# Global burden and strength of evidence for 88 risk factors in 204 countries and 811 subnational locations, 1990–2021: a systematic analysis for the Global Burden of Disease Study 2021

**DOI:** 10.1016/S0140-6736(24)00933-4

**Published:** 2024-05-18

**Authors:** GBD 2021 Risk Factors Collaborators, Michael Brauer, Gregory A Roth, Aleksandr Y Aravkin, Peng Zheng, Kalkidan Hassen Abate, Yohannes Habtegiorgis Abate, Cristiana Abbafati, Rouzbeh Abbasgholizadeh, Madineh Akram Abbasi, Mohammadreza Abbasian, Mitra Abbasifard, Mohsen Abbasi-Kangevari, Samar Abd ElHafeez, Sherief Abd-Elsalam, Parsa Abdi, Mohammad Abdollahi, Meriem Abdoun, Deldar Morad Abdulah, Auwal Abdullahi, Mesfin Abebe, Aidin Abedi, Armita Abedi, Tadesse M Abegaz, Roberto Ariel Abeldaño Zuñiga, Olumide Abiodun, Temesgen Lera Abiso, Richard Gyan Aboagye, Hassan Abolhassani, Mohamed Abouzid, Girma Beressa Aboye, Lucas Guimarães Abreu, Hasan Abualruz, Bilyaminu Abubakar, Eman Abu-Gharbieh, Hana Jihad Jihad Abukhadijah, Salahdein Aburuz, Ahmed Abu-Zaid, Mesafint Molla Adane, Isaac Yeboah Addo, Giovanni Addolorato, Rufus Adesoji Adedoyin, Victor Adekanmbi, Bashir Aden, Juliana Bunmi Adetunji, Temitayo Esther Adeyeoluwa, Rishan Adha, Amin Adibi, Qorinah Estiningtyas Sakilah Adnani, Leticia Akua Adzigbli, Aanuoluwapo Adeyimika Afolabi, Rotimi Felix Afolabi, Ashkan Afshin, Shadi Afyouni, Muhammad Sohail Afzal, Saira Afzal, Suneth Buddhika Agampodi, Faith Agbozo, Shahin Aghamiri, Antonella Agodi, Anurag Agrawal, Williams Agyemang-Duah, Bright Opoku Ahinkorah, Aqeel Ahmad, Danish Ahmad, Firdos Ahmad, Noah Ahmad, Shahzaib Ahmad, Tauseef Ahmad, Ali Ahmed, Anisuddin Ahmed, Ayman Ahmed, Luai A Ahmed, Muktar Beshir Ahmed, Safoora Ahmed, Syed Anees Ahmed, Marjan Ajami, Gizachew Taddesse Akalu, Essona Matatom Akara, Hossein Akbarialiabad, Shiva Akhlaghi, Karolina Akinosoglou, Tomi Akinyemiju, Mohammed Ahmed Akkaif, Sreelatha Akkala, Blessing Akombi-Inyang, Salah Al Awaidy, Syed Mahfuz Al Hasan, Fares Alahdab, Tareq Mohammed Ali AL-Ahdal, Samer O Alalalmeh, Tariq A Alalwan, Ziyad Al-Aly, Khurshid Alam, Nazmul Alam, Fahad Mashhour Alanezi, Turki M Alanzi, Almaza Albakri, Mohammad T AlBataineh, Wafa A Aldhaleei, Robert W Aldridge, Mulubirhan Assefa Alemayohu, Yihun Mulugeta Alemu, Bassam Al-Fatly, Adel Ali Saeed Al-Gheethi, Khairat Al-Habbal, Khalid F Alhabib, Robert Kaba Alhassan, Abid Ali, Amjad Ali, Beriwan Abdulqadir Ali, Iman Ali, Liaqat Ali, Mohammed Usman Ali, Rafat Ali, Syed Shujait Shujait Ali, Waad Ali, Gianfranco Alicandro, Sheikh Mohammad Alif, Syed Mohamed Aljunid, François Alla, Sabah Al-Marwani, Hesham M Al-Mekhlafi, Sami Almustanyir, Mahmoud A Alomari, Jordi Alonso, Jaber S Alqahtani, Ahmed Yaseen Alqutaibi, Rajaa M Al-Raddadi, Ahmad Alrawashdeh, Rami Hani Al-Rifai, Sahel Majed Alrousan, Salman Khalifah Al-Sabah, Najim Z Alshahrani, Zaid Altaany, Awais Altaf, Jaffar A Al-Tawfiq, Khalid A Altirkawi, Deborah Oyine Aluh, Nelson Alvis-Guzman, Nelson J Alvis-Zakzuk, Hassan Alwafi, Mohammad Sami Al-Wardat, Yaser Mohammed Al-Worafi, Hany Aly, Safwat Aly, Karem H Alzoubi, Walid Al-Zyoud, Uchenna Anderson Amaechi, Masous Aman Mohammadi, Reza Amani, Sohrab Amiri, Mohammad Hosein Amirzade-Iranaq, Enrico Ammirati, Hubert Amu, Dickson A Amugsi, Ganiyu Adeniyi Amusa, Robert Ancuceanu, Deanna Anderlini, Jason A Anderson, Pedro Prata Andrade, Catalina Liliana Andrei, Tudorel Andrei, Susan C Anenberg, Dhanalakshmi Angappan, Colin Angus, Abhishek Anil, Sneha Anil, Afifa Anjum, Amir Anoushiravani, Ippazio Cosimo Antonazzo, Catherine M Antony, Ernoiz Antriyandarti, Boluwatife Stephen Anuoluwa, Davood Anvari, Saeid Anvari, Saleha Anwar, Sumadi Lukman Anwar, Razique Anwer, Ekenedilichukwu Emmanuel Anyabolo, Anayochukwu Edward Anyasodor, Geminn Louis Carace Apostol, Jalal Arabloo, Razman Arabzadeh Bahri, Mosab Arafat, Demelash Areda, Brhane Berhe Aregawi, Abdulfatai Aremu, Benedetta Armocida, Michael Benjamin Arndt, Johan Ärnlöv, Mahwish Arooj, Anton A Artamonov, Kurnia Dwi Artanti, Idowu Thomas Aruleba, Ashokan Arumugam, Akram M Asbeutah, Saeed Asgary, Akeza Awealom Asgedom, Charlie Ashbaugh, Mubarek Yesse Ashemo, Tahira Ashraf, Amir Askarinejad, Michael Assmus, Thomas Astell-Burt, Mohammad Athar, Seyyed Shamsadin Athari, Prince Atorkey, Alok Atreya, Avinash Aujayeb, Marcel Ausloos, Leticia Avila-Burgos, Andargie Abate Awoke, Beatriz Paulina Ayala Quintanilla, Haleh Ayatollahi, Carlos Ayestas Portugal, Jose L Ayuso-Mateos, Sina Azadnajafabad, Rui M S Azevedo, Gulrez Shah Azhar, Hosein Azizi, Ahmed Y Azzam, Insa Linnea Backhaus, Muhammad Badar, Ashish D Badiye, Arvind Bagga, Soroush Baghdadi, Nasser Bagheri, Sara Bagherieh, Pegah Bahrami Taghanaki, Ruhai Bai, Atif Amin Baig, Jennifer L Baker, Shankar M Bakkannavar, Madhan Balasubramanian, Ovidiu Constantin Baltatu, Kiran Bam, Soham Bandyopadhyay, Biswajit Banik, Palash Chandra Banik, Aduragbemi Banke-Thomas, Hansi Bansal, Martina Barchitta, Mainak Bardhan, Erfan Bardideh, Suzanne Lyn Barker-Collo, Till Winfried Bärnighausen, Francesco Barone-Adesi, Hiba Jawdat Barqawi, Lope H Barrero, Amadou Barrow, Sandra Barteit, Zarrin Basharat, Afisu Basiru, João Diogo Basso, Mohammad-Mahdi Bastan, Sanjay Basu, Sai Batchu, Kavita Batra, Ravi Batra, Bernhard T Baune, Mohsen Bayati, Nebiyou Simegnew Bayileyegn, Thomas Beaney, Amir Hossein Behnoush, Maryam Beiranvand, Yannick Béjot, Alehegn Bekele, Uzma Iqbal Belgaumi, Arielle Wilder Bell, Michelle L Bell, Muhammad Bashir Bello, Olorunjuwon Omolaja Bello, Luis Belo, Apostolos Beloukas, Salaheddine Bendak, Derrick A Bennett, Fiona B Bennitt, Isabela M Bensenor, Habib Benzian, Azizullah Beran, Zombor Berezvai, Eduardo Bernabe, Robert S Bernstein, Paulo J G Bettencourt, Akshaya Srikanth Bhagavathula, Neeraj Bhala, Dinesh Bhandari, Nikha Bhardwaj, Pankaj Bhardwaj, Sonu Bhaskar, Ajay Nagesh Bhat, Vivek Bhat, Gurjit Kaur Bhatti, Jasvinder Singh Bhatti, Manpreet S Bhatti, Rajbir Bhatti, Mohiuddin Ahmed Bhuiyan, Zulfiqar A Bhutta, Boris Bikbov, Jessica Devin Bishai, Catherine Bisignano, Atanu Biswas, Bijit Biswas, Raaj Kishore Biswas, Tone Bjørge, Micheal Kofi Boachie, Hosea Boakye, Moses John Bockarie, Virginia Bodolica, Aadam Olalekan Bodunrin, Eyob Ketema Bogale, Srinivasa Rao Bolla, Archith Boloor, Milad Bonakdar Hashemi, Sri Harsha Boppana, Berrak Bora Basara, Hamed Borhany, Alejandro Botero Carvajal, Souad Bouaoud, Soufiane Boufous, Rupert Bourne, Christopher Boxe, Dejana Braithwaite, Luisa C Brant, Amanpreet Brar, Nicholas J K Breitborde, Susanne Breitner, Hermann Brenner, Andrey Nikolaevich Briko, Gabrielle Britton, Colin Stewart Brown, Annie J Browne, Andre R Brunoni, Dana Bryazka, Norma B Bulamu, Lemma N Bulto, Danilo Buonsenso, Katrin Burkart, Richard A Burns, Reinhard Busse, Yasser Bustanji, Nadeem Shafique Butt, Zahid A Butt, Florentino Luciano Caetano dos Santos, Jack Cagney, Lucero Cahuana-Hurtado, Daniela Calina, Luis Alberto Cámera, Luciana Aparecida Campos, Ismael R Campos-Nonato, Chao Cao, Fan Cao, Yubin Cao, Angelo Capodici, Rosario Cárdenas, Sinclair Carr, Giulia Carreras, Juan J Carrero, Andrea Carugno, Felix Carvalho, Márcia Carvalho, Joao Mauricio Castaldelli-Maia, Carlos A Castañeda-Orjuela, Giulio Castelpietra, Ferrán Catalá-López, Alberico L Catapano, Maria Sofia Cattaruzza, Arthur Caye, Christopher R Cederroth, Luca Cegolon, Muthia Cenderadewi, Kelly M Cercy, Ester Cerin, Joshua Chadwick, Chiranjib Chakraborty, Promit Ananyo Chakraborty, Sandip Chakraborty, Jeffrey Shi Kai Chan, Raymond N C Chan, Joht Singh Chandan, Rama Mohan Chandika, Pankaj Chaturvedi, An-Tian Chen, Catherine S Chen, Haowei Chen, Meng Xuan Chen, Mingling Chen, Simiao Chen, Ching-Yu Cheng, Esther T W Cheng, Nicolas Cherbuin, Gerald Chi, Fatemeh Chichagi, Odgerel Chimed-Ochir, Ritesh Chimoriya, Patrick R Ching, Jesus Lorenzo Chirinos-Caceres, Abdulaal Chitheer, William C S Cho, Bryan Chong, Hitesh Chopra, Rajiv Chowdhury, Devasahayam J Christopher, Dinh-Toi Chu, Isaac Sunday Chukwu, Eric Chung, Sheng-Chia Chung, Muhammad Chutiyami, Iolanda Cioffi, Rebecca M Cogen, Aaron J Cohen, Alyssa Columbus, Joao Conde, Alexandru Corlateanu, Samuele Cortese, Paolo Angelo Cortesi, Vera Marisa Costa, Simona Costanzo, Michael H Criqui, Jessica A Cruz, Natália Cruz-Martins, Garland T Culbreth, Alanna Gomes da Silva, Omid Dadras, Xiaochen Dai, Zhaoli Dai, Patience Unekwuojo Daikwo, Lachlan L Dalli, Giovanni Damiani, Emanuele D'Amico, Lucio D'Anna, Aso Mohammad Darwesh, Jai K Das, Subasish Das, Nihar Ranjan Dash, Mohsen Dashti, Claudio Alberto Dávila-Cervantes, Nicole Davis Weaver, Dragos Virgil Davitoiu, Fernando Pio De la Hoz, Alejandro de la Torre-Luque, Diego De Leo, Shayom Debopadhaya, Louisa Degenhardt, Cristian Del Bo', Ivan Delgado-Enciso, Juana Maria Delgado-Saborit, Chalachew Kassaw Demoze, Edgar Denova-Gutiérrez, Nikolaos Dervenis, Emina Dervišević, Hardik Dineshbhai Desai, Rupak Desai, Vinoth Gnana Chellaiyan Devanbu, Syed Masudur Rahman Dewan, Arkadeep Dhali, Kuldeep Dhama, Amol S Dhane, Mandira Lamichhane Dhimal, Meghnath Dhimal, Sameer Dhingra, Vishal R Dhulipala, Raja Ram Dhungana, Diana Dias da Silva, Daniel Diaz, Luis Antonio Diaz, Michael J Diaz, Adriana Dima, Delaney D Ding, Monica Dinu, Shirin Djalalinia, Thanh Chi Do, Thao Huynh Phuong Do, Camila Bruneli do Prado, Masoud Dodangeh, Sushil Dohare, Klara Georgieva Dokova, Wanyue Dong, Deepa Dongarwar, Mario D'Oria, Fariba Dorostkar, E Ray Dorsey, Rajkumar Doshi, Leila Doshmangir, Robert Kokou Dowou, Tim Robert Driscoll, Ashel Chelsea Dsouza, Haneil Larson Dsouza, Samuel C Dumith, Bruce B Duncan, Andre Rodrigues Duraes, Senbagam Duraisamy, Anar Dushpanova, Paulina Agnieszka Dzianach, Arkadiusz Marian Dziedzic, Alireza Ebrahimi, Chidiebere Peter Echieh, Abdelaziz Ed-Dra, Hisham Atan Edinur, David Edvardsson, Kristina Edvardsson, Ferry Efendi, Aziz Eftekharimehrabad, Ebrahim Eini, Michael Ekholuenetale, Temitope Cyrus Ekundayo, Rabie Adel El Arab, Maysaa El Sayed Zaki, Faris El-Dahiyat, Noha Mousaad Elemam, Frank J Elgar, Ghada Metwally Tawfik ElGohary, Hala Rashad Elhabashy, Muhammed Elhadi, Ahmed O Elmehrath, Omar Abdelsadek Abdou Elmeligy, Mohammed Elshaer, Ibrahim Elsohaby, Theophilus I Emeto, Negin Esfandiari, Babak Eshrati, Majid Eslami, Sayed Vahid Esmaeili, Kara Estep, Farshid Etaee, Natalia Fabin, Adeniyi Francis Fagbamigbe, Omotayo Francis Fagbule, Saman Fahimi, Luca Falzone, Mohammad Fareed, Carla Sofia e Sá Farinha, MoezAlIslam Ezzat Mahmoud Faris, Pawan Sirwan Faris, Andre Faro, Folorunso Oludayo Fasina, Ali Fatehizadeh, Nelsensius Klau Fauk, Timur Fazylov, Valery L Feigin, Xiaoqi Feng, Seyed-Mohammad Fereshtehnejad, Abdullah Hamid Feroze, Pietro Ferrara, Alize J Ferrari, Nuno Ferreira, Getahun Fetensa, Bikila Regassa Feyisa, Irina Filip, Florian Fischer, Ida Fitriana, Joanne Flavel, Carsten Flohr, David Flood, Luisa S Flor, Nataliya A Foigt, Morenike Oluwatoyin Folayan, Lisa M Force, Daniela Fortuna, Matteo Foschi, Richard Charles Franklin, Alberto Freitas, Sara D Friedman, Blima Fux, Sridevi G, Peter Andras Gaal, Santosh Gaihre, Márió Gajdács, Yaseen Galali, Silvano Gallus, Aravind P Gandhi, Balasankar Ganesan, Mohammad Arfat Ganiyani, Vanessa Garcia, William M Gardner, Ravindra K Garg, Rupesh K Gautam, Tilaye Gebru Gebi, Miglas W Gebregergis, Mesfin Gebrehiwot, Tesfay B B Gebremariam, Teferi Gebru Gebremeskel, Urge Gerema, Lemma Getacher, Genanew K a Getahun, Molla Getie, Fataneh Ghadirian, Sadegh Ghafarian, Amir Ghaffari Jolfayi, Khalid Yaser Ghailan, Alireza Ghajar, MohammadReza Ghasemi, Ghazal Ghasempour Dabaghi, Afsaneh Ghasemzadeh, Fariba Ghassemi, Ramy Mohamed Ghazy, Ali Gholami, Ali Gholamrezanezhad, Nasim Gholizadeh, Mahsa Ghorbani, Artyom Urievich Gil, Gabriela Fernanda Gil, Nora M Gilbertson, Paramjit Singh Gill, Tiffany K Gill, Ebisa Zerihun Gindaba, Alem Girmay, James C Glasbey, Elena V Gnedovskaya, Laszlo Göbölös, Myron Anthony Godinho, Amit Goel, Mahaveer Golechha, Pouya Goleij, Davide Golinelli, Nelson G M Gomes, Sameer Vali Gopalani, Giuseppe Gorini, Houman Goudarzi, Alessandra C Goulart, Mahdi Gouravani, Anmol Goyal, Simon Matthew Graham, Michal Grivna, Giuseppe Grosso, Shi-Yang Guan, Giovanni Guarducci, Mohammed Ibrahim Mohialdeen Gubari, Avirup Guha, Stefano Guicciardi, Snigdha Gulati, David Gulisashvili, Damitha Asanga Gunawardane, Cui Guo, Anish Kumar Gupta, Bhawna Gupta, Mohak Gupta, Rahul Gupta, Rajat Das Gupta, Rajeev Gupta, Sapna Gupta, Veer Bala Gupta, Vijai Kumar Gupta, Vivek Kumar Gupta, Farrokh Habibzadeh, Parham Habibzadeh, Tesfahun Simon Hadaro, Zahra Hadian, Nils Haep, Hamed Haghi-Aminjan, Dariush Haghmorad, Hailey Hagins, Demewoz Haile, Alemayehu Hailu, Adel Hajj Ali, Esam S Halboub, Aram Halimi, Brian J Hall, Sebastian Haller, Rabih Halwani, Randah R Hamadeh, Nadia M Hamdy, Sajid Hameed, Samer Hamidi, Ahmad Hammoud, Asif Hanif, Nasrin Hanifi, Zaim Anan Haq, Md Rabiul Haque, Harapan Harapan, Arief Hargono, Josep Maria Haro, Ahmed I Hasaballah, Ikramul Hasan, Mohammad Jahid Hasan, S M Mahmudul Hasan, Hamidreza Hasani, Mohammad Hasanian, Nadim Hashmeh, Md Saquib Hasnain, Amr Hassan, Ikrama Hassan, Mahgol Sadat Hassan Zadeh Tabatabaei, Shokoufeh Hassani, Soheil Hassanipour, Hadi Hassankhani, Johannes Haubold, Rasmus J Havmoeller, Simon I Hay, Jeffrey J Hebert, Omar E Hegazi, Tadele Yohannes Hegena, Golnaz Heidari, Mohammad Heidari, Bartosz Helfer, Mehdi Hemmati, Claire A Henson, Molly E Herbert, Claudiu Herteliu, Austin Heuer, Kamal Hezam, Thomas Kwadwo Hinneh, Yuta Hiraike, Nguyen Quoc Hoan, Ramesh Holla, Julia Hon, Mohammad Enamul Hoque, Nobuyuki Horita, Sahadat Hossain, Seyed Ehsan Hosseini, Hassan Hosseinzadeh, Mehdi Hosseinzadeh, Mihaela Hostiuc, Sorin Hostiuc, Hanno Hoven, Mohamed Hsairi, Johnathan M Hsu, Chengxi Hu, Junjie Huang, Md Nazmul Huda, Erin N Hulland, Michael Hultström, Kiavash Hushmandi, Javid Hussain, Nawfal R Hussein, Chantal K Huynh, Hong-Han Huynh, Segun Emmanuel Ibitoye, Oluwatope Olaniyi Idowu, Audrey L Ihler, Nayu Ikeda, Kevin S Ikuta, Olayinka Stephen Ilesanmi, Irena M Ilic, Milena D Ilic, Mohammad Tarique Imam, Mustapha Immurana, Leeberk Raja Inbaraj, Lalu Muhammad Irham, Mustafa Alhaji Isa, Md Rabiul Islam, Faisal Ismail, Nahlah Elkudssiah Ismail, Hiroyasu Iso, Gaetano Isola, Masao Iwagami, Chidozie C D Iwu, Chinwe Juliana Iwu-Jaja, Vinothini J, Jalil Jaafari, Louis Jacob, Kathryn H Jacobsen, Farhad Jadidi-Niaragh, Kasra Jahankhani, Nader Jahanmehr, Haitham Jahrami, Akhil Jain, Nityanand Jain, Ammar Abdulrahman Jairoun, Abhishek Jaiswal, Mihajlo Jakovljevic, Reza Jalilzadeh Yengejeh, Roland Dominic G Jamora, Abubakar Ibrahim Jatau, Sabzali Javadov, Tahereh Javaheri, Shubha Jayaram, Jayakumar Jeganathan, Bijay Mukesh Jeswani, Heng Jiang, Catherine O Johnson, Mohammad Jokar, Nabi Jomehzadeh, Jost B Jonas, Tamas Joo, Abel Joseph, Nitin Joseph, Vivek Joshi, Charity Ehimwenma Joshua, Jacek Jerzy Jozwiak, Mikk Jürisson, Billingsley Kaambwa, Ali Kabir, Zubair Kabir, Vidya Kadashetti, Ethan M Kahn, Rizwan Kalani, Feroze Kaliyadan, Sanjay Kalra, Rajesh Kamath, Thanigaivelan Kanagasabai, Tanuj Kanchan, Himal Kandel, Edmund Wedam Kanmiki, Kehinde Kazeem Kanmodi, Sushil Kumar Kansal, Daniel John Kapner, Neeti Kapoor, Efstratios Karagiannidis, Mehrdad Karajizadeh, Paschalis Karakasis, Shama D Karanth, Ibraheem M Karaye, André Karch, Asima Karim, Hanie Karimi, Shilpi Karmakar, Faizan Zaffar Kashoo, Hengameh Kasraei, Woldeteklehaymanot Dagne Kassahun, Nicholas J Kassebaum, Molly B Kassel, Srinivasa Vittal Katikireddi, Joonas H Kauppila, Norito Kawakami, Neda Kaydi, Gbenga A Kayode, Foad Kazemi, Peter Njenga Keiyoro, Laura Kemmer, John H Kempen, Jessica A Kerr, Emmanuelle Kesse-Guyot, Yousef Saleh Khader, Morteza Abdullatif Khafaie, Himanshu Khajuria, Amirmohammad Khalaji, Mariam Khalil, Alireza Khalilian, Faham Khamesipour, Asaduzzaman Khan, M Nuruzzaman Khan, Maseer Khan, Mohammad Jobair Khan, Moien AB Khan, Shaghayegh Khanmohammadi, Khaled Khatab, Haitham Khatatbeh, Moawiah Mohammad Khatatbeh, Mahalaqua Nazli Khatib, Armin Khavandegar, Hamid Reza Khayat Kashani, Feriha Fatima Khidri, Elaheh Khodadoust, Moein Khormali, Zahra Khorrami, Atulya Aman Khosla, Mahmood Khosrowjerdi, Haneen Khreis, Helda Khusun, Zemene Demelash Kifle, Kwanghyun Kim, Min Seo Kim, Yun Jin Kim, Ruth W Kimokoti, Adnan Kisa, Sezer Kisa, Luke D Knibbs, Ann Kristin Skrindo Knudsen, David S Q Koh, Ali-Asghar Kolahi, Farzad Kompani, Jianqiu Kong, Gerbrand Koren, Miikka Korja, Vladimir Andreevich Korshunov, Oleksii Korzh, Soewarta Kosen, Nikhil Kothari, Parvaiz A Koul, Sindhura Lakshmi Koulmane Laxminarayana, Kewal Krishan, Vijay Krishnamoorthy, Yuvaraj Krishnamoorthy, Bindu Krishnan, Kris J Krohn, Barthelemy Kuate Defo, Burcu Kucuk Bicer, Md Abdul Kuddus, Mohammed Kuddus, Nuworza Kugbey, Ilari Kuitunen, Mukhtar Kulimbet, Vishnutheertha Kulkarni, Ashish Kumar, Nithin Kumar, Vijay Kumar, Satyajit Kundu, Om P Kurmi, Asep Kusnali, Dian Kusuma, Tezer Kutluk, Carlo La Vecchia, Muhammad Awwal Ladan, Lucie Laflamme, Chandrakant Lahariya, Daphne Teck Ching Lai, Dharmesh Kumar Lal, Tea Lallukka, Judit Lám, Qing Lan, Tuo Lan, Iván Landires, Francesco Lanfranchi, Berthold Langguth, Van Charles Lansingh, Ariane Laplante-Lévesque, Bagher Larijani, Anders O Larsson, Savita Lasrado, Paolo Lauriola, Huu-Hoai Le, Long Khanh Dao Le, Nhi Huu Hanh Le, Thao Thi Thu Le, Janet L Leasher, Caterina Ledda, Munjae Lee, Paul H Lee, Seung Won Lee, Shaun Wen Huey Lee, Yo Han Lee, Kate E LeGrand, James Leigh, Elvynna Leong, Temesgen L Lerango, Haley Lescinsky, Janni Leung, Ming-Chieh Li, Wang-Zhong Li, Wei Li, Yichong Li, Zhihui Li, Virendra S Ligade, Lee-Ling Lim, Stephen S Lim, Ro-Ting Lin, Shuzhi Lin, Chaojie Liu, Gang Liu, Jinli Liu, Jue Liu, Richard T Liu, Shiwei Liu, Wei Liu, Xiaofeng Liu, Xuefeng Liu, Katherine M Livingstone, Erand Llanaj, Ayush Lohiya, Rubén López-Bueno, Platon D Lopukhov, Stefan Lorkowski, Paulo A Lotufo, Rafael Lozano, Jailos Lubinda, Giancarlo Lucchetti, Lisha Luo, Hengliang lv, Hawraz Ibrahim M Amin, Zheng Feei Ma, Kelsey Lynn Maass, Mahmoud Mabrok, Nikolaos Machairas, Monika Machoy, Asma Mafhoumi, Mohammed Magdy Abd El Razek, Azzam A Maghazachi, D R Mahadeshwara Prasad, Sandeep B Maharaj, Mansour Adam Mahmoud, Elham Mahmoudi, Azeem Majeed, Omar Mohamed Makram, Konstantinos Christos Makris, Satyaveni Malasala, Venkatesh Maled, Kashish Malhotra, Ahmad Azam Malik, Iram Malik, Lesibana Anthony Malinga, Deborah Carvalho Malta, Abdullah A Mamun, Ana Laura Manda, Yosef Manla, Ali Mansour, Borhan Mansouri, Pejman Mansouri, Marjan Mansourian, Mohammad Ali Mansournia, Lorenzo Giovanni Mantovani, Emmanuel Manu, Hamid Reza Marateb, Joemer C Maravilla, Elizabeth Marsh, Gabriel Martinez, Ramon Martinez-Piedra, Santi Martini, Francisco Rogerlândio Martins-Melo, Miquel Martorell, Wolfgang Marx, Sharmeen Maryam, Yasith Mathangasinghe, Alexander G Mathioudakis, Fernanda Penido Matozinhos, Jishanth Mattumpuram, Andrea Maugeri, Pallab K Maulik, Mahsa Mayeli, Mohsen Mazidi, Antonio Mazzotti, John J McGrath, Martin McKee, Anna Laura W McKowen, Susan A McLaughlin, Michael A McPhail, Steven M McPhail, Enkeleint A Mechili, Asim Mehmood, Khalid Mehmood, Kamran Mehrabani-Zeinabad, Entezar Mehrabi Nasab, Toni Meier, Fabiola Mejia-Rodriguez, Tesfahun Mekene Meto, Birye Dessalegn Mekonnen, Ritesh G Menezes, Belayneh Mengist, George A Mensah, Laverne G Mensah, Alexios-Fotios A Mentis, Sultan Ayoub Meo, Atte Meretoja, Tuomo J Meretoja, Abera M Mersha, Bezawit Afework Mesfin, Tomislav Mestrovic, Kukulege Chamila Dinushi Mettananda, Sachith Mettananda, Tomasz Miazgowski, Georgia Micha, Irmina Maria Michalek, Ana Carolina Micheletti Gomide Nogueira de Sá, Ted R Miller, Mojde Mirarefin, Mojgan Mirghafourvand, Andreea Mirica, Antonio Mirijello, Erkin M Mirrakhimov, Arvin Mirshahi, Maryam Mirzaei, Ajay Kumar Mishra, Vinaytosh Mishra, Philip B Mitchell, Prasanna Mithra, Chaitanya Mittal, Babak Moazen, Madeline E Moberg, Gabriele Mocciaro, Ashraf Mohamadkhani, Abdalla Z Mohamed, Ahmed Ismail Mohamed, Jama Mohamed, Mouhand F H Mohamed, Nouh Saad Mohamed, Esmaeil Mohammadi, Saeed Mohammadi, Abdollah Mohammadian-Hafshejani, Noushin Mohammadifard, Hussen Mohammed, Mustapha Mohammed, Salahuddin Mohammed, Shafiu Mohammed, Ali H Mokdad, Lorenzo Monasta, Stefania Mondello, Mohammad Ali Moni, AmirAli Moodi Ghalibaf, Catrin E Moore, Maryam Moradi, Yousef Moradi, Paula Moraga, Lidia Morawska, Rafael Silveira Moreira, Negar Morovatdar, Shane Douglas Morrison, Jakub Morze, Reza Mosaddeghi Heris, Elias Mossialos, Rohith Motappa, Vincent Mougin, Parsa Mousavi, Ahmed Msherghi, Sumaira Mubarik, Lorenzo Muccioli, Ulrich Otto Mueller, Francesk Mulita, Erin C Mullany, Kavita Munjal, Efrén Murillo-Zamora, BV Murlimanju, Ana-Maria Musina, Ghulam Mustafa, Sathish Muthu, Saravanan Muthupandian, Raman Muthusamy, Muhammad Muzaffar, Woojae Myung, Ayoub Nafei, Ahamarshan Jayaraman Nagarajan, Shankar Prasad Nagaraju, Gabriele Nagel, Mohsen Naghavi, Pirouz Naghavi, Ganesh R Naik, Gurudatta Naik, Firzan Nainu, Tapas Sadasivan Nair, Soroush Najdaghi, Noureddin Nakhostin Ansari, Dhairya P Nanavaty, Vinay Nangia, Sreenivas Narasimha Swamy, Delaram Narimani Davani, Bruno Ramos Nascimento, Gustavo G Nascimento, Abdulqadir J Nashwan, Zuhair S Natto, Javaid Nauman, Samidi N K Navaratna, Muhammad Naveed, Biswa Prakash Nayak, Vinod C Nayak, Rawlance Ndejjo, Sabina Onyinye Nduaguba, Hadush Negash, Ionut Negoi, Ruxandra Irina Negoi, Seyed Aria Nejadghaderi, Chakib Nejjari, Mohammad Hadi Nematollahi, Samata Nepal, Subas Neupane, Marie Ng, Georges Nguefack-Tsague, Josephine W Ngunjiri, Dang H Nguyen, Nhien Ngoc Y Nguyen, Phat Tuan Nguyen, Phuong The Nguyen, Van Thanh Nguyen, Duc Nguyen Tran Minh, Robina Khan Niazi, Sneha Ingle Nicholson, Jing Nie, Ali Nikoobar, Amin Reza Nikpoor, Dina Nur Anggraini Ningrum, Chukwudi A Nnaji, Efaq Ali Noman, Shuhei Nomura, Nafise Noroozi, Bo Norrving, Jean Jacques Noubiap, Chisom Adaobi Nri-Ezedi, George Ntaios, Mpiko Ntsekhe, Mengistu H Nunemo, Dieta Nurrika, Jerry John Nutor, Bogdan Oancea, Erin M O'Connell, Ismail A Odetokun, Martin James O'Donnell, Michael Safo Oduro, Adesola Adenike Ogunfowokan, Abiola Ogunkoya, In-Hwan Oh, Hassan Okati-Aliabad, Sylvester Reuben Okeke, Akinkunmi Paul Okekunle, Osaretin Christabel Okonji, Andrew T Olagunju, Omotola O Olasupo, Matthew Idowu Olatubi, Arão Belitardo Oliveira, Gláucia Maria Moraes Oliveira, Abdulhakeem Abayomi Olorukooba, Isaac Iyinoluwa Olufadewa, Bolajoko Olubukunola Olusanya, Jacob Olusegun Olusanya, Yinka Doris Oluwafemi, Hany A Omar, Ahmed Omar Bali, Goran Latif Omer, Kanyin Liane Ong, Sokking Ong, Obinna E Onwujekwe, Kenneth Ikenna Onyedibe, Anita Frimpomaa Oppong, Michal Ordak, Verner N Orish, Raffaele Ornello, Heather M Orpana, Alberto Ortiz, Esteban Ortiz-Prado, Wael M S Osman, Samuel M Ostroff, Uchechukwu Levi Osuagwu, Adrian Otoiu, Nikita Otstavnov, Stanislav S Otstavnov, Amel Ouyahia, Mayowa O Owolabi, Ifeoluwa Temitayo Oyeyemi, Oyetunde T Oyeyemi, Mahesh Padukudru P A, Kevin Pacheco-Barrios, Alicia Padron-Monedero, Jagadish Rao Padubidri, Pramod Kumar Pal, Tamás Palicz, Feng Pan, Hai-Feng Pan, Adrian Pana, Sujogya K Panda, Songhomitra Panda-Jonas, Ashok Pandey, Seithikurippu R Pandi-Perumal, Helena Ullyartha Pangaribuan, Ioannis Pantazopoulos, Anca Mihaela Pantea Stoian, Paraskevi Papadopoulou, Marie C Parent, Pragyan Paramita Parija, Romil R Parikh, Seoyeon Park, Sungchul Park, Nicholas Parsons, Ava Pashaei, Maja Pasovic, Roberto Passera, Shankargouda Patil, Dimitrios Patoulias, Venkata Suresh Patthipati, Uttam Paudel, Shrikant Pawar, Hamidreza Pazoki Toroudi, Amy E Peden, Paolo Pedersini, Minjin Peng, Umberto Pensato, Veincent Christian Filipino Pepito, Emmanuel K Peprah, Prince Peprah, Mario F P Peres, Arokiasamy Perianayagam, Norberto Perico, Simone Perna, Konrad Pesudovs, Ionela-Roxana Petcu, Fanny Emily Petermann-Rocha, Hoang Tran Pham, Anil K Philip, Michael R Phillips, Brandon V Pickering, Daniela Pierannunzio, Manon Pigeolet, David M Pigott, Zahra Zahid Piracha, Michael A Piradov, Enrico Pisoni, Mapa Prabhath Piyasena, Dietrich Plass, Evgenii Plotnikov, Dimitri Poddighe, Kevan R Polkinghorne, Ramesh Poluru, Constance Dimity Pond, Djordje S Popovic, Fabio Porru, Maarten J Postma, Govinda Raj Poudel, Ahmad Pour-Rashidi, Akram Pourshams, Naeimeh Pourtaheri, Disha Prabhu, Sergio I Prada, Jalandhar Pradhan, Pranil Man Singh Pradhan, Manya Prasad, Elton Junio Sady Prates, Hery Purnobasuki, Bharathi M Purohit, Jagadeesh Puvvula, Nameer Hashim Qasim, Ibrahim Qattea, Asma Saleem Qazi, Gangzhen Qian, Suli Qiu, Mehrdad Rabiee Rad, Amir Radfar, Raghu Anekal Radhakrishnan, Venkatraman Radhakrishnan, Hadi Raeisi Shahraki, Quinn Rafferty, Alireza Rafiei, Alberto Raggi, Pankaja Raghav Raghav, Nasiru Raheem, Fakher Rahim, Md Jillur Rahim, Mahban Rahimifard, Vafa Rahimi-Movaghar, Md Obaidur Rahman, Muhammad Aziz Rahman, Amir Masoud Rahmani, Bita Rahmani, Mohammad Rahmanian, Nazanin Rahmanian, Vahid Rahmanian, Masoud Rahmati, Setyaningrum Rahmawaty, Diego Raimondo, Sathish Rajaa, Vinoth Rajendran, Prashant Rajput, Mahmoud Mohammed Ramadan, Shakthi Kumaran Ramasamy, Premkumar Ramasubramani, Sheena Ramazanu, Pramod W Ramteke, Juwel Rana, Kritika Rana, Chhabi Lal Ranabhat, Amey Rane, Usha Rani, Annemarei Ranta, Chythra R Rao, Mithun Rao, Puja C Rao, Sowmya J Rao, Davide Rasella, Sina Rashedi, Vahid Rashedi, Mahsa Rashidi, Mohammad-Mahdi Rashidi, Ashkan Rasouli-Saravani, Zubair Ahmed Ratan, Giridhara Rathnaiah Babu, Santosh Kumar Rauniyar, Ilari Rautalin, David Laith Rawaf, Salman Rawaf, Reza Rawassizadeh, Christian Razo, Zinabu Ferede Ferede Reda, Murali Mohan Rama Krishna Reddy, Elrashdy Moustafa Mohamed Redwan, Lennart Reifels, Marissa B Reitsma, Giuseppe Remuzzi, Bhageerathy Reshmi, Serge Resnikoff, Stefano Restaino, Luis Felipe Reyes, Maryam Rezaei, Nazila Rezaei, Negar Rezaei, Mohsen Rezaeian, Taeho Gregory Rhee, Mavra A Riaz, Antonio Luiz P Ribeiro, Jennifer Rickard, Hannah Elizabeth Robinson-Oden, Célia Fortuna Rodrigues, Mónica Rodrigues, Jefferson Antonio Buendia Rodriguez, Leonardo Roever, Debby Syahru Romadlon, Luca Ronfani, Jennifer Jacqueline Rosauer, Gholamreza Roshandel, Morteza Rostamian, Kunle Rotimi, Himanshu Sekhar Rout, Bedanta Roy, Nitai Roy, Enrico Rubagotti, Guilherme de Andrade Ruela, Susan Fred Rumisha, Tilleye Runghien, Michele Russo, Sacha Walde Ruzzante, Chandan S N, Aly M A Saad, Korosh Saber, Maha Mohamed Saber-Ayad, Siamak Sabour, Simona Sacco, Perminder S Sachdev, Rajesh Sachdeva, Basema Saddik, Adam Saddler, Bashdar Abuzed Sadee, Ehsan Sadeghi, Masoumeh Sadeghi, Elham Sadeghi Majd, Mohammad Reza Saeb, Umar Saeed, Mehdi Safari, Sare Safi, Sher Zaman Safi, Rajesh Sagar, Dominic Sagoe, Fatemeh Saheb Sharif-Askari, Narjes Saheb Sharif-Askari, Amirhossein Sahebkar, Soumya Swaroop Sahoo, Monalisha Sahu, Zahra Saif, Mirza Rizwan Sajid, Joseph W Sakshaug, Nasir Salam, Payman Salamati, Afeez Abolarinwa Salami, Luciane B Salaroli, Leili Salehi, Sana Salehi, Marwa Rashad Salem, Mohammed Z Y Salem, Dauda Salihu, Sohrab Salimi, Giovanni A Salum, Hossein Samadi Kafil, Sara Samadzadeh, Yoseph Leonardo Samodra, Vijaya Paul Samuel, Abdallah M Samy, Juan Sanabria, Rama Krishna Sanjeev, Francesca Sanna, Damian Francesco Santomauro, Milena M Santric-Milicevic, Made Ary Sarasmita, Sivan Yegnanarayana Iyer Saraswathy, Aswini Saravanan, Babak Saravi, Yaser Sarikhani, Rodrigo Sarmiento-Suárez, Gargi Sachin Sarode, Sachin C Sarode, Benn Sartorius, Arash Sarveazad, Brijesh Sathian, Davide Sattin, Monika Sawhney, Ganesh Kumar Saya, Abu Sayeed, Md Abu Sayeed, Mehdi Sayyah, Christophe Schinckus, Maria Inês Schmidt, Art Schuermans, Austin E Schumacher, Aletta Elisabeth Schutte, Michaël Schwarzinger, David C Schwebel, Falk Schwendicke, Siddharthan Selvaraj, Mohammad H Semreen, Subramanian Senthilkumaran, Dragos Serban, Marc L Serre, Yashendra Sethi, Mahan Shafie, Humaira Shah, Nilay S Shah, Pritik A Shah, Syed Mahboob Shah, Ataollah Shahbandi, Amira A Shaheen, Samiah Shahid, Wajeehah Shahid, Hamid R Shahsavari, Moyad Jamal Shahwan, Masood Ali Shaikh, Summaiya Zareen Shaikh, Ali S Shalash, Sunder Sham, Muhammad Aaqib Shamim, Mehran Shams-Beyranvand, Mohammad Ali Shamshirgaran, Mohammad Anas Shamsi, Mohd Shanawaz, Abhishek Shankar, Sadaf Sharfaei, Amin Sharifan, Javad Sharifi-Rad, Manoj Sharma, Ujjawal Sharma, Vishal Sharma, Rajesh P Shastry, Amin Shavandi, Amr Mohamed Elsayed Shehabeldine, Somia Shehzadi, Aziz Sheikh, Jiabin Shen, Adithi Shetty, B Suresh Kumar Shetty, Pavanchand H Shetty, Amir Shiani, Desalegn Shiferaw, Mika Shigematsu, Min-Jeong Shin, Rahman Shiri, Aminu Shittu, Ivy Shiue, K M Shivakumar, Velizar Shivarov, Sina Shool, Seyed Afshin Shorofi, Rajan Shrestha, Sunil Shrestha, Kanwar Hamza Shuja, Kerem Shuval, Yafei Si, Emmanuel Edwar Siddig, Diego Augusto Santos Silva, Luís Manuel Lopes Rodrigues Silva, Soraia Silva, Thales Philipe R Silva, Colin R Simpson, Abhinav Singh, Balbir Bagicha Singh, Baljinder Singh, Garima Singh, Harmanjit Singh, Jasvinder A Singh, Mahendra Singh, Narinder Pal Singh, Paramdeep Singh, Surjit Singh, Robert Sinto, Shravan Sivakumar, Samarjeet Singh Siwal, Natia Skhvitaridze, Søren T Skou, David A Sleet, Farrukh Sobia, Matiwos Soboka, Bogdan Socea, Shahabaddin Solaimanian, Ranjan Solanki, Shipra Solanki, Sameh S M Soliman, Ranjani Somayaji, Yi Song, Reed J D Sorensen, Joan B Soriano, Ireneous N Soyiri, Michael Spartalis, Sandra Spearman, Cory N Spencer, Chandrashekhar T Sreeramareddy, Panagiotis Stachteas, Lauryn K Stafford, Jeffrey D Stanaway, Muhammad Haroon Stanikzai, Caroline Stein, Dan J Stein, Fridolin Steinbeis, Caitlyn Steiner, Sabine Steinke, Paschalis Steiropoulos, Leo Stockfelt, Mark A Stokes, Kurt Straif, Saverio Stranges, Narayan Subedi, Vetriselvan Subramaniyan, Muhammad Suleman, Rizwan Suliankatchi Abdulkader, Johan Sundström, David Sunkersing, Katharina S Sunnerhagen, Vinay Suresh, Chandan Kumar Swain, Lukasz Szarpak, Mindy D Szeto, Payam Tabaee Damavandi, Rafael Tabarés-Seisdedos, Seyyed Mohammad Tabatabaei, Ozra Tabatabaei Malazy, Seyed-Amir Tabatabaeizadeh, Shima Tabatabai, Celine Tabche, Mohammad Tabish, Santosh Kumar Tadakamadla, Yasaman Taheri Abkenar, Moslem Taheri Soodejani, Amir Taherkhani, Jabeen Taiba, Ken Takahashi, Iman M Talaat, Jacques Lukenze Tamuzi, Ker-Kan Tan, Haosu Tang, Nathan Y Tat, Nuno Taveira, Yibekal Manaye Tefera, Arash Tehrani-Banihashemi, Worku Animaw Temesgen, Mohamad-Hani Temsah, Masayuki Teramoto, Dufera Rikitu Terefa, Enoch Teye-Kwadjo, Ramna Thakur, Pugazhenthan Thangaraju, Kavumpurathu Raman Thankappan, Rekha Thapar, Rasiah Thayakaran, Sathish Thirunavukkarasu, Nihal Thomas, Nikhil Kenny Thomas, Jing Tian, Ales Tichopad, Jansje Henny Vera Ticoalu, Tenaw Yimer Tiruye, Ruoyan Tobe-Gai, Musliu Adetola Tolani, Tadesse Tolossa, Marcello Tonelli, Roman Topor-Madry, Fotis Topouzis, Mathilde Touvier, Marcos Roberto Tovani-Palone, Khaled Trabelsi, Jasmine T Tran, Mai Thi Ngoc Tran, Nghia Minh Tran, Domenico Trico, Indang Trihandini, Christopher E Troeger, Samuel Joseph Tromans, Thien Tan Tri Tai Truyen, Aristidis Tsatsakis, Evangelia Eirini Tsermpini, Munkhtuya Tumurkhuu, Aniefiok John Udoakang, Arit Udoh, Atta Ullah, Saeed Ullah, Sana Ullah, Muhammad Umair, Srikanth Umakanthan, Brigid Unim, Bhaskaran Unnikrishnan, Era Upadhyay, Daniele Urso, Jibrin Sammani Usman, Asokan Govindaraj Vaithinathan, Omid Vakili, Mario Valenti, Rohollah Valizadeh, Jef Van den Eynde, Aaron van Donkelaar, Orsolya Varga, Priya Vart, Shoban Babu Varthya, Tommi Juhani Vasankari, Milena Vasic, Siavash Vaziri, Narayanaswamy Venketasubramanian, Nicholas Alexander Verghese, Madhur Verma, Massimiliano Veroux, Georgios-Ioannis Verras, Dominique Vervoort, Jorge Hugo Villafañe, Victor E Villalobos-Daniel, Leonardo Villani, Gabriela Ines Villanueva, Manish Vinayak, Francesco S Violante, Vasily Vlassov, Bay Vo, Stein Emil Vollset, Simona Ruxandra Volovat, Theo Vos, Isidora S Vujcic, Yasir Waheed, Cong Wang, Fang Wang, Shu Wang, Yanzhong Wang, Yuan-Pang Wang, Mary Njeri Wanjau, Muhammad Waqas, Paul Ward, Abdul Waris, Emebet Gashaw Wassie, Kosala Gayan Weerakoon, Robert G Weintraub, Daniel J Weiss, Eli J Weiss, Haftom Legese Legese Weldetinsaa, Katherine M Wells, Yi Feng Wen, Taweewat Wiangkham, Nuwan Darshana Wickramasinghe, Caroline Wilkerson, Peter Willeit, Shadrach Wilson, Yen Jun Wong, Utoomporn Wongsin, Sarah Wozniak, Chenkai Wu, Dongze Wu, Felicia Wu, Zenghong Wu, Juan Xia, Hong Xiao, Suowen Xu, Xiaoyue Xu, Yvonne Yiru Xu, Mukesh Kumar Yadav, Sajad Yaghoubi, Kazumasa Yamagishi, Lin Yang, Yuichiro Yano, Habib Yaribeygi, Yuichi Yasufuku, Pengpeng Ye, Renjulal Yesodharan, Subah Abderehim Yesuf, Saber Yezli, Siyan Yi, Arzu Yiğit, Zeamanuel Anteneh Yigzaw, Dehui Yin, Paul Yip, Malede Berihun Yismaw, Dong Keon Yon, Naohiro Yonemoto, Yuyi You, Mustafa Z Younis, Zabihollah Yousefi, Chuanhua Yu, Yong Yu, Siddhesh Zadey, Vesna Zadnik, Fathiah Zakham, Nazar Zaki, Josefina Zakzuk, Giulia Zamagni, Sojib Bin Zaman, Ghazal G Z Zandieh, Aurora Zanghì, Heather J Zar, Iman Zare, Fatemeh Zarimeidani, Mikhail Sergeevich Zastrozhin, Youjie Zeng, Chunxia Zhai, Anthony Lin Zhang, Haijun Zhang, Liqun Zhang, Meixin Zhang, Yunquan Zhang, Zhenyu Zhang, Zhi-Jiang Zhang, Hanqing Zhao, Jeff T Zhao, Xiu-Ju George Zhao, Yang Zhao, Yong Zhao, Chenwen Zhong, Jingjing Zhou, Juexiao Zhou, Shangcheng Zhou, Bin Zhu, Lei Zhu, Zhaohua Zhu, Boback Ziaeian, Makan Ziafati, Magdalena Zielińska, Stephanie R M Zimsen, Ghazal Zoghi, Thomas Zoller, Alimuddin Zumla, Sa'ed H Zyoud, Samer H Zyoud, Christopher J L Murray, Emmanuela Gakidou

## Abstract

**Background:**

Understanding the health consequences associated with exposure to risk factors is necessary to inform public health policy and practice. To systematically quantify the contributions of risk factor exposures to specific health outcomes, the Global Burden of Diseases, Injuries, and Risk Factors Study (GBD) 2021 aims to provide comprehensive estimates of exposure levels, relative health risks, and attributable burden of disease for 88 risk factors in 204 countries and territories and 811 subnational locations, from 1990 to 2021.

**Methods:**

The GBD 2021 risk factor analysis used data from 54 561 total distinct sources to produce epidemiological estimates for 88 risk factors and their associated health outcomes for a total of 631 risk–outcome pairs. Pairs were included on the basis of data-driven determination of a risk–outcome association. Age-sex-location-year-specific estimates were generated at global, regional, and national levels. Our approach followed the comparative risk assessment framework predicated on a causal web of hierarchically organised, potentially combinative, modifiable risks. Relative risks (RRs) of a given outcome occurring as a function of risk factor exposure were estimated separately for each risk–outcome pair, and summary exposure values (SEVs), representing risk-weighted exposure prevalence, and theoretical minimum risk exposure levels (TMRELs) were estimated for each risk factor. These estimates were used to calculate the population attributable fraction (PAF; ie, the proportional change in health risk that would occur if exposure to a risk factor were reduced to the TMREL). The product of PAFs and disease burden associated with a given outcome, measured in disability-adjusted life-years (DALYs), yielded measures of attributable burden (ie, the proportion of total disease burden attributable to a particular risk factor or combination of risk factors). Adjustments for mediation were applied to account for relationships involving risk factors that act indirectly on outcomes via intermediate risks. Attributable burden estimates were stratified by Socio-demographic Index (SDI) quintile and presented as counts, age-standardised rates, and rankings. To complement estimates of RR and attributable burden, newly developed burden of proof risk function (BPRF) methods were applied to yield supplementary, conservative interpretations of risk–outcome associations based on the consistency of underlying evidence, accounting for unexplained heterogeneity between input data from different studies. Estimates reported represent the mean value across 500 draws from the estimate's distribution, with 95% uncertainty intervals (UIs) calculated as the 2·5th and 97·5th percentile values across the draws.

**Findings:**

Among the specific risk factors analysed for this study, particulate matter air pollution was the leading contributor to the global disease burden in 2021, contributing 8·0% (95% UI 6·7–9·4) of total DALYs, followed by high systolic blood pressure (SBP; 7·8% [6·4–9·2]), smoking (5·7% [4·7–6·8]), low birthweight and short gestation (5·6% [4·8–6·3]), and high fasting plasma glucose (FPG; 5·4% [4·8–6·0]). For younger demographics (ie, those aged 0–4 years and 5–14 years), risks such as low birthweight and short gestation and unsafe water, sanitation, and handwashing (WaSH) were among the leading risk factors, while for older age groups, metabolic risks such as high SBP, high body-mass index (BMI), high FPG, and high LDL cholesterol had a greater impact. From 2000 to 2021, there was an observable shift in global health challenges, marked by a decline in the number of all-age DALYs broadly attributable to behavioural risks (decrease of 20·7% [13·9–27·7]) and environmental and occupational risks (decrease of 22·0% [15·5–28·8]), coupled with a 49·4% (42·3–56·9) increase in DALYs attributable to metabolic risks, all reflecting ageing populations and changing lifestyles on a global scale. Age-standardised global DALY rates attributable to high BMI and high FPG rose considerably (15·7% [9·9–21·7] for high BMI and 7·9% [3·3–12·9] for high FPG) over this period, with exposure to these risks increasing annually at rates of 1·8% (1·6–1·9) for high BMI and 1·3% (1·1–1·5) for high FPG. By contrast, the global risk-attributable burden and exposure to many other risk factors declined, notably for risks such as child growth failure and unsafe water source, with age-standardised attributable DALYs decreasing by 71·5% (64·4–78·8) for child growth failure and 66·3% (60·2–72·0) for unsafe water source. We separated risk factors into three groups according to trajectory over time: those with a decreasing attributable burden, due largely to declining risk exposure (eg, diet high in trans-fat and household air pollution) but also to proportionally smaller child and youth populations (eg, child and maternal malnutrition); those for which the burden increased moderately in spite of declining risk exposure, due largely to population ageing (eg, smoking); and those for which the burden increased considerably due to both increasing risk exposure and population ageing (eg, ambient particulate matter air pollution, high BMI, high FPG, and high SBP).

**Interpretation:**

Substantial progress has been made in reducing the global disease burden attributable to a range of risk factors, particularly those related to maternal and child health, WaSH, and household air pollution. Maintaining efforts to minimise the impact of these risk factors, especially in low SDI locations, is necessary to sustain progress. Successes in moderating the smoking-related burden by reducing risk exposure highlight the need to advance policies that reduce exposure to other leading risk factors such as ambient particulate matter air pollution and high SBP. Troubling increases in high FPG, high BMI, and other risk factors related to obesity and metabolic syndrome indicate an urgent need to identify and implement interventions.

**Funding:**

Bill & Melinda Gates Foundation.

## Introduction

The COVID-19 pandemic revealed profound health disparities between individuals and across geographies.^1^ These differential impacts reflect a combination of multiple contributing risk factors affecting individuals and the varying capacities of health-care systems to protect and treat their populations. To strengthen the ability of health systems to meet future challenges, there is a need to focus on primary prevention.^2^, ^3^ In this context, and to regain momentum towards meeting UN Sustainable Development Goals,^1^, ^4^ identifying and quantifying the impact of key risk factors can help prioritise the use of scarce resources.

Targeting the reduction of modifiable risk factors is a powerful and essential strategy to prevent ill health and premature deaths due to diseases and injuries.^5^, ^6^, ^7^ Effective risk-reduction policies and practices are dependent on location-specific and population-specific information about relationships between risk factors and health outcomes, trends in the prevalence of leading risk factors, and the proportion of disease-specific mortality and morbidity that can be attributed to particular risk factors. Rigorous, well-sourced risk factor meta-analyses can highlight areas of public health progress, provide insight into persisting or emerging risks and consequent health challenges, and inform further modelling of plausible risk-factor reduction scenarios—including cost-effectiveness—to galvanise effective risk-reduction policies and practices. To produce these vital risk factor data, the Global Burden of Diseases, Injuries, and Risk Factors Study (GBD) has, since 1996, systematically estimated exposure to risk factors, relative health risk by exposure, and attributable disease burden for comprehensive sets of risk factors.^8^ For a specified set of causes of death and disability, attributable burden metrics are calculated to quantify the proportion of burden—measured in disability-adjusted life-years (DALYs), representing the sum of years of life lost to premature mortality and years lived with disability—that can be attributed to a particular risk factor or combination of risk factors. To improve estimation accuracy in response to an ever-changing global health landscape, successive iterations of GBD risk factor analyses have incorporated key methodological advancements and added emerging risk factors and health outcomes.^9^, ^10^, ^11^, ^12^, ^13^, ^14^ Other research efforts and networks of health scientists have contributed valuable global, multi-country, and population-level data on specific risk factors or groupings of risk factors; such endeavours include the NCD Risk Factor Collaboration (NCD-RisC), WHO's Noncommunicable Diseases Data Portal, and the Prospective Urban and Rural Epidemiological study (PURE).^15^, ^16^, ^17^, ^18^ However, only GBD produces systematic analyses of a comprehensive set of risk factors, identified on the basis of standardised data-driven criteria, in 204 countries and territories worldwide.

Here, we summarise GBD 2021 methods and present estimates of risk factor exposures and their relationships with health outcomes for 88 risk factors and combinations thereof included in the GBD 2021 hierarchical list of risk factors (appendix 1 table S1). Results are presented broadly within the Article and in more detail in appendix 2. Selected results are further accessible online through the Burden of Proof visualisation tool. This manuscript was produced as part of the GBD Collaborator Network and in accordance with the GBD Protocol.^19^

## Methods

### GBD overview

GBD publishes periodic updates, providing comprehensive estimates of risk exposure and risk-attributable health loss worldwide using all relevant available data. GBD 2021 estimated relevant metrics for 23 age groups from birth to age 95 years and older; for males, females, and all sexes combined; and for 204 countries and territories grouped into 21 regions and seven super-regions. GBD regions are made up of countries and territories that are geographically close and epidemiologically similar, and regions are grouped into super-regions on the basis of cause of death patterns.^20^ The seven super-regions are central Europe, eastern Europe, and central Asia; high income; Latin America and the Caribbean; north Africa and the Middle East; south Asia; southeast Asia, east Asia, and Oceania; and sub-Saharan Africa.^21^ GBD 2021 also includes subnational analyses for 21 countries and territories (see appendix 1 table S4 for the full GBD location hierarchy). Some results are presented stratified by Socio-demographic Index (SDI), a composite measure of lag-distributed income per capita, average years of education, and fertility rates among females younger than 25 years^22^ (appendix 1 table S5).

**Research in context**

**Evidence before this study**

The Global Burden of Diseases, Injuries, and Risk Factors Study (GBD) provides regularly updated estimates of risk factor exposure levels, relative health risk by exposure, and proportion of disease burden related to specific diseases or injuries that can be attributed to particular risk factors, categorised broadly into groups of environmental and occupational, behavioural, and metabolic risks. GBD has conducted analyses of risk-attributable burden since 1996, at which time ten risk factors were included in the analysis. GBD 2021 presents age-sex-location-year-specific estimates for 88 risk factors at global, regional, and national levels from 1990 through 2021. Although several research organisations and initiatives, including the NCD Risk Factor Collaboration (NCD-RisC) in partnership with WHO and the Prospective Urban and Rural Epidemiological (PURE) study, have provided valuable population-level evidence about specific risk factors or groupings in selected populations, GBD stands out for its systematic evaluation of the health effects of a comprehensive selection of risk factors across all countries and territories worldwide.

**Added value of this study**

GBD 2021 advances previous GBD estimates of risk factor exposure levels, relative risks (RRs), and the risk-attributable burden in several meaningful ways. RR estimates were systematically updated for risk–outcome pairs with burden of proof meta-regression methods that accounted for differences in exposure ranges in different comparison groups by integrating across the risk function and used an ensemble spline method to capture the (potentially non-linear) shape of the risk–outcome relationship from the data rather than imposing log-linearity. For 211 risk–outcome pairs, evidence of association was further quantified with burden of proof risk function (BPRF) analyses, which account for unexplained between-study heterogeneity in the input data, yielding a conservative interpretation of the risk–outcome association. For ease in interpreting and comparing BPRF measures across risk factors, summary risk–outcome scores were computed and mapped onto a star rating system (from one to five stars) summarising the relationships between risks and outcomes. Of the 211 risk–outcome pairs analysed with the BPRF methodology, 80 (37·9%) received a rating of three to five stars, indicating a well established (moderate to very strong) relationship between risk and outcome, based on a conservative interpretation of the available evidence, while 131 (62·1%) received one to two stars, suggesting that existing evidence for a robust relationship is weak. Additionally, mediation methods used to address risk–outcome relationships involving risk factors that act indirectly on outcomes via intermediate risks (eg, an association between low fruit consumption and heart disease mediated through systolic blood pressure [SBP]) were updated and systematised, resulting in a total of 158 mediated risk–outcome relationships. Nitrogen dioxide air pollution was added as a new risk factor, which resulted in the addition of one associated risk–outcome pair: nitrogen dioxide air pollution–asthma. 117 additional risk–outcome pairs were incorporated for risk factors already included in the study, based on new evidence, more detailed specification of outcomes, or refinements to mediation factors. Conversely, 25 risk–outcome pairs were excluded from GBD 2021 because they no longer met inclusion criteria. New or updated systematic reviews were conducted, as detailed in appendix 1 (section 2.1.3). Theoretical minimum risk exposure levels (TMRELs) were revised for 19 risk factors.

**Implications of all the available evidence**

We highlight three ways to interpret the GBD 2021 risk factor results to provide useful policy perspectives. The first approach is to emphasise the overall contribution of a given risk factor to disease burden. From this perspective, particulate matter air pollution, high SBP, smoking, low birthweight and short gestation, and high fasting plasma glucose (FPG) were associated with the highest levels of global death and disability in 2021. Strategies that address these risk factors will reduce the overall burden. A second approach, examining trends over time in the risk-attributable burden, reveals that although the risk exposure and burden attributable to many risk factors—such as child growth failure and unsafe water and sanitation—have decreased, there have also been increases for numerous other risk factors, including ambient particulate matter pollution, high SBP, high body-mass index (BMI), and high FPG. These findings suggest areas of concern and interactions between risk factor exposure and demographics. The third method—one that is new for GBD 2021—is to use BPRF methods to identify risk factors that contribute substantially to the overall burden and whose associated risk–outcome relationships are supported by strongly compelling evidence, in order to provide policy makers with a more informed approach to risk mitigation. Such risks include particulate matter air pollution, high SBP, smoking, high LDL cholesterol, high FPG, high alcohol use, kidney dysfunction, child growth failure, and, to a lesser extent, high BMI. These three complementary perspectives on our results can provide key audiences, including policy makers, health-care professionals, and the general public, with crucial information to effectively reduce disease burden.

The GBD 2021 analytical framework for risk factors generated estimates for the period 1990–2021. GBD has included an analysis of risk-attributable burden since 1996,^23^ with initial estimates published in WHO's annual World Health Reports^24^, ^25^, ^26^ and WHO GBD updates,^27^, ^28^ and estimates from 2010 onwards published in *The Lancet.*^9^, ^10^, ^11^, ^12^, ^13^, ^14^ An international GBD Collaborator Network provides, reviews, and analyses the available data to generate these metrics, with the GBD 2021 round drawing on the expertise of more than 11 000 collaborators in more than 160 countries and territories. In each iteration of GBD, newly available data and improved methods are used to update the full time series of estimates from 1990 through the latest year of analysis. GBD 2021 estimates for the entire 1990–2021 time series therefore supersede all previously published estimates.

### GBD risk factor hierarchy

GBD classifies all GBD risk factors into a risk factor hierarchy with four levels, plus an overarching aggregate of all risk factors combined. At Level 1, risk factors are categorised as environmental and occupational, behavioural, and metabolic risks. These Level 1 categories are disaggregated at Level 2 into 20 risk factors or clusters of risk factors (eg, dietary risks and air pollution). At Level 3, nine of the Level 2 risks are further broken down into 42 additional risk factors or clusters of risks; Level 3 also includes the 11 Level 2 risks that are not further disaggregated. At Level 4—the most granular level—five of the Level 3 risks are further disaggregated into 22 additional specific risk factors; Level 4 also includes the 11 Level 2 risks that were not disaggregated at Level 3 and 37 Level 3 risks not further disaggregated at Level 4. This hierarchy allows for evaluation of individual risk factors, such as low birthweight, as well as groups of risk factors that are of policy interest, such as child and maternal malnutrition or behavioural risks. In total, GBD 2021 covers 88 total risks (one aggregation of all risks combined plus three Level 1 risks plus 20 Level 2 risks plus 42 additional Level 3 risks plus 22 additional Level 4 risks), including one Level 3 risk factor being reported in GBD for the first time: nitrogen dioxide, an additional air pollution measure strongly influenced by motor vehicle emissions.^29^ See appendix 1 (table S1) for the full 2021 GBD risk factor hierarchy, along with appendix 1 (section 6) and the Methods Web Portal for risk factor-specific definitions and modelling details.

### Data sources

To generate relative risk (RR) estimates for risk–outcome pairs, GBD synthesises data from primary randomised controlled trials and cohort, pooled cohort, or case–control studies that report RRs of mortality or morbidity from a given health outcome as a function of risk exposure, in addition to meta-analyses summarising RRs (appendix 1 section 2.1.3). These data were obtained through systematic reviews, including updates of reviews conducted for past GBD cycles and new systematic reviews for risk factors such as diet high in unprocessed red meat, smoking, and diet low in vegetables.^30^, ^31^, ^32^ 3359 distinct data sources from 124 countries were used in the estimation of RRs, 1176 of which were new for GBD 2021, supplementing those previously included in GBD 2019. To estimate mean exposure for each risk factor, systematic literature reviews were conducted to identify risk factor exposure studies published or identified since GBD 2019, and were combined with data from other sources, including household and health examination surveys and censuses, ground-sensing or remote-sensing data, and administrative records. 51 337 distinct data sources from 204 countries and territories were used in estimating risk exposure, 14 252 of which were new, in addition to those previously included in GBD 2019. In total, the GBD 2021 risk factor analysis used 54 561 distinct data sources, which includes a small number of sources used to estimate both relative risk and risk exposure.

Available data sources for estimating RRs and exposure varied across risk factors; input data were highly heterogeneous, and quality varied across geography and time. See appendix 1 (section 2.1.3) for systematic review and bias assessment guidelines, and appendix 1 (section 6) for risk factor-specific details about data collection methods, systematic reviews, search strategies, data sources, bias assessment, and citations. The effort to systematically synthesise substantial quantities of heterogeneous data for large numbers of risk–outcome pairs in a comparable manner is ongoing, and protocols for performing systematic reviews and extracting and processing data will continue to be updated and integrated into methods in future GBD rounds. Detailed information on data sources used for risk factor estimation in GBD 2021 is also available online via the GBD 2021 Sources Tool in the Global Health Data Exchange (GHDx).

### Risk factor estimation

For GBD 2021, we estimated relationships between 88 risk factors and selected health outcomes—comprising 155 outcomes across risk factors—for a total of 631 risk–outcome pairs analysed. Notably, the present analysis did not formally incorporate or quantify the impact of the COVID-19 pandemic across risk factors or health outcomes due to data limitations. GBD 2021 produced risk-specific estimates of summary exposure value (SEV), RR, population attributable fraction (PAF), risk-attributable burden measured in disability-adjusted life-years (DALYs; the sum of years of life lost to premature mortality and years lived with disability),^33^ and deaths.^14^ Furthermore, a new method was introduced to complement RR estimates: burden of proof risk function (BPRF) analyses that account for unexplained between-study heterogeneity in RR input data and yield an additional, conservative interpretation of the risk–outcome association and its underlying input evidence.^34^ The methods employed to generate the measures from past GBD rounds closely followed those used for GBD 2019^14^ and have been extensively peer-reviewed over previous GBD rounds^9^, ^10^, ^11^, ^12^, ^13^, ^14^ and concurrently as part of the peer review process for GBD 2021. Here, we provide a methodological overview with an emphasis on the main changes since GBD 2019. A more comprehensive description of the analytical methods for GBD 2021 is provided in appendix 1, with extensive source details for input data available online via the GBD 2021 Sources Tool in the GHDx. Each of these materials was included in the peer review process of the present Article.

Our analysis was based on the comparative risk assessment (CRA) framework (appendix 1 table S2) established to compute risk factor estimates^8^, ^35^ and included seven primary inter-related methodological components. The first step entailed estimating effect size by quantifying the RR of the specified health outcome occurring as a function of exposure to the specified risk factor (appendix 1 section 2 step 1). Estimates were generated for risk–outcome pairs already included in GBD 2019 (based on convincing or probable evidence of an association assessed following World Cancer Research Fund methods and criteria^36^) and new pairs considered candidates for inclusion (based on informed judgements by GBD Collaborators and other subject experts on potential importance to disease burden or policy, in addition to sufficient data and appropriate methods to estimate key metrics) that met inclusionary criteria, described below (appendix 1 section 2.1.1). In our standard analytical process, the primary tool used to estimate RRs was meta-regression in the burden of proof approach,^34^, ^37^, ^38^ which was used to synthesise data identified and extracted through systematic reviews conducted for each risk–outcome pair in accordance with the Preferred Reporting Items for Systematic Reviews and Meta-Analyses (PRISMA) framework.^39^ Guidelines about systematic reviews and bias assessment are provided in appendix 1 (section 2.1.3), with risk factor-specific information—including details about data sources, systematic reviews, data extraction, and modelling strategies—provided in appendix 1 (section 6) and in the Methods Web Portal (cited earlier). The burden of proof approach relies on an ensemble spline method to capture the (potentially non-linear) shape of the RR function from the data rather than imposing a log-linear relationship. The approach also incorporates differences in exposure ranges for different comparison groups by integrating across the RR function, tests and adjusts for systematic biases to account for identified heterogeneity across input study designs and characteristics, and trims potentially distorting outliers in the input data.^37^ Methodological details about splines, knot placement, monotonicity constraints, trimming strategies, and bias adjustment are provided in appendix 1 (section 2.1.4). RR estimates provide the basis for including new risk–outcome pairs in GBD 2021. Inclusion criteria defined by the GBD Scientific Council state that the RR estimate's 95% uncertainty interval (UI), conventionally calculated, without accounting for unexplained between-study heterogeneity, must not cross the null RR value of 1 (ie, the mean RR estimate must be significantly higher [for harmful risks] or lower [for protective risks] than 1) for a risk–outcome pair to be included in GBD. On this basis, 118 new risk–outcome pairs were included in GBD 2021, for a total of 631 pairs. To maintain stability in included risk factors and risk–outcome pairs between GBD cycles, exclusion criteria for those pairs already included in GBD 2019 were less stringent; previously included pairs were excluded only if the conventionally calculated 90% UI crossed the null. On this basis, 25 risk–outcome pairs were dropped from GBD 2021. See appendix 1 (table S7) for a list of risk–outcome pairs included in GBD 2021 and details of pairs added or dropped since GBD 2019. New to GBD 2021, the burden of proof approach also evaluated potential publication or reporting bias (appendix 1 section 2.1.7) and quantified unexplained between-study heterogeneity (appendix 1 sections 2.1.5). Between-study heterogeneity was incorporated into estimates of uncertainty and used to generate BPRFs to complement mean RRs derived through our standard analytical process. BPRF metrics (ie, risk–outcome scores and star ratings) provide an additional, conservative interpretation of the risk–outcome effect and the consistency of underlying evidence (detailed below and in appendix 1 section 2.1.6).

The second step consisted of collecting exposure data and estimating the levels and distribution of exposure to each risk factor, primarily using two Bayesian statistical models (spatiotemporal Gaussian process regression [ST-GPR] and disease model meta-regression [DisMod-MR 2.1]^14^, ^33^) to pool heterogeneous data and to control and adjust for bias (appendix 1 section 2, step 2, and section 6). The third step involved determining theoretical minimum risk exposure levels (TMRELs; the counterfactual level of exposure that would minimise health risk) on the basis of epidemiological evidence^14^ (appendix 1 section 2 step 3). In a fourth step, estimates of PAFs,^14^ quantifying the proportional change in health that would occur if risk exposure was reduced to the TMREL, were independently computed for each risk–outcome pair with estimates of exposure, RR, and the TMREL (appendix 1 section 2 step 4). Fifth, SEVs,^14^ representing the age-specific risk-weighted prevalence of exposure, were calculated for each risk. SEVs are reported on a 0 to 100 scale, where 0 equates to a scenario in which the entire population (in age groups included in the evaluation, eg, those aged 0–27 days for low birthweight) is exposed at the TMREL, and 100 indicates that the entire population is exposed at the maximum risk exposure level (appendix 1 section 2 step 5). Sixth, because some risk factors affect other risks that lie on the physiological pathway to an outcome, mediation factors were estimated and used to correct for PAF overestimation if independence between risk factors was assumed and to compute the burden attributable to combinations of risk factors (appendix 1 section 2 step 6; table S6 presents the full mediation matrix). Finally, estimates of attributable burden (ie, the proportion of disease burden attributable to the risk factor, as quantified by the product of the PAF and the DALYs or deaths associated with the outcome) were calculated for each combination of age group, sex, location, and year (appendix 1 section 2 step 7). The majority of risk–outcome pairs were evaluated with this standard set of analytical processes. For some pairs, other methods were used as dictated by the evidence available for those risks (appendix 1 section 2 step 1 and section 6). For example, non-optimal temperature RR estimation and TMREL identification was conducted through primary analysis of the relationship between temperature and cause-specific mortality.^40^ For some risk–outcome pairs, PAFs were assumed by definition to be 100% (eg, 100% of diabetes is assumed to be, by definition, related to high fasting plasma glucose [FPG]). For other pairs in which the outcome is specific to a risk factor (eg, mesothelioma and occupational exposure to asbestos), direct PAFs were used, calculated directly from the disease rather than based on an RR estimate generated with the standard set of analytical processes (appendix 2 table 6).

Methodological improvements for estimating risk exposure and risk-attributable burden in the current GBD round focused on standardisation of RR estimation as described above and application of new BPRF methods to generate conservative assessments of risk–outcome relationships and their underlying evidence incorporating between-study heterogeneity; improved specification of the mediation matrix; and re-evaluation of TMRELs with meta-regression or other methods to incorporate new data, resulting in revised TMREL values for 19 risk factors—primarily dietary risks and high systolic blood pressure (SBP), high LDL cholesterol, and high body-mass index (BMI; see appendix 1 table S9 for changes to 2019 TMREL values). Details of these improvements are provided below or in appendix 1 (section 2).

### New for GBD 2021

#### Updates to the mediation matrix

To more fully and accurately account for mediated relationships involving distal risk factors that act indirectly on outcomes via intermediate risks (eg, an association between low fruit consumption and heart disease mediated through SBP), we reviewed and expanded the methods and evidence forming the basis of the GBD mediation matrix (appendix 1 table S6). A set of consistent rule-based inclusionary and exclusionary criteria were formalised and applied. First, a distal risk cannot be mediated by more than 100% through multiple mediators to the same outcome. Second, the full set of distal risks acting through a specific mediator should be applied to every outcome related to that mediator for all distal-mediator–outcome pathways previously included in GBD 2019 and new pathways that rated a three-star relationship or higher in the BPRF star rating system (exceptions to this included some pathways with smoking as a distal risk, and high FPG or high SBP as mediators). Last, outcomes previously absent from the mediation matrix in which a mediator has a direct causal effect in GBD should be added to the matrix (eg, chronic kidney disease due to diabetes was added as a mediated outcome for high FPG). Application of these criteria resulted in the addition of 87 new mediated risk–outcome pairs and the removal of 64 pairs previously in the matrix, resulting in a total of 158 pairs in the 2021 mediation matrix (appendix 1 table S8). See appendix 1 (section 2 step 6) for further details about GBD 2021 mediation methods. Specification of the matrix is ongoing and will be further updated for future GBD rounds.

#### Burden of proof risk function and star ratings

To complement our standard estimates of risk–outcome relationships, we further applied BPRF methods introduced by Zheng and colleagues^34^ that generate alternative metrics combining effect size and consistency of evidence. The motivation behind this methodology is to highlight risk factors for which the currently available data suggest there is either or both a large effect on health outcomes (and potentially high attributable burden) and robust evidence for the effect, in addition to risk factors that show large effects on outcomes but for which the evidence is less consistent, underscoring a need for additional research. For GBD 2021, BPRFs were generated for 211 risk–outcome pairs (ie, for most metabolic risks; all environmental but no occupational risks; and some behavioural risks such as dietary risks and high alcohol use; see appendix 2 table S6) to complement conventional estimates of RR used to calculate PAFs and attributable burden.

The BPRF is related to the mean RR relationship between exposure and health outcome, relying on 95% UIs inclusive of heterogeneity across estimates of effect from individual studies not accounted for by study design covariates (eg, confounding, selection bias, and exposure measurement; appendix 1 section 2.1.5).^41^ These 95% UIs are used to derive the BPRF, defined for harmful risks as the 5th quantile risk curve closest to null and for protective risks as the 95th quantile risk curve closest to null (RR=1; the function representing a relationship in which a change in risk exposure has no effect on health outcome). The BPRF therefore represents a conservative estimate, consistent with the available evidence, of the change in health outcome at each level of risk exposure. BPRF estimates are used to compute the risk–outcome score, defined as the signed value of the average log BPRF between the 15th and 85th percentiles of risk exposure levels observed across included studies.^34^ A higher positive risk–outcome score corresponds to either or both a greater average effect size (as represented by RRs) and stronger, more consistent evidence (as reflected in narrower 95% UIs), less distorted by spurious confounders or bias, for the specific risk–outcome relationship. For ease of interpretation and comparability across risk–outcome pairs, risk–outcome scores are mapped onto a star rating system (table 1; see appendix 2 table S6 for risk–outcome scores and star ratings for all risk–outcome pairs analysed using BPRF methods). All risk–outcome pairs receiving a one-star to five-star rating are eligible for inclusion in GBD. Application of the BPRF methodology might in some cases lead to 95% UIs including negative attributable burden estimates (eg, lower 95% UI <1) for one-star pairs; this is a result of values for the RR less than 1 in the 95% UIs, a consequence of including between-study heterogeneity in RR estimates. In these cases, the uncertainty includes the possibility of no effect or even protective effects of the exposure on the outcome. Although there might be biological plausibility for the protective effects for some risk factors (eg, metabolic and dietary), this is less likely for others (eg, air pollution and tobacco). In these cases, wide uncertainty suggests poorly understood or weak risk–outcome relationships. We report the full uncertainty distribution for transparency.

**Table 1:** BPRF risk–outcome score ranges associated with each star rating and number of risk–outcome pairs assigned to each star rating

	**Harmful: percentage increase in risk of outcome in those exposed**	**Protective: percentage decrease in risk of outcome in those exposed**	**Risk–outcome score range**	**Number of risk–outcome pairs (n=211)**
One star	0%	0%	<0·00	52
Two stars	0% to 15%	0% to 13%	0·00 to 0·14	79
Three stars	>15% to 50%	>13% to 34%	>0·14 to 0·41	55
Four stars	>50% to 85%	>34% to 46%	>0·41 to 0·62	13
Five stars	>85%	>46%	>0·62	12

BPRF=burden of proof risk function. BPRF refers to the most conservative estimate of the magnitude of the increase in risk (for harmful risk factors) or decrease in risk (for protective risk factors) of the specified outcome with exposure to the specified risk factor.

The BPRF methodology provides a structured analytical framework applied across the diversity of GBD risk factors to evaluate effect size and consistency across the underlying data. Although our core results are presented for all included risk–outcome pairs, BPRF metrics also allowed us to highlight risk factors with the strongest evidence of disease burden by re-calculating attributable burden estimates for three-star, four-star, and five-star risk–outcome pairs only. For further details on BPRF methods, see appendix 1 (sections 2.1.5 and 2.1.6), the paper by Zheng and colleagues 2022,^34^ and other publications associated with the methodology.^38^, ^42^ Development of BPRF methods and their application to GBD risk factor analyses are ongoing and will continue to be refined in future GBD rounds.

### Presentation of estimates

Risk-attributable burden estimates for 2021 are given as counts and age-standardised rates per 100 000 population, calculated with the GBD standard population structure to account for variation in age structures across populations.^22^ SEVs are given as age-standardised rates on a 0–100 scale. For changes over time, we present percentage changes during 2000–21 (see appendix 2 table S1 and table S3 for estimates for 1990–2021) and report annualised rates of change (ARCs) as the difference in the natural log of the values at the start and end of the time interval divided by the number of years in the interval. Estimates for all metrics are computed with the mean estimate across 500 draws, and 95% UIs are given as the 2·5th and 97·5th percentiles of that distribution. To reduce computing power and time, the number of computations per process was reduced from 1000 in previous GBD iterations to 500 for GBD 2021 based on simulations that revealed that estimates and uncertainty were not affected by this reduction.

### GBD research and reporting practices

GBD 2021 complies with the Guidelines for Accurate and Transparent Health Estimates Reporting (GATHER) statement (appendix 1 table S3).^43^ Analyses were completed with Python (version 3.10.4), Stata (version 13.1), and R (version 4.2.1). The statistical code used for GBD estimation is publicly available online.

### Role of the funding source

The funder of this study had no role in study design, data collection, data analysis, data interpretation, the writing of the report, or the decision to submit the manuscript for publication.

## Results

### Overview

Detailed estimates are available in appendix 2, which provides supplementary figures and links to tables in downloadable form through the Global Health Data exchange. All risk-related estimates are also available in searchable and downloadable form through the GBD Results tool and via visual exploration through the online tool GBD Compare and the Burden of Proof visualisation tool. Two-page summaries of results for each risk factor included in the analysis are also available online.

### Summary exposure values (SEVs)

Quantifying risk exposure with age-standardised SEVs, which account for both the severity and proportion of the population exposed and are comparable across risks with different patterns of exposure, global Level 2 risk exposure was highest in 2021 for high LDL cholesterol (SEV 45·3 [95% UI 30·7–63·1] on a 0–100 scale), dietary risks (37·6 [28·1–47·8]), air pollution (36·6 [29·6–45·1]), and high SBP (35·6 [25·9–47·0]; table 2). Disaggregated to Level 3 of the risk hierarchy, SEVs were highest for a variety of dietary factors, most notably diet low in omega-6 polyunsaturated fatty acids (75·6 [44·0–87·3]) and diet low in milk (64·9 [62·9–73·8]).

**Table 2:** Global age-standardised SEVs in 1990, 2000, 2010, and 2021, and annualised rate of change over 1990–2021, 2000–21, and 2010–21, by GBD risk factor

				**SEV 1990**	**SEV 2000**	**SEV 2010**	**SEV 2021**	**Annualised rate of change 1990 to 2021 (%)**	**Annualised rate of change 2000 to 2021 (%)**	**Annualised rate of change 2010 to 2021 (%)**
**All risk factors**				**29·4 (27·7 to 31·3)**	**28·8 (27·1 to 30·8)**	**28·1 (26·1 to 30·1)**	**27·5 (25·6 to 29·5)**	**–0·2% (−0·3 to −0·1)**	**–0·2% (−0·4 to −0·1)**	**–0·2% (−0·4 to −0·1)**
**Environmental and occupational risks**				**43·2 (37·8 to 46·8)**	**41·4 (36·1 to 45·2)**	**39·1 (33·6 to 43·2)**	**35·6 (30·2 to 39·9)**	**–0·6% (−0·8 to −0·5)**	**–0·7% (−0·9 to −0·5)**	**–0·8% (−1·0 to −0·7)**
	Unsafe water, sanitation, and handwashing			43·8 (29·2 to 49·5)	38·5 (24·7 to 44·5)	34·2 (21·0 to 40·0)	29·8 (17·3 to 35·3)	−1·2% (−1·7 to −1·0)	−1·2% (−1·7 to −0·9)	−1·3% (−1·9 to −0·8)
		Unsafe water source		44·8 (33·7 to 56·7)	41·0 (28·8 to 55·0)	38·4 (25·5 to 54·0)	35·4 (22·3 to 50·5)	−0·8% (−1·4 to −0·3)	−0·7% (−1·4 to −0·2)	−0·7% (−1·5 to −0·2)
		Unsafe sanitation		57·0 (54·4 to 59·6)	49·2 (46·6 to 52·2)	40·8 (38·0 to 44·0)	32·3 (29·4 to 35·3)	−1·8% (−2·0 to −1·6)	−2·0% (−2·3 to −1·8)	−2·1% (−2·4 to −1·8)
		No access to handwashing facility		31·5 (16·1 to 35·6)	28·9 (14·5 to 33·0)	26·8 (13·5 to 31·0)	24·1 (12·0 to 27·9)	−0·9% (−1·2 to −0·5)	−0·9% (−1·3 to −0·4)	−1·0% (−1·6 to −0·3)
	Air pollution			48·5 (41·0 to 56·6)	46·3 (38·9 to 54·1)	41·7 (34·1 to 50·1)	36·6 (29·6 to 45·1)	−0·9% (−1·1 to −0·7)	−1·1% (−1·4 to −0·9)	−1·2% (−1·5 to −1·0)
		Particulate matter pollution		53·1 (46·0 to 61·0)	50·6 (43·9 to 58·2)	45·0 (38·0 to 53·3)	39·6 (33·1 to 47·5)	−0·9% (−1·2 to −0·7)	−1·2% (−1·4 to −0·9)	−1·2% (−1·4 to −0·9)
			Ambient particulate matter pollution	19·9 (14·0 to 26·6)	21·8 (15·7 to 28·4)	24·6 (17·1 to 31·8)	28·4 (19·4 to 35·6)	1·1% (0·5 to 1·7)	1·3% (0·6 to 1·9)	1·3% (0·8 to 1·8)
			Household air pollution from solid fuels	33·9 (24·7 to 43·5)	30·2 (21·8 to 39·8)	24·1 (15·7 to 34·9)	17·3 (10·2 to 28·6)	−2·2% (−2·9 to −1·4)	−2·7% (−3·6 to −1·6)	−3·0% (−3·9 to −1·9)
		Ambient ozone pollution		16·4 (14·1 to 20·0)	18·4 (15·9 to 22·2)	20·7 (18·1 to 24·9)	23·8 (21·1 to 28·0)	1·2% (1·1 to 1·3)	1·2% (1·1 to 1·4)	1·3% (1·1 to 1·4)
		Ambient nitrogen dioxide pollution		19·5 (0·0 to 47·4)	18·0 (0·0 to 45·9)	18·4 (0·0 to 47·3)	15·0 (0·0 to 42·8)	−0·8% (−2·5 to 0·0)	−0·9% (−2·7 to 0·0)	−1·9% (−4·8 to 0·0)
	Non-optimal temperature			30·1 (26·0 to 34·8)	31·0 (27·0 to 35·6)	35·1 (30·7 to 40·1)	32·3 (28·1 to 37·0)	0·2% (0·1 to 0·3)	0·2% (0·1 to 0·3)	−0·8% (−0·9 to −0·6)
		High temperature		31·7 (26·0 to 37·5)	33·1 (27·6 to 39·0)	41·2 (35·1 to 47·1)	37·3 (31·5 to 43·3)	0·5% (0·4 to 0·7)	0·6% (0·4 to 0·7)	−0·9% (−1·1 to −0·7)
		Low temperature		24·9 (22·8 to 27·8)	25·8 (23·9 to 28·5)	25·5 (23·7 to 28·1)	24·2 (22·3 to 26·7)	−0·1% (−0·2 to 0·0)	−0·3% (−0·4 to −0·2)	−0·5% (−0·6 to −0·4)
	Other environmental risks			39·7 (8·8 to 47·3)	40·0 (8·9 to 47·8)	38·1 (8·8 to 45·6)	33·8 (8·5 to 40·8)	−0·5% (−0·7 to −0·1)	−0·8% (−1·1 to −0·1)	−1·1% (−1·4 to −0·1)
			Residential radon	24·5 (0·0 to 36·7)	24·2 (0·0 to 36·0)	24·0 (0·0 to 35·7)	23·8 (0·0 to 35·3)	−0·1% (−0·3 to 0·1)	−0·1% (−0·2 to 0·1)	−0·1% (−0·3 to 0·1)
			Lead exposure	46·7 (0·0 to 55·4)	47·2 (0·0 to 56·1)	44·5 (0·0 to 52·6)	38·2 (0·0 to 45·1)	−0·6% (−1·6 to −0·5)	−1·0% (−1·2 to −0·8)	−1·4% (−1·6 to 0·1)
		Occupational risks		3·6 (3·2 to 4·1)	3·7 (3·3 to 4·2)	3·7 (3·3 to 4·2)	3·6 (3·2 to 4·1)	0·0% (−0·2 to 0·1)	−0·2% (−0·3 to 0·0)	−0·3% (−0·5 to 0·0)
			Occupational carcinogens	0·9 (0·7 to 1·5)	1·0 (0·8 to 1·6)	1·1 (0·8 to 1·7)	1·1 (0·8 to 1·7)	0·6% (0·5 to 0·7)	0·5% (0·4 to 0·6)	0·4% (0·2 to 0·6)
			Occupational exposure to asbestos	2·3 (2·2 to 2·5)	2·2 (2·1 to 2·3)	2·3 (2·1 to 2·4)	2·0 (1·8 to 2·1)	−0·4% (−0·7 to −0·3)	−0·5% (−0·7 to −0·3)	−1·1% (−1·4 to −0·7)
			Occupational exposure to arsenic	0·4 (0·1 to 0·9)	0·5 (0·1 to 0·9)	0·5 (0·1 to 0·9)	0·5 (0·1 to 0·9)	0·3% (0·1 to 0·9)	0·2% (0·0 to 0·7)	0·1% (−0·1 to 0·6)
			Occupational exposure to benzene	0·8 (0·3 to 1·7)	0·8 (0·4 to 1·8)	0·9 (0·4 to 1·9)	1·0 (0·5 to 2·0)	0·8% (0·6 to 1·1)	0·8% (0·6 to 1·0)	0·7% (0·5 to 1·0)
			Occupational exposure to beryllium	0·1 (0·1 to 0·1)	0·1 (0·1 to 0·1)	0·1 (0·1 to 0·1)	0·1 (0·1 to 0·1)	0·5% (0·5 to 0·5)	0·3% (0·3 to 0·4)	0·3% (0·2 to 0·4)
			Occupational exposure to cadmium	0·2 (0·2 to 0·2)	0·2 (0·2 to 0·2)	0·2 (0·2 to 0·2)	0·2 (0·2 to 0·2)	0·7% (0·6 to 0·9)	0·6% (0·4 to 0·8)	0·5% (0·2 to 0·7)
			Occupational exposure to chromium	0·4 (0·4 to 0·4)	0·4 (0·4 to 0·4)	0·5 (0·4 to 0·5)	0·5 (0·5 to 0·5)	1·0% (0·9 to 1·1)	0·9% (0·7 to 1·1)	0·7% (0·5 to 1·0)
			Occupational exposure to diesel engine exhaust	1·7 (1·7 to 1·7)	2·0 (1·9 to 2·0)	2·3 (2·2 to 2·3)	2·6 (2·5 to 2·6)	1·3% (1·2 to 1·4)	1·3% (1·2 to 1·4)	1·2% (1·0 to 1·4)
			Occupational exposure to formaldehyde	0·8 (0·7 to 0·8)	0·9 (0·8 to 0·9)	0·9 (0·9 to 1·0)	1·0 (0·9 to 1·0)	0·8% (0·7 to 0·9)	0·6% (0·4 to 0·8)	0·5% (0·2 to 0·8)
			Occupational exposure to nickel	0·4 (0·1 to 1·3)	0·5 (0·1 to 1·3)	0·5 (0·1 to 1·3)	0·5 (0·1 to 1·3)	0·2% (0·0 to 0·8)	0·2% (−0·1 to 0·7)	0·1% (−0·2 to 0·6)
			Occupational exposure to polycyclic aromatic hydrocarbons	0·7 (0·7 to 0·7)	0·8 (0·8 to 0·9)	0·9 (0·9 to 1·0)	1·0 (1·0 to 1·0)	1·0% (0·9 to 1·1)	0·9% (0·7 to 1·1)	0·8% (0·5 to 1·0)
			Occupational exposure to silica	4·2 (1·7 to 10·7)	4·3 (1·9 to 10·7)	4·5 (2·1 to 11·0)	4·6 (2·2 to 11·2)	0·3% (0·1 to 0·7)	0·4% (0·2 to 0·7)	0·3% (0·1 to 0·6)
			Occupational exposure to sulphuric acid	1·0 (0·6 to 2·1)	1·0 (0·6 to 2·2)	1·0 (0·7 to 2·1)	1·0 (0·7 to 2·1)	0·2% (0·0 to 0·5)	0·0% (−0·2 to 0·3)	0·0% (−0·3 to 0·3)
			Occupational exposure to trichloroethylene	0·2 (0·2 to 0·2)	0·2 (0·2 to 0·2)	0·3 (0·3 to 0·3)	0·3 (0·3 to 0·3)	1·0% (1·0 to 1·1)	0·9% (0·8 to 1·0)	0·8% (0·6 to 1·0)
		Occupational asthmagens		17·9 (15·5 to 20·9)	18·3 (15·9 to 21·5)	18·1 (15·8 to 20·9)	17·6 (15·5 to 20·2)	−0·1% (−0·2 to 0·1)	−0·2% (−0·4 to 0·0)	−0·2% (−0·6 to 0·1)
		Occupational particulate matter, gases, and fumes		10·4 (8·4 to 12·8)	10·5 (8·6 to 12·9)	10·4 (8·5 to 12·7)	9·9 (8·2 to 12·0)	−0·1% (−0·2 to −0·1)	−0·3% (−0·4 to −0·2)	−0·4% (−0·5 to −0·3)
		Occupational noise		10·6 (10·2 to 11·2)	10·8 (10·4 to 11·4)	10·9 (10·5 to 11·5)	10·8 (10·4 to 11·3)	0·0% (0·0 to 0·1)	0·0% (−0·1 to 0·0)	−0·1% (−0·2 to −0·1)
		Occupational injuries		..	..	..	..	..	..	..
		Occupational ergonomic factors		20·3 (19·0 to 21·8)	20·0 (18·9 to 21·4)	18·3 (16·9 to 19·7)	16·3 (15·0 to 17·8)	−0·7% (−0·9 to −0·5)	−1·0% (−1·3 to −0·7)	−1·1% (−1·5 to −0·6)
**Behavioural risks**				**22·3 (20·6 to 24·5)**	**21·1 (19·5 to 23·3)**	**19·9 (18·2 to 22·2)**	**18·6 (17·0 to 20·7)**	**–0·6% (−0·6 to −0·5)**	**–0·6% (−0·7 to −0·5)**	**–0·6% (−0·7 to −0·5)**
	Child and maternal malnutrition			12·5 (8·4 to 17·9)	12·2 (8·3 to 17·2)	11·7 (7·9 to 16·6)	11·6 (7·7 to 16·6)	−0·3% (−0·3 to −0·2)	−0·2% (−0·4 to −0·1)	−0·1% (−0·3 to 0·0)
		Suboptimal breastfeeding		38·4 (34·9 to 42·8)	35·1 (31·8 to 39·2)	33·8 (30·5 to 37·5)	32·6 (29·8 to 36·1)	−0·5% (−0·6 to −0·5)	−0·4% (−0·4 to −0·3)	−0·3% (−0·5 to −0·2)
			Non-exclusive breastfeeding	43·5 (30·6 to 59·3)	40·7 (28·8 to 55·5)	38·7 (27·5 to 53·0)	35·9 (26·1 to 48·6)	−0·6% (−0·7 to −0·5)	−0·6% (−0·7 to −0·5)	−0·7% (−0·9 to −0·5)
			Discontinued breastfeeding	41·4 (40·5 to 42·4)	37·2 (36·5 to 38·0)	35·8 (35·2 to 36·6)	35·3 (34·5 to 36·2)	−0·5% (−0·6 to −0·4)	−0·2% (−0·3 to −0·1)	−0·1% (−0·3 to 0·0)
		Child growth failure		13·1 (9·0 to 20·1)	12·4 (8·5 to 19·1)	10·3 (6·8 to 16·3)	7·6 (4·6 to 12·9)	−1·7% (−2·2 to −1·4)	−2·3% (−3·0 to −1·8)	−2·7% (−3·6 to −2·0)
			Child underweight	19·8 (15·6 to 23·4)	19·1 (15·2 to 22·6)	16·5 (13·0 to 19·6)	13·6 (10·6 to 16·3)	−1·2% (−1·3 to −1·2)	−1·6% (−1·8 to −1·5)	−1·8% (−1·9 to −1·7)
			Child wasting	6·8 (4·5 to 8·5)	6·6 (4·3 to 8·2)	5·9 (3·9 to 7·5)	4·5 (3·0 to 5·9)	−1·3% (−1·5 to −1·2)	−1·8% (−2·0 to −1·6)	−2·5% (−2·8 to −2·1)
			Child stunting	24·7 (21·9 to 26·7)	23·5 (20·8 to 25·4)	20·2 (18·0 to 21·9)	16·2 (14·7 to 17·7)	−1·4% (−1·4 to −1·2)	−1·8% (−1·9 to −1·6)	−2·0% (−2·2 to −1·7)
		Low birthweight and short gestation		22·2 (20·0 to 24·5)	23·4 (21·1 to 25·8)	23·1 (20·9 to 25·5)	22·9 (20·7 to 25·3)	0·1% (0·1 to 0·1)	−0·1% (−0·2 to 0·0)	−0·1% (−0·2 to 0·0)
			Short gestation	32·5 (29·5 to 34·9)	33·7 (30·6 to 36·3)	33·0 (30·2 to 35·4)	32·5 (29·7 to 34·9)	0·0% (−0·1 to 0·1)	−0·2% (−0·2 to −0·1)	−0·1% (−0·2 to 0·0)
			Low birthweight	18·0 (17·0 to 19·0)	18·9 (17·8 to 19·9)	18·8 (17·8 to 19·9)	18·6 (17·6 to 19·6)	0·1% (0·1 to 0·1)	−0·1% (−0·1 to 0·0)	−0·1% (−0·2 to 0·0)
		Iron deficiency		8·6 (7·3 to 10·3)	8·4 (7·1 to 10·1)	7·9 (6·7 to 9·5)	7·5 (6·2 to 9·1)	−0·4% (−0·5 to −0·4)	−0·6% (−0·7 to −0·4)	−0·5% (−0·7 to −0·3)
		Vitamin A deficiency		25·2 (0·0 to 35·9)	22·0 (0·0 to 31·5)	16·4 (0·0 to 24·0)	10·6 (0·0 to 15·5)	−2·8% (−3·1 to 0·0)	−3·5% (−3·9 to 0·0)	−4·0% (−4·5 to 0·0)
		Zinc deficiency		10·3 (0·0 to 24·9)	10·3 (0·0 to 24·6)	8·6 (0·0 to 21·5)	6·4 (0·0 to 18·0)	−1·5% (−2·5 to 0·0)	−2·2% (−4·0 to 0·0)	−2·7% (−5·4 to 0·0)
	Tobacco			39·1 (38·2 to 39·7)	35·9 (35·1 to 36·5)	31·7 (31·1 to 32·3)	28·3 (27·6 to 28·9)	−1·0% (−1·1 to −1·0)	−1·1% (−1·2 to −1·1)	−1·0% (−1·2 to −0·9)
		Smoking		23·4 (22·9 to 24·0)	20·9 (20·5 to 21·4)	18·5 (18·1 to 18·9)	16·0 (15·6 to 16·5)	−1·2% (−1·3 to −1·2)	−1·3% (−1·4 to −1·2)	−1·3% (−1·5 to −1·2)
		Chewing tobacco		4·8 (4·3 to 5·2)	5·0 (4·7 to 5·3)	5·0 (4·7 to 5·3)	5·0 (4·6 to 5·4)	0·2% (−0·2 to 0·6)	0·0% (−0·5 to 0·5)	0·0% (−0·7 to 0·7)
		Second-hand smoke		44·6 (42·0 to 45·3)	42·1 (39·6 to 42·9)	37·8 (35·5 to 38·5)	34·3 (32·2 to 35·1)	−0·8% (−0·9 to −0·8)	−1·0% (−1·1 to −0·9)	−0·9% (−1·0 to −0·7)
	High alcohol use			13·8 (11·1 to 19·9)	13·1 (10·5 to 19·3)	13·0 (10·4 to 19·3)	12·6 (10·0 to 19·0)	−0·3% (−0·4 to −0·1)	−0·2% (−0·3 to 0·0)	−0·2% (−0·5 to 0·0)
	Drug use			0·4 (0·3 to 0·6)	0·4 (0·4 to 0·6)	0·4 (0·3 to 0·5)	0·5 (0·4 to 0·6)	0·5% (−0·6 to 1·4)	0·4% (−0·7 to 1·3)	1·7% (0·3 to 2·6)
	Dietary risks			40·3 (30·7 to 50·1)	38·9 (29·5 to 48·8)	38·0 (28·5 to 48·2)	37·6 (28·1 to 47·8)	−0·2% (−0·3 to −0·1)	−0·2% (−0·3 to −0·1)	−0·1% (−0·2 to 0·0)
		Diet low in fruits		45·3 (37·9 to 47·5)	43·7 (36·7 to 45·8)	41·7 (35·3 to 43·8)	40·9 (34·9 to 42·9)	−0·3% (−0·4 to −0·3)	−0·3% (−0·4 to −0·2)	−0·2% (−0·3 to −0·1)
		Diet low in vegetables		33·9 (20·6 to 40·6)	29·1 (18·2 to 35·4)	27·2 (17·0 to 32·9)	27·3 (17·2 to 32·9)	−0·7% (−0·8 to −0·6)	−0·3% (−0·4 to −0·2)	0·1% (−0·1 to 0·2)
		Diet low in legumes		39·6 (0·0 to 47·9)	35·3 (0·0 to 42·9)	32·4 (0·0 to 39·5)	31·7 (0·0 to 39·0)	−0·7% (−0·8 to 0·0)	−0·5% (−0·6 to 0·0)	−0·2% (−0·3 to 0·0)
		Diet low in whole grains		41·9 (34·4 to 46·7)	42·7 (35·2 to 47·7)	43·6 (36·1 to 48·8)	43·8 (36·1 to 49·2)	0·1% (0·1 to 0·2)	0·1% (0·0 to 0·2)	0·0% (−0·1 to 0·1)
		Diet low in nuts and seeds		42·9 (41·4 to 45·1)	37·4 (36·1 to 39·2)	34·0 (32·7 to 35·8)	31·4 (30·0 to 33·2)	−1·0% (−1·1 to −0·9)	−0·8% (−1·0 to −0·7)	−0·7% (−0·9 to −0·6)
		Diet low in milk		62·7 (60·6 to 71·8)	62·5 (60·4 to 71·5)	63·2 (61·1 to 72·0)	64·9 (62·9 to 73·8)	0·1% (0·1 to 0·1)	0·2% (0·1 to 0·2)	0·2% (0·2 to 0·3)
		Diet high in red meat		27·1 (0·0 to 37·2)	27·3 (0·0 to 37·4)	29·0 (0·0 to 39·5)	29·4 (0·0 to 39·7)	0·3% (0·0 to 1·0)	0·4% (0·1 to 1·6)	0·1% (−0·1 to 1·0)
		Diet high in processed meat		17·0 (13·4 to 17·9)	17·2 (13·6 to 18·1)	17·3 (13·8 to 18·2)	16·2 (13·0 to 17·1)	−0·2% (−0·2 to −0·1)	−0·3% (−0·4 to −0·2)	−0·6% (−0·7 to −0·5)
		Diet high in sugar-sweetened beverages		13·5 (11·1 to 14·3)	14·8 (12·2 to 15·6)	16·9 (13·8 to 17·8)	18·8 (15·3 to 19·9)	1·1% (1·0 to 1·2)	1·1% (1·0 to 1·2)	0·9% (0·7 to 1·1)
		Diet low in fibre		36·1 (19·1 to 38·7)	34·3 (18·0 to 36·9)	30·5 (16·1 to 33·0)	26·4 (14·3 to 28·7)	−1·0% (−1·2 to −0·8)	−1·2% (−1·5 to −1·0)	−1·3% (−1·6 to −1·0)
		Diet low in calcium		25·1 (23·5 to 36·2)	23·5 (22·0 to 34·1)	21·5 (20·0 to 31·3)	19·9 (18·5 to 29·1)	−0·7% (−0·8 to −0·7)	−0·8% (−0·9 to −0·7)	−0·7% (−0·7 to −0·6)
		Diet low in seafood omega-3 fatty acids		49·2 (40·2 to 58·6)	46·6 (37·9 to 55·7)	40·6 (32·4 to 49·0)	35·5 (28·0 to 43·3)	−1·1% (−1·2 to −0·9)	−1·3% (−1·5 to −1·1)	−1·2% (−1·4 to −1·0)
		Diet low in omega-6 polyunsaturated fatty acids		78·2 (45·0 to 90·1)	77·3 (44·7 to 89·0)	76·0 (44·1 to 87·6)	75·6 (44·0 to 87·3)	−0·1% (−0·1 to 0·0)	−0·1% (−0·1 to 0·0)	0·0% (−0·1 to 0·0)
		Diet high in trans fatty acids		15·3 (13·6 to 16·6)	15·3 (13·7 to 16·8)	10·2 (8·9 to 11·2)	6·4 (5·3 to 7·5)	−2·8% (−3·2 to −2·2)	−4·1% (−4·7 to −3·5)	−4·2% (−5·2 to −2·9)
		Diet high in sodium		40·6 (12·6 to 78·6)	40·9 (12·4 to 79·2)	40·8 (12·2 to 79·7)	40·0 (11·7 to 79·0)	0·0% (−0·4 to 0·1)	−0·1% (−0·6 to 0·1)	−0·2% (−0·7 to 0·0)
	Intimate partner violence			23·6 (14·7 to 26·0)	23·4 (16·2 to 25·3)	22·5 (15·9 to 24·5)	22·0 (14·5 to 24·2)	−0·2% (−0·5 to 0·1)	−0·3% (−0·6 to 0·0)	−0·2% (−0·6 to 0·2)
	Childhood sexual abuse and bullying			8·2 (5·1 to 13·1)	8·7 (5·4 to 13·8)	9·2 (5·7 to 14·8)	8·0 (5·1 to 12·5)	0·0% (−0·2 to 0·1)	−0·3% (−0·6 to −0·1)	−1·2% (−1·7 to −0·8)
		Childhood sexual abuse		6·9 (6·5 to 7·6)	6·7 (6·3 to 7·3)	6·6 (6·2 to 7·2)	6·3 (5·9 to 6·9)	−0·3% (−0·5 to −0·1)	−0·3% (−0·6 to 0·0)	−0·4% (−0·7 to −0·1)
		Bullying victimisation		7·9 (3·5 to 15·1)	8·7 (4·0 to 16·5)	9·6 (4·4 to 18·0)	8·0 (3·8 to 14·7)	0·0% (−0·2 to 0·3)	−0·4% (−0·7 to −0·1)	−1·7% (−2·1 to −1·3)
	Unsafe sex			..	..	..	..	..	..	..
	Low physical activity			18·2 (15·6 to 21·0)	18·2 (15·7 to 21·0)	18·8 (16·1 to 21·9)	19·8 (17·0 to 23·0)	0·3% (0·1 to 0·5)	0·4% (0·2 to 0·7)	0·5% (0·2 to 0·8)
**Metabolic risks**				**13·0 (11·5 to 14·9)**	**15·0 (13·4 to 16·9)**	**17·5 (15·8 to 19·4)**	**20·9 (18·9 to 22·9)**	**1·5% (1·4 to 1·7)**	**1·6% (1·4 to 1·7)**	**1·6% (1·4 to 1·7)**
	High fasting plasma glucose			10·6 (8·3 to 11·9)	12·2 (9·6 to 13·7)	14·1 (11·0 to 15·6)	16·2 (12·5 to 17·8)	1·4% (1·2 to 1·5)	1·3% (1·1 to 1·5)	1·2% (1·0 to 1·5)
	High LDL cholesterol			46·8 (31·8 to 64·8)	45·9 (31·2 to 63·8)	45·4 (30·8 to 63·2)	45·3 (30·7 to 63·1)	−0·1% (−0·1 to −0·1)	−0·1% (−0·1 to 0·0)	0·0% (0·0 to 0·0)
	High systolic blood pressure			33·0 (23·9 to 43·8)	32·9 (23·7 to 44·4)	33·5 (23·9 to 45·0)	35·6 (25·9 to 47·0)	0·2% (0·1 to 0·4)	0·4% (0·1 to 0·7)	0·6% (0·2 to 1·0)
	High body-mass index			12·6 (11·1 to 14·7)	14·8 (13·3 to 17·0)	17·7 (15·9 to 20·0)	21·5 (19·2 to 24·0)	1·7% (1·5 to 1·9)	1·8% (1·6 to 1·9)	1·8% (1·6 to 1·9)
	Low bone mineral density			24·7 (19·2 to 31·4)	24·2 (18·9 to 30·7)	23·8 (18·4 to 30·4)	23·5 (18·0 to 30·3)	−0·2% (−0·2 to −0·1)	−0·1% (−0·3 to 0·0)	−0·1% (−0·3 to 0·0)
	Kidney dysfunction			2·9 (2·3 to 3·8)	2·7 (2·2 to 3·6)	2·7 (2·2 to 3·6)	2·7 (2·1 to 3·6)	−0·2% (−0·3 to −0·2)	−0·1% (−0·2 to 0·0)	0·0% (−0·1 to 0·0)

Data in parentheses are 95% uncertainty intervals. GBD=Global Burden of Diseases, Injuries, and Risk Factors Study. SEV=summary exposure value.

We categorised trends in SEVs between 2000 and 2021 as either increasing substantially (ARCs of >0·5%), declining substantially (ARCs decreasing >0·5%), or as neither (ARC values between –0·5% and 0·5%). At Level 1 of the risk hierarchy, only metabolic risk factors increased in exposure between 2000 and 2021, with an annualised increase of 1·6% (95% UI 1·4–1·7; table 2). Among specific Level 2 metabolic risks, exposure increased considerably between 2000 and 2021 for high BMI at an annual rate of 1·8% (1·6–1·9) and for high FPG at a rate of 1·3% (1·1–1·5). Among Level 2 environmental and occupational risks, exposure to unsafe water, sanitation, and handwashing (WaSH) had the largest annual declines between 2000 and 2021, at 1·2% (0·9–1·7), followed by exposure to air pollution (an annual decline of 1·1% [0·9–1·4]). Changes in exposure to specific types of air pollution ranged from an annual decline of 2·7% (1·6–3·6) for household air pollution from solid fuels to an annual increase of 1·3% (0·6–1·9) for ambient particulate matter pollution. Among Level 2 behavioural risks, the largest declines in exposure were for tobacco use (an annual decline of 1·1% [1·1–1·2]), driven by an annual decline in smoking exposure of 1·3% (1·2–1·4). Among Level 3 risks, the largest annual decline was for diet high in trans fatty acids (a 4·1% [3·5–4·7] decrease). Only one behavioural risk had an annual increase in exposure higher than 0·5%: diet high in sugar-sweetened beverages, at 1·1% (1·0–1·2). See table 2 for a complete list of annualised rates of change in exposure to all GBD risk factors. See appendix 2 (table S3) for location-specific and sex-specific age-standardised SEVs and percentage change in SEVs over time for all risk factors over the period 1990–2021.

### Risk-attributable burden (DALYs)

**Figure 1:**
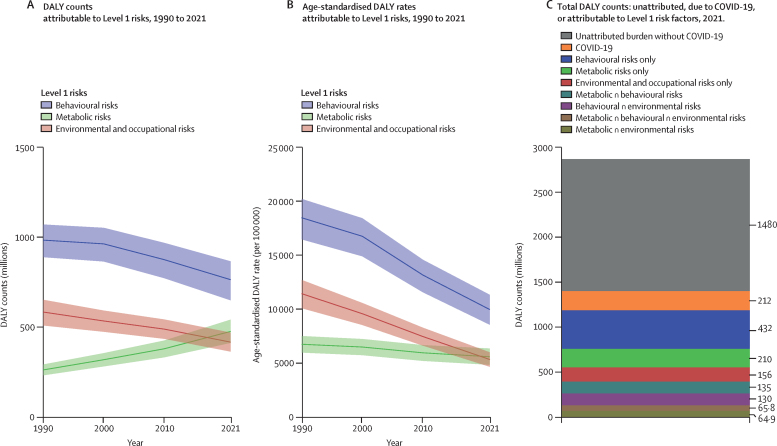
Global DALYs attributable to Level 1 risk factors, 1990–2021 (A) Global DALY counts attributable to Level 1 risks, 1990 to 2021. (B) Age-standardised DALY rates attributable to Level 1 risks, 1990 to 2021. (C) Global total DALY counts that were unattributed, due to COVID-19, or attributable to Level 1 risk factors, 2021. Mean estimates by Level 1 risk factor in panels A and B are represented by coloured lines; the shading indicates 95% uncertainty intervals. For panel C, ∩ refers to a burden that is attributed to two or all three Level 1 risk factors (ie, the intersecting set of DALYs that belong to both or all three risk factors). Mean estimates in panels A and B are aggregated to include all DALYs attributable exclusively to the specific Level 1 risk factor plus those attributable to the intersection of that risk and one or both of the other Level 1 risk factors (ie, for a single year, the DALY counts combined across the three lines sum to more than the total number of attributable DALYs for that year). DALYs due to COVID-19 were estimated as part of a separate GBD 2021 analysis by the GBD 2021 Diseases and Injuries Collaborators. They have been separated in this figure from the DALYs unattributed to a risk factor because attribution of COVID-19 DALYs to risk exposure was not conducted as part of this analysis. In GBD 2021, 41·4% of total global DALYs—or 44·7% excluding COVID-19 DALYs—were attributable to risk factors (see also appendix 2 figure S4); whereas in GBD 2019,^14^ 47·8% of total global DALYs were attributable to risk factors. DALY=disability-adjusted life-year. Environmental risks=environmental and occupational risks. GBD=Global Burden of Diseases, Injuries, and Risk Factors Study.

**Figure 2:**
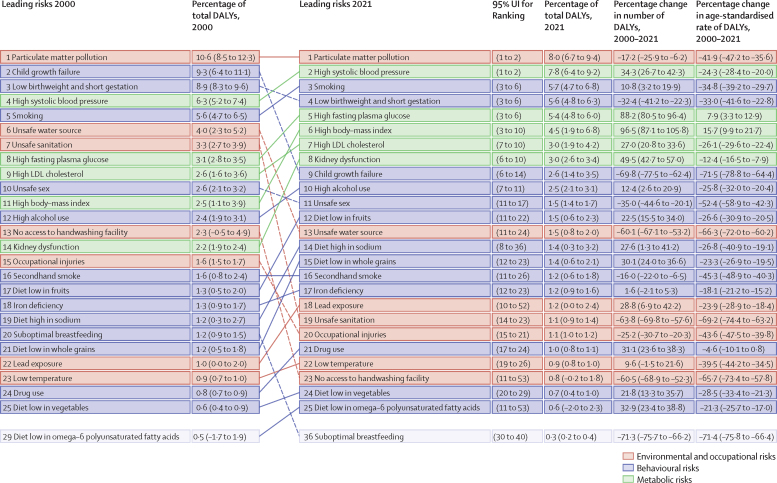
Leading 25 Level 3 risk factors by attributable DALYs, percentage of total DALYs (2000 and 2021), and percentage change in attributable DALY counts and age-standardised DALY rates from 2000 to 2021 Each column displays the top 25 risks in descending order for the specified year. Risk factors are connected by lines between time periods; solid lines represent an increase or lateral shift in ranking, dashed lines represent a decrease in rank. DALY=disability-adjusted life-year. UI=uncertainty interval.

For Level 1 risks, the attributable global disease burden—as measured in DALYs reflecting both premature death and years lived in poor health—was highest in 2021 for behavioural risks, followed by metabolic risks, then environmental and occupational risks (figure 1). 763 million (95% UI 650–865) DALYs were attributable to behavioural risks, 476 million (412–541) were attributable to metabolic risks, and 416 million (364–469) were attributable to environmental and occupational risks (appendix 2 table S1). In aggregate, 1190 million(1090–1330) global DALYs (41·4% of 2880 million DALYs in 2021)^33^ were attributable to all GBD 2021 risk factors combined, and 212 million (198–234; 7·4% of all DALYs in 2021) were due to COVID-19 (figure 1),^33^ for which there was no risk factor attributable burden estimation included in GBD 2021 due to data constraints and limitations in understanding of COVID-19 risk factors and interactions with comorbidities. Further disaggregation of risk-attributable burden estimates showed that particulate matter pollution (including ambient and household air pollution) was the leading Level 3 risk factor globally in 2021, contributing 8·0% (6·7–9·4) of total DALYs (figure 2; appendix 2 table S1; notably, 95% UIs associated with the 2021 rank orderings for risk factors are also presented in figure 2). Two of the other top five Level 3 risk factors were metabolic, with high SBP ranked as the second-leading risk (contributing 7·8% [6·4–9·2] of total DALYs) and high FPG ranked fifth (5·4% [4·8–6·0] of total DALYs). The remaining top five risks were behavioural, with smoking ranked third (5·7% [4·7–6·8] of total DALYs) and low birthweight and short gestation ranked fourth (5·6% [4·8–6·3] of total DALYs). Of the 25 leading Level 3 risk factors in 2021, 13 (ie, more than half) were behavioural risks. Five of the leading 25 Level 3 risk factors were metabolic risks—all of which were ranked in the top ten, with high BMI ranked sixth, high LDL cholesterol seventh, and kidney dysfunction eighth—and the remaining seven were environmental or occupational risk factors.

The contribution of Level 3 risk factors to global DALYs in 2021 varied considerably by age (appendix 2 figure S2a–e) and sex (appendix 2 figure S1a–b). Among children aged 0–5 years, low birthweight and short gestation, child growth failure, particulate matter pollution, unsafe water source, and unsafe sanitation were the leading five Level 3 risk factors. There were no metabolic risks in the top ten risk factors for this age group. For children and adolescents aged 5–14 years, iron deficiency, low birthweight and short gestation, and the three risks falling under the category of unsafe WaSH were the leading five risk factors, with only one metabolic risk—kidney dysfunction—in the top ten. Behavioural risk factors had a greater impact in those aged 15–49 years, with high alcohol use, unsafe sex, and smoking among the top five risks. Metabolic risk factors also gained influence, with high BMI, high FPG, high SBP, and high LDL cholesterol all in the top ten risk factors. For those aged 50–69 years, these same metabolic risks dominated the top ten ranking, and dietary risks (diets high in sodium, low in fruits, and low in whole grains) also gained in prominence. For those aged 70 years and older, metabolic risks remained dominant, and lead exposure and low temperature joined particulate matter pollution as environmental risks among the top ten risk factors.

**Figure 3:**
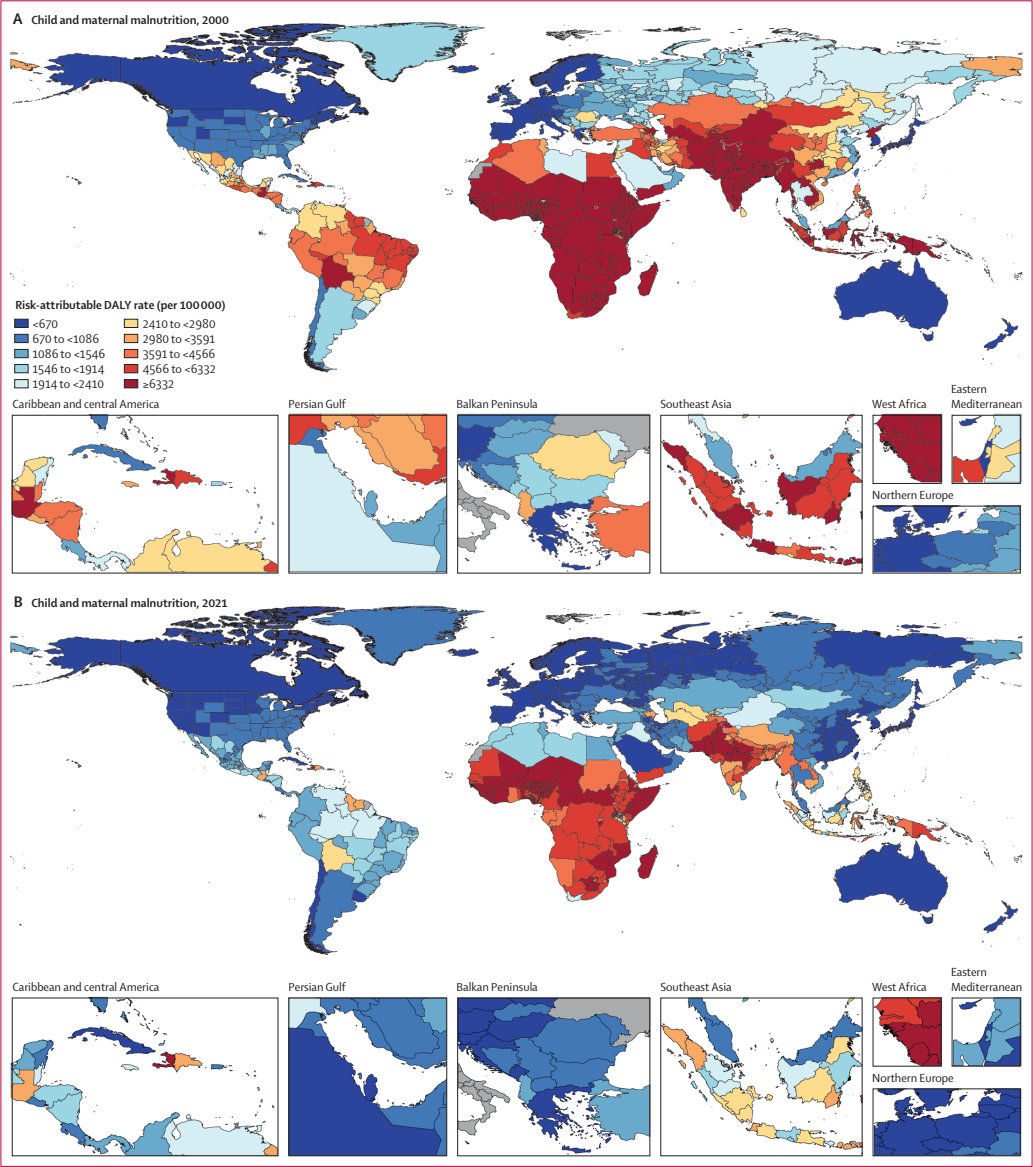

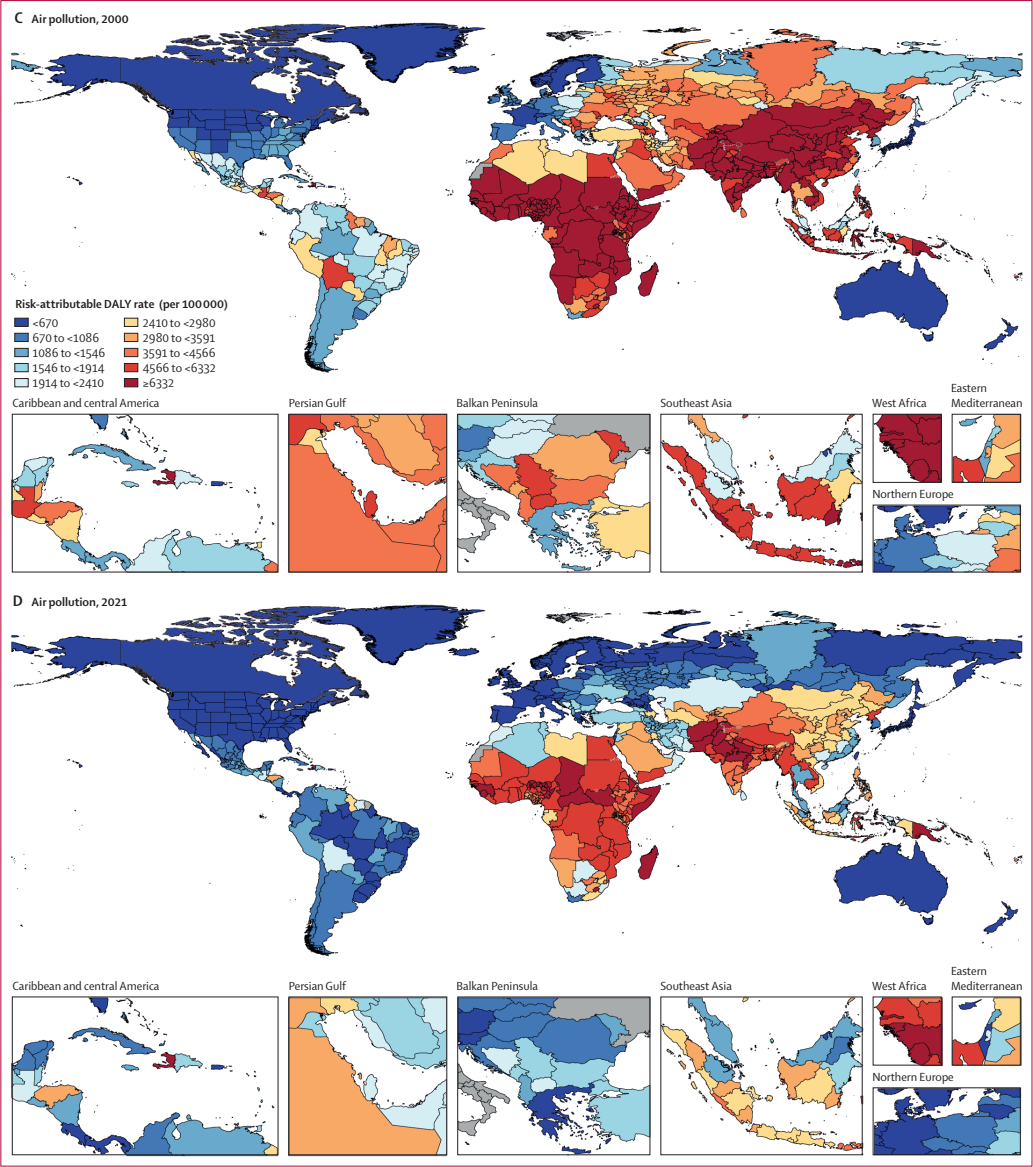

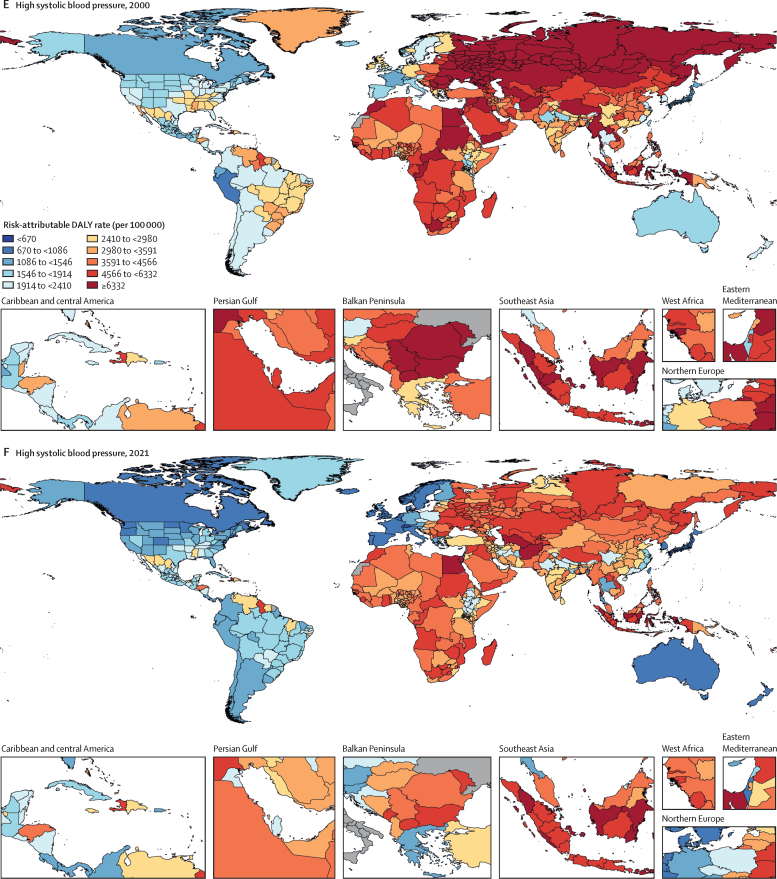

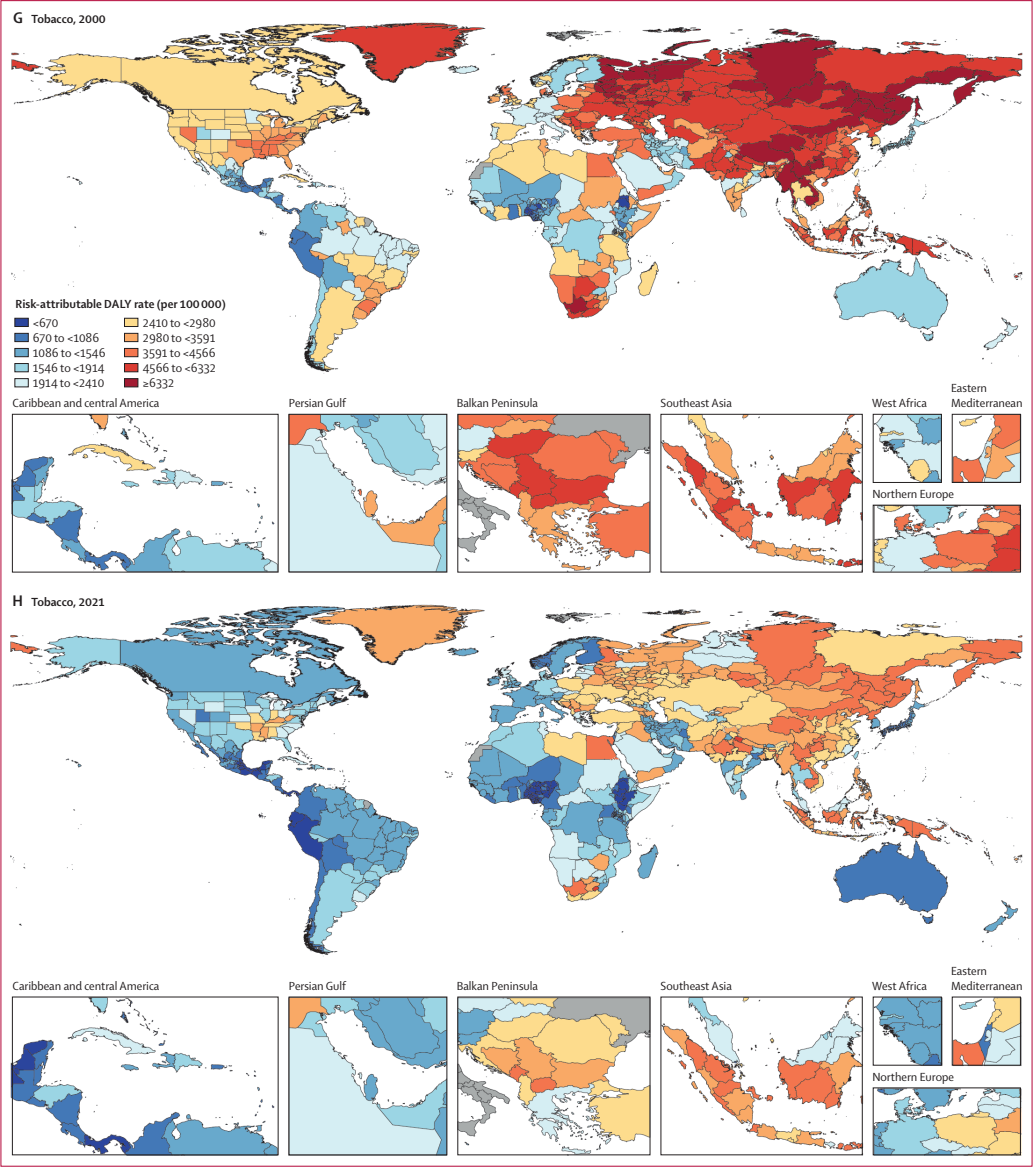

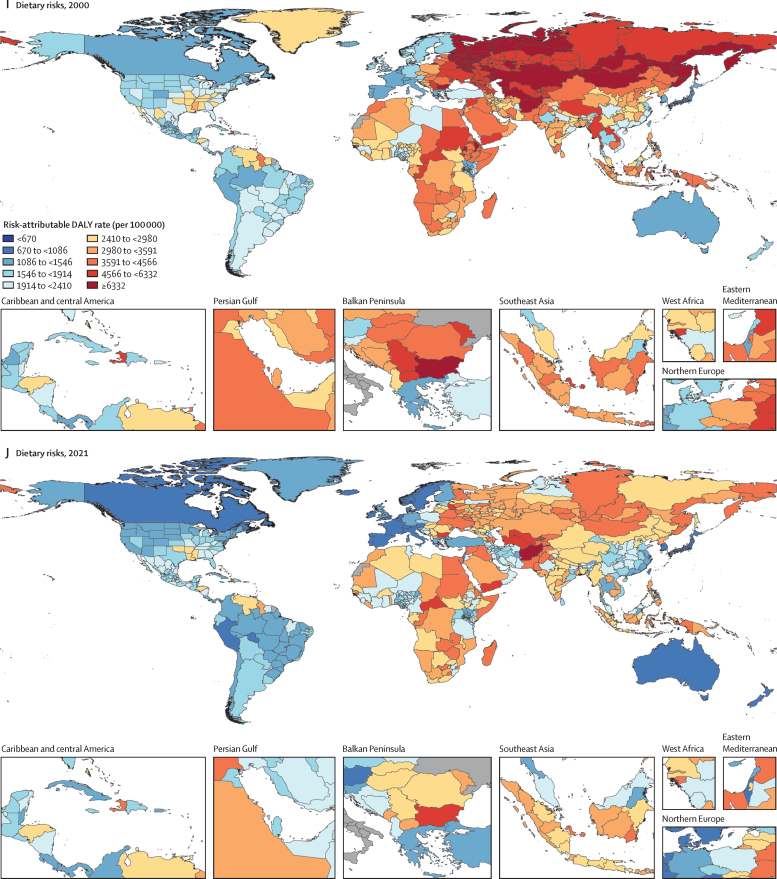
Age-standardised DALY rate attributable to the five leading Level 2 risk factors in 2000 and 2021 by location (A) Child and maternal malnutrition, 2000. (B) Child and maternal malnutrition, 2021. (C) Air pollution, 2000. (D) Air pollution, 2021. (E) High systolic blood pressure, 2000. (F) High systolic blood pressure, 2021. (G) Tobacco, 2000. (H) Tobacco, 2021. (I) Dietary risks, 2000. (J) Dietary risks, 2021. DALY=disability-adjusted life-year.

**Figure 4:**
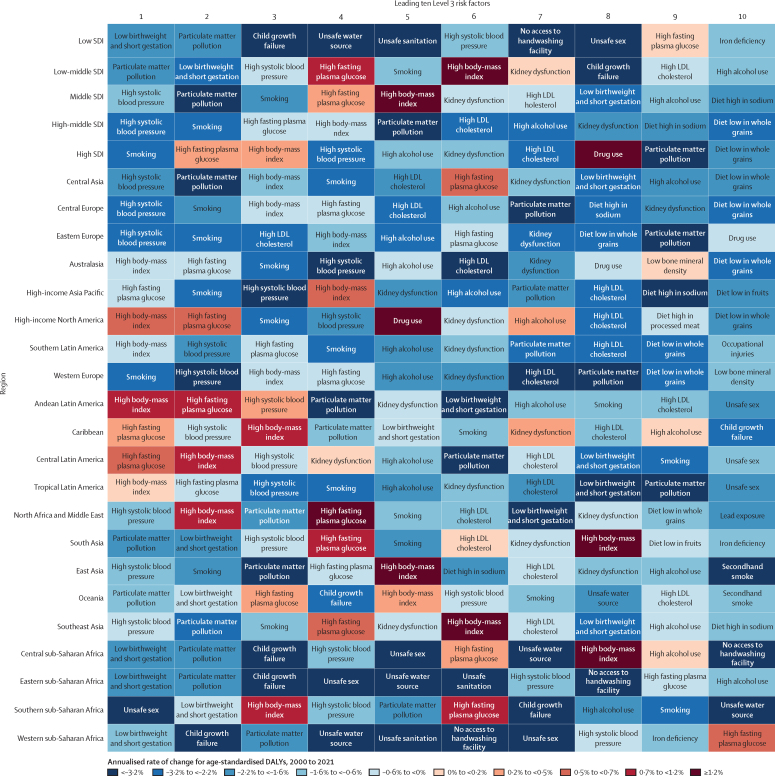
Annualised rate of change in age-standardised attributable DALY rates, 2000–21, for the leading ten Level 3 risk factors in 2021, by SDI quintile and GBD region For each region and SDI quintile, Level 3 risk factors are ranked by attributable DALY counts from left (first) to right (tenth). Risk factors are coloured by their annualised rates of change in age-standardised rates of attributable DALYs from 2000 to 2021. DALY=disability-adjusted life-year. GBD=Global Burden of Diseases, Injuries, and Risk Factors Study. SDI=Socio-demographic Index.

Age-standardised DALY rates for all outcomes combined attributable to all GBD risk–outcome pairs in 2021 varied by geography (appendix 2 figure S3), as did rates attributable to the five leading global Level 2 risk factors in 2021: child and maternal malnutrition, air pollution, high SBP, tobacco, and dietary risks (figure 3A–J). Age-standardised DALY rates for child and maternal malnutrition were highest in the GBD super-regions of sub-Saharan Africa; south Asia; areas of north Africa and the Middle East; and parts of southeast Asia, east Asia, and Oceania (figure 3B). Regionally, low birthweight and short gestation—a component of child and maternal malnutrition—was ranked as the leading Level 3 risk factor based on attributable all-age DALYs in eastern, central, and western sub-Saharan Africa (with a country-level high of 5668·0 [95% UI 4317·1–7321·4] age-standardised DALYs per 100  000 in South Sudan) and the second-leading risk factor in southern sub-Saharan Africa and Oceania (figure 4, appendix 2 table S1). Low birthweight and short gestation was the leading risk in the low SDI group and second-leading risk in the low-middle SDI group (figure 4).

The second-leading global Level 2 risk factor—air pollution—contributed attributable age-standardised DALY rates that were highest in south Asia, sub-Saharan Africa, and parts of north Africa and the Middle East (figure 3D). At a regional level, particulate matter pollution was the leading Level 3 risk factor for the attributable number of all-age DALYs in south Asia (highest in Pakistan, at 6278·3 [95% UI 5312·0–7405·6] age-standardised DALYs per 100 000) and Oceania (highest in the Solomon Islands, at 8813·4 [6860·2–11 307·0] age-standardised DALYs per 100 000) and the second-ranking risk factor in central Asia, southeast Asia, and eastern and central sub-Saharan Africa (figure 4, appendix 2 table S1). Particulate matter pollution was ranked as the leading Level 3 risk factor in the low-middle SDI group and the second-leading risk factor in both the low SDI and middle SDI groups (figure 4). World maps of the 2021 global burden attributable to the third-leading, fourth-leading, and fifth-leading global Level 2 risk factors—high SBP, tobacco, and dietary risks, respectively—are shown in figures 3F,H,J, and country-level estimates of the burden attributable to the associated Level 3 risks are shown in appendix 2 (table S1).

All-age PAFs—used to calculate the attributable burden—for all risk factors combined are provided in appendix 2 (table S4). Detailed information on estimates related to the attributable burden—PAFs, DALYs, and deaths—for each risk factor and outcome, across geography and time are provided in appendix 2 (table S1).

**Figure 5:**
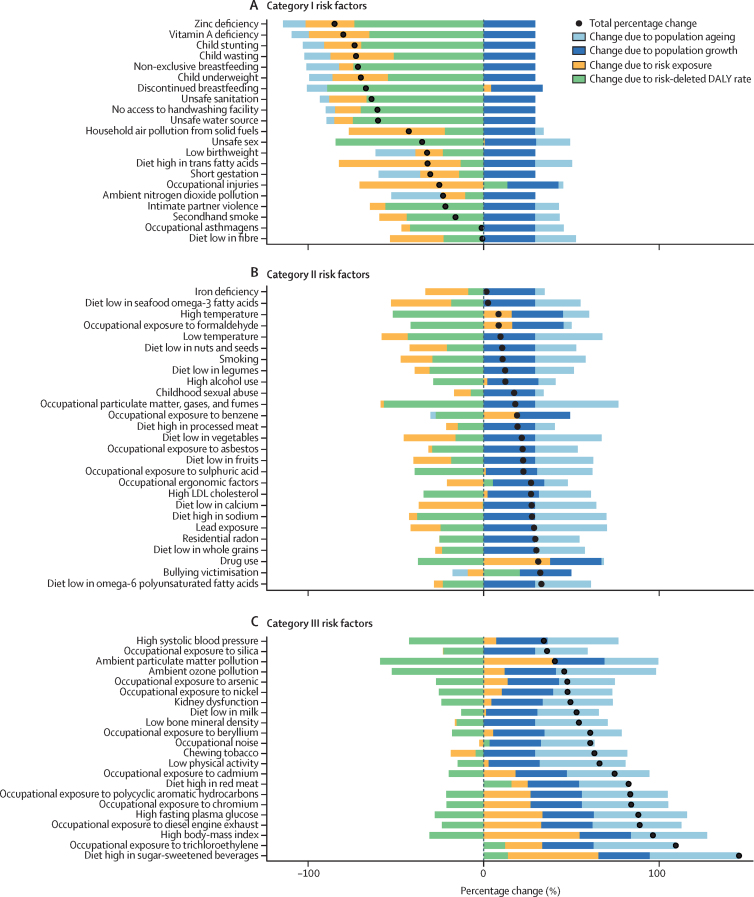
Percentage change in global DALY counts attributable to Level 4 risk factors from 2000 to 2021, due to population growth, population ageing, changes in risk factor exposure, and changes in risk-deleted DALY rates (A) Category I risk factors. (B) Category II risk factors. (C) Category III risk factors. This decomposition analysis visualises changes in risk-specific attributable DALYs from 2000 to 2021 due to changes in risk exposure, population growth, population age structure, and risk-deleted DALYs. Risk-deleted DALY rates are DALY rates after removing the effect of a risk factor or combination of risk factors on overall rates. They are calculated as the overall DALY rate multiplied by one minus the PAF for the risk or set of risks; this isolates the underlying changes in DALY rates unattributable to risk factors. Broadly grouped into three categories, category I risk factors are those for which the risk-attributable burden declined due in large part to decreased risk exposure, but in some cases also due to proportional declines in young populations due to population ageing. Category II risk factors are those for which the risk-attributable burden increased moderately despite decreased risk factor exposure, due largely to population ageing. Category III risk factors are those for which the risk-attributable burden increased considerably, due to both increased risk factor exposure and population ageing. DALY=disability-adjusted life-year. PAF=population attributable fraction.

Over the period 2000–2021, all-age DALY counts attributable to behavioural risks declined by 20·7% (95% UI 13·9–27·7), and those attributable to environmental and occupational risks declined by 22·0% (15·5–28·8). However, those attributable to metabolic risks increased by 49·4% (42·3–56·9; figure 1, appendix 2 table S1). This differential can be ascribed in part to the effect of metabolic risk factors in ageing populations, considering that age-standardised global DALY rates attributable to metabolic risks decreased over the same period (by 13·9% [9·54–18·0]); however, this decline was much less pronounced than that of other Level 1 risk factors (declines of 44·3% [40·0–48·7] for environmental and occupational risks and 40·5% [35·8–45·2] for behavioural risks; figure 1, appendix 2 table S1). The relatively small decline in age-standardised DALY rates attributable to metabolic risks was due largely to an increase in age-standardised attributable DALY rates for two prominent Level 3 metabolic risk factors: high BMI, which rose by 15·7% (9·9–21·7), and high FPG, which increased by 7·9% (3·3–12·9; figure 2). These increases stand in contrast to decreases in age-standardised risk-attributable global DALY rates over the same time period for all other leading 25 Level 3 risk factors (figure 2), including considerable declines in the attributable burden for risk factors related to child and maternal malnutrition, such as child growth failure (with age-standardised attributable DALY rates decreasing by 71·5% [64·4–78·8]), and low birthweight and short gestation (with rates decreasing by 33·0% [22·8–41·6]). The global burden attributable to unsafe water, unsafe sanitation, and no access to handwashing facility (all top 25 risks) likewise declined, with decreases in attributable age-standardised DALYs of 66·3% (60·2–72·0) for unsafe water source, 69·2% (63·2–74·4) for unsafe sanitation, and 65·7% (57·8–73·4) for no access to handwashing facility. Age-standardised rates attributable to particulate matter pollution—the leading Level 3 risk factor in 2021—also declined considerably (41·9% [35·6–47·2]) over the same period. Notably, although global age-standardised rates attributable to particulate matter pollution decreased between 2000 and 2021, further disaggregation into specific types of particulate matter pollution reveals that while global all-age DALY counts declined by 42·6% (26·5–54·6) for household air pollution from solid fuels, they increased by 40·6% (21·5–62·9) for ambient particulate matter pollution (figure 5, appendix 2 table S1). Decomposition analysis quantifying the drivers underlying the change in attributable burden indicates that exposure to household air pollution decreased over time but exposure to ambient particulate matter pollution increased, and further shows that population ageing played a larger role in increasing the burden attributable to ambient particulate matter pollution (figure 5; details on decomposition methods are provided in appendix 1 section 3).

Decomposing time trends of the number of global DALYs attributable to Level 4 risk factors revealed three main groups of risks (figure 5). The first group (category I) generally includes risk factors for which the global attributable burden decreased between 2000 and 2021 due in large part to declines in risk exposure (eg, occupational injuries, diet high in trans fatty acids, household air pollution from solid fuels, and diet low in fibre), and in some cases these exposure declines were enhanced by the positive effects of population ageing (ie, a given risk has a proportionally greater burden in younger individuals and therefore the risk-attributable burden declines in ageing populations). Category I risk factors in this latter class mainly fall under the broader umbrellas of child and maternal malnutrition and unsafe WaSH. The second group (category II) mainly comprises risk factors for which global attributable DALYs increased moderately between 2000 and 2021, in most cases despite decreasing risk exposure and in almost all cases related to the negative effects of population ageing. This group includes numerous dietary factors (eg, diets low in calcium, low in fruits, and low in vegetables), smoking, and risks related to environmental or occupational factors (eg, occupational exposure to asbestos and lead exposure). The third group (category III) includes risk factors for which the attributable global disease burden rose considerably over the study period due to both increasing risk exposure and the effects of population ageing. This group comprises many metabolic risks (eg, high BMI, high FPG, low bone mineral density, kidney dysfunction, and high SBP), occupational risks (occupational exposure to trichloroethylene, diesel engine exhaust, chromium, cadmium, and others), and some dietary risks (diets high in sugar-sweetened beverages, high in red meat, and low in milk). For nearly all risk factors across all three groups, risk-deleted global DALY rates (ie, change in DALYs not attributable to a risk factor included in our assessment, to population growth, or to ageing) exerted a downward effect on trends in risk-attributable global DALY counts between 2000 and 2021.

**Figure 6:**
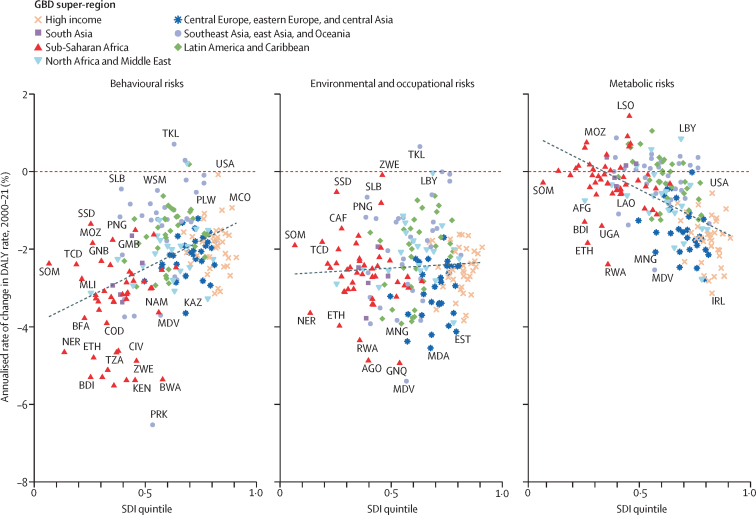
Annualised rate of change in age-standardised risk-attributable DALY rates by Level 1 risk, by SDI quintile and country or territory, 2000–21 The grey dashed lines depict the linear regression line. Country and territory points are categorised by GBD super-region. Selected countries and territories are labelled by ISO 3 codes. AFG=Afghanistan. AGO=Angola. BDI=Burundi. BFA=Burkina Faso. BWA=Botswana. CAF=Central African Republic. CIV=Côte D’Ivoire. COD=DR Congo. DALY=disability-adjusted life-year. EST=Estonia. ETH=Ethiopia. GBD=Global Burden of Diseases, Injuries, and Risk Factors Study. GMB=The Gambia. GNB=Guinea-Bissau. GNQ=Equatorial Guinea. IRL=Ireland. KAZ=Kazakhstan. KEN=Kenya. LAO=Laos. LBY=Libya. LSO=Lesotho. MCO=Monaco. MDA=Moldova. MDV=Maldives. MLI=Mali. MNG=Mongolia. MOZ=Mozambique. NAM=Namibia. NER=Niger. PNG=Papua New Guinea. PRK=North Korea. RWA=Rwanda. SDI=Socio-demographic Index. SLB=Solomon Islands. SOM=Somalia. SSD=South Sudan. TCD=Chad. TKL=Tokelau. TZA=Tanzania. UGA=Uganda. WSM=Samoa. ZWE=Zimbabwe.

Trends in the risk-attributable burden varied both by SDI level and by location. Figure 6 provides a high-level overview of ARCs between 2000 and 2021 in age-standardised DALY rates attributable to Level 1 risk factors, by SDI. For behavioural risks, the attributable burden declined at a lower rate in higher SDI areas than in lower SDI areas over this period. Conversely, the attributable burden for metabolic risks generally declined at a higher rate with increasing SDI. There was minimal association between SDI and all environmental and occupational risks combined. Disaggregating to a more specified level of risk factors, figure 4 presents ARCs between 2000 and 2021 for age-standardised DALYs attributable to the ten leading Level 3 risks, stratified by SDI and GBD region. Age-standardised rates of attributable DALYs declined at high rates for behavioural risk factors related to child and maternal malnutrition in the low and middle SDI quintiles; that is, child growth failure showed declines in ARCs of more than 3·2% and low birthweight and short gestation showed declines in ARCs of 1·6–3·2%. The DALY burden attributable to unsafe sex, another behavioural risk factor, also declined at high rates (ARC decrease of >3·2%) in the low SDI quintile. Although higher SDI quintiles had higher rates of decline (ARC decrease of 1·6–3·2%) in the burden attributable to the behavioural risk factor of smoking than did lower SDI quintiles, DALY rates attributable to drug use notably increased (ARC increase of >1·2%) in the high SDI quintile. With respect to metabolic risk factors, figure 4 shows that although the burden attributable to high SBP and high LDL cholesterol decreased over time across SDI strata, rates of decline were highest in the high-middle and high SDI quintiles (ARC decreases of 2·2–3·2%). Notably, the burden attributable to high BMI increased in the low-middle, middle, and high SDI quintiles, showing the highest rates of increase (ARC increase of >1·2%) in the low-middle and middle SDI quintiles, and decreased in the high-middle SDI quintile. For high FPG, attributable DALY rates increased across almost all SDI strata, with the highest rates of increase (ARC increase of 0·7–1·2%) in the low-middle SDI quintile. However, the high-middle SDI quintile was again an exception, showing slight declines between 2000 and 2021 in the burden attributable to high FPG. With respect to Level 3 environmental risk factors, the burden attributable to particulate matter pollution decreased between 2000 and 2021, with the highest rates of decline in the high, high-middle, and middle SDI quintiles (ARC decrease of >3·2%), and the burden attributable to all three Level 3 risks related to unsafe WaSH decreased at high rates (ARC decline of >3·2%) in the low SDI quintile.

Between 2000 and 2021, reductions in age-standardised DALY rates attributable to child and maternal malnutrition, the leading level 2 risk factor in 2021, were broadly evident in Latin America and the Caribbean, central Asia, and parts of east Asia and southeast Asia (figure 3A,B). Risk-attributable DALY rates also decreased in parts of sub-Saharan Africa, but child and maternal malnutrition remained a challenge in 2021 throughout this super-region, in addition to northern India and countries such as Pakistan, Afghanistan, Yemen, and Papua New Guinea. Location-specific reductions in Level 3 risks related to the child and maternal disease burden between 2000 and 2021 are reflected in decreases in age-standardised DALY rates attributable to low birthweight and short gestation, with high rates of decline (ARC decrease of >3·2%) in the regions of Andean Latin America and north Africa and the Middle East. Similarly, rates of decline in DALYs attributable to child growth failure were highest in the Caribbean; Oceania; and in central, eastern, southern, and western regions of sub-Saharan Africa (figure 4).

Declines in age-standardised DALY rates attributable to air pollution, the second-ranked Level 2 risk factor, between 2000 and 2021 can be seen in Andean Latin America, central Asia, eastern Europe, parts of east Asia, southeast Asia, and sub-Saharan Africa, but challenges remain in south Asia, sub-Saharan Africa, and parts of east Asia (figure 3C,D). Rates of decline between 2000 and 2021 for the age-standardised burden attributable to Level 3 particulate matter pollution were especially high (ARC decrease of >3·2%) in the regions of central Asia, central Europe, eastern Europe, western Europe, Andean Latin America, central and tropical Latin America, and east Asia. The burden attributable to particulate matter pollution declined between 2000 and 2021 in other regions, including south Asia, but at a slower rate (figure 4).

Declines over time in the burden attributable to high SBP, the third-ranked Level 2 risk factor, appear relatively limited in geographical scope, occurring primarily in central Asia, eastern Europe, and tropical Latin America (figure 3E,F). Although age-standardised DALY rates attributable to high SBP declined in 20 of 21 GBD regions (rising slightly in Andean Latin America; figure 4), high rates of decline in high SBP (ARC declines of >3·2%) occurred only in Australasia, high-income Asia Pacific, and western Europe, with increases or low declines elsewhere. As with SBP, the burden attributable to high LDL cholesterol declined in many regions between 2000 and 2021, with the highest rates of decline in Australasia and western Europe. Conversely, the burden attributable to high BMI and high FPG rose over this period in many regions, with the highest increases for high BMI (ARC increase of >1·2%) in south Asia, east Asia, southeast Asia, and central sub-Saharan Africa. The highest increases in the burden attributable to high FPG (ARC increase of >1·2%) occurred in north Africa and the Middle East, but increases were also high in other regions.

The burden attributable to tobacco, the fourth-ranked Level 2 risk factor, decreased between 2000 and 2021 throughout the world, notably in eastern Europe, central Asia, and east Asia, but remains a challenge in those and other areas (figure 3G,H). Decreases over the 2000–21 period in Level 3 risk-attributable DALYs to smoking—an important component of tobacco risk—were likewise high (ARC declines of 2·2–3·2%) in eastern Europe, central Asia, and east Asia, in addition to Australasia, high-income Asia Pacific, and western Europe. In east Asia, declines in the burden attributable to secondhand smoke were higher than the declining burden attributable to smoking.

The burden attributable to dietary risks, the fifth-leading Level 2 risk factor, declined in large areas of central Asia and eastern Europe between 2000 and 2021 (figure 3I,J), but the dietary burden remains concerning in these areas and parts of central Europe, and in countries such as Afghanistan and Yemen. High rates of decline (ARC decrease of >2·2%) in age-standardised risk-attributable DALY rates for many of the 15 diverse Level 3 dietary risk factors, from diet low in whole grains to diet high in sodium (figure 4) were seen in central and eastern Europe, in addition to Australasia, southern Latin America, and western Europe. High rates of decline (ARC decrease of >2·2%) in the burden attributable to diet high in sodium occurred in central Asia, eastern Europe, and high-income Asia Pacific. Other classes of risk factors related to unsafe WaSH and to unsafe sex also declined at high rates (ARC decrease of >3·2%) throughout sub-Saharan Africa (figure 4).

Detailed information on the change over time in estimates related to the attributable burden—DALYs and deaths—for each risk factor and outcome, across geography, are shown in appendix 2 (table S1).

### Burden of proof risk function assessments of effect size and strength of evidence

**Figure 7:**
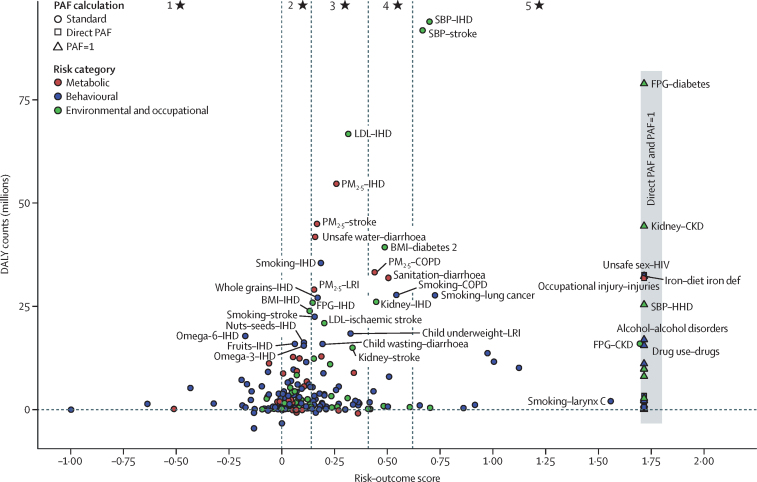
Global risk-attributable DALYs and risk–outcome score categorised by star rating for all risk–outcome pairs submitted to BPRF analysis, 2021 Risk–outcome score star ratings indicate a conservative assessment of the effect size and strength of evidence for each risk–outcome pair analysed using the BPRF framework. Each point represents a single risk–outcome pair, coloured by Level 1 risk factor category and shaped by type of PAF calculation. Risk–outcome pairs evaluated with direct PAFs and PAF=1 were not submitted to a BPRF analysis and thus did not receive a risk–outcome score or star rating. Risk–outcome pairs associated with more than 15 million attributable DALYs are labelled. BMI=high body-mass index. BPRF=burden of proof risk function. CKD=chronic kidney disease. COPD=chronic obstructive pulmonary disease. DALY=disability-adjusted life-year. Iron=iron deficiency. Diet iron def=dietary iron deficiency. FPG=high fasting plasma glucose. HHD=hypertensive heart disease. IHD=ischaemic heart disease. Larynx C=larynx cancer. LDL=high LDL cholesterol. LRI=lower respiratory infection. Occ injury=occupational injury. PAF=population attributable fraction. PM_2·5_=particulate matter pollution. SBP=high systolic blood pressure.

To complement conventional estimates of risk, we calculated risk–outcome scores, which quantify the effect size of an association and the strength of evidence for the effect (ie, the extent of between-study heterogeneity), and evaluated the relationship between attributable DALYs and risk–outcome scores. There was a positive relationship between the two values, indicating that risk–outcome pairs contributing the most to the overall attributable burden also had a stronger evidence of a risk–outcome association (figure 7; appendix 2 table S6). Of 211 risk–outcome pairs analysed by BPRF methods for GBD 2021, 12 (5·7%) were identified as having very strong (ie, five-star) relationships (appendix 2 table S6). The three five-star pairs responsible for the most risk-attributable DALYs in 2021 were high SBP–ischaemic heart disease (IHD; which contributed >75 million DALYs),^44^ high SBP–stroke (which contributed >75 million DALYs), and smoking–lung cancer^32^ (which contributed 25–50 million DALYs). 13 (6·1%) risk–outcome pairs were assessed as having strong (ie, four-star) associations and evidence. Five of those 13 risk–outcome pairs each contributed 25–50 million risk-attributable DALYs, with high BMI–type 2 diabetes generating the highest number of DALYs in the four-star group. 55 (26·0%) pairs were estimated as being three-star associations with moderate effect sizes and evidence strength; of those, high LDL cholesterol–IHD and particulate matter pollution–IHD contributed the most (50–75 million) attributable DALYs, and six others each contributed 25–50 million DALYs. 131 (62·1%) pairs were found to have weak relationships: 79 (37·4%) with two-star associations and 52 (24·6%) with one-star associations. None of the pairs in either of these groups contributed more than 25 million attributable DALYs. Relatively few risk–outcome pairs with more than 12·5 million attributable DALYs were rated as one-star or two-star pairs (appendix 2 table S6).

**Figure 8:**
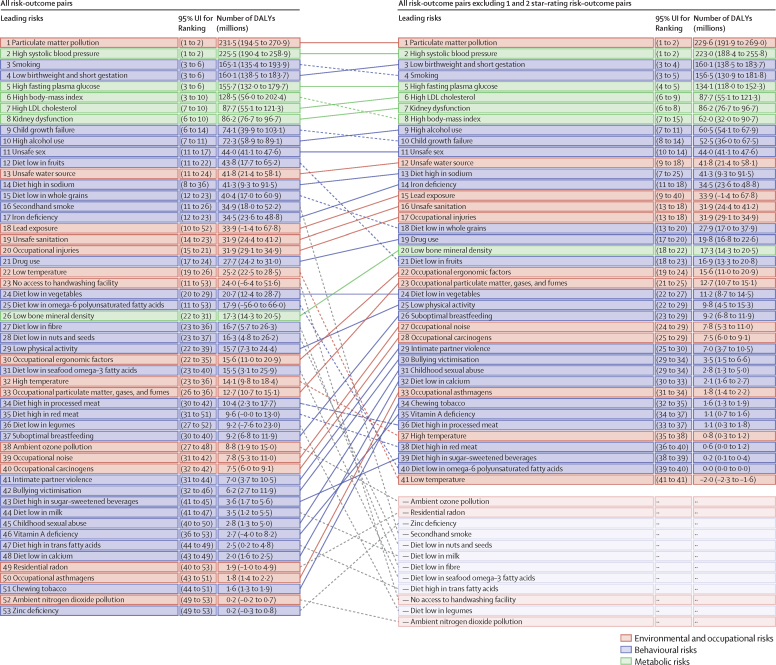
Level 3 risk factors rank ordered by risk-attributable DALYs inclusive of all GBD risk–outcome pairs versus GBD risk–outcome pairs excluding one-star and two-star associations, 2021 Each column displays Level 3 risk factors in descending order by risk-attributable DALYs. Risk factors for which no risk–outcome pairs have a better than two-star association are indicated in the right column with lighter shading and no attributable DALYs. One-star and two-star associations are those that are either or both weakly associated or lacking strong evidence, based on BPRF analysis. Risk factors are connected by lines, with solid lines representing an increase or lateral shift in risk-attributable burden ranking and dashed lines representing a decrease in rank. A number of risk factors—including low birthweight and short gestation, low bone mineral density, childhood sexual abuse, intimate partner violence, suboptimal breastfeeding, and all occupational risks—have not yet been submitted to BPRF analysis, and therefore no associated DALYs were removed due to low star rating. BPRF=burden of proof risk function. DALY=disability-adjusted life-year. UI=uncertainty interval.

To highlight risk factors for which there is a strong and consistent evidence base and those for which there is not a strong or consistent evidence base of robust health effects, according to available data, a secondary analysis was conducted that excluded one-star and two-star risk–outcome pairs. Because most of these pairs do not contribute large numbers of risk–attributable DALYs, the overall effect of excluding them was relatively modest, with the number of risk-attributable DALYs as a proportion of total DALYs (with total DALYs inclusive of estimated COVID-19 DALYs^33^) decreasing from 41·4% to 37·5% and DALYs unattributed to any risk factors rising from 51·4% to 55·2% when excluding one-star and two-star pairs (appendix 2 figure S4). This analysis found that various Level 3 risk factors in the top 20 for risk-attributable DALY counts in 2021, when calculated inclusive of all pairs, dropped considerably in ranking after removing one-star and two-star pairs, suggesting an absence of strong evidence for at least some of the health effects associated with these risk factors (figure 8). These include high BMI, for which attributable DALY counts declined from 128·5 million (95% UI 56·0–202·4) to 62·0 million (32·0–90·7); diet low in fruits, decreasing from 43·8 million (17·7–65·2) to 16·9 million (13·3–20·8); diet low in whole grains, declining from 40·4 million (17·0–60·9) to 27·9 million (17·0–37·9); and secondhand smoke, decreasing from 34·9 million (18·0–52·2) to no longer contributing risk-attributable DALYs. Conversely, the comparison reinforces the large health impact of Level 3 risk factors such as particulate matter pollution, high SBP, smoking, high FPG, high LDL cholesterol, kidney dysfunction, and high alcohol use, which remained leading contributors to risk-attributable DALYs even after dropping low-star-rated risk–outcome pairs from consideration. Other notable Level 3 risk factors that dropped considerably after removing low-star-rated risk–outcome pairs from the analysis include low temperature, no access to handwashing facility, and a large number of dietary risk factors, indicating the need for further research on the health effects associated with these risk factors.

Estimates related to the attributable burden—PAFs, DALYs, and deaths for each risk factor and outcome—that were made by excluding risk–outcome pairs rated as one star or two stars based on BPRF analysis are shown in appendix 2 (table S2). Maps of age-standardised DALY rates attributable to all risk factors combined, by location, based on datasets including all risk–outcome pairs and on all pairs exclusive of those rated with one or two stars using BPRF analysis are also presented in appendix 2 (figure S3).

## Discussion

### Main findings

This study presents comprehensive estimates of the risk-attributable burden for 204 countries and territories from 1990 to 2021. At the global level, particulate matter pollution, high SBP, smoking, low birthweight and short gestation, high FPG, and high BMI were the largest contributors to the burden in 2021, with considerable variation across ages, sexes, and locations. Broadly, the 2000–21 period saw sustained progress in reducing the number of global all-age DALYs attributable to environmental and occupational as well as behavioural risks, with approximately 20% reductions for both groups, while the number of DALYs attributable to metabolic risks increased by nearly 50% over the same period, reflecting global changes in demographics and lifestyle. The greatest declines in the risk-attributable burden occurred for risk factors related to maternal and child health and unsafe water, sanitation, and handwashing, due largely to decreases in risk exposure but also to proportionally smaller infant and youth populations. Among risk factors related to the leading Level 3 risks, the steepest increases in the risk-attributable burden occurred for ambient particulate matter air pollution and for risk factors related to obesity and metabolic syndrome—particularly high FPG and high BMI—due to a combination of population ageing and increasing risk exposure.

### Reducing the risk-attributable burden

Reducing the risk-attributable burden requires understanding the differing impact of specific risk factors, such as those under the broader umbrella of air pollution. The two forms of particulate matter pollution—the leading Level 3 contributor to the burden in 2021—are household air pollution and ambient particulate matter air pollution. Although global SEVs and risk-attributable DALYs for household air pollution decreased substantially between 2000 and 2021, they increased for ambient particulate matter pollution and ambient ozone pollution. Consequently, steep declines in the burden attributable to household air pollution in south Asia and China, for example, have been accompanied by increases in ambient particulate matter air pollution and ozone pollution, with some populations in these locations facing the substantial burden attributable to all three risk factors. Although it is tempting to suggest that these trends simply reflect a transfer of attributable burden from one risk factor to another, in the case of residential energy sources, the introduction of coupled policies has shown that success is possible on multiple fronts simultaneously. For example, SEVs decreased in China between 2015 and 2021 for both household (nearly 40%) and ambient particulate matter air pollution (approximately 9%; appendix 2 table S3). Indeed, one approach to improve ambient air pollution in Beijing has been a ban on the use of coal for residential energy in the surrounding region.^45^ As success with reducing both forms of particulate matter air pollution has been observed in China, there is also emerging evidence suggesting a recent peak in ambient air pollution in India coinciding with success in reducing household air pollution.^46^, ^47^ Introduction of a new air pollution risk factor in GBD 2021—nitrogen dioxide, a marker for motor vehicle pollution—adds another dimension to the air pollution picture. In contrast to other air pollution risk factors, SEVs for nitrogen dioxide are highest but decreasing in high, high-middle, and middle SDI locations, while they are increasing in low and low-middle SDI locations. This pattern reflects the high levels of vehicle use in higher SDI locations relative to low and low-middle SDI locations, while SEV trends in rapidly developing locations reflect the combination of overall increasing vehicle use with differing levels of adoption of lower-emissions vehicles.

The second-leading Level 3 contributor to the risk-attributable burden in 2021—high SBP—rose from fourth position in 2000, and along with high FPG and high BMI, shows a concerning trend of substantial burden attributable to key metabolic risks. The burden attributable to high SBP represents a continuing challenge and remains particularly impactful outside of most high-income countries, with reductions in burden limited in geographical scope. High FPG and high BMI stand out as risks for which both exposure and burden have increased considerably in almost all regions of the world, with the burden attributable to high BMI increasing with increasing SDI. Given projected increases in rates for outcomes such as type 2 diabetes^48^ and those related to high BMI (eg, musculoskeletal disorders^33^, ^49^), concerted policy actions addressing obesity and metabolic syndrome should be high priorities. Such actions could include evidence-based prevention efforts, treatment, or upstream socioeconomic policies to reduce underlying DALY rates.^50^ Balancing risk exposure reduction with other approaches might be more beneficial in cases where a single risk factor contributes to multiple outcomes (eg, low physical activity, high SBP, high BMI, and high FPG), especially when clear and compelling evidence exists of the effectiveness of specific interventions.

Smoking was the third-leading risk factor for all-age disease burden globally and a leading risk factor across most geographies and sociodemographic levels in 2021. Although actions by governmental agencies, multilateral institutions, and non-governmental organisations focused on tobacco control have contributed to a nearly 35% reduction in the age-standardised rate of global DALYs attributable to smoking over the period 2000–21, persistent and sustained action is needed to further reduce the burden of smoking, a highly consequential risk factor that can be addressed with proven interventions such as tobacco control policies, enforcing bans on tobacco advertising and sponsorship, encouraging current smokers to quit, and smoke-free policies.^51^

Low birthweight and short gestation remained the fourth-leading Level 3 contributor to all-age DALYs in 2021, despite major improvements in the risk-attributable burden related to child and maternal malnutrition and health over the study period, including a more than 70% reduction in the rate of age-standardised DALYs attributable to child growth failure; a nearly 35% decrease in the age-standardised DALY rate attributable to low birthweight and short gestation; and declines of more than 65% for risk factors related to unsafe WaSH. This reflects the major role of child and maternal risk factors in the overall global disease burden. In sub-Saharan Africa, south Asia, and parts of southeast Asia, child and maternal malnutrition remained the leading Level 2 risk factor for attributable DALYs in 2021. In these locations, there is a high need for evidence-based, locally relevant policies addressing the many complex factors that affect malnutrition, in order to maintain and further accelerate reductions in child and maternal malnutrition exposure.^52^ First, countries must implement or expand food and nutrition policies that have been developed with the best available evidence to maximise effectiveness.^53^ Second, beyond nutrition-based interventions, countries should consider and address the many other multifaceted factors influencing malnutrition, including increased access to health-care services; improved WaSH; and female education and empowerment.^54^, ^55^, ^56^, ^57^, ^58^

No specific Level 3 dietary risks were among the leading contributors to the burden either globally or in specific SDI quintiles or super-regions, yet—in aggregate—Level 2 dietary risks were a leading risk factor globally throughout the study period. This pattern was sustained despite broad reductions in the attributable burden due to improvements in eastern and central Europe and central Asia. Among specific leading dietary risk factors such as diets low in fruit, whole grains, and vegetables and diet high in sodium, there was a consistent pattern of increases in attributable DALY counts but an opposing decrease in age-standardised DALY rates, due to population growth and ageing. Specific dietary risks also contribute, via mediation, to multiple metabolic risk factors such as high FPG and high SBP that are leading contributors to the global burden.

By decomposing temporal patterns in risk factor exposure, population growth and ageing, and trends in underlying disease burden, we were able to broadly group risk factors into three categories. Category I includes risks such as maternal and childhood risk factors, household air pollution, and diets high in trans fatty acids for which the demonstrated reductions in risk-attributable burden have been substantial but not equitably distributed. There have been especially large decreases in the SEV for trans-fat, highlighting the effectiveness of trans-fat bans that have been implemented in a growing number of locations. For this and other category I risk factors, existing effective actions should be maintained, although the ability to sustain improvements could be challenged by other forces such as economic instability, conflict, absence of trust, and climate change. Category II includes many dietary risk factors for which exposure reduction actions have been successful but not sufficient to overcome demographic trends, particularly in older populations, and more action is required to counter demographic shifts. Category III entails those risk factors for which exposure has continued to increase since 2000, contributing to large increases in the risk-attributable burden. When combined with demographic and disease burden trends, insufficient actions to date to reduce exposure to this category of risk factors portend concern for the future. Category III risk factors include ambient particulate matter air pollution, drug use, and a group of risk factors related to the ongoing epidemics of obesity and metabolic syndrome: metabolic risks (including high BMI, high FPG, and high SBP), low physical activity, and diet high in sugar-sweetened beverages, which have been shown in decomposition analyses to be associated with the highest percentage increase in risk-attributable DALYs between 2000 and 2021. Taxing sugar-sweetened beverages is one strategy used to reduce the associated burden, but other policies such as public health campaigns to increase awareness of health risks might also be appropriate.^59^, ^60^

A new methodological component introduced in GBD 2021, BPRF analysis, offers an additional lens through which to prioritise actions and brings nuance to GBD estimates. For the first time, we present two distinct views of GBD results: one consistent with previous releases in which all considered risk–outcome pairs have been included, and an alternative view in which those with low risk–outcome scores—indicating weaker evidence or lower effect sizes, or both, based on a conservative interpretation of the evidence—have been excluded (one-star and two-star pairs). This secondary analysis suggests that for most of the leading risk factors with the highest attributable burden—such as particulate matter pollution, high SBP, smoking, high FPG, high LDL cholesterol, kidney dysfunction, child growth failure, and high alcohol use—the supporting evidence of their effects on specific health outcomes is also strong, strengthening the case that continued action is necessary. For some of these risk factors (eg, particulate matter pollution), actions will largely be related to public policy, whereas for others (eg, smoking), a combination of clinical guidance, individual action, and public policy are warranted. More research is needed on risk factors and risk–outcome pairs with a high attributable burden but low risk–outcome scores. Research should focus on one-star, two-star, and three-star pairs with more than 12·5 million risk-attributable DALYs. Research could also focus on risk factors and risk–outcome pairs with low risk–outcome scores but of specific geographical importance, such as child wasting and diarrhoea, as well as particulate matter pollution and lower respiratory infection. BPRF analysis can also be used as a transparent methodology to assess potential expansion of additional risk factors, such as pesticides and other chemical pollutants, to consider for inclusion in future GBD cycles.

The GBD risk factor analysis covers a broad array of risk factors including a complex and interconnected web of distal (eg, environmental and social), proximal (eg, diet and smoking), and biochemical (eg, FPG) risk factors. The BPRF scoring for specific risk–outcome pairs can be used as a standardised method to compare impacts across this diverse range and to prioritise research. Distal risk factors might be more amenable to upstream systemic change, whereas more proximal risks might be targets for individual control and clinical guidance. Prioritisation of policy action should be informed on the basis of all outcomes paired with a risk factor and the underlying DALY rate for the associated outcomes, as described by the attributable burden. For example, a three-star risk–outcome pair (eg, unsafe water and diarrhoea) with a very common outcome is a greater priority for public health than a three-star pair for a less common outcome (eg, diet high in sodium and stomach cancer). Furthermore, application of the precautionary principle would indicate that public health policy should still apply to risk factors with lower star ratings (eg, diet high in sugar-sweetened beverages), especially those with multiple outcomes. Risk factors for which a high proportion of the population is exposed (eg, ambient particulate matter air pollution) could also be a higher priority for public health policy than risk factors for which exposure prevalence is lower (eg, secondhand smoke), while also considering temporal and spatial variation in risk factor exposure. Although our analysis of burden and risk factor prioritisation is primarily relevant to public policy actions, our BPRF analysis and risk–outcome scores could also be useful for clinical guidance and individual behavioural actions to reduce risk factor exposure by highlighting those relationships for which the evidence is strongest.^34^

### Climate change: direct and indirect impacts

GBD 2021 partially captures the disease burden attributable to climate change via the high temperature risk factor, the most direct pathway through which climate change can affect health.^61^, ^62^ SEVs and DALYs attributable to high temperature increased modestly between 2000 and 2021. High temperature accounted for 0·5% of total global DALYs in 2021, contributing to 2·5% or more total DALYs in Saudi Arabia, Oman, Mauritania, and Iraq.^33^ Global attributable deaths due to high temperature in 2021 reached nearly 450 000 (appendix 2 table S1). The impact of high temperatures was modest in 2021 compared to that of other risk factors, including low temperatures, which had nearly double the number of attributable DALYs. While SEVs for high temperature increased between 2000 and 2021, the decrease in low temperature SEVs correspondingly decreased but to a much lesser degree. Beyond the direct impacts of temperature, climate change has been viewed as a crucial public health challenge and potentially presents an opportunity for health improvements if mitigation and adaptation actions are taken.^63^ Indirect effects of climate change, whether mediated by other GBD risk factors or via other pathways, have not yet been included in the GBD methodology, in part due to challenges associated with attribution of changes in risk factor exposure (eg, increases in ambient ozone air pollution and wildfires) or specific outcomes (eg, malaria and dengue), given their multifactorial nature, especially in the context of sociodemographic and health trends. For example, existing analyses of malaria suggest that climate-driven increases are likely to be restricted in geographical scope with relatively small impacts on burden.^64^ Furthermore, quantitative attribution of extreme weather events to climate change remains challenging, but if future warming leads to more frequent or severe events, or both, direct impacts on disease burden (eg, deaths due to drowning during floods) can be expected, whereas indirect effects such as reductions in safe drinking water and sanitation could be compromised. Additionally, climate change has disrupted and will continue to disrupt agricultural production.^65^ Although global undernutrition continues to decrease and this trend is unlikely to reverse, specific locations strongly affected by warm temperatures and large increases in extreme weather events (droughts or floods) could experience increases in wasting, but not stunting.^58^ The magnitude of future impacts on disease burden due to the direct effects of temperature, as well as those related to food security, extreme weather events, and rise in sea levels must also be considered in the context of population growth in areas most stressed by climate change and the extent to which populations may out-migrate from such areas. Along with climate change itself, actions taken to mitigate the emissions contributing to climate change could also indirectly affect disease burden and are the subject of substantial research. For example, actions to reduce fossil fuel combustion for energy and transportation are expected to lead to decreases in air pollution that would consequently decrease the attributable burden.^66^ Efforts to shift populations to more climate-friendly diets could also lead to reductions in the disease burden attributable to specific dietary factors such as diets low in whole grains and legumes.^67^

### Limitations

The GBD 2021 risk factor findings are limited by several considerations, including the omission of various potentially consequential risk factors and covariates. Importantly, the impact of the COVID-19 pandemic was not formally incorporated or quantified across risk factors or health outcomes, although COVID-19-related effects were included in the analyses for some risk–outcome pairs involving mental health, flu, pertussis, and malaria outcomes. As more information becomes available, future iterations of GBD could quantify risk factors for the COVID-19 burden, such as high BMI.^68^ Furthermore, drug use as well as stress, anxiety, depression, and other mental health conditions correlated with drug use increased sharply during the pandemic,^33^ but these changes have not yet been fully captured in the available data. Importantly, although there was no risk attribution for COVID-19, the overall numbers of deaths and non-fatal outcomes for risk attribution were smaller in 2021 than they would have been in the absence of COVID-19, as COVID-19 is likely to have accounted for a proportion of deaths that would have occurred due to other outcomes.

Another important limitation of this study is the inconsistent availability and variable quality of data to estimate RRs and risk exposure, with considerable disparities in risk exposure across socioeconomic factors that are further exacerbated in areas affected by violence and conflict.^69^, ^70^, ^71^ An additional limitation is that, although our methods to estimate RRs provide a standardised mechanism to code and test for bias, this functionality is constrained to the extent that differences across study-level characteristics are not always fully known or accurately described in individual studies. Moreover, more nuanced bias coding (eg, using dummy variables to expand to multiple categories) is possible in the current methodology, but is dependent on the availability of sufficient data in the input studies. Standardisation of our bias adjustment processes is ongoing and will be updated for future GBD rounds. While we applied new BPRF methods to 211 risk–outcome pairs—a strength of this study—we were unable to apply this analysis to all applicable pairs for GBD 2021. The BPRF work is ongoing, and the methods will be extended to additional risk–outcome pairs in the future. For one-star and two-star risk–outcome pairs, the evidence base is less consistent across studies and is likely to change in response to ongoing research.

To assess the joint effects of risk factors, our analyses were adjusted to account for the assumption that RRs are multiplicative. Known pathways in which one risk factor (eg, diet low in fruits) was mediated through another risk factor (eg, diet low in fibre) were incorporated into the estimation process. We computed non-mediated RRs and then assumed that non-mediated RRs are multiplicative to avoid overestimation of joint effects. One limitation of this approach is that it does not capture the possibility that some combinations of RRs might be super-multiplicative or sub-multiplicative. Given the centrality of nutrition in public policy discourses, as well as the large evidence base that diet-based interventions can produce positive health outcomes, more detailed work is needed to strengthen the scientific understandings of mediation. On estimating TMREL, we were confronted with several limitations. We generally assumed that the TMREL is 0 for harmful risks with monotonically increasing risk functions. However, for protective risks such as fruit or whole grain intake, selecting the minimum risk level of exposure required more careful analysis because extrapolating the risk function outside the range of where the available literature supports the protective effect could lead to both exaggerated estimates of attributable burden and implausible levels of consumption. We therefore set the TMREL for protective risks to be equal to the 85th percentile of exposure in the available cohorts and trials. A population-level study on red meat consumption is an example of how these proposed improvements and modifications to TMREL estimations improve outputs across the risk factor estimation process.^31^

Last, we faced challenges in achieving the generalisability of estimated risk–outcome relationships across time and place. We assumed in most cases that RRs as a function of exposure are universal; based on this assumption, RR functions apply to all locations and time periods. However, we did not make this assumption for all risk functions. Temperature is one exception. The risk functions of temperature depend on the annual mean temperature. Additionally, we did not assume RR functions for all locations and time periods for high BMI and breast cancer because of known differences between Asian and non-Asian populations.^72^ GBD methods require clear and substantive evidence of significant differences in the RR for different subgroups; based on such rules, few cases met this standard. We continue to assess the evidence of the RRs for different populations and will identify and incorporate more location-specific or sub-group RRs. Indeed, cases where risk–outcome relationships indicate substantial between-study heterogeneity suggest a need for further evaluation of the sources of this heterogeneity (eg, location or sub-group).

### Future directions

This analysis identifies a group of specific risk factors for which there is consistent evidence of strong risk–outcome relationships, which currently contribute considerably to the disease burden across all levels of SDI, and for which the risk exposure is either increasing or the declines are insufficient to reduce the attributable burden in the face of growing and ageing populations. These results therefore provide ample rationale for accelerated policy action on these risk factors. However, given that our analysis attributes 41·4% of total global DALYs to risk-included factors, a substantial proportion of currently unattributed disease burden remains. In addition to our eventual expansion of the BPRF methodology to all relevant risk–outcome pairs, future iterations of GBD will need to expand the scope of risk factors, especially for specific outcomes that are large and growing contributors to disease burden. For example, musculoskeletal disorders account for 5·6% of global DALYs,^33^ but only 20·5% of this burden is attributable to risk factors currently included in GBD. Similarly, mental disorders are responsible for 5·4% of the global burden, but only 8·0% of mental disorders are attributable to risk factors.^33^

Adding additional risk factors for these and other outcomes will require sufficient information on risk factor exposure and sufficiently strong evidence of a risk–outcome relationship. The BPRF methodology introduced in this GBD cycle provides a transparent and efficient approach for evidence scoring. Advances in artificial intelligence for summarising the literature and extracting relevant information to feed into this methodology could also accelerate the evaluation of new risk factors. Although many of the risk factors included here could mediate the impact of social determinants on disease burden, the inclusion of more distal social determinants of health as risk factors in GBD needs further development. For example, low educational attainment can be a strong determinant of health, at a level of attributable burden similar to the impacts of diet, physical activity, smoking, or alcohol.^73^ Accumulating data also suggest the plausibility of quantifying DALYs attributable to genetic risk factors for certain diseases, including major causes such as ischaemic heart disease, type 2 diabetes, and chronic obstructive pulmonary disease.^74^ Furthermore, greater attention is now being placed on commercial determinants of health such as tobacco and alcohol companies, the fossil fuel industry, and producers of ultra-processed foods, all of which can affect risk factor exposure and which suggest additional aggregation approaches are needed.^75^ Inclusion of distal risk factors such as education or commercial determinants in future iterations of GBD is likely to provide additional guidance to policy makers, including those sectors outside of health. However, inclusion of additional risk factors and especially such upstream factors will be challenging given the need for detailed understanding of complex mediation relationships. Numerous other individual risk factors such as sleep-related disorders, stress, and exposure to UV radiation, environmental noise, and heavy metals have been considered but are not yet included in the analysis. Multimorbidity, particularly in older age groups in whom health effects from exposure to multiple risk factors are more likely to occur, is another important factor to consider in future iterations of GBD.

### Conclusion

Attribution of disease burden to risk factors can help guide prioritisation of actions. Considering both the overall contribution to disease burden, trends in attributable burden, and the strength of evidence relating risk factor exposure to specific outcomes, we identified a highly consistent group of risk factors for which actions have been insufficient. Ambient particulate matter air pollution, high SBP, smoking, and high FPG are not only among the five leading risk factors globally but are also in the top three ranking risk factors for nearly all levels of SDI, suggesting a need for renewed and increased attention to exposure reduction. Low birthweight and short gestation is the leading risk factor at the lowest level of SDI and requires continued action to extend the reductions in attributable burden observed since 2000. By contrast, high BMI is the leading risk factor at the highest level of SDI. With increasing risk exposure—compounded by interactions with metabolic risk factors such as high FPG, high SBP, low physical activity, and diet high in sugar-sweetened beverages—there is an urgent need for interventions focused on obesity and metabolic syndrome. More generally, among the ten leading risk factors globally—each contributing to at least 2·5% of total global DALYs—all except child growth failure and low birthweight and short gestation have shown risk factor exposure trends from 2000 to 2021 that indicate inadequate action has been taken to reduce the attributable burden. Furthermore, all risk factors contributing the most to the attributable burden are supported by strong evidence of their association with specific outcomes, although in the case of high BMI the magnitude of the attributable burden changes when the strength of evidence for individual risk–outcome pairs is considered, suggesting a high-priority need for additional research on BMI–outcome relationships. Future iterations of GBD will continue to track levels and trends in risk factors and their attributable burden, assimilate a growing literature into the burden of proof framework, and incorporate additional risk factors to aid in prioritisation of actions to reduce the disease burden.

## Data sharing

To download the data used in these analyses, please visit the Global Health Data Exchange GBD 2021 website at http://ghdx.healthdata.org/gbd-2021/sources.

## Declaration of interests

S Afzal reports support for the present manuscript from King Edward Medical University, which provided study material, research articles, valid data sources, and authentic real-time information for this manuscript; payment or honoraria for lectures, presentations, speakers bureaus, manuscript writing, or educational events and webinars with King Edward Medical University and collaborative partners including University of Johns Hopkins, University of California, University of Massachusetts, KEMCAANA, KEMCA UK international scientific conferences, webinars, and meetings; support for attending meetings or travel, or both, from King Edward Medical University; participation on a data safety monitoring board or advisory board with the National Bioethics Committee Pakistan, King Edward Medical University Ethical Review Board, and the Ethical Review Board (Fatima Jinnah Medical University and Sir Ganga Ram Hospital); leadership or fiduciary roles in board, society, committee or advocacy groups, paid or unpaid with the Pakistan Association of Medical Editors and as a Fellow of Faculty of Public Health (Royal Colleges, UK), Society of Prevention, Advocacy And Research, King Edward Medical University (SPARK), and as a Member of the Pakistan Society of Infectious Diseases; other financial or non-financial interests as Dean of Public Health and Preventive Medicine (King Edward Medical University), Chief Editor Annals (King Edward Medical University), Director of Quality Enhancement Cell (King Edward Medical University), and a Fellow of Faculty of Public Health United Kingdom, an Advisory Board Member and Chair Scientific Session, KEMCA-UK Chairperson International Scientific Conference, KEMCAANA (at national level), Member Research and Publications Higher Education Commission, HEC Pakistan, Member Research and Journals Committee (Pakistan Medical and Dental Council), Pakistan Member National Bioethics Committee, Pakistan (at Punjab level), Member Corona Experts Advisory Group, a Member of the Dengue Experts Advisory Group, and Chair of the Punjab Residency Program Research Committee; outside the submitted work. E Ammirati reports grants or contracts from Italian Ministry of Health (GR-2019-12368506) and NextGenerationEU (PNRR-MAD-2022-12376225); consulting fees from Cytokinetics, Kiniksa, and AstraZeneca; outside the submitted work. R Ancuceanu reports consulting fees from AbbVie, payment or honoraria for lectures, presentations, speakers bureaus, manuscript writing, or educational events from AbbVie, Sandoz, B Braun, and Laropharm; outside the submitted work. J Ärnlöv reports payment or honoraria for lectures, presentations, speakers bureaus, manuscript writing, or educational events from AstraZeneca; and participation on a data safety monitoring board or advisory board with AstraZeneca and Astella; outside the submitted work. P Atorkey reports support from the present manuscript from the Australian College of Applied Professions (Discipline of Psychological Sciences, Sydney, Australia) and the School of Medicine and Public Health (The University of Newcastle, Australia). J L Baker reports grants or contracts from the World Cancer Research Fund, Novo Nordisk Foundation, and the Independent Research Fund Denmark; Consulting feeds from Novo Nordisk A/S Denmark; leadership or fiduciary roles in board, society, committee or advocacy groups, unpaid as an executive member of the European Association for the Study of Obesity; outside the submitted work. O C Baltatu reports support for the present manuscript from the National Council for Scientific and Technological Development (CNPq, 304224/2022-7) and Anima Institute (AI research professor fellowship); leadership or fiduciary roles in board, society, committee or advocacy groups, paid or unpaid with São José dos Campos Technology Park as a Biotech Board Member; outside the submitted work. T W Bärnighausen reports grants or contracts from the US National Institutes of Health (NIH), Alexander von Humboldt Foundation, German National Research Foundation (DFG), European Union, German Ministry of Education and Research, German Ministry of the Environment, Wellcome Trust, and KfW; payment or honoraria for lectures, presentations, speakers bureaus, manuscript writing, or educational events from PLOS Medicine as the editor-in-chief; participation on a data safety monitoring board or advisory board for NIH-funded research projects in Africa on climate change and health; and stock ownership in CHEERS; outside the submitted work. S Barteit reports grants or contracts from Carl-Zeiss Foundations and the German Research Foundation; and stock or stock options with CHEERS; outside the submitted work. Y Béjot reports grants or contracts from Medtronic paid to their institution; consulting fees from Medtronic, NovoNordisk, and Novartis; payment or honoraria for lectures, presentations, speakers bureaus, manuscript writing, or educational events from Bristol Myers Squibb, Pfizer, Servier, Amgen, and Medtronic; support for attending meetings or travel, or both, from Medtronic; and leadership or fiduciary roles in other board, society, committee or advocacy group, unpaid with Société Française Neurovasculaire; outside the submitted work. M L Bell reports grants or contracts from the US Environmental Protection Agency (EPA), NIH, High Tide Foundation, Health Effects Institute, Yale Women Faculty Forum, the Environmental Defense Fund, Wellcome Trust Foundation, Yale Climate Change and Health Center, Robert Wood Johnson Foundation, and the Hutchinson Postdoctoral Fellowship; consulting fees from Clinique; honoraria for lectures, presentations, speakers bureaus from Colorado School of Public Health, Duke University, University of Texas, Data4Justice, Korea University, Organization of Teratology Information Specialists, UPenn, and Boston University; honorarium for editorial duties from IOP Publishing; honorarium for grant review from NIH, Health Canada, PAC-10, UK Research and Innovation, and the AXA Research Fund Fellowship; honoraria for external advisory committee from Harvard University and the University of Montana; support for attending meetings or travel, or both, from the Colorado School of Public Health, University of Texas, Duke University, Boston University, UPenn, Harvard University, and the American Journal of Public Health; leadership or fiduciary roles in other board, society, committee or advocacy groups, unpaid with Fifth National Climate Assessment, the *Lancet* Countdown, Johns Hopkins EHE Advisory Board, Harvard external advisory committee for training grants, WHO Global Air Pollution and Health Technical Advisory group, and the National Academies Panels and Committees; leadership or fiduciary roles in other board, society, committee, or advocacy groups paid from the US EPA Clean Air Scientific Advisory Committee (CASAC); all outside the submitted work. L Belo reports support from Fundacao para a Ciencia e a Tecnologia in the scope of the project UIDP/04378/2020 and UIDB/04378/2020 of UCIBIO and the project LA/P/0140/2020 of i4HB; outside the submitted work. A Beloukas reports grants or contracts from Gilead and GSK; payment or honoraria for lectures, presentations, speakers bureaus, manuscript writing, or educational events from Gilead and GSK; support for attending meetings or travel, or both, from Gilead; receipt of equipment, materials, drugs, medical writing, gifts or other services from Cepheid; outside the submitted work. P G Bettencourt reports patents planned, issued, or pending: WO2020229805A1, EP3965809A1, US2023173050A1, EP4275700A2, BR112021022592A2, OA1202100511, EP4265271A2, EP4265271A3; other financial or non-financial support as a project reviewer at the Botnar Foundation; outside the submitted work. S Bhaskar reports grants or contracts from Japan Society for the Promotion of Science (JSPS), Japanese Ministry of Education, Culture, Sports, Science and Technology (MEXT), Grant-in-Aid for Scientific Research (KAKENHI), and JSPS and the Australian Academy of Science (JSPS International Fellowship); leadership or fiduciary roles in board, society, committee or advocacy groups, paid or unpaid with Rotary District 9675 as the District Chair, Diversity, Equity, and Inclusion; Global Health & Migration Hub Community, Global Health Hub Germany (Berlin, Germany) as the Chair and Manager; PLOS One, BMC Neurology, Frontiers in Neurology, Frontiers in Stroke, Frontiers in Public Health, and BMC Medical Research Methodology as an Editorial Board Member; and the College of Reviewers, Canadian Institutes of Health Research (CIHR), Government of Canada as a member; outside the submitted work. B Bikbov reports grants or contracts from the European Commission, University of Rome, and Politecnico di Milano; support for attending meetings or travel, or both, from the European Renal Association; leadership or fiduciary roles in board, society, committee, or advocacy groups, unpaid with the Advocacy Group of the International Society of Nephrology and the Western Europe Regional Board of the International Society of Nephrology; and other financial or non-financial support from Scientific-Tools.Org; outside the submitted work. A Biswas reports consulting fees from Lupin Pharmaceuticals (India), Alkem Laboratories (India), and Intas Pharmaceuticals (India); payment or honoraria for lectures, presentations, speakers bureaus, manuscript writing, or educational events from Roche Diagnostics (India); outside the submitted work. C S Brown reports other financial or non-financial interests from ad-hoc one-off market research advisory, on a variety of infection topics, all anonymously conducted via market research companies with no direct communication nor any knowledge of any pharmaceutical companies or products (and none specifically related to global burden of disease); outside the submitted work. M Carvalho reports other financial or non-financial interests from LAQV/REQUIMTE, University of Porto (Porto, Portugal) and acknowledges support from FCT/MCTES under the scope of the project UIDP/50006/2020 (DOI 10.54499/UIDP/50006/2020); outside the submitted work. A L Catapano reports grants or contracts from Amryt Pharma, Menarini, and Ultragenyx; consulting fees from Eli Lilly, Menarini Ricerche, and Sanofi; payment or honoraria for lectures, presentations, speakers bureaus, manuscript writing, or educational events from Amarin, Amgen, Amryt Pharma, AstraZeneca, Daiichi Sankyo, Esperion, Ionis Pharmaceuticals, Medscaper, Menarini, Merck, Novartis, NovoNordisk, PeerVoice, Pfizer, Recordati, Regeneron, Sandoz, Sanofi, The Corpus, Ultragenyx, and Viatris; participation on a data safety monitoring board or advisory board with Amarin, Amgen, Amryt Pharma, AstraZeneca, Daiichi Sankyo, Esperion, Ionis Pharmaceuticals, Medscaper, Menarini, Merck, Novartis, NovoNordisk, PeerVoice, Pfizer, Recordati, Regeneron, Sandoz, Sanofi, The Corpus, Ultragenyx, and Viatris; outside the submitted work. C R Cederroth reports support for attending meetings or travel, or both, from the Mérida Institute of Technology to present at the 18th international symposium from the AMCAOF at Mérida (Mexico), March 6–9, 2024; leadership or fiduciary roles in board, society, committee, or advocacy groups, paid or unpaid with the Professional Advisers Committee from the Tinnitus UK as a member, and with the Scientific Advisory Committee of the American Tinnitus Association as a member; outside the submitted work. J S Chandan reports grants or contracts from the National Institute of Health Research Youth Endowment Fund (Home Office) and the College of Policing, University of Birmingham (UK Research and Innovation); support for attending meetings or travel, or both, from the University of Miami; outside the submitted work. S Cortese reports grants or contracts from the National Institute of Health Research and the European Research Agency; and payment or honoraria for lectures, presentations, speakers bureaus, manuscript writing, or educational events from CADDRA, Medice, BAP, and ACAMH; outside the submitted work. L L Dalli reports support for the present manuscript from the National Heart Foundation of Australia (research fellowship support). L Degenhardt reports grants or contracts from Indivior and Seqirus. A Faro reports support for the present manuscript from the National Council for Scientific and Technological Development, CNPq (Brazil). I Filip reports financial support from Avicenna Medical and Clinical Research Institute; outside the submitted work. D Flood reports grants or contracts from the US National Heart, Lung, and Blood Institute (NHLBI; award number K23HL161271), the University of Michigan Claude D. Pepper Older Americans Independence Center (award number 5P30AG024824), and the University of Michigan Caswell Diabetes Institute Clinical Translational Research Scholars Program; consulting fees as a diabetes consultant for WHO; leadership or fiduciary roles in board, society, committee, or advocacy groups, unpaid as a staff physician for Maya Health Alliance, a non-governmental health organization in Guatemala; outside the submitted work. L M Force reports support for the present manuscript from the Bill & Melinda Gates Foundation; grants or contracts from the Conquer Cancer Foundation, St. Jude Children's Research Hospital, St. Baldrick's Foundation, and NIH Loan Repayment Program; and leadership or fiduciary roles in board, society, committee, or advocacy groups, unpaid as a member of the *Lancet Oncology* International Advisory Board; outside the submitted work. M Foschi reports consulting fees from Novartis and Roche; support for attending meetings or travel, or both, from Novartis, Roche, Biogen, Sanofi, Merck, and Bristol Myers Squibb; leadership or fiduciary roles in board, society, committee, or advocacy groups, paid or unpaid with MSBase Collaboration as a member of the scientific leadership group; outside the submitted work. R C Franklin reports grants or contracts from Heatwaves in Queensland – Queensland Government, Arc Flash – Human Factors – Queensland Government, and Mobile Plant Safety – Agrifutures; support for attending meetings or travel, or both, from ACTM – Tropical Medicine and Travel Medicine Conference 2022 and ISTM – Travel Medicine Conference, Basel 2023; leadership or fiduciary roles in board, society, committee, or advocacy groups, paid or unpaid with Kidsafe as a Director, Auschem as a Director, the International Society for Agricultural Safety and Health (ISASH) on the Governance Committee, Farmsafe as a Director, and the Public Health Association of Australia (PHAA) as the Injury Prevention SIG Convenor; outside the submitted work. R K Garg reports royalties or licenses for writing book chapters for UpToDate (Wolters Kluwer Health), MedLink Neurology USA, and Elsevier (publisher); outside the submitted work. G F Gil reports support for the present manuscript from Bloomberg Philanthropies and the Bill & Melinda Gates Foundation (through the Institute for Health Metrics and Evaluation); support for attending meetings or travel, or both, from The University of Washington; outside the submitted work. P S Gill reports support for the present manuscript from the National Institute of Health Research (UK). N G M Gomes reports grants or contracts from Fundação para a Ciência e Tecnologia (Research Contract 2022.07375.CEECIND); outside the submitted work. A Guha reports grants or contracts from the American Heart Association and the US Department of Defense; consulting fees from Pfizer, Novartis, and Myovant; leadership or fiduciary roles in board, society, committee, or advocacy groups, paid or unpaid with ZERO Prostate Cancer on the health equity task force and Doctorpedia as a founding medical partner; outside the submitted work. A Hassan reports consulting fees from Novartis, Sanofi Genzyme, Biologix, Merck, Hikma Pharma, Janssen, Inspire Pharma, Future Pharma, Elixir Pharma; payment or honoraria for lectures, presentations, speakers bureaus, manuscript writing, or educational events from Novartis, Allergan, Merck, Biologix, Janssen, Roche, Sanofi Genzyme, Bayer, Hikma Pharma, Al Andalus, Chemipharm, Lundbeck, Inspire Pharma, Future Pharma and Habib Scientific Office, and Everpharma; support for attending meetings or travel, or both, from Novartis, Allergan, Merck, Biologix, Roche, Sanofi Genzyme, Bayer, Hikma Pharma, Chemipharm, and Al Andalus and Clavita pharmaceuticals; leadership or fiduciary roles in other board, society, committee or advocacy groups, paid or unpaid as a member of educational, membership and regional committees of international headache societies; outside the submitted work. C Herteliu reports grants or contracts from the Romanian Ministry of Research Innovation and Digitalization, MCID (project number ID-585-CTR-42-PFE-2021), and a grant from the European Commission Horizon 4P-CAN (Personalized Cancer Primary Prevention Research through Citizen Participation and Digitally Enabled Social Innovation); outside the submitted work. M Hultström reports support for the present manuscript from Knut och Alice Wallenberg Foundation, Swedish Heart-Lung Foundation, Swedish Association Foundation; payment or honoraria for lectures, presentations, speakers bureaus, manuscript writing, or educational events from Swedish Society for Anaesthesiology and Intensive Care; support for attending meetings or travel, or both, from the American Physiological Society; leadership or fiduciary roles in board, society, committee, or advocacy groups, paid or unpaid with the American Physiological Society; outside the submitted work. I Ilic and M Ilic report support for the present manuscript from the Ministry of Education, Science and Technological development, Republic of Serbia (project numbers 175042, 2011–2023 and 451-03-47/2023-01/200111). N E Ismail reports leadership or fiduciary roles in board, society, committee or advocacy groups, unpaid as the Bursar and Council Member of the Malaysian Academy of Pharmacy (Malaysia); outside the submitted work. J J Jozwiak reports payment or honoraria for lectures, presentations, speakers bureaus, manuscript writing, or educational events from Novartis, ADAMed, and Amgen; outside the submitted work. E M Kahn reports grants or contracts from the US National Institute on Minority Health and Health Disparities, NIH (contract #75N94023C00004); support for attending meetings or travel, or both, from the Bill & Melinda Gates Foundation; all outside the submitted work. S V Katikireddi reports funding from the Medical Research Council (MC_UU_00022/2), the Scottish Government Chief Scientist Office (SPHSU17), and the European Research Council (949582); outside the submitted work. N Kawakami reports grants or contracts from the Junpukai Foundation; consulting fees from Riken Institute, JAXA, Sekisui Chemicals, SB@ Work; leadership or fiduciary roles in board, society, committee or advocacy groups, paid or unpaid with the Japan Society for Occupational Health; outside the submitted work. J H Kempen reports stock or stocks options with Betaliq and Tarsier; outside the submitted work. K Krishan reports non-financial support from the UGC Centre of Advanced Study, CAS II, awarded to the Department of Anthropology (Panjab University, Chandigarh, India); outside the submitted work. B Langguth reports grants or contracts from the German Research Foundation, German Bundesministerium für Bildung und Forschung, European Union, Bavarian-Czech University Association, Bavarian State; consulting fees from Schwabe, Neuromod, Sea Pharma, and Rovi; payment or honoraria for lectures, presentations, speakers bureaus, manuscript writing, or educational events from Schwabe and Neuromod; payment for expert testimony from Bavarian State; leadership or fiduciary roles in board, society, committee, or advocacy groups, paid or unpaid with the German Society for Brain Stimulation; stock or stocks options from Sea Pharma; receipt of equipment or materials from Deymed; outside the submitted work. M Lee reports support for the present manuscript from the Ministry of Education of the Republic of Korea and the National Research Foundation of Korea (NRF-2023S1A3A2A05095298) and Bio-convergence Technology Education Program through the Korea Institute for Advancement Technology (KIAT) funded by the Ministry of Trade, Industry and Energy (No. P0017805); outside the submitted work. M-C Li reports grants or contracts from the National Science and Technology Council, Taiwan (NSTC 112-2410-H-003-031); and leadership or fiduciary roles in board, society, committee or advocacy groups, paid or unpaid as the Technical Editor of the Journal of the American Heart Association; outside the submitted work. W-Z Li reports support for the present manuscript from the Postdoctoral Fellowship Program of CPSF (GZB20230179) and the Grant of State Key Laboratory of Respiratory Disease (SKLRD-Z-202401). R Lui reports grants or contracts from the National Institute of Mental Health (grants R01 MH115905, RF1 MH120830, R01 MH124899, R21 MH130767); consulting fees from Relmada Therapeutics; payment or honoraria for lectures, presentations, speakers bureaus, manuscript writing or educational events from Miami International Child and Adolescent Mental Health Conference, Massachusetts General Hospital, and University of California (San Francisco, CA, USA); support for attending meetings or travel, or both, from the America Foundation for Suicide Prevention; participation on a data safety monitoring board for the University of Pennsylvania, the University of Minnesota, and Massachusetts General Hospital; outside the submitted work. S Lorkowski reports grants or contracts from DSM Nutritional Products; consulting fees from Danone, Novartis Pharma, and Swedish Orphan Biovitrum (SOBI); payment or honoraria for lectures, presentations, speakers bureaus, manuscript writing, or educational events from Akcea Therapeutics Germany, AMARIN Germany, Amedes Holding, Amgen, Berlin-Chemie, Boehringer Ingelheim Pharma, Daiichi Sankyo Deutschland, Danone, Hubert Burda Media Holding, Janssen-Cilag, Lilly Deutschland, Novartis Pharma, Novo Nordisk Pharma, Roche Pharma, Sanofi-Aventis, SYNLAB Holding Deutschland, and SYNLAB Akademie; support for attending meetings or travel from Amgen; participation on a data safety monitoring board or advisory board with Akcea Therapeutics Germany, Amgen, Daiichi Sankyo Deutschland, Novartis Pharma, and Sanofi-Aventis; outside the submitted work. L G Mantovani reports support for the present manuscript from the Italian Ministry of Health (Ministero della Salute, Ricerca Corrente, IRCCS Istituto Auxologico Italiano). H R Marateb reports grants or contracts from Universitat Politècnica de Catalunya-Barcelona Tech (UPC), research funded by the Beatriu de Pinós post-doctoral programme from the Office of the Secretary of Universities and Research from the Ministry of Business and Knowledge of the Government of Catalonia programme (2020 BP 00261); outside the submitted work. A-F A Mentis reports grant or contract funding from “MilkSafe: a novel pipeline to enrich formula milk using omics technologies”, research co-financed by the European Regional Development Fund of the European Union and Greek national funds through the Operational Program Competitiveness, Entrepreneurship and Innovation, under the call RESEARCH - CREATE - INNOVATE (project code: T2EDK-02222), as well as from ELIDEK (Hellenic Foundation for Research and Innovation, MIMS-860); payment for expert testimony having served as external peer-reviewer for FONDAZIONE CARIPLO (Italy); participation on data safety monitoring board or advisory board as Editorial Board Member for *Systematic Reviews*, and *Annals of Epidemiology*, and as Associate Editor for *Translational Psychiatry*; stock or stock options from a family winery; other financial or non-financial support as a scientific officer (BGI Group); outside the submitted work. S A Meo reports grant or contract support from Researchers Supporting Project, King Saud University (Riyadh, Saudi Arabia) (RSP-2024 R47); outside the submitted work. T R Miller reports grants or contracts from AB InBev Foundation; payment or expert testimony in state and local opioid litigation; outside the submitted work. L Monasta and L Ronfani report support for the present manuscript from the Italian Ministry of Health (Ricerca Corrente 34/2017), and payments made to the Institute for Maternal and Child Health IRCCS Burlo Garofolo. C E Moore reports participation on a data safety monitoring board or advisory board as a member of an advisory board for MRC grant (no payments made), a member of the WHO Advisory Group and the REVIVE Advisory Group; leadership or fiduciary roles in board, society, committee, or advocacy groups, unpaid as a Co-Chair of the Impact and Influence Group in the Microbiology Society; outside the submitted work. R S Moreira reports grants or contracts from CNPq Research Productivity Scholarship (scholarship registration number is 316607/2021-5); outside the submitted work. S Nomura reports support for the present manuscript from the Ministry of Education, Culture, Sports, Science and Technology of Japan (21H03203), Precursory Research for Embryonic Science and Technology from the Japan Science and Technology Agency (JPMJPR22R8), and the National Cancer Center Research and Development Fund (2021-A16). B Norrving reports participation on a data safety monitoring board or advisory board with a data safety monitoring board for HOVID (trial, Simbec-Orion); outside the submitted work. A P Okekunle reports support for the present manuscript from the National Research Foundation of Korea funded by the Ministry of Science and ICT (2020H1D3A1A04081265); support for attending meetings or travel, or both, from the National Research Foundation of Korea funded by the Ministry of Science and ICT (2020H1D3A1A04081265); outside the submitted work. R Ornello reports grants or contracts from Novartis; consulting fees from Teva, AbbVie, and Eli Lilly; payment or honoraria for lectures, presentations, speakers bureaus, manuscript writing or educational events from Teva, AbbVie, Pfizer, and Eli Lilly; support for attending meetings or travel, or both, from Teva and Eli Lilly; participation on a data safety monitoring board or advisory board with Eli Lilly, AbbVie, and Pfizer; leadership or fiduciary roles in other board, society, committee, or advocacy groups, paid or unpaid as a Junior Editorial Board Member of the *Journal of Headache and Pain*, and Associate Editor for the section Headache and Neurogenic Pain with *Frontiers in Neurology*; and receipt of equipment, materials, drugs, medical writing, gifts, or other services from Pfizer; outside the submitted work. A Ortiz reports grants from Sanofi and grants or contracts from the Catedra Mundipharma-UAM for diabetic kidney disease and the Catedra AstraZeneca-UAM for chronic kidney disease and electrolytes (paid to Universidad Autonoma de Madrid); consulting fees from Advicciene, Astellas, AstraZeneca, Amicus, Amgen, Fresenius Medical Care, GSK, Bayer, Sanofi-Genzyme, Menarini, Kyowa Kirin, Alexion, Idorsia, Chiesi, Otsuka, Novo-Nordisk, and Vifor Fresenius Medical Care Renal Pharma; payment or honoraria for lectures, presentations, speakers bureaus, manuscript writing, or educational events from Advicciene, Astellas, AstraZeneca, Amicus, Amgen, Fresenius Medical Care, GSK, Bayer, Sanofi-Genzyme, Menarini, Kyowa Kirin, Alexion, Idorsia, Chiesi, Otsuka, Novo-Nordisk, and Vifor Fresenius Medical Care Renal Pharma; support for attending meetings or travel, or both, from Advicciene, Astellas, AstraZeneca, Fresenius Medical Care, Bayer, Sanofi-Genzyme, Menarini, Chiesi, and Otsuka; participation on a data safety monitoring board or advisory board with Astellas, AstraZeneca, Fresenius Medical Care, Bayer, Sanofi-Genzyme, Idorsia, Chiesi, and Otsuka; leadership or fiduciary roles in other board, society, committee or advocacy groups, unpaid on the Council of the European Renal Association and SOMANE; outside the submitted work. R Passera reports participation on a data safety monitoring board or advisory board for the non-profit clinical trial “Consolidation with ADCT-402 (loncastuximab tesirine) after immunochemotherapy: a phase II study in BTKi-treated/ineligible Relapse/Refractory Mantle Cell Lymphoma (MCL) patients” - sponsor FIL, Fondazione Italiana Linfomi, Alessandria-I; leadership or fiduciary roles in other board, society, committee or advocacy groups, unpaid as a member of the Statistical Committee of the EBMT, the European Society for Bone and Marrow Transplantation, Paris, a Member of the COST CA18218 working group - European Burden of Disease Network (burden-eu), the European Cooperation in Science & Technology, Brussels; outside the submitted work. A E Peden reports support for the present manuscript from the Australian National Health and Medical Research Council (Grant Number: APP2009306). V C F Pepito reports grants or contracts from Sanofi Consumer Healthcare and the International Initiative for Impact Evaluation; outside the submitted work. M Pigeolet reports grants or contracts from The Belgian Kids’ Fund for Pediatric Research; outside the submitted work. C D Pond reports grants or contracts from Valley to Coast Charitable Trust as payments to the University of Newcastle; consulting fees from HNECC Primary Health Network, SW Sydney Primary Health Network, Australian Department of Health and Aged Care, NSW Health, Royal Australian College of General Practitioners, Dementia Training Australia, Palliative Care Australia, University of Sydney, Monash University, Biogen, Roche, Medicines Australia, Dementia Training Australia, Sydney North Health Network, and In Vivo Academy; payment or honoraria for lectures, presentations, speakers bureaus, manuscript writing or educational events from Dementia Training Australia, Sydney North Health Network, and In Vivo Academy; payment for expert testimony from Legal Aid NSW; support for attending meetings or travel, or both, from the Royal Australian College of General Practitioners and Palliative Care Australia; leadership or fiduciary roles in other board, society, committee or advocacy groups, paid or unpaid as Provost, NSW Faculty, RACGP, Vice President, Doctors Reform Society, Chair, WONCA Special Interest Group, Ageing and Health, Board Member, Hunter Postgraduate Medical Institute, Adjunct Professor, School of Rural Medicine, University of New England, Adjunct Professor, School of Nursing and Midwifery, Western Sydney University, Clinical Professor, Wicking Dementia Research Education Centre, University of Tasmania, and Professor of General Practice, University of Newcastle (until August, 2021); all outside the submitted work. A Radfar reports other financial or non-financial interests in Avicenna Medical and Clinical Research Institute through their financial and logistical support, outside the submitted work. A Rane reports stock or stock options with Agios Pharmaceuticals as a full-time employee, outside the submitted work. A Ranta reports grants or contracts from NZ Health Research Council and NZ Ministry of Health; participation on a data safety monitoring board or advisory board with Phase II, Multicenter, Double-Blinded, Randomized, Placebo-Controlled, Parallel-Group, Single-Dose Study to Determine the Safety, Preliminary Efficacy, and Pharmacokinetics of ARG-007 in Acute Ischemic Stroke Patients; leadership or fiduciary roles in other board, society, committee, or advocacy groups, paid or unpaid with Australia and NZ Stroke Organization, World Stroke Organization, and NZ Stroke Foundation; outside the submitted work. L F Reyes reports grants or contracts from GSK; royalties for licences from GSK; consulting fees from GSK; payment or honoraria for lectures, presentations, speakers bureaus, manuscript writing, or educational events from GSK; payment for expert testimony from GSK; support for attending meetings or travel, or both, from GSK and Pfizer; and stock or stock options from GSK; outside the submitted work. S Sacco reports grants or contracts from Novartis and Uriach; consulting fees from Novartis, Allergan-AbbVie, Teva, Lilly, Lundbeck, Pfizer, NovoNordisk, Abbott, and AstraZeneca; payment or honoraria for lectures, presentations, speakers bureaus, manuscript writing, or educational events from Novartis, Allergan-AbbVie, Teva, Lilly, Lundbeck, Pfizer, NovoNordisk, Abbott, AstraZeneca; support for attending meetings or travel, or both, from Lilly, Novartis, Teva, Lundbeck, and Pfizer; leadership or fiduciary roles in other board, society, committee or advocacy groups, paid or unpaid as the President elect European Stroke Organization and Editor-in-Chief of *Cephalalgia*; receipt of equipment, materials, drugs, medical writing, gifts or other services from Allergan-AbbVie and NovoNordisk; outside the submitted work. P S Sachdev reports grants or contracts from the National Health and Medical Research Council of Australia and the NIH; payment or honoraria for lectures, presentations, speakers bureaus, manuscript writing, or educational events from Alkem Labs; participation on a data safety monitoring board or advisory board with Biogen Australia Medical Advisory Committee in 2020 and the 2021 Roche Australia Medical Advisory Committee in 2022; leadership or fiduciary roles in other board, society, committee or advocacy groups, unpaid with VASCOG Society on the executive committee and the World Psychiatric Association on the planning committee; outside the submitted work. Y L Samodra reports grants or contracts from Taipei Medical University; leadership or fiduciary roles in other board, society, committee, or advocacy groups, paid or unpaid as co-founder of Benang Merah Research Center; all outside the submitted work. J Sanabria reports support for attending meetings or travel, or both, from the Continuing Medical Education section of the University of Marshall School of Medicine; one patent issued and one patent pending; participation on a data safety monitoring board or advisory board with the Marshall University Department of Surgery; leadership or fiduciary roles in other board, society, committee, or advocacy groups, paid or unpaid, with the American Society of Transplant Surgeons, Society of Surgical Oncology, American Board of Surgery, Americas Hepato-Pancreato-Biliary Association, and International Hepato-Pancreato Biliary Association; all outside the submitted work. A E Schutte reports grants or contracts from the National Health and Medical Research Council of Australia (Investigator Grant); consulting feeds from Abbott, Medtronic, Servier, and Skylabs; payment or honoraria for lectures, presentations, speakers bureaus, manuscript writing, or educational events from Abbott, Medtronic, Servier, Skylabs, Aktiia, Sanofi, Omron, and Novartis; support for attending meetings or travel, or both, from Medtronic and Servier; leadership or fiduciary roles in other board, society, committee or advocacy groups, paid or unpaid as Co-Chair of the National Hypertension Taskforce of Australia, Secretary of the Australian Cardiovascular Alliance, Board Member of Hypertension Australia; outside the submitted work. A Sharifan reports leadership or fiduciary roles in other board, society, committee or advocacy groups, paid or unpaid from Cochrane; receipt of equipment, materials, drugs, medical writing, gifts, or other services from Elsevier; outside the submitted work. V Sharma acknowledges support from DFSS (MHA)'s research project (DFSS28(1)2019/EMR/6) at the Institute of Forensic Science & Criminology, Panjab University (Chandigarh, India); outside the submitted work. V Shivarov reports one patent pending and one utility model with the Bulgarian Patent Office; stock or stock options from RSUs with ICONplc; and a salary from ICONplc; outside the submitted work. S Shrestha reports support from the School of Pharmacy, Monash University Malaysia and the Graduate Research Merit Scholarship; outside the submitted work. C R Simpson reports grants or contracts from the Health Research Council of New Zealand, Ministry of Health (New Zealand), Ministry of Business, Innovation, and Employment (New Zealand), Chief Scientist Office (UK), and MRC (UK); leadership or fiduciary roles in other board, society, committee or advocacy groups, paid or unpaid as Data Ethics Advisory Group Chair for the New Zealand Government; outside the submitted work. J A Singh reports consulting fees from Schipher, Crealta/Horizon, Medisys, Fidia, PK Med, Two Labs, ANI Pharmaceuticals/Exeltis USA, Adept Field Solutions, Clinical Care options, ClearView Healthcare Partners, Putnam Associates, Focus Forward, Navigant Consulting, Spherix, MedIQ, Jupiter Life Science, UBM, Trio Health, Medscape, WebMD, and Practice Point Communications; and the NIH and the American College of Rheumatology; payment or honoraria for lectures, presentations, speakers bureaus, manuscript writing, or educational events as a member of the speaker's bureau of Simply Speaking; support for attending meetings or travel, or both, as a past steering committee member of OMERACT; participation on a data safety monitoring board or advisory board with the US Food and Drug Administration (FDA) Arthritis Advisory Committee; leadership or fiduciary roles in other board, society, committee or advocacy groups, paid or unpaid as a past steering committee member of the OMERACT, an international organization that develops measures for clinical trials and receives arm's length funding from 12 pharmaceutical companies, Chair of the Veterans Affairs Rheumatology Field Advisory Committee, and editor/Director of the UAB Cochrane Musculoskeletal Group Satellite Center on Network Meta-analysis; stock or stock options in Atai Life Sciences, Kintara Therapeutics, Intelligent Biosolutions, Acumen Pharmaceuticals, TPT Global Tech, Vaxart Pharmaceuticals, Aytu BioPharma, Adaptimmune Therapeutics, GeoVax Labs, Pieris Pharmaceuticals, Enzolytics, Seres Therapeutics, Tonix Pharmaceuticals Holding, Aebona Pharmaceuticals, and Charlotte's Web Holdings, and previously owned stock options in Amarin, Viking, and Moderna Pharmaceuticals; outside the submitted work. S T Skou reports grants or contracts from the European Research Council, European Union's Horizon 2020 research innovation programme, Region Zealand; Royalties or licences from Munksgaard and TrustMe-Ed; payment or honoraria for lectures, presentations, speakers bureaus, manuscript writing, or educational events from Nestlé Health Science; other financial support as a co-founder of GLA:D; outside the submitted work. R Somayaji reports grants or contracts through clinical research funding from the Canadian Institutes of Health Research, Cystic Fibrosis Foundation, Vertex Pharmaceuticals, and the University of Calgary; payment or honoraria for lectures, presentations, speakers bureaus, manuscript writing or educational events from educational events from Vertex Pharmaceuticals; participation on a data safety monitoring board or advisory board with Oncovir and the Cystic Fibrosis Foundation; leadership or fiduciary roles in other board, society, committee or advocacy groups, paid or unpaid, with the Canadian Pressure Injury Advisory Panel; all outside the submitted work. D J Stein reports personal fees from Discovery Vitality, Johnson & Johnson, Kanna, L’Oreal, Lundbeck, Orion, Sanofi, Servier, Takeda, and Vistagen; outside the submitted work. J H V Ticaolu reports leadership or fiduciary roles in other board, society, committee, or advocacy group, paid or unpaid, with the Benang Merah Research Center as co-founder, outside the submitted work. F Topouzis reports grants or contracts from Thea, Omikron, Pfizer, Alcon, AbbVie, and Bayer; consulting fees from Omikron, Thea, and Bausch and Lomb; payment or honoraria for lectures, presentations, speakers bureaus, manuscript writing, or educational events from Omikron, AbbVie, and Roche; leadership or fiduciary roles in other board, society, committee or advocacy groups, paid or unpaid, with the European Glaucoma Society, Greek Glaucoma Society, and World Glaucoma Association; all outside the submitted work. S J Tromans reports grants or contracts from NHS Digital via the Department of Health and Social Care; leadership or fiduciary roles in other board, society, committee or advocacy groups, paid or unpaid, with the Neurodevelopmental Psychiatry Special Interest Group and the Royal College of Psychiatrists; all outside the submitted work. E Upadhyay reports patents planned, issued, or pending: “A system and method of reusable filters for anti-pollution mask” (published), “A system and method for electricity generation through crop stubble by using microbial fuel cells” (published), “A system for disposed personal protection equipment (PPE) into biofuel through pyrolysis and method” (published), “A novel herbal pharmaceutical aid for formulation of gel and method thereof” (published), “Herbal drug formulation for treating lung tissue degenerated by particulate matter exposure” (filed), “A method to transform cow dung into the wall paint by using natural materials and composition thereof” (filed); leadership or fiduciary roles in other board, society, committee or advocacy groups, paid or unpaid as Joint Secretary of Indian Meteorological Society, Jaipur Chapter (India), Member Secretary of the DSTPURSE Program; outside the submitted work. P Willeit reports consulting fees from Novartis Pharmaceuticals; outside the submitted work. Y Yasufuku reports grants or contracts from Shionogi & Co; outside the submitted work. M Zielińska reports other financial or non-financial interests in AstraZeneca as their employee, outside the submitted work. A Zumla reports support for the present manuscript from the Pan-African Network on Emerging and Re-Emerging Infections (PANDORA-ID-NET) funded by the EDCTP - the EU Horizon 2020 Framework Programme, the UK NIHR Senior Investigator Award, Mahathir Science Award and EU-EDCTP Pascoal Mocumbi Prize Laureate; participation on a data safety monitoring board or advisory board as a member of the Scientific Expert Committee of the EC-EDCTP-Global Health Program; outside the submitted work. All other authors declare no competing interests.
