# Department of Error

**DOI:** 10.1016/S0140-6736(24)01458-2

**Published:** 2024-07-20

*GBD 2021 Risk Factors Collaborators. Global burden and strength of evidence for 88 risk factors in 204 countries and 811 subnational locations, 1990–2021: a systematic analysis for the Global Burden of Disease Study 2021.* Lancet *2024; **403:** 2162–203*—In this Article, the Risk category key in figure 7 has been updated with the correct colours. This correction has been made to the online version as of July 18, 2024.

