# Supplementary material for: Global burden and strength of evidence for 88 risk factors in 204 countries and 811 subnational locations, 1990–2021: a systematic analysis for the Global Burden of Disease Study 2021
Source: Lancet. 2024 May 18;403(10440):2162–203. doi: 10.1016/S0140-6736(24)00933-4 (PMC11120204; doi:10.1016/S0140-6736(24)00933-4)
Supplement: Supplementary appendix 1 [file mmc1.pdf]

THE LANCET

Supplementary appendix 1

This appendix formed part of the original submission and has been peer reviewed. We post it as supplied by the authors.

Supplement to: GBD 2021 Risk Factors Collaborators. Global burden and strength of evidence for 88 risk factors in 204 countries and 811 subnational locations, 1990–2021: a systematic analysis for the Global Burden of Disease Study 2021. *Lancet* 2024; **403:** 2162–203.

Appendix 1: Methods appendix to "Global burden and strength of evidence for 88 risk factors in 204 countries and territories and 811 subnational locations, 1990–2021: a systematic analysis for the Global Burden of Disease Study 2021"

Preamble

This appendix provides further methodological detail for "Global burden and strength of evidence for 88 risk factors in 204 countries and territories and 811 subnational locations, 1990–2021: a systematic analysis for the Global Burden of Disease Study 2021." This study complies with the Guidelines for Accurate and Transparent Health Estimates Reporting (GATHER) recommendations.[1] It includes detailed tables and information on data in an effort to maximise transparency in our estimation processes and provide a comprehensive description of analytical steps. We intend this appendix to be a living document, to be updated with each iteration of the Global Burden of Disease Study.

Portions of this appendix have been reproduced or adapted from the appendices of Lim et al 2012,[2] GBD 2015 Risk Factors Collaborators,[3] GBD 2016 Risk Factors Collaborators,[4] GBD 2017 Risk Factor Collaborators,[5] and GBD 2019 Risk Factors Collaborators.[6] References are provided for reproduced or adapted sections.

Contents

Preamble......2

List of methods appendix figures and tables 5

Section 1: GBD overview 6

Section 1.1: Global Burden of Diseases, Injuries, and Risk Factors Study 2021 6

Section 1.2: Geographical locations of the analysis...... 6

Section 1.3: Time period of the analysis 6

Section 1.4: Statement of GATHER compliance...... 6

Section 1.5: GBD risk factor hierarchy 6

Section 1.6: List of abbreviations 7

Section 1.7: Data input sources overview...... 9

Section 1.8: Funding sources 9

Section 2: Risk factor estimation 9

Overview 9

Step 1. Effect size estimation 10

Section 2.1.1: Criteria for inclusion of risk–outcome pairs[5]...... 10

Section 2.1.2: Overview of the effect size estimation pathway. 11

Section 2.1.3: Collate relative risk data 11

Section 2.1.4: Estimating the shape of the risk–outcome relationship. 16

Section 2.1.5: Quantifying between-study heterogeneity; accounting for heterogeneity, uncertainty, and small numbers of studies 21

Section 2.1.6: Estimating the burden of proof risk function...... 21

Section 2.1.7: Evaluating potential for publication or reporting bias...... 22

Section 2.1.8: MR-BRT and temperature 23

Step 2. Exposure estimation[5] 23

Section 2.2.1: Collate exposure data *Systematic reviews* 23

Section 2.2.2: Adjust exposure data...... 24

Section 2.2.3: Estimate exposure 27

Step 3. TMREL[5] 37

Step 4. Estimate population attributable fractions[5] 37

Step 5. Estimate summary exposure values[5]...... 38

Step 6. Mediation[5] 39

Section 2.6.1: Summary...... 39

Section 2.6.2: Calculating the burden of multiple risk factors 40
Section 2.6.3: Computing mediation factors using linear relationships 41
Section 2.6.4: Adjusting for mediation 41
Section 2.6.5: Calculating mediation factor 42
Section 2.6.6: Piecewise aggregation (Pattern 3) 43
Section 2.6.7: Uncertainty of aggregated and mediated PAFs 44
Section 2.6.8: Important assumptions in aggregating risk factors and including mediation 45
Step 7. Estimate attributable burden[5] 45
Section 3: Decomposition analysis of deaths and DALYs[5] 45
Section 4: Socio-demographic Index analysis[5] 47
Section 4.1: Development of the Socio-demographic Index 47
Section 4.2: Development of a revised SDI indicator 47
Section 5: References 49
Section 6: GBD 2021 risk factor-specific modelling descriptions 55
Section 7: Tables 436

List of methods appendix figures and tables

Table S1. GBD risk hierarchy with levels

Table S2. Types of comparative risk assessments (CRA) based on the time perspective and the nature of the counterfactual level or distribution of exposure

Table S3. Guidelines for Accurate and Transparent Health Estimates Reporting (GATHER) checklist

Table S4. GBD location hierarchy with levels

Table S5. Socio-demographic values for all estimated GBD 2021 locations, 1990–2021

Table S6. Mediation factor matrix

Table S7. Status of risk–outcome pairs considered for inclusion in GBD 2021: included in both GBD 2019 and GBD 2021, added in GBD 2021, or removed in GBD 2021

Table S8. Status of mediated risk–outcome pairs considered for inclusion in GBD 2021: included in both GBD 2019 and GBD 2021, added in GBD 2021, or removed in GBD 2021

Table S9. Theoretical minimum risk exposure levels (TMREL) changes compared to GBD 2019

Section 1: GBD overview

Section 1.1: Global Burden of Diseases, Injuries, and Risk Factors Study 2021

The Global Burden of Diseases, Injuries, and Risk Factors Study (GBD) is a collaborative research effort aimed at estimating morbidity and mortality from a comprehensive set of diseases, injuries, and risk factors. The GBD Collaborator Network draws on the expertise of over 10,000 contributors from around the world. For this paper, we estimated risk factor exposure levels, relative health risk by exposure, and risk-attributable burden by age, sex, and location from 1990 to 2021.

Section 1.2: Geographical locations of the analysis

We produced estimates for 204 countries and territories that were grouped into 21 regions and seven super-regions (table S4). The seven super-regions are central Europe, eastern Europe, and central Asia; high income; Latin America and the Caribbean; north Africa and the Middle East; south Asia; southeast Asia, east Asia, and Oceania; and sub-Saharan Africa. In GBD 2021, we continue to analyse at subnational levels countries that were added in previous cycles, including Brazil, China, Ethiopia, India, Indonesia, Iran, Italy, Japan, Kenya, Mexico, New Zealand, Nigeria, Norway, Pakistan, the Philippines, Poland, Russia, South Africa, Sweden, the United Kingdom, and the United States of America. All analyses are at the first level of administrative organisation within each country except for New Zealand (by Māori ethnicity), Sweden (by Stockholm and non-Stockholm), the Philippines (by provinces), and the UK (by local government authorities). Subnational estimates for these countries will be released in separate publications.

At the most detailed spatial resolution, we generated estimates for 983 unique locations. As was done in GBD 2019, in GBD 2021 we continue to use the set of locations defined as standard locations and non-standard locations. Standard GBD locations are defined as the set of all subnationals belonging to countries where data quality is high and with populations over 200 million, in addition to all other countries. Standard locations include the subnationals for China, India, the USA, and Brazil, but not Indonesia; data for China, India, the USA, and Brazil are also included at the country level. All other countries with subnational estimates are defined as non-standard locations.

Section 1.3: Time period of the analysis

A complete set of risk-specific exposures, relative risks (RRs), theoretical minimum risk exposure levels (TMRELs), and population attributable fractions (PAFs) were computed for the years 1990 to 2021.

Section 1.4: Statement of GATHER compliance

This study complies with the Guidelines for Accurate and Transparent Health Estimates Reporting (GATHER) recommendations.[1] We have documented the steps involved in our analytical procedures and detailed the data sources used. See table S3 for the GATHER checklist. The GATHER recommendations may be found here: http://gather-statement.org/.

Section 1.5: GBD risk factor hierarchy

The GBD 2021 risk factors hierarchy and levels are summarised in table S1. The risk hierarchy is based on common features of individual risks; for example, risk factors that represent behavioural factors are grouped together.

The GBD risk factor list continues to evolve to reflect the policy relevance, public health, and medical care importance of major risk factors. One risk was added to the list for GBD 2021: nitrogen dioxide air pollution.

Section 1.6: List of abbreviations

APCSC	Asia-Pacific Cohort Studies Collaboration
ARC	annualised rate of change
BMI	body-mass index
BPRF	Burden of Proof risk functions
BMD	bone mineral density
CDC	Centers for Disease Control and Prevention
CF	correction factor
CKD	chronic kidney disease
COD	causes of death
CODEm	Cause of Death Ensemble modelling
COPD	chronic obstructive pulmonary disease
COVID-19	coronavirus disease 2019
CRA	comparative risk assessment
CSA	childhood sexual abuse
CSV	comma-separated values
CRA	comparative risk assessment
CSMR	cause-specific mortality rate
CVD	cardiovascular disease
DALY	disability-adjusted life-year
DHS	Demographic and Health Survey
DRI	data representativeness index
EDU15+	mean education for those aged 15 years or older
EMR	excess mortality rate
FAO	Food and Agriculture Organization
FPG	fasting plasma glucose
GAM	generalised additive model
GATHER	Guidelines for Accurate and Transparent Health Estimates Reporting
GBD	Global Burden of Diseases, Injuries, and Risk Factors Study
GHDx	Global Health Data Exchange
GoF	goodness of fit
HAP	household air pollution
ID	iron deficiency
IDA	Iron-deficiency anaemia
IER	integrated exposure response
IHD	ischaemic heart disease
ILO	International Labour Organization
IPV	intimate partner violence
IQ	intelligence quotient
JMP	Joint Monitoring Project

KS	Kolmogorov-Smirnov
LDI	lag-distributed income
LDL	low-density lipoprotein
LMICs	low- and middle-income countries
LOESS	locally estimated scatterplot smoothing
LRI	lower respiratory infection
MCMC	Markov Chain Monte Carlo simulations
MDG	Millennium Development Goal
MF	mediation factor
MICS	Multiple Indicator Cluster Surveys
MoM	method of moments
MR-BRT	meta-regression—Bayesian, regularised, trimmed
NCD	non-communicable disease
NCD-RisC	Non-communicable Disease Risk Factor Collaboration
OER	observed-to-expected ratio
PAF	population attributable fraction
PDF	probability distribution factor
$PM_{2.5}$	particulate matter <2.5 μm in aerodynamic diameter
PRISMA	Preferred Reporting Items for Systematic Reviews and Meta-Analyses
PCS	prospective cohort study
RCT	randomised controlled trial
PURE	Prospective Urban and Rural Epidemiological Study
REDCap	Research Electronic Data Capture
RMSE	root mean square error
ROS	risk–outcome score
RR	relative risk
SARS-CoV 2	Severe acute respiratory syndrome coronavirus 2
SBP	systolic blood pressure
SD	standard deviation
SDG	Sustainable Development Goal
SDI	Socio-demographic Index
SEER	Surveillance, Epidemiology, and End Results Program
SEV	summary exposure value
SHS	secondhand smoke
SIR	smoking impact ratio
SSB	sugar-sweetened beverages
ST-GPR	spatiotemporal Gaussian process regression
TB	tuberculosis
TFU25	total fertility rate in those under 25 years old
TMREL	theoretical minimum risk exposure level
TSNA	tobacco-specific nitrosamines
UI	uncertainty interval
USD	United States dollars
WaSH	Water, sanitation, and handwashing

WCRF	World Cancer Research Fund
WHO	World Health Organization
YLDs	years lived with disability
YLLs	years of life lost

Section 1.7: Data input sources overview

GBD 2021 incorporated a large number and wide variety of input sources to estimate mortality, causes of death and illness, and risk factors for 204 countries and territories from 1990 to 2021. These input sources are accessible through an interactive citation tool available in the GHDx [https://ghdx.healthdata.org/].

Users can retrieve citations for a specific GBD component, cause or risk, and location by choosing from the available selection boxes. They can then view and access GHDx records for input sources and export a comma-separated value (CSV) file that includes the GHDx metadata, citations, and information about where the data were used in GBD. Additional metadata for each input source are available through the citation tool as required by the GATHER statement.

The citation tool is available online via the GBD 2021 Sources Tool in the GHDx [https://ghdx.healthdata.org/gbd-2021/sources].

Section 1.8: Funding sources

This publication and the research it presents were funded by the Bill & Melinda Gates Foundation; Bloomberg Philanthropies; the University of Melbourne; Queensland Department of Health, Australia; the National Health and Medical Research Council, Australia; Public Health England; the Norwegian Institute of Public Health; St. Jude Children's Research Hospital; the Cardiovascular Medical Research and Education Fund; the National Institute on Aging of the National Institutes of Health (award P30AG047845); and the National Institute of Mental Health of the National Institutes of Health (award R01MH110163). The funders of the study had no role in study design, data collection, data analysis, data interpretation, or writing of the report. All authors had full access to all data in the study and had final responsibility for the decision to submit for publication.

Section 2: Risk factor estimation

Overview

The comparative risk assessment (CRA) conceptual framework was developed by Murray and Lopez,[7] who established a causal web of hierarchically organised risks or causes that contribute to health outcomes, which allows for quantification of risks or causes at any level in the framework. In GBD 2021, as in previous iterations of the GBD study, we evaluated a set of behavioural, environmental and occupational, and metabolic risks, in which risk–outcome pairs were included based on evidence rules. These risks were organised in four hierarchical levels, where Level 1 represents the overarching categories (behavioural, environmental and occupational, and metabolic) nested within Level 1 risks; Level 2 contains both single risks and risk clusters (such as child and maternal malnutrition); Level 3 contains the disaggregated single risks from within Level 2 risk clusters (such as low birthweight and short gestation); and Level 4 details risks with the most granular disaggregation, such as for specific

occupational carcinogens, the subcomponents of child growth failure (stunting, wasting, underweight), and suboptimal breastfeeding (discontinued and non-exclusive breastfeeding). At each level of risk, we evaluated whether risk combinations were additive, multiplicative, or shared common pathways for intervention. This approach allows the quantification of the proportion of risk-attributable burden shared with another risk or combination of risks and the measurement of potential overlaps between behavioural, environmental and occupational, and metabolic risks. To date in GBD, we have not quantified the contribution of other classes of risk factors illustrated in table S2. We do provide some insights into the potential magnitude of distal social, cultural, and economic factors through an analysis of the relationship between risk exposures and development measured by using the Socio-demographic Index (SDI) (more details in section 4).

Two types of risk assessments are possible within the CRA framework: attributable burden and avoidable burden. Attributable burden is the reduction in current disease burden that would have been possible if past population exposure had shifted to an alternative or counterfactual distribution of risk exposure. Avoidable burden is the potential reduction in future disease burden that could be achieved by changing the current distribution of exposure to a counterfactual distribution of exposure. Murray and Lopez identified four types of counterfactual exposure distributions: (1) theoretical minimum risk; (2) plausible minimum risk; (3) feasible minimum risk; and (4) cost-effective minimum risk.[8] The theoretical minimum risk exposure level (TMREL) is the level of risk exposure that minimises risk at the population level. Other possible forms of risk quantification include plausible minimum risk and feasible minimum risk. Plausible minimum risk reflects the distribution of risk that is conceivably possible and would minimise population-level risk if achieved. Feasible minimum risk describes the lowest risk distribution that has been attained within a population, and cost-effective minimum risk is the lowest risk distribution for a population that can be attained in a cost-effective manner. Because no robust set of forecasts for all components of GBD is available, in this study we focus on quantifying attributable burden by using the theoretical minimum risk counterfactual distribution. Table S2 shows the eight possible types of risk quantification within the CRA framework; the grey box represents the type of CRA currently undertaken by the GBD study. According to the definition of avoidable burden, risk reversibility would be incorporated into this type of assessment because it would involve reducing risk to the counterfactual for the index year, given a history of past risk exposure. Given the focus in this study on attributable burden, risk reversibility is not a criterion used in estimation here.

In general, this analysis follows the CRA methods used since GBD 2015.[3] The methods described here provide a high-level overview of the analytical logic and focus on areas of notable change from the methods employed in GBD 2015 and since GBD 2019. Here we aim to provide sufficient detail on the methods and overall structure of the estimation process. This study complies with the GATHER recommendations proposed by the World Health Organization (WHO) and others, which include recommendations on documentation of data sources, estimation methods, and statistical analysis (table S3).[1]

Step 1. Effect size estimation

Section 2.1.1: Criteria for inclusion of risk–outcome pairs[5]

Beginning in GBD 2010, we included risk–outcome pairs that met the World Cancer Research Fund (WCRF) grades of convincing or probable evidence.[9] In this framework, convincing evidence consists of biologically plausible associations between exposure and disease established from multiple

epidemiological studies in different populations. Evidentiary studies must be substantial, include prospective observational studies, and, where relevant, randomised controlled trials (RCTs) of sufficient size, duration, and quality that show consistent effects. Probable evidence is similarly based on epidemiological studies with consistent associations between exposure and disease, but for which shortcomings in the evidence exist, such as insufficient available trials (or prospective observational studies). New to GBD 2021, we retained risk–outcome pairs included in GBD 2019 and evaluated the majority of these (table S7) using a new Burden of Proof methodology, described in more detail in section 2.1.6 below. Risk–outcome pairs previously included in GBD 2019 were retained in GBD 2021 provided that there was convincing or probable evidence. Entirely new risk factors were added based upon a minimal one-star Burden of Proof risk function (BPRF) (methods on BPRF detailed in section 2.1.6) and review and majority vote for inclusion by the GBD Scientific Council.

Section 2.1.2: Overview of the effect size estimation pathway

For most relative risks, our meta-analytic approach followed six main steps: 1) search and extract data from published studies using a standardised approach; 2) estimate the shape of the exposure versus relative risk relationship, integrating over exposure ranges in different comparison groups and avoiding the distorting effect of outliers; 3) test and adjust for systematic biases as a function of study attributes; 4) quantify remaining between-study heterogeneity while adjusting for within-study correlation induced by computing relative risks for several alternatives with the same reference, as well as the number of studies; 5) evaluate evidence for small-study effects to evaluate a potential risk of publication or reporting bias; and 6) estimate the BPRF, quantifying a conservative interpretation of the average risk increase across the range of exposure supported by the evidence to compute the risk–outcome score (ROS). Then, the ROS are mapped into five categories of risk as star ratings. Zheng and colleagues[10] published the technical developments required to implement this approach, which are also disseminated using open-source Python libraries.[11,12] Implementation details for each step of the approach used to find the ROS are described below. Custom models were used for some risk factors such as temperature (see GBD 2021 risk factor–specific modelling descriptions below for more details).

Section 2.1.3: Collate relative risk data

The relative risk (RR) by level of exposure or by cause for mortality or morbidity can be found in published and unpublished primary studies or in secondary studies that summarise RRs. We collated information from primarily RCTs, cohort, and pooled cohort studies; and in some instances, case-control studies. We used these data to determine the RR for the risk–outcome pairs included in GBD 2021 (table S7). For most risks, data from pooled cohorts or meta-analyses of cohorts were used; in the case of the risk of cataracts from household air pollution (HAP), cohort data were not available, and instead we used case-control data. We estimated RRs of mortality and morbidity for 88 risk factors for which we determined attributable burden by using RR and exposure. We incorporated RRs from studies that controlled for confounding but not for factors along the causal pathway between exposure and outcome. For risk–outcome pairs with evidence available for only one element of mortality or morbidity, we generally assumed that the estimated RRs applied equally to both. Given evidence of statistically different RRs for mortality and morbidity, we incorporated different RRs for each. Details and citation information for the data sources used for RRs are provided in searchable form through a web tool (http://ghdx.healthdata.org/). Available data sources for determining RRs varied across risks. Details on how RRs were calculated for each risk can be found in appendix section 6.

Systematic review protocol for relative risks

Task	Protocol
Develop inclusion criteria and search string	• Develop inclusion/exclusion criteria for systematic review based on GBD definition and expert knowledge.<br>• Develop search string in collaboration with GBD risk factor and cause teams and UW librarian (or host institution librarian as needed).
Identify existing meta-analysis / systematic review	• Use PubMed to identify existing meta-analysis/systematic review for risk-outcome pair. Criteria to identify meta-analysis:<br>1. PRISMA compliant – meta-analysis follows PRISMA reporting guidelines<br>2. Published in quality journal – journal ranks in the top two quartiles based on: https://www.scimagojr.com/journalrank.php?area=270 [scimagojr.com] (using the most appropriate subjects categories for the R-O pair)<br>3. Incorporates inclusion criteria that are the same as or more inclusive of final inclusion criteria for risk-outcome pair (e.g. include 'diarrhea' as outcome; whereas final inclusion criteria specifies 'WHO diarrhea definition')<br>4. Most recent
Pre-registration	• Pre-register systematic review on PROSPERO; example linked here.
Screening studies in meta-analysis	• One reviewer will be required to include an article in the data extraction phase, and two reviewers will be required to exclude an article. Discussion and consultation with senior personnel will occur as needed.<br>• Other considerations for data sparse topics – if meta-analysis yields 5 or fewer studies for inclusion:<br>1. Screen at least 2 additional published meta-analyses.<br>2. If still less than 5 included studies, conduct full literature review.
Updated search	• Conduct updated literature search from date published meta-analysis completed its search to present day (if applicable) or conduct literature review for studies published from at least 1985 to present if completing a full literature review.<br>• Use at least three databases in search that include PubMed (https://www.pubmed.gov), Embase + one topic-specific database<br>• Additional databases to consider:<br>o Web of Science<br>o Scopus<br>o CINAHL: Cumulative Index to Nursing & Allied Health Literature<br>o PsychINFO<br>o Cochrane Library<br>o LILACS: Latin American and Caribbean Health Sciences Literature<br>o CNKI: Chinese National Knowledge Infrastructure<br>o Global Index Medicus<br>o SciELO: Scientific Electronic Library Online

Title/abstract screening	• De-duplicate articles from multiple databases using DistillerSR (or similar).<br>• For reviews utilizing a single screener, a second reviewer will duplicate screen 100 studies or 10%, whichever is higher, of *excluded* articles as a quality check.<br>• Resolve conflicts with full-text screening and third reviewer (content expert) as needed. If the second reviewer identifies 2 or more studies that were incorrectly excluded after resolving conflicts with full-text screening, then retrain first reviewer on inclusion/exclusion criteria and redo full title/abstract screening from the beginning.<br>• If dual screening is employed, then this is considered to be above the minimum standard outlined in this document, so different criteria for % agreement and kappa can be used.
Full-text screening	• One reviewer will be required to include an article in the data extraction phase, and two reviewers will be required to exclude an article. Discussion and consultation with senior personnel will occur as needed.<br>• Other considerations: use team-level input to resolve edge cases; include specific information on how to handle data related to bias covariates and outcomes during team-specific training process.
The following steps apply to all included studies from meta-analyses and updated literature reviews:	
Duplicate cohorts or case-control (if applicable)	• Check for duplicate cohorts or case-control.<br>• If duplicates exist, select study to include based on exposure time, follow-up period, and covariates included in RR estimation (or other pre-determined criteria).
Data extraction	• Use one reviewer to extract data using the relative risk extraction template and validations.<br>• Use a second reviewer (content expert) to check for correctness and completeness of extracted data from at least 10% or 25 articles, whichever is higher.
Documentation and training	• Complete PRISMA flowchart (2020 version)<br>• Complete REDCap documentation. REDCap is a centralized web-based database used to document all GBD systematic reviews.<br>• Complete RR systematic review extended documentation.<br>○ Team-specific and GBD-wide trainings related to risk factors training, bundles, study design and measures of association, Distiller SR, evidence score/burden of proof.

Bias covariates: categories, conventions, and cases precluding inclusion of bias covariates

In the BPRF analysis, each source of bias is represented as a binary bias covariate, taking on the value '0' if the study has the gold standard in the bias covariate, and otherwise taking on the value '1.' The gold standard should be defined prior to data extraction. A value must be assigned for each bias covariate for each study, and each bias covariate must have at least two studies per group ('0' and '1').

The bias covariate selection methodology is entirely data driven. That means that any bias covariate included in the data must have sufficient studies with labels of ('0') and ('1'); in particular, a user cannot evaluate a bias covariate with no gold standard representatives, or only gold standard representatives.

Data sparsity issues that preclude bias covariate inclusion based on available data are:

- A bias covariate that has only zeros or ones, or only one study with the value '0' or '1,' cannot be included in the analysis. For inclusion, every bias covariate must have some studies that are the gold standard (have a value of '0'), and there must be at least two studies in both groups ('0' and '1'). Even with the use of priors, the model cannot include variables for which there is no variation in the covariate across observations.
- Redundant bias covariates cannot be included. If two or more bias covariates all have the same labels across studies, or nearly all the same labels across studies, all but one of the redundant bias covariates must be removed.

There are six categories of bias covariates, based on the GRADE criteria, that must be evaluated in a BPRF analysis (assuming sufficient data as discussed above). It is possible to include more than one potential bias covariate within each category, again assuming there is sufficient data to do this. These six study-level bias sources are listed below, with multiple examples of potential bias covariates:

1. **Representativeness of the study population**: assesses whether the study participants are representative of the target population, in terms of demographics and other relevant factors.

    - Typically named cov_representativeness
    - '0' for studies whose results are likely generalizable to the total population: sample was based on the general population with reasonable exclusions for pre-existing disease states.
    - '1' for studies performed on non-representative subpopulations, e.g., a high-risk group.

2. **Exposure**: assesses whether the exposure (i.e., risk factor) of interest is well-defined and measured accurately in the study.

    - Typically named cov_exposure_quality
    - '0' for studies whose exposure measurement characteristics are considered the gold standard for that risk-outcome pair and '1' otherwise.
    - May be broken into subgroups if sufficient information exists for each subgroup:
        - cov_exposure_population: '0' for individual-level exposure and '1' for population-level exposure.
        - cov_exposure_selfreport: '0' for measurements based on assays, tests, or physician observations and '1' for self-report.
        - cov_exposure_study: '0' if exposure was measured multiple times and '1' for only a baseline measurement. Case-control studies should be scored as '1' unless a detailed exposure history was solicited that allows for quantification of variation in exposure.

3. **Outcome**: assesses whether the outcome of interest is clearly defined, clinically relevant, and accurately measured in the study.

    - Typically named cov_outcome_quality
    - '0' for studies whose outcome measurement characteristics are considered the gold standard for that risk-outcome pair and '1' otherwise.
    - May be broken into subgroups if sufficient information exists for each subgroup:

    - cov_outcome_selfreport: ‘0’ if outcome measurement was based on death certificates, physician diagnosis or medical records and ‘1’ if based on self-report.
    - cov_outcome_unblinded: ‘0’ if outcome assessment is blind to the individual level of exposure and ‘1’ if unblinded.

4. **Reverse causation**: assesses the possibility that the observed relationship between the exposure and outcome could be due to the outcome causing the exposure, rather than the other way around.

    - Typically named cov_reverse_causation
    - ‘0’ for studies where reverse causation was accounted for (there is minimal or no risk of reverse causation).
    - ‘1’ for studies where reverse causation was not accounted for (there is a risk of reverse causation).

5. **Control for confounding**: assesses whether the study design and analysis adequately account for potential confounding factors that could affect the relationship between the exposure and outcome.

    - Typically named cov_confounder_quality
    - ‘0’ for studies where confounding factors were accounted for.
    - ‘1’ for studies where confounding factors were not accounted for.
    - May be broken into subgroups if sufficient information exists for each subgroup:
        - cov_confounder_nonrandom: ‘0’ if the study was randomized and ‘1’ if the study was nonrandomized.
        - cov_confounder_uncontrolled: ‘0’ for randomization or for a non-randomized study where the outcome is controlled for all major known confounders including age, sex, education, income, and other critical determinants of the outcome. ‘1’ for non-randomized studies with control for only some determinants.

6. **Selection bias**: assesses whether the study sample was selected in a way that could introduce bias and whether efforts were made to minimize bias in the study design and analysis.

    - Typically named cov_selection_bias
    - ‘0’ for studies with greater than 95% follow-up and ‘1’ for studies with less than 95% follow-up.
    - Case-control studies should be scored based on the percentage of cases and controls for which exposure data could be ascertained.

Additional bias covariates, following the same general criteria for collecting and assigning values, may be considered for certain risk factors. The decision to include any additional bias covariates is up to the modeler and their team leads. The names of the additional bias covariates may be defined arbitrarily but should reflect the content of the bias.

Section 2.1.4: Estimating the shape of the risk–outcome relationship

Most classic epidemiological analyses of exposure or dose–response risk relationships have either assumed the relationship between risk and outcome to be log-linear or have converted continuous exposure variables into dichotomous exposure categories. This assumption simplifies the analysis considerably. Unfortunately, while assuming a log-linear relationship is analytically convenient and allows for the use of simple open-source tools,[13] it is not necessarily biologically or clinically plausible (see model validation section for more details). For some risks, such as smoking, log-relative risk of outcome flattens at higher exposures. For others, such as BMI, the log-relative risk curves are J-shaped. We therefore chose to estimate the shape of the relationship directly from the data using a regularised spline.

In GBD 2021, for continuous and dichotomous risk factors, we modelled RRs using meta-regression—Bayesian, regularised, trimmed (MR-BRT) (see details below), relaxing the log-linear assumption to allow for monotonically increasing or decreasing but non-linear functions using cubic splines. Risk factors for which we undertook this reanalysis include all dietary risk factors, low physical activity, kidney dysfunction, unsafe water and sanitation, no access to handwashing facility, particulate matter air pollution, lead exposure, vitamin A deficiency, secondhand smoke, bullying victimisation, high body-mass index, high fasting plasma glucose, and high alcohol use. We did not conduct this reanalysis for risk factors with direct PAFs or PAFs=1 as well as select risk factors such as childhood sexual abuse, intimate partner violence, chewing tobacco, occupational risk factors, low bone mineral density, iron deficiency, suboptimal breastfeeding, and low birthweight and short gestation.

Because knot placement can affect the shape of the risk function when modelling with a cubic spline, we generated a wide range of knot placements and created an ensemble across these different knot placements. We also included in the final estimation 10% trimming of the data to avoid the results being sensitive to outliers.

Many meta-analyses convert RRs to per unit increase for convenience, particularly when studies choose different categories that could not otherwise be compared. If samples in the primary studies at high levels of exposure were sufficient to inform the shape of the tail of the distribution, we applied a cap to the maximum RR by using the midpoint of the last category for which a RR was reported.

Basis splines, measurement mechanism, and shape constraints

First, we used a Bayesian regularised spline to obtain the general shape of the non-linear relationship. Basis splines represent nonlinear curves as linear combination of recursively generated basis elements.[14] The basis elements were recursively generated using piecewise smooth polynomials and were roughly localised to certain regions of the exposure variable in the data. In most cases, quadratic or cubic polynomials were used, often with linear tails in the presence of sparse data. This approach allowed the use of the common restricted cubic spline, as well as constraints on the shape of the relationship (including non-decreasing and non-increasing).

Given basis functions $f_1, \dots, f_k$, the final curve is obtained as a β-linear combination

$$signal = \beta_1 f_1 + \cdots + \beta_k f_k.$$

Specifically, for any given exposure x, the prediction using the spline model is given by

$$signal(x) = \beta_1 f_1(x) + \cdots + \cdots + \beta_k f_k(x) = \langle X, \beta \rangle \tag{1}$$

where X is a vector containing $(f_1(x), \ldots, f_k(x))$. Derivatives and integrals of splines can likewise be expressed as linear combinations of spline coefficient sβ. For additional details about B-splines see Zheng et al.[10]

Many studies of exposure–response relationships report relative risks between categories defined by intervals of exposure. The relative risk between two exposure groups is a ratio of integrals of the spline across two specified intervals, so we used this exact non-linear mechanism to inform the fit.[10] Data from studies usually compare outcome rates in one exposure alternative group to those in a separate reference group. In mathematical notation, such observations are given by

$$y_{ij} = \frac{\frac{1}{d_{ij} - c_{ij}} \int_{c_{ij}}^{d_{ij}} f(x)dx}{\frac{1}{b_{ij} - a_{ij}} \int_{a_{ij}}^{b_{ij}} f(x)dx}, \tag{2}$$

where y_{ij} is the reported relative risk corresponding to measurement j in study i, $[a_{ij},\ b_{ij}]$ delineates the reference group exposure interval, and $[c_{ij},\ d_{ij}]$ delineates the alternative group exposure interval. When $f(x)$ is represented using a spline, each integral is a linear function of β similar to (1). The observation model (2) is then a non-linear function given as the ratio of linear functions,

$$y_{ij} = f_{ij}(\beta) := \frac{\langle X_{ij}^1, \beta \rangle}{X_{ij}^2, \beta}. \tag{3}$$

with the associated log-relative risk given by

$$\ln(y_{ij}) = \ln(\langle X_{ij}^1, \beta \rangle) - ln\left(\langle X_{ij}^2, \beta \rangle\right). \tag{4}$$

Equation (4) is the main model used to infer the spline, and it is a simple but non-linear function of the spline coefficient β.

When studying exposure–response relationships, we allow for shape constraints of the inferred mean response. For example, for some harmful risks, such as smoking and air pollution, we allow the relative risk to be specified as monotonically increasing with exposure. In order to introduce each of these constraints, we used the fact that all derivatives of splines are linear functions of spline coefficients, similar to (1).

Monotonicity. Monotonicity constraints can be imposed using linear inequality constraints based on exemplar exposures. Given an exemplar exposure x_i, the requirement that the slope of the spline at exposure x_i be non-negative can be formulated as

$$\langle X_{ij}, \beta \rangle \geq 0$$

for a particular vector X_i. Linear inequality constraints are strictly enforced by the optimisation solver used to fit the model; see Zheng et al.[10]

Robust trimming strategy

To make the estimation of the overall relationship less sensitive to potential outlying studies or observations within studies, we applied a robust, likelihood-based statistical approach—least trimmed squares (LTS)[15]—to our mixed effects models.[10] The goal of robust statistical methods is to ensure that estimates are robust to such outlying observations. Trimming approaches form a subclass of robust statistical methods, and LTS was originally developed in the context of linear regression.[16] LTS works by classifying observations into a majority of inliers and minority of outliers, while simultaneously fitting the model with respect to which the inlier/outlier classification is made. Compared with other robust approaches, such as M-estimators,[17] trimming methods are more effective in limiting influence than outliers, and have a high breakdown point[18]; ie, the proportion of the data than can be arbitrarily corrupted before the estimator becomes invalid.

Trimming estimators have been applied to a broad range of problems, from linear regression[15] to high-dimensional sparse regression and general machine learning problems.[19] In the context of mixed effects models, trimming methods are the most effective robust tools currently available for meta-analysis.[10] In practice, the approach requires only a specified inlier proportion, which was set to 90% across all examples, ie, we fit the 90% most self-coherent datapoints.

Using this approach, we trimmed 10% of the observations as part of the model fitting process, simultaneously discovering and fitting the most self-coherent 90% of the observations.[10] Numerical studies in data-rich cases have shown that quality of estimation is unaffected by trimming, even when there are no outliers in the data.[19] In the meta-analytic regime, the 90% level is a heuristic that balances the sparsity of available data with the need to improve estimates in the presence of outliers. As noted below, this step also substantially decreased the number of risk–outcome pairs with evidence of residual publication or reporting bias.

Spline ensemble

Third, to make non-linear risk function estimates robust to knot placement, we created 50 models based on random knot placement samples. Spline estimates depend on the choice of spline parameters, including spline degree, number of knots, and knot placement. To mitigate the effect of spline parameter selection on results, we developed an ensemble approach over knot placement, so that the modeller only had to specify the spline degree and number of knots.

Given the degree and number of knots, we automatically sampled a set of knot placements for a feasible knot distribution (described below). For each knot placement, we fit a spline (including non-linear measurements, shape constraints, and trimming as discussed above), evaluated each resulting model by computing its fit and curvature, and aggregated the final model as a weighted combination of the ensemble.

Sampling knots from simplex. We used a minimal set of rules that describe a feasible set from which to sample knots, and sample from this set uniformly. Given a number of knots, the rules specify feasible ranges for each knot and feasible gaps between knots. The set of knot placements that satisfy these four rules form a closed polyhedron (a volume in high-dimensional space delineated by hyperplanes). We calculated the vertices of the polyhedron using the double description method[20] and uniformly sampled knot placements from within the polyhedron. Each knot placement yielded a model, fit using the trimmed constrained spline approach described above.

Ensemble performance evaluation. Once the ensemble was created, we scored the resulting risk curves using two criteria: model fit (measured using the log-likelihood) and total variation (measured using the

highest-order derivative). These scores balanced competing objectives of fit and generalisability. Once we had these scores, we normalised them to the range $[0,1]$ and applied a logistic transformation. The transformation was used to make the scoring meaningful even in the presence of spurious curves in a large ensemble. We then multiplied the scores to down-weight models that were low under either criterion (fit or total variation). The final weights were normalised to sum to 1. Using a weighted combination of these metrics, we weighted the 50 models to create the ensemble model.

New non-linear covariates

Fourth, for risk–outcome pairs with non-linear relationships, we evaluated exposure levels since this information matters for non-log-linear pairs. To do this, we took advantage of the spline model and directly captured the typical data-generating mechanism. Specifically, we used the final model that we had estimated using the robust spline ensemble to generate a non-linear dose–response curve, which we encoded into new non-linear "signal" covariates that were later used to enable linear mixed effects analyses. Once the non-linear estimation was complete, the log-relative risk for each datapoint was a function of four parameters:

$$F\left(a_{ij}, b_{ij}, c_{ij}, d_{ij}\right) = \frac{\frac{1}{d_{ij} - c_{ij}} \int_{c_{ij}}^{d_{ij}} \hat{f}\,(x) dx}{\frac{1}{b_{ij} - a_{ij}} \int_{a_{ij}}^{b_{ij}} \hat{f}\,(x) dx}$$

where $\hat{f}$ is the non-linear function obtained by estimating spline coefficients $\hat{\beta}$, see (4), $\left[a_{ij}, b_{ij}\right]$ delineates the reference group exposure interval, and $\left[c_{ij}, d_{ij}\right]$ delineates the alternate group exposure interval.

We produced non-linear covariates for fixed and random effects. The non-linear fixed effects covariate, denoted $signal^f$, is given by

$$signal_{ij}^f = F(a_{ij}, b_{ij}, c_{ij}, d_{ij}). \tag{5}$$

The new non-linear random effect covariate, denoted by $signal^r$, is given by

$$signal_{ij}^r = F(t, t, c_{ij}, d_{ij}), \tag{6}$$

where t denotes a fixed reference, eg, the theoretical minimum risk exposure level (TMREL). We used these new covariates in linear mixed effects models in further stages of analysis:

$$y_{ij} = \; signal_{ij}^f \beta_s + \; signal_{ij}^r u_i + \; \epsilon_{ij} \tag{7}$$

where $\epsilon_{ij} \sim N(0, \sigma_{ij}^2)$ are known by each observation, β_s is a scalar linear covariate multiplier on the $signal^f$ covariate, and u_i is a random study-specific slope on the $signal^r$ covariate with unknown variance γ. The posterior for β_s in (7) was used as a reference for the prior in bias covariate selection, described in step 3.

For our visualisations (figures 1A–6A), we plotted each datapoint with x-value at the midpoint exposure of the alternative group, and y-value corresponding to the sum of the log relative risk and estimated

curve evaluated at the midpoint of the reference group. These visualisations allow the standard assessment of fit quality, with a perfect fit corresponding to the estimated non-linear relationship passing through the data.

Testing for bias across different study designs and characteristics

Following the approach of the GRADE criteria,[21] we quantified common sources of bias across six general domains: representativeness of the study population, exposure assessment, outcome ascertainment, reverse causation, control for confounding, and selection bias. In the illustrative cases presented here, these variables were quantified for each study during the study extraction phase. For the set of studies on a risk–outcome association, we tested systematic variation as a function of these risk of bias variables through meta-regression. We converted the dose–response relationship identified in step 1 into a new "signal" covariate, effectively linearising the non-log-linear relationship. For each bias covariate x (coded as an indicator variable), we defined a corresponding interaction covariate (ie, an effect modifier):

$$y_{ij} = signal_{ij}^{f} \times \left(\beta_s + x_{ij}^{1}\beta_1 + \cdots + x_{ij}^{k}\beta_k\right) + \epsilon_{ij}$$

that modified the slope of the "signal" covariate. We then tested risks of bias of the effect modifiers through linear meta-regression. To be included, every bias covariate must have some studies that are the "gold standard" (ie, at the standard of the best studies that have been conducted) for that covariate; otherwise, it is not possible to incorporate it into the regression framework. Further, in considering potential covariates, we enforced that every categorical covariate had at least two studies in each category. Since bias covariates were already study-specific, we only considered the fixed-effects model in bias covariate selection.

We used a robust approach to test for bias that limited the risk of over-interpreting differences with limited numbers of studies. We used the Lasso[22,23] approach—which augments the least squares loss typically solved in a linear regression by penalising the sum of absolute values of the bias covariate multipliers—to obtain a ranked list of bias covariates using the following equation:

$$\min_{\beta} \sum_{i,j} \frac{1}{2\sigma_{ij}^2} \left(y_{ij} - signal_{ij}^{f} \times \left(\beta_s + x_{ij}^{1}\beta_1 + \cdots + x_{ij}^{k}\beta_k\right)\right)^2 + \frac{1}{2}\beta^T \Sigma^{\{-1\}} \beta + \lambda \parallel \beta \parallel_1 \quad (8)$$

where β contains specifically bias covariate multipliers, $\sum$ is a diagonal matrix linked to the posterior on β_s from the basic linear model (7), and the term $\lambda \parallel \beta \parallel_1$ penalises the sum of the absolute values, pushing the bias covariate multipliers β to 0, with a strength determined by λ.[23]

We then selected bias covariates based on their Lasso ranking, obtained by sweeping through from high to low values of λ in (8). We then added the selected covariates to the linear meta-regression model one at a time, following this ranking. To stabilise the selection process and follow through on the "burden of proof" philosophy, we tested for significance of covariates using a Gaussian prior that biased all bias coefficients to 0 with a strength proportional to the posterior of the main dose–response relationship. If the coefficients were significant, they stayed in the model as the process continued; we terminated the process when the last added bias covariate was no longer significant after accounting for "signal" and higher-ranked covariates in the model. We predicted the risk function using the values of

the included bias covariates that reflected the preferred level of the covariate, such as the highest level of control for confounding.

Section 2.1.5: Quantifying between-study heterogeneity; accounting for heterogeneity, uncertainty, and small numbers of studies

Estimation of between-study heterogeneity is an important aspect of meta-analysis. It reflects the variation between studies and consistency across literature. In the following, we describe how we used the signal and bias covariates obtained in steps 2.4 and 3 to build a simple linear mixed effects model to capture the between-study heterogeneity.

After the selection procedure, we fit a final linear mixed-effects model that included the "signal" as well as selected bias covariates. Division by a common referent in the typical measurement mechanism induces correlation, (via an intercept shift in log-relative risk space); we therefore used a random intercept in the mixed-effects model to account for this induced within-study correlation. To capture the between-study heterogeneity, we used a study-specific random slope with respect to the "signal" model so that the random effect for each study effectively scaled the non-linear relative risk curve. Formally, we fit a linear mixed effects model of the form

$$y_{ij} = signal_{ij}^{f} \times (\beta_s + x_1\beta_1 + \cdots + x_k\beta_k) + signal_{ij}^{f} u_i + \epsilon_{ij}$$

where $\epsilon_{ij} \sim N(0, \sigma_{ij}^2)$ are the reported observation standard errors, and u_i are random effects with a common unknown variance,

$$u_i \sim N(0, \gamma).$$

Parameters β and γ were estimated simultaneously using maximum likelihood; see Zheng et al[10] for more details. We used the same prior on bias covariates in this analysis as we used in equation (8), ie, $\beta \sim N(0, \sum)$. For log-linear relative risks, this modelling choice reduced to the classic analysis, where the random slope with respect to exposure was equivalent to the random intercept for log-linear relative risk.

To account for the small studies problem—where in the setting of small numbers of studies, between-study heterogeneity (γ) can easily be under-estimated,[24] and in particular the estimate may be zero when too few studies are available—we quantified the uncertainty in heterogeneity estimation.[25] This estimate allowed a quantile of the heterogeneity parameter to be used, increasing the robustness of the estimate against the small study problem. Among several alternatives in the literature,[26,27] we used the Fisher information matrix (FIM)[26] to estimate the uncertainty of the between-study heterogeneity. The FIM is weakly dependent on observed data but is sensitive to the non-linear relationship, selected bias covariates, reported standard errors, and the number of studies. The final uncertainty intervals we report are composed of two components: (1) posterior uncertainty corresponding to fixed effect β_s, and (2) 95% quantile of γ, which depends on the estimate of γ and the estimate of the variance of γ using the inverse of Fisher information.

Section 2.1.6: Estimating the burden of proof risk function

The combined uncertainty for the mean, estimated between-study heterogeneity, and 95th quantile of the between-study heterogeneity obtained from the FIM estimate were used to generate a BPRF. The BPRF is defined as either the 5th (for harmful risks) or 95th (for protective risks) quantile curve closest to

the line of relative risk equal to 1 (the null) and can be interpreted as the smallest harmful or protective effect at each level of exposure consistent with the available evidence.

In the range of exposures defined by the 15th and 85th percentiles of exposure levels observed for each risk across available studies, the ROS is defined as the signed value of the average log BPRF. For example, a log BPRF of 0.4 for a harmful risk (where null = 0) and a log BPRF of –0.4 for a protective risk would both have an ROS of 0.4 because the magnitude of the log relative risk is the same. In contrast, for risk–outcome pairs with a BPRF opposite the null from the mean risk (ie, the BPRF suggests that the relationship is opposite of the expected relationship—a BPRF below 1 for a harmful risk and a BPRF above 1 for a protective risk), ROS would be calculated as negative.

Network analysis

Some relative risks were modelled using network analysis. Network analysis is a special case of the mixed effects linear model that is used to compare multiple treatment effects, for example, in the case of drinking water. To explain the coding, we use an example with four treatments A, B, C, D.

For simplicity, assume *A* is this reference treatment. We then have the following coding.

$$AB \to B - A: \quad [1 \quad 0 \quad 0]$$

$$AC \to C - A: \quad [0 \quad 1 \quad 0]$$

$$AD \to D - A: \quad [0 \quad 0 \quad 1].$$

We see from this example that the design matrix under the basic network assumption is always full rank, since a subset of rows forms the identity matrix.

Comparisons that do not include the reference can be computed. For example,

$$BC \to C - B = (C - A) - (B - A)$$

$$= [0 \quad 1 \quad 0] - [1 \quad 0 \quad 0]$$

$$= [-1 \quad 1 \quad 0]$$

Using this simple algebra, we obtain the remaining codings.

$$BC \to C - B: \quad [-1 \quad 1 \quad 0]$$

$$BD \to D - B: \quad [-1 \quad 0 \quad 1]$$

$$CD \to D - C: \quad [0 \quad -1 \quad 1]$$

Each row of the design matrix $\boldsymbol{X}$ is coded according to the comparison.

When doing network analysis, the design matrix $\boldsymbol{X}$ does not include the intercept term (**1** column).

Section 2.1.7: Evaluating potential for publication or reporting bias

A significant association between mean effect and standard deviation may indicate potential for publication or reporting bias, or methodological differences between large and small studies, which likewise lead to biased results. Publication bias is an important issue in meta-analysis,[28] and a formal test is typically done in addition to visual inspection of the funnel plot to decrease the chances of flagging

apparent bias due to chance alone. In the proposed approach, we checked whether the standard deviations were significant predictors of the observations in the presence of the "signal" and bias covariates. To detect publication bias, we used a data-driven approach known as Egger's regression.[29] The approach detects if there is a significant correlation between the residuals and their standard deviations. When Egger's regression failed to detect significant evidence of publication bias, we terminated the process. While we identified these pairs as having potential for publication or reporting bias, we followed the general literature and did not incorporate any correction to the risk function based on this finding.

Section 2.1.8: MR-BRT and temperature

While meta-regression of literature studies was applied to estimate relationships for risk–outcome pairs, for temperature, we conducted primary analysis of relationships with cause-specific mortality as described previously.[30] The relative risk, RR, of mortality was calculated for each daily and mean annual temperature category in each administrative unit. For this purpose, we calculated the daily mean temperature and aggregated the daily cause-specific death counts for each administration. We then calculated mortality rates for each cause, c, location, l, and daily mean temperature, ie, temperature category, t:t

$$MR_{clt} = \frac{deaths_{clt}}{person - days_{lt}}$$

With *MR* representing the mortality rate, *deaths* being the absolute number of cause-specific deaths, and *person-days* depicting the sum of the population in location, *l*, across all days with a daily temperature of *t*.

Following, we calculated the mean MR, $\overline{MR}$, for each cause, *c*, and location, *l*:

$$\overline{MR_{cl}} = \frac{deaths_{cl}}{person - days_{l}}$$

The daily temperature-specific mortality rate ratio, *MRR*, was then calculated as the ratio of the MR for each temperature category, location, and cause, and the average $\overline{MR}$:

$$MRR_{clt} = \frac{MR_{clt}}{\overline{MR_{cl}}}$$

In order to aggregate the *MMRs* to the first-level administrative unit, we calculate the population-weighted mean temperature (PWMT) for each location and across all days and then pooled all *MRRs* for each combination of daily temperature and PWMT.

Step 2. Exposure estimation[5]

Section 2.2.1: Collate exposure data

Systematic reviews

For GBD 2021, we conducted updated systematic literature reviews of risk factor exposure for two risks (high fasting plasma glucose and smoking). For other risk factors, only a fraction of the existing data

appears in the published literature, and other sources predominate, such as survey, measurement, or satellite data. Data were systematically screened from household surveys archived in the GHDx (http://ghdx.healthdata.org), including Demographic and Health Surveys, Multiple Indicator Cluster Surveys, Living Standards Measurement Surveys, and Reproductive Health Surveys. Other national health surveys were identified based on survey series that had yielded usable data for past rounds of GBD, sources suggested to us by in-country collaborators, and surveys identified in major multinational survey data catalogs, such as the International Household Survey Network and the WHO Central Data Catalog, as well as through country ministry of health and central statistical office websites. Certain risks, such as poor diet and excessive alcohol consumption, also incorporated administrative record systems. Citations for all data sources used for risk factor estimation in GBD 2021 are provided in searchable form through a web tool (http://ghdx.healthdata.org). A description of the search terms employed for risk-specific systematic reviews are detailed by cause in appendix section 6.

Information on systematic reviews were managed by using Research Electronic Data Capture (REDCap) electronic data capture tools hosted at the University of Washington.[31] REDCap is a secure, web-based application designed to support data capture for research studies that provides 1) an intuitive interface for validated data entry; 2) audit trails for tracking data manipulation and export procedures; 3) automated export procedures for seamless data downloads to common statistical packages; and 4) procedures for importing data from external sources.

Search terms

Search terms for updates of systematic reviews for GBD 2021 are shown by risk factor in appendix section 6.

Survey data preparation

Survey data constitute a substantial part of the underlying data used in the estimation process. During extraction, we concentrated on demographic variables (such as location, gender, age), survey design variables (such as sampling strategy and sampling weights), and the variables used to define the population estimate (such a prevalence or a proportion) and a measure of uncertainty (standard error, confidence interval, or sample size and number of cases).

Section 2.2.2: Adjust exposure data

Compiled Several adjustments were applied to extracted exposure sources to make the data more consistent and suitable for modelling. In GBD 2021, we implemented adjustments of risk exposure data to deal with alternative case definitons or study methods prior to entering data into our main analytical tools of DisMod-MR 2.1, ST-GPR, and MR-BRT. This decision also included the adjustment of data presented for both sexes to a male and female equivalent. The starting point was to explicitly state the reference case definition and study method and identify alternative definitions and study characteristics that fall withou our inclusion criteria.

We compiled data from both within-study comparisons (ie, data that used alternative and reference definitions in the same population) and between-study comparisons (ie, data that used an alternative definition in one population and a reference definition in another population that overlap in location, time, age, and sex) of different case definitions. For between-study comparisons, we allowed a maximum calendar year difference between studies of five years. Where validation studies (ie, those carried out at the introduction of a new set of diagnostic criteria comparing to previous criteria) were available, we extracted data on the comparison of alternative to reference. For quantities of interest

with multiple alternative definitions/methods, we also look for pairs comparing two alternatives. In a network analysis, if A is the reference and B and C are two alternatives, a comparison of A versus B and B versus C provides an indirect comparison of the alternative C against the reference A.

We pooled either the logit difference between alternative and reference or the natural log of the ratio of alternative to reference. From simulations, we found that the two methods provide almost identical results for quantities that after adjustment do not exceed a value of 0.5 (eg, prevalence or proportion). The logit difference method much better dealt with higher values and avoided prevalence or proportions to exceed 1. If the values of either the reference or alternative were zero, we aggregated values across age groups until both values had non-zero observations. We used the delta method to compute the standard error of the reference and alternative measures in logit space. The standard error of the logit difference was computed as the square root of the sum of the variances of each datapoint in a pair.

Data analysis

We used a network random effects meta-regression in MR-BRT (see section 2.1.4). In a network analysis, if A is the reference and B and C are two alternatives, a comparison of A versus B and B versus C provides an indirect comparison of the alternative C against the reference A. To implement the network, we included dummy variables with a particular structure. This was implemented as follows, where A is the reference definition/method:

- Create *k* dummy variables where *k* are all definitions/methods other than A (eg, *k* = B, C)
- Code dummy *k* as
    - 1 if the first term of the logit difference is *k*;
    - –1 if k is second term of the logit difference;
    - 0 otherwise

For example:

Study	Comparison	DummyB	DummyC
1	logit(B)-logit(A)	1	0
2	logit(B)-logit(A)	1	0
3	logit(C)-logit(A)	0	1

4	logit(C)-logit(A)	0	1
5	logit(C)-logit(B)	-1	1
6	logit(C)-logit(B)	-1	1

The coding structure outlined above in step 1 assumes that all case definitions are mutually exclusive. In some cases, however, individual case definitions are a function of different components or dimensions. For example, case definitions may vary by the type of symptoms that a respondent experiences as well as the recall period over which those symptoms are experienced. In the presence of sparse data, it may be difficult to find both direct and indirect comparisons of all individual case definitions. In these cases, an alternative approach is to assume different dimensions of case definitions have a multiplicative effect. In other words, the effect of recall period has the same relative effect across different categories of symptoms reported by respondents. To implement this coding scheme:

- Create *k* dummy variable columns for each case definition dimension
- For each dummy variable k:
    - Add 1 if *k* is a component of the first term in the logit difference
    - Subtract 1 if *k* is a component of the second term in the logit difference

In MR-BRT, we ran random effects meta-regression of the logit difference (or log ratio) with all the *k* dummy variables as covariates, omitting the intercept in the meta-regression. We used a study_id variable for be the unique combination of the NIDs of the reference and alternative studies (or alternative1 to alternative2). The coefficients on the *k* dummy variables represent the pooled logit difference of the *k* alternative definition to the reference taking into account evidence from both direct and indirect comparisons. In the example above, the coefficient on DummyA is the pooled logit difference of B minus A; the coefficient on DummyB is the pooled logit difference of C minus A. The standard error of the pooled logit difference incorporating the between-study variance was calculated as:

$$se(\text{logit}(difference_k)) = \sqrt{var_k + \gamma^2}$$

Where:

$se(\text{logit}(difference_k))$ = standard error of the pooled logit difference of alternative k to the reference

var_k= variance of the coefficient on dummy variable k
γ^2 = between-study variance

If both between- and within-study pairs were available, we examined whether there was a systematic difference between these. If there was a significant difference, we made judgement call as to whether within-study or between-study data comparisons were most appropriate. In general, this was the within-study data; however, there were important measurement or conceptual reasons for choosing between-study data. For example, for crosswalks between self-reported height and weight compared to measured height and weight, between-study comparisons may be preferable if respondents knew they would be measured and, therefore, were less likely to misreport their height and weight.

We also examined whether there were systematic differences in the adjustments by key demographics (age, sex, geographical location, year) and other potential factors that may lead to variation in crosswalks. This could only be done at present in a direct comparison model and not in a network. We did this when there was a strong rationale, eg, biological plausibility, for variation by such characteristics.

After obtaining the pooled logit difference or log ratio estimates, we predicted adjustments based on the statistical model, including uncertainty in the adjustment and sampling error of each datapoint. For non-significant logit differences or log ratios, we still applied the adjustments if there was a conceptual reason to believe that the alternative definition is biased. This expands the variance of these alternative definition datapoints.

Section 2.2.3: Estimate exposure

Mean exposure estimation

Once data were collected and compiled, the next step of the analytical flowchart was to apply adjustments, where necessary, to correct for bias. Examples of these adjustments include use of urban studies for lead; crosswalks between different measurements, methods, and definitions, such as for self-report of obesity and glycated haemoglobin (HbA_{1C}) for diabetes; and age-sex splitting of data, such as for fasting plasma glucose (FPG) level, cholesterol level, and systolic blood pressure that may be reported from broad age groups.

For the GBD, we developed two modelling approaches, a Bayesian meta-regression model (DisMod-MR 2.1) and a spatiotemporal Gaussian process regression model (ST-GPR), to pool data from different sources, control and adjust for bias in data, and incorporate other types of information such as country-level covariates. DisMod-MR 2.1 and ST-GPR are mixed effect models that borrow information across age, time, and locations to synthesise multiple data sources into unified estimates of levels and trends. A detailed description of the likelihood used for estimation and a full description of improvements made for DisMod-MR 2.1 were detailed by Vos and colleagues,[32] who provided additional detail in the appendix to that paper.[19] The ST-GPR model has three main hyper-parameters that control for smoothing across time, age, and location. Values for these hyper-parameters were selected on the basis of cross-validation. Cross-validation tests were conducted for different combinations of the hyper-parameters for three types of models: one data-sparse model, one data-moderate model, and one data-dense model. In each test, 20% of the data were held out, and the performance of each combination of hyper-parameters was evaluated on the held-out data. For each hyper-parameter combination, ten

cross-validation tests were conducted. The performance of each model in predicting the withheld 20% of the data was evaluated by using a combined measure based on root mean square error (RMSE) and uncertainty interval (UI) coverage. A detailed description of the ST-GPR process regression can be found below.

The main difference between these methods is their power to include unstructured types of data by sex and age group and their degree of flexibility. DisMod-MR 2.1 is the preferred tool in these cases because of its ability to integrate over age and adjust for different exposure definitions in the data; however, the use of Bayesian Markov Chain Monte Carlo (MCMC) simulations with large volumes of data renders the analysis computationally intensive and reduces the number of iterations that are possible. If standard age-group data are available – as is generally the case for metabolic risks – using ST-GPR becomes the preferred approach.

In some cases, we adapted our methods of modelling exposure to risks where necessary to account for complexities in the risk–outcome relationship or the need for particular handling of data, for example, dietary risks and ambient air pollution (see appendix section 6 for more detail). A complete list of risks is reported in table S1. Additional details for adjustments or adaptations to particular risk models are provided in appendix section 6.

DisMod-MR 2.1 description

Until GBD 2010, non-fatal estimates in burden of disease assessments were based on a single data source on prevalence, incidence, remission, or a mortality risk selected by the researcher as most relevant to a particular location and time. For GBD 2010, we set a more ambitious goal: to evaluate all available information on a disease that passes a minimum quality standard. That required a different analytical tool that would be able to pool disparate information presented for varying age groupings and from data sources by using different methods. The DisMod-MR 1.0 tool used in GBD 2010 evaluated and pooled all available data, adjusted data for systematic bias associated with methods that varied from the reference, and produced estimates by world regions with UIs by using Bayesian statistical methods. For GBD 2013, the improved DisMod-MR 2.0 increased computational speed, which allowed computations to be consistent between all disease parameters at the country rather than the region level. The hundred-fold increase in speed of DisMod-MR 2.0 was partly due to a more efficient rewrite of the code in C++ but also to changing to a model specification by using log rates rather than a negative binomial model used in DisMod-MR 1.0. In cross-validation tests, the log rates specification worked as well as or better than the negative binomial specification.[33] The sequence of estimation occurs at five levels: global, super-region, region, country and, where applicable, subnational location. The super-region priors are generated at the global level with mixed-effects, non-linear regression by using all available data; the super-region fit, in turn, informs the region fit, and so on down the cascade. The wrapper gives analysts the choice to branch the cascade in terms of time and sex at different levels depending on data density. The default used in most models is to branch by sex after the global fit but to retain all years of data until the lowest level in the cascade is reached.

The computational engine is limited to three levels of random effects; we differentiate estimates at the super-region, region, and country level. In GBD 2013, the subnational units of China, the UK, and Mexico were treated as "countries" to enable a random effect to be estimated for every location with contributing data. However, the lack of a hierarchy between country and subnational units meant that

the fit to country data contributed as much to the estimation of a subnational unit as the fits for all other countries in the region. We found inconsistency between the country fit and the aggregation of subnational estimates when the country's epidemiology varied from the average of the region. Adding an additional level of random effects required a prohibitively comprehensive rewrite of the underlying DisMod-MR engine. Instead, we added a fifth layer to the cascade, with subnational estimation informed by the country fit and country covariates, plus an adjustment based on the average of the residuals between the subnational location's available data and its prior. This technique mimicked the impact of a random effect on estimates between subnationals.

In GBD 2015, we also improved how country covariates differentiate non-fatal estimates for diseases with sparse data. The coefficients for country covariates are re-estimated at each level of the cascade. For a given location, country coefficients are calculated by using both data and prior information available for that location. In the absence of data, the coefficient of its parent location is used to utilise the predictive power of our covariates in data-sparse situations.

For GBD 2016, the computational engine (DisMod-MR 2.1) remained substantively unchanged from GBD 2015. We changed the prediction year set to generate fits for the years 1990, 1995, 2000, 2005, 2010, and 2016. We updated the age prediction sets to include age groups 80–84 years, 85–89 years, 90–94 years, and 95+ years to comply with changes across all functional areas of the GBD. We also expanded the set of locations where subnational units are modelled; the set now includes Brazil, China, England, India, Indonesia, Japan, Kenya, Mexico, South Africa, Sweden, and the USA.

In GBD 2017, we continued to use DisMod-MR 2.1 because no substantial changes were made. Updates to computation include extending the terminal prediction year to 2017 and additional subnational units in Ethiopia, Iran, New Zealand, Norway, and Russia. Saudi Arabia was also modelled only at the national level in 2017.

In GBD 2021, no substantial changes were made to DisMod-MR 2.1, but in GBD 2019 we made more substantial changes to how we use the tool. First, we added the current year of interest (ie, 2021) as an additional year of estimation. Second, we also included the option again to have random effects on cause-specific mortality rates (CSMR) and excess mortality rates (EMR). This functionality had been dropped a couple of GBD rounds earlier. Third, as we did all our adjustments for alternative case definition and study methods as well as adjustments to both-sex datapoints prior to entering data into DisMod-MR 2.1, we no longer used the functionality in DisMod-MR 2.1 to estimate coefficients for study covariates. Fourth, based on simulation testing, we found that coverage improved and errors reduced when passing down priors with a wider setting of minimum coefficient of variation (which determines the uncertainty around priors and hence how "informative" the priors are) than had generally been used in past GBD iterations. We settled on a default value of 0.8, where in the past, values of 0.4 or less had been more commonly used. We made some exceptions for high prevalent conditions where a lower minimum CV setting achieved the task of making priors less informative but not completely uninformative.

Fifth, we changed our approach to estimating excess mortality rates, the key link in the model between CSMR and incidence and prevalence. In the past two GBD rounds, we calculated priors on excess

mortality and entered these as datapoints by matching sex-specific prevalence data with an age width of 20 or less with the corresponding CSMR for the same location and year. For stability's sake, we excluded calculation of EMR for prevalence datapoints of less than 1 in a million. EMR is simply calculated as CSMR divided by prevalence. As with previous GBD years, for diseases with an average duration of less than a year (as indicated by a setting of remission greater than 1), we ran an initial global model to get an equivalent prevalence and used the following formula to calculate EMR:

EMR = CSMR * (remission + (ACMR - CSMR) + EMR_pred) / incidence

where, ACMR = all-cause mortality rate and EMR_pred = EMR fit from an initial global DisMod model

Despite using the log of lag-distributed income or the Healthcare Access and Quality (HAQ) Index as a covariate with a prior that the coefficient had to be negative, we found many disease models with an implausible distribution of mortality to prevalence (or incidence) ratios implying lower case fatality in locations with lower HAQ Index than in countries with higher HAQ Index. This likely signals an inconsistency between fatal and non-fatal data inputs. For GBD 2019, we decided to run regressions on EMR data (calculated as described above) first using MR-BRT with HAQ Index as a predictor. In general, we tend to think that CSMR estimates are more robust than non-fatal data because of much greater data availability and a lesser task in adjusting cause death data for garbage coding than the complex task of adjusting non-fatal data sources for alternative case definitions and study methods. To indicate that we would reduce the random effects on EMR and the minimum coefficient of variation for priors on EMR being created at each next level down the cascade. However, there were exceptions. For drug use disorders, the risk of overdose deaths is less a function of a country's quality of health services but is driven more by the availability of harm-reduction strategies such as opioid substitution therapy and the availability of highly potent opioids such as fentanyl which have been an important contributor to the large increase in overdose deaths in the USA in the last decade. We settled on a model for opioid use disorder with wider random effects and higher minimum coefficient of variation to give less emphasis on CSMR when enforcing consistency with prevalence data. In a next round, we will endeavour to find covariates that are more relevant to drug overdose deaths, such as a grading of harm-reduction strategies by country and over time. In the case of COPD, we noted that following the data on CSMR and EMR led to large increases in prevalence estimates in east Asia, Oceania and, to a lesser extent, south Asia. At oldest ages, prevalence estimates would be higher than the prevalence data for these locations and reach a level of close to 80% at oldest ages. In these locations, we will pay attention to how garbage codes are being redistributed onto COPD in the next round of GBD.

DisMod-MR 2.1 likelihood estimation

Analysts have the choice of using a Gaussian, log-Gaussian, Laplace, or Log-Laplace likelihood function in DisMod-MR 2.1. The default log-Gaussian equation for the data likelihood is

$$-log\left[p\left(y_j|\Phi\right)\right] = \log\left(\sqrt{2\pi}\right) + \log\left(\delta_j + s_j\right) + \frac{1}{2}\left(\frac{\log\left(a_j + \eta_j\right) - \log\left(m_j + \eta_j\right)}{\delta_j + s_j}\right)^2$$

where

y_j is a "measurement value" (ie, datapoint)

Φ denotes all model random variables

η_j is the offset value, *eta*, for a particular "integrand" (prevalence, incidence, remission, excess mortality rate, with-condition mortality rate, cause-specific mortality rate, relative risk, or standardised mortality ratio)

a_j is the adjusted measurement for datapoint j, defined by

$$a_j = e^{(-u_j - c_j)} y_j$$

Where:

u_j is the total "area effect" (ie, the sum of the random effects at three levels of the cascade: super-region, region, and country) and

c_j is the total covariate effect (ie, the mean combined fixed effects for sex, study-level, and country-level covariates), defined by

$$c_j = \sum_{k=0}^{K[I(j)]-1} \beta_{I(j),k} \hat{X}_{k,j}$$

with standard deviation

$$s_j = \sum_{l=0}^{L[I(j)]-1} \zeta_{I(j),l} \hat{Z}_{k,j}$$

Where:

k denotes the mean value of each datapoint in relation to a covariate (also called x-covariate)

I(j) denotes a datapoint for a particular integrand, *j*

$\beta_{I(j),k}$ is the multiplier of the kth x-covariate for the ith integrand

$\hat{X}_{k,j}$ is the covariate value corresponding to the datapoint *j* for covariate *k*;

l denotes the standard deviation of each datapoint in relation to a covariate (also called z-covariate)

$\zeta_{I(j),k}$ is the multiplier of the lth z-covariate for the ith integrand

δ_j is the standard deviation for adjusted measurement *j*, defined by:

$$\delta_j = log[y_j + e^{(-u_j - c_j)} \eta_j + c_j] - log[y_j + e^{(-u_j - c_j)} \eta_j]$$

Where:

m_j denotes the model for the jth measurement, not counting effects or measurement noise, and defined by:

$$m_j = \frac{1}{B(j)-A(j)} \int_{A(j)}^{B(j)} I_j(\text{a})\ \text{da}$$

Where:

A(j) is the lower bound of the age range for a datapoint

B(j) is the upper bound of the age range for a datapoint

I_j denotes the function of age corresponding to the integrand for datapoint *j*

Spatiotemporal Gaussian process regression

This type of regression has been used for risk factors for which the data density is sufficient to estimate a very flexible time trend. The approach is a stochastic modelling technique that is designed to detect signals amidst noisy data. It also serves as a powerful tool for interpolating non-linear trends.[34,35] Unlike classical linear models that assume that the trend underlying data follows a definitive functional form, GPR assumes that the specific trend of interest follows a Gaussian process, which is defined by a mean function $m(\cdot)$ and a covariance function $Cov(\cdot)$. For example, let $p_{c,a,s,t}$ be the exposure, in normal, log, or logit space, observed in country *c*, for age group *a*, and sex *s* at time t:

$$(p_{c,a,s,t}) = g_{c,a,s}(t) + \epsilon_{c,a,s,t}$$

where

$$\epsilon_{c,a,s,t} \sim Normal(0, \sigma_p^2),$$

$$g_{c,a,s}(t) \sim GP\left(m_{c,a,s}(t), Cov\left(g_{c,a,s}(t)\right)\right).$$

The derivation of the mean and covariance functions, $m_{c,a,s}(t)$ and $Cov\left(g_{c,a,s}(t)\right)$, along with a more detailed description of the error variance (σ_p^2), is described below.

Estimating mean functions

We estimated mean functions by using a two-step approach. To be more specific, $m_{c,a,s}(t)$ can be expressed, depending on the exposure transformation, as:

$$log(p_{c,a,s}(t)) = X_{c,a,s}\beta + h(r_{c,a,s,t})$$

$$logit(p_{c,a,s}(t)) = X_{c,a,s}\beta + h(r_{c,a,s,t})$$

$$p_{c,a,s}(t) = X_{c,a,s}\beta + h(r_{c,a,s,t})$$

where $X\beta$ is the summation of the components of a hierarchical mixed-effects linear regression, including the intercept and the product of covariates with their corresponding fixed-effect coefficients. Some models were run as hierarchical mixed-effects linear regressions with random effects on the levels of the geographical hierarchy. For most mixed-effects models, random effects were only used in the fit, not in the prediction. The second part of the equation, $h(r_{c,a,s,t})$, is a smoothing function for the residuals, $r_{c,a,s,t}$, derived from the linear model.[7] Descriptions of exposure transformations and which

covariates were used in linear models can be found in appendix section 6, which described the risk-specific estimation approaches. Some models used a custom stage-1 estimate. Detailed information on the mixed-effect estimation process for these risks may be found in the risk-specific appendix section 6.

Although the linear component captures the general trend in exposures over time, much of the data variability may still not be adequately accounted for. To address this, we fit a locally weighted polynomial regression (locally estimated scatterplot smoothing, or LOESS) function $h(r_{c,a,s,t})$ to systematically estimate this residual variability by borrowing strength across time, age, and space patterns (the spatiotemporal component of ST-GPR).[36,37] The time adjustment parameter, defined by λ , aims to borrow strength from neighbouring time points (ie, the exposure in this year is highly correlated with exposure in the previous year but less so further back in time). The age-adjustment parameter, defined by ω, borrows strength from data in neighbouring age groups. The space-adjustment parameter, defined by ξ, aims to borrow strength across the hierarchy of geographical locations. The spatial and temporal weights are combined into a single space-time weight to allow the amount of spatial weight given to a particular point $r_{c,a,s,t}$ to fluctuate given the data availability at each time *t* and location-level *l* in the location hierarchy.

Let $w_{c,a,s,t}$ be the final weight assigned to observation $r_{c,a,s,t}$ with reference to a focal observation r_{c_0,a_0,s_0,t_0}. We first generated a temporal weight $t.w_{c,a,s,t}$ for smoothing over time, which was based on the scaled distance along the time dimension of the two observations:[37]

$$t.w_{c,a,s,t} = \frac{1}{e^{\lambda|t-t_0|}}$$

Next, we generated a spatial weight to smooth over geography. Specifically, we defined a geospatial relationship by categorising data based on the GBD location hierarchy (table S4). *Zeta* acts as a scalar on a given datapoint given its proximity to the target location:

$$t.w_{c,a,s,t} = \zeta^{|c-c_0|}$$

For example, estimating a country would use the following weighting scheme:

- Country data: ζ^0 = 1
- Regional data not from the country being estimated: ζ^1
- Data from other regions in the same super-region: ξ^2
- Global data from other super-regions: ζ^3

Under the spatial weighting specification, typical values of ζ range from [0.001, 0.2], where ζ can be interpreted as the amount to down-weight regional datapoints compared to country datapoints for a given estimating country. For example, for a given datapoint $r_{c,a,s,t}$ and ζ = 0.01, a datapoint not within country *c* but within the same region *r* as $r_{c,a,s,t}$ would be assigned $\frac{1}{100}$ the weight of a datapoint within the country.

The spatial and temporal weights were then multiplied and summed across each level of the location hierarchy and normalised for each time period *t* . This procedure allowed the space-time weight to implicitly take into account the amount of data available at the country versus region versus super-region level and attribute spatial weight accordingly.

Given a normalisation constant,

$$K_i = \sum_{c\epsilon C} s.w_{c,t} * t.w_{c.t} + \sum_{c\epsilon R} s.w_{c,t} * t.w_{c.t} + \sum_{c\epsilon SR} s.w_{c,t} * t.w_{c.t}$$

the final space-time weight would then equal

$$w'_{c,a,s,t} = \frac{s.w_{c,t} * t.w_{c,t}}{K_i}$$

Finally, we calculated the weight $w''_{c,a,s,t}$ to smooth over age, which is based on a distance along the age dimension of two observations. For a point between the age a of the observation $r_{c,a,s,t}$ and a focal observation r_{c_0,a_0,s_0,t_0}, the weight is defined as follows:

$$w''_{c,a,s,t} = \frac{1}{e^{\omega|a-a_0|}}$$

The final weights would then be computed by simply multiplying the space-time weights and age weights and normalising so all weights for a given time period *t* sum to 1. A full derivation of weights for each category, assuming the location being estimated was a country, follows:

1) If the observation $r_{c,t}$ belongs to the same country c_0 of the focal observation r_{c_0,t_0}:

$$w_{c,a,s,t} = \frac{(w'_{c,a,s,t}w''_{c,a,s,t})}{\sum_{c=c_0}(w'_{c,a,s,t}w''_{c,a,s,t})} \qquad \forall c = c_0$$

2) If the observation $r_{c,t}$ belongs to a different country than the focal observation r_{c_0,t_0}, but both belong to the same region *R*:

$$w_{c,a,s,t} = \frac{(w'_{c,a,s,t}w''_{c,a,s,t})}{\sum_{c\neq c_0}(w'_{c,a,s,t}w''_{c,a,s,t})} \qquad \forall c \neq c_0 \cap R[c] = R[c_0]$$

3) If the observation $r_{c,t}$ belongs to the same super-region *SR* but to both a different country c_0 and a different region $R[c_0]$ than the focal observation r_{c_0,t_0}:

$$w_{c,a,s,t} = \frac{(w'_{c,a,s,t}w''_{c,a,s,t})}{\sum_{c\neq c_0}(w'_{c,a,s,t}w''_{c,a,s,t})} \qquad \forall c \neq c_0 \cap R[c] \neq R[c_0] \cap SR[c] = SR[c_0]$$

4) If the observation $r_{c,t}$ is from a different super-region than the focal observation r_{c_0,t_0}(Ie, all other data currently not receiving a weight):

$$w_{c,a,s,t} = \frac{(w'_{c,a,s,t}w''_{c,a,s,t})}{\sum_{c\neq c_0}(w'_{c,a,s,t}w''_{c,a,s,t})} \qquad \forall c \neq c_0 \cap R[c] \neq R[c_0] \cap SR[c] \neq SR[c_0]$$

Observations could be down-weighted by a factor of 0.1, usually because they were not geographically representative at the unit of estimation. Details of reasons for down-weighting can be found in risk-

specific modeling summaries. The final weights were then normalised such that the sum of weights across age, time, and geographical hierarchy for a reference group was 1.

Estimating error variance

σ_p^2 represents the error variance in normal or transformed space including the sampling variance of the estimates and prediction error from any crosswalks performed. First, variance was systematically imputed if the data extraction did not include any measure of uncertainty. When some sample sizes for data were available, missing sample sizes were imputed as the fifth percentile of available sample sizes. Missing variances were then calculated as $\sigma_p^2 = \frac{p*(1-p)}{n}$ for proportions or were predicted from the mean by using a regression for continuous values. When sample sizes were entirely missing and could not be imputed, the 95th percentile of available variances at the most granular geographical level (ie, first country, then region, etc.) were used to impute missing variances. For proportions where *p*n* or *(1-p)*n* is <20, variance was replaced by using the Wilson Interval Score method.

Next, if the exposure was modelled as a log transformation, the error variance was transformed into log-space by using the delta method approximation as follows:

$$\sigma_p^2 \cong \frac{\sigma_{p\prime}^2}{p_{c,a,s,t}^2}$$

where $\sigma_{p\prime}^2$ represents the error variance in normal space. If the exposure was modelled as a logit transformation, the error variance was transformed into logit-space by using the delta method approximation as follows:

$$\sigma_p^2 \cong \frac{\sigma_{p\prime}^2}{(p_{c,a,s,t} * (1 - p_{c,a,s,t}))^2}$$

Finally, prior to GPR, an approximation of non-sampling variance was added to the error variance. Calculations of non-sampling variance were done on normal-space variances. Non-sampling variance was calculated as the variance of inverse-variance-weighted residuals from the space-time estimate at a given location-level hierarchy. If there were <10 datapoints at a given level of the location hierarchy, the non-sampling variance was replaced with that of the next highest geography level with >10 datapoints.

Estimating the covariance function

The final input into GPR is the covariance function, which defines the shape and distribution of the trends. Here, we have chosen the Matern-Euclidian covariance function, which offers the flexibility to model a wide spectrum of trends with varying degrees of smoothness. The function is defined as follows:

$$M(t,t') = \sigma^2 \frac{2^{1-v}}{\Gamma(v)} \left(\frac{d(t,t')\sqrt{2v}}{l}\right)^v K_v\left(\frac{d(t,t')\sqrt{2v}}{l}\right)$$

where $d(\cdot)$is a distance function; σ^2, v, l, and K_v are hyperparameters of the covariance function—specifically σ^2 is the marginal variance, v is the smoothness parameter that defines the differentiability of the function, l is the length scale, which roughly defines the distance between which two points become uncorrelated, and K_v is the Bessel function. We approximated σ^2 by taking the normalised

median absolute deviation $MADN(r_c')$ of the difference, which is the normalised absolute deviation of the difference of the first-stage linear regression estimate from the second-stage spatiotemporal smoothing step for each country. We then took the mean of these country-level MADN estimates for all countries with 10+ country-years of data to ensure that differences between first- and second-stage estimates had sufficient data to truly convey meaningful information on model uncertainty. We used the parameter specification $v = 2$ for all models. The scale parameter l used for each risk is reported in appendix section 6.

Prediction using GPR

We integrated over $g_{c,t}(t_*)$ to predict a full time series for country c, age a, sex s, and prediction time t_*as follows:

$$p_{c,a,s}(t_*) \sim N\left(m_{c,a,s,t}(t_*), \sigma_p^2 I + Cov\left(g_{c,a,s,t}(t_*)\right)\right)$$

Random draws of 500 samples were obtained from the distributions above for every country for a given indicator. The final estimated mean for each country was the mean of the draws. In addition, 95% UIs were calculated by taking the 2.5 and 97.5 percentile of the sample distribution. The linear modelling process was implemented by using the lmer4 package in R, and the ST-GPR analysis was implemented through the PyMC2 package in Python.

Subnational scaling and aggregation

To ensure internal consistency of the estimates between countries and their respective subnational locations, national estimates were either created by population-weighted aggregation or subnational estimates were adjusted by population-weighted scaling to the national estimates, depending on the data coverage of a given country compared to that of its subnational locations. For example, if data coverage was better at the national level than at its corresponding subnational locations for a given country and risk across age, sex, and time, estimates were rescaled to be consistent with the national level. Conversely, if data coverage was better at the subnational level, estimates for its parent country were generated through population-weighted aggregation of subnational estimates.

Estimates can also be scaled within logit space. Scaling in logit space ensures that subnational estimates of proportion models do not exceed 1 after being rescaled to the national estimate.

Fitting a distribution to exposure data

The most informative data describing the distribution of risk factors within a population come from individual-level data; additional sources of data include reported means and variances. In cases in which a risk factor also defines a disease or disease severity cut-off, such as haemoglobin level and mild, moderate, or severe anaemia, or diabetes and FPG level, the prevalence of disease is also frequently reported. To model the distribution of any particular risk factor, we seek a family of probability density functions (PDF), a fitting method, and a model selection criterion. To make use of the most commonly available data describing most populations, we used the method of moments (MoM); the first two empirical moments from a population, the mean and the variance, were used to determine the parameters of two-parameter PDF describing the distribution of risk within any population. Exceptions to this rule are justified by context. We used the Kolmogorov-Smirnov[38] (KS) test to measure the goodness of fit (GoF) and compared the distance between the empirical and ensemble distributions, but in some cases, the GoF was based on the prediction error for the prevalence of disease.

We used an ensemble technique in which a model selection algorithm is used to choose the best model for each continuous risk factor.[38] We drew the initial set of candidate models from commonly used PDF families, including both right-skewed and left-skewed distributions. These included beta, exponential, gamma, gumbel, inverse gamma, inverse Weibull, log-logistic, lognormal, mirrored gamma, mirrored gumbel, normal, and Weibull. We fitted each PDF family candidate to each dataset by using the MoM and used the KS test as the measure of GoF. Preliminary analysis showed that the GoF ranking of PDF families varied across datasets for any particular risk factor and that combining the predictions of differently fitted PDF families could dramatically improve the GoF for each dataset. Therefore, we developed a new model for prediction by using the ensemble of candidate models, which is a weighted linear combination of all candidate models, $\{f\}$, where a set of weights $\{w\}$ is chosen such that $\sum_i w_i = 1$, and the values of the weights were determined by a second GoF criterion with its own validation process. For each risk, we pooled all available microdata and performed Nelder-Mead numeric optimisation across demographics subsets of data to derive a set of distribution-specific weights such that the average KS statistic across datasets would be minimised. The details can be summarised by 1) the summary statistics for each dataset; 2) a table showing the KS statistic for each candidate model; and 3) the weights defining the final ensemble model for each dataset. We then averaged across demographic subsets and datasets to determine the final weights for modelling the distribution of any particular risk factor.

Step 3. TMREL[5]

In this and all previous GBD studies, the counterfactual level of risk exposure used is the risk exposure that is both theoretically possible and minimises risk in the exposed population that consequently captures the maximum population attributable burden.[8] For each risk evaluated in GBD 2021, Step 4 of the analytical flowchart describes the use of the best available epidemiological evidence used to estimate relative risk by level of exposure and the lowest observed level of exposure from cohorts, used to select a single level of risk exposure that minimises risk from all causes of death combined to establish the TMREL. In principle, the TMREL for a given risk may vary by age, sex, and location if supported by clear evidence. Based on the available evidence, the TMREL itself can be uncertain, which is reflected in the 95% UIs in table S9.

Step 4. Estimate population attributable fractions[5]

Risks are categorised on the basis of how exposure was measured: dichotomous, polytomous, and continuous. High low-density lipoprotein (LDL) cholesterol level is an example of a risk measured on a continuous scale. The PAF, which represents the proportion of risk that would be reduced in a given year if the exposure to a risk factor in the past were reduced to an ideal exposure scenario, is defined for a continuous risk factor as:[39]

$$PAF_{joasgt} = \frac{\int_{x=l}^{u} RR_{joasg}(x)P_{jasgt}(x)dx - RR_{joasg}(TMREL_{jas})}{\int_{x=l}^{u} RR_{joasg}(x)P_{jasgt}(x)dx}$$

Where PAF_{joasgt} is the PAF for cause o due to risk factor j for age group a, sex s, location g, and year t. $RR_{joasg}(x)$ is the RR as a function of exposure level x for risk factor j for cause o, age group a, sex s, and location g with the lowest level of observed exposure as l and the highest as u; $P_{jasgt}(x)$ is the

distribution of exposure at x for age group a, sex s, location g, and year t; and $TMREL_{jas}$ is the TMREL for risk factor *j*, age group *a*, and sex *s*.

The PAF_{joasgt} for dichotomous and polytomous risk factors for every country is defined as:

$$PAF_{joasgt} = \frac{\sum_{x=l}^{u} RR_{joasg}(x)P_{jasgt}(x) \; - \; RR_{joasg}\left(TMREL_{jas}\right)}{\sum_{x=l}^{u} RR_{joasg}(x)P_{jasgt}(x)}$$

where PAF_{joasgt} is the PAF for cause o due to risk factor j for age group a, sex s, location g, and year t. $RR_{joasg}(x)$ is the RR as a function of exposure level x for risk factor *j* for cause o, age group a, sex s, and location g on a plausible range of exposure levels from *l* to *u; $P_{jasgt}(x)$* is the proportion of the population in risk group (prevalence) for age group a, sex s, location g, and year t; and $TMREL_{jas}$ is the TMREL for risk factor *j*, age group *a*, and sex *s*.

Step 5. Estimate summary exposure values[5]

Summary exposure value (SEV) is the RR-weighted prevalence of exposure, a univariate measure of risk-weighted exposure, taking the value zero when no excess risk for a population exists and the value 1 when the population is at the highest level of risk. We report SEVs on a scale from 0% to 100% on which a decline in SEV indicates reduced exposure to a given risk factor and an increase in SEV indicates increased exposure.

We first calculate risk, r, and cause, c, for specific SEVs by using the following equation,

$$SEV_{rc} = \frac{\frac{PAF_{rc}}{1 - PAF_{rc}}}{RR_{max} - 1}$$

for each most-detailed age, sex, location, year, and outcome. PAF is the YLL (except for any outcomes which are YLD only and thus use the YLD) PAF. RR_{max} for categorical risks is the RR at the highest category of exposure. For continuous risks, this is

$$RR_{max} = RR^{\frac{TMREL - 5^{th} \exp osure}{RR_{scalar}}} \text{ if protective, or}$$

$$= RR^{\frac{95^{th} \exp o\, sure - TMREL}{RR_{scalar}}}$$

otherwise, and for custom modelled risks like ambient particulate matter pollution, HAP from solid fuels, ozone pollution, alcohol, smoking, and bullying, the modeller provides draws of RR_{max}. Generally, RRs do not vary across time and space. However, exceptions exist, such as risks from secondhand smoke (SHS) or HAP for which the RR is based on the integrated exposure response (IER) curve. In these cases, the RR is averaged across location and year to ensure no time or space variation. If the PAF is negative, which signifies a protective effect for that outcome, the PAF is set to 0 and the SEV is then also 0 because the SEV is univariate and constrained to be a value between 0 and 1.

In most cases, risk – cause PAFs of 1 were not included in SEV calculations as the SEV function is undefined when the PAF value is 1. However, an alternate definition of SEV was used for a select set of risks for PAFs of 1: fasting plasma glucose, systolic blood pressure, and iron deficiency. For fasting plasma glucose, SEVs were set to risk prevalence above 7 mmol/L. For systolic blood pressure, SEVs were set to risk prevalence above 140 mmHg. Lastly, for iron deficiency, SEVs were set to the prevalence of moderate or severe anaemia.

Once we obtained a set of risk-cause-specific SEVs at the most-detailed risk, cause, age, sex, and location for all years, we averaged across causes to produce the final risk-specific SEV_r,

$$SEV_r = \frac{1}{N(c)} \sum_c SEV_{rc}$$

where $N(c)$is the total number of outcomes for a risk.

Step 6. Mediation[5]

Section 2.6.1: Summary

The portion of the burden of disease that is attributable to various combinations of risk factors or to all risk factors combined has been a topic of broad interest.[40] In GBD 2010, we only aggregated the burden of risk factors for some clusters of risks, including access to improved water and sanitation, child and maternal malnutrition, tobacco smoking, alcohol use, dietary risk factors, occupational risk factors, and sexual abuse and violence. We did not aggregate air pollution and metabolic risk factors. For GBD 2013 onward, we aggregated all risk factors into three large categories—behavioural, environmental and occupational, and metabolic risks—and aggregated all GBD risk factors into a single attributable fraction for each disease and eventually for all causes of burden. Please note that mediation is conducted as a separate process and is not part of the BPRF methodology. In our relative risk estimation, we include RRs that do not adjust for mediation as our goal is to capture the direct effect of a risk factor on an outcome.

Aggregating risk factors at different levels shares three essential challenges:

1. Risk factor coexistence or aggregation: for example, metabolic risk factors often occur together, or high-risk behaviours such as drug abuse and unsafe sex are related.
2. Mediation: a risk factor may affect another risk factor that lies in the physiological pathway to a disease outcome. It can be inside a cluster of risk factors, such as the effect of obesity through an increase in FPG level and later cardiovascular disease (CVD) outcomes, or between clusters of risk factors, such as the effect of fibre on cholesterol.
3. The formula used to calculate the aggregated PAF.

The aggregation method is conceptually applicable to other aggregations such as socioeconomic factors, education, homelessness, and refugee status that are being considered for inclusion in future GBD iterations. In the next section, we explain our approach to dealing with these challenges.

There are three patterns of associations between risk factors to consider (Figure C). The first concerns confounding; risk B affects risk A and outcome C (Pattern 1 in *Patterns of associations between risk*

factors). In these cases, the RR for A should be adjusted for B; for example, the fruit RR is adjusted for smoking. If part of the effect of A is through B, a mediator, we do not adjust the effect of A for B. For example, we do not adjust the RR of body-mass index (BMI) for cholesterol because cholesterol lies in the biological pathway between BMI and cardiovascular outcomes (Pattern 2 in *Patterns of associations between risk factors*). The third pattern occurs when risks A and B are proxies of a third variable Z and aggregation aims to estimate the total effect of a latent variable Z on C. An example is child growth failure, which is measured by stunting, wasting, and underweight as proxies.

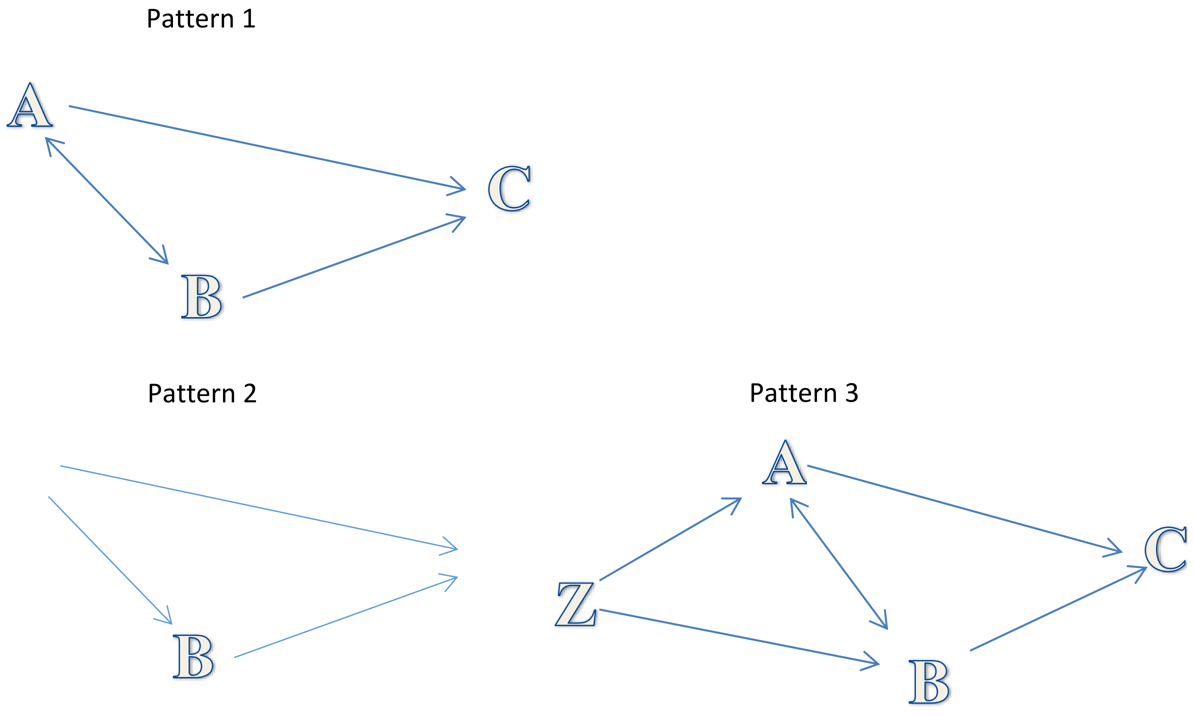

Figure C. Patterns of associations between risk factors

Section 2.6.2: Calculating the burden of multiple risk factors

Validation studies have reported congruency between the true risk associated with multiple risk factors affecting the same outcome and a multiplicative aggregation of the PAFs of the individual risk factors (formula below)[41]

$$PAF_{1..i} = 1 - \prod_{i=1}^{n}(1 - PAF_i)$$

where *PAF* is the population attributable fraction and *i* is each individual risk factor.

The same validation studies also found that the overestimation from ignoring the covariance between risk factors is small. This small overestimation was important to note because few data sources exist from which we can draw information on covariance.

We endeavoured to evaluate RRs that were controlled for confounders. However, because we had to rely on the literature for many RRs, we did not always have full control over the choice of confounders controlled for in each study.

Section 2.6.3: Computing mediation factors using linear relationships

If the relationship between the distal risk and the mediating risk factor is linear, eg, an increase in BMI of 1 kg/m^2 leads to an increase in FPG of $\delta_{B,A}$ mmol/L, we can use the linear relationship to estimate the mediated risk and hence the mediation factor. Specifically, the relative risk of C due to A mediated by B is computed as follows:

$$RR_{C,A}^{B} = \int_{b_{start}}^{b_{end}} \frac{1}{RR_{C,B}(b)} R_{C,B}\big(\delta_{B,A}(a-a_0)_{(+)}\big)p(b)db$$

The linear factor $\delta_{B,A}$ may be available from the literature or may be found using root finding in certain cases, as for example when the cause is defined purely by mediator exposure, such as for diabetes.

Section 2.6.4: Adjusting for mediation

When aggregating the effects of multiple risk factors, we included a mediation factor (MF) if a part of the effect of one risk factor was included in the effect estimated for in the mediator. First, we prepared a list of possible mediations, and especially between behavioural risks and metabolic risk factors with cardiometabolic outcomes. We did not assume any mediation effect between risk factors for cancers.

Danaei and colleagues assumed that part of the effect of BMI on ischaemic heart disease (IHD) is through high systolic blood pressure (SBP), cholesterol level, and FPG.[42] The proportion of the BMI effect that can be explained by other metabolic risk factors is the amount of mediation. The difference between the crude RR of BMI on IHD with the RR adjusted for SBP, FPG, and cholesterol level reflects the amount of BMI effect on IHD that is mediated and already included in SBP, FPG, and cholesterol level:

$$MF = \frac{RR_{crude} - RR_{adjusted}}{RR_{crude} - 1}$$

So, to aggregate the PAF of multiple risk factors, we first calculated the part of the excess risk (RR – 1) of every risk factor that is not mediated, re-compute the PAF so that it only includes the non-mediated risk then aggregated PAFs by assuming they are independent.

Therefore, if MF is the mediation factor of R2 through R1, the adjusted RR for R2 including only the non-mediated component of risk is:

$$RR_{1,2} = MF_{2/1}(RR_2 - 1) + 1$$

The PAF accounting for mediation is then computed using the adjusted RR and the joint PAF computed as detailed in Section 2.7.2. For every paired risk factor and outcome, the matrix of possible mediations was calculated and used.

Section 2.6.5: Calculating mediation factor

1 – Comparing crude RR versus mediator-adjusted RR

The best example is the mediation of BMI through SBP, FPG, and cholesterol level reported by Danaei et al.[42] In their meta-analysis, they report the adjusted and unadjusted RR of BMI on IHD and stroke based on combined data from individual cohorts. They calculated the MF by using the following equation, and we used it directly as the MF in risk factor aggregation. Using individual-level data from cohort studies, we estimated the MF for other metabolic risk factors and some dietary risks.

$$MF = \frac{RR_{crude} - RR_{adjusted}}{RR_{crude} - 1}$$

2 – Estimating the mediation factor by pathway of the effect

For many other risk factors, no data are available to enable the use of the first method. Instead, we searched studies to estimate the effect of the risk factor on the mediator and, finally, the expected increase in IHD risk. We pooled available studies to calculate the unit increase in the mediator per unit increase in the risk factor to calculate the size of the IHD RR (Figure D).

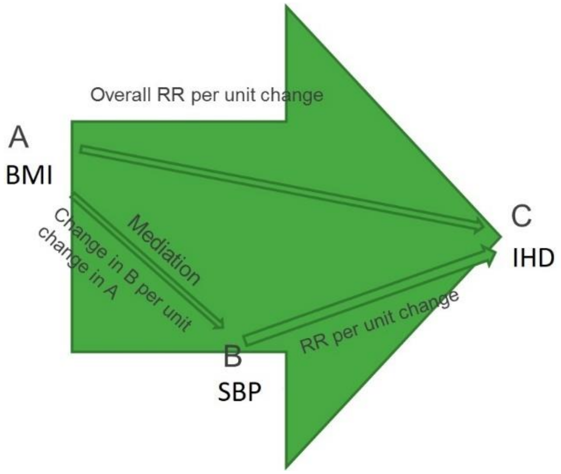

Figure D. Example of pathway between BMI, high systolic blood pressure, and ischemic heart disease

We have RRs for the effect of A on C and B on C in GBD from a meta-analysis of studies in the literature. The effect of A on B was estimated by analysis of trials.

$$RR_{ABC} = RR_{BC}{}^{\Delta_{AB}}$$

RR_{ABC} is the expected effect of A through B on C

RR_{BC} is the RR of each unit increase in mediator on outcome C

Δ_{AB} is the change in mediator level B per each unit change in A

If RR_{AB} is the overall effect of A on B, then:

$$MF = \frac{RR_{ABC} - 1}{RR_{AB} - 1}$$

We kept the uncertainty of each parameter to a minimum by generating and following 1000 draws of the estimates to calculate 1000 draws of the posterior distribution of the MF. We did not include risk-mediator pairs if the MF was not significant at the 5% level (more than 50 of 1000 draws were negative). We truncated the MF distribution at 1 when the whole effect of the risk factor on the outcome would be assumed to be exerted through the mediator pathway.

Some MFs equalled 1 when the whole effect was calculated through another risk factor (eg, the effect of salt through SBP) or when we assumed other risk factors were sources of the exposure (eg, fibre is provided by consuming fruit, vegetables, and whole grains, and all the beneficial effect of milk on colorectal cancer is mediated through calcium).

Air pollution

In GBD 2019, we considered mediation for particulate matter air pollution and SBP, FPG level, and cholesterol level, but in no case was the evidence strongly supportive. Review of the epidemiological evidence identified several cohort studies that reported increased prevalence and/or risk of hypertension due to long-term exposure to ambient $PM_{2.5}$, and several studies have found elevated SBP due to household solid fuel use as well. Studies of short-term exposure also reported acute elevations in blood pressure. However, there is not consensus as to whether the existing evidence with regard to the effects of long-term exposure is consistent with the current mechanistic understanding of the effect of air pollution exposure on blood pressure, and whether existing cohort studies have properly modelled that exposure.

Assumed mediations

For the risk factors with PAFs of 100%, such as FPG and diabetes, impaired kidney function and chronic kidney disease (CKD), SBP and hypertensive heart disease, alcohol and alcohol use disorders, child underweight and protein-energy malnutrition, child wasting and protein-energy malnutrition, and drug use and drug use disorders, no mediation is needed.

Section 2.6.6: Piecewise aggregation (Pattern 3)

There are three anthropometric indicators that are highly correlated: child underweight, stunting, and wasting, as shown in Figure E in this section. Available RRs for each indicator are not adjusted for the other two because these indicators are highly correlated and most of the burden occurs in an interaction. Estimating the total burden due to child growth failure, a latent variable, is difficult. The three anthropometric indicators are not independent, so the covariance between them should be considered. This consideration was the main reason that GBD 2010 only included child underweight. If covariance between these indicators is significant (as is shown in Figure E), aggregating these indicators by assuming they are independent would overestimate the total burden significantly.

To account for the high degree of correlation between CGF indicators, GBD uses a constrained optimisation method to adjust the observed univariate RRs that come out of the Burden of Proof analysis. First we created a joint distribution of stunting, underweight, and wasting from a population of children. Second, we generated 500 RR draws for each univariate indicator and severity based on the Burden of Proof analysis. Third, we altered these univariate RRs for the four causes (diarrhoea, LRI, malaria, and measles) and the two outcomes (mortality and morbidity) based upon interactions among the CGF indicators. An interaction occurs when the effect of one CGF indicator variable (eg, stunting) has a different effect on the outcome depending on the value of another CGF indicator variable (eg,

underweight). Interaction terms alter the risk of the outcome among children with more than one indicator of CGF. These interaction terms were extracted from a pooled cohort analysis of all-cause mortality published by McDonald et al.[43] Lastly, we optimised the adjusted relative risks by minimising the error between the observed RRs (generated from the Burden of Proof analysis) and the altered RRs derived from the joint distribution and accounting for the interaction terms while ensuring that no alteration resulted in a previously identified increase in relative risk becoming protective.

For GBD 2021, we made several changes to improve the four main steps of RR adjustment. From GBD 2013 to GBD 2019, a simulated joint distribution of stunting, underweight, and wasting measures was created from the Olofin et al. meta-analysis.[44] Sources in this meta-analysis were cross-sectional Demographic and Health Surveys (DHS).[44] In GBD 2021, we created age-specific joint distributions of stunting, underweight, and wasting measures from 15 longitudinal studies (from 26 locations) in the Ki database.[45] The RR adjustment method was strengthened in GBD 2021 by constraining optimisation in two ways. Optimisation was only permitted to alter the RR for an indicator/severity in draws where the observed RR was greater than 1, and constraints were placed on the error that penalise larger alterations to the RR. These changes enabled the estimation and utilisation of age-specific adjusted RRs for GBD 2021 burden estimation. The largest change for GBD 2021 was conducting Burden of Proof analyses for each cause/outcome/risk triplet using both data from Olofin et al as well as KI data. These changes result in identifying large differences in the relationship between CGF and mortality versus morbidity as well as identifying some impact of CGF on malaria.

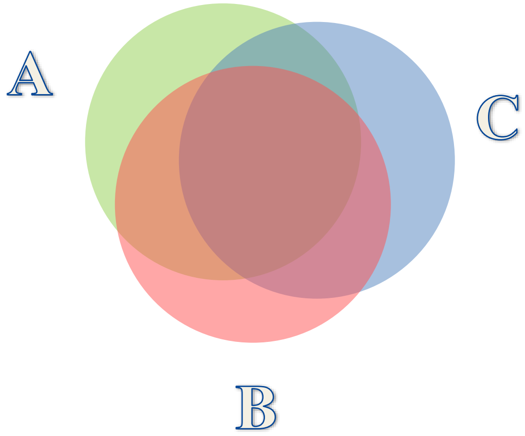

Figure E. Venn diagram demonstrating the correlation between child underweight, stunting, and wasting

After adjusting for the three risk factors, we calculated the PAFs and aggregated the underweight, stunting, and wasting burden.

Section 2.6.7: Uncertainty of aggregated and mediated PAFs

We generated 1000 draws of the posterior distribution of the MF calculated by different methods to use beside draws of other inputs to the PAF aggregation.

Section 2.6.8: Important assumptions in aggregating risk factors and including mediation

1 – The MFs or PAF adjustments are similar across countries, age, sex, and years. Although the size of mediation is probably different in different populations, few data are available to inform the covariance between different risk factors or the MF amount by age and country. For example, in some countries, the size of the mediated BMI-IHD PAF exerted through cholesterol level, as calculated by the MF, was even bigger than the total burden of cholesterol level. This finding indicated that less of the effect of BMI is mediated through cholesterol level and MFs are not similar across countries.

2 – For many risk-mediator-outcome pairs, no data are available, so we assumed the mediation is zero or one.

3 – Because the covariance between undernutrition indicators differs by location (and across time, but results were not reported), and an interaction exists between these indicators, the total burden might be underestimated.

4 – We assumed no significant covariance between PAFs, which might not be true between some risk factors, such as metabolic risk factors. Although this overestimation can be controlled by using adjusted RRs, using crude RRs for BMI and other metabolic risk factors may cause significant overestimation of the aggregated metabolic risks burden.

Step 7. Estimate attributable burden[5]

Four key components are included in the estimation of the burden attributable to a given risk factor: the metric of burden being assessed (the number of deaths, YLLs, YLDs, or DALYs [the sum of YLLs and YLDs]); the exposure levels for a risk factor; the RR of a given outcome due to exposure; and the counterfactual level of risk factor exposure. Uncertainty intervals for attributable burden were calculated using 500 draws for GBD 2021. Estimates of attributable burden as DALYs for risk–outcome pairs were generated by using the following model:

$$AB_{jasgt} = \sum_{o=1}^{w} DALY_{joasgt}\, PAF_{joasgt}$$

where AB_{jasgt} is the attributable burden for risk factor j for age group a, sex s, location g, and year t; $DALY_{joasgt}$ is total DALYs for cause o (of w relevant outcomes for risk factor j) for age group a, sex s, location g, and year t; and PAF_{joasgt} is the PAF for cause o due to risk factor j for age group a, sex s, location g, and year t. The proportions of deaths, YLLs, or YLDs attributable to a given risk factor or risk factor cluster were analogously computed by sequentially substituting each metric in place of DALYs in the equation provided.

Section 3: Decomposition analysis of deaths and DALYs[5]

We conducted a decomposition analysis of changes in DALYs from 2000 to 2021, decomposing changes in all-age cause-specific DALYs attributable to all risk factors and individual risk factors due to changes in population growth, population age structure, exposure to the given risk for a disease, and risk-deleted death and DALY rates. In this case, risk-deleted rates are the rates obtained after removing the effect of a risk factor or combination of risk factors — in other words, observed DALY rates multiplied by one minus the PAF for the risk or set of risks. Our decomposition analyses draw from methods developed by Das Gupta[46] to provide a computationally tractable solution for isolating drivers of burden changes

whereby all combinations of possible pathways are averaged across factors. Attributable burden was determined, following the methods of Das Gupta, as a product of three factors such that:

$$T_{asgt} = (A_{asgt}\, B_{asgt}\, C_{asgt})$$

where T_{asgt} represents the attributable burden at year t; $A_{sgt}$is the age-specific population size for a given age group a, sex s, and location g at year t; B_{asgt} is the underlying rate of the outcome unrelated to the risk factor or observed rate, multiplied by $1 - PAF$ for a given age group a, sex s, and location g at year t; and C_{asgt} is the ratio of the attributable burden to the underlying rate, which reflects the risk exposure effect for a given age group a, sex s, and location g at year t defined as $PAF/(1 - PAF)$when decomposing attributable burden to a risk. Risk exposure effects for individual risk factors are scaled such that they sum to the all-risk exposure effect by location, age, sex, and cause accounting for mediation. This process allows for aggregation of risks; the exposure for all risks for a disease can be split into exposure to metabolic, behavioural, and environmental risks. The contribution of each factor to total change in attributable burden was determined by changing the level of one factor from time t_0 to t_1 – here 2000 to 2021 – with all other factors held constant. Thus, the effect of any of the three factors, for example A_{asgt}on the change of the attributable burden between 2000 (A_{10}) and 2021 (A_{17}) is calculated as:

$$E_A = (A_{19} - A_{10})\left(\frac{B_{10}C_{10} + B_{19}C_{19}}{3} + \frac{B_{10}C_{19} + B_{19}C_{10}}{6}\right)$$

where E_A is the proportion of change due to factor A, and the subscripts for each factor in the equation denote the year for each estimate. Because the effect depends on the order of entry of the factor, we calculated the average of all combinations of the three factors. The proportion of change due to factorA_{sgt}, the age-specific population size for a given age group a, sex s, and location g at year t, is then further split, setting change in population growth equal to the percentage change in the all-age population from time t_0 to t_1 and change in population age structure to the residual, giving four factors.

This three-factor decomposition method does not work for risks for which the PAF, by definition, is 100% (such as high FPG level and type 2 diabetes) or for which the PAF is directly estimated (such as for unsafe sex and HIV). In the cases of child underweight and protein-energy malnutrition; child wasting and protein-energy malnutrition; short gestation for birthweight and neonatal preterm birth complications; low birthweight for gestation and neonatal preterm birth complications; iron deficiency (ID) and iron-deficiency anaemia (IDA); alcohol use and liver cancer due to alcohol use; alcohol use and cirrhosis and other chronic liver diseases due to alcohol use; alcohol use and alcohol use disorders; alcohol use and alcoholic cardiomyopathy; drug use and drug use disorders; occupational particulate matter, gases, and fumes and other pneumoconiosis; occupational particulate matter, gases, and fumes and coal workers pneumoconiosis; occupational exposure to asbestos and asbestosis; and occupational exposure to silica and silicosis, we used a two-factor decomposition method that examines the contribution of population, ageing, and risk exposure. Effectively, we assumed trends in these cases are driven by exposure, not change in the risk-deleted rates. Conversely, for unsafe sex and sexually transmitted diseases excluding HIV, we used a two-factor decomposition method that examines the contribution of population, ageing, and risk-deleted death and DALY rates and assumed that trends in these cases are driven by risk-deleted rates, not change in exposure. For high FPG level and type 1 and 2 diabetes, high FPG level and CKD due to type 1 and 2 diabetes, high SBP and hypertensive heart disease,

high SBP and CKD due to hypertension, and impaired kidney function and CKD, we used GBD estimates of SEVs for the given risk and the case-fatality rate to decompose trends into an estimate of the contribution of the three factors. Similarly, for unsafe sex and cervical cancer, we used GBD estimates of the incidence of cervical cancer and the case-fatality rate to decompose trends into an estimate of the contribution of the three factors. For unsafe sex and HIV, we used spectrum counterfactual and CD4 risk-weighted prevalence.

Section 4: Socio-demographic Index analysis[5]

Section 4.1: Development of the Socio-demographic Index

The SDI is a composite indicator of socio-demographic development status strongly correlated with health outcomes. In short, it is the geometric mean of 0 to 1 indices of total fertility rate in those under 25 years old (TFU25), mean education for those aged 15 years or older (EDU15+), and lag-distributed income per capita (LDI). For GBD 2021, after calculating SDI, values were multiplied by 100 for a scale of 0 to 100.

Section 4.2: Development of a revised SDI indicator

SDI was originally constructed for GBD 2015 by using the Human Development Index (HDI) methodology, wherein a 0 to 1 index value was determined for each of the original three covariate inputs (total fertility rate in those aged 15–49, EDU15+, and LDI per capita) by using the observed minima and maxima during the estimation period to set the scales.

During GBD 2016, we moved from using relative index scales to using absolute scales to enhance the stability of SDI interpretation over time because we noticed that the measure was highly sensitive to the addition of subnational units that tended to stretch the empirical minima and maxima. We selected the minima and maxima of the scales by examining the relationships each of the inputs had with life expectancy at birth and under-5 mortality and by identifying points of limiting returns at both high and low values if they occurred before theoretical limits (eg, a TFU25 of 0).

Thus, an index score of zero represents the minimum level of each covariate input past which selected health outcomes can get no worse, and an index score of 1 represents the maximum level of each covariate input past which selected health outcomes cease to improve. As a composite, a location with an SDI of zero would have a theoretical minimum level of socio-demographic development relevant to these health outcomes, and a location with an SDI of 1 (prior to multiplying by 100 for reporting purposes) would have a theoretical maximum level of socio-demographic development relevant to these health outcomes.

We computed the index scores underlying SDI as follows:

$$I_{Cly} = {(C_{ly} - C_{low})}\Big/{(C_{high} - C_{low})}$$

where I_{cly} – the index for covariate *C*, location *l*, and year *y* – is equal to the difference between the value of that covariate in that location-year and the lower bound of the covariate divided by the difference between the upper and lower bounds for that covariate.

If the values of input covariates fell outside the upper or lower bounds, they were mapped to the respective upper or lower bounds. We also note that the index value for TFU25 was computed as $1 -$

$I_{TFU25ly}$ because lower TFU25s correspond to higher levels of development and thus higher index scores. For GBD 2021, we computed SDI for 983 national and subnational locations spanning the time period 1950–2021.

The composite SDI is the geometric mean of these three indices for a given location-year. The cut-off values used to determine quintiles for analysis were then computed by using country-level estimates of SDI for the year 2021, excluding countries with populations less than 1 million. As stated above, for GBD 2021, final SDI values were multiplied by 100 in order to improve understanding of and broader engagement with the values, so final reporting values are on a 0 to 100 scale.

Example calculation

We present the following example calculation of SDI for "Country X":

$$TFU25 = 1.09;\ Mean\ educ\ yrs\ pc = 8.23;\ lnLDI = 9.60$$

$$I_{TFU25} = 1 - \frac{1.09 - 0}{3 - 0} = \ 0.637$$

$$I_{Educ} = \frac{8.23 - 0}{17 - 0} = \ 0.484$$

$$I_{lnLDI} = \frac{9.60 - 5.52}{11.00 - 5.52} = \ 0.744$$

$$SDI = \sqrt[3]{I_{TFU25} * I_{Educ} * I_{lnLDI}} = \sqrt[3]{.637 * .484 * \ .744} = \ 0.611$$

$$I_{lnLDI} = \frac{9.58 - 5.52}{11.00 - 5.52} = \ 0.741$$

$$SDI = \sqrt[3]{I_{TFR} * I_{Educ} * I_{lnLDI}} = \sqrt[3]{.855 * .543 * \ .741} = \ 0.701$$

$$GBD\ 2019\ reporting\ SDI = \ 0.701 * 100 = 70.1$$

SDI values by location can be found in table S5.

Section 5: References

1 Stevens GA, Alkema L, Black RE. Guidelines for Accurate and Transparent Health Estimates Reporting: the GATHER statement. *Lancet* 2016.

2 Lim SS, Vos T, Flaxman AD. A comparative risk assessment of burden of disease and injury attributable to 67 risk factors and risk factor clusters in 21 regions, 1990–2010: a systematic analysis for the Global Burden of Disease Study 2010. *The Lancet* 2012; **380**: 2224–60.

3 Forouzanfar M, Afshin A, Alexander LT, Anderson H, Bhutta Z, Murray CJL. Global, regional, and national comparative risk assessment of 79 behavioural, environmental and occupational, and metabolic risks or clusters of risks, 1990-2015: a systematic analysis for the Global Burden of Disease Study 2015. *Lancet* 2016; **388**: 1659–724.

4 Gakidou E, Afshin A, Abajobir AA, *et al.* Global, regional, and national comparative risk assessment of 84 behavioural, environmental and occupational, and metabolic risks or clusters of risks, 1990–2016: a systematic analysis for the Global Burden of Disease Study 2016. *The Lancet* 2017; **390**: 1345–422.

5 Stanaway JD, Afshin A, Gakidou E, *et al.* Global, regional, and national comparative risk assessment of 84 behavioural, environmental and occupational, and metabolic risks or clusters of risks for 195 countries and territories, 1990–2017: a systematic analysis for the Global Burden of Disease Study 2017. *The Lancet* 2018; **392**: 1923–94.

6 Murray CJL, Aravkin AY, Zheng P. Global burden of 87 risk factors in 204 countries and territories, 1990–2019: a systematic analysis for the Global Burden of Disease Study 2019. *The Lancet* 2020; **396**: 1223–49.

7 Murray CJ, Lopez AD. Global mortality, disability, and the contribution of risk factors: Global Burden of Disease Study. *Lancet* 1997; **349**: 1436–42.

8 Murray CJ, Lopez AD. On the comparable quantification of health risks: lessons from the Global Burden of Disease Study. *Epidemiology* 1999; **10**: 594–605.

9 Food, nutrition, physical activity and the prevention of cancer: a global perspective. Washington, D.C: World Cancer Research Fund & American Institute for Cancer Research, 2007.

10 Zheng P, Barber R, Sorensen RJ, Murray CJ, Aravkin AY. Trimmed constrained mixed effects models: formulations and algorithms. *Journal of Computational and Graphical Statistics* 2021; : 1–13.

11 Zheng P. limetr: limetr: linear mixed effects model with trimming. https://github.com/zhengp0/limetr (accessed July 28, 2021).

12 Zheng P. xspline: xspline: Advanced spline tools. https://github.com/zhengp0/xspline (accessed July 28, 2021).

13 Viechtbauer W. Conducting meta-analyses in R with the metafor package. *Journal of statistical software* 2010; **36**: 1–48.

14 de Boor C. A practical guide to splines (applied mathematical sciences, 27). New York: Springer, 2001 https://link.springer.com/book/9780387953663 (accessed April 4, 2022).

15 Rousseeuw PJ, Leroy AM. Robust regression and outlier detection. John wiley & sons, 2005.

16 Rousseeuw PJ. Least median of squares regression. *Journal of the American Statistical Association* 1984; **79**: 871–80.

17 Huber PJ. Robust Statistics. John Wiley & Sons, 2004.

18 Rousseeuw P. Multivariate estimation with high breakdown point. *Mathematical Statistics and Applications Vol B* 1985; 283–97.

19 Aravkin A, Davis D. Trimmed statistical estimation via variance reduction. *Mathematics of Operations Research* 2020; **45**: 292–322.

20 Motzkin TS, Raiffa H, Thompson GL, Thrall RM. 3. The Double Description Method. In: 3. The Double Description Method. Princeton University Press, 2016: 51–74.

21 Guyatt GH, Oxman AD, Vist G, *et al.* GRADE guidelines: 4. Rating the quality of evidence—study limitations (risk of bias). *Journal of clinical epidemiology* 2011; **64**: 407–15.

22 Efron B, Hastie T, Johnstone I, Tibshirani R. Least angle regression. *The Annals of statistics* 2004; **32**: 407–99.

23 Tibshirani R. Regression shrinkage and selection via the lasso. *Journal of the Royal Statistical Society: Series B (Methodological)* 1996; **58**: 267–88.

24 Kontopantelis E, Springate DA, Reeves D. A re-analysis of the Cochrane Library data: the dangers of unobserved heterogeneity in meta-analyses. *PloS one* 2013; **8**: e69930.

25 Ioannidis JP, Patsopoulos NA, Evangelou E. Uncertainty in heterogeneity estimates in meta-analyses. *Bmj* 2007; **335**: 914–6.

26 Biggerstaff BJ, Tweedie RL. Incorporating variability in estimates of heterogeneity in the random effects model in meta-analysis. *Statistics in medicine* 1997; **16**: 753–68.

27 Higgins JP, Thompson SG. Quantifying heterogeneity in a meta-analysis. *Statistics in medicine* 2002; **21**: 1539–58.

28 Sterne JA, Egger M. Chapter 6: Regression methods to detect publication and other bias in meta-analysis. In: Rothstein H, Sutton A, Borenstein M, eds. Publication bias in meta-analysis: Prevention, assessment and adjustments. John Wiley & Sons, Ltd, 2005.

29 Egger M, Smith GD, Schneider M, Minder C. Bias in meta-analysis detected by a simple, graphical test. *BMJ* 1997; **315**: 629–34.

30 Burkart KG, Brauer M, Aravkin AY. Estimating the cause-specific relative risks of non-optimal temperature on daily mortality: a two-part modelling approach applied to the Global Burden of Disease Study. *The Lancet* 2021; **398**: 685–97.

31 Harris PA, Taylor R, Thielke R, Payne J, Gonzalez N, Conde JG. Research Electronic Data Capture (REDCap) - A metadata-driven methodology and workflow process for providing translational research informatics support. *J Biomed Inform* 2009; **42**: 377–81.

32 GBD 2015 Diseases and Injury Incidence and prevalence Collaborators. Global, regional, and national incidence, prevalence, and years lived with disability (YLDs) for 310 acute and chronic diseases and injuries, 1990-2015: a systematic analysis for the Global Burden of Disease Study 2015. *Lancet* 2016 Oct 8;388(10053):1545-1602.

33 Flaxman AD, Vos T, Murray CJL, editors. *An integrative metaregression framework for descriptive epidemiology*, 1 edition. Seattle: University of Washington Press, 2015.

34 Vasudevan S, Ramos F, Nettleton E, Durrant-Whyte H, Blair A. Gaussian Process modeling of large scale terrain. In: 2009 IEEE International Conference on Robotics and Automation. 2009: 1047–53.

35 Rasmussen CE, Williams CKI. Gaussian Processes for Machine Learning. Cambridge, Mass: The MIT Press, 2005.

36 Ng M, Fleming T, Robinson M, *et al.* Global, regional, and national prevalence of overweight and obesity in children and adults during 1980–2013: a systematic analysis for the Global Burden of Disease Study 2013. *Lancet* 2014; **384**: 766–81.

37 Ng M, Freeman MK, Fleming TD, *et al.* Smoking Prevalence and Cigarette Consumption in 187 Countries, 1980-2012. *JAMA* 2014; **311**: 183–92.

38 Massey Jr FJ. The Kolmogorov-Smirnov test for goodness of fit. *Journal of the American statistical Association* 1951; **46**: 68–78.

39 Vander Hoorn S, Ezzati M, Rodgers A, Lopez AD, Murray CJL. Estimating attributable burden of disease from exposure and hazard data. In: Comparative Quanitifcation of Health Risks: Global and regional burden of disease attribution to selected major risk factors. World Health Organisation, 2004: 2129–40.

40 Preston SH. Causes and Consequences of Mortality Declines in Less Developed Countries during the Twentieth Century. In: Population and economic change in developing countries. Chicago: Univ. of Chicago Pr, 1980: 289–360.

41 Carnahan E, Lim SS, Nelson EC, *et al.* Validation of a new predictive risk model: measuring the impact of major modifiable risks of death for patients and populations. *The Lancet* 2013; **381**: S26.

42 Danaei G, Singh GM, Paciorek CJ, *et al.* The global cardiovascular risk transition: associations of four metabolic risk factors with national income, urbanization, and Western diet in 1980 and 2008. *Circulation* 2013; **127**: 1493–502, 1502e1-8.

43 McDonald CM, Olofin I, Flaxman S. The effect of multiple anthropometric deficits on child mortality: meta-analysis of individual data in 10 prospective studies from developing countries. *Am J Clin Nutr* 2013; **97**: 896–901.

44 Olofin I, McDonald CM, Ezzati M, et al. Associations of suboptimal growth with all-cause and cause specific mortality in children under five years: a pooled analysis of ten prospective studies. *PloS ONE*; **8**: e64636.

45 Ki Global Health. (n.d.). https://www.kiglobalhealth.org/.

46 Das Gupta P. Standardization and Decomposition of Rates: A User's Manual. Washington D.C.: U.S. Bureau of the Census, 1993.

47 WHO | WHO Global Database on Child Growth and Malnutrition. WHO. http://www.who.int/nutgrowthdb/en/ (accessed July 30, 2018).

48 Wang Y, Chen H-J. Use of Percentiles and Z-Scores in Anthropometry. In: Preedy VR, ed. Handbook of Anthropometry. New York, NY: Springer New York, 2012: 29–48.

49 Uribe Á, Cecilia M, López Gaviria A, Estrada Restrepo A. Concordance between Z scores from WHO 2006 and the NCHS 1978 growth standards of children younger than five. Antioquia-Colombia. *Perspectivas en Nutrición Humana* 2008; **10**: 177–87.

50 McDonald CM, Olofin I, Flaxman S, *et al.* The effect of multiple anthropometric deficits on child mortality: meta-analysis of individual data in 10 prospective studies from developing countries. *Am J Clin Nutr* 2013; **97**: 896–901.

51 Cole TJ, Lobstein T. Extended international (IOTF) body mass index cut-offs for thinness, overweight and obesity. *Pediatr Obes* 2012; **7**: 284–94.

52 Global, regional, and national comparative risk assessment of 79 behavioural, environmental and occupational, and metabolic risks or clusters of risks, 1990–2015: a systematic analysis for the Global Burden of Disease Study 2015. *Lancet* 2016; **388**: 1659–724.

53 Stanaway JD, Afshin A, Gakidou E, *et al.* Global, regional, and national comparative risk assessment of 84 behavioural, environmental and occupational, and metabolic risks or clusters of risks for 195 countries and territories, 1990–2017: a systematic analysis for the Global Burden of Disease Study 2017. *The Lancet* 2018; **392**: 1923–94.

54 Global Health Data Exchange | GHDx. http://ghdx.healthdata.org/ (accessed Oct 8, 2021).

55 Friedewald WT, Levy RI, Fredrickson DS. Estimation of the concentration of low-density lipoprotein cholesterol in plasma, without use of the preparative ultracentrifuge. *Clin Chem* 1972; **18**: 499–502.

56 Murray CJL, Aravkin AY, Zheng P, *et al.* Global burden of 87 risk factors in 204 countries and territories, 1990–2019: a systematic analysis for the Global Burden of Disease Study 2019. *The Lancet* 2020; **396**: 1223–49.

57 Singh GM, Danaei G, Farzadfar F, *et al.* The Age-Specific Quantitative Effects of Metabolic Risk Factors on Cardiovascular Diseases and Diabetes: A Pooled Analysis. *PLOS ONE* 2013; **8**: e65174.

58 Wang N, Fulcher J, Abeysuriya N, *et al.* Intensive LDL cholesterol-lowering treatment beyond current recommendations for the prevention of major vascular events: a systematic review and meta-analysis of randomised trials including 327 037 participants. *Lancet Diabetes Endocrinol* 2020; **8**: 36–49.

59 Moher D, Liberati A, Tetzlaff J, Altman DG, Group TP. Preferred Reporting Items for Systematic Reviews and Meta-Analyses: The PRISMA Statement. *PLOS Medicine* 2009; **6**: e1000097.

60 Ng M, Fleming T, Robinson M, *et al.* Global, regional, and national prevalence of overweight and obesity in children and adults during 1980–2013: a systematic analysis for the Global Burden of Disease Study 2013. *The Lancet* 2014; **384**: 766–81.

61 Boekholdt SM, Hovingh GK, Mora S, *et al.* Very Low Levels of Atherogenic Lipoproteins and the Risk for Cardiovascular Events: A Meta-Analysis of Statin Trials. *J Am Coll Cardiol* 2014; **64**: 485–94.

62 Vos T, Lim SS, Abbafati C, *et al.* Global burden of 369 diseases and injuries in 204 countries and territories, 1990–2019: a systematic analysis for the Global Burden of Disease Study 2019. *The Lancet* 2020; **396**: 1204–22.

63 Zheng P, Barber R, Sorensen RJD, Murray CJL, Aravkin AY. Trimmed Constrained Mixed Effects Models: Formulations and Algorithms. *Journal of Computational and Graphical Statistics* 2021; **30**: 544–56.

64 Sorensen (rsoren@uw.edu) R. Health Metrics Toolbox. https://ihmeuw-msca.github.io/index.html (accessed Oct 8, 2021).

65 Bias in meta-analysis detected by a simple, graphical test | The BMJ. https://www.bmj.com/content/315/7109/629.full (accessed Oct 8, 2021).

66 Prospective Studies Collaboration. Collaborative overview ('meta-analysis’) of prospective observational studies of the associations of usual blood pressure and usual cholesterol levels with common causes of death: protocol for the second cycle of the Prospective Studies Collaboration. *J Cardiovasc Risk* 1999; **6**: 315–20.

67 The effect of Blood Pressure on Kidney Failure: a systematic review and meta-analysis in 2.7 million participants [Unpublished] | GHDx. https://internal-ghdx.healthdata.org/record/effect-blood-pressure-kidney-failure-systematic-review-and-meta-analysis-27-million (accessed July 11, 2023).

68 Rapsomaniki E, Timmis A, George J, *et al.* Blood pressure and incidence of twelve cardiovascular diseases: lifetime risks, healthy life-years lost, and age-specific associations in 1·25 million people. *Lancet* 2014; **383**: 1899–911.

69 Denaxas SC, George J, Herrett E, *et al.* Data Resource Profile: Cardiovascular disease research using linked bespoke studies and electronic health records (CALIBER). *Int J Epidemiol* 2012; **41**: 1625–38.

70 Page MJ, McKenzie JE, Bossuyt PM, *et al.* The PRISMA 2020 statement: an updated guideline for reporting systematic reviews. *BMJ* 2021; **372**: n71.

71 Singh GM, Danaei G, Farzadfar F, *et al.* The age-specific quantitative effects of metabolic risk factors on cardiovascular diseases and diabetes: a pooled analysis. *PLoS ONE* 2013; **8**: e65174.

72 Collaboration APCS, others. Blood pressure and cardiovascular disease in the Asia Pacific region. *Journal of hypertension* 2003; **21**: 707–16.

73 Hersbach H, Bell B, Berrisford P, *et al.* The ERA5 global reanalysis: 2019. DOI:10.21957/vf291hehd7.

74 Copernicus Climate Change Service (C3S) (2017): ERA5: Fifth generation of ECMWF atmospheric reanalyses of the global climate. Copernicus Climate Change Service Climate Data Store (CDS), September 2019.

75 Geography and Environmental Science, University of Southampton. Age and Sex Structures, Global Per Country 2000-2020 - WorldPop. Southampton, United Kingdom: Geography and Environmental Science, University of Southampton, 2018.

76 Zheng P, Aravkin AY, Barber R, Sorensen RJD, Murray CJL. Trimmed Constrained Mixed Effects Models: Formulations and Algorithms. 2019; published online Sept 23.

77 Rousseeuw PJ. Least Median of Squares Regression. *Journal of the American Statistical Association* 1984; **79**: 871–80.

78 Aravkin A, Davis D. Trimmed Statistical Estimation via Variance Reduction. *Mathematics of Operations Research* 2019; moor.2019.0992.

79 Yang E, Lozano AC, Aravkin A. A general family of trimmed estimators for robust high-dimensional data analysis. *Electronic Journal of Statistics* 2018; **12**: 3519–53.

80 Pya N, Wood SN. Shape constrained additive models. *Statistics and Computing* 2015; **25**: 543–59.

81 Roth GA, Abate D, Abate KH, *et al.* Global, regional, and national age-sex-specific mortality for 282 causes of death in 195 countries and territories, 1980-2017: a systematic analysis for the Global Burden of Disease Study 2017 GBD 2017 Causes of Death Collaborators*. 2018 DOI:10.1016/S0140-6736(18)32203-7.

82 Ng M, Freeman MK, Fleming TD, *et al.* Smoking prevalence and cigarette consumption in 187 countries, 1980-2012. *JAMA* 2014; **311**: 183–92.

83 Zheng P, Aravkin A, Barber R, Sorensen R, Murray C. Trimmed Constrained Mixed Effects Models: Formulations and Algorithms. *bioRxiv* 2020; 2020.01.28.923599.

84 International Zinc Nutrition Consultative Group (IZiNCG), Brown KH, Rivera JA, *et al.* International Zinc Nutrition Consultative Group (IZiNCG) technical document #1. Assessment of the risk of zinc deficiency in populations and options for its control. *Food Nutr Bull* 2004; **25**: S99-203.

Section 6: GBD 2021 risk factor-specific modelling descriptions

1. Ambient nitrogen dioxide pollution
2. Ambient ozone pollution
3. Ambient particulate matter pollution
4. Bone mineral density
5. Bullying victimization
6. Chewing tobacco
7. Child growth failure
8. Childhood sexual abuse
9. Dietary risks
10. Drug use
11. High alcohol use
12. High bodymass index
13. High fasting plasma glucose
14. High LDL cholesterol
15. High systolic blood pressure
16. Household air pollution
17. Intimate partner violence
18. Iron deficiency
19. Kidney dysfunction
20. Lead exposure
21. Low birthweight and short gestation
22. Low physical activity
23. No access to handwashing facility
24. Non-optimal temperature
25. Occupational risk factors
26. Residential radon pollution
27. Secondhand smoke
28. Smoking
29. Suboptimal breastfeeding
30. Unsafe sanitation
31. Unsafe sex
32. Unsafe water
33. Vitamin A deficiency
34. Zinc deficiency

Ambient nitrogen dioxide pollution

Flowchart

Nitrogen Dioxide

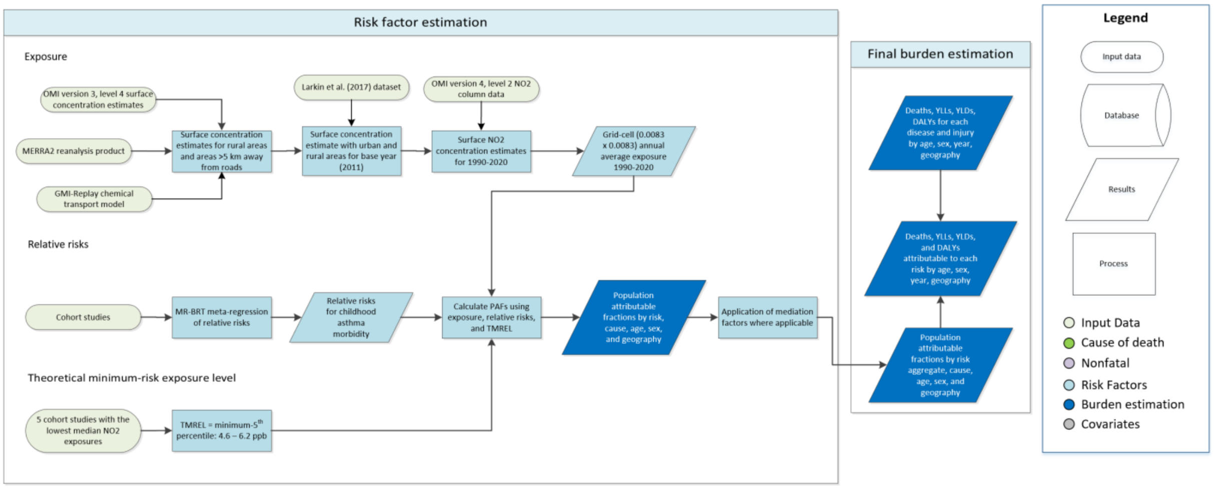

Input data and methodological summary

Exposure

Definition

Exposure to nitrogen dioxide (NO_2) pollution is defined as the population-weighted annual average ambient concentration of NO_2 gas measured in parts per billion (ppb).

Input data

The NO_2 exposure modelling process for the Global Burden of Disease (GBD) Study 2021 combines multiple and varied input data sources. These include ground measurements, satellite column measurements and satellite-based surface concentration estimates, land-use regression-based surface concentration estimates, urbanicity data, and population estimates.

Table 1: Data inputs for exposure for nitrogen dioxide pollution

	Countries with data	New sources	Total sources
Exposure	204	3876	3876

Land-use regression estimates

The first NO_2 exposure model input is a global NO_2 pollution surface concentration dataset published in 2017 by Larkin and colleagues, hereafter referred to as the Larkin et al. (2017) NO_2 dataset.[1] This dataset estimates a three-year average annual NO_2 concentration from the years 2010, 2011, and 2012. Land-use regression modelling was used to produce this dataset, with data inputs from road networks, other land use variables, ground measurements of NO_2, and satellite NO_2 column observations from the SCIAMACHY and GOME-2 satellite products (Larkin et al., 2017; Geddes et al., 2016).

Satellite-based and chemical transport model products

Satellite-based and chemical transport model inputs include the OMI NO_2 version 3, level 4 surface concentration dataset, MERRA2 reanalysis product (satellite-derived surface concentration estimates), GMI-Replay chemical transport model, and OMI version 4.0 level 2 annual average NO_2 column product. The OMI version 3, level 4 surface concentration estimates were derived from OMI satellite instrument NO_2 column observations following methods described by Lamsal et al., 2008 and were obtained from the NASA Goddard Space Flight Center (GSFC).[2] Though the newer version 4 OMI retrieval uses enhanced surface reflectivities in the calculation of the tropospheric column amounts, surface concentrations prepared by NASA GSFC are not currently available from the version 4 product.

Population and urbanicity data

The Global Human Settlement Model grid (Pesaresi et al. 2019) was used to classify grid cells as "urban" or "rural." Global population data on a high-resolution grid for the years 2000–2020 was obtained from the WorldPop database.[3] These data were provided at a resolution of $0.0083^{o} \times 0.0083^{o}$, which corresponds to the resolution of the GBD 2021 global NO_2 surface concentration estimates. Population estimates for 1990 and 1995 were estimated through an extrapolation process. For each $0.0083^{o} \times 0.0083^{o}$ grid cell, where the population growth rate for 2000–2005 was positive, the growth rate was applied backward in time to extrapolate prior population concentrations. Where the growth rate was negative, the population value for the year 2000 was used directly for prior years.

Modelling strategy

Surface concentration estimates for 2011

The following is a summary of the GBD 2021 global NO_2 exposure estimation approach, developed and implemented by Arash Mohegh, Dan Goldberg, and Susan Anenberg at George Washington University. For GBD 2021, surface annual average NO_2 concentrations were estimated at 1 km x 1 km resolution in five-year increments from 1990 to 2010 and annually from 2010 to 2019. Estimation was first conducted for 2011, the base year available from the Larkin et al. (2017) dataset.

To begin, the Larkin et al. (2017) NO_2 concentration dataset was aggregated from its native 100 m x 100 m resolution globally to 1 km x 1 km grid cells. In a previous study, Mohegh and colleagues determined that 1 km x 1 km is the optimum resolution for NO_2-attributable burden estimation to minimise computational resources while retaining a high enough resolution for accurate estimates.[4] Due to the lack of ground measurements in rural areas, the Larkin et al. (2017) dataset is finely tuned toward urban areas and overestimates NO_2 concentrations in rural areas, likely due to a high sensitivity to the normalised difference vegetation index (NDVI), one of the model's input land-use variables.

To better estimate NO_2 surface concentrations across urban and rural locations, multiple global NO_2 surface concentration estimates were combined. Given its good performance in urban areas, the Larkin et al. (2017) NO_2 dataset was applied in all 1 km x 1 km grid cells globally that are categorized as "urban" according to the Global Human Settlement Model grid.[5] The Larkin et al. (2017) dataset was also used for grid cells situated near major roadways. For grid cells >5 km away from roadways and in urban areas, new NO_2 surface concentration estimates were developed using a combination of satellite-based and chemical transport model products as described below.

For rural areas, NO_2 estimates derived from the OMI satellite instrument were used, with some adjustments to fill spatial and temporal gaps in the OMI satellite record and to estimate 24-hour

averages from the early afternoon OMI overpass time. The OMI NO_2 version 3, level 4 surface concentration dataset (0.1° x 0.1° resolution) was the basis for estimating NO_2 annual 2011 concentrations. Due to the lack of satellite dataset coverage over snow- and ice-covered areas, some grid cells, primarily in higher latitudes, were missing OMI observations for some months. The MERRA 2 surface-level reanalysis product (0.625° x 0.625° resolution) was used to generate a set of grid-cell-specific correction factors to ensure availability of NO_2 concentrations in all locations and months. Correction factors were calculated and applied to each grid cell missing complete OMI coverage in 2011 using the following approach:

$$Correction\ factor\ 1 = \frac{MERRA2_{annual\ average}}{MERRA2_{average\ for\ months\ with\ OMI\ level\ 4\ availability}}$$

A second correction factor was applied to convert surface NO_2 concentrations from the early afternoon OMI overpass time (13:00 local time) to 24-hour averages. NO_2 surface concentration estimates from the GMI-Replay chemical transport model simulations (2° x 2.5° resolution) were used to generate a correction factor for each grid cell, following the protocol described by Anenberg and colleagues (2018). These correction factors were calculated as follows:

$$Correction\ factor\ 2 = \frac{GMI_{24-hour\ average}}{GMI_{13:00}}$$

The NO_2 surface concentration estimates used for grid cells >5 km away from roads and in rural areas were then generated using the following formula:

$$Adjusted\ rural\ concentrations = OMI\ level\ 4\ \times Correction\ factor\ 1\ \times Correction\ factor\ 2$$

For rural grid cells within 5 km of major roadways, NO_2 surface concentration estimates were linearly scaled between the Larkin et al. (2017) values and the new adjusted rural concentrations for the span of the 5 km distance.

The methods described above produced a 1 km x 1 km annual average surface NO_2 concentration dataset for 2011 that uses Larkin et al. (2017) values in grid cells categorised as urban or near roadways, and a new surface concentration dataset derived from OMI satellite observations in rural areas and those >5 km away from roadways.

Figure 1: NO_2 surface concentration estimates, 2011

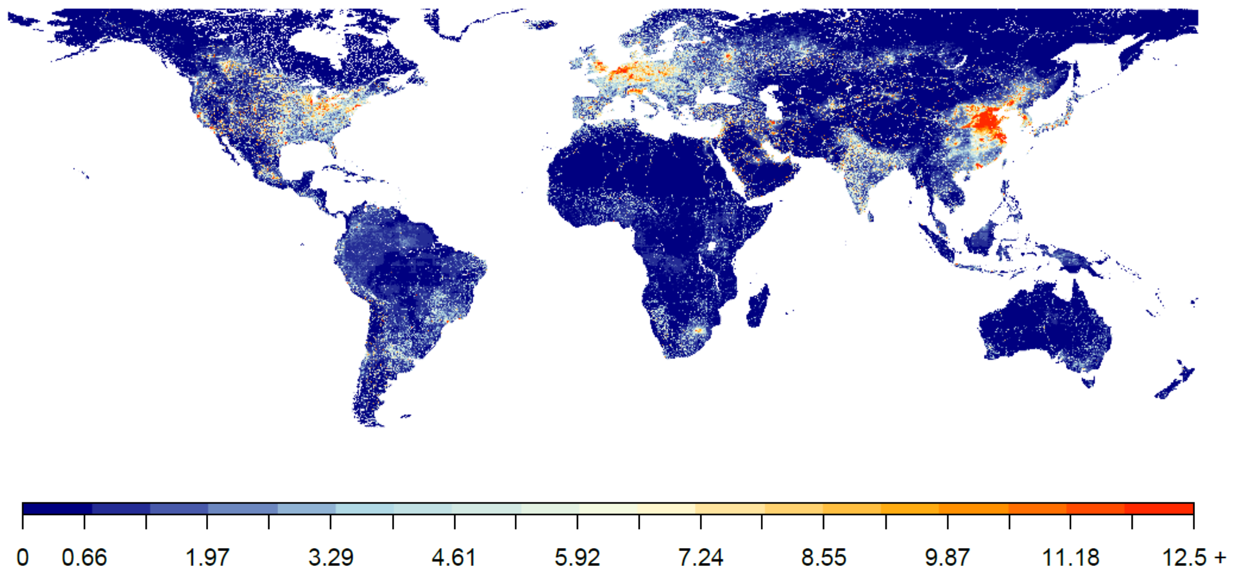

Scaling to GBD estimation years

This 2011 base-year surface NO_2 concentration dataset was then scaled to each year required for GBD burden analysis: 1990–2020 in five-year increments from 1990 to 2005 and annually from 2010 to 2020. For 2010–2018, surface NO_2 concentrations for 2011 were scaled to each year using three-year rolling averages of annual average NO_2 columns from the OMI version 4.0 level 2 product (13 km x 25 km resolution at nadir). NO_2 columns were used because surface concentrations derived from the version 4 OMI retrieval are not yet available. The NO_2 column dataset was oversampled to a 0.1° x 0.1° resolution and regridded to a resolution of 0.0083° x 0.0083° (approximately 1 km x 1 km). Three-year rolling averages were used to remove noise from the satellite data.

Estimates for 2005, 2019, and 2020 were scaled with satellite data from their respective years as described above; however, three-year rolling averages were not used. Instead, each year's NO_2 columns were used directly. 2005 and 2020 lacked sufficient data to create three-year averages, and due to the dramatic changes from the COVID-19 pandemic, 2020 estimates were not used to inform estimates for 2019.

The years 1990, 1995, and 2000 predate the OMI observational record. For these years, the NO_2 concentrations from the MERRA2 reanalysis product were used to scale the 2011 NO_2 surface concentrations to those years.[6] To remove model noise, these scaling factors were created across world regions (Giorgi Regions) as opposed to applying grid-cell-specific scaling factors.[7] These steps resulted in a global 0.0083° x 0.0083° (approximately 1 km x 1 km) resolution dataset of annual average surface NO_2 concentrations from 1990 to 2020.

Uncertainty estimation

To incorporate estimates of model uncertainty, evaluation results for the Larkin et al. (2017) NO_2 concentration dataset were used. The mean absolute error (MAE) results in the table below were used to generate distributions of each grid cell's exposure for use in burden assessment. The Larkin et al.

(2017) evaluation regions are not equivalent to GBD regions; for the north Africa and Middle East region, the mean of the Larkin et al. (2017) MAE estimates for Asia and Africa was used. For the central Latin America Region, the MAE estimate for South America was used. Due to lack of information on posterior correlation relationships, we chose a conservative approach and assumed complete correlation between deviation estimates when generating exposure distributions for each grid cell.

Table 2: Mean absolute error (ppb) of NO_2 surface concentration estimate for the Larkin et al. (2017) dataset by region

Region	Mean absolute error (ppb)
North America	3.8
South America	2.6
Europe	3.3
Africa	2.4
Asia	3.5
Oceania	2.3

Theoretical minimum risk exposure level

To calculate population attributable fractions (PAFs), we used a theoretical minimum risk exposure level (TMREL) of a uniform distribution between 4.6 and 6.2 ppb. This TMREL was calculated by imputing the 0.1 and 5th percentiles of study-specific exposure distributions (when the minimum and/or 5th percentile was unreported) for all NO_2 relative risk cohort studies that reported a median and standard deviation. The TMREL bounds are the means of the minimum/0.1 and 5th percentiles for the five studies with the lowest reported median values. A uniform distribution was used instead of a single fixed value to convey uncertainty in scientific literature regarding the level at which no harmful effects of NO_2 pollution exist. This TMREL calculation method is consistent with that used for the other GBD ambient air pollution risk factors, ambient particulate matter pollution and ozone.

Relative risks and population attributable fractions

Input data and systematic review

To assess the dose–response relationship between NO_2 exposure and paediatric asthma, we first extracted the 31 NO_2-related component studies from a recent (2017) and comprehensive meta-analysis of traffic-related air pollution's effects on childhood asthma development conducted by Khreis and colleagues.[8] We then searched PubMed and Embase for updates to relevant literature published from September 9, 2016, to December 19, 2019 (date of search), using the search string below.

Search string: (((((("child*") AND "air pollution") AND "asthma")) OR ((("child*") AND "air quality") AND "asthma")) OR ((("child*") AND "vehicle emissions") AND "asthma")) OR ((("child*") AND "ultra-fine particles") AND "asthma")

We excluded all cross-sectional studies, as this type of study design is less robust. Apart from this exclusion, we employed inclusion and exclusion criteria identical to those used in the Khreis et al., 2017 meta-analysis. Input data included in the GBD 2021 NO_2-childhood asthma relative risk analysis are as follows, with all sources detailed below:

Table 3: Data inputs for relative risks for nitrogen dioxide pollution

	Countries with data	New sources	Total sources
Relative risks	12	27	27

Figure 2: PRISMA diagram for systematic review of nitrogen dioxide pollution and childhood asthma

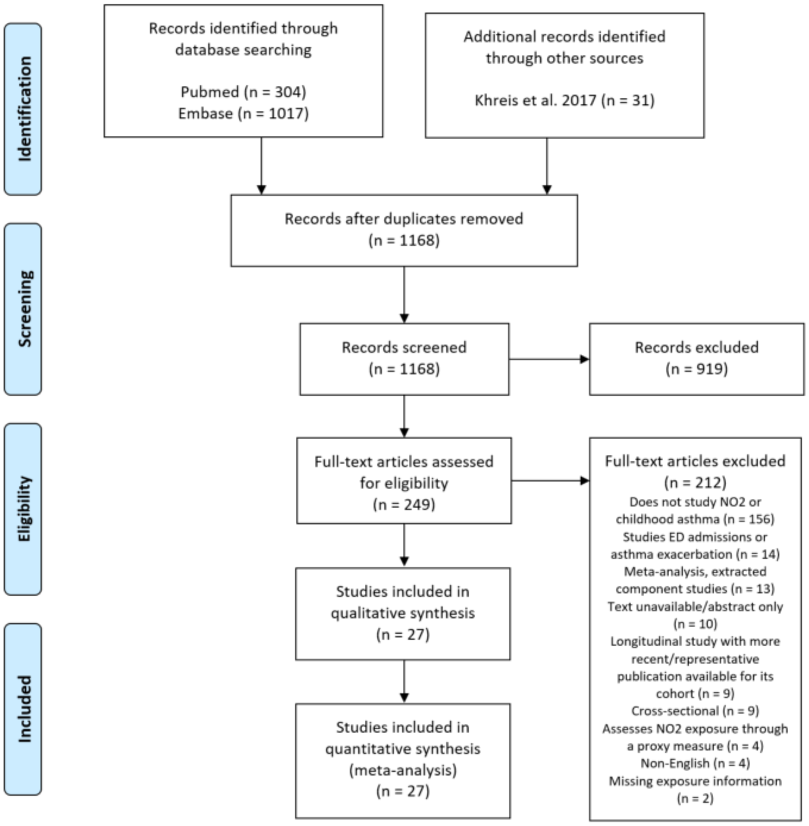

Table 4: Studies included in NO_2-paediatric asthma meta-analysis

Source	Citation
1	Brauer M, Hoek G, Smit HA, de Jongste JC, Gerritsen J, Postma DS, Kerkhof M, Brunekreef B. Air pollution and development of asthma, allergy and infections in a birth cohort. Eur Respir J. 2007; 29(5): 879-88.
2	Carlsten C, Dybuncio A, Becker A, Chan-Yeung M, Brauer M. Traffic-related air pollution and incident asthma in a high-risk birth cohort. Occup Environ Med. 2011; 68(4): 291-5.
3	Clark NA, Demers PA, Karr CJ, Koehoorn M, Lencar C, Tamburic L, Brauer M. Effect of Early Life Exposure to Air Pollution on Development of Childhood Asthma. Environ Health Perspect. 2010; 118(2): 284-90.
4	Clougherty JE, Levy JI, Kubzansky LD, Ryan PB, Suglia SF, Canner MJ, Wright RJ. Synergistic effects of traffic-related air pollution and exposure to violence on urban asthma etiology. Environ Health Perspect. 2007; 115: 1140-6.
5	Dell SD, Jerrett M, Beckerman B, Brook JR, Foty RG, Gilbert NL, Marshall L, Miller JD, To T, Walter SD, Stieb DM. Presence of other allergic disease modifies the effect of early childhood traffic-related air pollution exposure on asthma prevalence. Environ Int. 2014; 65: 83-92.
6	Deng Q, Lu C, Norbäck D, Bornehag CG, Zhang Y, Liu W, Yuan H, Sundell J. Early life exposure to ambient air pollution and childhood asthma in China. Environ Res. 2015; 143: 83-92.

7	Deng Q, Lu C, Ou C, Chen L, Yuan H. Preconceptional, prenatal and postnatal exposure to outdoor and indoor environmental factors on allergic diseases/symptoms in preschool children. Chemosphere. 2016; 152: 459-67.
8	Fuertes E, Standl M, Cyrys J, Berdel D, von Berg A, Bauer CP, Krämer U, Sugiri D, Lehmann I, Koletzko S, Carlsten C, Brauer M, Heinrich J. A longitudinal analysis of associations between traffic-related air pollution with asthma, allergies and sensitization in the GINIplus and LISAplus birth cohorts. PeerJ. 2003; 1: e193.
9	Gehring U, Wijga AH, Hoek G, Bellander T, Berdel D, Brüske I, Fuertes E, Gruzieva O, Heinrich J, Hoffmann B, de Jongste JC, Klümper C, Koppelman GH, Korek M, Krämer U, Maier D, Melén E, Pershagen G, Postma DS, Standl M, von Berg A, Anto JM, Bousquet J, Keil T, Smit HA, Brunekreef B. Exposure to air pollution and development of asthma and rhinoconjunctivitis throughout childhood and adolescence: a population-based birth cohort study. Lancet Respir Med. 2015; 3(12): 933-42.
10	Jerrett M, Shankardass K, Berhane K, Gauderman WJ, Kunzli N, Avol E, Gilliland F, Lurmann F, Molitor JN, Molitor JT, Thomas DC, Peters J, McConnell R. Traffic-Related Air Pollution and Asthma Onset in Children: A Prospective Cohort Study with Individual Exposure Measurement. Environ Health Perspect. 2008; 116(10): 1433-8.
11	Kravitz-Wirtz N, Teixeira S, Hajat A, Woo B, Crowder K, Takeuchi D. Early-Life Air Pollution Exposure, Neighborhood Poverty, and Childhood Asthma in the United States, 19902014. Int J Environ Res Public Health. 2018; 15(6).
12	Krämer U, Sugiri D, Ranft U, Krutmann J, von Berg A, Berdel D, Behrendt H, Kuhlbusch T, Hochadel M, Wichmann HE, Heinrich J, GINIplus and LISAplus study groups. Eczema, respiratory allergies, and traffic-related air pollution in birth cohorts from small-town areas. J Dermatol Sci. 2009; 56(2): 99-105.
13	Lavigne É, Bélair MA, Rodriguez Duque D, Do MT, Stieb DM, Hystad P, van Donkelaar A, Martin RV, Crouse DL, Crighton E, Chen H, Burnett RT, Weichenthal S, Villeneuve PJ, To T, Brook JR, Johnson M, Cakmak S, Yasseen AS 3rd, Walker M. Effect modification of perinatal exposure to air pollution and childhood asthma incidence. Eur Respir J. 2018.
14	Liu W, Huang C, Hu Y, Fu Q, Zou Z, Sun C, Shen L, Wang X, Cai J, Pan J, Huang Y, Chang J, Sun Y, Sundell J. Associations of gestational and early life exposures to ambient air pollution with childhood respiratory diseases in Shanghai, China: A retrospective cohort study. Environ Int. 2016; 92-93: 284-93.
15	McConnell R, Islam T, Shankardass K, Jerrett M, Lurmann F, Gilliland F, Gauderman J, Avol E, Künzli N, Yao L, Peters J, Berhane K. Childhood incident asthma and traffic-related air pollution at home and school. Environ Health Perspect. 2010; 118(7): 1021-6.
16	Morgenstern V, Zutavern A, Cyrys J, Brockow I, Koletzko S, Krämer U, Behrendt H, Herbarth O, von Berg A, Bauer CP, Wichmann HE, Heinrich J, GINI Study Group, LISA Study Group. Atopic diseases, allergic sensitization, and exposure to traffic-related air pollution in children. Am J Respir Crit Care Med. 2008; 177(12): 1331-7.
17	Mölter A, Agius R, de Vocht F, Lindley S, Gerrard W, Custovic A, Simpson A. Effects of long-term exposure to PM10 and NO2 on asthma and wheeze in a prospective birth cohort. J Epidemiol Community Health. 2014; 68(1): 21-8.
18	Nishimura KK, Galanter JM, Roth LA, Oh SS, Thakur N, Nguyen EA, Thyne S, Farber HJ, Serebrisky D, Kumar R, Brigino-Buenaventura E, Davis A, LeNoir MA, Meade K, Rodriguez-Cintron W, Avila PC, Borrell LN, Bibbins-Domingo K, Rodriguez-Santana JR, Sen Ś, Lurmann F, Balmes JR, Burchard EG. Early-life air pollution and asthma risk in minority children. The GALA II and SAGE II studies. Am J Respir Crit Care Med. 2013; 188(3); 309-18.
19	Norbäck D, Lu C, Wang J, Zhang Y, Li B, Zhao Z, Huang C, Zhang X, Qian H, Sun Y, Sundell J, Deng Q. Asthma and rhinitis among Chinese children - Indoor and outdoor air pollution and indicators of socioeconomic status (SES). Environ Int. 2018; 115: 1-8.
20	Oftedal B, Nystad W, Brunekreef B, Nafstad P. Long-term traffic-related exposures and asthma onset in schoolchildren in Oslo, Norway. Environ Health Perspect. 2009; 117(5): 839-44.
21	Ranzi A, Porta D, Badaloni C, Cesaroni G, Lauriola P, Davoli M, Forastiere F. Exposure to air pollution and respiratory symptoms during the first 7 years of life in an Italian birth cohort. Occup Environ Med. 2014; 71(6): 430-6.
22	Sbihi H, Koehoorn M, Tamburic L, Brauer M. Asthma Trajectories in a Population-based Birth Cohort. Impacts of Air Pollution and Greenness. Am J Respir Crit Care Med. 2017; 195(5): 607-613.
23	Shima M, Adachi M. Effect of outdoor and indoor nitrogen dioxide on respiratory symptoms in schoolchildren. Int J Epidemiol. 2000; 29(5): 862-70.
24	Shima M, Nitta Y, Ando M, Adachi M. Effects of air pollution on the prevalence and incidence of asthma in children. Arch Environ Health. 2002; 57(6): 529-35.
25	To T, Zhu J, Stieb D, Gray N, Fong I, Pinault L, Jerrett M, Robichaud A, Ménard R, van Donkelaar A, Martin RV, Hystad P, Brook JR, Dell S. Early Life Exposure to Air Pollution and Incidence of Childhood Asthma, Allergic Rhinitis and Eczema. Eur Respir J. 2019.

26	Tétreault LF, Doucet M, Gamache P, Fournier M, Brand A, Kosatsky T, Smargiassi A. Childhood Exposure to Ambient Air Pollutants and the Onset of Asthma: An Administrative Cohort Study in Québec. Environ Health Perspect. 2016; 124(8): 1276-82.
27	Voros K, Koi T, Magyar D, Rudnai P, Paldy A. The influence of air pollution on respiratory allergies, asthma and wheeze in childhood in Hungary. Minerva Pediatr. 2019.

When extracting data for analysis, we also extracted a set of study-specific covariates for use in quantifying unexplained between-study heterogeneity. These covariates include subpopulation, individual- or population-level exposure, self-reported exposure, exposure measured multiple times throughout the study or only at baseline, unblinded assessment of outcome or exposure, a randomised study design, uncontrolled confounders (age, sex, secondhand smoke, parental asthma/allergy), selection bias, follow-up duration (gold standard data extracted as greater than or equal to 85% follow-up), and controlled for PM (2.5 or 10).

Modelling strategy

Meta-analysis

To assess the summary effect size of NO_2 exposure on childhood asthma, we conducted a meta-analysis using the meta-regression—Bayesian, regularised, trimmed (MR-BRT) tool, a meta-regression tool used widely across GBD risk factor teams.[9] We first tested fitting a third-order spline with three interior knots on the dataset; however, this analysis determined that there is currently insufficient evidence across the global NO_2 exposure range to support a non-linear relationship between NO_2 and childhood asthma. The final model we used was a log-linear meta-analysis weighting each estimated effect size by its inverse standard error and allowing each estimate to inform the meta-analysis between the 5th and 95th percentiles of its study-specific exposure distribution. 10% of all observations were trimmed during model fitting, in accordance with GBD protocol across risk factor teams.

We made two adjustments to prevent a single cohort or study from unduly weighting the final estimate. When multiple individual publications were available for the same cohort, we extracted only the peak RR estimate across follow-up time to account for patterns of asthma incidence and remission across age. Additionally, the standard error of observations from studies with multiple observations for a single cohort reporting an unstratified sample size were multiplied by the square root of n, where n is the total number of observations for a given cohort.

We performed covariate selection to empirically select significant covariates from those extracted to quantify between-study heterogeneity. The MR-BRT automated covariate selection tool implements a two-step process. First, a series of loosening Lasso penalty parameters are applied to a log-linear meta-regression on all input effect size observations. Then, covariates with a non-zero coefficient are tested for significance using a Gaussian prior (significance threshold = 0.05). Significant covariates selected were confounding uncontrolled, selection bias, and self-reported outcome. A Gaussian prior was used on each covariate's beta during curve fitting with a mean 0 and variance of 0.1 multiplied by the standard deviation of the beta from the initial log-linear meta-regression.

1000 predictions of the effect size were generated across the exposure distribution for use in calculating burden estimates. These predictions were created incorporating predictions of between-study heterogeneity and Fisher information. The Fisher scoring correction corrects for data-sparse situations when applied to the heterogeneity parameter. In such cases, the between-study heterogeneity parameter estimate may be 0, simply from lack of data. The Fisher scoring correction uses a quantile of

gamma, which is sensitive to the number of studies, study design, and reported uncertainty. The summary relative risk for childhood asthma at 5 ppb NO_2 exposure is 1.082 (95% UI 1.010–1.160) relative to 0 ppb.

Table 5: MR-BRT relative risk model parameters for nitrogen dioxide pollution

Covariate	Gamma (95% UI)	Beta coefficient, log (95% UI)
Exposure (per 1 ppb)	3.536×10^{-5} (1.658×10^{-5} – 6.455×10^{-5})	0.0155 (0.0085 – 0.0224)
Confounding uncontrolled		-1.344×10^{-6} (-6.955×10^{-4} – 7.007×10^{-4})
Selection bias (<85%)		1.201×10^{-6} (-6.729×10^{-4} – 6.607×10^{-4})
Self-reported outcome		7.709×10^{-7} (-6.359×10^{-4} – 7.156×10^{-4})

Figure 3: Nitrogen dioxide pollution and childhood asthma risk literature funnel plot

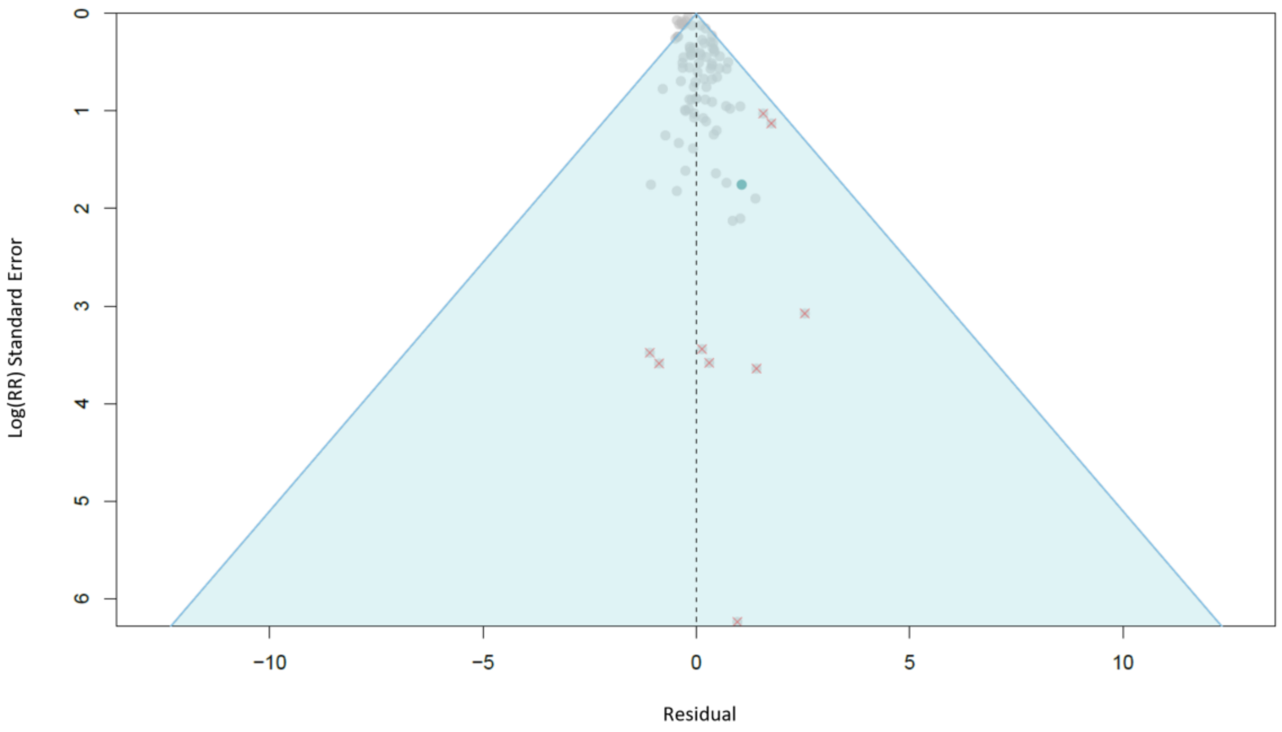

Figure 4: Nitrogen dioxide pollution and childhood asthma log-linear relative risk curve

Each number represents one study effect size. Each study's effect size is plotted at the 95th percentile of its study-specific NO_2 exposure distribution. The relative risk is plotted relative to the predicted relative risk at the 5th percentile of the study-specific exposure distribution.

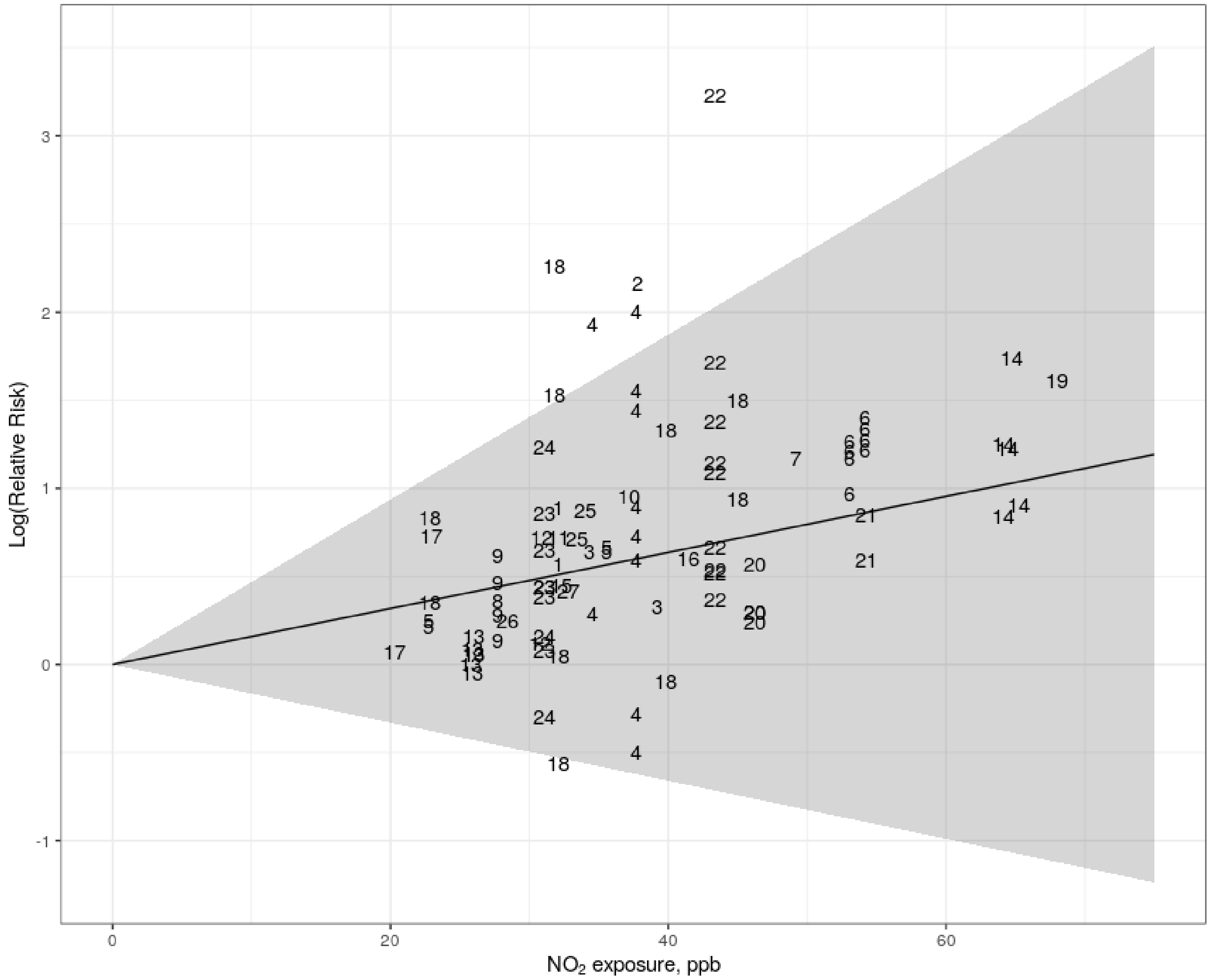

Risk-outcome scoring

Risk-outcome scores provide an empirical measure of the strength of evidence for risk–outcome pairs across risk factors in the GBD and are therefore useful for standardised comparison. Risk-outcome scores evaluate the area between the lower bound of the 95% uncertainty interval and the x-axis for harmful risk factors, including NO_2 pollution.

Prior to generating a risk-outcome score, we conducted an additional post-analysis step to detect and flag publication bias in the input data. This approach is based on the classic Egger's regression strategy, which is applied to the residuals in our model. In the current implementation, we do not correct for publication bias, but flag the risk–outcome pairs where the risk for publication bias is significant. We flagged NO_2-childhood asthma for publication bias after detecting significant association between observation residuals and their standard errors (p-value = 0.014, Egger mean = –0.244, Egger SD =

0.111). To calculate the risk-outcome score, we generated an uncertainty interval from 500 draws of the adjusted summary effect size (retaining uncertainty information from between-study heterogeneity predictions and the Fisher information boost). We then evaluated the risk-outcome score between the 15th and 85th percentiles of the input data exposure distribution (9.99–53.00 ppb). The final risk-outcome score is –0.51, which corresponds to a star rating of 1.

Population attributable fractions

PAFs were calculated for individuals between 0 and 19 years of age and contributed to morbidity estimates only. This is because available literature provides sufficient evidence for NO_2's association with childhood asthma incidence (not adult asthma or asthma-related mortality).[10] PAFs were calculated on the 0.0083° x 0.0083° (~1 x 1 km) grid-cell level. We aggregated PAFs to most-detailed GBD modeling locations using population rasters from the WorldPop Database (described above).

References

1. Larkin, A., Geddes, J. A., Martin, R. V, Xiao, Q., Liu, Y., Marshall, J. D., … Hystad, P. (2017). Global Land Use Regression Model for Nitrogen Dioxide Air Pollution. Environmental Science & Technology, 51(12), 6957–6964. https://doi.org/10.1021/acs.est.7b01148.
2. Lamsal LN, Martin R V., van Donkelaar A, Steinbacher M, Celarier EA, Bucsela E, et al. 2008. Ground-level nitrogen dioxide concentrations inferred from the satellite-borne Ozone Monitoring Instrument. J Geophys Res Atmos 113:1–15; doi:10.1029/2007JD009235.
3. Geography and Environmental Science, University of Southampton, WorldPop. Age and Sex Structures, Global Per Country 2000-2020. Southampton, United Kingdom: WorldPop, 2018.
4. Mohegh A., Goldberg D., Achakulwisut P., Anenberg S. Sensitivity of estimated NO2-attributable pediatric asthma incidence to grid resolution and urbanicity. Environ. Res. Lett. 2021; 16: 14019. https://doi.org/10.1088/1748-9326/abce25
5. Pesaresi, Martino; Florczyk, Aneta; Schiavina, Marcello; Melchiorri, Michele; Maffenini, Luca (2019): GHS settlement grid, updated and refined REGIO model 2014 in application to GHS-BUILT R2018A and GHS-POP R2019A, multitemporal (1975-1990-2000-2015), R2019A. European Commission, Joint Research Centre (JRC) DOI: 10.2905/42E8BE89-54FF-464E-BE7B-BF9E64DA5218
6. Gelaro, R., McCarty, W., Suárez, M. J., Todling, R., Molod, A., Takacs, L., Randles, C. A., Darmenov, A., Bosilovich, M. G., Reichle, R., Wargan, K., Coy, L., Cullather, R., Draper, C., Akella, S., Buchard, V., Conaty, A., da Silva, A. M., Gu, W., … Zhao, B. (2017): The Modern-Era Retrospective Analysis for Research and Applications, Version 2 (MERRA-2). J. Climate, 30, 5419–5454, https://doi.org/10.1175/JCLI-D-16-0758.1.
7. Giorgi, F., Francisco, R. Uncertainties in regional climate change prediction: a regional analysis of ensemble simulations with the HADCM2 coupled AOGCM. Climate Dynamics **16,** 169–182 (2000). https://doi-org.offcampus.lib.washington.edu/10.1007/PL00013733
8. Khreis H, Kelly C, Tate J, Parslow R, Lucas K, Nieuwenhuijsen M. Exposure to traffic-related air pollution and risk of development of childhood asthma: A systematic review and meta-analysis. Environ Int. 2017 Mar; 100:1-31. doi: 10.1016/j.envint.2016.11.012. Epub 2016 Nov 21. PMID: 27881237.
9. Zheng P, Barber R, Sorensen RJD, Murray CJL, Aravkin AY. Trimmed Constrained Mixed Effects Models: Formulations and Algorithms. J Comput Graph Stat. Published online February 12, 2021:1-13. doi:10.1080/10618600.2020.1868303

10. Achakulwisut P, Brauer M, Hystad P, Anenberg SC. Global, national, and urban burdens of paediatric asthma incidence attributable to ambient NO_2 pollution: estimates from global datasets. Lancet Planet Health. 2019; 3 (4): E166-E178. DOI:https://doi.org/10.1016/S2542-5196(19)30046-4.

Ambient ozone pollution

Flowchart

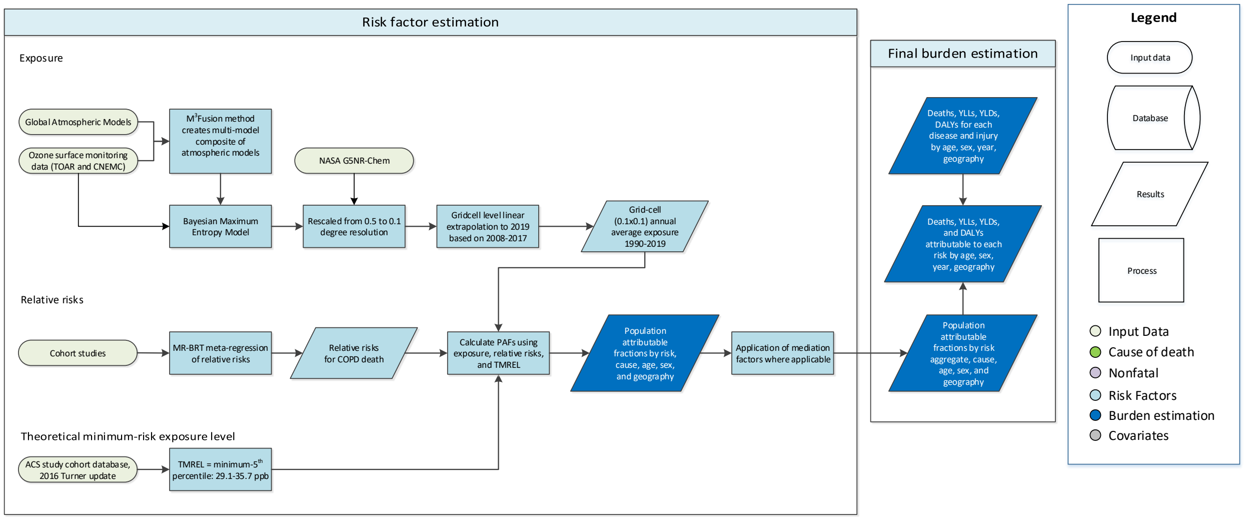

Input data and methodological summary

Exposure

Definition

Exposure to ambient ozone pollution is defined as the population-weighted highest seasonal (6-month) average of 8-hour daily maximum ozone concentrations. This measurement is reported in parts per billion (ppb).

Input data

Table 1: Data inputs for exposure for ambient ozone pollution

Input data	Exposure
Site-years (total)	5712
Number of countries with data	204
Number of GBD regions with data (out of 21 regions)	21
Number of GBD super-regions with data (out of seven super-regions)	7

To estimate the global distribution of exposure to ambient ozone pollution for the years 1990 to 2017, ozone ground measurement data were combined with chemical transport model estimates using Bayesian maximum entropy. Exposure estimates for 2018–2020 were extrapolated from these results, as described below.

1) Measurements and model combination

1.1) Measurement data

Ozone monitoring data were obtained from the Tropospheric Ozone Assessment Report (TOAR), which contains the world's largest collection of surface ozone metrics (DeLang et al., 2021; Schultz et al., 2017). Since TOAR has released data until 2015 to the public, an update was made to include readily available datasets until 2017. In addition to TOAR, our analysis included ozone data from the China National Environmental Monitoring Center (CNEMC) Network, which contains surface ozone measurements for 2013–2017 in China (Lu et al., 2018). All observations were processed to provide the 6-month ozone season average of 8-hour daily maximum ozone concentrations.

1.2) Model combination

We used a combination of global atmospheric chemical transport models in our analysis, many of which simulated specified dynamics for the Chemistry-Climate Model Initiative (CCMI). Note that some of these modelling teams completed extra years of simulations beyond 2010 specifically for this project. The eight models and years available include the following: CHASER (1990–2010), MOCAGE (1988–2016), MRI-ESM (1988–2017), NASA MERRA2-GMI (1988–2017), NCAR CESM-Chem (1988–2010), NCAR WACCM (1988–2010), GFDL AM3 (1988–2014), and GFDL AM4 (2010–2016).

We obtained hourly ozone data for each of these models and then calculated the six-month maximum daily eight-hour maximum ozone mixing ratio (ppb). The M^{3}Fusion method (Chang et al., 2019) was used to create a multi-model composite of the specified-dynamics models in each year from 1990 to 2017. This multi-model composite finds the linear combination of models available for each year that minimises the mean square error as compared to the observations in each world region, and in the process, it corrects to minimise the mean model bias in each region. The world was divided geographically into eight regions: North America, South America, Europe, Africa, south central Asia, east Asia, Russia, and Oceania. In every region, each model was weighted to minimise the difference between the multi-model average and observations as described by the following:

Let s_g be the grid cell at resolution 0.5° × 0.5°, $\hat{y}(sg)$ be the interpolated observations, $\{\eta_k(sg); k = 1, \ldots, n\}$ be the model output registered onto the same grid from the n models available in a given year. α_r is a constant that allows adjustment to the overall (regional) underestimation or overestimation and β_{rk} is an optimal weight for the k-th model in region r.

$$\underset{\{\alpha_r, \beta_{rk}; k=1,..,n\}}{minimize} \sum_{s_g \in Region\ r} \left(\hat{y}(s_g) - \alpha_r - \sum_{k=1}^{n} \beta_{rk}\eta_k(s_g) \right)^2,$$

$$subject\ to \sum_{k=1}^{n} \beta_{rk} = 1\ and\ \beta_{rk} \geq 0$$

In the M^{3}Fusion method, weights are constrained to be positive and sum to 1. A constant offset, α_r was included to guarantee that the residuals from this optimisation have a zero mean, through which the mean model bias is corrected (Chang et al., 2019). In most regions and years, the multi-model mean ozone was biased high, so this method tends to decrease the average ozone.

Since the M^{3}Fusion method relies on surface measurements to change the weights, regions with sparse data had to be taken into account. North America and Europe use weights-based model and observation values for each individual year. The rest of the world regions (South America, Africa, south central Asia,

east Asia, Russia, and Oceania) use individual year weights for 2000–2010, and apply weights calculated from the aggregated 2000 to 2010 period for 1990–1999. For 2011–2017, east Asia uses individual year weights, while South America, Africa, south central Asia, Russia, Oceania, and Antarctica use weights from the aggregated 2011–2014 period.

An example of the weighted values used to create the M^{3}Fusion model in North America and Europe are shown below, accompanied by a map of the M^{3}Fusion model in 2005.

	North America		Europe	
Year	*1995*	*2005*	*1995*	*2005*
CESM-Chem	0.38	0.29		
CHASER	0.04			
GFDL-AM3			0.18	0.62
MERRA		0.32	0.82	0.38
MOCAGE	0.36	0.01		
MRI-ESM	0.22	0.27		
WACCM		0.11		

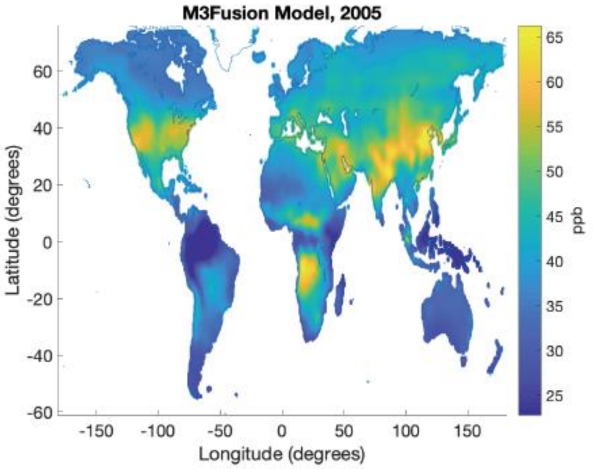

2) Bayesian maximum entropy (BME)

BME is a geostatistical modelling tool that can be used to combine various knowledge bases for an air pollutant and combine them to create a single product. In this case, we use BME to combine site-specific measurements and modelled concentrations, making use of the correlations between measurement locations. BME uses the measurement values to correct the M^{3}Fusion Model locally around each station spatially and temporally, allowing future and past observations to provide input. Since more measurement locations became available through time, this method allows later measurements to influence ozone surfaces earlier in the period, which is particularly important in China and data-sparse regions. The range over which each measurement can correct the M^{3}Fusion Model and how each measurement's impact decreases over distance in time and space are calculated as part of BME.

Beyond combining these knowledge bases to provide an estimate of ozone pollution, BME also estimates a variance, which can be used to assess estimation confidence at different locations.

In short, the steps are:

1. Let **Z(p)** be a field of ozone concentration estimations in space and time and let **mo(p)** be the M^{3}Fusion model output values in space and time.
2. Subtract the M^{3}Fusion model output values at each measure point from the observed values, **z(p)**, to obtain residuals **x(p)**=**z(p)**-**mo(p)**. Examples for 2005 of **z** and **x** are shown below on the left and right, respectively:

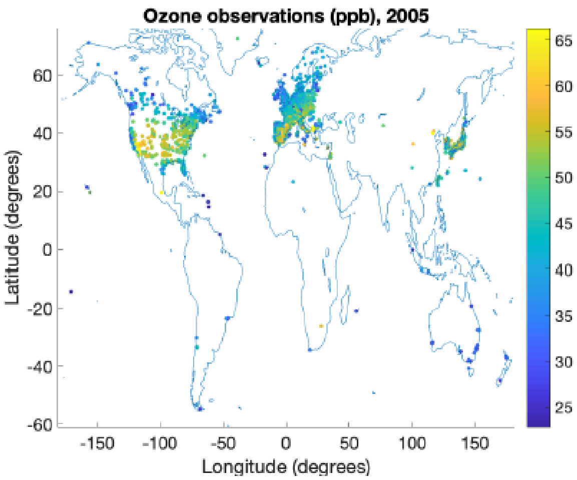

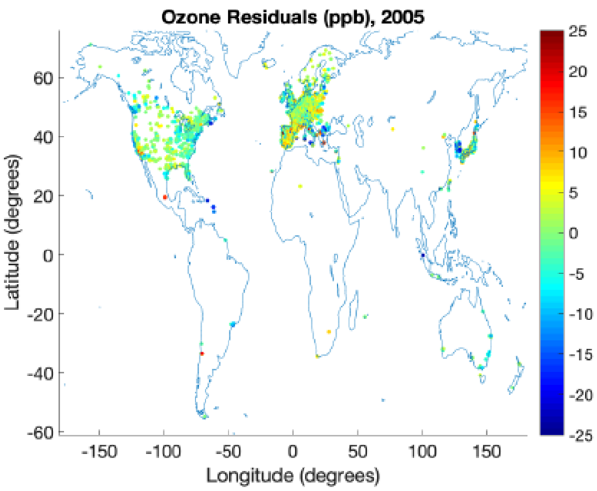

3. Model the covariance (the correlation between locations in space and time) $\mathbf{c_x}$ based on the residuals $\mathbf{x}$. The covariance of the residuals is the range of influence of a measurement to predict other concentrations in space and time. A shallower curve indicates that ozone values are correlated over a greater distance, while a steep drop-off indicates the reverse. The modelled spatial and temporal covariance are displayed below with the corresponding equation:

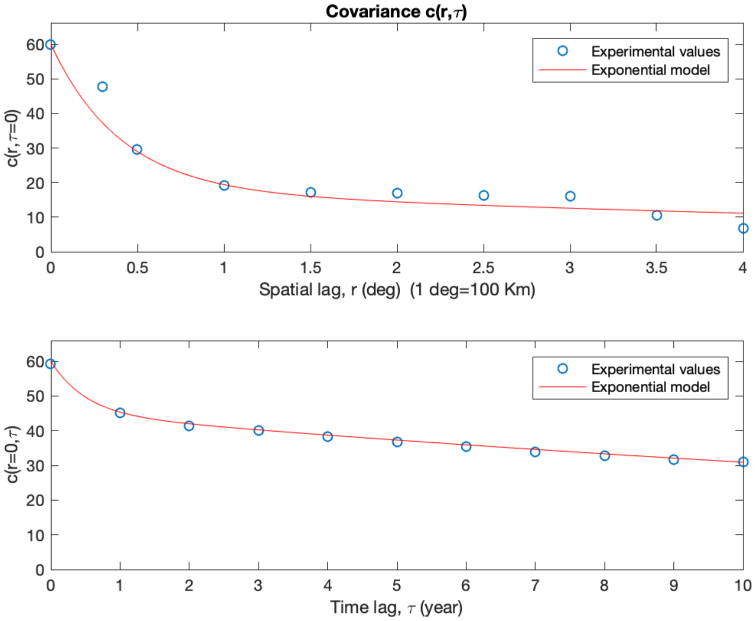

$$C_x(r,\tau) = 59.9938\ ppb^2(0.7\exp\left(-\frac{3r}{1.2\ degrees}\right)\exp\left(-\frac{3\tau}{80\ years}\right) + 0.05\exp\left(-\frac{3r}{25\ degrees}\right)\exp\left(-\frac{3\tau}{80\ years}\right) + 0.25\exp\left(-\frac{3r}{25\ degrees}\right)\exp\left(-\frac{3\tau}{1.5\ years}\right)$$

Where τ is temporal distance and r is spatial distance

Note that the spatial covariance drops off steeply spatially such that the influence of a measurement location becomes very small beyond 1 degree of distance. However, the temporal covariance remains high, meaning that observations can influence ozone estimates through time over several years.

4. Combine the observation data residuals **(x)**, covariance **(cx),** and estimation parameters to get the BME estimation **(xk)** and variance **(vk)** on a 0.5º by 0.5º grid, shown for 2005.

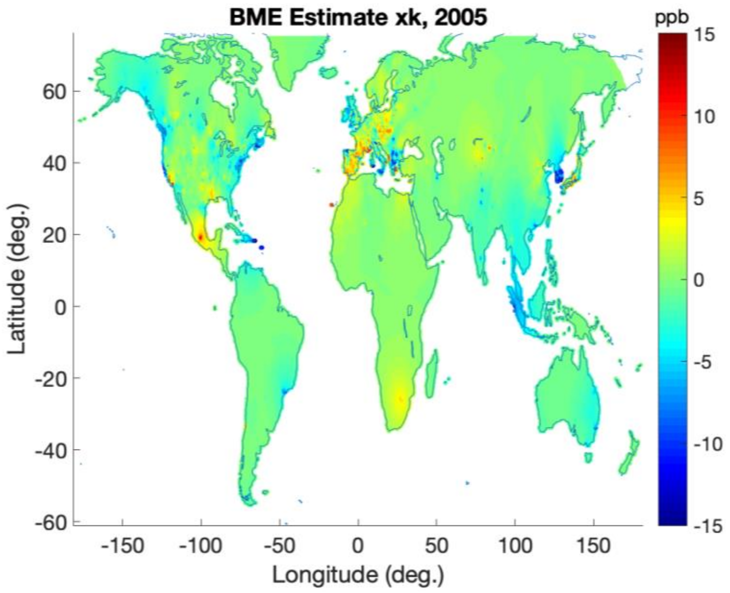

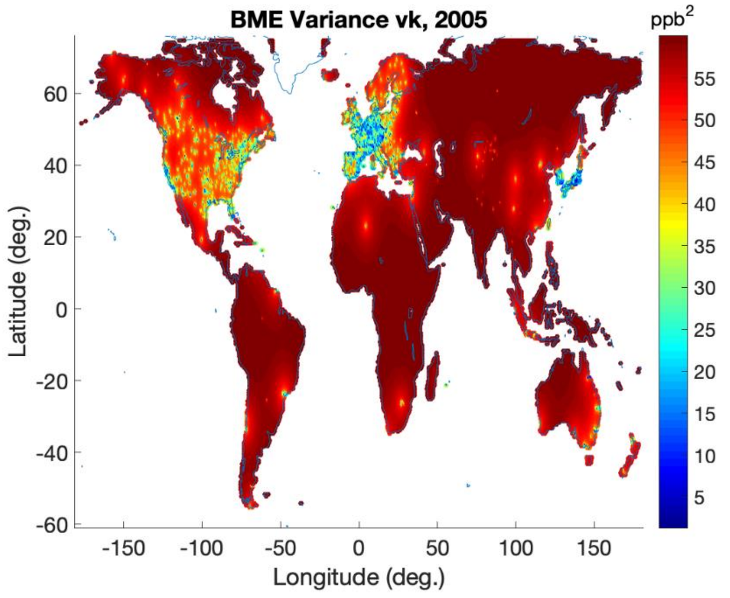

The variance is zero where the location of the estimation point matches an observation point in that year. The variance increases as the space time distance from an observation increases, until the variance reaches a maximum value equal to the sill of the covariance equation (59.9938 ppb^2).

5. Obtain final BME estimation values **(z_k)**, shown for 2005 in the figure below, by adding back the previously subtracted model values **mo(p_k)** to the BME estimation **(x_k)**.

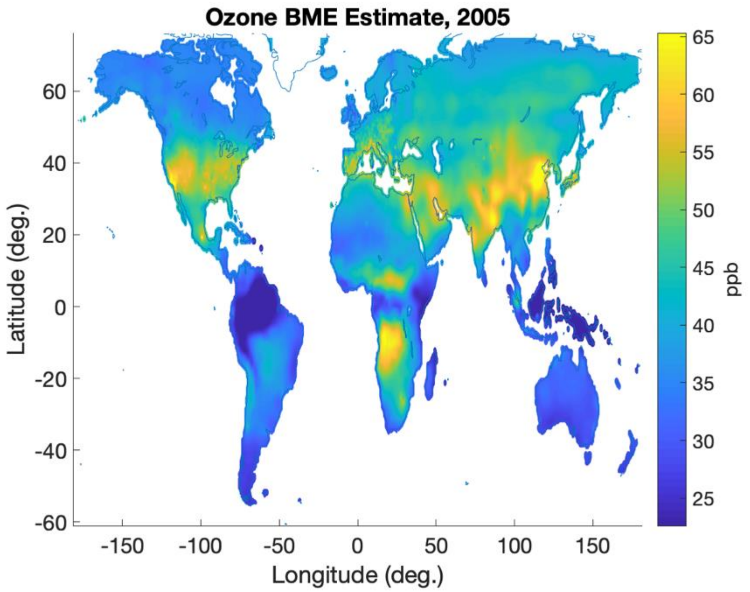

3) Adding fine resolution

Our results were calculated at 0.5° resolution, so to downscale estimates at finer resolution, we used the NASA G5NR-Chem model. The NASA G5NR-Chem model simulates surface ozone concentrations at 0.125° by 0.125° resolution for July 2013 to June 2014 (Hu et al., 2018). We regridded the G5NR-Chem model from 0.125° resolution to 0.1° resolution. While we do not expect that the raw values for 2013–2014 hold true for every year, we believe that the spatial distribution of this model can be used to inform the fine-scale spatial pattern for each year. To add fine resolution, we performed the following steps:

1. Regrid NASA G5NR-Chem from 0.125° resolution to 0.1° resolution
2. Average each 0.5° NASA G5NR-Chem grid cell
3. Calculate the difference between our BME estimation results at 0.5° and the average NASA G5NR-Chem at 0.5°
4. Add the calculated difference to NASA G5NR-Chem at 0.1° to obtain our BME estimation at 0.1°

Adding fine resolution to our results keeps the average of each 0.5° grid cell the same as the original estimation at 0.5°, as well as the global average.

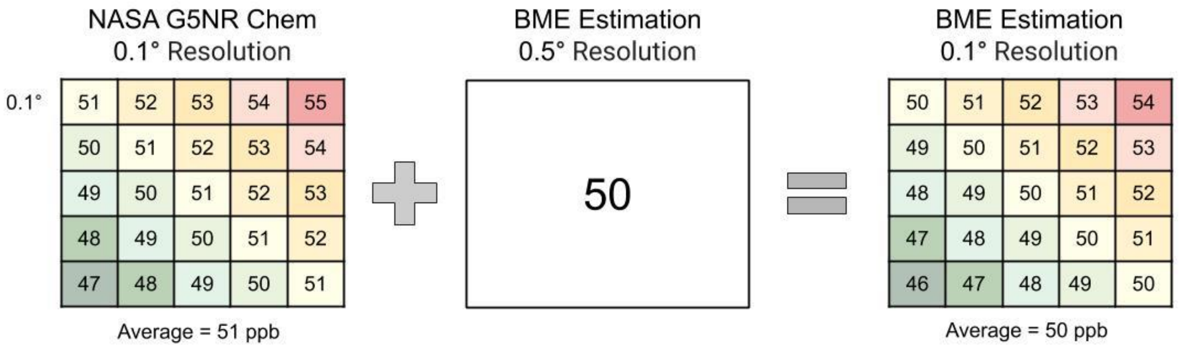

4) Final output

Three years are shown as an example below: 1995, 2005, and 2015. For each year, there are five maps displayed: the observations, M^3Fusion Model, BME Estimate, the difference between the BME Estimate and the M^3Fusion Model, and the variance. The difference map shows that the BME method corrects the M^3Fusion Model near monitoring stations, including stations in other years.

1995

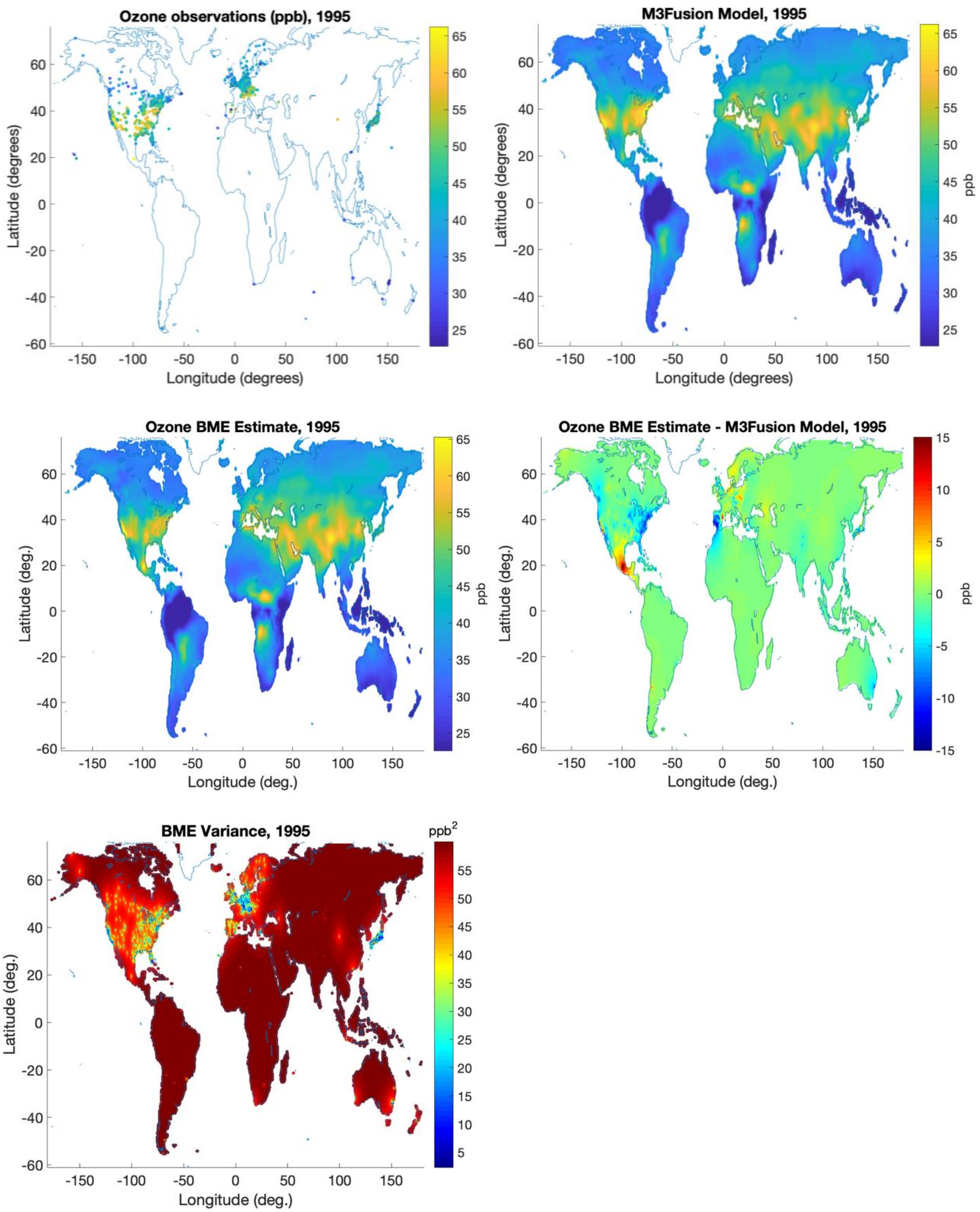
Ozone observations (ppb), 1995
Latitude (degrees)
Longitude (degrees)
M3Fusion Model, 1995
Latitude (degrees)
Longitude (degrees)
ppb
Ozone BME Estimate, 1995
Latitude (deg.)
Longitude (deg.)
ppb
Ozone BME Estimate - M3Fusion Model, 1995
Latitude (deg.)
Longitude (deg.)
ppb
BME Variance, 1995
Latitude (deg.)
Longitude (deg.)
ppb^2

2005

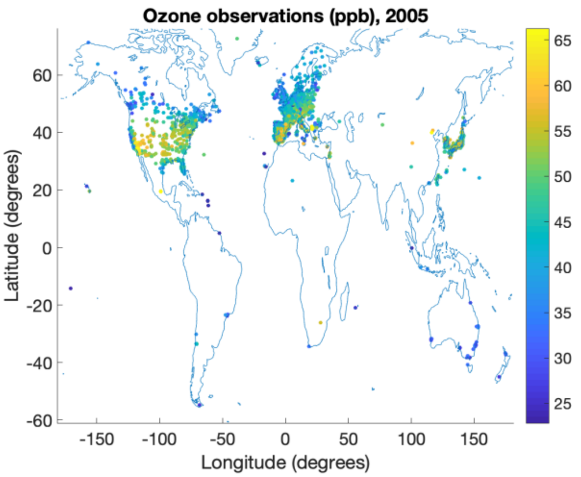
Ozone observations (ppb), 2005
Latitude (degrees)
Longitude (degrees)

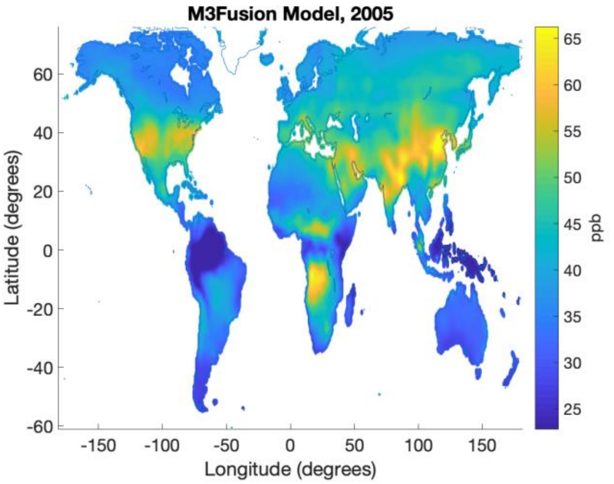
M3Fusion Model, 2005
Latitude (degrees)
Longitude (degrees)
ppb

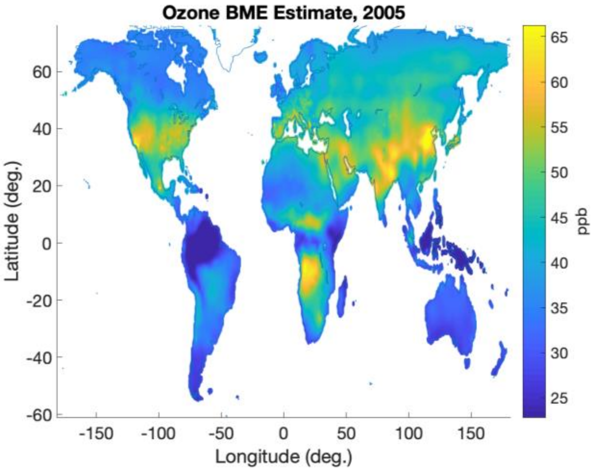
Ozone BME Estimate, 2005
Latitude (deg.)
Longitude (deg.)
ppb

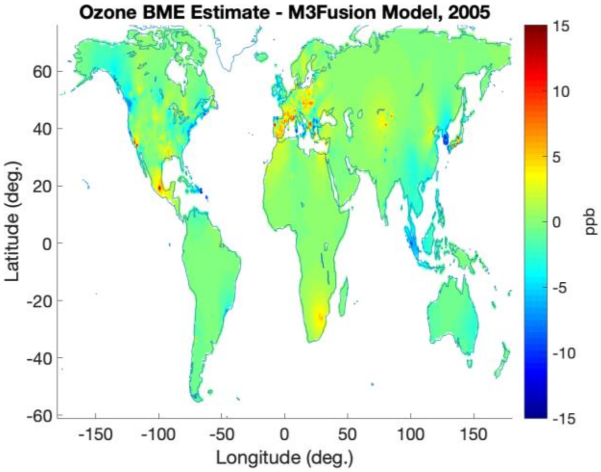
Ozone BME Estimate - M3Fusion Model, 2005
Latitude (deg.)
Longitude (deg.)
ppb

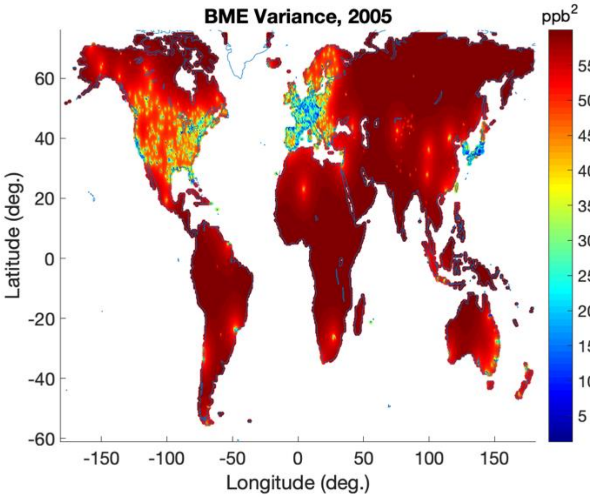
BME Variance, 2005
ppb^2
Latitude (deg.)
Longitude (deg.)

2015

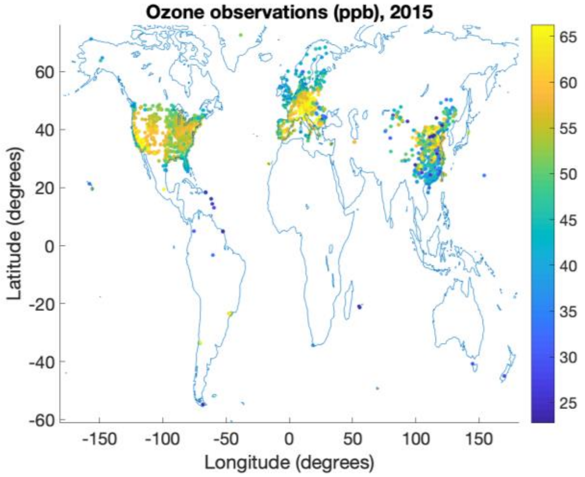
Ozone observations (ppb), 2015
Latitude (degrees)
Longitude (degrees)

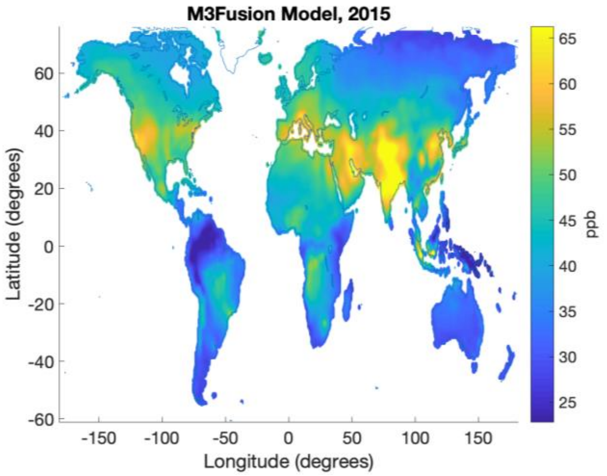
M3Fusion Model, 2015
Latitude (degrees)
Longitude (degrees)
ppb

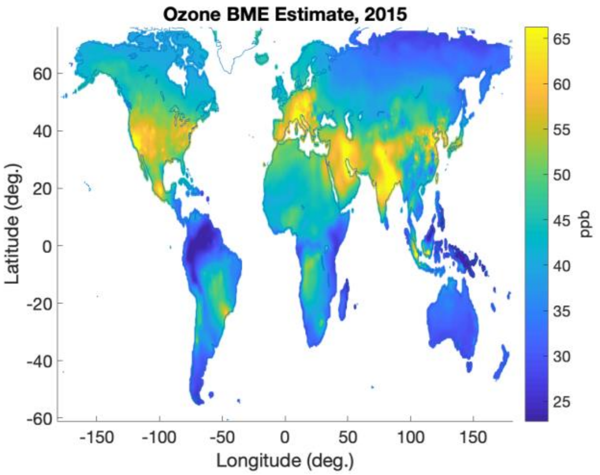
Ozone BME Estimate, 2015
Latitude (deg.)
Longitude (deg.)
ppb

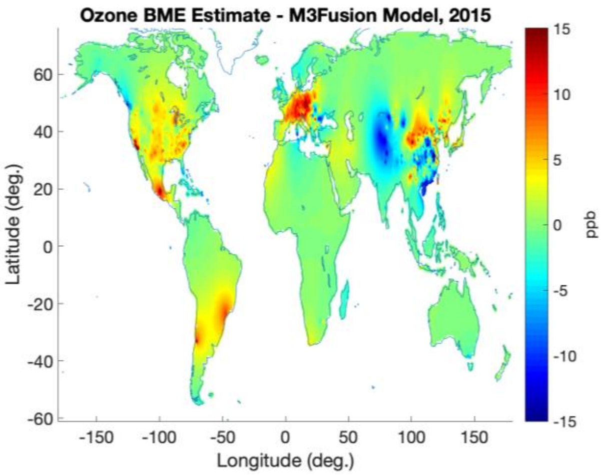
Ozone BME Estimate - M3Fusion Model, 2015
Latitude (deg.)
Longitude (deg.)
ppb

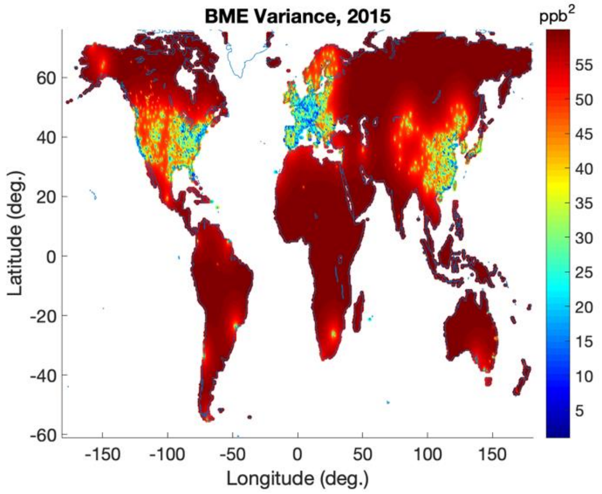
BME Variance, 2015
ppb2
Latitude (deg.)
Longitude (deg.)

5) Extrapolation and annual means

To estimate global ozone exposures in 2018, 2019, and 2020 for each 0.1° grid cell, we ran a log-linear model of the ozone estimates on year for the most recent ten years (2008–2017) of the following form:

$$\log(\text{ozone}) \sim \text{year}+1.$$

We considered using splines, but due to annual variation of ozone, we found this to be the most reasonable prediction.

For burden estimation, we are more interested in long-term trends and effects than annual variation; therefore, for the years 1991–2016, we used a three-year mean of exposure centered on the year of interest. This strategy aligns with the methodology used in ambient air pollution. For 1990 and 2017, we used two-year means (1990/1991 and 2016/2017, respectively) because 1989 and 2018 were not available in the estimates.

To estimate the variance for the three-year mean to generate confidence intervals, we did not have information on the covariance between years, so a conservative estimate of the variance was made:

Let *X*, *Y*, and *Z*, be random variables describing ozone exposure in a given 0.1-degree grid cell for years *i-1*, *i,* and *i+1*, respectively. By the laws of variance,

$$Var\left(\frac{1}{3}(X+Y+Z)\right)=\frac{1}{9}\big(Var(X)+Var(Y)+Var(Z)+2Cov(X,Y)+2Cov(X,Z)+2Cov(Y,Z)\big).$$

We do not know the covariance, but by the Cauchy-Schwartz inequality,

$$Cov(A,B)\leq\sqrt{Var(A)*Var(B)}.$$

Therefore,

$$Var\left(\frac{1}{3}(X+Y+Z)\right)\leq\frac{1}{9}\Big(Var(X)+Var(Y)+Var(Z)+2\sqrt{Var(X)*Var(Y)}+2\sqrt{Var(X)*Var(Z)}+2\sqrt{Var(Y)*Var(Z)}\Big).$$

This is a conservative estimate of the variance used when taking a three-year mean.

6) Difference from previous estimations

This method, implemented in the Global Burden of Disease (GBD) Study 2019 and unchanged for GBD 2021, improves upon the GBD 2017 ozone exposure estimates (Chang et al, 2019) in the following ways:

1. The previous estimates used observations in a certain year to correct the model within 2° of a monitoring station. In the current method, the radius of influence of each observation is defined by the spatial covariance. The spatial covariance shows that much of the influence of an observation is lost after 1°.
2. Measurements not only bias-correct the model in the year in which they were observed, but also influence other years according to the temporal covariance. This is important for regions that were not monitored over the entire 1990–2017 period.
3. The fine spatial structure of the final product represents the spatial distribution of the 0.125° NASA G5NR-Chem model.

Theoretical minimum-risk exposure level

As in GBD 2019, the TMREL is based on the exposure distribution from the ACS CPS-II study (Turner et al., 2016). It is a uniform distribution around the minimum and 5th percentile values observed in the cohort, ~U(29.1, 35.7), in ppb.

Relative risks

COPD is the only included outcome for ambient ozone pollution.

In GBD 2017, we performed a literature review of studies examining long-term ozone exposure and COPD. We included five cohorts from Canada, the UK, and the USA, all of which reported ozone effects on COPD mortality (Turner et al., 2016; Carey et al., 2013, and Burnett RT. "Cox…").

Table 2: Data inputs for relative risks for ambient ozone pollution

Input data	Relative risk
Site-years (total)	5
Number of countries with data	3
Number of GBD regions with data (out of 21 regions)	2
Number of GBD super-regions with data (out of seven super-regions)	1

As in GBD 2019, we used the meta-regression—Bayesian, regularised, trimmed (MR-BRT) meta-regression tool to conduct a meta-analysis on these five observations. For GBD 2021, there were several key updates to the meta-regression process. First, we implemented automated covariate selection to detect significant covariates from those extracted (see table below) to quantify between-study heterogeneity. The MR-BRT automated covariate selection tool implements a two-step process. First, a series of loosening Lasso penalty parameters are applied to a log-linear meta-regression on all input effect size observations. Then, covariates with a non-zero coefficient are tested for significance using a Gaussian prior (significance threshold = 0.05). No significant covariates were detected for ozone.

Table 3: Extracted covariates for ambient ozone pollution and COPD risk literature

Covariate name	Covariate description
cv_subpopulation	Study represents the general population; study represents a subgroup (eg, high-risk group)
cv_exposure_population	Study measures individual-level exposure (≤1 km radius); study measures population-level exposure
cv_exposure_selfreport	Exposure is self-reported; exposure is measured externally
cv_exposure_study	Exposure is measured multiple times; exposure is measured only at baseline
cv_outcome_selfreport	Outcome is self-reported; outcomes is based on death certificate or medical record
cv_outcome_unblinded	Study implements unblinded assessment; assessment of outcome is blind to exposure (and vice versa)
cv_reverse_causation	Study presents no risk of reverse causation; risk of reverse causation

cv_confounding_nonrandom	Non-randomised study; randomised study
cv_confounding_uncontrolled	Study is randomised/outcome controlled for age, sex, education, income, and all critical determinants of outcome; study is controlled for age, sex, and other critical determinants of outcome; study is controlled for only age and sex
cv_selection_bias	Study reports >95% follow-up; study reports 85–95% follow-up; study reports <85% follow-up

The standard error of observations based on a single cohort that reported an unstratified sample size were multiplied by the square root of n, where n is the total number of observations for a given cohort. This adjustment was made to prevent a single cohort or study from having an outsize weight on the summary effect size. Additionally, we trimmed one of the input observations (Carey et al., 2013) during model fitting in accordance with GBD protocol across risk factor teams to trim 10% of input data.

We generated 1000 predictions of the effect size for use in calculating burden estimates. These predictions were created using predictions of between-study heterogeneity. We implemented the Fisher scoring correction to the heterogeneity parameter, which corrects for data-sparse situations. In such cases, the between-study heterogeneity parameter estimate may be 0, simply from lack of data. The Fisher scoring correction uses a quantile of gamma, which is sensitive to the number of studies, study design, and reported uncertainty.

The inverse-standard error weighted meta-analysis provided an estimated relative risk of 1.074 (95% CI 1.014–1.137) per 10 ppb with an estimated gamma (including between-study heterogeneity) of 0.

Table 4: MR-BRT relative risk model parameters for ambient ozone pollution

Covariate	Gamma (95% CI)	Beta coefficient, log (95% UI)	Exponentiated coefficient (95% UI)
Exposure (per 1 ppb)	0 (0–0)	7.133e-3 (4.488e-3 to 9.925e-3)	1.007 (1.004–1.010)

Ambient ozone pollution and COPD risk literature funnel plot

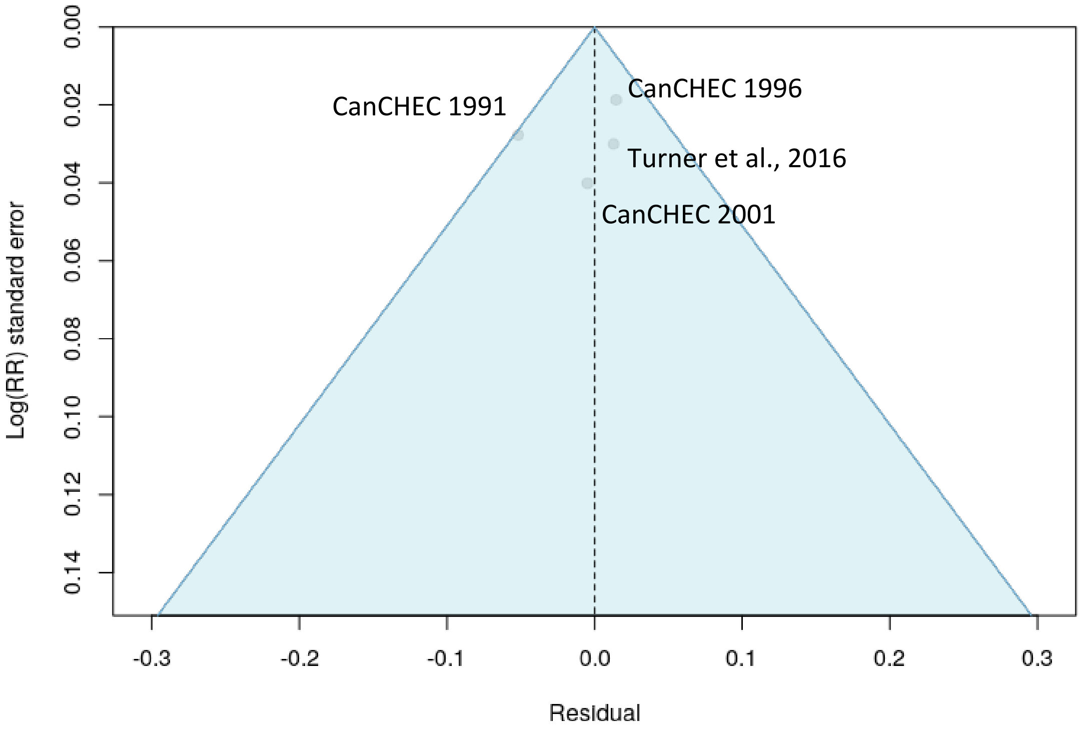

Risk-outcome scoring

For GBD 2021, we also implemented risk-outcome scoring. Risk-outcome scores provide an empirical measure of the strength of evidence for risk-outcome pairs across risk factors in the GBD and are therefore useful for standardised comparison. Risk-outcome scores evaluate the area between the lower bound of the 95% uncertainty interval and the x-axis for harmful risk factors, including ambient ozone pollution. Prior to generating a risk-outcome score, we conducted an additional post-analysis step to detect and flag publication bias in the input data. This approach is based on the classic Egger's regression strategy, which is applied to the residuals in our model. In the current implementation, we do not correct for publication bias, but flag the risk-outcome pairs where the risk for publication bias is significant. Publication bias was not detected for ambient ozone pollution and COPD risk literature.

To calculate the risk-outcome score, we generated an uncertainty interval from 1000 draws of the adjusted summary effect size (retaining uncertainty information from between-study heterogeneity predictions and the Fisher information boost). We then evaluated the risk-outcome score between the 15th and 85th percentiles of the input data exposure distribution (0–10 ppb). The final risk-outcome score is 0.011, which corresponds to a star rating of 2.

We calculated PAFs at the grid-cell level and aggregated up to GBD locations using population data from the Gridded Population of the World database. Estimates came from version 4 except for estimates for 1990 and 1995 from version 3. More details on these estimates are available in the Ambient Particulate Matter Pollution Methods Appendix.

Citations

1. DeLang MN, Becker JS, Chang KL, Serre ML, Cooper OR, Schultz MG, Schröder S, Lu X, Zhang L, Deushi M, Josse B, Keller CA, Lamarque JF, Lin M, Liu J, Marécal V, Strode AS, Sudo K, Tilmes S, Zhang L, Cleland SE, Collins EL, Brauer M, West JJ. Mapping Yearly Fine Resolution Global Surface Ozone through the Bayesian Maximum Entropy Data Fusion of Observations and Model Output for 1990–2017. Environ Sci Technol. 2021; 55 (8): 4389-4398. DOI: 10.1021/acs.est.0c07742.
2. Schultz MG, Schröder S, Lyapina O, Cooper O, Galbally I, Petropavlovskikh I, et al. Tropospheric Ozone Assessment Report: Database and Metrics Data of Global Surface Ozone Observations. Elem Sci Anth. 2017;5:58. DOI: http://doi.org/10.1525/elementa.244.
3. Lu, Xiao, Jiayun Hong, Lin Zhang, Owen R. Cooper, Martin G. Schultz, Xiaobin Xu, Tao Wang, Meng Gao, Yuanhong Zhao, and Yuanhang Zhang. Severe surface ozone pollution in China: a global perspective. Environmental Science & Technology Letters 2018 *5* (8), 487-49 DOI: 10.1021/acs.estlett.8b00366.
4. Chang, Kai-Lan, R. Cooper, Owen, West, Jason, L. Serre, Marc, G. Schultz, Martin, Lin, Meiyun, Marecal, Virginie, Josse, B, Deushi, Makoto, Sudo, Kengo, Liu, Junhua & A. Keller, Christoph. (2019). A new method (M^3Fusion v1) for combining observations and multiple model output for an improved estimate of the global surface ozone distribution. Geoscientific Model Development. 12. 955-978. 10.5194/gmd-12-955-2019.
5. Hu, L., C. A. Keller, M. S. Long, T. Sherwen, B. Auer, A. Da Silva, J. E. Nielsen, S. Pawson, M. A. Thompson, A. L. Trayanov, K. R. Travis, S. K. Grange, M. J. Evans, D. J. Jacob (2018) Global simulation of tropospheric chemistry at 12.5 km resolution: performance and evaluation of the GEOS-Chem chemical module (v10-1) within the NASA GEOS Earth system model (GEOS-5 ESM), Geoscientific Model Development, 11, 4603-4620. 10.5194/gmd-11-4603-2018.
6. Turner MC,Jerrett M, Pope CA 3rd, Krewski D, Gapstur SM, Diver WR, Beckerman BS, Marshall JD, Su J, Crouse DL, Burnett RT. Long-term ozone exposure and mortality in a large prospective study. *Am J Respir Crit Care Med*. 2016; 193(10): 1134-42.
7. Carey IM, Atkinson RW, Kent AJ, van Staa T, Cook DG, Anderson HR. Mortality associations with long-term exposure to outdoor air pollution in a national English cohort. *Am J Respir Crit Care Med.* 2013; 187(11): 1226-33.
8. Burnett RT. Cox Proportional Survival Model Hazard Ratios from Census Years (1991, 1996, 2001) to 2011 for Adults Aged 25 to 89 in CanCHEC Cohort. Custom Analysis for GBD 2017.

Citations for Atmospheric Chemical Transport Models

Ozone exposure information was provided by the following collaborators:

- Marissa DeLang, Jacob S. Becker, Stephanie Cleland, Elyssa Collins, Marc L. Serre, J. Jason West, University of North Carolina at Chapel Hill
- Owen R. Cooper and Kai-Lan Chang, CIRES, University of Colorado, Boulder/NOAA Earth System Research Laboratory, Boulder, USA
- Martin G. Schultz and Sabine Schröder, Jülich Supercomputing Centre (JSC), Forschungszentrum Jülich, Jülich, DE
- Xiao Lu and Lin Zhang, Laboratory for Climate and Ocean-Atmosphere Studies, Dept. of Atmospheric and Oceanic Sciences, School of Physics, Peking University, Beijing, China

- CCMI and NASA modellers

- CESM:
  - Tilmes, S., Lamarque, J.-F., Emmons, L. K., Kinnison, D. E., Ma, P.-L., Liu, X., Ghan, S., Bardeen, C., Arnold, S., Deeter, M., Vitt, F., Ryerson, T., Elkins, J. W., Moore, F., Spackman, J. R., and Val Martin, M.: Description and evaluation of tropospheric chemistry and aerosols in the Community Earth System Model (CESM1.2), Geosci. Model Dev., 8, 1395–1426, doi:10.5194/gmd-8-1395-2015, 2015
- CESM WACCM:
  - Garcia, R. R., Smith, A. K., Kinnison, D. E., de la Cámara, Á., and Murphy, D.: Modifications of the gravity wave parameterisation in the Whole Atmosphere Community Climate Model: Motivation and results, J. Geophys. Res.-Atmos., doi:10.1175/JAS-D16-0104.1, 2016.
  - Marsh, D., Mills, M. J., Kinnison, D. E., Garcia, R. R., Lamarque, J.-F., and Calvo, N.: Climate change from 1850–2005 simulated in CESM1 (WACCM), J. Climate, 26, 7372–7391, doi:10.1175/JCLI-D-12-00558.1, 2013
- CHASER:
  - Sudo, K., Takahashi, M., and Akimoto, H.: CHASER: A global chemical model of the troposphere, 2. Model results and evaluation, J. Geophys. Res., 107, 4586, https://doi.org/10.1029/2001JD001114, 2002a.
  - Sudo, K., Takahashi, M., Kurokawa, J., and Akimoto, H.: CHASER: A global chemical model of the troposphere, 1. Model description, J. Geophys. Res., 107, 4339, https://doi.org/10.1029/2001JD001113, 2002b.

  - Watanabe, S., Hajima, T., Sudo, K., Nagashima, T., Takemura, T., Okajima, H., Nozawa, T., Kawase, H., Abe, M., Yokohata, T., Ise, T., Sato, H., Kato, E., Takata, K., Emori, S., and Kawamiya, M.: MIROC-ESM 2010: model description and basic results of CMIP5-20c3m experiments, Geosci. Model Dev., 4, 845–872, https://doi.org/10.5194/gmd-4-845-2011, 2011.
- GEOSCCM:
  - Oman, L. D., Ziemke, J. R., Douglass, A. R., Waugh, D. W., Lang, C., Rodriguez, J. M., and Nielsen, J. E.: The response of tropical tropospheric ozone to ENSO, Geophys. Res. Lett., 38, L13706, https://doi.org/10.1029/2011GL047865, 2011.
- GFDL AM3 & AM4:
  - Lin, M., Fiore, A. M., Horowitz, L. W., Cooper, O. R., Naik, V., Holloway, J., Johnson, B. J., Middlebrook, A. M., Oltmans, S. J., Pollack, I. B., Ryerson, T. B., Warner, J. X., Wiedinmyer, C., Wilson, J., and Wyman, B.: Transport of Asian ozone pollution into surface air over the western United States in spring, J. Geophys. Res., 117, D00V07, https://doi.org/10.1029/2011JD016961, 2012.
  - Lin, M., Horowitz, L. W., Oltmans, S. J., Fiore, A. M., and Fan, S.: Tropospheric ozone trends at Mauna Loa Observatory tied to decadal climate variability, Nat. Geosci., 7, 136–143, https://doi.org/10.1038/NGEO2066, 2014.

- Lin, M., Horowitz, L. W., Payton, R., Fiore, A. M., and Tonnesen, G.: US surface ozone trends and extremes from 1980 to 2014: quantifying the roles of rising Asian emissions, domestic controls, wildfires, and climate, Atmos. Chem. Phys., 17, 2943–2970, https://doi.org/10.5194/acp-17-2943-2017, 2017.

- MERRA GMI:
  - Ziemke, J. R., Oman, L. D., Strode, S. A., Douglass, A. R., Olsen, M. A., McPeters, R. D., Bhartia, P. K., Froidevaux, L., Labow, G. J., Witte, J. C., Thompson, A. M., Haffner, D. P., Kramarova, N. A., Frith, S. M., Huang, L.-K., Jaross, G. R., Seftor, C. J., Deland, M. T., and Taylor, S. L.: Trends in global tropospheric ozone inferred from a composite record of TOMS/OMI/MLS/OMPS satellite measurements and the MERRA-2 GMI simulation , Atmos. Chem. Phys., 19, 3257–3269, https://doi.org/10.5194/acp-19-3257-2019, 2019.
- MOCAGE:
  - Josse, B., Simon, P., and Peuch, V.-H.: Radon global simulations with the multiscale chemistry and transport model MOCAGE, Tellus B, 56, 339–356, https://doi.org/10.1111/j.1600-0889.2004.00112.x, 2004.
  - Teyssèdre, H., Michou, M., Clark, H. L., Josse, B., Karcher, F., Olivié, D., Peuch, V.-H., Saint-Martin, D., Cariolle, D., Attié, J.-L., Nédélec, P., Ricaud, P., Thouret, V., van der A, R. J., Volz-Thomas, A., and Chéroux, F.: A new tropospheric and stratospheric Chemistry and Transport Model MOCAGE-Climat for multi-year studies: evaluation of the present-day climatology and sensitivity to surface processes, Atmos. Chem. Phys., 7, 5815–5860, https://doi.org/10.5194/acp-7-5815-2007, 2007.
- MRI-ESM1r1:
  - Adachi, Y., Yukimoto, S., Deushi, M., Obata, A., Taichu. Y. Tanaka, H. N., Hosaka, M., Sakami, T., Yoshimura, H., Hirabara, M., Shindo, E., Tsujino, H., Mizuta, R., Yabu, S., Koshiro, T., Ose, T., and Kitoh, A.: Basic performance of a new earth system model of the Meteorological Research Institute (MRI-ESM1), Pap. Meteorol. Geophys, 64, 1–18, https://doi.org/10.2467/mripapers.64.1, 2013.

Ambient particulate matter pollution

Flowchart

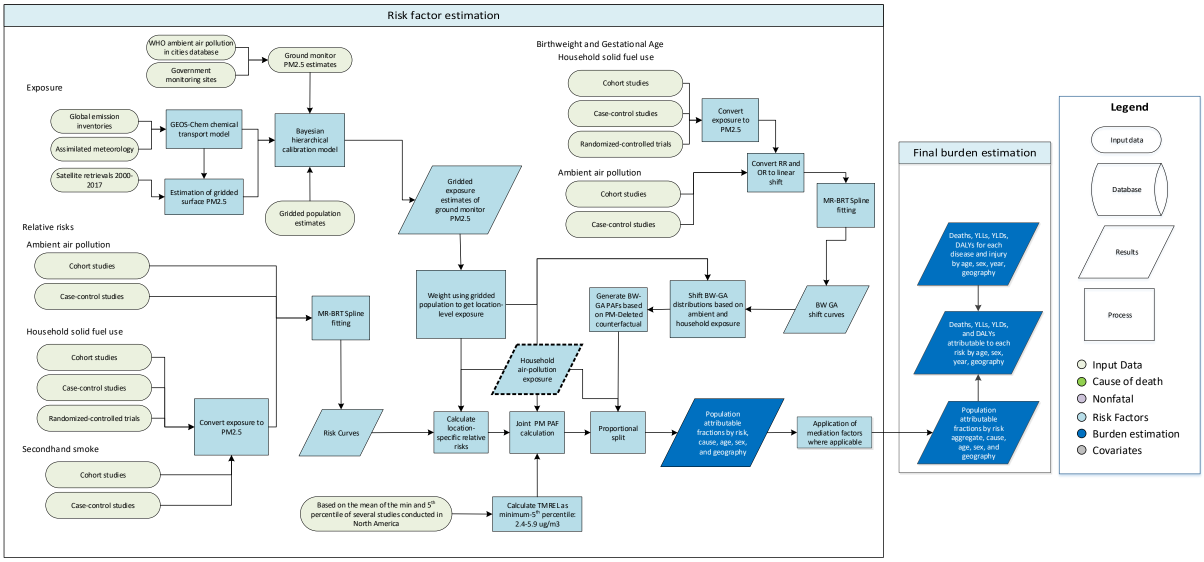

Input data and methodological summary

Exposure

Definition

Exposure to ambient particulate matter pollution is defined as the population-weighted annual average mass concentration of particles with an aerodynamic diameter less than 2.5 micrometers ($PM_{2.5}$) in a cubic meter of air. This measurement is reported in µg/m^3.

Input data

Ambient air pollution exposure estimates use input data from multiple sources. These include satellite observations of aerosols in the atmosphere, ground monitor measurements, chemical transport model simulations, population estimates, and land-use data.

Table 1: Data inputs for exposure for ambient particulate matter pollution

Input data	Exposure
Site-years (total)	5442
Number of countries with data	204
Number of GBD regions with data (out of 21 regions)	21
Number of GBD super-regions with data (out of 7 super-regions)	7

Details for updates in exposure methodology and input data for the Global Burden of Disease (GBD) Study 2021 are as follows.

$PM_{2.5}$ ground measurement database

For GBD 2021, ground monitor measurements were updated to include more recent measurements from sites included in GBD 2019 and additional measurements from new monitors. New data were added to the database from several sources, including the European Environment Agency, United States Environmental Protection Agency, and the OpenAQ database. The complete, updated dataset included measurements of PM_{10} and $PM_{2.5}$ concentrations between 2018 and 2020 from 18,406 ground monitors from 120 countries, primarily from the USA, China, European countries, and USA embassies and consulates. Annual averages were excluded if they were based on less than 75% coverage within a year unless there was already sufficient data within the country of interest (monitor density greater than 0.1). If information on coverage was not available, data were included.

For sites with PM_{10} measurements only, these observations were converted from PM_{10} to $PM_{2.5}$ measurements using a hierarchy of conversion factors ($PM_{2.5}/PM_{10}$ ratios): (i) where possible, a "local" conversion factor was used, constructed as the ratio of the average measurements (of $PM_{2.5}$ and PM_{10}) from within 50 km of the location of the PM_{10} measurement, and within the same country, if such measurements were available; (ii) where local information was not sufficient to construct a conversion factor, a country-wide conversion factor was used; and (ii) where appropriate information within a country did not exist, a region-level factor was used. In each case, to avoid the possible effects of outliers in the measured $PM_{2.5}$ and PM_{10} data, extreme values of the ratios were excluded. These extreme values were defined as those greater/lesser than the 95th and 5th quantiles of the empirical distributions of conversion factors. As with the GBD 2013, 2015, 2016, 2017, and 2019 databases, in addition to values of $PM_{2.5}$ and whether they were direct measurements or conversions from PM_{10}, the GBD 2021 database also included additional information (where available) concerning the ground measurements, such as monitor geo-coordinates and monitor site type.

Satellite-based estimates

Global satellite-derived estimates (V4.GL.03.NoGWR) used as inputs to DIMAQ2 for 1998–2019 and for January to August 2020 are used at 0.1^{o} x 0.1^{o} resolution (~11 x 11 km resolution at the equator) and follow the methodology described in Hammer et al., 2020.[1] The algorithm uses aerosol optical depth (AOD) from several updated satellite products (MAIAC, MODIS, and MISR). Ground-based observations from a global sunphotometer network (AERONET version 3) are used to combine different AOD information sources. The GEOS-Chem chemical transport model was used for geophysical relationships between surface $PM_{2.5}$ and AOD. For GBD 2021, an additional update to biomass burning emissions from 2015 to 2020 was made. This update allows for time-varying biomass burning emissions in the simulation for those years, where they had previously been unavailable after 2014. Given lags in releases of available meteorological information used in the GEOS Chem simulations, for September to December 2020, the estimates incorporate satellite retrievals from 2020, but GEOS-Chem simulated values for 2019 as well as biomass burning emissions from 2019. Further, satellite retrievals for all of 2020 were limited to MODIS DT, DB, and MAIAC. We included MISR inputs for January to June 2020 only, as this product was not available past June when the satellite-based estimates were generated.

Chemical transport model simulations

Estimates of the sum of particulate sulfate, nitrate, ammonium, and organic carbon and the compositional concentrations of mineral dust simulated using the GEOS-Chem chemical transport

model, and a measure combining elevation and the distance to the nearest urban land surface (as described in van Donkelaar et al. 2016[2] and Hammer et al. 2020)[1] were available for 2000–2020 for each $0.1^{o} \times 0.1^{o}$ grid cell.

Population data

We obtained a comprehensive, high-resolution gridded population dataset from the Gridded Population of the World (GPW) database. Estimates for 2000, 2005, 2010, 2015, and 2020 were available from the GPW version 4, with estimates for 1990 and 1995 obtained from the GPW version 3. These data are provided on a $0.0083^{o} \times 0.0083^{o}$ resolution. Aggregation to each $0.1^{o} \times 0.1^{o}$ grid cell was accomplished by summing the central 12 × 12 population cells. Populations estimates for 2001–2004, 2006–2009, 2011–2014, and 2016–2019 were obtained by interpolation using natural splines with knots placed at 2000, 2005, 2010, 2015, and 2020. This was performed for each grid cell.

Modelling strategy

The following is a summary of the modelling approach, known as the Data Integration Model for Air Quality (DIMAQ) used in GBD 2015, 2016, 2017, 2019, and 2020. [3,4]

Before the implementation of DIMAQ in GBD 2010 and 2013, exposure estimates were obtained using a single global function to calibrate available ground measurements to a “fused” estimate of $PM_{2.5}$: the mean of satellite-based estimates and those from the TM5 chemical transport model, calculated for each $0.1^{o} \times 0.1^{o}$ grid cell. This approach was recognised to represent a trade-off between accuracy and computational efficiency when utilising all the available data sources. In particular, the GBD 2013 exposure estimates were known to underestimate ground measurements in specific locations (see discussion in Brauer et al., 2015).[5] This underestimation was largely due to the use of a single, global calibration function, whereas in reality, the relationship between ground measurements and other variables varies spatially.

In GBD 2015 and 2016, coefficients in the calibration model were estimated for each country through DIMAQ. Where data were insufficient within a country, information was “borrowed” from a region-level aggregation, and where information was still insufficient, from the super-region-level aggregation. Individual country-level estimates were therefore based on a combination of information from the country and its region and super-region. This was implemented within a Bayesian hierarchical modelling (BHM) framework. BHMs provide an extremely useful and flexible framework in which to model complex relationships and dependencies in data. Uncertainty can also be propagated through the model, allowing uncertainty arising from different components (both data sources and models) to be incorporated within estimates of uncertainty associated with the final estimates. The results of the modelling comprise a posterior distribution for each grid cell, rather than just a single point estimate, allowing a variety of summaries to be calculated. The primary outputs for this process are the median and 95% uncertainty intervals for each grid cell. Based on the availability of ground measurement data, modelling and evaluation were focused on the year 2016.

The model used from GBD 2017 onward (GBD 2017, 2019, and now 2021) also included within-country calibration variation.[6] This model, henceforth referred to as DIMAQ2, provides a number of substantial improvements over the initial formulation of DIMAQ. In DIMAQ, ground measurements from different years were all assumed to have been made in the primary year of interest and then regressed against values from other inputs (satellites, etc.) made in that year. In the presence of changes over time,

therefore, and particularly in areas where no recent measurements were available, there was the possibility of mismatches between the ground measurements and other variables. In DIMAQ2, ground measurements are matched with other inputs (over time), and the (global-level) coefficients are allowed to vary over time, subject to smoothing that is induced by a first-order random walk process. In addition, the manner in which spatial variation can be incorporated within the model has developed: where there are sufficient data, the calibration equations can now vary (smoothly) both within and between countries, achieved by allowing the coefficients to follow (smooth) Gaussian processes. Where there are insufficient data within a country, to produce accurate equations, information is borrowed as before from lower down the hierarchy and is supplemented with information from the wider region.

DIMAQ2 as described above was used for all regions except for the north Africa/Middle East and sub-Saharan Africa super-regions, where there are insufficient data across years to allow the extra complexities of the new model to be implemented. In these super-regions, a simplified version of DIMAQ2 is used in which the temporal component is dropped.

Inference and prediction

Continuous explanatory variables:

- (SAT) Estimate of $PM_{2.5}$ (in $\mu g/m^3$) from satellite remote sensing on the log-scale.
- (POP) Estimate of population for the same year as SAT on the log-scale.
- (SANOC) Estimate of the sum of sulfate, nitrate, ammonium, and organic carbon simulated using the GEOS-Chem chemical transport model.
- (DST) Estimate of compositional concentrations of mineral dust simulated using the GEOS-Chem chemical transport model.
- (EDxDU) The log of the elevation difference between the elevation at the ground measurement location and the mean elevation within the GEOS-Chem simulation grid cell multiplied by the inverse distance to the nearest urban land surface.

Discrete explanatory variables:

- (LOC) Binary variable indicating whether exact location of ground measurement is known.
- (TYPE) Binary variable indicating whether exact type of ground monitor is known.
- (CONV) Binary variable indicating whether ground measurement is $PM_{2.5}$ or converted from PM_{10}.

Interactions:

- Interactions between the binary variables and the effects of SAT.

Random effects:

- Regional temporal (random walk) hierarchical random-effects on the intercept
- Regional hierarchical random-effects for the coefficient associated with SAT
- Regional hierarchical random-effects for the coefficient associated with POP
- Smoothed, spatially varying, random-effects for the intercept
- Smoothed, spatially varying, random-effects for the coefficient associated with SAT

Due to both the complexity of the models and the size of the data, notably the number of spatial predictions that are required, recently developed techniques that perform “approximate” Bayesian inference based on integrated nested Laplace approximations (INLA) were used.[7] Computation was performed using the R interface to the INLA computational engine (R-INLA). For GBD 2019 and GBD 2021, the model also implements an innovative way to use samples from the (Bayesian) model to represent distributions of estimated concentrations in each grid cell. Estimates, and distributions representing uncertainty, of concentrations for each grid cell are obtained by taking repeated (joint) samples from the posterior distributions of the parameters and calculating estimates based on a linear combination of those samples and the input variables.[8]

DIMAQ2 was used to produce grid-cell-level ($0.1^{o} \times 0.1^{o}$) estimates of ambient $PM_{2.5}$ for 1990, 1995, and 2010–2020 by matching the gridded estimates with the corresponding coefficients from the calibration. For the year 2020, additional analysis was conducted to incorporate updated ground monitor (1777 observations for 2020) and satellite-based data (as described above) to examine potential impacts of the COVID-19 pandemic on ambient particulate matter pollution.

Model evaluation

Model development and comparison was performed using within- and out-of-sample assessment. For evaluation, cross-validation was performed using 25 combinations of training (80%) and validation (20%) datasets. Validation sets were obtained by taking a stratified random sample, using sampling probabilities based on the cross-tabulation of $PM_{2.5}$ categories (0–24.9, 25–49.9, 50–74.9, 75–99.9, 100+ $\mu g/m^3$) and super-regions, resulting in sets with the same distribution of $PM_{2.5}$ concentrations and super-regions as the overall set of sites. The following metrics were calculated for each training/validation set combination: for model fit—R^2; for predictive accuracy—root mean squared error (RMSE) and population-weighted root mean squared error (PwRMSE).

Evaluation of model results for GBD 2021 were comparable to those from GBD 2013 and GBD 2017 (the most recent model evaluation prior to GBD 2021). For GBD 2021, DIMAQ2 predictions of ground measurements in all super-regions produced a mean out-of-sample population-weighted RMSE of 8.50 (95% UI 6.17–12.77) $\mu g/m^3$ and an R^2 of 0.909 (0.886–0.926). The high-income super-region produced the most accurate predictions, with a mean population-weighted RMSE of 2.16 (2.09–2.23) $\mu g/m^3$, while south Asia produced the largest population-weighted mean RMSE, 31.56 (18.95– 51.88) $\mu g/m^3$. Trends in relative magnitude of PwRMSE are consistent with previous DIMAQ evaluations in GBD 2017 and 2019.

Figure 1: Summary measure of predictive ability, globally and by super-region. Points denote median values of out-of-sample population-weighted root mean square error ($\mu g/m^3$) from 25 validation sets. Vertical lines denote 95% uncertainty interval bounds.

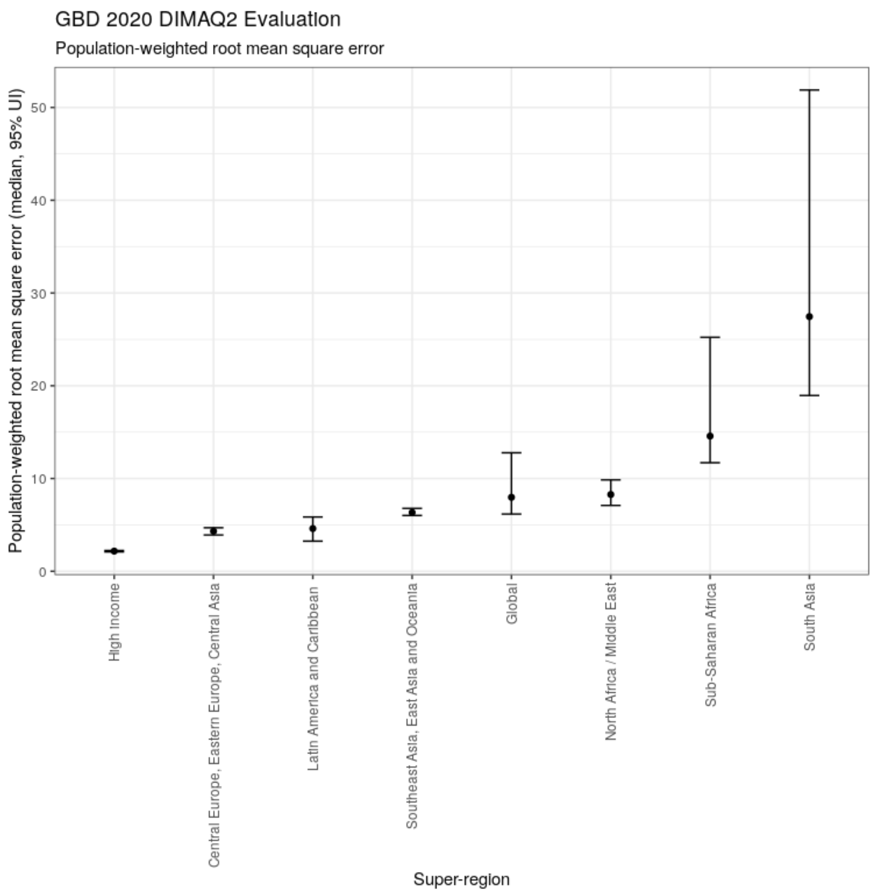

Table 2: Summary measure of predictive ability, globally and by super-region. Values denote median, lower, and upper 95% uncertainty interval bounds of out-of-sample population-weighted relative error (root mean square error/mean $PM_{2.5}$ prediction reported in μg/m^3) from 25 validation sets.

Location	Median	Lower	Upper
Global	0.115	0.105	0.133
Central Europe, eastern Europe, central Asia	0.189	0.180	0.199
High income	0.151	0.147	0.155
Latin America and Caribbean	0.234	0.179	0.313
North Africa and Middle East	0.243	0.217	0.263
South Asia	0.452	0.349	0.616
Southeast Asia, east Asia, and Oceania	0.174	0.169	0.184
Sub-Saharan Africa	0.322	0.256	0.409

Theoretical minimum-risk exposure level

The theoretical minimum-risk exposure level (TMREL) was assigned a uniform distribution with lower/upper bounds given by the average of the minimum and 5th percentiles of outdoor air pollution cohort studies exposure distributions conducted in North America, with the assumption that current evidence was insufficient to precisely characterise the shape of the concentration-response function below the 5th percentile of the exposure distributions. The TMREL was defined as a uniform distribution rather than a fixed value in order to represent the uncertainty regarding the level at which the scientific evidence was consistent with adverse effects of exposure. The specific outdoor air pollution cohort studies selected for this averaging were based on the criteria that their 5th percentiles were less than that of the American Cancer Society Cancer Prevention II (CPSII) cohort's 5th percentile of 8.2 based on Turner et al. (2016).[9] This criterion was selected because GBD 2010 used the minimum, 5.8, and 5th percentile solely from the CPS II cohort. The resulting lower/upper bounds of the distribution for GBD 2021 were 2.4 and 5.9. This has not changed since GBD 2015.

Relative risks and population attributable fractions

Input data

For GBD 2021, as in previous GBD cycles, we created one set of cause-specific risk curves for both household air pollution and ambient particulate matter pollution as two different sources of $PM_{2.5}$. In GBD 2017, we estimated the particulate-matter-attributable burden of disease based on the relation of long-term exposure to $PM_{2.5}$ with ischaemic heart disease, stroke (ischaemic and haemorrhagic), COPD, lung cancer, acute lower respiratory infection, and type 2 diabetes. In GBD 2019, we added adverse birth outcomes including low birthweight and short gestation as contributors to $PM_{2.5}$-attributable burden. Because these are risk factors (not outcomes) included in the GBD study, we performed a mediation analysis, in which a proportion of the burden attributable to low birthweight and short gestation is attributed to $PM_{2.5}$ pollution. For GBD 2021, as in previous cycles, we used risk curves to calculate burden for ages 25+ for ischaemic heart disease, stroke (ischaemic and haemorrhagic), COPD, lung cancer, and type 2 diabetes and for all ages for acute lower respiratory infection. Burden calculation for mediated outcomes is described below.

For the six non-mediated outcomes, we used results from cohort and case-control studies of ambient $PM_{2.5}$ pollution and cohort studies, case-control studies, and randomised-controlled trials of household use of solid fuel for cooking. For GBD 2021, we excluded secondhand smoke cohort and case-control studies from risk curve input data.

We conducted a literature review for studies of $PM_{2.5}$ (ambient and household air pollution) and risk of lower respiratory infection using the search string below. We searched the PubMed database for studies published between January 1, 2017, and July 22, 2020 (date of search). 32 initial results were obtained from the database, 31 of which were excluded during title-abstract and full-text screening. The remaining study was later excluded due to insufficient information reported on the study-specific exposure distribution.

Search string: ((("Air Pollution"[Mesh] OR "Particulate Matter"[Mesh] OR "air pollution"[Title/Abstract] OR "urban air pollution"[Title/Abstract] OR "ambient air pollution"[Title/Abstract] OR "airborne particulate matter"[Title/Abstract]) OR ("Air Pollution, Indoor"[Mesh] OR "Household air"[Title/Abstract] OR "Indoor air pollution"[Title/Abstract] OR "Indoor fine particulate matter"[Title/Abstract] OR "Indoor particulate matter"[Title/Abstract] OR "Indoor air quality"[Title/Abstract])) AND ("lower respiratory infection"[Title/Abstract] OR "LRI"[Title/Abstract]))

Table 3: Data inputs for relative risks for ambient particulate matter pollution

Input data	Relative risk
Site-years (total)	196
Number of countries with data	53
Number of GBD regions with data (out of 21 regions)	18
Number of GBD super-regions with data (out of 7 super-regions)	7

For GBD 2021, as in GBD 2019, the meta-regression—Bayesian, regularised, trimmed (MR-BRT) meta-regression tool was used to create relative risk estimates, with three key updates to input data. In GBD 2017, we used relative estimates for active smoking and secondhand smoke (converting cigarettes per day to $PM_{2.5}$ exposure) to estimate relative risk predictions for $PM_{2.5}$ exposure at the highest end of the exposure–response curve. These data were included because the majority of the air pollution epidemiological studies have been performed in high-income countries which have lower levels of ambient $PM_{2.5}$ pollution. This posed a barrier to extrapolating relative risk estimates from the steep relationship at the beginning of the exposure range to locations with high exposures but no relative risk estimates, such as India and China. In GBD 2019, we incorporated estimates at high $PM_{2.5}$ levels by adding recently published ambient $PM_{2.5}$ studies conducted in China and other higher-exposure settings and additional HAP studies.[10,11,12,13,14] Additionally, the switch to MR-BRT splines in GBD 2019 (instead of the integrated exposure–response function employed in GBD 2017) presented a more flexible approach that allowed the curve to fit ambient and household data and removed the need for active smoking data to anchor the curve at higher exposures. The inclusion of active smoking and secondhand smoking data in previous GBD cycles required conversion from cigarettes per day to $PM_{2.5}$ exposure and introduced other differences, including differences in dose rates and those between voluntary (active smoking) and involuntary (ambient $PM_{2.5}$, household air pollution, secondhand smoke) exposures. Due to these factors, in GBD 2019, we removed active smoking data from the relative risk model's input data. In GBD 2021, we also removed secondhand smoking data, completing the transition to only using $PM_{2.5}$ and HAP relative risk input data. This removes important sources of uncertainty in our earlier estimates.[15,16] The following plot displays $PM_{2.5}$ risk curves from GBD 2019 and from GBD 2021, with and without secondhand smoking RR input data:

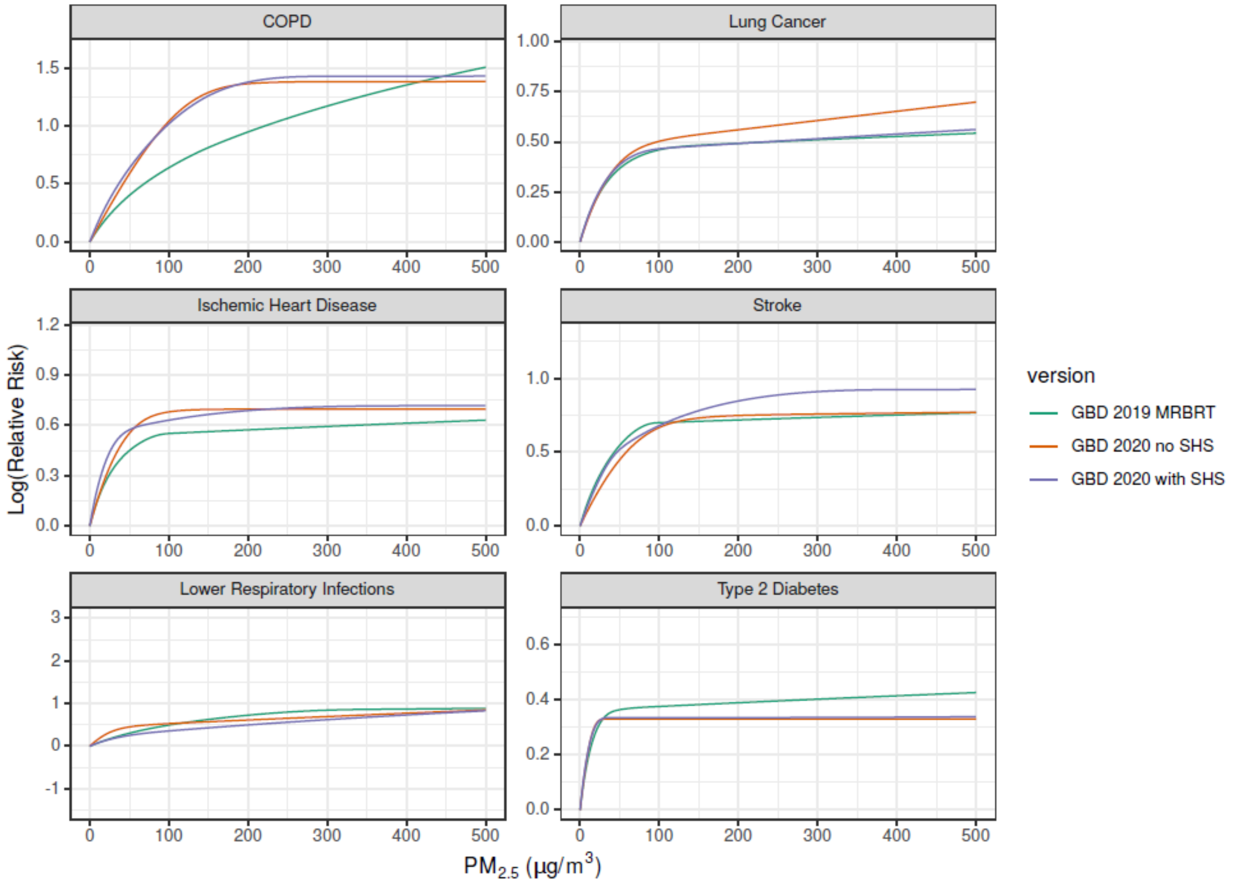

For GBD 2019, we implemented age-specific risk curves for cardiovascular diseases (ischaemic heart disease and stroke) due to evidence suggesting relative risk decreases with age for these outcomes.[17] These risk curves were created for five-year age groups from 25–29 to 95+. For GBD 2021, we dropped the use of age-specific risk curves for cardiovascular disease outcomes. Linear regressions on cardiovascular disease input data predicting log(RR) by mean cohort age, with and without random effects on study ID, were fit to ischaemic heart disease and stroke input data separately. None of these regressions showed evidence for a significant association between the two variables. Additionally, we used the MR-BRT automated covariate selection tool (detailed below) to test mean cohort age for significance as a bias covariate and found no significant results. We therefore generated a single risk curve for each of the cardiovascular outcomes and applied it across all age groups.

For all $PM_{2.5}$ outcomes, the standard error of observations from studies with multiple observations for a single cohort that reported an unstratified sample size were multiplied by the square root of *n*, where *n* is the total number of observations for a given cohort. This adjustment was made to prevent a single cohort or study from unduly weighting the final risk curve.

As in previous GBD cycles, we considered the published relative risk over a range of exposure data when fitting the risk curves. For OAP studies, the relative risk informs the curve from the 5th to the 95th percentile of observed exposure. When this is not available in the published study, we estimate the distribution from the provided information (mean and standard deviation, mean and IQR, etc.). We scale the RR to this range. For HAP studies, we allow each study to inform the curve from the Exp_{OAP} to the

Exp_{OAP}+Exp_{HAP}, where Exp_{OAP} is the GBD 2019 estimate of the ambient exposure level in the study location and year, and Exp_{HAP} is the GBD 2021 estimate of the excess exposure for those who use solid fuel for cooking in the study location and year.

MR-BRT risk splines

To estimate relative risk curves for each of the $PM_{2.5}$ outcomes, we used the MR-BRT meta-regression tool to fit splines on the input datasets of OAP and HAP studies. We used the following functional form, where X and X_{CF} represent the range of exposure characterised by the effect size:

$$log\left(\frac{MRBRT(X)}{MRBRT(X_{CF})}\right) \sim \log\left(Published\ Effect\ Size\right)$$

Several key updates were made to the model fitting methods. For each risk–outcome pair, model settings and priors were tested when fitting the MR-BRT splines. The final models used third-order splines with three interior knots and a constraint on the right-most segment forcing the fit to be linear rather than cubic. Splines were also constrained to be concave and monotonically increasing, the most biologically plausible shape for the $PM_{2.5}$ risk curve. We used an ensemble approach to generate final spline predictions, in which 50 different models were run with randomly placed knots, then weighted and combined based on a measure of fit that penalises excessive changes in the maximum derivative of the curve. Knots were free to be placed across the entire domain of the input exposure data. To prevent over-fitting, on the non-linear segments, we implemented a Gaussian prior on the third derivative of mean 0 and variance 1e-4. On the linear segment, a stronger prior of mean 0 and variance 1e-6 was used to ensure that the risk curves do not continue to increase beyond the range of the data. 10% of all observations were trimmed during model fitting, in accordance with GBD protocol across risk factor teams.

To select significant covariates from those extracted (see table below) to quantify between-study heterogeneity, we performed covariate selection. The MR-BRT automated covariate selection tool implements a two-step process. First, a series of loosening Lasso penalty parameters are applied to a log-linear meta-regression on all input effect size observations. Then, covariates with a non-zero coefficient are tested for significance using a Gaussian prior (significance threshold = 0.05). A Gaussian prior was used on each covariate's beta during spline fitting with a mean 0 and variance of 0.1 multiplied by the standard deviation of the beta from the log-linear meta-regression. Type 2 diabetes was the only outcome for which a significant covariate was identified. Its selected covariate was cv_hap, a binary indicator for whether or not an observation was from a household air pollution study.

**Covariate name**	**Covariate description**
cv_subpopulation	Study represents the general population; study represents a subgroup (eg, high-risk group)
cv_exposure_population	Study measures individual-level exposure (≤1 km radius); study measures population-level exposure
cv_exposure_self_report	Exposure is self-reported; exposure is measured externally
cv_exposure_study	Exposure is measured multiple times; exposure is measured only at baseline
cv_outcome_self_report	Outcome is self-reported; outcomes is based on death certificate or medical record

cv_outcome_unblinded	Study implements unblinded assessment; assessment of outcome is blind to exposure (and vice versa)
cv_reverse_causation	Study presents no risk of reverse causation; risk of reverse causation
cv_confounding_nonrandom	Non-randomised study; randomised study
cv_confounding_uncontrolled	Study is randomised/outcome controlled for age, sex, education, income, and all critical determinants of outcome; study is controlled for age, sex, and other critical determinants of outcome; study is controlled for only age and sex
cv_selection_bias	Study reports >95% follow-up; study reports 85–95% follow-up; study reports <85% follow-up
cv_hap	Studies household air pollution; studies ambient air pollution

1000 predictions of the effect size were generated across the exposure distribution for use in calculating burden estimates. These predictions were created by incorporating predictions of between-study heterogeneity to characterise the model's uncertainty. We implemented the Fisher scoring correction to the heterogeneity parameter, which corrects for data-sparse situations. In such cases, the between-study heterogeneity parameter estimate may be 0, simply from lack of data. The Fisher scoring correction uses a quantile of gamma, which is sensitive to the number of studies, study design, and reported uncertainty.

Risk-outcome scoring

Risk-outcome scores provide an empirical measure of the strength of evidence for risk-outcome pairs across risk factors in the GBD and are therefore useful for standardised comparison. Risk-outcome scores evaluate the area between the lower bound of the 95% uncertainty interval and the x-axis for harmful risk factors, including $PM_{2.5}$ pollution.

Prior to generating a risk-outcome score, we conducted an additional post-analysis step to detect and flag publication bias in the input data. This approach is based on the classic Egger's regression strategy, which is applied to the residuals in our model. In the current implementation, we do not correct for publication bias, but flag the risk–outcome pairs where the risk for publication bias is significant. Of the $PM_{2.5}$ outcomes, three were flagged for publication bias: birthweight, ischaemic heart disease, and type 2 diabetes.

Outcome	Egger p-value	Egger mean	Egger SD	Publication bias
Birthweight	0.0208	–0.322	0.158	X
Gestational age	0.249	–0.130	0.192	
Ischaemic heart disease	0.0164	0.322	0.151	X
Stroke	0.0717	0.186	0.127	
LRI	0.178	0.102	0.110	
Lung cancer	0.191	0.108	0.123	
COPD	0.423	0.0359	0.186	

Type 2 diabetes	0.0419	0.408	0.236	X

To calculate the risk-outcome score, we generated an uncertainty interval from 1000 draws of the adjusted summary effect size (retaining uncertainty information from between-study heterogeneity predictions and the Fisher information correction). We then evaluated the risk-outcome score between the 15th and 85th percentiles of the input data exposure distribution. Risk-outcome scores and star ratings are below. Risk-outcome scores are not reported for birthweight and gestational age because these are mediated outcomes.

Outcome	Risk-outcome score	Star rating
Ischaemic heart disease	0.259	3
Stroke	0.167	3
LRI	0.126	2
Lung cancer	0.342	3
COPD	0.441	4
Type 2 diabetes	0.188	3

The following table includes all ambient and household sources used to generate GBD 2021 risk curves.

Source	Reference
1	Abusalah A, Gavana M, Haidich AB, Smyrnakis E, Papadakis N, Papanikolaou A, Benos A. Low birth weight and prenatal exposure to indoor pollution from tobacco smoke and wood fuel smoke: a matched case-control study in Gaza Strip. Matern Child Health J. 2012; 16(8): 1718-27.
2	Akhtar T, Ullah Z, Khan MH, Nazli R. Chronic bronchitis in women using solid biomass fuel in rural Peshawar, Pakistan. Chest. 2007; 132(5): 1472–5.
3	Al-Sonboli N, Hart CA, Al-Aghbari N, Al-Ansi A, Ashoor O, Cuevas LE. Human metapneumovirus and respiratory syncytial virus disease in children, Yemen. Emerg Infect Dis. 2006; 12(9): 1437–9.
4	Alam DS, Chowdhury MAH, Siddiquee AT, Ahmed S, Hossain MD, Pervin S, Streatfield K, Cravioto A, Niessen LW. Adult Cardiopulmonary Mortality and Indoor Air Pollution: A 10-Year Retrospective Cohort Study in a Low-Income Rural Setting. Glob Heart. 2012; 7(3): 215–21.
5	Alexander DA, Northcross A, Karrison T, Morhasson-Bello O, Wilson N, Atalabi OM, Dutta A, Adu D, Ibigbami T, Olamijulo J, Adepoju D, Ojengbede O, Olopade CO. Pregnancy outcomes and ethanol cook stove intervention: A randomized-controlled trial in Ibadan, Nigeria. Environ Int. 2018; 111: 152-163.
6	Atkinson RW, Carey IM, Kent AJ, van Staa TP, Anderson HR, Cook DG. Long-term exposure to outdoor air pollution and the incidence of chronic obstructive pulmonary disease in a national English cohort. Occup Environ Med. 2015; 72(1): 42–8.

7	Azizi BH, Zulkifli HI, Kasim MS. Protective and risk factors for acute respiratory infections in hospitalized urban Malaysian children: a case control study. Southeast Asian J Trop Med Public Health. 1995; 26(2): 280–5.
8	Balakrishnan K, Ghosh S, Thangavel G, Sambandam S, Mukhopadhyay K, Puttaswamy N, Sadasivam A, Ramaswamy P, Johnson P, Kuppuswamy R, Natesan D, Maheshwari U, Natarajan A, Rajendran G, Ramasami R, Madhav S, Manivannan S, Nargunanadan S, Natarajan S, Saidam S, Chakraborty M, Balakrishnan L, Thanasekaraan V. Exposures to fine particulate matter (PM2.5) and birthweight in a rural-urban, mother-child cohort in Tamil Nadu, India. Environ Res. 2018; 161: 524–31.
9	Basu R, Harris M, Sie L, Malig B, Broadwin R, Green R. Effects of fine particulate matter and its constituents on low birth weight among full-term infants in California. Environ Res. 2014; 128: 42–51.
10	Basu R, Pearson D, Ebisu K, Malig B. Association between PM2.5 and PM2.5 Constituents and Preterm Delivery in California, 2000-2006. Paediatr Perinat Epidemiol. 2017; 31(5): 424-434.
11	Beelen R, Hoek G, van den Brandt PA, Goldbohm RA, Fischer P, Schouten LJ, Jerrett M, Hughes E, Armstrong B, Brunekreef B. Long-Term Effects of Traffic-Related Air Pollution on Mortality in a Dutch Cohort (NLCS-AIR Study) [Unpublished data]. Environ Health Perspect. 2008; 116(2): 196–202.
12	Beelen R, Hoek G, van den Brandt PA, Goldbohm RA, Fischer P, Schouten LJ, Jerrett M, Hughes E, Armstrong B, Brunekreef B. Long-Term Effects of Traffic-Related Air Pollution on Mortality in a Dutch Cohort (NLCS-AIR Study). Environ Health Perspect. 2008; 116(2): 196–202.
13	Beelen R, Stafoggia M, Raaschou-Nielsen O, Andersen ZJ, Xun WW, Katsouyanni K, Dimakopoulou K, Brunekreef B, Weinmayr G, Hoffmann B, Wolf K, Samoli E, Houthuijs D, Nieuwenhuijsen M, Oudin A, Forsberg B, Olsson D, Salomaa V, Lanki T, Yli-Tuomi T, Oftedal B, Aamodt G, Nafstad P, De Faire U, Pedersen NL, Östenson CG, Fratiglioni L, Penell J, Korek M, Pyko A, Eriksen KT, Tjønneland A, Becker T, Eeftens M, Bots M, Meliefste K, Wang M, Bueno-de-Mesquita B, Sugiri D, Krämer U, Heinrich J, de Hoogh K, Key T, Peters A, Cyrys J, Concin H, Nagel G, Ineichen A, Schaffner E, Probst-Hensch N, Dratva J, Ducret-Stich R, Vilier A, Clavel-Chapelon F, Stempfelet M, Grioni S, Krogh V, Tsai MY, Marcon A, Ricceri F, Sacerdote C, Galassi C, Migliore E, Ranzi A, Cesaroni G, Badaloni C, Forastiere F, Tamayo I, Amiano P, Dorronsoro M, Katsoulis M, Trichopoulou A, Vineis P, Hoek G. Long-term exposure to air pollution and cardiovascular mortality: an analysis of 22 European cohorts. Epidemiology. 2014; 25(3): 368–378.
14	Bell ML, Belanger K, Ebisu K, Gent JF, Lee HJ, Koutrakis P, Leaderer BP. Prenatal Exposure to Fine Particulate Matter and Birth Weight: Variations by Particulate Constituents and Sources. Epidemiology. 2010; 21(6): 884–91.
15	Bell ML, Ebisu K, Belanger K. Ambient Air Pollution and Low Birth Weight in Connecticut and Massachusetts. Environ Health Perspect. 2007; 115(7): 1118–24.
16	Benmarhnia T, Huang J, Basu R, Wu J, Bruckner TA. Decomposition Analysis of Black-White Disparities in Birth Outcomes: The Relative Contribution of Air Pollution and Social Factors in California. Environ Health Perspect. 2017; 125(10): 107003.

17	Bowe B, Xie Y, Li T, Yan Y, Xian H, Al-Aly Z. The 2016 global and national burden of diabetes mellitus attributable to PM2·5 air pollution. Lancet Planet Health. 2018; 2(7): e301–12.
18	Boy E, Bruce N, Delgado H. Birth weight and exposure to kitchen wood smoke during pregnancy in rural Guatemala. Environ Health Perspect. 2002; 110(1): 109-14.
19	Brauer M, Lencar C, Tamburic L, Koehoorn M, Demers P, Karr C. A cohort study of traffic-related air pollution impacts on birth outcomes. Environ Health Perspect. 2008; 116(5): 680-6.
20	Broor S, Pandey RM, Ghosh M, Maitreyi RS, Lodha R, Singhal T, Kabra SK. Risk factors for severe acute lower respiratory tract infection in under-five children. Indian Pediatr. 2001; 1361-9.
21	Burnett RT. Cox Proportional Survival Model Hazard Ratios from Census Year to 2011 for Adults Aged 25 to 89 in CanCHEC Cohort.
22	Cai J, Zhao Y, Kan J, Chen R, Martin R, van Donkelaar A, Ao J, Zhang J, Kan H, Hua J. Prenatal Exposure to Specific PM2.5 Chemical Constituents and Preterm Birth in China: A Nationwide Cohort Study. Environ Sci Technol. 2020; 54(22): 14494-14501.
23	Cakmak S, Hebbern C, Pinault L, Lavigne E, Vanos J, Crouse DL, Tjepkema M. Associations between long-term $PM_{2.5}$ and ozone exposure and mortality in the Canadian Census Health and Environment Cohort (CANCHEC), by spatial synoptic classification zone. Environ Int. 2018; 111: 200-211.
24	Carey IM, Atkinson RW, Kent AJ, van Staa T, Cook DG, Anderson HR. Mortality associations with long-term exposure to outdoor air pollution in a national English cohort. Am J Respir Crit Care Med. 2013; 187(11): 1226-33.
25	Cassidy-Bushrow AE, Burmeister C, Lamerato L, Lemke LD, Mathieu M, O'Leary BF, Sperone FG, Straughen JK, Reiners JJ Jr. Prenatal airshed pollutants and preterm birth in an observational birth cohort study in Detroit, Michigan, USA. Environ Res. 2020; 189: 109845.
26	Cesaroni G, Badaloni C, Gariazzo C, Stafoggia M, Sozzi R, Davoli M, Forastiere F. Long-term exposure to urban air pollution and mortality in a cohort of more than a million adults in Rome. Environ Health Perspect. 2013; 121(3): 324–31.
27	Chang HH, Reich BJ, Miranda ML. A spatial time-to-event approach for estimating associations between air pollution and preterm birth. J R Stat Soc Ser C Appl Stat. 2013; 62(2).
28	Chen H, Burnett RT, Kwong JC, Villeneuve PJ, Goldberg MS, Brook RD, van Donkelaar A, Jerrett M, Martin RV, Brook JR, Copes R. Risk of incident diabetes in relation to long-term exposure to fine particulate matter in Ontario, Canada. Environ Health Perspect. 2013; 121(7): 804–10.
29	Chen LH, Knutsen SF, Shavlik D, Beeson WL, Petersen F, Ghamsary M, Abbey D. The association between fatal coronary heart disease and ambient particulate air pollution: Are females at greater risk? Environ Health Perspect. 2005; 113(12): 1723-9.
30	Chen G, Guo Y, Abramson MJ, Williams G, Li S. Exposure to low concentrations of air pollutants and adverse birth outcomes in Brisbane, Australia, 2003-2013. Sci Total Environ. 2018; 622-623: 721-726.

31	Chen J, Fang J, Zhang Y, Xu Z, Byun HM, Li PH, Deng F, Guo X, Guo L, Wu S. Associations of adverse pregnancy outcomes with high ambient air pollution exposure: Results from the Project ELEFANT. Sci Total Environ. 2021; 761: 143218.
32	Clark C, Sbihi H, Tamburic L, Brauer M, Frank LD, Davies HW. Association of Long-Term Exposure to Transportation Noise and Traffic-Related Air Pollution with the Incidence of Diabetes: A Prospective Cohort Study. Environ Health Perspect. 2017; 125(8): 087025.
33	Clemens T, Turner S, Dibben C. Maternal exposure to ambient air pollution and fetal growth in North-East Scotland: A population-based study using routine ultrasound scans. Environ Int. 2017; 107: 216–26.
34	Coker E, Ghosh J, Jerrett M, Gomez-Rubio V, Beckerman B, Cockburn M, Liverani S, Su J, Li A, Kile ML, Ritz B, Molitor J. Modeling spatial effects of PM(2.5) on term low birth weight in Los Angeles County. Environ Res. 2015; 142: 354-64.
35	Collings DA, Sithole SD, Martin KS. Indoor woodsmoke pollution causing lower respiratory disease in children. Trop Doct. 1990; 20(4): 151–5.
36	Coogan PF, White LF, Yu J, Burnett RT, Seto E, Brook RD, Palmer JR, Rosenberg L, Jerrett M. PM2.5 and Diabetes and Hypertension Incidence in the Black Women's Health Study. Epidemiology. 2016; 27(2): 202–10.
37	Cramer J, Jørgensen JT, Hoffmann B, et al. Long-Term Exposure to Air Pollution and Incidence of Myocardial Infarction: A Danish Nurse Cohort Study. Environ Health Perspect. 2020;128(5):57003. doi:10.1289/EHP5818
38	Dadvand P, Ostro B, Figueras F, Foraster M, Basagaña X, Valentín A, Martinez D, Beelen R, Cirach M, Hoek G, Jerrett M, Brunekreef B, Nieuwenhuijsen MJ. Residential proximity to major roads and term low birth weight: the roles of air pollution, heat, noise, and road-adjacent trees. Epidemiology. 2014; 25(4): 518-25.
39	Darrow LA, Klein M, Strickland MJ, Mulholland JA, Tolbert PE. Ambient Air Pollution and Birth Weight in Full-Term Infants in Atlanta, 1994–2004. Environ Health Perspect. 2011; 119(5): 731–7.
40	Dennis RJ, Maldonado D, Norman S, Baena E, Martinez G. Woodsmoke exposure and risk for obstructive airways disease among women. Chest. 1996; 109(1): 115–9.
41	Dherani M, Pope D, Mascarenhas M, Smith KR, Weber M, Bruce N. Indoor air pollution from unprocessed solid fuel use and pneumonia risk in children aged under five years: a systematic review and meta-analysis. Bull World Health Organ. 2008; 86(5): 390-398C and Kossove D. and Jeena PM, Ayannusi OE, Annamalai K, Naidoo P, Coovadia HM, Guldner P. Risk factors for admission and the role of respiratory syncytial virus-specific cytotoxic T-lymphocyte responses in children with acute bronchiolitis. S Afr Med J. 2003; 93(4): 291–4.
42	Ebisu K, Bell ML. Airborne PM2.5 chemical components and low birth weight in the northeastern and mid-Atlantic regions of the United States. Environ Health Perspect. 2012; 120(12): 1746-52.
43	Ebisu K, Berman JD, Bell ML. Exposure to coarse particulate matter during gestation and birth weight in the U.S. Environ Int. 2016; 94: 519–24.

44	Ebisu K, Belanger K, Bell ML. The Association between Airborne PM2.5 Chemical Constituents and Birth Weight-Implication of Buffer Exposure Assignment. Environ Res Lett. 2014; 9(8).
45	Erickson AC, Ostry A, Chan LH, Arbour L. The reduction of birth weight by fine particulate matter and its modification by maternal and neighbourhood-level factors: a multilevel analysis in British Columbia, Canada. Environ Health. 2016; 15: 51.
46	Fleischer NL, Merialdi M, van Donkelaar A, Vadillo-Ortega F, Martin RV, Betran AP, Souza JP. Outdoor air pollution, preterm birth, and low birth weight: analysis of the world health organization global survey on maternal and perinatal health. Environ Health Perspect. 2014; 122(4): 425-30.
47	Fong KC, Kosheleva A, Kloog I, Koutrakis P, Laden F, Coull BA, Schwartz JD. Fine Particulate Air Pollution and Birthweight: Differences in Associations Along the Birthweight Distribution. Epidemiology. 2019; 30(5): 617-623.
48	Fonseca W, Kirkwood BR, Victora CG, Fuchs SR, Flores JA, Misago C. Risk factors for childhood pneumonia among the urban poor in Fortaleza, Brazil: a case-control study. Bull World Health Organ. 1996; 74(2): 199–208.
49	Galeone C, Pelucchi C, La Vecchia C, Negri E, Bosetti C, Hu J. Indoor air pollution from solid fuel use, chronic lung diseases and lung cancer in Harbin, Northeast China. Eur J Cancer Prev. 2008; 17(5): 473–8.
50	Gan WQ, FitzGerald JM, Carlsten C, Sadatsafavi M, Brauer M. Associations of ambient air pollution with chronic obstructive pulmonary disease hospitalization and mortality. Am J Respir Crit Care Med. 2013; 187(7): 721–7.
51	Gan WQ, Koehoorn M, Davies HW, Demers PA, Tamburic L, Brauer M. Long-Term Exposure to Traffic-Related Air Pollution and the Risk of Coronary Heart Disease Hospitalization and Mortality. Environ Health Perspect. 2011; 119(4): 501–7.
52	Garcia CA, Yap PS, Park HY, Weller BL. 2016. Association of long-term PM2.5 exposure with mortality using different air pollution exposure models: impacts in rural and urban California. International Journal of Environmental Health Research, 26(2), 145-15.
53	Geer LA, Weedon J, Bell ML. Ambient air pollution and term birth weight in Texas from 1998 to 2004. J Air Waste Manag Assoc. 2012; 62(11): 1285–95.
54	Gehring U, Tamburic L, Sbihi H, Davies HW, Brauer M. Impact of Noise and Air Pollution on Pregnancy Outcomes. Epidemiology. 2014; 25(3): 351–8.
55	Gehring U, Wijga AH, Fischer P, de Jongste JC, Kerkhof M, Koppelman GH, Smit HA, Brunekreef B. Traffic-related air pollution, preterm birth and term birth weight in the PIAMA birth cohort study. Environ Res. 2011; 111(1): 125–35.
56	Ger LP, Hsu WL, Chen KT, Chen CJ. Risk Factors of Lung Cancer by Histological Category in Taiwan. Anticancer Res. 1993; 13(5A): 1491–500.
57	Giorgis-Allemand L, Pedersen M, Bernard C, Aguilera I, Beelen RM, Chatzi L, Cirach M, Danileviciute A, Dedele A, van Eijsden M, Estarlich M, Fernández-Somoano A, Fernández MF, Forastiere F, Gehring U, Grazuleviciene R, Gruzieva O, Heude B, Hoek G, de Hoogh K, van den Hooven EH, Håberg SE, Iñiguez C, Jaddoe VW, Korek M, Lertxundi A, Lepeule J, Nafstad P, Nystad W, Patelarou E, Porta D, Postma D, Raaschou-Nielsen O, Rudnai P, Siroux V, Sunyer J, Stephanou E, Sørensen M, Eriksen

	KT, Tuffnell D, Varró MJ, Vrijkotte TG, Wijga A, Wright J, Nieuwenhuijsen MJ, Pershagen G, Brunekreef B, Kogevinas M, Slama R. The Influence of Meteorological Factors and Atmospheric Pollutants on the Risk of Preterm Birth. Am J Epidemiol. 2017; 185(4): 247-258.
58	Gray SC, Edwards SE, Schultz BD, Miranda ML. Assessing the impact of race, social factors and air pollution on birth outcomes: a population-based study. Environ Health. 2014; 13(1): 4.
59	Gray SC, Gelfand AE, Miranda ML. Hierarchical spatial modeling of uncertainty in air pollution and birth weight study. Stat Med. 2011; 30(17): 2187-98.
60	Guo T, Wang Y, Zhang H, Zhang Y, Zhao J, Wang Q, Shen H, Wang Y, Xie X, Wang L, Xu Z, Zhang Y, Yan D, He Y, Yang Y, Xu J, Peng Z, Ma X. The association between ambient PM2.5 exposure and the risk of preterm birth in China: A retrospective cohort study. Sci Total Environ. 2018; 633: 1453-1459.
61	Gupta D, Boffetta P, Gaborieau V, Jindal SK. Risk factors of lung cancer in Chandigarh, India. Indian J Med Res. 2001; 113: 142–50.
62	Ha S, Hu H, Roussos-Ross D, Haidong K, Roth J, Xu X. The effects of air pollution on adverse birth outcomes. Environ Res. 2014; 134: 198-204.
63	Ha S, Zhu Y, Liu D, Sherman S, Mendola P. Ambient temperature and air quality in relation to small for gestational age and term low birthweight. Environ Res. 2017; 15 5: 394-400.
64	Han Y, Ji Y, Kang S, Dong T, Zhou Z, Zhang Y, Chen M, Wu W, Tang Q, Chen T, Wang Y, Xia Y. Effects of particulate matter exposure during pregnancy on birth weight: A retrospective cohort study in Suzhou, China. Sci Total Environ. 2018; 615: 369-374.
65	Hansen AB, Ravnskjær L, Loft S, Andersen KK, Bräuner EV, Baastrup R, Yao C, Ketzel M, Becker T, Brandt J, Hertel O, Andersen ZJ. Long-term exposure to fine particulate matter and incidence of diabetes in the Danish Nurse Cohort. Environ Int. 2016; 91: 243–50.
66	Hao H, Chang HH, Holmes HA, Mulholland JA, Klein M, Darrow LA, Strickland MJ. Air Pollution and Preterm Birth in the U.S. State of Georgia (2002-2006): Associations with Concentrations of 11 Ambient Air Pollutants Estimated by Combining Community Multiscale Air Quality Model (CMAQ) Simulations with Stationary Monitor Measurements. Environ Health Perspect. 2016; 124(6): 875-80.
67	Hao Y, Strosnider H, Balluz L, Qualters JR. Geographic Variation in the Association between Ambient Fine Particulate Matter (PM2.5) and Term Low Birth Weight in the United States. Environ Health Perspect. 2016; 124(2): 250-5.
68	Harris G, Thompson WD, Fitzgerald E, Wartenberg D. The association of PM(2.5) with full term low birth weight at different spatial scales. Environ Res. 2014; 134: 427-34.
69	Hart J, Garshick E, Dockery D, Smith T, Ryan L, Laden F. Long-Term Ambient Multipollutant Exposures and Mortality. Am J Respir Crit Care Med. 2011; 183: 75–8.
70	Hart JE, Puett RC, Rexrode KM, Albert CM, Laden F. Effect Modification of Long-Term Air Pollution Exposures and the Risk of Incident Cardiovascular Disease in US Women. J Am Heart Assoc. 2015; 4(12).
71	Heft-Neal S, Burney J, Bendavid E, Burke M. Robust relationship between air quality and infant mortality in Africa. Nature. 2018; 559(7713): 2548.

72	Hertz-Picciotto I, Baker RJ, Yap P-S, Dostál M, Joad JP, Lipsett M, Greenfield T, Herr CEW, Benes I, Shumway RH, Pinkerton KE, Srám R. Early childhood lower respiratory illness and air pollution. Environ Health Perspect. 2007; 115(10): 1510-8.
73	Honda T, Pun VC, Manjourides J, et al. Associations between long-term exposure to air pollution, glycosylated hemoglobin and diabetes. Int J Hyg Environ Health. 2017, 220 (7): 1124-1132.
74	Huang C, Zhang X, Qiao Z, Guan L, Peng S, Liu J, Xie R, Zheng L. A case-control study of dietary factors in patients with lung cancer. Biomed Environ Sci. 1992; 5(3): 257–65.
75	Huang H, Woodruff TJ, Baer RJ, Bangia K, August LM, Jellife-Palowski LL, Padula AM, Sirota M. Investigation of association between environmental and socioeconomic factors and preterm birth in California. Environ Int. 2018; 121(Pt 2): 1066-1078.
76	Huang K, Liang F, Yang X, Liu F, Li J, Xiao Q, Chen J, Liu X, Cao J, Shen C, Yu L, Lu F, Wu X, Zhao L, Wu X, Li Y, Hu D, Huang J, Liu Y, Lu X, Gu D. Long term exposure to ambient fine particulate matter and incidence of stroke: prospective cohort study from the China-PAR project. BMJ. 2019; 367: l6720.
77	Huynh M, Woodruff TJ, Parker JD, Schoendorf KC. Relationships between air pollution and preterm birth in California. Paediatr Perinat Epidemiol. 2006; 20(6): 454-61.
78	Hyder A, Lee HJ, Ebisu K, Koutrakis P, Belanger K, Bell ML. PM2.5 Exposure and Birth Outcomes: Use of Satellite- and Monitor-Based Data. Epidemiology. 2014; 25(1): 58–67.
79	Hystad P, Demers PA, Johnson KC, Carpiano RM, Brauer M. Long-term residential exposure to air pollution and lung cancer risk. Epidemiology. 2013; 24(5): 762-72.
80	Hystad P, Duong M, Brauer M, Larkin A, Arku R, Kurmi OP, Fan WQ, Avezum A, Azam I, Chifamba J, Dans A, du Plessis JL, Gupta R, Kumar R, Lanas F, Liu Z, Lu Y, Lopez-Jaramillo P, Mony P, Mohan V, Mohan D, Nair S, Puoane T, Rahman O, Lap AT, Wang Y, Wei L, Yeates K, Rangarajan S, Teo K, Yusuf S, on behalf of Prospective Urban and Rural Epidemiological (PURE) Study investigators. Health Effects of Household Solid Fuel Use: Findings from 11 Countries within the Prospective Urban and Rural Epidemiology Study [Unpublished]. Environ Health Perspect. 2019; 127(5): 57003.
81	Hystad P, Duong M, Brauer M, Larkin A, Arku R, Kurmi OP, Fan WQ, Avezum A, Azam I, Chifamba J, Dans A, du Plessis JL, Gupta R, Kumar R, Lanas F, Liu Z, Lu Y, Lopez-Jaramillo P, Mony P, Mohan V, Mohan D, Nair S, Puoane T, Rahman O, Lap AT, Wang Y, Wei L, Yeates K, Rangarajan S, Teo K, Yusuf S, on behalf of Prospective Urban and Rural Epidemiological (PURE) Study investigators. Health Effects of Household Solid Fuel Use: Findings from 11 Countries within the Prospective Urban and Rural Epidemiology Study. Environ Health Perspect. 2019; 127(5): 57003.
82	Hystad P, Larkin A, Rangarajan S, PURE country investigators, Yusuf S, Brauer M. Outdoor fine particulate matter air pollution and cardiovascular disease: Results from 747 communities across 21 countries in the PURE Study [Unpublished].
83	Jedrychowski W, Perera F, Mrozek-Budzyn D, Mroz E, Flak E, Spengler JD, Edwards S, Jacek R, Kaim I, Skolicki Z. Gender differences in fetal growth of newborns exposed prenatally to airborne fine particulate matter. Environ Res. 2009; 109(4): 447-56.

84	Jerrett M, Burnett RT, Beckerman BS, et al. 2013. Spatial analysis of air pollution and mortality in California. American Journal of Respiratory and Critical Care Medicine, 188(5), 593-599.
85	Jin C, Rossignol AM. Effects of passive smoking on respiratory illness from birth to age eighteen months, in Shanghai, People's Republic of China. J Pediatr. 1993; 123(4): 553–8.
86	Johnson AW, Aderele WI. The association of household pollutants and socio-economic risk factors with the short-term outcome of acute lower respiratory infections in hospitalized pre-school Nigerian children. Ann Trop Paediatr. 1992; 12(4): 421–32.
87	Karr C, Lumley T, Schreuder A, Davis R, Larson T, Ritz B, Kaufman J. Effects of subchronic and chronic exposure to ambient air pollutants on infant bronchiolitis. Am J Epidemiol. 2007; 165(5): 553-60.
88	Karr CJ, Rudra CB, Miller KA, Gould TR, Larson T, Sathyanarayana S, Koenig JQ. Infant exposure to fine particulate matter and traffic and risk of hospitalization for RSV bronchiolitis in a region with lower ambient air pollution. Environ Res. 2009; 109(3): 321-7.
89	Katanoda K, Sobue T, Satoh H, Tajima K, Suzuki T, Nakatsuka H, Takezaki T, Nakayama T, Nitta H, Tanabe K, Tominaga S. An association between long-term exposure to ambient air pollution and mortality from lung cancer and respiratory diseases in Japan. J Epidemiol. 2011; 21(2): 132-43.
90	Kim C, Seow WJ, Shu X-O, Bassig BA, Rothman N, Chen BE, Xiang Y-B, Hosgood HD, Ji B-T, Hu W, Wen C, Chow W-H, Cai Q, Yang G, Gao Y-T, Zheng W, Lan Q. Cooking Coal Use and All-Cause and Cause-Specific Mortality in a Prospective Cohort Study of Women in Shanghai, China. Environ Health Perspect. 2016; 124(9): 1384–9.
91	Kingsley SL, Eliot MN, Glazer K, Awad YA, Schwartz JD, Savitz DA, Kelsey KT, Marsit CJ, Wellenius GA. Maternal ambient air pollution, preterm birth and markers of fetal growth in Rhode Island: results of a hospital-based linkage study. J Epidemiol Community Health. 2017; 71(12): 1131-1136.
92	Kirwa K, McConnell-Rios R, Manjourides J, Cordero J, Alshawabekeh A, Suh HH. Low birth weight and PM2.5 in Puerto Rico. Environ Epidemiol. 2019; 3(4).
93	Kleinerman RA, Wang Z, Wang L, Metayer C, Zhang S, Brenner AV, Zhang S, Xia Y, Shang B, Lubin JH. Lung cancer and indoor exposure to coal and biomass in rural China. J Occup Environ Med. 2002; 44(4): 338–44.
94	Kloog I, Melly SJ, Ridgway WL, Coull BA, Schwartz J. Using new satellite based exposure methods to study the association between pregnancy pm2.5 exposure, premature birth and birth weight in Massachusetts. Environ Health. 2012; 11(1).
95	Ko YC, Lee CH, Chen MJ, Huang CC, Chang WY, Lin HJ, Wang HZ, Chang PY. Risk factors for primary lung cancer among non-smoking women in Taiwan. Int J Epidemiol. 1997; 26(1): 24-31.
96	Kumar N. Uncertainty in the relationship between criteria pollutants and low birth weight in Chicago. Atmos Environ. 2012; 49: 171–9.
97	Kumar S, Awasthi S, Jain A, Srivastava RC. Blood zinc levels in children hospitalized with severe pneumonia: a case control study. Indian Pediatr. 2004; 41(5): 486–91.

98	Lamichhane DK, Lee SY, Ahn K, Kim KW, Shin YH, Suh DI, Hong SJ, Kim HC. Quantile regression analysis of the socioeconomic inequalities in air pollution and birth weight. Environ Int. 2020; 142: 105875.
99	Lan Q, He X, Shen M, Tian L, Liu LZ, Lai H, Chen W, Berndt SI, Hosgood HD, Lee K-M, Zheng T, Blair A, Chapman RS. Variation in lung cancer risk by smoky coal subtype in Xuanwei, China. Int J Cancer. 2008; 123(9): 2164–9.
100	Laurent O, Hu J, Li L, Cockburn M, Escobedo L, Kleeman MJ, Wu J. Sources and contents of air pollution affecting term low birth weight in Los Angeles County, California, 2001-2008. Environ Res. 2014; 134: 488-95.
101	Laurent O, Hu J, Li L, Kleeman MJ, Bartell SM, Cockburn M, Escobedo L, Wu J. A Statewide Nested Case-Control Study of Preterm Birth and Air Pollution by Source and Composition: California, 2001-2008. Environ Health Perspect. 2016; 124(9): 1479-86.
102	Laurent O, Hu J, Li L, Kleeman MJ, Bartell SM, Cockburn M, Escobedo L, Wu J. Low birth weight and air pollution in California: Which sources and components drive the risk? Environ Int. 2016; 92-93: 471-7.
103	Laurent O, Wu J, Li L, Chung J, Bartell S. Investigating the association between birth weight and complementary air pollution metrics: a cohort study. Environ Health. 2013; 12(1).
104	Lavigne E, Yasseen AS 3rd, Stieb DM, Hystad P, van Donkelaar A, Martin RV, Brook JR, Crouse DL, Burnett RT, Chen H, Weichenthal S, Johnson M, Villeneuve PJ, Walker M. Ambient air pollution and adverse birth outcomes: Differences by maternal comorbidities. Environ Res. 2016; 148: 457-466.
105	Lavigne É, Burnett RT, Stieb DM, Evans GJ, Godri Pollitt KJ, Chen H, van Rijswijk D, Weichenthal S. Fine Particulate Air Pollution and Adverse Birth Outcomes: Effect Modification by Regional Nonvolatile Oxidative Potential. Environ Health Perspect. 2018; 126(7): 077012.
106	Le CH, Ko YC, Cheng LS, Lin YC, Lin HJ, Huang MS, Huang JJ, Kao EL, Wang HZ. The heterogeneity in risk factors of lung cancer and the difference of histologic distribution between genders in Taiwan. Cancer Causes Control. 2001; 12(4): 289–300.
107	Lepeule J, Laden F, Dockery D, Schwartz J. Chronic exposure to fine particles and mortality: an extended follow-up of the Harvard Six Cities study from 1974 to 2009 - Unpublished data. Environ Health Perspect. 2012; 120(7): 965-70.
108	Lepeule J, Laden F, Dockery D, Schwartz J. Chronic exposure to fine particles and mortality: an extended follow-up of the Harvard Six Cities study from 1974 to 2009. Environ Health Perspect. 2012; 120(7): 965-70.
109	Li Q, Wang YY, Guo Y, Zhou H, Wang X, Wang Q, Shen H, Zhang Y, Yan D, Zhang Y, Zhang H, Li S, Chen G, Lin L, Zhao J, He Y, Yang Y, Xu J, Wang Y, Peng Z, Wang HJ, Ma X . Effect of airborne particulate matter of 2.5m or less on preterm birth: A national birth cohort study in China. Environ Int. 2018; 121(Pt 2): 1128-1136.
110	Li Q, Wang YY, Guo Y, Zhou H, Wang X, Wang QM, Shen HP, Zhang YP, Yan DH, Li S, Chen G, Lin L, He Y, Yang Y, Peng ZQ, Wang HJ, Ma X. Folic Acid Supplementation and the Association between Maternal Airborne Particulate Matter

	Exposure and Preterm Delivery: A National Birth Cohort Study in China. Environ Health Perspect. 2020; 128(12): 127010.
111	Li Z, Yuan X, Fu J, Zhang L, Hong L, Hu L, Liu L. Association of ambient air pollutants an d birth weight in Ningbo, 2015-2017. Environ Pollut. 2019; 249: 629-637.
112	Lim CC, Hayes RB, Ahn J, Shao Y, Silverman DT, Jones RR, Garcia C, Thurston GD. Association between long-term exposure to ambient air pollution and diabetes mortality in the US. Environ Res. 2018; 165: 330-36
113	Lin L, Li Q, Yang J, Han N, Jin C, Xu X, Liu Z, Liu J, Luo S, Raat H, Wang H. The associations of particulate matters with fetal growth in utero and birth weight: A birth cohort study in Beijing, China. Sci Total Environ. 2020; 709: 136246.
114	Lipsett MJ, Ostro BD, Reynolds P, Goldberg D, Hertz A, Jerrett M, Smith DF, Garcia C, Chang ET, Bernstein L. Long-term exposure to air pollution and cardiorespiratory disease in the California teachers study cohort [Unpublished data]. Am J Respir Crit Care Med. 2011; 184(7): 828-35.
115	Lipsett MJ, Ostro BD, Reynolds P, Goldberg D, Hertz A, Jerrett M, Smith DF, Garcia C, Chang ET, Bernstein L. Long-term exposure to air pollution and cardiorespiratory disease in the California teachers study cohort. Am J Respir Crit Care Med. 2011; 184(7): 828-35.
116	Lissowska J, Bardin-Mikolajczak A, Fletcher T, Zaridze D, Szeszenia-Dabrowska N, Rudnai P, Fabianova E, Cassidy A, Mates D, Holcatova I, Vitova V, Janout V, Mannetje A, Brennan P, Boffetta P. Lung cancer and indoor pollution from heating and cooking with solid fuels: the IARC international multicentre case-control study in Eastern/Central Europe and the United Kingdom. Am J Epidemiol. 2005; 162(4): 326–33.
117	Luo RX, Wu B, Yi YN, Huang ZW, Lin RT. Indoor burning coal air pollution and lung cancer--a case-control study in Fuzhou, China. Lung Cancer. 1996; 14 Suppl 1: S113-119.
118	MacIntyre EA, Gehring U, Mölter A, Fuertes E, Klümper C, Krämer U, Quass U, Hoffmann B, Gascon M, Brunekreef B, Koppelman GH, Beelen R, Hoek G, Birk M, de Jongste JC, Smit HA, Cyrys J, Gruzieva O, Korek M, Bergström A, Agius RM, de Vocht F, Simpson A, Porta D, Forastiere F, Badaloni C, Cesaroni G, Esplugues A, Fernández-Somoano A, Lerxundi A, Sunyer J, Cirach M, Nieuwenhuijsen MJ, Pershagen G, Heinrich J. Air Pollution and Respiratory Infections during Early Childhood: An Analysis of 10 European Birth Cohorts within the ESCAPE Project. Environ Health Perspect. 2014; 122(1): 107–13.
119	Mahalanabis D, Gupta S, Paul D, Gupta A, Lahiri M, Khaled MA. Risk factors for pneumonia in infants and young children and the role of solid fuel for cooking: a case-control study. Epidemiol Infect. 2002; 129(1): 65–71.
120	Melody S, Wills K, Knibbs LD, Ford J, Venn A, Johnston F. Adverse birth outcomes in Victoria, Australia in association with maternal exposure to low levels of ambient air pollution. Environ Res. 2020; 188: 109784.
121	Miller KA, Siscovick DS, Sheppard L, Shepherd K, Sullivan JH, Anderson GL, Kaufman JD. Long-term exposure to air pollution and incidence of cardiovascular events in women. N Engl J Med. 2007; 356(5): 447-58.

122	Morello-Frosch R, Jesdale BM, Sadd JL, Pastor M. Ambient air pollution exposure and full-term birth weight in California. Environ Health. 2010; 9(1).
123	Naess Ø, Nafstad P, Aamodt G, Claussen B, Rosland P. Relation between concentration of air pollution and cause-specific mortality: four-year exposures to nitrogen dioxide and particulate matter pollutants in 470 neighborhoods in Oslo, Norway. Am J Epidemiol. 2007; 165(4): 435-43.
124	Ng C, Malig B, Hasheminassab S, Sioutas C, Basu R, Ebisu K. Source apportionment of fine particulate matter and risk of term low birth weight in California: Exploring modification by region and maternal characteristics. Sci Total Environ. 2017; 605-606: 647-654.
125	Ostro B, Hu J, Goldberg D, et al. 2015. Associations of mortality with long-term exposures to fine and ultrafine particles, species and sources: results from the California Teachers Study Cohort. Environmental Health Perspectives, 123(6), 549-556.
126	Ottone M, Broccoli S, Parmagnani F, Giannini S, Scotto F, Bonvicini L, Luberto F, Bacco D, Trentini A, Poluzzi V, Angelini P, Colacci A, Giorgi Rossi P, Ranzi A. Source-related components of fine particulate matter and risk of adverse birth outcomes in Northern Italy. Environ Res. 2020; 186: 109564.
127	Park SK, Adar SD, O'Neill MS, Auchincloss AH, Szpiro A, Bertoni AG, Navas-Acien A, Kaufman JD, Diez-Roux AV. Long-term exposure to air pollution and type 2 diabetes mellitus in a multiethnic cohort. Am J Epidemiol. 2015; 181(5): 327–36.
128	Parker JD, Woodruff TJ, Basu R, Schoendorf KC. Air Pollution and Birth Weight Among Term Infants in California. Pediatrics. 2005; 115(1): 121–8.
129	Parker JD, Woodruff TJ. Influences of study design and location on the relationship between particulate matter air pollution and birthweight. Paediatr Perinat Epidemiol. 2008; 22(3): 214–27.
130	Parker JD, Kravets N, Vaidyanathan A. 2018. Particulate matter air pollution exposure and heart disease mortality risks by race and ethnicity in the United States: 1997 to 2009 National Health Interview Survey with mortality follow-up through 2011. Circulation, 137(16), 1688-1697.
131	Pedersen M, Giorgis-Allemand L, Bernard C, Aguilera I, Andersen AM, Ballester F, Beelen RM, Chatzi L, Cirach M, Danileviciute A, Dedele A, Eijsden Mv, Estarlich M, Fernández-Somoano A, Fernández MF, Forastiere F, Gehring U, Grazuleviciene R, Gruzieva O, Heude B, Hoek G, de Hoogh K, van den Hooven EH, Håberg SE, Jaddoe VW, Klümper C, Korek M, Krämer U, Lerchundi A, Lepeule J, Nafstad P, Nystad W, Patelarou E, Porta D, Postma D, Raaschou-Nielsen O, Rudnai P, Sunyer J, Stephanou E, Sørensen M, Thiering E, Tuffnell D, Varró MJ, Vrijkotte TG, Wijga A, Wilhelm M, Wright J, Nieuwenhuijsen MJ, Pershagen G, Brunekreef B, Kogevinas M, Slama R. Ambient air pollution and low birthweight: a European cohort study (ESCAPE). Lancet Respir Med. 2013; 1(9): 695-704.
132	Pereira G, Belanger K, Ebisu K, Bell ML. Fine particulate matter and risk of preterm birth in Connecticut in 2000-2006: a longitudinal study. Am J Epidemiol. 2014; 179(1): 67-74.

133	Pereira G, Bell ML, Belanger K, de Klerk N. Fine particulate matter and risk of preterm birth and pre-labor rupture of membranes in Perth, Western Australia 1997-2007: a longitudinal study. Environ Int. 2014; 73: 143-9.
134	Pinault L, Tjepkema M, Crouse DL, Weichenthal S, van Donkelaar A, Martin RV, Brauer M, Chen H, Burnett RT. Risk estimates of mortality attributed to low concentrations of ambient fine particulate matter in the Canadian community health survey cohort [Unpublished]. Environ Health. 2016; 15: 18.
135	Pinault L, Tjepkema M, Crouse DL, Weichenthal S, van Donkelaar A, Martin RV, Brauer M, Chen H, Burnett RT. Risk estimates of mortality attributed to low concentrations of ambient fine particulate matter in the Canadian community health survey cohort. Environ Health. 2016; 15(1): 18.
136	Pinault L, Brauer M, Crouse DL, et al. 2018. Diabetes status and susceptibility to the effects of PM2.5 exposure on cardiovascular mortality in a National Canadian Cohort. Epidemiology, 29(6), 784-794.
137	Pope CA, Burnett R, Thun M, Calle E, Krewski D, Ito K, Thurston G. Lung Cancer, Cardiopulmonary Mortality, and Long-term Exposure to Fine Particulate Air Pollution. JAMA. 2002; 287(9): 1132–41.
138	Pope CA, Lefler JS, Ezzati M, et al. 2019. Mortality Risk and Fine Particulate Air Pollution in a Large, Representative Cohort of US Adults. Environmental Health Perspectives, 127(7), 077007.
139	Puett RC, Hart JE, Suh H, Mittleman M, Laden F. Particulate matter exposures, mortality, and cardiovascular disease in the health professionals follow-up study. Environ Health Perspect. 2011; 119(8): 1130-5.
140	Puett RC, Hart JE, Yanosky JD, Paciorek C, Schwartz J, Suh H, Speizer FE, Laden F. Chronic fine and coarse particulate exposure, mortality, and coronary heart disease in the Nurses' Health Study. Environ Health Perspect. 2009; 117(11): 1697-701.
141	Qian Z, Liang S, Yang S, Trevathan E, Huang Z, Yang R, Wang J, Hu K, Zhang Y, Vaughn M, Shen L, Liu W, Li P, Ward P, Yang L, Zhang W, Chen W, Dong G, Zheng T, Xu S, Zhang B. Ambient air pollution and preterm birth: A prospective birth cohort study in Wuhan, China. Int J Hyg Environ Health. 2016; 219(2): 195-203.
142	Qiu H, Schooling CM, Sun S, Tsang H, Yang Y, Lee RS, Wong CM, Tian L. Long-term exposure to fine particulate matter air pollution and type 2 diabetes mellitus in elderly: A cohort study in Hong Kong. Environ Int. 2018; 113: 350-56.
143	Qiu H, Sun S, Tsang H, Wong CM, Lee RS, Schooling CM, Tian L. Fine particulate matte r exposure and incidence of stroke: A cohort study in Hong Kong. Neurology. 2017; 88(18): 1709-1717.
144	Raaschou-Nielsen O, Andersen ZJ, Beelen R, Samoli E, Stafoggia M, Weinmayr G, Hoffmann B, Fischer P, Nieuwenhuijsen MJ, Brunekreef B, Xun WW, Katsouyanni K, Dimakopoulou K, Sommar J, Forsberg B, Modig L, Oudin A, Oftedal B, Schwarze PE, Nafstad P, De Faire U, Pedersen NL, Ostenson C-G, Fratiglioni L, Penell J, Korek M, Pershagen G, Eriksen KT, Sørensen M, Tjønneland A, Ellermann T, Eeftens M, Peeters PH, Meliefste K, Wang M, Bueno-de-Mesquita B, Key TJ, de Hoogh K, Concin H, Nagel G, Vilier A, Grioni S, Krogh V, Tsai M-Y, Ricceri F, Sacerdote C, Galassi C, Migliore E, Ranzi A, Cesaroni G, Badaloni C, Forastiere F, Tamayo I, Amiano P, Dorronsoro M,

	Trichopoulou A, Bamia C, Vineis P, Hoek G. Air pollution and lung cancer incidence in 17 European cohorts: prospective analyses from the European Study of Cohorts for Air Pollution Effects (ESCAPE). Lancet Oncol. 2013; 14(9): 813–22.
145	Renzi M, Cerza F, Gariazzo C, et al. Air pollution and occurrence of type 2 diabetes in a large cohort study. Environ Int. 2018, 112: 68-76.
146	Robin LF, Less PS, Winget M, Steinhoff M, Moulton LH, Santosham M, Correa A. Wood-burning stoves and lower respiratory illnesses in Navajo children. Pediatr Infect Dis J. 1996; 15(10): 859–65.
147	Sapkota A, Gajalakshmi V, Jetly DH, Roychowdhury S, Dikshit RP, Brennan P, Hashibe M, Boffetta P. Indoor air pollution from solid fuels and risk of hypopharyngeal/laryngeal and lung cancers: a multicentric case-control study from India. Int J Epidemiol. 2008; 37(2): 321–8.
148	Sasco AJ, Merrill RM, Dari I, Benhaïm-Luzon V, Carriot F, Cann CI, Bartal M. A case-control study of lung cancer in Casablanca, Morocco. Cancer Causes Control. 2002; 13(7): 609–16.
149	Savitha MR, Nandeeshwara SB, Pradeep Kumar MJ, ul-Haque F, Raju CK. Modifiable risk factors for acute lower respiratory tract infections. Indian J Pediatr. 2007; 74(5): 477–82.
150	Savitz DA, Bobb JF, Carr JL, Clougherty JE, Dominici F, Elston B, Ito K, Ross Z, Yee M, Matte TD. Ambient Fine Particulate Matter, Nitrogen Dioxide, and Term Birth Weight in New York, New York. Am J Epidemiol. 2014; 179(4): 457–66.
151	Schembari A, de Hoogh K, Pedersen M, Dadvand P, Martinez D, Hoek G, Petherick ES, Wright J, Nieuwenhuijsen MJ. Ambient Air Pollution and Newborn Size and Adiposity at Birth: Differences by Maternal Ethnicity (the Born in Bradford Study Cohort). Environ Health Perspect. 2015; 123(11): 1208-15.
152	Sezer H, Akkurt I, Guler N, Marakoglu K, Berk S. A case-control study on the effect of exposure to different substances on the development of COPD. Ann Epidemiol. 2006; 16(1): 59–62.
153	Shah N, Ramankutty V, Premila PG, Sathy N. Risk factors for severe pneumonia in children in south Kerala: a hospital-based case-control study. J Trop Pediatr. 1994; 40(4): 201–6.
154	Shang L, Huang L, Yang L, Leng L, Qi C, Xie G, Wang R, Guo L, Yang W, Chung MC. Impact of air pollution exposure during various periods of pregnancy on term birth weight: a large-sample, retrospective population-based cohort study. Environ Sci Pollut Res Int. 2021; 28(3): 3296-3306.
155	Shen M, Chapman RS, Vermeulen R, Tian L, Zheng T, Chen BE, Engels EA, He X, Blair A, Lan Q. Coal use, stove improvement, and adult pneumonia mortality in Xuanwei, China: a retrospective cohort study. Environ Health Perspect. 2009; 117(2): 261–6.
156	Sheridan P, Ilango S, Bruckner TA, Wang Q, Basu R, Benmarhnia T. Ambient Fine Particulate Matter and Preterm Birth in California: Identification of Critical Exposure Windows. Am J Epidemiol. 2019; 188(9): 1608-1615.
157	Siddiqui AR, Gold EB, Yang X, Lee K, Brown KH, Bhutta ZA. Prenatal exposure to wood fuel smoke and low birth weight. Environ Health Perspect. 2008; 116(4): 543-9.

158	Smith KR, McCracken JP, Weber MW, Hubbard A, Jenny A, Thompson LM, Balmes J, Diaz A, Arana B, Bruce N. Effect of reduction in household air pollution on childhood pneumonia in Guatemala (RESPIRE): a randomised controlled trial. Lancet. 2011; 378(9804): 1717-26.
159	Smith RB, Fecht D, Gulliver J, Beevers SD, Dajnak D, Blangiardo M, Ghosh RE, Hansell AL, Kelly FJ, Anderson HR, Toledano MB. Impact of London's road traffic air and noise pollution on birth weight: retrospective population based cohort study. BMJ. 2017; 359: j5299.
160	Stafoggia M, Cesaroni G, Peters A, Andersen ZJ, Badaloni C, Beelen R, Caracciolo B, Cyrys J, de Faire U, de Hoogh K, Eriksen KT, Fratiglioni L, Galassi C, Gigante B, Havulinna AS, Hennig F, Hilding A, Hoek G, Hoffmann B, Houthuijs D, Korek M, Lanki T, Leander K, Magnusson PK, Meisinger C, Migliore E, Overvad K, Ostenson C-G, Pedersen NL, Pekkanen J, Penell J, Pershagen G, Pundt N, Pyko A, Raaschou-Nielsen O, Ranzi A, Ricceri F, Sacerdote C, Swart WJR, Turunen AW, Vineis P, Weimar C, Weinmayr G, Wolf K, Brunekreef B, Forastiere F. Long-term exposure to ambient air pollution and incidence of cerebrovascular events: results from 11 European cohorts within the ESCAPE project. Environ Health Perspect. 2014; 122(9): 919–25.
161	Starling AP, Moore BF, Thomas DSK, Peel JL, Zhang W, Adgate JL, Magzamen S, Martenies SE, Allshouse WB, Dabelea D. Prenatal exposure to traffic and ambient air pollution and infant weight and adiposity: The Healthy Start study. Environ Res. 2020; 182: 109130.
162	Stieb DM, Chen L, Beckerman BS, Jerrett M, Crouse DL, Omariba DW, Peters PA, van Donkelaar A, Martin RV, Burnett RT, Gilbert NL, Tjepkema M, Liu S, Dugandzic RM. Associations of Pregnancy Outcomes and PM2.5 in a National Canadian Study. Environ Health Perspect. 2016; 124(2): 243-9.
163	Strickland MJ, Lin Y, Darrow LA, Warren JL, Mulholland JA, Chang HH. Associations Between Ambient Air Pollutant Concentrations and Birth Weight: A Quantile Regression Analysis. Epidemiology. 2019; 30(5): 624-632.
164	Sun Z, Yang L, Bai X, Du W, Shen G, Fei J, Wang Y, Chen A, Chen Y, Zhao M. Maternal ambient air pollution exposure with spatial-temporal variations and preterm birth risk assessment during 2013-2017 in Zhejiang Province, China. Environ Int. 2019; 133(Pt B): 105242.
165	Tapia VL, Vasquez BV, Vu B, Liu Y, Steenland K, Gonzales GF. Association between maternal exposure to particulate matter (PM2.5) and adverse pregnancy outcomes in Lima, Peru. J Expo Sci Environ Epidemiol. 2020; 30(4): 689-697.
166	Thompson LM, Bruce N, Eskenazi B, Diaz A, Pope D, Smith KR. Impact of reduced maternal exposures to wood smoke from an introduced chimney stove on newborn birth weight in rural Guatemala. Environ Health Perspect. 2011; 119(10): 1489-94.
167	Thurston GD, Ahn J, Cromar KR, Shao Y, Reynolds HR, Jerrett M, Lim CC, Shanley R, Park Y, Hayes RB. Ambient Particulate Matter Air Pollution Exposure and Mortality in the NIH-AARP Diet and Health Cohort [Unpublished]. Environ Health Perspect. 2016; 124(4): 484-90.
168	Tielsch JM, Katz J, Thulasiraj RD, Coles CL, Sheeladevi S, Yanik EL, Rahmathullah L. Exposure to indoor biomass fuel and tobacco smoke and risk of adverse reproductive

	outcomes, mortality, respiratory morbidity and growth among newborn infants in south India. Int J Epidemiol. 2009; 38(5): 1351-63.
169	To T, Zhu J, Villeneuve PJ, Simatovic J, Feldman L, Gao C, Williams D, Chen H, Weichenthal S, Wall C, Miller AB. Chronic disease prevalence in women and air pollution--A 30-year longitudinal cohort study. Environ Int. 2015; 80: 26–32.
170	Tseng E, Ho W-C, Lin M-H, Cheng T-J, Chen P-C, Lin H-H. Chronic exposure to particulate matter and risk of cardiovascular mortality: cohort study from Taiwan. BMC Public Health. 2015; 15: 936.
171	Turner MC, Jerrett M, Pope CA 3rd, Krewski D, Gapstur SM, Diver WR, Beckerman BS, Marshall JD, Su J, Crouse DL, Burnett RT. Long-term ozone exposure and mortality in a large prospective study. Am J Respir Crit Care Med. 2016; 193(10): 1134-42.
172	Turner MC, Krewski D, Pope CA, et al. 2011. Long-term ambient fine particulate matter air pollution and lung cancer in a large cohort of never-smokers. American Journal of Respiratory and Critical Care Medicine, 184(12), 1374-1381.
173	Victora CG, Fuchs SC, Flores JA, Fonseca W, Kirkwood B. Risk factors for pneumonia among children in a Brazilian metropolitan area. Pediatrics. 1994; 977-85.
174	Villeneuve PJ, Weichenthal SA, Crouse D, Miller AB, To T, Martin RV, van Donkelaar A, Wall C, Burnett RT. Long-term exposure to fine particulate matter air pollution and mortality among Canadian women. Epidemiology. 2015; 26(4): 536-45.
175	Wang Q, Benmarhnia T, Zhang H, Knibbs LD, Sheridan P, Li C, Bao J, Ren M, Wang S, H e Y, Zhang Y, Zhao Q, Huang C. Identifying windows of susceptibility for maternal exposure to ambient air pollution and preterm birth. Environ Int. 2018; 121(Pt 1): 317-324.
176	Wayse V, Yousafzai A, Mogale K, Filteau S. Association of subclinical vitamin D deficiency with severe acute lower respiratory infection in Indian children under 5 y. Eur J Clin Nutr. 2004; 58(4): 563–7.
177	Weichenthal S, Villeneuve PJ, Burnett RT, van Donkelaar A, Martin RV, Jones RR, DellaValle CT, Sandler DP, Ward MH, Hoppin JA. Long-term exposure to fine particulate matter: association with nonaccidental and cardiovascular mortality in the agricultural health study cohort. Environ Health Perspect. 2014; 122(6): 609-15.
178	Weinmayr G, Hennig F, Fuks K, Nonnemacher M, Jakobs H, Möhlenkamp S, Erbel R, Jöckel K-H, Hoffmann B, Moebus S, Heinz Nixdorf Recall Investigator Group. Long-term exposure to fine particulate matter and incidence of type 2 diabetes mellitus in a cohort study: effects of total and traffic-specific air pollution. Environ Health. 2015; 14: 53.
179	Wesley AG, Loening WE. Assessment and 2-year follow-up of some factors associated with severity of respiratory infections in early childhood. S Afr Med J. 1996; 86(4): 365–8.
180	Wilhelm M, Ghosh JK, Su J, Cockburn M, Jerrett M, Ritz B. Traffic-related air toxics and preterm birth: a population-based case-control study in Los Angeles County, California. Environ Health. 2011; 10: 89.
181	Wong CM, Lai HK, Tsang H, Thach TQ, Thomas GN, Lam KBH, Chan KP, Yang L, Lau AKH, Ayres JG, Lee SY, Man Chan W, Hedley AJ, Lam TH. Satellite-Based Estimates of

	Long-Term Exposure to Fine Particles and Association with Mortality in Elderly Hong Kong Residents. Environ Health Perspect. 2015; 123(11): 1167-72.
182	Wu AH, Henderson BE, Pike MC, Yu MC. Smoking and other risk factors for lung cancer in women. J Natl Cancer Inst. 1985; 74(4): 747-51.
183	Wu J, Wilhelm M, Chung J, Ritz B. Comparing exposure assessment methods for traffic-related air pollution in an adverse pregnancy outcome study. Environ Res. 2011; 111(5): 685-92.
184	Wu H, Jiang B, Geng X, Zhu P, Liu Z, Cui L, Yang L. Exposure to fine particulate matter during pregnancy and risk of term low birth weight in Jinan, China, 2014-2016. Int J Hyg Environ Health. 2018; 221(2): 183-190.
185	Wylie BJ, Coull BA, Hamer DH, Singh MP, Jack D, Yeboah-Antwi K, Sabin L, Singh N, MacLeod WB. Impact of biomass fuels on pregnancy outcomes in central East India. Environ Health. 2014; 13(1): 1.
186	Wylie BJ, Kishashu Y, Matechi E, Zhou Z, Coull B, Abioye AI, Dionisio KL, Mugusi F, Premji Z, Fawzi W, Hauser R, Ezzati M. Maternal exposure to carbon monoxide and fine particulate matter during pregnancy in an urban Tanzanian cohort. Indoor Air. 2017; 27(1): 136-146.
187	Xiao Q, Chen H, Strickland MJ, Kan H, Chang HH, Klein M, Yang C, Meng X, Liu Y. Associations between birth outcomes and maternal PM2.5 exposure in Shanghai: A comparison of three exposure assessment approaches. Environ Int. 2018; 117: 226-236.
188	Ye L, Ji Y, Lv W, Zhu Y, Lu C, Xu B, Xia Y. Associations between maternal exposure to ai r pollution and birth outcomes: a retrospective cohort study in Taizhou, China. Enviro n Sci Pollut Res Int. 2018; 25(22): 21927-21936.
189	Yin P, Brauer M, Cohen A, Burnett RT, Liu J, Liu Y, Liang R, Wang W, Qi J, Wang L, Zhou M. Long-term Fine Particulate Matter Exposure and Nonaccidental and Cause-specific Mortality in a Large National Cohort of Chinese Men [Unpublished]. Environ Health Perspect. 2017; 125(11): 117002.
190	Yin P, Brauer M, Cohen A, Burnett RT, Liu J, Liu Y, Liang R, Wang W, Qi J, Wang L, Zhou M. Long-term Fine Particulate Matter Exposure and Nonaccidental and Cause-specific Mortality in a Large National Cohort of Chinese Men. Environ Health Perspect. 2017; 125(11): 117002.
191	Yu K, Qiu G, Chan K-H, Lam K-BH, Kurmi OP, Bennett DA, Yu C, Pan A, Lv J, Guo Y, Bian Z, Yang L, Chen Y, Hu FB, Chen Z, Li L, Wu T. Association of Solid Fuel Use With Risk of Cardiovascular and All-Cause Mortality in Rural China. JAMA. 2018; 319(13): 1351–61.
192	Yuan L, Zhang Y, Wang W, Chen R, Liu Y, Liu C, Kan H, Gao Y, Tian Y, Shanghai Birth Cohort Study. Critical windows for maternal fine particulate matter exposure and adverse birth outcomes: The Shanghai birth cohort study. Chemosphere. 2020; 240: 124904.
193	Yucra S, Tapia V, Steenland K, Naeher LP, Gonzales GF. Association between biofuel exposure and adverse birth outcomes at high altitudes in Peru: a matched case-control study. Int J Occup Environ Health. 2011; 17(4): 307-13.

The following figures display risk curves for each outcome. The dashed line depicts the GBD 2017 IER including active smoking data, the dotted line depicts the GBD 2019 MR-BRT curve without active smoking but with secondhand smoking data, and the solid line depicts the GBD 2021 MR-BRT curve without the inclusion of active smoking or secondhand smoking data. For GBD 2021, a single curve is used for cardiovascular diseases (ischaemic heart disease, stroke) for all ages, so only one plot is displayed for each of these outcomes. For the GBD 2017 and GBD 2021 curves, the curve for the age group 60–64 is plotted for the cardiovascular disease outcomes because these cycles used age-specific cardiovascular disease curves. For birthweight and gestational age, no curve is displayed for GBD 2017 because these outcomes were added to the GBD in the 2019 cycle. The grey shaded areas represent the 95% CI. The red box represents the TMREL area of the curve. On each page, the first figure depicts the typical range of outdoor exposure, whereas the second plot includes higher levels typical of household air pollution exposure.

Each point or number represents one study effect size. Each is plotted at the 95th percentile of the exposure distribution (OAP) or the expected level of exposure for individual using solid fuel (HAP). The relative risk is plotted relative to the predicted relative risk at the 5th percentile of exposure distribution (OAP) or the expected (ambient only) level of exposure for individuals not using solid fuel (HAP). For example, a study predicting a relative risk of 1.5 for an exposure range of 10 to 20 would be plotted at (20, MRBRT(10)*1.5). Arrows represent studies that would have been outside the range of the plot but have been shifted to be included in the figure.

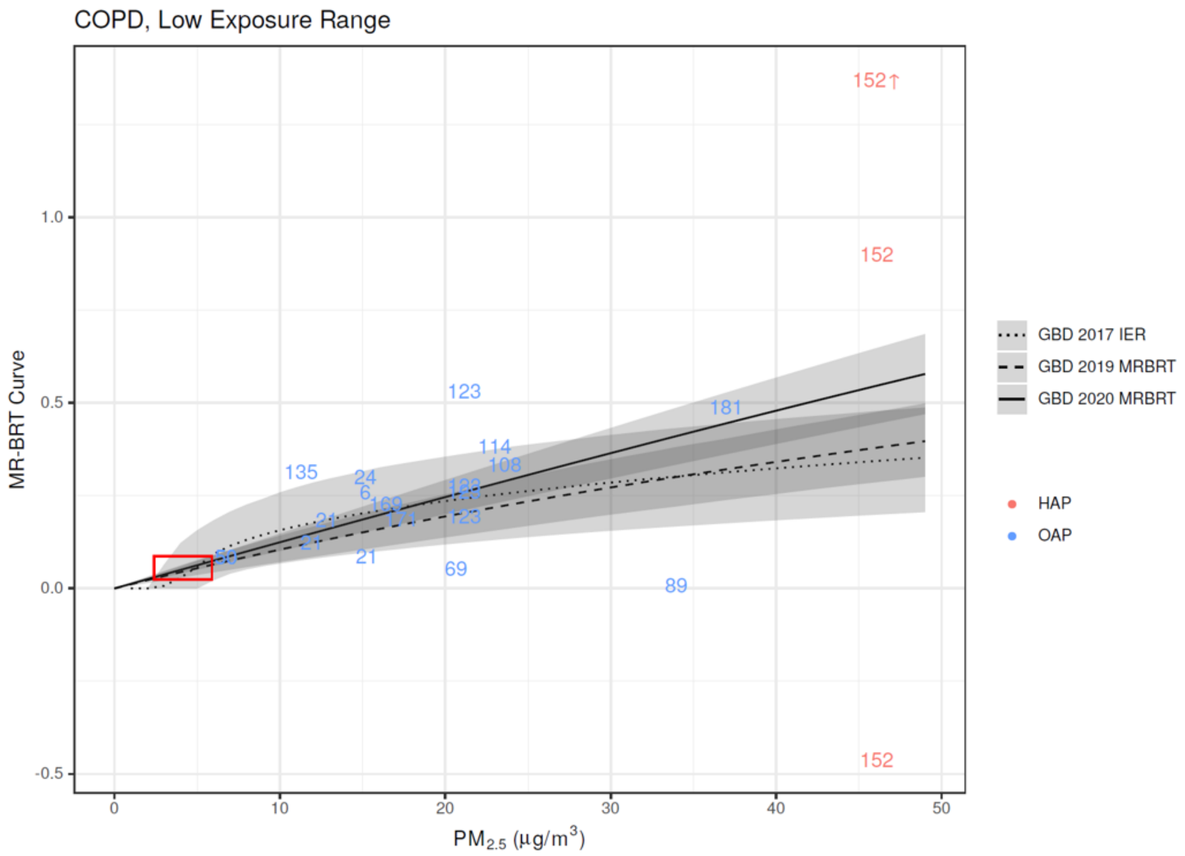
COPD, Low Exposure Range
MR-BRT Curve
PM2.5 (µg/m3)
GBD 2017 IER
GBD 2019 MRBRT
GBD 2020 MRBRT
HAP
OAP

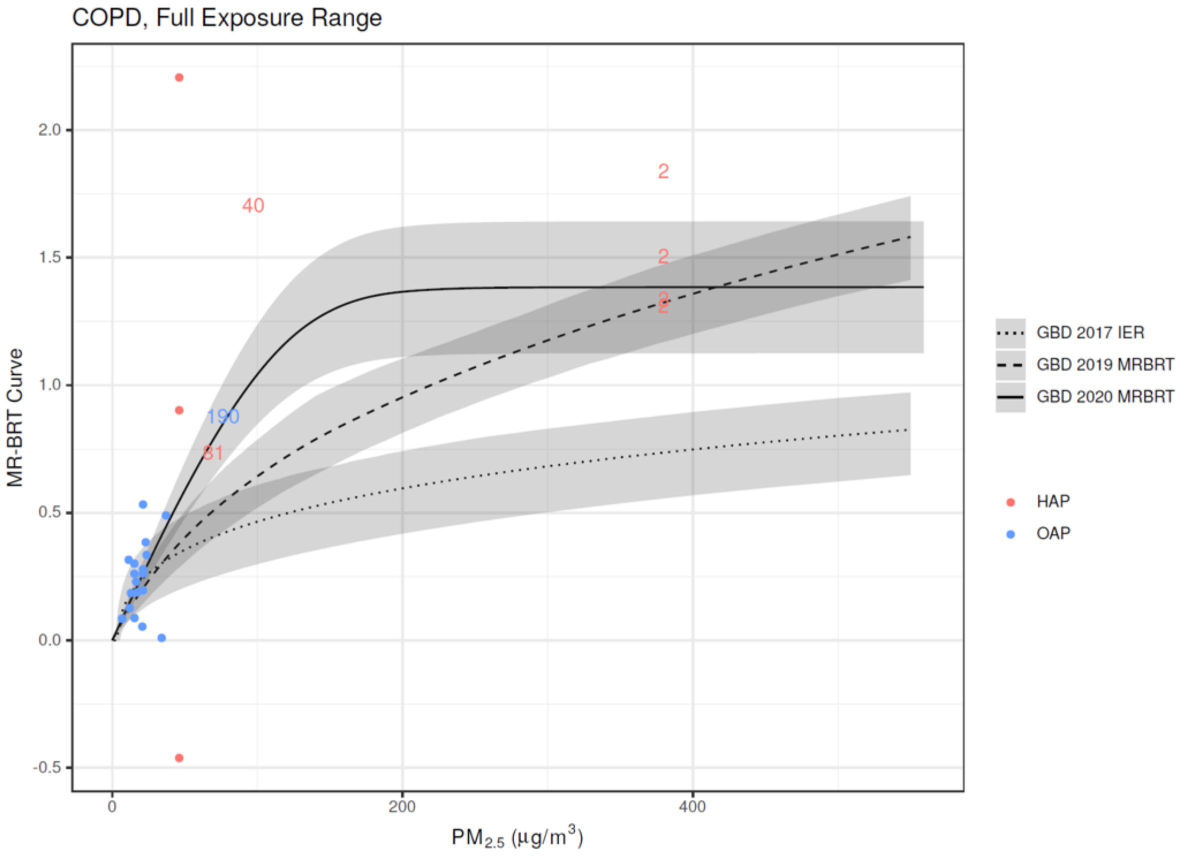
COPD, Full Exposure Range
MR-BRT Curve
PM2.5 (µg/m3)
GBD 2017 IER
GBD 2019 MRBRT
GBD 2020 MRBRT
HAP
OAP

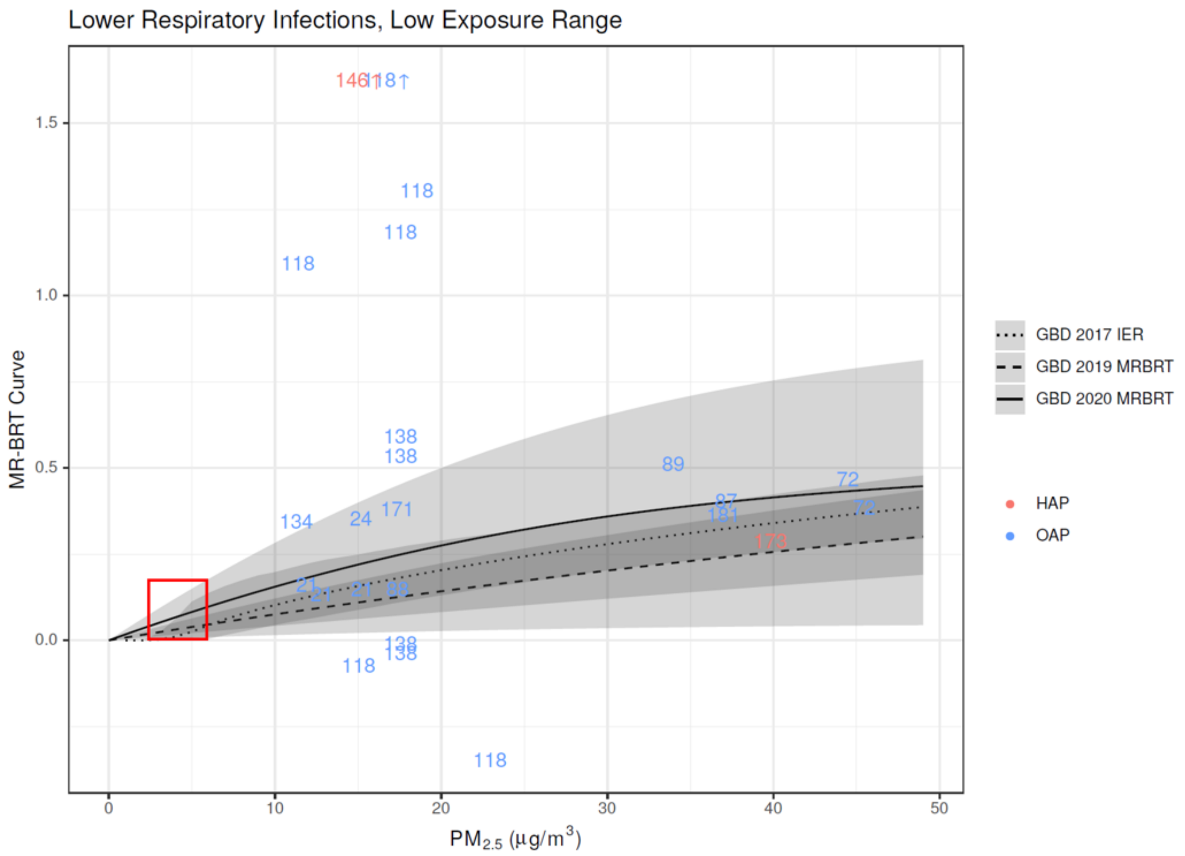
Lower Respiratory Infections, Low Exposure Range
MR-BRT Curve
1.5
1.0
0.5
0.0
0
10
20
30
40
50
PM2.5 (µg/m3)
GBD 2017 IER
GBD 2019 MRBRT
GBD 2020 MRBRT
HAP
OAP

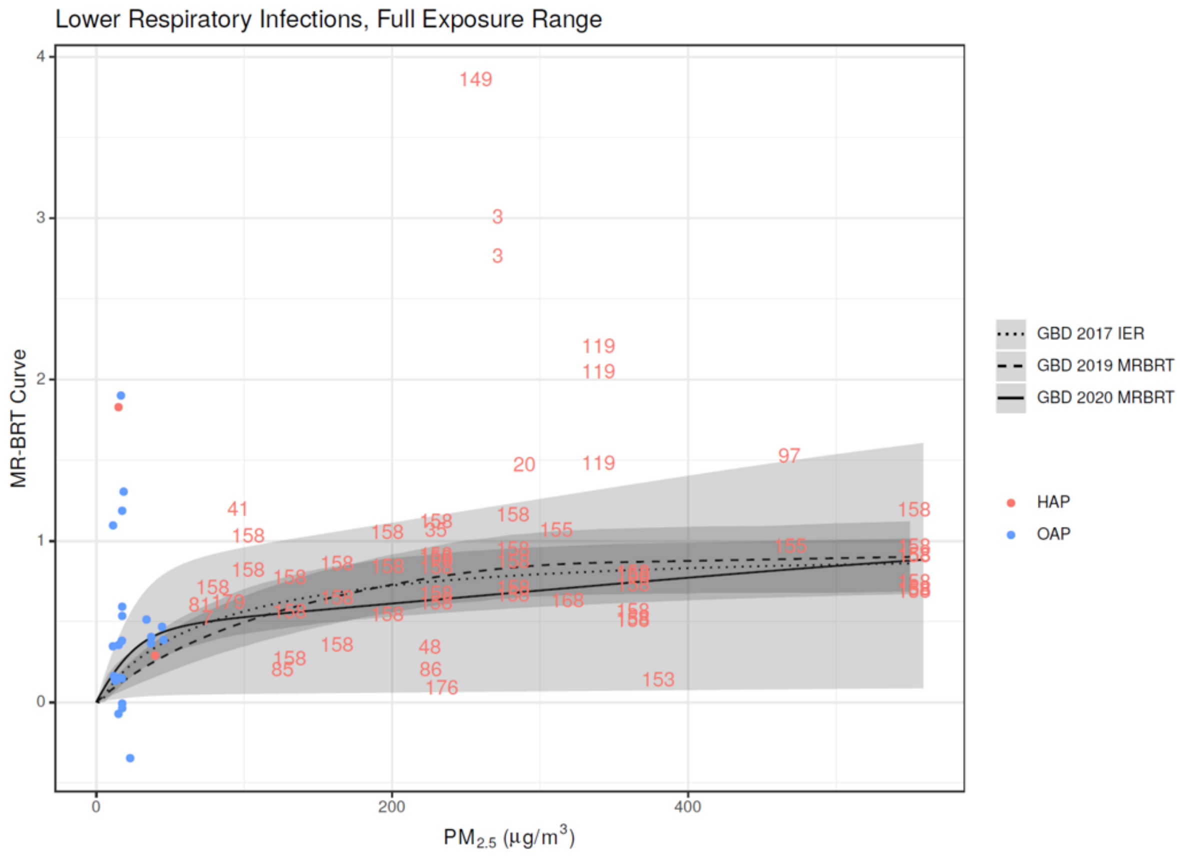
Lower Respiratory Infections, Full Exposure Range
MR-BRT Curve
4
3
2
1
0
0
200
400
PM2.5 (µg/m3)
GBD 2017 IER
GBD 2019 MRBRT
GBD 2020 MRBRT
HAP
OAP

Lung Cancer, Low Exposure Range

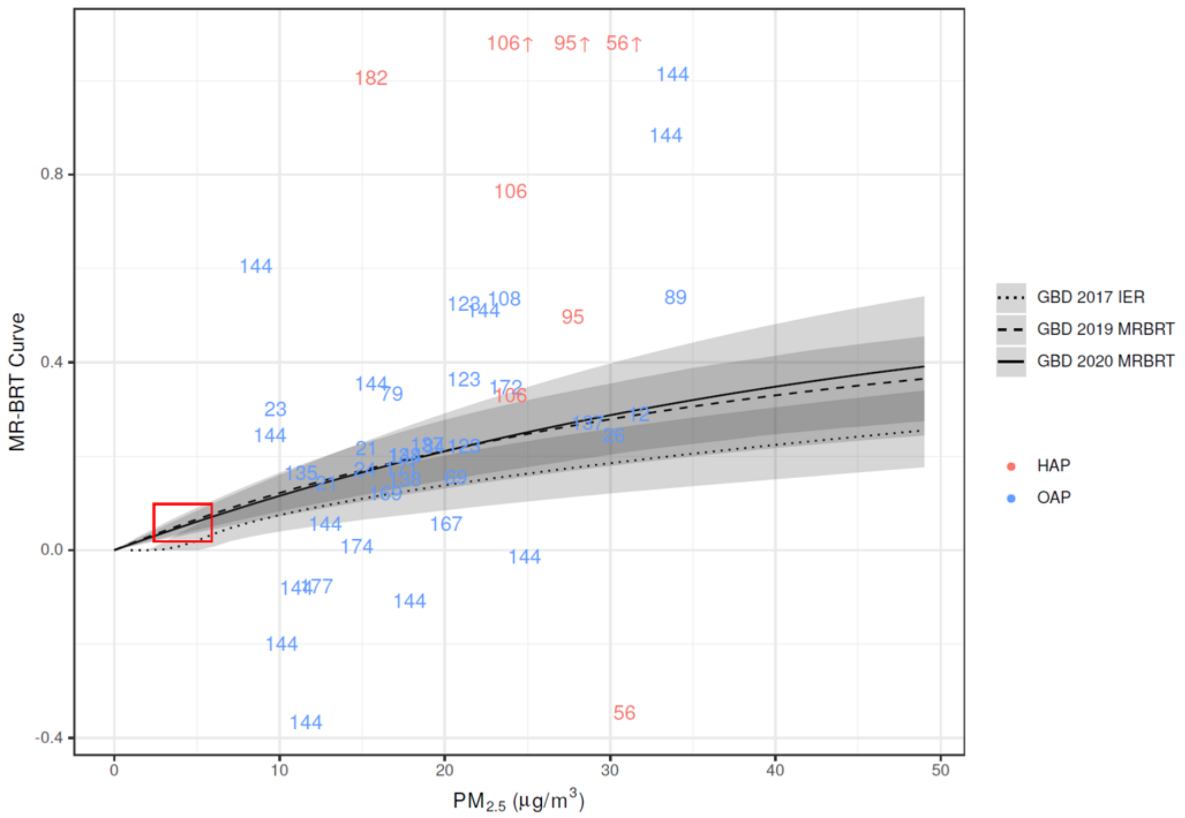

Lung Cancer, Full Exposure Range

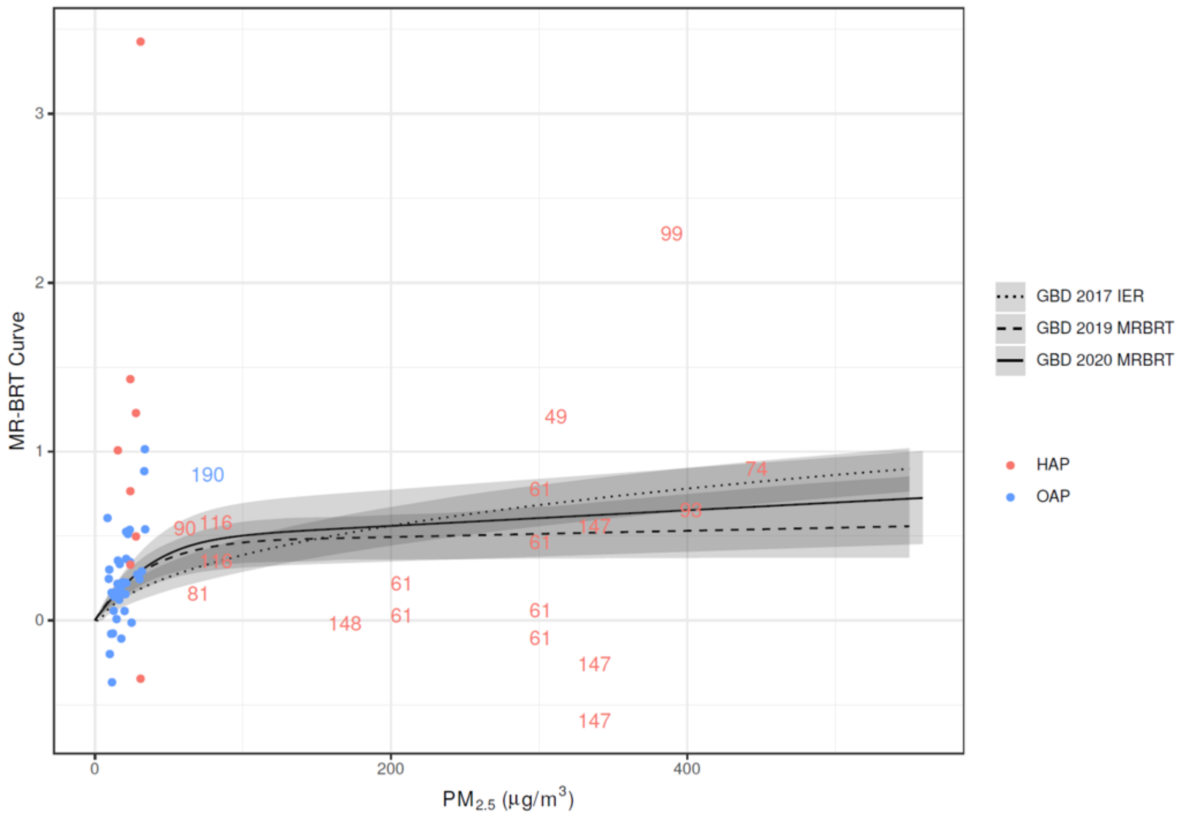

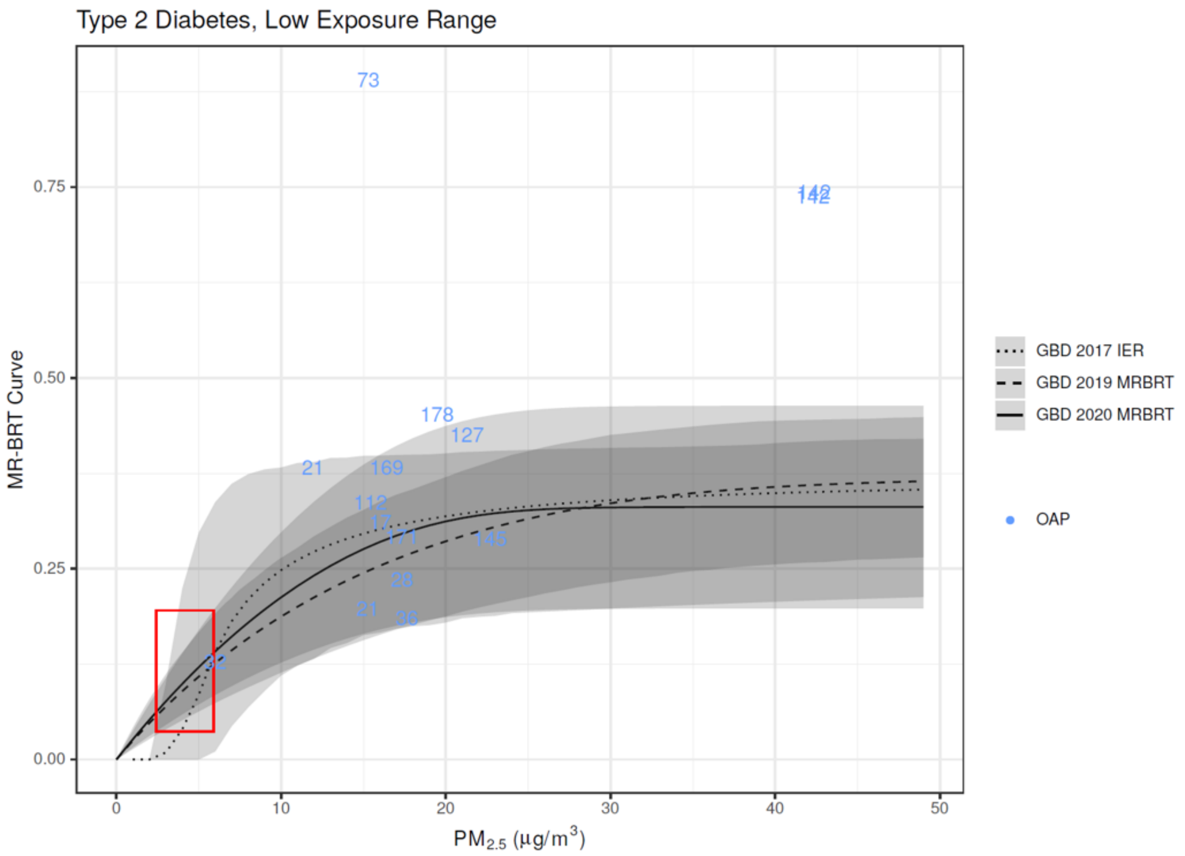

Type 2 Diabetes, Low Exposure Range
MR-BRT Curve
PM2.5 (µg/m3)
GBD 2017 IER
GBD 2019 MRBRT
GBD 2020 MRBRT
OAP

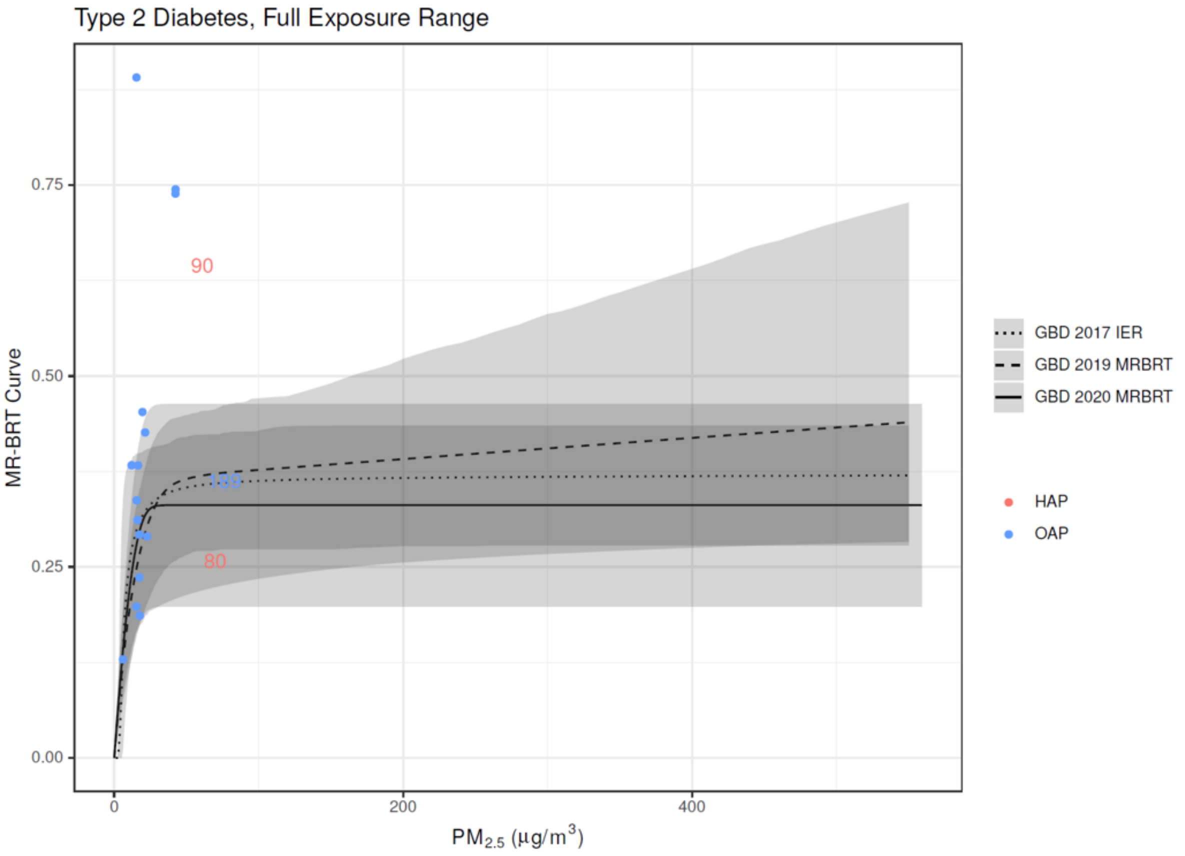

Type 2 Diabetes, Full Exposure Range
MR-BRT Curve
PM2.5 (µg/m3)
GBD 2017 IER
GBD 2019 MRBRT
GBD 2020 MRBRT
HAP
OAP

Ischemic Heart Disease, Low Exposure Range

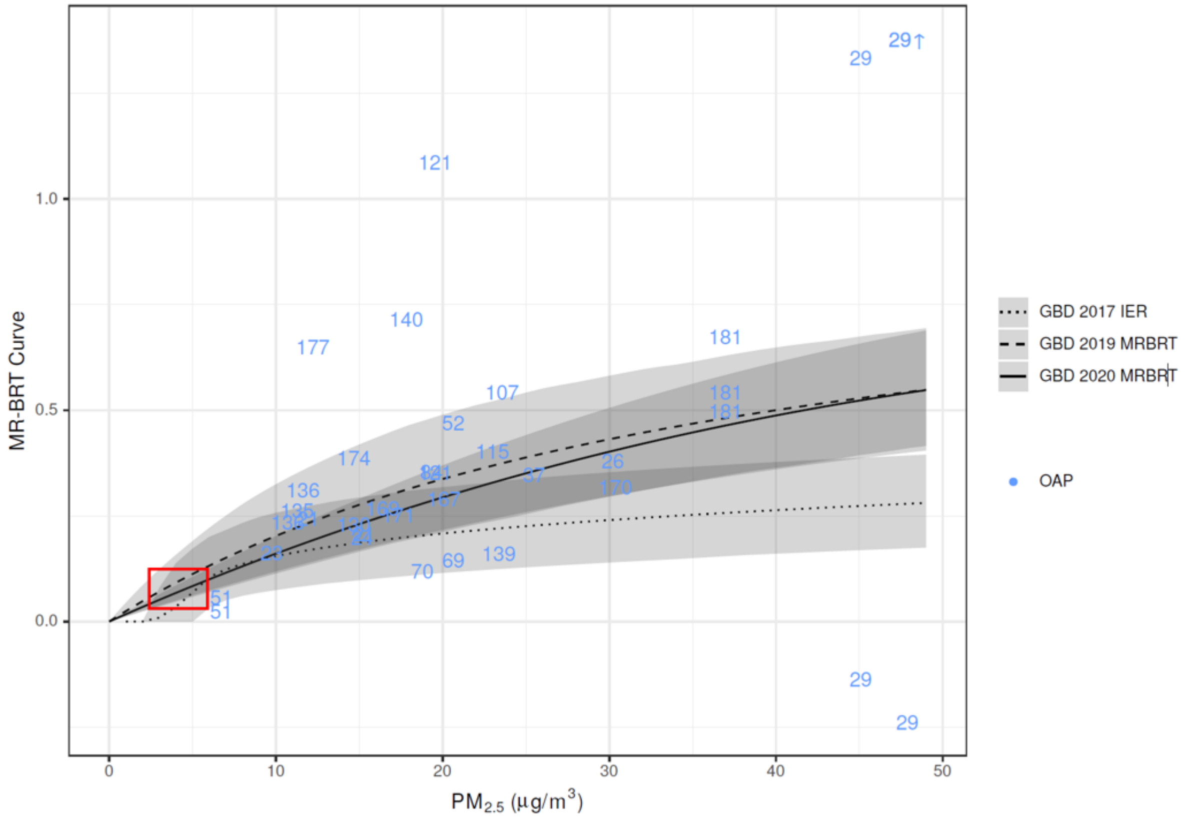

Ischemic Heart Disease, Full Exposure Range

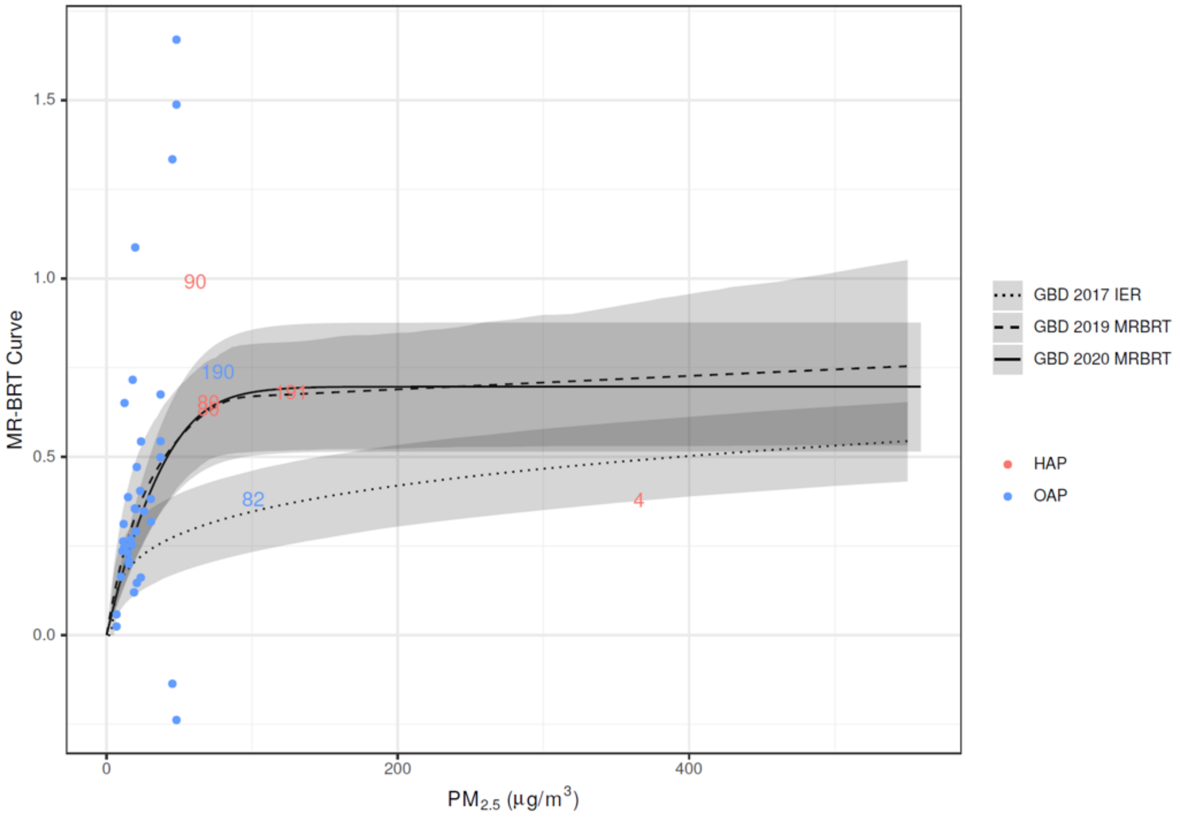

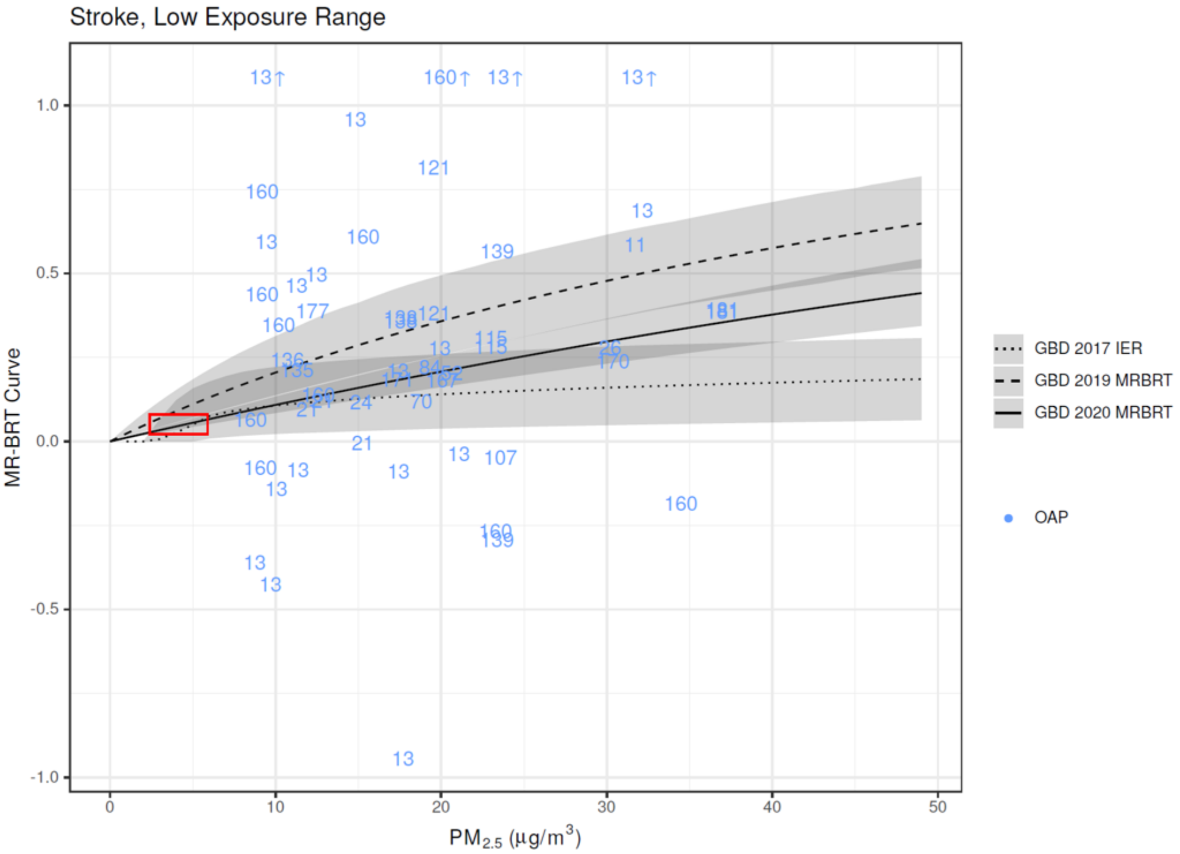
Stroke, Low Exposure Range
MR-BRT Curve
PM2.5 (μg/m3)
GBD 2017 IER
GBD 2019 MRBRT
GBD 2020 MRBRT
OAP

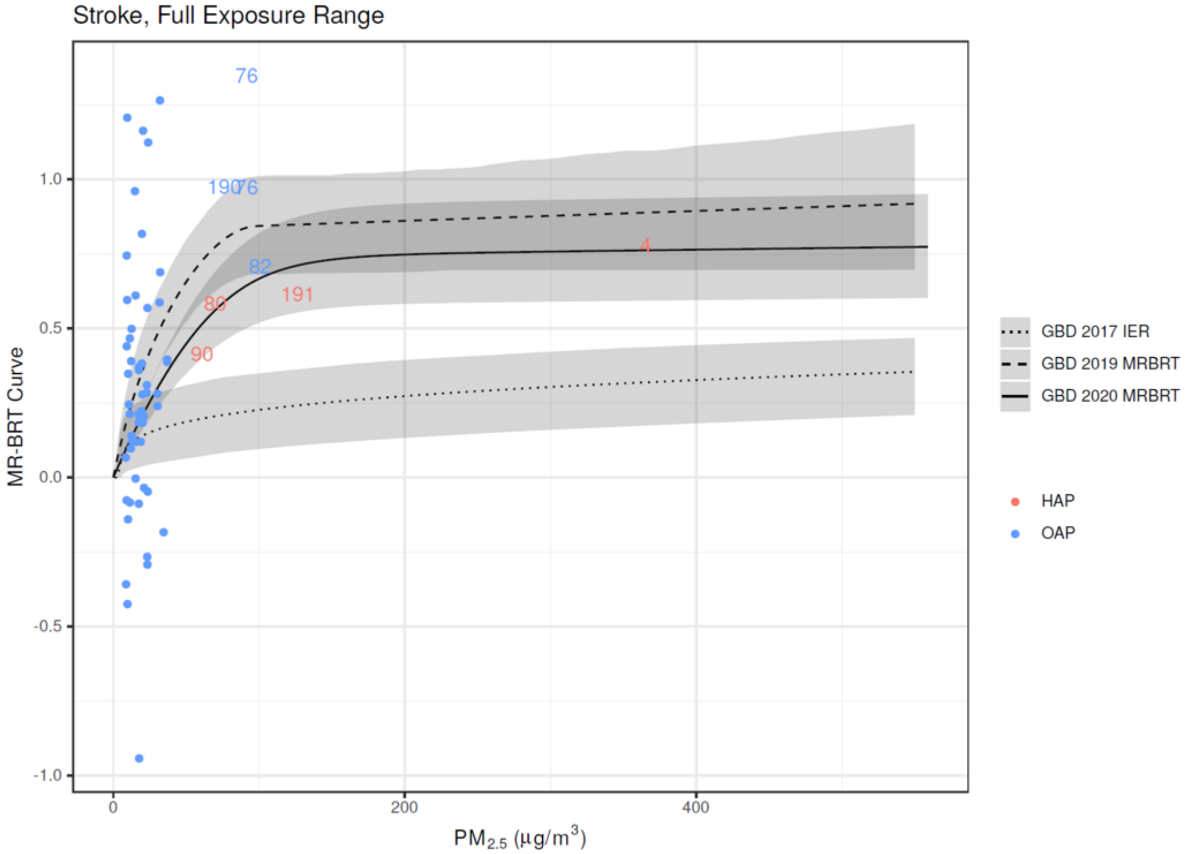
Stroke, Full Exposure Range
MR-BRT Curve
PM2.5 (μg/m3)
GBD 2017 IER
GBD 2019 MRBRT
GBD 2020 MRBRT
HAP
OAP

Low birthweight and short gestation mediation analysis

As in GBD 2019, in GBD 2021, low birthweight and short gestation were included as $PM_{2.5}$ outcomes via a mediation analysis. Low birthweight and short gestation includes mortality due to diarrhoeal diseases, lower respiratory infections, upper respiratory infections, otitis media, meningitis, encephalitis, neonatal preterm birth, neonatal encephalopathy due to birth asphyxia and trauma, neonatal sepsis and other neonatal infections, haemolytic disease and other neonatal jaundice, and other neonatal disorders. Morbidity estimates were also calculated for neonatal preterm birth. These outcomes are specific to the neonatal ages: 0–6 days and 7–27 days.

The following is a summary of methods used to conduct the mediation analysis. For GBD 2019, we conducted a systematic review of all cohort, case-control, or randomised-controlled trial studies of ambient $PM_{2.5}$ pollution or household air pollution and birthweight or gestational age outcomes for GBD 2019.[18] Outcomes measured included continuous birthweight (bw), continuous gestational age (ga), low birthweight (LBW) (<2500 g), preterm birth (PTB) (<37 weeks), and very preterm birth (VPTB) (<32 weeks). We included any papers published until April 4, 2021.

Birthweight and gestational age are modelled using a continuous joint distribution for the GBD. To determine how these distributions are influenced by $PM_{2.5}$ pollution, we used available literature to model the continuous shift in birthweight (bw, grams) and gestational age (ga, weeks) at a given $PM_{2.5}$ exposure level. When available, we used estimates of continuous shifts in bw or ga directly from each study. When shifts were not available, we converted the published OR/RR/HR for LBW, PTB, or VPTB using the following strategy:

1. Extract the OR/RR/HR from the study.
2. Select the GBD 2017 estimated bw-ga joint distribution for the study location and year.
3. Calculate the number of grams or weeks required to shift the distribution such that the proportion of births under the specified threshold (P) is reduced by the study effect size to a counterfactual level (P_{cf}).
4. Save the resulting shift and 95% CI as the continuous effect.

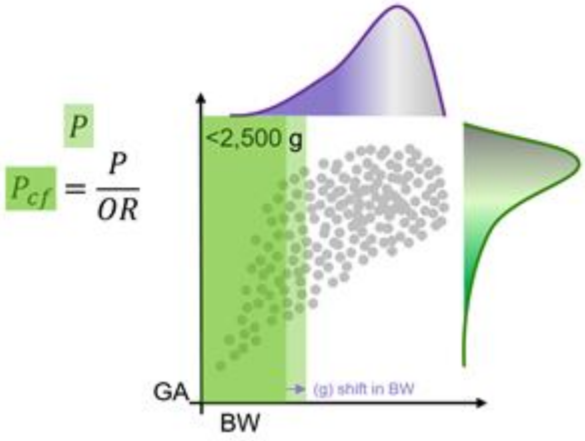

When preparing HAP data to fit splines, we used the same strategy described above for other outcomes to map HAP input data to $PM_{2.5}$ exposure values. We then fit MR-BRT splines to the input studies, where the difference in the value of the model at the upper concentration (X) and the value of the model at the counterfactual concentration (X_{CF}) is equal to the published or calculated shift in bw or ga:

$$MRBRT(X) - MRBRT(X_{CF}) \sim Shift$$

We used the same model fitting process, settings, and covariate selection process as described above for the other outcomes. The only exception is that, because the change in birthweight and gestational age was expected to be negative, the splines were constrained to be monotonically decreasing.

The following figures display MR-BRT curves for linear shift in grams (bw) and weeks (ga).

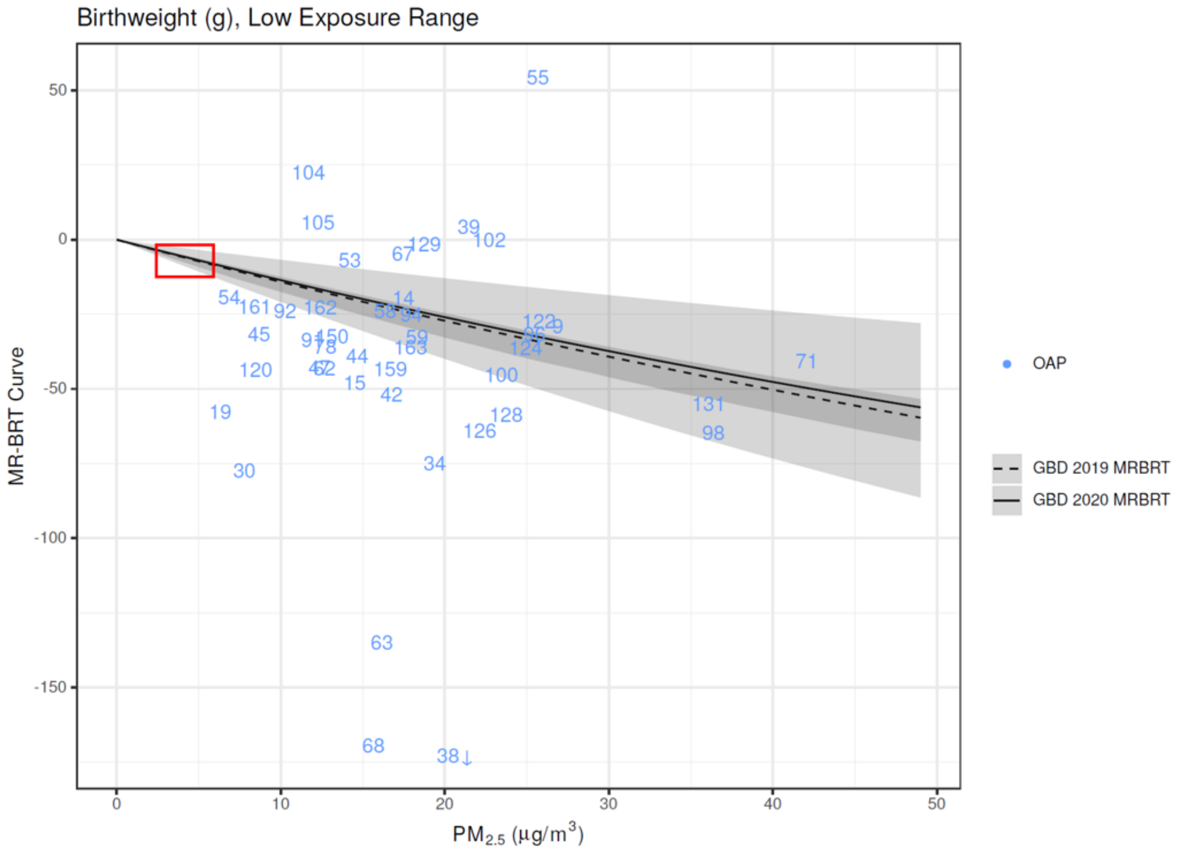
Birthweight (g), Low Exposure Range
MR-BRT Curve
PM2.5 (μg/m3)
OAP
GBD 2019 MRBRT
GBD 2020 MRBRT

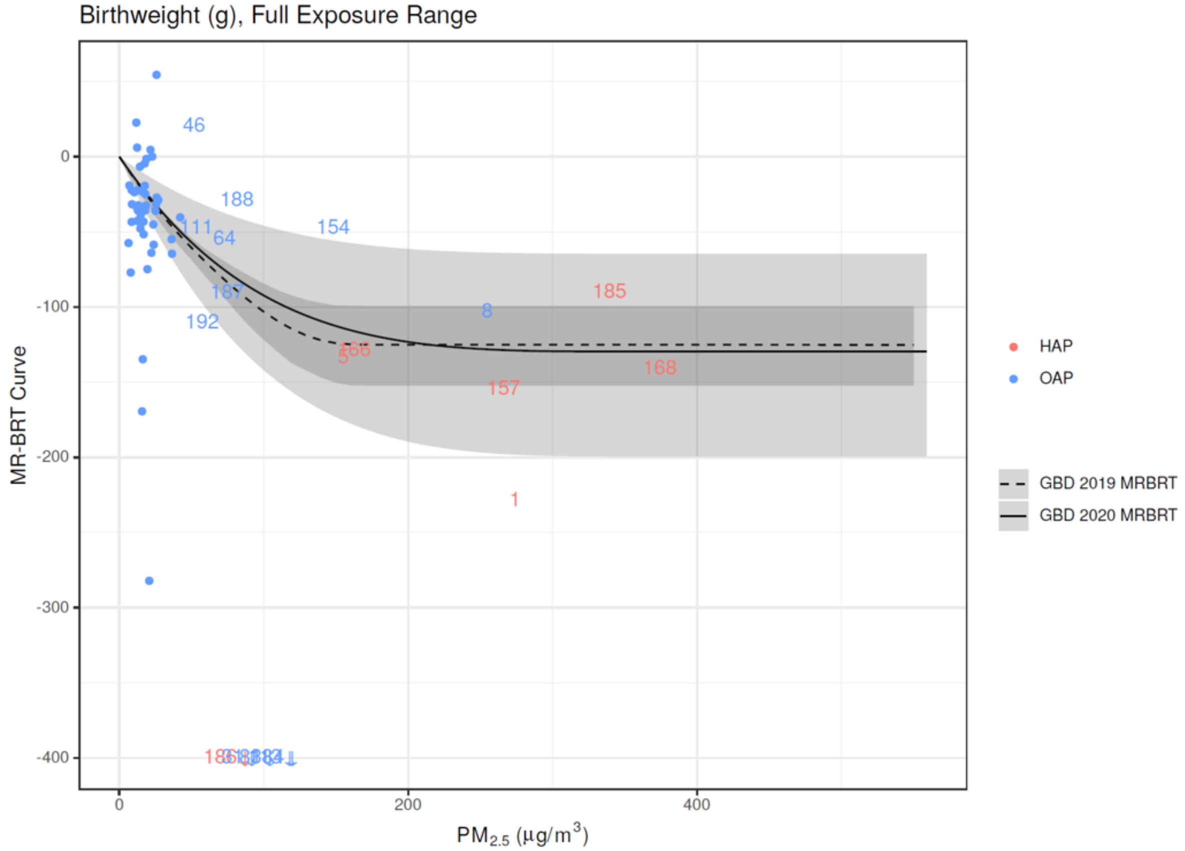
Birthweight (g), Full Exposure Range
MR-BRT Curve
PM2.5 (μg/m3)
HAP
OAP
GBD 2019 MRBRT
GBD 2020 MRBRT

Gestational Age (weeks), Low Exposure Range

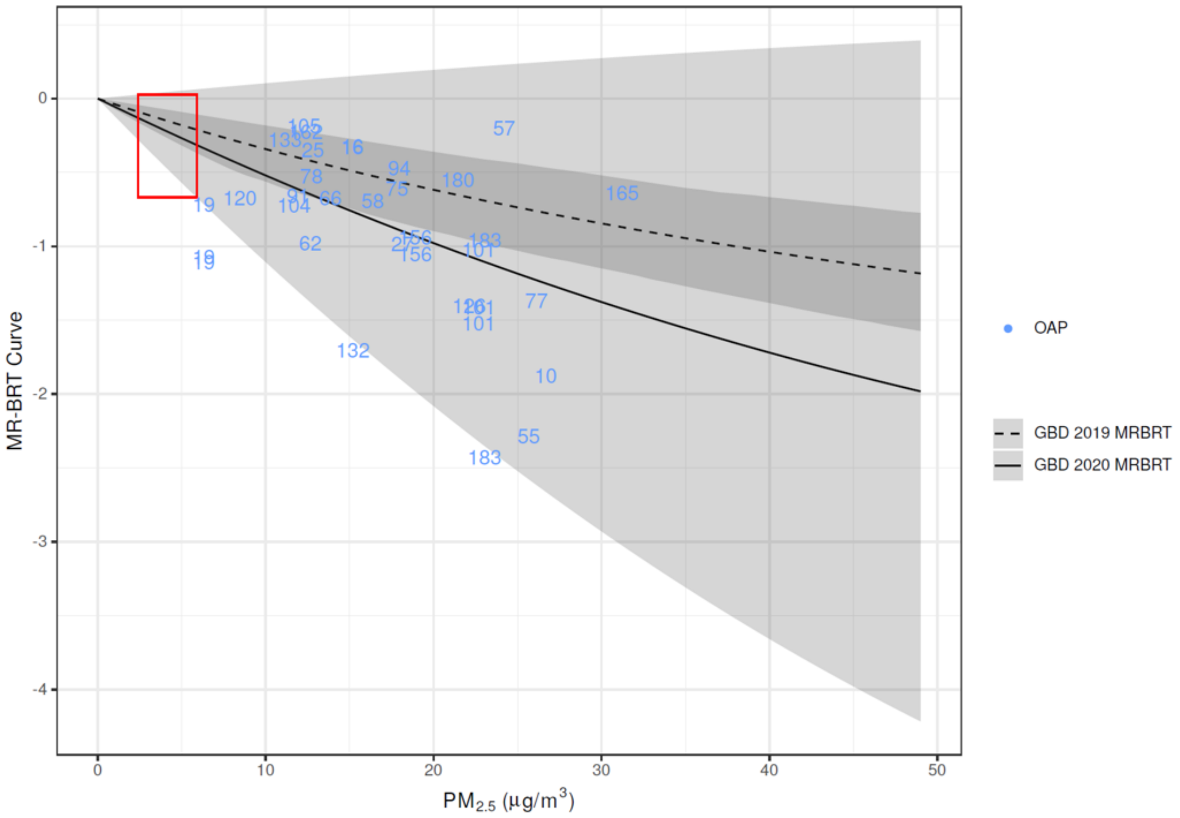

Gestational Age (weeks), Full Exposure Range

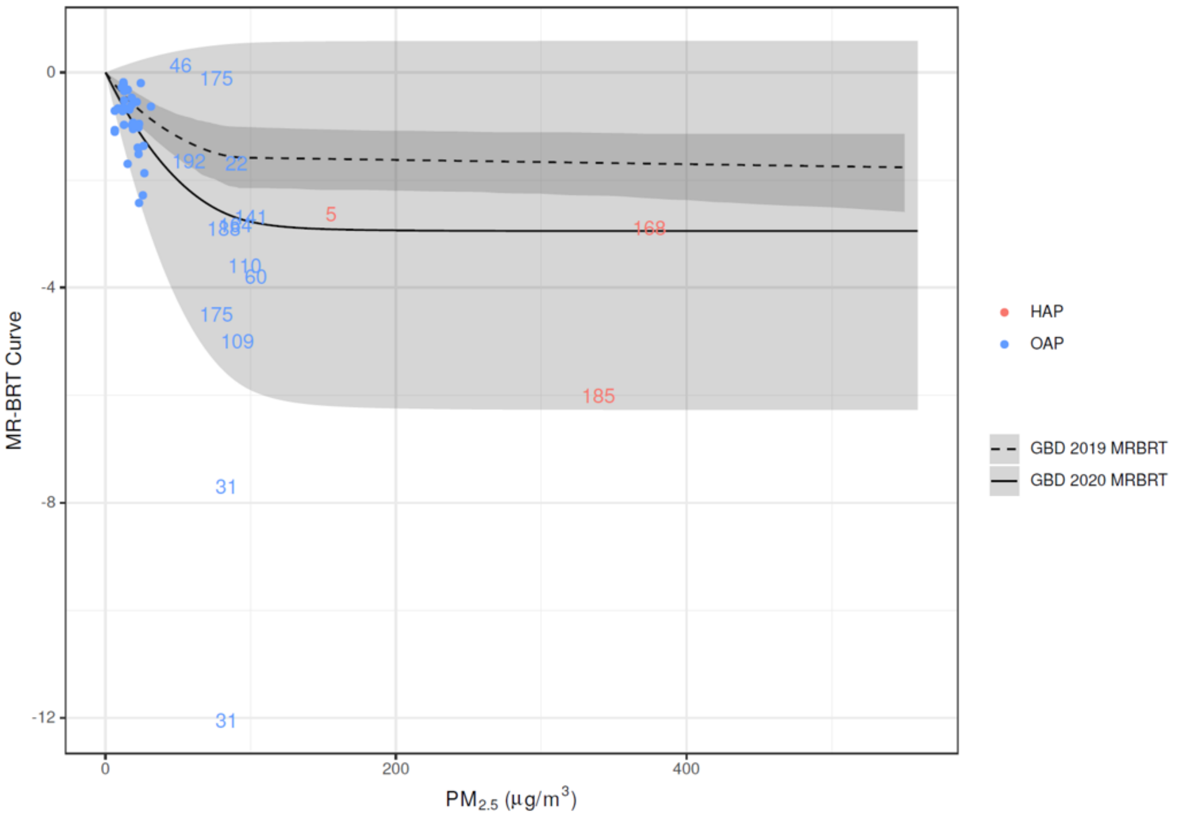

We used the curves of estimated shifts across the exposure range to predict the shift in both birthweight and gestational age for total female particulate matter pollution exposure in each location and year. Because the epidemiological studies mutually controlled for birthweight and gestational age, we assumed these shifts are independent. We then shifted the observed distributions to reflect the expected bwga distribution in the absence of particulate matter pollution. These shifted distributions were used as the counterfactual in the PAF calculation equation to calculate the burden attributable to $PM_{2.5}$ pollution.

To calculate PAFs, the distribution is divided into 56 bw-ga categories, each with a unique RR. Let p_i be the observed proportion of babies in category, *i* and p_i' be the counterfactual proportion of babies in category, *i* if there were no particulate matter pollution.

$$PAF_{PM} = \frac{\sum_{i \in bwga\ category} RR_i p_i - \sum_{i \in bwga\ category} RR_i p_i'}{\sum_{i \in bwga} RR_i p_i}$$

We proportionately split this PAF to ambient and HAP based on exposure as described below. One important assumption to note is that we assume the shift in bw and ga is linear across the bwga distribution.

For lower respiratory infections, $PM_{2.5}$-attributable PAFs are directly estimated in addition to estimated through bwga mediation. We expect that some of the directly estimated PAFs are mediated through bw and ga. Additionally, the directly estimated PAF is based on a summary of relative risks for all children under 5 years, so there is a possibility that the mediated PAF, which is more finely resolved, could be greater. To avoid double counting, for the two neonatal age groups (0–6 days and 0–27 days), we take the maximum of the two PAF estimates. If the directly estimated PAF is greater than the bwga-mediated PAF, we take the direct estimate, and if the mediated PAF is greater, we take the mediated estimate.

PTB incidence and mortality are both outcomes measured in the GBD. 100% of the burden for this cause is attributable to short gestation. To calculate the percentage attributable to particulate matter pollution, we estimated the percentage of babies born at less than 37 weeks (p_{ptb}) and the percentage of babies that would have been born at less than 37 weeks in the counterfactual scenario of no particulate matter pollution (p_{ptb}').

$$PAF_{ptb,pm} = 1 - \frac{p_{ptb}'}{p_{ptb}}$$

Limitations

Although for GBD 2021 we have not used active smoking or secondhand smoking data to estimate $PM_{2.5}$ risk curves, we still use an integrated exposure–response approach because we integrate relative risk estimates across ambient and HAP sources. The use of both source types to construct a risk curve with $PM_{2.5}$ as the exposure indicator assumes equitoxicity of particles regardless of source, despite evidence suggesting differences in health impacts by specific PM source (eg, motor vehicles, coal-fired power plant), size, and/or chemical composition. However, in the absence of sufficient estimates of source- or composition-specific exposure–response relationships and consistent and robust evidence of differential

toxicity by source, integrating across all OAP and HAP studies is the approach most consistent with the current evidence, as reviewed by USA EPA and WHO.[19,20]

Proportional PAF approach

Prior to GBD 2017, relative risks for both ambient and HAP exposures were obtained from the risk curve as a function of exposure, relative to the same TMREL. In reality, were a country to reduce only one of these risk factors, the other would remain. We did not consider the joint effects of particulate matter from outdoor exposure and burning solid fuels for cooking. For GBD 2017, we developed a new approach to use the risk curve for obtaining PAFs for both OAP and HAP, which was also implemented in GBD 2019 and 2021.

Let Exp_{OAP} be the ambient $PM_{2.5}$ exposure level and Exp_{HAP} be the excess exposure for those who use solid fuel for cooking. Let P_{HAP} be the proportion of the population using solid fuel for cooking. We calculated PAFs at each $0.1^{o} \times 0.1^{o}$ grid cell. We assumed that the distribution of those using solid fuel for cooking (HAP) was equivalent across all grid cells of the GBD location.

For the proportion of the population not exposed to HAP the relative risk was:

$$RR_{OAP} = MRBRT(z = Exp_{OAP})/MRBRT(z = TMREL),$$

And for those exposed to HAP, the relative risk was

$$RR_{HAP} = MRBRT(z = Exp_{OAP} + Exp_{HAP})/MRBRT(z = TMREL).$$

We then calculate a population-level RR and PAF for all particulate matter exposure:

$$RR_{PM} = RR_{OAP}(1 - P_{HAP}) + RR_{HAP}P_{HAP}$$

$$PAF_{PM} = \frac{RR_{PM} - 1}{RR_{PM}}$$

We population weight the grid-cell level particulate matter PAFs to get a country-level PAF, and finally, we split this PAF based on the average exposure to each OAP and HAP:

$$PAF_{OAP} = \frac{Exp_{OAP}}{Exp_{OAP}+P_{HAP}*Exp_{HAP}} PAF_{PM}, \text{ and } PAF_{HAP} = \frac{P_{HAP}*Exp_{HAP}}{Exp_{OAP}+P_{HAP}*Exp_{HAP}} PAF_{PM}.$$

With this strategy, $PAF_{PM} = PAF_{HAP} + PAF_{OAP}$, and no burden is counted twice.

References

1. Hammer M. S., van Donkelaar A., Li C., Lyapustin A., Sayer A. M., Hsu C. N., Levy R. C., Garay M. J., Kalashnikova O. V., Kahn R. A., Brauer M., Apte J. S., Henze D. K., Zhang L., Zhang Q., Ford B., Pierce J. R., Martin R. V. Global Estimates and Long-Term Trends of Fine Particulate Matter Concentrations (1998–2018). Environ. Sci. Technol. 2020; 54(13): 7879-7890. DOI: 10.1021/acs.est.0c01764

2. van Donkelaar, A.; Martin, R. V; Brauer, M.; Hsu, N. C.; Kahn, R. A.; Levy, R. C.; Lyapustin, A.; Sayer, A. M.; Winker, D. M. Global Estimates of Fine Particulate Matter using a Combined Geophysical-Statistical Method with Information from Satellites, Models, and Monitors. Environ. Sci. Technol. 2016, 50 (7), 3762–3772
3. Shaddick, G., Thomas, M.L., Jobling, A., Brauer, M., van Donkelaar, A., Burnett, R., Chang, H., Cohen, A., Van Dingenen, R., Dora, C. and Gumy, S., 2016. Data Integration Model for Air Quality: A Hierarchical Approach to the Global Estimation of Exposures to Ambient Air Pollution. Journal of Royal Statistical Society Series C (Applied Statistics). 2017. DOI: 10.1111/rssc.12227
4. Shaddick, G., Thomas, M. L., Mudu, P., Ruggeri, G. and Gumy, S. Half the world's population are exposed to increasing air pollution. Accepted by Nature Climate and Atmospheric Science.
5. Brauer, M.; Freedman, G.; Frostad, J.; van Donkelaar, A.; Martin, R. V; Dentener, F.; Van Dingenen, R.; Estep, K.; Amini, H.; Apte, J. S.; et al. Ambient Air Pollution Exposure Estimation for the Global Burden of Disease 2013. Environ. Sci. Technol. 2015, 50 (1), 79–88.
6. Shaddick G, Thomas M, Amini H, Broday DM, Cohen A, Frostad J, Green A, Gumy S, Liu Y, Martin RV, Prüss-Üstün A, Simpson D, van Donkelaar A, Brauer M. Data integration for the assessment of population exposure to ambient air pollution for global burden of disease assessment. Environ Sci Technol. 2018 Jun 29. doi: 10.1021/acs.est.8b02864
7. Rue, H.; Martino, S.; Chopin, N.; Approximate Bayesian inference for latent Gaussian models by using integrated nested Laplace approximations. Journal of the royal statistical society: Series b (statistical methodology). 2009;71(2):319-92.
8. Thomas, M. L., Shaddick, G., Simpson, D., de Hoogh, K. and Zidek, J. V. Spatio-temporal downscaling for continental–scale estimation of air pollution concentrations. arXiv preprint arXiv:1907.00093 (also been Submitted to the Journal of the Royal Statistical Society: Series C (Applied Statistics)).
9. Turner MC, Jerrett M, Pope CA 3rd, Krewski D, Gapstur SM, Diver WR, Beckerman BS, Marshall JD, Su J, Crouse DL, Burnett RT. Long-term ozone exposure and mortality in a large prospective study. Am J Respir Crit Care Med. 2016; 193(10): 1134-42.
10. Yin P, Brauer M, Cohen A, et al. Long-term Fine Particulate Matter Exposure and Nonaccidental and Cause-specific Mortality in a Large National Cohort of Chinese Men. Environ Health Perspect 2017; 125: 117002.
11. Li T, Zhang Y, Wang J, et al. All-cause mortality risk associated with long-term exposure to ambient PM2·5 in China: a cohort study. Lancet Public Health 2018; 3: e470–7.
12. Yang Y, Tang R, Qiu H, et al. Long term exposure to air pollution and mortality in an elderly cohort in Hong Kong. Environ Int 2018; 117: 99–106.
13. Hystad P, Larkin A, Rangarajan S, AlHabib KF, Avezum A, Tumerdem Calik KB; Chifamba J, Dans A, Diaz R, du Plessis JL, Gupta R, Iqbal R, Khatib R, Kelishadi R, Lanas F, Liu Z, Lopez-Jaramillo P, Nair S, Poirier P, Rahman O, Rosengren A, Swidan H, Tse L-A, Wei L, Wielgosz A, Yeates K, Yusoff K, Zatoński T, Yusuf S, Brauer M. Outdoor fine particulate matter air pollution and cardiovascular disease: Results from 747 communities across 21 countries in the PURE Study. (Submitted to Lancet Global Health)
14. Joseph P, Rangarajan S, Islam S, Mente A, Hystad P, Brauer M, Raman Kutty V, Gupta R, Wielgosz A, AlHabib KF, Dans A, Lopez-Jaramillo P, Avezum A, Lanas F, Oguz A, Kruger IM, Diaz R, Yusoff K, Mony P, Chifamba J, Yeates K, Kelishadi R, Yusufali A, Khatib R, Rahman O, Zatonska K, Iqbal R, Wei L, Bo H, Rosengren A, Kaur M, Mohan V, Lear SA, Teo KK, O'Donnell M, McKee M, Dagenais G, Yusuf S. Modifiable risk factors, cardiovascular disease and mortality in 155,722 individuals

from 21 high-, middle-, and low-income countries (PURE): a prospective cohort study. The Lancet. 2019. doi:10.1016/S0140-6736(19)32008-2
15. Burnett RT, Pope CA 3rd, Ezzati M, Olives C, Lim SS, Mehta S, Shin HH, Singh G, Hubbell B, Brauer M, Anderson HR, Smith KR, Balmes JR, Bruce NG, Kan H, Laden F, Prüss-Ustün A, Turner MC, Gapstur SM, Diver WR, Cohen A. An integrated risk function for estimating the global burden of disease attributable to ambient fine particulate matter exposure. *Environ Health Perspect*. 2014; 122(4): 397-403.
16. Pope CA III, Cohen AJ, Burnett RT. Cardiovascular Disease and Fine Particulate Matter: Lessons and Limitations of an Integrated Exposure Response Approach. Circulation Research. 2018;122:1645-1647.
17. Lind L, Sundström J, Ärnlöv J, Lampa E. Impact of Aging on the Strength of Cardiovascular Risk Factors: A Longitudinal Study Over 40 Years. *J Am Heart Assoc.* 2018;7(1):e007061. Published 2018 Jan 6. doi:10.1161/JAHA.117.007061
18. Ghosh R, Causey K, Burkart K, Wozniak S, Cohen A, Brauer M. Ambient and household $PM_{2.5}$ pollution and adverse perinatal outcomes: A meta-regression and analysis of attributable global burden for 204 countries and territories. *PLoS Med* 2021. Accepted; in press.
19. US Environmental Protection Agency. Integrated science assessment (ISA) for particulate matter (Final Report, Dec 2009). EPA/600/R-08/139F, 2009. Washington, DC: US Environmental Protection Agency; 2009. Available at: http://cfpub.epa.gov/ncea/risk/recordisplay.cfm?deid=216546
20. World Health Organization. Review of evidence on health aspects of air pollution – REVIHAAP Project technical report. Copenhagen: WHO Regional Office for Europe; 2013. Available at: http://www.euro.who.int/__data/assets/pdf_file/0004/193108/REVIHAAP-Final-technical-report-final-version.pdf?ua=1

Bone mineral density

Flowchart

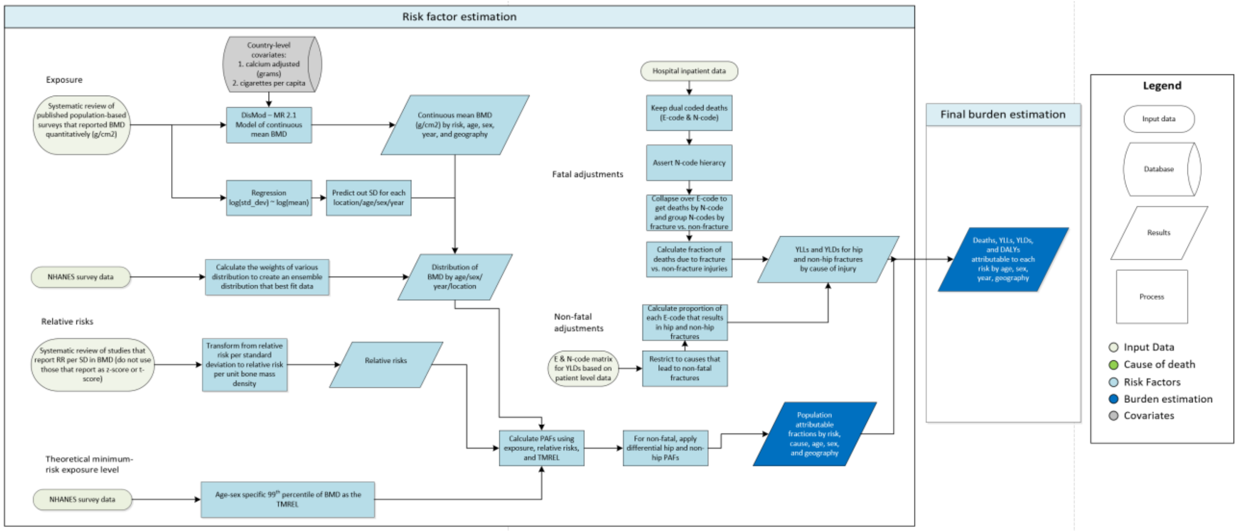

Input data and methodological summary

Definition

Exposure

Bone mineral density (BMD) is a continuous variable measured by dual-X-ray-absorptiometry (DXA) at the femoral neck (FN) and is presented in g/cm^2 after standardising for the brand of densitometer

(sBMD). Low BMD is measured in terms of the difference between BMD of a population and the 99th percentile of a reference population at the same age and sex (theoretical minimum-risk exposure level, TMREL). The burden attributed to low BMD is estimated for adults 20 years and older.

Input data

Exposure

A systematic review (search string at the end of document) was conducted in GBD 2010 and updated for GBD 2013 and 2015 using the same search string. It was not scheduled for systematic review in GBD 2016, 2017, 2019, or 2020. Inclusion criteria that informed the search are:

- Representative, population-based surveys
- Reporting of quantitative BMD
  - measured by DXA
  - performed at the FN region
  - measured in g/cm^2

Mean BMD was occasionally reported in stratified groups, eg, by fracture status but not for total sample. In these cases, the stratified means were aggregated to obtain a total mean BMD at the population level for an age or sex category. Two additional studies provided by collaborators were added for GBD 2019.

For GBD 2019, we also began tagging existing data with study covariates for BMD measured at sites other than the FN: the greater trochanter, intertrochanter, Ward's triangle, total femur, total hip, distal radius, and lumbar spine. In the future, these covariates can inform potential bias adjustments in the event that new data are added that do not report BMD measured at our reference site. The data in the current BMD model, however, do not require any bias adjustment.

Table 1. Data inputs for exposure for BMD

	Countries with data	New sources	Total sources
Exposure	49	1	169

Relative risk

Relative risks (RR) must be reported per standard deviation or per unit bone-mass density in order for us to use the data. Studies reporting relative risk in an osteoporotic group versus a non-osteoporotic group were excluded.

For GBD 2017, 12 prospective observational studies were found, but one meta-analysis of 12 studies[1] reported the dose–response relationship between low BMD and high relative risk of hip and other fractures that are prone to osteoporosis, as shown in the below table.

Figure 1: Dose-response between low BMD and RR of fracture

BMD z score	Any fracture RR	Any fracture 95% CI	Osteoporotic fracture RR	Osteoporotic fracture 95% CI	Hip fracture RR	Hip fracture 95% CI
−4	1.79	1.44–2.23	2.10	1.63–2.71	2.14	1.40–3.26
−3	1.71	1.44–2.02	1.96	1.61–2.39	2.12	1.54–2.92
−2	1.63	1.45–1.84	1.84	1.60–2.12	2.11	1.70–2.62
−1	1.56	1.45–1.69	1.73	1.59–1.89	2.11	1.86–2.39
0	1.50	1.44–1.56	1.62	1.54–1.71	2.08	1.91–2.26
1	1.39	1.32–1.46	1.42	1.34–1.51	2.04	1.78–2.34
2	1.32	1.21–1.45	1.33	1.19–1.48	2.03	1.60–2.56
3	1.26	1.10–1.45	1.25	1.06–1.47	2.01	1.44–2.81
4	1.21	1.00–1.45	1.17	0.93–1.46	1.99	1.28–3.10

The z score ranged from −5.1 to +5.8.

For GBD 2019, we re-estimated relative risk estimates for hip and non-hip fractures using the meta-regression—Bayesian, regularised, trimmed (MR-BRT) meta-analysis method. Input data studies consisted of those identified through a re-review of the 12 studies included in the 2005 meta-analysis that provided our previous relative risk dose-response estimates. We extracted relative risk data from six of those 12 studies, excluding cohorts that used a measure of exposure other than BMD measured by DXA at the FN and those that used mortality as an outcome of interest instead of fracture.

In addition, a systematic review of the relative risk of fracture due to low BMD was conducted for the years 2010 to 2020 on PubMed using the following search terms: (((BMD OR BMDs OR bone density) AND (mean OR average) AND risk) AND fracture). Results were filtered for comparative studies, journal articles, meta-analyses, or observational studies published in English. This search yielded 611 results. Cohorts were excluded for the same reasons mentioned above. We extracted relative risk data from six sources. Nine countries were represented among the 12 cohort studies included in the meta-analysis.[2-13]

Table 2: Data inputs for relative risks for BMD

	Countries with data	New sources	Total sources
Relative risks	9	0	12

Figure 2: PRISMA diagram of BMD RR systematic review from 2019

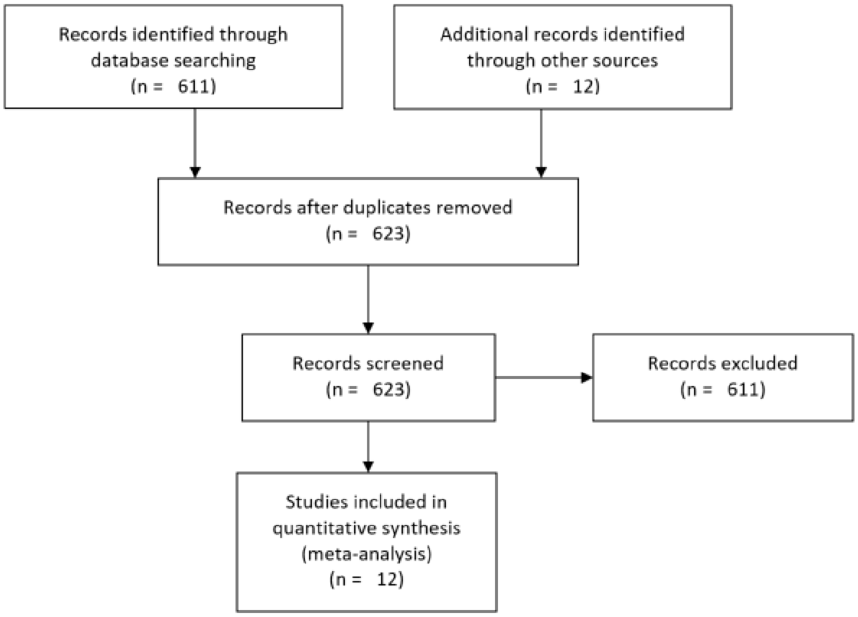

Modelling strategy

Exposure

We modelled mean BMD in DisMod-MR 2.1 as a single "continuous" parameter model by age and sex, and all GBD locations for years 1990–2019. The model had age mesh points at 0 10 20 25 30 40 50 60 70 80 90 & 100, a time window of ten years for fitting data, and a minimum coefficient of variation of 0.1 for global, 0.06 for super-regions, and 0.08 for the region level. We made no substantive changes in the modelling strategy from GBD 2017.

The country covariates of total physical activity (MET-min/week), tobacco consumption (cigarettes per capita), mean BMI, and unadjusted calcium intake (g) were included in modelling.

Table 3. Summary of covariates used in the BMD DisMod-MR meta-regression model

Covariate	Type	Parameter	Exponentiated beta (95% uncertainty interval)
Total physical activity (MET-min/week), age-standardised	Country-level	Continuous	1.00 (1.00 to 1.00)
Tobacco consumption (cigarettes per capita)	Country-level	Continuous	0.98 (0.96 to 1.00)
Mean BMI	Country-level	Continuous	1.01 (1.00 to 1.01)
Calcium intake (g), unadjusted	Country-level	Continuous	1.00 (1.00 to 1.03)

We consider the risk of fatal and non-fatal outcomes for hip/non-hip fractures, separately, as relative risk data provide different estimates. Thus, there were various steps after DisMod-MR 2.1 exposure modelling to arrive at attributable fractions that can be applied to fatal and non-fatal fracture outcomes. Osteoporotic non-hip fractures include fractures of vertebrae, clavicle, scapula, humerus, skull, sternum, face bone, radius or ulna, femur, patella, tibia, fibula, ankle, and pelvis.

First, we calculated the proportion of injury deaths that are due to fractures. This proportion of deaths caused by fracture is the envelope that we use to attribute death to BMD. In order to do this, we assumed that hip fracture and some non-hip fractures (any fractures apart from those of fingers and toes) are potentially fatal fractures. As cause of death data from vital registration and verbal autopsy attribute injury deaths to causes of death (eg, fall or road injury) and not nature of injury (such as fractures), we used available hospital data to estimate the proportion of injury deaths during admission that could be ascribed to fractures. We restricted our analysis to cases that were dual-coded with both the cause of injury ("E-code") and nature of injury ("N-code"). As injury cases may have multiple forms of trauma, we applied a severity hierarchy to the fatal hospital data to determine the proportion of the deaths that could be attributed to the chosen fracture types but were not accompanied by more severe fatal trauma such as head trauma, spinal cord lesion, and intra-abdominal or thoracic organ damage. We collapsed all deaths over E-code to determine the ratio of deaths attributable to fracture versus non-fracture injuries. We applied this ratio to the YLLs.

We restricted non-fatal estimates of low BMD to a list of causes that were deemed to cause osteoporotic fractures. Below is the list of injuries for which a population attributable fraction (PAF) was calculated:

- Transport injuries
- Road injuries
- Pedestrian road injuries
- Cyclist road injuries
- Motorcyclist road injuries
- Motor vehicle road injuries
- Other road injuries
- Other transport injuries
- Unintentional injuries
- Falls
- Exposure to mechanical forces
- Other exposure to mechanical forces
- Non-venomous animal contact
- Interpersonal violence
- Assault by other means

We made use of the E- to N-code matrix generated from dual-coded (E-code/N-code) patient-level data in our injury analyses to determine the proportion of each E-code that results in a certain N-code. The hip and non-hip fracture population attributable fractions were applied to the appropriate combinations of external cause and fracture estimates of YLD and then summed together to produce a single estimate.

Theoretical minimum-risk exposure level

The theoretical minimum of risk exposure level, or TMREL, was chosen as the age-sex specific 99th percentile of BMD from five cycles of NHANES study as the reference population. Below is a descriptive table of the five NHANES cycles used.

Table 4. TMREL. Summary of NHANES reference population

NHANES cycle	Age range (years)	Number of people tested	BMD range (g/cm^2)
1988	20–90	14,646	0.23–1.84
2005	20–85	3,494	0.40–1.50
2007	20–80	4,726	0.34–1.46
2009	20–80	5,052	0.33–1.63
2013	40–80	3,127	0.39–1.36

Figure 3: Plot of 99th percentile of BMD at FN in each cycle of NHANES

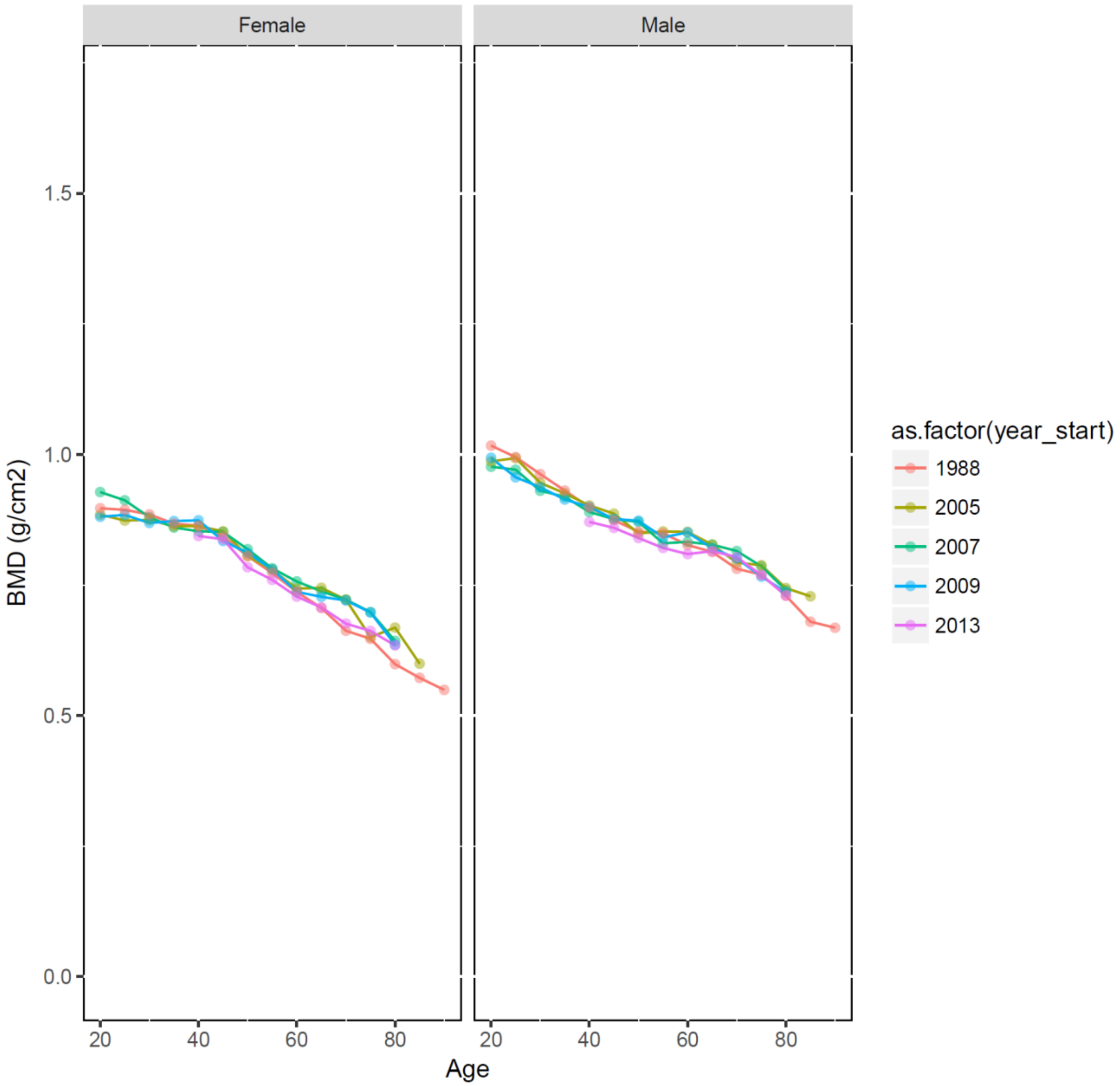

Relative risk

Relative risk estimates were updated during GBD 2019. No further updates were made during GBD 2021. We used study covariates for studies that reported the relative risk of low BMD on hip fracture and non-hip fractures, and for the percentage of the cohort that was male (which was always either 1 or 0). The mean and standard error for the coefficients were calculated using the MR-BRT crosswalk adjustment method with a cubic spline on cohort mean age. An adjustment for percentage male was not included in the final model, as we did not find a significant difference between relative risks for males and females. The age spline was also not included in the final model. After testing four iterations with two and three knots placed evenly or by data frequency, it was clear that there was not a reliable relationship between cohort mean age and relative risk. Betas and exponentiated values (which can be

interpreted as the relative risks) for the remaining hip and non-hip fracture covariates are shown in the table below:

Table 5. MR-BRT Crosswalk Results for RR of Fracture due to low BMD

Data input	Gamma	Beta coefficient, log (95% CI)	RR per SD unit of BMD
Hip fracture	0.13	0.77 (0.45 to 1.08)	2.18 (1.66 to 2.80)
Non-hip fracture		0.57 (0.30 to 0.84)	1.79 (1.41 to 2.23)

Figure 4: MR-BRT crosswalk results for RR of fracture due to low BMD

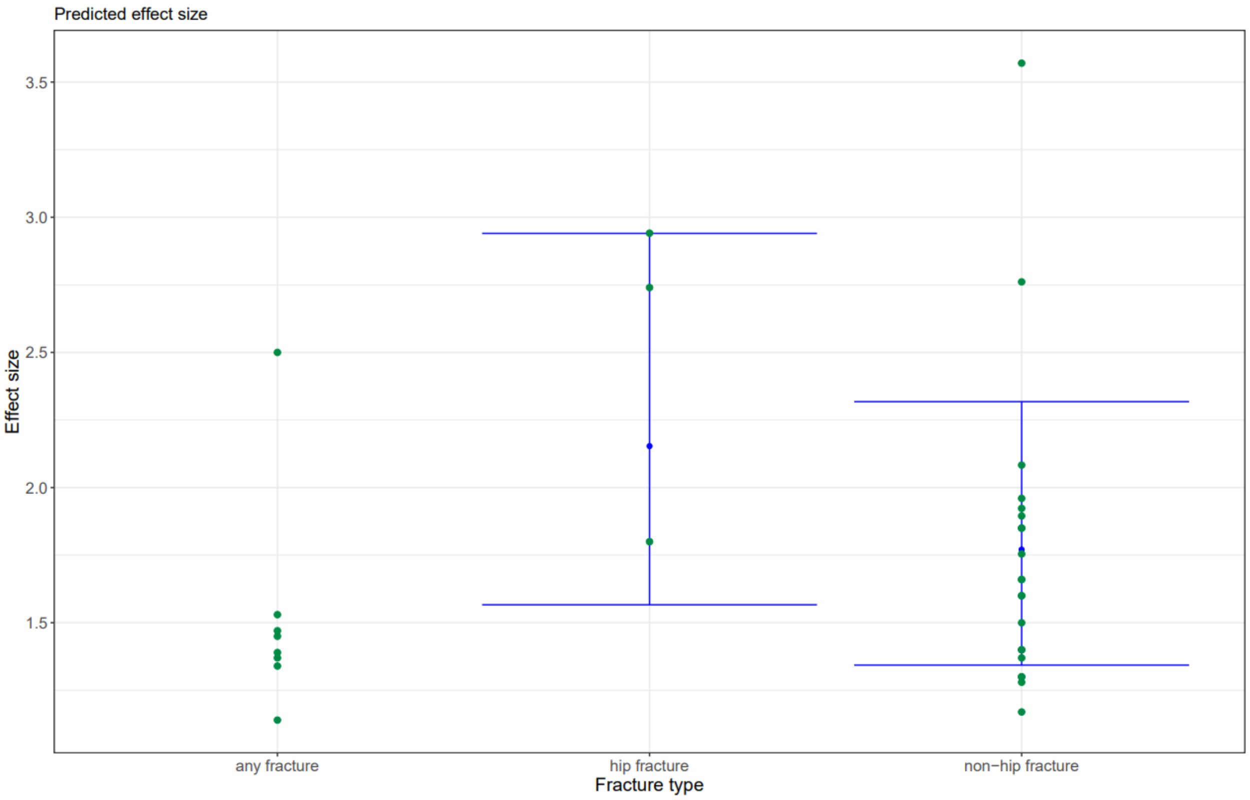

Citations

1. Johnell O, Kanis JA, Oden A, Johansson H, De Laet C, Delmas P, Eisman JA, Fujiwara S, Kroger H, Mellstrom D, Meunier PJ, Melton LJ, 3rd, O'Neill T, Pols H, Reeve J, Silman A, Tenenhouse A (2005) Predictive value of BMD for hip and other fractures. J Bone Miner Res 20 (7):1185-1194
2. Berger C, Langsetmo L, Joseph L, Hanley DA, Davison KS, Josse RG, Prior JC, Kreiger N, Tenenhouse A, Goltzman D, CaMos Research Group. Association between change in BMD and fragility fracture in women and men. J Bone Miner Res. 2009; 24(2): 361-70.

3. Bow CH, Tsang SW, Loong CH, Soong CS, Yeung SC, Kung AW. Bone mineral density enhances use of clinical risk factors in predicting ten-year risk of osteoporotic fractures in Chinese men: the Hong Kong Osteoporosis Study. Osteoporosis Int. 2011; 22(11): 2799-807.
4. Chalhoub D, Orwoll ES, Cawthon PM, Ensrud KE, Boudreau R, Greenspan S, Newman AB, Zmuda J, Bauer D, Cummings S, Cauley JA, Osteoporotic Fractures in Men (MrOS) Study Research Group
5. Crandall CJ, Hovey KM, Andrews CA, Cauley JA, Manson JE, Wactawski-Wende J, Wright NC, Li W, Beavers K, Curtis JR, LeBoff MS. Bone mineral density as a predictor of subsequent wrist fractures: Findings from the Women's Health Initiative Study. J Clin Endocrinal Metab. 2015; 100(11): 4315-24.
6. Dargent-Molina P, Favier F, Grandjean H, Baudoin C, Schott AM, Hausherr E, Meunier PJ, Bréart G. Fall-related factors and risk of hip fracture: the EPIDOS prospective study. Lancet. 1996; 348(9021): 145-9.
7. Fujiwara S, Kasagi F, Masunari N, Naito K, Suzuki G, Fukunaga M. Fracture Prediction From Bone Mineral Density in Japanese Men and Women. J Bone Miner Res. 2003; 18(8): 1547-53.
8. Huopio J, Kröger H, Honkanen R, Saarikoski S, Alhava E. Risk factors for perimenopausal fractures: a prospective study. Osteoporos Int. 2000; 11(3): 219-27.
9. Kwok AW, Gong JS, Wang YX, Leung JC, Kwok T, Griffith JF, Leung PC. Prevalence and risk factors of radiographic vertebral fractures in elderly Chinese men and women: Results of Ms. OS (Hong Kong) and Ms. OS (Hong Kong) studies. Osteoporosis Int. 2013; 23(3); 877-85.
10. Melton LJ, Crowson CS, O'Fallon WM, Wahner HW, Riggs BL. Relative contributions of bone density, bone turnover, and clinical risk factors to long-term fracture prediction. J Bone Miner Res. 2003; 18(2): 312–8.
11. Nguyen TV, Eisman JA, Kelly PJ, Sambrook PN. Risk factors for osteoporotic fractures in elderly men. Am J Epidemiol. 1996; 144(3): 255–63.
12. Sheu Y, Cauley JA, Patrick AL, Wheeler VW, Bunker CH, Zmuda JM. Risk factors for fracture in middle-age and older-age men of African descent. J Bone Miner Res. 2014; 29(1): 234-41.
13. Shin CS, Kim MJ, Shim SM, Kim JT, Yu SH, Koo BK, Cho HY, Choi HJ, Cho SW, Kim SW, Kim SY, Yang SO, Cho NH. The prevalence and risk factors of vertebral fractures in Korea. J Bone Miner Metab. 2012; 30(2): 183-192

Bullying victimisation

Flowchart

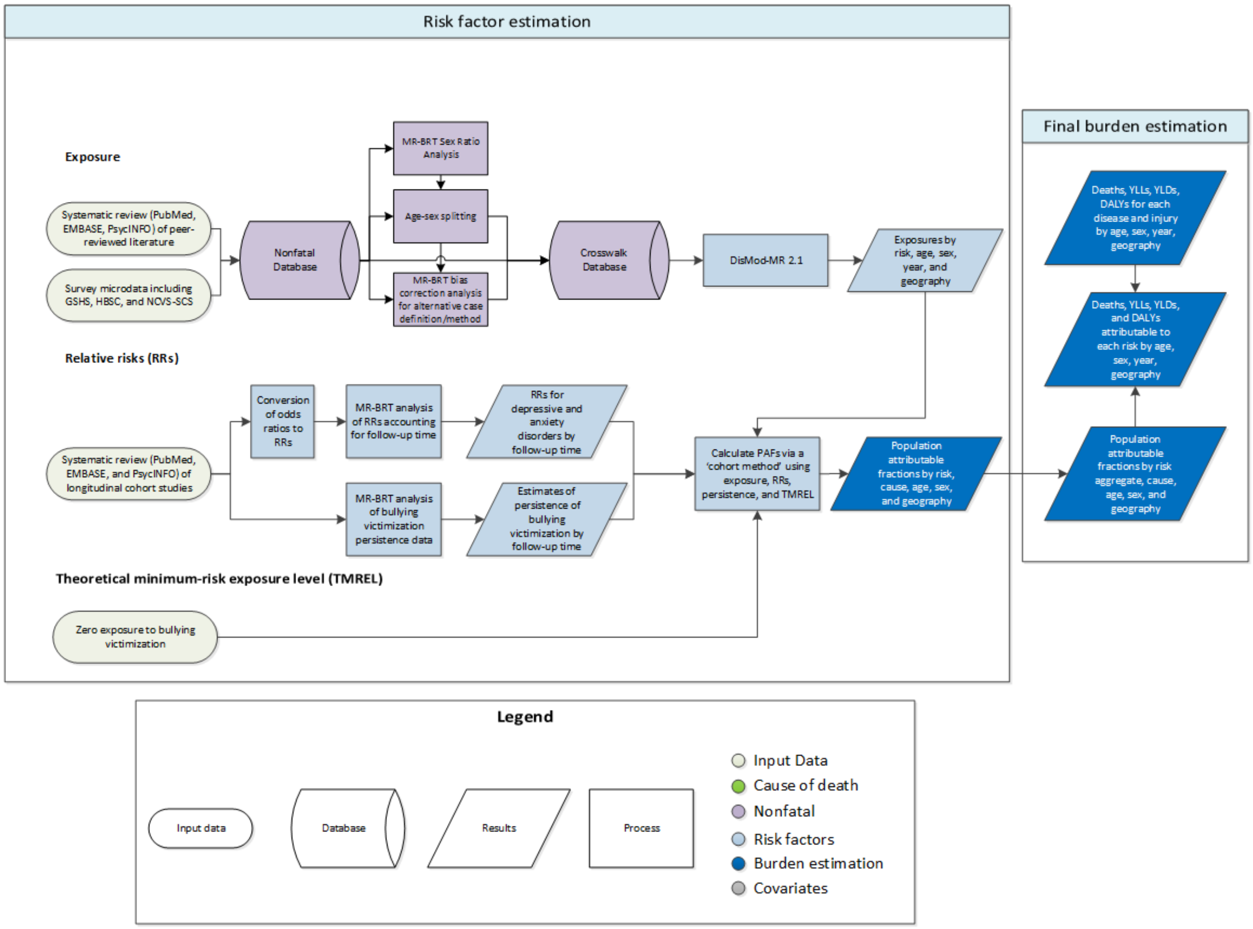

Input data and methodological summary

Exposure

Case definition

Bullying victimisation is commonly conceptualised as the intentional and repeated harm of a less powerful individual by peers.[1] This differentiates bullying victimisation from disagreements, conflicts, or playful teasing. The case definition of bullying victimisation in the GBD context is "bullying victimisation of children and adolescents attending school by peers". This definition includes the global concept of bullying victimisation, which incorporates combined estimates of subtypes such as physical, verbal, relational, and cyberbullying victimisation. It excludes abuse/harassment by siblings, intimate partners, and adults (eg, teachers). While bullying can be experienced as either a victim or perpetrator, perpetration (ie, those who bully others) is not included in this definition although some victims will also be perpetrators.

Input data

Exposure

In order for a study to be included, it must report the prevalence of bullying victimisation and 1) have been published since 1980, 2) ask participants about bullying victimisation in the previous year or more recently, 3) use an appropriate frequency threshold to define bullying victimisation (approximating at least once a week or greater than "occasionally"), 4) be representative of the general population rather than a special population (eg, ethnic minorities), and 5) report prevalence for bullying victimisation overall rather than a subtype (eg, physical bullying victimisation).

A new systematic review for bullying victimisation was conducted for GBD 2017, with the next electronic literature update due for the next round of GBD. Included studies were sourced from a systematic review of three electronic databases (PubMed, EMBASE, and PsycINFO), covering the period 1980 to 2017. No restriction was set on the language of publication. The Global Health Data Exchange (GHDx) was also used to source microdata from survey series meeting the above inclusion criteria. Estimates from the Global School-based Student Health Survey (GSHS), the Health Behavior in School-aged Children (HBSC), and the National Crime Victimization Survey – School Crime Supplement (NCVS-SCS) were extracted and included in the dataset. The grey literature search and expert consultation conducted for GBD 2019 did not reveal any additional studies. The table below summarises exposure data inputs for bullying victimization.

Table 1: Exposure input data for bullying victimisation

	Countries with data	New sources	Total sources
Exposure	119	0	308

Age and sex splitting

The extracted data underwent two types of age and sex splitting processes:

1. Where possible, estimates were further split by sex and age based on the data that were available. For instance, if studies reported prevalence for broad age groups by sex (eg, prevalence in 5–17-year-old males and females separately), and also by specific age groups, but for both sexes combined (eg, prevalence in 5–12-year-olds, then in 13–17-year-olds, for males and females combined), age-specific estimates were split by sex using the reported sex ratio and bounds of uncertainty.
2. A meta-regression—Bayesian, regularised, trimmed (MR-BRT) analysis was used to split the remaining both-sex estimates in the dataset. For each parameter, sex-specific estimates were matched by location, age, and year, and a MR-BRT network meta-analysis was used to estimate pooled sex ratios and bounds of uncertainty. These were then used to split the both-sex estimates in the dataset. The male:female prevalence ratio was 1.20 (95% CI: 0.88–1.53).

Bias corrections/crosswalks

Estimates with known biases were adjusted/crosswalked accordingly prior to DisMod-MR 2.1. Within the bullying victimisation epidemiological dataset, within and between-study estimates were paired by age, sex, location, and year, between the reference and alternative estimates. Pairs were also made between the different alternative estimates. The ratios between these estimates were then used as inputs in a MR-BRT network meta-analysis. This analysis produced pooled ratios between the reference

estimates and alternative estimates. These ratios (see Table 2) were used to adjust all alternative estimates in the dataset. Bullying victimisation had four alternative definitions to crosswalk:

1. Suboptimal frequency threshold used, eg, “sometimes + frequently”
2. No definition of bullying victimisation presented to participants or not specified
3. Asked about bullying victimisation in the past year

Table 2: MR-BRT crosswalk adjustment factors for bullying victimisation

Data input	Reference or alternative case definition	Gamma	Beta coefficient, log (95% CI)*	Adjustment factor (95% CI)**
School survey	Reference: Point proportion of children and adolescents attending school who have been exposed to bullying victimisation by peers at least once a week or greater than “occasionally”. Participants are given a definition of bullying victimisation prior to being asked about exposure.	0.33	---	---
School survey	Alternative: Suboptimal frequency threshold used, eg, “sometimes + frequently”		1.17 (0.51–1.86)	3.22 (1.68–6.45)
School survey	Alternative: No definition of bullying victimisation presented to participants or not specified		0.27 (-0.39 to 0.94)	1.30 (0.68–2.55)
School survey	Alternative: Past-year proportion		0.16 (-0.49 to 0.81)	1.17 (0.61–2.25)

**MR-BRT crosswalk adjustments can be interpreted as the factor the alternative case definition is adjusted by to reflect what it would have been had it been measured using the reference case definition. If the log/logit beta coefficient is negative, then the alternative is adjusted up to the reference. If the log/logit beta coefficient is positive, then the alternative is adjusted down to the reference.*

***The adjustment factor column is the exponentiated beta coefficient. For log beta coefficients, this is the relative rate between the two case definitions. For logit beta coefficients, this is the relative odds between the two case definitions.*

Confidence intervals incorporate gamma, which represents the between-study variance across all input data in the model. This added uncertainty widens the confidence intervals for crosswalks with significant fixed effects.

Relative risk

For GBD 2017, studies reporting the prospective longitudinal association between these outcomes and bullying victimisation were sourced from a systematic review of three electronic databases (PubMed, EMBASE, and PsycINFO), covering the period 1980–2017. No restriction was set on the language of publication. Studies had to report relative risks (RRs), odds ratios, or sufficient data to calculate RRs (ie, exposed/non-exposed cases/non-cases). Altogether, there were 23 studies reporting on 14 cohorts (see

Table 3) across six countries. The electronic literature update for relative risk estimates is due for the next round of GBD.

Table 3: Relative risk input data for bullying victimisation

	Countries with data	New sources	Total sources
Relative risks	6	0	23

Modelling strategy

Exposure

After the above data processes were applied, DisMod-MR 2.1 was used to model the prevalence of bullying victimisation. Bullying victimisation prevalence was modelled as a single parameter prevalence model. The DisMod-MR modelling strategy for bullying victimisation followed the standard GBD 2019 decomposition structure. At each decomposition step, we compared the new model against the GBD 2017 best model and the best model from the previous step. All substantial changes between models were explored and explained. Adjustments to model priors or the dataset were made where appropriate. Where outliers were identified in the data, we reassessed the study's methodology and quality before a decision was made to exclude or include the data. We assumed no prevalence prior to 5 years or after 20 years of age.

Adjustment for years of schooling

In order to better represent the prevalence of bullying victimisation, prevalence estimates were adjusted for the proportion of children and adolescents attending school by ages 5–9, 10–14, and 15–19 years by sex, location, and year. Data on the proportion of children and adolescents attending school was sourced from the online database (http://data.uis.unesco.org/) published by the United Nations Educational, Scientific, and Culture Organization (UNESCO). The data covered 18,441 country-years for age groups 6–11, 12–14, and 15–17 years by sex. These data were modelled in ST-GPR, with average years of education as a country-level covariate, to predict the proportion of children and adolescents attending school by these age groups. This gave estimates of the proportion of children and adolescents attending school by age, sex, year, and location.

Theoretical minimum-risk exposure level

The theoretical minimum-risk exposure level was assumed to be zero exposure to bullying victimisation.

Relative risks

We estimate burden attributable to bullying victimisation for major depressive disorder (MDD) and anxiety disorders. Data on the association between bullying victimisation and self-harm was also reviewed but not included due to variation in the definition of "self-harm" and only one study looking at suicide.

Estimation of pooled relative risks

MR-BRT meta-regressions were conducted to determine the impact of follow-up time on the risk of MDD and anxiety disorders following exposure to bullying victimisation. These analyses controlled for percent of the sample female and four bias covariates: 1) Exposure reported by parent only, 2) Estimate derived from multiple logistic regression, 3) Suboptimal exposure frequency threshold, 4) 15%+ attrition

at follow-up. Estimates were nested within cohorts. All available estimates by cohort that varied across the covariates were extracted to better inform the covariates. An initial log-linear model containing both MDD and anxiety disorders was run to obtain priors for sex and the bias covariates for disorder-specific ensemble random splines models with decreasing monotonicity priors.

Population attributable fractions (PAFs)

A cohort method was developed to accommodate the waning risk over time observed in the MR-BRT meta-regression of the RRs. The following steps are conducted for each point of estimation (ie, by age, sex, location, and year), hereafter referred to as a "cohort":

1. Pull current and past bullying victimisation prevalence for the cohort from the DisMod-MR 2.1 exposure model.
2. Adjust each bullying victimisation prevalence estimate for the proportion of the cohort attending school in that year.
3. Estimate incidence of bullying victimisation within the cohort for each year using the following formula:

$$I_k = P_k - \sum_{n=0}^{k-1} (I_n \times r_{k-n})$$

   Where *I* represents incidence, *P* represents prevalence, *r* represents the estimate of persistence, and *k* represents the time between the incidence estimate and the earliest possible time of exposure in the cohort. I_k requires I_0 through to I_{k-1} to first be calculated and so we complete this process by first estimating I_0, then I_1, and so on until we have estimated incidence for the latest possible year of exposure for this cohort. The persistence estimate is based on a separate MR-BRT meta-regression of seven studies.[2-8]
4. Use the incidence estimates to divide the cohort into proportions based on time since first exposed to bullying victimisation:

$$p_t = I_{\max(k)-k}$$

   Where t is the time since first exposed to bullying victimisation, and p is the proportion of the cohort first exposed to bullying victimisation at time t.
5. Estimate PAFs via the following formula:

$$PAF = \frac{\sum(p_t \times RR_t) + p_{no\ exposure} - 1}{\sum(p_t \times RR_t) + p_{no\ exposure}}$$

   Where *t* is the time since first exposed to bullying victimisation, *p* is the proportion of the cohort first exposed to bullying victimisation at time *t* or the proportion not exposed to bullying victimisation, and *RR* is the relative risk for depressive and anxiety disorders given *t*.

GBD 2021 prevalence and burden estimates for MDD and anxiety disorders incorporated the impact of the COVID-19 pandemic, leading to an increase in estimated prevalent cases and burden in 2020 for these two disorders. After consultation with our GBD collaborators regarding this, we removed the impact of COVID-19 from the PAF estimation for bullying victimisation. This was achieved by removing the additional prevalence due to the COVID-19 pandemic from the final PAFs for the year 2020. To date, there was insufficient evidence to suggest that bullying victimisation would explain part of this additional prevalence or burden. This will be reviewed as more epidemiological data relating to the impacts of the pandemic on bullying victimisation emerge.

Changes between GBD 2019 and GBD 2021

There were two main changes in the GBD 2021 modelling strategy compared to GBD 2019:

1. In GBD 2019, the RRs for MDD and anxiety disorders following bullying victimisation were estimated in a single log-linear MR-BRT model. In GBD 2021, disorder-specific ensemble random spline MR-BRT models were conducted to estimate RRs. The MR-BRT analysis resulted in RRs that were slightly higher for MDD than in GBD 2019. Sex-specific RRs were also estimated from this model resulting in larger PAFs for males compared to females.
2. We developed a meta-analytic approach and burden of proof risk function to operationalise the relationship between bullying victimisation and MDD and anxiety disorders respectively in GBD 2021. This work is currently being prepared for publication.

References

1. Olweus D. Bullying at school: What we know and what we can do. MA, USA; Oxford, UK; Vic, Australia: Blackwell; 1993.

2. Baly MW, Cornell DG, Lovegrove P. A Longitudinal Investigation of Self- and Peer Reports of Bullying Victimization Across Middle School. *Psychology in the Schools* 2014; **51**(3): 217-40.

3. Bowes L, Maughan B, Ball H, et al. Chronic bullying victimization across school transitions: the role of genetic and environmental influences. *Development and psychopathology* 2013; **25**(2): 333-46.

4. Kumpulainen K, Rasanen E, Henttonen I. Children involved in bullying: psychological disturbance and the persistence of the involvement. *Child Abuse Negl* 1999; **23**(12): 1253-62.

5. Lereya ST, Copeland WE, Zammit S, Wolke D. Bully/victims: A longitudinal, population-based cohort study of their mental health. *European Child & Adolescent Psychiatry* 2015; **24**(12): 1461-71.

6. Lien L, Welander-Vatn A. Factors Associated with the Persistence of Bullying Victimization From 10th grade to 13th Grade: A Longitudinal Study. *Clinical practice and epidemiology in mental health : CP & EMH* 2013; **9**: 243-50.

7. Sourander A, Helstela L, Helenius H, Piha J. Persistence of bullying from childhood to adolescence--a longitudinal 8-year follow-up study. *Child Abuse Negl* 2000; **24**(7): 873-81.

8. Winsper C, Lereya T, Zanarini M, Wolke D. Involvement in bullying and suicide-related behavior at 11 years: a prospective birth cohort study. *Journal of the American Academy of Child and Adolescent Psychiatry* 2012; **51**(3): 271-82.e3.

Chewing tobacco

Flowchart

Chewing Tobacco

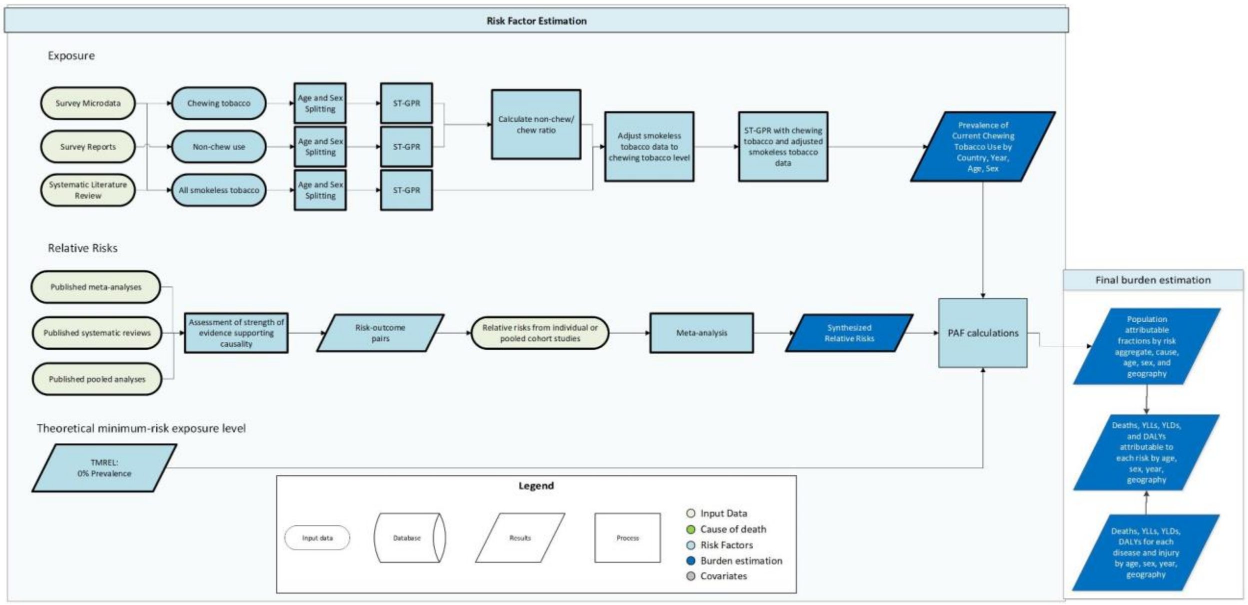

Input data and methodological summary

Definition

Exposure

Current chewing tobacco use is defined as current use (use within the last 30 days where possible, or according to the closest definition available from the survey) of any frequency (any, daily, or less than daily). Chewing tobacco includes local products, such as betel quid with tobacco.

Input data

Exposure

As in GBD 2019, we included sources that reported primary chewing tobacco, non-chew smokeless tobacco, and all smokeless tobacco use among respondents over age 10. To be eligible for inclusion, sources had to be representative for their level of estimation (ie, national sources needed to be nationally representative, and subnational sources had to be subnationally representative). We included only self-reported use data and excluded data from questions asking about others' tobacco use behaviors.

We extracted primary data from individual-level microdata and survey report tabulations on chewing tobacco, non-chewing smokeless tobacco, and any smokeless tobacco use. We extracted data on current, former, and/or ever use as well as frequency of use (daily, occasional, and unspecified, which

includes both daily and occasional smokers). Products that do not include tobacco, such as betel quid without tobacco, were excluded or estimated separately as part of the drug use risk factor, if applicable.

For microdata, we extracted relevant demographic information, including age, sex, location, and year, as well as survey metadata, including survey weights, primary sampling units, and strata. This information allowed us to tabulate individual-level data in the standard GBD five-year age-sex groups and produce accurate estimates of uncertainty. For survey report tabulations, we extracted data at the most granular age-sex group provided. Compared to GBD 2019, we have identified 64 new input sources to inform our chewing tobacco exposure estimates.

Relative risk

As in GBD 2019, we included outcomes based on the strength of available evidence supporting a causal relationship. There was sufficient evidence to include oral cancer and esophageal cancer as health outcomes caused by chewing tobacco use.

Relative risk estimates were derived from prospective cohort studies and population-based case-control studies. We used the same underlying effect size estimates from prospective cohort studies and population-based case-control studies as in GBD 2019. Briefly, we did not include hospital-based case-control studies due to concerns of over representativeness. We only included sources that adequately adjusted for major confounders, especially smoking status.

Data processing

Age- and sex-splitting

We split data reported in broader age groups than the GBD five-year age groups or as both sexes combined by adapting the method reported in Ng, et al[1] to split using a sex-geography-time-specific reference age pattern. We separated the data into two sets: a training dataset, with data already falling into GBD sex-specific five-year age groups, and a split dataset, which reported data in aggregated age or sex groups. We then used spatiotemporal Gaussian process regression (ST-GPR) to estimate sex-geography-time-specific age patterns using data in the training dataset. The estimated age patterns were then used to split each source in the split dataset.

The ST-GPR model used to estimate the age patterns for age-sex splitting used an age weight parameter value that minimizes the effect of any age smoothing. This parameter choice allows the estimated age pattern to be driven by data, rather than being enforced by any smoothing parameters of the model. Because these age-sex-split datapoints will be incorporated in the final ST-GPR exposure model, we do not want to doubly enforce a modelled age pattern for a given sex-location-year on a given aggregate datapoint. We run three separate age-sex splitting ST-GPR models for – one for each smokeless tobacco category (chew, non-chew, and all smokeless).

Table 1: Data inputs for exposure for chewing tobacco

	Countries with data	New sources	Total sources
Exposure	188	64	782

Table 2: Data inputs for relative risks for chewing tobacco

	Countries with data	New sources	Total sources
Relative risks	1	0	6

Modelling strategy

Exposure

We used a ST-GPR to model chewing tobacco prevalence. Full details on the ST-GPR method are reported elsewhere in the Appendix. Briefly, the mean function input to GPR is a complete time series of estimates generated from a mixed effects hierarchical linear model plus weighted residuals smoothed across time, space, and age. The linear model formula for chewing tobacco, fit separately by sex using restricted maximum likelihood in R, is:

$$logit(p_{g,a,t}) = \beta_0 + \sum_{k=1}^{18} \beta_k I_{A[a]} + \alpha_s + \alpha_r + \alpha_g + \epsilon_{g,a,t}$$

Where $I_{A[a]}$ is a dummy variable indicating a specific age group A that the prevalence point $p_{g,a,t}$captures, and α_s, α_r, and α_g are super-region, region, and geography random intercepts, respectively. The hyperparameters are the same as in GBD 2019. We run three ST-GPR models for each prevalence category – one for each smokeless tobacco category (chew, non-chew, and all smokeless).

All smokeless tobacco prevalence adjustment

Using the 1000 draws from each of the prevalence ST-GPR models, we calculated 1000 draws of chewing tobacco prevalence divided by the sum of chewing tobacco and non-chewing tobacco prevalence for each location, age group, sex, and year. The draws were unordered, as we did not want to enforce an assumption about the relationship between the levels of chewing tobacco and non-chewing tobacco prevalence.

The draws of the ratio of chewing to non-chewing tobacco were then multiplied by the draws from the all smokeless tobacco prevalence model to adjust the estimates to chewing tobacco prevalence. These were then averaged to get the mean estimate. The variance across the ratios was calculated for each

location, year, age, and sex, and was added to the variance from the original all smokeless tobacco draws.

Final chewing tobacco prevalence model

To calculate the final chewing tobacco prevalence, we ran an additional ST-GPR model with both the original chewing tobacco data (post-age-sex splitting), as well as the adjusted data. These adjusted data add more information to the model – as surveys will often only ask about all smokeless tobacco consumption – while taking into consideration the uncertainty from the ratio calculation.

Theoretical minimum risk exposure level

The theoretical minimum risk exposure level is that everyone in the population has been a lifelong non-user of chewing tobacco.

Relative risk

As in GBD 2019, summary effect size estimates were calculated in R, using the 'metafor' package. We performed a random effects meta-analysis using the DerSimonian and Laird method, which does not assume a true effect size but considers each input study as selected from a random sample of all possible sets of studies for the outcome of interest. The random effects method allows for more variation between the studies and incorporates this variance into the estimation process. We used an inverse-variance weighting method to determine component study weights. We found significantly different relative risks for oral cancer for males and females and estimated relative risks separately by sex for oral cancer alone.

Citations

1. Ng, M., Freeman, M. K., Fleming, T. D., Robinson, M., Dwyer-Lindgren, L., Thomson, B., Wollum, A., Sanman, E., Wulf, S., Lopez, A. D., Murray, C. J. L., & Gakidou, E. (2014). Smoking Prevalence and Cigarette Consumption in 187 Countries, 1980-2012. *JAMA*, *311*(2), 183. https://doi.org/10.1001/jama.2013.284692

Child growth failure

Combined Flowchart for Child Growth Failure and Protein Energy Malnutrition

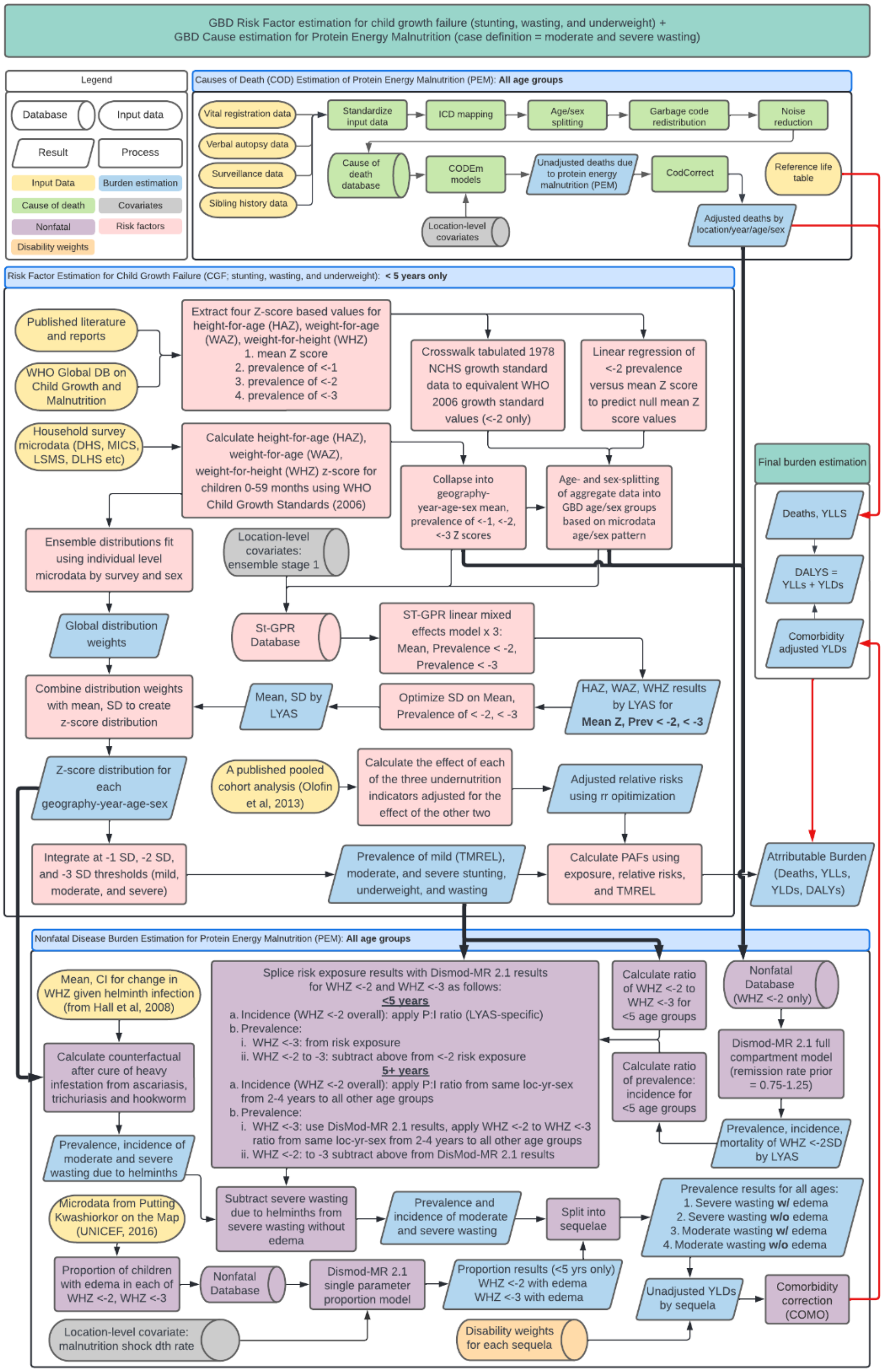

Input data and methodological summary

Exposure

Case definition

Child growth failure (CGF) is estimated using three indicators (stunting, wasting, and underweight), all of which are based on categorical definitions using the WHO 2006 growth standards for children 0-59 months. Definitions are based on Z scores from the growth standards, which were derived from an international reference population. Mild (<-1 to -2 Z score), moderate (<-2 to -3 Z score), and severe (<3 Z score) categorical prevalences were estimated for each of the three indicators.

Input data

There are three main inputs for the GBD child growth failure models: microdata from population surveys, tabulated data from reports and published literature, and the WHO Global Database on Child Growth and Malnutrition.[47] The primary data additions in GBD 2021 for child growth failure were from population surveys that include anthropometry. Population surveys include a variety of multi-country and country-specific survey series such as Multiple Indicator Cluster Surveys (MICS), Demographic and Health Surveys (DHS), Living Standards Measurement Surveys (LSMS), and the China Health and Nutrition Survey (CHNS), as well as other one-time country-specific surveys such as the Indonesia Family Life Survey and the Brazil National Demographic and Health Survey of Children and Women. These microdata contain information about each individual child's age (from which age in weeks and age in months are calculated), as well as height and/or weight. From that information, a height-for-age z-score (HAZ), weight-for-age z-score (WAZ), and weight-for-height z-score (WHZ) are calculated using the WHO 2006 Child Growth Standards and the LMS method.[48] Data that did not meet the following three criteria were dropped: 1) non-sex-specific data, 2) data with invalid Z-scores (HAZ, WAZ, WHZ, or BMI above 6 SD or below -6 SD), and 3) data with impossible values (negative height, weight, or age).

All available data from the WHO Global Database on Child Growth and Malnutrition were extracted in GBD 2016 – much of which are from published studies. Exclusions included examination date prior to 1985, non-population-representative studies, and those based on self-report. A systematic literature review was last completed in GBD 2010. We looked for four metrics from all sources with tabulated data: mean Z score, prevalence <-1 Z score, prevalence <-2 Z score, and prevalence <-3 Z score. All data for each metric were extracted for each of stunting (height-for-age Z score; HAZ), wasting (weight-for-height Z score; WHZ), and underweight (weight-for-age Z score; WAZ).

Table 1: Input data counts for Child wasting exposure models

Input data	Exposure
Source count (total)	1908
Number of countries with data	159

Table 2: Input data counts for Child underweight exposure models

Input data	Exposure
Source count (total)	1897
Number of countries with data	160

Table 3: Input data counts for Child stunting exposure models

Input data	Exposure
Source count (total)	1897
Number of countries with data	160

Data processing

To maximise internal consistency and comprehensiveness of the modelling dataset, we performed three data transformations. First, any data that were reported using the National Center for Health Statistics (NCHS) 1978 growth standards were crosswalked to corresponding values on the WHO 2006 Growth Standards curves based on a study that evaluated growth standard concordance.[49] Crosswalks from 1978 to 2006 growth standards were performed using OLS linear regression only on <-2 (ie, moderate) prevalence data, as that is where the concordance was most consistent. Second, for any study that lacked a measure of mean Z score for any of stunting, wasting, or underweight, we predicted a mean value for that study based on an ordinary-least-squares regression of mean Z score versus <-2 prevalence for that metric from all sources where both were available. Third, for any data that were presented as both sexes combined or for 0-59 months combined, we used the age and sex pattern from all data sources that included that detail to split into corresponding and age- and sex-specific data.

Exposure estimation

The following four-step modelling process was applied in parallel to each of stunting, wasting, and underweight.

First, all microdata were fit using an ensemble modelling. A series of 10 individual distributions (normal, log-normal, log-logistic, exponential, gamma, mirror gamma, inverse gamma, gumbel, mirror gumbel, and Weibull) were fit simultaneously to each microdata source in the dataset. All component distributions that were used to derive weights were parameterised using "method of moments," meaning that each corresponding probability density function (PDF) could be described as a function of the mean and variance of the quantity of interest. From these distribution families, an ensemble distribution was parameterised using an updated methodology for GBD 2021, which has 2 main advantages over GBD 2019 methodology. Those advantages are described below.

The new ensemble modeling strategy is considered an advancement, in part, because the models were specifically fit on the portions of the distributions that constitute mild, moderate, and severe CGF. While

previous methods aimed to minimise predictive error across the entire distribution, the new GBD 2021 method aimed to minimise absolute prediction error in highly relevant areas of the curve.

The second advancement is that the optimisation process considers the fit across all microdata sources simultaneously. Therefore, the algorithm targets the set of ensemble weights that minimises the predictive error across all microdata sources collectively, as opposed to finding one set of weights for each individual microdata source and averaging those sets of weights together.

After ensemble distributions have been parameterised, the second modeling step begins. Models were developed for mean Z scores and prevalence of moderate and severe growth failure. Individual-level microdata were collapsed to calculate three metrics: mean z-score, moderate prevalence, and severe prevalence. These data were combined with those derived from literature, GHDx review, and the WHO Global Database on Child Growth and Malnutrition. Each of the three metrics was then modelled using spatiotemporal Gaussian process regression (ST-GPR), a common modelling framework used across GBD, generating estimates for each age group, sex, year, and location. Location-level covariates used in all models included Socio-demographic Index (SDI) and logit-transformed proportion of households with improved sanitation.

Third, we combined estimates of mean, prevalence (moderate and severe) with ensemble weights in an optimisation framework in order to derive the variance that would best correspond to the predicted mean and prevalence. This variance was then paired with the mean and, using the method of moments equation for each of the component distributions of the ensemble, PDF of the distribution of Z-scores were calculated for each location, year, age group, and sex.

Fourth, PDFs were integrated to determine the prevalence between -1 and -2 Z scores (mild), between -2 and -3 Z scores (moderate), and below -3 Z scores (severe). These were categorical exposures used for subsequent attributable risk analysis.

Theoretical minimum-risk exposure level

Theoretical minimum risk exposure level (TMREL) for underweight, stunting, and wasting was assigned to be greater than or equal to -1 SD of the WHO 2006 standard weight-for-age, height-for-age, and weight-for-height curves, respectively. This has not changed since GBD 2010.

Relative risks

The final list of outcomes paired with child growth failure risks included mortality and morbidity for lower respiratory infections (LRI), diarrhoea, malaria, measles, and protein-energy malnutrition (PEM), as shown in Table 6. These were derived from a Burden of Proof analysis that incorporated both a pooled analysis of ten prospective cohort studies by Olofin and colleagues as well as relative risk estimates from Knowledge Integration (KI) studies (Table 5). For the KI studies, aggregated relative risks of disease or cause-specific mortality were calculated for 1-unit z-score bins for stunting, wasting, and underweight (e.g., relative risk of diarrhea-attributable death in children 1 to 2 years of age and with a HAZ score between -4 and -3). The burden of proof analysis uses all available relative risks with corresponding uncertainty to create continuous relative risk curves for each outcome/risk pair. These continuous risk curves are then combined with the global exposure curves for HAZ, WAZ, and WHZ, to calculate exposure-weighted relative risks for severe, moderate, and mild stunting, wasting, and underweight with uncertainty. Of historical note, upper respiratory infections and otitis media were

included as outcomes in the GBD 2013 risk analysis, based on the “analogy” causal criterion, assuming there is similar pathway as LRI outcome. However, closer review for GBD 2015 did not find sufficient evidence to support their inclusion and they were excluded, a decision that was carried forward into GBD 2016. We also attributed 100% of PEM to childhood wasting and underweight but not stunting. To build on the existing literature base for GBD on riskoutcome pairs, a literature search was conducted for GBD 2017 searching for case-control studies published after January 1, 1985 did not return any sources that were usable.

Table 4: Input data counts for Child growth failure relative risk models

Input data	Risk
Source count (total)	53

There is a high degree of correlation between stunting, wasting, and underweight. Failing to account for their covariance and assuming independence would overestimate the total burden significantly and misrepresent the attributable burden of individual CGF indicators. Inability to address these correlations is the main reason that GBD 2010 only included childhood underweight.

In order to account for the high degree of correlation between CGF indicators, GBD uses a constrained optimisation method to adjust the observed univariate RRs that come out of the Burden of Proof analysis. First we created a joint distribution of stunting, underweight, and wasting from a population of children. Second, we generated one thousand RR draws for each univariate indicator and severity based from the Burden of Proof analysis. Third, we altered these univariate RRs for the four causes (diarrhoea, LRI, malaria, and measles) and the two outcomes (mortality and morbidity) based upon interactions among the CGF indicators. An interaction occurs when the effect of one CGF indicator variable (eg, stunting) has a different effect on the outcome depending on the value of another CGF indicator variable (eg, underweight). Interaction terms alter the risk of the outcome among children with more than one indicator of CGF. These interaction terms were extracted from a pooled cohort analysis of all-cause mortality published by McDonald et al. [50] Lastly, we optimised the adjusted relative risks by minimising the error between the observed RRs (generated from Olofin et al.) and the altered RRs derived from the joint distribution and accounting for the interaction terms while ensuring that no alteration resulted in a previously identified increase in relative risk becoming protective.

For GBD 2021, we made several changes to improve the four main steps of RR adjustment. From GBD 2013 to GBD 2019, a simulated joint distribution of stunting, underweight, and wasting measures was created from the Olofin et al. meta-analysis. Sources in this meta-analysis were cross-sectional Demographic and Health Surveys (DHS). In GBD 2021, we created age-specific joint distributions of stunting, underweight, and wasting measures from 15 longitudinal studies (from 26 locations) in the Bill and Melinda Gates Foundation’s Knowledge Integration (Ki) database[6]. (Study details are provided in Table 5). The RR adjustment method was strengthened in GBD 2021 by constraining optimisation in two ways. Optimisation was only permitted to alter the RR for an indicator/severity in draws where the observed RR was greater than 1, and constraints were placed on the error that penalise larger alterations to the RR. These changes enabled the estimation and utilisation of age-specific adjusted RRs for GBD 2021 burden estimation. The largest changes for GBD 2021 was conducting Burden of Proof

Analyses for each cause/outcome/risk triplet using both data from Olofin et al as well as KI data. These changes result in identifying large differences in the relationship between CGF and mortality versus morbidity as well as identifying some impact of CGF on malaria.

Study name	Country	Sample	Years conducted
Zimbabwe Vitamin A for Mothers and Babies Trial	ZWE	14,110	1997-2001
CMC Vellore Birth Cohort Study	IND	373	2002-2006
International Lipid-Based Nutrient Supplements Project	MWI	1,206	2011-2014
Malnutrition and Enteric Disease Study	BGD	265	2009-2017
Malnutrition and Enteric Disease Study	IND	251	2009-2017
Malnutrition and Enteric Disease Study	NEP	240	2009-2017
Malnutrition and Enteric Disease Study	PER	303	2009-2017
Malnutrition and Enteric Disease Study	BRA	233	2009-2017
Malnutrition and Enteric Disease Study	ZAF	314	2009-2017
Malnutrition and Enteric Disease Study	TZA	262	2009-2017
Medical Research Council Keneba	GMB	2,867	-
Performance of Rotavirus and Oral Polio Vaccines In Developing Countries	BGD	700	2011-2014
Community-based Intervention Trial to Compare the Impact of Preventive and Therapeutic Zinc Supplementation Programs Among Young Children in Burkina Faso	BFA	7,634	2010-2012
WASH Benefits Bangladesh	BGD	4,423	2011-2014
WASH Benefits Kenya	KEN	5,649	2012-2016
Promotion of Breastfeeding Intervention Trial	BLR	16,897	1996-1998
Childhood Malnutrition and Infection Network	BGD	477	1993-1996
Childhood Malnutrition and Infection Network	BRA	119	1989-1998
Childhood Malnutrition and Infection Network	GNB	350	1987-1990
Childhood Malnutrition and Infection Network	GNB	885	1996-1997
Childhood Malnutrition and Infection Network	PER	210	1989-1991
Childhood Malnutrition and Infection Network	PER	224	1995-1998
Delhi Infant Vitamin D Study	IND	2,100	2007-2010

Characterization of Respiratory pathogens endemic to Pakistan in pregnant women and newborns in urban settings	PAK	380	2012-2013
Impact of Zinc Supplementation in Low Birth Weight Infants on Severe Morbidity, Mortality and Zinc Status: A Randomized Controlled Trial	IND	2,052	2005-2007
A Trial of Zinc and Micronutrients in Tanzanian Children	TZA	2,400	2007-2012

Table 5: Bill and Melinda Gates Foundation Knowledge Integration (KI) database study details

Table 6: Age-Specific Adjusted RRs for each riskoutcome pair for child growth failure

**1 to 5 months**		**Incidence**			**Mortality**		
**Cause**		**<-3**	**-3,-2**	**-2,-1**	**<-3**	**-3,-2**	**-2,-1**
**Diarrhoea**	HAZ	1.2 (0.8, 1.7)	1.2 (0.8, 1.6)	1.1 (0.9, 1.5)	3.6 (2.1, 4.4)	2.1 (1.6, 2.6)	1.4 (1.2, 1.6)
	WAZ	1.6 (0.9, 2.7)	1.6 (0.9, 2.6)	1.5 (0.9, 2.4)	6.7 (4.4, 9.3)	3.4 (2.2, 4.7)	1.8 (1.3, 2.2)
	WHZ	1.3 (0.8, 1.8)	1.2 (0.9, 1.7)	1.2 (0.9, 1.6)	40.8 (0.8, 224.7)	12.8 (0.8, 51.5)	4.1 (0.9, 10.6)
**LRI**	HAZ	1.3 (0.6, 2.5)	1.1 (0.7, 1.8)	1.1 (0.8, 1.5)	5.1 (2.7, 7.6)	2.9 (1.7, 4.2)	1.8 (1.2, 2.4)
	WAZ	1.6 (0.6, 4.1)	1.3 (0.7, 2.2)	1.2 (0.8, 1.7)	31.1 (1.0, 191.4)	13.9 (1.0, 69.2)	4.9 (1.0, 16.8)
	WHZ	1.3 (0.9, 1.8)	1.1 (1.0, 1.2)	1.0 (1.0, 1.1)	6.5 (4.5, 8.8)	3.6 (2.6, 4.6)	1.8 (1.5, 2.1)
**Malaria**	HAZ	1.0 (1.0, 1.0)	1.0 (1.0, 1.0)	1.0 (1.0, 1.0)	3.0 (0.6, 12.4)	1.1 (0.9, 1.2)	1.0 (1.0, 1.0)
	WAZ	2.3 (0.6, 6.6)	2.3 (0.6, 6.6)	2.3 (0.6, 6.6)	2.7 (0.6, 8.4)	1.8 (0.7, 3.9)	1.5 (0.8, 2.5)

	WHZ	1.0 (1.0, 1.0)	1.0 (1.0, 1.0)	1.0 (1.0, 1.0)	1.0 (1.0, 1.0)	1.0 (1.0, 1.0)	1.0 (1.0, 1.0)
**Measles**	HAZ	1.3 (0.9, 2.3)	1.0 (1.0, 1.0)	1.0 (1.0, 1.0)	4.1 (1.8, 5.7)	2.2 (1.4, 2.9)	1.4 (1.1, 1.5)
	WAZ	1.0 (1.0, 1.0)	1.0 (1.0, 1.0)	1.0 (1.0, 1.0)	5.1 (2.2, 7.8)	2.7 (1.6, 3.5)	1.4 (1.2, 1.6)
	WHZ	1.7 (0.8, 3.3)	1.3 (0.9, 1.8)	1.3 (0.9, 1.8)	6.7 (2.5, 15.4)	2.6 (1.6, 4.2)	1.2 (1.1, 1.4)
**PEM**	HAZ		0% PAF			0% PAF	
	WAZ		100% PAF			100% PAF	
	WHZ		100% PAF			100% PAF	
**6 to 11 months**		**Incidence**			**Mortality**		
**Cause**		**<-3**	**-3,-2**	**-2,-1**	**<-3**	**-3,-2**	**-2,-1**
**Diarrhoea**	HAZ	1.2 (0.8, 1.7)	1.2 (0.8, 1.6)	1.1 (0.9, 1.5)	3.1 (2.2, 3.9)	1.9 (1.5, 2.4)	1.3 (1.2, 1.6)
	WAZ	1.6 (0.9, 2.7)	1.6 (0.9, 2.6)	1.5 (0.9, 2.4)	5.9 (3.7, 8.9)	3.1 (2.0, 4.5)	1.7 (1.3, 2.2)
	WHZ	1.3 (0.8, 1.8)	1.2 (0.9, 1.7)	1.2 (0.9, 1.6)	40.7 (0.8, 224.7)	12.7 (0.8, 51.5)	4.1 (0.9, 10.6)
**LRI**	HAZ	1.3 (0.6, 2.5)	1.1 (0.7, 1.8)	1.1 (0.8, 1.5)	4.3 (2.2, 6.8)	2.6 (1.5, 3.9)	1.7 (1.2, 2.3)
	WAZ	1.6 (0.6, 4.1)	1.3 (0.7, 2.2)	1.2 (0.8, 1.7)	31.0 (1.0, 191.4)	13.8 (1.0, 69.2)	4.9 (1.0, 16.8)
	WHZ	1.3 (0.9, 1.8)	1.1 (1.0, 1.2)	1.0 (1.0, 1.1)	6.2 (4.3, 8.4)	3.5 (2.5, 4.5)	1.8 (1.5, 2.1)
**Malaria**	HAZ	1.0 (1.0, 1.0)	1.0 (1.0, 1.0)	1.0 (1.0, 1.0)	3.0 (0.6, 12.4)	1.1 (0.9, 1.2)	1.0 (1.0, 1.0)
	WAZ	2.3 (0.6, 6.6)	2.3 (0.6, 6.6)	2.3 (0.6, 6.6)	2.7 (0.6, 8.4)	1.8 (0.7, 3.9)	1.5 (0.8, 2.5)
	WHZ	1.0 (1.0, 1.0)	1.0 (1.0, 1.0)	1.0 (1.0, 1.0)	1.0 (1.0, 1.0)	1.0 (1.0, 1.0)	1.0 (1.0, 1.0)
**Measles**	HAZ	1.3 (0.9, 2.3)	1.0 (1.0, 1.0)	1.0 (1.0, 1.0)	3.6 (1.8, 4.9)	2.1 (1.4, 2.6)	1.3 (1.1, 1.5)
	WAZ	1.0 (1.0, 1.0)	1.0 (1.0, 1.0)	1.0 (1.0, 1.0)	5.2 (2.2, 8.1)	2.7 (1.6, 3.6)	1.4 (1.2, 1.6)
	WHZ	1.7 (0.8, 3.3)	1.3 (0.9, 1.8)	1.3 (0.9, 1.8)	6.7 (2.5, 15.4)	2.6 (1.6, 4.2)	1.2 (1.1, 1.4)
**PEM**	HAZ		0% PAF			0% PAF	

	WAZ		100% PAF			100% PAF	
	WHZ		100% PAF			100% PAF	

**12 to 23 months**		**Incidence**			**Mortality**		
**Cause**		**<-3**	**-3,-2**	**-2,-1**	**<-3**	**-3,-2**	**-2,-1**
**Diarrhea**	HAZ	1.2 (0.8, 1.7)	1.2 (0.8, 1.6)	1.1 (0.9, 1.5)	2.7 (2.2, 3.4)	1.7 (1.4, 2.2)	1.3 (1.1, 1.5)
	WAZ	1.6 (0.9, 2.7)	1.6 (0.9, 2.6)	1.5 (0.9, 2.4)	5.4 (3.7, 8.4)	2.9 (2.0, 4.4)	1.6 (1.3, 2.1)
	WHZ	1.3 (0.8, 1.8)	1.2 (0.9, 1.7)	1.2 (0.9, 1.6)	40.7 (0.8, 225.5)	12.7 (0.8, 51.7)	4.0 (0.9, 10.6)
**LRI**	HAZ	1.3 (0.6, 2.5)	1.1 (0.7, 1.8)	1.1 (0.8, 1.5)	4.1 (2.3, 5.9)	2.5 (1.5, 3.5)	1.6 (1.2, 2.2)
	WAZ	1.6 (0.6, 4.1)	1.3 (0.7, 2.2)	1.2 (0.8, 1.7)	29.3 (1.0, 186.4)	13.0 (1.0, 67.8)	4.6 (1.0, 16.5)
	WHZ	1.3 (0.9, 1.8)	1.1 (1.0, 1.2)	1.0 (1.0, 1.1)	5.4 (3.5, 7.6)	3.1 (2.1, 4.2)	1.7 (1.3, 2.0)
**Malaria**	HAZ	1.0 (1.0, 1.0)	1.0 (1.0, 1.0)	1.0 (1.0, 1.0)	3.0 (0.6, 12.4)	1.1 (0.9, 1.2)	1.0 (1.0, 1.0)
	WAZ	2.3 (0.6, 6.6)	2.3 (0.6, 6.6)	2.3 (0.6, 6.6)	2.7 (0.6, 8.4)	1.8 (0.7, 3.9)	1.5 (0.8, 2.5)
	WHZ	1.0 (1.0, 1.0)	1.0 (1.0, 1.0)	1.0 (1.0, 1.0)	1.0 (1.0, 1.0)	1.0 (1.0, 1.0)	1.0 (1.0, 1.0)
**Measles**	HAZ	1.3 (0.9, 2.3)	1.0 (1.0, 1.0)	1.0 (1.0, 1.0)	3.5 (1.8, 4.4)	2.0 (1.4, 2.5)	1.3 (1.1, 1.4)
	WAZ	1.0 (1.0, 1.0)	1.0 (1.0, 1.0)	1.0 (1.0, 1.0)	4.6 (2.2, 6.1)	2.5 (1.6, 3.0)	1.4 (1.2, 1.5)
	WHZ	1.7 (0.8, 3.3)	1.3 (0.9, 1.8)	1.3 (0.9, 1.8)	6.7 (2.5, 15.4)	2.6 (1.6, 4.2)	1.2 (1.1, 1.4)
**PEM**	HAZ		0% PAF			0% PAF	
	WAZ		100% PAF			100% PAF	
	WHZ		100% PAF			100% PAF	

2 to 4 years		Incidence			Mortality		
Cause		<-3	-3,-2	-2,-1	<-3	-3,-2	-2,-1
Diarrhoea	HAZ	1.2 (0.8, 1.7)	1.2 (0.8, 1.6)	1.1 (0.9, 1.5)	2.8 (2.1, 3.4)	1.8 (1.5, 2.2)	1.3 (1.2, 1.5)
	WAZ	1.6 (0.9, 2.7)	1.6 (0.9, 2.6)	1.5 (0.9, 2.4)	5.6 (4.0, 8.3)	2.9 (2.1, 4.3)	1.6 (1.3, 2.1)
	WHZ	1.3 (0.8, 1.8)	1.2 (0.9, 1.7)	1.2 (0.9, 1.6)	41.3 (0.8, 229.9)	12.8 (0.8, 52.4)	4.0 (0.9, 10.7)
LRI	HAZ	1.3 (0.6, 2.5)	1.1 (0.7, 1.8)	1.1 (0.8, 1.5)	9.5 (4.7, 21.2)	4.7 (3.0, 8.6)	2.5 (2.0, 3.8)
	WAZ	1.6 (0.6, 4.1)	1.3 (0.7, 2.2)	1.2 (0.8, 1.7)	14.5 (1.0, 60.4)	7.1 (1.0, 21.7)	3.1 (1.0, 5.6)
	WHZ	1.3 (0.9, 1.8)	1.1 (1.0, 1.2)	1.0 (1.0, 1.1)	5.1 (2.3, 7.9)	3.0 (1.5, 4.3)	1.6 (1.2, 2.1)
Malaria	HAZ	1.0 (1.0, 1.0)	1.0 (1.0, 1.0)	1.0 (1.0, 1.0)	3.0 (0.6, 12.4)	1.1 (0.9, 1.2)	1.0 (1.0, 1.0)
	WAZ	2.3 (0.6, 6.6)	2.3 (0.6, 6.6)	2.3 (0.6, 6.6)	2.7 (0.6, 8.4)	1.8 (0.7, 3.9)	1.5 (0.8, 2.5)
	WHZ	1.0 (1.0, 1.0)	1.0 (1.0, 1.0)	1.0 (1.0, 1.0)	1.0 (1.0, 1.0)	1.0 (1.0, 1.0)	1.0 (1.0, 1.0)
Measles	HAZ	1.3 (0.9, 2.3)	1.0 (1.0, 1.0)	1.0 (1.0, 1.0)	3.5 (1.8, 4.4)	2.0 (1.4, 2.5)	1.3 (1.1, 1.4)
	WAZ	1.0 (1.0, 1.0)	1.0 (1.0, 1.0)	1.0 (1.0, 1.0)	4.6 (2.2, 6.1)	2.5 (1.6, 3.0)	1.4 (1.2, 1.5)
	WHZ	1.7 (0.8, 3.3)	1.3 (0.9, 1.8)	1.3 (0.9, 1.8)	6.7 (2.5, 15.4)	2.6 (1.6, 4.2)	1.2 (1.1, 1.4)
PEM	HAZ		0% PAF			0% PAF	
	WAZ		100% PAF			100% PAF	
	WHZ		100% PAF			100% PAF	

References

1 Stevens GA, Alkema L, Black RE. Guidelines for Accurate and Transparent Health Estimates Reporting: the GATHER statement. *Lancet* 2016.

2 Lim SS, Vos T, Flaxman AD. A comparative risk assessment of burden of disease and injury attributable to 67 risk factors and risk factor clusters in 21 regions, 1990–2010: a systematic analysis for the Global Burden of Disease Study 2010. *The Lancet* 2012; **380**: 2224–60.

3 Forouzanfar M, Afshin A, Alexander LT, Anderson H, Bhutta Z, Murray CJL. Global, regional, and national comparative risk assessment of 79 behavioural, environmental and occupational, and metabolic risks or clusters of risks, 1990-2015: a systematic analysis for the Global Burden of Disease Study 2015. *Lancet* 2016; **388**: 1659–724.

4 Gakidou E, Afshin A, Abajobir AA, *et al.* Global, regional, and national comparative risk assessment of 84 behavioural, environmental and occupational, and metabolic risks or clusters of risks, 1990–2016: a systematic analysis for the Global Burden of Disease Study 2016. *The Lancet* 2017; **390**: 1345–422.

5 Stanaway JD, Afshin A, Gakidou E, *et al.* Global, regional, and national comparative risk assessment of 84 behavioural, environmental and occupational, and metabolic risks or clusters of risks for 195 countries and territories, 1990–2017: a systematic analysis for the Global Burden of Disease Study 2017. *The Lancet* 2018; **392**: 1923–94.

6 Murray CJL, Aravkin AY, Zheng P. Global burden of 87 risk factors in 204 countries and territories, 1990–2019: a systematic analysis for the Global Burden of Disease Study 2019. *The Lancet* 2020; **396**: 1223–49.

7 Murray CJ, Lopez AD. Global mortality, disability, and the contribution of risk factors: Global Burden of Disease Study. *Lancet* 1997; **349**: 1436–42.

8 Murray CJ, Lopez AD. On the comparable quantification of health risks: lessons from the Global Burden of Disease Study. *Epidemiology* 1999; **10**: 594–605.

9 Food, nutrition, physical activity and the prevention of cancer: a global perspective. Washington, D.C: World Cancer Research Fund & American Institute for Cancer Research, 2007.

10 Zheng P, Barber R, Sorensen RJ, Murray CJ, Aravkin AY. Trimmed constrained mixed effects models: formulations and algorithms. *Journal of Computational and Graphical Statistics* 2021; : 1–13.

11 Zheng P. limetr: limetr: linear mixed effects model with trimming. https://github.com/zhengp0/limetr (accessed July 28, 2021).

12 Zheng P. xspline: xspline: Advanced spline tools. https://github.com/zhengp0/xspline (accessed July 28, 2021).

13 Viechtbauer W. Conducting meta-analyses in R with the metafor package. *Journal of statistical software* 2010; **36**: 1–48.

14 de Boor C. A practical guide to splines (applied mathematical sciences, 27). New York: Springer, 2001 https://link.springer.com/book/9780387953663 (accessed April 4, 2022).

15 Rousseeuw PJ, Leroy AM. Robust regression and outlier detection. John wiley & sons, 2005.

16 Rousseeuw PJ. Least median of squares regression. *Journal of the American Statistical Association* 1984; **79**: 871–80.

17 Huber PJ. Robust Statistics. John Wiley & Sons, 2004.

18 Rousseeuw P. Multivariate estimation with high breakdown point. *Mathematical Statistics and Applications Vol B* 1985; : 283–97.

19 Aravkin A, Davis D. Trimmed statistical estimation via variance reduction. *Mathematics of Operations Research* 2020; **45**: 292–322.

20 Motzkin TS, Raiffa H, Thompson GL, Thrall RM. 3. The Double Description Method. In: 3. The Double Description Method. Princeton University Press, 2016: 51–74.

21 Guyatt GH, Oxman AD, Vist G, *et al.* GRADE guidelines: 4. Rating the quality of evidence—study limitations (risk of bias). *Journal of clinical epidemiology* 2011; **64**: 407–15.

22 Efron B, Hastie T, Johnstone I, Tibshirani R. Least angle regression. *The Annals of statistics* 2004; **32**: 407–99.

23 Tibshirani R. Regression shrinkage and selection via the lasso. *Journal of the Royal Statistical Society: Series B (Methodological)* 1996; **58**: 267–88.

24 Kontopantelis E, Springate DA, Reeves D. A re-analysis of the Cochrane Library data: the dangers of unobserved heterogeneity in meta-analyses. *PloS one* 2013; **8**: e69930.

25 Ioannidis JP, Patsopoulos NA, Evangelou E. Uncertainty in heterogeneity estimates in meta-analyses. *Bmj* 2007; **335**: 914–6.

26 Biggerstaff BJ, Tweedie RL. Incorporating variability in estimates of heterogeneity in the random effects model in meta-analysis. *Statistics in medicine* 1997; **16**: 753–68.

27 Higgins JP, Thompson SG. Quantifying heterogeneity in a meta-analysis. *Statistics in medicine* 2002; **21**: 1539–58.

28 Sterne JA, Egger M. Chapter 6: Regression methods to detect publication and other bias in meta-analysis. In: Rothstein H, Sutton A, Borenstein M, eds. Publication bias in meta-analysis: Prevention, assessment and adjustments. John Wiley & Sons, Ltd, 2005.

29 Egger M, Smith GD, Schneider M, Minder C. Bias in meta-analysis detected by a simple, graphical test. *BMJ* 1997; **315**: 629–34.

30 Burkart KG, Brauer M, Aravkin AY. Estimating the cause-specific relative risks of non-optimal temperature on daily mortality: a two-part modelling approach applied to the Global Burden of Disease Study. *The Lancet* 2021; **398**: 685–97.

31 Harris PA, Taylor R, Thielke R, Payne J, Gonzalez N, Conde JG. Research Electronic Data Capture (REDCap) - A metadata-driven methodology and workflow process for providing translational research informatics support. *J Biomed Inform* 2009; **42**: 377–81.

32 GBD 2015 Diseases and Injury Incidence and prevalence Collaborators. Global, regional, and national incidence, prevalence, and years lived with disability (YLDs) for 310 acute and chronic diseases and injuries, 1990-2015: a systematic analysis for the Global Burden of Disease Study 2015. *The Lancet Under review*.

33 Flaxman AD, Vos T, Murray CJL, Kiyono P, editors. An integrative metaregression framework for descriptive epidemiology, 1 edition. Seattle: University of Washington Press, 2015.

34 Vasudevan S, Ramos F, Nettleton E, Durrant-Whyte H, Blair A. Gaussian Process modeling of large scale terrain. In: 2009 IEEE International Conference on Robotics and Automation. 2009: 1047–53.

35 Rasmussen CE, Williams CKI. Gaussian Processes for Machine Learning. Cambridge, Mass: The MIT Press, 2005.

36 Ng M, Fleming T, Robinson M, *et al.* Global, regional, and national prevalence of overweight and obesity in children and adults during 1980–2013: a systematic analysis for the Global Burden of Disease Study 2013. *Lancet* 2014; **384**: 766–81.

37 Ng M, Freeman MK, Fleming TD, *et al.* Smoking Prevalence and Cigarette Consumption in 187 Countries, 1980-2012. *JAMA* 2014; **311**: 183–92.

38 Massey Jr FJ. The Kolmogorov-Smirnov test for goodness of fit. *Journal of the American statistical Association* 1951; **46**: 68–78.

39 Vander Hoorn S, Ezzati M, Rodgers A, Lopez AD, Murray CJL. Estimating attributable burden of disease from exposure and hazard data. In: Comparative Quanitifcation of Health Risks: Global and regional burden of disease attribution to selected major risk factors. World Health Organisation, 2004: 2129–40.

40 Preston SH. Causes and Consequences of Mortality Declines in Less Developed Countries during the Twentieth Century. In: Population and economic change in developing countries. Chicago: Univ. of Chicago Pr, 1980: 289–360.

41 Carnahan E, Lim SS, Nelson EC, *et al.* Validation of a new predictive risk model: measuring the impact of major modifiable risks of death for patients and populations. *The Lancet* 2013; **381**: S26.

42 Danaei G, Singh GM, Paciorek CJ, *et al.* The global cardiovascular risk transition: associations of four metabolic risk factors with national income, urbanization, and Western diet in 1980 and 2008. *Circulation* 2013; **127**: 1493–502, 1502e1-8.

43 McDonald CM, Olofin I, Flaxman S. The effect of multiple anthropometric deficits on child mortality: meta-analysis of individual data in 10 prospective studies from developing countries. *Am J Clin Nutr* 2013; **97**: 896–901.

44 Olofin I, McDonald CM, Ezzati M, et al. Associations of suboptimal growth with all-cause and cause specific mortality in children under five years: a pooled analysis of ten prospective studies. *PloS ONE*; **8**: e64636.

45 Ki Global Health. (n.d.). https://www.kiglobalhealth.org/.

46 Das Gupta P. Standardization and Decomposition of Rates: A User's Manual. Washington D.C.: U.S. Bureau of the Census, 1993.

47 WHO | WHO Global Database on Child Growth and Malnutrition. WHO. http://www.who.int/nutgrowthdb/en/ (accessed July 30, 2018).

48 Wang Y, Chen H-J. Use of Percentiles and Z-Scores in Anthropometry. In: Preedy VR, ed. Handbook of Anthropometry. New York, NY: Springer New York, 2012: 29–48.

49 Uribe Á, Cecilia M, López Gaviria A, Estrada Restrepo A. Concordance between Z scores from WHO 2006 and the NCHS 1978 growth standards of children younger than five. Antioquia-Colombia. *Perspectivas en Nutrición Humana* 2008; **10**: 177–87.

50 McDonald CM, Olofin I, Flaxman S, *et al.* The effect of multiple anthropometric deficits on child mortality: meta-analysis of individual data in 10 prospective studies from developing countries. *Am J Clin Nutr* 2013; **97**: 896–901.

51 Cole TJ, Lobstein T. Extended international (IOTF) body mass index cut-offs for thinness, overweight and obesity. *Pediatr Obes* 2012; **7**: 284–94.

52 Global, regional, and national comparative risk assessment of 79 behavioural, environmental and occupational, and metabolic risks or clusters of risks, 1990–2015: a systematic analysis for the Global Burden of Disease Study 2015. *Lancet* 2016; **388**: 1659–724.

53 Stanaway JD, Afshin A, Gakidou E, *et al.* Global, regional, and national comparative risk assessment of 84 behavioural, environmental and occupational, and metabolic risks or clusters of risks for 195 countries and territories, 1990–2017: a systematic analysis for the Global Burden of Disease Study 2017. *The Lancet* 2018; **392**: 1923–94.

54 Global Health Data Exchange | GHDx. http://ghdx.healthdata.org/ (accessed Oct 8, 2021).

55 Friedewald WT, Levy RI, Fredrickson DS. Estimation of the concentration of low-density lipoprotein cholesterol in plasma, without use of the preparative ultracentrifuge. *Clin Chem* 1972; **18**: 499–502.

56 Murray CJL, Aravkin AY, Zheng P, *et al.* Global burden of 87 risk factors in 204 countries and territories, 1990–2019: a systematic analysis for the Global Burden of Disease Study 2019. *The Lancet* 2020; **396**: 1223–49.

57 Singh GM, Danaei G, Farzadfar F, *et al.* The Age-Specific Quantitative Effects of Metabolic Risk Factors on Cardiovascular Diseases and Diabetes: A Pooled Analysis. *PLOS ONE* 2013; **8**: e65174.

58 Wang N, Fulcher J, Abeysuriya N, *et al.* Intensive LDL cholesterol-lowering treatment beyond current recommendations for the prevention of major vascular events: a systematic review and meta-analysis of randomised trials including 327 037 participants. *Lancet Diabetes Endocrinol* 2020; **8**: 36–49.

59 Moher D, Liberati A, Tetzlaff J, Altman DG, Group TP. Preferred Reporting Items for Systematic Reviews and Meta-Analyses: The PRISMA Statement. *PLOS Medicine* 2009; **6**: e1000097.

60 Ng M, Fleming T, Robinson M, *et al.* Global, regional, and national prevalence of overweight and obesity in children and adults during 1980–2013: a systematic analysis for the Global Burden of Disease Study 2013. *The Lancet* 2014; **384**: 766–81.

61 Boekholdt SM, Hovingh GK, Mora S, *et al.* Very Low Levels of Atherogenic Lipoproteins and the Risk for Cardiovascular EventsA Meta-Analysis of Statin Trials. *J Am Coll Cardiol* 2014; **64**: 485–94.

62 Vos T, Lim SS, Abbafati C, *et al.* Global burden of 369 diseases and injuries in 204 countries and territories, 1990–2019: a systematic analysis for the Global Burden of Disease Study 2019. *The Lancet* 2020; **396**: 1204–22.

63 Zheng P, Barber R, Sorensen RJD, Murray CJL, Aravkin AY. Trimmed Constrained Mixed Effects Models: Formulations and Algorithms. *Journal of Computational and Graphical Statistics* 2021; **30**: 544–56.

64 Sorensen (rsoren@uw.edu) R. Health Metrics Toolbox. https://ihmeuw-msca.github.io/index.html (accessed Oct 8, 2021).

65 Bias in meta-analysis detected by a simple, graphical test | The BMJ. https://www.bmj.com/content/315/7109/629.full (accessed Oct 8, 2021).

66 Prospective Studies Collaboration. Collaborative overview ('meta-analysis') of prospective observational studies of the associations of usual blood pressure and usual cholesterol levels with common causes of death: protocol for the second cycle of the Prospective Studies Collaboration. *J Cardiovasc Risk* 1999; **6**: 315–20.

67 The effect of Blood Pressure on Kidney Failure: a systematic review and meta-analysis in 2.7 million participants [Unpublished] | GHDx. https://internal-ghdx.healthdata.org/record/effect-blood-pressure-kidney-failure-systematic-review-and-meta-analysis-27-million (accessed July 11, 2023).

68 Rapsomaniki E, Timmis A, George J, *et al.* Blood pressure and incidence of twelve cardiovascular diseases: lifetime risks, healthy life-years lost, and age-specific associations in 1·25 million people. *Lancet* 2014; **383**: 1899–911.

69 Denaxas SC, George J, Herrett E, *et al.* Data Resource Profile: Cardiovascular disease research using linked bespoke studies and electronic health records (CALIBER). *Int J Epidemiol* 2012; **41**: 1625–38.

70 Page MJ, McKenzie JE, Bossuyt PM, *et al.* The PRISMA 2020 statement: an updated guideline for reporting systematic reviews. *BMJ* 2021; **372**: n71.

71 Singh GM, Danaei G, Farzadfar F, *et al.* The age-specific quantitative effects of metabolic risk factors on cardiovascular diseases and diabetes: a pooled analysis. *PLoS ONE* 2013; **8**: e65174.

72 Collaboration APCS, others. Blood pressure and cardiovascular disease in the Asia Pacific region. *Journal of hypertension* 2003; **21**: 707–16.

73 Hersbach H, Bell B, Berrisford P, *et al.* Global reanalysis : goodbye Global reanalysis : goodbye ERA-Interim , hello. 2019. DOI:10.21957/vf291hehd7.

74 Copernicus Climate Change Service (C3S) (2017): ERA5: Fifth generation of ECMWF atmospheric reanalyses of the global climate. Copernicus Climate Change Service Climate Data Store (CDS), September 2019. .

75 Geography and Environmental Science, University of Southampton. Age and Sex Structures, Global Per Country 2000-2020 - WorldPop. Southampton , United Kingdom: Geography and Environmental Science, University of Southampton, 2018. .

76 Zheng P, Aravkin AY, Barber R, Sorensen RJD, Murray CJL. Trimmed Constrained Mixed Effects Models: Formulations and Algorithms. 2019; published online Sept 23.

77 Rousseeuw PJ. Least Median of Squares Regression. *Journal of the American Statistical Association* 1984; **79**: 871–80.

78 Aravkin A, Davis D. Trimmed Statistical Estimation via Variance Reduction. *Mathematics of Operations Research* 2019; : moor.2019.0992.

79 Yang E, Lozano AC, Aravkin A. A general family of trimmed estimators for robust high-dimensional data analysis. *Electronic Journal of Statistics* 2018; **12**: 3519–53.

80 Pya N, Wood SN. Shape constrained additive models. *Statistics and Computing* 2015; **25**: 543–59.

81 Roth GA, Abate D, Abate KH, *et al.* Global, regional, and national age-sex-specific mortality for 282 causes of death in 195 countries and territories, 1980-2017: a systematic analysis for the Global Burden of Disease Study 2017 GBD 2017 Causes of Death Collaborators*. 2018 DOI:10.1016/S0140-6736(18)32203-7.

82 Ng M, Freeman MK, Fleming TD, *et al.* Smoking prevalence and cigarette consumption in 187 countries, 1980-2012. *JAMA* 2014; **311**: 183–92.

83 Zheng P, Aravkin A, Barber R, Sorensen R, Murray C. Trimmed Constrained Mixed Effects Models: Formulations and Algorithms. *bioRxiv* 2020; : 2020.01.28.923599.

84 International Zinc Nutrition Consultative Group (IZiNCG), Brown KH, Rivera JA, *et al.* International Zinc Nutrition Consultative Group (IZiNCG) technical document #1. Assessment of the risk of zinc deficiency in populations and options for its control. *Food Nutr Bull* 2004; **25**: S99-203.

6 Jumbe NL, Murray JC, Kern S. Data Sharing and Inductive Learning – Toward Healthy Birth, Growth, and Development. N Engl J Med. 2016 Jun 23;374(25):2415-7. doj: 10.1056/NEJMp1605441. Epub 2016 May 11. PMID: 27168111.

Childhood sexual abuse

Flowchart

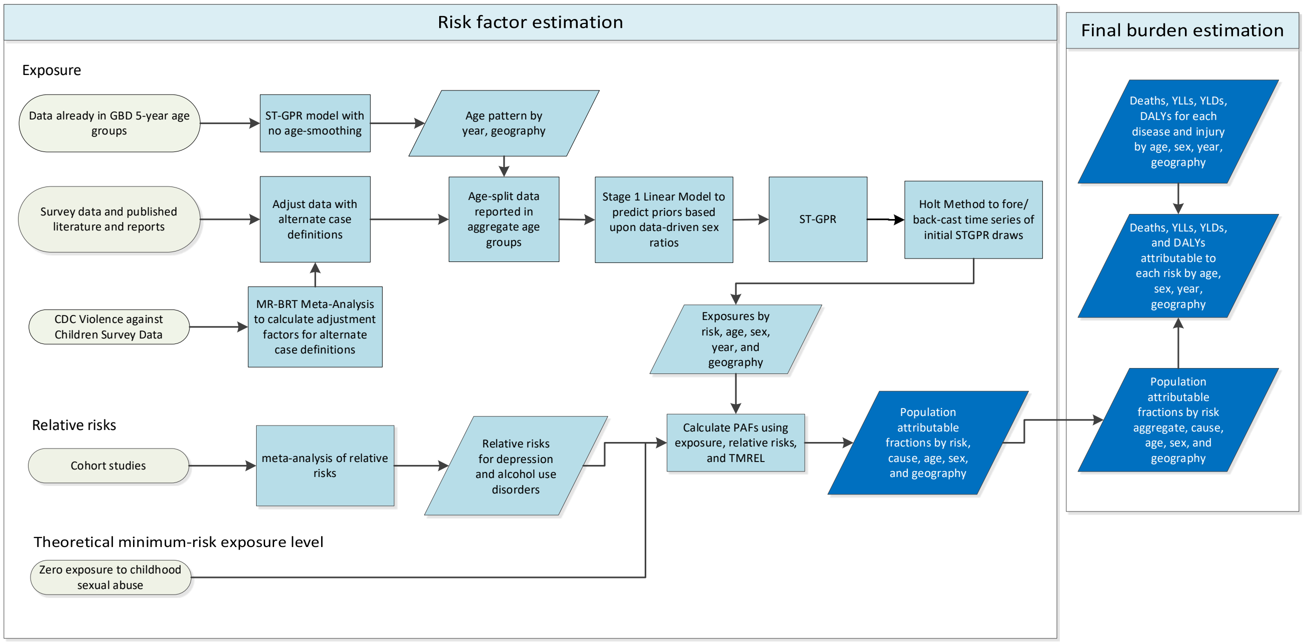

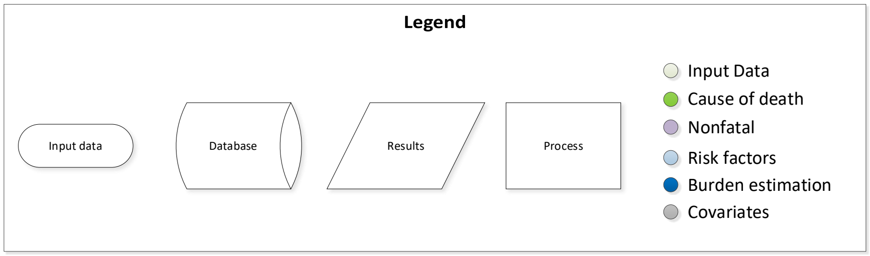

Input data and methodological summary

Definition

Exposure

The case definition for childhood sexual abuse (CSA) is ever having experienced intercourse or other contact abuse (ie, fondling and other sexual touching) when aged 15 years or younger, in which the contact was unwanted or the perpetrator was five or more years older than the victim.

Input data

Exposure

Currently, we use self-reported survey data to measure CSA prevalence, and we do not use data from Child Protection Services (CPS) or other crime data. The reliability and comprehensiveness of CPS and crime statistics vary too much geographically to warrant inclusion (typically identifying only a small proportion of cases). In addition, there is a lack of reliable survey data available for children under the age of 10, so our model is restricted to individuals 10 years and older.

For GBD 2021, we incorporated new exposure data sources identified through the GHDx and shared with us by collaborators. We included all sources that provided population-representative data on the proportion of males or females under the age of 15 who have experienced sexual abuse. In addition, we accepted sources reporting on the following non-reference cases and populations:

1. Proportion of individuals who experienced intercourse CSA
2. Proportion of individuals who experienced contact or non-contact CSA
3. Proportion of individuals who experienced CSA in which the definition of perpetrator is restricted (eg, CSA committed by a father)
4. Proportion of individuals whose first sexual debut was CSA (before age 15 and forced or with a partner 5 or more years older than the respondent)
5. Proportion of individuals who experienced sexual abuse before some age greater than 15 (such as before age 18)
6. Proportion of individuals who experienced sexual abuse before some age less than 15 (such as before age 12)
7. Proportion of individuals who experienced CSA, measured from a student population
8. Non-nationally representative populations who experienced CSA

Table 1: Data inputs for exposure for childhood sexual abuse.

	**Countries with data**	**New sources**	**Total sources**
Exposure	87	43	228

Relative risk

We did not conduct a new systematic review for CSA relative risks in GBD 2021.

Table 2: Data inputs for relative risks for childhood sexual abuse.

	**Countries with data**	**New sources**	**Total sources**
Relative risks	4	0	10

Data processing

Crosswalking

For alternate case definitions of CSA, we used gold-standard data from the CDC Violence Against Children Surveys (n=12) to run a logit-difference meta-regression with the MR-BRT tool to estimate correction factors. MR-BRT is described in detail in a separate section of this appendix. While we originally intended to fit separate models for each sex, we decided to model one set of crosswalk adjustments as we did not have rich data to inform significant differences between the sexes and observed implausible differences in adjustments made to the same source between the sexes when modelling adjustment factors separately. Our models were fit using 10% trimming and two priors, 1) contact-only (reference) definitions should be less than definitions including contact or non-contact cases, and 2) intercourse-only definitions be less than contact-only (reference) definitions.

Table 3: MR-BRT crosswalk adjustment factors for CSA exposure

Data input	Reference or alternative case definition	Gamma	Beta coefficient, logit (95% UI)*	Adjustment factor**
Contact CSA	Ref	0.0042	---	---
Contact or non-contact CSA	Alt		0.081 (-0.052, 0.213)	1.084
Intercourse-only CSA	Alt		-0.747 (-0.877, -0.616)	0.474
CSA before an age less than 15	Alt		-0.837 (-0.968, -0.707)	0.433
CSA before an age greater than 15	Alt		0.683 (0.553, 0.813)	1.98

**MR-BRT crosswalk adjustments can be interpreted as the factor the alternative case definition is adjusted by to reflect what it would have been had it been measured using the reference case definition. If the logit beta coefficient is negative, then the alternative is adjusted up to the reference. If the logit beta coefficient is positive, then the alternative is adjusted down to the reference.*

***The adjustment factor column is the exponentiated beta coefficient. For logit beta coefficients, this is the relative odds between the two case definitions.*

For studies restricting the perpetrator or reporting cases of CSA at a respondent's sexual debut, due to insufficient data comparing our reference and alternate definitions, we refrained from calculating under-informed correction factors.

Age-splitting

We split data reported in broader age groups than the GBD five-year age groups by adapting the method reported in Ng et al. to split aggregate data using a reference age pattern. We divided the data into two sets: 1) a training dataset, containing data that already fell into GBD five-year age groups, and 2) a split dataset, which contained data reported in aggregate age groups broader than GBD five-year bins. We then used spatiotemporal Gaussian process regression (ST-GPR) to estimate geography-time-specific age patterns using the training dataset. The ST-GPR model used an age-weight parameter value that minimised the effect of any age smoothing within the model. This parameter choice allowed the estimated age pattern to be driven by data rather than enforced by smoothing parameters of the model. Due to data sparsity within the training dataset, estimated geography-time age patterns were aggregated to the GBD region level. For female CSA, the age pattern from the GBD world region with the most training datapoints (high-income North America) was used to adjust all non-standard age data. Due to extreme data sparsity within the male CSA model, even the aggregated regional age-pattern for the GBD region with the most training datapoints (high-income North America) was unrealistically variable across neighbouring age groups. Therefore, countries within this region were visually examined and the most stable age pattern (Canada) was selected to adjust non-standard age data.

Modelling strategy

Exposure

We used ST-GPR to model lifetime CSA prevalence. Input data were prepared by first adjusting data with alternate case definitions and then splitting data in aggregate age groups by applying modelled reference age patterns, as described above. In previous rounds of the GBD, CSA exposure was modelled

independently for males and females because we observe little correlation between the prevalence of child abuse among females and males. However, in GBD 2021, we found that modelling male and female CSA entirely independently resulted in non-data-driven sex trends, in which priors from the male model fit in the absence of data were higher than female model fits informed by female-only data. In order to leverage data-driven sex trends, we introduced a stage one linear model that predicted trends using CSA data from both males and females. This method allowed the global ratio of CSA exposure between sexes to inform the priors of consequent ST-GPR models in the absence of (super) regional data. Full details on the ST-GPR method are reported elsewhere in the appendix. Briefly, the mean function input to GPR is a complete time series of estimates generated from the mixed effects hierarchical linear model described above plus weighted residuals smoothed across time, space, and age.

The stage one linear model formula is as follows:

$$logit(p_{g,a,t}) = \beta_0 + \beta_1 S_{A[a],g,t} + \sum_{k=2}^{20} \beta_k I_{A[a]} + \alpha_s + \alpha_r + \alpha_g + \epsilon_{g,a,t}$$

Where $S_{A[a],g,t}$ is the sex of the prevalence point by specific age group A, geography g, and time t, $I_{A[a]}$ is a dummy variable indicating specific age group A that the prevalence point $p_{g,a,t}$ captures, and α_s, α_r, and α_g are super-region, region, and geography random intercepts, respectively. Random effects were used in model fitting and prediction.

Data sparsity within the CSA models caused poor model fits over time. Thus, we introduced Holt's linear trend method (extended simple exponential smoothing) to fore- and back-cast draws from the initial ST-GPR model. Holt's linear trend method allows forecasting of data with a linear trend using a weighted average of past observations, with weights decaying exponentially as observations get older (Hyndman et al. 2018). We applied this method to location-age-specific draws from our initial ST-GPR model, with the year range of the ST-GPR draws to be used as the initial time series defined based upon location-age data availability. For location-age combinations with available data spanning more than three years, draws were bounded from the minimum year to the maximum year of location-age-specific data. Otherwise, draws were bounded from the minimum year to the maximum year of super-region-age-specific data. For male CSA, there is one super-region (north Africa and the Middle East) for which we have no data. In this case, we preserved most of the ST-GPR fit by using the time range of 1990–2019 (ie, forecasting only 2019–2022). To avoid over-forecasting for longer time periods (ie, in locations where only very old data were available), we used a damping parameter (phi=0.9) to enforce a zero-slope linear trend over time. Finally, due to our adjustment to ST-GPR draws, we needed to re-enforce consistency between subnational and national means, so we logit-raked subnational draws to fit national means for countries with subnational estimation.

Theoretical minimum-risk exposure level

The theoretical minimum-risk exposure level is zero exposure to contact childhood sexual abuse.

Relative risk

Depression

No changes were introduced to the GBD 2019 depression result. From nine studies (Brown J et al, J Am Acad Child Adolesc Psychiatry 1999, Chapman DP et al, J Affect Disord 2004, Dinwiddie S et al, Psychol Med 2000, Dube SR et al, Am J Prev Med 2005, Jaffee SR et al, Arch Gen Psychiatry 2002, Kendler KS et al, Arch Gen Psychiatry 2000, Nelson EC et al, Arch Gen Psychiatry 2002, Widom CS et al, Arch Gen Psychiatry 2007, Cheasty et all, BMJ 1998), the relative risk of depression was calculated as 1.56 (95% UI 1.30–1.86) using MR-BRT 2019. The bias covariates were selected as significant and adjusted for within the final model are listed in Table 4.

Alcohol use disorder

No changes were introduced to the GBD 2019 alcohol use disorder result. From four studies (Dinwiddie S et al, Psychol Med 2000, Fleming J et al, Addiction 1998, Kendler KS et al, Arch Gen Psychiatry 2000, & Nelson EC et al, Arch Gen Psychiatry 2002), the relative risk of alcohol use disorder was calculated as 2.21 (90% UI 1.15–4.04) using MR-BRT 2019. The bias covariates were selected as significant and adjusted for within the final model are listed in Table 4.

Table 4. MR-BRT bias covariates by risk-outcome pair

Risk-outcome pair	Selected bias covariates
CSA – Depression	cv_symptom_scale, cv_confounding_uncontrolled
CSA – Alcohol Use Disorder	cv_symptom_scale

References

1. Ng M, Freeman MK, Fleming TD, Robinson M, Dwyer-Lindgren L, Thomson B, et al. Smoking Prevalence and Cigarette Consumption in 187 Countries, 1980–2012. *JAMA*. 2014 Jan 8;311(2):183–92.
2. Hyndman, R.J., & Athanasopoulos, G. (2018) *Forecasting: principles and practice*, 2nd edition, OTexts: Melbourne, Australia. OTexts.com/fpp2.
3. Brown J, Cohen P, Johnson JG., and Smailes EM. Childhood abuse and neglect: specificity of effects on adolescent and young adult depression and suicidality. Journal of the American Academy of Child & Adolescent Psychiatry. 1999; 38(12): 1490-1496.
4. Chapman DP, Whitfield CL, Felitti VJ, Dube SR, Edwards VJ and Anda RF. Adverse childhood experiences and the risk of depressive disorders in adulthood. Journal of affective disorders. 2004; 82(2): 217-225.
5. Cheasty M, Clare AW and Collins C. Relation between sexual abuse in childhood and adult depression: case-control study. Bmj. 1998; 316(7126): 198-201.
6. Dinwiddie S, Heath AC, Dunne MP, Bucholz KK, Madden PA, Slutske WS, Bierut LJ, Statham DB, Martin NG. Early sexual abuse and lifetime psychopathology: a co-twin-control study. *Psychol Med*. 2000; 30(1): 41–52.
7. Dube SR, Anda RF, Whitfield CL, Brown DW, Felitti VJ, Dong M and Giles WH. Long-term consequences of childhood sexual abuse by gender of victim. American journal of preventive medicine. 2005; 28(5): 430-438.
8. Ernst C, Angst J, Földényi M. The Zurich Study. XVII. Sexual abuse in childhood. Frequency and relevance for adult morbidity data of a longitudinal epidemiological study. *Eur Arch Psychiatry Clin Neurosci*. 1993; 242(5): 293–300.
9. Fleming J, Mullen PE, Sibthorpe B, Attewell R and Bammer G. The relationship between childhood sexual abuse and alcohol abuse in women-a case-control study. Addiction. 1998; 93(12): 1787-1798.

10. Jaffee SR, Moffitt TE, Caspi A, Fombonne E, Poulton R, Martin J. Differences in early childhood risk factors for juvenile-onset and adult-onset depression. *Arch Gen Psychiatry*. 2002; 59(3): 215-22.
11. Kendler KS, Bulik CM, Silberg J, Hettema JM, Myers J, Prescott CA. Childhood sexual abuse and adult psychiatric and substance use disorders in women: an epidemiological and cotwin control analysis. *Arch Gen Psychiatry*. 2000; 57(10): 953–9.
12. Molnar BE, Buka SL and Kessler, RC. Child sexual abuse and subsequent psychopathology: results from the National Comorbidity Survey. American journal of public health. 2001; 91(5): 753.
13. Nelson EC, Heath AC, Madden PA, Cooper ML, Dinwiddie SH, Bucholz KK, Glowinski A, McLaughlin T, Dunne MP, Statham DJ, Martin NG. Association between self-reported sexual abuse and adverse psychosocial outcomes: results from a twin study. Arch Gen Psychiatry. 2002; 59(2): 139-45.
14. Peleikis DE, Mykletun A and Dahl AA. The relative influence of childhood sexual abuse and other family background risk factors on adult adversities in female outpatients treated for anxiety disorders and depression. Child Abuse & Neglect. 2004; 28(1): 61-76.
15. Sartor CE, Lynskey MT, Bucholz KK, McCutcheon VV, Nelson EC, Waldron M, Heath AC. Childhood sexual abuse and the course of alcohol dependence development: findings from a female twin sample. *Drug Alcohol Depend*. 2007; 89(2-3): 139–44.
16. Silverman AB, Reinherz HZ and Giaconia RM. The long-term sequelae of child and adolescent abuse: A longitudinal community study. Child abuse & neglect. 1996; 20(8): 709-723.
17. Widom CS, DuMont K and Czaja SJ. A prospective investigation of major depressive disorder and comorbidity in abused and neglected children grown up. Archives of general psychiatry. 2007; 64(1): 49-56.

Dietary risks

List of abbreviations used in this section

DR	Dietary recall surveys
FAO	Food and Agriculture Organization of the United Nations
FBS	FAO Food Balance Sheets
FFQ	Food frequency questionnaires
FPG	Fasting plasma glucose
GHDx	IHME Global Health Data Exchange
HBS	Household budget surveys
IHD	Ischaemic heart disease
IHME	Institute for Health Metrics and Evaluation
MR-BRT	Meta Regression; Bayesian; Regularized; Trimmed
PRISMA	Preferred Reporting Items for Systematic Reviews and Meta-Analyses
PUFA	Polyunsaturated fatty acids
Sales	Euromonitor Fresh and Packaged Food Sales data
SBP	Systolic blood pressure
SSBs	Sugar-sweetened beverages
ST-GPR	Spatiotemporal gaussian process regression
SUA	FAO Supply Utilization Accounts
TMREL	Theoretical minimum-risk exposure level
USDA	United States Department of Agriculture

Flowchart

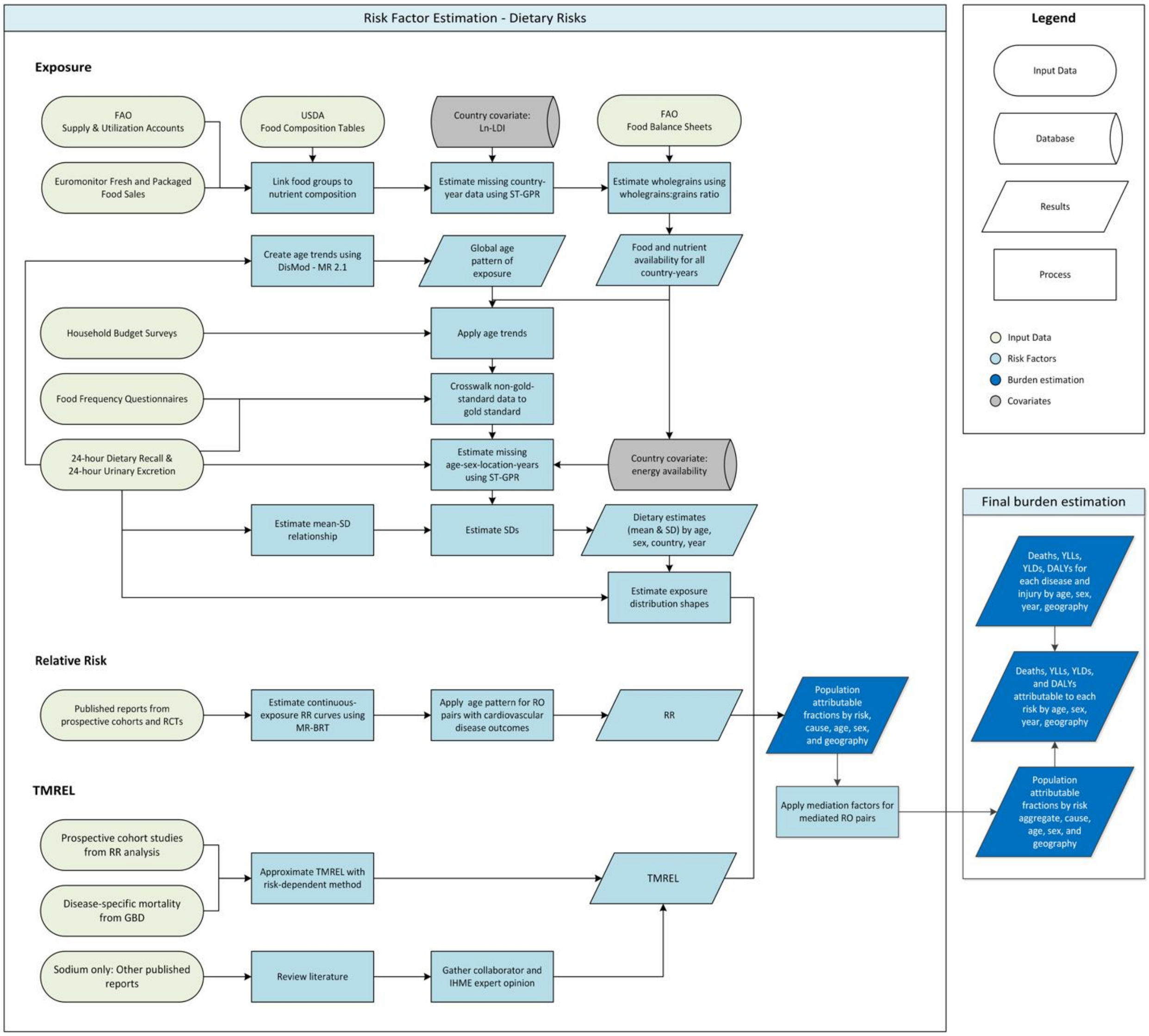

“Ln-LDI" stands for log lag-distributed income per capita; “RO pair” stands for risk-outcome pair; "SD” stands for standard deviation; “RR” stands for relative risk. Other abbreviations defined in list of abbreviations for this section.

Input data and methodological summary

Note that GBD dietary risk factor modelling is for adult populations only (ages 25+).

Definition

Exposure

Table 1: Dietary risk factor exposure definitions and optimal level of exposure as defined by GBD 2021

Dietary Risk Factor	Definition of exposure	Optimal level or range of intake*
**Diet low in fruit**	Average daily consumption (in grams per day) of fruit including fresh, frozen, cooked, canned, or dried fruit, excluding fruit juices and salted or pickled fruits	340–350 g/day

**Diet low in vegetables**	Average daily consumption (in grams per day) of vegetables, including fresh, frozen, cooked, canned, or dried vegetables and excluding legumes and salted or pickled vegetables, juices, nuts and seeds, and starchy vegetables such as potatoes or corn	306–372 g/day
**Diet low in whole grains**	Average daily consumption (in grams per day) of whole grains (bran, germ, and endosperm in their natural proportion) from breakfast cereals, bread, rice, pasta, biscuits, muffins, tortillas, pancakes, and other sources	160–210 g/day
**Diet low in nuts and seeds**	Average daily consumption (in grams per day) of nuts and seeds, including tree nuts and seeds and peanuts	19–24 g/day
**Diet low in fibre**	Average daily consumption (in grams per day) of fibre from all sources including fruits, vegetables, grains, legumes, and pulses	22–25 g/day
**Diet low in seafood omega-3 fatty acids**	Average daily consumption (in milligrams per day) of eicosapentaenoic acid (EPA) and docosahexaenoic acid (DHA)	470–660 mg/day
**Diet low in omega-6 polyunsaturated fatty acids**	Average daily consumption (in % daily energy) from omega-6 polyunsaturated fatty acids (PUFA) (specifically linoleic acid, γ-linolenic acid, eicosadienoic acid, dihomo-γ-linolenic acid, arachidonic acid)	9–10% of total daily energy
**Diet low in calcium**	Average daily consumption (in grams per day) of calcium from all sources, including milk, yoghurt, and cheese	0.72–0.86 g/day (males)<br>1.1–1.2 g/day (females)
**Diet low in milk**	Average daily consumption (in grams per day) of dairy milk including non-fat, low-fat, and full-fat milk, but excluding plant-based milks, fermented milk products such as buttermilk, and other dairy products such as cheese	280–340 g/day (males)<br>500–610 g/day (females)
**Diet low in legumes**	Average daily consumption (in grams per day) of legumes and pulses, including fresh, frozen, cooked, canned, or dried legumes	100–110 g/day
**Diet high in red meat**	Average daily consumption (in grams per day) of unprocessed red meat including pork and bovine meats such as beef, pork, lamb, and goat, but excluding all processed meats, poultry, fish, and eggs	0–200 g/day
**Diet high in processed meat**	Average daily consumption (in grams per day) of meat preserved by smoking, curing, salting, or addition of chemical preservatives	0 g/day

**Diet high in sugar-sweetened beverages (SSBs)**	Average daily consumption (in grams per day) of beverages with ≥50 kcal per 226.8 gram serving, including carbonated beverages, sodas, energy drinks, and fruit drinks, but excluding 100% fruit and vegetable juices	0 g/day
**Diet high in trans fatty acids**	Average daily consumption (in percent daily energy) of trans fat from all sources, mainly partially hydrogenated vegetable oils and ruminant products	0–1.1% of total daily energy
**Diet high in sodium**	Average 24-hour urinary sodium excretion (in grams per day)	1–5 g/day

** "Optimal level" is equivalent to "TMREL" throughout this document.*

Input data

Exposure

The dietary data that we used in the exposure models come from multiple sources, including nationally and subnationally representative nutrition surveys using 24-hour dietary recall methodology (DR), food frequency questionnaires (FFQ), household budget surveys (HBS), accounts of national sales from the Euromonitor ("sales"), and food availability data from the Food and Agriculture Organization of the United Nations (FAO). Table 2 provides a summary of data inputs used for each dietary risk factor modelled in GBD 2021. For sodium, data from the 24-hour urinary sodium excretion and 24-hour dietary recall surveys was used. In GBD 2021, we did not make any updates to the exposure data sources used in the dietary risk factor models. In GBD 2019, we had included new DR sources from a literature search of PubMed and updates to yearly known survey series from the Institute for Health Metrics and Evaluation Global Health Data Exchange (IHME GHDx) in our models; we had also conducted a new systematic review for sodium.

Table 2: Data inputs for exposure for dietary risk factors

Dietary risk factor	Countries with exposure data	Total exposure sources
All dietary risks	194	1515
Calcium	178	160
Fibre	180	155
Fruit	180	869
Legumes	169	683
Milk	177	1148
Nuts and seeds	158	100
Omega-3	178	20
Processed meat	66	737
Omega-6	180	70
Red meat	178	760
Sodium	52	146

SSBs	66	720
Trans fat	72	924
Vegetables	180	871
Whole grains	188	52

Through GBD 2017, the availability data for food groups in GBD was based on the FAO Food Balance Sheets (FBS), which provide tabulated and processed data of national food supply. In GBD 2019, to characterise the national availability of various food groups more accurately, we used more disaggregated data on food commodities that were included in FAO Supply Utilization Accounts (SUA) and recreated the national availability of each food group based on the GBD definition of the food group. In some cases where it serves as an appropriate supplement to FAO SUA data, we continued to use FAO FBS data. We used this GBD 2019 approach in GBD 2021.

To estimate nutrient availability, we used USDA Food Composition Tables and constructed a global nutrient database, as indicated in Schmidhuber et al., 2018.[i, ii]

Our primary, gold-standard data sources are 24-hour dietary recall surveys where food and nutrient intake are reported or convertible to grams/milligrams per person per day for all dietary risk factors; for sodium, the gold standard is 24-hour urinary sodium excretion. For other data types, not every type was used for every dietary risk factor. Table 3 summarizes which sources were used in modelling each dietary risk factor.

Table 3. Types of data sources (other than 24-hour dietary) and covariates used in modelling of each dietary risk factor

**Dietary risk factor**	**Data sources**				**Country-level covariate**
	**Sales**	**FFQ[1]**	**HBS[2]**	**FAO [3]**	
Diet low in fruits	•	•	•	•	Lag-distributed income
Diet low in vegetables	•	•	•	•	Energy availability (kcal)
Diet low in whole grains	-	•	-	•	Energy availability (kcal)
Diet low in nuts and seeds	-	-	•	•	Energy availability (kcal)
Diet low in milk	•	•	•	•	Energy availability (kcal)
Diet high in red meat	•	•	•	•	Energy availability (kcal)
Diet high in processed meat	•	•	•	-	Energy availability (kcal), pigs per capita
Diet low in legumes	•	•	-	•	Energy availability (kcal)
Diet high in sugar-sweetened beverages	•	•	•	-	Energy availability (kcal), availability of sugar
Diet low in fibre	-	•	-	•	Energy availability (kcal)
Diet low in calcium	-	•	-	•	Energy availability (kcal)
Diet low in seafood omega-3 fatty acids	-	-	-	•	Lag-distributed income, proportion landlocked area
Diet low in omega-6 polyunsaturated fatty acids	-	•	-	•	Lag-distributed income
Diet high in trans fatty acids	•	•	-	-	
Diet high in sodium[4]	-	-	-	-	

[1] *Food Frequency Questionnaire*
[2] *Household Budget Survey*
[3] *Food and Agriculture Organization*
[4] *For sodium, we used data from the 24-hour urinary sodium excretion and 24-hour dietary recall.*

Relative Risk

Table 5: Data inputs used to estimate relative risk for dietary risk factors

Dietary risk factor	Countries with relative risk data	Total relative risk sources
Calcium	9	29
Fibre	13	51
Fruit	14	81
Legumes	4	9
Milk	8	24
Nuts and seeds	8	21
Omega-3	16	48
Processed meat	10	34
Omega-6	7	16
Red meat	14	55
Sodium	2	4
SSBs	5	12
Trans fat	4	9
Vegetables	11	50
Whole grains	9	36

In GBD 2021, we conducted two additional systematic reviews that examine the health effects of vegetable and red meat consumption.

The red meat systematic review evaluated the association between unprocessed red meat consumption and six health outcomes based on 37 prospective cohort studies and one nested case-control study. The health outcomes included in the systematic review were haemorrhagic stroke, type 2 diabetes, colorectal cancer, ischaemic heart disease (IHD), breast cancer, and ischaemic stroke. The study identification process is described in the PRISMA flow diagram (Figure 1). The detailed methods and results of the study were published elsewhere.[iii]

The vegetable systematic review examined the health effects of vegetable consumption and five health outcomes: IHD, ischaemic stroke, haemorrhagic stroke, type 2 diabetes, and oesophageal cancer. A total of 49 prospective cohort studies and one nested case-control study, with a total of 4,609,296 participants, were included in the analysis. Most of the studies used food frequency questionnaires to assess vegetable consumption. The study identification process is depicted in the PRISMA diagram (Figure 2-5). The detailed methods and full findings of the systematic review were published elsewhere.[iv]

Figure 1: PRISMA flow diagram of unprocessed red meat data-seeking approach

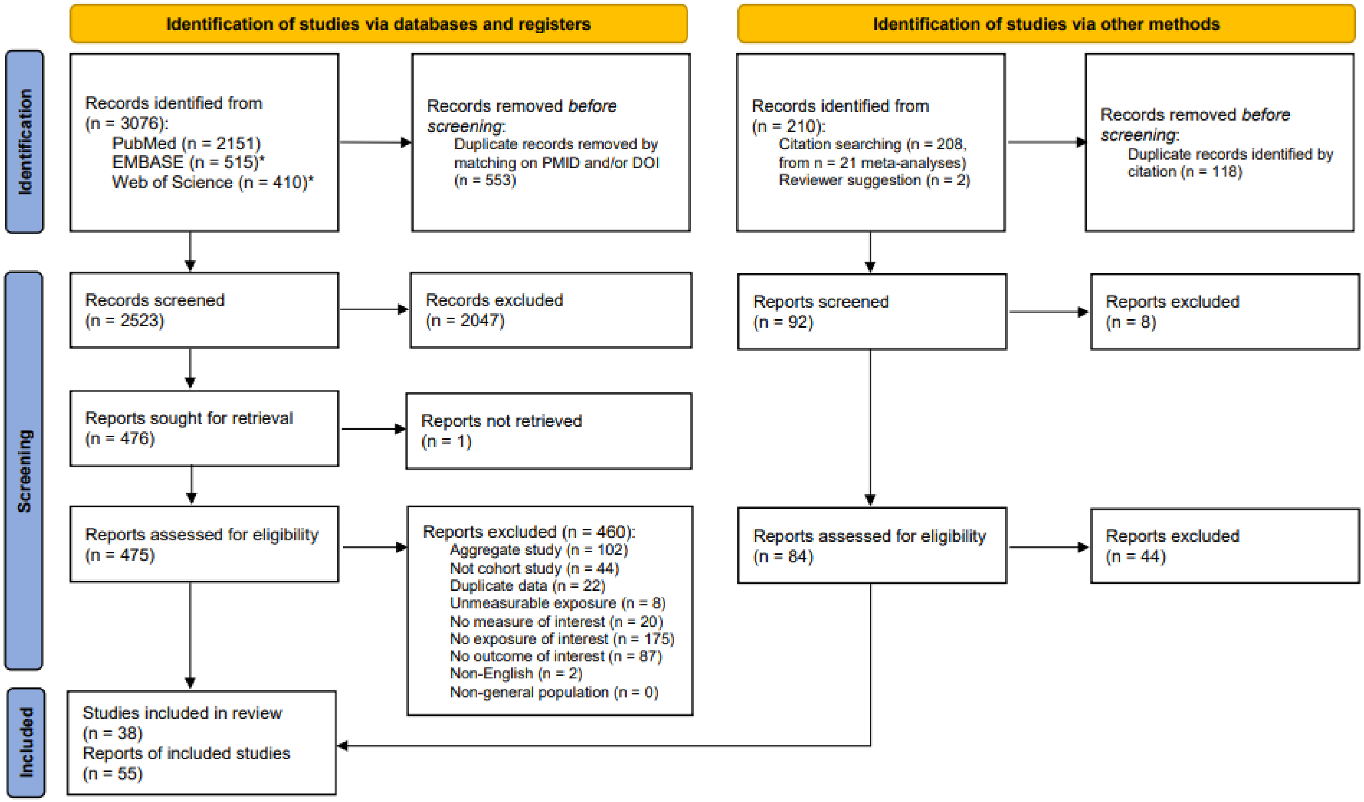

Figure 2: PRISMA flow diagram of vegetable consumption and stroke subtypes (ischaemic stroke and haemorrhagic stroke) data-seeking approach

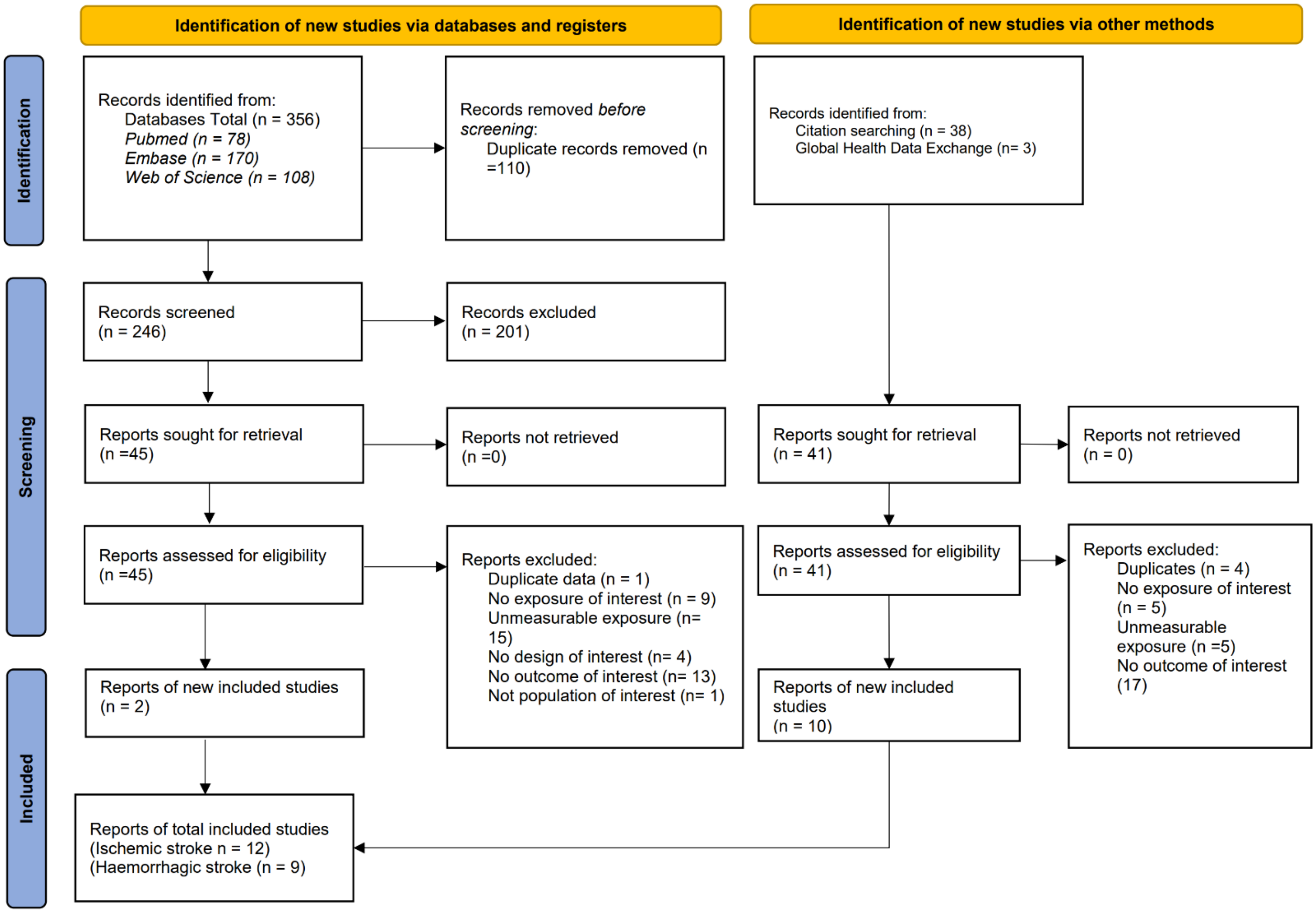

Figure 3: PRISMA flow diagram of vegetable consumption and IHD data-seeking approach

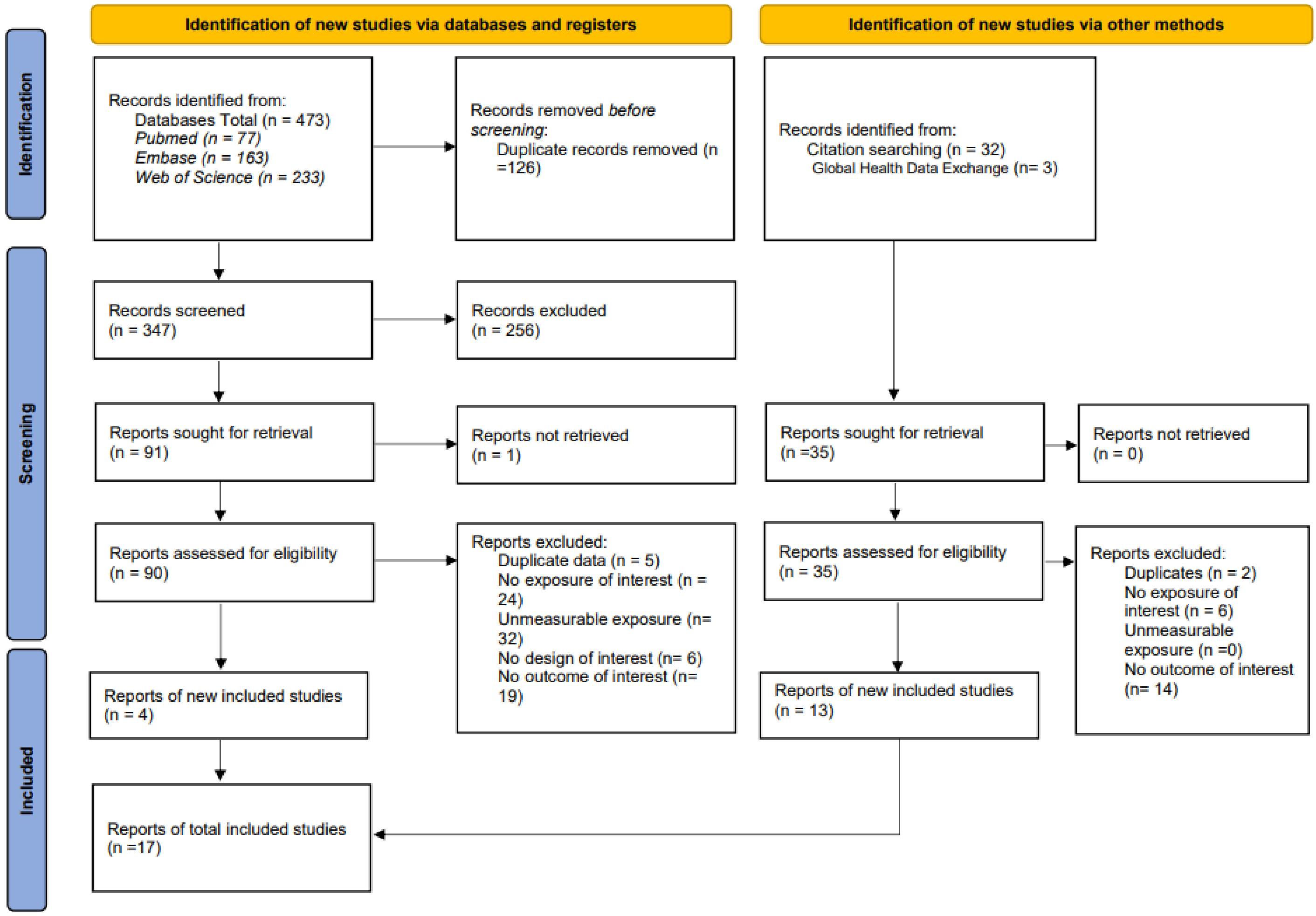

Figure 4: PRISMA flow diagram of vegetable consumption and oesophageal cancer data-seeking approach

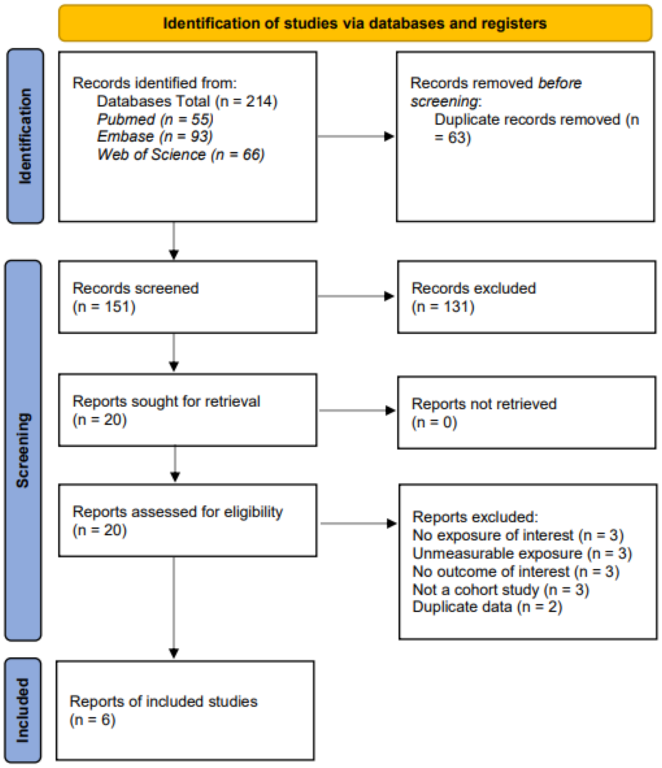

Figure 5: PRISMA flow diagram of vegetable consumption and type 2 diabetes data-seeking approach

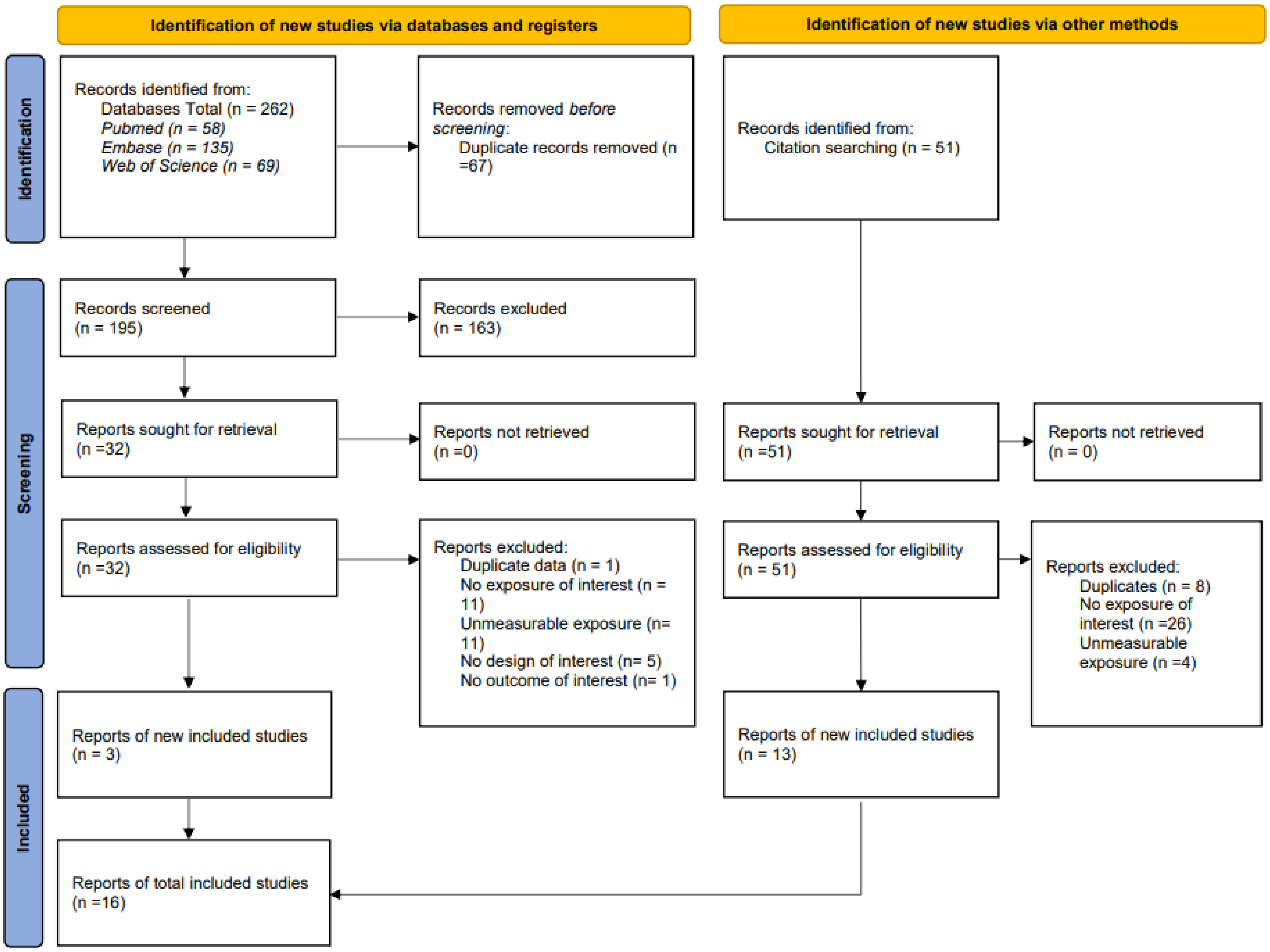

Data processing

Exposure

Data processing for exposure data largely followed the methodology of previous GBD rounds, apart from a change for trans fat and a correction to omega-3 fatty acids. For trans fat, we implemented an adjustment for locations that have implemented gold-standard trans-fat policy bans.[v] Given the relatively poor data coverage of trans fatty acids intake in recent years, in the absence of any policy assumptions, the temporal trend does not decrease in locations that have banned trans fat. To account for this, we assume that trans fat intake becomes zero percent of energy per day in the year following the introduction of a gold-standard policy ban. A gold-standard ban is defined as a mandatory national limit of 2 grams of industrially produced trans fat per 100 grams of total fat in all foods or mandatory national ban on the production or use of partially hydrogenated oils as an ingredient in all foods. For omega-3 fatty acids, we implemented a correction that had the overall effect of increasing estimates of omega-3 fatty acids exposure and decreasing burden associated with low omega-3 fatty acids intake in some locations. Standard deviations for omega-3 exposure also increased overall. The general data processing process for all dietary risk factors is described in more detail in the following paragraphs.

First, we prepared the FAO data. We mapped specific food items in the FAO data to our risk factors (e.g., a type of vegetable is included in the risk factor category "vegetables"). For omega-3, omega-6, calcium, and fibre, availability was estimated by mapping the FAO food item to a corresponding entry in the USDA Food and Nutrient Database for Dietary Studies. Missing country-year data for each risk was estimated by using a spatiotemporal Gaussian process regression with log lag-distributed income per capita as the covariate. This process also yielded our energy availability covariate for a later stage of diet exposure modelling (see Table 3).

Second, we applied information about consumption by age group to data sources that are not age specific. For each dietary risk factor, we estimated the global age pattern of consumption based on 24-hour dietary recall surveys and applied that age pattern to the all-age data (FAO, sales, and HBS).

Third, we used gold-standard data to adjust non-gold-standard data for bias. Our gold-standard data source for all dietary risks (except sodium) is 24-hour dietary recall surveys where food and nutrient intake are reported or convertible to grams per person per day; the gold-standard data source for sodium is 24-hour urinary sodium excretion. The other data sources we used—household budget surveys, food frequency questionnaires, sales, and availability—were treated as alternate definitions for dietary intake and crosswalked to the gold-standard definition. For non-sex-specific sources (FAO, sales, and HBS), we applied the sex split by using sex-specific gold-standard intake data. In GBD 2016 and GBD 2017, we determined the bias adjustment factors from a mixed effects linear regression. In GBD 2019 and GBD 2021, we used MR-BRT (a tool developed by researchers at IHME, standing for Meta Regression; Bayesian; Regularized; Trimmed) to determine the adjustment factors for non-gold-standard datapoints. In cases where we had more than one data input type serving as an alternative definition, we used a network meta-regression implemented with MR-BRT. Coefficients for these models can be found in Table 4.

Table 4: MR-BRT crosswalk adjustment factors for dietary risk factor exposure

Dietary risk	Sex	Data input	Reference or alternative case definition	Gamma	Beta coefficient, log (95% UI)*	Adjustment factor**
Calcium	---	DR	Reference	0.24	---	---
Calcium	Female	FAO	Alternative		0.04 (-0.45 to 0.50)	0.96 (0.60–1.57)
Calcium	Female	FFQ	Alternative		-0.04 (-0.53 to 0.43)	1.04 (0.65–1.70)
Calcium	Male	FAO	Alternative		0.17 (-0.32 to 0.63)	0.84 (0.53–1.38)
Calcium	Male	FFQ	Alternative		0.09 (-0.40 to 0.55)	0.91 (0.58–1.49)
Fibre	---	DR	Reference	0.33	---	---
Fibre	Female	FAO	Alternative		0.56 (-0.07 to 1.17)	0.57 (0.31–1.08)
Fibre	Female	FFQ	Alternative		0.27 (-0.37 to 0.88)	0.76 (0.42–1.45)
Fibre	Male	FAO	Alternative		0.55 (-0.08 to 1.17)	0.57 (0.31–1.08)
Fibre	Male	FFQ	Alternative		0.26 (-0.38 to 0.88)	0.77 (0.42–1.46)
Fruit	---	DR	Reference	0.76	---	---
Fruit	Female	FAO	Alternative		0.36 (-1.16 to 1.83)	0.70 (0.16–3.20)
Fruit	Female	Sales	Alternative		0.73 (-0.80 to 2.19)	0.48 (0.11–2.22)
Fruit	Female	FFQ	Alternative		-0.15 (-1.68 to 1.32)	1.17 (0.27–5.35)
Fruit	Female	HBS	Alternative		0.23 (-1.30 to 1.71)	0.79 (0.18–3.65)
Fruit	Male	FAO	Alternative		0.32 (-1.20 to 1.79)	0.73 (0.17–3.33)
Fruit	Male	Sales	Alternative		0.69 (-0.84 to 2.16)	0.50 (0.12–2.31)
Fruit	Male	FFQ	Alternative		-0.19 (-1.72 to 1.28)	1.21 (0.28–5.56)
Fruit	Male	HBS	Alternative		0.19 (-1.34 to 1.66)	0.83 (0.19–3.80)
Legumes	---	DR	Reference	0.74	---	---
Legumes	Female	FAO	Alternative		-0.08 (-1.49 to 1.39)	1.08 (0.25–4.45)
Legumes	Female	Sales	Alternative		-0.90 (-2.31 to 0.56)	2.47 (0.57–10.04)
Legumes	Female	FFQ	Alternative		-0.53 (-1.94 to 0.95)	1.70 (0.39–6.95)
Legumes	Male	FAO	Alternative		0.06 (-1.35 to 1.53)	0.94 (0.22–3.85)
Legumes	Male	Sales	Alternative		-0.76 (-2.16 to 0.70)	2.14 (0.50–8.68)
Legumes	Male	FFQ	Alternative		-0.39 (-1.79 to 1.09)	1.47 (0.34–6.01)
Milk	---	DR	Reference	1.06	---	---
Milk	Female	FAO	Alternative		0.27 (-1.81 to 2.57)	0.76 (0.08–6.09)
Milk	Female	Sales	Alternative		0.01 (-2.06 to 2.31)	0.99 (0.10–7.86)
Milk	Female	FFQ	Alternative		0.46 (-1.74 to 2.78)	0.63 (0.06–5.69)
Milk	Female	HBS	Alternative		-0.61 (-2.69 to 1.69)	1.84 (0.19–14.71)
Milk	Male	FAO	Alternative		0.28 (-1.79 to 2.58)	0.75 (0.08–6.00)
Milk	Male	Sales	Alternative		0.03 (-2.05 to 2.33)	0.97 (0.10–7.74)
Milk	Male	FFQ	Alternative		0.48 (-1.72 to 2.80)	0.62 (0.06–5.60)
Milk	Male	HBS	Alternative		-0.59 (-2.68 to 1.70)	1.81 (0.18–14.52)
Nuts & seeds	---	DR	Reference	1.58	---	---
Nuts & seeds	Female	FAO	Alternative		0.49 (-2.79 to 3.63)	0.62 (0.03–16.25)
Nuts & seeds	Female	FFQ	Alternative		-0.34 (-3.70 to 2.76)	1.41 (0.06–40.51)

Dietary risk	Sex	Data input	Reference or alternative case definition	Gamma	Beta coefficient, log (95% UI)*	Adjustment factor**
Nuts & seeds	Female	HBS	Alternative		-0.72 (-3.99 to 2.42)	2.06 (0.09–53.95)
Nuts & seeds	Male	FAO	Alternative		0.60 (-2.68 to 3.73)	0.55 (0.02–14.63)
Nuts & seeds	Male	FFQ	Alternative		-0.23 (-3.58 to 2.87)	1.26 (0.06–35.94)
Nuts & seeds	Male	HBS	Alternative		-0.62 (-3.88 to 2.54)	1.85 (0.08–48.56)
Omega-3	---	DR	Reference	0.12	---	---
Omega-3	Female	FAO	Alternative		-0.26 (-1.92 to 1.46)	1.29 (0.23–6.82)
Omega-3	Male	FAO	Alternative		-0.57 (-2.24 to 1.15)	1.76 (0.32–9.39)
Proc. meat	---	DR	Reference	1.21	---	---
Proc. meat	Female	Sales	Alternative		0.79 (-1.68 to 3.14)	0.46 (0.04–5.35)
Proc. meat	Female	FFQ	Alternative		-0.30 (-2.98 to 2.25)	1.35 (0.11–19.65)
Proc. meat	Female	HBS	Alternative		-0.46 (-2.92 to 1.89)	1.59 (0.15–18.53)
Proc. meat	Male	Sales	Alternative		0.95 (-1.51 to 3.30)	0.39 (0.04–4.55)
Proc. meat	Male	FFQ	Alternative		-0.13 (-2.82 to 2.42)	1.14 (0.09–16.78)
Proc. meat	Male	HBS	Alternative		-0.30 (-2.76 to 2.06)	1.35 (0.13–15.74)
Omega-6	---	DR	Reference	0.14	---	---
Omega-6	Female	FAO	Alternative		-0.14 (-0.43 to 0.14)	1.15 (0.87–1.54)
Omega-6	Female	FFQ	Alternative		1.05 (0.68 to 1.43)	0.35 (0.24–0.51)
Omega-6	Male	FAO	Alternative		-0.18 (-0.48 to 0.10)	1.20 (0.91–1.62)
Omega-6	Male	FFQ	Alternative		1.00 (0.63–1.38)	0.37 (0.25–0.53)
Red meat	---	DR	Reference	0.83	---	---
Red meat	Female	FAO	Alternative		0.89 (-0.80 to 2.54)	0.41 (0.08–2.23)
Red meat	Female	Sales	Alternative		1.09 (-0.61 to 2.74)	0.34 (0.06–1.84)
Red meat	Female	FFQ	Alternative		-0.34 (-2.22 to 1.60)	1.40 (0.20–9.21)
Red meat	Female	HBS	Alternative		0.45 (-1.24 to 2.10)	0.64 (0.12–3.45)
Red meat	Male	FAO	Alternative		0.89 (-0.80 to 2.54)	0.41 (0.08–2.23)
Red meat	Male	Sales	Alternative		1.09 (-0.61 to 2.74)	0.34 (0.06–1.84)
Red meat	Male	FFQ	Alternative		-0.34 (-2.22 to 1.60)	1.40 (0.20–9.22)
Red meat	Male	HBS	Alternative		0.45 (-1.24 to 2.10)	0.64 (0.12–3.46)
Sodium	---	Urinary sodium excreti on	Reference	0.39	---	---
Sodium	Female	DR	Alternative		-0.02 (-0.97 to 0.85)	1.02 (0.43–2.63)
Sodium	Female	FFQ	Alternative		0.47 (-0.37 to 1.29)	0.63 (0.27–1.44)
Sodium	Male	DR	Alternative		-0.06 (-0.97 to 0.80)	1.06 (0.45–2.65)
Sodium	Male	FFQ	Alternative		0.43 (-0.40 to 1.26)	0.65 (0.28–1.49)
SSBs	---	DR	Reference	0.61	---	---
SSBs	Female	Sales	Alternative		0.15 (-0.99 to 1.43)	0.86 (0.24–2.71)
SSBs	Female	FFQ	Alternative		-0.01 (-1.20 to 1.32)	1.01 (0.27–3.31)

Dietary risk	Sex	Data input	Reference or alternative case definition	Gamma	Beta coefficient, log (95% UI)*	Adjustment factor**
SSBs	Female	HBS	Alternative		-0.59 (-1.74 to 0.68)	1.80 (0.50–5.69)
SSBs	Male	Sales	Alternative		0.35 (-0.79 to 1.63)	0.70 (0.20–2.21)
SSBs	Male	FFQ	Alternative		0.19 (-0.99 to 1.53)	0.83 (0.22–2.70)
SSBs	Male	HBS	Alternative		-0.39 (-1.53 to 0.89)	1.48 (0.41–4.62)
Trans fat	---	DR	Reference	0.22	---	---
Trans fat	Male	Sales	Alternative		-0.23 (-1.27 to 0.94)	1.25 (0.39–3.54)
Trans fat	Female	Sales	Alternative		-0.23 (-1.27 to 0.94)	1.25 (0.39–3.54)
Trans fat	Male	FFQ	Alternative		0.59 (-2.72 to 4.23)	0.56 (0.01–15.18)
Trans fat	Female	FFQ	Alternative		0.86 (-2.63 to 4.90)	0.42 (0.01–13.87)
Vegetables	---	DR	Reference	0.64	---	---
Vegetables	Female	FAO	Alternative		0.12 (-1.18 to 1.33)	0.89 (0.26–3.24)
Vegetables	Female	Sales	Alternative		0.62 (-0.68 to 1.83)	0.54 (0.16–1.97)
Vegetables	Female	FFQ	Alternative		-0.05 (-1.34 to 1.16)	1.05 (0.31–3.83)
Vegetables	Female	HBS	Alternative		0.10 (-1.20 to 1.31)	0.91 (0.27–3.32)
Vegetables	Male	FAO	Alternative		0.16 (-1.13 to 1.37)	0.85 (0.25–3.10)
Vegetables	Male	Sales	Alternative		0.66 (-0.63 to 1.87)	0.52 (0.15–1.89)
Vegetables	Male	FFQ	Alternative		-0.01 (-1.30 to 1.20)	1.01 (0.30–3.67)
Vegetables	Male	HBS	Alternative		0.14 (-1.16 to 1.35)	0.87 (0.26–3.17)
Whole grains	---	DR	Reference	0.69	---	---
Whole grains	Female	FAO	Alternative		1.94 (0.60–3.37)	0.14 (0.03–0.55)
Whole grains	Female	FFQ	Alternative		-0.35 (-2.07 to 1.37)	1.42 (0.25–7.89)
Whole grains	Male	FAO	Alternative		2.09 (0.75–3.52)	0.12 (0.03–0.47)
Whole grains	Male	FFQ	Alternative		-0.20 (-1.91 to 1.52)	1.22 (0.22–6.76)

** MR-BRT crosswalk adjustments can be interpreted as the factor the alternative case definition is adjusted by to reflect what it would have been had it been measured using the reference case definition. If the log/logit beta coefficient is negative, then the alternative is adjusted up to the reference. If the log/logit beta coefficient is positive, then the alternative is adjusted down to the reference.*

*** The adjustment factor column is the exponentiated beta coefficient. For log beta coefficients, this is the relative rate between the two case definitions. For logit beta coefficients, this is the relative odds between the two case definitions.*

Modelling strategy

Exposure

We made no changes to the exposure modelling strategy in GBD 2021. We used a spatiotemporal Gaussian process regression (ST-GPR) framework to estimate the mean intake of each dietary risk factor by age, sex, location, and year. The covariates used in the model can be seen in Table 3.

We determined the standard deviation of each population's consumption through a linear regression that captured the relationship between the standard deviation and mean intake. We estimated this model using a subset of our gold-standard data (i.e., nationally representative surveys capturing 24-hour urinary sodium excretion for the sodium risk factor and 24-hour dietary recall data for other dietary risk factors):

$$ln\ (Standard\ deviation) = \beta_0 + \beta_1 \times ln\ (Mean_i)$$

Then we applied the coefficients of this regression to the outputs of our ST-GPR model to calculate the standard deviation of intake by age, sex, year, and location. The standard deviations were adjusted for within-person variation. Previous adjustment factors were updated for GBD 2021 by this method.

We characterised the shape of the distribution of exposure for each dietary risk factor with an approach that uses an ensemble of distribution types. First, we selected microdata suitable for estimating the shape of the distribution of dietary risk factor exposure within a population. The selected microdata we used to create the fit include a subset of 24-hour urinary sodium excretion for sodium and a subset of 24-hour dietary recall data for other dietary risk factors. Second, we separately fit 12 types of distributions (including, for example, normal, log normal, gamma, etc.) to this microdata. Then, the respective goodness of fit of each distribution type was assessed to determine the weight for each distribution type. Finally, we created the global weighted ensemble distribution shape, which is applied during PAF calculation.

Theoretical minimum-risk exposure level

We used several different approaches to set the dietary theoretical minimum-risk exposure levels (TMRELs).

Sodium: For sodium, we use a TMREL range of 1-5 grams per day of urinary sodium excretion, based on a literature review, collaborator discussion, and a final decision taken by the GBD Scientific Council. This TMREL has remained the same since GBD 2013.[vi]

Harmful risks: For strictly harmful dietary risks, the TMREL was set to zero, following the same approach as GBD 2019.

Protective risks: For strictly protective dietary risks except vegetables, we used data from the cohort studies used in the relative risk analysis to create a TMREL. The aim of the approach was to select an exposure level that reflects high real-world consumption and for which we have solid evidence about the associated risks. First, for each risk-outcome pair, we calculated the 85th percentile of each the lower bound and the midpoints of all alternative (non-lowest) exposure ranges reported by the underlying

study. Second, for each the lower bound and the midpoint statistic, we took a weighted average across outcomes, weighting by the global number of deaths due to each outcome as reported by GBD 2019. Finally, we constructed a uniform distribution between these two values to serve as the TMREL. For vegetables, a similar but slightly different approach was followed, as described in Stanaway et al., 2022.[4] This calculation method differs only slightly from GBD 2019.

Mixed protective and harmful risks: For risks that have both protective and harmful associations with outcomes (calcium and milk), the approach was similar to protective risk TMREL calculation. However, in the first step, the 15th percentile of the midpoints of the reference exposure ranges (i.e., the lowest exposure range reported by the study) and the 15th percentile of the upper bound of the reference exposure ranges were used for harmful risk-outcome pairs. In the second step, sex-specific death weights were used. The final step of constructing the uniform distribution remained the same. In GBD 2019, calcium and milk were both protective risks, so the TMREL calculation followed the protective risk methodology. Furthermore, these TMRELs were not sex specific in GBD 2019. In GBD 2021, new, harmful associations with prostate cancer were added for both calcium and milk, so these risks became "mixed" and the TMREL became sex-specific due to the sex-specificity of prostate cancer.

J-shaped risks: For J-shaped risks, we used an approach that minimizes the mean relative risk curve.

- Red meat: First, we estimated 1,000 draws of the relative risk curve for each risk-outcome pair. Second, relative risk draws from each risk-outcome pair were weighted by global number of deaths from the outcome, as estimated by GBD 2019, and combined, resulting in 1,000 draws of an across-outcome, death-weighted relative risk curve for red meat. Finally, each draw of the death-weighted relative risk curve was minimized, yielding 1,000 draws of the TMREL. The non-normal distribution can be seen in Figure 6.
- Trans fat: The trans-fat TMREL is a set of 1,000 draws, calculated as the minimum of each of 1,000 draws of an intermediate estimation of the relative risk curve for the single trans fat risk-outcome pair. (The final model was not used, since the nature of modelling a j-shaped risk implies that the minimum of each draw in the final model will necessarily occur at one of the knots, i.e. exposure values, that was estimated in intermediate modelling then passed as a parameter to create the final model. In this specific case, this would have resulted in all 1,000 draws occurring at just one of two exposure levels).

Because the distribution of the TMREL is not normal for the j-shaped risks, 1,000 draws of the TMREL were used in downstream calculations. The 95% uncertainty interval of these TMRELs are reported in Table 8. In GBD 2019, all dietary risk factors were modelled with a monotonic constraint, so red meat and trans fat both followed the harmful risk of TMREL calculation in that round.

An overarching limitation in the calculation of dietary TMRELs was that only direct risk-outcome relationships are considered in calculation; mediation is not accounted for. Furthermore, while GBD accounts for key risk-outcome relationships, risk-outcome relationships that were not accounted for in GBD were not accounted for in TMREL calculation.

Table 8. Theoretical minimum risk exposure level for dietary risk factors, GBD 2019 and GBD 2021

Risk type	Dietary risk factor	GBD 2019	GBD 2021
Protective	**Fruits**	310–340 g/day	340–350 g/day

	**Vegetables**	280–320 g/day	306–372 g/day
	**Whole grains**	140–160 g/day	160–210 g/day
	**Nuts and seeds**	10–19 g/day	19–24 g/day
	**Dietary fibre**	21–22 g/day	22–25 g/day
	**Legumes**	90–100 g/day	100–110 g/day
	**Omega-6 polyunsaturated fatty acids**	7–9% of total daily energy	9–10% of total daily energy
	**Seafood omega-3 fatty acids**	430–470 mg/day	470–660 mg/day
Harmful	**Processed meats**	0 g/day	0 g/day
	**Dietary sodium**	1–5 g/day	1–5 g/day
	**Sugar-sweetened beverages**	0 g/day	0 g/day
J-shaped	**Red meats**	0 g/day	0–200 g/day
	**Trans fatty acids**	0% of total daily energy	0–1.1% of total daily energy
Mixed protective and harmful	**Dietary calcium**	1.06–1.1 g/day	0.72–0.86 g/day (males)<br>1.1–1.2 g/day (females)
	**Milk**	360–500 g/day	280–340 g/day (males)<br>500–610 g/day (females)

Figure 6. The TMREL draws for red meat, as based on the cause-weighted mortality curve. Note that about 50 draws of the relative risk curve are monotonically decreasing; therefore, relative risk is minimised at the maximum exposure value (in this case, 200 g/day).

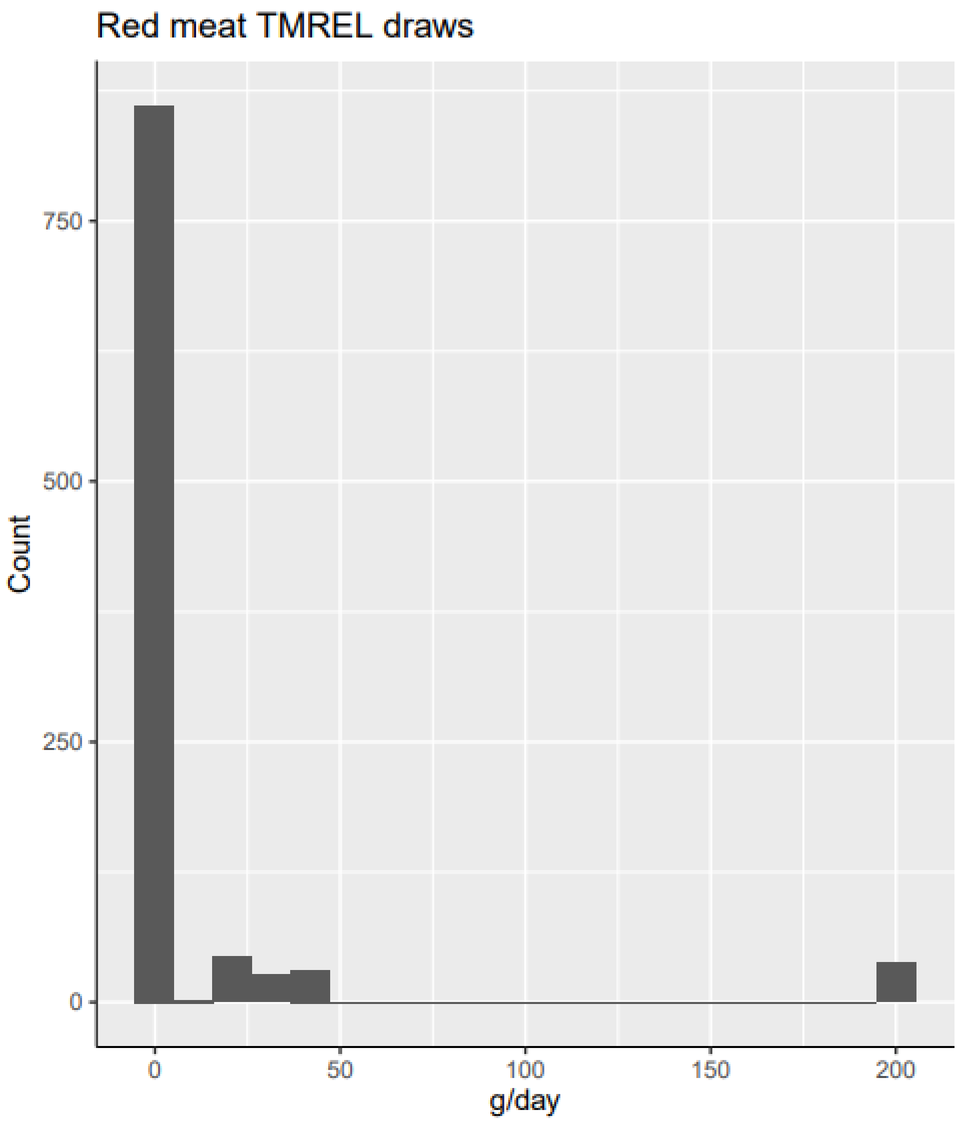

Relative risk

In GBD 2019, dietary risk factors were modelled using MR-BRT for the first time, as part of piloting the new MR-BRT tool for continuous relative risk curves. For GBD 2021, we updated the relative risk curves using an updated version of MR-BRT and new model specifications. To determine the shape of the risk curve for each risk-outcome pair, we first modelled it without any constraints. If a risk curve was generally increasing or decreasing across the full exposure domain, it was considered a monotonic (i.e., strictly protective or strictly harmful) risk. Otherwise, if the risk curve had j-shape behaviour (i.e., it decreases before increasing, or the inverse, or does not demonstrate a change in relative risk until some

threshold), it was considered a j-shaped risk. Then, each risk-outcome pair was modelled according to the priors and constraints outlined in Table 6.

For each risk-outcome pair meta-regression, we considered study-level covariates that could potentially bias the study's reported effect size estimates. These study-level covariates included indication of whether the study had a follow-up period >10 months, whether the study used a wider definition of the exposure or outcome, whether the study was a randomised controlled trial or a cohort study, whether the effect sizes were relative risks or odds ratios, whether the effect sizes were for incidence or mortality, whether the study measured intake with a single or repeat measurements, whether the study determined outcomes based on administrative records or self-reports, and the level of adjustment for relevant confounders like age, sex, smoking, education, and income. Additionally for our vegetable risk-outcome pairs, we considered whether the studies adjusted for energy intake as a covariate.[4] We adjusted for these covariates in our meta-regression if they significantly biased our estimated relative risk function.

We implemented the Fisher scoring correction to the heterogeneity parameter, which corrects for data-sparse situations. In such cases, the between-study heterogeneity parameter estimate may be 0, simply from lack of data. The Fisher scoring correction uses a quantile of gamma, which is sensitive to the number of studies, study design, and reported uncertainty.

We have also added methodology that can detect and flag publication bias. The approach was based on the classic Egger's regression strategy, which was applied to the residuals in our model. In the current implementation, we do not correct for publication bias but flag the risk-outcome pairs where the risk for publication bias is significant.

In the tables below, we list each dietary risk-outcome pair used in GBD 2021 along with several of the key modelling parameters and results. The formulation for MR-BRT is described in detail in the general methods of the GBD appendix.

Table 6: MR-BRT model specifications

Shape of risk	Spline degree, # interior knots	Priors & constraints
Protective	Quadratic, 2 I knots	Monotonic decreasing, right linear tail, Gaussian max derivative prior on the right tail (0, 0.001)
Harmful	Quadratic, 2 I knots	Monotonic increasing, right linear tail, Gaussian max derivative prior on the right tail (0, 0.001)
J-shaped	Quadratic, 3 I knots	No monotonicity constraint, right and left linear tails, Gaussian max derivative prior on the right and left tail (0, 0.001)

Table 7: Risk-outcome pair model specifications and results

Risk-outcome	Shape of risk	Selected covariates	Mean gamma solution	Publication bias result
Calcium & colorectal cancer	Protective	--	0	No publication bias

Risk-outcome	Shape of risk	Selected covariates	Mean gamma solution	Publication bias result
Calcium & prostate cancer	Harmful	--	0	No publication bias
Fibre & colorectal cancer	Protective	--	0.012	No publication bias
Fibre & type 2 diabetes	Protective	--	0	Publication bias observed
Fibre & haemorrhagic stroke	Protective	--	0	No publication bias
Fibre & IHD	Protective	--	0	No publication bias
Fibre & ischaemic stroke	Protective	Total stroke	0	No publication bias
Fruit & type 2 diabetes	Protective	--	0	No publication bias
Fruit & haemorrhagic stroke	Protective	Total stroke	0.079	Publication bias observed
Fruit & IHD	Protective	--	0.006	No publication bias
Fruit & ischaemic stroke	Protective	Total stroke	0	Publication bias observed
Fruit & lung cancer	Protective	--	0	No publication bias
Legumes & IHD	Protective	--	0.250	No publication bias
Milk & colorectal cancer	Protective	--	0	No publication bias
Milk & prostate cancer	Harmful	--	0.434	No publication bias
Nuts and seeds & IHD	Protective	--	0.018	No publication bias
Omega-3 & IHD	Protective	Study type	0.028	No publication bias
Processed meat & colorectal cancer	Harmful	--	0.061	No publication bias
Processed meat & type 2 diabetes	Harmful	Incidence	0.028	Publication bias observed
Processed meat & IHD	Harmful	--	0	No publication bias
Omega-6 & IHD	Protective	Study type	0.809	No publication bias
Red meat & breast cancer	Harmful		0	No publication bias
Red meat & colorectal cancer	Harmful		0.099	No publication bias
Red meat & type 2 diabetes	J-shaped	--	0.096	No publication bias
Red meat & haemorrhagic stroke	Protective	--	0.930	No publication bias
Red meat & IHD	J-shaped	--	0	No publication bias
Red meat & ischaemic stroke	J-shaped		0.016	Publication bias observed
Sodium & stomach cancer	Harmful	--	0.006	No publication bias
SSB & type 2 diabetes	Harmful	--	0	No publication bias
SSB & IHD	Harmful	--	0	No publication bias
Trans fatty acids & IHD	J-shaped	--	0	No publication bias

Risk-outcome	Shape of risk	Selected covariates	Mean gamma solution	Publication bias result
Veg & type 2 diabetes	Protective	Outcome assessment, confounder adjustment, exposure measurement, length of follow-up	0.236	No publication bias
Veg & haemorrhagic stroke	Protective		0	No publication bias
Veg & IHD	Protective	Confounder measurement, incidence	0.020	Publication bias observed
Veg & ischaemic stroke	Protective		0	No publication bias
Veg & oesophageal cancer	Protective	--	0.006	Publication bias observed
Whole grain & colorectal cancer	Protective	--	0	No publication bias
Whole grain & type 2 diabetes	Protective	Follow-up period, Odds ratio	0	No publication bias
Whole grain & IHD	Protective	--	0	No publication bias
Whole grain & ischaemic stroke	Protective	Total stroke	0.211	No publication bias

After evaluating all available evidence, we found sufficient evidence on the casual relationship for three new risk-outcome pairs and insufficient evidence for one old risk-outcome pair. Based on these results, we updated the risk-outcome pairs used the GBD dietary risk factor analysis with the removals and additions listed below. Please note that this list only includes changes due to direct modelling; any additions or removals due solely to changes in mediation are not included here:

Removed:

    Diet low in nuts/seeds and type 2 diabetes
    Diet low in fruit and oesophageal cancer

Note that diet low in fruit and oesophageal cancer was reported as removed in GBD 2019 documentation. However, it was accidentally included in calculation and results reporting. We removed it for GBD 2021.

Added:

    Diet low in vegetables and type 2 diabetes
    Diet low in milk and prostate cancer
    Diet low in calcium and prostate cancer

Note that, while we retain the risk factor names of "diet *low* in milk" and "diet *low* in calcium", each of these has a harmful association with prostate cancer.

There is a well-documented attenuation of the risk for cardiovascular disease due to metabolic risks factors throughout one's life.[vii] To incorporate this age trend in the relative risks, we first identified the median age-at-event across all cohorts and considered that as the reference age group. We then assigned our risk curves to this reference age group. Then, we derived attenuation factors by taking the ratio of excess risk between each age group and the reference. Finally, we applied 1000 draws of the age-specific attenuation factors to 1000 draws of the reference age group's risk curve to determine age-specific risk curves that propagated the uncertainty of both the risk function and age pattern. The three cardiovascular disease outcomes for dietary risks are haemorrhagic stroke (including intracerebral haemorrhage and subarachnoid haemorrhage), ischaemic stroke, and IHD; the effects of dietary risks on them are mediated through high systolic blood pressure and cholesterol (not included for haemorrhagic stroke). There was no evidence found for an age pattern in fasting plasma glucose, and since the effect of diet is estimated independently of body-mass index in the GBD study, body-mass index was not included as a mediator in the relative risk age trend analysis.

Risk-outcome pairs modelled through mediation

This dietary-risk-specific modelling section has dealt almost exclusively with dietary risks associated *directly* with outcomes in the GBD 2021 framework. Dietary risks were also associated with outcomes *indirectly* through mediation.

Below is a list of the dietary risk-outcome pairs modelled through mediation in GBD 2021, where dietary risk factors (first column) affect mediators (middle column), which in turn affect outcomes (last column). Mediators are themselves risk factors. The last column contains the value of the mediation factor. See the section of the GBD 2021 appendix on mediation for more details about mediation modelling.

Table 8. Mediation factor matrix – Dietary risk factors

For IHD, stroke, and diabetes we pooled all available cohorts and estimated relative risks with and without adjustment across all combinations of metabolic risk factors. We then computed the excess attenuated risk for each mediation-risk-cause set.

Risk Factor	Mediator	Cause	Mediation Factor
Diet low in fruits	High fasting plasma glucose	Ischemic stroke	0.05 (0.04 to 0.06)
Diet low in fruits	High fasting plasma glucose	Lower extremity peripheral arterial disease	1 (1 to 1)
Diet low in fruits	High fasting plasma glucose	Diabetes mellitus type 2	1 (1 to 1)
Diet low in fruits	High fasting plasma glucose	Drug-susceptible tuberculosis	1 (1 to 1)
Diet low in fruits	High fasting plasma glucose	Multidrug-resistant tuberculosis without extensive drug resistance	1 (1 to 1)
Diet low in fruits	High fasting plasma glucose	Extensively drug-resistant tuberculosis	1 (1 to 1)
Diet low in fruits	High fasting plasma glucose	Chronic kidney disease	0.15 (0.02 to 0.31)
Diet low in fruits	High LDL cholesterol	Ischemic heart disease	0.07 (0.05 to 0.08)
Diet low in fruits	High LDL cholesterol	Ischemic stroke	0.05 (0.04 to 0.06)
Diet low in fruits	High systolic blood pressure	Ischemic heart disease	0.07 (0.05 to 0.08)

Diet low in fruits	High systolic blood pressure	Ischemic stroke	0.05 (0.04 to 0.06)
Diet low in fruits	High systolic blood pressure	Intracerebral hemorrhage	0.02 (0.02 to 0.03)
Diet low in fruits	High systolic blood pressure	Subarachnoid hemorrhage	0.02 (0.02 to 0.03)
Diet low in fruits	High systolic blood pressure	Hypertensive heart disease	1 (1 to 1)
Diet low in fruits	High systolic blood pressure	Aortic aneurysm	1 (1 to 1)
Diet low in fruits	High systolic blood pressure	Chronic kidney disease	0.85 (0.69 to 0.98)
Diet low in vegetables	High fasting plasma glucose	Ischemic heart disease	0.06 (0.01 to 0.20)
Diet low in vegetables	High fasting plasma glucose	Ischemic stroke	0.08 (0.04 to 0.16)
Diet low in vegetables	High fasting plasma glucose	Intracerebral hemorrhage	0.08 (0.04 to 0.16)
Diet low in vegetables	High fasting plasma glucose	Lower extremity peripheral arterial disease	1 (1 to 1)
Diet low in vegetables	High fasting plasma glucose	Diabetes mellitus type 2	1 (1 to 1)
Diet low in vegetables	High fasting plasma glucose	Drug-susceptible tuberculosis	1 (1 to 1)
Diet low in vegetables	High fasting plasma glucose	Multidrug-resistant tuberculosis without extensive drug resistance	1 (1 to 1)
Diet low in vegetables	High fasting plasma glucose	Extensively drug-resistant tuberculosis	1 (1 to 1)
Diet low in vegetables	High fasting plasma glucose	Chronic kidney disease	0.06 (-0.01 to 0.14)
Diet low in vegetables	High LDL cholesterol	Ischemic heart disease	0.04 (0.03 to 0.05)
Diet low in vegetables	High LDL cholesterol	Ischemic stroke	0.09 (0.04 to 0.16)
Diet low in vegetables	High systolic blood pressure	Ischemic heart disease	0.04 (0.03 to 0.05)
Diet low in vegetables	High systolic blood pressure	Ischemic stroke	0.03 (0.02 to 0.04)
Diet low in vegetables	High systolic blood pressure	Intracerebral hemorrhage	0.04 (0.02 to 0.05)
Diet low in vegetables	High systolic blood pressure	Subarachnoid hemorrhage	0.04 (0.02 to 0.05)
Diet low in vegetables	High systolic blood pressure	Hypertensive heart disease	1 (1 to 1)
Diet low in vegetables	High systolic blood pressure	Aortic aneurysm	1 (1 to 1)
Diet low in vegetables	High systolic blood pressure	Chronic kidney disease	0.95 (0.86 to 1.01)
Diet low in whole grains	High fasting plasma glucose	Lower extremity peripheral arterial disease	1 (1 to 1)
Diet low in whole grains	High fasting plasma glucose	Diabetes mellitus type 2	1 (1 to 1)
Diet low in whole grains	High fasting plasma glucose	Drug-susceptible tuberculosis	1 (1 to 1)
Diet low in whole grains	High fasting plasma glucose	Multidrug-resistant tuberculosis without extensive drug resistance	1 (1 to 1)
Diet low in whole grains	High fasting plasma glucose	Extensively drug-resistant tuberculosis	1 (1 to 1)

Diet low in whole grains	High fasting plasma glucose	Chronic kidney disease	1<br>(1 to 1)
Diet low in whole grains	High LDL cholesterol	Ischemic heart disease	0.39<br>(0.17 to 0.54)
Diet low in whole grains	High LDL cholesterol	Ischemic stroke	0.17<br>(0.05 to 0.37)
Diet low in nuts and seeds	High LDL cholesterol	Ischemic heart disease	0.20<br>(0.01 to 0.76)
Diet low in milk	Diet low in calcium	Colon and rectum cancer	1<br>(1 to 1)
Diet high in red meat	High fasting plasma glucose	Lower extremity peripheral arterial disease	1<br>(1 to 1)
Diet high in red meat	High fasting plasma glucose	Diabetes mellitus type 2	1<br>(1 to 1)
Diet high in red meat	High fasting plasma glucose	Drug-susceptible tuberculosis	1<br>(1 to 1)
Diet high in red meat	High fasting plasma glucose	Multidrug-resistant tuberculosis without extensive drug resistance	1<br>(1 to 1)
Diet high in red meat	High fasting plasma glucose	Extensively drug-resistant tuberculosis	1<br>(1 to 1)
Diet high in red meat	High fasting plasma glucose	Chronic kidney disease	1<br>(1 to 1)
Diet high in processed meat	High fasting plasma glucose	Ischemic heart disease	0.01<br>(0.01 to 0.02)
Diet high in processed meat	High fasting plasma glucose	Ischemic stroke	1<br>(1 to 1)
Diet high in processed meat	High fasting plasma glucose	Lower extremity peripheral arterial disease	1<br>(1 to 1)
Diet high in processed meat	High fasting plasma glucose	Diabetes mellitus type 2	1<br>(1 to 1)
Diet high in processed meat	High fasting plasma glucose	Drug-susceptible tuberculosis	1<br>(1 to 1)
Diet high in processed meat	High fasting plasma glucose	Multidrug-resistant tuberculosis without extensive drug resistance	1<br>(1 to 1)
Diet high in processed meat	High fasting plasma glucose	Extensively drug-resistant tuberculosis	1<br>(1 to 1)
Diet high in processed meat	High fasting plasma glucose	Chronic kidney disease	1<br>(1 to 1)
Diet high in sugar-sweetened beverages	High fasting plasma glucose	Ischemic stroke	1<br>(1 to 1)
Diet high in sugar-sweetened beverages	High fasting plasma glucose	Lower extremity peripheral arterial disease	1<br>(1 to 1)
Diet high in sugar-sweetened beverages	High fasting plasma glucose	Diabetes mellitus type 2	1<br>(1 to 1)
Diet high in sugar-sweetened beverages	High fasting plasma glucose	Drug-susceptible tuberculosis	1<br>(1 to 1)
Diet high in sugar-sweetened beverages	High fasting plasma glucose	Multidrug-resistant tuberculosis without extensive drug resistance	1<br>(1 to 1)
Diet high in sugar-sweetened beverages	High fasting plasma glucose	Extensively drug-resistant tuberculosis	1<br>(1 to 1)
Diet high in sugar-sweetened beverages	High fasting plasma glucose	Chronic kidney disease	1<br>(1 to 1)
Diet low in polyunsaturated fatty acids	High LDL cholesterol	Ischemic heart disease	0.00<br>(-0.03 to 0.03)
Diet low in polyunsaturated fatty acids	High LDL cholesterol	Ischemic stroke	1<br>(1 to 1)
Diet high in trans fatty acids	High LDL cholesterol	Ischemic heart disease	0.15<br>(0.02 to 0.24)

Diet high in sodium	High systolic blood pressure	Ischemic heart disease	1<br>(1 to 1)
Diet high in sodium	High systolic blood pressure	Ischemic stroke	1<br>(1 to 1)
Diet high in sodium	High systolic blood pressure	Intracerebral hemorrhage	1<br>(1 to 1)
Diet high in sodium	High systolic blood pressure	Subarachnoid hemorrhage	1<br>(1 to 1)
Diet high in sodium	High systolic blood pressure	Hypertensive heart disease	1<br>(1 to 1)
Diet high in sodium	High systolic blood pressure	Atrial fibrillation and flutter	1<br>(1 to 1)
Diet high in sodium	High systolic blood pressure	Aortic aneurysm	1<br>(1 to 1)
Diet high in sodium	High systolic blood pressure	Lower extremity peripheral arterial disease	1<br>(1 to 1)
Diet high in sodium	High systolic blood pressure	Chronic kidney disease	1<br>(1 to 1)

Citations

1. United States Department of Agriculture, Agricultural Research Service, Nutrient Data Laboratory. USDA National Nutrient Database for Standard Reference, Release 28 (Slightly revised). Version Current: May 2016. Accessed from http://www.ars.usda.gov/ba/bhnrc/ndl.
2. Schmidhuber J, Sur P, Fay K, et al. The Global Nutrient Database: availability of macronutrients and micronutrients in 195 countries from 1980 to 2013. Lancet Planet Health. 2018;2:e353-368. doi: https://doi.org/10.1016/S2542-5196(18)30170-0.
3. Lescinsky H, Afshin A, Ashbaugh C, et al. Health effects associated with consumption of unprocessed red meat: a Burden of Proof study. Nat Med. 2022;28:2075-2082. doi: https://doi.org/10.1038/s41591-022-01968-z.
4. Stanaway JD, Afshin A, Ashbaugh C, et al. Health effects associated with vegetable consumption: a Burden of Proof study. Nat Med. 2022;28:2066-2074. doi: https://doi.org/10.1038/s41591-022-01970-5.
5. World Health Organization. TFA Country Score Card. c2012. Available at: https://extranet.who.int/nutrition/gina/en/scorecard/TFA.

6. GBD 2013 Risk Factors Collaborators. Global, regional, and national comparative risk assessment of 79 behavioural, environmental and occupational, and metabolic risks or clusters of risks in 188 countries, 1990-2013: a systematic analysis for the Global Burden of Disease Study 2013. Lancet. 2015 Dec 5;386(10010):2287-323. doi: 10.1016/S0140-6736(15)00128-2. Epub 2015 Sep 11. PMID: 26364544; PMCID: PMC4685753.
7. Singh GM, Danaei G, Farzadfar F, et al. The Age-Specific Quantitative Effects of Metabolic Risk Factors on Cardiovascular Diseases and Diabetes: A Pooled Analysis. PLOS ONE. 2013;8:e65174.

Drug use

Flowchart

Drug Use

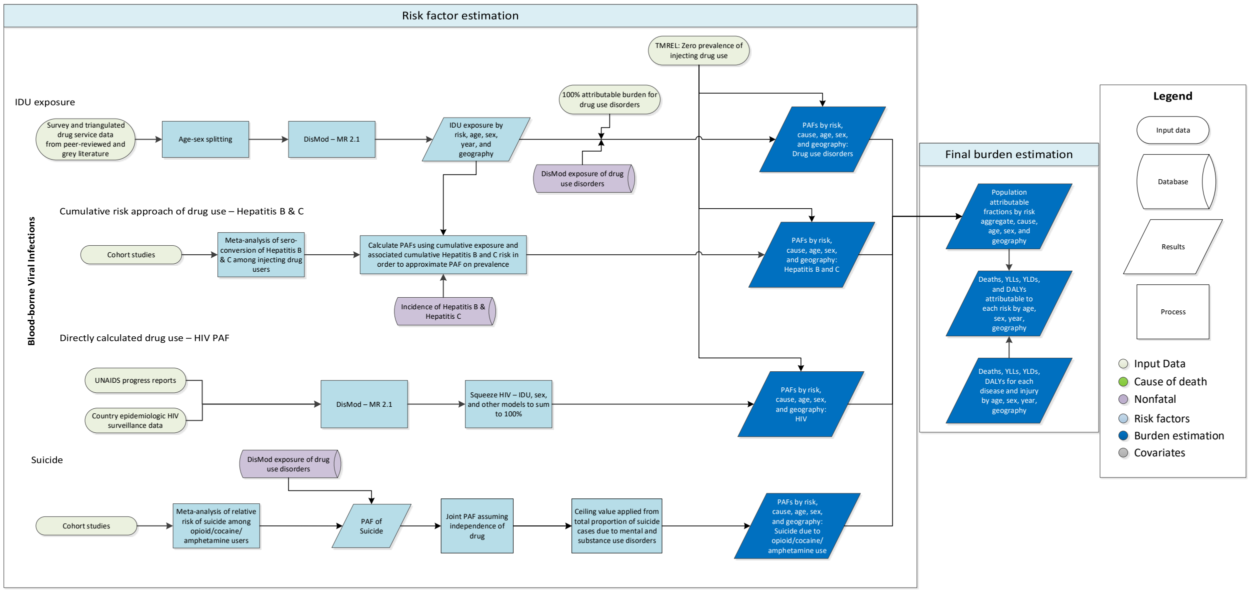

Input data and methodological summary

Exposure definition

The drug use risk factor includes four dimensions of exposure. First, we include 100% attribution of drug use disorder estimates. Second, estimates of prevalence of opioid, amphetamine, and cocaine use disorder are used as exposures for risk of suicide. These drug use disorders are defined based on DSM or ICD diagnostic criteria. Third, instead of starting with an exposure model to estimate the proportion of HIV cases due to injection drug use (IDU), we model the PAF[1] (population attributable fractions, described in appendix) directly, alongside proportion of HIV cases due to sexual transmission and other routes of transmission, which mainly includes blood transfusions. Finally, prevalence of IDU is used to model risk of Hepatitis B and C viruses (HBV and HCV, respectively). Injecting drug users are at high risk of bloodborne infections due to the use of shared needles and injection equipment. Injecting drug use is defined as current IDU among individuals aged 15–64. The theoretical minimum risk exposure level[i] (TMREL, described in appendix) for drug use is defined as zero exposure to drug use.

[1] Murray CJL, Aravkin AY, Zheng P, *et al*. Global burden of 87 risk factors in 204 countries and territories, 1990–2019: a systematic analysis for the Global Burden of Disease Study 2019. *The Lancet* 2020; **396**: 1223–49. doi: https://doi.org/10.1016/S0140-6736(20)30752-2

Input data

To estimate the burden of HIV cases attributable to IDU, we extracted data on the proportion of notified HIV cases by transmission route – sexual intercourse, injecting drug use, and other – from a number of agencies that conduct surveillance of HIV across the globe.[1-8]

The prevalence of current injecting drug use was estimated using data from a multistage process of systematic review. It involved multiple stages of peer and expert review, including by the Reference Group to the UN on HIV and injecting drug use,[9] with searches of the peer-reviewed literature in addition to an extensive review of online grey literature databases in the drug and alcohol and HIV fields.

In order to generate a pooled incidence rate/absolute relative risk for viral hepatitis among people who inject drugs, we conducted a meta-analysis of longitudinal epidemiological studies that reported a hepatitis B or hepatitis C incidence rate among persons who inject drugs.[10-25] We calculated confidence intervals for the incidence rate (where no confidence interval was reported) from a Poisson distribution around the number of cases.

We excluded studies that focused on non-representative subgroups, such as recent injectors or adolescents, because hepatitis incidence is far higher in those groups than for all people who inject drugs (eg, Larney and colleagues[26]). We did not vary incidence among active injectors according to the availability of bloodborne virus-prevention strategies (eg, needle and syringe programs (NSPs), opioid substitution therapy) because too few studies have examined different levels of incidence according to variable coverage, and we were not able to estimate coverage by country over time. In any case, in most countries, effective coverage of virus-prevention strategies remains low among people who inject drugs.[27]

Inputs to the model also include estimates of the incidence of hepatitis B and hepatitis C, coming from estimation of non-fatal health outcomes in GBD. Full details on the inputs and modelling process to produce these estimates are available in the disease-specific appendices in the GBD 2021 diseases and injuries manuscript.

Table 1: Input data summary

Input data	Countries with data	New sources	Total sources
Exposure	64	15	144
Relative risk	15	4	45

Modelling strategy

Burden of HIV attributable to injecting drug use

We estimated the proportion of HIV cases attributable to three transmission categories (sex, IDU, and

other) for all country-time periods using DisMod-MR 2.1[2] (Disease Model – Bayesian Meta-regression, described in appendix 1, section 4.5). In previous GBD rounds, data for estimating the proportion of HIV cases attributable to IDU were age-split using the age pattern of the IDU exposure model and sex-split in DisMod. In GBD 2019, these data were age- and sex-split using the estimated IDU exposure age-sex pattern, resulting in increases in the proportion of HIV due to IDU among men and decreases among women. We scaled the proportions from each of the three transmission models (sex, IDU, and other) to ensure that they fit the total HIV transmission envelope by country, year, age, and sex. Scaled estimates are used as direct PAFs, meaning that the proportion coming from the model is the proportion of HIV deaths or DALYs attributable to IDU.

Burden of hepatitis B and hepatitis C attributable to injecting drug use

To estimate the relative contribution of IDU to hepatitis B and C disease burden at the country, regional, and global level, we used a cohort method. We recalibrated individuals according to history of injecting drug use and their accumulated risk of incident hepatitis B and C due to IDU. We made use of data on prevalence of current injecting drug use, pooled in DisMod-MR 2.1; a meta-analysis of incidence rates of hepatitis B and hepatitis C among people who inject drugs; and estimates of population-level incidence of hepatitis B and C between 1990 and 2019. We used back-extrapolations to estimate incidence before 1990. These steps are detailed below.

To estimate the lifetime risk of being infected with hepatitis B or C, we undertook a cohort analysis for each country, year, age, and sex category and estimated the probability of an individual having been infected in each preceding year. One of the main inputs to this cohort method was the probability of having injected drugs in a specific age cohort in a given calendar year. For example, for a cohort of 40-year-olds in 2015, the relevant probability in 2005 is the estimated prevalence of injecting drug use among 30-year-olds.

DisMod-MR 2.1 was used to estimate the prevalence of injecting drug use with year as a covariate to estimate the trends over time. DisMod makes an average estimate of the change in drug use over the time period 1990–2019, and we took draws from a normal distribution of the coefficient to project IDU prevalence backward in time to 1960 from baseline level in 1990 (assuming there was little injecting drug use before the 1960s). In GBD 2019, prevalence of IDU was estimated as a single parameter prevalence model in DisMod, as opposed to a full compartmental model, because factoring in cause-specific mortality resulted in underestimating prevalence in certain locations, particularly in the North Africa and Middle East and South Asia super-regions. In future rounds, we plan to test bias covariates for potential use in these models.

Theoretical minimum risk exposure level

The TMREL is defined as zero exposure to drug use.

[2] Vos T, Lim SS, Abbafati C, et al. Global burden of 369 diseases and injuries in 204 countries and territories, 1990–2019: a systematic analysis for the Global Burden of Disease Study 2019. The Lancet 2020; 396: 1204–22. doi: https://doi.org/10.1016/S0140-6736(20)30925-9

Relative risk

Table 2: Relative risk input data summary

Input data	Relative risk
Source count (total)	42
Number of countries with data	14

We used a pooled absolute risk of hepatitis C and hepatitis B among those who have ever used injecting drugs. Input data for this pooled absolute risk are described above, and there were no methodological or data changes to this parameter in GBD 2021.

Figure 1: Forest plot of absolute risk of HBV incidence among cohorts of people who inject drugs

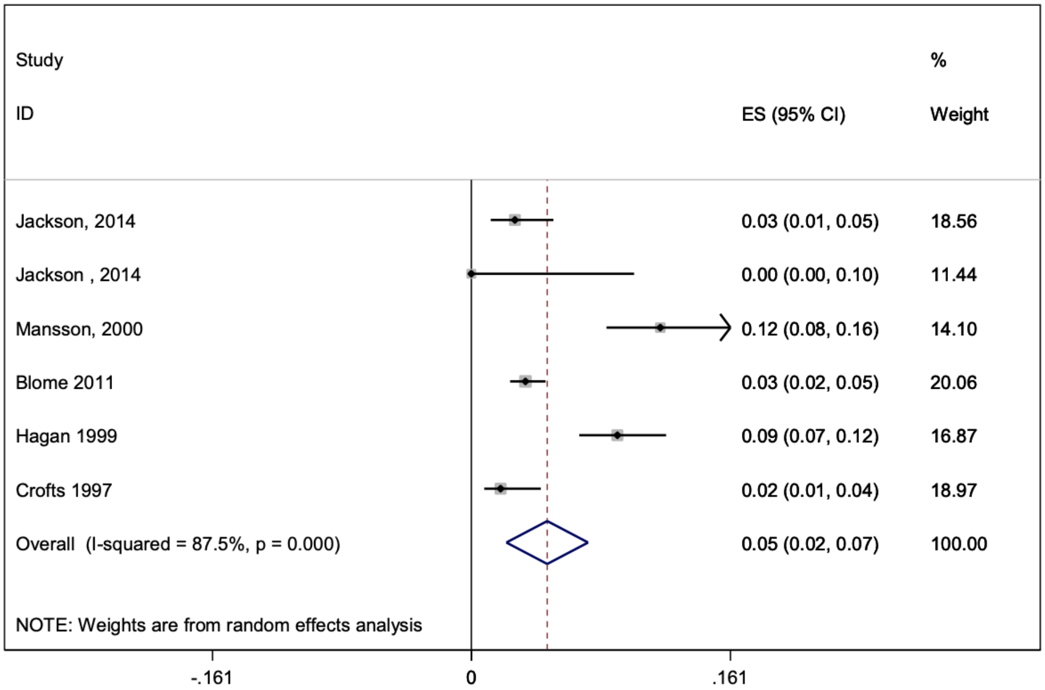

Figure 2: Forest plot of absolute risk of HCV incidence among cohorts of people who inject drugs

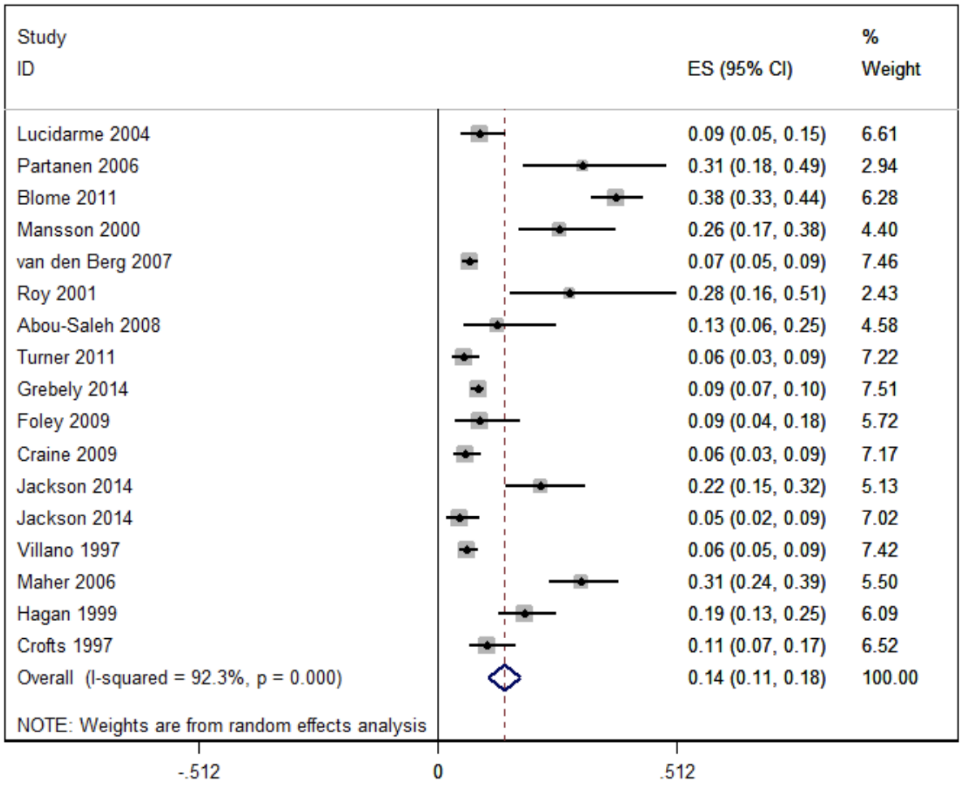

In GBD 2021, we used previously published data[28-54] and estimated the relative risk – as well as evaluated the strength of evidence of the association between drug use disorders and outcomes – for relationships that are not a PAF of 1 or use a direct PAF approach. A description of the methods and approach can be found in the evidence score documentation, section 6.2 (Binary risk-outcome pairs). Funnel plots of the results are shown in Figures X and Y.

Figure 3: Amphetamine use and cocaine use (risk) and suicide (outcome): The data sources used are the same.

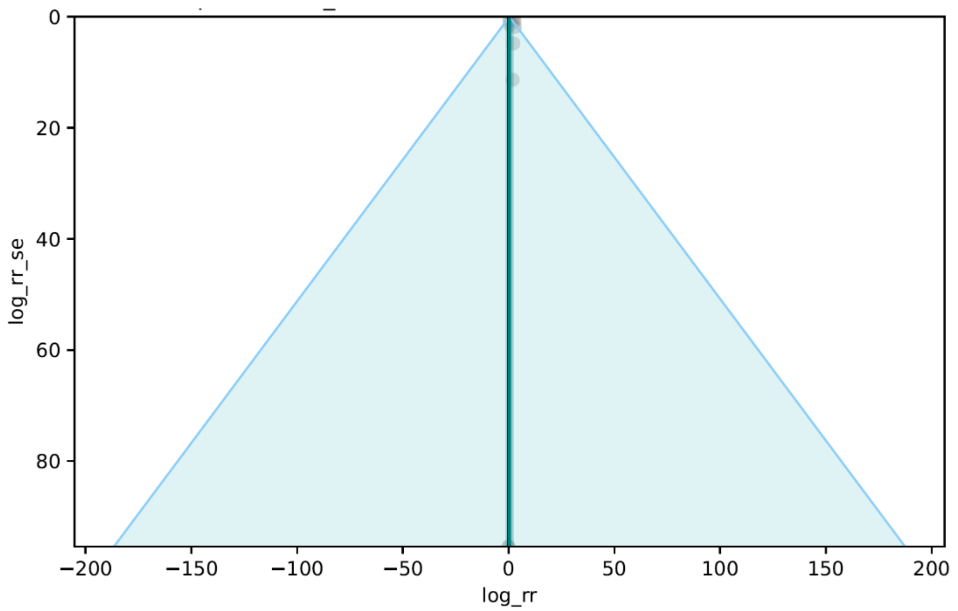

Figure 4: Opioid use (risk) and suicide (outcome)

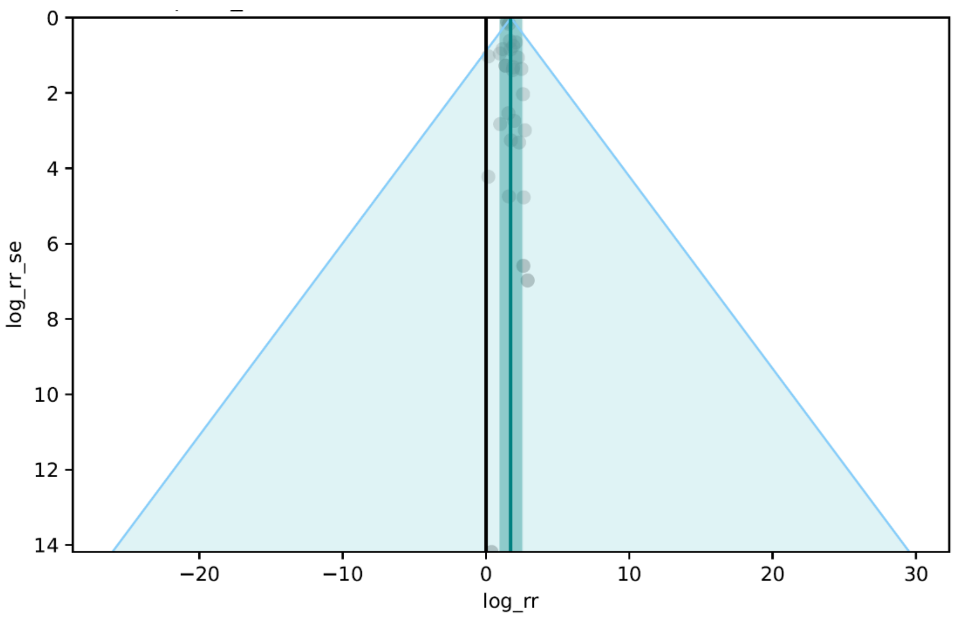

Figure 5: Injection drug use (risk) and hepatitis B (outcome)

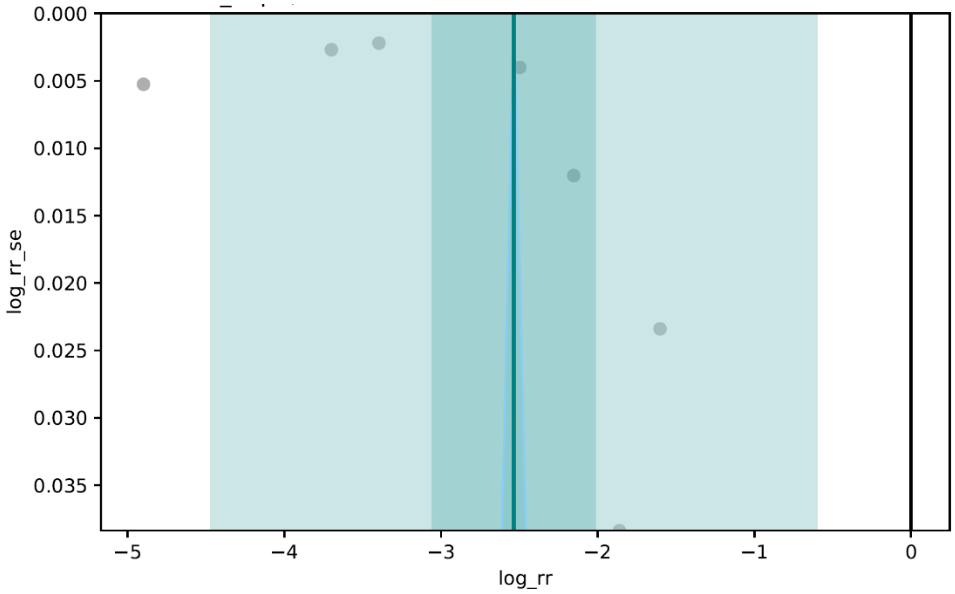

Figure 6: Injection drug use (risk) and hepatitis C (outcome)

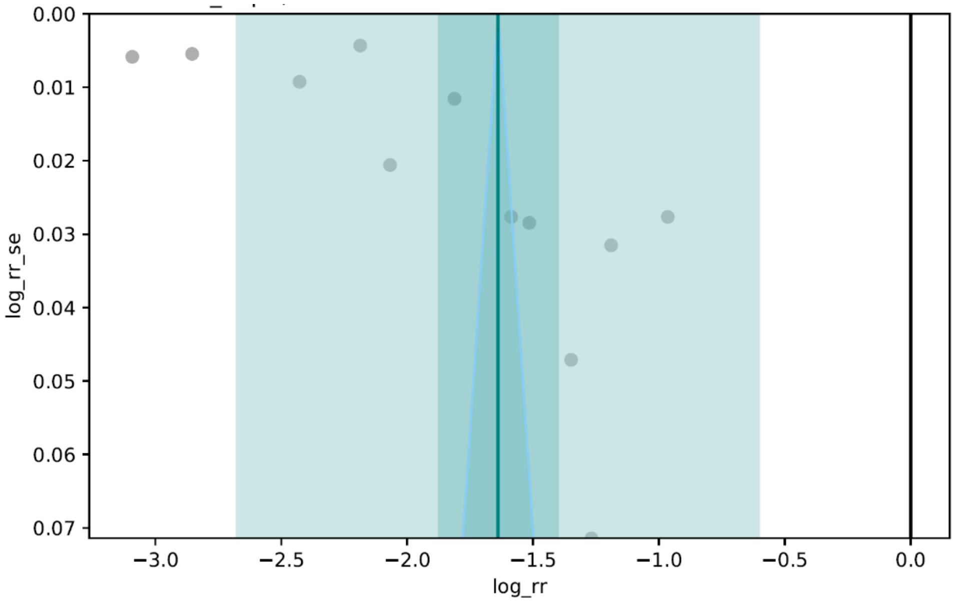

References

1. European Centre for Disease Prevention. HIV/AIDS surveillance in Europe 2014 Solna, Sweden. http://ecdc.europa.eu/en/publications/surveillance_reports/HIV_STI_and_blood_borne_viruses/Pages/HIV_STI_and_blood_borne_viruses.aspx: ECDC, 2014.
2. Family Health International, Bureau of AIDS TB and STIs Department of Disease Control. The Asian Epidemic Model (AEM) Projections for HIV/AIDS in Thailand:2005-2025. Bangkok: Family Health International (FHI) and Bureau of AIDS, TB and STIs, Department of Disease Control, Ministry of Public Health, Thailand, 2008.
3. Kirby Institute. 2015 Annual Surveillance Report of HIV, viral hepatitis, STIs. Sydney, New South Wales. https://kirby.unsw.edu.au/surveillance/2015-annual-surveillance-report-hiv-viral-hepatitis-stis: Kirby Institute, UNSW Australia, 2015.
4. Kirby Institute. Australian NSP survey national data report 2015. Sydney, New South Wales: Kirby Institute, University of New South Wales, 2015.
5. Country reports for Global AIDS Response Progress Reporting [Internet]. UNAIDS. 2014.
6. UNAIDS. UNAIDS Country reports. Geneva: Joint United Nations Programme on HIV/AIDS. http://www.unaids.org/en/regionscountries/countries, 2015.
7. United States Center for Disease Control and Prevention. HIV/AIDS Statistics. Atlanta, Georgia: US CDC. http://www.cdc.gov/hiv/statistics/index.html, 2015.
8. Gouws E, White PJ, Stover J, Brown T. Short term estimates of adult HIV incidence by mode of transmission: Kenya and Thailand as examples. Sex Transm Infect. 2006;82 Suppl 3:iii51-5.
9. Mathers BM, Degenhardt L, Phillips B, Wiessing L, Hickman M, Strathdee SA, et al. Global epidemiology of injecting drug use and HIV among people who inject drugs: a systematic review. Lancet. 2008;372(9651):1733-45.
10. Jackson JB, Wei L, Liping F, Aramrattana A, Celentano DD, Walshe L, et al. Prevalence and Seroincidence of Hepatitis B and Hepatitis C Infection in High Risk People Who Inject Drugs in China and Thailand. Hepatitis research and treatment. 2014;2014.
11. Månsson A-S, Moestrup T, Nordenfelt E, Widell A. Continued transmission of hepatitis B and C viruses, but no transmission of human immunodeficiency virus among intravenous drug users participating in a syringe/needle exchange program. Scandinavian Journal of Infectious Diseases. 2000;32(3):253-8.
12. Blomé MA, Björkman P, Flamholc L, Jacobsson H, Molnegren V, Widell A. Minimal transmission of HIV despite persistently high transmission of hepatitis C virus in a Swedish needle exchange program. Journal of viral hepatitis. 2011;18(12):831-9.
13. Hagan H, McGough JP, Thiede H, Weiss NS, Hopkins S, Alexander ER. Syringe exchange and risk of infection with hepatitis B and C viruses. American journal of epidemiology. 1999;149(3):203-13.
14. Crofts N, Aitken CK. Incidence of bloodborne virus infection and risk behaviours in a cohort of injecting drug users in Victoria in 1990-1995. Medical Journal of Australia. 1997;167(1):17-20.
15. Roy K, Goldberg D, Taylor A, Hutchinson S, MacDonald L, Wilson K, et al. A method to detect the incidence of hepatitis C infection among injecting drug users in Glasgow 1993–98. Journal of Infection. 2001;43(3):200-5.

16. Abou-Saleh M, Davis P, Rice P, Checinski K, Drummond C, Maxwell D, et al. The effectiveness of behavioural interventions in the primary prevention of hepatitis C amongst injecting drug users: a randomised controlled trial and lessons learned. Harm reduction journal. 2008;5(1):1.
17. Turner KM, Hutchinson S, Vickerman P, Hope V, Craine N, Palmateer N, et al. The impact of needle and syringe provision and opiate substitution therapy on the incidence of hepatitis C virus in injecting drug users: pooling of UK evidence. Addiction. 2011;106(11):1978-88.
18. Grebely J, Lima VD, Marshall BD, Milloy M, DeBeck K, Montaner J, et al. Declining incidence of hepatitis C virus infection among people who inject drugs in a Canadian setting, 1996-2012. PloS one. 2014;9(6):e97726.
19. Foley S, Abou-Saleh MT. Risk behaviors and transmission of hepatitis C in injecting drug users. Addictive Disorders & Their Treatment. 2009;8(1):13-21.
20. Craine N, Hickman M, Parry J, Smith J, Walker A, Russell D, et al. Incidence of hepatitis C in drug injectors: the role of homelessness, opiate substitution treatment, equipment sharing, and community size. Epidemiology and Infection. 2009;137(09):1255-65.
21. Villano SA, Vlahov D, Nelson KE, Lyles CM, Cohn S, Thomas DL. Incidence and risk factors for hepatitis C among injection drug users in Baltimore, Maryland. Journal of clinical microbiology. 1997;35(12):3274-7.
22. Maher L, Jalaludin B, Chant KG, Jayasuriya R, Sladden T, Kaldor JM, et al. Incidence and risk factors for hepatitis C seroconversion in injecting drug users in Australia. Addiction. 2006;101(10):1499-508.
23. Lucidarme D, Bruandet A, Ilef D, Harbonnier J, Jacob C, Decoster A, et al. Incidence and risk factors of HCV and HIV infections in a cohort of intravenous drug users in the North and East of France. Epidemiology and infection. 2004;132(04):699-708.
24. Partanen A, Malin K, Perälä R, Harju O, Holopainen A, Holmström P, et al. Riski-tutkimus 2000-2003. Pistämällä huumeita käyttävien seurantatutkimus. A-Klinikkasäätiön Raporttisarja nro 52. Helsinki: A-Klinikkasäätiön, 2006.
25. Van Den Berg C, Smit C, Van Brussel G, Coutinho R, Prins M. Full participation in harm reduction programmes is associated with decreased risk for human immunodeficiency virus and hepatitis C virus: evidence from the Amsterdam Cohort Studies among drug users. Addiction. 2007;102(9):1454-62.
26. Larney S, Kopinski H, Beckwith CG, Zaller ND, Jarlais DD, Hagan H, et al. Incidence and prevalence of hepatitis C in prisons and other closed settings: results of a systematic review and meta-analysis. Hepatology. 2013;58(4):1215-24.
27. Degenhardt L, Mathers B, Vickerman P, Rhodes T, Latkin C, Hickman M. Prevention of HIV infection for people who inject drugs: Why individual, structural, and combination approaches are needed. The Lancet. 2010;376:285-301.
28. Pavarin RM. Cocaine consumption and death risk: a follow-up study on 347 cocaine addicts in the metropolitan area of Bologna. Ann Ist Super Sanita. 2008; 44(1): 91-8.
29. Tyndall MW, Craib KJ, Currie S, Li K, O'Shaughnessy MV, Schechter MT. Impact of HIV infection on mortality in a cohort of injection drug users. J Acquir Immune Defic Syndr. 2001; 28(4): 351-7.
30. Miller CL, Kerr T, Strathdee SA, Li K, Wood E. Factors associated with premature mortality among young injection drug users in Vancouver. Harm Reduct J. 2007; 4: 1.

31. Galli M, Musicco M. Mortality of intravenous drug users living in Milan, Italy: role of HIV-1 infection. COMCAT Study Group. AIDS. 1994; 8(10): 1457-63.
32. Manfredi R, Sabbatani S, Agostini D. Trend of mortality observed in a cohort of drug addicts of the metropolitan area of Bologna, North-Eastern Italy, during a 25-year-period. Coll Antropol. 2006; 30(3): 479-88.
33. Eskild A, Magnus P, Samuelsen SO, Sohlberg C, Kittelsen P. Differences in mortality rates and causes of death between HIV positive and HIV negative intravenous drug users. Int J Epidemiol. 1993; 22(2): 315-20.
34. Ødegård E, Amundsen EJ, Kielland KB. Fatal overdoses and deaths by other causes in a cohort of Norwegian drug abusers – a competing risk approach. Drug Alcohol Depend. 2007; 89(2-3): 176-82.
35. Rossow I. Suicide among drug addicts in Norway. Addiction. 1994; 89(12): 1667-73.
36. Risser D, Hönigschnabl S, Stichenwirth M, Pfudl S, Sebald D, Kaff A, Bauer G. Mortality of opiate users in Vienna, Austria. Drug Alcohol Depend. 2001; 64(3): 251-6.
37. Bartu A, Freeman NC, Gawthorne GS, Codde JP, Holman CDJ. Mortality in a cohort of opiate and amphetamine users in Perth, Western Australia. Addiction. 2004; 99(1): 53-60.
38. Degenhardt L, Randall D, Hall W, Law M, Butler T, Burns L. Mortality among clients of a state-wide opioid pharmacotherapy program over 20 years: risk factors and lives saved. Drug Alcohol Depend. 2009; 105(1): 9–15.
39. Tait RJ, Ngo HTT, Hulse GK. Mortality in heroin users 3 years after naltrexone implant or methadone maintenance treatment. J Subst Abuse Treat. 2008; 35(2): 116-24.
40. Vlahov D, Galai N, Safaeian M, Galea S, Kirk GD, Lucas GM, Sterling TR. Effectiveness of highly active antiretroviral therapy among injection drug users with late-stage human immunodeficiency virus infection. Am J Epidemiol. 2005; 161(11): 999-1012.
41. Vlahov D, Wang C, Ompad D, Fuller CM, Caceres W, Ouellet L, Kerndt P, Jarlais DCD, Garfein RS, Collaborative Injection Drug User Study. Mortality risk among recent-onset injection drug users in five U.S. cities. Subst Use Misuse. 2008; 43(3-4): 413-28.
42. Oppenheimer E, Tobutt C, Taylor C, Andrew T. Death and survival in a cohort of heroin addicts from London clinics: a 22-year follow-up study. Addiction. 1994; 89(10): 1299-308.
43. Goldstein A, Herrera J. Heroin addicts and methadone treatment in Albuquerque: a 22-year follow-up. Drug Alcohol Depend. 1995; 40(2): 139-50.
44. Soyka M, Apelt SM, Lieb M, Wittchen H-U. One-year mortality rates of patients receiving methadone and buprenorphine maintenance therapy: a nationally representative cohort study in 2694 patients. J Clin Psychopharmacol. 2006; 26(6): 657-60.
45. Fugelstad A, Agren G, Romelsjö A. Changes in mortality, arrests, and hospitalizations in nonvoluntarily treated heroin addicts in relation to methadone treatment. Subst Use Misuse. 1998; 33(14): 2803-17.
46. Stenbacka M, Leifman A, Romelsjo A. Mortality Among Opiate Abusers in Stockholm: A Longitudinal Study. Heroin Addict Relate Clin Probl. 2007; 9(3): 41-50.
47. Fugelstad A, Annell A, Rajs J, Agren G. Mortality and causes and manner of death among drug addicts in Stockholm during the period 1981-1992. Acta Psychiatr Scand. 1997; 96(3): 169-75.
48. Antolini G, Pirani M, Morandi G, Sorio C. [Gender difference and mortality in a cohort of heroin users in the Provinces of Modena and Ferrara, 1975-1999]. Epidemiol Prev. 2006; 30(2): 91-9.

49. Digiusto E, Shakeshaft A, Ritter A, O'Brien S, Mattick RP, NEPOD Research Group. Serious adverse events in the Australian National Evaluation of Pharmacotherapies for Opioid Dependence (NEPOD). Addiction. 2004; 99(4): 450-60.
50. Brancato V, Delvecchio G, Simone P. [Survival and mortality in a cohort of heroin addicts in 1985-1994]. Minerva Med. 1995; 86(3): 97-9.
51. Wang C, Vlahov D, Galai N, Cole SR, Bareta J, Pollini R, Mehta SH, Nelson KE, Galea S. The effect of HIV infection on overdose mortality. AIDS. 2005; 19(9): 935-42.
52. Auckloo MBKM, Davies BB. Post-mortem toxicology in violent fatalities in Capte Town, South Africa: A preliminary investigation. J Foresnsic Leg Med. 2019; 63:18-25.
53. Brådvik L. Suicide risk and mental disorders. Int J Environ Res Publ Health. 2019; 15(9):2028.
54 Merrall E, Bird S, Hutchinson SJ. A record-linkage study of drug-related death and suicide after hospital discharge among drug-treatment clients in Scotland, 1996-2006. Addiction. 2012; 102(2).

High alcohol use

Flowchart

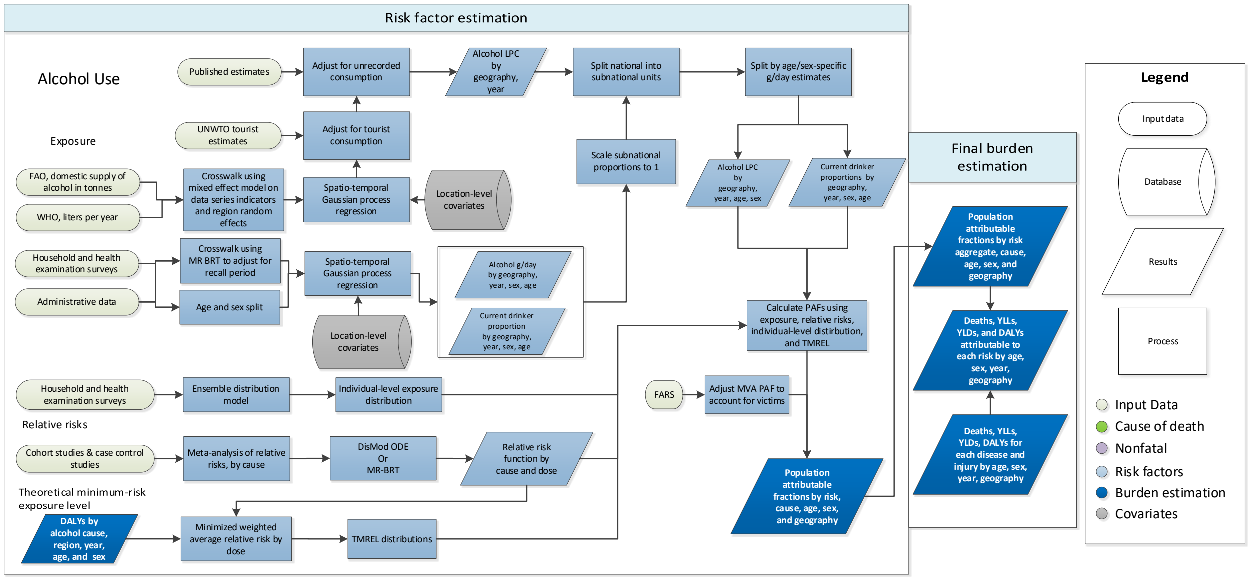

Input data and methodological summary

Definition

Exposure

High alcohol use is defined as alcohol consumption in excess of the theoretical minimum risk exposure level (TMREL), the level of alcohol consumption at which all-cause risk is minimised. Prior to GBD 2021, this risk factor was simply “Alcohol use” and quantified the burden of alcohol consumption over the entire exposure range. More details on the changes to the methodology can be found in the TMREL and “Population attributable fraction” sections of this appendix.

We defined exposure as the grams per day of pure alcohol consumed among current drinkers. We constructed this exposure using the indicators outlined below:

1. Current drinkers, defined as the proportion of individuals who have consumed at least one alcoholic beverage (or some approximation) in a 12-month period.
2. Alcohol consumption (in grams per day), defined as grams of alcohol consumed by current drinkers, per day, over a 12-month period.
3. Alcohol litres per capita (LPC) stock, defined in LPC of pure alcohol, over a 12-month period.

We also used three additional indicators to adjust alcohol exposure estimates to account for different types of bias:

1. Number of tourists within a location, defined as the total amount of visitors to a location within a 12-month period.
2. Tourists' duration of stay, defined as the number of days resided in a hosting country.
3. Unrecorded alcohol stock, defined as a percentage of the total alcohol stock produced outside established markets.

Input data

Exposure

A systematic review of the literature was performed to extract data on our primary indicators. The Global Health Exchange (GHDx), IHME's online database of health-related data, was searched for population survey data containing participant-level information from which we could formulate the required alcohol use indicators on current drinkers and alcohol consumption. Data sources were included if they captured a sample representative of the geographical location under study. We documented relevant survey variables from each data source in a spreadsheet and extracted using STATA 13.1 and R 3.3. A total of 6926 potential data sources were available in the GHDx, of which 5764 have been screened and 1206 accepted.

Table 1: Data inputs for exposure for alcohol use

	Countries with data	New sources	Total sources
Exposure	202	323	10,724

Relative risk

For relative risks, in GBD 2016 we performed a systematic literature review of all cohort and case-control studies reporting a relative risk, hazard ratio, or odds ratio for any riskoutcome pairs studied in GBD 2016. Studies were included if they reported a categorical or continuous dose for alcohol consumption, as well as uncertainty measures for their outcomes, and the population under study was representative.

In GBD 2021, we undertook an effort to update the relative risk curves, beginning with six riskoutcome pairs that were among those associated with the greatest burden: ischaemic heart disease, ischaemic stroke, intracerebral haemorrhage, diabetes mellitus type II, lower respiratory infection, and tuberculosis. We refined the search strings to capture a larger number of studies than identified by previous searches. Studies published between 01/01/1970 and 12/31/2019 were reviewed. Of those articles captured, cohort and case-control studies were included if they reported an association between alcohol use and a GBD outcome, a continuous dose for alcohol consumption, and effect size (relative risk, hazard ratio, or odds ratio) with uncertainty. Information on study type, confounders controlled for, sample representativeness, and measurement of exposure and outcomes was also extracted.

Table 2: Data inputs for relative risks for alcohol use

	Countries with data	New sources	Total sources
Relative risks	63	110	566

Data processing

Estimates of current drinking prevalence were split by age and sex where necessary. First, studies that reported prevalence for both sexes combined were split using a region-specific sex ratio estimated using meta-regression—Bayesian, regularised, trimmed (MR-BRT). Second, where studies reported estimates across non-GBD age groups, these were split into standard five-year age groups using the global age pattern estimated by ST-GPR.

Table 3: MR-BRT sex splitting adjustment factors for current drinking

Data input	Gamma	Beta coefficient, log (95% CI)	Adjustment factor*
Female: Male	0	-0.16 (-0.17 to -0.14)	0.85
Age <50	0	0.06 (0.06 to 0.06)	1.07
East Asia	0.36	-1.02 (-1.74 to -0.29)	0.36
Southeast Asia	0.64	-1.06 (-2.34 to 0.22)	0.35
Central Asia	0.41	-0.35 (-1.16 to 0.46)	0.70
Central Europe	0.18	-0.21 (-0.58 to 0.14)	0.80
Eastern Europe	0.10	-0.07 (-0.28 to 0.14)	0.93
High-income Asia Pacific	1.27	-1.11 (-4.90 to 2.68)	0.33
Western Europe	0.08	0.03 (-0.14 to 0.20)	1.03
Southern Latin America	1.26	-0.67 (-4.18 to 2.84)	0.51
High-income North America	0.09	-0.07 (-0.26 to 0.11)	0.93
Caribbean	0.25	-0.52 (-1.02 to -0.03)	0.59

Andean Latin America	0.76	-0.16 (-1.66 to 1.34)	0.85
Central Latin America	0.30	-0.52 (-1.12 to 0.08)	0.59
Tropical Latin America	0.08	-0.61 (-0.79 to -0.44)	0.54
North Africa and Middle East	1.21	-1.44 (-3.91 to 1.03)	0.24
South Asia	0.71	-1.17 (-2.57 to 0.23)	0.31
Eastern sub-Saharan Africa	0.28	-0.53 (-1.10 to, 0.03)	0.58
Southern sub-Saharan Africa	0.20	-0.16 (-0.56 to 0.23)	0.85
Western sub-Saharan Africa	0.32	-0.19 (-0.83 to 0.45)	0.83
Oceania	0.94	-0.54 (-2.42 to 1.34)	0.58

**Adjustment factor is the transformed beta coefficient in normal space and can be interpreted as the factor by which the alternative case definition is adjusted to reflect the ratio by which both-sex datapoints were split.*

To allow for the inclusion of data that did not meet our reference definition for current drinking, two crosswalks were performed using MR-BRT. The first crosswalk converted estimates of one-month drinking prevalence to what they would be if data represented estimates of 12-month drinking prevalence. This crosswalk incorporated two binary covariates: male sex and age ≥50. The second crosswalk converted estimates of one-week drinking prevalence to 12-month drinking prevalence. This crosswalk incorporated age <20 and male sex as covariates. The covariates utilised in both crosswalks were included as both x and z covariates. A uniform prior of 0 was set as the upper bound for the beta coefficients to enforce the logical constraint that one-month and one-week prevalence could not be greater than 12-month prevalence.

Table 4: MR-BRT crosswalk adjustment factors for alcohol use current drinking model

Data input	Reference or alternative case definition	Gamma	Beta coefficient, logit (95% UI)*	Adjustment factor**

12-month prevalence	Ref	---	---	---
1-month prevalence	Alt	0.22	-0.60 (-1.05, -0.16)	0.55 (0.35, 0.85)
Age ≥50		0.13	0.16 (-0.10, 0.43)	1.17 (0.9, 1.54)
Male		0.29	0.01 (-0.57, 0.59)	1.01 (0.57, 1.8)
1-week prevalence	Alt	0.46	-1.51 (-2.42, -0.59)	0.22 (0.09, 0.55)
Age <20		0.47	-0.29 (-1.34, 0.76)	0.75 (0.26, 2.14)
Male		0.00	0.38 (0.15, 0.60)	1.46 (1.16, 1.82)

**MR-BRT crosswalk adjustments can be interpreted as the factor the alternative case definition is adjusted by to reflect what it would have been had it been measured using the reference case definition. If the log/logit beta coefficient is negative, then the alternative is adjusted up to the reference. If the log/logit beta coefficient is positive, then the alternative is adjusted down to the reference.*
***The adjustment factor column is the exponentiated beta coefficient. For log beta coefficients, this is the relative rate between the two case definitions. For logit beta coefficients, this is the relative odds between the two case definitions.*

The raw data included in the supply-side model are domestic supply (FAO[1]; WHO GISAH[2]) and retail supply (Euromonitor[3]) of litres of pure ethanol consumed. Domestic supply is calculated as the sum of production and imports, subtracting exports. The WHO and FAO sources were combined, such that FAO data were only used if there were no data available for that location-year from WHO. This was done to account for the WHO source considering FAO values when available in its estimates. Because the WHO data are given in more granular alcohol types than used in modelling, the following adjustments were made:

$$LPC\ Pure\ Ethanol = 0.13 * \left(\frac{Wine}{0.973}\right)$$

$$LPC\ Pure\ Ethanol = 0.05 * \left(\frac{Beer}{0.989}\right)$$

$$LPC\ Pure\ Ethanol = 0.4 * \left(\frac{Spirits}{0.91}\right)$$

Three outliering strategies are used to omit implausible datapoints and data that created implausible model fluctuations. First, estimates from the current drinking model are used to calculate the grams of alcohol consumed per drinker per day. A point is outliered if the grams of pure ethanol per drinker per day estimate for a given source-location-year is greater than 100 (approximately 10 drinks). These thresholds were selected by consulting expert knowledge about reasonable consumption levels. In the second round of outliering, the mean LPC value over a 10-year window is calculated. If an individual datapoint differs from the calculated mean value by greater than 70% of the calculated mean value, it is considered an outlier. The 70% limit was determined by examining histograms of these distances. Additionally, manual outliering is performed to account for edge cases. Finally, data smoothing is performed by taking a three-year rolling mean over each location-year.

Next, an imputation to fill in missing years is performed for all series to compensate for compositional bias from our final estimates. Because our main sources report data from different time periods, imputing a complete time series for each data series reduces the probability that compositional bias of the sources results in biased final estimates. To impute missing years for each series, we model the log ratio of each pair of sources as a function of an intercept and nested random effects on super-region, region, and location. The corresponding predicted ratio is multiplied by the relevant existing source data, generating an estimated value for the missing source. For some locations where there was limited overlap between series, the predicted ratio appeared nonsensical, and thus a regional ratio was used.

Finally, variance was calculated both across series (within a location-year) as well as across years (within a location-source). Additionally, if a location-year had an imputed datapoint, the variance was inflated by a factor 2. If a location-year had two imputed datapoints, the variance was subsequently multiplied by 4. The average estimates in each location-year served as the input to an ST-GPR model. This uses a mixed-effects model modelled in log space with nested location random effects.

We obtained data on the number of tourists and their duration of stay from the UN World Tourism Organization.[4] We applied a crosswalk across different tourist categories, mimicking the technique applied to the LPC data, to arrive at a consistent definition (ie, visitors to a country).

We then obtained estimates on unrecorded alcohol stock from data available in WHO GISAH database,[2] consisting of 189 locations. For locations with no data available, the national or regional average was used.

Modelling strategy

Exposure

While population-based surveys provide accurate estimates of the prevalence of current drinkers, they typically underestimate real alcohol consumption levels.[5-7] As a result, we considered the LPC input to be a better estimate of overall volume of consumption. Per capita consumption, however, does not provide age- and sex-specific consumption estimates needed to compute alcohol-attributable burden of disease. Therefore, we use the age-sex pattern of consumption among drinkers modelled from the population survey data and the overall volume of consumption from FAO[1], GISAH[2], and Euromonitor[3] to determine the total amount of alcohol consumed within a location. In the paragraphs that follow, we outline how we estimated each primary input in the alcohol exposure model, as well as how we combined these inputs to arrive at our final estimate of grams per day of pure alcohol. We estimated all models using 1000 draws.

For data obtained through surveys, we used spatiotemporal Gaussian process regression (ST-GPR) to construct estimates for each location/year/age/sex. We chose to use ST-GPR due to its ability to leverage information across the nearby locations or time periods. We also modelled the alcohol LPC data, as well as the total number of tourists, using ST-GPR. To improve the LPC model fit for years beyond those in which data was available, we forecasted ST-GPR estimates using a damped holt function.

Given the heterogeneous nature of the estimates on unrecorded consumption, as well as the wide variation across countries and time periods, we took 1000 draws from the uniform distribution of the

lowest and highest estimates available for a given country. We did this to incorporate the diffuse uncertainty within the unrecorded estimates reported. We used these 1000 draws in the equation below.

We adjusted the alcohol LPC for unrecorded consumption using the following equation:

$$Alcohol\ LPC = \frac{Alcohol\ LPC}{(1 - \%\ Unrecorded)}$$

We then adjusted the estimates for alcohol LPC for tourist consumption by adding in the per capita rate of consumption abroad and subtracting the per capita rate of tourist consumption domestically.

$$Alcohol\ LPC_d = Unadjusted\ Alcohol\ LPC_d + Alcohol\ LPC_{Domestic\ consumption\ abroad} - Alcohol\ LPC_{Tourist\ consumption\ domestically}$$

$$Alcohol\ LPC_i = \frac{\sum_l Tourist\ Population_l * Proportion\ of\ tourists_{i,l} * Unadjusted\ Alcohol\ LPC_l * \frac{Average\ length\ of\ stay_{i,l}}{365} *}{Population_d}$$

where:

l *is the set of all locations,* i *is either* Domestic consumption abroad *or* Tourist consumption domestically, *and* d *is a domestic location.*

After adjusting alcohol LPC by tourist consumption and unrecorded consumption for all location/years reported, sex-specific and age-specific estimates were generated by incorporating estimates modelled in ST-GPR to determine the overall percentage of current drinkers within a location/year/sex/age. Consumption trends were also modelled in ST-GPR using the grams per day model. We do this by first calculating the proportion of total consumption for a given location/year by age and sex, using the estimates of alcohol consumed per day, the population size, and the percentage of current drinkers. We then multiply this proportion of total stock for a given location/year/sex/age by the total stock for a given location/year to calculate the consumption in terms of LPC for a given location/year/sex/age. We then convert these estimates to be in terms of grams/per day. The following equations describe these calculations:

$$Proportion\ of\ total\ consumption_{l,y,s,a} = \frac{Alcohol\ \ g/day_{l,y,s,a} * Population_{l,y,s,a} * \%\ Current\ drinkers_{l,y,s,a}}{\sum_{s,a} Alcohol\ \ g/day_{l,y,s,a} * Population_{l,y,s,a} * \%\ Current\ drinkers_{l,y,s,a}}$$

$$Alcohol\ LPC_{l,y,s,a} = \frac{Alcohol\ LPC_{l,y} * Population_{l,y} * Proportion\ of\ total\ consumption_{l,y,s,a}}{\%\ Current\ drinkers_{l,y,s,a} * Population_{l,y,s,a}}$$

$$Alcohol\ g/day_{\,l,y,s,a} = Alcohol\ LPC_{\,l,y,s,a} * \frac{789\ g/L}{365}$$

where:

$l\ is\ a\ location, y\ is\ a\ year, s\ is\ a\ sex, and\ a\ is\ an\ age\ group.$

We then used the gamma distribution to estimate individual-level variation within location, year, sex, age drinking populations, following the recommendations of other published alcohol studies.[7,8] We chose parameters of the gamma distribution based on the mean and standard deviation of the 1000 draws of alcohol g/day exposure for a given population. Standard deviation was calculated using the following formula. We tested several alternative models using our data and found this model performed best.

$$standard\ deviation = mean * (0.087 * female + 1.171)$$

Theoretical minimum risk exposure level

The methods for calculating the TMREL were updated for GBD 2021. Previously, one global estimate of the TMREL was calculated. However, the contributions of each cause to overall health loss vary over geography, age, time, and sex, suggesting that the amount of alcohol that minimises health loss similarly varies over these domains. For this reason, in GBD 2021 we estimated an individual TMREL for each region, age, sex, and year.

For each region, age, sex, and year, we calculated TMREL by first calculating the overall risk attributable to alcohol. We did this by weighting each relative risk curve by the share of overall DALYs for a given cause. We then took the minimum of this overall-risk curve as the TMREL of alcohol use. More formally,

$$TMREL = argmin\ average\ overall\ risk_{\omega}(g/day)$$

$$Average\ overall\ risk_{\omega,l,y,a,s}(g/day) = \sum_{i}^{\omega} \log\left(RR_i(g/day)\right) * \frac{DALY_{i,l,y,a,s}}{\sum_i^{\omega} DALY_{i,l,y,a,s}}$$

Where:

$\omega\ is\ the\ set\ of\ causes\ associated\ with\ alcohol, i\ is\ a\ given\ cause\ from\ that\ set,$
$l\ is\ a\ location,$ y $is\ a\ year, s\ is\ a\ sex, a\ is\ an\ age\ group, DALY\ is\ the\ DALY\ rate,$
$and\ RR\ is\ the\ dose\ response\ curve\ for\ a\ given\ cause\ and\ exposure\ level\ in\ grams\ per\ day.$

In other words, we chose TMREL as being the exposure that minimises the risk of suffering burden from any given cause related to alcohol. We weight the risk for a particular cause in our aggregation by the proportion of DALYs due to that cause (eg, since more observed people die from ischaemic heart disease, we weight the risk for ischaemic heart disease more in the above calculation of average risk compared to, say, diabetes, even if both have the same relative risk for a given level of consumption).

Figure 1: TMREL by region, age, and sex, 2020

Female

Region	15 to 19	20 to 24	25 to 29	30 to 34	35 to 39	40 to 44	45 to 49	50 to 54	55 to 59	60 to 64	65 to 69	70 to 74	75 to 79	80 plus
Global	0.1	0.2	0.2	0.3	0.4	0.4	0.5	0.5	0.6	0.6	0.6	0.6	0.7	0.7
Eastern Europe	0	0	0.1	0.2	0.3	0.3	0.4	0.5	0.6	0.7	0.8	1.1	1.4	1.7
Central Europe	0	0	0.1	0.2	0.3	0.4	0.4	0.5	0.6	0.6	0.7	0.7	0.7	0.9
Central Asia	0.1	0.2	0.3	0.3	0.4	0.5	0.5	0.6	0.7	0.8	0.9	0.9	1.2	1.4
Western Europe	0	0	0.1	0.2	0.2	0.2	0.3	0.3	0.4	0.5	0.6	0.6	0.6	0.6
Southern Latin America	0	0	0	0.1	0.2	0.3	0.4	0.5	0.5	0.6	0.6	0.6	0.6	0.7
High−income North America	0	0	0.1	0.2	0.3	0.4	0.5	0.6	0.6	0.7	0.7	0.7	0.7	0.8
High−income Asia Pacific	0	0.1	0.1	0.2	0.2	0.3	0.3	0.4	0.4	0.5	0.5	0.5	0.6	0.6
Australasia	0	0	0	0	0.1	0.1	0.2	0.3	0.4	0.5	0.5	0.6	0.6	0.7
Tropical Latin America	0	0	0.1	0.2	0.4	0.5	0.6	0.6	0.7	0.7	0.8	0.7	0.7	0.7
Central Latin America	0	0.1	0.2	0.3	0.5	0.6	0.7	0.8	0.8	0.8	0.9	0.9	0.9	1
Caribbean	0.1	0.2	0.3	0.4	0.5	0.6	0.6	0.7	0.7	0.8	0.8	0.8	0.8	0.8
Andean Latin America	0.1	0.1	0.2	0.2	0.3	0.4	0.5	0.6	0.6	0.7	0.7	0.7	0.7	0.7
North Africa and Middle East	0.3	0.3	0.3	0.4	0.5	0.5	0.6	0.7	0.8	0.9	0.9	0.9	0.9	0.9
South Asia	0.1	0.1	0.2	0.4	0.4	0.5	0.6	0.6	0.7	0.7	0.7	0.7	0.7	0.7
Southeast Asia	0.2	0.4	0.4	0.4	0.5	0.5	0.5	0.5	0.6	0.6	0.6	0.6	0.6	0.6
Oceania	0.2	0.3	0.4	0.5	0.6	0.7	0.7	0.7	0.8	0.8	0.8	0.8	0.7	0.7
East Asia	0.1	0.2	0.3	0.3	0.4	0.4	0.5	0.5	0.5	0.6	0.6	0.6	0.6	0.7
Western Sub−Saharan Africa	0.2	0.2	0.2	0.3	0.4	0.4	0.5	0.5	0.5	0.6	0.6	0.6	0.6	0.6
Southern Sub−Saharan Africa	0.3	0.4	0.4	0.3	0.3	0.4	0.5	0.6	0.6	0.7	0.7	0.7	0.7	0.7
Eastern Sub−Saharan Africa	0.2	0.3	0.3	0.4	0.4	0.4	0.4	0.5	0.5	0.5	0.5	0.5	0.5	0.6
Central Sub−Saharan Africa	0.2	0.4	0.5	0.5	0.5	0.5	0.5	0.6	0.6	0.6	0.6	0.6	0.6	0.6

Male

Region	15 to 19	20 to 24	25 to 29	30 to 34	35 to 39	40 to 44	45 to 49	50 to 54	55 to 59	60 to 64	65 to 69	70 to 74	75 to 79	80 plus
Global	0	0	0.1	0.2	0.3	0.4	0.5	0.5	0.6	0.6	0.6	0.6	0.6	0.7
Eastern Europe	0	0	0	0.1	0.2	0.4	0.4	0.5	0.6	0.8	1	1.2	1.5	1.9
Central Europe	0	0	0	0.1	0.3	0.4	0.5	0.5	0.6	0.6	0.7	0.7	0.7	0.9
Central Asia	0	0	0.1	0.2	0.4	0.5	0.6	0.7	0.8	0.8	0.9	1	1.4	1.4
Western Europe	0	0	0	0.1	0.3	0.4	0.4	0.5	0.6	0.6	0.7	0.7	0.7	0.7
Southern Latin America	0	0	0	0	0.1	0.3	0.4	0.5	0.6	0.6	0.7	0.7	0.7	0.7
High−income North America	0	0	0	0.1	0.3	0.5	0.6	0.7	0.7	0.8	0.8	0.9	0.9	1.1
High−income Asia Pacific	0	0.1	0.1	0.2	0.3	0.4	0.4	0.5	0.5	0.5	0.5	0.5	0.5	0.6
Australasia	0	0	0	0	0.1	0.3	0.4	0.5	0.5	0.6	0.7	0.7	0.7	0.9
Tropical Latin America	0	0	0	0	0.1	0.3	0.4	0.5	0.6	0.6	0.7	0.7	0.7	0.7
Central Latin America	0	0	0	0.1	0.2	0.3	0.5	0.7	0.8	0.8	0.9	0.9	0.9	1
Caribbean	0	0	0	0.1	0.3	0.4	0.5	0.6	0.7	0.7	0.8	0.8	0.8	0.8
Andean Latin America	0	0	0	0.1	0.1	0.3	0.4	0.5	0.6	0.6	0.7	0.7	0.7	0.7
North Africa and Middle East	0.1	0.1	0.2	0.3	0.4	0.5	0.7	0.9	0.9	0.9	1	0.9	0.9	0.9
South Asia	0	0	0.1	0.3	0.4	0.5	0.6	0.6	0.7	0.7	0.7	0.7	0.8	0.8
Southeast Asia	0	0.1	0.2	0.3	0.4	0.4	0.5	0.5	0.5	0.6	0.6	0.6	0.6	0.6
Oceania	0	0.1	0.2	0.5	0.6	0.7	0.8	0.9	0.9	0.9	0.9	0.8	0.8	0.8
East Asia	0	0.1	0.2	0.3	0.4	0.4	0.4	0.5	0.5	0.5	0.5	0.5	0.5	0.6
Western Sub−Saharan Africa	0	0	0	0.1	0.2	0.4	0.4	0.5	0.5	0.5	0.6	0.6	0.6	0.6
Southern Sub−Saharan Africa	0	0	0	0	0.1	0.3	0.4	0.5	0.6	0.6	0.6	0.7	0.7	0.7
Eastern Sub−Saharan Africa	0	0	0.1	0.3	0.3	0.4	0.4	0.5	0.5	0.5	0.5	0.5	0.5	0.6
Central Sub−Saharan Africa	0	0	0.1	0.1	0.2	0.4	0.5	0.6	0.6	0.6	0.6	0.6	0.6	0.7

Relative risk

For GBD 2016 through 2019, we used the studies identified through a systematic review to calculate a dose–response, modelled using DisMod ODE. We chose DisMod ODE rather than a conventional mixed-effects meta-regression because of its ability to estimate non-parametric splines over doses (ie, for most alcohol causes, there is a non-linear relationship with different doses) and incorporate heterogeneous doses through dose-integration (ie, most studies report doses categorically in wide ranges. DisMod ODE estimates specific doses when categories overlap across studies, through an integration step.). We used the results of the meta-regression to estimate a non-parametric curve for all doses between zero and 100 g/day and their corresponding relative risks. For all causes, we assumed the relative risk was the same for all ages and sexes.

For GBD 2021, we used the studies identified through the updated systematic review to estimate new dose–response curves using MR-BRT for six outcomes among those associated with the greatest burden: ischaemic heart disease, ischaemic stroke, intracerebral haemorrhage, diabetes mellitus type II, lower

respiratory infection, and tuberculosis. The relative risk curves for the remaining outcomes will be modelled using MR-BRT instead of DisMod ODE in the coming GBD rounds. Importantly, this new method takes into account the risk of biases in the relative risk estimation and incorporates unexplained between-study heterogeneity into the uncertainty of the relative risk estimates. The results of the meta-regression were used to estimate a non-parametric curve for all doses between zero and 100 g/day and their corresponding relative risks.

We implemented the Fisher Scoring correction to the heterogeneity parameter, which corrects for data-sparse situations. In such cases, the between-study heterogeneity parameter estimate may be 0, simply from lack of data. The Fisher Scoring correction uses a quantile of gamma, which is sensitive to the number of studies, study design, and reported uncertainty.

We have also added methodology that can detect and flag publication bias. The approach is based on the classic Egger's Regression strategy, which is applied to the residuals in our model. In the current implementation, we do not correct for publication bias, but flag the risk-outcome pairs where the risk for publication bias is significant.

In the table below, we list each risk-outcome pair that is updated in GBD 2021 along with several of the key modelling parameters and results. The formulation for MR-BRT is described in detail in the MR-BRT section of the appendix.

Table 5: MR-BRT splines and priors by type of risk

Riskoutcome	Type of risk	Spline degree, # interior knots	Priors and constraints
Ischaemic heart disease	J-shaped	Quadratic, 2 I knots	No monotonicity constraint
Ischaemic stroke	J-shaped	Quadratic, 3 I knots	No monotonicity constraint, right linear tail
Intracerebral haemorrhage	J-shaped	Cubic, 3 I knots	No monotonicity constraint, right linear tail
Type II diabetes mellitus	J-shaped	Cubic, 3 I knots	No monotonicity constraint, right linear tail
Tuberculosis	Harmful	Quadratic, 3 I knots	Monotonic increasing, right linear tail, Gaussian max derivative prior on the right tail (0, 0.001)
Lower respiratory infection	Harmful	Quadratic, 3 I knots	Monotonic increasing, right linear tail, Gaussian max derivative prior on the right tail (0, 0.001)

Table 6: MR-BRT parameters by riskoutcome pair

Riskoutcome	Type of risk	Selected covariates	Mean gamma solution	Publication bias result
Ischaemic heart disease	J-shaped	cv_incidence	0.158	No publication bias
Ischaemic stroke	J-shaped	cv_incidence	0.234	No publication bias
Intracerebral haemorrhage	J-shaped	cv_adjusted_2, cv_adjusted_1	0.09	No publication bias
Type II diabetes mellitus	J-shaped	None	0.117	No publication bias
Tuberculosis	Harmful	cv_sick_quitters, cv_incidence	19.488	No publication bias
Lower respiratory infection	Harmful	None	0	No publication bias

After evaluating all available evidence, we found insufficient evidence for a relationship between alcohol use and lower respiratory infection. Specifically, a simplified log-linear model was run, including only exposed and reference group dose data and study id as covariates, and a one-sided z-test was performed for the fixed-effects only model at alpha value set to 0.1. Based on this test, we removed alcohol use vs. lower respiratory infection as a risk-outcome pair for GBD 2021.

Regarding injuries outcomes, we constructed relative risks based on chronic exposure to alcohol rather than acute exposure immediately preceding injury, which has a weaker relationship to the outcome, though still significant.[9-15] We decided to use chronic exposure given the lack of available data on acute exposure, as well as the lack of cohort studies using acute exposure as a metric. Further, using chronic exposure allowed us to construct relative risks curves for unintentional injuries, interpersonal violence, motor vehicle accidents, and self-harm using the same method as reported above.

Population attributable fraction

We calculated population attributable fractions (PAFs) by setting the relative risk of alcohol consumption among abstainers and drinkers consuming alcohol below the TMREL to be 1. We then calculated PAFs for drinkers consuming alcohol in excess of the TMREL as we have previously. For each location, age, sex, year, and cause, we defined PAF as:

$$PAF(x) = \frac{P_A + \int_0^{TMREL} P(x)\ dx + \int_{TMREL}^{100} P(x) * RR_C(x)\ dx - RR_C(TMREL)}{P_A + \int_0^{TMREL} P(x)\ dx + \int_{TMREL}^{100} P(x) * RR_C(x)\ dx} \qquad P(x) = P_C * \Gamma(\boldsymbol{p})$$

where:

$$P_C \text{ is the prevalence of current drinkers}, P_A \text{ is the prevalence of abstainers, and}$$

$$\boldsymbol{p} \text{ are parameters determined by the mean and sd of exposure for that location, age, sex, and year;}$$

$$RR_c(x) \text{ is the global relative risk function for current drinkers for a given cause, and}$$

$$TMREL \text{ is the theoretical minimum risk exposure level for that location's region, age, sex, and year}$$

We performed the above equation for 1000 draws of the exposure and relative risk models. We then used the estimated PAF draws to calculate YLL, YLDs, and DALYs, as per the other risk factors.

For outcomes that are by definition caused by alcohol, such as liver cancer or cirrhosis due to alcohol use, PAFs are set to 1. PAFs for cirrhosis due to all causes that are in excess of the proportion of all cirrhosis burden due to alcohol are proportionally redistributed over cirrhosis due to hepatitis B, hepatitis C, and other causes. Similarly, PAFs for liver cancer due to all causes that are in excess of the proportion of all liver cancer burden due to alcohol are proportionally redistributed over liver cancer due to hepatitis B, hepatitis C, and other causes.

In the case of motor vehicle accidents, we adjusted the PAF to account for victims of drunk drivers who are involved in accidents. Using data from the Fatality Analysis Reporting System (FARS) in the US,[16] we calculated the average number of fatalities in a car crash involving alcohol, as well as the percentage of those fatalities distributed by age and sex (Figures 2 and 3). We aggregated FARS data across the years 1985–2015, given there was little variation in the data temporally and the number of cases in old age groups had too much variance when constructing estimates by year. To adjust PAFs, we multiplied attributable deaths by the average number of fatalities from FARS and redistributed the PAF among each population, based on the probability of being a victim to a certain drunk driver by age and sex, based on the FARS data. The following equation describes this process:

$$Adjusted\ PAF_i = \frac{\sum_d PAF_d * DALY_d * Avg\ Fatalities_d * \ P(i \text{ is a victim})_d}{DALY_i}$$

where:

$$i \text{ is a population by location, year, age, sex and}$$
$$d \text{ is the set of all age and sex exposed groups within that location and year.}$$

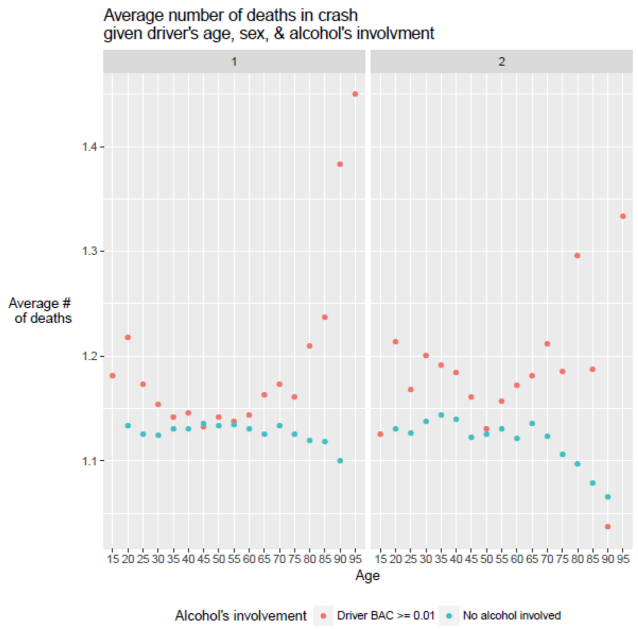

Figure 2

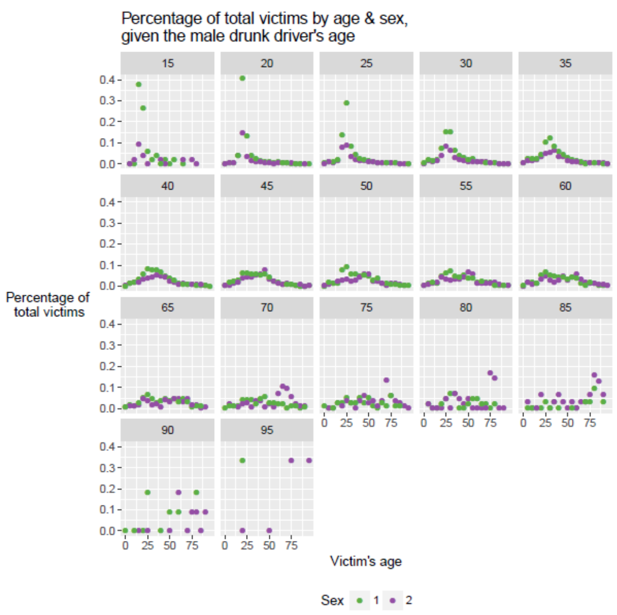

Figure 3

Citations

1. Food and Agriculture Organization of the United Nations (FAO). FAOSTAT Food Balance Sheets, October 2014. Rome, Italy: Food and Agriculture Organization of the United Nations (FAO).
2. World Health Organization (WHO). WHO Global Health Observatory - Recorded adult per capita alcohol consumption, Total per country. Geneva, Switzerland: World Health Organization (WHO).
3. Euromonitor International. Euromonitor Passport - Alcoholic Drinks Statistics. London, United Kingdom: Euromonitor International.
4. UN World Tourism Organization (UNWTO). UN World Tourism Organization Compendium of Tourism Statistics 2015 [Electronic]. Madrid, Spain: UN World Tourism Organization (UNWTO), 2016.

5. Ramstedt, Mats. "How much alcohol do you buy? A comparison of self-reported alcohol purchases with actual sales." *Addiction* 105.4 (2010): 649-654.
6. Stockwell, Tim, et al. "Under-reporting of alcohol consumption in household surveys: a comparison of quantity–frequency, graduated–frequency and recent recall." *Addiction* 99.8 (2004): 1024-1033.
7. Kerr, William C., and Thomas K. Greenfield. "Distribution of alcohol consumption and expenditures and the impact of improved measurement on coverage of alcohol sales in the 2000 National Alcohol Survey." *Alcoholism: Clinical and Experimental Research* 31.10 (2007): 1714-1722.
8. Kehoe, Tara et al. "Determining the best population-level alcohol consumption model and its impact on estimates of alcohol-attributable harms." Population health metrics 10 6. (2012)
9. Taylor, Bruce, et al. "The more you drink, the harder you fall: a systematic review and meta-analysis of how acute alcohol consumption and injury or collision risk increase together." *Drug and alcohol dependence* 110.1 (2010): 108-116.
10. Vinson, Daniel C., Guilherme Borges, and Cheryl J. Cherpitel. "The risk of intentional injury with acute and chronic alcohol exposures: a case-control and case-crossover study." *Journal of studies on alcohol* 64.3 (2003): 350-357.
11. Vinson, Daniel C., et al. "A population-based case-crossover and case-control study of alcohol and the risk of injury." *Journal of studies on alcohol*64.3 (2003): 358-366.
12. Chen, Li-Hui, Susan P. Baker, and Guohua Li. "Drinking history and risk of fatal injury: comparison among specific injury causes." Accident Analysis & Prevention 37.2 (2005): 245-251.
13. Bell, Nicole S., et al. "Self-reported risk-taking behaviors and hospitalization for motor vehicle injury among active duty army personnel." American journal of preventive medicine 18.3 (2000): 85-95.
14. Margolis, Karen L., et al. "Risk factors for motor vehicle crashes in older women." The Journals of Gerontology Series A: Biological Sciences and Medical Sciences 57.3 (2002): M186-M191.
15. Sorock, Gary S., et al. "Alcohol-drinking history and fatal injury in older adults." Alcohol 40.3 (2006): 193-199.
16. Fatal Accident Reporting System (FARS). **National Highway Traffic Safety Administration, National Center for Statistics and Analysis Data Reporting and Information Division (NVS-424); 1985, 1990, 1995, 2000, 2005, 2010, 2015**

High body-mass index

Flowchart

Adult (Ages 20+) High Body-Mass Index: Data and Model Flow Chart

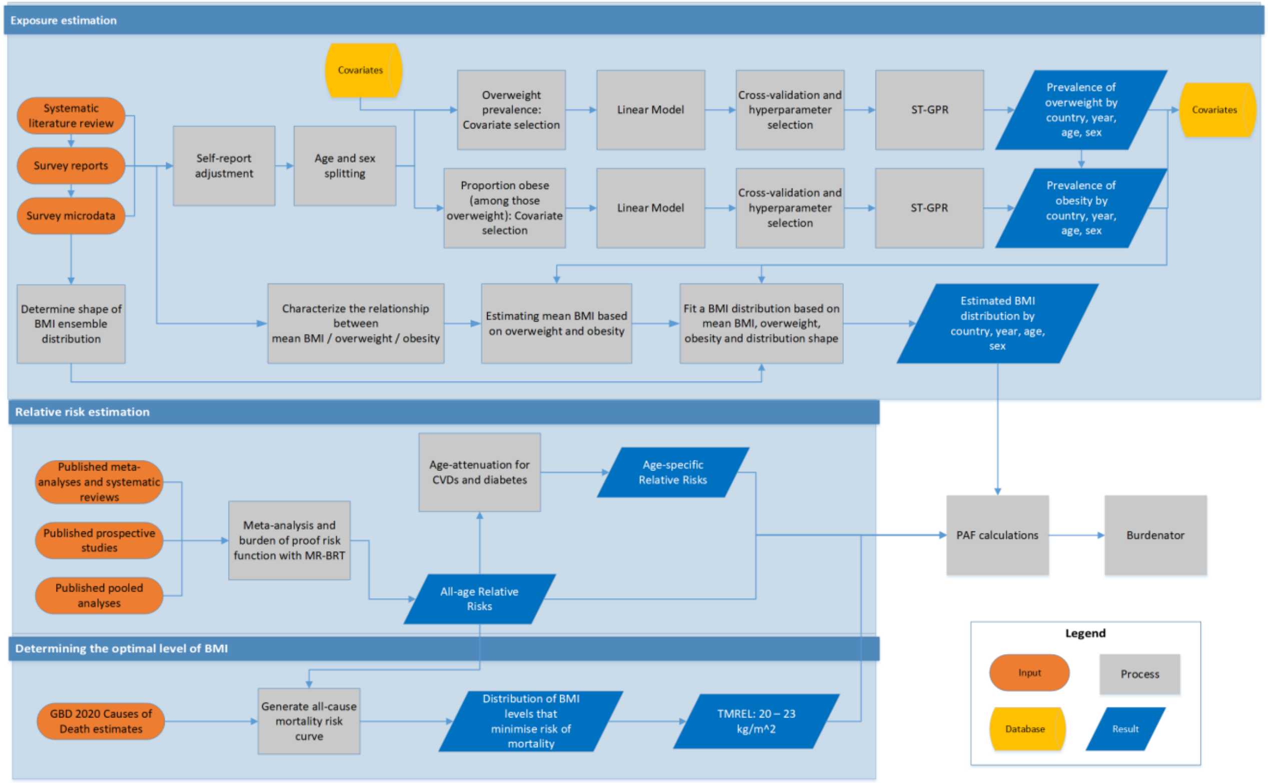

Childhood (Ages 2-19) High Body-Mass Index: Data and Model Flow Chart

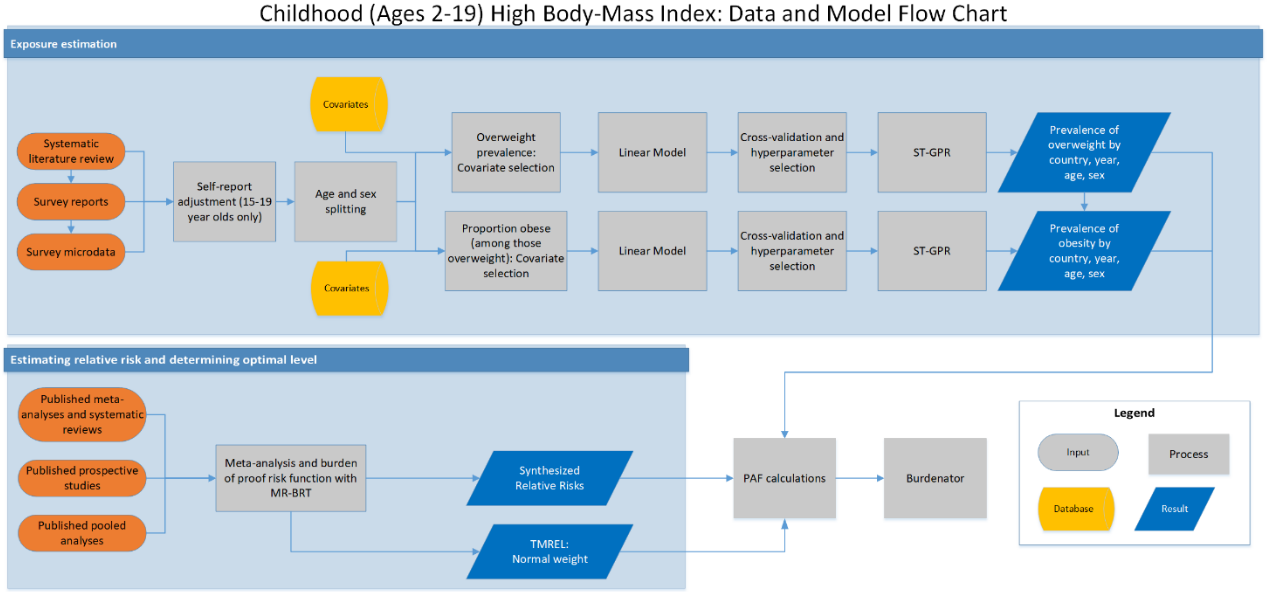

Input data and methodological summary

Case definitions

Exposure

High body-mass index (BMI) for adults (ages 20+) is defined as BMI greater than 20 to 23 kg/m^2. High BMI for children and adolescents (ages 2–19) is defined as being overweight or obese based on International Obesity Task Force standards.[1]

Input data

Exposure

In GBD 2021, new data were added from sources included in the annual GHDx update of known survey series. We conducted a systematic review in GBD 2017 to identify studies providing nationally or subnationally representative estimates of overweight prevalence, obesity prevalence, or mean body-mass index (BMI). We limited the search to literature published between January 1, 2016, and December 31, 2016, to update the systematic literature search previously performed as part of GBD 2015.

The search for adults was conducted on 4 January 2017, using the following terms:

((("Body Mass Index"[Mesh] OR "Overweight"[Mesh] OR "Obesity"[Mesh]) AND ("Geographic Locations"[Mesh] NOT "United States"[Mesh]) AND ("humans"[Mesh] AND "adult"[MeSH]) AND ("Data Collection"[Mesh] OR "Health Services Research"[Mesh] OR "Population Surveillance"[Mesh] OR "Vital statistics"[Mesh] OR "Population"[Mesh] OR "Epidemiology"[Mesh] OR "surve*"[TiAb]) NOT (Comment[ptyp] OR Case Reports[ptyp] OR "hospital"[TiAb])) AND ("2016/01/01"[Date - Publication] : "2016/12/31"[Date - Publication]))

The search for children was conducted on 4 August 2016, using the following terms:
((("Body Mass Index"[Mesh] OR "Overweight"[Mesh] OR "Obesity"[Mesh]) AND ("Geographic Locations"[Mesh] NOT "United States"[Mesh]) AND ("humans"[Mesh] AND "child"[MeSH]) AND ("Data Collection"[Mesh] OR "Health Services Research"[Mesh] OR "Population Surveillance"[Mesh] OR "Vital statistics"[Mesh] OR "Population"[Mesh] OR "Epidemiology"[Mesh] OR "surve*"[TiAb]) NOT (Comment[ptyp] OR Case Reports[ptyp] OR "hospital"[TiAb])) AND ("2016/01/01"[Date - Publication] : "2016/12/31"[Date - Publication]))

Table 1: Data inputs for exposure for high body-mass index.

Input data	Exposure
Source count (total)	2016
Number of countries with data	194

Eligibility criteria

We included representative studies providing data on mean BMI or prevalence of overweight or obesity among adults or children. For adults, studies were included if they defined overweight as BMI ≥25 kg/m^2 and obesity as BMI ≥30 kg/m^2, or if estimates using those cutoffs could be back-calculated from reported categories. For children (children ages 2–19), studies were included if they used International Obesity Task Force (IOTF) standards to define overweight and obesity thresholds.[1] We only included

studies reporting data collected after January 1, 1980. Studies were excluded if they used non-random samples (eg, case-control studies or convenience samples), conducted among specific subpopulations (eg, pregnant women, racial or ethnic minorities, immigrants, or individuals with specific diseases), used alternative methods to assess adiposity (eg, waist circumference, skin-fold thickness, or hydrodensitometry), had sample sizes of less than 20 per age-sex group, or provided inadequate information on any of the inclusion criteria. We also excluded review articles and non-English-language articles.

Data collection process

Where individual-level survey data were available, we computed mean BMI using weight and height. We then used BMI to determine the prevalence of overweight and obesity. For individuals aged over 19 years, we considered them to be overweight if their BMI was greater than or equal to 25 kg/m^2, and obese if their BMI was greater than or equal to 30 kg/m^2. For individuals aged 2 to 19 years, we used monthly IOTF cutoffs[2] to determine overweight and obese status when age in months was available. When only age in years was available, we used the cutoff for the midpoint of that year. Obese individuals were also considered to be overweight. We excluded studies using the World Health Organization (WHO) standards or country-specific cutoffs to define childhood overweight and obesity. At the individual level, we considered BMI <10 kg/m^2 and BMI >70 kg/m^2 to be biologically implausible and excluded those observations.

The rationale for choosing to use the IOTF cutoffs over the WHO standards has been described elsewhere.[1] Briefly, the IOTF cutoffs provide consistent child-specific standards for ages 2–18 derived from surveys covering multiple countries. By contrast, the WHO growth standards apply to children under age 5, and the WHO growth reference applies to children ages 5–19. The WHO growth reference for children ages 5–19 was derived from United States data, which are less representative than the multinational data used by IOTF. Additionally, the switch between references at age 5 can produce artificial discontinuities. Given that we estimate global childhood overweight and obesity for ages 2–19 (with age 19 using standard adult cutoffs), the IOTF cutoffs were preferable. Additionally, we found that IOTF cutoffs were more commonly used in scientific literature covering childhood obesity.

From report and literature data, we extracted data on mean BMI, prevalence of overweight, and prevalence of obesity, measures of uncertainty for each, and sample size, by the most granular age and sex groups available. Additionally, we extracted the same study-level covariates as were extracted from microdata (measurement, urbanicity, and representativeness), as well as location and year.

In addition to the primary indicators described above, we extracted relevant survey-design variables, including primary sampling unit, strata, and survey weights, which were used to tabulate individual-level microdata and produce accurate measures of uncertainty. We extracted three study-level covariates: 1) whether height and weight data were measured or self-reported; 2) whether the study was predominantly conducted in an urban area, rural area, or both; and 3) the level of representativeness of the study (national or subnational).

Finally, we extracted relevant demographic indicators, including location, year, age, and sex. We estimated the standard error of the mean from individual-level data, where available, and used the reported standard error of the mean for published data. When multiple data sources were available for the same country, we included all of them in our analysis. If data from the same data source were

available in multiple formats such as individual-level data and tabulated data, we used individual-level data.

Relative risk

In GBD 2021, we did not conduct an updated systematic review to identify new relative risk data sources. The last date of search in PubMed for evidence studying the health effects of high BMI on cardiovascular diseases and diabetes was 6 June 2019 using the following terms: ("Diabetes Mellitus"[Mesh] OR "diabetes"[title] OR "Stroke"[Mesh] OR "stroke[title]" OR "Heart Diseases"[Mesh] OR "Heart Diseases"[title] OR "Cardiovascular Diseases"[Mesh] OR "Cardiovascular Diseases"[title]) AND ("Obesity"[Mesh] OR "Obesity"[title] OR "Overweight"[Mesh] OR "Overweight"[title] OR "Body Mass Index"[Mesh] OR "Body Mass Index"[title]) AND ("cohort"[tiab]). For other risk-outcome pairs, we used existing meta-analyses and systematic reviews to identify and extract pooled cohorts and prospective studies for analysis.

Table 2: Data inputs for relative risks for high body-mass index.

Input data	Relative risk
Source count (total)	313
Number of countries with data	26

Data processing

Age and sex splitting

Any report or literature data provided in age groups wider than the standard five-year age groups or as both sexes combined were split using the approach used by Ng and colleagues.[2] *We first modelled age-sex patterns with spatiotemporal Gaussian process regression (ST-GPR) using data sources reporting in sex-specific, standard five-year age units. To account for the large heterogeneity in overweight and obesity prevalence across geographical regions, we categorised each location into three categories of overweight and obesity prevalence. We then aggregated the modelled age and sex patterns into tertiles of overweight and obesity prevalence. Finally, the aggregated patterns were applied to split report and literature data based on the data source's location and its respective tertile of overweight or obesity prevalence.* We did not propagate the uncertainty in the age pattern and sex pattern used to split the data as they seemed to have small effect.

Self-report bias adjustment

We included both measured and self-reported data. We tested for bias in self-report data compared to measured data, which is considered to be the gold standard. There was no clear direction of bias for children ages 2–14, so for these age groups we only included measured data. For individuals ages 15 and older, we *adjusted self-reported data for overweight prevalence and obesity prevalence. We used MR-BRT to determine the level of self-report bias adjustment. For both overweight and obesity, we fit sex-specific MR-BRT models on the logit difference between measured and self-reported with a fixed effect on super-region. The bias coefficients derived from these two models are in Table 1 and 2.*

A separate self-report bias adjustment was completed for the USA. Self-report data was compared to measured data from the NHANES survey series, which were selected as the gold standard for the USA. For individuals ages 2 and older, we *adjusted self-reported data for overweight prevalence and obesity*

prevalence. We used MR-BRT to determine the level of self-report bias adjustment. For both overweight and obesity, we fit sex-specific MR-BRT models on the logit difference between NHANES measured and self-reported with a fixed effect on 5-year age groups and decade when the data was collected. The bias coefficients derived from these two models are in Table 3.

Table 1: MR-BRT self-report crosswalk adjustment factors for overweight prevalence

Model	Data input	Reference or alternative case definition	Gamma	Beta coefficient, logit (95% CI)
Females	Measured data	Ref	0.26	---
	Self-reported data (southeast Asia, east Asia, and Oceania)	Alt		–0.53 (–1.03, –0.04)
	Self-reported data (central Europe, eastern Europe, and central Asia)	Alt		–0.20 (–0.69, 0.30)
	Self-reported data (high-income)	Alt		–0.25 (–0.75, 0.24)
	Self-reported data (Latin America and Caribbean)	Alt		–0.19 (–0.69, 0.31)
	Self-report data (north Africa and Middle East)	Alt		–0.38 (–0.89, 0.11)
	Self-report data (south Asia)	Alt		0.36 (–0.14, 0.85)
	Self-report data (sub-Saharan Africa)	Alt		–0.26 (–0.76, 0.24)
Males	Measured data	Ref	0.43	---
	Self-reported data (southeast Asia, east Asia, and Oceania)	Alt		–0.36 (–1.17, 0.50)
	Self-reported data (central Europe, eastern Europe, and central Asia)	Alt		–0.03 (–0.84, 0.82)
	Self-reported data (high-income)	Alt		0.05 (–0.77, 0.87)
	Self-reported data (Latin America and Caribbean)	Alt		–0.02 (–0.84, 0.81)
	Self-report data (north Africa and Middle East)	Alt		–0.21 (–1.04, 0.61)
	Self-report data (south Asia)	Alt		0.53 (–0.28, 1.37)
	Self-report data (sub-Saharan Africa)	Alt		–0.27 (–1.09, 0.55)

Table 2: MR-BRT self-report crosswalk adjustment factors for obesity prevalence

Model	Data input	Reference or alternative case definition	Gamma	Beta coefficient, logit (95% UI) *
Females	Measured data	Ref	0.38	---

	Self-reported data (southeast Asia, east Asia, and Oceania)	Alt		–0.11 (–0.86, 0.64)
	Self-reported data (central Europe, eastern Europe, and central Asia)	Alt		–0.95 (–1.70, –0.19)
	Self-reported data (high-income)	Alt		–0.42 (–1.16, 0.34)
	Self-reported data (Latin America and Caribbean)	Alt		–0.41 (–1.16, 0.34)
	Self-report data (north Africa and Middle East)	Alt		–0.48 (–1.23, 0.27)
	Self-report data (south Asia)	Alt		0.50 (–0.25, 1.26)
	Self-report data (sub-Saharan Africa)	Alt		–0.41 (–1.16, 0.34)
Males	Measured data	Ref	0.74	
	Self-reported data (southeast Asia, east Asia, and Oceania)	Alt		0.04 (–1.41, 1.53)
	Self-reported data (central Europe, eastern Europe, and central Asia)	Alt		–0.79 (–2.25, 0.71)
	Self-reported data (high-income)	Alt		–0.13 (–1.58, 1.40)
	Self-reported data (Latin America and Caribbean)	Alt		–0.26 (–1.70, 1.21)
	Self-report data (north Africa and Middle East)	Alt		–0.33 (–1.77, 1.16)
	Self-report data (south Asia)	Alt		0.66 (–0.78, 2.15)
	Self-report data (sub-Saharan Africa)	Alt		–0.41 (–1.86, 1.08)

**MR-BRT crosswalk adjustments can be interpreted as the factor the alternative case definition is adjusted by to reflect what it would have been had it been measured using the reference case definition. If the log/logit beta coefficient is negative, then the alternative is adjusted up to the reference. If the log/logit beta coefficient is positive, then the alternative is adjusted down to the reference.*

Table 3: MR-BRT self-report crosswalk adjustment factors for USA overweight and obesity prevalence

**Model**	**Data input**	**Reference or alternative case definition**	**Gamma**	**Beta coefficient, logit (95% UI) ***
**Overweight prevalence**				
Females	Measured NHANES data	Ref	0.0052	---
	Self-report (intercept)	Alt		0.08 (0.05, 0.11)
	Self-report (5-year age group)	Alt		–0.02 (–0.02, -0.01)
	Self-report data (decade)	Alt		0 (–0.02, 0.02)
Males	Measured NHANES data	Ref	0.016	---
	Self-report (intercept)	Alt		-0.42 (-0.46, -0.37)
	Self-report (5-year age group)	Alt		-0.003 (–0.005, -0.001)
	Self-report data (decade)	Alt		0 (–0.03, 0.03)
**Obesity prevalence**				
Females	Measured NHANES data	Ref	0.012	---
	Self-report (intercept)	Alt		-0.45 (-0.49, -0.41)

	Self-report (5-year age group)	Alt		0.003 (0.001, 0.004)
	Self-report data (decade)	Alt		0.01 (-0.02, 0.04)
Males	Measured NHANES data	Ref	0.018	---
	Self-report (intercept)	Alt		-0.46 (-0.50, -0.41)
	Self-report (5-year age group)	Alt		0 (–0.002, 0.001)
	Self-report data (decade)	Alt		0.01(–0.02, 0.04)

**MR-BRT crosswalk adjustments can be interpreted as the factor the alternative case definition is adjusted by to reflect what it would have been had it been measured using the reference case definition. If the log/logit beta coefficient is negative, then the alternative is adjusted up to the reference. If the log/logit beta coefficient is positive, then the alternative is adjusted down to the reference.*

Modelling strategy

Exposure

Prevalence estimation for overweight and obesity

After adjusting for self-report bias and splitting aggregated data into five-year age-sex groups, we used ST-GPR to estimate the prevalence of overweight and obesity. This modelling approach has been described in detail elsewhere.

The linear model, which when added to the smoothed residuals forms the mean prior for GPR is as follows:

$$\text{logit}(\text{overweight})_{c,a,t} = \beta_0 + \beta_1 \text{educ}_{c,t} + \beta_2 \text{urban}_{c,t} + \beta_3 \text{agriculture}_{c,t} + \sum_{k=1}^{16} \beta_k I_{A[a]} + \alpha_s + \alpha_r + \alpha_c$$

$$\text{logit}(\text{obesity/overweight})_{c,a,t} = \beta_0 + \beta_1 \text{educ}_{c,t} + \beta_2 \text{urban}_{c,t} + \beta_3 \text{agriculture}_{c,t} + \sum_{k=1}^{16} \beta_k I_{A[a]} + \alpha_s + \alpha_r + \alpha_c$$

where educ *is the age-standardised level of educational attainment;* urban *is the proportion of the population living in an urban area ; and* agriculture *is the proportion of the population working in agriculture.* $I_{A[a]}$ is a dummy variable indicating specific age group A that the prevalence point captures, and α_s, α_r, and α_c are super-region, region, and country nested random intercepts, respectively. Random effects were used in model fitting but were not used in prediction.

We tested all combinations of the following covariates to see which performed best in terms of in-sample AIC for the overweight linear model and the obesity as a proportion of overweight linear model: ten-year lag-distributed energy per capita, proportion of the population living in urban areas, SDI, lag-distributed income per capita, educational attainment (years) per capita, proportion of the population working in agriculture, grams of sugar adjusted for energy per capita, grams of sugar not adjusted for energy per capita, and the number of two- or four-wheeled vehicles per capita. We selected these candidate covariates based on theory as well as reviewing covariates used in other publications. The final linear model was selected based on 1) if the direction of covariates matched what is expected from theory, 2) all the included covariates were significant, and 3) minimising in-sample AIC. The covariate selection process was performed using the dredge package in R.

Estimating mean BMI

To estimate the mean BMI for adults in each country, age, sex, and time period 1980–2021, we first used the following nested hierarchical mixed-effects model, fit using restricted maximum likelihood on

data from sources containing estimates of all three indicators (prevalence of overweight, prevalence of obesity, and mean BMI), in order to characterise the relationship between overweight, obesity, and mean BMI:

$$\log(\mathrm{BMI}_{c,a,s,t}) = \beta_0 + \beta_1 \mathrm{ow}_{c,a,s,t} + \beta_2 \mathrm{ob}_{c,a,s,t} + \beta_3 \mathrm{sex} + \sum_{k=1}^{12} \beta_k I_{A[a]} + \alpha_s(1 + \mathrm{ow}_{c,a,s,t} + \mathrm{ob}_{c,a,s,t}) + \alpha_r(1 + \mathrm{ow}_{c,a,s,t} + \mathrm{ob}_{c,a,s,t}) + \alpha_c(1 + \mathrm{ow}_{c,a,s,t} + \mathrm{ob}_{c,a,s,t}) + \epsilon_{c,a,s,t}$$

where $\mathrm{ow}_{c,a,s,t}$ *is the prevalence of overweight in country c, age a, sex s, and year t,* $\mathrm{ob}_{c,a,s,t}$ *is the prevalence of obesity in country c, age a, sex s, and year t,* sex *is a fixed effect on sex,* $I_{A[a]}$ *is an indicator variable for age, and* α_s, α_r, *and* α_c *are random effects at the super-region, region, and country level, respectively. The model was run in Stata 13.*

We applied 1000 draws of the regression coefficients to the 1000 draws of overweight prevalence and obesity prevalence produced through ST-GPR to estimate 1000 draws of mean BMI for each country, year, age, and sex. This approach ensured that overweight prevalence, obesity prevalence, and mean BMI were correlated at the draw level and uncertainty was propagated.

Estimating BMI distribution

We used the ensemble distribution approach described in the manuscript. We fit ensemble weights by source and sex, with source- and sex-specific weights averaged across all sources included to produce the final global weights. The ensemble weights were fit on measured microdata. The final ensemble weights were exponential = 0.002, gamma = 0.028, inverse gamma = 0.085, log-logistic = 0.187, Gumbel = 0.220, Weibull = 0.011, log-normal = 0.058, normal = 0.012, beta = 0.136, mirror gamma = 0.008, and mirror Gumbel = 0.113.

1000 draws of BMI distributions for each location, year, age group, and sex estimated were produced by fitting an ensemble distribution using 1000 draws of estimated mean BMI, 1000 draws of estimated standard deviation, and the ensemble weights. Estimated standard deviation was produced by optimising a standard deviation to fit estimated overweight prevalence draws and estimated obesity prevalence draws.

Relative risk

In previous rounds of GBD, we reported the relative risk per five-unit change in BMI for disease endpoints using meta-analyses, and where available, pooled analyses of prospective observational studies. In GBD 2021, we assessed risk–outcome pairs included in previous rounds of the GBD based on the available evidence supporting a causal effect. We used MR-BRT to estimate the non-linear dose–response relationships between high BMI and risk for 26 disease endpoints. Specifically, we used the evidence score framework to systematically determine the risk function and evaluate the strength of evidence for each risk–outcome pair. Further details on the evidence score framework are available in the general methods of the Appendix.

The shape of dose–response relationships between BMI and risk for diseases has been well defined.[3,4] To best account for the various shapes (eg, J-shaped, increasing, and decreasing) of these relationships, we used the MR-BRT tool to estimate the log relative risk associated with each level of BMI on a continuous scale. Outcome-specific model characteristics are described in Table 4.

For each risk–outcome pair meta-regression, we considered study-level covariates that could potentially bias the study's reported effect size estimates. These study-level covariates included indication of whether the study used a washout period, whether the study population was randomly sampled from the general population, whether the study measured or asked participants to self-report baseline BMI levels, whether the study determined outcomes based on administrative records or self-reports, and the level of adjustment for relevant confounders like age, sex, smoking, education, and income. We adjusted for these covariates in our meta-regression if they significantly biased our estimated relative risk function.

We implemented the Fisher scoring correction to the heterogeneity parameter, which corrects for data-sparse situations. In such cases, the between-study heterogeneity parameter estimate may be 0, simply from lack of data. The Fisher scoring correction uses a quantile of gamma, which is sensitive to the number of studies, study design, and reported uncertainty.

We also added methodology to detect and flag publication bias. The approach is based on the classic Egger's regression strategy, which is applied to the residuals in our model. In the current implementation, we do not correct for publication bias, but flag the risk–outcome pairs where the risk for publication bias is significant. We found no evidence of publication bias for the outcomes associated with high body-mass index.

There is a well-documented attenuation of the risk for cardiovascular disease and diabetes due to metabolic risks factors throughout one's life.[5] To incorporate this age trend in the relative risks, we first identified the median age-at-event across all cohorts and considered that as the reference age group. We then assigned our risk curves to this reference age group. Then, we derived attenuation factors by taking the ratio of excess risk between each age group and the reference. Finally, we applied 1000 draws of the age-specific attenuation factors to 1000 draws of the reference age group's risk curve to determine age-specific risk curves that propagated the uncertainty of both the risk function and age pattern.

For children and adolescent outcomes (ages 2–19), we computed dichotomous relative risks for overweight and obesity by modelling the log difference in relative risk between alternative groups (ie, overweight or obese) and reference groups (ie, normal weight) from prospective cohort studies.

Table 4: Model characteristics for outcomes related to high body-mass index in adults

Outcome	Non-linear specifications and constraints	Selected covariates	Mean gamma solution	Publication bias
Alzheimer's disease and other dementias	*	Reverse causality; representative population	0.332	No
Asthma	*		0.020	No
Atrial fibrillation and flutter	*		0.016	No

Breast cancer (in premenopausal women)	*		0.000	No
Breast cancer (in postmenopausal women)	*	Representative population	0.110	No
Cataract	**		0.157	No
Colon and rectum cancer	*		0.000	No
Diabetes mellitus type 2	*	Objective exposure measurement; objective outcome ascertainment	0.087	No
Gallbladder and biliary diseases	*	Objective outcome ascertainment; level of adjusted confounders	0.049	No
Gallbladder and biliary tract cancer	*		0.000	No
Gout	*		0.000	No
Intracerebral haemorrhage and Subarachnoid haemorrhage	*	Objective exposure measurement	0.118	No
Ischaemic heart disease	*	Objective exposure measurement	0.106	No
Ischaemic stroke	*		0.458	No
Kidney cancer	*		0.036	No
Leukaemia	*		0.000	No
Liver cancer	*		0.032	No
Low back pain	*		0.000	No
Multiple myeloma	**		0.000	No
Non-Hodgkin lymphoma	*		0.058	No
Osteoarthritis	*		0.045	No
Ovarian cancer	*		0.000	No
Pancreatic cancer	*		0.019	No
Thyroid cancer	*		0.000	No
Uterine cancer	*		0.008	No

** Cubic splines with 5 knots; left and right linear tails; Gaussian prior (0, 0.01) on max derivative of non-linear intervals.*

*** Cubic splines with 5 knots; left and right linear tails; Gaussian prior (0, 0.01) on max derivative of non-linear intervals.*

Theoretical minimum risk exposure level

For adults (ages 20+), the theoretical minimum risk exposure level (TMREL) of BMI (20–23 kg/m^2) was determined based on the BMI level that was associated with the lowest risk of all-cause mortality. Briefly, after estimating all-age, cause-specific dose–response risk curves, we generated 1000 draws of an all-cause mortality risk curve by taking weighted averages of 1000 draws of cause-specific risk curves. The weights were determined from the number of cause-specific global deaths from the GBD 2021 Causes of Death analysis. By generating the all-cause risk curve at the draw level, we were able to determine a distribution of the BMI levels that minimised all-cause mortality by assessing the level of BMI that minimised the risk for each of the 1000 draws.

For children and adolescents (ages 2–19), the TMREL is "normal weight," that is, not overweight or obese, based on IOTF cutoffs.[1]

References

1.) Cole TJ, Lobstein T. Extended international (IOTF) body mass index cut-offs for thinness, overweight and obesity. *Pediatr Obes* 2012;**7**(4):284—94.
2.) Ng M, Fleming T, Robinson M, et al. Global, regional, and national prevalence of overweight and obesity in children and adults during 1980–2013: a systematic analysis for the Global Burden of Disease Study 2013. *Lancet* 2014; **384**: 766–81.
3.) Angelantonio ED, Bhupathiraju SN, Wormser D, et al. Body-mass index and all-cause mortality: individual-participant-data meta-analysis of 239 prospective studies in four continents. *Lancet* 2016; **388**: 776—86. doi: 10.1016/S0140-6736(16)30175-1.
4.) Bhaskaran K, Dos-Santos-Silva I, Leon DA, Douglas IJ, Smeeth L. Association of BMI with overall and cause-specific mortality: a population-based cohort study of 3·6 million adults in the UK. *Lancet Diabetes Endocrinol* 2018; **6**(12): 944—53.
5.) Singh GM, Danaei G, Farzadfar F, et al. The age-specific quantitative effects of metabolic risk factors on cardiovascular diseases and diabetes: a pooled analysis. *PLoS One* 2013; **8**(7): e65174.

High fasting plasma glucose

Flowchart

Figure 1: *Calculating high fasting plasma glucose attributable burden*

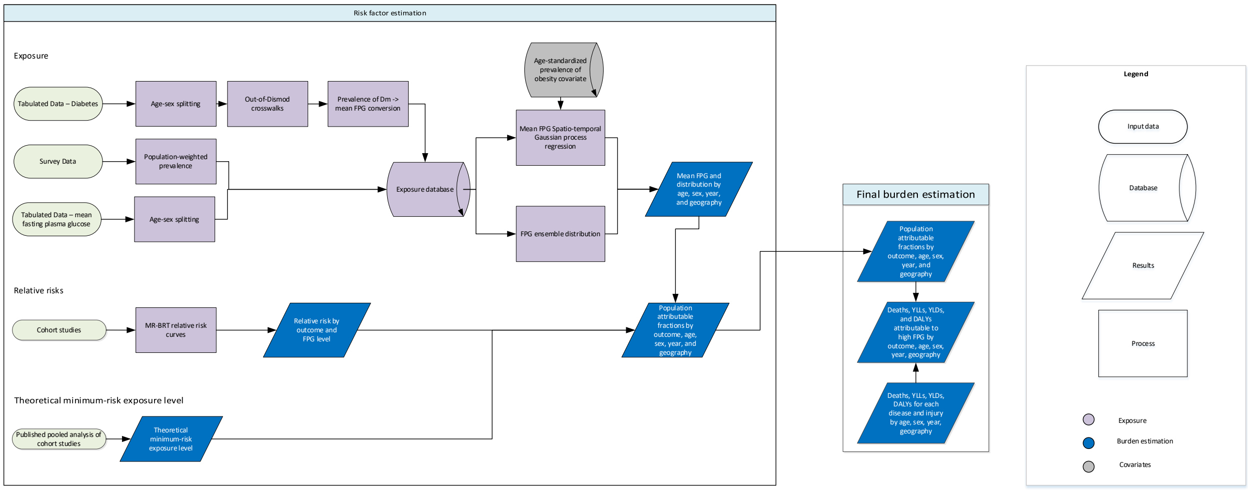

Input data and methodological summary

Definition

Exposure

High fasting plasma glucose (FPG) is measured as the mean FPG in a population, where FPG is a continuous exposure in units of mmol/L. Since FPG is along a continuum, we define high FPG as any level above the theoretical minimum-risk exposure level (TMREL), which is 4.9–5.3 mmol/L.

Data seeking

Exposure

Collaborator-provided sources that were either shared directly with us or were identified through searching the Global Health Data Exchange (GHDx) were reviewed for inclusion.

- 139 new sources were included in the FPG exposure model for GBD 2021.

No systematic review was conducted for the FPG exposure model for GBD 2021; the most recent systematic review was conducted for GBD 2019. In place of a systematic review, an "audit" of the current data in the FPG model was undertaken. The audit process involved returning to each data source to re-evaluate inclusion into the model, and to re-check data extractions for those sources that remain eligible for inclusion. Both GBD 2019 sources and the 139 new GBD 2021 sources were included in the audit.

Figure 2: *Diagram of data sources in the GBD 2021 FPG exposure model*

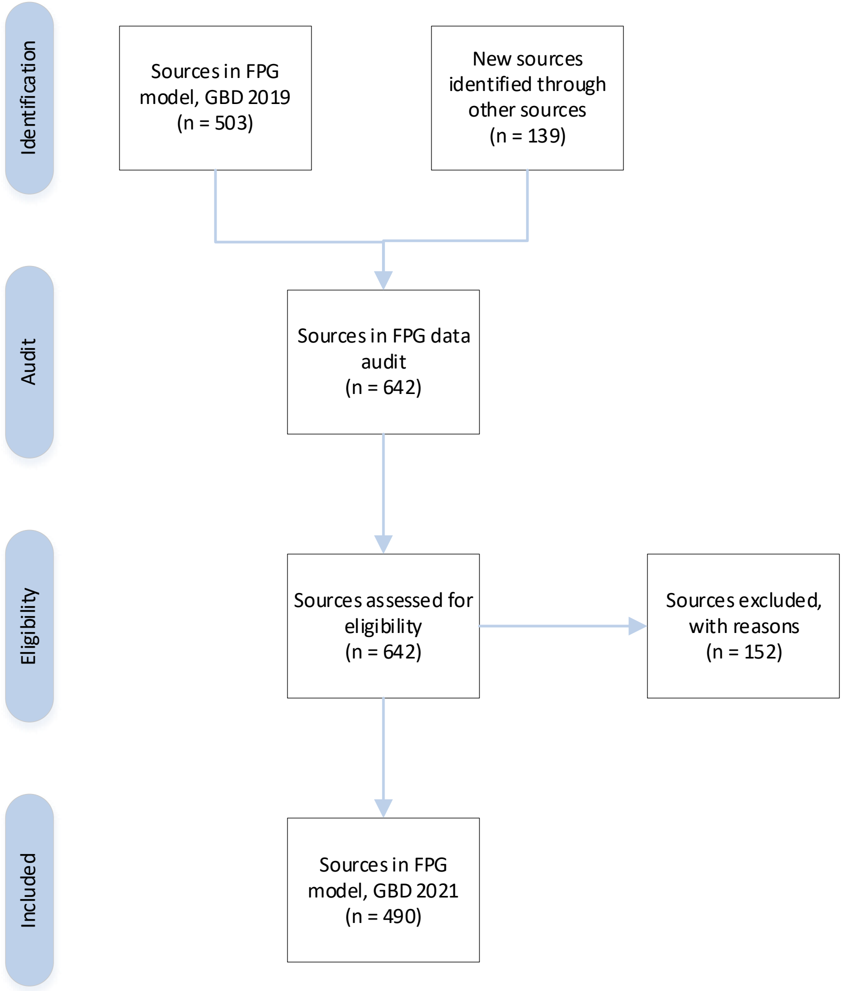

Common exclusion reasons include duplicative studies and not population representative.

Relative risk

For each outcome, our goal was to extract the estimates from the original cohort study. To accomplish this, we re-reviewed the relative risk studies used in previous GBD rounds and looked for the original studies used in any meta-analysis. In the event that a study in the meta-analysis was a pooled analysis or we found a study in our PubMed search was a pooled study, we made an effort to document the cohorts used and attempted to identify different studies that reported estimates for each individual

cohort. The search strings were grouped based on topics due to reports of multiple outcomes within the same study. Below are diagrams for the review of studies used in past rounds of GBD as well as the studies found in the review of sources.

Due to the number of relative risk studies we found and amount of time and personnel resources, we prioritised our search for additional studies. First, we looked for original articles used in the studies we accepted in previous GBD rounds. Second, we looked for original articles used in the additional meta-analysis studies we identified. Finally, we reviewed as many sources from the search string as we were able to within a 4-month time period. Below are the results for each effort.

Outcomes: ischaemic stroke, ischaemic heart disease, intracerebral haemorrhage

- Search string

(((((diabetes[MeSH Terms] OR diabetes[Title/Abstract] OR hyperglycemia[MeSH Terms] OR hyperglycemia[Title/Abstract] OR blood glucose[MeSH Terms] OR blood glucose[Title])) AND (Case-Control Studies[MeSH Terms] OR Cross-Over Studies[MeSH Terms] OR Cohort Studies[MeSH Terms] OR Systematic Review[Publication Type] OR Meta-Analysis[Publication Type] OR systematic review[Title/Abstract] OR meta-analysis[Title/Abstract] OR cohort[Title/Abstract] OR cross-over[Title/Abstract] OR crossover[Title/Abstract] OR case-control[Title/Abstract] OR prospective[Title/Abstract] OR retrospective[Title/Abstract] OR longitudinal[Title/Abstract] OR follow-up[Title/Abstract] OR Dose-Response Relationship, Drug[MeSH Terms] OR dose-response[Title/Abstract]) AND (Risk[MeSH Terms] OR Odds Ratio[MeSH Terms] OR risk[Title/Abstract] OR odds ratio[Title/Abstract] OR cross-product ratio[Title/Abstract] OR hazards ratio[Title/Abstract] OR hazard ratio[Title/Abstract])) AND ((1970/01/01[PDat] : 2019/12/31[PDat]) NOT (animals[MeSH Terms] NOT Humans[MeSH Terms])))) AND (intracranial hemorrhage[MeSH Terms] OR stroke[MeSH Terms] OR brain infarction[MeSH Terms] OR cerebral infarction[MeSH Terms] OR intracerebral hemorrhage[MeSH Terms] OR subarachnoid hemorrhage[MeSH Terms] OR cerebrovascular stroke[MeSH Terms] OR heart disease[MeSH Terms] OR peripheral artery disease[MeSH Terms] OR intracranial hemorrhage[Title/Abstract] OR stroke[Title/Abstract] OR brain infarction[Title/Abstract] OR cerebral infarction[Title/Abstract] OR intracerebral hemorrhage[Title/Abstract] OR subarachnoid hemorrhage[Title/Abstract] OR cerebrovascular stroke[Title/Abstract] OR heart disease[Title/Abstract] OR peripheral artery disease[Title/Abstract])

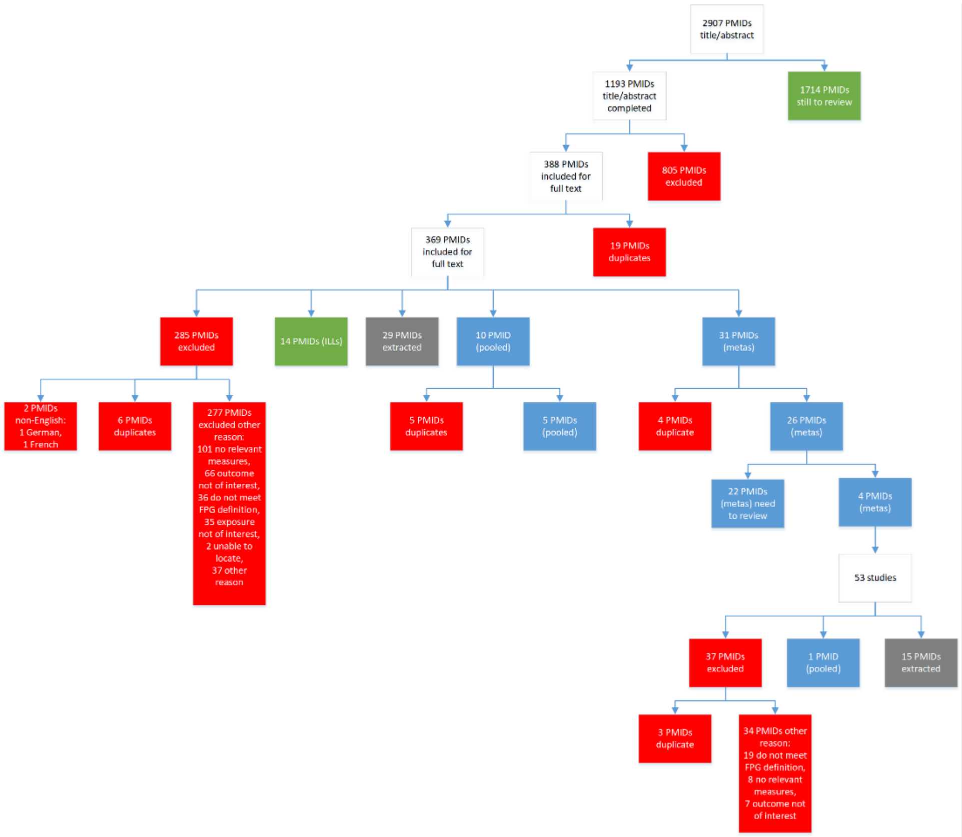

- Previously accepted studies

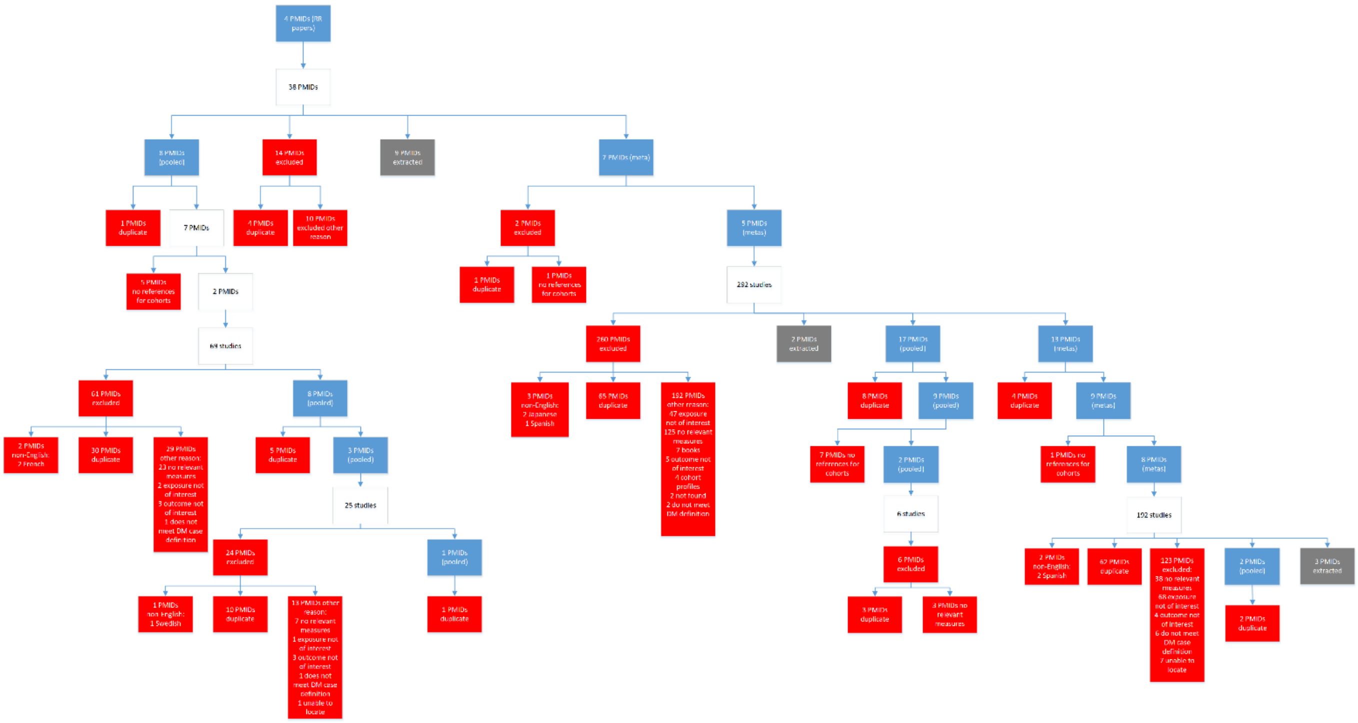

- Prospectively found additional meta-analyses

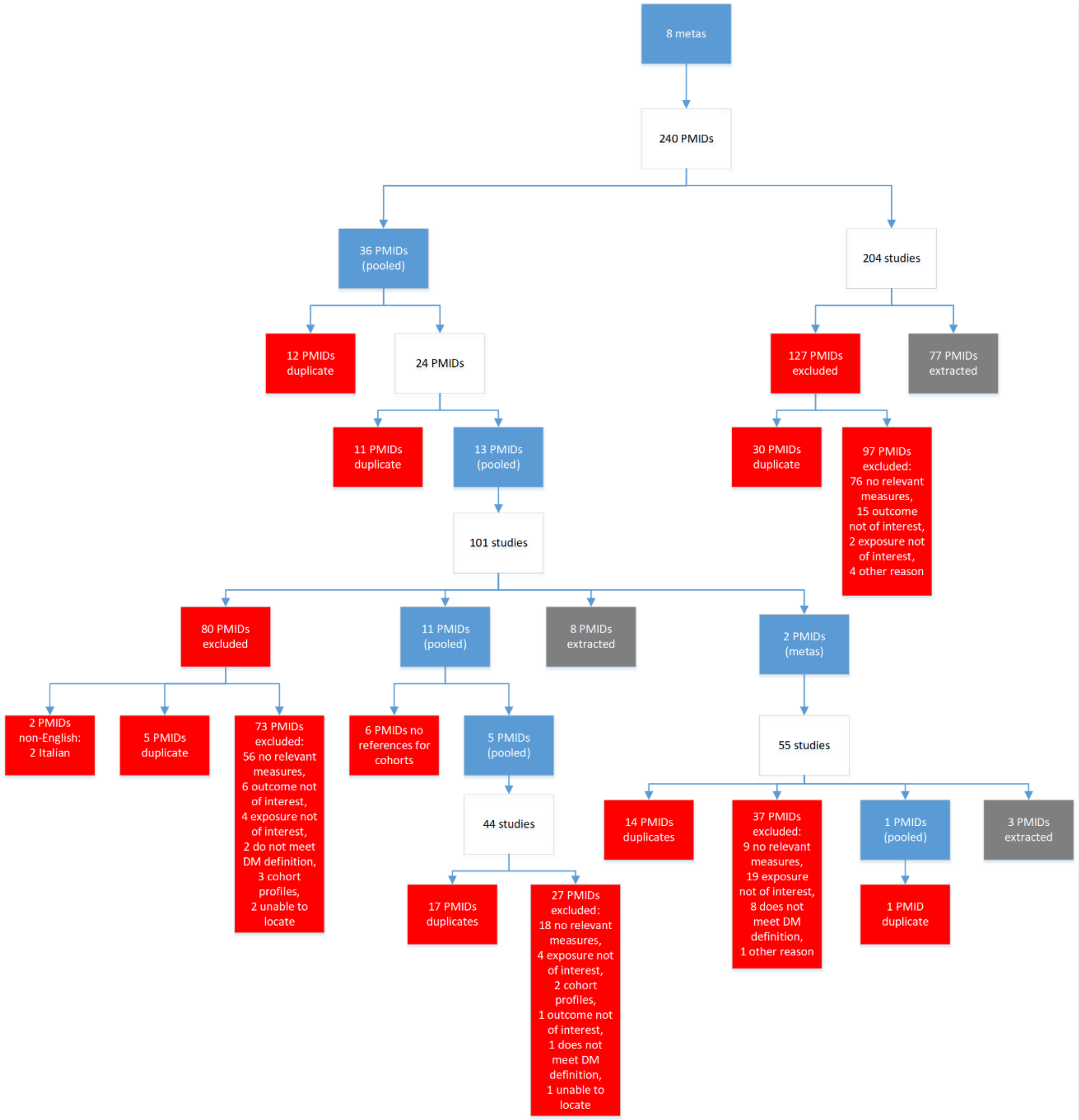

An additional effort for the ischaemic stroke, ischaemic heart disease, and intracerebral haemorrhage outcomes was undertaken in 2022 to re-review the relative risk data. Major updates included excluding data for composite cardiovascular outcomes that were previously included in the ischaemic heart disease model, and excluding data for unspecified stroke that were previously included in the ischaemic stroke model. An opportunistic search of PubMed was also undertaken to add recently published cohort studies (post-2010) and data reporting exposure using glycated hemoglobin (HbA1c) or oral glucose tolerance tests (OGTT). HbA1c (%) and OGTT (mmol/L) exposure levels were converted to FPG (mmol/L) equivalent exposure levels using the conversion values of 7/6.5 and 7/11.1, respectively. Below are the final source and data point counts:

- Ischaemic stroke: 26 sources, 79 data points
- Ischaemic heart disease: 49 sources, 144 data points
- Intracerebral haemorrhage: 9 sources, 28 data points

As a result of this additional effort, subarachnoid haemorrhage was dropped as an outcome of high FPG for GBD 2021, due to an implausible biological mechanism for this risk-outcome pair and a lack of data informing this relationship (only 1 data point specific to subarachnoid haemorrhage was found acceptable for inclusion).

Outcome: chronic kidney disease

- Previously accepted studies

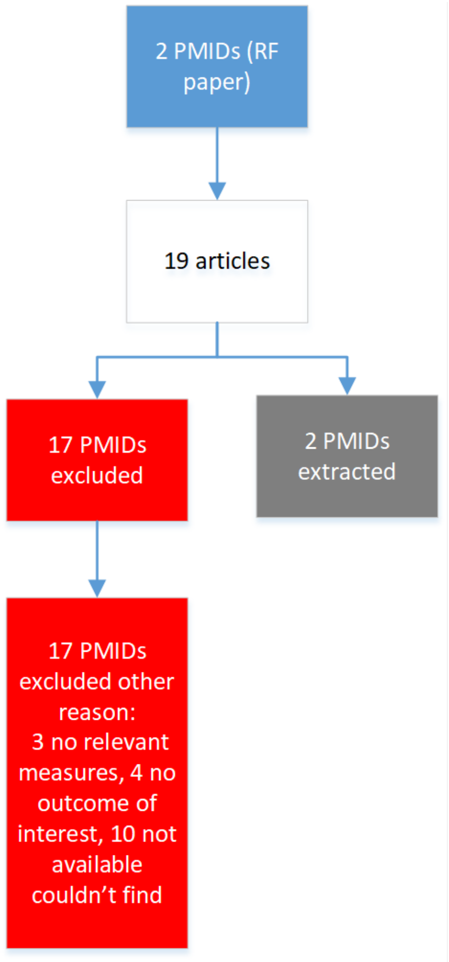

- Search string

((((((chronic kidney failure[MeSH Terms]) OR chronic kidney disease[Title/Abstract]) OR glomerulonephritis[MeSH Terms]) OR glomerulonephritis[Title/Abstract]) OR chronic kidney failure[Title/Abstract])) AND (((((diabetes[MeSH Terms]) OR diabetes[Title/Abstract]) OR hyperglycemia[MeSH Terms]) OR hyperglycemia[Title/Abstract]) OR blood glucose[MeSH Terms]) OR blood glucose[Title])) AND (((((((((diabetes[MeSH Terms]) OR diabetes[Title/Abstract]) OR hyperglycemia[MeSH Terms]) OR hyperglycemia[Title/Abstract]) OR blood glucose[MeSH Terms]) OR

blood glucose[Title])) AND ((Case-Control Studies[MeSH Terms] OR Cross-Over Studies[MeSH Terms] OR Cohort Studies[MeSH Terms] OR Systematic Review[Publication Type] OR Meta-Analysis[Publication Type] OR systematic review[Title/Abstract] OR meta-analysis[Title/Abstract] OR cohort[Title/Abstract] OR cross-over[Title/Abstract] OR crossover[Title/Abstract] OR case-control[Title/Abstract] OR prospective[Title/Abstract] OR retrospective[Title/Abstract] OR longitudinal[Title/Abstract] OR follow-up[Title/Abstract] OR Dose-Response Relationship, Drug[MeSH Terms] OR dose-response[Title/Abstract]) AND (Risk[MeSH Terms] OR Odds Ratio[MeSH Terms] OR risk[Title/Abstract] OR odds ratio[Title/Abstract] OR cross-product ratio[Title/Abstract] OR hazards ratio[Title/Abstract] OR hazard ratio[Title/Abstract]) AND (1970/01/01[PDat] : 2019/12/31[PDat]) NOT (animals[MeSH Terms] NOT Humans[MeSH Terms])))

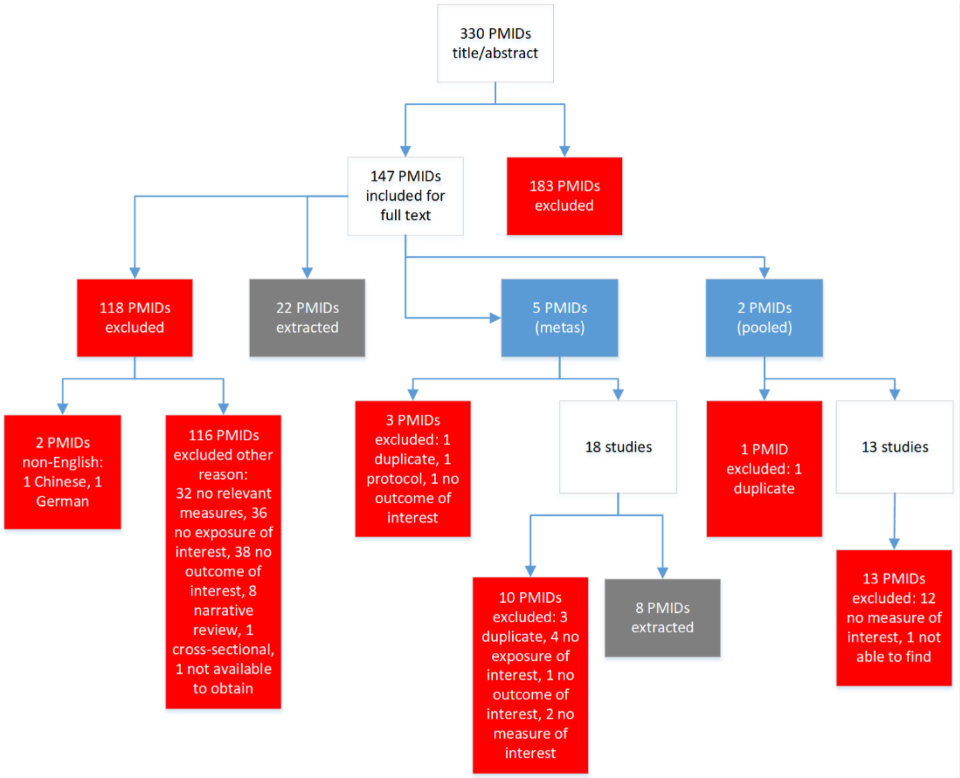

Outcome: tuberculosis

- Previously accepted studies

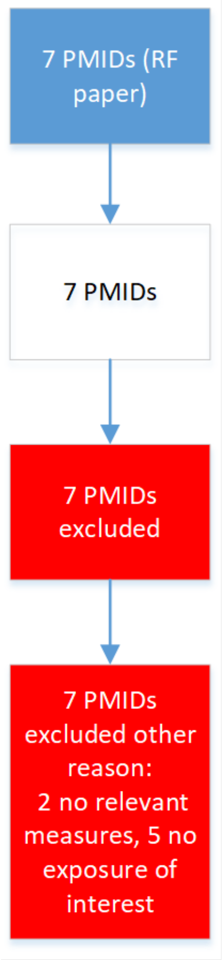

- Search string

((((tuberculosis[MeSH Terms]) OR tuberculosis[Title/Abstract])) AND (((((((Case-Control Studies[MeSH Terms] OR Cross-Over Studies[MeSH Terms] OR Cohort Studies[MeSH Terms] OR Systematic Review[Publication Type] OR Meta-Analysis[Publication Type] OR systematic review[Title/Abstract] OR meta-analysis[Title/Abstract] OR cohort[Title/Abstract] OR cross-over[Title/Abstract] OR crossover[Title/Abstract] OR case-control[Title/Abstract] OR prospective[Title/Abstract] OR retrospective[Title/Abstract] OR longitudinal[Title/Abstract] OR follow-up[Title/Abstract] OR Dose-Response Relationship, Drug[MeSH Terms] OR dose-response[Title/Abstract]) AND (Risk[MeSH Terms] OR Odds Ratio[MeSH Terms] OR risk[Title/Abstract] OR odds ratio[Title/Abstract] OR cross-product ratio[Title/Abstract] OR hazards ratio[Title/Abstract] OR hazard ratio[Title/Abstract]) AND (1970/01/01[PDat] : 2019/12/31[PDat]) NOT (animals[MeSH Terms] NOT Humans[MeSH Terms]))))))) AND ((((((diabetes[MeSH Terms]) OR diabetes[Title/Abstract]) OR hyperglycemia[MeSH Terms]) OR hyperglycemia[Title/Abstract]) OR blood glucose[MeSH Terms]) OR blood glucose[Title])

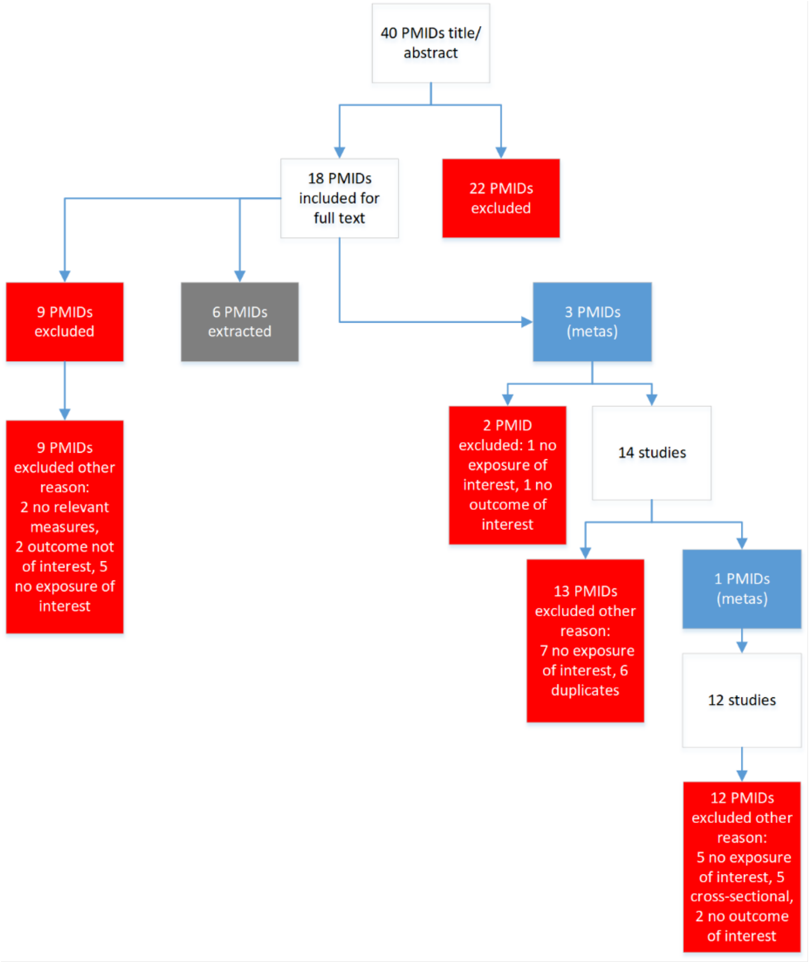

Outcome: Alzheimer's disease and other dementias

- Search string

(((((diabetes[MeSH Terms] OR diabetes[Title/Abstract] OR hyperglycemia[MeSH Terms] OR hyperglycemia[Title/Abstract] OR blood glucose[MeSH Terms] OR blood glucose[Title])) AND (Case-Control Studies[MeSH Terms] OR Cross-Over Studies[MeSH Terms] OR Cohort Studies[MeSH Terms] OR Systematic Review[Publication Type] OR Meta-Analysis[Publication Type] OR systematic review[Title/Abstract] OR meta-analysis[Title/Abstract] OR cohort[Title/Abstract] OR cross-over[Title/Abstract] OR crossover[Title/Abstract] OR case-control[Title/Abstract] OR prospective[Title/Abstract] OR retrospective[Title/Abstract] OR longitudinal[Title/Abstract] OR follow-up[Title/Abstract] OR Dose-Response Relationship, Drug[MeSH Terms] OR dose-response[Title/Abstract]) AND (Risk[MeSH Terms] OR Odds Ratio[MeSH Terms] OR risk[Title/Abstract] OR odds ratio[Title/Abstract] OR cross-product ratio[Title/Abstract] OR hazards ratio[Title/Abstract] OR hazard ratio[Title/Abstract])) AND ((1970/01/01[PDat] : 2019/12/31[PDat]) NOT (animals[MeSH Terms] NOT Humans[MeSH Terms])))) AND (alzheimer disease[MeSH Terms] OR dementia[MeSH Terms] OR alzheimer[Title/Abstract] OR dementia[Title/Abstract])

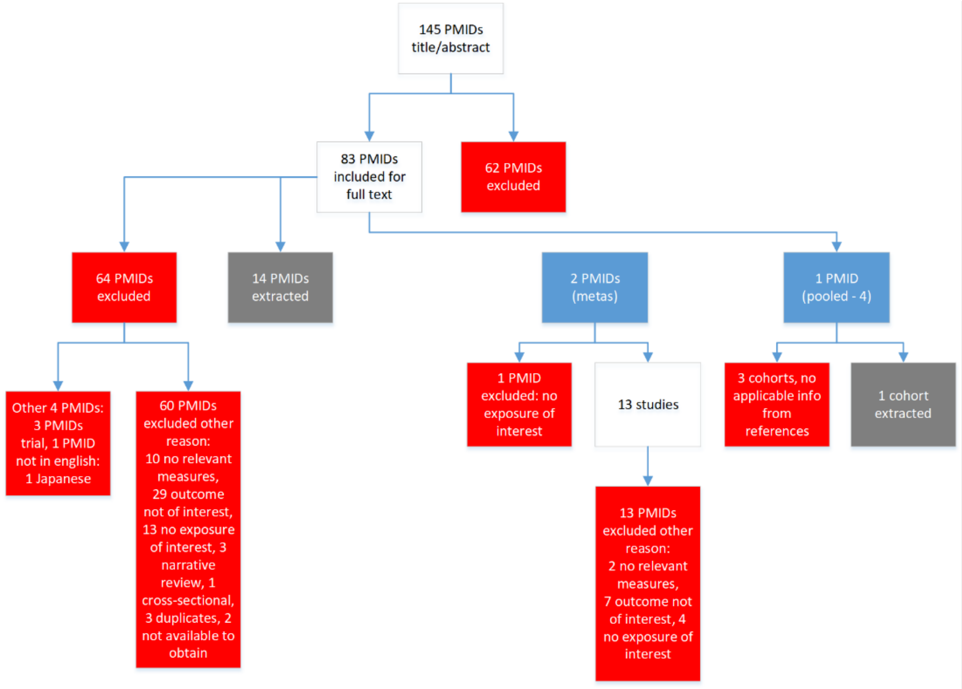

- Previously accepted studies

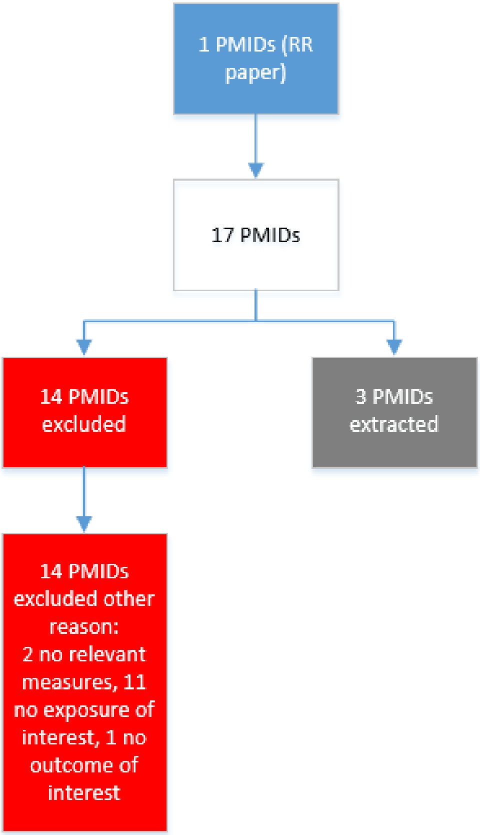

Outcomes: glaucoma, cataracts

- Previously accepted studies

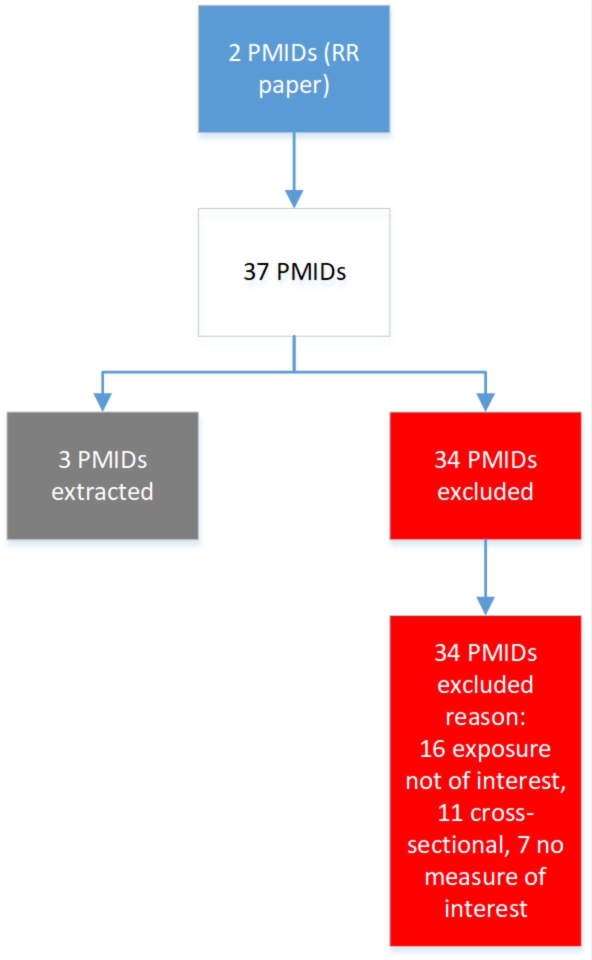

- Search string

(((((diabetes[MeSH Terms] OR diabetes[Title/Abstract] OR hyperglycemia[MeSH Terms] OR hyperglycemia[Title/Abstract] OR blood glucose[MeSH Terms] OR blood glucose[Title])) AND (Case-Control Studies[MeSH Terms] OR Cross-Over Studies[MeSH Terms] OR Cohort Studies[MeSH Terms] OR Systematic Review[Publication Type] OR Meta-Analysis[Publication Type] OR systematic review[Title/Abstract] OR meta-analysis[Title/Abstract] OR cohort[Title/Abstract] OR cross-over[Title/Abstract] OR crossover[Title/Abstract] OR case-control[Title/Abstract] OR prospective[Title/Abstract] OR retrospective[Title/Abstract] OR longitudinal[Title/Abstract] OR follow-up[Title/Abstract] OR Dose-Response Relationship, Drug[MeSH Terms] OR dose-response[Title/Abstract]) AND (Risk[MeSH Terms] OR Odds Ratio[MeSH Terms] OR risk[Title/Abstract] OR odds ratio[Title/Abstract] OR cross-product ratio[Title/Abstract] OR hazards ratio[Title/Abstract] OR hazard ratio[Title/Abstract])) AND ((1970/01/01[PDat] : 2019/12/31[PDat]) NOT (animals[MeSH Terms] NOT Humans[MeSH Terms])))) AND (glaucoma[MeSH Terms] OR cataract[MeSH Terms] OR glaucoma[Title/Abstract] OR cataract[Title/Abstract])

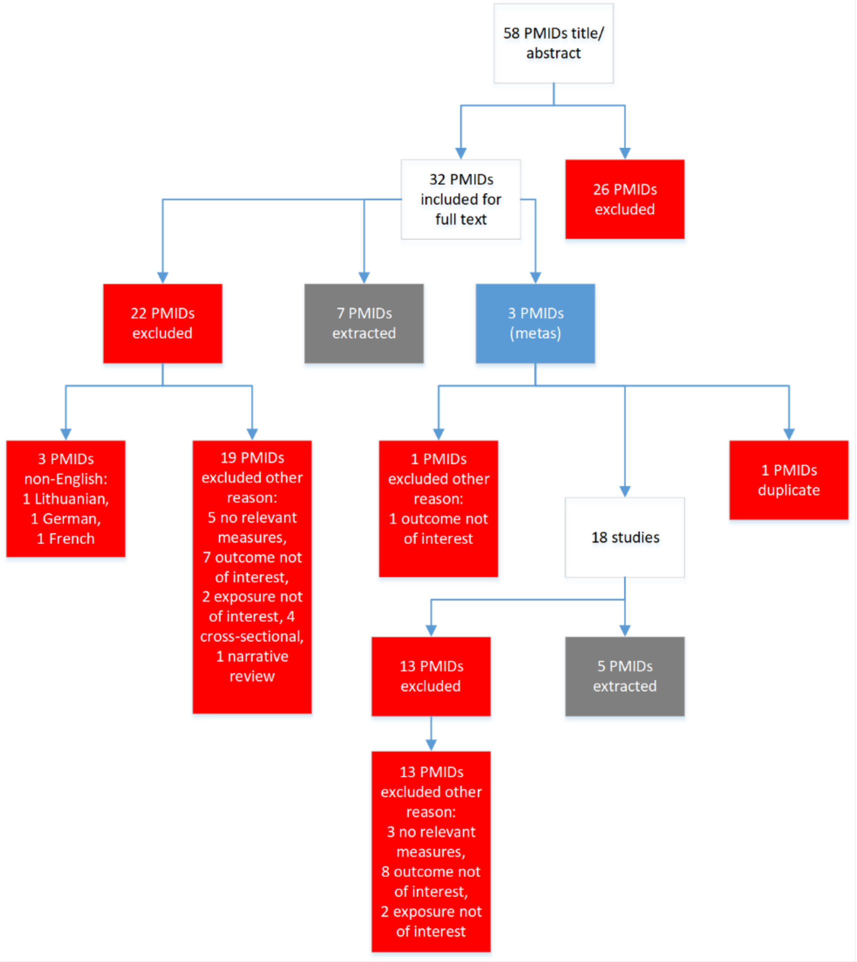

Outcomes: lung cancer, liver cancer, colorectal cancer, breast cancer, pancreas cancer, bladder cancer

- Previously accepted studies

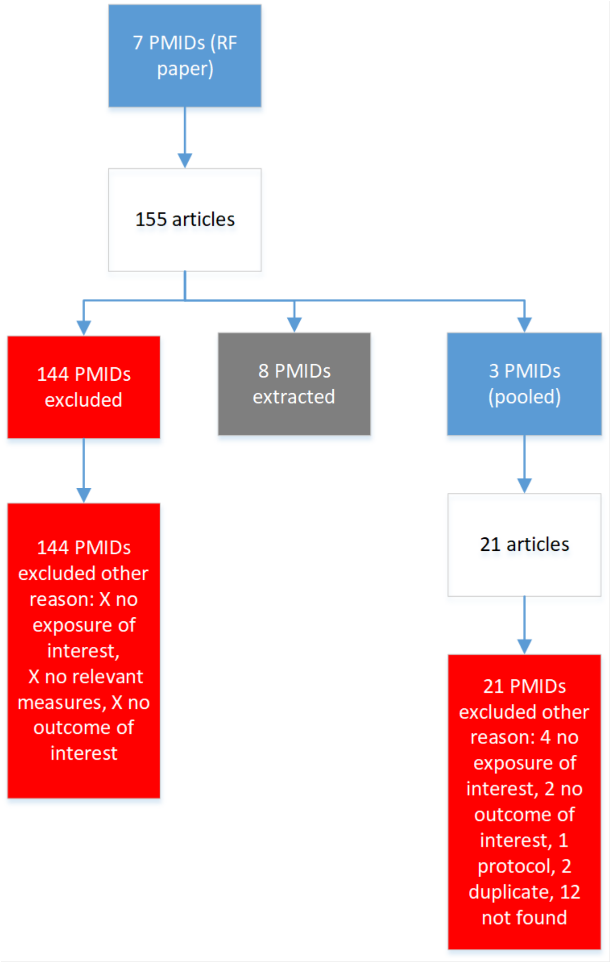

- Search string

(((((diabetes[MeSH Terms] OR diabetes[Title/Abstract] OR hyperglycemia[MeSH Terms] OR hyperglycemia[Title/Abstract] OR blood glucose[MeSH Terms] OR blood glucose[Title])) AND (Case-Control Studies[MeSH Terms] OR Cross-Over Studies[MeSH Terms] OR Cohort Studies[MeSH Terms] OR Systematic Review[Publication Type] OR Meta-Analysis[Publication Type] OR systematic review[Title/Abstract] OR meta-analysis[Title/Abstract] OR cohort[Title/Abstract] OR cross-over[Title/Abstract] OR crossover[Title/Abstract] OR case-control[Title/Abstract] OR prospective[Title/Abstract] OR retrospective[Title/Abstract] OR longitudinal[Title/Abstract] OR follow-up[Title/Abstract] OR Dose-Response Relationship, Drug[MeSH Terms] OR dose-response[Title/Abstract]) AND (Risk[MeSH Terms] OR Odds Ratio[MeSH Terms] OR risk[Title/Abstract] OR odds ratio[Title/Abstract] OR cross-product ratio[Title/Abstract] OR hazards ratio[Title/Abstract] OR hazard ratio[Title/Abstract])) AND ((1970/01/01[PDat] : 2019/12/31[PDat]) NOT (animals[MeSH Terms] NOT Humans[MeSH Terms])))) AND (cancer[MeSH Terms] OR neoplasm[MeSH Terms] OR cancer[Title/Abstract] OR neoplasm[Title/Abstract])

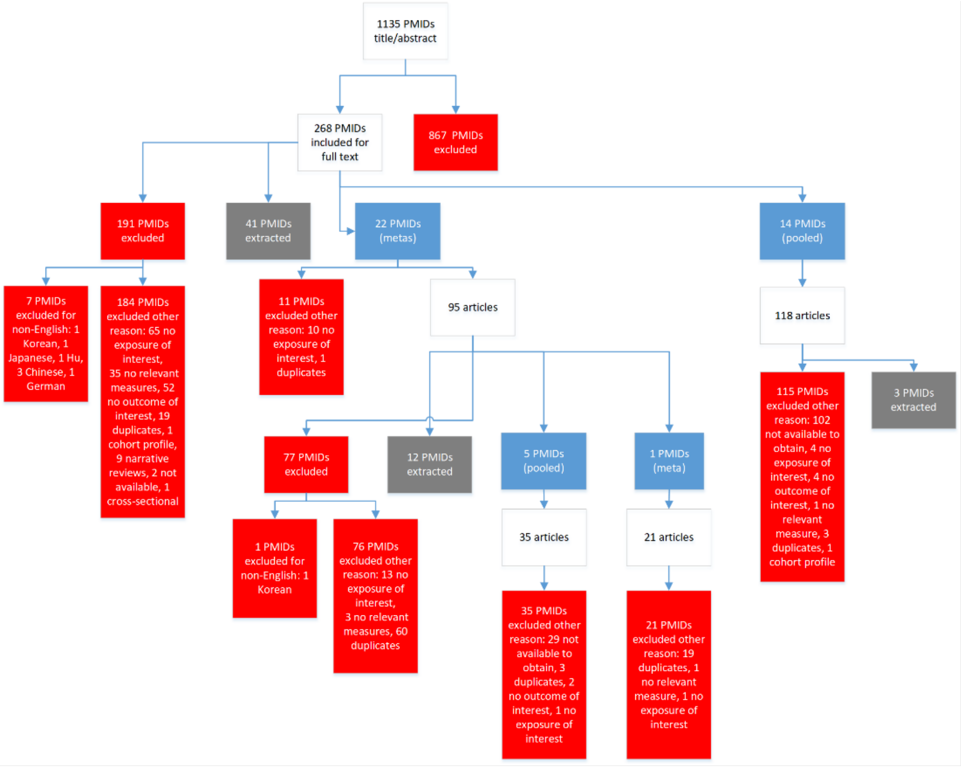

Outcome: peripheral arterial disease

An opportunistic search was undertaken for peripheral arterial disease in 2022. A review of studies included in previous GBD rounds and original studies described in meta-analyses for the relationship between peripheral arterial disease and high FPG was prioritized due to resource constraints. As a result of this opportunistic search, 10 sources and 15 data points were included for peripheral arterial disease in GBD 2021.

Data inputs

Exposure

We used all available sources on FPG and prevalence of diabetes in the FPG model. Data inputs came from three sources:

- Estimates of mean FPG in a representative population
- Individual-level data of FPG measured from surveys
- Estimates of diabetes prevalence in a representative population

Data sources that did not report mean FPG or prevalence of diabetes were excluded from analysis. When a study reported both mean FPG and prevalence of diabetes, we used the mean FPG for exposure estimates. Where possible, individual-level data supersede any data presented in a published study or report. Individual-level data were aggregated to produce estimates for each 5-year age group, sex, location, and year of a survey.

Table 1: *Data inputs for exposure for high FPG*

	Countries with data	New sources	Total sources
Exposure	151	133	493

Relative risk

Table 2: *Data inputs for relative risks for high FPG*

	Countries with data	New sources	Total sources
Relative risks	37	236	240

Data processing

We performed several processing steps to the data in order to address sampling and measurement inconsistencies that ensure the data are comparable across data sources and between diabetes mellitus prevalence modelling efforts.

1. *Small sample size*
   Data with a sample size of 10 or less were outliered prior to modelling.
2. *Diabetes prevalence processing*
   We used an ensemble distribution to estimate mean FPG based on prevalence of diabetes for sources where data on mean FPG were not available, but there were data on diabetes prevalence. Essentially, we constructed a distribution based on unit-level data available in 31 countries. Before predicting mean FPG from prevalence of diabetes, we ensured that the prevalence of diabetes was based on the reference case definition: FPG greater than or equal to 126 mg/dL (7 mmol/L) or on treatment. For more details on how the case definition crosswalk is conducted, please see the diabetes mellitus appendix section. Then, we predicted out the mean FPG by age and sex.
3. *Age and sex splitting*: Reported estimates of mean FPG were split by age and sex where possible. First, if studies reported mean FPG for broad age groups by sex, and also by specific age groups but for both sexes combined, age-specific estimates were split by sex using the sex ratio from within the study. Second, input data reporting mean FPG for both sexes that could not be split using a within-study ratio were split using a sex ratio derived from a meta-analysis of existing sex-specific data using meta-regression—Bayesian, regularised, trimmed (MR-BRT).[1] Finally, where studies reported estimates across age groups spanning more than five years, these were split into five-year age groups using either the age midpoint of the estimate or the diabetes prevalence age pattern estimated by disease model—Bayesian meta-regression (DisMod-MR 2.1)[2] from a model that contained the subset of diabetes prevalence data with age range less than 25 years. Additional information on DisMod-MR 2.1 can be found in appendix 1, section 4.5 of the reference article.

Modelling strategy

Exposure

Exposure estimates were produced for every year between 1980 and 2021 for each national and subnational location, sex, and for each 5-year age group starting from 25 years. As in previous rounds of GBD, we used a spatiotemporal Gaussian process regression (ST-GPR)[3] framework to model the mean

FPG at the location-, year-, age-, and sex-level. Additional information on ST-GPR can be found in appendix 1, section 3.3.3 of the reference article.

To inform our estimates in data-sparse countries, we systematically tested a range of covariates and selected age-specific prevalence of obesity as a covariate based on direction of the coefficient and significance level.

Mean FPG was estimated using a mixed-effects linear regression, run separately by sex:

$$\text{logit}(\text{FPG}_{c,a,t}) = \beta_0 + \beta_1 p_{\text{overweight}_{c,a,t}} + \sum_{k=2}^{16} \beta_k I_{A[a]} + \alpha_s + \alpha_r + \alpha_c + \epsilon_{c,a,t}$$

where $p_{\text{overweight}_{c,a,t}}$ is the prevalence of overweight, $I_{A[a]}$ is an indicator variable for a fixed effect on a given 5-year age group, and α_s α_r α_c are random effects at the super-region, region, and country level, respectively. The estimates were then propagated through the ST-GPR framework to obtain 1000 draws for each location, year, age, and sex.

FPG distributions were created using an ensemble distribution of FPG individual-level data.

Theoretical minimum-risk exposure level

The TMREL for FPG is 4.9–5.3 mmol/L. This was calculated by taking the person-year weighted average of the levels of FPG that were associated with the lowest risk of mortality in the pooled analyses of prospective cohort studies.[4]

Relative risk

In GBD 2021, we attributed burden of 25 level 4 diseases to high FPG. We made several updates in relative risk estimation and population attributable fraction (PAF) estimation detailed below.

First, we re-reviewed all the literature and opportunistically searched for new studies with information on association between blood glucose and each outcome. Please see the Data seeking section for relative risk above for more details.

Second, we used all the available data to create risk curves across the exposure domain for all outcomes. This resulted in transitioning all risk curves to continuous FPG exposure domains. In previous GBD rounds, some high FPG outcomes were modeled as categorical risk-outcome pairs due to the nature of the relative risk data.

We incorporated data in a meta-analytic tool to estimate the relative risk, adjust for bias covariates, as well as evaluate the strength of evidence of the association between FPG and each outcome. Study level bias covariates that were adjusted for in the analysis include study representativeness, quality of confounder adjustment, imputed FPG bounds, exposure range reported in HbA1c values, exposure range reported in OGTT values, exposure defined based on diabetes diagnosis based on anti-diabetic medication use as part of the criteria, studies that exclude people with known/previously diagnosed diabetes from analysis. Additionally, for CVD outcomes, the relative risk was also adjusted for studies where total hemorrhage was reported instead of intracerebral hemorrhage, and the quality of PAD outcome assessment/definition; for CKD outcome, stage of kidney disease was adjusted for.

For relationships that are not a PAF of 1, which include type 1 and type 2 diabetes, 100% of type 1 and type 2 diabetes burden is attributable to high FPG. A description of the methods and approach can be found in the Evidence score documentation, section 6.1 (Continuous Risk-Outcome Pairs). Risk curves for each outcome can be found in the Burden of Proof tool.

Third, from this analysis we determined that the risk of ovarian cancer is not associated with FPG and so was dropped as an outcome, as well as subarachnoid hemorrhage (as described in the Data seeking for relative risk section above).

Finally, we made updates to the relative risk modeling for chronic kidney disease (CKD). We used the risk curve for CKD for total CKD. We do not have separate risk curves for each CKD aetiology due to the paucity of data. Instead, we assumed that estimates from end-stage renal disease registries of the proportion of CKD due to diabetes is the PAF and matched that information by age, sex, year, and location to estimates of diabetes prevalence. Then we back-calculated the relative risk. That modeled data, in addition to data from studies where end stage renal disease was the outcome, were used to calculate the PAF for CKD and high FPG. Then, we subtracted out 100% of the burden of CKD due to type 1 and type 2 diabetes (where the PAF is 1) and proportionally allocated the remaining burden among the remaining three aetiologies and back-calculated the PAF for each aetiology. The GBD 2021 FPG-CKD risk curve can be found in the Burden of Proof tool.

References

1. Zheng, P., Barber, R., Sorensen, R. J., Murray, C. J., & Aravkin, A. Y. (2021). Trimmed constrained mixed effects models: formulations and algorithms. Journal of Computational and Graphical Statistics, 1-13. https://www.tandfonline.com/doi/full/10.1080/10618600.2020.1868303

2. GBD 2019 Diseases and Injuries Collaborators. Global burden of 369 diseases and injuries in 204 countries and territories, 1990–2019: a systematic analysis for the Global Burden of Disease Study 2019. Lancet 2020; 396: 1204–22. doi: https://doi.org/10.1016/S0140-6736(20)30925-9

3. GBD 2019 Risk Factors Collaborators. Global burden of 87 risk factors in 204 countries and territories, 1990–2019: a systematic analysis for the Global Burden of Disease Study 2019. Lancet 2020; 396: 1223–49. doi: https://doi.org/10.1016/S0140-6736(20)30752-2

4. Singh GM, Danaei G, Farzadfar F, et al. The age-specific quantitative effects of metabolic risk factors on cardiovascular diseases and diabetes: a pooled analysis. PloS One 2013; 8: e65174.

High LDL cholesterol

Flowchart

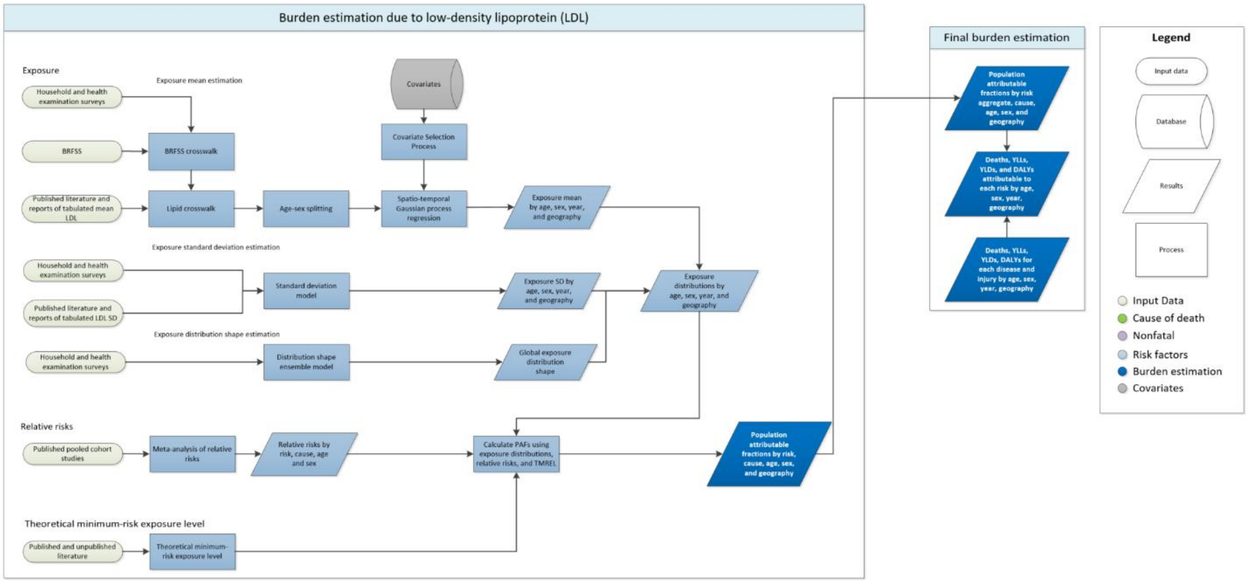

Input data and methodological summary

Definition

Exposure

In earlier iterations of the GBD study, including GBD 2010, 2015, and 2016, we estimated the burden attributable to total cholesterol (TC).[52] Beginning in GBD 2017, we modelled blood concentration of low-density lipoprotein cholesterol (LDL) in units of mmol/L.[53] We used data on blood levels for LDL, TC, triglyceride (TGL), and high-density lipoprotein cholesterol (HDL) from literature and from household survey microdata and reports. We adjusted data for TC, TGL, and HDL using the correction approach described in the "Lipid Crosswalk" section below.

Input data

Exposure

For GBD 2021, a systematic review of data sources included in the LDL exposure model was not performed. However, we updated our original list of sources using the Global Health Data Exchange (GHDx) repository[54] and added six new data sources including STEPS surveys from Vietnam and Zambia, and national surveys from the UK, South Africa, Singapore, Ethiopia, and Colombia. The total counts of the data inputs used for GBD 2021 are shown in Table 1. Details of inclusion and exclusion criteria and data processing steps follow.

Inclusion criteria

Studies were included if they were population-based and reported measurements of total LDL, TC, HDL,

and/or TG from blood tests or if LDL was calculated using the Friedewald equation.[55] We assumed the data were representative of the location if the geography or population chosen was not related to the diseases.

Outliers

All data were used in the modelling process unless an assessment of data strongly suggested that the data were biased. A candidate source was excluded if the quality of study did not warrant a valid estimate because of selection (non-representative populations) or if the study did not provide methodological details for evaluation. In a small number of cases, a datapoint was an outlier candidate if it deviated significantly from other datapoints within the respective country or region or the level was implausibly low or high based on expert judgement and other country data.

Data extraction

Where possible, individual-level data on LDL estimates were extracted from survey microdata, and these were collapsed across demographic groupings to produce mean estimates in the standard GBD five-year age-sex groups. If microdata were unavailable, information from survey reports or from literature were extracted along with any available measure of uncertainty, including standard error, uncertainty intervals, and sample size. Standard deviations were also extracted. Where LDL was reported split by groups other than age, sex, location, and year (eg, by diabetes status), a weighted mean was calculated.

Table 1: Data inputs for exposure for LDL cholesterol

	**Countries with data**	**New sources**	**Total sources**
Exposure	145	6	714

Relative risk

In all versions of GBD up to and including 2019[53,56] we estimated relative risks for TC (total cholesterol) and cardiovascular disease. These risks were derived from a meta-regression analysis of two combined epidemiological studies: the Asia Pacific Cohort Studies Collaboration (APCSC) and the Prospective Studies Collaboration (PSC).[57] For GBD 2017 and 2019, we use the relative risks for TC and cardiovascular disease to approximate the age-specific relative risks for LDL based on the knowledge that the relative risks for LDL and TC exhibit substantial similarity. Additionally, this approach relied on the strong linear correlation observed between TC and LDL at the individual level.

For GBD 2021, we revisited the underlying 52 randomised trials used in a previously published systematic review and meta-analysis of intensive LDL-lowering treatment for the prevention of major vascular events conducted by Wang and colleagues[58] and re-analysed the evidence on LDL as a risk factor of ischaemic heart disease and ischaemic stroke.

The original systematic review was done in accordance with Preferred Reporting Items for Systematic Reviews and Meta-Analyses (PRISMA) guidelines for meta-analyses of interventional studies.[59] Details of the methods, including the PRISMA diagram are reported elsewhere.[58] In brief, using the search terms *"low-density lipoprotein cholesterol" or "cholesterol" or "lowering" or "statins" or "ezetimibe" or "proprotein convertase subtilisin kexin type 9 antibody"*, the following databases were searched: MEDLINE, Embase, and the Cochrane Central Register of Controlled Trials from inception to August 1,

2018. A second search was repeated on June 15, 2019. For the purpose of the GBD final analysis, the search was updated on July 7, 2020, and the search results were updated accordingly. Table 2 shows the search strings and search strategy used for each outcome included in this analysis. The PRISMA flow diagram is shown below.

Table 2: Search strings used in the literature review for LDL cholesterol and cardiovascular outcomes

Outcome	Search strategy	Search string	Number of studies selected
Cardiovascular outcomes	Past systematic review plus literature review of trials	Search string adapted from Wang et al.<br>Updated search string:<br>(hydroxymethylglutaryl coenzyme a reductase inhibitor[tw] OR statin[tw] OR statins[tw] OR ezetimibe[tw] OR PCSK9 inhibitor[tw] OR PCSK9 antibody[tw] OR Proprotein convertase subtilisin kexin type 9 antibody[tw]) AND (LDL-C[tw] OR Low-density lipoprotein cholesterol[tw] OR cholesterol[tw] OR lipid lowering[tw]) AND randomized controlled trial[Publication Type] AND (2019/06/15[PDAT] : 2020/07/07[PDAT])	38

Inclusion criteria were published randomised controlled trials of treatment to reduce cholesterol using statins, ezetimibe, or PCSK9 inhibitors, with at least 1000 patient-years of follow-up, and that reported cardiovascular outcomes of interest. All trials comparing therapy versus no therapy, more-intensive versus less-intensive intervention, or higher versus lower doses of a medication were eligible for inclusion. Primary and secondary prevention trials were included. Trials were excluded if patients were followed up for less than six months or treatment was compared with medications other than cholesterol-lowering drugs or placebo. Trials including enrolling patients on haemodialysis were excluded. The total counts of the relative risks data sources used for GBD 2021 are shown in Table 3.

Table 3: Data inputs for relative risks for LDL cholesterol

	Countries with data	New sources	Total sources
Relative risks for ischaemic heart disease	-	38	38
Relative risks for ischaemic stroke	-	30	30

Total relative risks (unique sources)	-	38	38

No countries reported given the assumption of global relative risks.

Data processing

For GBD 2021, there were no changes to data processing or methods used to estimate the LDL exposure model.

Lipid crosswalk

Total cholesterol consists of three major components: LDL, HDL, and TGL. LDL is often calculated for an individual using the Friedewald equation,[55] shown below:

$$LDL = TC - \left(HDL + \frac{TGL}{2.2}\right)$$

We used this relationship at the individual level to impute the mean LDL for a study population when only data on TC, HDL, and TGL were available. Because studies report different combinations of TC, HDL, and TGL, we constructed a single regression to utilise all available data to evaluate the relationship between each lipid and LDL at the population level. We used the following regression:

$$LDL = ind_{tc}\beta_1 TC - \left(ind_{hdl}\beta_2 HDL + ind_{tgl}\beta_3 TGL\right) + \sum \alpha_l I_l$$

Where ind_{tc}, ind_{hdl}, and ind_{tgl} are indicator variables for whether data are available for a given lipid, I_l is an indicator variable for a given set of available lipids l, and α_l is a unique intercept for each set of available lipid combinations. For example, for sources that only reported TC and HDL, $\alpha_{l=TC,HDL}$ should account for the missing lipid data, ie, TGL. The form of this regression allows us to estimate the betas for each lipid using all available data. As a sensitivity analysis, we also ran separate regressions for each set of available lipids and found that the single regression method had much lower root-mean-squared error. We found almost no relationship between LDL and HDL or TGL when TC was not available, so only studies that reported TC were adjusted to LDL.

Incorporating USA prevalence data

Survey reports and literature often report information only about the prevalence, but not the level, of hypercholesterolemia in the population studied. These sources were not used to model LDL, except for data from the Behavioral Risk Factors Surveillance System (BRFSS) because of the availability of a similarly structured exam survey covering the identical population, the National Health and Nutrition Examination Survey (NHANES). BRFSS is a telephone survey conducted in the USA for all counties. It collects self-reported diagnosis of hypercholesterolemia. These self-reported values of prevalence of raised TC in each age group, sex, USA state, and year were used to predict a mean TC for the same strata with a regression using data from the NHANES, a nationally representative health examination survey of the USA adult population. The regression was:

$$\mathrm{TC}_{l,a,t,s} = \beta_0 + \beta_1 \mathrm{prev}_{l,a,t,s}$$

where $\mathrm{TC}_{\mathrm{l,a,t,s}}$ *is the location, age, time, and sex specific mean total cholesterol and* $\mathrm{prev}_{\mathrm{l,a,t,s}}$ *is the location, age, time, and sex specific prevalence of raised total cholesterol. The coefficients for both models are reported in Table 4.*

Table 4: Coefficients in the sex-specific USA states total cholesterol prediction models

Term	Male model	Female model
Intercept	4.23	4.36
Prevalence	6.25	5.22

Out-of-sample root-mean-square error (RMSE) was used to quantify the predictive validity of the model. The regression was repeated ten times for each sex, each time randomly holding out 20% of the data. The RMSEs from each holdout analysis were averaged to get the average out-of-sample RMSE. The results of this holdout analysis are reported in Table 5. Total cholesterol estimates were crosswalked to LDL using the lipid crosswalk reported above.

Table 5: Out of sample RMSEs of the sex-specific USA states TC prediction models

	Male model	Female model
Out-of-sample RMSE	0.21 mmol/L	0.20 mmol/L

Age and sex splitting

Prior to modelling, data provided in age groups wider than the GBD five-year age groups were processed using the approach outlined in Ng and colleagues.[60] Briefly, age-sex patterns were identified using person-level microdata (60 sources) and estimate age-sex-specific levels of TC from aggregated results reported in published literature or survey reports. To incorporate uncertainty into this process and borrow strength across age groups when constructing the age-sex pattern, we used a model with auto-regression on the change in mean LDL over age groups:

$$\mu_a = \mu_{a-1} + \omega_a$$
$$\omega_a \sim N(\omega_{a-1}, \tau)$$

Where μ_a is the mean predicted value for age group a, μ_{a-1} is the mean predicted value for the age group previous to age group a, ω_a is the difference in mean between age group a and age group *a-1*, ω_{a-1} is the difference between age group *a-1* and age group *a-2*, and τ is a user-input prior on how quickly the mean LDL changes for each unit increase in age. We used a τ of 0.05 mmol/L for this model. Draws of the age-sex pattern were combined with draws of the input data needing to be split to calculate the new variance of age-sex-split datapoints.

Modelling strategy

Exposure

For GBD 2021, there were no changes in the modelling strategy used to estimate LDL exposure. Exposure estimates were produced from 1980 to 2020 for each national and subnational location, sex, and each five-year age group starting from 25. As in GBD 2019, we used a spatiotemporal Gaussian

process regression (ST-GPR) framework to model the mean LDL at the location, year, age, and sex level. Details of the ST-GPR method used in GBD 2021 can be found elsewhere in the appendix.

Covariate selection

The first step of the ST-GPR framework requires the creation of a linear model for predicting LDL at the location, year, age, sex level. Covariates for this model were selected in two stages. First, a list of variables with an expected causal relationship with LDL was created based on significant association found within high-quality prospective cohort studies reported in the published scientific literature. Covariates included in the first step were mean body-mass index (BMI), prevalence of obesity, age- and sex-specific SEV for low fruit, age- and sex-specific SEV for low nuts and seeds, age- and sex-specific SEV for low vegetables, Healthcare Access and Quality (HAQ) Index, and Socio-demographic Index (SDI). The second stage in covariate selection was to test the predictive validity of every possible combination of covariates in the linear model, given the covariates selected above. This was done separately for each sex. Predictive validity was measured without sample root-mean-squared error.

In GBD 2016, the linear model with the lowest root-mean-squared error for each sex was then used in the ST-GPR model. Beginning in GBD 2017, we used an ensemble model of the 50 models with the lowest root-mean-squared error for each sex. This allows us to utilise covariate information from many plausible linear mixed-effects models. The 50 models were each used to predict the mean LDL for every age, sex, location, and year, and the inverse-RMSE-weighted average of this set of 50 predictions was used as the linear prior. The relative weight contributed by each covariate is plotted by sex in Figure 2. The results of the ensemble linear model were used for the first stage in an ST-GPR model. The result of the ST-GPR model are estimates of the mean LDL for each age, sex, location, and year.

Estimate of standard deviation

The standard deviation of LDL within a population was estimated for each national and subnational location, sex, and five-year age group starting from age 25 using the standard deviation from person-level and some tabulated data sources. Person-level microdata accounted for 3009 of the total 4001 rows of data on standard deviation. The remaining 992 rows came from tabulated data. Tabulated data were only used to model standard deviation if they were sex-specific and five-year-age-group-specific and reported a population standard deviation LDL. The LDL standard deviation function was estimated using a linear regression:

$$\log\left(SD_{c,a,t,s}\right) = \beta_0 + \beta_1 \log\left(mean\_LDL_{c,a,t,s}\right) + \beta_2 sex + \sum_{k=3}^{17} \beta_k I_{A[a]}$$

where $mean\_LDL_{c,a,t,s}$ is the country-, age-, time-, and sex-specific mean LDL estimate from ST-GPR and $I_{A[a]}$ is a dummy variable for a fixed effect on a given five-year age group.

Figure 1: Covariates relative weights

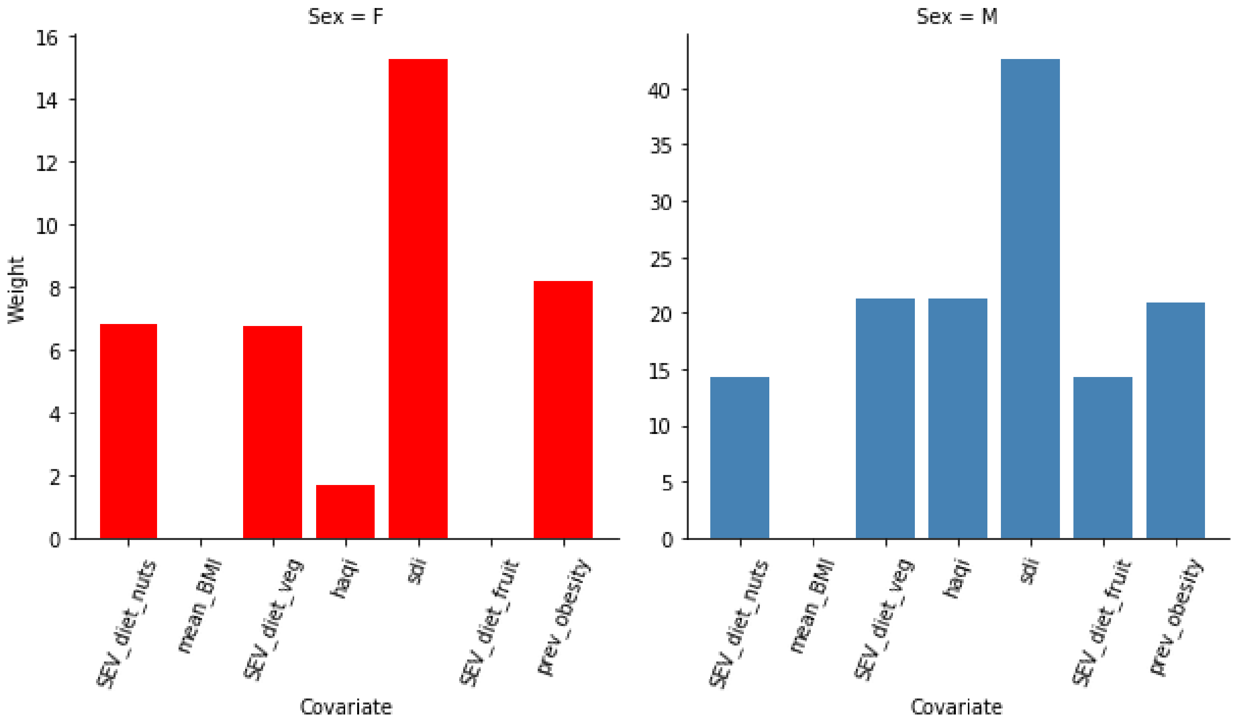

Distribution shape modelling

The shape of the distribution of LDL was estimated using all available person-level microdata sources, which was a subset of the input data into the modelling process. The distribution shape modelling framework for GBD 2021 is detailed elsewhere in the appendix. Briefly, an ensemble distribution created from a weighted average of distribution families was fit for each individual microdata source, separately by age and sex. The weights for the distribution families for each individual source were then averaged and weighted to create a global ensemble distribution for each sex. Figure 2 shows the final ensemble distribution for both sexes combined.

Figure 2. Global ensemble distribution fit and distribution-specific weights for LDL

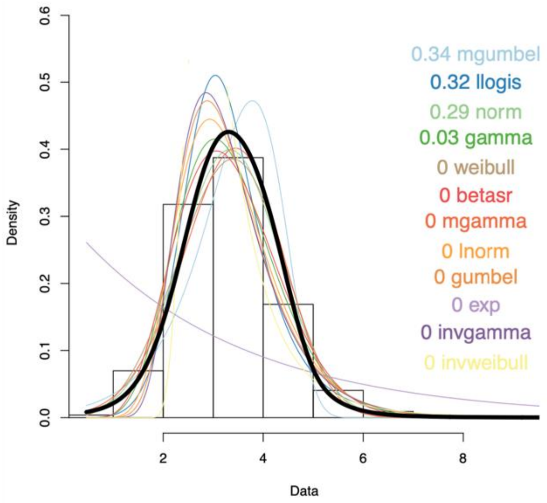

Theoretical minimum risk exposure level

For GBD 2017, based on a meta-analysis of randomised trials that showed that outcomes can be improved even at low levels of LDL cholesterol, below 1.3 mmol/L,[61] the TMREL for LDL was define as a uniform distribution between 0.7 and 1.3 mmol/L. This value remained unchanged for GBD 2019. For GBD 2021, we used the LDL values reported in the randomised trials used to estimate the LDL relative risks to update the TMREL as well. We first identify the LDL levels from the reference exposure (or control) and alternate (or comparison) groups of the studies for all outcomes associated to LDL. Then, we compute the midpoint of the lower and upper bounds of the reference exposure groups and define the TMREL as a uniform distribution with lower/upper bounds of 0.9 and 1.4. The lower bound was given by the percentile 15 of the midpoints calculated above, and the upper bound was given by the percentile 15 of only the upper bounds of the reference exposure groups. We calculated these ranges for each RO pair, and then took the weighted average of the ranges using global cause-specific deaths as the weights.

The TMREL was defined as a uniform distribution rather than a fixed value to represent the uncertainty regarding the level at which the scientific evidence was consistent with adverse effects of exposure.

Relative risk

For GBD 2021, we switched from using DisMod-MR 2.1[62] to meta-regression—Bayesian, regularised, Trimmed (MR-BRT)[63] to estimate the effect sizes and generate a dose–response curve for each of the outcomes associated with LDL (ischaemic heart disease and ischaemic stroke). This new approach enabled us to incorporate random effects across studies accounting for between-study heterogeneity, data sparsity, and publication bias. Relative risks (RR) for ischaemic heart disease and ischaemic stroke were modelled with log (RR) as the dependent variable and LDL exposure values as the independent variable. Due to data sparsity, and given that most of the studies included in the meta-regression do not report information disaggregated by stroke subtypes (ie, ischaemic vs. haemorrhagic stroke), we decided to combine data sources that reported "stroke" and "ischaemic stroke" as outcomes in a single model, assuming that physiologically LDL can be associated with ischaemic stroke only. Further technical

details supporting estimation of non-log-linear risk curves using relative risks, trimming, and general meta-analysis models are detailed elsewhere[64] and can be found in a different section of this appendix. We implemented the Fisher scoring correction to the heterogeneity parameter, which corrects for data-sparse situations. In such cases, the between-study heterogeneity parameter estimate may be 0, simply from lack of data. The Fisher scoring correction uses a quantile of gamma, which is sensitive to the number of studies, study design, and reported uncertainty. In addition, we have added methodology that can detect and flag publication bias. The approach is based on the classic Egger's regression strategy,[65] which is applied to the residuals of the model. In the current implementation, we do not correct for publication bias, but flag the risk–outcome pairs where the risk for publication bias is significant. For this analysis, no risk of publication bias was detected for LDL and related outcomes, as shown in the funnel plots in figures 3 and 4. Given the data limitations. we assumed that the estimated RRs were universal for all countries and sex categories and were the same for incidence and mortality.

Figure 3. Low-density lipoprotein cholesterol and ischaemic heart disease log relative risk (a) and residuals by estimated standard deviation (b)

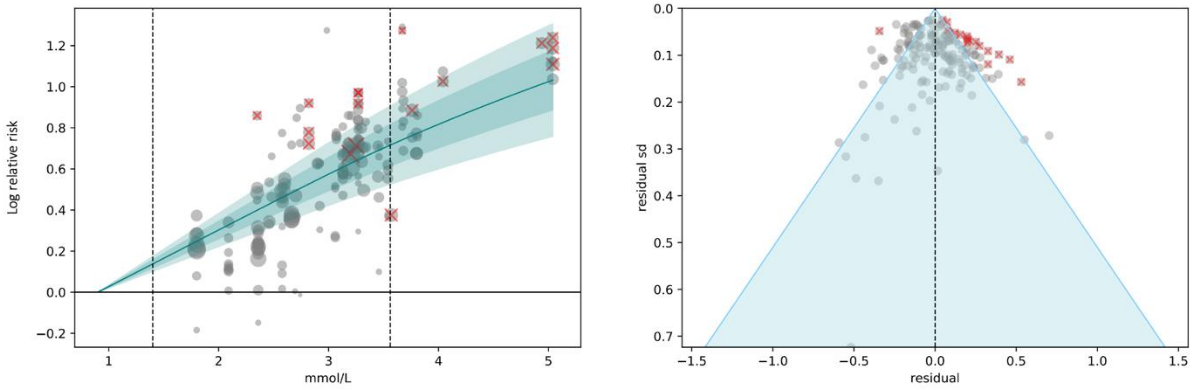

Figure 4: Low-density lipoprotein cholesterol and ischaemic stroke log relative risk (a) and residuals by estimated standard deviation (b)

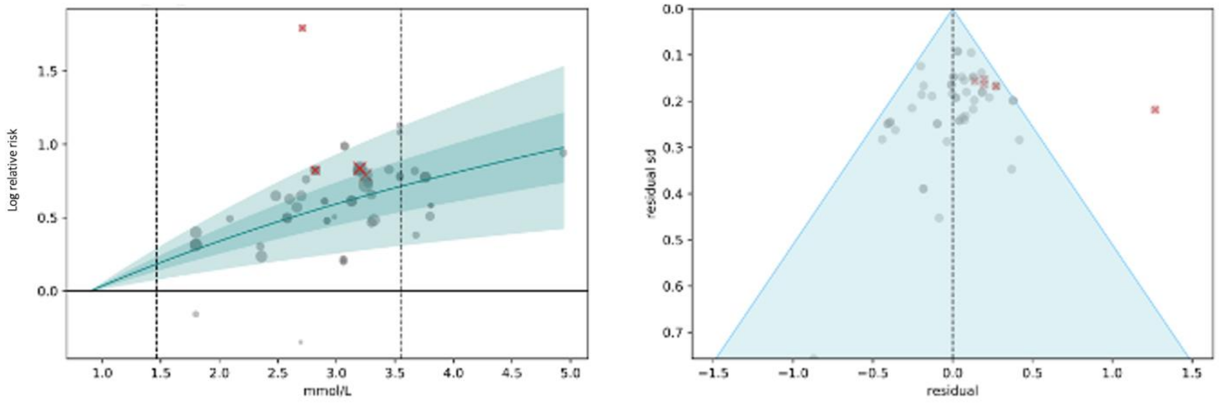

Figures 3 and 4. The risk curves are computed relative to an LDL cholesterol value of 0.9 mmol/L. In panel (a) the dark line indicates mean relative risk across LDL cholesterol exposure levels; the light and dark shading show 95% uncertainty intervals with and without between-study heterogeneity, respectively; the size of the datapoints corresponds to the inverse of the standard error, with those trimmed during the model fitting process marked by a red x; and the dashed lines represent the 15th percentile of the reference exposure and the 85th percentile of the alternative exposure. To visualise log-relative-risk points in panel (a), we plotted each datapoint with the x-value at the midpoint of the alternative group and the y-value offset by the difference between the reported and predicted log risk. Panel (b) depicts a customised funnel plot, with the x-axis

representing residuals between predicted and observed relative risks, and the y-axis representing uncertainty from both measurement error and between-study heterogeneity.

To account for the heterogeneity of the effect size by age and given the limitations of both the available data and MR-BRT in terms of lack of age-specific data and estimates, we estimated cause-specific age attenuation factors using a second MR-BRT model with log (RR) as the dependent variable and age as an independent variable, including data for TC from the APCSC and the PSC cohorts only reported by Singh and colleagues. We then applied these cause-specific attenuation factors to the corresponding RR curve using the mid age at event observed in these two cohort studies (60–64 years) as the reference group to finally generate RR for standard five-year GBD age categories starting at age 25. With this new methodology, we removed the previous assumption that there is not a protective effect of LDL and stroke after age 70.

In future iterations of GBD, we plan to update the MR-BRT tool to be able to incorporate a second spline on age and generate more accurate age-specific RR curves.

PRISMA 2020 flow diagram. Study selection flow diagram for meta-analysis of LDLc and ischaemic heart disease and ischaemic stroke combined

From: Page MJ, McKenzie JE, Bossuyt PM, Boutron I, Hoffmann TC, Mulrow CD, et al. The PRISMA 2020 statement: an updated guideline for reporting systematic reviews. *BMJ* 2021;372:n71. doi: 10.1136/bmj.n71

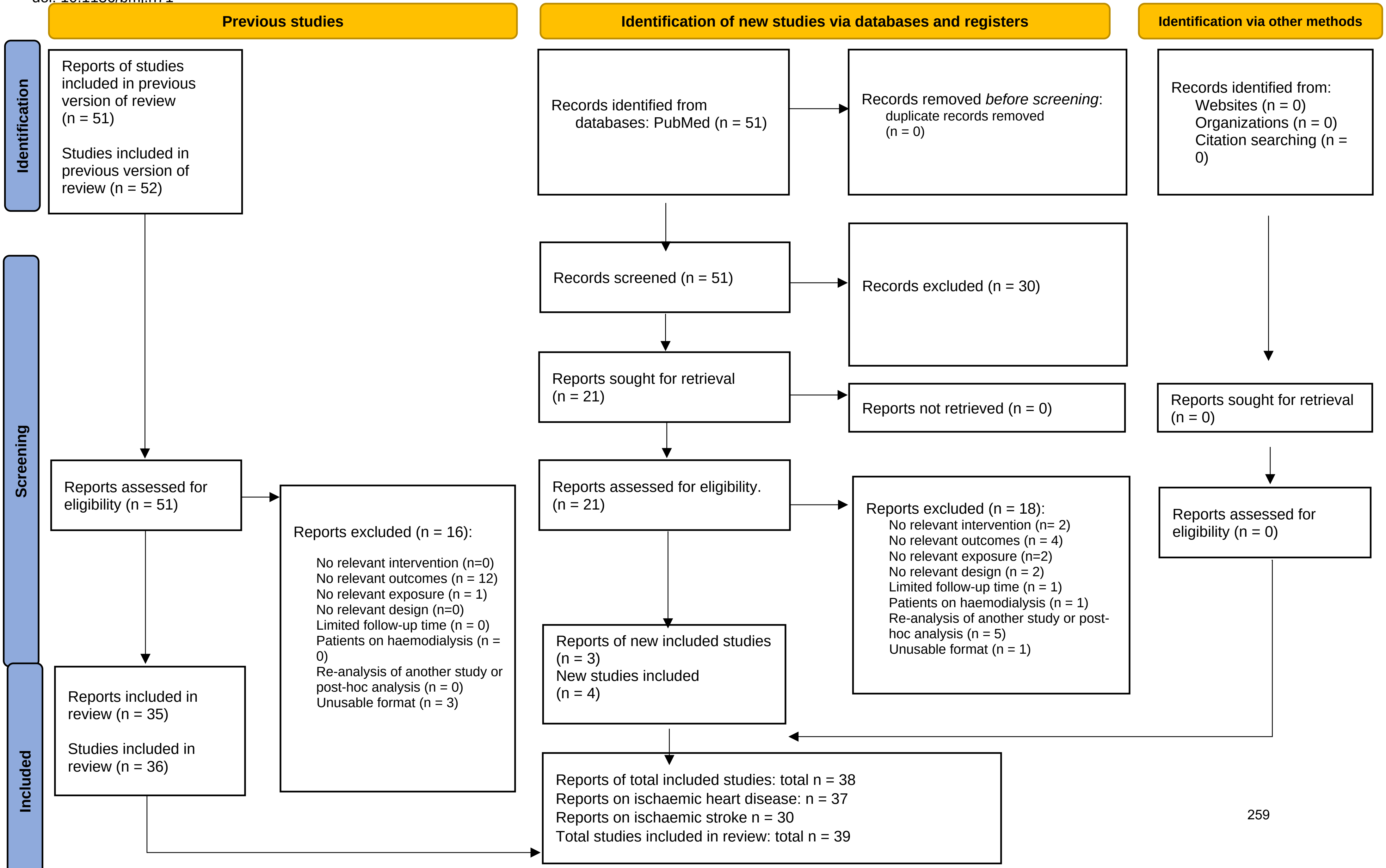

Citations

1 Stevens GA, Alkema L, Black RE. Guidelines for Accurate and Transparent Health Estimates Reporting: the GATHER statement. *Lancet* 2016.

2 Lim SS, Vos T, Flaxman AD. A comparative risk assessment of burden of disease and injury attributable to 67 risk factors and risk factor clusters in 21 regions, 1990–2010: a systematic analysis for the Global Burden of Disease Study 2010. *The Lancet* 2012; **380**: 2224–60.

3 Forouzanfar M, Afshin A, Alexander LT, Anderson H, Bhutta Z, Murray CJL. Global, regional, and national comparative risk assessment of 79 behavioural, environmental and occupational, and metabolic risks or clusters of risks, 1990-2015: a systematic analysis for the Global Burden of Disease Study 2015. *Lancet* 2016; **388**: 1659–724.

4 Gakidou E, Afshin A, Abajobir AA, *et al.* Global, regional, and national comparative risk assessment of 84 behavioural, environmental and occupational, and metabolic risks or clusters of risks, 1990–2016: a systematic analysis for the Global Burden of Disease Study 2016. *The Lancet* 2017; **390**: 1345–422.

5 Stanaway JD, Afshin A, Gakidou E, *et al.* Global, regional, and national comparative risk assessment of 84 behavioural, environmental and occupational, and metabolic risks or clusters of risks for 195 countries and territories, 1990–2017: a systematic analysis for the Global Burden of Disease Study 2017. *The Lancet* 2018; **392**: 1923–94.

6 Murray CJL, Aravkin AY, Zheng P. Global burden of 87 risk factors in 204 countries and territories, 1990–2019: a systematic analysis for the Global Burden of Disease Study 2019. *The Lancet* 2020; **396**: 1223–49.

7 Murray CJ, Lopez AD. Global mortality, disability, and the contribution of risk factors: Global Burden of Disease Study. *Lancet* 1997; **349**: 1436–42.

8 Murray CJ, Lopez AD. On the comparable quantification of health risks: lessons from the Global Burden of Disease Study. *Epidemiology* 1999; **10**: 594–605.

9 Food, nutrition, physical activity and the prevention of cancer: a global perspective. Washington, D.C: World Cancer Research Fund & American Institute for Cancer Research, 2007.

10 Zheng P, Barber R, Sorensen RJ, Murray CJ, Aravkin AY. Trimmed constrained mixed effects models: formulations and algorithms. *Journal of Computational and Graphical Statistics* 2021; 1–13.

11 Zheng P. limetr: limetr: linear mixed effects model with trimming. https://github.com/zhengp0/limetr (accessed July 28, 2021).

12 Zheng P. xspline: xspline: Advanced spline tools. https://github.com/zhengp0/xspline (accessed July 28, 2021).

13 Viechtbauer W. Conducting meta-analyses in R with the metafor package. *Journal of statistical software* 2010; **36**: 1–48.

14 de Boor C. A practical guide to splines (applied mathematical sciences, 27). New York: Springer, 2001 https://link.springer.com/book/9780387953663 (accessed April 4, 2022).

15 Rousseeuw PJ, Leroy AM. Robust regression and outlier detection. John Wiley & Sons, 2005.

16 Rousseeuw PJ. Least median of squares regression. *Journal of the American Statistical Association* 1984; **79**: 871–80.

17 Huber PJ. Robust Statistics. John Wiley & Sons, 2004.

18 Rousseeuw P. Multivariate estimation with high breakdown point. *Mathematical Statistics and Applications Vol B* 1985; : 283–97.

19 Aravkin A, Davis D. Trimmed statistical estimation via variance reduction. *Mathematics of Operations Research* 2020; **45**: 292–322.

20 Motzkin TS, Raiffa H, Thompson GL, Thrall RM. 3. The Double Description Method. In: 3. The Double Description Method. Princeton University Press, 2016: 51–74.

21 Guyatt GH, Oxman AD, Vist G, *et al.* GRADE guidelines: 4. Rating the quality of evidence—study limitations (risk of bias). *Journal of clinical epidemiology* 2011; **64**: 407–15.

22 Efron B, Hastie T, Johnstone I, Tibshirani R. Least angle regression. *The Annals of statistics* 2004; **32**: 407–99.

23 Tibshirani R. Regression shrinkage and selection via the lasso. *Journal of the Royal Statistical Society: Series B (Methodological)* 1996; **58**: 267–88.

24 Kontopantelis E, Springate DA, Reeves D. A re-analysis of the Cochrane Library data: the dangers of unobserved heterogeneity in meta-analyses. *PloS one* 2013; **8**: e69930.

25 Ioannidis JP, Patsopoulos NA, Evangelou E. Uncertainty in heterogeneity estimates in meta-analyses. *Bmj* 2007; **335**: 914–6.

26 Biggerstaff BJ, Tweedie RL. Incorporating variability in estimates of heterogeneity in the random effects model in meta-analysis. *Statistics in medicine* 1997; **16**: 753–68.

27 Higgins JP, Thompson SG. Quantifying heterogeneity in a meta-analysis. *Statistics in medicine* 2002; **21**: 1539–58.

28 Sterne JA, Egger M. Chapter 6: Regression methods to detect publication and other bias in meta-analysis. In: Rothstein H, Sutton A, Borenstein M, eds. Publication bias in meta-analysis: Prevention, assessment and adjustments. John Wiley & Sons, Ltd, 2005.

29 Egger M, Smith GD, Schneider M, Minder C. Bias in meta-analysis detected by a simple, graphical test. *BMJ* 1997; **315**: 629–34.

30 Burkart KG, Brauer M, Aravkin AY. Estimating the cause-specific relative risks of non-optimal temperature on daily mortality: a two-part modelling approach applied to the Global Burden of Disease Study. *The Lancet* 2021; **398**: 685–97.

31 Harris PA, Taylor R, Thielke R, Payne J, Gonzalez N, Conde JG. Research Electronic Data Capture (REDCap) - A metadata-driven methodology and workflow process for providing translational research informatics support. *J Biomed Inform* 2009; **42**: 377–81.

32 GBD 2015 Diseases and Injury Incidence and prevalence Collaborators. Global, regional, and national incidence, prevalence, and years lived with disability (YLDs) for 310 acute and chronic diseases and injuries, 1990-2015: a systematic analysis for the Global Burden of Disease Study 2015. *The Lancet Under review*.

33 Flaxman AD, Vos T, Murray CJL, Kiyono P, editors. An integrative metaregression framework for descriptive epidemiology, 1 edition. Seattle: University of Washington Press, 2015.

34 Vasudevan S, Ramos F, Nettleton E, Durrant-Whyte H, Blair A. Gaussian Process modeling of large scale terrain. In: 2009 IEEE International Conference on Robotics and Automation. 2009: 1047–53.

35 Rasmussen CE, Williams CKI. Gaussian Processes for Machine Learning. Cambridge, Mass: The MIT Press, 2005.

36 Ng M, Fleming T, Robinson M, *et al.* Global, regional, and national prevalence of overweight and obesity in children and adults during 1980–2013: a systematic analysis for the Global Burden of Disease Study 2013. *Lancet* 2014; **384**: 766–81.

37 Ng M, Freeman MK, Fleming TD, *et al.* Smoking Prevalence and Cigarette Consumption in 187 Countries, 1980-2012. *JAMA* 2014; **311**: 183–92.

38 Massey Jr FJ. The Kolmogorov-Smirnov test for goodness of fit. *Journal of the American statistical Association* 1951; **46**: 68–78.

39 Vander Hoorn S, Ezzati M, Rodgers A, Lopez AD, Murray CJL. Estimating attributable burden of disease from exposure and hazard data. In: Comparative Quanitifcation of Health Risks: Global and regional burden of disease attribution to selected major risk factors. World Health Organisation, 2004: 2129–40.

40 Preston SH. Causes and Consequences of Mortality Declines in Less Developed Countries during the Twentieth Century. In: Population and economic change in developing countries. Chicago: Univ. of Chicago Pr, 1980: 289–360.

41 Carnahan E, Lim SS, Nelson EC, *et al.* Validation of a new predictive risk model: measuring the impact of major modifiable risks of death for patients and populations. *The Lancet* 2013; **381**: S26.

42 Danaei G, Singh GM, Paciorek CJ, *et al.* The global cardiovascular risk transition: associations of four metabolic risk factors with national income, urbanization, and Western diet in 1980 and 2008. *Circulation* 2013; **127**: 1493–502, 1502e1-8.

43 McDonald CM, Olofin I, Flaxman S. The effect of multiple anthropometric deficits on child mortality: meta-analysis of individual data in 10 prospective studies from developing countries. *Am J Clin Nutr* 2013; **97**: 896–901.

44 Olofin I, McDonald CM, Ezzati M, et al. Associations of suboptimal growth with all-cause and cause specific mortality in children under five years: a pooled analysis of ten prospective studies. *PloS ONE*; **8**: e64636.

45 Ki Global Health. (n.d.). https://www.kiglobalhealth.org/.

46 Das Gupta P. Standardization and Decomposition of Rates: A User's Manual. Washington D.C.: U.S. Bureau of the Census, 1993.

47 WHO | WHO Global Database on Child Growth and Malnutrition. WHO. http://www.who.int/nutgrowthdb/en/ (accessed July 30, 2018).

48 Wang Y, Chen H-J. Use of Percentiles and Z-Scores in Anthropometry. In: Preedy VR, ed. Handbook of Anthropometry. New York, NY: Springer New York, 2012: 29–48.

49 Uribe Á, Cecilia M, López Gaviria A, Estrada Restrepo A. Concordance between Z scores from WHO 2006 and the NCHS 1978 growth standards of children younger than five. Antioquia-Colombia. *Perspectivas en Nutrición Humana* 2008; **10**: 177–87.

50 McDonald CM, Olofin I, Flaxman S, *et al.* The effect of multiple anthropometric deficits on child mortality: meta-analysis of individual data in 10 prospective studies from developing countries. *Am J Clin Nutr* 2013; **97**: 896–901.

51 Cole TJ, Lobstein T. Extended international (IOTF) body mass index cut-offs for thinness, overweight and obesity. *Pediatr Obes* 2012; **7**: 284–94.

52 Global, regional, and national comparative risk assessment of 79 behavioural, environmental and occupational, and metabolic risks or clusters of risks, 1990–2015: a systematic analysis for the Global Burden of Disease Study 2015. *Lancet* 2016; **388**: 1659–724.

53 Stanaway JD, Afshin A, Gakidou E, *et al.* Global, regional, and national comparative risk assessment of 84 behavioural, environmental and occupational, and metabolic risks or clusters of risks for 195 countries and territories, 1990–2017: a systematic analysis for the Global Burden of Disease Study 2017. *The Lancet* 2018; **392**: 1923–94.

54 Global Health Data Exchange | GHDx. http://ghdx.healthdata.org/ (accessed Oct 8, 2021).

55 Friedewald WT, Levy RI, Fredrickson DS. Estimation of the concentration of low-density lipoprotein cholesterol in plasma, without use of the preparative ultracentrifuge. *Clin Chem* 1972; **18**: 499–502.

56 Murray CJL, Aravkin AY, Zheng P, *et al.* Global burden of 87 risk factors in 204 countries and territories, 1990–2019: a systematic analysis for the Global Burden of Disease Study 2019. *The Lancet* 2020; **396**: 1223–49.

57 Singh GM, Danaei G, Farzadfar F, *et al.* The Age-Specific Quantitative Effects of Metabolic Risk Factors on Cardiovascular Diseases and Diabetes: A Pooled Analysis. *PLOS ONE* 2013; **8**: e65174.

58 Wang N, Fulcher J, Abeysuriya N, *et al.* Intensive LDL cholesterol-lowering treatment beyond current recommendations for the prevention of major vascular events: a systematic review and meta-analysis of randomised trials including 327 037 participants. *Lancet Diabetes Endocrinol* 2020; **8**: 36–49.

59 Moher D, Liberati A, Tetzlaff J, Altman DG, Group TP. Preferred Reporting Items for Systematic Reviews and Meta-Analyses: The PRISMA Statement. *PLOS Medicine* 2009; **6**: e1000097.

60 Ng M, Fleming T, Robinson M, *et al.* Global, regional, and national prevalence of overweight and obesity in children and adults during 1980–2013: a systematic analysis for the Global Burden of Disease Study 2013. *The Lancet* 2014; **384**: 766–81.

61 Boekholdt SM, Hovingh GK, Mora S, *et al.* Very Low Levels of Atherogenic Lipoproteins and the Risk for Cardiovascular EventsA Meta-Analysis of Statin Trials. *J Am Coll Cardiol* 2014; **64**: 485–94.

62 Vos T, Lim SS, Abbafati C, *et al.* Global burden of 369 diseases and injuries in 204 countries and territories, 1990–2019: a systematic analysis for the Global Burden of Disease Study 2019. *The Lancet* 2020; **396**: 1204–22.

63 Zheng P, Barber R, Sorensen RJD, Murray CJL, Aravkin AY. Trimmed Constrained Mixed Effects Models: Formulations and Algorithms. *Journal of Computational and Graphical Statistics* 2021; **30**: 544–56.

64 Sorensen (rsoren@uw.edu) R. Health Metrics Toolbox. https://ihmeuw-msca.github.io/index.html (accessed Oct 8, 2021).

65 Bias in meta-analysis detected by a simple, graphical test | The BMJ. https://www.bmj.com/content/315/7109/629.full (accessed Oct 8, 2021).

66 Prospective Studies Collaboration. Collaborative overview ('meta-analysis’) of prospective observational studies of the associations of usual blood pressure and usual cholesterol levels with common causes of death: protocol for the second cycle of the Prospective Studies Collaboration. *J Cardiovasc Risk* 1999; **6**: 315–20.

67 The effect of Blood Pressure on Kidney Failure: a systematic review and meta-analysis in 2.7 million participants [Unpublished] | GHDx. https://internal-ghdx.healthdata.org/record/effect-blood-pressure-kidney-failure-systematic-review-and-meta-analysis-27-million (accessed July 11, 2023).

68 Rapsomaniki E, Timmis A, George J, *et al.* Blood pressure and incidence of twelve cardiovascular diseases: lifetime risks, healthy life-years lost, and age-specific associations in 1·25 million people. *Lancet* 2014; **383**: 1899–911.

69 Denaxas SC, George J, Herrett E, *et al.* Data Resource Profile: Cardiovascular disease research using linked bespoke studies and electronic health records (CALIBER). *Int J Epidemiol* 2012; **41**: 1625–38.

70 Page MJ, McKenzie JE, Bossuyt PM, *et al.* The PRISMA 2020 statement: an updated guideline for reporting systematic reviews. *BMJ* 2021; **372**: n71.

71 Singh GM, Danaei G, Farzadfar F, *et al.* The age-specific quantitative effects of metabolic risk factors on cardiovascular diseases and diabetes: a pooled analysis. *PLoS ONE* 2013; **8**: e65174.

72 Collaboration APCS, others. Blood pressure and cardiovascular disease in the Asia Pacific region. *Journal of hypertension* 2003; **21**: 707–16.

73 Hersbach H, Bell B, Berrisford P, *et al.* Global reanalysis : goodbye Global reanalysis : goodbye ERA-Interim , hello. 2019. DOI:10.21957/vf291hehd7.

74 Copernicus Climate Change Service (C3S) (2017): ERA5: Fifth generation of ECMWF atmospheric reanalyses of the global climate. Copernicus Climate Change Service Climate Data Store (CDS), September 2019. .

75 Geography and Environmental Science, University of Southampton. Age and Sex Structures, Global Per Country 2000-2020 - WorldPop. Southampton , United Kingdom: Geography and Environmental Science, University of Southampton, 2018. .

76 Zheng P, Aravkin AY, Barber R, Sorensen RJD, Murray CJL. Trimmed Constrained Mixed Effects Models: Formulations and Algorithms. 2019; published online Sept 23.

77 Rousseeuw PJ. Least Median of Squares Regression. *Journal of the American Statistical Association* 1984; **79**: 871–80.

78 Aravkin A, Davis D. Trimmed Statistical Estimation via Variance Reduction. *Mathematics of Operations Research* 2019; : moor.2019.0992.

79 Yang E, Lozano AC, Aravkin A. A general family of trimmed estimators for robust high-dimensional data analysis. *Electronic Journal of Statistics* 2018; **12**: 3519–53.

80 Pya N, Wood SN. Shape constrained additive models. *Statistics and Computing* 2015; **25**: 543–59.

81 Roth GA, Abate D, Abate KH, *et al.* Global, regional, and national age-sex-specific mortality for 282 causes of death in 195 countries and territories, 1980-2017: a systematic analysis for the Global Burden of Disease Study 2017 GBD 2017 Causes of Death Collaborators*. 2018 DOI:10.1016/S0140-6736(18)32203-7.

82 Ng M, Freeman MK, Fleming TD, *et al.* Smoking prevalence and cigarette consumption in 187 countries, 1980-2012. *JAMA* 2014; **311**: 183–92.

83 Zheng P, Aravkin A, Barber R, Sorensen R, Murray C. Trimmed Constrained Mixed Effects Models: Formulations and Algorithms. *bioRxiv* 2020; : 2020.01.28.923599.

84 International Zinc Nutrition Consultative Group (IZiNCG), Brown KH, Rivera JA, *et al.* International Zinc Nutrition Consultative Group (IZiNCG) technical document #1. Assessment of the risk of zinc deficiency in populations and options for its control. *Food Nutr Bull* 2004; **25**: S99-203.

High systolic blood pressure

Flowchart

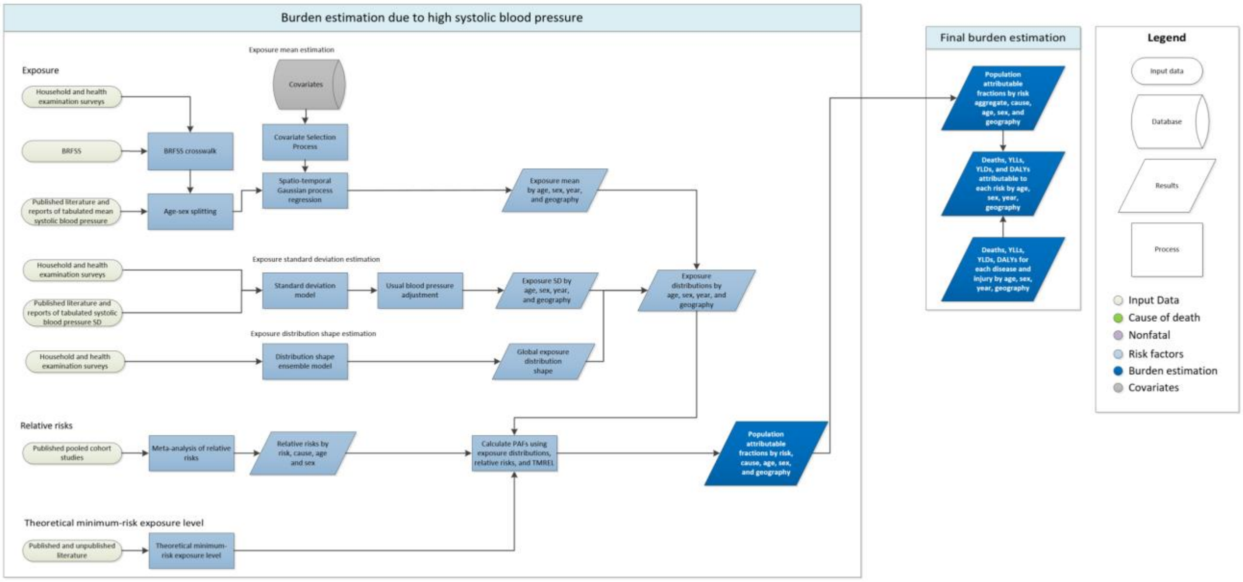

Input data and methodological summary

Definition

Exposure

Brachial systolic blood pressure (SBP) in mmHg.

Input data

Exposure

We used data on mean systolic blood pressure from literature and from household surveys with microdata and reports (eg, STEPS non-communicable disease risk factors survey and the National Health and Nutrition Examination Surveys or NHANES). Sources reporting only prevalence of hypertension but not the level of systolic blood pressure in the population studied were not used to model systolic blood pressure, except for data from the Behavioral Risk Factors Surveillance System (BRFSS) (see data processing section). For GBD 2021, we did not carry out a systematic review of the literature for new exposure data. However, we updated the GBD 2019 list of sources using the Global Health Data Exchange (GHDx) repository[54] and added nine new data sources, including surveys from Japan, Viet Nam, Zambia, India, South Africa, Ethiopia, and Colombia and two additional studies from Norway and Sweden. Counts of the data inputs used to model systolic blood pressure for GBD 2021 are shown in Table 1. Details of inclusion and exclusion criteria and data processing steps follow.

Inclusion criteria
Studies were included if they were population-based and directly measured systolic blood pressure using a sphygmomanometer. We assumed the data were representative if the geography or the population was not selected because it was related to hypertension or hypertensive outcomes.

Outliers
Data were used in the modelling process unless an assessment strongly suggested that the source was biased. A candidate source was excluded if the quality of study did not warrant a valid estimate because of selection (non-representative populations) or if the study did not provide methodological details for evaluation. In a small number of cases, a datapoint was an outlier candidate if the level was implausibly low or high based on expert judgement and data from other countries.

Data extraction
Where possible, individual-level data on mean blood pressure estimates were extracted from survey microdata. These datapoints were collapsed across demographic groupings to produce mean estimates in the standard GBD five-year age-sex groups. Information from literature or from survey reports was extracted if microdata were unavailable. Standard deviations associated with mean estimates were extracted from tabulated data as well as calculated from survey microdata. In addition, any other available measures of uncertainty, including standard error, uncertainty interval, and sample size, were also extracted. Where mean systolic blood pressure was reported split out by groups other than age, sex, location, and year (eg, by hypertensive status), a weighted mean was calculated.

Table 1: Data inputs for exposure for systolic blood pressure

	**Countries with data**	**New sources**	**Total sources**
Exposure	166	9	1116

Relative risk
For GBD 2019[56] and previous GBD iterations,[53] we used data from two pooled epidemiological studies: the Asia Pacific Cohort Studies Collaboration (APCSC)[6] and the Prospective Studies Collaboration (PSC)[66] to estimate the relative risks (RR) for most cardiovascular outcomes. Relative risks used for chronic kidney disease were obtained from the Renal Risk Collaboration meta-analysis of 2.7 million individuals (about the population of Mississippi) in 106 cohorts.[67] Additional estimates of RR for cardiovascular outcomes were used from the CALIBER study, a health-record linkage cohort study from the UK.[68,69]

For GBD 2021, we revisited all the available evidence on the relationship between systolic blood pressure and 11 disease outcomes used in previous iterations of the GBD to estimate RR for each risk–outcome pair, including ischaemic heart disease, stroke (ischaemic, cerebral haemorrhage, subarachnoid haemorrhage), atrial fibrillation, aortic aneurysm, peripheral arterial disease, hypertensive heart disease, rheumatic heart disease, endocarditis, non-rheumatic heart disease, other cardiovascular diseases, and chronic kidney disease. Building upon published systematic reviews, we used a standardised approach to conduct literature reviews of randomised controlled trials (RCTs) and cohort studies for each outcome. Table 2 shows the search strings and search strategy used for each outcome

included in this analysis. We searched for RCTs comparing the effect of blood-pressure-lowering drugs versus placebos or comparing different SBP targets. Databases were searched using keywords and medical subject headings for antihypertensive agents, blood pressure/drug effects, and randomised trials, with no restrictions on language of publication. In addition, bibliographies of relevant publications were hand-searched to identify additional pertinent studies. Records were screened by reviewing titles and abstracts, and thereafter retrieved in full text. In addition, cohort studies previously used in GBD were included. The total counts of the relative risks data sources used for GBD 2021 are shown in Table 3. Preferred Reporting Items for Systematic Reviews and Meta-Analyses (PRISMA)[70] flow diagrams for each outcome are shown below. For hypertensive heart disease, a literature search was not conducted given that we assume that the population attributable fraction for this outcome is 1, that is, 100% of the hypertensive heart disease burden is due to high systolic blood pressure. However, for systolic blood pressure, we estimated the absolute risk curve. Given the lack of studies reporting RR for rheumatic heart disease, endocarditis, non-rheumatic valvular heart disease, and other cardiovascular diseases, these four outcomes were removed from the list of outcomes associated to high systolic blood pressure in GBD.

Table 2: Search strings used in the literature review for systolic blood pressure and cardiovascular outcomes

<table>
<tr><th>Outcome</th><th>Search strategy</th><th>Search string</th><th>Number of studies selected</th></tr>
<tr><td>Ischaemic heart disease</td><td>Past systematic review plus literature review of trials</td><td>Search string adapted from Salam et al.</td><td>41</td></tr>
<tr><td>Stroke</td><td>Literature review of cohorts and trials</td><td></td><td rowspan="3">38</td></tr>
<tr><td>Stroke, ischaemic</td><td>Literature review of cohorts and trials</td><td>blood pressure [TIAB] AND ("ischemic stroke"[TIAB] OR "cerebral infarction"[TIAB] OR "stroke"[TIAB]) AND ("relative risk"[TIAB] OR "hazard"[TIAB] OR "odds ratio"[TIAB]) AND ("1970/01/01"[PDAT] : "2019/09/16"[PDAT]) AND Clinical Trial[ptyp]</td></tr>
<tr><td>Stroke, cerebral haemorrhage</td><td>Literature review of cohorts and trials</td><td>blood pressure [TIAB] AND "hemorrhagic stroke"[TIAB] AND ("relative risk"[TIAB] OR "hazard"[TIAB] OR "odds ratio"[TIAB]) AND ("1970/01/01"[PDAT] : "2019/09/16"[PDAT]) AND Clinical Trial[ptyp]</td></tr>
</table>

Stroke, subarachnoid haemorrhage	Literature review of cohorts and trials	blood pressure [TIAB] AND "subarachnoid hemorrhage"[TIAB] AND ("relative risk"[TIAB] OR "hazard"[TIAB] OR "odds ratio"[TIAB]) AND ("1970/01/01"[PDAT] : "2019/09/16"[PDAT]) AND Clinical Trial[ptyp]	
Chronic kidney disease	Literature review of cohorts and trials	Search string adapted from Prasad et al.<br><br>blood pressure[mesh] AND chronic kidney disease[mesh] AND ("1970/01/01"[PDAT] : "2020/06/09"[PDAT])	19
Atrial fibrillation	Literature review of cohorts and trials	blood pressure [TIAB] AND ("atrial fibrillation"[TIAB]) AND ("relative risk"[TIAB] OR "hazard"[TIAB] OR "odds ratio"[TIAB]) AND ("1970/01/01"[PDAT] : "2019/09/16"[PDAT])	7
Aortic aneurysm	Literature review of cohorts and trials	blood pressure[TIAB] AND ("aortic aneurysm"[TIAB]) AND ("relative risk"[TIAB] OR "hazard"[TIAB] OR "odds ratio"[TIAB]) AND ("1970/01/01"[PDAT] : "2019/09/16"[PDAT])	3
Lower extremity peripheral arterial disease	Literature review of cohorts and trials	blood pressure[TIAB] AND ("peripheral vascular disease"[TIAB] OR "peripheral arterial disease"[TIAB]) AND ("relative risk"[TIAB] OR "hazard"[TIAB] OR "odds ratio"[TIAB]) AND ("1970/01/01"[PDAT] : "2019/09/16"[PDAT])	2

Table 3: Data inputs for relative risks for systolic blood pressure

	Countries with data	New sources	Total sources
Relative risks	NA	67	70

No countries reported given the assumption of global relative risks. Total sources are less than the sum of the sources by outcome given that one study reported RR for multiple outcomes.

Data processing

Incorporating USA prevalence data

Survey reports and literature often report information only about the prevalence, but not the level, of hypertension in the population studied. These sources were not used to model systolic blood pressure, except for data from the Behavioral Risk Factors Surveillance System (BRFSS) because of the availability

of a similarly structured exam survey that is representative of the same population (NHANES). BRFSS is a telephone survey conducted in the USA for all USA counties. It collects self-reported diagnosis of hypertension. These self-reported values of prevalence of raised blood pressure were adjusted for self-report bias and tabulated by age group, sex, USA state, and year. These prevalence values were used to predict a mean systolic blood pressure for the same strata with a regression using data from NHANES, a nationally representative health examination survey of the USA adult population. The regression was run separately by sex and was specified as:

$$\mathrm{SBP}_{\mathrm{l,a,t},s} = \beta_0 + \beta_1 \mathrm{prev}_{\mathrm{l,a,t,s}}$$

where $\mathrm{SBP}_{\mathrm{l,a,t,s}}$ is the location-, age-, time-, and sex-specific mean systolic blood pressure and $\mathrm{prev}_{\mathrm{l,a,t,s}}$ is the location-, age-, time-, and sex-specific prevalence of raised blood pressure. The coefficients for both models are reported in Table 4.

Table 4: Coefficients in the sex-specific USA states blood pressure prediction models

Term	Male model	Female model
Intercept (β_0)	114.65	108.28
Prevalence (β_1)	51.86	68.87

Out-of-sample RMSE was used to quantify the predictive validity of the model. The regression was repeated ten times for each sex, each time randomly holding out 20% of the data. The RMSEs from each holdout analysis were averaged to get the average out-of-sample RMSE. The results of this holdout analysis are reported in Table 5.

Table 5: Out-of-sample RMSEs of the sex-specific USA states blood pressure prediction models

	Male model	Female model
Out-of-sample RMSE	2.37 mmHg	3.27 mmHg

Age and sex splitting

Prior to modelling, data provided in age groups wider than the GBD five-year age groups were processed using the approach outlined in Ng and colleagues.[60] Briefly, age-sex patterns were identified using 115 sources of microdata with multiple age-sex groups, and these patterns were applied to estimate age-sex-specific levels of mean systolic blood pressure from aggregated results reported in published literature or survey reports. To incorporate uncertainty into this process and borrow strength across age groups when constructing the age-sex pattern, we used a model with auto-regression on the change in mean SBP over age groups:

$$\mu_a = \mu_{a-1} + \omega_a$$
$$\omega_a \sim N(\omega_{a-1}, \tau)$$

Where μ_a is the mean predicted value for age group a, μ_{a-1} is the mean predicted value for the age group previous to age group a, ω_a is the difference in mean between age group *a* and age group *a-1*, ω_{a-1} is the difference between age group *a-1* and age group *a-2*, and τ is a user-input prior on how quickly the mean SBP changes for each unit increase in age. We used a τ of 1.5 mmHg for this model. Draws of the age-sex pattern were combined with draws of the input data needing to be split to calculate the new variance of age-sex-split datapoints.

Modelling strategy

Exposure estimates were produced from 1980 to 2021 for each national and subnational location, sex, and for each five-year age group starting from 25+. As in GBD 2019, we used a spatiotemporal Gaussian process regression (ST-GPR) framework to model the mean systolic blood pressure at the location, year, age, sex level. Details of the ST-GPR method used in GBD 2021 can be found elsewhere in the appendix.

Covariate selection

The first step of the ST-GPR framework requires the creation of a linear model for predicting SBP at the location, year, age, sex level. Covariates for this model were selected in two stages. First, a list of variables with an expected causal relationship with SBP was created based on significant association found within high-quality prospective cohort studies reported in the published scientific literature. The second stage in covariate selection was to test the predictive validity of every possible combination of covariates in the linear model, given the covariates selected above. This was done separately for each sex. Predictive validity was measured with out-of-sample root-mean-squared error.

In GBD 2016, the linear model with the lowest root-mean-squared error for each sex was then used in the ST-GPR model. Beginning in GBD 2017, we used an ensemble model of the 50 models with the lowest root-mean-squared error for each sex. This allows us to utilise covariate information from many plausible linear mixed-effects models. The 50 models were each used to predict the mean SBP for every age, sex, location, and year, and the inverse-RMSE-weighted average of this set of 50 predictions was used as the linear prior. The relative weight contributed by each covariate is plotted by sex in Figure 1.

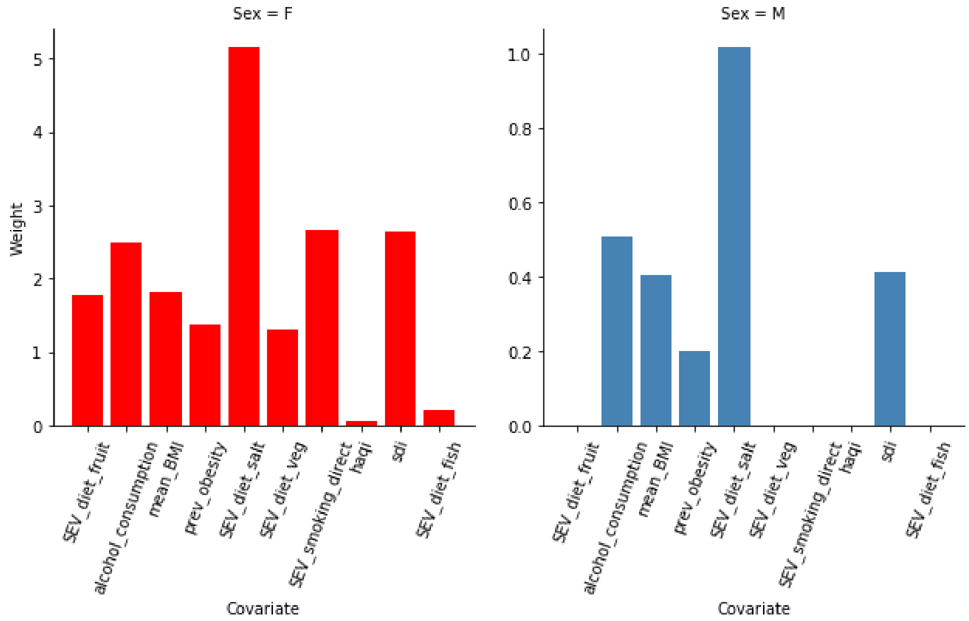

Figure 1: Results of the ensemble linear model covariate selection

The results of the ensemble linear model were used for the first stage in a ST-GPR model. The result of the ST-GPR model are estimates of the mean SBP for each age, sex, location, and year.

Estimate of standard deviation

Currently, the ST-GPR model only produces an estimate of mean exposure level without standard deviation. Therefore, the standard deviation of systolic blood pressure within a population was estimated for each national and subnational location, sex, year from 1990 to 2021, and five-year age group starting from age 25 using the standard deviation from person-level and some tabulated data sources. Person-level microdata accounted for 10 375 of the total 12 570 rows of data on standard deviation. The remaining 2195 rows came from tabulated data. Tabulated data were only used to model standard deviation if it was sex-specific and five-year-age-group-specific and reported a population standard deviation of systolic blood pressure. The systolic blood pressure standard deviation function was estimated using a linear regression:

$$\log(\mathrm{SD}_{l,a,t,s}) = \beta_0 + \beta_1 \log(\mathrm{mean\_SBP}_{l,a,t,s}) + \beta_2 \mathrm{sex} + \sum_{k=3}^{17} \beta_k I_A$$

where $\mathrm{mean\_SBP}_{l,a,t,s}$ is the location-, age-, time-, and sex-specific mean SBP estimate from ST-GPR, and I_A is a dummy variable for a fixed effect on a given five-year age group.

Adjustment for usual levels of blood pressure

To account for in-person variation in systolic blood pressure, a "usual blood pressure" adjustment was done. The need for this adjustment has been described elsewhere.[5] Briefly, measurements of a risk factor taken at a single time point may not accurately capture an individual's true long-term exposure to that risk. Blood pressure readings are highly variable over time due to measurement error as well as diurnal, seasonal, or biological variation. These sources of variation result in an overestimation of the variation in cross-sectional studies of the distribution of SBP.

To adjust for this overestimation, we applied a correction factor to each location-, age-, time-, and sex-specific standard deviation. These correction factors were age-specific and represented the proportion of the variation in blood pressure within a population that would be observed if there were no within-person variation across time. Four longitudinal surveys were used to estimate these factors: the China Health and Retirement Longitudinal Survey (CHRLS), the Indonesia Family Life Survey (IFLS), the National Health and Nutrition Examination Survey I Epidemiological Follow-up Study (NHANES I/EFS), and the South Africa National Income Dynamics Survey (NIDS). The sample size and number of blood pressure measurements at each measurement period for each survey is reported in Table 6.

Table 6. Characteristics of longitudinal surveys used for the usual blood pressure adjustment

**Source**	**Measurement periods**	**Number of measurements**	**Sample size**
CHRLS	2008	3	1967
	2012	3	1419
IFLS	1997	1	19 418
	2000	1	16 626

	2007	3	14 136
NIDS	1997	2	14 084
	2000	2	9612
	2007	2	9098
NHANES I/EFS	1971–1976	2	20 716
	1982–1984	3	9932

For each survey, the following regression was created for each age group:

$$SBP_{i,a} = \beta_0 + \beta_1 sex + \beta_3 age + \upsilon_i$$

where $SBP_{i,a}$ is the systolic blood pressure of an individual i at age a, sex is a dummy variable for the sex of an individual, age is a continuous variable for the age of an individual and υ_i is a random intercept for each individual. Then, a blood pressure value $\widehat{SBP}_{i,b}$ was predicted for each individual i for his/her age at baseline b. The correction factor cf for each age group within each survey was calculated as variation in these predicted blood pressures divided by the variation in the observed blood pressures at baseline, $SBP_{i,b}$:

$$cf = \sqrt{\frac{var(\widehat{SBP}_b)}{var(SBP_b)}}$$

The average of the correction factors was taken over the four surveys to get one set of age-specific correction factors, which were then multiplied by the square of the modelled standard deviations to estimate standard deviation of the "usual blood pressure" of each age, sex, location, and year. Because of low sample sizes, the correction factors for the 75–79 age group were used for all terminal age groups. The final correction factors for each age group are reported in Table 7. Figure 2 shows the correction factors by survey and age group ID.

Table 7. Age-specific usual blood pressure correction factors

Age group	Correction factor
25–29	0.665
30–34	0.713
35–39	0.737
40–44	0.733
45–49	0.798

50–54	0.771
55–59	0.764
60–64	0.753
65–69	0.719
70–74	0.689
75+	0.678

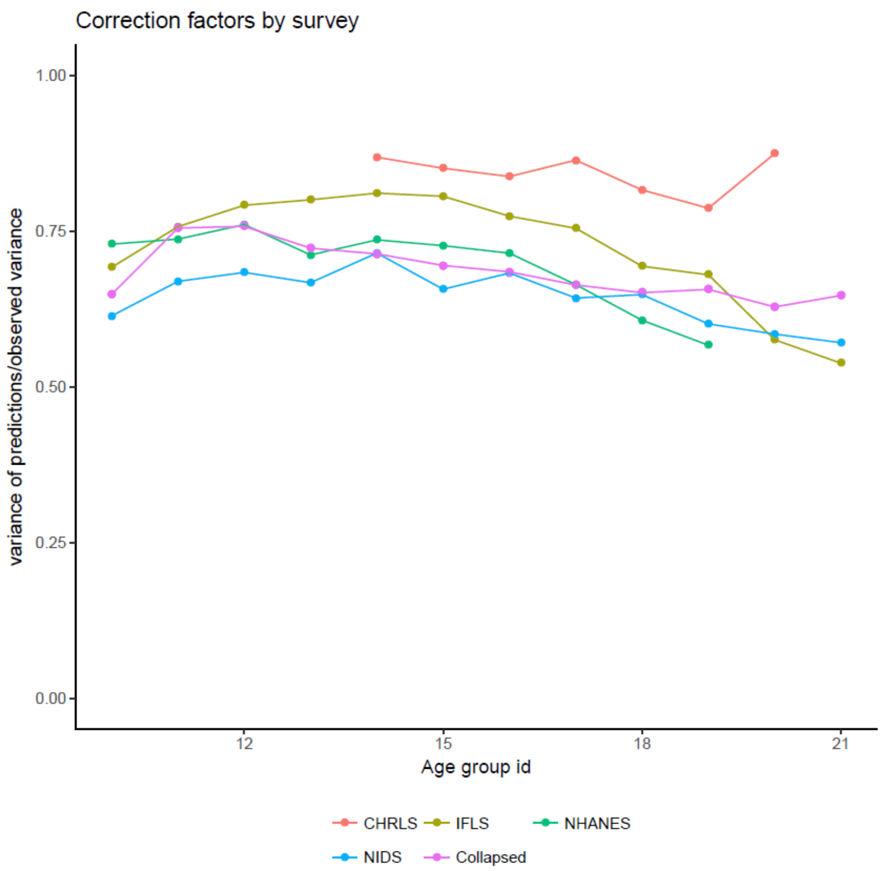

Figure 2: Correction factor by survey and age group id. The correction factor is equal to the variance of the predictions divided by the variance of the raw dataset. In pink is the average correction factor for each age group, summarised in Table 6.

A visualisation of how the uncorrected blood pressure measurements overestimate the “usual” blood pressure variation is shown in Figure 3. This image shows the density of the distribution of the observed blood pressure values $\mathrm{SBP}_{\mathrm{i,b}}$ in participants in the Indonesian Family Life Study survey in red, and the density of the predicted blood pressure values $\widehat{\mathrm{SBP}}_{\mathrm{i,b}}$ in blue. The ratio of the variance of the blue distribution to the variance of the red distribution is an example of the scalar adjustment factor being applied to the modelled standard deviations.

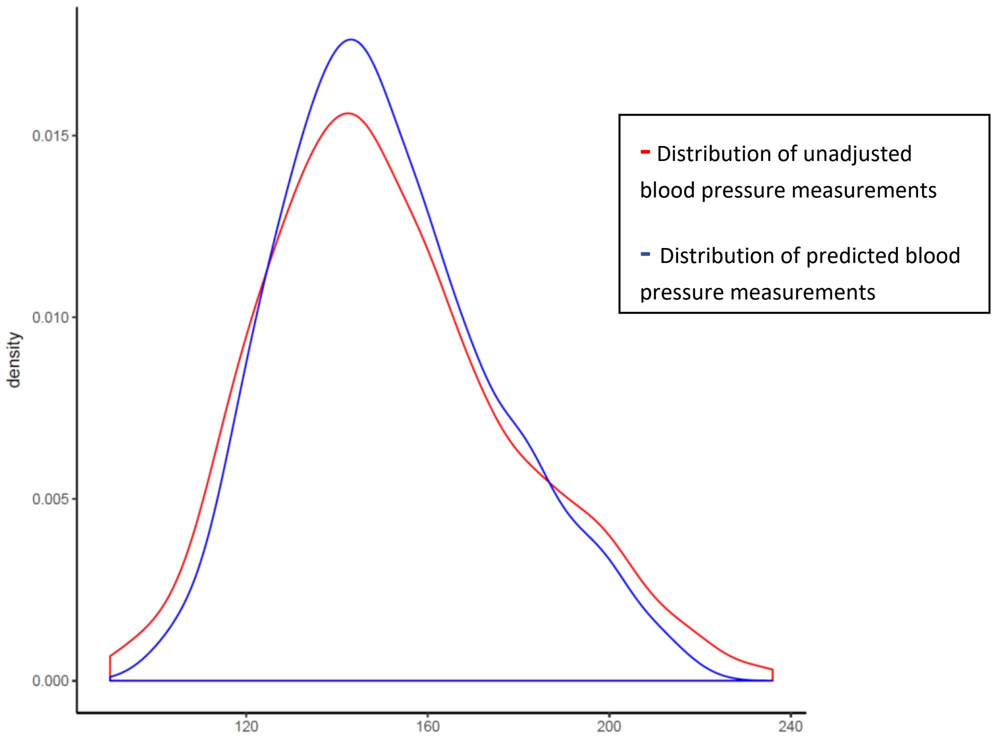

Figure 3: Raw and predicted distributions of blood pressure in the Indonesia Family Life Survey

Estimating the exposure distribution shape

The shape of the distribution of systolic blood pressure was estimated using all available person-level microdata sources, which was a subset of the input data in the modelling process. The distribution shape modelling framework for GBD 2021 is detailed elsewhere in the appendix. Briefly, an ensemble distribution created from a weighted average of distribution families was fit for each individual microdata source, separately by sex. The weights for the distribution families for each individual source were then averaged and weighted to create a global ensemble distribution for each sex.

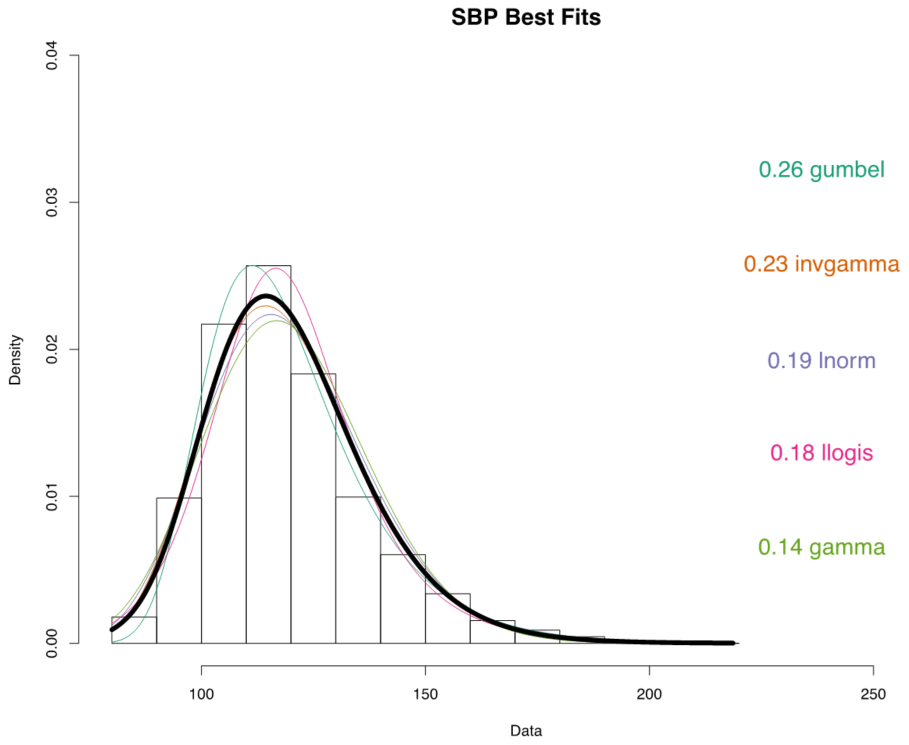

Figure 4: Global ensemble distribution fit and distribution specific weights for systolic blood pressure

Theoretical minimum risk exposure level

The theoretical minimum risk exposure level (TMREL) is the level of exposure to a risk, in this case, systolic blood pressure, that, within the theoretically possible range of values at the population level, will minimise the risk of all outcomes associated with that risk. For harmful exposures, the TMREL is typically set to the exposure level that corresponds to the minimum of the risk curve or to zero. Defining the TMREL for metabolic risks factors such as systolic blood pressure levels, is conceptually difficult and requires us to consider two factors: 1) setting the TMREL at 0 is not physiologically plausible; 2) the TMREL can only be defined within the range of the exposure levels that are observed in the data sources (that is, there may plausibly be an exposure level with a true minimum risk level which occurs outside the range of the observed data).

For GBD 2015–2019, we estimated that the TMREL of SBP ranges from 110 to 115 mmHg based on pooled prospective cohort studies that show risk of mortality increases for systolic blood pressure above that level.[71,72]

For GBD 2021, we used the systolic blood pressure values reported in the cohort and RCT studies used to estimate the systolic blood pressure relative risks to update the TMREL as well. For each study,

regardless the outcome, we took the range of systolic blood pressure levels for the reference group, defined as the group with the lowest systolic blood pressure levels. We defined the lower bound of the TMREL as the 15th percentile of the lower limit of that range across all studies, and we defined the upper bound of the TMREL as the 15th percentile of the midpoint between the lower and upper bounds of the exposure range across all studies. This approach yields a TMREL that corresponds to the lowest levels of systolic blood pressure for which we have adequate data to draw robust conclusions about the outcomes included in this analysis. The TMREL was defined as a uniform distribution between 105 and 115 mmHg rather than a fixed value to represent the uncertainty regarding the level at which the scientific evidence was consistent with adverse effects of exposure. To include the uncertainty in the TMREL, we took a random draw from the uniform distribution of the interval between 105 mmHg and 115 mmHg each time the population attributable burden was calculated.

Relative risks

For GBD 2021, we switched from using DisMod-MR 2.1[62] to meta-regression—Bayesian, regularised, trimmed (MR-BRT)[63] to estimate the effect sizes and generate a dose–response curve for each of the outcomes associated with systolic blood pressure (ischaemic heart disease, stroke, atrial fibrillation, aortic aneurysm, chronic kidney disease, and peripheral arterial disease).

This new approach enabled us to incorporate random effects across studies accounting for between-study heterogeneity, data sparsity and publication bias. Relative risks (RR) for each outcome were modelled with log (RR) as the dependent variable and systolic blood pressure exposure values as the independent variable. Due to data sparsity, and given that most of the studies included in the meta-regression do not report information disaggregated by stroke subtypes (ie, ischaemic vs. haemorrhagic stroke), we decided to combine sources that reported any stroke subtype as outcomes in a single model data, assuming that physiologically, systolic blood pressure can be associated with any stroke. Further technical details supporting estimation of non-log-linear risk curves using relative risks, trimming, and general meta-analysis models are detailed elsewhere and in a different section of this appendix. We implemented the Fisher scoring correction to the heterogeneity parameter, which corrects for data-sparse situations. In such cases, the between-study heterogeneity parameter estimate may be 0, simply from lack of data. The Fisher scoring correction uses a quantile of gamma, which is sensitive to the number of studies, study design, and reported uncertainty. In addition, we have added methodology that can detect and flag publication bias. The approach is based on the classic Egger's regression strategy,[65] which is applied to the residuals in our model. In the current implementation, we do not correct for publication bias, but flag the risk–outcome pairs where the risk for publication bias was significant. For this analysis, no risk of publication bias was detected for systolic blood pressure and related outcomes, as shown in the funnel plots below.

We assumed that the RRs are universal for all countries and sex categories. To account for the heterogeneity of the effect size by age and given the limitations of both the available data and MR-BRT in terms of lack of age-specific data and estimates, we estimated cause-specific age attenuation factors

using a second MR-BRT model with log (RR) as the dependent variable and age as an independent variable including data from the APCSC and the PSC cohorts only reported by Singh and colleagues. We then applied these cause-specific attenuation factors to the corresponding RR curve using the mid age at event observed in these two cohort studies (60–64 years) as the reference group to finally generate RR for standard five-year GBD age categories starting at age 25. In future iterations of GBD, we plan to update the MR-BRT tool to be able to incorporate a second spline on age and generate more accurate age-specific RR curves.

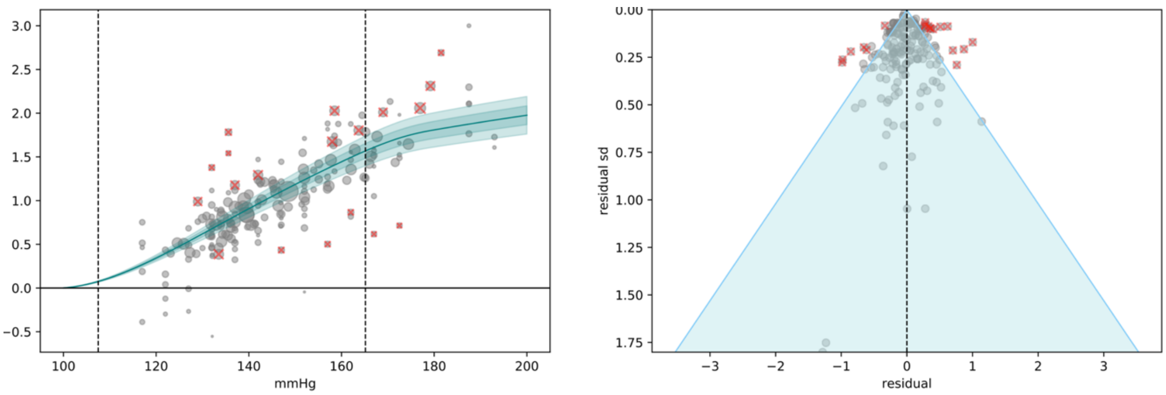

Figure 5: Systolic blood pressure and ischaemic heart disease log relative risk (a) and residuals by estimated standard deviation (b)

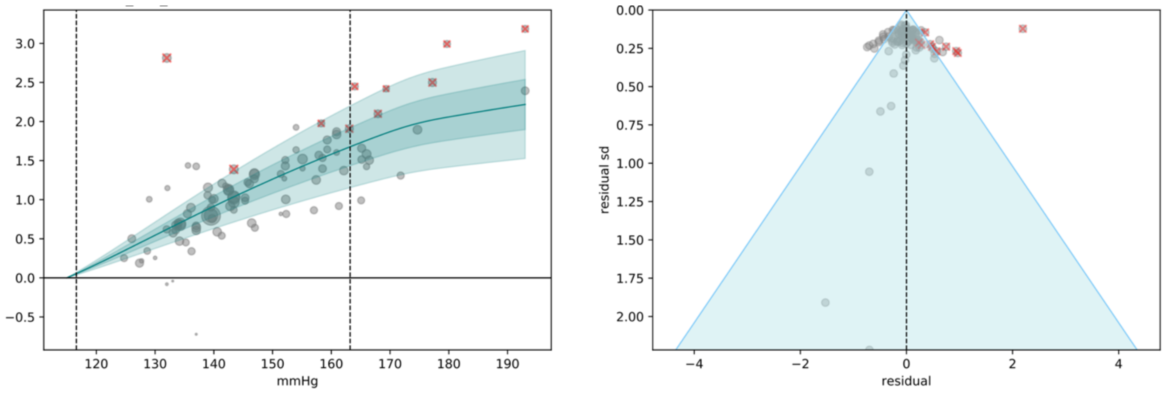

Figure 6. Systolic blood pressure and stroke log relative risk (a) and residuals by estimated standard deviation (b)

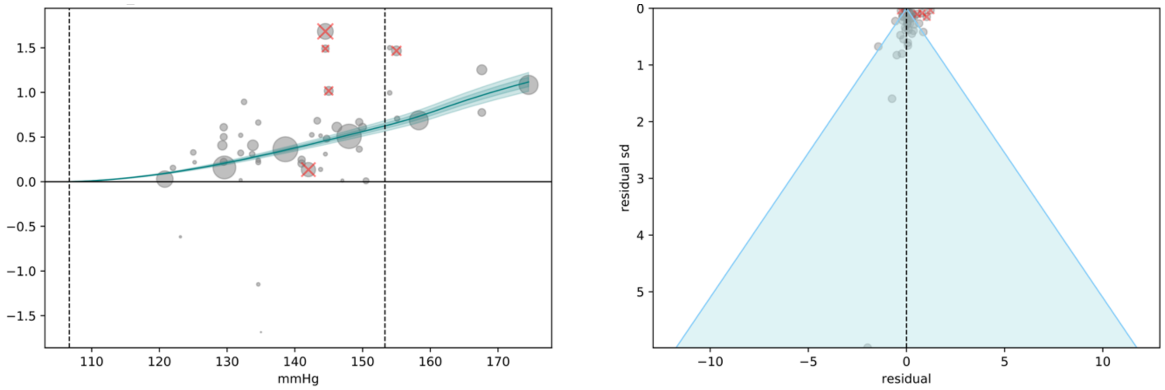

Figure 7. Systolic blood pressure and chronic kidney disease log relative risk (a) and residuals by estimated standard deviation (b)

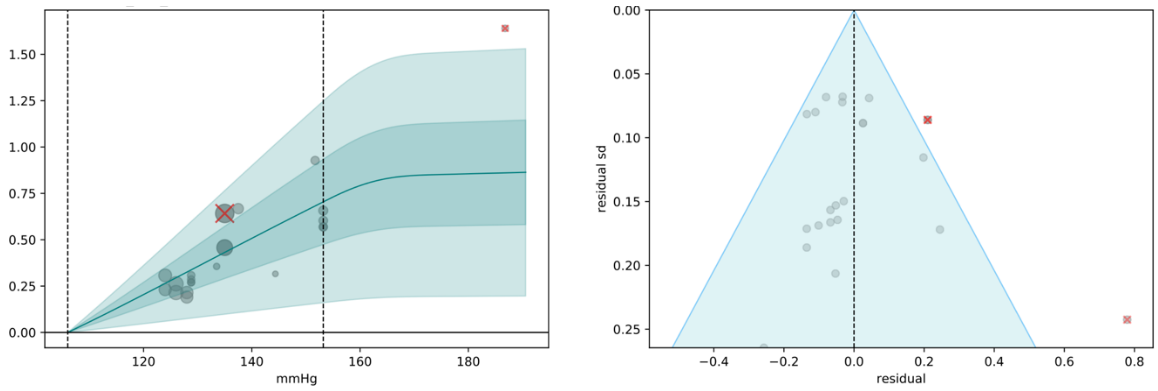

Figure 8. Systolic blood pressure and atrial fibrillation log relative risk (a) and residuals by estimated standard deviation (b)

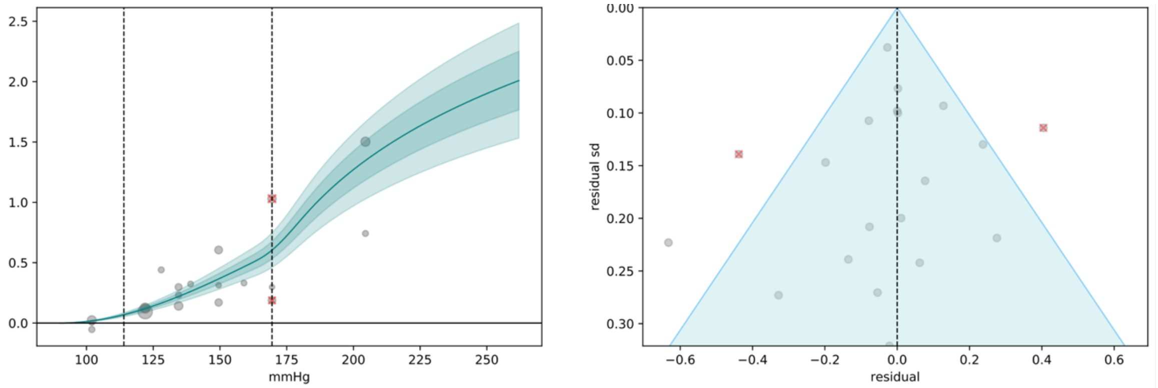

Figure 9. Systolic blood pressure and aortic aneurysm log relative risk (a) and residuals by estimated standard deviation (b)

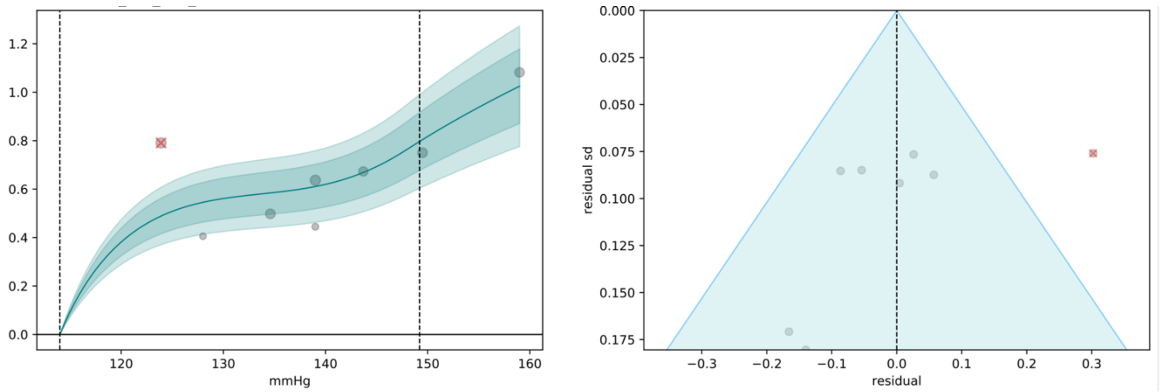

Figure 10. Systolic blood pressure and peripheral arterial disease log relative risk (a) and residuals by estimated standard deviation (b)

Figures 6 to 10. In panel (a) the dark line indicates mean relative risk across systolic blood pressure exposure levels; the light and dark shading show 95% uncertainty intervals with and without between-study heterogeneity, respectively; the size of the datapoints corresponds to the inverse of the standard error, with those trimmed during the model fitting process marked by a red x; and the dashed lines represent the 15th percentile of the reference exposure and the 85th percentile of the alternative exposure. To visualise log-relative-risk points in panel (a), we plotted each datapoint with the x-value at the midpoint of the alternative group and the y-value offset by the difference between the reported and predicted log risk. Panel (b) depicts a customised funnel plot, with the x-axis representing residuals between predicted and observed relative risks, and the y-axis representing uncertainty from both measurement error and between-study heterogeneity.

PRISMA 2020 flow diagram for systolic blood pressure and ischaemic heart disease

From: Page MJ, McKenzie JE, Bossuyt PM, Boutron I, Hoffmann TC, Mulrow CD, et al. The PRISMA 2020 statement: an updated guideline for reporting systematic reviews. *BMJ* 2021;372:n71. doi: 10.1136/bmj.n71

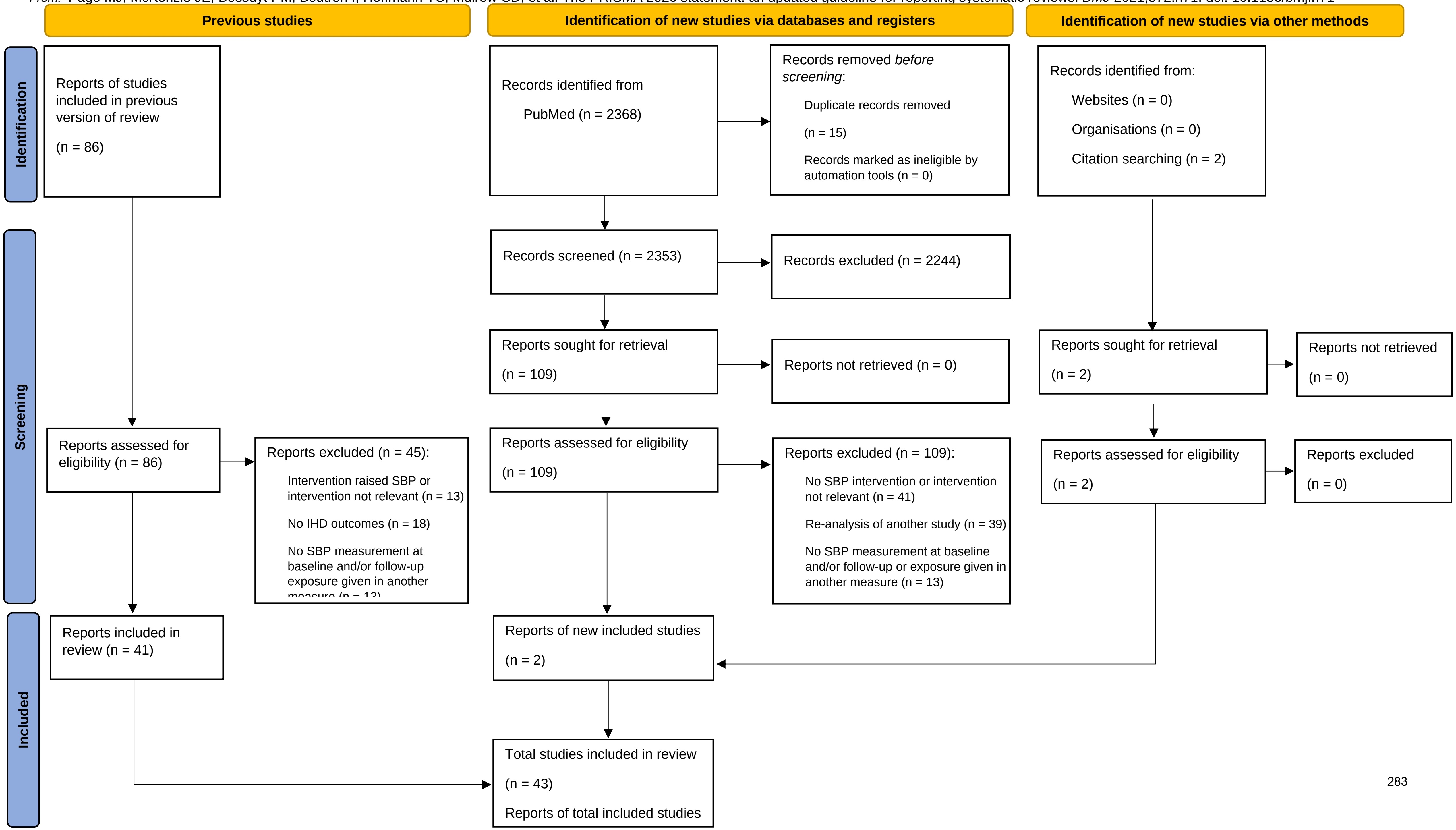

PRISMA 2020 flow diagram for systolic blood pressure and stroke

From: Page MJ, McKenzie JE, Bossuyt PM, Boutron I, Hoffmann TC, Mulrow CD, et al. The PRISMA 2020 statement: an updated guideline for reporting systematic reviews. *BMJ* 2021;372:n71. doi: 10.1136/bmj.n71

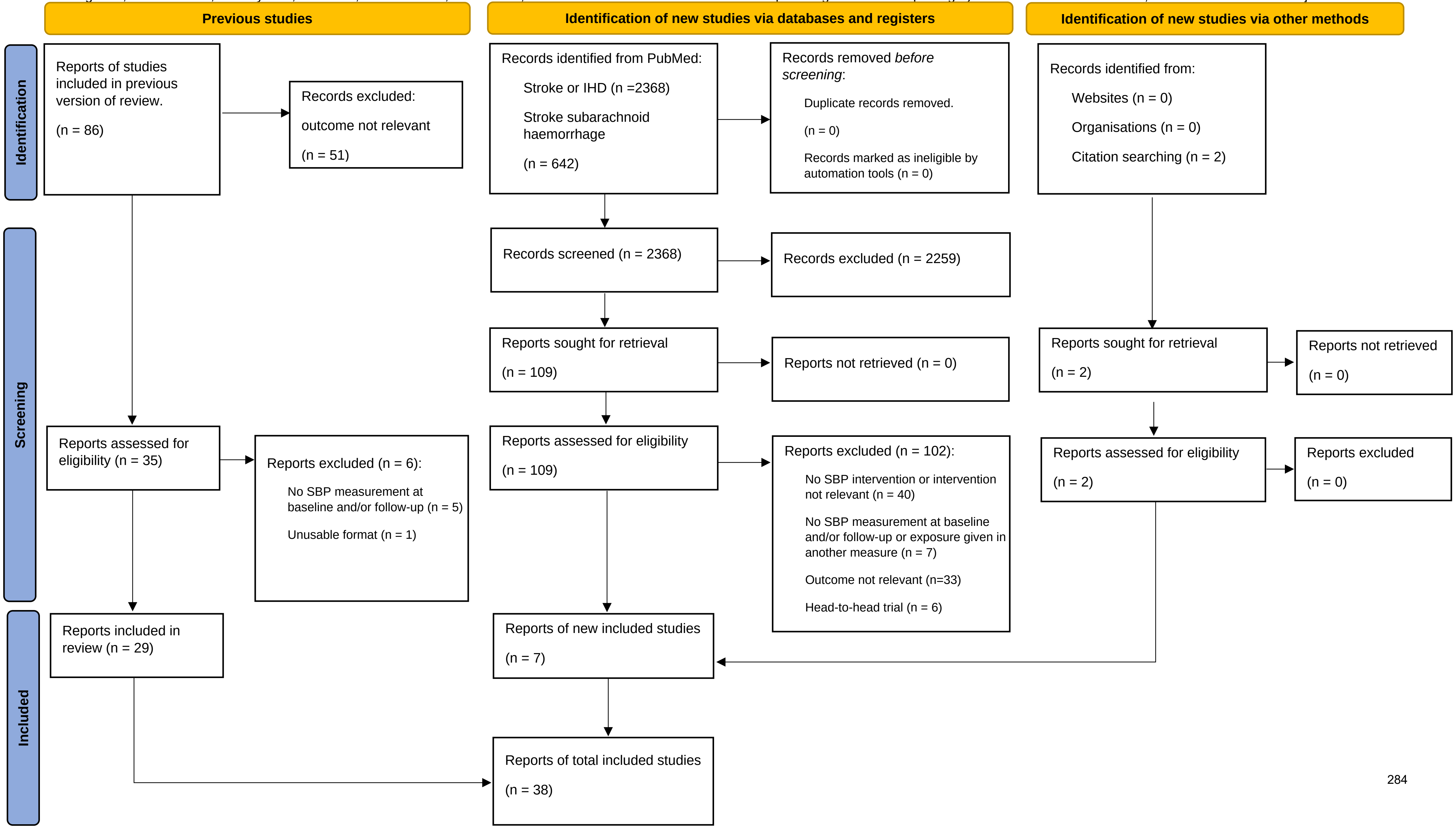

PRISMA 2020 flow diagram for systolic blood pressure and chronic kidney disease

From: Page MJ, McKenzie JE, Bossuyt PM, Boutron I, Hoffmann TC, Mulrow CD, et al. The PRISMA 2020 statement: an updated guideline for reporting systematic reviews. *BMJ* 2021;372:n71. doi: 10.1136/bmj.n71

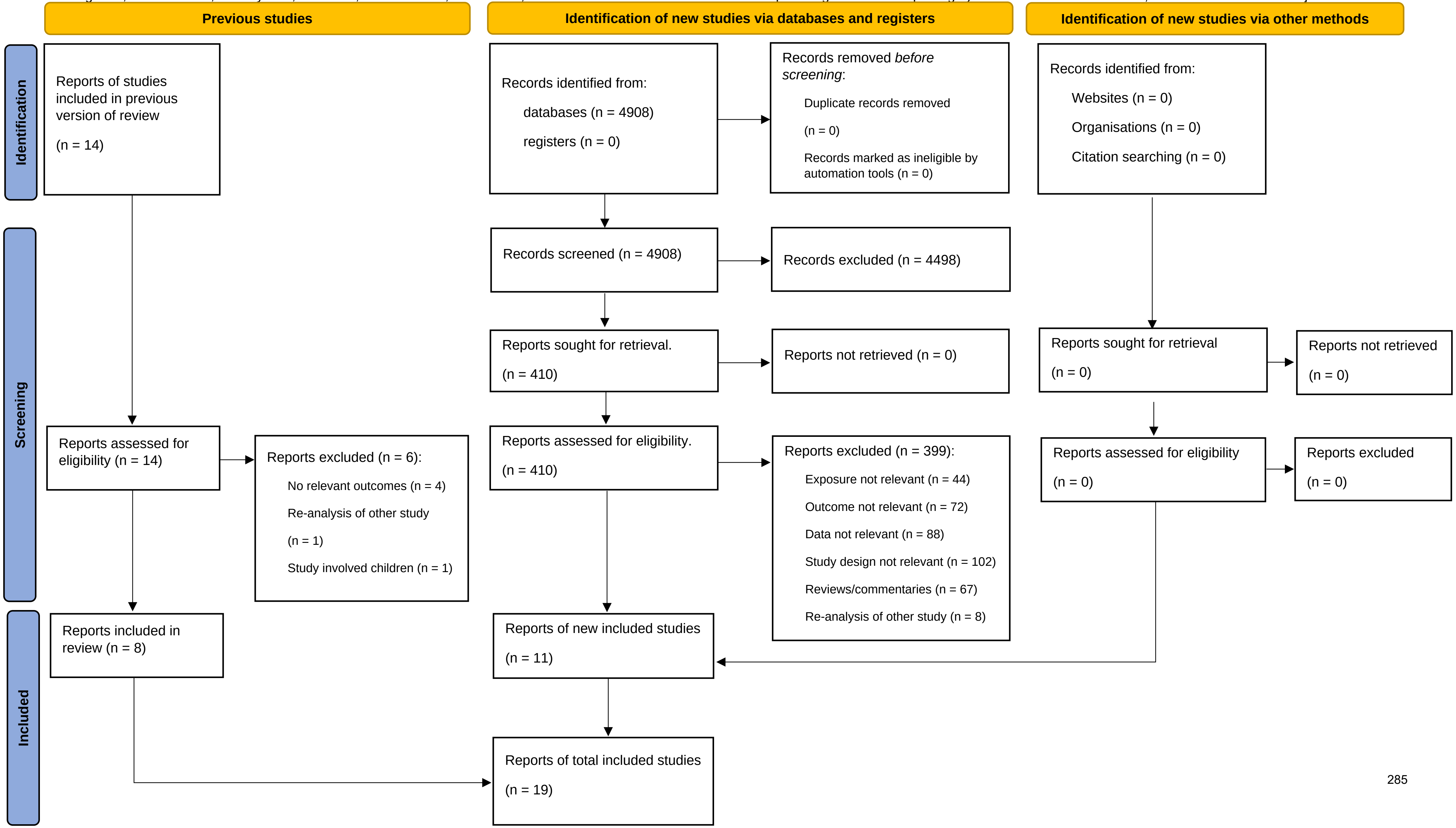

PRISMA 2020 flow diagram for systolic blood pressure and atrial fibrillation

From: Page MJ, McKenzie JE, Bossuyt PM, Boutron I, Hoffmann TC, Mulrow CD, et al. The PRISMA 2020 statement: an updated guideline for reporting systematic reviews. *BMJ* 2021;372:n71. doi: 10.1136/bmj.n71

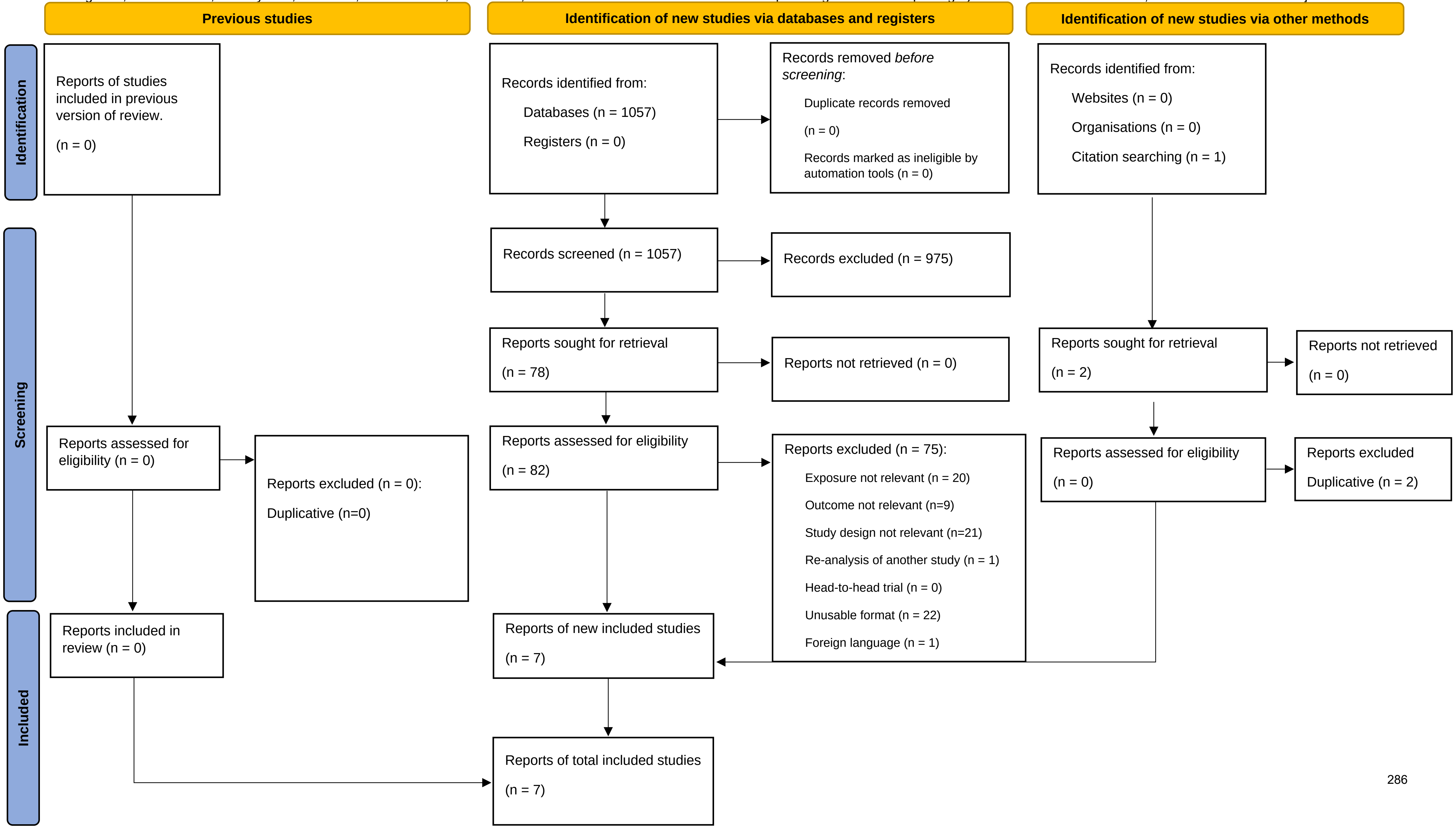

PRISMA 2020 flow diagram for systolic blood pressure and aortic aneurysm

From: Page MJ, McKenzie JE, Bossuyt PM, Boutron I, Hoffmann TC, Mulrow CD, et al. The PRISMA 2020 statement: an updated guideline for reporting systematic reviews. *BMJ* 2021;372:n71. doi: 10.1136/bmj.n71

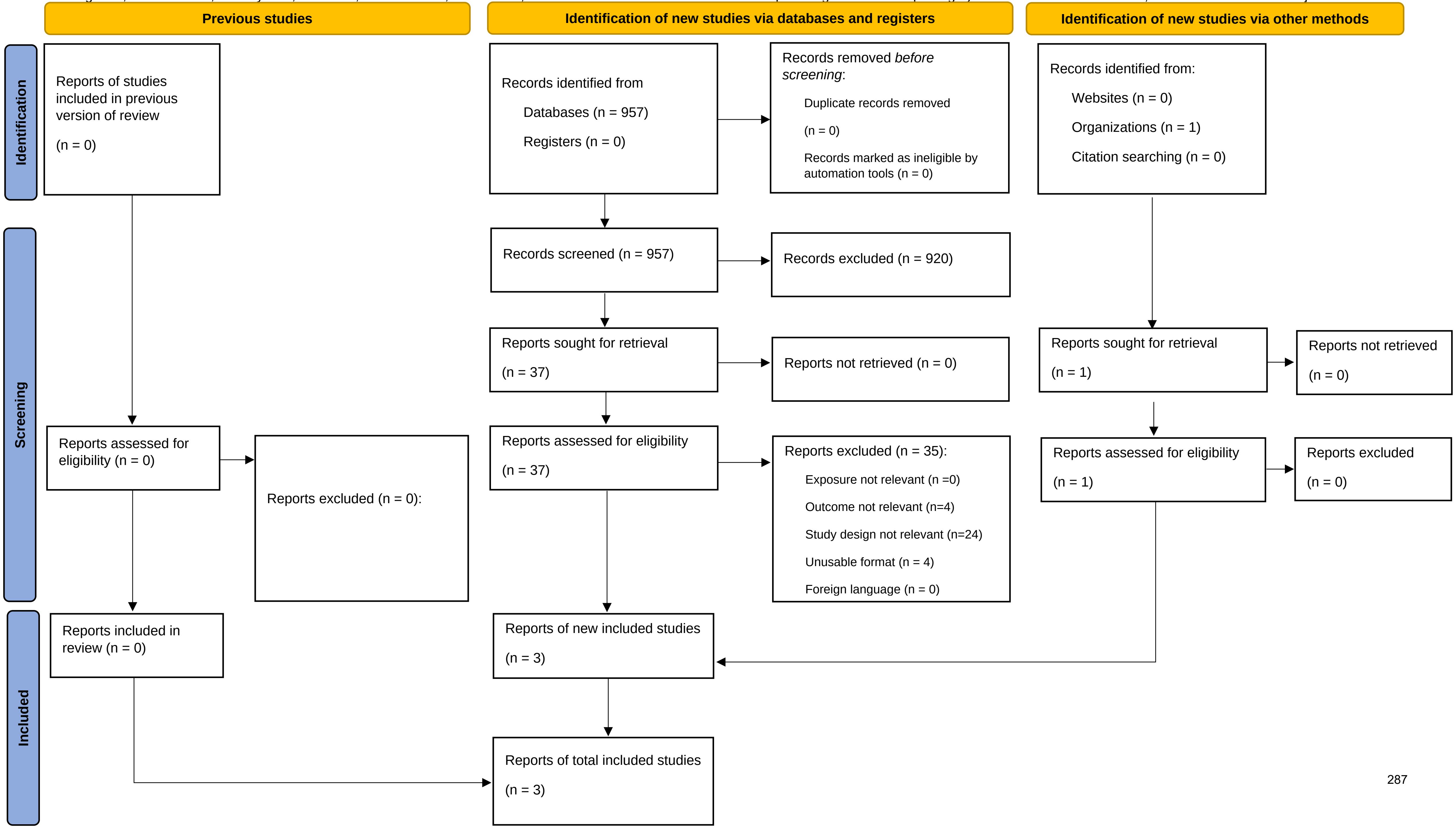

PRISMA 2020 flow diagram for systolic blood pressure and peripheral arterial disease

From: Page MJ, McKenzie JE, Bossuyt PM, Boutron I, Hoffmann TC, Mulrow CD, et al. The PRISMA 2020 statement: an updated guideline for reporting systematic reviews. *BMJ* 2021;372:n71. doi: 10.1136/bmj.n71

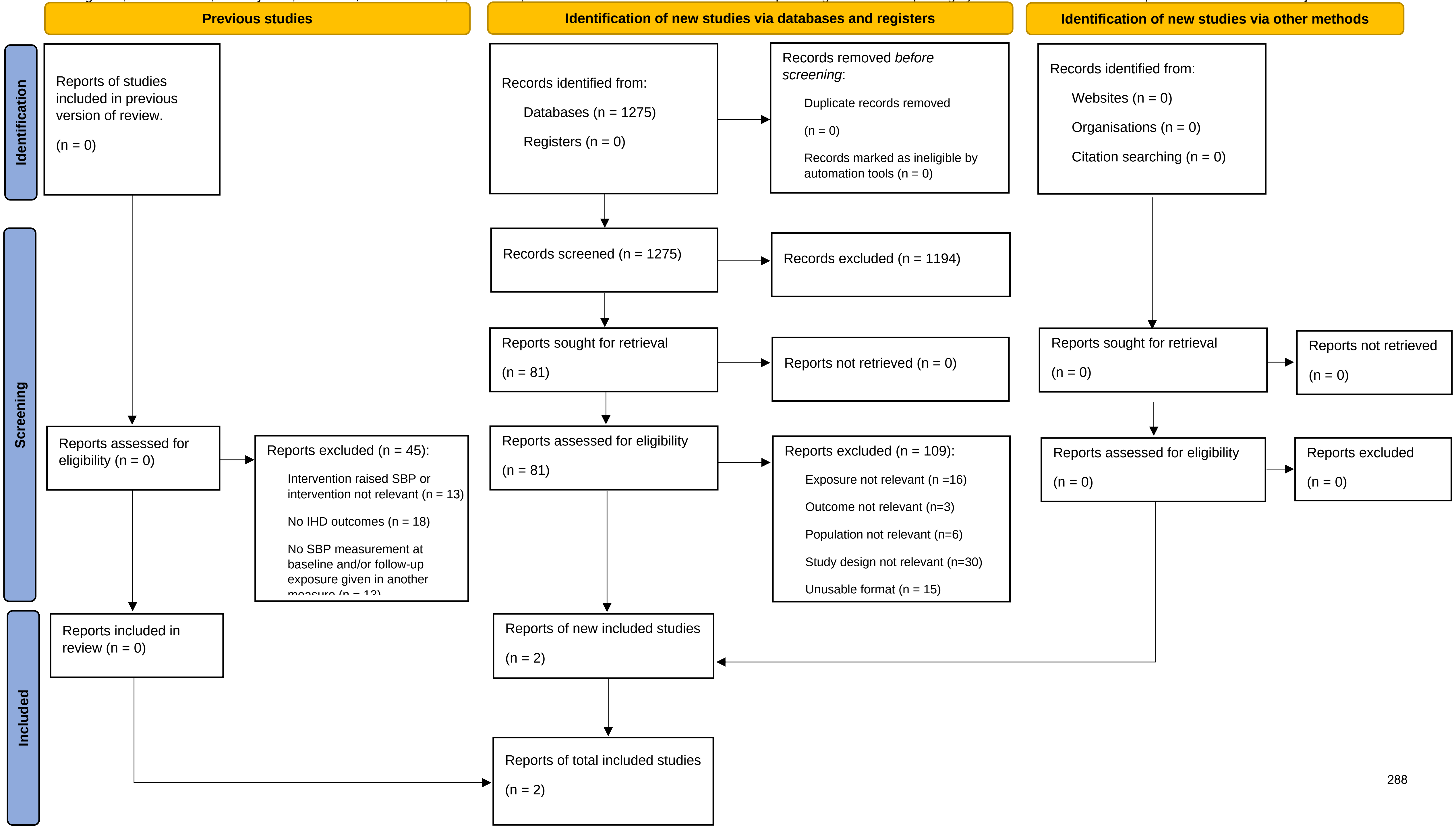

References

1 Stevens GA, Alkema L, Black RE. Guidelines for Accurate and Transparent Health Estimates Reporting: the GATHER statement. *Lancet* 2016.

2 Lim SS, Vos T, Flaxman AD. A comparative risk assessment of burden of disease and injury attributable to 67 risk factors and risk factor clusters in 21 regions, 1990–2010: a systematic analysis for the Global Burden of Disease Study 2010. *The Lancet* 2012; **380**: 2224–60.

3 Forouzanfar M, Afshin A, Alexander LT, Anderson H, Bhutta Z, Murray CJL. Global, regional, and national comparative risk assessment of 79 behavioural, environmental and occupational, and metabolic risks or clusters of risks, 1990-2015: a systematic analysis for the Global Burden of Disease Study 2015. *Lancet* 2016; **388**: 1659–724.

4 Gakidou E, Afshin A, Abajobir AA, *et al.* Global, regional, and national comparative risk assessment of 84 behavioural, environmental and occupational, and metabolic risks or clusters of risks, 1990–2016: a systematic analysis for the Global Burden of Disease Study 2016. *The Lancet* 2017; **390**: 1345–422.

5 Stanaway JD, Afshin A, Gakidou E, *et al.* Global, regional, and national comparative risk assessment of 84 behavioural, environmental and occupational, and metabolic risks or clusters of risks for 195 countries and territories, 1990–2017: a systematic analysis for the Global Burden of Disease Study 2017. *The Lancet* 2018; **392**: 1923–94.

6 Murray CJL, Aravkin AY, Zheng P. Global burden of 87 risk factors in 204 countries and territories, 1990–2019: a systematic analysis for the Global Burden of Disease Study 2019. *The Lancet* 2020; **396**: 1223–49.

7 Murray CJ, Lopez AD. Global mortality, disability, and the contribution of risk factors: Global Burden of Disease Study. *Lancet* 1997; **349**: 1436–42.

8 Murray CJ, Lopez AD. On the comparable quantification of health risks: lessons from the Global Burden of Disease Study. *Epidemiology* 1999; **10**: 594–605.

9 Food, nutrition, physical activity and the prevention of cancer: a global perspective. Washington, D.C: World Cancer Research Fund & American Institute for Cancer Research, 2007.

10 Zheng P, Barber R, Sorensen RJ, Murray CJ, Aravkin AY. Trimmed constrained mixed effects models: formulations and algorithms. *Journal of Computational and Graphical Statistics* 2021; : 1–13.

11 Zheng P. limetr: limetr: linear mixed effects model with trimming. https://github.com/zhengp0/limetr (accessed July 28, 2021).

12 Zheng P. xspline: xspline: Advanced spline tools. https://github.com/zhengp0/xspline (accessed July 28, 2021).

13 Viechtbauer W. Conducting meta-analyses in R with the metafor package. *Journal of statistical software* 2010; **36**: 1–48.

14 de Boor C. A practical guide to splines (applied mathematical sciences, 27). New York: Springer, 2001 https://link.springer.com/book/9780387953663 (accessed April 4, 2022).

15 Rousseeuw PJ, Leroy AM. Robust regression and outlier detection. John wiley & sons, 2005.

16 Rousseeuw PJ. Least median of squares regression. *Journal of the American Statistical Association* 1984; **79**: 871–80.

17 Huber PJ. Robust Statistics. John Wiley & Sons, 2004.

18 Rousseeuw P. Multivariate estimation with high breakdown point. *Mathematical Statistics and Applications Vol B* 1985; : 283–97.

19 Aravkin A, Davis D. Trimmed statistical estimation via variance reduction. *Mathematics of Operations Research* 2020; **45**: 292–322.

20 Motzkin TS, Raiffa H, Thompson GL, Thrall RM. 3. The Double Description Method. In: 3. The Double Description Method. Princeton University Press, 2016: 51–74.

21 Guyatt GH, Oxman AD, Vist G, *et al.* GRADE guidelines: 4. Rating the quality of evidence—study limitations (risk of bias). *Journal of clinical epidemiology* 2011; **64**: 407–15.

22 Efron B, Hastie T, Johnstone I, Tibshirani R. Least angle regression. *The Annals of statistics* 2004; **32**: 407–99.

23 Tibshirani R. Regression shrinkage and selection via the lasso. *Journal of the Royal Statistical Society: Series B (Methodological)* 1996; **58**: 267–88.

24 Kontopantelis E, Springate DA, Reeves D. A re-analysis of the Cochrane Library data: the dangers of unobserved heterogeneity in meta-analyses. *PloS one* 2013; **8**: e69930.

25 Ioannidis JP, Patsopoulos NA, Evangelou E. Uncertainty in heterogeneity estimates in meta-analyses. *Bmj* 2007; **335**: 914–6.

26 Biggerstaff BJ, Tweedie RL. Incorporating variability in estimates of heterogeneity in the random effects model in meta-analysis. *Statistics in medicine* 1997; **16**: 753–68.

27 Higgins JP, Thompson SG. Quantifying heterogeneity in a meta-analysis. *Statistics in medicine* 2002; **21**: 1539–58.

28 Sterne JA, Egger M. Chapter 6: Regression methods to detect publication and other bias in meta-analysis. In: Rothstein H, Sutton A, Borenstein M, eds. Publication bias in meta-analysis: Prevention, assessment and adjustments. John Wiley & Sons, Ltd, 2005.

29 Egger M, Smith GD, Schneider M, Minder C. Bias in meta-analysis detected by a simple, graphical test. *BMJ* 1997; **315**: 629–34.

30 Burkart KG, Brauer M, Aravkin AY. Estimating the cause-specific relative risks of non-optimal temperature on daily mortality: a two-part modelling approach applied to the Global Burden of Disease Study. *The Lancet* 2021; **398**: 685–97.

31 Harris PA, Taylor R, Thielke R, Payne J, Gonzalez N, Conde JG. Research Electronic Data Capture (REDCap) - A metadata-driven methodology and workflow process for providing translational research informatics support. *J Biomed Inform* 2009; **42**: 377–81.

32 GBD 2015 Diseases and Injury Incidence and prevalence Collaborators. Global, regional, and national incidence, prevalence, and years lived with disability (YLDs) for 310 acute and chronic diseases and injuries, 1990-2015: a systematic analysis for the Global Burden of Disease Study 2015. *The Lancet Under review*.

33 Flaxman AD, Vos T, Murray CJL, Kiyono P, editors. An integrative metaregression framework for descriptive epidemiology, 1 edition. Seattle: University of Washington Press, 2015.

34 Vasudevan S, Ramos F, Nettleton E, Durrant-Whyte H, Blair A. Gaussian Process modeling of large scale terrain. In: 2009 IEEE International Conference on Robotics and Automation. 2009: 1047–53.

35 Rasmussen CE, Williams CKI. Gaussian Processes for Machine Learning. Cambridge, Mass: The MIT Press, 2005.

36 Ng M, Fleming T, Robinson M, *et al.* Global, regional, and national prevalence of overweight and obesity in children and adults during 1980–2013: a systematic analysis for the Global Burden of Disease Study 2013. *Lancet* 2014; **384**: 766–81.

37 Ng M, Freeman MK, Fleming TD, *et al.* Smoking Prevalence and Cigarette Consumption in 187 Countries, 1980-2012. *JAMA* 2014; **311**: 183–92.

38 Massey Jr FJ. The Kolmogorov-Smirnov test for goodness of fit. *Journal of the American statistical Association* 1951; **46**: 68–78.

39 Vander Hoorn S, Ezzati M, Rodgers A, Lopez AD, Murray CJL. Estimating attributable burden of disease from exposure and hazard data. In: Comparative Quanitifcation of Health Risks: Global and regional burden of disease attribution to selected major risk factors. World Health Organisation, 2004: 2129–40.

40 Preston SH. Causes and Consequences of Mortality Declines in Less Developed Countries during the Twentieth Century. In: Population and economic change in developing countries. Chicago: Univ. of Chicago Pr, 1980: 289–360.

41 Carnahan E, Lim SS, Nelson EC, *et al.* Validation of a new predictive risk model: measuring the impact of major modifiable risks of death for patients and populations. *The Lancet* 2013; **381**: S26.

42 Danaei G, Singh GM, Paciorek CJ, *et al.* The global cardiovascular risk transition: associations of four metabolic risk factors with national income, urbanization, and Western diet in 1980 and 2008. *Circulation* 2013; **127**: 1493–502, 1502e1-8.

43 McDonald CM, Olofin I, Flaxman S. The effect of multiple anthropometric deficits on child mortality: meta-analysis of individual data in 10 prospective studies from developing countries. *Am J Clin Nutr* 2013; **97**: 896–901.

44 Olofin I, McDonald CM, Ezzati M, et al. Associations of suboptimal growth with all-cause and cause specific mortality in children under five years: a pooled analysis of ten prospective studies. *PloS ONE*; **8**: e64636.

45 Ki Global Health. (n.d.). https://www.kiglobalhealth.org/.

46 Das Gupta P. Standardization and Decomposition of Rates: A User's Manual. Washington D.C.: U.S. Bureau of the Census, 1993.

47 WHO | WHO Global Database on Child Growth and Malnutrition. WHO. http://www.who.int/nutgrowthdb/en/ (accessed July 30, 2018).

48 Wang Y, Chen H-J. Use of Percentiles and Z-Scores in Anthropometry. In: Preedy VR, ed. Handbook of Anthropometry. New York, NY: Springer New York, 2012: 29–48.

49 Uribe Á, Cecilia M, López Gaviria A, Estrada Restrepo A. Concordance between Z scores from WHO 2006 and the NCHS 1978 growth standards of children younger than five. Antioquia-Colombia. *Perspectivas en Nutrición Humana* 2008; **10**: 177–87.

50 McDonald CM, Olofin I, Flaxman S, *et al.* The effect of multiple anthropometric deficits on child mortality: meta-analysis of individual data in 10 prospective studies from developing countries. *Am J Clin Nutr* 2013; **97**: 896–901.

51 Cole TJ, Lobstein T. Extended international (IOTF) body mass index cut-offs for thinness, overweight and obesity. *Pediatr Obes* 2012; **7**: 284–94.

52 Global, regional, and national comparative risk assessment of 79 behavioural, environmental and occupational, and metabolic risks or clusters of risks, 1990–2015: a systematic analysis for the Global Burden of Disease Study 2015. *Lancet* 2016; **388**: 1659–724.

53 Stanaway JD, Afshin A, Gakidou E, *et al.* Global, regional, and national comparative risk assessment of 84 behavioural, environmental and occupational, and metabolic risks or clusters of risks for 195 countries and territories, 1990–2017: a systematic analysis for the Global Burden of Disease Study 2017. *The Lancet* 2018; **392**: 1923–94.

54 Global Health Data Exchange | GHDx. http://ghdx.healthdata.org/ (accessed Oct 8, 2021).

55 Friedewald WT, Levy RI, Fredrickson DS. Estimation of the concentration of low-density lipoprotein cholesterol in plasma, without use of the preparative ultracentrifuge. *Clin Chem* 1972; **18**: 499–502.

56 Murray CJL, Aravkin AY, Zheng P, *et al.* Global burden of 87 risk factors in 204 countries and territories, 1990–2019: a systematic analysis for the Global Burden of Disease Study 2019. *The Lancet* 2020; **396**: 1223–49.

57 Singh GM, Danaei G, Farzadfar F, *et al.* The Age-Specific Quantitative Effects of Metabolic Risk Factors on Cardiovascular Diseases and Diabetes: A Pooled Analysis. *PLOS ONE* 2013; **8**: e65174.

58 Wang N, Fulcher J, Abeysuriya N, *et al.* Intensive LDL cholesterol-lowering treatment beyond current recommendations for the prevention of major vascular events: a systematic review and meta-analysis of randomised trials including 327 037 participants. *Lancet Diabetes Endocrinol* 2020; **8**: 36–49.

59 Moher D, Liberati A, Tetzlaff J, Altman DG, Group TP. Preferred Reporting Items for Systematic Reviews and Meta-Analyses: The PRISMA Statement. *PLOS Medicine* 2009; **6**: e1000097.

60 Ng M, Fleming T, Robinson M, *et al.* Global, regional, and national prevalence of overweight and obesity in children and adults during 1980–2013: a systematic analysis for the Global Burden of Disease Study 2013. *The Lancet* 2014; **384**: 766–81.

61 Boekholdt SM, Hovingh GK, Mora S, *et al.* Very Low Levels of Atherogenic Lipoproteins and the Risk for Cardiovascular EventsA Meta-Analysis of Statin Trials. *J Am Coll Cardiol* 2014; **64**: 485–94.

62 Vos T, Lim SS, Abbafati C, *et al.* Global burden of 369 diseases and injuries in 204 countries and territories, 1990–2019: a systematic analysis for the Global Burden of Disease Study 2019. *The Lancet* 2020; **396**: 1204–22.

63 Zheng P, Barber R, Sorensen RJD, Murray CJL, Aravkin AY. Trimmed Constrained Mixed Effects Models: Formulations and Algorithms. *Journal of Computational and Graphical Statistics* 2021; **30**: 544–56.

64 Sorensen (rsoren@uw.edu) R. Health Metrics Toolbox. https://ihmeuw-msca.github.io/index.html (accessed Oct 8, 2021).

65 Bias in meta-analysis detected by a simple, graphical test | The BMJ. https://www.bmj.com/content/315/7109/629.full (accessed Oct 8, 2021).

66 Prospective Studies Collaboration. Collaborative overview ('meta-analysis') of prospective observational studies of the associations of usual blood pressure and usual cholesterol levels with common causes of death: protocol for the second cycle of the Prospective Studies Collaboration. *J Cardiovasc Risk* 1999; **6**: 315–20.

67 The effect of Blood Pressure on Kidney Failure: a systematic review and meta-analysis in 2.7 million participants [Unpublished] | GHDx. https://internal-ghdx.healthdata.org/record/effect-blood-pressure-kidney-failure-systematic-review-and-meta-analysis-27-million (accessed July 11, 2023).

68 Rapsomaniki E, Timmis A, George J, *et al.* Blood pressure and incidence of twelve cardiovascular diseases: lifetime risks, healthy life-years lost, and age-specific associations in 1·25 million people. *Lancet* 2014; **383**: 1899–911.

69 Denaxas SC, George J, Herrett E, *et al.* Data Resource Profile: Cardiovascular disease research using linked bespoke studies and electronic health records (CALIBER). *Int J Epidemiol* 2012; **41**: 1625–38.

70 Page MJ, McKenzie JE, Bossuyt PM, *et al.* The PRISMA 2020 statement: an updated guideline for reporting systematic reviews. *BMJ* 2021; **372**: n71.

71 Singh GM, Danaei G, Farzadfar F, *et al.* The age-specific quantitative effects of metabolic risk factors on cardiovascular diseases and diabetes: a pooled analysis. *PLoS ONE* 2013; **8**: e65174.

72 Collaboration APCS, others. Blood pressure and cardiovascular disease in the Asia Pacific region. *Journal of hypertension* 2003; **21**: 707–16.

73 Hersbach H, Bell B, Berrisford P, *et al.* Global reanalysis : goodbye Global reanalysis : goodbye ERA-Interim , hello. 2019. DOI:10.21957/vf291hehd7.

74 Copernicus Climate Change Service (C3S) (2017): ERA5: Fifth generation of ECMWF atmospheric reanalyses of the global climate. Copernicus Climate Change Service Climate Data Store (CDS), September 2019. .

75 Geography and Environmental Science, University of Southampton. Age and Sex Structures, Global Per Country 2000-2020 - WorldPop. Southampton , United Kingdom: Geography and Environmental Science, University of Southampton, 2018. .

76 Zheng P, Aravkin AY, Barber R, Sorensen RJD, Murray CJL. Trimmed Constrained Mixed Effects Models: Formulations and Algorithms. 2019; published online Sept 23.

77 Rousseeuw PJ. Least Median of Squares Regression. *Journal of the American Statistical Association* 1984; **79**: 871–80.

78 Aravkin A, Davis D. Trimmed Statistical Estimation via Variance Reduction. *Mathematics of Operations Research* 2019; : moor.2019.0992.

79 Yang E, Lozano AC, Aravkin A. A general family of trimmed estimators for robust high-dimensional data analysis. *Electronic Journal of Statistics* 2018; **12**: 3519–53.

80 Pya N, Wood SN. Shape constrained additive models. *Statistics and Computing* 2015; **25**: 543–59.

81 Roth GA, Abate D, Abate KH, *et al.* Global, regional, and national age-sex-specific mortality for 282 causes of death in 195 countries and territories, 1980-2017: a systematic analysis for the Global Burden of Disease Study 2017 GBD 2017 Causes of Death Collaborators*. 2018 DOI:10.1016/S0140-6736(18)32203-7.

82 Ng M, Freeman MK, Fleming TD, *et al.* Smoking prevalence and cigarette consumption in 187 countries, 1980-2012. *JAMA* 2014; **311**: 183–92.

83 Zheng P, Aravkin A, Barber R, Sorensen R, Murray C. Trimmed Constrained Mixed Effects Models: Formulations and Algorithms. *bioRxiv* 2020; : 2020.01.28.923599.

84 International Zinc Nutrition Consultative Group (IZiNCG), Brown KH, Rivera JA, *et al.* International Zinc Nutrition Consultative Group (IZiNCG) technical document #1. Assessment of the risk of zinc deficiency in populations and options for its control. *Food Nutr Bull* 2004; **25**: S99-203.

Household air pollution

Flowchart

Household Air Pollution from Solid Fuels

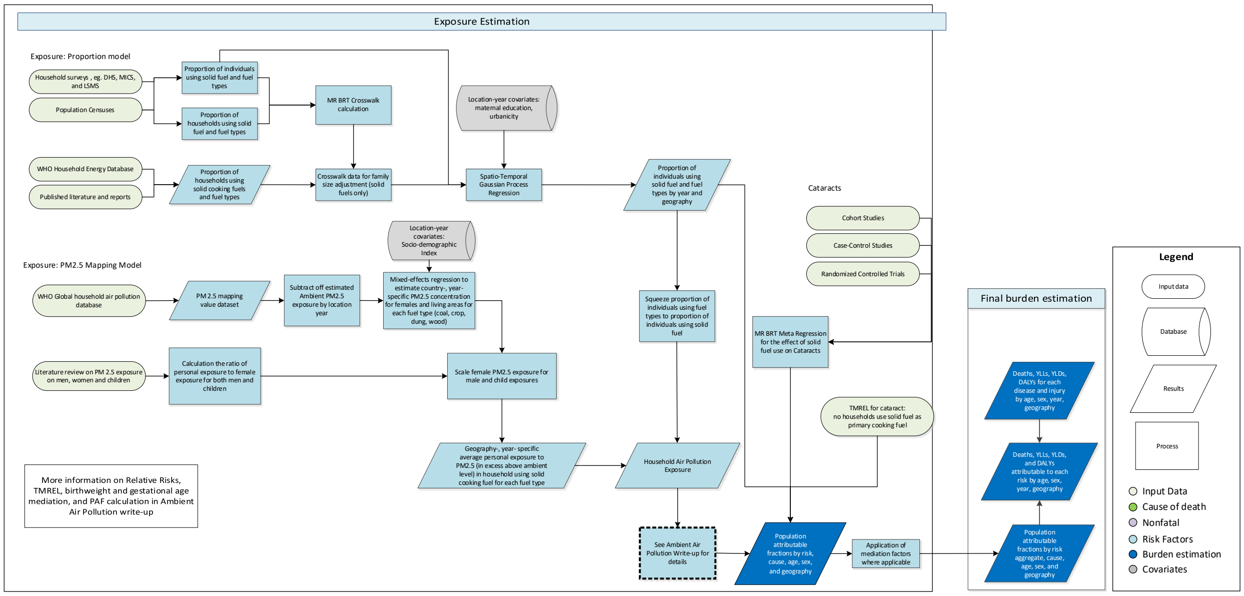

Input data and methodological summary

Exposure

Definition

Exposure to household air pollution from solid fuels (HAP) is estimated from both the proportion of individuals using solid cooking fuels and the level of exposure to particulate matter less than 2.5 micrometres in diameter ($PM_{2.5}$) air pollution for these individuals. Solid fuels in our analysis include wood, coal/charcoal, dung, and agricultural residues.

Input data

We extracted information on the use of solid fuels for cooking from standard multi-country survey series, including the Demographic and Health Surveys (DHS), Living Standards Measurement Surveys (LSMS), Multiple Indicator Cluster Surveys (MICS), and World Health Surveys (WHS). We also used data from censuses and country-specific survey series, such as the Kenya Welfare Monitoring Survey and South Africa General Household Survey. To fill remaining gaps in survey and census data, we downloaded the WHO Household Energy Database and updated estimates using extracted information from literature through a systematic review.[1] From this combined body of input data, each nationally or subnationally representative datapoint provided an estimate of the percentage of households or individuals using solid cooking fuels. We used studies from 1980 to 2020 to inform our time series estimates.

We excluded sources that did not distinguish specific primary fuel types, estimated fuel used for purposes other than cooking (eg, lighting or heating), failed to report standard error or sample size, reported over 15% missingness for households surveyed, reported fuel use in physical units, or were secondary sources referencing primary analyses.

Table 1: Data inputs for exposure for household air pollution.

Input data	Exposure
Site-years (total)	1173
Number of countries with data	161
Number of GBD regions with data (out of 21 regions)	20
Number of GBD super-regions with data (out of 7 super-regions)	7

Family size crosswalk

Many estimates in the WHO Energy Database and other reports quantify the proportion of households using solid fuel for cooking; however, we are interested in the proportion of individuals using solid fuel for cooking for exposure and burden assessment. To crosswalk these estimates, where available, we extracted fuel use at both the individual and household levels. We used studies that reported values for both household and individual solid fuel use and did not report a mean of 0 or 1. This resulted in 8074 source-specific pairs used as input data for the crosswalk model, which was modelled with the meta-regression—Bayesian, regularised, trimmed (MR-BRT) meta-regression tool. We applied this crosswalk only to proportion estimates for the parent solid fuel category. We did not adjust fuel-specific (coal/charcoal, crop, dung, or wood) proportion estimates due to lack of sufficient data for each individual fuel type.

Table 2: MR-BRT crosswalk adjustment factors for household air pollution exposure

Data input	Reference or alternative case definition	Gamma	Beta coefficient, logit (95% UI)*	Adjustment factor**
Proportion of individuals	Ref	0.095	---	---
Proportion of households	Alt		-0.094 (-0.097, -0.090)	1.099 (1.094–1.102)

**MR-BRT crosswalk adjustments can be interpreted as the factor the alternative case definition is adjusted by to reflect what it would have been had it been measured using the reference case definition. If the log/logit beta coefficient is negative, then the alternative is adjusted up to the reference. If the log/logit beta coefficient is positive, then the alternative is adjusted down to the reference.*

***The adjustment factor column is the exponentiated negative beta coefficient. For log beta coefficients, this is the relative rate between the two case definitions. For logit beta coefficients, this is the relative odds between the two case definitions.*

We applied this coefficient to household-only solid fuel reports with the following formula:

$prop_{individ}$ = the proportion of individuals using solid fuel for cooking, and

$prop_{hh}$ = the proportion of households using solid fuel for cooking.

$$\log\left(\frac{prop_{individ}}{1 - prop_{individ}}\right) = \log\left(\frac{prop_{hh}}{1 - prop_{hh}}\right) - \beta$$

or

$$prop_{individ} = \frac{prop_{hh} * e^{-\beta}}{1 - prop_{hh} + prop_{hh} * e^{-\beta}}$$

As a result, household studies were inflated to account for bias in size between households that use solid cooking fuels and those that do not. Larger households are more likely to use solid fuels for cooking. The following figure depicts the 8074 datapoints that informed the crosswalk model. Red points indicate the 10% of studies trimmed as outliers during model fitting.

Figure 1: MR-BRT crosswalk for household air pollution exposure

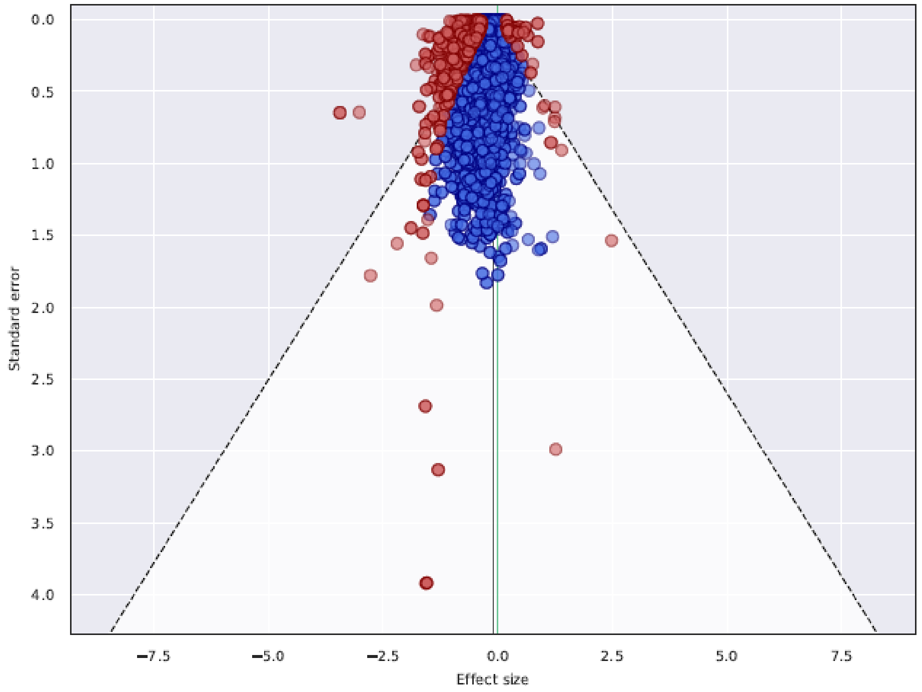

$$logit(prop_{hh}) - logit(prop_{individual})$$

Modelling strategy

As in the Global Burden of Disease (GBD) Study 2019, household air pollution was modelled at the individual level using a three-step modelling strategy implementing linear regression, spatiotemporal regression, and Gaussian process regression (GPR). The full ST-GPR process is specified elsewhere in this appendix.

For GBD 2021, we updated the HAP proportion model to disaggregate estimates of solid fuel use to estimate the proportion of individuals using each of the following component fuel type categories: 1) coal or charcoal, 2) crop residue, 3) dung, and 4) wood. With this strategy, we can more finely characterise individual exposure to $PM_{2.5}$ due to solid fuel use by applying fuel-specific mapping values to fuel-specific proportion estimates. This change addresses an important limitation in our model, in that it previously assumed equal $PM_{2.5}$ exposure for all solid fuel categories.

Fuel type-specific estimates were generated by first using ST-GPR to generate location- and year-specific estimates for coal, crop, dung, and wood. ST-GPR was also used to create estimates for the parent solid fuel category, as in GBD 2019. The first step of the ST-GPR modelling process is a mixed-effect linear regression of logit-transformed proportion of individuals using solid cooking fuels. For each of the linear models, maternal education and the proportion of population

living in urban areas were used as covariates. These models also included nested random effects by GBD region and GBD super-region.

Table 3: First-stage linear model and coefficients (solid model)

Variable	Beta (95% UI)
Intercept	3.36 (2.01, 4.71)
Maternal education (years per capita)	-0.55 (-0.58, -0.51)
Urbanicity (proportion of population living in urban areas)	-0.14 (-0.67, 0.39)

The four fuel-type-specific proportion estimates were then squeezed to the estimates for the overall proportion of individuals using solid fuel for cooking. For each location and year, we used the following formula, where $prop_{coal}$, $prop_{crop}$, $prop_{dung}$, $prop_{wood}$, and $prop_{solid}$ indicate the proportion of individuals using coal, crop, dung, wood, or any type of solid fuel, respectively.

$$\text{Let } prop_{total} = prop_{coal} + prop_{wood} + prop_{crop} + prop_{dung}$$

$$S = prop_{total} / prop_{solid}$$

For each fuel category, with coal shown below as an example, the adjusted (squeezed) proportion is calculated as

$$prop_{coal}' = prop_{coal} / S$$

In preliminary model iterations, we mapped mixed fuel strings (eg, “wood and agricultural residues") to the category associated with highest $PM_{2.5}$ exposure to avoid underestimating HAP exposure. However, fuel-specific ST-GPR models were unstable with this approach. We therefore excluded mixed-fuel string studies from final estimates for fuel-specific proportions, though we retained these studies when modelling the proportion of overall solid fuel use.

Theoretical minimum-risk exposure level

For all HAP outcomes except cataract, burden is related to both ambient and household air pollution. These PAFs are estimated jointly, and the theoretical minimum-risk exposure level (TMREL) is defined as a uniform distribution between 2.4 and 5.9 μg/m^3 $PM_{2.5}$. For cataract, the TMREL is defined as no individuals using solid cooking fuel.

Relative risks

The outcomes associated with household air pollution are lower respiratory infections (LRI), stroke, ischaemic heart disease (IHD), chronic obstructive pulmonary disease (COPD), lung cancer, type 2 diabetes, and cataract. Low birthweight and short gestation are also outcomes attributable to household air pollution through a mediation analysis. With the exception of cataract, all causes share risk curves and are calculated jointly with ambient particulate matter pollution.

Cataract relative risk meta-analysis

Prior to GBD 2019, we used the results of an external meta-analysis with a summary relative of 2.47 (95% UI 1.63–3.73) for cataract risk estimates.[2] While this effect estimate was for both sexes, in the past we estimated burden for women only because women are known to have higher HAP exposure than men. In GBD 2019, we performed our own meta-regression analysis of household air pollution and cataracts. We updated this meta-regression for GBD 2021.

We extracted all the component studies of the above meta-analysis paper but excluded one cross-sectional study. GBD risk factor analyses typically do not include cross-sectional analyses due to their weaker evidence base. In literature search conducted in GBD 2019, we found one additional paper describing different fuel types and cataracts.[3] We excluded this study because there was no comparison group without solid fuel use. We conducted an additional literature search in GBD 2021 but found no new studies to include. The following search string was used to identify studies in the PubMed database published between January 1, 2017, and July 22, 2020 (date of search).

Search string: ((“Air Pollution, Indoor”[Mesh] OR “Household air”[Title/Abstract] OR “Indoor air pollution”[Title/Abstract] OR “Indoor fine particulate matter”[Title/Abstract] OR “Indoor particulate matter”[Title/Abstract] OR “Indoor air quality”[Title/Abstract] OR “Airborne particulate matter”[Title/Abstract]) AND (“Cataract”[Title/Abstract] OR “Cataracts”[Title/Abstract] OR “Cataracts”[Mesh] OR “Lens Opacities”[Mesh] OR “Lens Opacity”[Mesh] OR “Opacities, Lens”[Mesh] OR “Opacity, Lens”[Mesh] OR “Cataract, Membranous”[Mesh] OR “Cataracts, Membranous”[Mesh] OR “Membranous Cataract”[Mesh] OR “Membranous Cataracts”[Mesh] OR “Pseudoaphakia”[Mesh] OR “Pseudoaphakias”[Mesh]))

Our resulting dataset contained eight estimates from six sources in India and Nepal. We ran a MR-BRT meta-regression on these eight estimates to generate a summary effect size of 2.52 (95% UI 1.42–4.57). We did not trim any of the observations due to the relatively few input studies available compared to other GBD risk factors. We used the MR-BRT automated covariate selection process to identify significant covariates from those extracted to quantify between-study heterogeneity. Briefly, a series of loosening Lasso penalty parameters were applied to a log-linear meta-regression on all input effect size observations. Then, covariates with a non-zero coefficient were tested for significance using a Gaussian prior (significance threshold = 0.05). No significant covariates were identified. The table and figure below provide the model coefficients and a visual representation.

Table 4: MR-BRT relative risk meta-analysis for household air pollution and cataract

Covariate	Gamma	Beta coefficient, logit (95% UI)	Beta coefficient, adjusted (95% UI)
Intercept	0.109	0.939 (0.623–1.278)	2.56 (1.86–3.59)

Figure 2: Household air pollution and cataract risk literature funnel plot

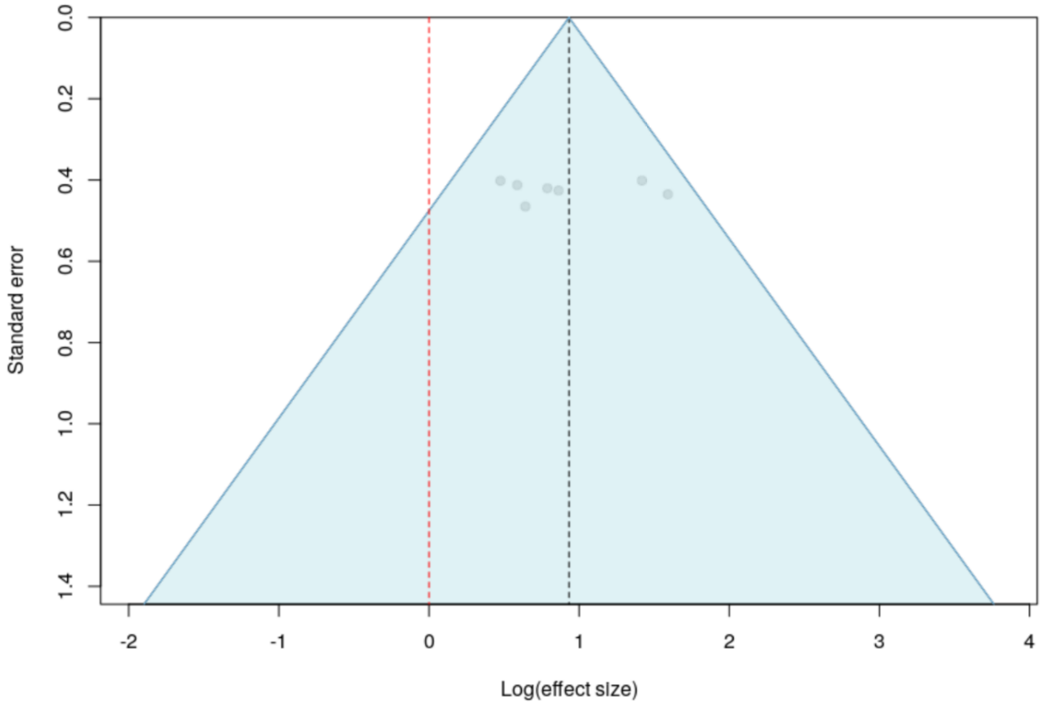

Studies reported effect sizes for males, females, and/or both sexes. In a sensitivity analysis conducted in GBD 2019 and repeated in GBD 2021, we included a covariate for sex and found no significant difference in effect size by sex. We therefore estimate cataract as an outcome of household air pollution in both males and females.

For GBD 2021, we also implemented risk-outcome scoring to provide an empirical measure of strength of evidence for risk-outcome pairs across risk factors in the GBD study (described in more detail elsewhere). Prior to generating a risk-outcome score, we conducted an additional post-analysis step to detect and flag publication bias in the input data. This approach is based on the classic Egger's Regression strategy, which is applied to the residuals in our model. In the current implementation, we do not correct for publication bias, but flag the risk-outcome pairs where the risk for publication bias is significant. Publication bias was not detected for HAP-cataract risk literature. The resulting risk-outcome score for HAP and cataract was -0.009, which corresponds to a star rating of 1.

In GBD 2021, we also made key changes to our particulate matter risk curves. These risk curves, the mediation analysis for birthweight and gestational age, and the joint-estimation PAF approach are described in the Ambient Particulate Matter Pollution appendix.

$PM_{2.5}$ mapping value estimation

To calculate relative risks from particulate matter risk curves for individuals using solid fuels for cooking, we first estimated the $PM_{2.5}$ exposure level resulting from usage of each fuel type. Input data for the HAP mapping model included indoor and personal measurement data from the WHO Global Household Air Pollution Measurements database, which contains 196 studies with measurements from 43 countries of various pollution metrics in households using solid fuel for cooking.[4] For GBD 2021, we also added data from the PURE-AIR study published in 2020, which includes additional measurements from 120 rural locations in Bangladesh, Chile, China, Colombia, India, Pakistan, Tanzania, and Zimbabwe.[5] The final dataset included 390 estimates from 76 studies in 47 unique locations. We included 281, 81, 9, and 19 measurements for indoor exposure and personal monitors for females, children (under 5), and males, respectively. 314 estimates were in households using solid fuels, 61 in households using clean fuels (gas or electricity) only, and 15 in households using a mixture of solid and clean fuels. Of measurements from households using solid fuels, we included 40, 20, 13, 155, and 86 measurements for coal, crop, dung, wood, and mixed fuels, respectively.

The following models were used to predict log-transformed estimates of excess $PM_{2.5}$ for each individual fuel type (coal, crop, dung, wood) and for the parent solid category. Predictions for the parent solid category were used only to prepare relative risk input data for analysis, not for predicting individual exposure to $PM_{2.5}$ from solid fuel use.

Fuel types:

$$\log(excess\ PM) \sim crop + coal + dung + wood + measure\ group + 24\ hr\ measurement + LDI + (1|study)$$

Solid:

$$\log(excess\ PM) \sim solid + measure\ group + 24\ hr\ measurement + LDI + (1|study)$$

Where,

- 24-hour measurement: binary variable equal to 1 if the measurement occurred over at least a 24-hour period and not only during mealtimes
- Measure group: categorical variable indicating indoor, female, male, or children
- Solid: indicator variable equal to 1 if the measurements were among households using solid fuel only, 0.5 if the measurements represented a mix of clean and solid fuels, and 0 if the households only used clean fuels.

For previous GBD cycles, we also included the Socio-demographic Index (SDI) as a variable to predict a unique value of HAP for each location and year based on development. For GBD 2021, we updated the HAP mapping model to predict unique values from the lag-distributed income per capita (LDI). Evaluations of model fit using root mean square error (RMSE) indicated that LDI is a more suitable predictor of excess $PM_{2.5}$. We also included a random effect on study and weighted each study by the square root of its sample size.

Before modelling, we subtracted off the GBD 2019 prediction of ambient $PM_{2.5}$ in the study location and year to calculate the excess particulate matter for individuals using solid fuel. The final model coefficients are included below:

Table 5: HAP mapping model and coefficients

**Variable**	**Beta, log (95% UI)**	**Beta, exponentiated (95% UI)**
Intercept	5.34 (5.16–5.52)	208.51 (174.16–249.64)
Fuel type		
• Clean (ref)		
• Crop	3.15 (3.06–3.25)	23.34 (21.33–25.79)
• Coal	1.66 (1.57–1.73)	5.26 (4.81–5.64)
• Dung	2.35 (2.22–2.48)	10.49 (9.21–11.94)
• Wood	1.99 (1.94–2.04)	7.32 (6.96–7.69)
Measure group		
• Indoor (ref)		
• Female	-0.37 (-0.42 to -0.32)	0.69 (0.66–0.73)
• Male	-0.27 (-0.36 to -0.18)	0.76 (0.70–0.84)
• Child	-1.09 (-1.19 to -1.00)	0.34 (0.30–0.37)

24-hour measurement	-0.68 (-0.83 to -0.54)	0.51 (0.44–0.58)
LDI	-2.93e-4 (-4.94e-4 to -8.37e-5)	1.00 (1.00–1.00)

To derive final predicted $PM_{2.5}$ exposure values due to solid fuel usage, instead of using direct model outputs for males and children, we scaled $PM_{2.5}$ exposure values for females to the other two groups. There are few studies of personal monitoring in men and children, so we derived ratios of female-male and female-child exposures using studies that reported PM exposure values for females and one or both of the other groups. To calculate these ratios, we first subtracted off the outdoor value from each PM measurement (using GBD 2019 ambient $PM_{2.5}$ predictions as above for $PM_{2.5}$ studies and the studies' published values for PM_4 and PM_{10} studies) and then calculated ratios weighted by sample size.

Table 6: HAP mapping personal monitoring input observations

Study	Location	Year	Pollutant	Female N	Female PM	Group	N	PM	Outdoor
Balakrishnan et al, 2004	Andhra Pradesh, Rural	2004	PM4	591	352	male	503	187	94
Gao X et al, 2009.	Tibet	2009	PM2.5	52	127	male	85	111	78
Dasgupta et al, 2006	Bangladesh	2006	PM10	944	209	male	944	166	50
Devkumar et al, 2014	Nepal	2014	PM2.5	405	169	male	429	167	167
Balakrishnan et al, 2004	Andhra Pradesh, Rural	2004	PM4	591	352	child	56	262	94
Dionisio et al, 2008.	Republic of the Gambia	2008	PM2.5	13	275	child	13	219	147
Dasgupta et al, 2006	Bangladesh	2006	PM10	944	209	child	944	199	50
Gurley et al, 2013	Bangladesh	2013	PM2.5			child	37	308	
Shupler et al, 2020	Sub-Saharan Africa	2018	PM2.5	37	153	male	20	120	26.05
Shupler et al, 2020	India	2018	PM2.5	11	150	male	5	178	42.3
Shupler et al, 2020	India	2018	PM2.5	63	89	male	48	82	42.3
Shupler et al, 2020	South Asia	2018	PM2.5	5	148	male	3	147	64
Shupler et al, 2020	South Asia	2018	PM2.5	27	148	male	17	90	64
Shupler et al, 2020	South Asia	2018	PM2.5	5	147	male	2	73	64
Shupler et al, 2020	South Asia	2018	PM2.5	15	183	male	6	135	64
Shupler et al, 2020	Latin America and Caribbean	2018	PM2.5	24	39	male	12	40	27.2
Shupler et al, 2020	China	2018	PM2.5	36	71	male	35	61	58.9
Shupler et al, 2020	China	2018	PM2.5	23	94	male	21	93	58.9
Shupler et al, 2020	China	2018	PM2.5	55	45	male	47	44	58.9
Shupler et al, 2020	China	2018	PM2.5	4	64	male	3	37	58.9

The final ratios, updated with information from the 2020 PURE-AIR study, were 0.85 (95% UI 0.67–1.09) for children and 0.64 (0.52–0.79) for males compared to 0.85 (0.56–1.31) for children and 0.64 (0.45–0.91) for males in GBD 2019. These results were used to scale the $PM_{2.5}$ mapping model fuel-type-specific predictions for these age and sex groups to calculate relative risks from the $PM_{2.5}$ risk curves.

HAP population-attributable fractions (PAFs) are calculated jointly with those for ambient particulate matter pollution. Details of PAF calculation, relative risks, and evidence scores for all outcomes besides cataract are provided in the Ambient Particulate Matter Pollution appendix.

References

1. World Health Organization (WHO). WHO Household Energy Database 1960-2017. Geneva, Switzerland: World Health Organization (WHO), 2019.
2. Smith KR, Bruce N, Balakrishnan K, Adair-Rohani H, Balmes J, Chafe Z, *et al*. Millions Dead: How Do We Know and What Does It Mean? Methods Used in the Comparative Risk Assessment of Household Air Pollution. Annu Rev Public Health. 2014; **35**(1):185–206.
3. Tanchangya J, Geater AF. Use of traditional cooking fuels and the risk of young adult cataract in rural Bangladesh: a hospital-based case-control study. BMC Ophthalmology. 2011; **11**.
4. Shupler M, Balakrishnan K, Ghosh S, *et al*. Global household air pollution database: Kitchen concentrations and personal exposures of particulate matter and carbon monoxide. Data in Brief 2018; **21**: 1292–5.
5. Shupler M, Hystad P, Birch A, Miller-Lionberg D, Jeronimo M, Arku R, *et al*. Household and personal air pollution exposure measurements from 120 communities in eight countries: results from the PURE-AIR study. Lancet Planet Health. 2020; **4**(10): E451-E462.
6. Shupler M, Godwin W, Frostad J, Gustafson P, Arku RE, Brauer M. Global estimation of exposure to fine particulate matter ($PM_{2.5}$) from household air pollution. Environment International 2018; **120**: 354–63.

Intimate partner violence

Flowchart

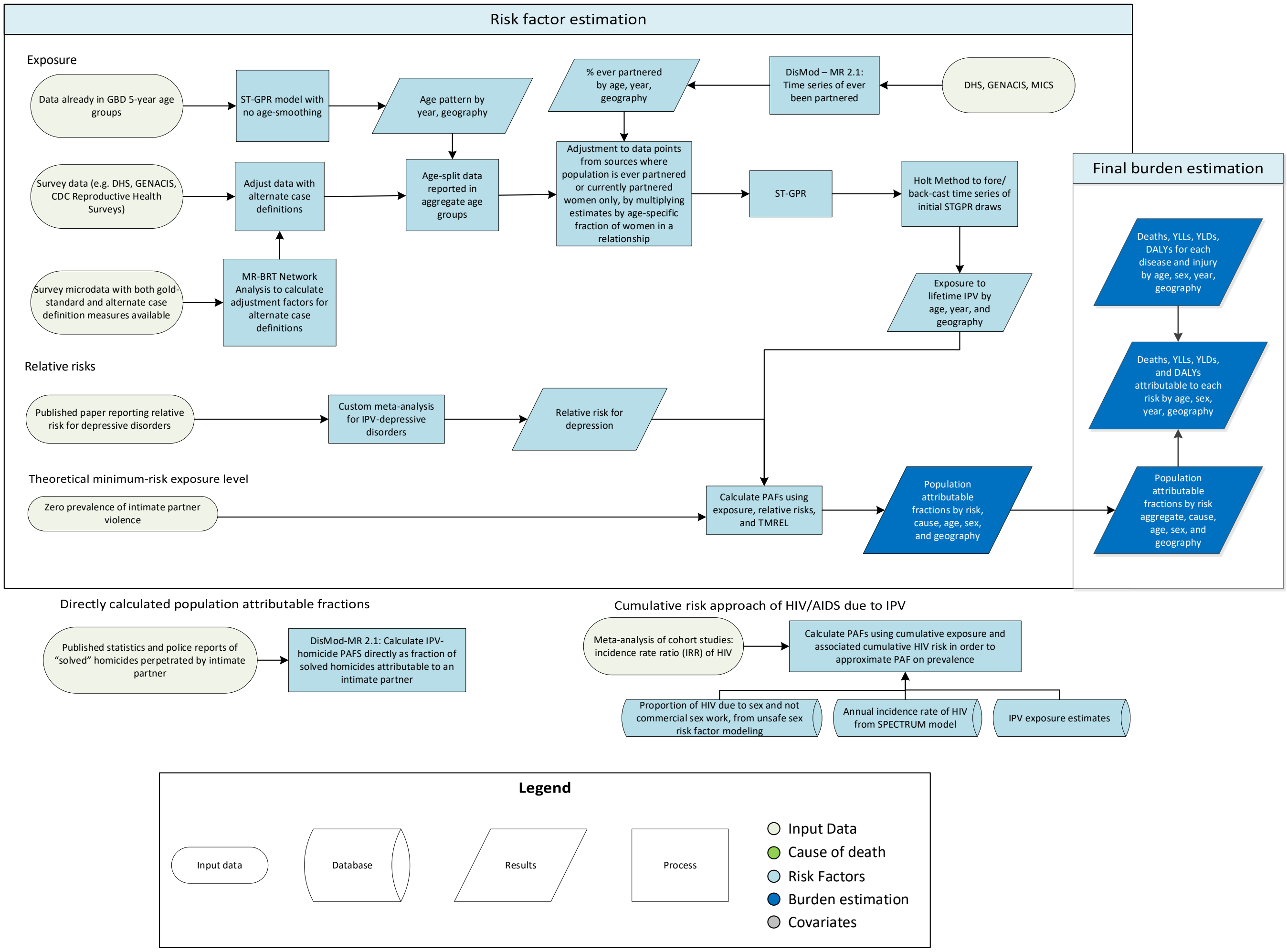

Input data and methodological summary

Definition

Exposure

The case definition for intimate partner violence (IPV) is ever having experienced one or more acts of physical and/or sexual violence by a current or former intimate partner since the age of 15 years. IPV is estimated in females only because evidence of riskoutcomes for males does not meet our inclusion criteria.

- Physical violence is defined as “being slapped or having something thrown at you that could hurt you, being pushed or shoved, being hit with a fist or something else that could hurt, being kicked, dragged, or beaten up, being choked or burnt on purpose, and/or being threatened with or actually having a gun, knife, or other weapon used on you.”

- Sexual violence is defined as "being physically forced to have intercourse when you did not want to, having sexual intercourse because you were afraid of what your partner might do, and/or being forced to do something that you found humiliating or degrading" (the definition of humiliating and degrading may vary across studies depending on the regional and cultural setting).
- Intimate partner is defined as "a partner to whom you are married or with whom you cohabit." In countries where people date, dating partners will also be considered (a partner with whom you have an intimate [sexual] relationship with but are not married to or cohabiting).

Input data

Exposure

For GBD 2021, we incorporated new exposure data sources identified through the GHDx and shared with us by collaborators. We made no updates to the systematic review conducted in 2019 for the fraction of homicides against women attributable to an intimate partner.

We included all sources that provided population-representative data on the proportion of women who have ever experienced physical or sexual violence by a current or former intimate partner. We also accepted sources reporting on the following alternate case definitions and non-reference populations:

1. Women who have ever experienced any physical IPV
2. Women who have ever experienced any sexual IPV
3. Women who have ever experienced severe physical IPV
4. Women who have experienced IPV in the past year
5. Women who have ever had an intimate partner who have experienced IPV
6. Women who currently have an intimate partner who have experienced IPV
7. Women who have experienced intimate partner violence by a spouse
8. Women who have experienced intimate partner violence by a current spouse

Table 1: Data inputs for exposure for intimate partner violence.

	**Countries with data**	**New sources**	**Total sources**
Exposure	142	115	712

Relative risk

We did not conduct a new systematic review for IPV relative risks in GBD 2021.

Table 2: Data inputs for relative risks for intimate partner violence.

	**Countries with data**	**New sources**	**Total sources**
Relative risks	7	0	9

Data processing

Crosswalking

For data that reported IPV using alternate case definitions (ie, just physical, just sexual, past-year IPV), we ran a logit-difference meta-regression with the MR-BRT tool to estimate correction factors. MR-BRT is described in detail in a separate section of this appendix. Only within-study comparisons were used to inform the meta-regression, and data

from subnational locations were not used unless they were the only data available from that location (ie, in order to avoid biasing crosswalk calculations towards locations with estimates available from multiple subnational units). In comparing alternate definitions against gold-standard, it was observed that the difference between 12-month recall and lifetime recall definitions differed by abuse type (ie, the difference in 12-month and lifetime recall was larger for physical violence only definitions when compared to sexual violence only definitions). In addition, the difference between definitions varied by age of respondent (ie, the difference between 12-month and lifetime recall widens as participants get older). For these reasons, a network meta-analysis was created with a spline on age and mutually exclusive definitions (eg, recall and abuse type alternate definition adjustment factors are interactive, not additive). The model was fit using 10% trimming and priors assuming that past 12-month prevalence would be less than lifetime prevalence of the same definition type and component definition prevalence would be less than aggregate within the same recall period (ie, severe only IPV over the lifetime would be less than physical and/or sexual over the lifetime).

Figure 1. MR-BRT network meta-analysis predictions by definition type, age

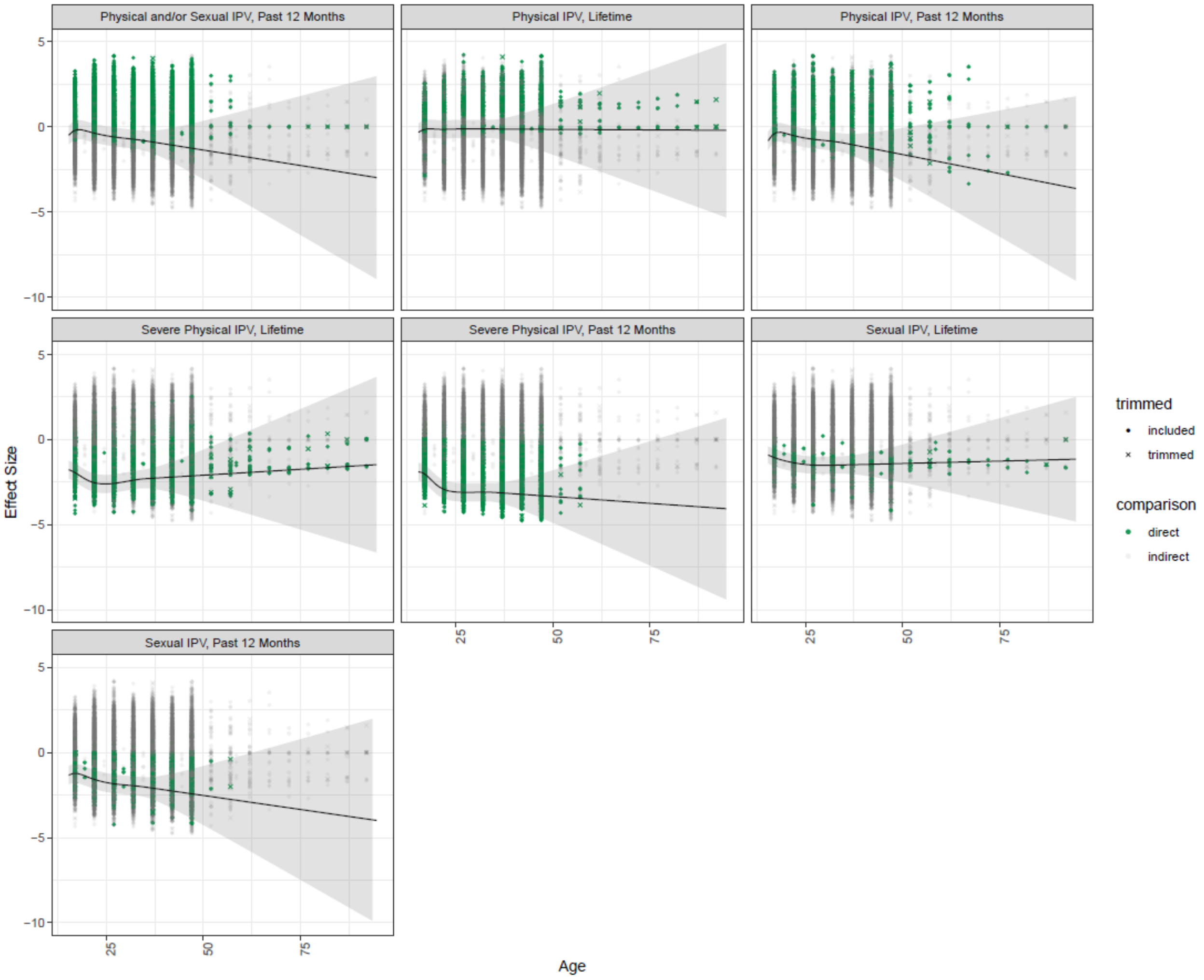

Age-splitting

We split data reported in broader age groups than the GBD five-year age groups by adapting the method reported in Ng et al. to split aggregate data using a reference age pattern. We divided the data into two sets: 1) a training dataset, containing data that already fell into GBD five-year age groups, and 2) a split dataset, which contained data reported in aggregate age groups broader than GBD five-year bins. We then used spatiotemporal Gaussian process regression (ST-GPR) to estimate geography-time-specific age patterns using the training dataset. The ST-GPR model used an age-weight parameter value that minimised the effect of any age smoothing within the model. This parameter choice allowed the

estimated age pattern to be driven by data rather than enforced by smoothing parameters of the model. Due to data sparsity within the training dataset, estimated geography-age patterns were aggregated to the GBD region level. The age pattern from the GBD region with the most training datapoints (south Asia) was used to adjust data reported in aggregated age groups.

Ever-partnered sample adjustment

To correct for studies reporting IPV prevalence out of only ever- or currently partnered women, we multiplied estimates from these studies by the age-specific fraction of women who had ever been partnered. We generated ever-partnered estimates using MICS and DHS data in a single parameter DisMod-MR 2.1 model to reflect the most recent data on proportion of women who have ever been partnered.

For studies restricting the perpetrator to spouses or current spouses, due to insufficient data comparing our reference and alternate populations in specific age-location-years, we refrained from calculating under-informed correction factors.

Modelling strategy

We use three distinct approaches to estimate burden attributable to IPV, including 1) the traditional exposure and relative risk (RR) to population attributable fraction (PAF) method for depression; 2) the direct PAF approach for estimating the proportion of homicides that are perpetrated by an intimate partner; and 3) a cumulative risk approach for estimating the burden of HIV/AIDS attributable to IPV.

Exposure

We used ST-GPR to model lifetime IPV prevalence. Input data were prepared by first adjusting data with alternate case definitions and then splitting data in aggregate age groups by applying modelled reference age patterns, as described above. Full details on the ST-GPR method are reported elsewhere in the appendix. Briefly, the mean function input to GPR is a complete time series of estimates generated from a mixed effects hierarchical linear model plus weighted residuals smoothed across time, space, and age. The linear model formula for IPV is:

$$logit(p_{g,a,t}) = \beta_0 + \sum_{k=1}^{18} \beta_k I_{A[a]} + \alpha_s + \alpha_r + \alpha_g + \epsilon_{g,a,t}$$

Where, $I_{A[a]}$ is a dummy variable indicating specific age group A that the prevalence point $p_{g,a,t}$ captures, and α_s, α_r, and α_g are super-region, region, and geography random intercepts, respectively. Random effects were used in model fitting but not in prediction.

Data sparsity within the IPV model caused poor model fits over time. Thus, we introduced Holt's linear trend method (extended simple exponential smoothing) to forecast and back-cast draws from the initial ST-GPR model. Holt's linear trend method allows forecasting of data with a linear trend using a weighted average of past observations, with weights decaying exponentially as observations get older (Hyndman et al. 2018). We applied this method to location-age-specific draws from our initial ST-GPR model, with the year range of the ST-GPR draws to be used as the initial time series defined based upon location-age data availability. For location-age combinations with available data spanning more than three years, draws were bounded from the minimum year to the maximum year of location-age-specific data. Otherwise, draws were bounded from the minimum year to the maximum year of super-region-age-specific data. To avoid over-forecasting for longer time periods (ie, in locations where only very old data were available), we used a damping parameter (phi=0.9) to enforce a zero-slope linear trend over time. Finally, due to our adjustment to ST-GPR draws we needed to re-enforce consistency between subnational and national means, so we logit-raked subnational draws to fit national means for countries with subnational estimation.

Theoretical minimum-risk exposure level

The theoretical minimum-risk exposure level for IPV is zero.

Direct PAF for female homicides

The burden of homicides attributable to intimate partner violence was modelled as a direct PAF. Input data sources provided the direct measurement of proportion of homicide cases where an intimate partner was the perpetrator. A single-parameter proportion DisMod-MR 2.1 model was run on input data to estimate geography-age-specific estimates of the fraction of homicides perpetrated by an intimate partner, which were then used as PAFs for homicide outcomes.

Cumulative risk approach for PAF of HIV/AIDS due to IPV

The third and final modelling approach that we used to assess burden attributable to intimate partner violence was a cumulative risk approach to measure the burden of HIV/AIDS attributable to IPV. As we measure burden based on deaths and prevalence, we needed to quantify attributable fractions for prevalence and death rather than incidence. To get a PAF for prevalence, we needed to consider the history of exposure to IPV and the accumulated associated risk of incident HIV due to IPV, relative to the overall risk of HIV at the population level. The ratio of cumulative IPV-attributable HIV incidence to total HIV incidence was an approximation of the relevant PAF for HIV prevalence, and we assumed this PAF can also be applied to mortality.

$$\frac{Cumulative\ HIV\ incidence\ due\ to\ IPV}{Cumulative\ HIV\ incidence\ overall} = \frac{1-\prod_{a=o}^{a=n}\left(1-PAF_{ay} * I_{ay}\right)}{1-\prod_{a=o}^{a=n}\left(1-I_{ay}\right)}$$

where:

I = annual incidence rate of HIV

a = age (15-95)

y = year (1990-2021)

$$PAF_{HIV\ incidence} = \frac{[Prevalence\ of\ IPV]_{ay} * (IRR-1)}{[Prevalence\ of\ IPV]_{ay} * (IRR-1)+1}$$

Relative risk

Depression

No changes were introduced to the GBD 2019 depression result. From six studies (Ackard DM et al, J Pediatr. 2007, Chowdhary et al, J Obstet Gynaecol Can 2007, Loxton D et al, J Interpers Violence 2006, Suglia SF et al, J Urban Health 2011 & Ouellet-Morin et al, Depression and anxiety 2015, Han et al, Journal of Affective Disorders 2019), the relative risk of depression was calculated as 1.54 (95% UI 1.00–2.36) using MR-BRT 2019. The bias covariates were selected as significant and adjusted for within the final model are listed in Table 3.

HIV incidence

No changes were introduced to the GBD 2019 HIV incidence result. From two cohort studies (Jewkes et al, Lancet 2010 & Kouyoumdijian et al, AIDS 2013) the relative risk of HIV incidence was calculated as 1.60 (95% UI 1.31–1.93) using a regression with MR-BRT 2019. The bias covariates were selected as significant and adjusted for within the final model are listed in Table 3.

Table 3. MR-BRT bias covariates by risk-outcome pair

Risk-outcome pair	Selected bias covariates

IPV – Depression	cv_symptom_scale, cv_reverse_causation, cv_subpopulation
IPV – HIV	None

Citations

1) Ng M, Freeman MK, Fleming TD, Robinson M, Dwyer-Lindgren L, Thomson B, et al. Smoking Prevalence and Cigarette Consumption in 187 Countries, 1980–2012. *JAMA*. 2014 Jan 8;311(2):183–92.
2) Hyndman, R.J., & Athanasopoulos, G. (2018) *Forecasting: principles and practice*, 2nd edition, OTexts: Melbourne, Australia. OTexts.com/fpp2.
3) Ackard DM, Eisenberg ME, Neumark-Sztainer D. Long-term impact of adolescent dating violence on the behavioral and psychological health of male and female youth. J Pediatr. 2007; 151(5): 476-81.
4) Bourassa D, Bérubé J. The prevalence of intimate partner violence among women and teenagers seeking abortion compared with those continuing pregnancy. J Obstet Gynaecol Can. 2007; 29(5): 415-23.
5) Chowdhary N, Patel V. The effect of spousal violence on women's health: findings from the Stree Arogya Shodh in Goa, India. J Postgrad Med. 2008; 54(4): 306–12.
6) Han KM, Jee HJ, An H, Shin C, Yoon HK, Ko YH, Ham BJ, Kim YK, Han C. Intimate partner violence and incidence of depression in married women: A longitudinal study of a nationally representative sample. J Affect Disord. 2019; 245():305-311
7) Jewkes RK, Dunkle K, Nduna M, Shai N. Intimate partner violence, relationship power inequity, and incidence of HIV infection in young women in South Africa: a cohort study. Lancet. 2010; 41-48.
8) Kouyoumdjian FG, Calzavara LM, Bondy SJ, O'Campo P, Serwadda D, Nalugoda F, Kagaayi J, Kigozi G, Wawer M, Gray R. Intimate partner violence is associated with incident HIV infection in women in Uganda. AIDS. 2013; 27(8): 1331-8.
9) Leung TW, Leung WC, Chan PL, Ho PC. A comparison of the prevalence of domestic violence between patients seeking termination of pregnancy and other general gynecology patients. Int J Gynaecol Obstet. 2002; 77(1): 47-54.
10) Loxton D, Schofield M, Hussain R. Psychological health in midlife among women who have ever lived with a violent partner or spouse. J Interpers Violence. 2006; 21(8): 1092-107.
11) Ouellet-Morin I, Fisher HL, York-Smith M, Fincham-Campbell S, Moffitt TE, Arseneault L. Intimate partner violence and new-onset depression: a longitudinal study of women's childhood and adult histories of abuse. Depression and anxiety. 2015;32(5):316-324.
12) Romito P, Escribà-Agüir V, Pomicino L, Lucchetta C, Scrimin F, Molzan Turan J. Violence in the lives of women in Italy who have an elective abortion. Women's Health Issues. 2009; 19(5): 335-43.
13) Suglia SF, Duarte CS, Sandel MT. Housing quality, housing instability, and maternal mental health. J Urban Health. 2011; 88(6): 1105–16.
14) Taft AJ, Watson LF. Termination of pregnancy: associations with partner violence and other factors in a national cohort of young Australian women. Aust N Z J Public Health. 2007; 31(2): 135-42.

Iron deficiency

Flowchart

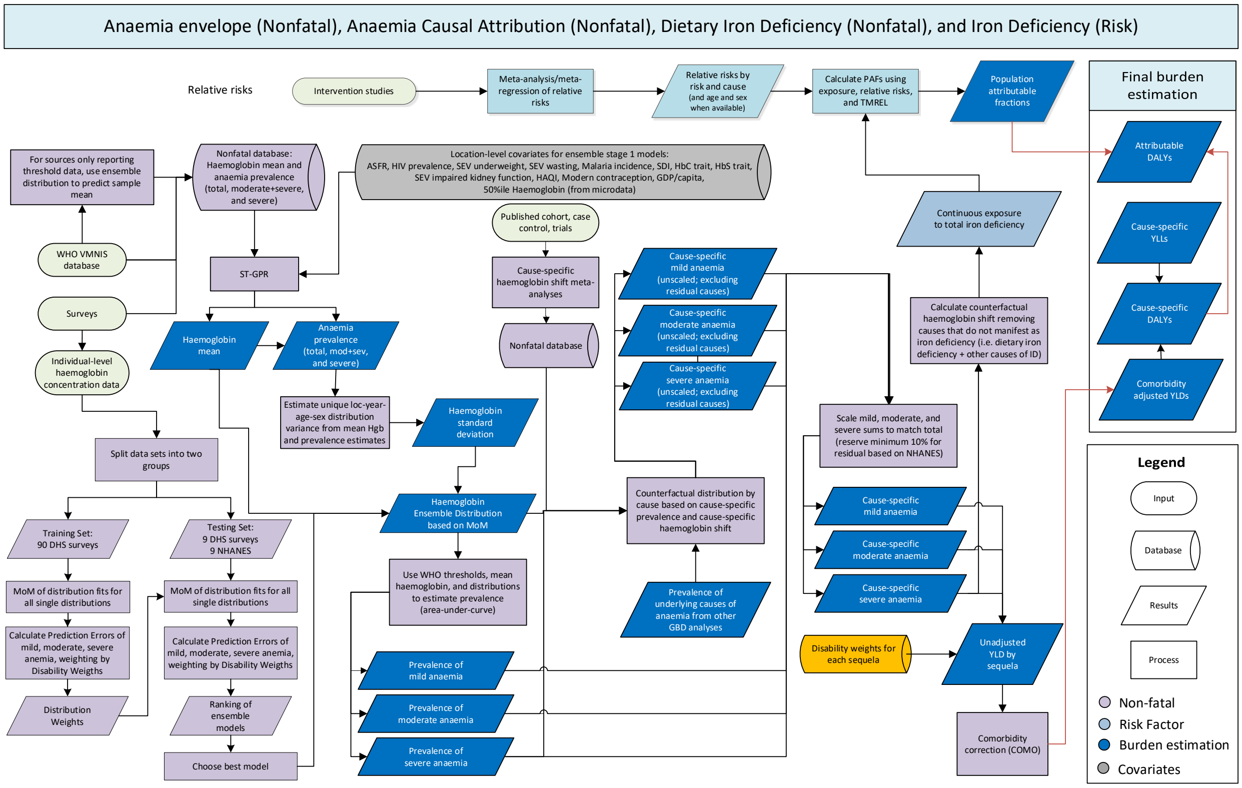

Input data and methodological summary

Definition

Exposure

Iron deficiency in the GBD risk factors analysis is defined as inadequate iron to meet the body's needs. Exposure is quantified in terms of mean haemoglobin concentration at the population level from the cumulative effect of all causes that lead to iron deficiency. This is distinct from the GBD cause of "dietary iron deficiency" that only includes the subset of anaemia that is due to inadequate intake of elemental iron and excludes other diseases that manifest as iron deficiency (eg, maternal haemorrhage, uterine fibroids, menstrual disorders, hookworm, schistosomiasis, gastritis and duodenitis, inflammatory bowel disease, etc.).

Input data

The Table 1 provides a summary of data inputs for iron deficiency.

Table 1: Data inputs iron deficiency

Measure	Countries with data	New sources	Total sources
Exposure	152	40	722
Relative risk	1	0	1

Exposure

Data informing estimates of population mean haemoglobin concentration were extracted from a variety of sources, primarily population-based household surveys such as the Demographic and Health Surveys, Multiple Indicator Cluster Surveys, and country-specific micronutrient and nutrition surveys. We supplemented these data with additional sources reporting mean haemoglobin from the WHO Vitamin and Mineral Nutrition Information System (available at http://www.who.int/vmnis/database/anaemia/countries/en/) and other literature sources where available. Additional detail on data sources and processing of haemoglobin data can be found in the "Anaemia (impairment)" documentation.

Relative risk

Data directly linking iron deficiency to other outcomes is sparse. The CHERG iron report [1] presents data supporting low haemoglobin as a risk factor for maternal outcomes, which has traditionally been used as a proxy for assigning the corresponding relative risks from that report to all maternal outcomes in the GBD study. No other outcomes have been identified as having sufficient evidence of causal relationship with iron deficiency.

In GBD 2021, we undertook a systematic review of iron supplementation trials. We identified 17 trials that reported the effect of oral or IV iron supplementation on haemoglobin levels. Fitting a network meta-regression—Bayesian, regularised, trimmed (MR-BRT) analysis of these studies, similar to the approach used for GBD crosswalking, yielded a mean haemoglobin shift of 14.3 g/L(95% UL:3.58 -25.59) resulting from IV iron supplementation. This shift best approximates the haemoglobin shift that would occur in the absence of all iron-responsive deficiencies.

Modelling strategy

Exposure

Iron deficiency was quantified as an output of the GBD Anaemia Causal Attribution framework. The GBD anaemia model has two main steps – estimation of the anaemia envelope and causal attribution – both of which inherently impact estimates of iron deficiency. See the methodological description of "Anaemia (impairment)" for detailed description of the analytic approach and inputs.

Briefly, the first step is estimating the anaemia envelope – the prevalence of mild, moderate, and severe anaemia prevalence for each GBD location, age group, sex, and year. The inputs to the envelope model are mean and standard deviation (SD) of haemoglobin [Hb] concentration. Mean haemoglobin is modelled directly in spatiotemporal Gaussian process regression (ST-GPR), and SD is estimated using a variance optimisation algorithm that takes as inputs the modelled mean haemoglobin estimates and estimates of the prevalence of severe, moderate+severe, and total anaemia (also modelled in ST-GPR). For every location, year, age, and sex we anchor the distributions at the estimated mean [Hb] value and find the variance value that minimises the error between our ST-GPR estimates of severe, moderate+severe, and total anaemia and the corresponding values implied by a given mean and variance [Hb] combination.

Individual-level data sources are then used to develop a set of ensemble distribution weights using method of moments, which are then paired with mean and SD results to produce estimates of the entire distribution of haemoglobin for each population group. A population group is a specific geography, sex, age group, and year combination. The second step is anaemia causal attribution, which generates counterfactual haemoglobin distributions for each cause of anaemia based on the cause-level prevalence (or incidence, in the case of maternal haemorrhage) estimates from the respective GBD analyses and cause-specific haemoglobin shifts that were determined via meta-analysis for each cause. The counterfactual distribution methods used the same ensemble distribution weights as the overall anaemia envelope because there is inadequate data to guide alternate distributions for each sub-cause. Mild, moderate, and severe anaemia were assigned to each cause based on the difference between the counterfactual and observed haemoglobin distributions in each population group. The sum of severity-specific prevalence was then summed to match the total, with a minimum residual of 10%,[2,3] and then the remainder was distributed between five GBD causes using fixed

proportion redistribution methods: 1) dietary iron deficiency (GBD cause), 2) other haemoglobinopathies and haemolytic anaemias, 3) other infectious diseases, 4) other neglected tropical disease, and 5) endocrine, metabolic, blood, and immune disorders.

Iron deficiency exposure for GBD risk factors analysis is the haemoglobin concentration for each population group for all diseases and injuries that manifest with iron deficiency. This was operationalised by using the observed mean haemoglobin concentration in each population as the actual exposure and then calculating a separate theoretical minimum risk exposure level (TMREL) for each population group as described below.

Theoretical minimum-risk exposure level

The implied mean haemoglobin in the absence of iron deficiency is the TMREL. To calculate the TMREL, we took an estimate of "normal" haemoglobin concentration and subtracted the prevalence-weighted haemoglobin shifts corresponding to causes that are not "iron responsive" (including all haemoglobinopathies and malaria) for each location, year, age, and sex. Normal haemoglobin was operationalised as the 95th percentile of mean haemoglobin concentration by age and sex across all locations. From this calculation we estimate a location, year, age, and sex-specific TMREL that corresponds to the expected normal haemglobin concentration after subtracting out causes that would not be responsive to supplemental iron.

Relative risk

For GBD 2021, we updated the relative risk analysis to account for the mediation of low haemoglobin on the relationship between iron deficiency and maternal outcomes. As mentioned above, we still used the relative risk values from the CHERG iron report, but adjusted the exposure values based on the haemoglobin shift that would occur under iron supplementation.

We calculated the population attributable fraction (PAF) of maternal outcomes due to low haemoglobin under two scenarios. Firstly, with the observed haemoglobin exposure. Secondly, with the haemoglobin level that would exist if everyone had received iron supplementation (the current haemoglobin exposure + iron supplementation haemoglobin shift). Explicitly, the limiting assumption here is that 100% of the population has iron deficiency. Then, we calculated the iron deficiency burden as the difference between these two PAFs such that the proportion of the risk that is from low haemoglobin and not amenable to iron supplementation is removed. In other words, the final PAF is the PAF of iron deficiency without low haemoglobin.

Notably, this approach to mediation only allows us to assign risk for iron responsive iron deficiency; in future years of the GBD study, additional trials would be needed to assess functional iron deficiency (eg, EPO, inflammation, chronic disease).

Citations

1. Murray-Kolb LE, Chen L, Chen P, Shapiro M, Caulfield L. CHERG Iron Report: Maternal Mortality, Child Mortality, Perinatal Mortality, Child Cognition, and Estimates of Prevalence of Anemia due to Iron Deficiency | GHDx. 2013. http://ghdx.healthdata.org/record/cherg-iron-report-maternal-mortality-child-mortality-perinatal-mortality-child-cognition-and (accessed Nov 12, 2019).

2. Centers for Disease Control and Prevention (CDC). Iron deficiency--United States, 1999-2000. MMWR Morb Mortal Wkly Rep 2002; 51: 897–9.

3. Looker AC, Dallman PR, Carroll MD, Gunter EW, Johnson CL. Prevalence of iron deficiency in the United States. JAMA 1997; 277: 973–6.

Kidney dysfunction

Flowchart

Kidney Dysfunction

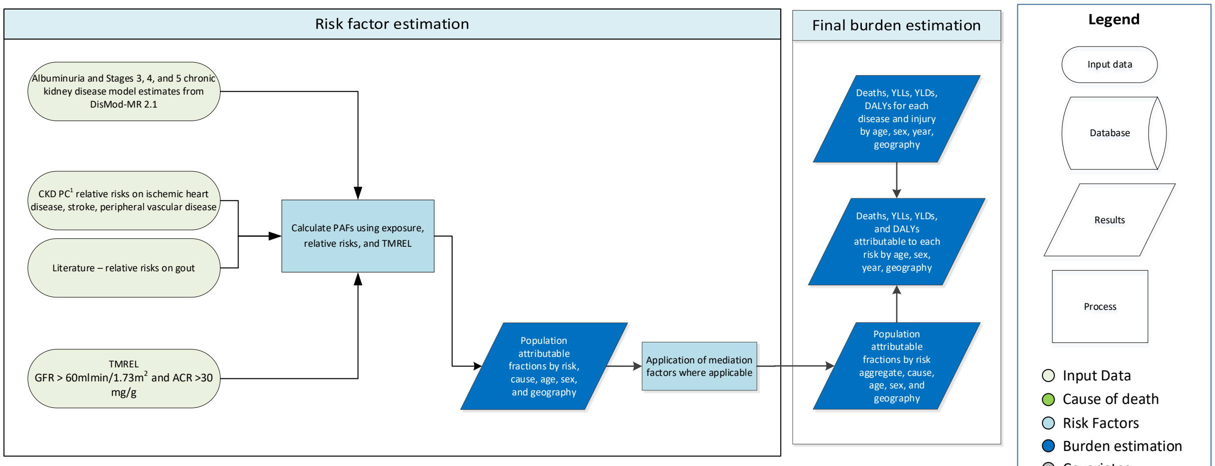

1. The Chronic Kidney Disease Prognosis Consortium collects population-level cohort data from around the world for the purpose of collective meta-analyses

Input data and methodological summary

Definition

Exposure

The kidney dysfunction (KD) risk factor exposure is divided into four categories of renal function defined by urinary albumin to creatinine ratio (ACR) and estimated glomerular filtration rate (eGFR). The definitions of KD exposures can be found in Table 1.

Table 1: Case Definitions of KD Exposure

Exposure	Parameter	Status	Definition
Stages 1&2 chronic kidney disease (CKD)	Prevalence	Reference	ACR of >=30 mg/g and estimated glomerular filtration rate (eGFR) > 60 mL/min/1.73m^2 as estimated using the CKD-EPI equation for individuals age >18 and the Schwartz equation for those <18.
Stage 3 chronic kidney disease	Prevalence	Reference	Estimated glomerular filtration rate (eGFR) 30-60 mL/min/1.73m^2 as estimated using the CKD-EPI equation for individuals age >18 and the Schwartz equation for those <18 not on renal replacement therapy.
Stage 3 chronic kidney disease	Prevalence	Alternative	Estimated glomerular filtration rate (eGFR) 30-60 mL/min/1.73m^2 as estimated using the MDRD equation (or modifications thereof) for individuals age >18 not on renal replacement therapy.
Stage 3 chronic kidney disease	Prevalence	Alternative	Estimated glomerular filtration rate (eGFR) 30-60 mL/min/1.73m^2 as estimated using the Cockcroft-Gault equation (standardised for body surface area) for individuals age >18 not on renal replacement therapy.
Stage 4 chronic kidney disease	Prevalence	Reference	Estimated glomerular filtration rate (eGFR) 15-30 mL/min/1.73m^2 as estimated using the CKD-EPI equation for

			individuals age >18 and the Schwartz equation for those <18 not on renal replacement therapy.
Stage 4 chronic kidney disease	Prevalence	Alternative	Estimated glomerular filtration rate (eGFR) 15-30 mL/min/1.73m^2 as estimated using the MDRD equation (or modifications thereof) for individuals age >18 not on renal replacement therapy.
Stage 4 chronic kidney disease	Prevalence	Alternative	Estimated glomerular filtration rate (eGFR) 15-30 mL/min/1.73m^2 as estimated using the Cockcroft-Gault equation (standardised for body surface area) for individuals age >18 not on renal replacement therapy.
Stage 5 chronic kidney disease	Prevalence	Reference	Estimated glomerular filtration rate (eGFR) <15 mL/min/1.73m^2 as estimated using the CKD-EPI equation for individuals age >18 and the Schwartz equation for those <18 not on renal replacement therapy.
Stage 5 chronic kidney disease	Prevalence	Alternative	Estimated glomerular filtration rate (eGFR) <15 mL/min/1.73m^2 as estimated using the MDRD equation (or modifications thereof) for individuals age >18 not on renal replacement therapy.
Stage 5 chronic kidney disease	Prevalence	Alternative	Estimated glomerular filtration rate (eGFR) <15 mL/min/1.73m^2 as estimated using the Cockcroft-Gault equation (standardised for body surface area) for individuals age >18 not on renal replacement therapy.

The modelling of renal function prevalence estimates is described in detail in the CKD section of the appendix to the GBD 2021 disease and injury paper.

Input data

Exposure

The last systematic review of prevalence of low glomerular filtration rate was conducted for GBD 2017, updating searches done in GBD 2016, GBD 2015, GBD 2013, and GBD 2010. Exclusion criteria included surveys that were not population-representative and studies not reporting on CKD by stage.

Relative risk

For GBD 2021, we reviewed the existing four KD-Gout sources used in previous GBD rounds. We determined that only two of the four sources were in line with our exclusion criteria. Additionally, we opportunistically extracted a new source that was found under the PubMed search (gout [Title/Abstract]) AND ((dialysis [Title/Abstract]) OR "chronic kidney disease"[Title/Abstract]). Overall, we had three sources for KD-Gout relative risks.

For our relative risks for ischaemic heart disease, peripheral artery disease, and stroke, we obtained data from the Chronic Kidney Disease Prognosis Consortium (CKD-PC). The CKD-PC is a research group composed of investigators representing cohorts from around the world. Investigators share data for the purpose of collaborative meta-analyses to study prognosis in CKD.

Data processing

The following table below is the source count for KD exposure and relative risk.

Table 2. Data inputs for KD by parameter

Parameter	Count	Number of countries
Relative risk	23	2
Exposure	226	60

For our relative risk data, we processed the data similarly to our GBD 2019 processing. We ran the relative risk data through a meta-regression—Bayesian, regularised, trimmed model[1] (MR-BRT) for the KD exposures can be found in Table 3. Moreover, we ran sensitivity analyses with and without controlling for blood pressure. This is because KD increases the risk of cardiovascular diseases directly, as well as through blood pressure. We wanted to understand how estimates of risk would differ. Generally, the relative risk of cardiovascular disease was lower when controlling for blood pressure. Thus, we decided to go with the lower risks that controlled for hypertension for a more conservative estimate.

Table 3. MR-BRT crosswalk adjustment factors for KD exposure data

Data input	Reference or alternative case definition	Gamma	Beta coefficient, Logit (95% CI)*	Adjustment factor**
Stage 3, Stage 4, Stage 5, Stage 3–5 CKD-EPI	Reference	---	---	---
Stage 3 CG	Alternative	0.25	0.24 (-0.28–0.76)	0.56 (0.43–0.68)
Stage 3 MDRD	Alternative	0.03	0.49 (0.34–0.64)	0.62 (0.58–0.66)
Stage 4 CG	Alternative	0	0.09 (-0.05–0.24)	0.52 (0.49–0.56)
Stage 4 MDRD	Alternative	0	-0.07 (-0.19–0.04)	0.48 (0.45–0.51)
Stage 5 CG	Alternative	0	-0.18 (-0.45–0.09)	0.45 (0.39–0.52)
Stage 5 MDRD	Alternative	0	-0.06 (-0.28–0.18)	0.49 (0.43–0.54)
Stage 3-5 CG	Alternative	0.26	0.23 (-0.29–0.75)	0.56 (0.43–0.68)
Stage 3-5 MDRD	Alternative	0.03	0.47 (0.32–0.62)	0.62 (0.58–0.65)

**MR-BRT crosswalk adjustments can be interpreted as the factor the alternative case definition is adjusted by to reflect what it would have been had it been measured using the reference case definition. If the log/logit beta coefficient is negative, then the alternative is adjusted up to the reference. If the log/logit beta coefficient is positive, then the alternative is adjusted down to the reference.*

***The adjustment factor column is the exponentiated beta coefficient. For log beta coefficients, this is the relative rate between the two case definitions. For logit beta coefficients, this is the relative odds between the two case definitions.*

Modelling strategy

We model the proportion of cardiovascular and musculoskeletal diseases attributable to KD. This is performed by 1) Running a disease model—Bayesian meta-regression[1] (DisMod-MR 2.1) to estimate the prevalence of stage 1–2, stage 3 CKD, stage 4 CKD, and stage 5 CKD; 2) Estimating relative risks from available data on cardiovascular outcomes and gout; and 3) Calculating the population attributable fraction of those outcomes to KD.

Exposure

The prevalence of exposure to Stage 1-2, Stage 3, Stage 4 and Stage 5 CKD were obtained from the GBD 2021 non-fatal burden of disease analysis. No bias covariates are currently used in these models, but bias adjustments were made to standardized the alternative case definitions to our reference definitions. In future rounds, we plan to test and potentially incorporate any relevant bias covariates.

Relative risk

We ran the data through MR-BRT meta-regression to determine the relationship between age and outcomes based on exposure to KD and adjust for bias covariates such as selection bias. A three-degree spline was placed on age with decreasing monotonicity. All relative risk estimates for stroke, peripheral artery disease, and ischaemic heart disease above age 85 were set equal to the risk at age 85 to control for lack of data in older age groups. Moreover, for GBD 2021, we implemented the Fisher Scoring correction to the heterogeneity parameter, which corrects for data-sparse situations. In such cases, the between-study heterogeneity parameter estimate may be zero, simply from lack of data. The Fisher Scoring correction uses a quantile of gamma, which is sensitive to the number of studies, study design, and reported uncertainty. Additionally, we have added methodology that can detect and flag publication bias. The approach is based on the classic Egger's Regression strategy, which is applied to the residuals in our model. In the current implementation, we do not correct for publication bias, but flag the risk-outcome pairs where the risk for publication bias is significant.

The following plot shows the mean relative risks for ischemic heart disease, stroke, and peripheral arterial disease by each stage of CKD. Stage 5 and stage 4 CKD have higher risks overall. Risks are also higher at younger ages and lower at the oldest age, likely reflecting competing risk factors.

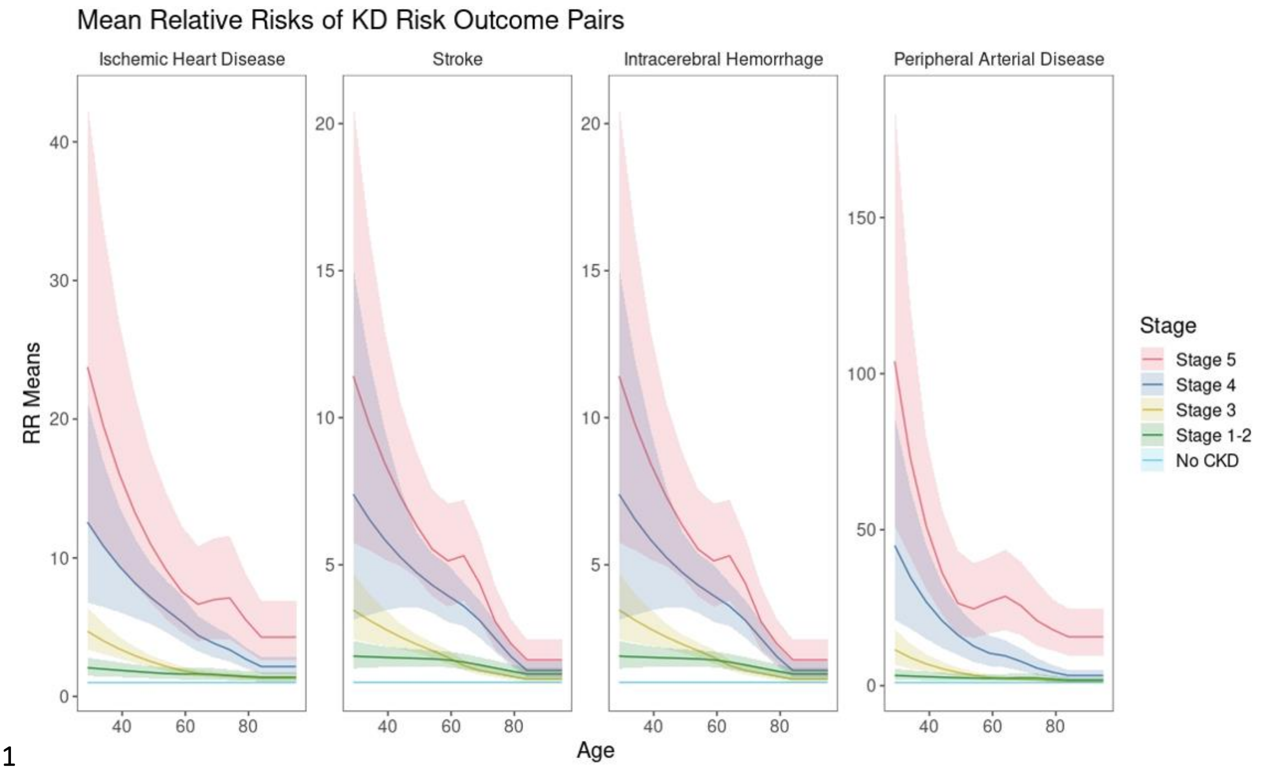

Figure 1

We also included three forest plots to show the distribution of risk estimates for heart disease, stroke, and peripheral artery disease across the studies provided by CKD-PC.

Figure 2

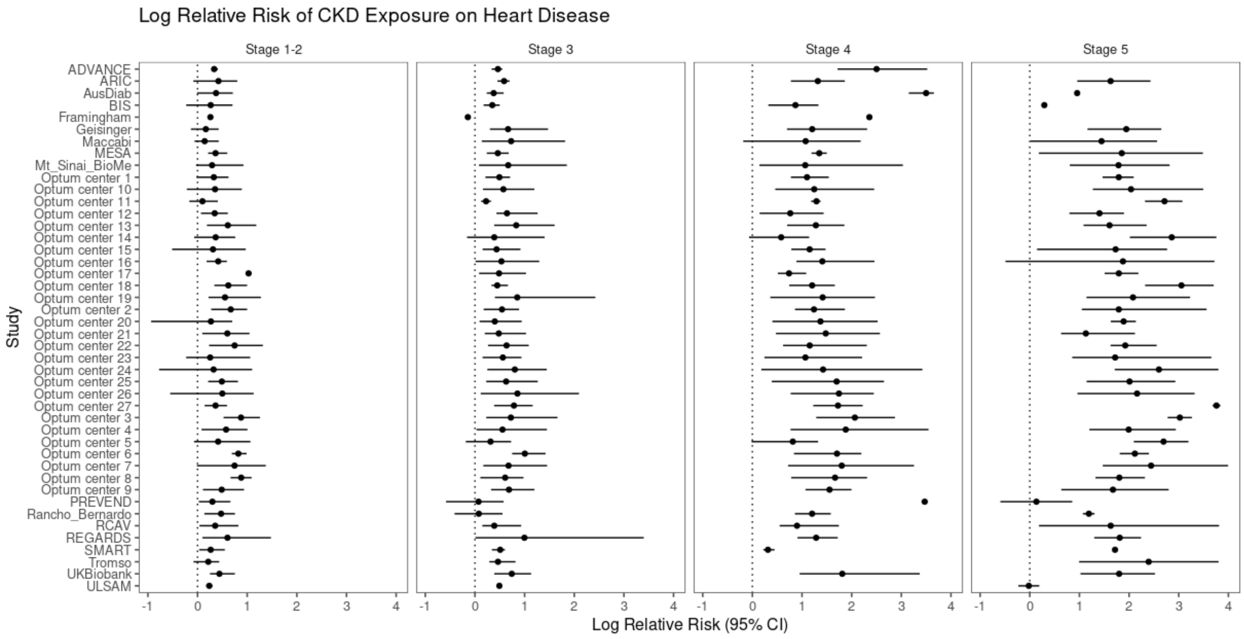

Figure 3

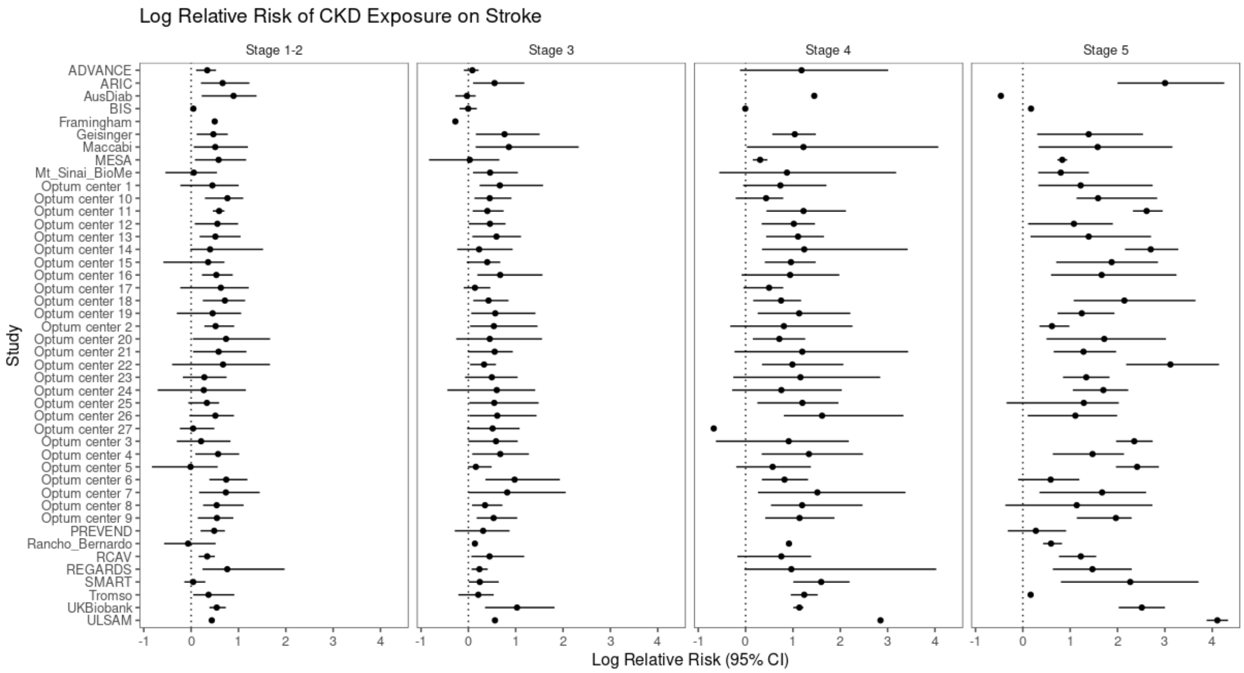

Figure 4

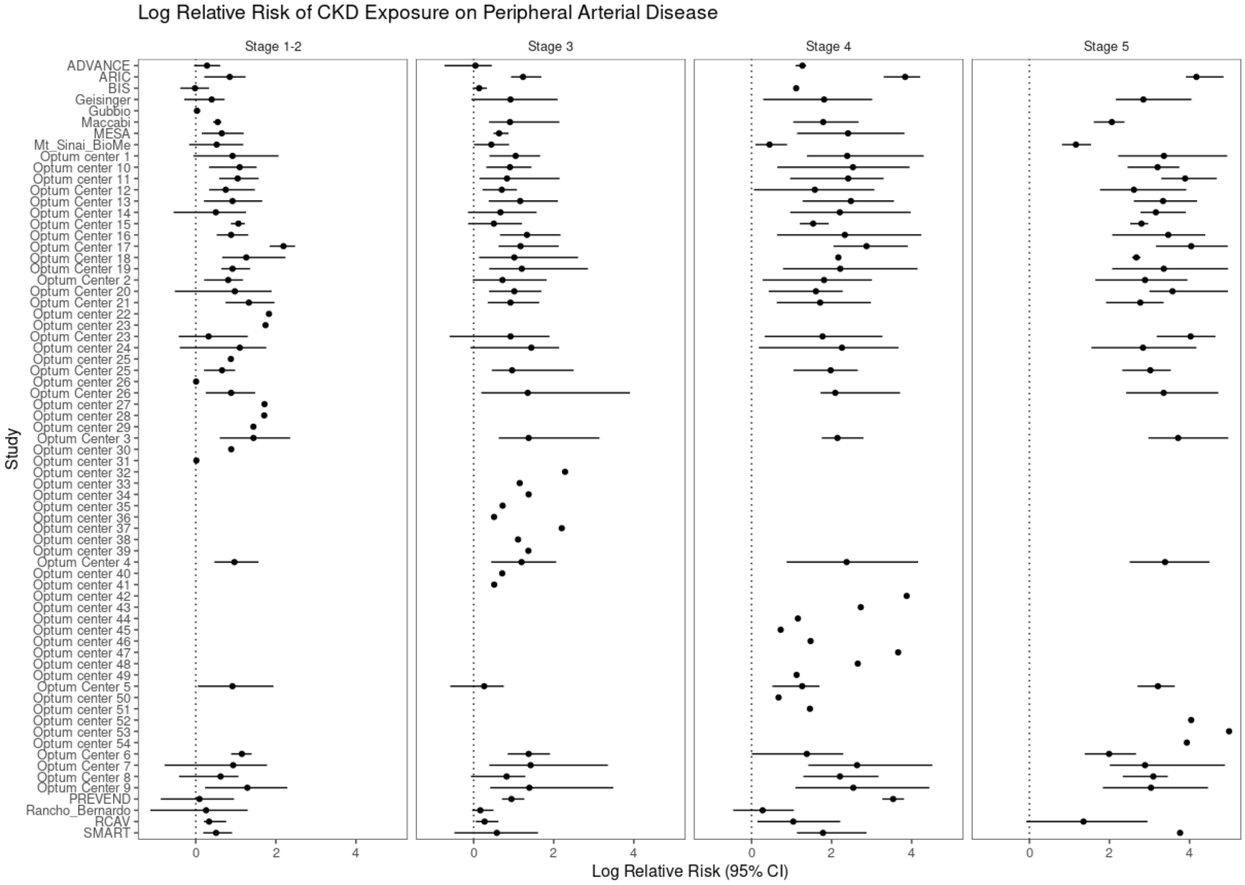

These plots further demonstrate our understanding of how the relative risks of KD shift throughout stages.

Theoretical minimum-risk exposure level

The theoretical minimum-risk exposure level is ACR 30 mg/g or less and eGFR greater than 60ml/min/1.73m^2. An ACR above 30 mg/g and eGFR below 60ml/min/1.73m^2 have been demonstrated in the literature to be the thresholds at which increased cardiovascular and gout events occur secondary to KD (2-11).

Population attributable fraction

We calculated the cardiovascular and gout fatal and non-fatal burden attributable to the categorical exposure to KD using the following equation:

$$PAF = \frac{\sum_{i=1}^{n} P_i(RR_i - 1)}{\sum_{i=1}^{n} P_i(RR_i - 1) + 1}$$

Equation 1. PAF based on categorical exposure where RR_i is the relative risk for exposure level *i*, P_i is the proportion of the population in that exposure category, and *n* is the number of exposure categories. (12)

Citations

1.Vos T, Lim SS, Abbafati C, *et al.* Global burden of 369 diseases and injuries in 204 countries and territories, 1990–2019: a systematic analysis for the Global Burden of Disease Study 2019. *The Lancet* 2020; **396**: 1204–22. doi: https://doi.org/10.1016/S0140-6736(20)30925-9 . Details found in appendix 1, section 4.4.1 and 4.5

2. Go AS, Chertow GM, Fan D, McCulloch CE, Hsu CY. Chronic kidney disease and the risks of death, cardiovascular events, and hospitalization. N Engl J Med. 2004; 351(13):1296-305.

3. Ninomiya T, Kiyohara Y, Kubo M, Tanizaki Y, Doi Y, Okubo K, et al. Chronic kidney disease and cardiovascular disease in a general Japanese population: the Hisayama Study. Kidney international. 2005; 68(1):228-36.

4. Shara NM, Wang H, Mete M, Al-Balha YR, Azalddin N, Lee ET, et al. Estimated GFR and incident cardiovascular disease events in American Indians: the Strong Heart Study. American journal of kidney diseases: the official journal of the National Kidney Foundation. 2012; 60(5):795-803.

5. Mann JF, Gerstein HC, Pogue J, Bosch J, Yusuf S. Renal insufficiency as a predictor of cardiovascular outcomes and the impact of ramipril: the HOPE randomized trial. Annals of internal medicine. 2001; 134(8):629-36.

6. Chronic Kidney Disease Prognosis C, Matsushita K, van der Velde M, Astor BC, Woodward M, Levey AS, et al. Association of estimated glomerular filtration rate and albuminuria with all-cause and cardiovascular mortality in general population cohorts: a collaborative meta-analysis. Lancet. 2010; 375(9731):2073-81.

7. De Graauw J, Chonchol M, Poppert H, Etgen T, Sander D. Relationship between kidney function and risk of asymptomatic peripheral arterial disease in elderly subjects. Nephrology, dialysis, transplantation: official publication of the European Dialysis and Transplant Association - European Renal Association. 2011;26(3):927-32.

8. Wattanakit K, Folsom AR, Selvin E, Coresh J, Hirsch AT, Weatherley BD. Kidney function and risk of peripheral arterial disease: results from the Atherosclerosis Risk in Communities (ARIC) Study. Journal of the American Society of Nephrology: JASN. 2007; 18(2):629-36.

9. O'Hare AM, Vittinghoff E, Hsia J, Shlipak MG. Renal insufficiency and the risk of lower extremity peripheral arterial disease: results from the Heart and Estrogen/Progestin Replacement Study (HERS). Journal of the American Society of Nephrology: JASN. 2004; 15(4):1046-51.

10. Manjunath G, Tighiouart H, Coresh J, Macleod B, Salem DN, Griffith JL, et al. Level of kidney function as a risk factor for cardiovascular outcomes in the elderly. Kidney international. 2003; 63(3):1121-9.

11. Manjunath G, Tighiouart H, Ibrahim H, MacLeod B, Salem DN, Griffith JL, et al. Level of kidney function as a risk factor for atherosclerotic cardiovascular outcomes in the community. Journal of the American College of Cardiology. 2003; 41(1):47-55.

12. Miettinen OS. Proportion of disease caused or prevented by a given exposure, trait or intervention. American journal of epidemiology. 1974; 99(5):325-32.

13. Tan VS, Garg AX, McArthur E, Lam NN, Sood MM, Naylor KL. The 3-Year Incidence of Gout in Elderly Patients with CKD. Clin J Am Soc Nephrol. 2017; 12(4): 577-584.

14. Krishnan E. Chronic kidney disease and the risk of incident gout among middle-aged men: a seven-year prospective observational study. Arthritis Rheum. 2013; 65(12): 3271-8.

15. McAdams-DeMarco MA, Maynard JW, Baer AN, Coresh J. Hypertension and the risk of incident gout in a population-based study: the atherosclerosis risk in communities cohort. J Clin Hypertens (Greenwich). 2012; 14(10): 675-9.

Lead exposure

Flowchart

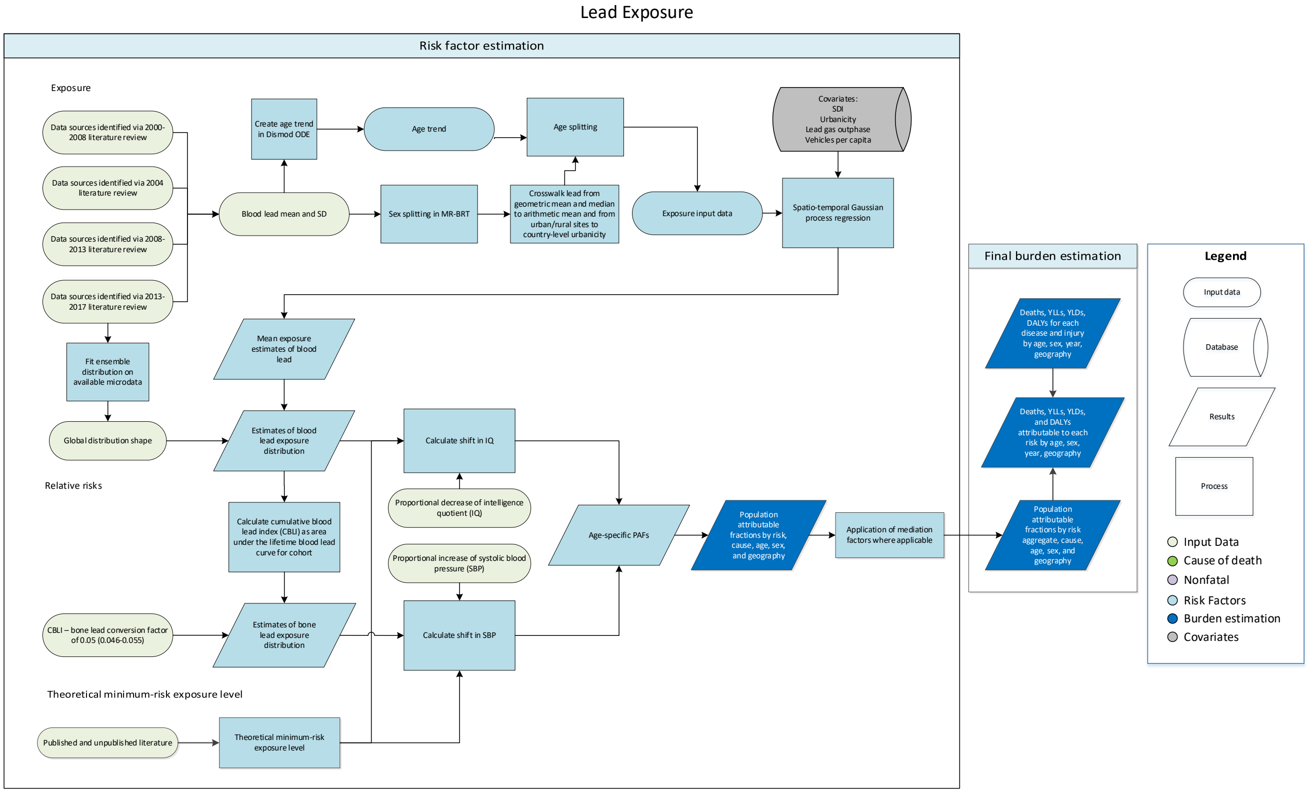

Definitions

Exposure to lead is defined in two different ways according to the currently known pathways of attributable health loss. Acute lead exposure, measured as micrograms of lead per decilitre of blood (µg/dL), is associated with IQ loss in children. Chronic lead exposure, measured as micrograms of lead per gram of bone (µg/g), is associated with cardiovascular diseases, mediated by the impact of lead on increased systolic blood pressure.

Input data

The input data for lead exposure is primarily extracted from literature reports of blood lead levels, in addition to a few blood lead surveys. Blood lead values are derived from studies that take blood samples and analyse them using various techniques to determine the level of lead present. The second pathway of burden, bone lead exposure, was estimated by calculating a cumulative blood lead index for cohorts using estimated blood lead exposure over their lifetime. The cumulative blood lead index is then used to estimate bone lead using a scalar defined in literature.[1] Table 1 provides a summary of the exposure input data used.

Table 1: Data inputs for exposure

Input data	Exposure
Source count (total)	553
Number of countries with data	85

Data processing

In the Global Burden of Diseases, Injuries, and Risk Factors Study (GBD) 2021, we used a tool called Meta-Regression, Bayesian, Regularised, Trimmed (MR-BRT) to crosswalk our data. Blood lead exposure data are reported in the literature as either an arithmetic mean, a geometric mean, or a median. To standardise the data, we adjusted all values reported as a geometric mean or median to reflect what they would have been had the study reported the arithmetic mean. Additionally, the data come from locations of varying urbanicity (proportion of individuals in a given location living in an urban area). Because we expected the urbanicity of a location to affect our estimates, we adjusted our data so that they were equivalent to the average urbanicity of the country from which the data were collected. Tables 2 and 3 show the MR-BRT crosswalk adjustment factors.

Table 2: MR-BRT crosswalk adjustment factors for lead exposure (mean)

Reference or alternative case definition	Gamma	Beta coefficient, log (95% UI)*	Adjustment factor**
Reference (data reported as arithmetic mean)	0.0052	---	---
Alternative (data reported as geometric mean)		-0.025 (-0.026 to -0.024)	0.975 (0.974–0.976)
Alternative (data reported as median)		-0.110 (-0.111 to -0.110)	0.896 (0.895–0.896)

Table 3: MR-BRT crosswalk adjustment factors for lead exposure (urbanicity)

Reference or alternative case definition	Gamma	Beta coefficient, log (95% UI)*	Adjustment factor**
Reference (study urbanicity equals national average urbanicity)	0.0138	---	---
Alternative (study urbanicity does not equal national average urbanicity)		-0.110 (-0.197 to -0.023)	0.896 (0.821–0.977)

**MR-BRT crosswalk adjustments can be interpreted as the factor the alternative case definition is adjusted by to reflect what it would have been had it been measured using the reference case definition. If the log/logit beta coefficient is negative, then the alternative is adjusted up to the reference. If the log/logit beta coefficient is positive, then the alternative is adjusted down to the reference.*

***The adjustment factor column is the exponentiated beta coefficient. For log beta coefficients, this is the relative rate between the two case definitions. For logit beta coefficients, this is the relative odds between the two case definitions.*

Exposure modelling

The methodology to estimate lead exposure last underwent significant change in GBD 2013. Global exposure had been previously modelled using age-integrating Bayesian hierarchal modelling (DisMod-MR). The modelling process was updated for GBD 2013 by shifting to a spatiotemporal Gaussian process regression methodology (ST-GPR). This allowed for estimates of all country-age-sex-year groups for single years instead of five-year periods. This approach improved the granularity of estimates for bone lead, which requires back-estimation of previous blood lead to calculate a cumulative blood lead index.

For GBD 2021, the ST-GPR modelling methodology was updated as detailed in the appendix specific to this analytical technique, which is common to a variety of risk factors. In order to predict blood lead in country-years with insufficient data, covariates that have been produced across time and space relevant to this analysis were used. For blood lead exposure, the covariates determined to have predictive ability were the Socio-demographic Index (SDI), urbanicity, the combined number of two- and four-wheeled vehicles per capita, and a covariate indicating whether leaded gasoline had been phased out in a given country-year (smoothed over the first five years of phase-out to reflect its gradual implementation). ST-GPR was used to produce estimates of mean and standard deviation of blood lead for all age groups, for both sexes, and for all GBD locations from 1970 to 2021. The linear regression equation is shown below.

$$log(data) \sim sdi + urbanicity + (leaded\ gas\ outphase * vehicles\ per\ capita) + (1|level\_1) + age$$

SDI = Socio-demographic Index
Urbanicity = proportion of population living in urban areas
Leaded gas outphase = whether or not a country has banned use of leaded gasoline
Vehicles per capita = number of 2- and 4-wheeled vehicles per capita
(1|level_1) = super-region-level random effects
Age = factor variable of all age groups

In earlier iterations of GBD, the distribution of lead exposure was assumed to be lognormal. Since GBD 2016, ensemble modelling techniques were used to find an optimal global distribution by fitting a variety of distributions to the available blood lead microdata. This was a common update for all continuous risk factors in the GBD. The ST-GPR mean and standard deviation estimates for blood lead were used with the global distribution shape to determine distributions for blood lead exposure. The distribution ultimately included 11 different probability distributions: exponential, gamma, inverse-gamma, mirrored gamma, log-logistic, Gumbel, mirrored Gumbel, Weibull, lognormal, normal, and beta. A little over 80% of the final distribution was log-logistic (35%), inverse-gamma (18%), lognormal (16%), or mirrored Gumbel (12%), with the seven other distributions comprising the remaining 20%.

To calculate blood lead over the lifetime of a given cohort, blood lead was assumed to grow linearly from 0.016 µg/dL in 1920 (see section *Theoretical minimum-risk exposure level*) to the estimated value for that cohort in 1970. Using the exposure distributions of blood lead over time and space, cohorts were constructed such that lifetime blood lead could be expressed as a curve over each year of life. The

area under this curve was the cumulative blood lead index, which was used to estimate bone lead in a given year with the aforementioned scalar from Hu et al (2007).[1]

Estimating attributable burden

Assessment of risk-outcome pairs

We included outcomes based on the strength of available evidence supporting a causal relationship. Blood lead level (a measure of acute lead exposure) was paired with idiopathic developmental intellectual disability as modelled through the impact of blood lead levels on IQ in children. Bone lead level (a measure of chronic lead exposure) was paired with systolic blood pressure, and subsequently to all cardiovascular outcomes to which systolic blood pressure is paired, which include the following: ischaemic heart disease, ischaemic stroke, intracerebral haemorrhage, subarachnoid haemorrhage, atrial fibrillation and flutter, aortic aneurysm, lower extremity peripheral arterial disease, chronic kidney disease due to hypertension, and hypertensive heart disease.

Theoretical minimum-risk exposure level

In previous iterations of GBD, the Theoretical minimum-risk exposure level (TMREL) was estimated to be 2.0 micrograms of lead per decilitre of blood. For GBD 2021, we re-evaluated our approach and concluded that the blood lead TMREL should be lowered to 0.016 µg/dL, the level experienced by pre-industrial humans, as estimated by Flegal and Smith (1992).[4] Additionally, given that bone lead levels are cumulative, we updated our bone lead TMREL to be age-specific. To do this, we created synthetic cohorts where every age and sex was exposed to the blood lead TMREL for all years of their lives. Then, we calculated the cumulative blood lead exposure and converted that to bone lead exposure using the scalar from Hu et al (2007).[1] The following table describes the bone lead TMRELs.

Table 4: Bone lead TMRELs

Age group	TMREL in µg/g (95% UI)
25–29	0.022 (0.020–0.024)
30–34	0.026 (0.024–0.028)
35–39	0.030 (0.027–0.033)
40–44	0.034 (0.031–0.037)
45–49	0.038 (0.035–0.041)
50–54	0.042 (0.038–0.046)
55–59	0.046 (0.042–0.050)
60–64	0.050 (0.045–0.054)
65–69	0.054 (0.049–0.059)
70–74	0.058 (0.053–0.063)
75–79	0.062 (0.056–0.067)
80–84	0.066 (0.060–0.072)
85–89	0.070 (0.064–0.076)
90–94	0.074 (0.067–0.080)
95 plus	0.078 (0.071–0.085)

Relative risks

Because the relative risk of IQ loss from lead exposure is specific to children, in previous iterations of GBD, no burden of lead via IQ loss was estimated in the population aged 15 and above. To better account for the continued burden of past lead exposure on IQ in older age groups, since GBD 2016 we have constructed cohorts from the entire population. Estimates of a cohort's lead exposure in early childhood (at 24 months of age) were used to determine past IQ loss, and thus calculate burden via the impact on concurrent IQ in the older population.

Blood lead relative risks were previously taken from a 2005 pooled analysis that was first incorporated in GBD 2010.[5] Those relative risks were then updated for GBD 2017 using a 2013 re-analysis of the findings of that 2005 paper, providing slightly adjusted relative risk estimates specific to exposure at 24 months of age.[6] The bone lead relative risks were adapted from a 2008 meta-analysis.[7] In GBD 2010, we modified the meta-analysis results by re-running it after removing one study (a cross-sectional study of lead workers in Korea). The revised meta-analysis results showed a 0.61 mm Hg increase in systolic blood pressure (SBP) per 10 µg/g bone lead (95% UI: -0.01 to 1.34). Because bone lead is associated with increases in SBP, all of the health loss attributable to exposure to bone lead is mediated through SBP (Table 7). As such, the relative risks for bone lead exposure are all the same as the relative risks that SBP has for its outcomes. Table 5 shows the relative risks for exposure to blood lead.

Table 5: Data inputs for relative risks

Input data	Relative risk
Source count (total)	2

Table 6: Relative risks for exposure to blood lead

Exposure level	IQ shift (95% UI)
0.016 µg/dL	0.0 (0.0–0.0)
2 µg/dL	2.172 (0.813–3.552)
4 µg/dL	3.182 (1.191-5.204)
6 µg/dL	3.847 (1.440-6.291)
8 µg/dL	4.344 (1.626-7.104)
10 µg/dL	4.741 (1.775-7.753)
12 µg/dL	5.071 (1.898-8.293)
15 µg/dL	5.482 (2.052-8.964)
20 µg/dL	6.019 (2.253-9.843)
25 µg/dL	6.442 (2.411-10.534)
30 µg/dL	6.789 (2.542-11.103)
35 µg/dL	7.085 (2.652-11.586)
40 µg/dL	7.342 (2.748-12.006)

Table 7: SBP mediation factor for bone lead

Cause Name	Mediation Factor
Ischemic heart disease	1.000 (1.000 to 1.000)

Ischemic stroke	1.000 (1.000 to 1.000)
Intracerebral hemorrhage	1.000 (1.000 to 1.000)
Subarachnoid hemorrhage	1.000 (1.000 to 1.000)
Hypertensive heart disease	1.000 (1.000 to 1.000)
Atrial fibrillation and flutter	1.000 (1.000 to 1.000)
Aortic aneurysm	1.000 (1.000 to 1.000)
Lower extremity peripheral arterial disease	1.000 (1.000 to 1.000)
Chronic kidney disease	1.000 (1.000 to 1.000)

Population attributable fraction

We used the standard GBD population attributable fraction (PAF) equation to calculate PAFs for bone lead exposure and each of its paired outcomes using exposure estimates and relative risks. We used a similar approach for estimating PAFs for the burden of intellectual disability attributable to blood lead, which uses the estimated distribution of intellectual disability and the modelled shifts in IQ due to blood lead levels to determine the PAF.

References

1. Hu H, Shih R, Rothenberg S, Schwartz BS. The epidemiology of lead toxicity in adults: measuring dose and consideration of other methodologic issues. Environ Health Perspect. 2007;115(3):455-62.
2. Pruss-Astun A, Fewtrell L, Landrigan PJ, Ayuso-Mateos JL. Lead Exposure. In: Ezzati M, Lopez AD, Rodgers A, Murray CJ, eds. Comparative quantifications of health risks: Global and regional burden of disease attrituable to selected major risk factors. Geneva, World Health Organization, 2004: 1496-542
3. CDC - Adult Blood Lead Epidemiology and Surveillance (ABLES): Program Description: NIOSH Workplace Safety and Health Topic." Centers for Disease Control and Prevention, 11 May 2018, www.cdc.gov/niosh/topics/ables/description.html.
4. Flegal AR, Smith DR. Lead levels in preindustrial humans. N Engl J Med. 1992;326(19):1293-4.
5. Lanphear BP, Hornung R, Khoury J, et al. Low-level environmental lead exposure and children's intellectual function: an international pooled analysis. Environ Health Perspect. 2005;113(7):894-9.
6. Crump K, Van Landingham C, Bowers T, Cahoy D, Chandalia J. A statistical reevaluation of the data used in the Lanphear et al. (2005) pooled-analysis that related low levels of blood lead to intellectual deficits in children. Critical Reviews in Toxicology. 2013;43(9):785-799.
7. Navas-Acien A, Schwartz BS, Rothenberg SJ, Hu H, Silbergeld EK, Guallar E. Bone lead levels and blood pressure endpoints: a meta-analysis. Epidemiology. 2008;19(3):496-504.

Low birthweight and short gestation

Flowchart

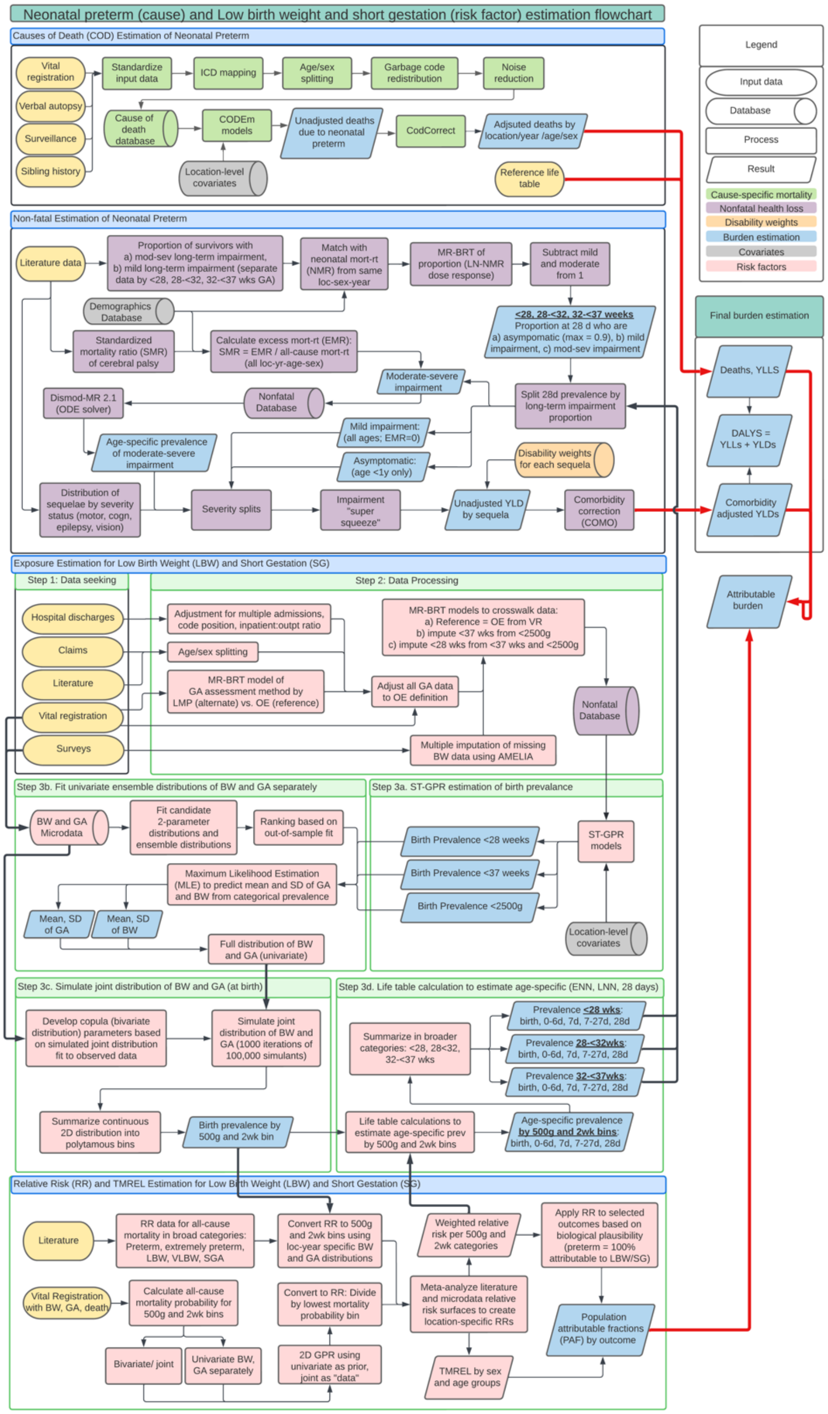

Input data and methodological summary

Short gestational age and low birthweight are highly correlated risk factors associated with poor child health outcomes. The "low birthweight and short gestation" (LBWSG) risk factor quantifies the burden of disease attributable to increased risk of death and disability due to 1) less than ideal birthweight ("low birthweight") and 2) shorter than ideal length of gestation ("short gestation").

Within GBD, attributable burden is generally estimated separately for each individual risk factor, but the combined burden attributable to multiple risk factors is of general interest. In GBD, attributable burden due to multiple risk factors is typically estimated through a "mediation analysis" that is applied after independent estimation of each risk factor's exposure, relative risk, theoretical minimum risk exposure level (TMREL), and population attributable fraction (PAF). In the mediation analysis, a "mediation factor" adjusts the PAF of each risk factor by the amount of attributable burden mediated through the other GBD risk factors. While mediation may be common, direct quantification of the joint exposure, relative risk, and PAF of the combined risk factors is conceptually more straightforward.

In GBD 2016, LBWSG became the first (and, as of GBD 2021, only) group of GBD risk factors in which combined attributable burden is quantified by direct estimation of the joint exposure, relative risk, TMREL, and PAF of multiple risk factors. After first directly estimating the joint exposure, relative risk, TMREL, and PAF of birthweight and gestational age together, we then separate out the independent PAFs due to birthweight only or gestational age only. Because of this modelling strategy, the joint GBD risk factor quantifying the burden of disease due to both less than ideal birthweight ("low birthweight") and shorter than ideal gestational age ("short gestation") is grouped into a single "parent" risk factor termed "low birthweight and short gestation". LBWSG is disaggregated into two "child" risk factors: "low birthweight for gestation" and "short gestation for birthweight". Low birthweight for gestation quantifies the burden of disease attributable to less than ideal birthweight, after adjusting for the influence of gestational age. Likewise, short gestation for birthweight quantifies the burden of disease attributable to shortened gestational age, after adjusting for the influence of birthweight.

Ideally, the model for joint exposure and joint relative risk would be fully continuous. To simplify the computation for the analysis, a grid of 500-gram and 2-week units ("bins") is used as the LBWSG dimensions and to approximate a fully continuous joint distribution model (see Figure 1).

Figure 3: Fully continuous analysis of joint gestational age and birthweight (left) is approximated with a grid of birthweight and gestational age with 500-gram and 2-week "bins" (right)

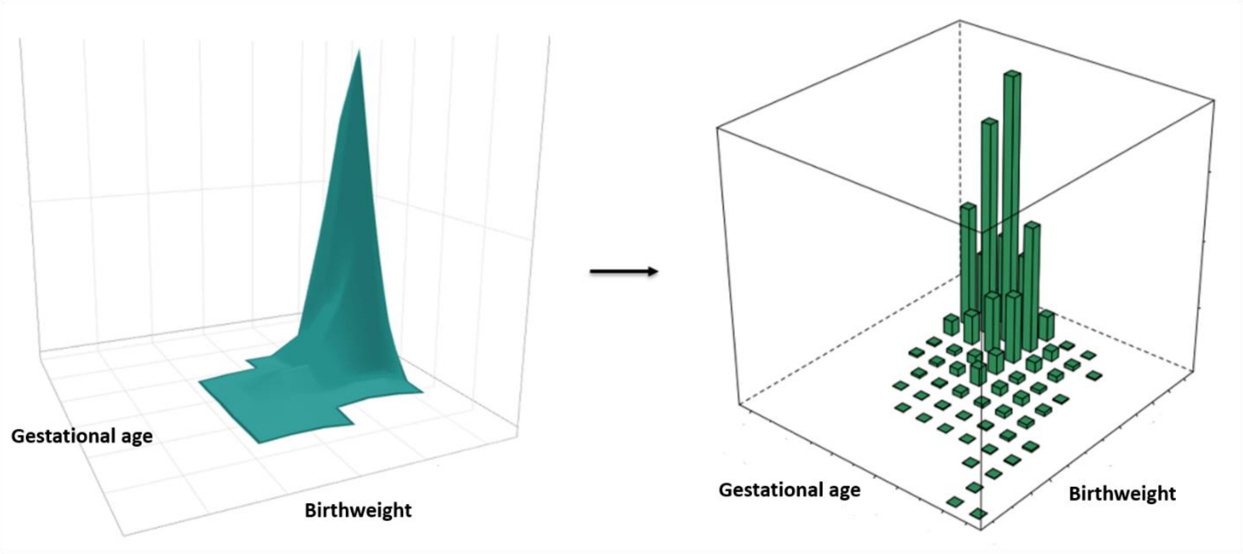

Case definition

"Low birthweight" has historically referred to any birthweight less than 2500 grams, dichotomising birthweight into two categories: "normal" and "low". In the context of the GBD LBWSG risk factor, low birthweight refers to any birthweight less than the birthweight TMREL (the birthweight that minimises risk at the population level). Because LBWSG is estimated in a grid of 500-gram and 2-week bins, any 500-gram birthweight unit less than the TMREL, which was determined as [38, 40) weeks and [3500, 4000) g for the LBWSG parent risk factor, is considered "low birthweight". This includes, for example, birthweight of [2500, 3000) grams, which the traditional, dichotomous definition of "low birthweight" would not include.

Like birthweight, gestational age is typically classified into broad categories. "Preterm" is used to describe any newborn baby born less than 37 completed weeks of gestation. In the GBD context, "short gestation" is used to refer to all gestational ages below the gestational age TMREL.

Exposure

In LBWSG, exposure refers to the portion of the joint distribution of gestational age and birthweight less than the TMREL, by location/year/sex (l/y/s), from birth to the end of the neonatal period. Modelling LBWSG exposure can be summarised in three steps:

A. Model univariate gestational age and birthweight distributions at birth, by l/y/s
B. Model joint distributions of gestational age and birthweight at birth, by l/y/s
C. Model joint distributions from birth to the end of the neonatal period, by l/y/s

Table 1: Analytic steps in estimation of YLDs due to preterm birth

	Summary of exposure modelling strategy
**Step A**<br>Model univariate distributions at birth	1. Model mean gestational age, prevalence of gestational age <28 weeks, and prevalence of gestational age <37 weeks, by l/y/s<br>2. Model mean birthweight and prevalence of birthweight <2500 grams, by l/y/s<br>3. Model univariate gestational age and birthweight distributions separately at birth, by l/y/s
**Step B**<br>Model joint distributions at birth	1. Use copulae to model the correlation structure of the joint distribution of gestational age and birthweight, globally<br>2. Model the joint distribution of gestational age and birthweight, by location/year/sex at birth, by applying the globally modelled correlation structure to the location/year/sex-specific univariate models of gestational age and birthweight distributions
**Step C**<br>Model joint distributions from birth to 28 days	1. Model all-cause mortality rates by gestational age and birthweight<br>2. Model gestational age and birthweight distributions of surviving neonates for all l/y/s from birth to end of the neonatal period, using all-cause mortality rates by gestational age and birthweight

Section 2.1.4: Input data and data processing

Input data needed to model univariate gestational age and birthweight distributions at birth (Step A):

- Prevalence of preterm birth (<37 weeks), by l/y/s
- Prevalence of preterm birth (<28 weeks), by l/y/s
- Mean gestational age, by l/y/s
- Gestational age microdata
- Prevalence of low birthweight (<2500 grams), by l/y/s
- Mean birthweight, by l/y/s
- Birthweight microdata

To model joint distributions of gestational age and birthweight (Step B), joint microdata of gestational age and birthweight are also required. Additional inputs to modelling joint distributions from birth to 28 days (Step C) are all-cause mortality by l/y/s and joint birthweight and gestational age microdata linked to mortality outcomes.

Prevalence of extremely preterm birth (<28 weeks) and preterm birth (<37 weeks) were modelled using vital registration, survey, and clinical data. For the preterm models, only inpatient and insurance claims data were included from clinical informatics datasets; outpatient data were excluded because they were more likely to capture repeated visits by the same child rather than unique visits. Prevalence of low birthweight (<2500 grams) was modelled using only vital registration and survey data.

Literature review

Before GBD 2016, available preterm birth data were sourced by a technical working group. In GBD 2016 and GBD 2017, we conducted systematic reviews to identify additional sources beyond the data already used in the models. The PubMed database was searched using the following search string:

> ((("Infant, Premature"[Mesh] OR ("infant"[All Fields] AND "premature"[All Fields]) OR "premature infant"[All Fields] OR ("preterm"[All Fields] AND "infant"[All Fields]) OR "preterm infant"[All Fields] OR ("infant, newborn"[MeSH Terms] OR ("infant"[All Fields] AND "newborn"[All Fields]) OR "newborn infant"[All Fields] OR ("newborn"[All Fields] AND "infant"[All Fields])) AND (premature[All Fields] OR preterm[All Fields]) OR "premature birth"[MeSH Terms] OR ("premature"[All Fields] AND "birth"[All Fields]) OR "premature birth"[All Fields] OR ("preterm"[All Fields] AND "birth"[All Fields]) OR "preterm birth"[All Fields]) ((("Infant, Premature"[Mesh] OR ("infant"[All Fields] AND "premature"[All Fields]) OR "premature infant"[All Fields] OR ("preterm"[All Fields] AND "infant"[All Fields]) OR "preterm infant"[All Fields] OR ("infant, newborn"[MeSH Terms] OR ("infant"[All Fields] AND "newborn"[All Fields]) OR "newborn infant"[All Fields] OR ("newborn"[All Fields] AND "infant"[All Fields])) AND (premature[All Fields] OR preterm[All Fields]) OR "premature birth"[MeSH Terms] OR ("premature"[All Fields] AND "birth"[All Fields]) OR "premature birth"[All Fields] OR ("preterm"[All Fields] AND "birth"[All Fields]) OR "preterm birth"[All Fields]) AND ("1985"[PDAT] : "3000"[PDAT]) AND "humans"[MeSH Terms].

The exclusion criteria were: studies that did not provide primary data on epidemiological parameters, non-representative studies (eg, only high-risk pregnancies), and reviews. Table 3 shows the search hits, number of full-texts reviewed, and number of extracted sources.

Table 3. LBWSG search hits, full-text review, extracted sources

Search	Hits	Full-text review	Extracted	Search date
GBD 2017	16,174	2200	154	6/6/2017

Table 4. Input data for exposure models

Input data	Exposure
Source count (total)	2233
Number of countries with data	176

Section 2.1.5:

Section 2.1.6: *Data processing*

Any data that didn't fit a GBD age groups was split into age groups using a model that was run using only age-specific data. Starting in GBD 2019, as was the case with all other non-fatal analyses, we applied empirical age and sex ratios from previous models to disaggregate observations that did not entirely fit in one GBD age category or sex. Ratios were determined by dividing the result for a specific age and sex by the result for the aggregate age and sex specified in a given observation.

Low birthweight (<2500 grams) data were extracted from literature, vital registration systems, and surveys. Survey data (most commonly from DHS and MICS) were observed to have high missingness of birthweight responses. We evaluated the patterns of missingness and found a number of distinct patterns that suggested non-random omission of birthweight observations. We therefore imputed missing birthweight values using the Amelia II (Version 1.7.6) package in R. Birthweight was predicted using the following variables also in the DHS surveys: urbanicity, sex, birthweight recorded on card, birth order, maternal education, paternal education, child age, child weight, child height, mother's age at birth, mother's weight, shared toilet facility, and household water treated.

After imputation, we completed a number of additional steps to standardize the dataset by applying a series of crosswalks. “Crosswalking” is a process of reducing non-random bias by adjusting non-standard data to the likely value had the data been collected using a reference definition, technique, or sample. Three crosswalks were applied for birthweight and gestational age data, all of the statistical models for which were developed using meta-regression – regularized, Bayesian, trimmed (MR-BRT).

First was a crosswalk for method of gestational age assessment that included three separate models. All microdata that reported GA and both obstetric estimate (OE) and last menstrual period were crosswalked to OE using the relationship derived from USA GA microdata (Figure 2). This crosswalk was developed with a spline on LMP in order to reliably match on the data that needed to be crosswalked.

Next, for all data that were only categorical, we adjusted all gestational age data to a reference definition of obstetric estimate (OE), which also included tabulations of the crosswalked microdata above. Two alternate definitions regularly appeared and both were crosswalked separately. These were Last Menstrual Period (LMP) for each of <37 weeks and <28 weeks gestation (Tables 5 and 6) and other measure of gestation age (Table 7 and 8).

The second set of crosswalks adjusted data derived from clinical administrative sources (ie. Hospital discharges and insurance claims) to matched vital registration data using OE (Tables 9 and 10).

The third set of crosswalks served to “square the input dataset” to ensure that every location-year with data had an observation for each of <2500g (birthweight), <37 weeks, and <28 weeks. This process utilized relationships between input data types to maximize the volume of data later input to models. Low birthweight data (<2500g) were crosswalked to preterm (<37 weeks) data (Table 11), preterm to extremely preterm (Table 12), and extremely preterm to preterm (Table 13).

Figure 2. MR-BRT OE-LMP crosswalk adjustment factor by LMP-reported gestational age

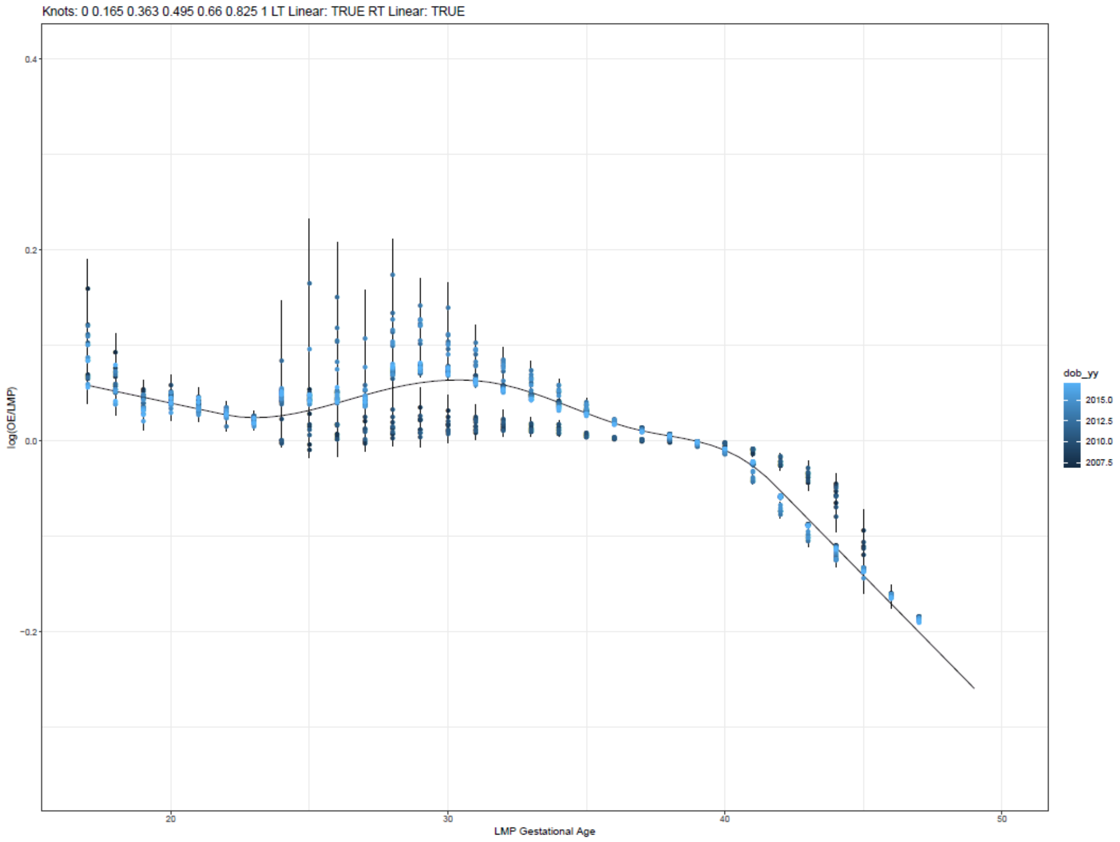

Table 5. MR-BRT OE-LMP crosswalk adjustment factor for preterm birth (<37 weeks of gestation)

Data input	Reference or alternative case definition	Gamma	Beta coefficient, log (95% CI)	Adjustment factor*
Obstetric estimate	Reference	0.01	---	---
Last menstrual period	Alternative		0.187 (0.142,0.231)	1.205 (1.153, 1.260)

Table 6. MR-BRT OE-LMP crosswalk adjustment factor for extremely preterm (<28 weeks gestation)

Data input	Reference or alternative case definition	Gamma	Beta coefficient, log (95% CI)	Adjustment factor*
Obstetric estimate	Reference	0.00	---	---
Last menstrual period	Alternative		0.0284 (0.268,0.300)	1.328 (1.308, 1.349)

Table 7. MR-BRT OE-other measure crosswalk adjustment factor for preterm birth (<37 weeks gestation)

Data input	Reference or alternative case definition	Gamma	Beta coefficient, log (95% CI)	Adjustment factor*
Obstetric estimate	Reference	0.10	---	---
Other measurement	Alternative		-0.243 (--0.494, 0.009)	0.785 (0.610, 1.01)

Table 8. MR-BRT OE-other measure crosswalk adjustment factor for extremely preterm birth (<28 weeks gestation)

Data input	Reference or alternative case definition	Gamma	Beta coefficient, log (95% CI)	Adjustment factor*
Obstetric estimate	Reference	0.37	---	---
Other measurement	Alternative		0.154 (-0.486, 0.793)	1.166 (0.615, 2.210)

Table 9. MR-BRT VR-claims crosswalk adjustment factor for preterm birth (<37 weeks gestation)

Data input	Reference or alternative case definition	Gamma	Beta coefficient, log (95% CI)	Adjustment factor*
Vital registration	Reference	0.07	---	---
Insurance claims	Alternative		-0.712 (-0.909, -0.515)	0.491 (0.403, 0.597)

Table 10. MR-BRT VR-insurance claims crosswalk adjustment factor for extremely preterm birth (<28 weeks of gestation)

Data input	Reference or alternative case definition	Gamma	Beta coefficient, log (95% CI)	Adjustment factor*
Vital registration	Reference	0.02	---	---
Insurance claims	Alternative		-1.258 (-1.447, -1.07)	0.284 (0.235, 0.344)

Table 11. MR-BRT low birthweight to preterm birth (<37 weeks gestation)

Data input	Reference or alternative case definition	Gamma	Beta coefficient, log (95% CI)	Adjustment factor*
Preterm birth	Reference	0.08	---	---
Low birthweight	Alternative		-0.479 (-0.518, -0.440)	0.620 (0.596, 0.644)

Table 12. MR-BRT preterm (<37 weeks gestation) to extremely preterm (<28 weeks gestation)

Data input	Reference or alternative case definition	Gamma	Beta coefficient, log (95% CI)	Adjustment factor*
28 weeks	Reference	0.06	---	---
37 weeks	Alternative		3.221 (3.161, 3.281)	25.053 (23.600, 26.604)

Table 13. MR-BRT extremely preterm (<28 weeks gestation) to preterm (<37 weeks gestation)

Data input	Reference or alternative case definition	Gamma	Beta coefficient, log (95% CI)	Adjustment factor*
37 weeks	Reference	0.05	---	---
28 weeks	Alternative		-3.208 (-3.266, -3.150)	0.0404 (0.0381, 0.0428)

**MR-BRT crosswalk adjustments can be interpreted as the factor the alternative case definition is adjusted by to reflect what it would have been had it been measured using the reference case definition. If the log/logit beta coefficient is negative, then the alternative is adjusted up to the reference. If the log/logit beta coefficient is positive, then the alternative is adjusted down to the reference.*

***The adjustment factor column is the exponentiated beta coefficient. For log beta coefficients, this is the relative rate between the two case definitions. For logit beta coefficients, this is the relative odds between the two case definitions.*

These data adjustments had the effect of dramatically increasing the size of each of the modelling datasets and are primarily responsible for most changes in preterm estimates between GBD 2019 and GBD 2021. After all crosswalks, we performed a deduplication step on GA models. Namely, if low birthweight data in countries that were 1) categorised as "data-rich" locations in cause-of-death modelling or had at least 10 consecutive years of vital registration data recording gestational age, and 2) had both preterm birth and low birthweight data, then crosswalked low birthweight data were outliered so that the model was informed only by the gestational age data.

Modelling strategy

Step A: Model univariate birthweight and gestational age distributions at birth, by l/y/s

Microdata are the ideal data source for modelling distributions; however, microdata are not widely available for birthweight and are scarcer for gestational age. Categorical prevalence data are more readily available from a wider range of locations and years for low birthweight (<2500g), extremely preterm (<28 weeks of gestation), and preterm birth (<37 weeks of gestation). Because categorical prevalence has wider availability than microdata, we use prevalence data to assist in modelling birthweight and gestational age ensemble distributions.

Ensemble distribution models can be constructed with three pieces of information: mean of the distribution, variance of the distribution, and the weights of the distributions being used in the ensemble. To model mean and variance for all l/y/s for birthweight and gestational age, we first used spatiotemporal Gaussian process regression (ST-GPR) models to model prevalence of low birthweight, extremely preterm, and preterm birth for all l/y/s at birth. To model mean birthweight for all l/y/s, OLS linear regression was used to regress mean birthweight on log-transformed low birthweight prevalence. This model was then used to predict mean birthweight for all l/y/s, using the prevalence of low birthweight (<2500 grams) modelled for all l/y/s in ST-GPR. Similarly, to model gestational age mean for all l/y/s, OLS linear regression model was used to regress mean gestational age on log-transformed preterm prevalence. Mean gestational age for all l/y/s was predicted using the preterm birth (<37 weeks) estimated modelled in ST-GPR.

Global ensemble weights for gestational age were derived by using all available gestational age and birthweight microdata in Table 14 to select the ensemble weights. The distribution families included in the optimization process were exponential, gamma, gumbel, Weibull, log-normal, normal, mirrored gamma, and mirrored gumbel. As an advancement in GBD 2021, ensemble weights were fit that specifically targeted the fit at 28 weeks and 37 weeks for gestational age and 1500 grams and 2500 grams for low birthweight. In previous GBD cycles the fit of these models had been optimized to reduce error across the entire distribution. Additionally, as an improvement in GBD 2021, this ensemble weight fitting strategy optimized on all microdata sources simultaneously, as opposed to separately.

For each l/y/s, given the mean and ensemble weights, the variance was optimised to minimise error on the prevalence of preterm birth (<37 weeks) for the gestational age distribution and prevalence of low birthweight (<2500 grams) for the birthweight distribution.

Step B: Model joint birthweight and gestational age distributions at birth, by l/y/s

In order to model the joint distribution of gestational age and birthweight from separate distributions, information was needed about the correlation between the two distributions. Distributions of gestational age and birthweight are not independent; the Spearman correlation for each country where

joint microdata were available (Table 14), pooling across all years of data available, ranged from 0.25 to 0.49. The overall Spearman correlation was 0.38, pooling across all countries in the dataset.

Table 14. Summary of microdata inputs

Location	Years of data	Total births*	Format of data	Spearman correlation	Used in ensemble weight selection	Used in copula parameter selection	Used in relative risk models
BRA	2016	2,854,380	Microdata	0.37	Yes	Yes	No
ECU	2003–2015	2,473,039	Microdata	0.34	Yes	Yes	No
ESP	1990–2014	8,537,220	Microdata	0.42	Yes	Yes	No
JPN	1995–2015	23,644,506	Tabulations	0.41	No	No	Yes
MEX	2008–2012	10,256,117	Microdata	0.35	Yes	Yes	No
NOR	1990–2014	1,489,210	Microdata	0.44	Yes	Yes	Yes
NZL	1990–2016	1,600,501	Microdata	0.25	Yes	Yes	Yes
SGP	1993–2015	972,775	Tabulations	0.41	No	No	Yes
TWN	1998–2002	1,331,760	Tabulations	0.38	No	No	Yes
URY	1996–2014	698,622	Microdata	0.49	Yes	Yes	No
USA	1990–2014	81,929,879	Microdata	0.38	Yes	Yes	Yes

** Pooled across all years and sexes, excluding data missing year of birth, gestational age, or birthweight*

Joint distributions between the birthweight and gestational age marginal distributions were modelled with copulae. The Copula and VineCopula packages in R were used to select the optimal copula family and copula parameters to model the joint distribution, using joint microdata from the country-years in Table 14. The copula family selected from the microdata was "Survival BB8", with theta parameter set to 1.75 and delta parameter set to 1.

The joint distribution of birthweight and gestational age per location-year-sex was modelled using the global copula family and parameters selected and the location-year-sex gestational age and birthweight distributions. The joint distribution was simulated 100 times to capture uncertainty. Each simulation consisted of 10,000 simulated joint birthweight and gestational age datapoints. Each joint distribution was divided into 500g by 2-week bins to match the categorical bins of the relative risk surface. Birth prevalence was then calculated for each 500g by 2-week bin.

Step C: Model joint distributions from birth to the end of the neonatal period, by l/y/s

Early neonatal prevalence and late neonatal prevalence were estimated using life table approaches for each 500g and 2-week bin. Using the all-cause early neonatal mortality rate for each location-year-sex, births per location-year-sex-bin, and the relative risks for each location-year-sex-bin in the early neonatal period, the all-cause early neonatal mortality rate was calculated for each location-year-sex-bin. The early neonatal mortality rate per bin was used to calculate the number of survivors at seven days and prevalence in the early neonatal period. Using the same process, the all-cause late neonatal mortality rate for each location-year-sex was paired with the number of survivors at seven days and late neonatal relative risks per bin to calculate late neonatal prevalence and survivors at 28 days.

Relative risks and theoretical minimum risk exposure level

LBWSG is paired with the outcomes listed in Table 15 and is only attributed to burden in the early and late neonatal period.

Table 15: Cause list of outcomes for low birthweight and short gestation

Cause name
Diarrhoeal diseases
Lower respiratory infections
Upper respiratory infections
Otitis media
Pneumococcal meningitis
H influenzae type B meningitis
Meningococcal meningitis
Other meningitis
Encephalitis
Neonatal preterm birth complications
Neonatal encephalopathy due to birth asphyxia and trauma
Neonatal sepsis and other neonatal infections
Haemolytic disease and other neonatal jaundice
Other neonatal disorders
Sudden infant death syndrome

Causes

The available data for deriving relative risk was only for all-cause mortality. The exception was the USA linked infant birth-death cohort data, which contained three-digit ICD causes of death, but also had nearly 30% of deaths coded to causes that are ill-defined, or intermediate, in the GBD cause classification system. We analysed the relative risk of all-cause mortality across all available sources and selected outcomes based on criteria of biological plausibility. Some causes, most notably congenital birth defects, haemoglobinopathies, malaria, and HIV/AIDS, were excluded based on the criteria that reverse causality could not be excluded.

Input data

In the Norway, New Zealand, and USA Linked Birth/Death Cohort microdata datasets, livebirths are reported with gestational age, birthweight, and an indicator of death at 7 days and 28 days. For this analysis, gestational age was grouped into two-week categories, and birthweight was grouped into 500-gram categories. The Taiwan, Japan, and Singapore datasets were prepared in tabulations of joint 500-gram and two-week categories. A pooled country analysis of mortality risk in the early neonatal period and late neonatal period by “small-for-gestational-age” category in developing countries in Asia and sub-Saharan Africa were also used to inform the relative risk analysis.

Table 16: Input data for relative risk models

Input data	Relative risk
Source count (total)	113
Number of countries with data	6

Modelling strategy

For each location, data were pooled across years, and the risk of all-cause mortality at the early neonatal period and late neonatal period at joint birthweight and gestational age combinations was calculated. In all datasets except for the USA, sex-specific data were combined to maximise sample size. The USA analyses were sex-specific. To calculate relative risk at each 500-gram and two-week combination, logistic regression was first used to calculate mortality odds for each joint two-week gestational age and 500-gram birthweight category. Mortality odds were smoothed with Gaussian process regression, with the independent distributions of mortality odds by birthweight and mortality odds by gestational age serving as priors in the regression.

A pooled country analysis of mortality risk in the early neonatal period and late neonatal period by SGA category in developing countries in Asia and sub-Saharan Africa were also converted into 500-gram and two-week bin mortality odds surfaces. The relative risk surfaces produced from microdata and the Asia and Africa surfaces produced from the pooled country analysis were meta-analysed, resulting in a meta-analysed mortality odds surface for each location. The meta-analysed mortality odds surface for each location was smoothed using Gaussian process regression and then converted into mortality risk. To calculate mortality relative risks, the risk of each joint two-week gestational age and 500-gram birthweight category were divided by the risk of mortality in the joint gestational age and birthweight category with the lowest mortality risk.

For each of the country-derived relative risk surfaces, the 500-gram and two-week gestational age joint bin with the lowest risk was identified. This bin differed within each country dataset. To identify the universal 500-gram and two-week gestational age category that would serve as the universal TMREL for our analysis, we chose the bins that was identified to be the TMREL in each country dataset to contribute to the universal TMREL. Therefore, the joint categories that served as our universal TMREL for the LBWSG risk factor were “38–40 weeks of gestation and 3500–4000 grams”, “38–40 weeks of gestation and 4000–4500 grams”, and “40–42 weeks of gestation and 4000–4500 grams”. As the joint TMREL, all three categories were assigned to a relative risk equal to 1.

Population attributable fraction

The total PAF for the low birthweight and short gestation joint risk factor was calculated by summing the PAF calculated from each 500g x two-week category, with the lowest risk category among all the 500g x two-week categories serving as the TMREL. The equation for calculating PAF for each 500g x two-week category is:

$$PAF_{joasgt} = \frac{\sum_{x=1}^{u} RR_{joast}(x)P_{jasgt}(x) - RR_{joasg}(TMRE_{jas})}{\sum_{x=1}^{u} RR_{joas}(x)P_{jasgt}(x)}$$

To calculate the PAFs for the univariate risks (‘short gestation for birthweight’ and ‘low birthweight for gestation’), relative risks are first weighted by global exposure in 2019, summed across one of the dimensions (gestational age or birthweight), and then rescaled by the maximum relative risk in the TMREL block (38-42 weeks of gestation and 3500-4500 grams). Any relative risk less than 1 was set to 1. Exposure was also summed across the same dimension, and the univariate PAF equalled the sum of the product of the weighted relative risks and exposures.

Citations

1. Katz J, Lee AC, Kozuki N, Lawn JE, Cousens S, Blencowe H, et al. Mortality risk in preterm and small-for-gestational-age infants in low-income and middle-income countries: a pooled country analysis. The Lancet. 2013;382(9890):417–25.

Low physical activity

Flowchart

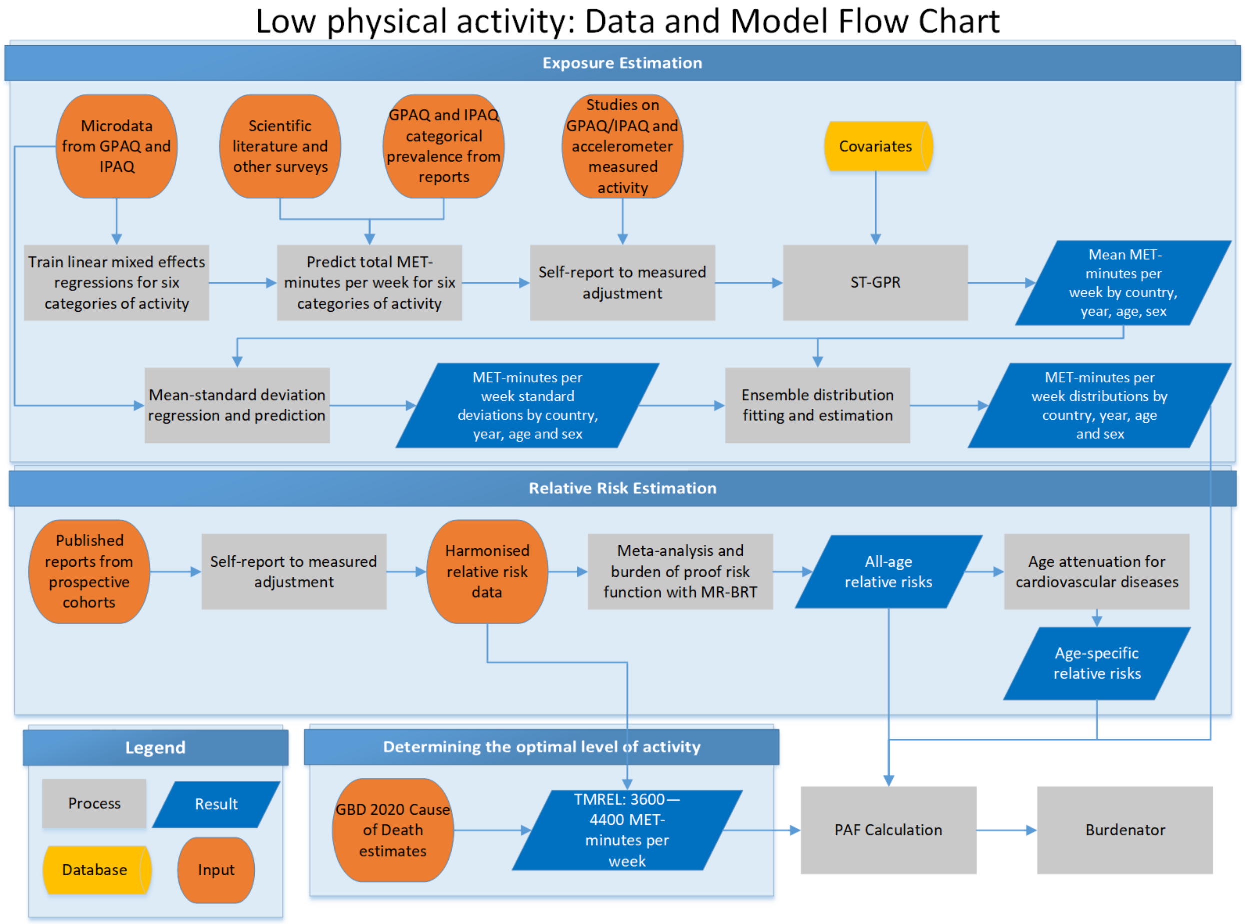

Input data and methodological summary

Definition

Exposure

Low physical activity is defined as objectively measured, total physical activity less than 3600 to 4400 MET-minutes per week. We assess physical activity performed by adults older than 25 years of age, for duration of at least ten minutes at a time, across all domains of life (leisure/recreation, work, household, and transport). We use frequency, duration, and intensity of activity to calculate total metabolic equivalent (MET)-minutes per week. MET is the ratio of the working metabolic rate to the resting metabolic rate. One MET is equivalent to 1 kcal/kg/hour and is equal to the energy cost of sitting quietly. A MET is also defined as the oxygen uptake in ml/kg/min with one MET equal to the oxygen cost of sitting quietly, around 3.5 ml/kg/min.

Input data

Exposure

We included surveys of the general adult population that captured self-reported physical activity in all domains of life (leisure/recreation, work/household, and transport), where random sampling was used.

Data were primarily derived from two standardised questionnaires: The Global Physical Activity Questionnaire (GPAQ)[1] and the International Physical Activity Questionnaire (IPAQ)[2], although we included other survey instruments that asked about intensity, frequency, and duration of physical activity performed across all activity domains.

Due to a lack of a consistent relationship on the individual level between activity performed in each domain and total activity, we were not able to use studies that only assessed recreational/leisure activity.

Physical activity level is categorised by total MET-minutes per week using four categories based on rounded values closest to the quartiles of the global distribution of total MET-minutes/week. The lower limit for the Level 1 category (600 MET-min/week) is the recommended minimum amount of physical activity to get any health benefit. We used four categories with higher thresholds rather than the GPAQ and IPAQ recommended three categories to better capture any additional protective effects from higher activity levels.

- Level 0: <600 MET-min/week (inactive)
- Level 1: 600–3999 MET-min/week (low-active)
- Level 2: 4000–7999 MET-min/week (moderately active)
- Level 3: ≥8000 MET-min/week (highly active)

The GHDx was used to locate all surveys that use the GPAQ or IPAQ questionnaire. Although there were many other surveys that focused specifically on leisure activity, we were unable to use these sources because they did not include all three domains (work, transport, and leisure). In addition, we excluded any surveys that did not report frequency, duration, and intensity of activity.

Table 1: Data inputs for exposure for low physical activity.

Input data	Exposure
Source-count (total)	261
Number of countries with data	131

Relative risk

In GBD 2021, we conducted an updated systematic review for studies published before December 31, 2019, evaluating the relationship between physical activity and risk of breast cancer, colon and rectum cancer, diabetes, ischaemic heart disease, and ischaemic stroke. We searched for studies in PubMed using the search strings reported in table 3. We included prospective cohort studies that assessed total physical activity or leisure-time physical activity as the exposure variable and at least one of the five diseases as an outcome. Further, we only included studies that reported risk estimates (relative risk,

hazard ratio, or odds ratio) with confidence intervals, standard errors, or enough information to quantify uncertainty. In addition, we only included studies that reported the frequency and duration of activity achieved, excluding studies that reported physical activity using categorical or custom component scores. In future rounds of the GBD, we aim to incorporate new evidence as it becomes available. In addition, we will evaluate the evidence of low physical activity and risk for additional disease endpoints and add these riskoutcome pairs if general GBD inclusion criteria are met.

Table 2: Data inputs for relative risks for low physical activity.

Input data	Relative risk
Source count (total)	100
Number of countries with data	17

Table 3: Search strings used to search PubMed database

Outcome	String
Breast cancer	physical activity[Title/Abstract] AND breast cancer [Title/Abstract] AND "humans"[MeSH Terms] AND English[lang] AND ("2014/10/01"[PDAT] : "2019/12/31" [PDAT])
Colon and rectum cancer	physical activity[Title/Abstract] AND colon cancer [Title/Abstract] AND "humans"[MeSH Terms] AND English[lang] AND ("2014/10/01"[PDAT] : "2019/12/31" [PDAT])
Type 2 diabetes	physical activity[Title/Abstract] AND type 2 diabetes[Title/Abstract] AND "humans"[MeSH Terms] AND English[lang] AND ("2014/10/01"[PDAT] : "2019/12/31" [PDAT])
	physical activity[Title/Abstract] AND noninsulin dependent diabetes mellitus [Title/Abstract] AND "humans"[MeSH Terms] AND English[lang] AND ("2014/10/01"[PDAT] : "2019/12/31" [PDAT])
	physical activity[Title/Abstract] AND niddm[Title/Abstract] AND "humans"[MeSH Terms] AND English[lang] AND ("2014/10/01"[PDAT] : "2019/12/31" [PDAT])
Ischaemic heart disease	physical activity[Title/Abstract] AND ischemic heart disease [Title/Abstract] AND "humans"[MeSH Terms] AND English[lang] AND ("2014/10/01"[PDAT] : "2019/12/31" [PDAT])

	physical activity[Title/Abstract] AND ischaemic heart disease [Title/Abstract] AND "humans"[MeSH Terms] AND English[lang] AND ("2014/10/01"[PDAT] : "2019/12/31" [PDAT])
	physical activity[Title/Abstract] AND coronary heart disease [Title/Abstract] AND "humans"[MeSH Terms] AND English[lang] AND ("2014/10/01"[PDAT] : "2019/12/31" [PDAT])
Ischaemic stroke	physical activity[Title/Abstract] AND ischemic stroke [Title/Abstract] AND "humans"[MeSH Terms] AND English[lang] AND ("2014/10/01"[PDAT] : "2019/12/31" [PDAT])
	physical activity[Title/Abstract] AND ischaemic stroke[Title/Abstract] AND "humans"[MeSH Terms] AND English[lang] AND ("2014/10/01"[PDAT] : "2019/12/31" [PDAT])

Data processing

Exposure

Mixed effects modelling

For this round of the GBD, we used six separate linear mixed effects regressions to capture the relationship of reported activity in total MET-minutes per week with the prevalence of the six activity categories (Table 4). We fit the models using tabulated individual level IPAQ and GPAQ data. The general form of the equation was as follows:

$$\ln(MET_i) = \beta_0 + \beta_1 cat_i + \beta_2 age_i + \beta_3 fem_i + \beta_4 IPAQ_i + \beta_5 IPAQ_i * cat_i + \alpha_s + \alpha_r + \alpha_c$$

where MET_i was the reported MET-minutes/week achieved; cat_i was the categorical prevalence of activity (ie, inactive, low/moderately/highly active, low active, moderately/highly active, moderately active, or highly active); age_i was the midpoint of the tabulated age group; fem_i was an indicator for whether the data were for females; $IPAQ_i$ was an indicator for whether the data were from an IPAQ survey; and $\alpha_s, \alpha_r, \alpha_c$ were nested random intercepts at the super-region, region, and country level, respectively. Once these coefficients were estimated, we applied the relationships to the rest of our categorical prevalence data from GPAQ and IPAQ to determine the corresponding levels of MET-minutes per week.

Table 4: Definitions of categorical prevalence of physical activity exposure data

Category	MET-min/week
inactive	<600
low/moderately/highly active	≥600
low active	600–3999
moderately/highly active	>4000
moderately active	4000–7999
highly active	≥8,000

Self-report adjustment

Our input data of MET-minutes per week were collected through self-reported questionnaires like GPAQ and IPAQ. Self-reported activity can lead to biased responses and result in exposure measurement error compared to other direct measurement tools (eg, accelerometer and doubly labelled water).[3] To best account for the measurement error, we relied on existing systematic reviews to identify studies that assessed the relationship between self-reported physical activity using GPAQ or IPAQ and accelerometer-measured physical activity.[4,5,6] We extracted 15 studies usable for analysis, collecting information on study name, location, age, sex, type of activity, self-report instrument, self-reported values of activity, accelerometer instrument, and accelerometer-measured values of activity. We used MR-BRT to model the log difference between activity reported through GPAQ or IPAQ questionnaires to activity measured with accelerometers. The results of our crosswalk adjustment are summarised in Table 5. The adjustment factor was then applied to the input data of self-reported total MET-minutes per week to approximate accelerometer-adjusted total MET-minutes per week.

Table 5: MR-BRT crosswalk adjustment factors for low physical activity

Data input	Reference or alternative case definition	Gamma	Beta coefficient, log (95% UI)*	Adjustment factor **
Accelerometer measured	Ref	0.707	---	---
Self-reported with GPAQ or IPAQ	Alt		0.730 (–0.721, 2.181)	2.075

**MR-BRT crosswalk adjustments can be interpreted as the factor the alternative case definition is adjusted by to reflect what it would have been had it been measured using the reference case definition. If the log/logit beta coefficient is negative, then the alternative is adjusted up to the reference. If the log/logit beta coefficient is positive, then the alternative is adjusted down to the reference.*

***The adjustment factor column is the exponentiated beta coefficient. For log beta coefficients, this is the relative rate between the two case definitions. For logit beta coefficients, this is the relative odds between the two case definitions.*

Modelling strategy

Exposure

Accelerometer-adjusted total MET-minutes per week data were then used as inputs into our spatiotemporal Gaussian process regression (ST-GPR) model to generate complete estimates of MET-minutes per week for every country, age group, and sex from 1990 to 2021. Further details on ST-GPR are available in the general methods appendix.

The linear model, which when added to the smoothed residuals forms the mean prior for GPR, is as follows:

$$\log(\mathrm{MET})_{c,a,t} = \beta_0 + \beta_1 \mathrm{agriculture}_{c,t} + \beta_2 \mathrm{vehicles}_{c,t} + \alpha_s + \alpha_r + \alpha_c$$

where $\mathrm{agriculture}$ *is the proportion of the population working in agriculture and* $\mathrm{vehicles}$ *is the number of four-wheeled vehicles per capita, and* α_s, α_r, and α_c are super-region, region, and country nested random intercepts, respectively. Random effects were used in model fitting but were not used in prediction.

Utilising microdata on total MET-minutes/week from individual-level surveys, we characterised the distribution of activity level at the population level. We then used an ensemble approach for distribution fitting, borrowing characteristics from individual distributions to tailor a unique distribution to fit the data using a weighting scheme. We characterised the standard deviation of each population's activity through a linear regression that captured the relationship between standard deviation and mean activity levels in nationally representative IPAQ surveys:

$$\begin{aligned} ln\,(Standard\ deviation) \\ = \beta_0 + \beta_1 \times ln\,(Mean_i) + \beta_2 \times Age_i + \beta_3 \times SR_i + \beta_4 \times Fem_i \end{aligned}$$

Age_i is the youngest age in population *i*'s age group, SR_i is the super-region in which the population lives, and Fem_i is a Boolean value depicting whether the population is female. We then applied the coefficients of this regression to the outputs of our estimate of total MET-minutes per week regression outputs to calculate the standard deviation by country, year, age, and sex.

Relative risk

In previous rounds of GBD, we used a dose–response meta-analysis of prospective cohort studies to estimate the effect size of the change in physical activity level on breast cancer, colon cancer, diabetes, ischaemic heart disease, and ischaemic stroke.[7] In GBD 2021, we conducted an updated systematic review and evaluated the relationship between physical activity and risk of the five disease endpoints.

We used MR-BRT to estimate the non-linear dose–response relationships between low physical activity and risk for breast cancer, colon cancer, diabetes, ischaemic heart disease and ischaemic stroke. Specifically, we used the evidence score framework to systematically determine the risk function and evaluate the strength of evidence for each risk–outcome pair. Further details on the evidence score framework are available in the general methods of the Appendix.

The non-linear inverse relationships between physical activity and risk for diseases has been well-defined.[7] We used the MR-BRT tool to estimate the log relative risk associated with each level of total physical activity on a continuous scale. Outcome-specific model characteristics are described in Table 6.

For each risk–outcome pair meta-regression, we considered study-level covariates that could potentially bias the study's reported effect size estimates. These study-level covariates included indication of whether the study used a washout period, whether the study determined outcomes based on administrative records or self-reports, the level of adjustment for relevant confounders like age, sex, smoking, education, and income, and whether the study adjusted for body-mass index (BMI) or another indicator for adiposity. We adjusted for these covariates in our meta-regression if they significantly biased our estimated relative risk function.

When available, we extracted and analysed effect sizes that controlled for BMI or another indicator for adiposity. We evaluated adjustment for BMI as a study-level variable in our covariate selection process described above. We found that this covariate was only significant in our physical activity – breast cancer model, suggesting that our predicted risk functions reflected the protective health effects of total physical activity independent of BMI.

We implemented the Fisher scoring correction to the heterogeneity parameter, which corrects for data-sparse situations. In such cases, the between-study heterogeneity parameter estimate may be 0, simply from lack of data. The Fisher scoring correction uses a quantile of gamma, which is sensitive to the number of studies, study design, and reported uncertainty.

We also added methodology to detect and flag publication bias. The approach is based on the classic Egger's regression strategy, which is applied to the residuals in our model. In the current implementation, we do not correct for publication bias, but flag the risk–outcome pairs where the risk for publication bias is significant. We found no evidence of publication bias for the outcomes associated with physical activity.

There is a well-documented attenuation of the risk for cardiovascular disease due to metabolic risks factors throughout one's life.[8] To incorporate this age trend in the relative risks, we first identified the median age-at-event across all cohorts and considered that as the reference age group. We then assigned our risk curves to this reference age group. Then, we derived attenuation factors for each metabolic mediator (ie, systolic blood pressure and total cholesterol) by taking the ratio of excess risk between each age group and the reference. Finally, we applied 1000 draws of the age-specific attenuation factors averaged across the two metabolic mediators to 1000 draws of the reference age group's risk curve to determine age-specific risk curves that propagated the uncertainty of both the risk function and age pattern.

Table 6: Model characteristics for outcomes related to high body-mass index in adults

Outcome	Non-linear specifications and constraints	Selected covariates	Mean gamma solution	Publication bias
Breast cancer	*	Adjustment for BMI	0.000	No
Colon and rectum cancer	*		0.000	No
Diabetes mellitus type 2	**		0.105	No
Ischaemic heart disease	**		0.079	No
Ischaemic stroke	*		0.015	No

** Quadratic splines with 4 knots; right linear tail; monotonically decreasing constraint; Gaussian prior (0, 0.01) on max derivative of non-linear intervals*

*** Quadratic splines with 5 knots; right linear tail; monotonically decreasing constraint; Gaussian prior (0, 0.01) on max derivative of non-linear intervals*

Theoretical minimum risk exposure level

The theoretical minimum risk exposure level for physical inactivity is 3600–4400 MET-minutes per week. We calculated this range by taking the weighted average of the 85th percentile of activity achieved across all five risk–outcome pairs. We first determined the 85th percentile of activity for a given study by taking the 85th percentiles of the lower bound and midpoints of the alternative comparison groups. For studies where the reference group was the highest level of activity, we instead took the 85th percentile of the lower bound and midpoint of the reference exposure range. Next, we took the simple average of the 85th percentile ranges across all the studies for a given riskoutcome pair to derive five cause-specific ranges. Finally, using the GBD 2021 Causes of Death analysis, we used the number of global deaths in the year 2021 for adults aged 25+ as weights to estimate our weighted average of the minimum risk level.

References

1. World Health Organization. Global Physical Activity Questionnaire (GPAQ) Analysis Guide. 2011. Geneva, Switzerland: WHO Google Scholar. 2013
2. IPAQ Research Committee. Guidelines for data processing and analysis of the International Physical Activity Questionnaire (IPAQ)–short and long forms. Retrieved September. 2005;17:2008.
3. Shephard RJ. Limits to the measurement of habitual physical activity by questionnaires. *Br J Sports Med* 2003; **37**(3): 197—206.

4. Prince SA, Adamo KB, Hamel ME, Hardt J, Connor Gorber S, Tremblay M. A comparison of direct versus self-report measures for assessing physical activity in adults: a systematic review. *Int J Behav Nutr Phys Act* 2008; 5–56.
5. Skender S, Ose J, Chang-Claude J, et al. Accelerometry and physical activity questionnaires - a systematic review. *BMC Public Health* 2016; 16–515.
6. Sember V, Meh K, Sorić M, Starc G, Rocha P, Jurak G. Validity and Reliability of International Physical Activity Questionnaires for Adults across EU Countries: Systematic Review and Meta Analysis*. Int J Environ Res Public Health* 2020; **17**(19): 7161.
7. Kyu HH, Bachman VF, Alexander LT, et al. Physical activity and risk of breast cancer, colon cancer, diabetes, ischemic heart disease, and ischemic stroke events: systematic review and dose-response meta-analysis for the Global Burden of Disease Study 2013. *BMJ* 2016; 354:i3857.
8. Singh GM, Danaei G, Farzadfar F, et al. The age-specific quantitative effects of metabolic risk factors on cardiovascular diseases and diabetes: a pooled analysis. *PLoS One* 2013; **8**(7)

No access to handwashing facility

Flowchart

Unsafe Handwashing

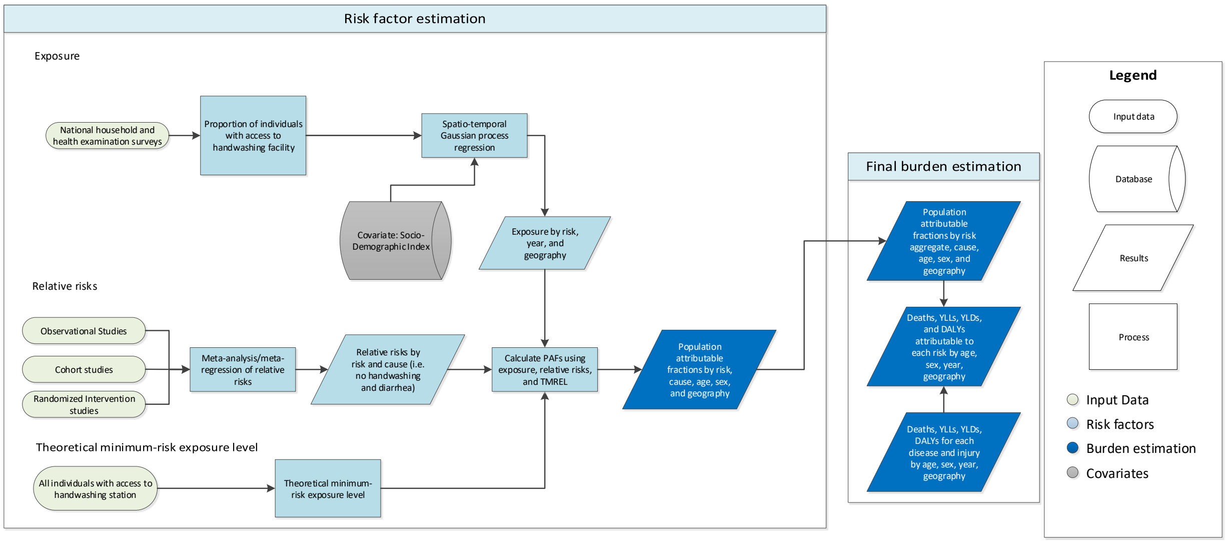

Input data and methodological summary

Exposure

Case definition

This risk is defined as the proportion of the population without access to a handwashing facility with soap (bar, liquid, or powder/detergent), water, and wash station (either permanent or mobile).[1] If any of these is missing, then the individual is counted as not having access.

Input data

Input data came primarily from geographically representative household surveys, including the Demographic and Health Surveys (DHS), Multiple Indicator Cluster Surveys (MICS), and Performance Monitoring and Accountability 2020 (PMA2020) surveys. For the GBD 2021 study, a large number of new data sources were added, nearly doubling the total number of sources from 98 in GBD 2019 to 177 in GBD 2021. We also re-extracted all sources previously used to ensure that we were capturing individual-level exposure, in an effort to align with the WHO/UNICEF Joint Monitoring Programme's methodology.[1] As a result of this effort, we excluded several studies used in previous GBD rounds that included ash, sand, or soil in their definition of "soap." Table 1 provides a summary of the exposure input data.

Table 1: Exposure input data

Input data	Exposure
Source count (total)	177
Number of countries with data	89

Modelling strategy

We modelled exposure to this risk using a three-step modelling scheme of mixed effect linear regression followed by spatiotemporal Gaussian process regression (ST-GPR), which outputs full time-series

estimates for each GBD 2021 location. Two covariates were used as fixed effects in the linear regression: Socio-demographic Index (SDI), which is a composite measure of development that includes income per capita, education, and fertility, and proportion of individuals with access to piped water (see below for model equation). Random effects were set at GBD 2021 region and super-region levels to fit the model but were not used in the predictions.

$$logit(data) \sim sdi + piped\ water\ access + (1|level\_1) + (1|level\_2)$$

SDI = Socio-demographic Index
Piped water access = proportion of individuals with access to piped water
(1|level_1) = super-region-level random effects
(2|level_2) = region-level random effects

The process of vetting and validating models was accomplished primarily through an examination of ST-GPR scatterplots by GBD 2021 location from 1990 to 2021. Any poorly fitting datapoints were re-inspected for error at the level of extraction and survey implementation. If errors in data extraction were found, the study in question was re-extracted. In addition to SDI, a number of different potential fixed effects were considered, including lag-distributed income and urbanicity. However, SDI proved to be the strongest predictor.

Theoretical minimum-risk exposure level

The theoretical minimum-risk exposure level for unsafe hygiene is defined as having access to a handwashing facility with soap (bar, liquid, or powder/detergent), water, and wash station (either permanent or mobile).

Relative risks

Input data

Input data included in the GBD 2021 hygiene relative risk analysis are as follows:

Table 2: Relative risk input data

Input data	Relative risk
Source count (total)	41
Number of countries with data	22

For GBD 2021, unsafe hygiene was paired with two outcomes: diarrhoeal diseases and lower respiratory infections (LRI). A meta-analysis by Cairncross and colleagues 2010[2] provided relative risk values describing the relationship between lack of facility access and diarrhoeal diseases. A meta-analysis by Rabie & Curtis 2006[3] provided relative risk evidence for the relationship between lack of facility access and LRI, including the years 1997-2004.

Additionally, a literature review on the relationship between hygiene and LRI was conducted in GBD 2021, which added 15 new studies (Figure 1). A meta-analysis by Jefferson et al 2011[4] was included in the literature review, covering the years 2004-2010. Thirty-five studies were identified from Jefferson et al 2011[4] that matched our criteria, of which nine were already extracted in previous rounds. After initial title/abstract screening and full-text review, eight new studies were added. We then searched PubMed

for updates to relevant literature published from January 1, 2010 to August 20, 2020 (date of search), using the search string below:

("Hand Hygiene"[MESH] OR "handwashing"[TiAb] OR "hand hygiene"[TiAb] OR "hand cleansing"[TiAb] OR "hand cleaning"[TiAb]) AND ("lower respiratory tract infection"[TiAb] OR "LTRI"[TiAb] OR "lower respiratory infection"[TiAb] OR "acute respiratory infection" [TiAb] OR "sinusitis"[TiAb] OR "common cold"[TiAb] OR "otitis media"[TiAb] OR "pharyngitis"[TiAb] OR "influenza"[TiAb] OR "coryza"[TiAb] OR "laryngitis"[TiAb] OR "epiglottis"[TiAb] OR "croup"[TiAb] OR "pneumonia"[TiAb] OR "bronchitis"[TiAb] OR "bronchiolitis"[TiAb] OR "pertussis"[TiAb] OR "whooping cough"[TiAb] OR "pneumococcal pneumonia "[TiAb] OR "influenza"[TiAb] OR "respiratory syncytial virus"[TiAb] OR "h influenzae type b"[TiAb] OR "Respiratory Tract Infections"[MESH] OR "Otitis Media"[MESH] OR "Respiratory Syncytial Viruses"[MESH] OR "Croup"[MESH] OR "Haemophilus influenzae type b"[MESH]) AND (2010[PDAT]:3000[PDAT]) NOT(animals[MESH] NOT humans[MESH])

Figure 1: PRISMA diagram for systematic review of hygiene and lower respiratory infection (LRI)

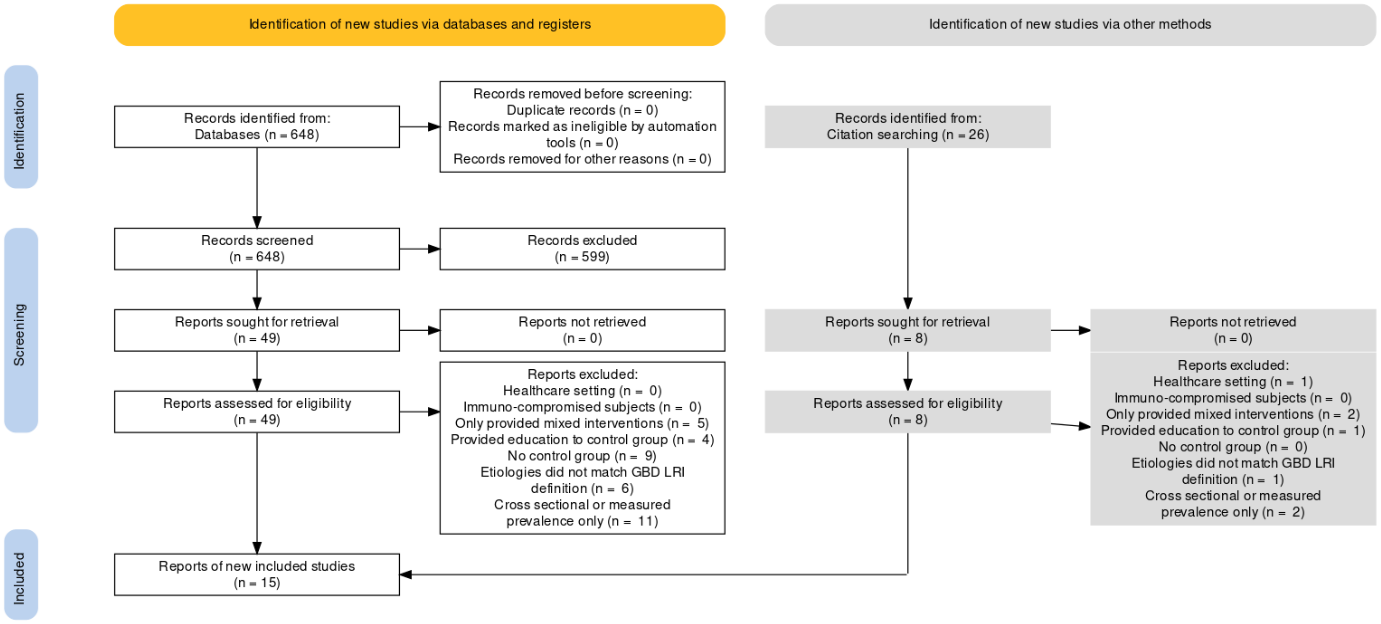

Modelling strategy

In GBD 2021, relative risk values were calculated using a tool called meta-regression—Bayesian, regularised, trimmed (MR-BRT). For the both the diarrhoea model and the LRI model, two study-level covariates were included – whether or not the study was randomised and whether or not the percentage of the study population lost to follow-up was greater than 15%. No priors were used. Table 3 shows the results of the relative risk analyses.

Table 3: Relative risks (reference: access to handwashing facility)

Outcome	Relative risk (95% CI)

Diarrhoeal diseases	1.52 (1.06–2.12)
Lower respiratory infections	1.43 (0.82–2.30)

Figures 2 and 3 show the funnel plots for each MR-BRT analysis, along with the associated “risk-outcome scores,” which measure how good the evidence is for that particular relative risk estimate. Prior to generating an risk-outcome score, we conducted an additional post-analysis step to detect and flag publication bias in the input data. This approach is based on the classic Egger’s regression strategy, which is applied to the residuals in our model. In the current implementation, we do not correct for publication bias, but flag the risk–outcome pairs where the risk for publication bias is significant.

For hygiene and diarrhoea, we detected publication bias based on the association between observation residuals and their standard errors (p-value = 0.0265, Egger mean = -0.302, Egger SD = 0.156). The risk-outcome score was 0.000828.

Figure 2: Hygiene–diarrhoea funnel plot and risk-outcome score

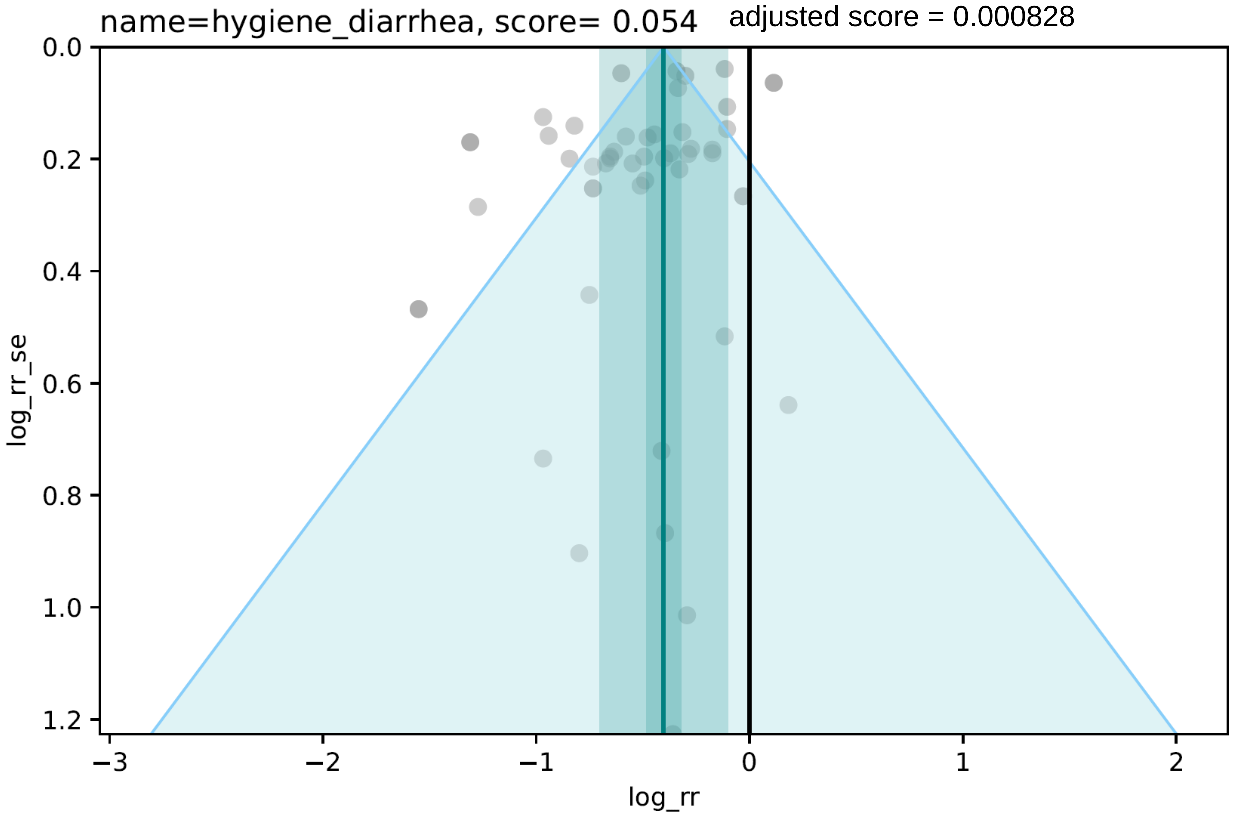

For hygiene and LRI, we did not detect publication bias (p-value = 0.379, Egger mean = -0.0570, Egger SD = 0.186). The risk-outcome score was -0.060.

Figure 3: Hygiene–LRI funnel plot and risk-outcome score

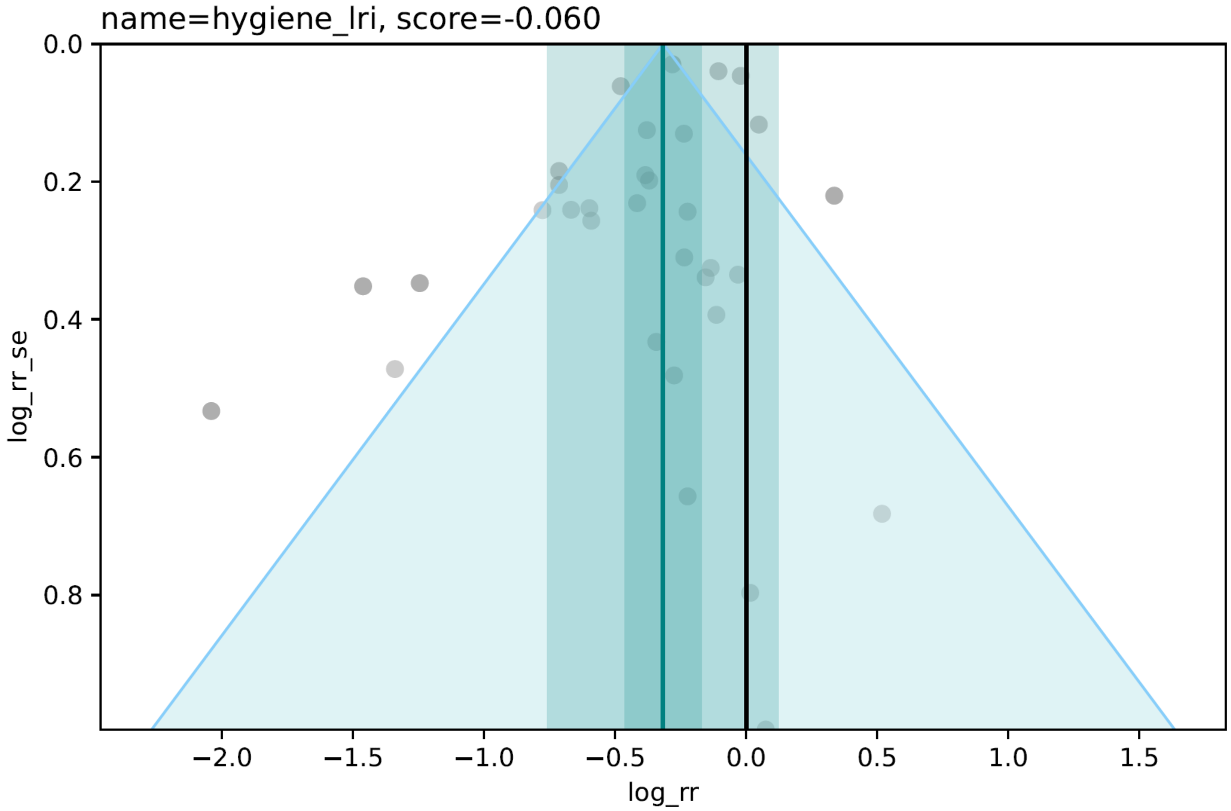

References

1. WHO/UNICEF. JMP Methodology: 2017 update and SDG baselines. 2018.

2. Cairncross S, Hunt C, Boisson S, *et al.* Water, sanitation and hygiene for the prevention of diarrhoea. *International Journal of Epidemiology* 2010; **39**: i193–205.

3. Rabie T, Curtis V. Handwashing and risk of respiratory infections: a quantitative systematic review. *Tropical Medicine and International Health* 2006; **11**: 258–67.

4. Jefferson T, Del Mar CB, Dooley L, Ferroni E, Al-Ansary LA, Bawazeer GA, van Driel ML, Jones MA, Thorning S, Beller EM, Clark J, Hoffmann TC, Glasziou PP, Conly JM. Physical interventions to interrupt or reduce the spread of respiratory viruses. Cochrane Database of Systematic Reviews 2020, Issue 11. Art. No.: CD006207. DOI: 10.1002/14651858.CD006207.pub5. Accessed 13 April 2022.

Non-optimal temperature

Flowchart

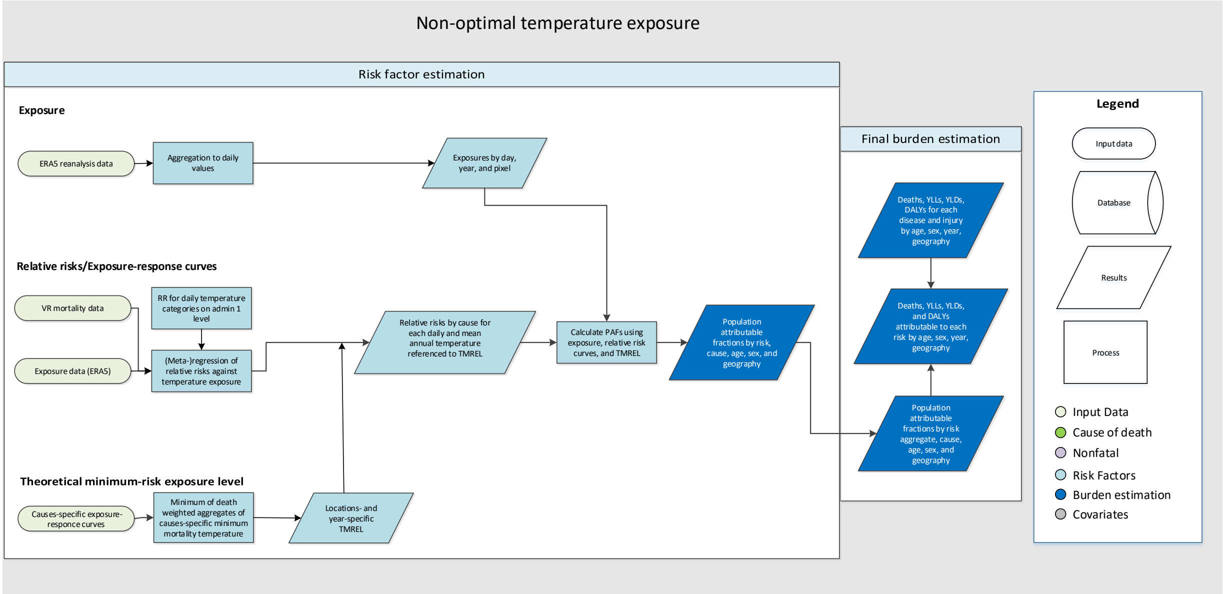

Input data and methodological summary

Definition

The non-optimal temperature risk factor is defined as the same-day exposure to ambient temperature that is either warmer or colder than the temperature of minimum mortality risk. Specifically, we define the theoretical minimum risk exposure level (TMREL) for non-optimal temperature as the temperature that is associated with the lowest overall mortality attributable to this risk, in a given location and year. Given varying exposure–response curves for different mean annual temperature zones, as well as spatially and temporally varying cause of death composition, we estimate TMRELs by year and location and not a globally uniform TMREL. High temperature (heat) exposure is defined as exposure to temperatures warmer than the TMREL, and low temperature (cold) is defined as temperatures colder than the TMREL.

Exposure

We assess the daily exposure to non-optimal temperature, which is defined for high temperatures (above the TMREL) and low temperatures (below TMREL). TMRELs for non-optimal temperature exposure vary by year and location and reflect location-specific adaptation to temperature as well as specific composition of causes of death.

Input data

ERA5 data

We derived exposure estimates from the ERA5 reanalysis dataset from the European Centre for Medium-Range Weather Forecasts (ECMWF). ECMWF produced ERA5 estimates using their Integrated Forecast System (IFS). Hourly values of surface temperature are available for a spatial resolution of 0.25° x 0.25°. Uncertainty estimates for these temperature values, ie, the ensemble spread (standard

deviation) is available for every three hours (00:00, 03:00, 06:00, 09:00, 12:00, 15:00, 18:00, 21:00) for a spatial resolution of 0.5° x 0.5°. At the time of analysis, data were available from 1979 to December 2021.[1,2] We calculated daily averages of temperature and spread for each pixel and then assigned an uncertainty value to each daily temperature value. Based on the spread we derived 1000 draws of each daily temperature pixel.

Population data

Population data for calculating population-weighted location means were derived from WorldPop, which is an open-source project initiated in 2013.[3] Multi-temporal, globally consistent, high-resolution human population data at 1 km x 1 km resolution can be downloaded from http://www.worldpop.org.uk/ for 2000, 2005, 2010, 2015, and 2020. For the purpose of our work, we interpolated in between the five-year estimation bins to obtain annual data. Further, we extrapolated until 1990 by using the 2000–2005 growth rate for back-casting.

Mortality data

Deaths at the individual level that included information regarding the cause (ie, ICD code), date, and the location at the second administrative level (admin2) or finer were collected from the Global Burden of disease (GBD) cause-of-death (CoD) database for vital registration data sources. We adapted the GBD standard procedure for garbage code redistribution to redistribute daily mortality data rather than annual data and mapped ICD causes to GBD causes for Level 3. In total, we analysed 64.9 million deaths from nine different countries and 15,198 administrative units. For Brazil, the data cover a period from 1999 to 2016 for 5570 municipalities and 19.9 million deaths. For Chile, the data cover the period from 1990 to 1996 and 2009 to 2011 for 15 regions and 0.82 million deaths. For Colombia, the data cover a period from 2001 to 2005 for 1125 municipalities and 0.95 million deaths. For Guatemala, the data cover a period from 2009 to 2016 for 333 municipalities and 0.49 million deaths. For Mexico, the data cover a period from 1996 to 2015 for 2438 municipalities and 9.88 million deaths. For New Zealand, the data cover a period from 1988 to 2014 for 20 district health boards and 0.76 million deaths. For South Africa, the data cover the years 1997 to 2016 for one province and 1.8 million deaths. For the United States, the data cover a period from 1980 to 1988 for 3140 municipalities and 18.1 million deaths. For China, the data cover the years 2015 to 2016 for 2556 counties and 12.2 million deaths.

Table 1: Data inputs for exposure for non-optimal temperature

Input data	Exposure
Total sources	6324
Number of countries with data	204
Number of GBD regions with data (out of 21 regions)	21
Number of GBD super-regions with data (out of 7 super-regions)	7

Table 2: Data inputs for relative risks for non-optimal temperature

Input data	Relative risk
Total sources	127
Number of countries with data	9
Number of GBD regions with data (out of 21 regions)	7
Number of GBD super-regions with data (out of 7 super-regions)	4

Modelling strategy

To estimate cause-specific mortality, based on average daily temperature and temperature zone (defined by mean annual temperature), we used a robust meta-regression framework, implemented through the MR-BRT (Bayesian, regularised, trimmed) tool. The tool allows three features that are essential to the analysis:[4]

- A meta-analytic framework that can handle heterogeneous data sources
- A robust approach to outlier detection and removal (trimming)
- Specification of the functional dependence of outcome versus average daily temperature and temperature zone as a two-dimensional surface through a spline interface.

The use of trimming in a vast array of inference and machine learning problems is standard.[5–7] The use of high-dimensional splines has been proposed before,[8] but the methods used for estimation go beyond prior work, and we explain them below.

The functional relationship between any outcome y and input variables (t1, t2) models y as a linear combination of 2d spline basis elements. Each spline basis element is a product of individual basis elements for 1D splines for t1 and t2. Therefore, the inference problem looks for a combination of simple curvilinear 2D elements that fit the data while preserving smoothness across element boundaries. The MR-BRT tool also allows prior information to influence the shape of the spline, particularly in areas with sparse data.

For the purpose of modelling the relationship between mortality and mean annual and daily temperature, we imposed monotonicity in the direction of daily temperature. For all J-shaped curves that depicted an increase in mortality above and below a threshold, we forced the curve to monotonically decrease at the lower end of the temperature distribution and to monotonically increase at the upper end. For all external causes that displayed a monotonic increase over the entire temperature range, we imposed monotonicity only in the direction of warmer temperatures. We placed two knots of degree 3 in the direction of mean annual temperature when fitting the surface. In the direction of daily mean temperature, we placed three knots of degree 3 for J-shaped causes and two knots of degree 1 for external causes that monotonically increase over temperature range. Figure 1 shows an example of a relative risk (RR) surface along daily and annual mean temperature for drowning. We extrapolated daily temperature-mortality curves beyond the range of observed temperatures by either linear interpolation, in the case of monotonically increasing response relationships (observed for

external causes) or cubic polynomial extrapolation for J-and V-shaped curves (observed for non-external causes).

We estimated uncertainty using a two-step approach. First, we derived the uncertainty of the mean surface from the measurement error using the fit-retrofit error. Second, we added uncertainty from the random effects by sampling it separately from the cold and warm side.

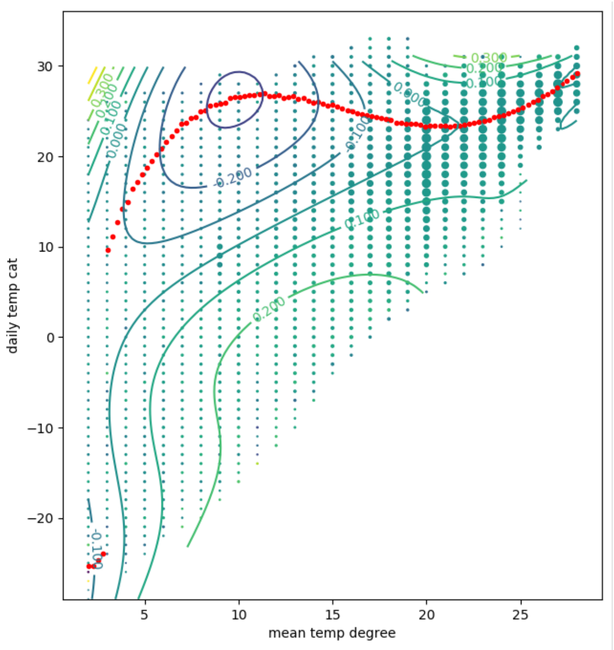

Figure 1: Log relative risk of death from lower respiratory infection along mean annual temperature (mean temp cat) and daily mean temperature (daily temp cat). The red dotted line depicts minimum mortality temperature along mean annual temperature zones. Green and blue lines depict isopleths, ie, lines of equal log RR of mortality

Cause selection

We assessed the effect of temperature on all causes included in Level 3 of the GBD cause hierarchy (N=176). We then reduced the set of potential causes by excluding causes for which no deaths were recorded in our mortality dataset (N=44) or for which classification practices are highly inconsistent across countries, which applied to dementia and protein-energy malnutrition (N=2).[30] We estimated RR surfaces and uncertainties for all remaining (N=130) Level 3 causes. Cause selection or inclusion was based on a risk-outcome score developed for evaluating risk-outcome pairs in the GBD study. To derive this risk-outcome score, we normalised the risk curve for each cause and each temperature zone so that the RR was equal to 1·0 at the temperature associated with the lowest risk, and log-transformed the resulting normalised curves. The risk-outcome score is based on the area between the lower bound of the 95% UI and the null (ie, log-RR of 0·0). Regions of the risk curve for which the 95% UI includes the null will produce negative scores, and regions that exclude the null will produce positive scores; a score of zero indicates that the lower bound is equal to the null. We then averaged scores across all climate

zones for each individual cause and included all causes for which the mean risk-outcome score exceeded zero. A table with average risk-outcome scores for individual causes is provided below:

Table 3: Average risk-outcome scores across climate zones for individual causes. Causes with an average score above 0 were included into the GBD.

Rank	Cause (level 3 [a])	Parent cause (level 2 [a])	Cause label (level 3 [a])	Score average
1	Drowning	Unintentional injuries	inj_drowning	0.361087
2	Other unintentional injuries	Unintentional injuries	inj_othunintent	0.269039
3	Lower respiratory infections	Respiratory infections and tuberculosis	lri	0.118761
4	Chronic obstructive pulmonary disease	Chronic respiratory diseases	resp_copd	0.105613
5	Animal-related injuries	Unintentional injuries	inj_animal	0.09697
6	Ischaemic heart disease	Cardiovascular diseases	cvd_ihd	0.08433
7	Stroke	Cardiovascular diseases	cvd_stroke	0.071678
8	Suicide	Self−harm and interpersonal violence	inj_suicide	0.070617
9	Disaster-related injuries	Unintentional injuries	inj_disaster	0.068935
10	Homicide	Self−harm and interpersonal violence	inj_homicide	0.068804
11	Mechanical injuries	Unintentional injuries	inj_mech	0.066174
12	Other transport-related injuries	Transport injuries	inj_trans_other	0.052465
13	Hypertensive heart disease	Cardiovascular diseases	cvd_htn	0.05067
14	Transport-related injuries	Transport injuries	inj_trans_road	0.048922
15	Diabetes	Diabetes and kidney diseases	diabetes	0.044704
16	Cardiomyopathy and myocarditis	Cardiovascular diseases	cvd_cmp	0.044378
17	Chronic kidney disease	Diabetes and kidney diseases	ckd	0.032174

Theoretical minimum-risk exposure level

For the purpose of this analysis, the TMREL was defined as the temperature associated with the lowest mortality for all included causes. We calculated a death-weighted average of the cause-specific exposure–response curves with the minimum of this average curve being the TMREL. This was done for each year and each of the 990 GBD locations using CoD estimates produced for the GBD 2019 study. As climate zones or mean annual temperature can vary within a location, we calculated the TMREL for every mean annual temperature, assuming a consistent cause composition within a location. This approach represents the first use of spatially and temporally varying TMRELs within the GBD study.

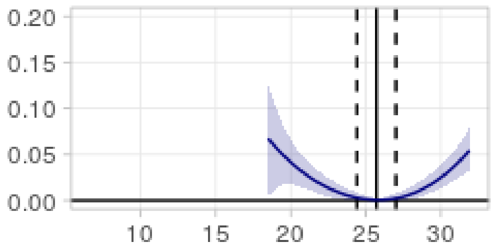

Figure 3: Schematic illustration of the exposure–response relationship between temperature and mortality and associated low temperature (cold) and high temperature effects beyond the theoretical minimum exposure level (TMREL). The blue line depicts the exposure–response curve with blue shaded line showing 95% uncertainty range. The black solid line depicts the TMREL with dashed black lines displaying 95% uncertainty range. Effects left of the TMREL are counted towards cold PAFs and right of the TMREL towards heat PAFs.

Relative risk

We followed the GBD 2019 methods for estimating relative risks.

Population attributable fractions

The population attributable fraction (PAF) was calculated for each temperature pixel and each day of the year (ie, pixel-day). Subsequently, we population-weighted each pixel using the fraction of the population living in a given pixel relative to the GBD location. Depending on whether the daily mean temperature was below or above the TMREL, the effect was assigned to either low or high temperature. Daily population-weighted high and low temperature PAFs were then aggregated for the location and the year. Temperature effects can be either harmful or protective depending on whether the RR is above or below 1. For harmful temperature effects, ie, effects with a RR above 1, we used the following equation to derive PAFs: PAF=(RR-1)/RR; For temperature effects exhibiting a protective effect, the equation was adapted by implementing the reverse RR: PAF=-((1/RR)-1)/(1/RR). The PAF associated with non-optimal temperature exposure is an aggregate, ie, the sum of heat and cold effects in each location and year. We estimated the temperature-attributable burden as the product of the total burden for that cause and the corresponding PAF for each GBD location, year, age group, and sex.

Citations

1 Hersbach H, Bell B, Berrisford P, *et al.* Global reanalysis : goodbye Global reanalysis : goodbye ERA-Interim , hello. 2019. DOI:10.21957/vf291hehd7.

2 Copernicus Climate Change Service (C3S) (2017): ERA5: Fifth generation of ECMWF atmospheric reanalyses of the global climate. Copernicus Climate Change Service Climate Data Store (CDS), September 2019.

3 Geography and Environmental Science, University of Southampton. Age and Sex Structures, Global Per Country 2000-2020 - WorldPop. Southampton, United Kingdom: Geography and Environmental Science, University of Southampton, 2018.

4 Zheng P, Aravkin AY, Barber R, Sorensen RJD, Murray CJL. Trimmed Constrained Mixed Effects Models: Formulations and Algorithms. 2019; published online Sept 23.

5 Rousseeuw PJ. Least Median of Squares Regression. *Journal of the American Statistical Association* 1984; **79**: 871–80.

6 Aravkin A, Davis D. Trimmed Statistical Estimation via Variance Reduction. *Mathematics of Operations Research* 2019; : moor.2019.0992.

7 Yang E, Lozano AC, Aravkin A. A general family of trimmed estimators for robust high-dimensional data analysis. *Electronic Journal of Statistics* 2018; **12**: 3519–53.

8 Pya N, Wood SN. Shape constrained additive models. *Statistics and Computing* 2015; **25**: 543–59.

Occupational risk factors

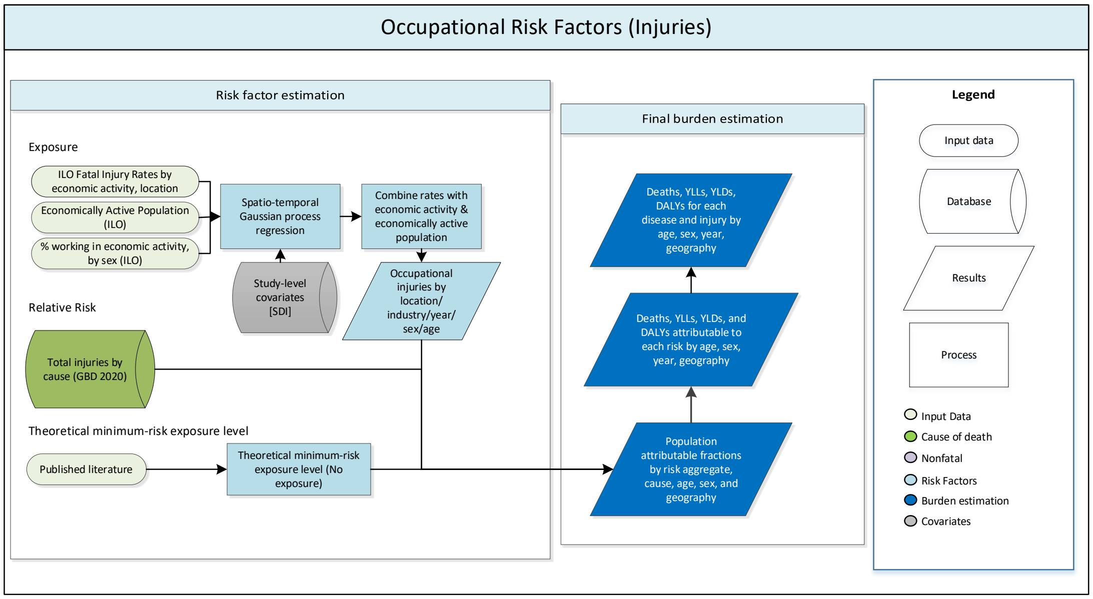

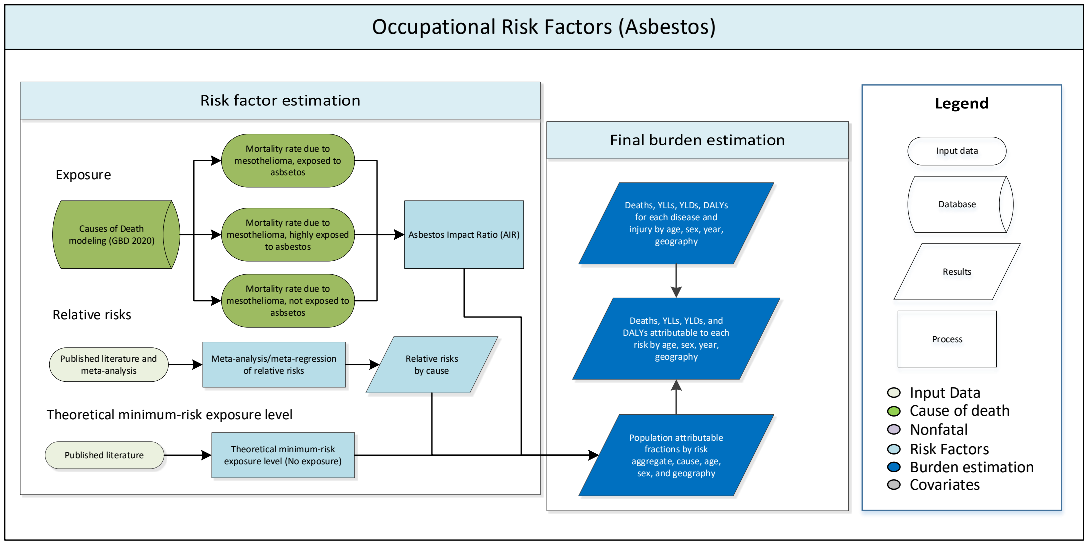

Exposure

The following definitions were used for occupational risk factor exposures. All exposures were estimated for ages 15 and older.

Occupational asbestos	Proportion of the population occupationally exposed to asbestos, using the mesothelioma death rate as an analogue
Occupational asthmagens	Proportion of the working population exposed to asthmagens, based on population distributions across nine occupational categories (listed below)

Occupational carcinogens (arsenic, benzene, beryllium, cadmium, chromium, diesel engine exhaust, formaldehyde, nickel, polycyclic aromatic hydrocarbons, silica, sulfuric acid, and trichloroethylene)	Proportion of the population that was ever occupationally exposed to carcinogens at high or low exposure levels, based on population distributions across 17 economic activities (listed below)
Occupational ergonomic factors	Proportion of the working population exposed to work that causes low back pain, based on population distributions across nine occupational categories
Occupational injuries	Proportion of injuries in the working-age population attributable to occupation, based on fatal injury rates in 17 economic activities
Occupational noise	Proportion of the population occupationally exposed to 85+ decibels of noise, based on population distributions across 17 economic activities
Occupational particulates	Proportion of the population occupationally exposed to particles, based on population distributions across 17 economic activities

Economic activities and occupations were coded according to the following categories:

**Economic activities**
Agriculture, hunting, forestry
Fishing
Mining and quarrying
Manufacturing
Electricity, gas, and water
Construction
Wholesale and retail trade/repair
Hospitality
Transport, storage, and communication
Financial intermediation
Real estate/renting
Public administration/defense; compulsory social security
Education
Health and social work
Other community/social/personal service activities
Private households
Extra-territorial organisations/bodies

**Occupational categories**
Legislators, senior officials, and managers

Professionals
Technicians and associate professionals
Clerks
Service workers and shop/market sales workers
Skilled agricultural and fishery workers
Plant and machine operators and assemblers
Craft and related workers
Elementary occupations

Input data

Primary inputs were obtained from the International Labour Organization (ILO).[1-4] These inputs included raw data on economic activity proportions, occupation proportions, fatal injury rates, and employment to population ratio estimates. No data on informal employment was included due to data sparseness. In 2017, a systematic review was conducted to collect the underlying microdata from the ILO's estimates to aid in re-extraction at greater levels of granularity. Where freely available, survey datasets were downloaded from the survey organisations in question. Other datasets were obtained through submission of requests to agencies and through the GBD collaborator network. Microdata were tabulated in order to create survey-weighted estimates of economic activities and occupations for the GBD geographies and years. Various classification systems were adjusted to match the ISIC Rev.3 classification (for economic activities) and ISCO 1988 classification (for occupations). For the current GBD cycle, we updated our ILO data by downloading the most recent data files from their website.

For occupational asbestos, primary inputs were obtained through GBD 2021 cause of death estimates and published studies.[7,13,14]

Uncertainty for inputs where microdata were unavailable was generated by fitting a Loess curve to the data and determining the standard deviation of the data from the fitted curve.

Table 1 provides a summary of the exposure input data used.

Table 1: Data inputs for exposure

Input data	Exposure
Source count (total)	5173
Number of countries with data	197

Modelling strategies

A spatiotemporal Gaussian process regression (ST-GPR) was used to generate estimates for all years and locations for the primary inputs. Space-time parameters were chosen by maximising out-of-sample cross-validation and minimising RMSE. A number of different study-level covariates were used in the linear regression models. The linear models for each of the 46 different ST-GPR models used in occupational exposure estimation are listed below. Although there might appear to be duplicates, there is a distinction between occupation and economic activity (detailed in the footnotes). For example, "skilled agriculture/fisheries" involves the proportion of the workforce doing agricultural and fishing work, while "agriculture, hunting, forestry" and "fishing" involve the proportion of the workforce employed in those respective industries (ie, one doesn't have to be actually doing agricultural or fishing

work – someone who transports crops would count as being employed in this industry, but their occupation would fall under "plant and machine operators & assemblers"). Additionally, each model included random effects at the region and super-region levels. The covariates are explained in greater detail below.

**ST-GPR model**	**Linear regression equation**
Employment (% of population employed)	$logit(data) = gov\_exp + prop\_muslim + education$
Armed forces*	$logit(data) = sdi + education + urbanicity$
Management*	$logit(data) = sdi + education + urbanicity$
Professional occupations*	$logit(data) = sdi + education + urbanicity$
Scientific/technicians*	$logit(data) = sdi + education + urbanicity$
Clerical work*	$logit(data) = sdi + education + urbanicity$
Service & shop/market sales workers*	$logit(data) = sdi + education + urbanicity$
Skilled agriculture/fisheries*	$logit(data) = sdi + latitude + urbanicity$
Craft and related trades*	$logit(data) = sdi + education + urbanicity$
Plant and machine operators & assemblers*	$logit(data) = sdi + education + urbanicity$
Elementary occupations*	$logit(data) = sdi + education + urbanicity$
Agriculture, hunting, forestry†	$logit(data) = sdi + latitude + urbanicity$
Fishing†	$logit(data) = \log(coastal\_prop + 0.01)$
Mining/quarrying†	$logit(data) = sdi + \log(coastal\_prop + 0.01) + urbanicity + asbestos$
Manufacturing†	$logit(data) = sdi + education + urbanicity$
Electricity/gas/water supply†	$logit(data) = \log(sdi) + urbanicity + temperature$
Construction†	$logit(data) = sdi + urbanicity$
Wholesale and retail trade/repair†	$logit(data) = sdi + education + urbanicity$
Hospitality†	$logit(data) = sdi + urbanicity$
Transport/storage/communications†	$logit(data) = sdi + urbanicity + vehicles\_pc$
Financial intermediation†	$logit(data) = sdi + urbanicity$
Real estate/renting†	$logit(data) = sdi + urbanicity$
Public administration/defence†	$logit(data) = sdi + urbanicity$
Education†	$logit(data) = sdi + education + urbanicity$
Health and social work†	$logit(data) = \log(sdi) + \log(health\_exp)$
Other community/social/personal service activities†	$logit(data) = sdi + urbanicity$
Private households†	$logit(data) = sdi + urbanicity$
Extraterritorial organisations and bodies†	$logit(data) = sdi + urbanicity$
All occupational injuries models‡	$logit(data) = sdi$

**Proportion of workforce working this type of occupation*

†Proportion of workforce employed in this type of economic activity

‡There are 18 different models, corresponding to one for each type of economic activity and a "total" model

**Covariate**	**Description**
gov_exp	Total government expenditure
prop_muslim	Proportion of population that is Muslim

education	Age-standardised years of education per capita
sdi	Socio-demographic Index
urbanicity	Proportion of population living in urban areas
latitude	Absolute value of average latitude of country's center point
coastal_prop	Percentage of total country area within 10 km of a coastal zone
asbestos	Asbestos consumption (metric tons per year per capita)
temperature	Population-weighted mean temperature
vehicles_pc	Number of 2- and 4-wheeled vehicles per capita
health_exp	Total health expenditure per capita

For economic activity and occupation proportions, estimates from ST-GPR were then re-scaled to sum to 1 across categories by dividing each estimate by the sum of all the estimates.

The following sections describe the modelling approaches for each occupational risk's exposure prevalence. These approaches were developed for GBD 2016 and have not changed substantially since then.

Occupational carcinogens, occupational noise, and occupational particulates

Prevalence of exposure to these risks was determined using the following equation:

$$Prevalence\ of\ Exposure_{c,y,s,a,r,l} = \sum_{EA} Proportion_{EA,c,y} * EAP_{c,y,s,a} * Exposure\ rate_{EA,r,l,d}$$

where:

EAP = economically active population	c = country	r = risk
EA = economic activity	d = duration	s = sex
a = age	l = level of exposure	y = year

Exposure rate (proportion of population exposed) was provided by expert group recommendations and literature.[8-11] The CAREX (carcinogen exposure) database[7] was used to quantify the association between exposure by industry/carcinogen to SDI across all the countries in the database. This effect was used to predict exposure in countries that were not included in CAREX. Duration was considered for occupational carcinogens through application of occupational turnover factors[12] and for occupational noise and particulates by calculating cumulative exposure as the average exposure over the lifetime (the past 50 years) for each age/sex cohort.

Occupational ergonomic factors and occupational asthmagens

Prevalence of exposure to these risks was determined using the following equation:

$$Prevalence\ of\ Exposure_{c,y,s,a,r} = \sum_{EA} Proportion_{OCC,c,y} * EAP_{c,y,s,a}$$

where:

EAP = economically active population	c = country	r = risk
EA = economic activity	a = age	s = sex
OCC = occupation	y = year	

Occupational injuries
Occupational injury counts were estimated using the following equation:

$$Occupational\ fatal\ injuries_{c,y,a,s} = \sum_{EA} Injury\ rate_{EA,c,y,s} * Population_{c,y,a,s} * EAP_{c,y,s,a} * Proportion_{EA,c,y}$$

where:

EAP = economically active population	c = country	y = year
EA = economic activity	a = age	s = sex

In GBD 2021, we used MR-BRT to crosswalk our data. Occupational injuries exposure data come from a number of different sources: insurance records, labour inspectorate records, establishment surveys, establishment or business registers, labour force surveys, economic or establishment censuses, official estimates, records of employers' organizations, and other administrative records and related sources. We expect insurance records to be the gold-standard source, because people should have more incentive to report injuries when they stand to benefit from their insurance plans. As such, we wanted to correct the data reported from other sources for under-reporting, and so we crosswalked all of the data with insurance records data as our reference. To do so, we ran a mixed-effects log-linear regression using MR-BRT, with fixed effects on type of data source and random effects on super-region and region. Table 2 shows the beta coefficients from the MR-BRT model, as well as the crosswalk adjustment factors.

Table 2: MR-BRT crosswalk adjustment factors for occupational injuries

Reference or alternative case definition	Gamma	Beta coefficient, log (95% UI)*	Adjustment factor**
Reference (insurance records)	0.29	---	---
Alternative (labour inspectorate records)		-0.22 (-0.24, -0.21)	0.80 (0.79, 0.81)
Alternative (establishment surveys)		-0.46 (-0.47, -0.44)	0.63 (0.62, 0.65)
Alternative (labour force surveys)		0.01 (0.00, 0.02)	1.01 (1.00, 1.02)
Alternative (economic or establishment censuses)		0.17 (0.17, 0.18)	1.19 (1.18, 1.20)
Alternative (official estimates)		-0.54 (-0.56, -0.52)	0.58 (0.57, 0.60)
Alternative (establishment or business registers)		-0.69 (-0.76, -0.62)	0.50 (0.47, 0.54)

Alternative (other administrative records and related sources)		-0.29 (-0.30, -0.28)	0.75 (0.74, 0.75)

**MR-BRT crosswalk adjustments can be interpreted as the factor the alternative case definition is adjusted by to reflect what it would have been had it been measured using the reference case definition. If the beta coefficient is negative, then the alternative is adjusted up to the reference. If the beta coefficient is positive, then the alternative is adjusted down to the reference.*

***The adjustment factor column is the exponentiated beta coefficient. For log beta coefficients, this is the relative rate between the two case definitions.*

Occupational asbestos

Prevalence of exposure to asbestos was estimated using the asbestos impact ratio (AIR), which is equivalent to the excess deaths due to mesothelioma observed in a population divided by excess deaths due to mesothelioma in a population heavily exposed to asbestos. Formally, this is defined using the following equation:

$$AIR = \frac{Mort_{c,y,s} - N_{c,y,s}}{Mort^{*}_{c,ys,} - N_{c,y,s}}$$

where:

Mort = Mortality rate due to mesothelioma
Mort* = Mortality rate due to mesothelioma in population highly exposed to asbestos
N = Mortality rate due to mesothelioma in population not exposed to asbestos

c = country
y = year
s = sex

Mortality rate due to mesothelioma was estimated using GBD 2021 causes of death results. Mortality rate due to mesothelioma in populations not exposed to asbestos was calculated using the model in Lin and colleagues,[13] while the mortality rate due to high exposure to asbestos was estimated using Goodman and colleagues' model.[14] Asbestos exposure prevalence created using the AIR was used to estimate population attributable fractions (PAFs) for all asbestos-associated causes except for mesothelioma. Custom PAFs were calculated for mesothelioma by using the ratio of the excess mortality with respect to an unexposed population (Mort – N) divided by the mortality rate in the population in question (Mort). This calculation assumes that all mesothelioma is a product of occupational asbestos exposure and could potentially overestimate the burden due to occupational asbestos exposure in populations with high non-occupational asbestos exposure.

Theoretical minimum-risk exposure level

For all occupational risks, the theoretical minimum-risk exposure level was assumed to be no exposure to that risk.

Relative risks

Relative risks were obtained for all occupational risks by conducting a systematic review of published meta-analyses. This review was last updated for GBD 2016. Table 3 provides a summary of the relative risk input data used. The full table of relative risks can be found in a separate section of the Appendix.

Table 3: Data inputs for relative risks

Input data	Relative risk
Source count (total)	21

Population attributable fractions (PAFs)

For all occupational risks, with the exception of injuries (outlined below) and asbestos (outlined above), PAFs were calculated using the exposure prevalences estimated above, using the PAF formula outlined in the GBD 2021 methods appendix.

Section 2.1.7: *Occupational injuries PAF*

The PAFs for occupational injuries were calculated using the following formula:

$$PAF_{c,y,a,s} = \frac{Occupational\ fatal\ injuries_{c,y,a,s} - TMREL}{Fatal\ injuries_{c,y,a,s}}$$

where:

c = country
y = year
a = age
s = sex

Since the TMREL is zero, the occupational injuries PAF is simply the ratio of occupational fatal injuries to total fatal injuries. Fatal injury totals were obtained from GBD 2021 causes of death.

References

1. International Labour Organization (ILO). International Labour Organization Database (ILOSTAT) - Employment by Sex and Economic Activity. International Labour Organization (ILO).

2. International Labour Organization (ILO). International Labour Organization Database (ILOSTAT) - Employment by Sex and Occupation. International Labour Organization (ILO).

3. International Labour Organization (ILO). International Labour Organization Database (ILOSTAT) - Fatal Injuries by Sex and Economic Activity. International Labour Organization (ILO).

4. International Labour Organization (ILO). International Labour Organization LABORSTA Economically Active Population, Estimates and Projections, October 2011. International Labour Organization (ILO), 2011.

5. Office for National Statistics (United Kingdom). Nomis Official Labor Market Statistics - Annual Population Survey. Newport, United Kingdom: Office for National Statistics (United Kingdom).

6. National Bureau of Statistics of China. China 1% National Population Sample Survey 1995. Ann Arbor, United States: China Data Center, University of Michigan.

7. Kauppinen T, Toikkanen J, Pedersen D, *et al.* Occupational exposure to carcinogens in the European Union. *Occupational and Environmental Medicine* 2000; **57**: 10–8.

8. Wilson DH, Walsh PG, Sanchez L, *et al.* The epidemiology of hearing impairment in an Australian adult population. *Int J Epidemiol* 1999; 28: 247–52

9. Kauppinen T, Toikkanen J, Pederson D, Young R, Kogevinas M, Ahrens W, et al. Occupational Exposure to Carcinogens in the European Union in 1990-93. Helsinki, Finland: Finnish Institute of Occupational Health; 1998.

10. Kauppinen T, Toikkanen J, Pedersen D, Young R, Ahrens W, Boffetta P, et al. Occupational exposure to carcinogens in the European Union. *Occup Environ Med* 2000; 57(1): 10–18.

11. Driscoll T, et al. The global burden of non-malignant respiratory disease due to occupational airborne exposures. *American Journal of Industrial Medicine 2005; 48*(6): 432-445.

12. Nelson, D. I., Concha-Barrientos, M., Driscoll, T., Steenland, K., Fingerhut, M., Punnett, L. & Corvalan, C. (2005). The global burden of selected occupational diseases and injury risks: Methodology and summary. *American journal of industrial medicine*, *48*(6), 400-418

13. Lin R-T, Takahashi K, Karjalainen A, *et al.* Ecological association between asbestos-related diseases and historical asbestos consumption: an international analysis. *Lancet* 2007; **369**: 844–9.

14. Goodman M, Morgan RW, Ray R, Malloy CD, Zhao K. Cancer in asbestos-exposed occupational cohorts: a meta-analysis. *Cancer Causes Control* 1999; **10**: 453–65.

Residential radon pollution

Flowchart

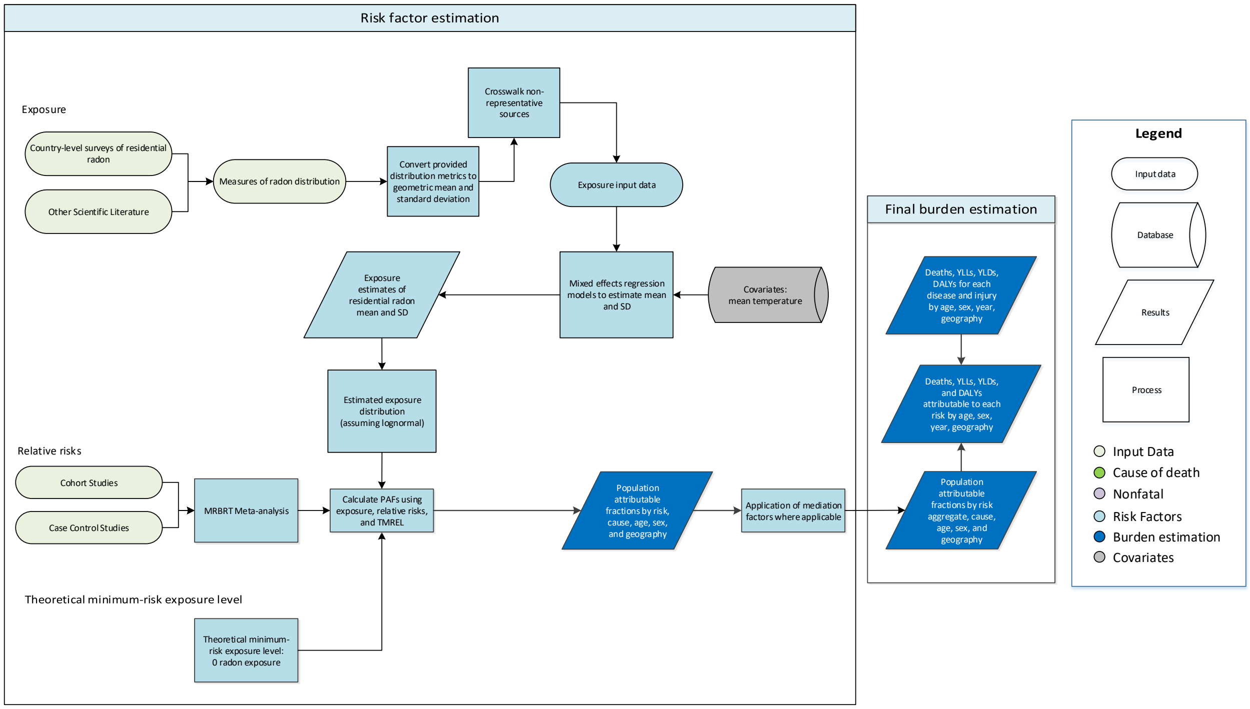

Input data and methodological summary

Exposure

Definition

Radon is a radioactive gas produced as a byproduct of the decay chain of uranium, occurring naturally within the Earth's crust. Some fraction of this natural radon production escapes into the atmosphere, where it is present at low concentrations unless buildup is caused by release into enclosed spaces such as homes, mines, or caves. Radon exposure is expressed as average daily exposure to indoor air radon gas levels measured in Becquerels (disintegrations per second) per cubic metre (Bq/m^3). In the GBD study, we specifically quantify the burden due to indoor radon exposure.

Input data

An expert group curated the original dataset for residential radon exposure. We updated data sources for GBD 2019, and no additional data were included for GBD 2021. Data sources include national surveys, government reports, and scientific literature. We include any sources that report results of residential radon measurement in homes (not schools or workplaces). Due to limited availability of data, we also include sources that are not representative of an entire population, but exclude studies or surveys explicitly conducted in high radon areas.

Table 1: Data inputs for exposure for residential radon pollution

	Countries with data	New sources	Total sources
Exposure	77	0	207

From each source, we extracted all available information required to estimate the distribution of radon exposure, including arithmetic mean and standard deviation, geometric mean and standard deviation, median, IQR, range, max, sample size, confidence interval, and/or standard error.

Modelling strategy

Literature suggests that radon exposure follows a lognormal distribution both on the household and national levels.[1] We therefore assume that the distribution of radon exposure is lognormal within any one GBD geography or study. For studies reporting at least one measure of central tendency (arithmetic mean, geometric mean, or median) and a measure of spread (standard deviation [arithmetic or geometric], IQR, confidence interval, or standard error), we are able to directly calculate the geometric mean and geometric standard deviation of the underlying distribution. For those only reporting a measure of central tendency and range, max, or sample size, we estimate the geometric mean and standard deviation based on several assumptions.

- When the range or max is provided, we assume that the range divided by 4 is a reasonable estimate of standard deviation, because 95% of observations occur within 2 standard deviations of the mean. We perform this calculation on log-transformed observations.
- For studies that only provide a measure of central tendency and sample size, we impute standard deviation based on sample size from observed associations between standard deviation and sample size for other available input data.
- If we only have the mean, we impute the median standard deviation of all other studies.

Once we convert all estimates to the mean and standard deviation of a lognormal distribution, we run all analyses on log-transformed data to meet assumptions of normality.

Though we exclude studies intentionally performed in high-exposure areas, we still see a bias in studies that are not representative of their geography. To account for this difference, we perform a crosswalk adjustment using the meta-regression—Bayesian, regularised, trimmed (MR-BRT) tool. We match all locations where we have both representative and non-representative sources. These locations include Canada, Egypt, Gansu, Greece, Hiroshima, Ireland, Jordan, Portugal, Puebla, Querétaro, Romania, San Luis Potosí, Saudi Arabia, Shanghai, Spain, Syria, Taiwan (province of China), Turkey, Urban Andhra Pradesh, Urban Assam, Urban Gujarat, Urban Haryana, Urban Karnataka, Urban Kerala, Urban Maharashtra, Urban Meghalaya, Urban Punjab, Urban Rajasthan, Urban Tripura, and Urban Uttar Pradesh. We perform the following model on the log difference of the log of the geometric means:

Let $ref = \log(geometric\ mean\ representative)$, and

$alt = \log(geometric\ mean\ non\ representative)$.

$$log\left(\frac{alt}{ref}\right) \sim Beta$$

$$ref \sim e^{-Beta} * alt$$

$$ref \sim (adjustment\ factor) * alt$$

We use the results of this crosswalk to downscale all non-representative input sources and inflate their uncertainty in the model. The effect is equivalent to scaling the log of the geometric mean of non-representative sources by a factor of 0.887.

Table 2: MR-BRT crosswalk adjustment factor for residential radon exposure

Data input	Reference or alternative case definition	Gamma	Beta coefficient, log (95% UI)*	Adjustment factor**
Geographically representative survey or report	Reference	0.25	---	
Estimate not representative of geographic unit	Alternative		0.120 (0.107–0.132)	0.887 (0.876–0.899)

**MR-BRT crosswalk adjustments can be interpreted as the factor the alternative case definition is adjusted by to reflect what it would have been had it been measured using the reference case definition. If the log/logit beta coefficient is negative, then the alternative is adjusted up to the reference. If the log/logit beta coefficient is positive, then the alternative is adjusted down to the reference.*
***The adjustment factor column is the exponentiated negative beta coefficient. For log beta coefficients, this is the relative rate between the two case definitions. For logit beta coefficients, this is the relative odds between the two case definitions.*

After crosswalking non-representative sources, we run a model to estimate the log of the geometric mean residential radon exposure for each GBD most-detailed location. Because radon is naturally occurring and is not considered to have much long-term temporal fluctuation, we used a mixed effects linear model independent of time.[2] The model included nested random effects on super-region, region, and location (most detailed) and one fixed effect covariate: long-term mean temperature (average annual temperature averaged over 1990 to 2020) as a proxy for adequate building ventilation. We weighted the model by inverse standard error. We also tested weighting by inverse variance and sample size, but this resulted in an unstable fit. To predict the log of the geometric mean we used the following model:

$$\log(geometric\ mean) \sim \beta * long\ term\ mean\ temp + (1|super\ region) + (1|region) + (1|location)$$

Table 3: Regression coefficients for predicting mean radon

Input	Coefficient (95% UI)
Intercept	4.008 (3.519 to 4.502)
Long-term mean temperature	–0.0388 (–0.0619 to –0.0145)

We also ran a model to predict the standard deviation (in log space) for each location. We included all studies that were representative of a geography and that included a measure of spread for which we were able to directly calculate the standard deviation. We used a mixed effects linear regression of standard deviation on mean, including random effects on location (most detailed) and region. The

model was not stable when including super-region. To predict the log of the geometric standard deviation we used the following model:

$$\log(geometric\ standard\ deviation) \sim \beta * \log(geometric\ mean) + (1|region) + (1|location)$$

Table 4: Regression coefficients for predicting standard deviation of radon

Input	Coefficient (95% UI)
Intercept	0.619 (0.397 to 0.848)
Log(geometric mean)	0.014 (–0.027 to 0.054)

We used the estimated mean and standard deviation for each location to generate an exposure distribution for use in population attributable fraction calculation.

Theoretical minimum risk exposure level

The GBD 2017 study defined the theoretical minimum risk exposure level (TMREL) as a uniform distribution from 7 to 14 Bq/m^3 representing outdoor air. However, in GBD 2019 we updated the radon TMREL to 0. The basis for this decision is that it is theoretically possible with mitigation strategies to reduce all indoor radon exposure to 0. The TMREL of 0 remains unchanged for GBD 2021.

Relative risks

Input data

In GBD 2017, the relative risk (RR) was based on a single meta-analysis (Darby et al., 2005) which reported an RR of 1.16 (1.05–1.31) per 100 Bq/m^3 increase in radon exposure.[3] In GBD 2019, we conducted a systematic review of studies examining residential exposure to radon and lung cancer incidence or mortality. We extracted the component studies from the following meta-analyses: Lubin et al. 2003, Darby et al. 2005, Krewski et al. 2005, Zhang et al. 2012, Torres-Durán et al. 2014, and Dobrzynski et al. 2018.[3,4,5,6,7,8] We excluded studies that were cross-sectional or ecological, studied high-risk populations such as miners, or were not available in English. When multiple studies were published on the same dataset, we retained only the study with the longest follow-up. We also excluded studies that only reported cumulative exposure, because this does not align with our exposure definition.

For GBD 2021, as in GBD 2019, we assumed a log-linear relationship between RR and radon exposure. As before, we converted all reported risks to the RR increase per 100-unit increase in Bq/m^3. Some studies only reported RR between exposure categories. In these instances, we took the mean, median, or midpoint of the exposed and unexposed categories to calculate an "exposure range." We then scaled the reported RR based on that exposure range to estimate the corresponding increase per 100 units. This resulted in a total of 49 estimates from 25 studies in 12 countries including England, the Czech Republic, Finland, France, Germany, Italy, Spain, Sweden, the United States, China, Denmark, and Japan.

Table 5: Data inputs for relative risks for residential radon pollution

	Countries with data	New sources	Total sources
Relative risks	12	0	24

For those studies that reported no confidence intervals or standard error, we imputed the standard error based on sample size. To do this we created a model of the following form:

$$se \sim \beta * \frac{1}{\sqrt{n}}$$

Where we predict the standard error, se, as a function of some constant, β, times the inverse square root of the sample size, n. Here β is an estimate of the population level standard deviation.

MR-BRT meta-regression

As in GBD 2019, we used the MR-BRT meta-regression tool to estimate a summary effect size from the 49 estimates of the RR increase per 100-unit change in exposure. For GBD 2021, there were several key updates to the meta-regression process. First, we implemented automated covariate selection to detect significant covariates from those extracted to quantify between-study heterogeneity. The MR-BRT automated covariate selection tool implements a two-step process. First, a series of loosening Lasso penalty parameters are applied to a log-linear meta-regression on all input effect size observations. Then, covariates with a non-zero coefficient are tested for significance using a Gaussian prior (significance threshold = 0.05). No significant covariates were detected for residential radon, so we did not include covariates for selection bias (percentage of cohort retained through follow-up) and quality of exposure measurement (binary indicator for whether or not studies included a full residential history) as we did in GBD 2019. Additionally, we trimmed 10% of input data during model fitting in accordance with GBD protocol across risk factor teams.

We generated 1000 predictions of the effect size for use in calculating burden estimates. These predictions were created using predictions of between-study heterogeneity to characterise the model's uncertainty. We implemented the Fisher scoring correction to the heterogeneity parameter, which corrects for data-sparse situations. In such cases, the between-study heterogeneity parameter estimate may be 0, simply from lack of data. The Fisher scoring correction uses a quantile of gamma, which is sensitive to the number of studies, study design, and reported uncertainty.

The summary effect size for RR of lung cancer per 100 Bq/m^3 of radon exposure is 1.102 (95% UI 0.962–1.266).

Table 6: MR-BRT relative risk model parameters for residential radon pollution

**Covariate**	**Gamma (95% UI)**	**Beta coefficient, log (95% UI)**	**Exponentiated coefficient (95% UI)**
Exposure (per 1 Bq/m^3)	0 (0–0)	9.907e-4 (5.189e-4 to 1.484e-3)	1.00099 (1.00052–1.00149)

Figure 1: Residential radon pollution and lung cancer risk literature funnel plot

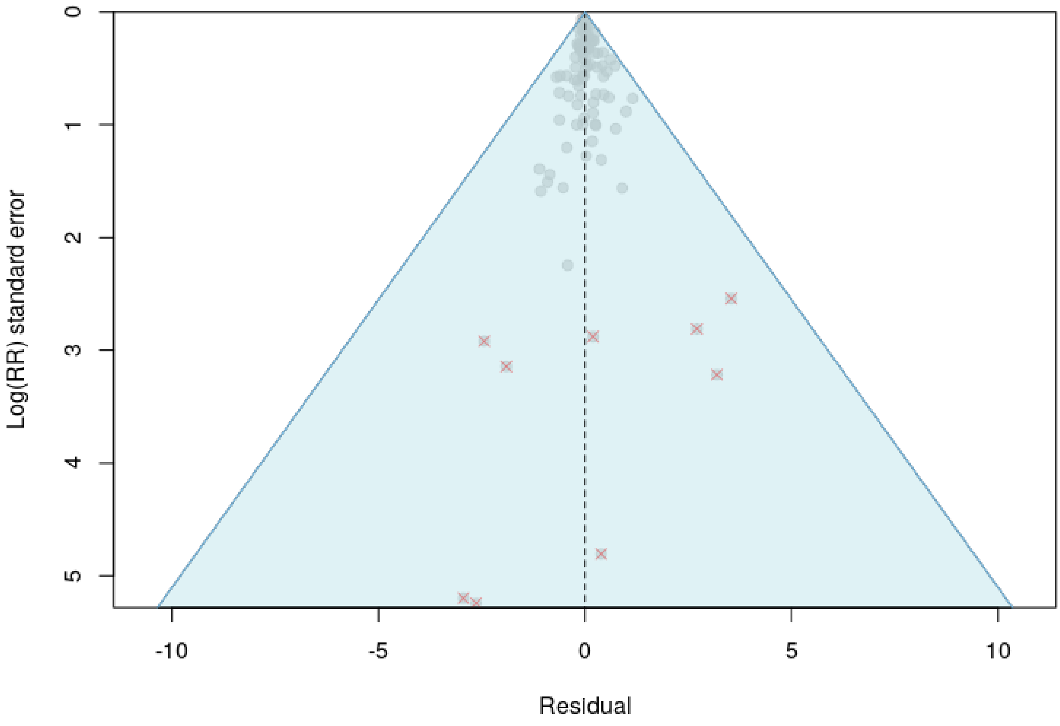

Figure 2: Residential radon pollution and lung cancer log-linear relative risk curve

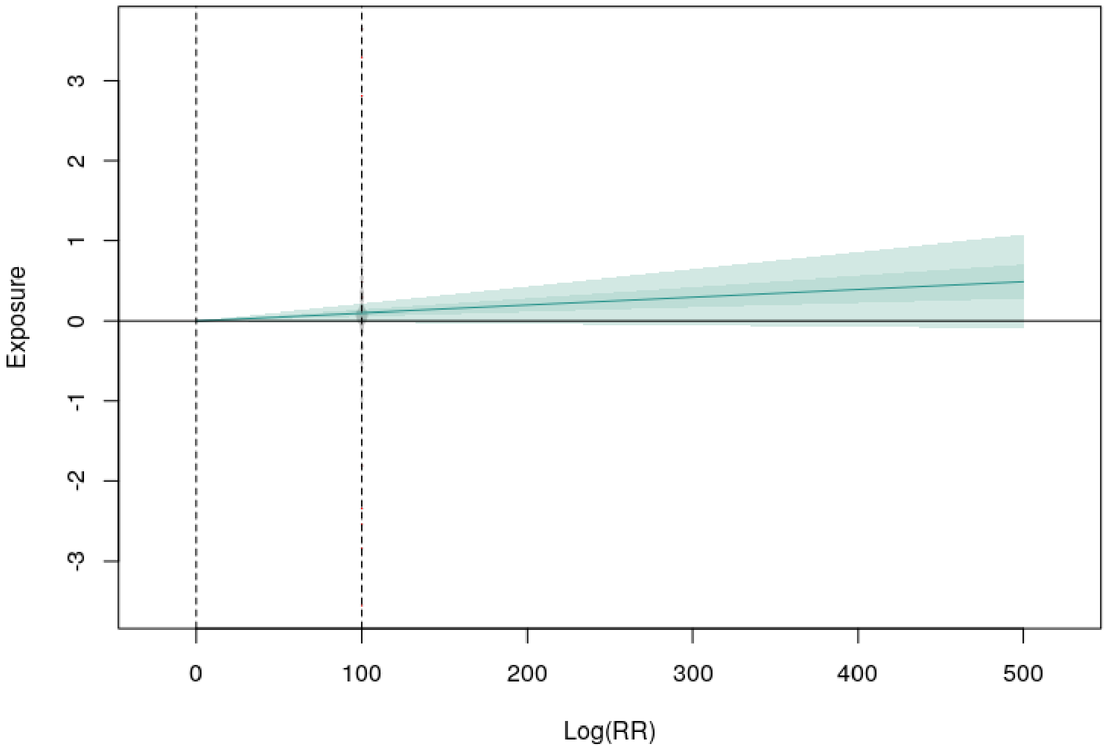

Risk-outcome scoring

For GBD 2021, we also implemented risk-outcome scoring. Risk-outcome scores provide an empirical measure of the strength of evidence for risk–outcome pairs across risk factors in the GBD and are therefore useful for standardised comparison. Risk-outcome scores evaluate the area between the lower bound of the 95% uncertainty interval and the x-axis for harmful risk factors, including residential radon pollution.

Prior to generating a risk-outcome score, we conducted an additional post-analysis step to detect and flag publication bias in the input data. This approach is based on the classic Egger's regression strategy, which is applied to the residuals in our model. In the current implementation, we do not correct for publication bias, but flag the risk–outcome pairs where the risk for publication bias is significant. Publication bias was not detected for residential radon pollution.

To calculate the risk-outcome score, we generated an uncertainty interval from 1000 draws of the adjusted summary effect size (retaining uncertainty information from between-study heterogeneity predictions and the Fisher information boost). We then evaluated the risk-outcome score between the 15th and 85th percentiles of the input data exposure distribution (0–100 Bq/m^3). The final risk-outcome score is –0.009, which corresponds to a star rating of 1.

References

1. Daraktchieva Z, Miles JCH, McColl N. Radon, the lognormal distribution and deviation from it. J Radiol Prot 2014; 34: 183–190.
2. Steck DJ. Annual average indoor radon variations over two decades. Health Phys. 2009;96(1):37-47.
3. Darby S, Hill D, Auvinen A, et al. Radon in homes and risk of lung cancer: collaborative analysis of individual data from 13 European case-control studies. BMJ. 2005;330(7485):223
4. Lubin JH. Studies of radon and lung cancer in North America and China. Radiation Protection Dosimetry. 2003Jan;104(4):315–9.
5. Krewski D, Lubin JH, Zielinski JM, Alavanja M, Catalan VS, Field RW, et al. Residential Radon and Risk of Lung Cancer. Epidemiology. 2005;16(2):137–45.
6. Zhang Z-L, Sun J, Dong J-Y, et al. Residential radon and lung cancer risk: an updated meta- analysis of case-control studies. Asian Pac J Cancer Prev 2012; 13: 2459–65.
7. Torres-Durán MCAD, Barros-Dios JM, Fernández-Villar A, Ruano-Ravina A. Residential radon and lung cancer in never smokers. A systematic review. Cancer Letters. 2014;345(1):21–6.
8. Dobrzyński L, Fornalski KW, Reszczyńska J. Meta-analysis of thirty-two case–control and two ecological radon studies of lung cancer. J Radiat Res 2018; 59: 149–63.

Secondhand smoke

Flowchart

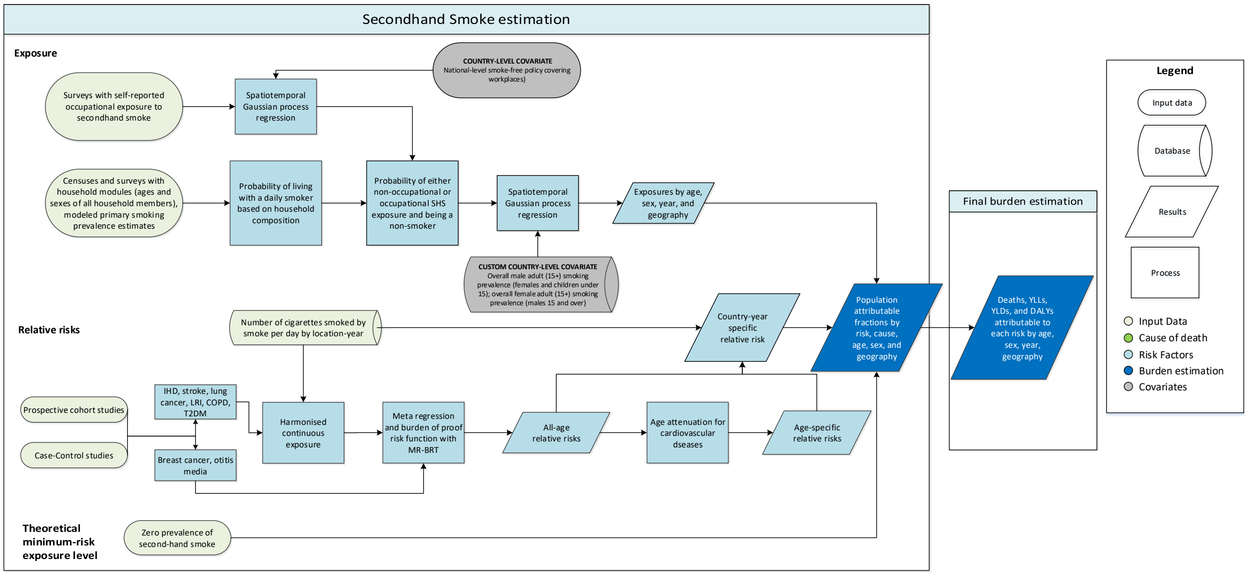

Exposure

Case definition

We define secondhand smoke exposure as current exposure to secondhand tobacco smoke at home or at work. We use household composition as a proxy for household secondhand smoke exposure and make the assumption that all persons living with a daily smoker are exposed to tobacco smoke. We use surveys to estimate the proportion of the population exposed to secondhand smoke at work. We only consider non-smokers to be exposed to secondhand smoke. Non-smokers are defined as all persons who are not daily smokers. Ex-smokers and occasional smokers are considered non-smokers in this analysis. Exposure is evaluated for both children and adults.

Input data

To calculate the proportion of non-smokers who live with at least one daily smoker, two types of data were used: 1) unit record data on household composition, which included the ages and sexes of all persons living in the same household, and 2) GBD daily smoking estimates for each location, year, sex, and age group. Major survey series with a household composition module – including the Demographic Health Surveys (DHS), the Multiple Indicator Cluster Surveys (MICS), and the Living Standards Measurement Surveys (LSMS) – and national and subnational censuses, which included those captured in the Integrated Public Use Microdata Series (IPUMS) project, were used.

To calculate the proportion of the population exposed to secondhand smoke at work, by age and sex, we used cross-sectional surveys that ask respondents about self-reported occupational secondhand smoke exposure. Sources include the Global Adult Tobacco Surveys (GATS), Eurobarometer Surveys,

WHO Stepwise Approach to NCD Risk Factor Surveillance (STEPS) Surveys, and other regional and national survey series.

We updated our systematic review in GBD 2021 by searching the Global Health Data Exchange (GHDx) using the keywords “environmental tobacco smoke”, for workplace exposure, and “family composition”, for identifying household composition modules. We prioritised extraction of surveys used for estimating

Modelling strategy

exposure at the workplace and of new household modules for filing in location and time gaps. Sources that reported exposure to secondhand smoke in a setting other than the workplace were not used. Due to the type of analysis performed, we restricted our data sources to those with available microdata (tabulated data-only sources were excluded). Given the nature of the data used in our models (microdata), no crosswalk for case definition adjustment or age and sex splitting processes were required. Table 1 provides a summary of the exposure input data.

Table 1: Data inputs for exposure for secondhand smoke

	Countries with data	New sources	Total sources
Exposure	176	480	1198

Identical to GBD 2019, we estimated the probability that each person is living with a smoker and is also a non-smoker themselves using set theory. Household composition data were used at the individual level to capture the ages and sexes of each person in the household. In the past, we analysed surveys with both household composition data and tobacco use questions and determined that the distribution of household size, mean age of the household members, and the age distribution were not significantly different between households with and without a self-reported smoker. Since we did not find that household composition varied between smokers and non-smokers, we then used the updated GBD 2021 daily smoking prevalence estimates to calculate the probability that each household member is a daily smoker. Next, we used the probability of the union of sets on each individual household member to calculate the overall probability that at least one of the other household members was a daily smoker.

As in GBD 2019, we incorporated occupational exposure by modelling prevalence of current exposure to secondhand smoke at work, by age, sex, location, and year, in a three-step spatiotemporal Gaussian process regression (ST-GPR), which generates exposure estimates from a mixed-effects hierarchical linear model plus weighted residuals smoothed across time, space, and age. For this, we first processed all data to capture exposure to secondhand smoke at work among anyone working primarily indoors. Using information from survey-specific gateway questions, we considered all those not currently working or not currently working primarily indoors not exposed to secondhand smoke.

The processed microdata was used to generate a complete time series from 1990 to 2022 for the proportion of the population exposed to tobacco smoke at the workplace using the ST-GPR. The first step of the ST-GPR process is a linear mixed-effects regression of our data on a set of potentially predictive covariates. In addition to the daily smoking prevalence estimates taken from the GBD study covariates database, in GBD 2021 we incorporated a dummy covariate to reflect if a national-level

smoking ban covering workplaces was in place in each location-year. The data used to create this covariate came mainly from several iterations of the WHO report on the global tobacco epidemic.

With the estimated workplace exposure from ST-GPR, in order to avoid double counting, we calculated the probability that an individual is exposed through either household exposure or occupational exposure, given their age, sex, and household composition. Lastly, we multiplied this probability of exposure by the probability that the individual is not a smoker themselves (ie, 1 minus primary daily smoking prevalence for that person's location, year, age, and sex). We then collapsed these individual-level probabilities to produce average probabilities of exposure by location, year, age, and sex.

These final probabilities were modelled in the GBD ST-GPR framework. The linear model formula was fit separately by sex using restricted maximum likelihood in R. We used the sex-specific overall daily smoking prevalence for adults (age 15 and older) as a country-level covariate in the model. The overall male adult daily smoking prevalence was used as the covariate for females of all ages and for males under age 15. The overall female adult daily smoking prevalence was used as the covariate for males aged 15 and older.

All input datapoints from the probability calculation had a measure of uncertainty (variance and sample size) coming from the uncertainty of the primary smoking prevalence model and the sample size from the unit record data going into the modelling process. Geographical r*andom effects were used in model fitting but were not used in prediction.*

Theoretical minimum risk exposure level

The theoretical minimum risk exposure level for secondhand smoke is zero exposure among non-smokers, meaning that non-smokers would not live with any daily smokers and would not be exposed to tobacco smoke at their workplace.

Relative risks

The same riskoutcome pairs from GBD 2019 were used. For children ages 0–14, we estimated the burden of otitis media attributable to secondhand smoke exposure. For all ages, we estimated the burden of lower respiratory infections (LRI) and for adults greater than or equal to 25 years of age, we estimated the burden of lung cancer, chronic obstructive pulmonary disease (COPD), ischaemic heart disease (IHD), ischaemic stroke, breast cancer, and type 2 diabetes (T2DM).

Input data

In GBD 2021, we moved from deriving our relative risks from the integrated exposure response curves (IER) for $PM_{2.5}$ air pollution to creating relative risk curves using secondhand smoke-specific studies. We conducted an updated systematic review for studies published before December 31, 2019, evaluating the relationship between exposure to secondhand smoke and risk of IHD, stroke, COPD, breast cancer, and otitis media. We searched for studies in PubMed using the search strings reported in Table 2. Meta-analysis identified through our search were reviewed and underlying studies were considered for inclusion if not previously captured by our search strings. For the remaining outcomes – lung cancer, LRI, and T2DM – we selected the secondhand smoke studies from the database that was used in GBD 2019 for generating the IER curve.

Table 2: Search strings used to search PubMed database

Outcome	String
Ischaemic heart disease	(Tobacco smoke pollution [MeSH Terms] OR second-hand[Title/Abstract] OR secondhand[Title/Abstract] OR environmental tobacco[Title/Abstract] OR tobacco smoke[Title/Abstract] OR cigarette smoke[Title/Abstract] OR passive smok*[Title/Abstract] OR involuntary smok*[Title/Abstract] OR parental smoking[Title/Abstract] OR maternal smoking[Title/Abstract]) AND (Coronary Artery Disease[MeSH] OR Myocardial Ischemia[MeSH] OR atherosclerosis[MeSH] OR Coronary Artery Disease[Title/Abstract] OR Myocardial Ischemia[Title/Abstract] OR cardiac ischemia[Title/Abstract] OR silent ischemia[Title/Abstract] OR atherosclerosis [Title/Abstract] OR Ischaemic heart disease[Title/Abstract] OR Ischemic heart disease[Title/Abstract] OR coronary heart disease[Title/Abstract] OR myocardial infarction[Title/Abstract] OR heart attack[Title/Abstract] OR heart infarction[Title/Abstract]) AND (Case-Control Studies[MeSH Terms] OR Cross-Over Studies[MeSH Terms] OR Cohort Studies[MeSH Terms] OR Systematic Review[Publication Type] OR Meta-Analysis[Publication Type] OR “systematic review”[Title/Abstract] OR “meta-analysis”[Title/Abstract] OR “cohort”[Title/Abstract] OR “cross-over”[Title/Abstract] OR “crossover”[Title/Abstract] OR “case-control”[Title/Abstract] OR “prospective”[Title/Abstract] OR “retrospective”[Title/Abstract] OR “longitudinal”[Title/Abstract] OR “follow-up”[Title/Abstract] OR Dose-Response Relationship, Drug[MeSH Terms] OR “dose-response”[Title/Abstract]) AND (Risk[MeSH Terms] OR Odds Ratio[MeSH Terms] OR “risk”[Title/Abstract] OR “odds ratio”[Title/Abstract] OR “cross-product ratio”[Title/Abstract] OR “hazards ratio”[Title/Abstract] OR “hazard ratio”[Title/Abstract]) AND ("1970/01/01"[PDat] : "2019/12/31"[PDat]) AND (English[LA]) NOT (animals[MeSH Terms] NOT Humans[MeSH Terms])
Ischaemic stroke	(Tobacco smoke pollution [MeSH Terms] OR second-hand[Title/Abstract] OR secondhand[Title/Abstract] OR environmental tobacco[Title/Abstract] OR tobacco smoke[Title/Abstract] OR cigarette smoke[Title/Abstract] OR passive smok*[Title/Abstract] OR involuntary

	smok*[Title/Abstract] OR parental smoking[Title/Abstract] OR maternal smoking[Title/Abstract]) AND (brain infarction[MeSH Terms] OR stroke[MeSH Terms] OR intracranial hemorrhages[MeSH Terms] OR "stroke"[Title/Abstract] OR “brain infarction”[Title/Abstract] OR “cerebral infarction”[Title/Abstract] OR “intracerebral hemorrhage”[Title/Abstract] OR “intracerebral haemorrhage”[Title/Abstract] OR “subarachnoid hemorrhage”[Title/Abstract] OR “subarachnoid haemorrhage”[Title/Abstract]) AND (Case-Control Studies[MeSH Terms] OR Cross-Over Studies[MeSH Terms] OR Cohort Studies[MeSH Terms] OR Systematic Review[Publication Type] OR Meta-Analysis[Publication Type] OR “systematic review”[Title/Abstract] OR “meta-analysis”[Title/Abstract] OR “cohort”[Title/Abstract] OR “cross-over”[Title/Abstract] OR “crossover”[Title/Abstract] OR “case-control”[Title/Abstract] OR “prospective”[Title/Abstract] OR “retrospective”[Title/Abstract] OR “longitudinal”[Title/Abstract] OR “follow-up”[Title/Abstract] OR Dose-Response Relationship, Drug[MeSH Terms] OR “dose-response”[Title/Abstract]) AND (Risk[MeSH Terms] OR Odds Ratio[MeSH Terms] OR “risk”[Title/Abstract] OR “odds ratio”[Title/Abstract] OR “cross-product ratio”[Title/Abstract] OR “hazards ratio”[Title/Abstract] OR “hazard ratio”[Title/Abstract]) AND ("1970/01/01"[PDat] : "2019/12/31"[PDat]) AND (English[LA]) NOT (animals[MeSH Terms] NOT Humans[MeSH Terms])
Chronic obstructive pulmonary disease	(Tobacco smoke pollution [MeSH Terms] OR second-hand[Title/Abstract] OR secondhand[Title/Abstract] OR environmental tobacco[Title/Abstract] OR tobacco smoke[Title/Abstract] OR cigarette smoke[Title/Abstract] OR passive smok*[Title/Abstract] OR involuntary smok*[Title/Abstract] OR parental smoking[Title/Abstract] OR maternal smoking[Title/Abstract]) AND (Pulmonary Disease, Chronic Obstructive[MeSH] OR "COPD"[Title/Abstract] OR "emphysema"[Title/Abstract] OR "chronic obstructive pulmonary disease"[Title/Abstract]) AND (Case-Control Studies[MeSH Terms] OR Cross-Over Studies[MeSH Terms] OR Cohort Studies[MeSH Terms] OR Systematic Review[Publication Type] OR Meta-Analysis[Publication Type] OR “systematic review”[Title/Abstract] OR “meta-analysis”[Title/Abstract] OR “cohort”[Title/Abstract] OR “cross-over”[Title/Abstract] OR “crossover”[Title/Abstract] OR “case-control”[Title/Abstract] OR “prospective”[Title/Abstract] OR “retrospective”[Title/Abstract] OR “longitudinal”[Title/Abstract] OR “follow-up”[Title/Abstract] OR Dose-Response Relationship, Drug[MeSH Terms] OR “dose-response”[Title/Abstract]) AND (Risk[MeSH Terms] OR Odds Ratio[MeSH Terms] OR “risk”[Title/Abstract] OR “odds ratio”[Title/Abstract] OR “cross-product ratio”[Title/Abstract] OR “hazards ratio”[Title/Abstract] OR

	“hazard ratio”[Title/Abstract]) AND ("1970/01/01"[PDat] : "2019/12/31"[PDat]) AND (English[LA]) NOT (animals[MeSH Terms] NOT Humans[MeSH Terms])
Breast cancer	(Tobacco smoke pollution [MeSH Terms] OR second-hand[Title/Abstract] OR secondhand[Title/Abstract] OR environmental tobacco[Title/Abstract] OR tobacco smoke[Title/Abstract] OR cigarette smoke[Title/Abstract] OR passive smok*[Title/Abstract] OR involuntary smok*[Title/Abstract] OR parental smoking[Title/Abstract] OR maternal smoking[Title/Abstract]) AND (breast neoplasm[MeSH Terms] OR “breast cancer”[Title/Abstract] OR “breast cancers”[Title/Abstract] OR “breast neoplasm”[Title/Abstract] OR “breast neoplasms”[Title/Abstract] OR “mammary cancer”[MeSH Terms] OR “mammary cancers”[Title/Abstract] OR “breast malignant neoplasm”[Title/Abstract] OR “breast malignant neoplasms”[Title/Abstract] OR “mammary carcinoma”[Title/Abstract] OR “mammary carcinomas”[Title/Abstract] OR “breast carcinoma”[Title/Abstract] OR “breast carcinomas”[Title/Abstract] OR “mammary neoplasm”[Title/Abstract] OR “mammary neoplasms”[Title/Abstract] OR “breast tumor”[Title/Abstract] OR “breast tumors”[Title/Abstract] OR “cancer of the breast”[Title/Abstract] OR “cancers of the breast”[Title/Abstract] OR “neoplasm of the breast”[Title/Abstract] OR “tumor of the breast”[Title/Abstract]) AND (Case-Control Studies[MeSH Terms] OR Cross-Over Studies[MeSH Terms] OR Cohort Studies[MeSH Terms] OR Systematic Review[Publication Type] OR Meta-Analysis[Publication Type] OR “systematic review”[Title/Abstract] OR “meta-analysis”[Title/Abstract] OR “cohort”[Title/Abstract] OR “cross-over”[Title/Abstract] OR “crossover”[Title/Abstract] OR “case-control”[Title/Abstract] OR “prospective”[Title/Abstract] OR “retrospective”[Title/Abstract] OR “longitudinal”[Title/Abstract] OR “follow-up”[Title/Abstract] OR Dose-Response Relationship, Drug[MeSH Terms] OR “dose-response”[Title/Abstract]) AND (Risk[MeSH Terms] OR Odds Ratio[MeSH Terms] OR “risk”[Title/Abstract] OR “odds ratio”[Title/Abstract] OR “cross-product ratio”[Title/Abstract] OR “hazards ratio”[Title/Abstract] OR “hazard ratio”[Title/Abstract]) AND ("1970/01/01"[PDat] : "2019/12/31"[PDat]) AND (English[LA]) NOT (animals[MeSH Terms] NOT Humans[MeSH Terms])

Otitis media	(Tobacco smoke pollution [MeSH Terms] OR second-hand[Title/Abstract] OR secondhand[Title/Abstract] OR environmental tobacco[Title/Abstract] OR tobacco smoke[Title/Abstract] OR cigarette smoke[Title/Abstract] OR passive smok*[Title/Abstract] OR involuntary smok*[Title/Abstract] OR parental smoking[Title/Abstract] OR maternal smoking[Title/Abstract]) AND (Otitis Media[MeSH Terms] OR “otitis media”[Title/Abstract] OR “middle ear infection” [Title/Abstract] OR “middle ear disease” [Title/Abstract] OR “ear infection”[Title/Abstract] OR “ear disease”[Title/Abstract] OR “otitis” [Title/Abstract]) AND (Case-Control Studies[MeSH Terms] OR Cross-Over Studies[MeSH Terms] OR Cohort Studies[MeSH Terms] OR Systematic Review[Publication Type] OR Meta-Analysis[Publication Type] OR “systematic review”[Title/Abstract] OR “meta-analysis”[Title/Abstract] OR “cohort”[Title/Abstract] OR “cross-over”[Title/Abstract] OR “crossover”[Title/Abstract] OR “case-control”[Title/Abstract] OR “prospective”[Title/Abstract] OR “retrospective”[Title/Abstract] OR “longitudinal”[Title/Abstract] OR “follow-up”[Title/Abstract] OR Dose-Response Relationship, Drug[MeSH Terms] OR “dose-response”[Title/Abstract]) AND (Risk[MeSH Terms] OR Odds Ratio[MeSH Terms] OR “risk”[Title/Abstract] OR “odds ratio”[Title/Abstract] OR “cross-product ratio”[Title/Abstract] OR “hazards ratio”[Title/Abstract] OR “hazard ratio”[Title/Abstract]) AND ("1970/01/01"[PDat] : "2019/12/31"[PDat]) AND (English[LA]) NOT (animals[MeSH Terms] NOT Humans[MeSH Terms])

PRISMA 2020 flow diagram for a new systematic review of the secondhand smoke and ischaemic heart disease risk–outcome pair in GBD 2021

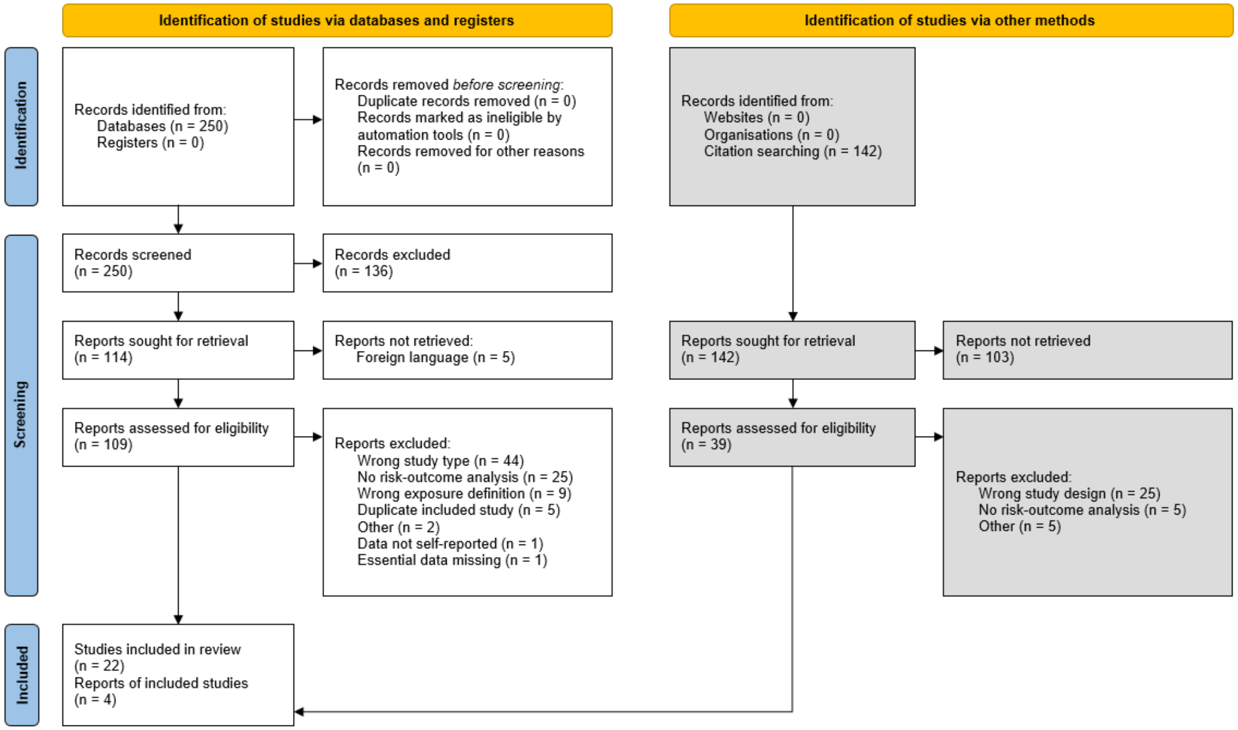

From: Page MJ, McKenzie JE, Bossuyt PM, Boutron I, Hoffmann TC, Mulrow CD, et al. The PRISMA 2020 statement: an updated guideline for reporting systematic reviews. BMJ 2021;372:n71. doi: 10.1136/bmj.n71. For more information, visit: http://www.prisma-statement.org/

PRISMA 2020 flow diagram for a new systematic review of the secondhand smoke and ischaemic stroke risk–outcome pair in GBD 2021

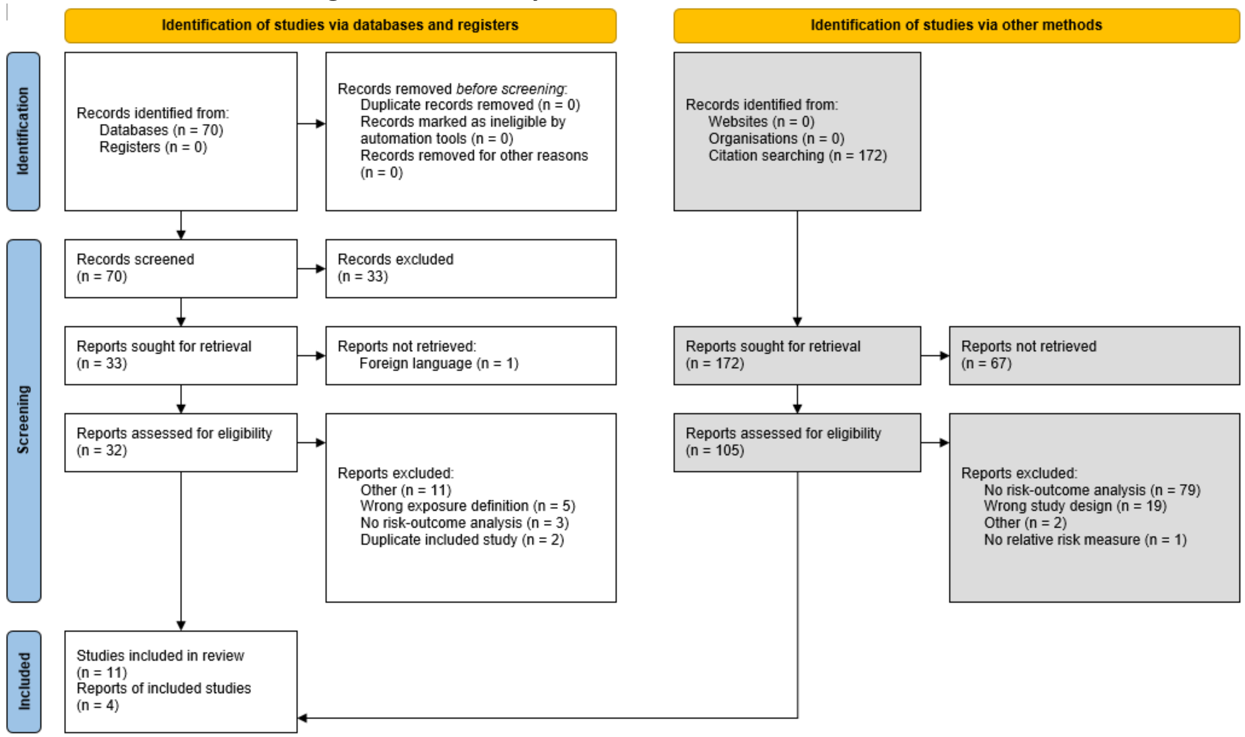

From: Page MJ, McKenzie JE, Bossuyt PM, Boutron I, Hoffmann TC, Mulrow CD, et al. The PRISMA 2020 statement: an updated guideline for reporting systematic reviews. BMJ 2021;372:n71. doi: 10.1136/bmj.n71. For more information, visit: http://www.prisma-statement.org/

PRISMA 2020 flow diagram for a new systematic review of the secondhand smoke and chronic obstructive pulmonary disease risk–outcome pair in GBD 2021

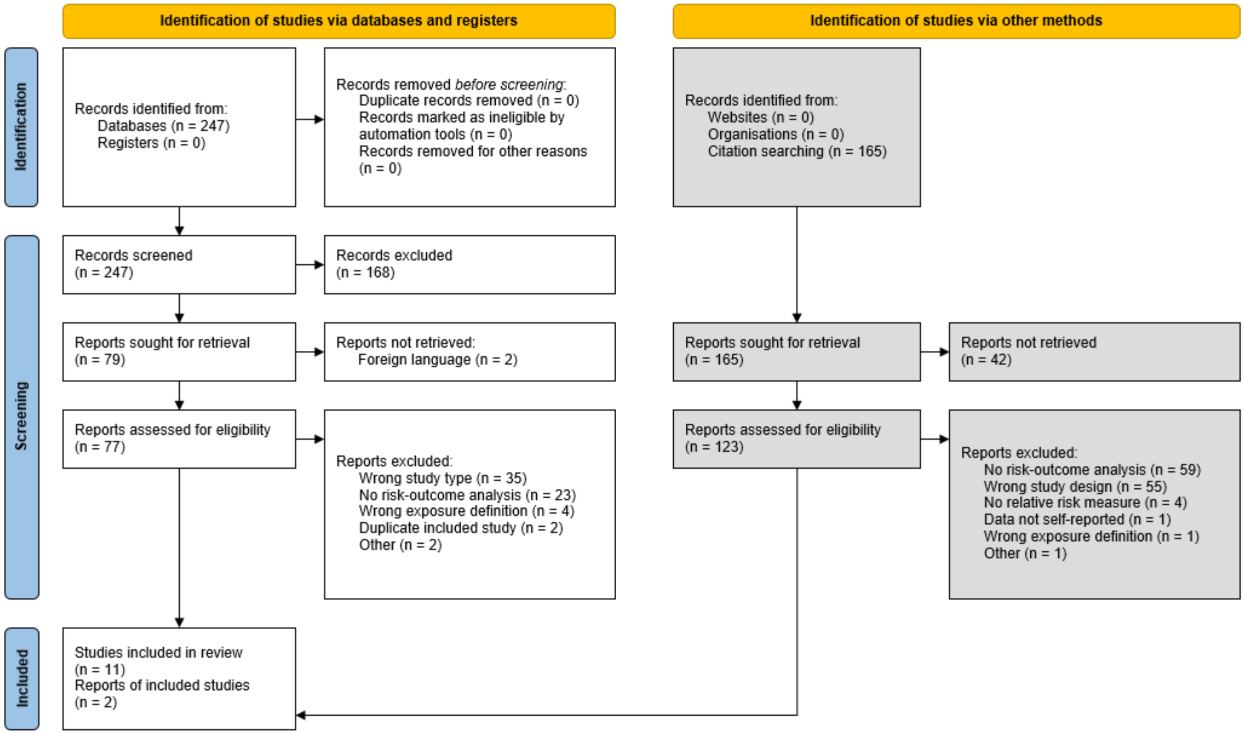

From: Page MJ, McKenzie JE, Bossuyt PM, Boutron I, Hoffmann TC, Mulrow CD, et al. The PRISMA 2020 statement: an updated guideline for reporting systematic reviews. BMJ 2021;372:n71. doi: 10.1136/bmj.n71. For more information, visit: http://www.prisma-statement.org/

PRISMA 2020 flow diagram for a new systematic review of the secondhand smoke and breast cancer risk–outcome pair in GBD 2021

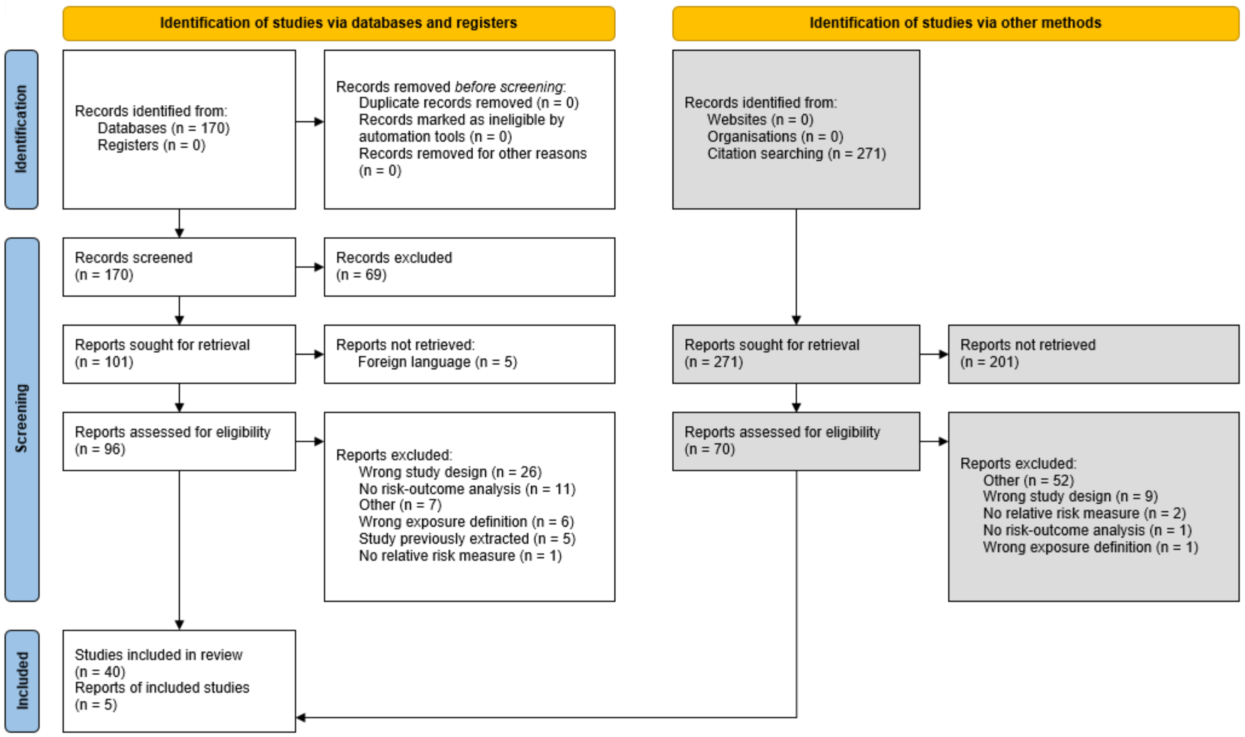

From: Page MJ, McKenzie JE, Bossuyt PM, Boutron I, Hoffmann TC, Mulrow CD, et al. The PRISMA 2020 statement: an updated guideline for reporting systematic reviews. BMJ 2021;372:n71. doi: 10.1136/bmj.n71. For more information, visit: http://www.prisma-statement.org/

PRISMA 2020 flow diagram for a new systematic review of the secondhand smoke and otitis media risk–outcome pair in GBD 2021

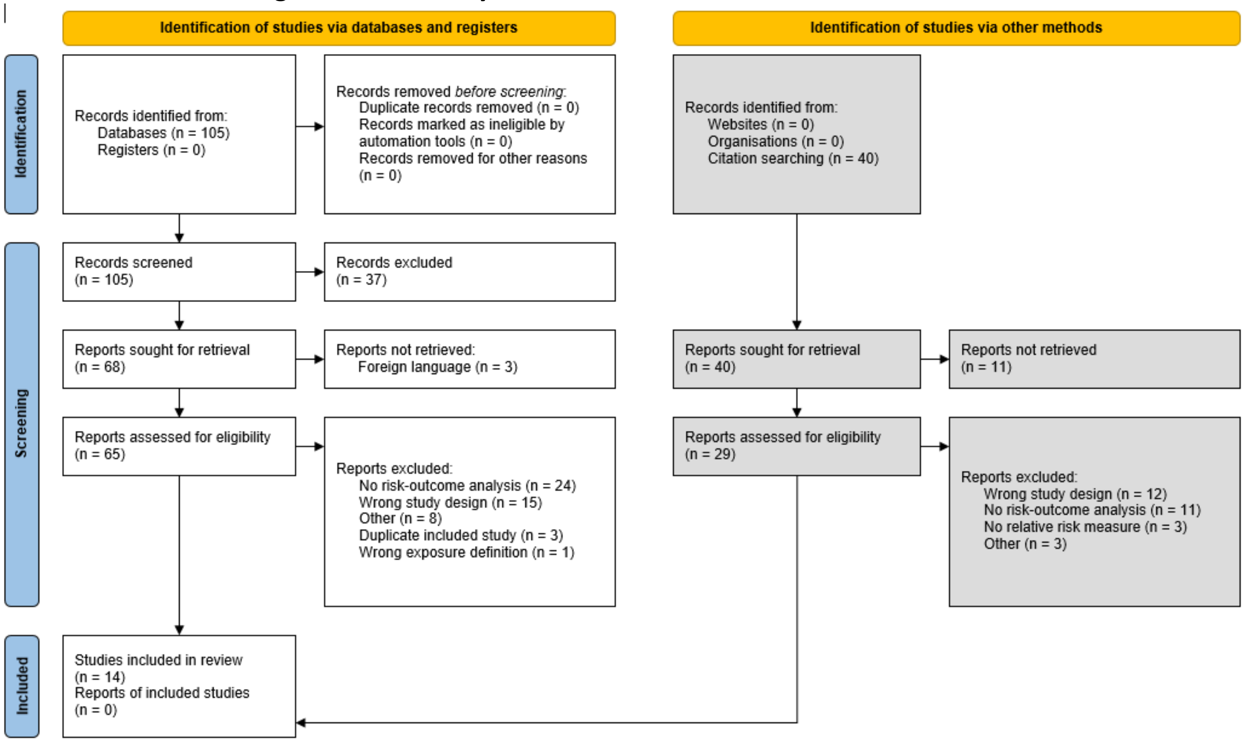

From: Page MJ, McKenzie JE, Bossuyt PM, Boutron I, Hoffmann TC, Mulrow CD, et al. The PRISMA 2020 statement: an updated guideline for reporting systematic reviews. BMJ 2021;372:n71. doi: 10.1136/bmj.n71. For more information, visit: http://www.prisma-statement.org/

We included prospective cohort studies and case-control studies that assessed exposure to secondhand smoke as a binary, categorical (level of exposure: low, moderate, high), or continuous (cigarettes per day) exposure, excluding studies that reported exposure using a different continuous metric (eg, number of hours, number of people, number of days, level of cotinine, etc.) or score. Further, we only included studies that reported risk estimates (relative risk, hazard ratio, or odds ratio) with confidence intervals, standard errors, or enough information to quantify uncertainty. In addition, we excluded studies that only reported former exposure to secondhand smoke (eg, child exposure during pregnancy) or only exposure among current smokers. Table 3 summarises the relative risk input data used in GBD 2021.

Table 3: Data inputs for relative risk for secondhand smoke

Input data	Relative risk
Source count (total)	124
Number of countries with data	33

In future rounds of the GBD, we aim to conduct systematic reviews for the outcomes not updated this round and incorporate new evidence for all outcomes as they become available. In addition, we will evaluate the evidence concerning the relationship between exposure to secondhand smoke and other diseases and add these riskoutcome pairs if general GBD inclusion criteria are met.

Modelling strategy

Prior to GBD 2021, lung cancer, IHD, stroke, and COPD risk curves were calculated jointly with ambient particulate matter pollution, while relative risks for otitis media, breast cancer, and diabetes were derived from published meta-analyses. In GBD 2021, we used the meta-regression—Bayesian, regularised, trimmed (MR-BRT) tool to estimate the log relative risk associated with each level of secondhand smoke exposure on a continuous scale for lung cancer, IHD, stroke, COPD, LRI, and T2DM. For this, we converted binary and categorical exposures reported in each study to a common continuous metric representing the number of cigarettes smoked per smoker per day in each location-year (Table 4). If a study reported exposure in number of cigarettes, we used that number directly.

Table 4: Converting exposure to a continuous scale

Study reported exposure	Matched continuous exposure
Binary	Median of the distribution of cigarettes smoked per smoker per day in the study location-year
Categorical	**Low:** 25th percentile of the distribution of cigarettes smoked per smoker per day in a specific location-year<br>**Medium:** Median of the distribution of cigarettes smoked per smoker per day in a specific location-year<br>**High:** 75th percentile of the distribution of cigarettes smoked per smoker per day in a specific location-year
Continuous (cigarettes per day)	Direct number reported associated with the relative risk reported in the study

For breast cancer and otitis media, we used the MR-BRT tool to perform our own meta-regression analysis of the risk of developing these conditions for those currently exposed to tobacco smoke relative to the reference category of those not exposed. For these outcomes, only studies reporting a binary exposure were included in the analysis. Table 5 shows the results of the MR-BRT analyses for the outcomes with dichotomous exposure.

Table 5: Otitis media and breast cancer MR-BRT network meta-analysis results (reference: not exposed to secondhand smoke)

Outcome	GBD 2019 relative risk	GBD 2021 MR-BRT relative risk
Otitis media	1.37 (1.25–1.50)	1.23 (1.051.45)
Breast cancer	1.07 (1.02–1.13)	1.04 (0.951.13)

For each riskoutcome pair meta-regression, we considered study-level covariates that could potentially bias the study's reported effect size estimates. These study-level covariates included indication of the study design, whether the study used a washout period, whether the study determined outcomes based on administrative records or self-reports, whether the study was generalisable to the general population, and the level of adjustment for relevant confounders like age, sex, smoking, education, and income. We also created covariates to indicate aspects related to the secondhand smoke exposure reported in each study, such as source of exposure (ie, spouse, maternal), exposure setting (ie, work, home, any), exposed population (ie, never smoker, non-smokers), and others. We adjusted for these covariates in our meta-regression if they significantly biased our estimated relative risk function. We used the MR-BRT automated covariate selection process to identify the statistically significant covariates (significance threshold = 0.05). For outcomes with enough datapoints, we introduce likelihood-based trimming to detect and remove outliers (10% trimming) before fitting the model. Outcome-specific model characteristics are described in Table 6.

Table 6: Risk–outcome pair model specifications and results.

Outcome	MR-BRT models specifications	Trimming	Selected covariates	Mean gamma solution	Publication bias
Continuous (risk curves)					
Ischaemic heart disease	Quadratic splines with 3 internal knots; right linear tail; monotonically increasing constraint; Gaussian prior (0, 0.01) on max derivative of non-linear intervals	Yes	Cohort study	0.028	No

Ischaemic stroke	Cubic splines with 3 internal knots; right linear tail; monotonically increasing constraint; Gaussian prior (0, 0.01) on max derivative of non-linear intervals	Yes	-	0.000	No
Chronic obstructive pulmonary disease	Cubic splines with 3 internal knots; right linear tail; monotonically increasing constraint; Gaussian prior (0, 0.01) on max derivative of non-linear intervals	Yes	-	0.082	No
Lung cancer	Cubic splines with 3 internal knots; right linear tail; monotonically increasing constraint; Gaussian prior (0, 0.01) on max derivative of non-linear intervals	Yes	-	0.000	No
Lower respiratory infection	Cubic splines with 3 internal knots; right linear tail; monotonically increasing constraint; Gaussian prior (0, 0.01) on max derivative of non-linear intervals	Yes	Adjusted model; Multiple exposure measurements; >95% follow-up	2.377	Yes
Type 2 diabetes	Cubic splines with 3 internal knots; right linear tail; monotonically increasing constraint; Gaussian prior (0, 0.01) on max derivative of non-linear intervals	No	-	0.126	No
Dichotomous					
Breast cancer	NA	Yes	Non-smoker population	0.006	No
Otitis media	NA	Yes	Adjusted model	0.014	No

We implemented the Fisher Scoring correction to the heterogeneity parameter, which corrects for data-sparse situations. In such cases, the between-study heterogeneity parameter estimate may be zero, simply from lack of data. The Fisher Scoring correction uses a quantile of gamma, which is sensitive to the number of studies, study design, and reported uncertainty.

Prior to generating an evidence score, we conducted an additional post-analysis step to test and adjust for publication bias in the input data. This approach is based on the classic Egger's regression strategy, which is applied to the residuals in our model. In the current implementation, we do not correct for

publication bias, but flag the riskoutcome pairs where the risk for publication bias is significant. We found evidence of publication bias for LRI studies only.

There is a well-documented attenuation of the risk for cardiovascular disease throughout one's life. Thus, in GBD 2021, to incorporate this age trend in the relative risks, we first identified the median age-at-event across all IHD and stroke cohorts and considered that as the reference age group. We then assigned our risk curves to this reference age group. Next, we applied 1000 draws of the age-specific attenuation factors produced for the smoking curves to 1000 draws of our reference age group's risk curve to determine age-specific risk curves that propagated the uncertainty of both the risk function and age pattern.

Population attributable fraction

For outcomes with a risk a curve, we assigned a specific relative risk to each country-year based on the average number of cigarettes smoked per smoker in that location-year. Relative risks for otitis media and breast cancer from MR-BRT were applied to all countries for all years. Except for IHD and stroke, relative risks were applied to all estimated ages. There was no variation in relative risk by sex. We used the standard GBD population attributable fraction equation for dichotomous risks to estimate burden based on exposure, relative risks, and theoretical minimum risk exposure level.

Smoking

Flowchart

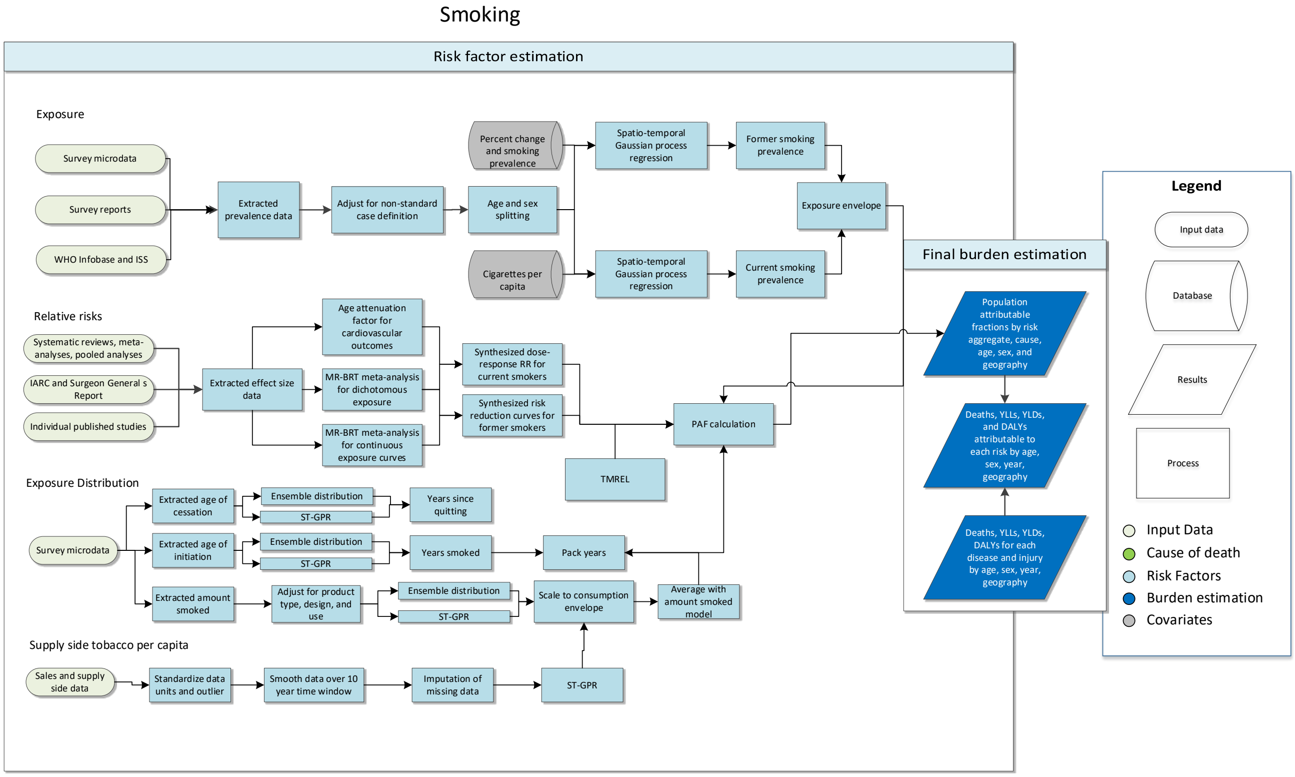

Input data and methodological summary

Definition

Exposure

As in previous GBD cycles, we estimated the prevalence of current smoking and the prevalence of former smoking using data from cross-sectional nationally representative household surveys. We defined current smokers as individuals who currently use any smoked tobacco product on a daily or occasional basis. We defined former smokers as individuals who quit using all smoked tobacco products for at least six months, where possible, or according to the definition used by the given survey.

Input data

Exposure

Our survey data extraction method for smoking exposure has not changed from previous GBD cycles. A systematic review of literature was performed to extract data on our primary exposure indicators. We searched the Global Health Data Exchange (GHDx), a comprehensive online catalog of health-related data created by IHME, for population survey data. We also included surveys that were recommended by our collaborators but were not in the GHDx. Regarding inclusion and exclusion criteria, we only included

surveys that are nationally or subnationally representative (on state/province level). Surveys conducted among specific populations (eg, pregnant women, physicians) were excluded.

We extracted primary data from individual-level microdata and survey report tabulations. Specifically, we extracted data on current, former, and/or ever smoked tobacco use reported as any combination of frequency of use (daily, occasional, and unspecified, which includes both daily and occasional smokers) and type of smoked tobacco used (all smoked tobacco, cigarettes, hookah, and other smoked tobacco products such as cigars or pipes), resulting in 36 possible combinations. Other variants of tobacco products, for example hand-rolled cigarettes, were grouped into the four type categories listed above based on product similarities.

For microdata, we extracted relevant demographic information, including age, sex, location, and year, as well as survey metadata, including survey weights, primary sampling units, and strata. This information allowed us to tabulate individual-level data in the standard GBD five-year age-sex groups and produce accurate estimates of uncertainty. For survey report tabulations, we extracted data at the most granular age-sex group provided. After data were extracted, we carefully vetted the extracted data, fixed any extraction error and cautiously outliered problematic data due to quality concerns based on expert opinion. We documented relevant survey variables from each data source as well as outliered data in spreadsheets. We extracted data using STATA 13.1 and R 3.3.

Table 1: Data inputs for exposure for smoked tobacco

	Countries with data	New sources	Total sources
Exposure	201	164	3603

Relative risk

Since GBD 2016 we had performed systematic review and meta-analysis of all case-control and prospective cohort studies reporting a relative risk, hazard ratio, or odds ratio for any risk-outcome pair studied in GBD 2016. In GBD 2019, we had included 36 risk-outcome pairs for smoking. Studies were included if they reported a categorical or continuous dose for smoked tobacco consumption (pack-years or cigarettes per day) as well as uncertainty measures of the estimated risk, and the population under study was general population. Studies were excluded if they used cross-sectional or retrospective cohort design or if the study was conducted among specific populations (eg, people with diabetes or drug users, etc.).

In GBD 2021, we undertook an effort to improve our relative risk curves by refining our search strings to capture a larger number of studies than was identified by previous search strings. Studies published between 01/01/1970 and 05/31/2022 were reviewed. Of those articles captured in PubMed, prospective cohort and case-control studies were included if they reported the effect sizes (relative risk, hazard ratio, or odds ratio) of an association between a continuous or categorical dose for smoked tobacco consumption and a GBD outcome with uncertainty. Information on study design, confounders controlled for, sample representativeness, and measurement of exposure and outcomes was also extracted.

In GBD 2021, we also employed a new approach to produce age-specific relative risk (RR) curves for CVD outcomes, which involves estimating an age pattern of excess risk (ie, RR–1) of smoking for CVD

outcomes. To estimate the age pattern, we performed a systematic review of literature on risk of smoking for five CVD outcomes, namely, ischaemic heart disease, stroke, atrial fibrillation and flutter, aortic aneurysm, and peripheral arterial disease. We developed a search string to search for articles reporting any association of binary smoking status (ie, current, former, and ever smokers) on the five CVD outcomes from 01/01/1970 to 12/31/2019 and only included studies reporting age-specific risk (RR, OR, HR) of smoking status, which is different from the estimation of dose–response risk of smoking for which we only included studies reporting dose-specific risk. Information on study design, confounders controlled for, sample representativeness, type of exposure (ie, current, former, and ever smoker), measurement of exposure and outcomes was also extracted for bias adjustment. Table 2 summarises the number of studies included for estimating the dose–response risk curve and the age pattern of risk for the CVD outcomes.

Table 2: Data inputs for relative risks for smoked tobacco use

	Countries with data	New sources	Total sources
Relative risks	55	218	730

Data processing

Crosswalk

Our GBD smoking case definitions were current smoking of any tobacco product and former smoking of any tobacco product. All other definitions were adjusted to be consistent with either of these definitions. Some sources contained information on more than one case definition, and these sources were used to develop the adjustment coefficients to transform alternative case definitions to the GBD case definition. The adjustment coefficients were the beta values derived from linear regression models with one predictor and no intercept. We used the same crosswalk adjustment coefficients as in GBD 2019, and thus we have not included a methods explanation in this appendix, as it has been detailed previously.

Age and sex splitting

As in GBD 2019, we split data reported in broader age groups than the GBD five-year age groups or as both sexes combined by adapting the method reported in Ng et al[1] to split using a sex-geography-time-specific reference age pattern. We separated the data into two sets: a training dataset, with data already falling into GBD sex-specific five-year age groups, and a split dataset, which reported data in aggregated age or sex groups. We then used spatiotemporal Gaussian process regression (ST-GPR) to estimate sex-geography-time-specific age patterns using data in the training dataset. The estimated age patterns were used to split each source in the split dataset.

The ST-GPR model used to estimate the age patterns for age-sex splitting used an age weight parameter value that minimises the effect of any age smoothing. This parameter choice allowed the estimated age pattern to be driven by data, rather than being enforced by any smoothing parameters of the model. These age-sex-split datapoints were to be incorporated in the final ST-GPR exposure model; thus, we did

not want to doubly enforce a modelled age pattern for a given sex-location-year on a given aggregate datapoint.

Modelling strategy

Smoking prevalence modelling

We used ST-GPR to model current and former smoking prevalence. The model is identical to that in GBD 2019. Full details on the ST-GPR method are reported elsewhere in the appendix. Briefly, the mean function input to GPR is a complete time series of estimates generated from a mixed effects hierarchical linear model plus weighted residuals smoothed across time, space, and age. The linear model formula for current smoking, fit separately by sex using restricted maximum likelihood in R, is:

$$logit(p_{g,a,t}) = \beta_0 + \beta_1 CPC_{g,t} + \sum_{k=2}^{19} \beta_k I_{A[a]} + \alpha_s + \alpha_r + \alpha_g + \epsilon_{g,a,t}$$

Where $CPC_{g,t}$ is the tobacco consumption per capita covariate by geography g and time t, described above, $I_{A[a]}$ is a dummy variable indicating specific age group that the prevalence point $p_{g,a,t}$ captures, and a_s, a_r, and a_g are super-region, region, and geography random intercepts, respectively. Random effects were used in model fitting but not in prediction.

The linear model formula for former smoking is:

$$logit(p_{g,a,t}) = \beta_0 + \beta_1 PctChange_{A[a],g,t} + \beta_3 CSP_{A[a],g,t} + \sum_{k=3}^{20} \beta_k I_{A[a]} + \alpha_s + \alpha_r + \alpha_g + \epsilon_{g,a,t}$$

Where $PctChange_{A[a],g,t}$ is the percentage change in current smoking prevalence from the previous year, and $CSP_{A[a],g,t}$ is the current smoking prevalence by specific age group A, geography g, and time t that point $p_{g,a,t}$ captures, both derived from the current smoking ST-GPR model defined above.

Supply-side estimation

The methods for modelling supply-side-level data were consistent with those used in GBD 2019. The raw data were domestic supply (USDA Global Surveillance Database and UN FAO) and retail supply (Euromonitor) of tobacco. Domestic supply was calculated as production + imports – exports. The data went through three rounds of outliering based on reasonable consumption thresholds of number of cigarettes per smoker per day, distance from the ten-year rolling mean tobacco per capita, and manual outliering for edge cases. Finally, data smoothing was performed by taking a three-year rolling mean over each location-year.

Next, to impute the missing years for each series and remove compositional bias from our final estimates, we modelled the log ratio of each pair of sources as a function of an intercept and nested

random effects on super-region, region, and location. The appropriate predicted ratio was multiplied by each source that we did have, and then the predictions were averaged to get the final imputed value. For some locations where there was limited overlap between series, the predicted ratio did not make sense, and a regional ratio was used.

Finally, variance was calculated both across series (within a location-year) as well as across years (within a location-source). Additionally, if a location-year had one imputed point, the variance was multiplied by 2. If a location-year had two imputed points, the variance was multiplied by 4. The average estimates in each location-year were the input to an ST-GPR model. For this, we used a simple mixed effects model, which was modelled in log space with nested location random effects. Subnational estimates were then further modelled by splitting the country-level estimates using current smoking prevalence.

Theoretical minimum-risk exposure level

The theoretical minimum-risk exposure level is 0.

Exposure among current and former smokers

Identical to GBD 2019, we estimated exposure among current smokers for two continuous indicators: cigarettes per smoker per day and pack-years. Pack-years incorporates aspects of both duration and amount. One pack-year represents the equivalent of smoking one pack of cigarettes (assuming a 20-cigarette pack) per day for one year. Since the pack-years indicator collapses duration and intensity into a single dimension, one pack-year of exposure can reflect smoking 40 cigarettes per day for six months or smoking 10 cigarettes per day for two years.

To produce these indicators, we simulated individual smoking histories based on distributions of age of initiation and amount smoked. We informed the simulation with cross-sectional survey data capturing these indicators, modelled at the mean level for all locations, years, ages, and sexes using ST-GPR. We rescaled estimates of cigarettes per smoker per day to an envelope of cigarette consumption based on supply-side data. We estimated pack-years of exposure by summing samples from age- and time-specific distributions of cigarettes per smoker for a birth cohort to capture both age trends and time trends and avoid the common assumption that the amount someone currently smokes is the amount they have smoked since they began smoking. All distributions were age-, sex-, and region-specific ensemble distributions, which were found to outperform any single distribution.

We estimated exposure among former smokers using years since cessation. We used ST-GPR to model mean age of cessation using cross-sectional survey data capturing age of cessation. Using these estimates, we generated ensemble distributions of years since cessation for every location, year, age group, and sex.

Relative risk

The same risk-outcome pairs from GBD 2019 were used for GBD 2021: tuberculosis, lower respiratory tract infections, oesophageal cancer, stomach cancer, bladder cancer, liver cancer, laryngeal cancer, lung cancer, breast cancer, cervical cancer, colorectal cancer, lip and oral cancer, nasopharyngeal cancer, other pharyngeal cancer, pancreatic cancer, kidney cancer, leukaemia, ischaemic heart disease,

ischaemic stroke, haemorrhagic stroke, subarachnoid haemorrhage, atrial fibrillation and flutter, aortic aneurysm, peripheral arterial disease, chronic obstructive pulmonary disease, other chronic respiratory diseases, asthma, peptic ulcer disease, gallbladder and biliary tract diseases, Alzheimer's disease and other dementias, Parkinson's disease (protective), multiple sclerosis, type 2 diabetes, rheumatoid arthritis, low back pain, cataracts, macular degeneration, and fracture.

For GBD 2021, the risk of all risk-outcome pairs is evaluated by continuous smoking exposure level (ie, pack-year, cigarettes per smoker per day, and years since cessation), expect for fracture, whose risk is evaluated by binary smoking exposure (ie, smoker versus non-smoker/former smoker).

Dose–response risk curves

Since GBD 2016, we had used the studies identified through the systematic review to estimate dose–response risk of smoking on related health outcomes using DisMod ODE. We chose DisMod ODE rather than a conventional mixed effects meta-regression because of its ability to estimate nonparametric splines over doses (ie, there is usually a non-linear relationship between smoking exposure level and outcome risk) and incorporate heterogeneous doses through dose integration (ie, most studies report smoking exposure level categorically in wide ranges, and DisMod ODE can estimate risk of specific exposure level when categories overlap across studies, through an integration step).

For GBD 2021, we used the studies identified through the updated systematic review to estimate new dose–response curves using MR-BRT for all outcomes. Importantly, this new method takes into account the risk of biases in the RR estimation by selecting and including important covariates of the risk estimates in the model (eg, measurement of exposure and outcomes, representativeness, and adjustment level of the risk estimates) and incorporates unexplained between-study heterogeneity into the uncertainty of the RR estimates. The results of the meta-regression were used to estimate a non-parametric curve for all doses between zero and 100 pack-years or cigarettes per smoker per day and their corresponding relative risks. For all outcomes, we assumed the relative risk was the same for both sexes, expect for breast cancer, cervical cancer, and prostate cancer, which were assumed to apply only to female or to male.

For data-sparse risk–outcome pairs, we implemented the Fisher scoring correction to the heterogeneity parameter. When data are sparse, the between-study heterogeneity parameter estimate may be 0, simply due to lack of data. The Fisher scoring correction uses a quantile of gamma, which is sensitive to the number of studies, study design, and reported uncertainty.

We have also added methodology that can detect and flag publication bias. The approach is based on the classic Egger's regression strategy, which is applied to the residuals in our model. In the current implementation, we do not correct for publication bias, but flag the risk–outcome pairs where the risk for publication bias is significant.

For risk of former smokers, we estimated risk curves of former smokers compared to never smokers taking into account the rate of risk reduction among former smokers seen in the cohort and case-control studies, and the cumulative exposure among former smokers within each age, sex, location, and year

group. For GBD 2021, we did not include new data or change the method of estimating the risk curves of former smokers.

In the table below, we list each risk–outcome pair that is updated in GBD 2021 along with several of the key modelling parameters and results. The formulation for MR-BRT is described in detail in the appendix. More detail on the modeling strategy and results can be found in Dai et al., 2022.[3]

Table 5: MR-BRT model specifications by risk–outcome pair

Risk-outcome	Type of risk	Spline degree, # interior knots	Priors & constraints
Atrial fibrillation and flutter	Continuous, harmful	Quadratic, 3 I knots	Monotonic increasing, right linear tail, Gaussian max derivative prior on the right tail (0, 0.001)
Alzheimer's and other dementias	Continuous, harmful	Quadratic, 3 I knots	Monotonic increasing, right linear tail, Gaussian max derivative prior on the right tail (0, 0.001)
Aortic aneurism	Continuous, harmful	Quadratic, 3 I knots	Monotonic increasing, right linear tail, Gaussian max derivative prior on the right tail (0, 0.001)
Asthma	Continuous, harmful	Quadratic, 3 I knots	Monotonic increasing, right linear tail, Gaussian max derivative prior on the right tail (0, 0.001)
Bladder cancer	Continuous, harmful	Quadratic, 3 I knots	Monotonic increasing, right linear tail, Gaussian max derivative prior on the right tail (0, 0.001)
Breast cancer	Continuous, harmful	Quadratic, 3 I knots	Monotonic increasing, right linear tail, Gaussian max derivative prior on the right tail (0, 0.001)
Cataracts	Continuous, harmful	Quadratic, 3 I knots	Monotonic increasing, right linear tail, Gaussian max derivative prior on the right tail (0, 0.001)
Cervical cancer	Continuous, harmful	Quadratic, 3 I knots	Monotonic increasing, right linear tail, Gaussian max derivative prior on the right tail (0, 0.001)
Colon and rectum cancer	Continuous, harmful	Quadratic, 3 I knots	Monotonic increasing, right linear tail, Gaussian max derivative prior on the right tail (0, 0.001)
COPD	Continuous, harmful	Quadratic, 3 I knots	Monotonic increasing, right linear tail, Gaussian max derivative prior on the right tail (0, 0.001)
Diabetes	Continuous, harmful	Quadratic, 3 I knots	Monotonic increasing, right linear tail, Gaussian max derivative prior on the right tail (0, 0.001)
Oesophageal cancer	Continuous, harmful	Quadratic, 3 I knots	Monotonic increasing, right linear tail, Gaussian max derivative prior on the right tail (0, 0.001)

Gallbladder diseases	Continuous, harmful	Quadratic, 3 l knots	Monotonic increasing, right linear tail, Gaussian max derivative prior on the right tail (0, 0.001)
Fracture (hip and non-hip)	Dichotomous, harmful	N/A	N/A
Ischaemic heart disease	Continuous, harmful	Quadratic, 3 l knots	Monotonic increasing, right linear tail, Gaussian max derivative prior on the right tail (0, 0.001)
Kidney cancer	Continuous, harmful	Quadratic, 3 l knots	Monotonic increasing, right linear tail, Gaussian max derivative prior on the right tail (0, 0.001)
Laryngeal cancer	Continuous, harmful	Quadratic, 3 l knots	Monotonic increasing, right linear tail, Gaussian max derivative prior on the right tail (0, 0.001)
Lower back pain	Continuous, harmful	Quadratic, 3 l knots	Monotonic increasing, right linear tail, Gaussian max derivative prior on the right tail (0, 0.001)
Leukemia	Continuous, harmful	Quadratic, 3 l knots	Monotonic increasing, right linear tail, Gaussian max derivative prior on the right tail (0, 0.001)
Lip and oral cavity cancer	Continuous, harmful	Quadratic, 3 l knots	Monotonic increasing, right linear tail, Gaussian max derivative prior on the right tail (0, 0.001)
Liver cancer	Continuous, harmful	Quadratic, 3 l knots	Monotonic increasing, right linear tail, Gaussian max derivative prior on the right tail (0, 0.001)
Lower respiratory infections	Continuous, harmful	Quadratic, 3 l knots	Monotonic increasing, right linear tail, Gaussian max derivative prior on the right tail (0, 0.001)
Lung cancer	Continuous, harmful	Quadratic, 3 l knots	Monotonic increasing, right linear tail, Gaussian max derivative prior on the right tail (0, 0.001)
Macular degeneration	Continuous, harmful	Quadratic, 3 l knots	Monotonic increasing, right linear tail, Gaussian max derivative prior on the right tail (0, 0.001)
Multiple sclerosis	Continuous, harmful	Quadratic, 3 l knots	Monotonic increasing, right linear tail, Gaussian max derivative prior on the right tail (0, 0.001)
Nasopharyngeal cancer	Continuous, harmful	Quadratic, 3 l knots	Monotonic increasing, right linear tail, Gaussian max derivative prior on the right tail (0, 0.001)
Other pharynx cancer	Continuous, harmful	Quadratic, 3 l knots	Monotonic increasing, right linear tail, Gaussian max derivative prior on the right tail (0, 0.001)
Pancreatic cancer	Continuous, Harmful	Quadratic, 3 l knots	Monotonic increasing, right linear tail, Gaussian max derivative prior on the right tail (0, 0.001)

Parkinson's disease	Continuous, protective	Quadratic, 3 I knots	Monotonic decreasing, right linear tail, Gaussian max derivative prior on the right tail (0, 0.001)
Peptic ulcer	Continuous, harmful	Quadratic, 3 I knots	Monotonic increasing, right linear tail, Gaussian max derivative prior on the right tail (0, 0.001)
Peripheral artery disease	Continuous, harmful	Quadratic, 3 I knots	Monotonic increasing, right linear tail, Gaussian max derivative prior on the right tail (0, 0.001)
Prostate cancer	Continuous, harmful	Quadratic, 3 I knots	Monotonic increasing, right linear tail, Gaussian max derivative prior on the right tail (0, 0.001)
Rheumatoid arthritis	Continuous, harmful	Quadratic, 3 I knots	Monotonic increasing, right linear tail, Gaussian max derivative prior on the right tail (0, 0.001)
Stomach cancer	Continuous, harmful	Quadratic, 3 I knots	Monotonic increasing, right linear tail, Gaussian max derivative prior on the right tail (0, 0.001)
Stroke (ischaemic stroke, haemorrhagic stroke, and subarachnoid haemorrhage)	Continuous, harmful	Quadratic, 3 I knots	Monotonic increasing, right linear tail, Gaussian max derivative prior on the right tail (0, 0.001)
Tuberculosis	Continuous, harmful	Quadratic, 3 I knots	Monotonic increasing, right linear tail, Gaussian max derivative prior on the right tail (0, 0.001)

Table 6: MR-BRT estimated parameters and bias covariates by risk–outcome pair

Risk–outcome	Unit of risk	Selected bias covariates	Mean gamma solution	publication bias
Atrial fibrillation and flutter	cigarettes per day	None	0.000	
Alzheimer's and other dementias	cigarettes per day	None	0.054	
Aortic aneurism	cigarettes per day	None	0.000	
Asthma	cigarettes per day	None	1.651	
Bladder cancer	pack-year	None	0.052	
Breast cancer	pack-year	cv_subpopulation	0.000	
Cataracts	cigarettes per day	None	0.000	
Cervical cancer	pack-year	None	0.000	
Colon and rectum cancer	pack-year	None	0.090	
COPD	pack-year	cv_older, cv_adj_L1	0.022	
Diabetes (type 2)	cigarettes per day	cv_subpopulation	0.055	
Oesophageal cancer	pack-year	None	0.106	
Gallbladder diseases	cigarettes per day	cv_adj_L0	0.000	

Fracture (hip and non-hip)	Binary smoking status	cv_subpopulation, cv_risk_measure, cv_adj_L2	0.032	
Ischaemic heart disease	cigarettes per day	cv_adj_L2, cv_subpopulation, cv_older	0.190	
Kidney cancer	pack-year	None	0.078	
Laryngeal cancer	pack-year	None	0.000	
Lower back pain	cigarettes per day	None	0.000	
Leukemia	pack-year	None	0.000	
Lip and oral cavity cancer	pack-year	cv_adj_L1	0.158	
Liver cancer	pack-year	None	0.429	
Lower respiratory infection	cigarettes per day	None	0.000	
Lung cancer	pack-year	cv_adj_L1, cv_adj_L0, cv_adj_L2	0.063	
Macular degeneration	cigarettes per day	None	0.000	
Multiple sclerosis	cigarettes per day	None	0.000	
Nasopharyngeal cancer	pack-year	cv_adj_L0	0.065	
Other pharynx cancer	pack-year	None	0.000	
Pancreatic cancer	pack-year	None	0.000	
Parkinson's disease	cigarettes per day	cv_adj_L2, cv_outcome_selfreport	0.000	
Peptic ulcer	cigarettes per day	cv_adj_L1, cv_subpopulation	0.000	
Peripheral artery disease	cigarettes per day	cv_subpopulation	0.000	
Prostate cancer	cigarettes per day	None	0.170	
Rheumatoid arthritis	cigarettes per day	None	0.000	
Stomach cancer	pack-year	None	0.000	
Stroke (ischaemic stroke, haemorrhagic stroke, and subarachnoid haemorrhage)	cigarettes per day	None	0.146	
Tuberculosis	cigarettes per day	None	0.038	

† definitions of bias covariates:

cv_subpopulation: 0 for risk estimates are likely generalisable to the general population because the sample was based on the general population with reasonable exclusions for pre-existing disease states; 1 for risk estimates of sub-groups such as high-risk groups

cv_adj_L0, cv_adj_L1, cv_adj_L2: cascading dummy variables for adjustment level of the risk estimates (ie, how many confounders are adjusted for in the regression model for the risk estimate). There are four adjustment levels, namely, 1. no adjustment, 2. only adjusting for age and sex, 3. adjusting for age and sex and ≤3 other covariates, and 4. adjusting for age and sex and >3 other covariates. If the adjustment level is 1, cv_adj_L0=1, cv_adj_L1=1, cv_adj_L2=1; if the adjustment level is 2, cv_adj_L0=1, cv_adj_L1=1, cv_adj_L2=0; if the adjustment level is 3, then cv_adj_L0=1, cv_adj_L1=0, cv_adj_L2=0; if the adjustment level is 4, then cv_adj_L0=0, cv_adj_L1=0, cv_adj_L2=0.

cv_outcome_selfreport: 0 for measurement of outcome based on assays, tests, or physician observation and 1 for self-report outcome.

cv_older: 0 if the population contains both young and old people; 1 if the population only contains old people.

cv_risk_measure: 0 if the risk is reported as Relative Risk; 1 if the risk is reported as Odds Ratio or Hazard Ratios.

Age-specific dose–response risk curves for CVD outcomes

For all non-CVD outcomes, we assumed the risk curve to be the same for all ages. However, the risk of smoking on CVD outcomes (ie, stroke, ischaemic heart disease, atrial fibrillation and flutter, aortic aneurysm, and peripheral arterial disease) is well known to attenuate with increasing age, and thus we produced age-specific risk curves for all CVD outcomes. Previously, we used a linear relationship between age and log risk to adjust all RR data to a specific age group (eg, 45–49). Then, we modelled the risk curve for each age group using the adjusted age-group-specific data. This approach often produced curves with different shapes for different age groups and tended to underestimate the risk for older age groups since we set the log RR to be 0 for the terminal age group (eg, 95+) in the linear function.

In GBD 2021, we adopted a new approach to produce the age-specific risk curves by producing an age pattern of smoking risk on CVD outcomes and adjusting the risk curve of the reference age group using the age pattern of risk to produce age-group-specific risk curves. Briefly, we first estimated the reference dose–response risk of smoking for each CVD outcome using dose-specific RR data of each outcome regardless of the age group information. This step was the same with other non-CVD outcomes. Once we had the reference curve, we determined the age group of the reference curve by calculating the weighted mean age across all dose-specific RR data (weighted by 1/SE of each datum). For example, if the weighted mean age of all dose-specific RR data was 56.5, we determined the age group of the reference risk curve to be 55–59. For cohort studies, the mean age was calculated as mean age at baseline plus the mean/median years of follow-up (if only maximum years of follow-up is reported, we added half of the maximum years to the mean age at baseline). For the case-control studies, the mean age was just the reported mean age at baseline (in case the mean age is not reported, we used the midpoint of age range as the mean age instead). In the third step, we extracted age-group-specific RR data and relevant bias covariates from literature identified in the systematic review mentioned above, and we used MR-BRT to model the age pattern of excess risk (ie, RR–1) of smoking on CVD outcomes with age-group-specific excess RR data of all CVD outcomes. In the final model, we included age as spline, random effects of study, and the bias covariates of exposure types (ie, current, former, and ever smokers), which were selected by an algorithm described elsewhere.[2] When predicting the age pattern of the excess risk of smoking on CVD outcomes using the fitted model, we did not include between-study heterogeneity to reduce uncertainty in the prediction. Figure S1 below shows the estimated age pattern of excess risk of smoking on CVD along with its 95% uncertainty intervals. In the fourth step, we calculated the age attenuation factors (AF) of excess risk compared with the reference age group for each CVD outcome as ratio of the estimated excess risk of each age group to that of the reference age group. We did the calculation at the draw level to obtain 1000 draws of the AF for each age group. Figure S2 below shows the AF for stroke along with its 95% uncertainty intervals. Once we had the AF, in the last step, we adjusted the risk curve of the reference age group from step 1 using equation (1) to produce the age-group-specific risk curves for each CVD outcome.

$$rr_{age_i} = \left(rr_{ref} - 1\right) * AF_{age_i} + 1 \qquad (1)$$

We did the age adjustment on draw level so that the uncertainty of the AF can be naturally incorporated in the final adjusted age-specific RR curves. Figure S3 shows the age AF adjusted age-group-specific RR curves for stroke outcome.

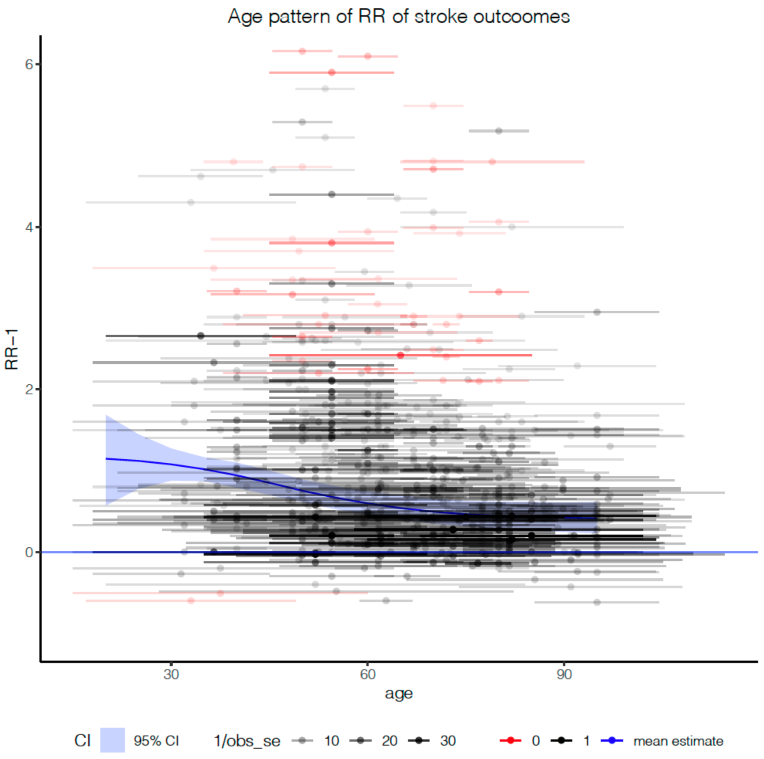

Figure S1: estimated age pattern of excess risk of smoking on CVD outcomes

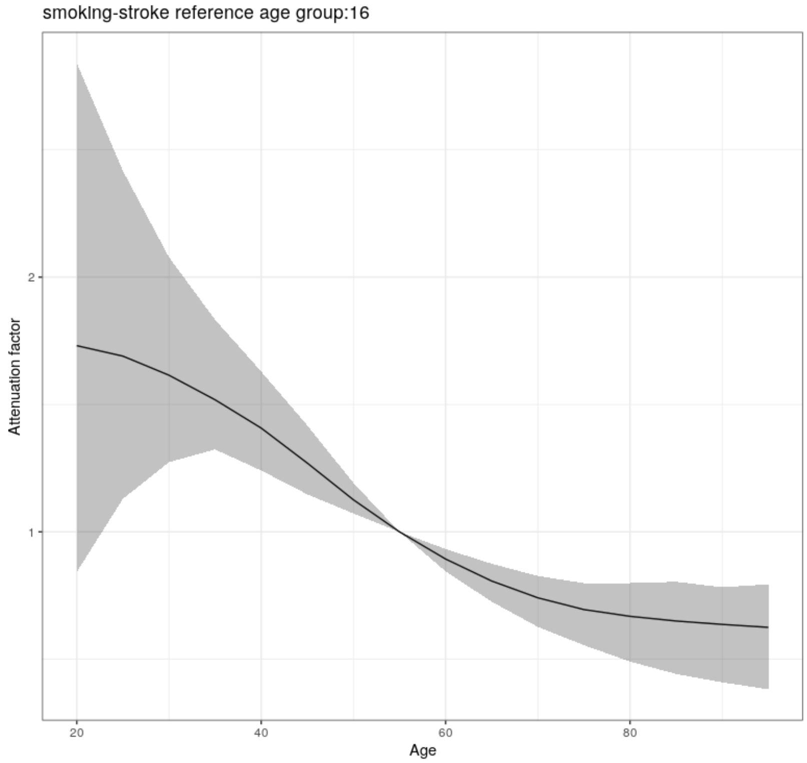

Figure S2: attenuation factors of excess risk of smoking on stroke campared with the reference age group

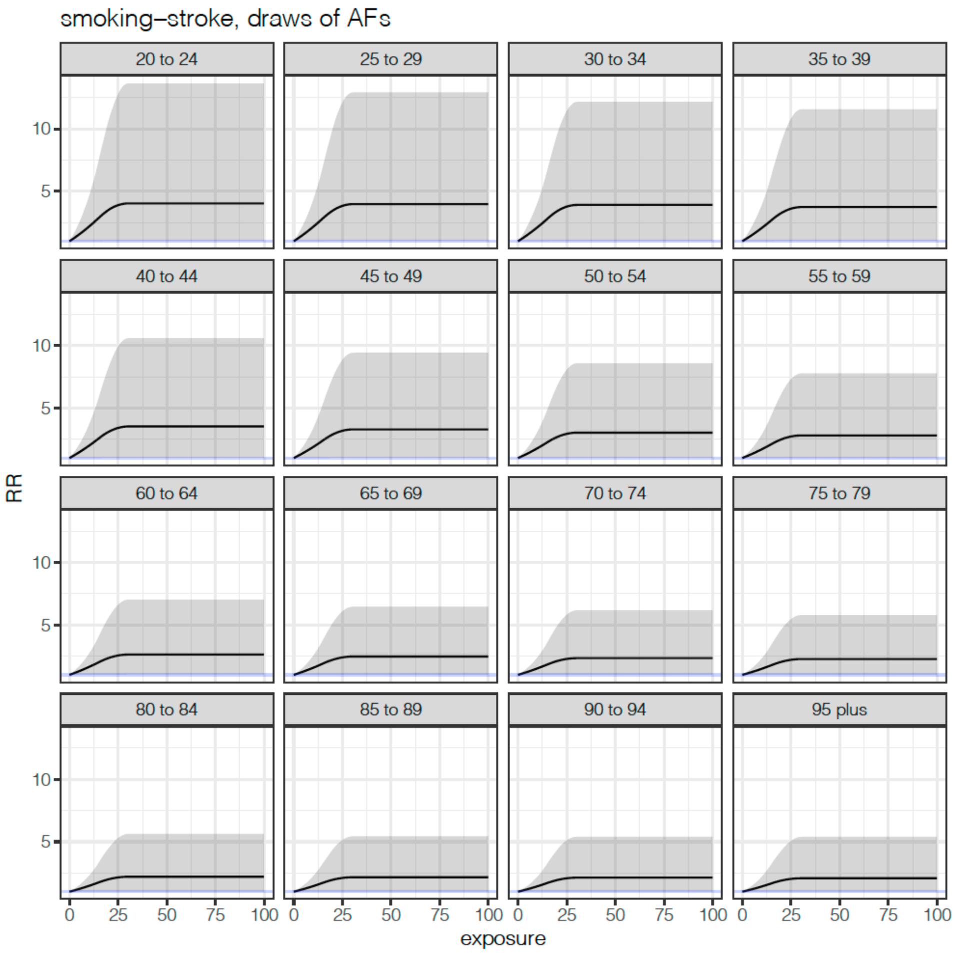

Figure S3: AF adjusted age-group specific RR curves for stroke, reference age group 55–59. The exposure is in units of cigarette-equivalents per smoker per day.

Population attributable fraction (PAF)

As in GBD 2019, we estimated PAFs based on the following equation:

$$PAF = \frac{p(n) + p(f)\int \exp(x) * rr(x) + p(c)\int \exp(y) * rr(y) - 1}{p(n) + p(f)\int \exp(x) * rr(x) + p(c)\int \exp(y) * rr(y)}$$

where $p(n)$ is the prevalence of never smokers, $p(f)$ is the prevalence of former smokers, $p(c)$ is the prevalence of current smokers, $exp(x)$ is a distribution of years since quitting among former smokers, $rr(x)$ is the relative risk for years since quitting, $exp(y)$ is a distribution of cigarettes per smoker per day or pack-years, and $rr(y)$ is the relative risk for cigarettes per smoker per day or pack-years.

We used pack-years as the exposure definition for cancers and chronic respiratory diseases, and cigarettes per smoker per day for cardiovascular diseases and all other health outcomes.

Citations

1. Ng M, Freeman MK, Fleming TD, Robinson M, Dwyer-Lindgren L, Thomson B, et al. Smoking prevalence and cigarette consumption in 187 countries, 1980-2012. JAMA. 2014 Jan 8;311(2):183–92.

2. Zheng P, Aravkin A, Barber R, Sorensen R, Murray C. Trimmed Constrained Mixed Effects Models: Formulations and Algorithms. bioRxiv. 2020 Jan 29;2020.01.28.923599.

3. Dai, X., Gil, G. F., Reitsma, M. B., Ahmad, N. S., Anderson, J. A., Bisignano, C., Carr, S., Feldman, R., Hay, S. I., He, J., Iannucci, V., Lawlor, H. R., Malloy, M. J., Marczak, L. B., McLaughlin, S. A., Morikawa, L., Mullany, E. C., Nicholson, S. I., O'Connell, E. M., … Gakidou, E. (2022). Health effects associated with smoking: A Burden of Proof study. Nature Medicine, 28(10), 2045–2055. https://doi.org/10.1038/s41591-022-01978-x

Suboptimal breastfeeding

Flowchart

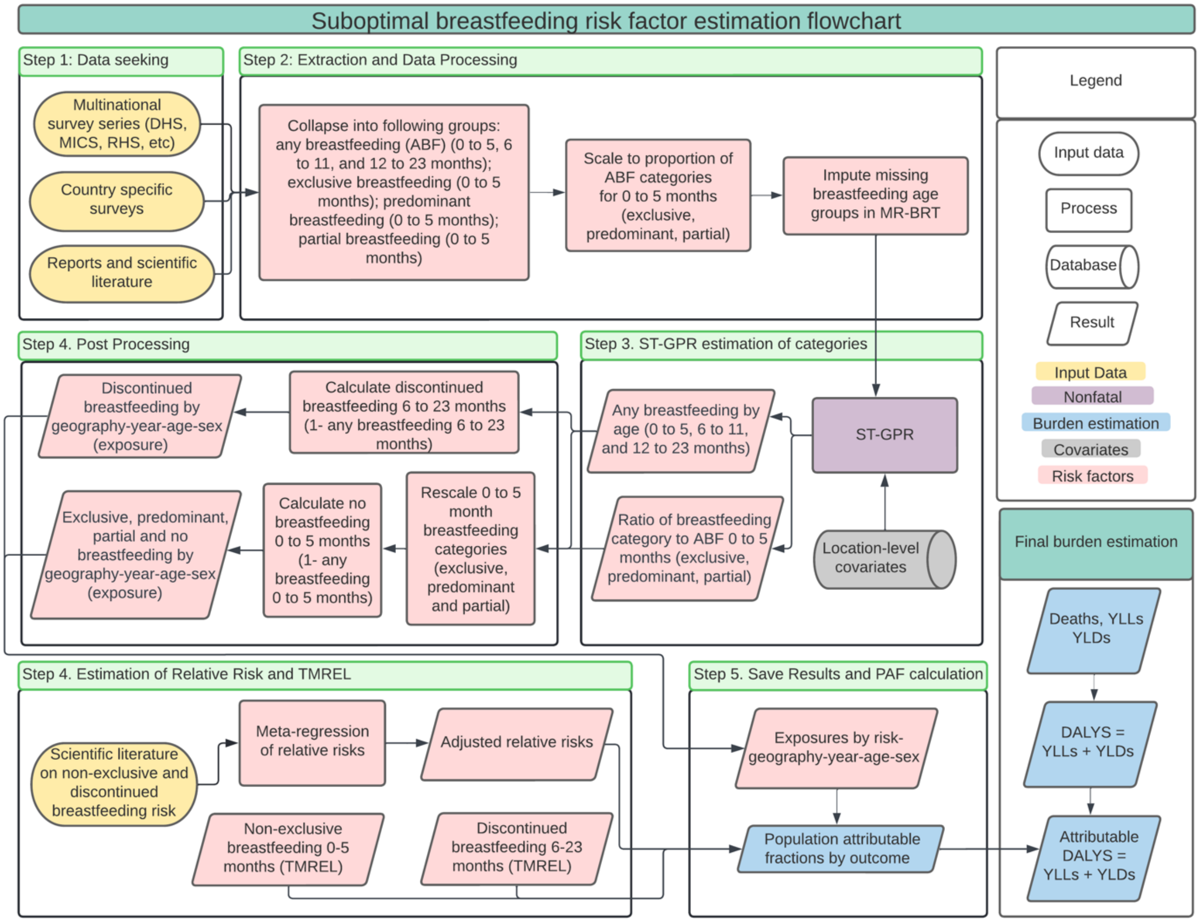

Input data and methodological summary

Definition

Exposure

Exposure to suboptimal breastfeeding is composed of two distinct categories: non-exclusive breastfeeding and discontinued breastfeeding.

Non-exclusive breastfeeding is defined as the proportion of children under 6 months of age who are not exclusively breastfed. We then parse those not exclusively breastfed into three categories – predominant, partial, and no breastfeeding. Exclusive breastfeeding is defined as the proportion of children who receive no other food or drink except breastmilk (allowing for ORS, drops, or syrups containing vitamins, minerals, or medicines). Predominant breastfeeding is the proportion of children whose predominant source of nourishment is breastmilk but also receive other liquids. Partial breastfeeding refers to those infants who receive breastmilk as well as food and liquids, including non-

human milk and formula. No breastfeeding refers to infants who do not receive breastmilk as a source of nourishment.

Discontinued breastfeeding is defined as the proportion of children between 6 and 23 months who receive no breastmilk as a source of nourishment.

Input data

Exposure

The data used in the analysis consist mostly of processed individual-level microdata from surveys; in the cases where microdata were unavailable, we used reported tabulated data from survey reports and scientific literature. Data used to categorise type of non-exclusive breastfeeding (predominant, partial, and none) come from surveys with 24-hour dietary logs based on maternal recall.

We updated our systematic review in GBD 2021 by searching the Global Health Data Exchange (GHDx) using the keyword "breastfeeding." We prioritised extraction of surveys with microdata and new surveys from major survey series such as Demographic and Health Surveys (DHS) and Multiple Indicator Cluster Surveys (MICS).

Table 1. Input data counts – suboptimal breastfeeding exposure

	**Countries with data**	**New sources**	**Total sources**
Non-exclusive breastfeeding	169	49	737
Discontinued breastfeeding	162	50	679

To better ensure consistency in estimates across age groups, we identified location-years where we had data for "any breastfeeding 6–11 months" but no data for "any breastfeeding 12–23 months." We then imputed data for "any breastfeeding 12–23 months" based on the observed 6–11month datapoint in that location-year. We estimated the imputation adjustment by meta-analysing proportion ratios of matched pairs by source-location-year for any breastfeeding in these two age groups in meta-regression—Bayesian, regularised, trimmed (MR-BRT)[1], a Bayesian meta-analytic tool.

Table 2. MR-BRT adjustment factor for any breastfeeding 12–23 months imputation

**Data input**	**Reference or alternative definition**	**Gamma**	**Beta coefficient, logit (95% UI)***	**Adjustment factor****
Any breastfeeding 6–11 months	Ref	0.19	---	---
Any breastfeeding 12–23 months	Alt		1.54 (-1.58 1.50)	0.21 (0.21–0.22)

**MR-BRT crosswalk adjustments can be interpreted as the factor the alternative definition is adjusted by to reflect what it would have been had it been measured using the reference definition.*
***The adjustment factor column is the exponentiated beta coefficient. For logit beta coefficients, this is the relative odds between the two definitions.*

Relative risk

We included outcomes based on the strength of available evidence supporting a causal relationship. Studies evaluating the causal evidence for our riskoutcome pairs came primarily from studies compiled in a published review by the World Health Organization.[2] Non-exclusive breastfeeding was paired with diarrhoea and lower respiratory infection as diseases outcomes. Discontinued breastfeeding was paired with diarrhoea as an outcome.

Table 3. Input data counts – suboptimal breastfeeding relative risk

	**Countries with data**	**New sources**	**Total sources**
Relative risk	26	0	43

Modelling strategy

Exposure

Using the processed microdata and tabulated data from reports, we generated a complete time series from 1980 to 2022 for 1) any breastfeeding 0–5 months, 6–11 months, and 12–23 months, 2) ratio of exclusive breastfeeding to any breastfeeding 0–5 months, 3) ratio of predominant breastfeeding to any breastfeeding 0–5 months, and 4) ratio of partial breastfeeding to any breastfeeding 0–5 months using a three-step spatiotemporal Gaussian process regression. In previous GBD rounds, “any breastfeeding” was modelled separately for each of the estimated age groups. In GBD 2021 with the addition of new under-5 age groups that aligned with those ages we model, we incorporated the three age groups into a single model of “any breastfeeding.” This allowed us to borrow additional strength over space, age, and time by incorporating data from all sources in one model.

The first step of the ST-GPR process is an ensemble linear mixed-effects regression of our data on a set of potentially predictive covariates taken from the GBD covariates database. We tested every combination of these covariates in individual, sex-specific mixed-effects linear regressions with nested random effects at the super-region, region, and location levels. We then evaluated and ranked each of these sub-models by their out-of-sample root-mean-squared error. Finally, to produce initial estimates for every location, year, age, and sex in the analysis, we averaged the 50 top-performing models where the estimated coefficients were 1) statistically significant at $p < 0.05$ and 2) in the expected direction. We tested the following covariates in the ensemble prior: Socio-demographic Index, SEV unsafe water, total fertility rate, maternal education, antenatal care (4+ visits), HIV mortality in women of reproductive age, high BMI in women of reproductive age, and underweight in women of reproductive age.

The second, spatiotemporal smoothing step of ST-GPR calculates the residual between our stage 1 regression estimate and each of our observed datapoints and then smooths this residual, drawing

strength over space, age, and time and producing a revised stage 2 estimate of birth prevalence for every location, year, and sex. The third step of ST-GPR is a Gaussian process regression, using the stage 2 estimates as a prior and the observed datapoints and their variance to 1) further smooth the residual between the stage 2 predictions and observed data and produce a final mean estimate for each location, year, and sex, and 2) estimate uncertainty around this mean estimate, quantified by taking 1000 draws from the posterior Gaussian process. More detailed information on the ST-GPR modelling process can be found in the main text methods appendix.

To generate exposure categories for non-exclusive breastfeeding, we converted the modelled ratios of exclusive, predominant, and partial breastfeeding to the total category prevalence by multiplying each ratio by the estimates of any breastfeeding among infants aged 0–5 months. This ensured that these categories sum correctly to the "any breastfeeding 0–5 months" envelope. We calculated the proportion of infants receiving no breastmilk 0–5 months of age by subtracting the estimates of current breastfeeding from 1. We performed the same operation to estimate discontinued breastfeeding in the 6–11 months and 12–23 months categories.

Theoretical minimumrisk exposure level

For non-exclusive breastfeeding, those children that received no source of nourishment other than breastmilk ("exclusively breastfed") were considered to be at the lowest risk of any of the disease outcomes. For discontinued breastfeeding, we assumed that children aged 6–23 months who received any breastmilk as a source of nourishment to be at the lowest risk of disease outcome.

Relative risk

We estimated relative risks for both non-exclusive and discontinued breastfeeding in a meta-analysis using the "metareg" package in Stata. For the 0–5 month age group we included diarrhoea and lower respiratory infection as outcomes, and for the 6–23 month age group we included diarrhoea as an outcome. We did not estimate separate relative risks for morbidity and mortality. The estimated relative risks are detailed in Table 4.

Table 4. Suboptimal breastfeeding relative risk estimates

**Exposure category**	**Diarrhoea**		**Lower respiratory infection**	
	**Mortality**	**Morbidity**	**Mortality**	**Morbidity**
0–5 months				
Exclusive breastfeeding	1.00	1.00	1.00	1.00
Predominant breastfeeding	2.35 (1.67–3.23)	2.35 (1.67–3.23)	1.37 (1.06–1.80)	1.37 (1.06–1.80)
Partial breastfeeding	2.63 (1.94–3.48)	2.63 (1.94–3.48)	1.48 (1.21–1.79)	1.48 (1.21–1.79)

No breastfeeding	3.60<br>(2.72–4.70)	3.60<br>(2.72–4.70)	1.74<br>(1.49–2.03)	1.74<br>(1.49–2.03)
6–23 months				
Any breastfeeding	1.00	1.00	--	--
Discontinued breastfeeding	1.31<br>(1.11–1.55)	1.31<br>(1.11–1.55)	--	--

Population attributable fraction

We used the standard GBD population attributable fraction (PAF) equation to calculate PAFs for non-exclusive breastfeeding and discontinued breastfeeding and each of their paired outcomes using exposure estimates, the theoretical minimumrisk exposure level, and relative risks.

Citations

1. Vos T, Lim SS, Abbafati C, et al. Global burden of 369 diseases and injuries in 204 countries and territories, 1990–2019: a systematic analysis for the Global Burden of Disease Study 2019. The Lancet 2020; 396: 1204–22.
2. Horta, B., Voctora, C. (2013) Short-term effects of breastfeeding: a systematic review on the benefits of breastfeeding on diarrhoea and pneumonia mortality. The World Health Organization.

Unsafe sanitation

Flowchart

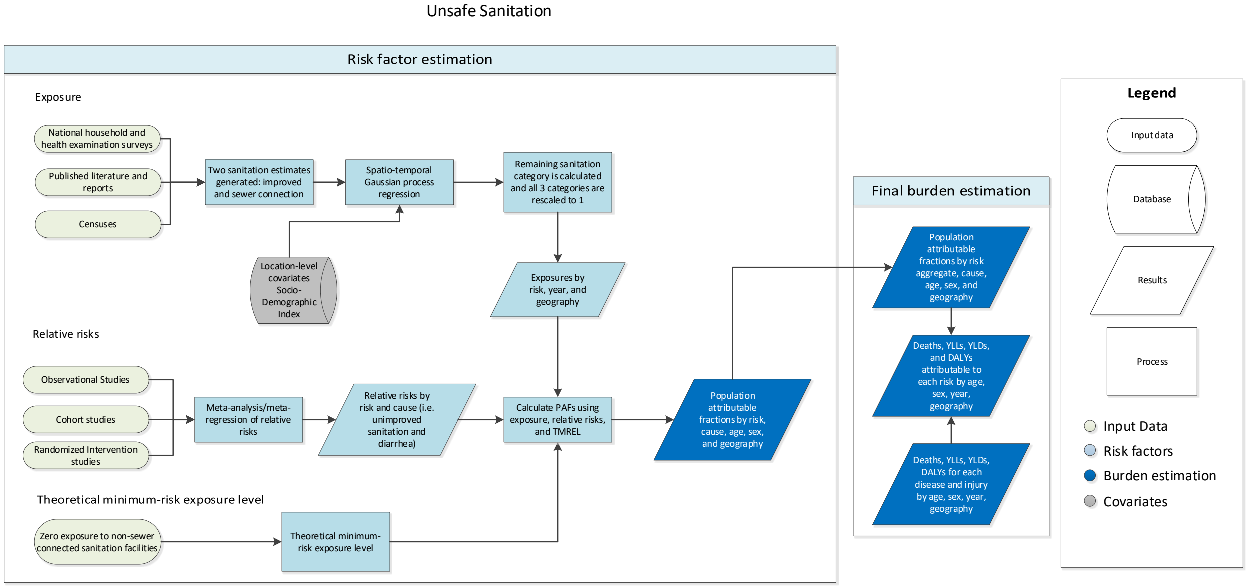

Input data and methodological summary

Exposure

Case definition

Exposure to unsafe sanitation is defined based on the primary toilet type used by households. For the Global Burden of Diseases, Injuries, and Risk Factors Study (GBD) 2021, we modelled three different categories of sanitation: unimproved, improved, and facilities with a sewer connection or septic tank. These categories were defined according to the WHO/UNICEF Joint Monitoring Programme for Water Supply, Sanitation and Hygiene (JMP).[1] Examples of "improved" sanitation facilities include ventilated improved pit latrines, composting toilets, and pit latrines with slabs. Examples of "unimproved" facilities include open pit latrines, open defecation, and toilets that flush into creeks or open fields. Sewer connection toilets include flush toilets or any toilet with connection to the sewer or septic tank.

Input data

The search for usable data sources was conducted using the Global Health Data Exchange (GHDx) database. Input data came primarily from nationally representative surveys, such as the Demographic and Health Survey (DHS), the Multiple Indicator Cluster Surveys (MICS), the World Health Survey (WHS), and the DHS AIDS Indicator Survey (AIS). Surveys that reported results at the household level were converted to the individual level using household size data, to ensure that our models estimated the proportion of individuals, rather than households, exposed to a given indicator. Surveys and censuses were then tabulated to two sanitation categories, sewer connection and improved sanitation, for each location. Table 1 provides a summary of the input data used.

Table 1: Exposure input data

Input data	Exposure
Source count (total)	1153
Number of countries with data	159

Modelling

For GBD 2021, sanitation was modelled in an ordinal framework. Two distinct indicators were estimated: (1) the proportion of the total population using sewer connection or septic tank facilities, and (2) the proportion of individuals using improved sanitation within the population not connected to a sewer or septic tank. This ordinal framework allows us to estimate the category with the most data (sewer connection/septic tank prevalence) and leverage that estimate to anchor the estimates for the improved and unimproved sanitation categories. The results of the improved-proportion model are multiplied by one minus the sewer connection/septic tank prevalence to calculate improved sanitation prevalence. The sum of improved and sewer connection/septic tank prevalence are subtracted from 1 to yield unimproved sanitation prevalence.

$$\boldsymbol{Sewer} = \frac{\#\, persons\ with\ sewer\ or\ septic\ connection}{\#\, persons\ with\ nonmissing\ response}$$

$$\boldsymbol{Improved} = \left(\frac{\#\, persons\ using\ improved\ facilities}{\#\, persons\ without\ sewer\ or\ septic\ connection}\right) * (1 - \boldsymbol{Sewer})$$

$$\boldsymbol{Unimproved} = 1 - (\boldsymbol{Sewer} + \boldsymbol{Improved})$$

The two indicators were each modelled using a three-step modelling scheme of mixed effect linear regression followed by spatiotemporal Gaussian process regression (ST-GPR), which produced full time-series estimates for each GBD 2021 location. Socio-demographic Index (SDI), a composite metric combining education per capita, income per capita, and fertility, was set as a fixed effect in the linear regression since it proved to be a significant predictor. Random effects were set at GBD 2021 region and super-region levels to fit the models but were not used in the predictions. The same linear regression equation was used for both ST-GPR models (see below).

$$logit(data) \sim sdi + (1|level\_1) + (1|level\_2)$$

SDI = Socio-demographic Index
(1|level_1) = super-region-level random effects
(2|level_2) = region-level random effects

The process of vetting and validating models was accomplished primarily through an examination of ST-GPR scatterplots by GBD 2021 location from 1990 to 2021. Any poorly fitting datapoints were re-inspected for error at the level of extraction and survey implementation. If errors in data extraction were found, the study in question was re-extracted. In addition to SDI, a number of different potential fixed effects were considered, including lag-distributed income and urbanicity, but SDI proved to be the strongest predictor of unsafe sanitation in terms of magnitude of the coefficient. Uncertainty in the estimates was initially constructed based on standard deviation around each survey mean, then propagated through ST-GPR modelling by incorporating the variance of each datapoint in the Gaussian process regression step. A datapoint with high variance, for example, would contribute relatively less influence to the model than a datapoint with lower variance.

Once models are vetted, full time-series outputs from ST-GPR modelling are then rescaled using the above equations to form three mutually exclusive categories that sum up to 1 for each location-year combination. Table 2 provides the final result of this rescaling.

Table 2: Exposure categories and definitions

Category	Definition
Unimproved sanitation	Proportion of individuals that use unimproved sanitation facilities.
Improved sanitation	Proportion of individuals that use improved sanitation facilities, excluding sewer connection or septic tank.
Sanitation facilities with sewer connection or septic tank	Proportion of individuals that use toilet facilities with sewer connection or septic tank.

Theoretical minimum-risk exposure level

The theoretical minimum-risk exposure level for unsafe sanitation was defined as having access to a sanitation facility with sewer connection or septic tank.

Relative risks

For GBD 2021, unsafe sanitation was paired with one outcome, diarrhoeal diseases. Two meta-analyses, by Wolf et al 2014 and Wolf et al 2018, along with a literature review that used the same search terms as Wolf et al 2014, were used to identify relative risk studies.[2,3] Table 3 provides a summary of the relative risk data used.

Table 3: Relative risk input data

Input data	Relative risk
Source count (total)	16
Number of countries with data	13

In GBD 2021, relative risk values were calculated using a network meta-analysis approach with a tool called meta-egression—Bayesian, regularised, trimmed (MR-BRT). One study-level covariate – whether or not the study was generalisable to the general population – was included in the network meta-analysis. Several other covariates (whether exposure was captured at the individual level or population level; whether conformity with the study interventions was self-reported or confirmed by the researchers; whether or not the study was randomised; whether or not the study adjusted for all major known confounders; and what the study's follow-up percentage was) were considered but ultimately were not statistically significant and so were not used in analysis. No priors were used. We calculated the risk of developing diarrhoea for those using improved sanitation facilities and sewer or septic facilities, relative to the reference category of those using unimproved facilities. Those model results were then rescaled so that the relative risk for using sewer or septic facilities was the reference category, in order to match with our exposure definition. Table 4 shows the results of the MR-BRT analyses. Table 5 shows the relative risks that were ultimately used for modelling.

Table 4: MR-BRT network meta-analysis results (reference: unimproved sanitation)

Intervention	Relative risk (95% CI)
Improved sanitation	0.795 (0.739–0.856)
Sanitation facilities with sewer connection or septic tank	0.310 (0.274–0.352)

Table 5: Relative risks for each exposure category (reference: sewer or septic facilities)

Exposure category	Relative risk (95% CI)
Unimproved sanitation	3.22 (2.74–3.76)
Improved sanitation	2.57 (2.08–3.12)
Sanitation facilities with sewer connection or septic tank	1 (reference)

Figure 1 shows the results of the MR-BRT analysis in graphical form, along with the associated "risk-outcome scores" for each category, which is a measure of how good the evidence is for that particular relative risk estimate. Prior to generating an risk-outcome score, we conducted an additional post-analysis step to detect and flag publication bias in the input data. This approach is based on the classic Egger's regression strategy, which is applied to the residuals in our model. In the current implementation, we do not correct for publication bias, but flag the risk–outcome pairs where the risk for publication bias is significant.

We did not detect publication bias based on the association between observation residuals and their standard errors (p-value = 0.337, Egger mean = -0.102, Egger SD = 0.243). The overall risk-outcome score for unsafe sanitation is 0.518, which is the maximum of the individual category scores.

Figure 1: Risk-outcome scores

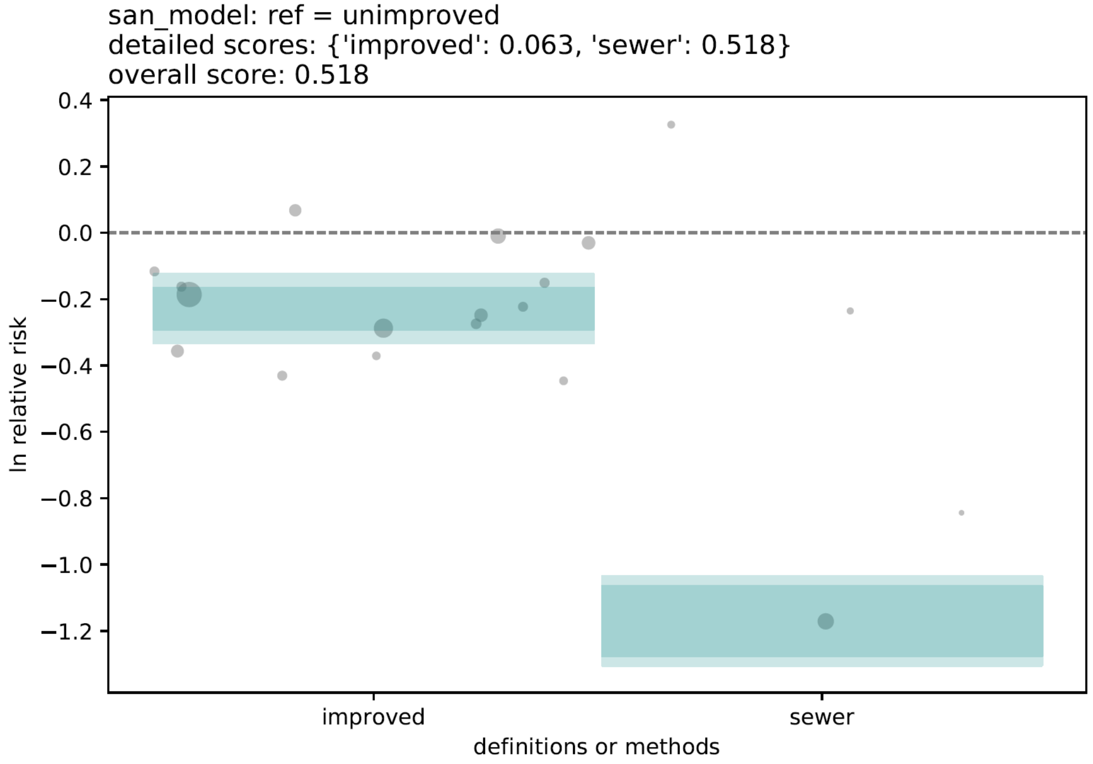

References

1. WHO/UNICEF Joint Monitoring Programme: Sanitation. https://washdata.org/monitoring/sanitation (accessed Oct 31, 2019).

2. Wolf J, Pruss-Ustun A, Cumming O, *et al.* Assessing the impact of drinking water and sanitation on diarrhoeal disease in low- and middle-income settings: systematic review and meta-regression. *Tropical Medicine and International Health* 2014; **19**: 928–42.
3. Wolf J, Hunter PR, Freeman MC, *et al.* Impact of drinking water, sanitation and handwashing with soap on childhood diarrhoeal disease: updated meta-analysis and meta-regression. *Tropical Medicine and International Health* 2018; **23**: 508–25.

Unsafe sex

Flowchart

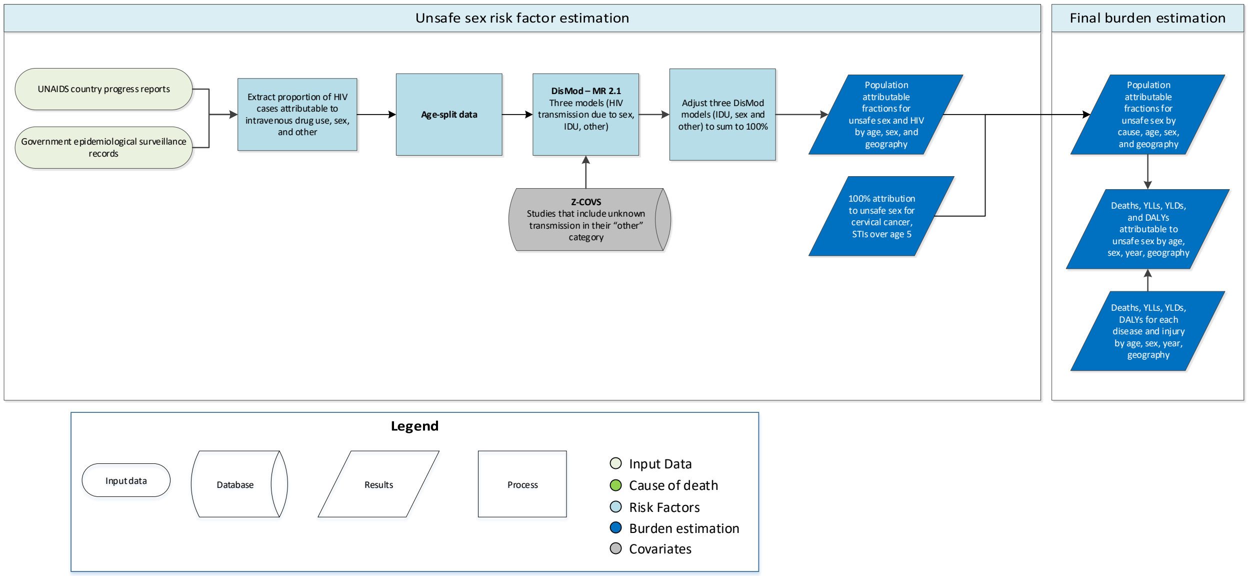

Input data and methodological summary

Definition

Exposure

Unsafe sex is defined as the risk of disease due to sexual transmission. The outcomes associated with unsafe sex that we estimate for the GBD study include HIV, cervical cancer, and all sexually transmitted diseases (STDs) except for those in neonates from vertical transmission, including HIV, *Opthalmia neonatorum*, and neonatal syphilis. We assumed 100% of cervical cancer and STDs were attributable to unsafe sex and modelled the proportion of HIV incidence occurring through sexual transmission to estimate the attributable burden for HIV due to unsafe sex. The theoretical minimum risk exposure level[2] (TMREL, described in Appendix for unsafe sex is defined as the absence of disease transmission due to sexual contact.

Input data

To be used in our models, sources must report HIV cases attributable to various modes of transmission. We screened UNAIDS country progress reports and searched government epidemiological surveillance records for these data. The primary data sources we used were UNAIDS, the European CDC, and the US CDC.

We excluded all extractions where the "other" category for HIV transmissions accounted for greater than 25% of all cases. We believe that such high proportions raise concerns about the quality of reporting.

Input data	Countries with data	New sources	Total sources
Exposures	97	0	948

Modelling strategy

We modelled the proportion of HIV cases attributable to unsafe sex. To do this we collected and cleaned data, ran three DisMod-MR 2.1[1] (Bayesian -regression, described in Appendix) models (HIV attributable to sex, HIV attributable to injection drug use, HIV attributable to other routes of transmission), adjusted results of the three DisMod-MR models to sum to , and then assigned the proportions as direct PAFs[2] (Fractions, described in Appendix.

No country-level covariates were included in the models. We tested an injection drug use (IDU) covariate – an opioid use covariate in the proportion HIV due to drug use model – but found no significant coefficients, so excluded them from the final model.

Since all-age and both-sex datapoints represent the vast majority of the available data, we derived an age-sex pattern for the HIV-IDU transmission model from the age-sex pattern present in the GBD 2017 PAF for hepatitis B attributable to IDU (the model for injecting drug use and hepatitis estimates the cumulative exposure to injecting drug use to capture all infections in people with a history of injecting even if in a more distant past). Assuming the proportion of HIV due to other transmission is constant over age and by sex, the age-sex pattern for the proportion of HIV due to sexual transmission was set to be the complement to 1 of the age-sex pattern for the proportion of HIV due to IDU. The all-age and both-sex data were split according to these age-sex patterns, and the three HIV transmission DisMod-MR models were run on the age- and sex-split data. In previous GBD rounds, only age-splitting had used this approach, while sex-splitting occurred within DisMod-MR. Since most data are for both sexes combined, using the sex ratio – in addition to the age pattern from the IDU-Hepatitis B PAF – is much more informative. The impact of this change resulted in general increases in proportion HIV due to sexual transmission among females, as they generally had lower IDU rates compared to males.

In GBD 2019, we also changed the proportion HIV due to sex DisMod-MR model to run in complement (1-proportion) space. Since proportions were high in most countries, modelling in complement space resulted in a better model fit. Additional priors were set to inform an age pattern: zero proportion HIV transmission due to IDU before age 15, zero proportion HIV transmission due to sex before age 10 (100 in complement space), and 100% transmission due to other before age 10. The results from these HIV transmission models were adjusted to sum to 100% for a given country-year-age-sex group at each of 1000 draws. This process was continued in GBD 2021.

Squeezed global HIV transmission model, Male

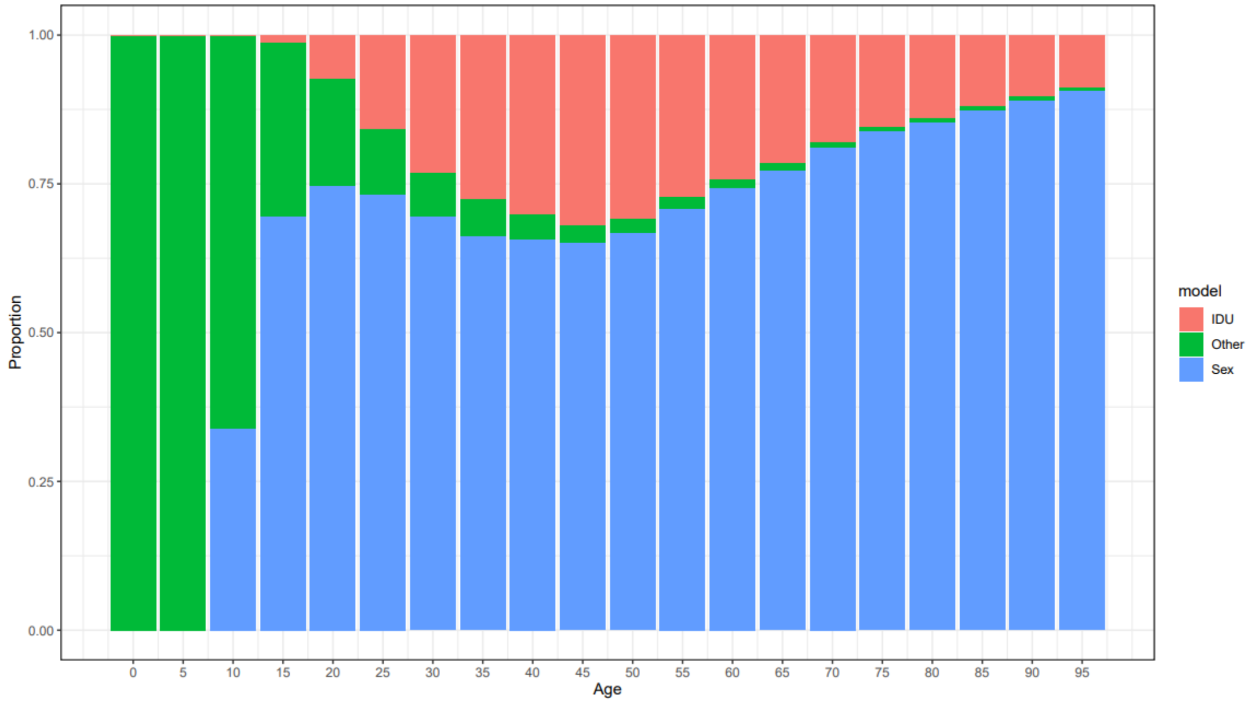

Squeezed global HIV transmission model, Female

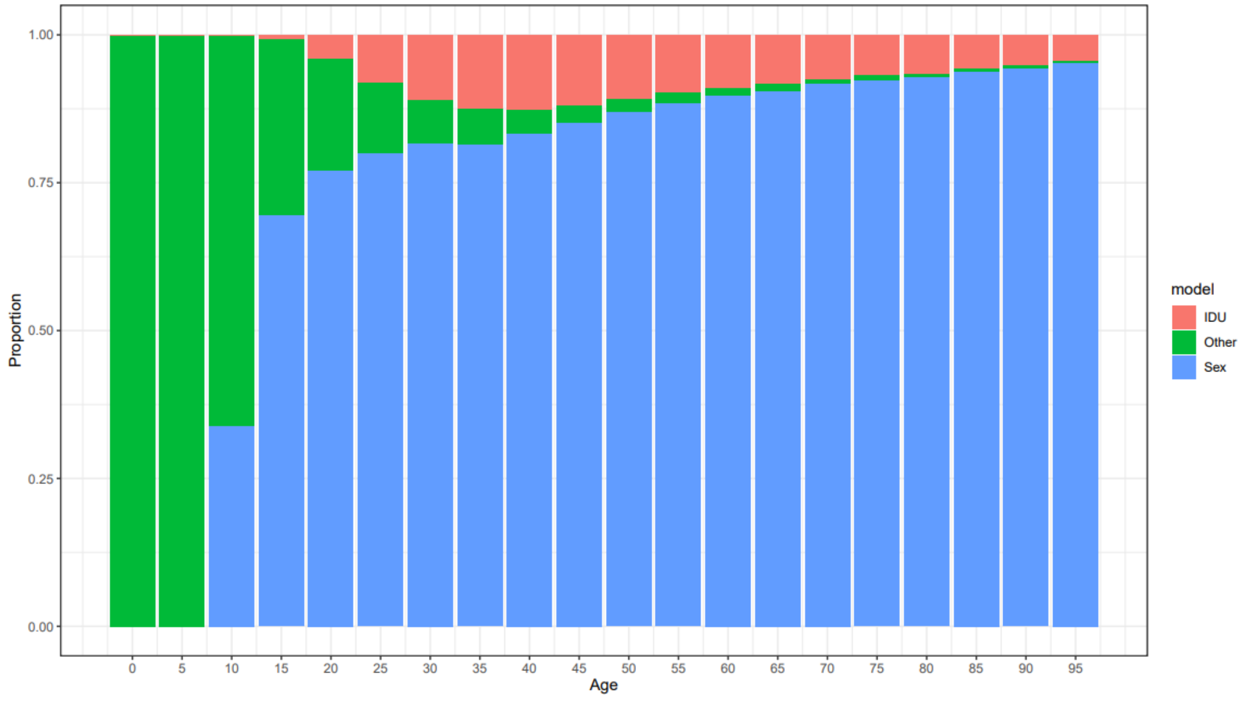

Theoretical minimum risk exposure level

The TMREL used for unsafe sex is the absence of disease transmission due to sexual contact.

Population attributable fraction calculations

Based on evidence in the literature, we attributed 100% of cervical cancer to unsafe sex. These sources state that HPV infection is necessary for cervical cancer to develop and that HPV is only spread through sexual contact. The proportion of STDs attributable to unsafe sex was also 100%.

For HIV, the results from the single parameter proportion DisMod-MR model for HIV transmission due to sex after squeezing were used directly as the population attributable fraction.

References

1. Vos T, Lim SS, Abbafati C, et al. Global burden of 369 diseases and injuries in 204 countries and territories, 1990–2019: a systematic analysis for the Global Burden of Disease Study 2019. The Lancet 2020; 396: 1204–22. doi: https://doi.org/10.1016/S0140-6736(20)30925-9
2. Murray CJL, Aravkin AY, Zheng P, *et al*. Global burden of 87 risk factors in 204 countries and territories, 1990–2019: a systematic analysis for the Global Burden of Disease Study 2019. *The Lancet* 2020; **396**: 1223–49. doi: https://doi.org/10.1016/S0140-6736(20)30752-2

Unsafe water

Flowchart

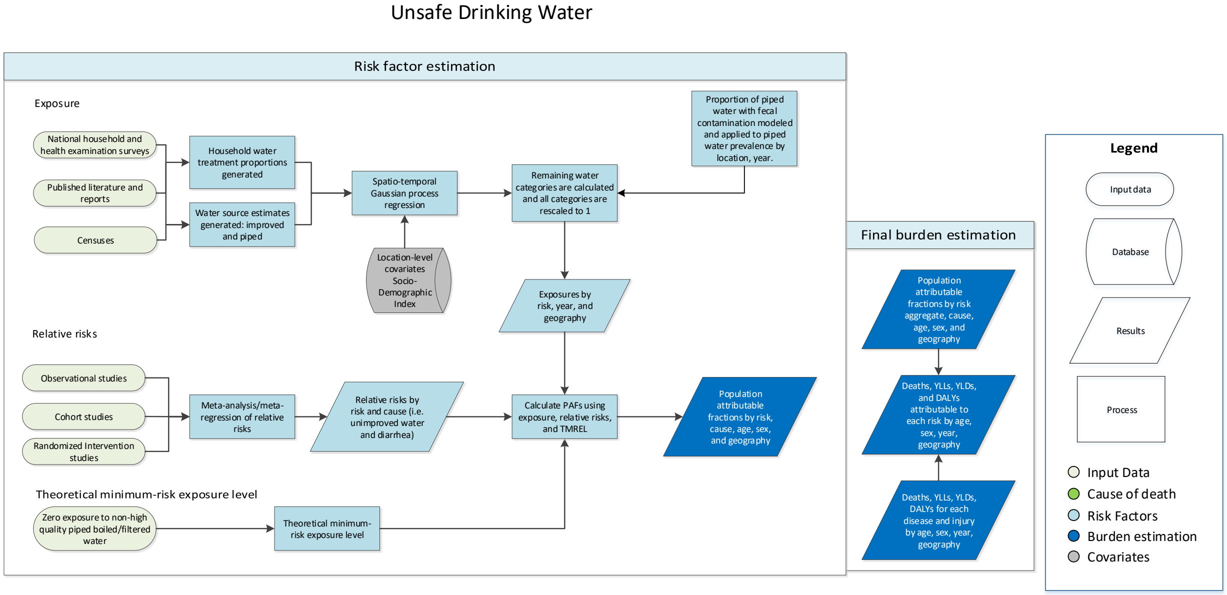

Input data and methodological summary

Exposure

Case definition

For the Global Burden of Diseases, Injuries, and Risk Factors Study (GBD) 2021, exposure to unsafe water was defined based on (1) reported primary water source used by the household, and (2) use of household water treatment (HWT) to improve the quality of drinking water before consumption. Water sources were defined based on the WHO/UNICEF Joint Monitoring Programme for Water Supply, Sanitation and Hygiene (JMP).[1] Examples of "improved" sources include boreholes, tube wells, protected wells, and packaged or delivered water. Piped water is also considered "improved" by the JMP but is placed into its own category for GBD purposes. Examples of "unimproved" sources include unprotected springs, unprotected wells, and surface water. Additionally, four different HWTs were determined to be effective point-of-use treatments based on effect sizes calculated from a network meta-analysis: solar treatment, chlorine treatment, boiling, and filtering. For modelling purposes, we grouped solar and chlorine treatment together, as well as boiling and filtering.

Input data

The search for usable data sources was conducted using the Global Health Data Exchange (GHDx) database. Water source input data came primarily from nationally representative surveys, such as the Demographic and Health Survey (DHS), the Multiple Indicator Cluster Surveys (MICS), the World Health Survey (WHS), and the DHS AIDS Indicator Survey (AIS). HWT input data were largely limited to the DHS and MICS due to data availability. Surveys that reported results at the household level were converted to the individual level using household size data to ensure that our models estimated the proportion of individuals, rather than households, exposed to a given indicator. For GBD 2021, we re-extracted nearly all of our sources from 2000 to present in an effort to standardise extraction outputs and fix past extraction errors. Additionally, we added 64 new sources for this cycle. After extraction, surveys and

censuses were then tabulated to the water source and water treatment categories of interest for each location. Table 1 provides a summary of the exposure input data.

Table 1: Exposure input data

Input data	Exposure
Source count (total)	1221
Number of countries with data	170

Modelling strategy

Water source data were modelled using an ordinal framework, with two distinct models: (1) proportion of the total population that uses piped water sources, and (2) proportion of the non-piped population that uses improved water sources. Both models were estimated for all ages and both sexes combined, and produced results for each unique location-year combination. This ordinal framework allowed estimating the category with the most data (piped water prevalence) and leveraging that estimate to anchor the estimates for the improved and unimproved water categories. The results of the improved proportion model were multiplied by 1 minus the piped water prevalence to calculate improved water prevalence. The sum of improved and piped water prevalence was then subtracted from 1 to yield unimproved water prevalence.

$$\boldsymbol{Piped} = \frac{\#\ persons\ using\ piped\ water}{\#\ persons\ with\ nonmissing\ response}$$

$$\boldsymbol{Improved} = \left(\frac{\#\ persons\ using\ improved\ water}{\#\ persons\ without\ piped\ water}\right) * (1 - \boldsymbol{Piped})$$

$$\boldsymbol{Unimproved} = 1 - (\boldsymbol{Piped} + \boldsymbol{Improved})$$

HWT categories were estimated in a similar ordinal framework. Its two models were (1) proportion of the total population that does not use any water treatment methods, and (2) proportion of the population that treat their water that use boiling or filtering as their primary HWT. Like the water source models, both HWT models were estimated for all ages and both sexes combined and produced results for each unique location-year combination. The proportion of individuals who boil/filter drinking water was calculated by multiplying the proportion who boil/filter modelled previously multiplied by the prevalence of any water treatment (estimated by subtracting the prevalence of no treatment from 1). The proportion of individuals who treat their water using solar/chlorine methods was estimated by subtracting from 1 the sum of prevalence of no treatment estimates and prevalence of filter/boil treatment.

$$\boldsymbol{No\ HWT} = \frac{\#\ persons\ who\ do\ not\ treat\ water}{\#\ persons\ with\ nonmissing\ response}$$

$$\boldsymbol{Boil\ or\ filter} = \left(\frac{\#\ persons\ who\ treat\ water\ with\ boil\ or\ filter}{\#\ persons\ who\ treat\ water\ with\ any\ method}\right) * (1 - \boldsymbol{No\ HWT})$$

$$\boldsymbol{Chlorine\ or\ solar} = 1 - (\boldsymbol{No\ HWT} + \boldsymbol{Boil\ or\ filter})$$

Additionally, we modelled the microbiological quality of piped water sources primarily using data from a review by Bain et al 2014[2] that measured the proportion of piped water sources contaminated with

faecal matter. We used the results from this model to split the prevalence of piped water into basic piped water and high-quality piped water by location and year. High-quality piped water is piped water that enters the household free of contamination. Thus, HWT is irrelevant for this category, since treatment is only necessary if the water is contaminated.

$$\boldsymbol{Faecal\ contamination} = \frac{\#\ contaminated\ piped\ water\ systems}{\#\ piped\ water\ systems\ sampled}$$

$$\boldsymbol{Basic\ piped = Piped * Faecal\ contamination}$$

$$\boldsymbol{HQ\ piped = Piped - Basic\ piped}$$

Each of the models described above was modelled using a three-step modelling scheme of mixed-effect linear regression followed by spatiotemporal Gaussian process regression (ST-GPR), which produced full time-series estimates for each GBD 2021 location. Socio-demographic Index (SDI), a composite measure of development combining education per capita, income per capita, and fertility, was set as a fixed effect in the linear regression since it proved to be a significant predictor. The proportion of individuals with access to piped water was also used as a covariate in the faecal matter model. Random effects were set at GBD 2021 region and super-region levels to fit the models but were not used in the predictions. The linear regression equations for each of the five ST-GPR models used for this risk factor are listed below.

Proportion using piped water: $logit(data) \sim SDI + (1|level\_1) + (1|level\_2)$

Proportion of non-piped population using improved water: $logit(data) \sim SDI + (1|level\_1) + (1|level\_2)$

Proportion using no HWT: $logit(data) \sim SDI + (1|level\_1) + (1|level\_2)$

Proportion of HWT-using population that boils/filters: $logit(data) \sim SDI + (1|level\_1) + (1|level\_2)$

Proportion of piped water systems contaminated with faecal matter: $logit(data) \sim SDI + piped\ water\ access + (1|level\_1) + (1|level\_2)$

Piped water access = proportion of individuals with access to piped water
(1|level_1) = super-region-level random effects
(2|level_2) = region-level random effects

The process of vetting and validating models was accomplished primarily through an examination of ST-GPR scatter plots by GBD 2021 location from 1990 to 2021. Any poorly fitting datapoints were re-inspected for error at the level of extraction and survey implementation. If errors in data extraction were found, the study in question was re-extracted. In addition to SDI, a number of different potential fixed effects were considered, including lag-distributed income and urbanicity, but SDI proved to be the strongest predictor of the unsafe water categories. Uncertainty in the estimates was initially formed based on standard deviation by survey, then propagated through ST-GPR modelling by means of confidence intervals around each datapoint that reflected the point-estimate-specific variance. A datapoint with high variance, for example, would contribute relatively less influence to the model than a datapoint with lower variance.

Once models were vetted, full time-series outputs from ST-GPR modelling were then rescaled to form ten mutually exclusive categories that summed to 1 for each location-year combination. Table 2 provides the final result of this rescaling and also includes the formulas for each category.

Table 2: Exposure categories, definitions, and formulas

Exposure category	Definition	Formula
Unimproved, no HWT	Proportion of individuals who primarily use unimproved source and *do not* use any HWT to purify their drinking water.	$[1-(Piped+Improved)]*\left[\frac{\#\ persons\ who\ do\ not\ treat\ water}{\#\ persons\ with\ nonmissing\ response}\right]$
Unimproved, chlorine/solar	Proportion of individuals who primarily use unimproved source, and who use solar or chlorine treatment to purify their drinking water.	$[1-(Piped+Improved)]*[1-(No\ HWT+Boil\ or\ filter)]$
Unimproved, boil/filter	Proportion of individuals who primarily use unimproved source and who boil or filter to purify their drinking water.	$[1-(Piped+Improved)]*\left[\left(\frac{\#\ persons\ who\ treat\ water\ with\ boil\ or\ filter}{\#\ persons\ who\ treat\ water\ with\ any\ method}\right)*(1-No\ HWT)\right]$
Improved water except piped, no HWT	Proportion of individuals who primarily use improved sources other than piped water supply and *do not* use any HWT to purify their drinking water.	$\left[\left(\frac{\#\ persons\ using\ improved\ water}{\#\ persons\ without\ piped\ water}\right)\right]*\left[\frac{\#\ persons\ who\ do\ not\ treat\ water}{\#\ persons\ with\ nonmissing\ response}\right]$
Improved water except piped, chlorine/solar	Proportion of individuals who primarily use improved sources other than piped water supply, and who use solar or chlorine treatment to purify their drinking water.	$\left[\left(\frac{\#\ persons\ using\ improved\ water}{\#\ persons\ without\ piped\ water}\right)\right]*[1-(No\ HWT+Boil\ or\ filter)]$
Improved water except piped, boil/filter	Proportion of individuals who primarily use improved sources other than piped water supply and who boil/filter their drinking water.	$\left[\left(\frac{\#\ persons\ using\ improved\ water}{\#\ persons\ without\ piped\ water}\right)\right]*\left[\left(\frac{\#\ persons\ who\ treat\ water\ with\ boil\ or\ filter}{\#\ persons\ who\ treat\ water\ with\ any\ method}\right)*(1-No\ HWT)\right]$
Basic piped water, no HWT	Proportion of individuals who primarily use basic piped water supply and *do not* use any HWT to purify their drinking water.	$\left[\left[\frac{\#\ persons\ using\ piped\ water}{\#\ persons\ with\ nonmissing\ response}\right]*\left[\frac{\#\ contaminated\ piped\ water\ systems}{\#\ piped\ water\ systems\ sampled}\right]\right]*\left[\frac{\#\ persons\ who\ do\ not\ treat\ water}{\#\ persons\ with\ nonmissing\ response}\right]$
Basic piped water, chlorine/solar	Proportion of individuals who primarily use basic piped water supply, and who *use* solar or chlorine water treatment, to purify their drinking water.	$\left[\left[\frac{\#\ persons\ using\ piped\ water}{\#\ persons\ with\ nonmissing\ response}\right]*\left[\frac{\#\ contaminated\ piped\ water\ systems}{\#\ piped\ water\ systems\ sampled}\right]\right]*[1-(No\ HWT+Boil\ or\ filter)]$
Basic piped water, boil/filter	Proportion of individuals who primarily use basic piped water supply and who boil or filter to purify their drinking water.	$\left[\left[\frac{\#\ persons\ using\ piped\ water}{\#\ persons\ with\ nonmissing\ response}\right]*\left[\frac{\#\ contaminated\ piped\ water\ systems}{\#\ piped\ water\ systems\ sampled}\right]\right]*\left[\left(\frac{\#\ persons\ who\ treat\ water\ with\ boil\ or\ filter}{\#\ persons\ who\ treat\ water\ with\ any\ method}\right)*(1-No\ HWT)\right]$

High-quality piped water	Proportion of individuals who primarily use high-quality piped water.	$\left[\frac{\#\ persons\ using\ piped\ water}{\#\ persons\ with\ nonmissing\ response}\right] - \left[\left[\frac{\#\ persons\ using\ piped\ water}{\#\ persons\ with\ nonmissing\ response}\right] * \left[\frac{\#\ contaminated\ piped\ water\ systems}{\#\ piped\ water\ systems\ sampled}\right]\right]$

Theoretical minimum-risk exposure level

The theoretical minimum-risk exposure level for unsafe water is defined as having access to high-quality piped water.

Relative risks

Input data

For GBD 2021, unsafe water was paired with one outcome – diarrhoeal diseases – given evidence provided by relative risk studies. Input data included in the GBD 2021 unsafe water relative risk analysis are as follows:

Table 3: Relative risk input data

Input data	Relative risk
Source count (total)	73
Number of countries with data	36

Two meta-analyses (Wolf et al 2014 and Wolf et al 2018) were used to identify relative risk studies, including years 1970-2016.[3,4] Additionally, a literature review on the relationship between high-quality piped water and diarrhoea was conducted for GBD 2021, which yielded three new studies (Figure 1). We searched PubMed for relevant literature published from January 1, 1970 to July 2, 2020 (date of search), using the search string below:

("Drinking Water"[Mesh] OR "Water Quality"[Mesh] OR "Water Supply"[Mesh] OR "Piped water"[TIAB] OR "Tap water"[TIAB] OR "Potable water"[TIAB]) AND ("Diarrhea"[Mesh] OR Diarrh*[TIAB] OR "Diarrhea incidence"[TIAB] OR "Bacteriological"[TIAB] OR "Microbial water quality"[TIAB]) AND (1970[PDAT] : 3000[PDAT) NOT (animals[MeSH] NOT humans[MeSH])

Figure 1: PRISMA diagram for systematic review of high-quality piped water and diarrheal

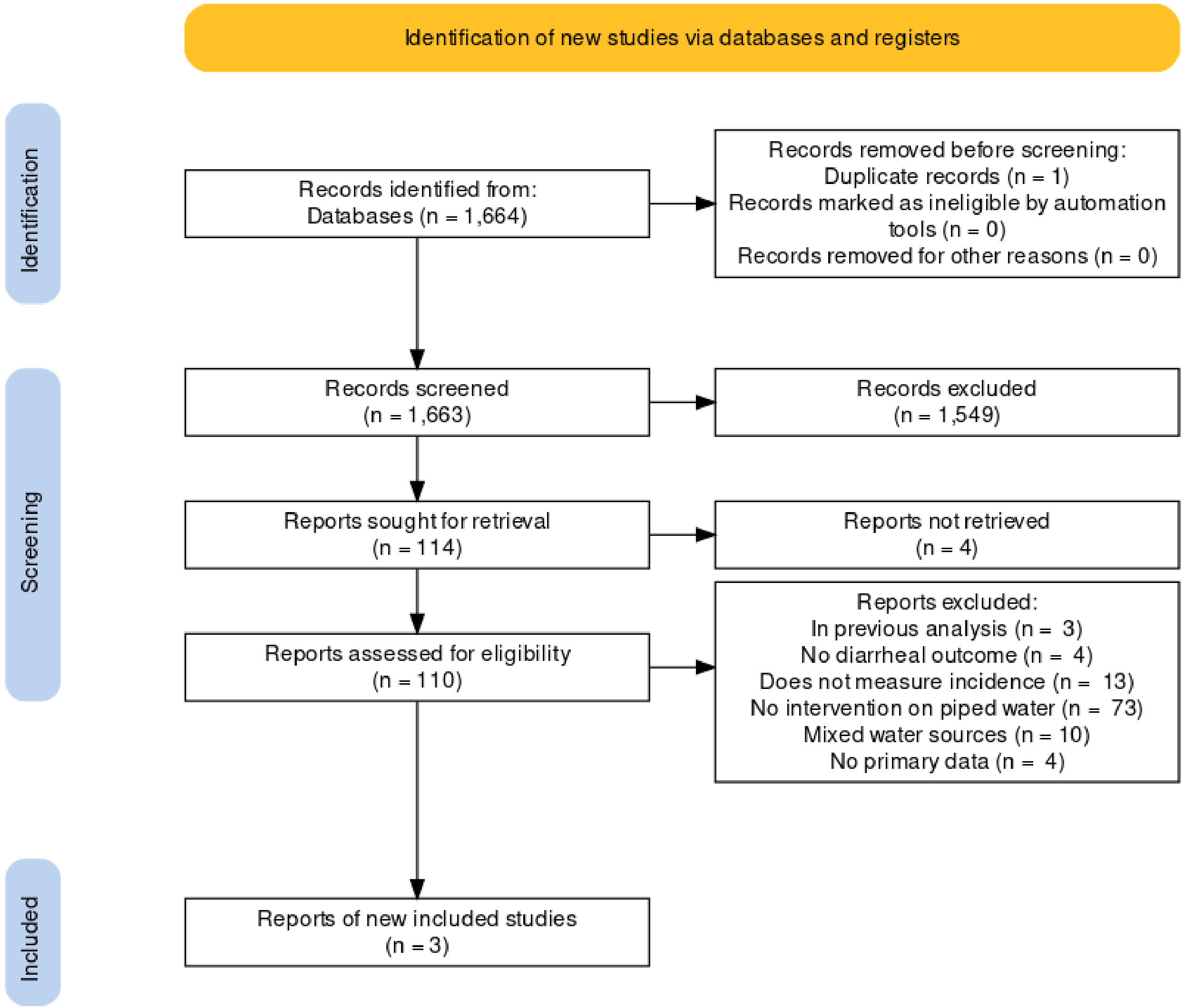

disease

Modelling strategy

In GBD 2021, relative risk values were calculated using a network meta-analysis approach with a tool called meta-regression—Bayesian, regularised, trimmed (MR-BRT). One study-level covariate – whether conformity with the study interventions was self-reported or confirmed by the researchers – was included in the network meta-analysis. Several other covariates (whether exposure was captured at the individual level or population level; whether or not the study was randomised; whether or not the study adjusted for all major known confounders; and what the study's follow-up percentage was) were considered but ultimately were not statistically significant and so were not included in the analysis. No priors were used. The risk of developing diarrhoea relative to using an unimproved water source was calculated for each of the following categories: boil or filter, solar or chlorine, improved, piped, and high-quality piped (Table 4). These model results were then combined and rescaled to match with our exposure definitions (Table 5). The combined effects of source interventions (ie, improved, piped, high-quality piped) and point-of-use interventions (ie, boil/filter, solar/chlorine) were assumed to be

multiplicative. Additionally, we assumed that the lowest possible risk level is using the best source type (high-quality piped water) combined with the best point-of-use treatment (boil/filter).

Table 4: MR-BRT network meta-analysis results (reference: unimproved water source)

Intervention	Relative risk (95% UI)
Boil/filter water treatment	0.57 (0.37–0.87)
Chlorine/solar water treatment	0.77 (0.51–1.15)
Improved water source	0.84 (0.54–1.28)
Piped water source	0.67 (0.44–1.03)
High-quality piped water source	0.29 (0.12–0.70)

Table 5: Relative risks for each exposure category (reference: high-quality piped water)

Exposure category	Relative risk (95% UI)
Unimproved, no HWT	6.93 (2.20–17.54)
Unimproved, chlorine/solar	5.41 (1.59–14.02)
Unimproved, boil/filter	3.87 (1.42–8.66)
Improved water except piped, no HWT	5.97 (1.77–16.16)
Improved water except piped, chlorine/solar	4.67 (1.25–13.20)
Improved water except piped, boil/filter	3.33 (1.13–8.20)
Basic piped water, no HWT	4.73 (1.33–12.59)
Basic piped water, chlorine/solar	3.69 (1.01–10.14)
Basic piped water, boil/filter	2.64 (0.86–6.34)
High-quality piped water	1 (reference)

Figure 2 shows the results of the MR-BRT analysis in graphical form, along with the associated "risk-outcome scores" for each category, which is a measure of how good the evidence is for that particular relative risk estimate. Prior to generating an risk-outcome score, we conducted an additional post-analysis step to detect and flag publication bias in the input data. This approach is based on the classic Egger's regression strategy, which is applied to the residuals in our model. In the current implementation, we do not correct for publication bias, but flag the risk–outcome pairs where the risk for publication bias is significant.

We did not detect publication bias based on the association between observation residuals and their standard errors (p-value = 0.195, Egger mean = –0.101, Egger SD = 0.118). The overall risk-outcome score for this risk factor is 0.231, which is the maximum of the individual category scores.

Figure 2: Risk-outcome scores

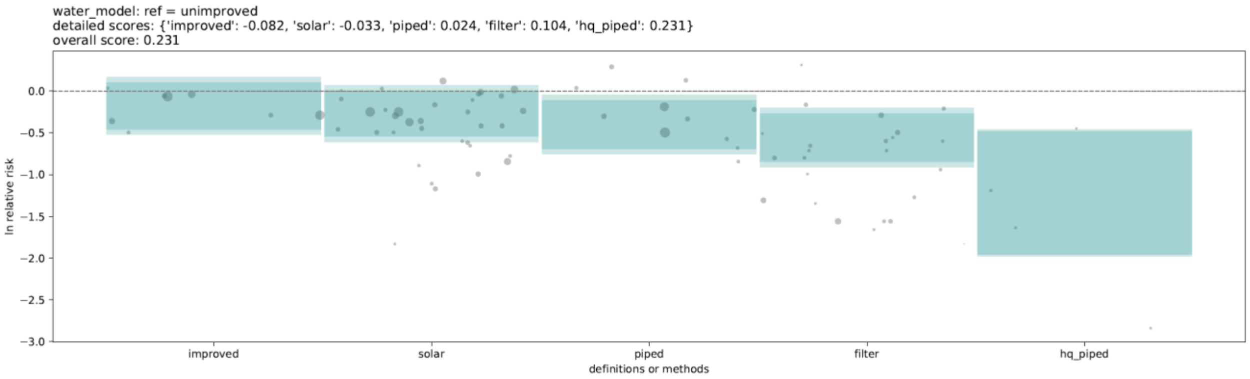

References

1. WHO/UNICEF Joint Monitoring Programme: Drinking water. https://washdata.org/monitoring/drinking-water (accessed Oct 31, 2019).
2. Bain R, Cronk R, Wright J, Yang H, Slaymaker T, Bartram J. Fecal Contamination of Drinking-Water in Low- and Middle-Income Countries: A Systematic Review and Meta-Analysis. *PLOS Medicine* 2014; **11**: e1001644.
3. Wolf J, Pruss-Ustun A, Cumming O, *et al.* Assessing the impact of drinking water and sanitation on diarrhoeal disease in low- and middle-income settings: systematic review and meta-regression. *Tropical Medicine and International Health* 2014; **19**: 928–42.
4. Wolf J, Hunter PR, Freeman MC, *et al.* Impact of drinking water, sanitation and handwashing with soap on childhood diarrhoeal disease: updated meta-analysis and meta-regression. *Tropical Medicine and International Health* 2018; **23**: 508–25.

Vitamin A deficiency

Flowchart

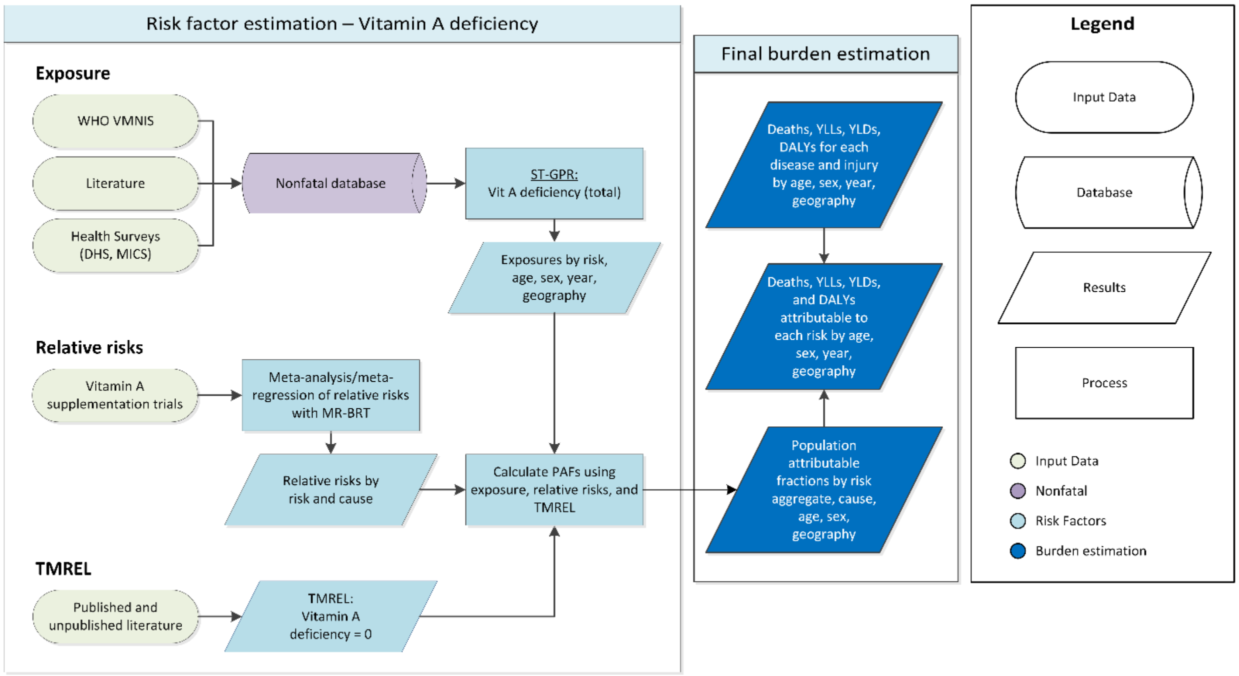

Input data and methodological summary

Definition

Exposure

Vitamin A deficiency is a condition due to low dietary intake or bioavailability of vitamin A that is inadequate to satisfy physiological needs, which is characterized by low serum or/and breast milk retinol or /and retinol binding concentration, or /and clinical symptoms such as night blindness, xerophthalmia. For GBD 2021, vitamin A deficiency is defined as serum retinol <70 μmol/L. We examined vitamin A deficiency as a risk factor in children aged 6 months to 5 years.

Input data

For GBD 2021, we used data from the WHO Vitamin and Mineral Nutrition Information System, health surveys such as DHS and MICS, and studies identified through literature review for the vitamin A deficiency model. Tables 1 and 2 provide a summary of data inputs for vitamin A deficiency risk factor modelling**.** A systematic review was last conducted for GBD 2013. The PubMed search terms were: ((vitamin A deficiency[Title/Abstract] AND prevalence[Title/Abstract]) AND (“2009”[Date – Publication] : “2013”[Date – Publication])). Exclusion criteria were:

1. Studies that were not population-based, eg, hospital or clinic-based studies
2. Studies that did not provide primary data on epidemiological parameters, eg, commentaries
3. Review articles
4. Case series
5. Self-reported cases

Table 1: Data inputs for exposure for vitamin A deficiency

Input data	Exposure
Source count	274
Number of countries with data	96

Table 2: Data inputs for relative risks for vitamin A deficiency

Input data	Relative risk
Source count (total)	19
Number of countries with data	10

Modelling strategy

Exposure

No major changes to the modelling strategy for vitamin A deficiency were made in GBD 2021 as compared to GBD 2019. However, the covariates used in the vitamin A deficiency model were updated in 2021. Specifically, the vitamin A deficiency model now uses logit SDI instead of SDI, and no longer includes vitamin A supplementation coverage as a covariate.

We estimated the age- and sex-specific prevalence of vitamin A deficiency (serum retinol <0.7 µmol/L). Firstly, we sex- and age-split the data using a sex ratio model (fit in meta-regression—Bayesian, regularised, trimmed [MR-BRT]) and an age pattern model (fit in DisMod). In GBD 2021, the vitamin A ST-GPR model utilized three location-level covariates: age-specific stunting SEV, Socio-demographic Index (logit scale), and the availability of retinol activity equivalent (RAE) units in foods. Vitamin A supplementation was omitted as a covariate in the vitamin A deficiency model due to its lack of statistical significance in the ST-GPR model. We also observed that when the coverage of vitamin A supplementation was included as a covariate in the vitamin A deficiency STGPR model, it resulted in an implausible temporal trend. Since GBD 2019, we have introduced the assumption that the duration of vitamin A deficiency is one year, which implies that prevalence and incidence are equal.

Theoretical minimum risk exposure level

The theoretical minimum risk exposure is that the prevalence of vitamin A deficiency is zero.

Relative risk

The relative risk data were updated in GBD 2017 to reflect studies included in the most recently

published systematic review by Imdad and colleagues.[1] In GBD 2019, we revisited the underlying studies reported in this analysis and re-analysed and evaluated the evidence on vitamin A deficiency as a risk factor for diarrhoea, measles, and lower respiratory infections using MR-BRT. Lower respiratory infections were removed as an outcome due to insufficient evidence, and the relative risks for diarrhoea and measles were updated. Additionally, in GBD 2019, we found no significant relationship between background vitamin A deficiency prevalence and the magnitude of the relative risk. Thus, starting in GBD 2019, we no longer adjust relative risks for background vitamin A deficiency prevalence.

For GBD 2021, we updated the relative risks with MR-BRT using new model specifications and the most-up-to-date epidemiological evidence. In both GBD 2019 and GBD 2021, inclusion and exclusion of a risk-outcome pair was assessed on the risk curve without between-study-heterogeneity. However, we used the relative risk from the models with between-study-heterogeneity for the population attributable fraction (PAF) calculation in GBD 2021, while in GBD 2019 we used the relative risk that did not incorporate between-study-heterogeneity.

All input data for the relative risk model came from randomized controlled supplementation trials. Bias covariates were extracted for representativeness, exposure measurement, outcome measurement, blinding, confounder adjustment, study design variation, reverse causation, selection bias, and incidence. Most bias covariates did not have variation amongst studies, and only incidence, which indicates if the RR estimate was for cause-specific mortality or incidence, was used in the analysis.

Table 3: Relative risks for risk-outcome pairs.

Cause	GBD 2017 relative risk	GBD 2019 relative risk	GBD 2021 relative risk
Diarrhoea	2.35 (2.17–2.54)	1.14 (1.03–1.26)	1.25 (0.62–2.25)
Measles	2.76 (2.01–3.78)	1.39 (1.03–1.90)	1.510.64–3.04)

Citations

1. Imdad A, Ahmed Z, Bhutta ZA. Vitamin A supplementation for the prevention of morbidity and mortality in infants one to six months of age. *Cochrane Database of Systematic Reviews* 2016; Sep 28; 9. Art. No: CD007480.

Zinc deficiency

Flowchart

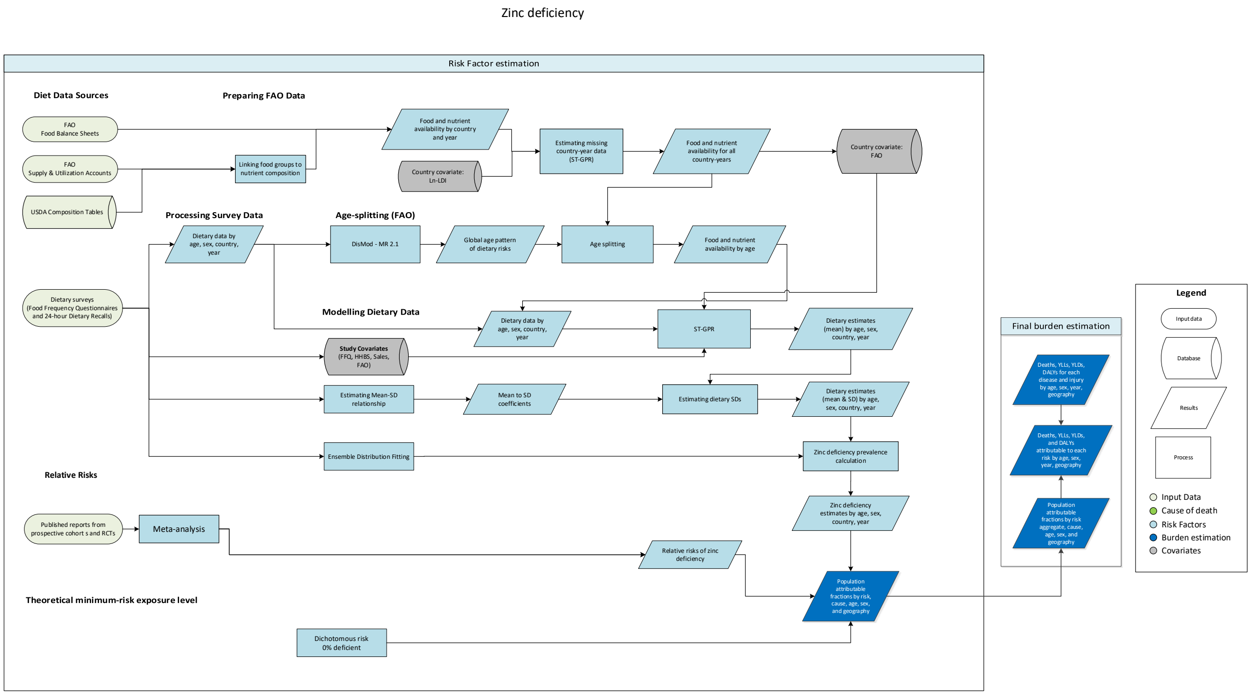

Input data and methodological summary

Definition

Exposure

Exposure to zinc deficiency is defined as consumption of less than 2-3 milligrams of zinc per day among children between the ages of 1 and 4 years old.

Input data

Exposure

We used dietary data from nationally and subnationally representative nutrition surveys, food frequency questionnaires, and United Nations FAO Supply and Utilization Accounts to estimate the mean intake of zinc at the population level.

Table 1: Data inputs for exposure for zinc deficiency.

Input data	Exposure
Source count (total)	28
Number of countries with data	175

Relative risk

Table 2: Data inputs for relative risks for zinc deficiency.

Input data	Relative risk
Source count (total)	39

In GBD 2021, a new systematic review was conducted on the effect of zinc supplementation on diarrhoea. In the review, only orally administered zinc supplementation trials were included. Sources were excluded if study designs were not randomised controlled trials (i.e., observational, cross-sectional, commentary pieces), included populations with disease of no interest (i.e., children less than 6 months of age), had no outcome of interest, or components for analysis were not available (i.e., relative risk not measured). Also, dietary zinc and zinc-fortified foods were excluded from the analysis. Details of the systematic review are provided below in Figure 1.

Search strings: "(zinc [tiab] OR Zn [tiab]) AND (supplement* [tiab]) AND (infant [mh] OR child [mh] OR adolescent [mh] OR pediatric [tiab]) AND (randomized controlled trial [pt] OR controlled clinical trial [pt] OR randomized [tiab] OR placebo [tiab] OR clinical trials as topic [mesh: noexp] OR randomly [tiab] OR trial [ti]) NOT (animals [mh] NOT humans [mh])" from all past sources to 12/02/19.

Figure 1: PRISMA 2021 flow diagram for systematic review of zinc deficiency and risk of diarrhoea

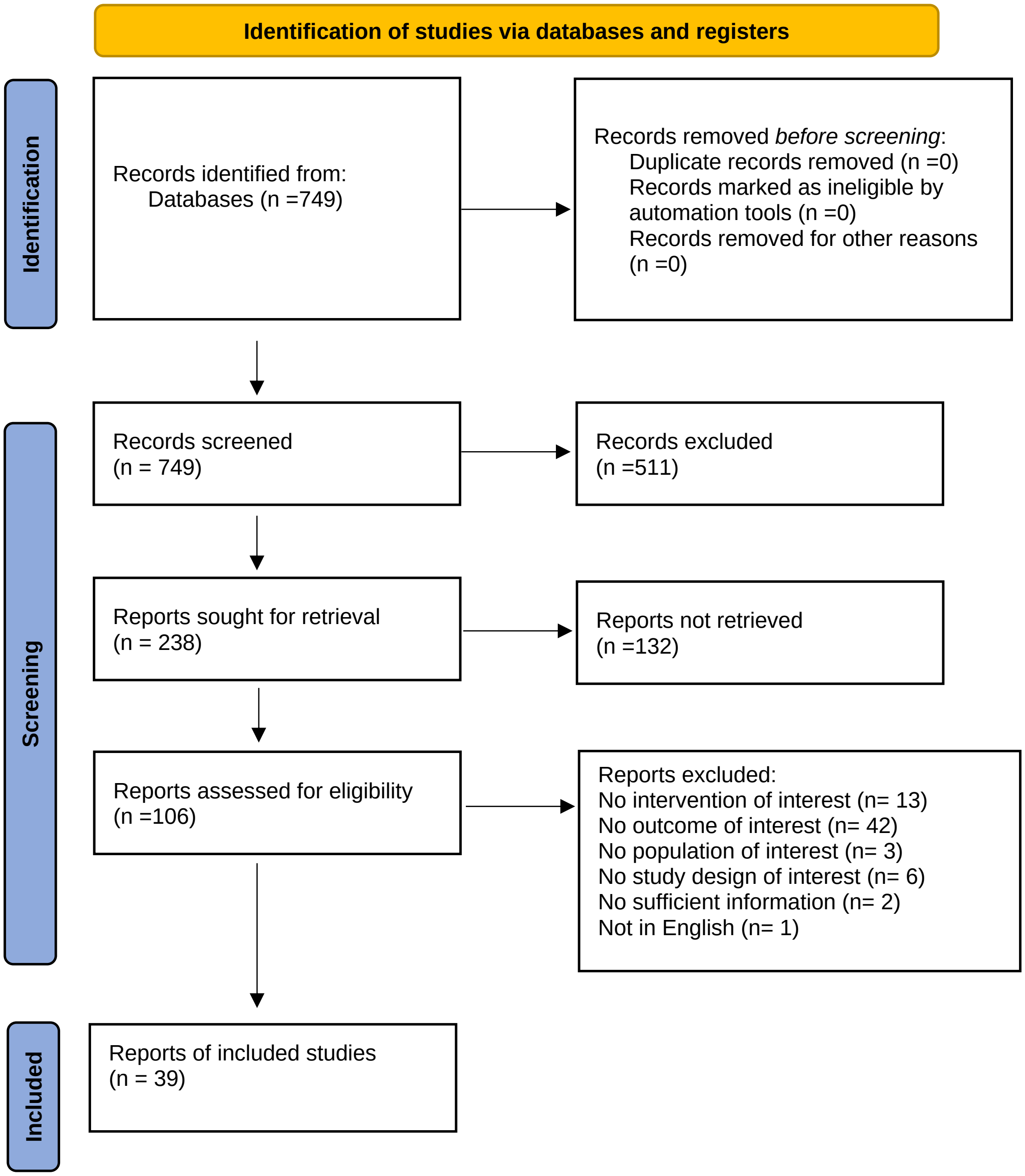

Data processing

As with the rest of the dietary risks, the gold-standard data source are 24-hour dietary recall surveys, and we perform a bias adjustment to crosswalk alternate definitions to dietary recall. In GBD 2019, we updated the crosswalk regression to utilise meta-regression – Bayesian, regularised, trimmed (MR-BRT) and in GBD 2021 we used the same adjustment factors.

Table 3: MR-BRT crosswalk adjustment factors for zinc deficiency

Sex	Data input	Reference or alternative case definition	Gamma	Beta coefficient, log (95% CI)	Adjustment factor*
---	DR	Ref	0.41	---	---
Male	FAO	Alt		0.58 (-0.26,1.42)	0.56 (0.77,4.15)
Female	FAO	Alt		0.57 (-0.26,1.41)	0.56 (0.77,4.11)
Male	FFQ	Alt		0.64 (-0.3,1.51)	0.53 (0.74,4.53)
Female	FFQ	Alt		0.63 (-0.32,1.51)	0.53 (0.73,4.52)

***Adjustment factor is the transformed beta coefficient in normal space and can be interpreted as the factor by which the alternative case definition is adjusted to reflect what it would have been if measured as the reference.**

Modelling strategy

Exposure model

In GBD 2021, there were no updates made to the modelling strategy for zinc deficiency. We first used a spatiotemporal Gaussian process regression (ST-GPR) framework to estimate the mean intake of zinc by age, sex, country, and year. To assist with estimation for locations and years without data, we used the lag-distributed income and energy availability (kcal) of that location-year as a covariate. Using the method described in the dietary risks section, we characterised the distribution of zinc intake for children between ages of 1 and 4 years old and then integrated to determine the proportion of the children with intake of less than 2-3 milligrams of zinc per day[1].

Theoretical minimum-risk exposure level

The theoretical minimum-risk exposure is that the prevalence of zinc deficiency is zero.

Relative risk

In GBD 2019, we revisited the most recent meta-analysis evaluating the effects of zinc supplementation on disease endpoints. Specifically, we re-analysed and evaluated the evidence of zinc deficiency as a risk factor for diarrhoea and lower respiratory infections (GBD 2017 outcomes) using MR-BRT. Lower respiratory infections were removed as an outcome due to insufficient evidence, and the relative risks for diarrhoea were updated. Additionally, in GBD 2019 we found no significant relationship between background zinc deficiency prevalence and the magnitude of the relative risk. Thus, starting in GBD 2019, the relative risks were no longer adjusted for background zinc deficiency prevalence. Between GBD 2019 and GBD 2021, the main updates in the MR-BRT approach were the inclusion of new data from the systematic review we conducted and the inclusion of gamma in the uncertainty.

All input data for the relative risk model came from randomized controlled supplementation trials. Bias covariates were extracted for representativeness, exposure measurement, outcome measurement, blinding, confounder adjustment, study design variation, reverse causation, selection bias, and incidence. Most bias covariates did not have variation amongst studies, and only incidence, which indicates if the RR estimate was for cause-specific mortality or incidence, was used in the analysis.

Table 4: Relative risks for risk-outcome pairs.

Cause	GBD 2017 RR<br>Mortality<br>incidence	GBD 2019 RR<br>Mortality + incidence	GBD 2021 RR<br>Mortality + incidence
Diarrhoea	1.95 (0.91–3.91)<br>1.90 (1.52–2.33)	1.14 (1.07–1.21)	1.18 (0.72–1.84)

References

1 International Zinc Nutrition Consultative Group (IZiNCG), Brown KH, Rivera JA, *et al.* International Zinc Nutrition Consultative Group (IZiNCG) technical document #1. Assessment of the risk of zinc deficiency in populations and options for its control. *Food Nutr Bull* 2004; **25**: S99-203.

Table S1. GBD risk hierarchy with levels	
**Risk**	**level**
All risk factors	0
Environmental/occupational risks	1
Unsafe water, sanitation, and handwashing	2
Unsafe water source	3
Unsafe sanitation	3
No access to handwashing facility	3
Air pollution	2
Particulate matter pollution	3
Ambient particulate matter pollution	4
Household air pollution from solid fuels	4
Ambient ozone pollution	3
Nitrogen dioxide pollution	3
Non-optimal temperature	2
High temperature	3
Low temperature	3
Other environmental risks	2
Residential radon	3
Lead exposure	3
Occupational risks	2
Occupational carcinogens	3
Occupational exposure to asbestos	4
Occupational exposure to arsenic	4
Occupational exposure to benzene	4
Occupational exposure to beryllium	4
Occupational exposure to cadmium	4
Occupational exposure to chromium	4
Occupational exposure to diesel engine exhaust	4
Occupational exposure to formaldehyde	4
Occupational exposure to nickel	4
Occupational exposure to polycyclic aromatic hydrocarbons	4
Occupational exposure to silica	4
Occupational exposure to sulfuric acid	4
Occupational exposure to trichloroethylene	4
Occupational asthmagens	3
Occupational particulate matter, gases, and fumes	3
Occupational noise	3
Occupational injuries	3
Occupational ergonomic factors	3
Behavioral risks	1
Child and maternal malnutrition	2
Suboptimal breastfeeding	3
Non-exclusive breastfeeding	4
Discontinued breastfeeding	4
Child growth failure	3

Table S1. GBD risk hierarchy with levels	
**Risk**	**level**
Child underweight	4
Child wasting	4
Child stunting	4
Low birth weight and short gestation	3
Short gestation	4
Low birth weight	4
Iron deficiency	3
Vitamin A deficiency	3
Zinc deficiency	3
Tobacco	2
Smoking	3
Chewing tobacco	3
Secondhand smoke	3
High alcohol use	2
Drug use	2
Dietary risks	2
Diet low in fruits	3
Diet low in vegetables	3
Diet low in legumes	3
Diet low in whole grains	3
Diet low in nuts and seeds	3
Diet low in milk	3
Diet high in red meat	3
Diet high in processed meat	3
Diet high in sugar-sweetened beverages	3
Diet low in fiber	3
Diet low in calcium	3
Diet low in seafood omega-3 fatty acids	3
Diet low in omega-6 polyunsaturated fatty acids	3
Diet high in trans fatty acids	3
Diet high in sodium	3
Intimate partner violence	2
Childhood sexual abuse and bullying	2
Childhood sexual abuse	3
Bullying victimization	3
Unsafe sex	2
Low physical activity	2
Metabolic risks	1
High fasting plasma glucose	2
High LDL cholesterol	2
High systolic blood pressure	2
High body-mass index	2
Low bone mineral density	2
Kidney dysfunction	2

Table 2. Types of Comparative Risk Assessments (CRA) based on the time perspective and the nature of the counterfactual level or distribution of exposure.
The shaded box represents the type of CRA currently undertaken in GBD 2021. GBD = Global Burden of Disease

	**Counterfactual distributions of exposure**			
**Construct**	Theoretical minimum risk: level of risk with the lowest level of burden	Plausible minimum risk: level of risk with the lowest level of burden that could be imagined by current technology and knowledge	Feasible minimum risk: level of risk with the lowest level of burden that has been achieved in any population	Cost-effective minimum risk: lowest level of risk that can be achieved cost-effectively in a given population
**Attributable burden:** burden of disease today that would be avoided if each individual in the past had been exposed to counterfactual level of exposure	Currently in GBD			
**Avoidable burden:** burden of disease in the future that would be avoided if each individual today shifted to the counterfactual level of expsoure				

Table S3. GATHER checklist of information that should be included in reports of global health estimates, with description of compliance and location of information for "Global burden of 288 causes of death and life-expectancy decomposition in 204 countries and territories and 811 subnational locations, 1990–2021: a systematic analysis for the Global Burden of Disease Study 2021"			
**#**	**GATHER checklist item**	**Description of compliance**	**Reference**
**Objectives and funding**			
1	Define the indicators, populations, and time periods for which estimates were made.	Narrative provided in paper and methods appendix describing indicators, definitions, and populations	Manuscript (Methods) and methods appendix
2	List the funding sources for the work.	Funding sources listed in paper	Summary (Funding Sources)
**Data Inputs**			
For all data inputs from multiple sources that are synthesized as part of the study:			
3	Describe how the data were identified and how the data were accessed.	Narrative description of data seeking methods provided	Manuscript (Methods) and methods appendix
4	Specify the inclusion and exclusion criteria. Identify all ad-hoc exclusions.	Narrative about inclusion and exclusion criteria by data type provided; ad-hoc exclusions in cause-specific write ups	Methods appendix
5	Provide information on all included data sources and their main characteristics. For each data source used, report reference information or contact name/institution, population represented, data collection method, year(s) of data collection, sex and age range, diagnostic criteria or measurement method, and sample size, as relevant.	An interactive, online data source tool that provides metadata for data sources by component, geography, cause, risk, or impairment has been developed	Online data citation tool https://ghdx.healthdata.org/gbd-2021/sources
6	Identify and describe any categories of input data that have potentially important biases (e.g., based on characteristics listed in item 5).	Summary of known biases by cause included in methods appendix	Methods appendix
For data inputs that contribute to the analysis but were not synthesized as part of the study:			
7	Describe and give sources for any other data inputs.	Included in online data source tool	https://ghdx.healthdata.org/gbd-2021/sources
For all data inputs:			

8	Provide all data inputs in a file format from which data can be efficiently extracted (e.g., a spreadsheet as opposed to a PDF), including all relevant meta-data listed in item 5. For any data inputs that cannot be shared due to ethical or legal reasons, such as third-party ownership, provide a contact name or the name of the institution that retains the right to the data.	Downloads of input data available through online tools, including data visualization tools and data query tools; input data not available in tools will be made available upon request	Online data visualization tools, data query tools, and the Global Health Data Exchange https://ghdx.healthdata.org/gbd-2021/sources
**Data analysis**			
9	Provide a conceptual overview of the data analysis method. A diagram may be helpful.	Flow diagrams of the overall methodological processes, as well as cause-specific modeling processes, have been provided	Manuscript (Methods) and methods appendix
10	Provide a detailed description of all steps of the analysis, including mathematical formulae. This description should cover, as relevant, data cleaning, data pre-processing, data adjustments and weighting of data sources, and mathematical or statistical model(s).	Flow diagrams and corresponding methodological write-ups for each cause, as well as the databases and modeling processes, have been provided	Manuscript (Methods) and methods appendix
11	Describe how candidate models were evaluated and how the final model(s) were selected.	Provided in the methodological write-ups	Methods appendix
12	Provide the results of an evaluation of model performance, if done, as well as the results of any relevant sensitivity analysis.	Provided in the methodological write-ups	Methods appendix
13	Describe methods for calculating uncertainty of the estimates. State which sources of uncertainty were, and were not, accounted for in the uncertainty analysis.	Provided in the methodological write-ups	Methods appendix
14	State how analytic or statistical source code used to generate estimates can be accessed.	Access statement provided	Code is provided at https://ghdx.healthdata.org/gbd-2021/code
**Results and Discussion**			

15	Provide published estimates in a file format from which data can be efficiently extracted.	GBD 2021 results are available through online data visualization tools, the Global Health Data Exchange, and the online data query tool	Manuscript, supplementary results, and online data tools (data visualization tools, data query tools, and the Global Health Data Exchange); http://ghdx.healthdata.org/gbd-data-tool
16	Report a quantitative measure of the uncertainty of the estimates (e.g. uncertainty intervals).	Uncertainty intervals are provided with all results	Manuscript, supplementary results, and online data tools (data visualization tools, data query tools, and the Global Health Data Exchange); http://ghdx.healthdata.org/gbd-data-tool
17	Interpret results in light of existing evidence. If updating a previous set of estimates, describe the reasons for changes in estimates.	Discussion of methodological changes between GBD rounds provided in the narrative of the manuscript and methods appendix	Manuscript (Research in Context and Discussion) and methods appendix
18	Discuss limitations of the estimates. Include a discussion of any modeling assumptions or data limitations that affect interpretation of the estimates.	Discussion of limitations provided in the narrative of the manuscript, as well as in the methodological write-ups in the methods appendix	Manuscript (Limitations) and methods appendix

Table S4. GBD location hierarchy with levels	
**Geography**	**level**
Global	0
Central Europe, eastern Europe, and central Asia	1
Central Asia	2
Armenia	3
Azerbaijan	3
Georgia	3
Kazakhstan	3
Kyrgyzstan	3
Mongolia	3
Tajikistan	3
Turkmenistan	3
Uzbekistan	3
Central Europe	2
Albania	3
Bosnia and Herzegovina	3
Bulgaria	3
Croatia	3
Czechia	3
Hungary	3
Montenegro	3
North Macedonia	3
Poland	3
Romania	3
Serbia	3
Slovakia	3
Slovenia	3
Eastern Europe	2
Belarus	3
Estonia	3
Latvia	3
Lithuania	3
Moldova	3
Russia	3
Ukraine	3
High income	1
Australasia	2
Australia	3
New Zealand	3
High-income Asia Pacific	2
Brunei	3
Japan	3
Aichi	4
Akita	4
Aomori	4

Table S4. GBD location hierarchy with levels	
**Geography**	**level**
Chiba	4
Ehime	4
Fukui	4
Fukuoka	4
Fukushima	4
Gifu	4
Gunma	4
Hiroshima	4
Hokkaidō	4
Hyōgo	4
Ibaraki	4
Ishikawa	4
Iwate	4
Kagawa	4
Kagoshima	4
Kanagawa	4
Kōchi	4
Kumamoto	4
Kyōto	4
Mie	4
Miyagi	4
Miyazaki	4
Nagano	4
Nagasaki	4
Nara	4
Niigata	4
Ōita	4
Okayama	4
Okinawa	4
Ōsaka	4
Saga	4
Saitama	4
Shiga	4
Shimane	4
Shizuoka	4
Tochigi	4
Tokushima	4
Tōkyō	4
Tottori	4
Toyama	4
Wakayama	4
Yamagata	4
Yamaguchi	4
Yamanashi	4

Table S4. GBD location hierarchy with levels	
**Geography**	**level**
South Korea	3
Singapore	3
High-income North America	2
Canada	3
Greenland	3
USA	3
Alabama	4
Alaska	4
Arizona	4
Arkansas	4
California	4
Colorado	4
Connecticut	4
Delaware	4
Washington, DC	4
Florida	4
Georgia	4
Hawaii	4
Idaho	4
Illinois	4
Indiana	4
Iowa	4
Kansas	4
Kentucky	4
Louisiana	4
Maine	4
Maryland	4
Massachusetts	4
Michigan	4
Minnesota	4
Mississippi	4
Missouri	4
Montana	4
Nebraska	4
Nevada	4
New Hampshire	4
New Jersey	4
New Mexico	4
New York	4
North Carolina	4
North Dakota	4
Ohio	4
Oklahoma	4
Oregon	4

Table S4. GBD location hierarchy with levels	
**Geography**	**level**
Pennsylvania	4
Rhode Island	4
South Carolina	4
South Dakota	4
Tennessee	4
Texas	4
Utah	4
Vermont	4
Virginia	4
Washington	4
West Virginia	4
Wisconsin	4
Wyoming	4
Southern Latin America	2
Argentina	3
Chile	3
Uruguay	3
Western Europe	2
Andorra	3
Austria	3
Belgium	3
Cyprus	3
Denmark	3
Finland	3
France	3
Germany	3
Greece	3
Iceland	3
Ireland	3
Israel	3
Italy	3
Abruzzo	4
Basilicata	4
Calabria	4
Campania	4
Emilia-Romagna	4
Friuli-Venezia Giulia	4
Lazio	4
Liguria	4
Lombardia	4
Marche	4
Molise	4
Piemonte	4
Provincia autonoma di Bolzano	4

Table S4. GBD location hierarchy with levels	
**Geography**	**level**
Provincia autonoma di Trento	4
Puglia	4
Sardegna	4
Sicilia	4
Toscana	4
Umbria	4
Valle d'Aosta	4
Veneto	4
Luxembourg	3
Malta	3
Monaco	3
Netherlands	3
Norway	3
Agder	4
Innlandet	4
Møre og Romsdal	4
Nordland	4
Oslo	4
Rogaland	4
Troms og Finnmark	4
Trøndelag	4
Vestfold og Telemark	4
Vestland	4
Viken	4
Portugal	3
San Marino	3
Spain	3
Sweden	3
Stockholm	4
Sweden except Stockholm	4
Switzerland	3
UK	3
England	4
East Midlands	5
Derby	6
Derbyshire	6
Leicester	6
Leicestershire	6
Lincolnshire	6
Northamptonshire	6
Nottingham	6
Nottinghamshire	6
Rutland	6
East of England	5

Table S4. GBD location hierarchy with levels	
**Geography**	**level**
Bedford	6
Cambridgeshire	6
Central Bedfordshire	6
Essex	6
Hertfordshire	6
Luton	6
Norfolk	6
Peterborough	6
Southend-on-Sea	6
Suffolk	6
Thurrock	6
Greater London	5
Barking and Dagenham	6
Barnet	6
Bexley	6
Brent	6
Bromley	6
Camden	6
Croydon	6
Ealing	6
Enfield	6
Greenwich	6
Hackney	6
Hammersmith and Fulham	6
Haringey	6
Harrow	6
Havering	6
Hillingdon	6
Hounslow	6
Islington	6
Kensington and Chelsea	6
Kingston upon Thames	6
Lambeth	6
Lewisham	6
Merton	6
Newham	6
Redbridge	6
Richmond upon Thames	6
Southwark	6
Sutton	6
Tower Hamlets	6
Waltham Forest	6
Wandsworth	6
Westminster	6

Table S4. GBD location hierarchy with levels

Geography	level
North East England	5
County Durham	6
Darlington	6
Gateshead	6
Hartlepool	6
Middlesbrough	6
Newcastle upon Tyne	6
North Tyneside	6
Northumberland	6
Redcar and Cleveland	6
South Tyneside	6
Stockton-on-Tees	6
Sunderland	6
North West England	5
Blackburn with Darwen	6
Blackpool	6
Bolton	6
Bury	6
Cheshire East	6
Cheshire West and Chester	6
Cumbria	6
Halton	6
Knowsley	6
Lancashire	6
Liverpool	6
Manchester	6
Oldham	6
Rochdale	6
Salford	6
Sefton	6
St Helens	6
Stockport	6
Tameside	6
Trafford	6
Warrington	6
Wigan	6
Wirral	6
South East England	5
Bracknell Forest	6
Brighton and Hove	6
Buckinghamshire	6
East Sussex	6
Hampshire	6
Isle of Wight	6

Table S4. GBD location hierarchy with levels

Geography	level
Kent	6
Medway	6
Milton Keynes	6
Oxfordshire	6
Portsmouth	6
Reading	6
Slough	6
Southampton	6
Surrey	6
West Berkshire	6
West Sussex	6
Windsor and Maidenhead	6
Wokingham	6
South West England	5
Bath and North East Somerset	6
Bournemouth	6
Bristol, City of	6
Cornwall	6
Devon	6
Dorset	6
Gloucestershire	6
North Somerset	6
Plymouth	6
Poole	6
Somerset	6
South Gloucestershire	6
Swindon	6
Torbay	6
Wiltshire	6
West Midlands	5
Birmingham	6
Coventry	6
Dudley	6
Herefordshire, County of	6
Sandwell	6
Shropshire	6
Solihull	6
Staffordshire	6
Stoke-on-Trent	6
Telford and Wrekin	6
Walsall	6
Warwickshire	6
Wolverhampton	6
Worcestershire	6

Table S4. GBD location hierarchy with levels

Geography	level
Yorkshire and the Humber	5
Barnsley	6
Bradford	6
Calderdale	6
Doncaster	6
East Riding of Yorkshire	6
Kingston upon Hull, City of	6
Kirklees	6
Leeds	6
North East Lincolnshire	6
North Lincolnshire	6
North Yorkshire	6
Rotherham	6
Sheffield	6
Wakefield	6
York	6
Northern Ireland	4
Scotland	4
Wales	4
Latin America and Caribbean	1
Andean Latin America	2
Bolivia	3
Ecuador	3
Peru	3
Caribbean	2
Antigua and Barbuda	3
The Bahamas	3
Barbados	3
Belize	3
Bermuda	3
Cuba	3
Dominica	3
Dominican Republic	3
Grenada	3
Guyana	3
Haiti	3
Jamaica	3
Puerto Rico	3
Saint Kitts and Nevis	3
Saint Lucia	3
Saint Vincent and the Grenadines	3
Suriname	3
Trinidad and Tobago	3
Virgin Islands	3

Table S4. GBD location hierarchy with levels

Geography	level
Central Latin America	2
Colombia	3
Costa Rica	3
El Salvador	3
Guatemala	3
Honduras	3
Mexico	3
Aguascalientes	4
Baja California	4
Baja California Sur	4
Campeche	4
Chiapas	4
Chihuahua	4
Coahuila	4
Colima	4
Durango	4
Guanajuato	4
Guerrero	4
Hidalgo	4
Jalisco	4
México	4
Mexico City	4
Michoacán de Ocampo	4
Morelos	4
Nayarit	4
Nuevo León	4
Oaxaca	4
Puebla	4
Querétaro	4
Quintana Roo	4
San Luis Potosí	4
Sinaloa	4
Sonora	4
Tabasco	4
Tamaulipas	4
Tlaxcala	4
Veracruz de Ignacio de la Llave	4
Yucatán	4
Zacatecas	4
Nicaragua	3
Panama	3
Venezuela	3
Tropical Latin America	2
Brazil	3

Table S4. GBD location hierarchy with levels	
**Geography**	**level**
Acre	4
Alagoas	4
Amapá	4
Amazonas	4
Bahia	4
Ceará	4
Distrito Federal	4
Espírito Santo	4
Goiás	4
Maranhão	4
Mato Grosso	4
Mato Grosso do Sul	4
Minas Gerais	4
Pará	4
Paraíba	4
Paraná	4
Pernambuco	4
Piauí	4
Rio de Janeiro	4
Rio Grande do Norte	4
Rio Grande do Sul	4
Rondônia	4
Roraima	4
Santa Catarina	4
São Paulo	4
Sergipe	4
Tocantins	4
Paraguay	3
North Africa and Middle East	1
North Africa and Middle East	2
Afghanistan	3
Algeria	3
Bahrain	3
Egypt	3
Iran	3
Alborz	4
Ardebil	4
Bushehr	4
Chahar Mahaal and Bakhtiari	4
East Azarbayejan	4
Fars	4
Gilan	4
Golestan	4
Hamadan	4

Table S4. GBD location hierarchy with levels	
Geography	level
Hormozgan	4
Ilam	4
Isfahan	4
Kerman	4
Kermanshah	4
Khorasan-e-Razavi	4
Khuzestan	4
Kohgiluyeh and Boyer-Ahmad	4
Kurdistan	4
Lorestan	4
Markazi	4
Mazandaran	4
North Khorasan	4
Qazvin	4
Qom	4
Semnan	4
Sistan and Baluchistan	4
South Khorasan	4
Tehran	4
West Azarbayejan	4
Yazd	4
Zanjan	4
Iraq	3
Jordan	3
Kuwait	3
Lebanon	3
Libya	3
Morocco	3
Oman	3
Palestine	3
Qatar	3
Saudi Arabia	3
Sudan	3
Syria	3
Tunisia	3
Türkiye	3
United Arab Emirates	3
Yemen	3
South Asia	1
South Asia	2
Bangladesh	3
Bhutan	3
India	3
Nepal	3

Table S4. GBD location hierarchy with levels	
**Geography**	**level**
Pakistan	3
Azad Jammu & Kashmir	4
Balochistan	4
Gilgit-Baltistan	4
Islamabad Capital Territory	4
Khyber Pakhtunkhwa	4
Punjab	4
Sindh	4
Southeast Asia, east Asia, and Oceania	1
East Asia	2
China	3
North Korea	3
Taiwan (province of China)	3
Oceania	2
American Samoa	3
Cook Islands	3
Fiji	3
Guam	3
Kiribati	3
Marshall Islands	3
Federated States of Micronesia	3
Nauru	3
Niue	3
Northern Mariana Islands	3
Palau	3
Papua New Guinea	3
Samoa	3
Solomon Islands	3
Tokelau	3
Tonga	3
Tuvalu	3
Vanuatu	3
Southeast Asia	2
Cambodia	3
Indonesia	3
Aceh	4
Bali	4
Bangka-Belitung Islands	4
Banten	4
Bengkulu	4
Gorontalo	4
Jakarta	4
Jambi	4
West Java	4

Table S4. GBD location hierarchy with levels	
**Geography**	**level**
Central Java	4
East Java	4
West Kalimantan	4
South Kalimantan	4
Central Kalimantan	4
East Kalimantan	4
North Kalimantan	4
Riau Islands	4
Lampung	4
Maluku	4
North Maluku	4
West Nusa Tenggara	4
East Nusa Tenggara	4
Papua	4
West Papua	4
Riau	4
West Sulawesi	4
South Sulawesi	4
Central Sulawesi	4
Southeast Sulawesi	4
North Sulawesi	4
West Sumatra	4
South Sumatra	4
North Sumatra	4
Yogyakarta	4
Laos	3
Malaysia	3
Maldives	3
Mauritius	3
Myanmar	3
Philippines	3
Abra	4
Agusan Del Norte	4
Agusan Del Sur	4
Aklan	4
Albay	4
Antique	4
Apayao	4
Aurora	4
Basilan	4
Bataan	4
Batanes	4
Batangas	4
Benguet	4

Table S4. GBD location hierarchy with levels	
**Geography**	**level**
Biliran	4
Bohol	4
Bukidnon	4
Bulacan	4
Cagayan	4
Camarines Norte	4
Camarines Sur	4
Camiguin	4
Capiz	4
Catanduanes	4
Cavite	4
Cebu	4
Cotabato (North Cotabato)	4
Davao de Oro	4
Davao Del Norte	4
Davao Del Sur	4
Davao Occidental	4
Davao Oriental	4
Dinagat Islands	4
Eastern Samar	4
Guimaras	4
Ifugao	4
Ilocos Norte	4
Ilocos Sur	4
Iloilo	4
Isabela	4
Kalinga	4
La Union	4
Laguna	4
Lanao Del Norte	4
Lanao Del Sur	4
Leyte	4
Maguindanao	4
Marinduque	4
Masbate	4
Misamis Occidental	4
Misamis Oriental	4
Mountain Province	4
National Capital Region	4
Negros Occidental	4
Negros Oriental	4
Northern Samar	4
Nueva Ecija	4
Nueva Vizcaya	4

Table S4. GBD location hierarchy with levels

Geography	level
Occidental Mindoro	4
Oriental Mindoro	4
Palawan	4
Pampanga	4
Pangasinan	4
Quezon	4
Quirino	4
Rizal	4
Romblon	4
Samar (Western Samar)	4
Sarangani	4
Siquijor	4
Sorsogon	4
South Cotabato	4
Southern Leyte	4
Sultan Kudarat	4
Sulu	4
Surigao Del Norte	4
Surigao Del Sur	4
Tarlac	4
Tawi-Tawi	4
Zambales	4
Zamboanga Del Norte	4
Zamboanga Del Sur	4
Zamboanga Sibugay	4
Seychelles	3
Sri Lanka	3
Thailand	3
Timor-Leste	3
Viet Nam	3
Sub-Saharan Africa	1
Central sub-Saharan Africa	2
Angola	3
Central African Republic	3
Congo (Brazzaville)	3
DR Congo	3
Equatorial Guinea	3
Gabon	3
Eastern sub-Saharan Africa	2
Burundi	3
Comoros	3
Djibouti	3
Eritrea	3
Ethiopia	3

Table S4. GBD location hierarchy with levels	
**Geography**	**level**
Addis Ababa	4
Afar	4
Amhara	4
Benishangul-Gumuz	4
Dire Dawa	4
Gambella	4
Harari	4
Oromia	4
Somali	4
Southern Nations, Nationalities, and Peoples	4
Tigray	4
Kenya	3
Baringo	4
Bomet	4
Bungoma	4
Busia	4
Elgeyo Marakwet	4
Embu	4
Garissa	4
Homa Bay	4
Isiolo	4
Kajiado	4
Kakamega	4
Kericho	4
Kiambu	4
Kilifi	4
Kirinyaga	4
Kisii	4
Kisumu	4
Kitui	4
Kwale	4
Laikipia	4
Lamu	4
Machakos	4
Makueni	4
Mandera	4
Marsabit	4
Meru	4
Migori	4
Mombasa	4
Murang'a	4
Nairobi	4
Nakuru	4
Nandi	4

Table S4. GBD location hierarchy with levels	
**Geography**	**level**
Narok	4
Nyamira	4
Nyandarua	4
Nyeri	4
Samburu	4
Siaya	4
Taita Taveta	4
Tana River	4
Tharaka Nithi	4
Trans Nzoia	4
Turkana	4
Uasin Gishu	4
Vihiga	4
Wajir	4
West Pokot	4
Madagascar	3
Malawi	3
Mozambique	3
Rwanda	3
Somalia	3
South Sudan	3
Uganda	3
Tanzania	3
Zambia	3
Southern sub-Saharan Africa	2
Botswana	3
Eswatini	3
Lesotho	3
Namibia	3
South Africa	3
Eastern Cape	4
Free State	4
Gauteng	4
KwaZulu-Natal	4
Limpopo	4
Mpumalanga	4
North West	4
Northern Cape	4
Western Cape	4
Zimbabwe	3
Western sub-Saharan Africa	2
Benin	3
Burkina Faso	3
Cabo Verde	3

Table S4. GBD location hierarchy with levels	
**Geography**	**level**
Cameroon	3
Chad	3
Côte d'Ivoire	3
The Gambia	3
Ghana	3
Guinea	3
Guinea-Bissau	3
Liberia	3
Mali	3
Mauritania	3
Niger	3
Nigeria	3
São Tomé and Príncipe	3
Senegal	3
Sierra Leone	3
Togo	3

Table S5. Socio-demographic Index values for all estimated GBD 2021 locations, 1990-2021																																
**Location**	**1990**	**1991**	**1992**	**1993**	**1994**	**1995**	**1996**	**1997**	**1998**	**1999**	**2000**	**2001**	**2002**	**2003**	**2004**	**2005**	**2006**	**2007**	**2008**	**2009**	**2010**	**2011**	**2012**	**2013**	**2014**	**2015**	**2016**	**2017**	**2018**	**2019**	**2020**	**2021**
Global	0.52552886	0.53027248	0.53463654	0.53859832	0.54238943	0.546328	0.55070341	0.55513943	0.55938057	0.56373802	0.56800316	0.57177753	0.57568796	0.57990708	0.58437859	0.58897852	0.59375662	0.59864137	0.6033701	0.60775944	0.6129525	0.61775922	0.62184498	0.62634328	0.63098237	0.63544149	0.64015359	0.6455643	0.65104847	0.6565759	0.66134354	0.66582097
Central Europe, eastern Europe, and central Asia	0.63831682	0.64426072	0.65030023	0.65498411	0.6588964	0.66323061	0.6673985	0.67106161	0.67444171	0.67804438	0.68204328	0.68616546	0.69041062	0.69548048	0.70141441	0.70707179	0.71222406	0.71722196	0.72255514	0.72727958	0.73207766	0.73607829	0.73967451	0.74328837	0.74665736	0.74987717	0.75335999	0.75709847	0.76076037	0.76422712	0.76683705	0.76946635
Central Asia	0.55336195	0.55514777	0.55726954	0.56008289	0.56269953	0.5655856	0.56876058	0.57176648	0.57465939	0.5781207	0.58215554	0.5866458	0.59145711	0.59652977	0.60173672	0.60729382	0.61307828	0.61910458	0.62493038	0.63010289	0.6352956	0.64031356	0.64467352	0.64888814	0.65299988	0.65681783	0.66031242	0.66357678	0.66666645	0.6698373	0.67248671	0.67516398
Armenia	0.54441453	0.54756186	0.54897472	0.55121467	0.55378384	0.55681551	0.56053778	0.56462746	0.56955666	0.57432306	0.57943757	0.5854193	0.59271061	0.60080917	0.60903177	0.61775963	0.62668834	0.63587098	0.64443159	0.64971484	0.65481153	0.65981809	0.66483314	0.6695345	0.67393219	0.67805463	0.6817464	0.68598027	0.69027687	0.69476596	0.6982689	0.70183319
Azerbaijan	0.59598603	0.59580771	0.59465134	0.59354268	0.59208879	0.58944146	0.58631595	0.58322181	0.58039289	0.57919251	0.58002057	0.58219371	0.58571614	0.59003871	0.59464608	0.60248519	0.6137711	0.62611093	0.63690111	0.64516334	0.65205651	0.65797163	0.662897	0.66765878	0.67216274	0.67624491	0.67980764	0.6832632	0.68656109	0.68983471	0.69260519	0.69485127
Georgia	0.65613604	0.66211319	0.66502143	0.66346686	0.65813414	0.6513636	0.64455809	0.63852563	0.63334793	0.63027795	0.63026599	0.6333883	0.63837011	0.64406558	0.64892098	0.653616	0.65840337	0.66369797	0.6684425	0.67244938	0.67703489	0.68156704	0.68551922	0.68860327	0.69215521	0.69731613	0.70373149	0.71087472	0.71799365	0.72453646	0.72912589	0.7324736
Kazakhstan	0.5894358	0.59096783	0.59612766	0.60313833	0.6105911	0.61857358	0.62601314	0.63116663	0.63538862	0.64018049	0.64468962	0.64839726	0.65152934	0.65483593	0.65873307	0.66294394	0.66704886	0.67136721	0.67635287	0.68160684	0.68699664	0.69175895	0.69531277	0.69905359	0.70351192	0.70822974	0.71256904	0.71602669	0.718532	0.7207976	0.72278877	0.7251445
Kyrgyzstan	0.51940765	0.52374671	0.52790155	0.53243101	0.53623298	0.53905282	0.54162893	0.54334115	0.54325855	0.54286728	0.54303649	0.54380555	0.54437467	0.54564926	0.54726234	0.54826045	0.54938359	0.55114619	0.55320638	0.55512387	0.55641654	0.55892333	0.56154157	0.56577677	0.57056936	0.57546323	0.58038855	0.5857661	0.59123713	0.59650164	0.60049955	0.60397933
Mongolia	0.46655012	0.47219901	0.47758655	0.48272673	0.48827668	0.49500678	0.50170372	0.50855827	0.51479338	0.52078119	0.52665034	0.5326202	0.53842483	0.54397165	0.5495515	0.55468028	0.55956114	0.5642368	0.5685006	0.57134169	0.57440667	0.57860066	0.58325589	0.58808081	0.59286735	0.59685965	0.60026077	0.60372196	0.60750183	0.61125068	0.61462964	0.61762156
Tajikistan	0.46615541	0.47234217	0.47574519	0.47823881	0.47872601	0.47758687	0.47434603	0.4702748	0.46534174	0.46050562	0.45706133	0.45625651	0.45880778	0.46332971	0.46915421	0.47463779	0.47965183	0.48421116	0.48844114	0.49206404	0.49589396	0.4998698	0.50395388	0.50797068	0.51198952	0.51578324	0.519897	0.52449261	0.52915392	0.53376995	0.53769053	0.54151119
Turkmenistan	0.56312689	0.56493939	0.56558751	0.56717445	0.56728898	0.56719863	0.56706126	0.56562308	0.56440526	0.56471601	0.56742103	0.57104382	0.57559617	0.58082013	0.58671235	0.59338889	0.59989607	0.6060422	0.61192331	0.61765291	0.62323459	0.62923372	0.63526318	0.64136189	0.64754242	0.65335279	0.65894561	0.66428469	0.6694336	0.67430354	0.67829818	0.68216078
Uzbekistan	0.50024174	0.50154461	0.50290519	0.50655662	0.51101852	0.51698626	0.52439291	0.53268633	0.54127907	0.54973939	0.55752231	0.56480338	0.57169014	0.57800654	0.58394299	0.58953078	0.59449806	0.59962805	0.60488916	0.61035175	0.61609791	0.62134462	0.62620213	0.6307964	0.63525705	0.63980564	0.6442306	0.64811935	0.65191814	0.65583696	0.6591242	0.66262169
Central Europe	0.63727217	0.64308262	0.64877215	0.65446991	0.66121939	0.66795495	0.67412117	0.68003726	0.68596837	0.69205618	0.69877971	0.70565678	0.7120006	0.71782364	0.72349425	0.72867409	0.73347644	0.73807667	0.74314784	0.74876572	0.75491949	0.76034929	0.76480327	0.76856771	0.77175547	0.77484401	0.77781725	0.78135932	0.78547633	0.78974898	0.79311112	0.79624445
Albania	0.5577733	0.55562941	0.5531079	0.55245333	0.55376367	0.55704089	0.56224889	0.5668028	0.5725341	0.57829546	0.58466419	0.59241295	0.59986598	0.60697117	0.61386425	0.62067961	0.62748562	0.63416797	0.64076613	0.64681613	0.6526542	0.65819677	0.66355678	0.66873956	0.67411299	0.6796686	0.68503665	0.6903792	0.69556688	0.70028786	0.70379025	0.70684979
Bosnia and Herzegovina	0.54113254	0.54134625	0.53930323	0.53603188	0.53385339	0.53402554	0.54554922	0.56356541	0.58182952	0.59799747	0.61095068	0.62198946	0.63207552	0.6406351	0.64779373	0.65385914	0.65981366	0.66557874	0.67110081	0.6760329	0.6808242	0.68539926	0.68950657	0.69350871	0.69703189	0.70044605	0.70407934	0.70805343	0.7123473	0.71685712	0.72020206	0.72307789
Bulgaria	0.6334465	0.64306949	0.65118688	0.65916157	0.66743046	0.67399998	0.67886258	0.68022147	0.67849688	0.67744654	0.68100452	0.68648737	0.69151167	0.69556596	0.69988195	0.70447121	0.70886829	0.7131924	0.71767245	0.72367663	0.73070174	0.73574928	0.73917752	0.74213595	0.74537264	0.74897906	0.7522592	0.75566586	0.75914846	0.76235979	0.76508971	0.76815094
Croatia	0.66890636	0.6747246	0.67809313	0.67822105	0.67754813	0.67702947	0.67950732	0.68536501	0.69136552	0.69663641	0.7030399	0.70953684	0.71538342	0.72113038	0.72632765	0.73215414	0.73806688	0.74314878	0.7484696	0.75406561	0.75947225	0.76368638	0.76740534	0.77115188	0.77447924	0.77786589	0.78144361	0.78507454	0.78867141	0.79247542	0.79546227	0.79834103
Czechia	0.68184802	0.68800286	0.69757781	0.71085342	0.72596969	0.73798172	0.74604138	0.75168034	0.75703376	0.76242888	0.76776616	0.77216724	0.77692793	0.78244808	0.7871264	0.79133395	0.79492278	0.79825412	0.80176485	0.80496222	0.80874733	0.81205478	0.8142304	0.81561536	0.81622726	0.81656468	0.81715303	0.81906768	0.82203449	0.82478447	0.82662663	0.82845043
Hungary	0.64941999	0.65370715	0.66038291	0.66754356	0.67505141	0.68318641	0.69088333	0.69793551	0.70420272	0.70998063	0.71580528	0.72209906	0.7283846	0.73445066	0.74009997	0.74535681	0.75051538	0.75494294	0.75931747	0.76356369	0.76732865	0.76970194	0.77078425	0.7715617	0.77257505	0.77404738	0.77615031	0.77911642	0.78208366	0.78491031	0.78762039	0.79075477
Montenegro	0.67422572	0.67430572	0.67266477	0.66866572	0.66501193	0.66276544	0.66341477	0.66554203	0.66902477	0.6720961	0.67730685	0.68343345	0.68993948	0.69651751	0.70299828	0.70906546	0.71545907	0.72250394	0.72998176	0.73653893	0.74334787	0.75008033	0.75557838	0.76088691	0.76577653	0.77056128	0.77520077	0.77976463	0.78440897	0.7890269	0.79255439	0.79580058
North Macedonia	0.60902609	0.61133745	0.61270886	0.6131951	0.61436291	0.61699488	0.62094402	0.62622381	0.63205941	0.63668726	0.64165124	0.64804968	0.65539879	0.66340889	0.67123752	0.67804727	0.68404434	0.68972948	0.69537696	0.70045305	0.70558932	0.71059252	0.71517315	0.71967115	0.72408959	0.72860377	0.73317362	0.73733996	0.74133437	0.74519357	0.7478499	0.7506297
Poland	0.62722789	0.63267147	0.64066179	0.6489505	0.65801051	0.66667887	0.67437883	0.68258761	0.69080567	0.69853606	0.70666205	0.7148313	0.72200946	0.72815525	0.73367319	0.73842877	0.74236371	0.74600882	0.75052143	0.75642738	0.76318497	0.76940716	0.77468077	0.77943426	0.78368237	0.78744534	0.79065101	0.79453034	0.79947898	0.80479133	0.80879532	0.81204281
Romania	0.61929886	0.62679563	0.63047508	0.63380142	0.63837193	0.64371888	0.6481042	0.65122558	0.65492777	0.65954485	0.66529435	0.67202017	0.67829742	0.68407493	0.69041705	0.69499895	0.69949077	0.70452373	0.71075713	0.71744512	0.72389135	0.72901827	0.73389248	0.73788047	0.74039547	0.74347353	0.74705871	0.75111737	0.75553333	0.76028397	0.76427644	0.76845386
Serbia	0.63051102	0.63801693	0.64197199	0.64209603	0.64222322	0.64273967	0.64484642	0.64716209	0.65024599	0.65553417	0.6607528	0.66540733	0.67151107	0.67833493	0.68681984	0.69609757	0.70494502	0.7132423	0.72150531	0.7299656	0.74009131	0.75007276	0.75710508	0.76217091	0.76604309	0.76984222	0.77365408	0.77746038	0.78149497	0.7857683	0.78918333	0.79241629
Slovakia	0.65385351	0.6587314	0.66583011	0.67596794	0.68813648	0.69747654	0.704405	0.71151321	0.71905877	0.72614417	0.73404624	0.74139255	0.74679114	0.75116727	0.75583669	0.76172419	0.76767304	0.77291415	0.7771974	0.78105226	0.78625442	0.79146538	0.79545622	0.79790372	0.7996188	0.80039418	0.80087821	0.80227732	0.8043573	0.80644846	0.80830597	0.81061053
Slovenia	0.72746393	0.73266699	0.73739155	0.74177417	0.74623486	0.75099497	0.75634899	0.76227529	0.76772853	0.77323619	0.77957801	0.78624078	0.79180557	0.79637153	0.80096224	0.80560125	0.8097086	0.81300632	0.81634725	0.81887856	0.82117849	0.82302356	0.82433656	0.82582628	0.82779925	0.82983707	0.83136792	0.83303814	0.83547248	0.83826678	0.84037385	0.84243073
Eastern Europe	0.66425016	0.67145237	0.67875289	0.6833592	0.68601159	0.68945934	0.69279135	0.69520295	0.69693983	0.69881365	0.70101118	0.70350622	0.70672714	0.71202517	0.71921544	0.72626571	0.73261745	0.73878686	0.74545042	0.75107016	0.75660648	0.7610253	0.76542275	0.77033829	0.7750917	0.77966348	0.78480469	0.79001487	0.79448558	0.79813838	0.80050506	0.80285101
Belarus	0.62244658	0.62703827	0.63289174	0.63939417	0.64465278	0.6489595	0.65285522	0.6556273	0.65724647	0.66017101	0.66470582	0.67133327	0.67812178	0.68486856	0.69250214	0.69898548	0.7051191	0.71149656	0.71912087	0.72729278	0.73495463	0.73950433	0.74346884	0.74852932	0.75351148	0.75820574	0.76432822	0.77113147	0.77656896	0.78050802	0.78238156	0.78448471
Estonia	0.67496763	0.68522309	0.69558667	0.70369315	0.70845618	0.71317273	0.71881302	0.72465244	0.72902704	0.73325062	0.73971315	0.74660221	0.7526299	0.75781568	0.76322373	0.76911218	0.77445184	0.78107444	0.78859887	0.79472821	0.80082972	0.80663308	0.81110936	0.81488718	0.81877073	0.82322128	0.82736436	0.83097531	0.8345663	0.83857351	0.84181714	0.84491779
Latvia	0.68019364	0.6881173	0.69615704	0.70524986	0.71253135	0.71763946	0.72113954	0.72374658	0.72542734	0.72721968	0.73095145	0.7362942	0.74187044	0.74816816	0.75526321	0.76192943	0.76845993	0.77648196	0.78632457	0.79450443	0.79817159	0.79908078	0.80102647	0.80266049	0.80372149	0.80698599	0.81146359	0.81692312	0.82141981	0.82478178	0.82790045	0.83066352
Lithuania	0.66850394	0.67323412	0.68099288	0.68748062	0.69115165	0.69473438	0.6985913	0.70284095	0.70746818	0.71283417	0.71989696	0.72713358	0.7337934	0.7412296	0.74894976	0.75635236	0.76305912	0.77022964	0.77827371	0.78568974	0.79336468	0.80092499	0.80782703	0.81321972	0.81788638	0.82311263	0.8293037	0.83587174	0.84191891	0.84790634	0.85275239	0.85648405
Moldova	0.60425176	0.60727077	0.60966456	0.61415292	0.61760377	0.62095132	0.62311398	0.6243701	0.62411676	0.62283453	0.62200493	0.62293641	0.62587484	0.62998504	0.63539482	0.64153097	0.64799626	0.65421585	0.6610466	0.66628439	0.67198962	0.67800414	0.6829798	0.68851917	0.69410456	0.69935517	0.70514454	0.71120664	0.71724443	0.72301165	0.72771054	0.73221488
Russia	0.67160058	0.68009921	0.68806802	0.69203619	0.69414497	0.69785421	0.70146823	0.70375287	0.70519852	0.70686354	0.70864365	0.71057036	0.71346096	0.71887512	0.72630532	0.73359278	0.74006466	0.74619151	0.75262324	0.7579604	0.76332012	0.76763359	0.77192038	0.77652698	0.78061752	0.78491377	0.79033015	0.79566948	0.80010814	0.8036582	0.80601147	0.808536
Ukraine	0.64746147	0.65195002	0.65760033	0.6628674	0.66550637	0.66695017	0.66810474	0.66920785	0.6703223	0.67064291	0.671095	0.67246782	0.67454322	0.67861464	0.68538094	0.6920233	0.69809338	0.70420231	0.71098018	0.71618547	0.72101987	0.72489196	0.7288172	0.73403357	0.73988356	0.74379086	0.74685799	0.75059332	0.75445173	0.75798008	0.75992987	0.76077391
High income	0.75158965	0.75589794	0.76045019	0.76467829	0.76869262	0.77236267	0.77564621	0.77871449	0.78156786	0.78449535	0.78791261	0.79147794	0.79480055	0.7975774	0.80008676	0.80217744	0.80403794	0.80641939	0.80949267	0.8128359	0.81652603	0.820039	0.82331217	0.8264454	0.82954203	0.83297246	0.83654703	0.84019794	0.84388377	0.84753265	0.8498679	0.85188481
Australasia	0.7312344	0.73491529	0.73900562	0.74342601	0.74772604	0.75201507	0.7563873	0.76099009	0.7652995	0.76952127	0.77379116	0.77807392	0.78248002	0.78628529	0.78942762	0.79172173	0.79285655	0.79394958	0.79640196	0.79993153	0.80411233	0.80816063	0.81246356	0.81734922	0.82164728	0.82561103	0.82945687	0.83292442	0.83661889	0.84058158	0.84333343	0.84551406
Australia	0.72598252	0.72975986	0.7339014	0.73837474	0.74284657	0.74742682	0.75199174	0.7566339	0.76113327	0.76569751	0.77022384	0.77466279	0.77936094	0.78360018	0.78701654	0.78961552	0.79115569	0.79253268	0.7952929	0.79917437	0.80356154	0.80752022	0.81164446	0.81645366	0.82084085	0.82486072	0.82855994	0.83179827	0.83535462	0.83931743	0.84205131	0.84425281
New Zealand	0.75232165	0.75561609	0.75946137	0.7636346	0.76711866	0.76999804	0.77357528	0.77820578	0.78173076	0.78442864	0.78765956	0.79142608	0.79451959	0.79628688	0.79817748	0.79910729	0.79845687	0.79844762	0.79966951	0.80172753	0.80513318	0.80976035	0.81495756	0.82014599	0.82390894	0.82746249	0.83184397	0.83630179	0.84059786	0.84453321	0.84739874	0.8494425
High-income Asia Pacific	0.76780379	0.77333291	0.77883445	0.78396734	0.78902846	0.79409924	0.79886294	0.80312776	0.80683134	0.81046897	0.81408902	0.81754476	0.82111049	0.82463581	0.82807376	0.83106214	0.83376633	0.83654611	0.83927964	0.84177063	0.84468204	0.84772196	0.85069345	0.85367141	0.85661156	0.85960261	0.86273318	0.86587024	0.8690726	0.87221884	0.87443725	0.876767
Brunei	0.66608192	0.67123383	0.67670253	0.68217544	0.68739409	0.69247116	0.69760223	0.70294439	0.70863012	0.71485124	0.72155249	0.72853507	0.73534392	0.74157809	0.74731171	0.75248179	0.75731866	0.76164313	0.76571426	0.76960168	0.77321922	0.77663189	0.78021655	0.78401704	0.78773329	0.79136757	0.79498077	0.79830994	0.80143655	0.80449939	0.80742409	0.81023437
Japan	0.79025352	0.79461619	0.79915622	0.80297299	0.80668948	0.81051077	0.81387075	0.81667578	0.8190162	0.82106664	0.822939	0.82472324	0.82693677	0.82939222	0.83203738	0.83428869	0.83622226	0.83829416	0.84030122	0.84213366	0.84436181	0.84675027	0.84906149	0.85135699	0.85369275	0.8560975	0.85869817	0.86139331	0.86423714	0.86714818	0.86910688	0.87124181
Aichi	0.80085483	0.80571372	0.8105257	0.81442873	0.81815669	0.82199186	0.82535932	0.82798029	0.83002089	0.83175264	0.8332455	0.83490721	0.83708961	0.8394057	0.84194956	0.84427199	0.84666045	0.84920441	0.85111704	0.85277674	0.8546368	0.85683271	0.85932579	0.86194822	0.86460482	0.86723678	0.87003729	0.87290738	0.87590019	0.8788931	0.88091865	0.88304884
Akita	0.74385628	0.74757474	0.75158244	0.7552731	0.75943629	0.76401447	0.76851064	0.77234586	0.77556893	0.77827903	0.78054579	0.78279736	0.78541866	0.78816362	0.79094398	0.7931404	0.79441252	0.79573206	0.79717433	0.79884151	0.80086617	0.80326262	0.80585363	0.80861305	0.81154735	0.8144146	0.81764909	0.82098122	0.82435531	0.82767269	0.82984444	0.83224902
Aomori	0.73463228	0.73822059	0.74250369	0.74633139	0.75074594	0.75539088	0.75933032	0.76240201	0.76505175	0.76759461	0.76987495	0.77227942	0.77527108	0.77851107	0.78187887	0.78447708	0.78680775	0.78906722	0.79106378	0.79324665	0.79589154	0.79885447	0.80177429	0.8043424	0.80705578	0.80993558	0.81318129	0.81656442	0.82007856	0.8235253	0.8258641	0.82834421
Chiba	0.78871661	0.79342279	0.79819204	0.80219287	0.80628592	0.81024286	0.81386043	0.81676476	0.81899728	0.82058206	0.82190151	0.82296686	0.82447662	0.82641886	0.82857765	0.83034976	0.83172731	0.83338772	0.83513779	0.83673563	0.83861528	0.84053708	0.84235991	0.84447398	0.84657607	0.8486335	0.85084245	0.85309259	0.85544962	0.85794583	0.85947215	0.86129264
Ehime	0.75600835	0.75977639	0.76417931	0.76834458	0.77270038	0.77722728	0.78169862	0.78553159	0.78912502	0.79219341	0.79488205	0.79715003	0.79948284	0.8020362	0.80478449	0.80663557	0.80764538	0.80872204	0.80990072	0.81141894	0.81355548	0.81615461	0.81822375	0.82040592	0.82277036	0.82550713	0.82868215	0.83195796	0.83543283	0.83887631	0.84113997	0.84350171
Fukui	0.76648177	0.77099568	0.77591989	0.78010778	0.78439208	0.78868773	0.7927842	0.79652314	0.79977006	0.80261373	0.80520343	0.80742616	0.8098834	0.81242651	0.81496141	0.81723055	0.81944211	0.82197871	0.82462179	0.82713517	0.82991351	0.83265551	0.8347411	0.83667757	0.83847613	0.84057593	0.84311726	0.84585546	0.84878473	0.85184471	0.85383607	0.85614258
Fukuoka	0.78194094	0.78554334	0.78962864	0.793282	0.79704352	0.8008801	0.80443674	0.80758852	0.81029308	0.81268464	0.81482105	0.81667403	0.81896676	0.82151248	0.82408526	0.82607366	0.82711703	0.82834918	0.82969873	0.83135498	0.83363064	0.83594027	0.83803372	0.83992485	0.84181947	0.84386213	0.84614641	0.84860519	0.85138688	0.85430094	0.85623286	0.85834771
Fukushima	0.74906731	0.75289555	0.75734119	0.76091254	0.76465986	0.76884583	0.7725602	0.77557452	0.77788178	0.78001063	0.78182647	0.78360994	0.78646247	0.78964778	0.79327859	0.79625177	0.79860764	0.80083603	0.80285161	0.8048784	0.80708522	0.80894285	0.81102141	0.81340328	0.81636905	0.81945357	0.82303381	0.82699751	0.83125774	0.83539288	0.83816268	0.84094147
Gifu	0.77061016	0.77516716	0.7799211	0.7840638	0.78793547	0.79189924	0.79571377	0.79884546	0.80141302	0.80359933	0.8055828	0.80757215	0.80983054	0.81238671	0.815011	0.81723705	0.81911886	0.82105667	0.82296334	0.82466643	0.8267801	0.8291073	0.83144978	0.83361806	0.83587588	0.83819813	0.84075883	0.84340128	0.8462117	0.84913588	0.85112348	0.85334719
Gunma	0.77266928	0.77684152	0.78130249	0.78512725	0.78911035	0.7932894	0.79675256	0.79974658	0.80198384	0.80373666	0.80504171	0.80644285	0.80891736	0.81196848	0.81493971	0.8172735	0.81911682	0.82122607	0.82330426	0.82547856	0.82826356	0.83123679	0.83399606	0.83686271	0.83994849	0.84315203	0.84645534	0.84973033	0.85313864	0.85656404	0.85880568	0.86111136

Table S5. Socio-demographic Index values for all estimated GBD 2021 locations, 1990-2021

Location	1990	1991	1992	1993	1994	1995	1996	1997	1998	1999	2000	2001	2002	2003	2004	2005	2006	2007	2008	2009	2010	2011	2012	2013	2014	2015	2016	2017	2018	2019	2020	2021
Hiroshima	0.78873994	0.7929969	0.79772105	0.80168578	0.80556365	0.8096871	0.81343513	0.81649148	0.8188832	0.82097232	0.82297162	0.82467757	0.82664335	0.82894115	0.83136415	0.83335881	0.83457326	0.8360478	0.83730996	0.83866117	0.8405394	0.8429427	0.84510631	0.84740061	0.8500611	0.85299511	0.85609248	0.85915007	0.86231082	0.86549114	0.86765875	0.8699086
Hokkaidō	0.76661876	0.77054505	0.7750022	0.7789979	0.78302704	0.78710436	0.7906244	0.79347298	0.79584126	0.79797199	0.799858	0.80178408	0.80427382	0.80697344	0.80974588	0.81163841	0.81267846	0.81377925	0.8149429	0.81626318	0.8179953	0.8199283	0.82188928	0.82389077	0.82607466	0.82841474	0.83100338	0.83370273	0.83654063	0.83953173	0.84155411	0.84374398
Hyōgo	0.79342225	0.79785413	0.8025584	0.80673976	0.8105915	0.81502147	0.81893551	0.82196384	0.82427579	0.82620486	0.82789823	0.82919088	0.83063265	0.83210027	0.83380631	0.83513412	0.83627039	0.83752276	0.838957	0.84023015	0.84229974	0.84448463	0.84658012	0.84878255	0.85117382	0.85366344	0.85625707	0.85889748	0.86159612	0.86434714	0.8661734	0.86817016
Ibaraki	0.7719232	0.77624027	0.78082861	0.78486436	0.7891977	0.7934051	0.79681591	0.79962031	0.80165805	0.80333471	0.80498668	0.80652655	0.80886007	0.81155625	0.81427517	0.81643648	0.81877336	0.82163186	0.82438561	0.82686706	0.829813	0.83285232	0.83559506	0.83793715	0.84032202	0.84291606	0.84584205	0.84894424	0.85222168	0.85547838	0.857592	0.85980457
Ishikawa	0.77225722	0.7777437	0.78304892	0.78747837	0.79167522	0.79612946	0.80035965	0.80385955	0.80707235	0.81019908	0.81315169	0.81580031	0.81838035	0.8209175	0.82355549	0.82573252	0.82689538	0.82802182	0.82927008	0.83031172	0.83167657	0.8331578	0.83485392	0.83699127	0.8393891	0.84207206	0.84509037	0.84817826	0.85146605	0.85474863	0.85697088	0.85937894
Iwate	0.73618491	0.74002245	0.74475024	0.74897498	0.75361378	0.75846562	0.76270062	0.76612863	0.7689439	0.77169128	0.774133	0.77606918	0.77871648	0.78171599	0.78509849	0.78775726	0.78932272	0.79099356	0.79280502	0.79477593	0.7970759	0.79988635	0.80294938	0.80620657	0.80977659	0.81336791	0.81736283	0.82153995	0.82569305	0.82961876	0.83230429	0.83497969
Kagawa	0.77118347	0.77604986	0.78131297	0.78569121	0.78998734	0.79442244	0.79792066	0.80063888	0.80305194	0.80515161	0.80713888	0.80924832	0.81188091	0.81461316	0.81759622	0.81969154	0.8208718	0.8221698	0.82375216	0.82577386	0.82807738	0.83093591	0.83367028	0.83603564	0.83852418	0.84112252	0.84413236	0.84715498	0.85026935	0.85340351	0.85555665	0.85781836
Kagoshima	0.74333244	0.74734116	0.7516214	0.75529704	0.75920802	0.76328877	0.76695547	0.77018783	0.773109	0.7758911	0.7784486	0.78087689	0.78373284	0.78661203	0.78943215	0.79164939	0.79327096	0.79535829	0.79737271	0.79924869	0.80152326	0.80401512	0.80652573	0.80899375	0.81157205	0.81436806	0.81737057	0.82038433	0.82366461	0.82713188	0.82953918	0.83204731
Kanagawa	0.81702186	0.82108275	0.82515599	0.82863982	0.83206456	0.83557849	0.83900604	0.84182612	0.84399815	0.84561912	0.84694841	0.84806167	0.84948108	0.85124523	0.85315852	0.85483109	0.85659608	0.85864149	0.86061805	0.8619109	0.8636736	0.86550684	0.86713614	0.86864031	0.87001606	0.87161307	0.87338986	0.87530079	0.87742255	0.87967672	0.88103038	0.88265439
Kōchi	0.74200445	0.74530611	0.74941884	0.75338103	0.75789865	0.76267174	0.76703224	0.77086611	0.77427281	0.77745194	0.78045311	0.78341967	0.786654	0.78961915	0.79221878	0.79404857	0.79541345	0.79680627	0.79841258	0.80023214	0.80257547	0.80524945	0.80774471	0.81030636	0.81302166	0.81595017	0.81925189	0.82272476	0.82632776	0.82989924	0.832364	0.83490616
Kumamoto	0.75373116	0.75762005	0.76191929	0.76536387	0.76882678	0.77248233	0.77535205	0.77763261	0.77939577	0.78105345	0.78293029	0.78506212	0.7878304	0.79091605	0.79398795	0.79632154	0.79754144	0.79891207	0.80041557	0.80189814	0.80396303	0.80650876	0.80927457	0.81208558	0.81493941	0.8176735	0.82066318	0.82364727	0.82677894	0.8300365	0.83222153	0.83453659
Kyōto	0.79990393	0.80411073	0.80847183	0.81238528	0.81606628	0.8196826	0.8229264	0.82561566	0.82803527	0.83024396	0.83257268	0.83446341	0.83660877	0.83902629	0.8416575	0.84395744	0.84570039	0.84752644	0.84908256	0.85045396	0.85227442	0.85413512	0.85590821	0.85762926	0.8596532	0.86206125	0.86452426	0.86698786	0.86957116	0.8722428	0.87404097	0.87601835
Mie	0.76383004	0.76882189	0.77394322	0.7781197	0.78216613	0.78628572	0.79048262	0.79399001	0.79686977	0.79952132	0.80211902	0.8046487	0.80764589	0.81079547	0.8142186	0.81711858	0.81946188	0.82195069	0.82390435	0.82599892	0.82861707	0.83114417	0.8337827	0.83666254	0.83962612	0.8427325	0.84601697	0.84924362	0.85253608	0.85582009	0.85812473	0.86056119
Miyagi	0.77250193	0.77700569	0.78184337	0.78588159	0.78994364	0.79393884	0.79715256	0.79974963	0.80173629	0.80353098	0.80523442	0.80695671	0.80927991	0.81199468	0.81489873	0.81738953	0.81927682	0.82094772	0.82257561	0.82420999	0.8261855	0.8284245	0.83139288	0.83452577	0.83789656	0.84134574	0.84497345	0.84854135	0.85199609	0.85535643	0.85759114	0.85988657
Miyazaki	0.73910863	0.74249764	0.74629663	0.74947547	0.75293606	0.75675515	0.76070268	0.76443568	0.76782981	0.77098375	0.77361448	0.77604665	0.77908425	0.78222887	0.78535599	0.7874564	0.78823714	0.78939737	0.790878	0.79254962	0.79461097	0.7972054	0.79990634	0.80274632	0.805668	0.80854478	0.81162327	0.81466352	0.81794356	0.82146135	0.82397392	0.82658986
Nagano	0.77659984	0.78065074	0.78482894	0.78830185	0.79179262	0.79578257	0.79954	0.80274737	0.80540674	0.80787798	0.81026469	0.81234308	0.81448684	0.8167925	0.81933682	0.8216002	0.82351302	0.82558167	0.82750706	0.82924148	0.83131053	0.83351826	0.83539514	0.83724094	0.83932896	0.84186063	0.84471469	0.84768055	0.85078159	0.85396811	0.85619146	0.85857784
Nagasaki	0.74196583	0.74579612	0.75045173	0.75456316	0.75883105	0.76324704	0.76690791	0.76988383	0.77244373	0.77492483	0.77718146	0.7792525	0.78166337	0.78414904	0.7868197	0.78909626	0.79025376	0.79147035	0.7928075	0.79446748	0.7968678	0.79978117	0.80265729	0.80533637	0.807847	0.81074883	0.81396269	0.81740776	0.82095367	0.82439151	0.82674305	0.82921388
Nara	0.77849492	0.78311529	0.78771718	0.79188812	0.79602123	0.80010327	0.80419018	0.80748444	0.81006833	0.81241853	0.81465722	0.81672094	0.81892478	0.82114727	0.8233192	0.82502828	0.82607449	0.82719269	0.8284619	0.82958941	0.83127913	0.83279357	0.83436246	0.83615687	0.83794932	0.83973697	0.84173331	0.84378632	0.84606016	0.84844766	0.8499352	0.8516522
Niigata	0.75594262	0.76014411	0.76475705	0.76869099	0.77286292	0.77731692	0.78110278	0.78439407	0.78731349	0.79006443	0.79270107	0.79536472	0.79837768	0.80136145	0.80442238	0.80697612	0.80856016	0.81027861	0.81194731	0.81366644	0.81595803	0.81846309	0.82109167	0.82387172	0.82660024	0.82918937	0.83193627	0.83476685	0.83769159	0.84074048	0.84285295	0.8451521
Ōita	0.7639453	0.76820385	0.77285722	0.77686507	0.78092744	0.78517787	0.78878235	0.79217702	0.79496134	0.79746945	0.80007512	0.80245469	0.80536363	0.80858375	0.81173714	0.81398563	0.81528513	0.81650498	0.81780326	0.81893576	0.82088887	0.8233097	0.8256154	0.82769824	0.82994155	0.83239437	0.8352293	0.8382653	0.8414539	0.84471141	0.84693883	0.84928244
Okayama	0.77737818	0.7817814	0.78662038	0.79051231	0.79423738	0.79818739	0.80132481	0.80391333	0.80640833	0.80877309	0.81068495	0.81281515	0.81547384	0.8181863	0.82098671	0.82343597	0.8257387	0.82809923	0.83024672	0.8322331	0.83446952	0.83699498	0.83923484	0.84150667	0.84377162	0.84627974	0.84896787	0.85182062	0.85489701	0.85807399	0.86027156	0.86256022
Okinawa	0.73545322	0.73906647	0.743244	0.74680788	0.75059463	0.75451693	0.75719137	0.7590796	0.76065024	0.76228189	0.76401725	0.76644775	0.77006847	0.77378759	0.77766314	0.78078458	0.78279709	0.78447154	0.78614472	0.78818894	0.7903229	0.79259381	0.79462211	0.79684744	0.79950476	0.80230924	0.80553625	0.80906299	0.81286285	0.81669732	0.8192232	0.82180305
Ōsaka	0.80461579	0.809254	0.81429293	0.81846243	0.82232746	0.82624333	0.82939708	0.83192722	0.83407757	0.83593791	0.8374334	0.83874731	0.8403772	0.84214541	0.84411331	0.84567855	0.84682826	0.84810578	0.8494596	0.85077574	0.85258357	0.8546753	0.85666015	0.85852076	0.86051359	0.86260459	0.8649027	0.8673414	0.86993601	0.8726364	0.87443418	0.87640925
Saga	0.75102028	0.75455215	0.75898869	0.76307863	0.76719548	0.77135671	0.77482347	0.77780846	0.78049642	0.78300603	0.78523171	0.78749215	0.79047822	0.79362705	0.79678813	0.79925514	0.8005295	0.80216197	0.80391131	0.80541167	0.80748932	0.80954996	0.81136767	0.81317367	0.81523093	0.81760275	0.82035528	0.82336197	0.82668387	0.83011189	0.83245558	0.8349259
Saitama	0.78247338	0.78702645	0.7919327	0.796063	0.79995023	0.80374055	0.80702273	0.80977368	0.81185246	0.81346806	0.814842	0.81604769	0.81767503	0.81975178	0.82218282	0.82436765	0.82602183	0.82767181	0.82943717	0.83108422	0.83309052	0.8352491	0.83719716	0.83924866	0.84128281	0.84335055	0.84560362	0.8479581	0.85046102	0.85300708	0.85459453	0.85637105
Shiga	0.79294074	0.79811013	0.80365653	0.80822836	0.81263083	0.81693876	0.82059857	0.82377741	0.82613589	0.8281362	0.82986369	0.83156427	0.83427903	0.83720508	0.84008554	0.84221347	0.84351544	0.84523639	0.84689308	0.84839055	0.85033482	0.85220357	0.85417981	0.85621791	0.85827185	0.86042497	0.86271728	0.86536673	0.86808768	0.87074143	0.87242284	0.87432337
Shimane	0.73955349	0.74360272	0.74804078	0.75188809	0.75616336	0.76086466	0.7655098	0.76977742	0.77358661	0.77687824	0.7796426	0.78232731	0.78556393	0.78911113	0.79279072	0.79552484	0.79748084	0.7996164	0.80143878	0.80306571	0.80532182	0.80832427	0.8114897	0.81450981	0.81719972	0.81995576	0.82325668	0.82653441	0.83002969	0.8336211	0.83608512	0.83867226
Shizuoka	0.78019983	0.7846306	0.78910727	0.79273118	0.79635836	0.80007905	0.80357397	0.80649175	0.80882886	0.81084234	0.81292247	0.81477053	0.81741221	0.82035273	0.82338208	0.82598914	0.82816662	0.83038886	0.83253433	0.83441062	0.83666622	0.83912754	0.8415696	0.84412845	0.84662065	0.84925416	0.85215925	0.85512197	0.85814927	0.86120135	0.86325076	0.86545045
Tochigi	0.77223438	0.77675398	0.78163154	0.78586733	0.78994171	0.79409413	0.79750186	0.80017151	0.80223525	0.80389371	0.80540865	0.80715178	0.8096336	0.81245611	0.81556709	0.81824367	0.82039122	0.82263	0.82476673	0.82701468	0.82970196	0.83231255	0.8347045	0.83713149	0.83990111	0.84308497	0.84643456	0.84970327	0.85313389	0.85652734	0.85879538	0.8611865
Tokushima	0.7538988	0.75862093	0.7637023	0.76799454	0.77217749	0.77663977	0.78069532	0.78424733	0.78748805	0.79080249	0.79397171	0.79725871	0.80114346	0.80546339	0.80963532	0.81277519	0.81507553	0.81712433	0.81927235	0.82144075	0.82423053	0.82720682	0.82999467	0.83275736	0.83565784	0.83864297	0.84195162	0.84547443	0.84917384	0.85272355	0.85506056	0.85748626
Tōkyō	0.86628094	0.87077885	0.87507404	0.87865756	0.88189487	0.8851046	0.8878536	0.89025655	0.8924091	0.89427115	0.8960117	0.8976953	0.89948479	0.90125465	0.90300785	0.90451959	0.90585958	0.90724926	0.90879511	0.91061338	0.912389	0.91424523	0.91590124	0.91740228	0.91888298	0.92027589	0.92171952	0.92310058	0.92457188	0.92606561	0.9274607	0.92881011
Tottori	0.75780225	0.76159698	0.76601127	0.76977314	0.77384583	0.77824226	0.78211928	0.78543809	0.78820708	0.79069086	0.7927611	0.79476359	0.7970453	0.79943445	0.8020004	0.80388624	0.80530368	0.80667932	0.80797056	0.80919693	0.810385	0.81188448	0.81345937	0.81516252	0.81712678	0.81933739	0.82183984	0.82465627	0.82792754	0.83130918	0.83357647	0.83595696
Toyama	0.77680956	0.78182027	0.78668339	0.7907912	0.79468102	0.7987416	0.80289581	0.80627593	0.80921716	0.81190368	0.8145953	0.81715206	0.81999204	0.82317001	0.82639689	0.82912046	0.83097413	0.8328277	0.83471494	0.83622232	0.83836985	0.84062285	0.84267203	0.84483545	0.84712486	0.84954174	0.85221074	0.85493825	0.85785099	0.86084067	0.86288001	0.86518113
Wakayama	0.75373911	0.75774515	0.76215474	0.76611614	0.76993836	0.77391491	0.77768766	0.78084073	0.78355184	0.78608523	0.78860826	0.79118409	0.7940618	0.79710686	0.80036864	0.80339604	0.80590873	0.80822047	0.81048014	0.81255037	0.81535727	0.81868589	0.82200534	0.82512279	0.82811175	0.83091736	0.8338982	0.8367232	0.83965402	0.84273187	0.84476128	0.84703748
Yamagata	0.74624613	0.75007447	0.75422501	0.7576385	0.76116284	0.764953	0.76854048	0.7716565	0.77428777	0.77660916	0.77863534	0.78059314	0.78336657	0.78646429	0.78985381	0.79282314	0.7953713	0.79815611	0.80046852	0.802631	0.80530324	0.80805942	0.8107854	0.81366333	0.81652403	0.81960797	0.82298556	0.82634022	0.82975998	0.83316026	0.83545631	0.83793315
Yamaguchi	0.77051275	0.77421339	0.77863726	0.7821224	0.78573517	0.78967882	0.79312984	0.7961712	0.7987228	0.80097584	0.8031616	0.80535395	0.80828079	0.81121021	0.81415528	0.81655968	0.81818226	0.82006813	0.82208683	0.824121	0.82683188	0.82979826	0.83254721	0.83524197	0.83777225	0.83989353	0.8424799	0.84538757	0.84859169	0.85179793	0.85396251	0.85625589
Yamanashi	0.77735452	0.78150079	0.78566277	0.78921375	0.79292952	0.79688217	0.80025899	0.80310846	0.80506544	0.80681889	0.80863842	0.8104224	0.81257207	0.81505088	0.81792044	0.82047173	0.82319808	0.82601919	0.8285141	0.83019439	0.83272313	0.83546504	0.83777821	0.83984945	0.84196589	0.84419901	0.84649973	0.84875021	0.85117713	0.85388208	0.85570176	0.85778285
South Korea	0.69232931	0.70263113	0.71244084	0.72228674	0.73219791	0.74194003	0.75155324	0.76059168	0.76828189	0.77677344	0.78542882	0.79349628	0.8010243	0.80768779	0.81372093	0.8190934	0.82417982	0.82912795	0.83389463	0.83810833	0.84251143	0.84691323	0.85114026	0.855441	0.85966557	0.86390741	0.86831782	0.87261776	0.87682332	0.88073151	0.88371757	0.88667527
Singapore	0.68640444	0.69505556	0.70342783	0.7123152	0.7212568	0.72998223	0.73902823	0.7475719	0.7548732	0.76083114	0.76771737	0.77429466	0.77973577	0.78416556	0.78985277	0.79645575	0.80334269	0.8106253	0.81812416	0.82425152	0.83022216	0.83382961	0.83739181	0.84042018	0.84289454	0.84540186	0.847597	0.84905152	0.85076273	0.85249734	0.85429601	0.85609777
High-income North America	0.76565986	0.76804998	0.77154982	0.77497763	0.77820675	0.78134824	0.78410475	0.78634761	0.78856903	0.79119629	0.79499703	0.79927482	0.80296133	0.80582125	0.80799119	0.80888669	0.80972116	0.81248611	0.81708393	0.82247001	0.82781414	0.83206088	0.83566894	0.83905868	0.84234095	0.8458767	0.84942337	0.85296011	0.85631618	0.8597494	0.86187425	0.86346547
Canada	0.78197786	0.78415936	0.78703054	0.78971659	0.79259852	0.79652117	0.80106497	0.80483336	0.80813544	0.81226594	0.81668506	0.82069625	0.82440227	0.8278011	0.83092811	0.83328829	0.83491566	0.8366251	0.83891368	0.84158367	0.84485349	0.84801783	0.85097805	0.85384495	0.85675993	0.8595329	0.86217423	0.86505383	0.86790554	0.87055448	0.87199263	0.87317068
Greenland	0.73225825	0.73062653	0.7297811	0.72989484	0.7308759	0.73083164	0.73021623	0.73055109	0.73324259	0.73655713	0.74085701	0.74628961	0.75287837	0.75944415	0.76540157	0.77209958	0.77914141	0.78532192	0.79144126	0.79702575	0.80300313	0.80810519	0.81168456	0.81436831	0.81652706	0.81771919	0.8189619	0.8197811	0.82081551	0.82234	0.82435903	0.82621034
USA	0.76364769	0.76604429	0.76959549	0.77310352	0.77636859	0.77941025	0.78193477	0.78397928	0.78607633	0.78854454	0.79231665	0.79667679	0.80040332	0.80322802	0.80530469	0.80603308	0.80678358	0.80968805	0.8145771	0.82029457	0.82588466	0.83026639	0.83395819	0.83741299	0.84074653	0.84437034	0.84801552	0.8516303	0.85504919	0.85857806	0.86079277	0.86244835
Alabama	0.72397152	0.72675173	0.7311724	0.73542278	0.73915722	0.7424488	0.74477923	0.74596417	0.74729842	0.74978298	0.75562221	0.7634569	0.76967632	0.77372193	0.77544902	0.7737957	0.77140728	0.77285401	0.77897235	0.78761845	0.79555018	0.8008662	0.8045349	0.80709759	0.80885174	0.81000381	0.81118185	0.81358806	0.81642184	0.82052655	0.82377652	0.82560514
Alaska	0.7454567	0.74507701	0.74883217	0.755756	0.76225407	0.76758647	0.77115338	0.77336962	0.77481424	0.77683942	0.78083077	0.78521972	0.7889819	0.79222383	0.79491632	0.79540335	0.79443709	0.79555489	0.79964399	0.80458663	0.80994445	0.81596775	0.82090836	0.82457178	0.82889147	0.83450064	0.84015487	0.84488933	0.84859956	0.85323225	0.85613572	0.8575246
Arizona	0.73653711	0.73878969	0.74148919	0.7438978	0.74586189	0.74756578	0.74918804	0.75062739	0.75212472	0.75368999	0.75636002	0.75953736	0.76246112	0.76501479	0.7674259	0.76937805	0.77264764	0.77881287	0.78717604	0.79577678	0.80338079	0.80908547	0.81356378	0.81774779	0.82165123	0.82564794	0.82971346	0.8340636	0.83821615	0.84226178	0.84475283	0.84673287
Arkansas	0.70264572	0.70789711	0.71465441	0.71895062	0.7212195	0.72252494	0.72404437	0.72662946	0.72987824	0.73309065	0.73748229	0.74320166	0.74775529	0.75153552	0.75372879	0.75302192	0.7514672	0.75412153	0.76043973	0.76857519	0.77587478	0.78133396	0.78603757	0.78957499	0.79265214	0.79556586	0.79882242	0.8027062	0.80635235	0.81171342	0.8156793	0.81770902
California	0.75549857	0.75635433	0.75931059	0.76314174	0.76721932	0.77153682	0.77579064	0.77980965	0.78378332	0.78758629	0.79193486	0.79616917	0.79984861	0.80304853	0.80590143	0.8079627	0.81048972	0.81448132	0.81964069	0.82507426	0.83048851	0.83516311	0.83936823	0.84342579	0.84738175	0.85153146	0.85567482	0.8599178	0.86388959	0.86717602	0.86897947	0.87021505
Colorado	0.78396654	0.7848009	0.78806271	0.79244475	0.7964655	0.79967381	0.80183999	0.80245221	0.80204853	0.80196446	0.80418719	0.80819566	0.81279125	0.81694526	0.81988706	0.82109149	0.82171493	0.82398986	0.82833782	0.83413826	0.84036149	0.84514076	0.8485599	0.85151037	0.85496469	0.86041387	0.86588934	0.87024427	0.87330682	0.87475059	0.875278	0.87665477
Connecticut	0.82431218	0.82783302	0.83156958	0.83447056	0.8369576	0.83914665	0.84055057	0.84161208	0.84366458	0.84714975	0.85175425	0.85669719	0.86069919	0.86256747	0.86334374	0.86325105	0.86340962	0.86495183	0.86811132	0.8723255	0.87642813	0.8796926	0.88265225	0.88567728	0.88874147	0.89176531	0.89437426	0.89643387	0.89825006	0.90042212	0.90214477	0.90310756
Delaware	0.78351977	0.78670763	0.79025099	0.79323986	0.79565634	0.79781879	0.79950599	0.80048766	0.80152795	0.80317951	0.80628035	0.81003888	0.8131746	0.81552757	0.81744644	0.81845518	0.81992419	0.82298905	0.82741328	0.83262738	0.83771046	0.84208282	0.84554445	0.84850874	0.85080132	0.85294475	0.85513302	0.85761662	0.86022942	0.86307653	0.86480183	0.86636623

Table S5. Socio-demographic Index values for all estimated GBD 2021 locations, 1990-2021

Location	1990	1991	1992	1993	1994	1995	1996	1997	1998	1999	2000	2001	2002	2003	2004
Washington, DC	0.78511962	0.78899516	0.79461714	0.80100268	0.80785127	0.81498919	0.82199836	0.82832584	0.8340342	0.83947401	0.84538819	0.85135363	0.85648338	0.85952174	0.86200405
Florida	0.75529951	0.75957736	0.76421451	0.7686355	0.77275674	0.77611663	0.77817577	0.77953506	0.78122149	0.78327984	0.78706618	0.79234156	0.7965943	0.79874702	0.79966403
Georgia	0.73951162	0.74349072	0.74828627	0.75277282	0.75650886	0.75943308	0.76149699	0.76281708	0.76406893	0.76560824	0.76834849	0.77184119	0.7748813	0.77719682	0.77900131
Hawaii	0.76536379	0.76737079	0.77056592	0.77453181	0.78020118	0.78566323	0.79061745	0.79472236	0.7958315	0.79727384	0.80052211	0.80419143	0.80717508	0.81010875	0.81356265
Idaho	0.73646241	0.73732233	0.74041199	0.74437181	0.74781858	0.75100526	0.75455719	0.75760319	0.7603815	0.76363089	0.7676966	0.77187798	0.77524921	0.77750502	0.77896201
Illinois	0.76924489	0.77125388	0.77466917	0.77829484	0.78237699	0.78715847	0.79153833	0.79467225	0.79724958	0.79989308	0.80421124	0.80999749	0.81550795	0.81969255	0.8227012
Indiana	0.75032879	0.75278371	0.75662732	0.76033561	0.76337022	0.76560925	0.76748519	0.76893091	0.77055891	0.77291208	0.77761538	0.78309917	0.78675308	0.78833451	0.78895133
Iowa	0.77326663	0.77542453	0.77895088	0.78234147	0.78534145	0.78770768	0.79046665	0.79301472	0.7950669	0.79778215	0.80196891	0.80658782	0.80977591	0.81121385	0.8112952
Kansas	0.76578268	0.76758298	0.77009548	0.77337761	0.77686084	0.7800481	0.78199239	0.78282797	0.78389532	0.78541963	0.78938978	0.79410424	0.79756874	0.79995935	0.8006555
Kentucky	0.72169882	0.72457641	0.72931033	0.73369416	0.73752802	0.74099727	0.7435904	0.7457364	0.74772197	0.74978603	0.75420833	0.76000231	0.7650894	0.76902435	0.7707573
Louisiana	0.71735673	0.71864455	0.72229255	0.72738957	0.73305248	0.73756337	0.73983977	0.74064879	0.7420071	0.74457604	0.74980683	0.75602604	0.76109762	0.76553255	0.7706367
Maine	0.76873693	0.77426146	0.78075317	0.7864066	0.79084064	0.79401877	0.79595675	0.7969726	0.79854867	0.80111861	0.80478314	0.80919862	0.81319548	0.81615377	0.81817186
Maryland	0.79602368	0.80012387	0.80564034	0.81016513	0.81371748	0.81693609	0.81930476	0.82046271	0.82111379	0.82231727	0.82571292	0.83077548	0.83635915	0.84126649	0.84435198
Massachusetts	0.82570141	0.82979418	0.83357919	0.83688148	0.84010735	0.84330711	0.84579162	0.84771077	0.85003678	0.85315139	0.85738343	0.86187639	0.86574216	0.8686235	0.87068959
Michigan	0.76690804	0.77091804	0.77670253	0.78183197	0.78626406	0.79016802	0.79299145	0.79517439	0.79715201	0.79925975	0.80337512	0.80849511	0.81240646	0.81477789	0.81641922
Minnesota	0.79954949	0.80280014	0.80665799	0.80985858	0.81271152	0.81503988	0.81685386	0.81860652	0.82074059	0.82313167	0.82630704	0.82986937	0.83301535	0.83560568	0.83763177
Mississippi	0.69770961	0.70021718	0.7048727	0.70986045	0.71479328	0.71970347	0.7227825	0.72348113	0.72491065	0.72754967	0.73300925	0.73981595	0.74419718	0.74613448	0.74634436
Missouri	0.75122171	0.75414479	0.75903346	0.76352804	0.76772788	0.77067698	0.77261613	0.77418908	0.77583323	0.77857091	0.78301074	0.78769155	0.79081752	0.7925965	0.79337314
Montana	0.75665039	0.75793747	0.76127463	0.76598227	0.77066705	0.77517149	0.77934286	0.78209283	0.78516835	0.78834873	0.79165948	0.79471095	0.79732911	0.79948287	0.80167168
Nebraska	0.77552129	0.77857512	0.78219833	0.78500835	0.78729054	0.79037557	0.79353353	0.79574494	0.79740392	0.79882666	0.80074384	0.80284267	0.80547258	0.80843384	0.81044858
Nevada	0.74440899	0.74779561	0.75322181	0.75659181	0.75858673	0.76094727	0.76333927	0.76462656	0.76630507	0.76947594	0.77461151	0.78005427	0.78373812	0.78586148	0.78656206
New Hampshire	0.8113287	0.81700015	0.82112844	0.8231773	0.824704	0.82610851	0.82713737	0.82900962	0.83244901	0.83597563	0.84029615	0.84544038	0.84970579	0.85248253	0.85443341
New Jersey	0.81123262	0.81487177	0.81932112	0.82253072	0.82533577	0.82857221	0.83094535	0.83222171	0.83352842	0.83545331	0.83848901	0.84240382	0.84660477	0.85003077	0.85246915
New Mexico	0.71566806	0.7165368	0.71917226	0.72282471	0.72674302	0.73067078	0.73416471	0.73730235	0.74040767	0.74357526	0.74758474	0.75190787	0.75553552	0.75837515	0.76050242
New York	0.79741955	0.8001792	0.80344953	0.80650451	0.80945965	0.81248909	0.8153925	0.81800017	0.82049498	0.82339725	0.82716208	0.83122233	0.83486238	0.83795089	0.8404858
North Carolina	0.74397762	0.74718191	0.75142845	0.75544606	0.75870264	0.76124132	0.76304092	0.76430052	0.76561436	0.76747617	0.77077581	0.77480776	0.77830555	0.78103261	0.78319075
North Dakota	0.77143028	0.77417877	0.77720499	0.78010132	0.78337244	0.78679763	0.79092773	0.79486793	0.79916856	0.8029663	0.80659318	0.80925588	0.81089045	0.81204049	0.81248854
Ohio	0.76134778	0.76346782	0.76672036	0.76998337	0.77302377	0.77582609	0.77819338	0.78025896	0.7824083	0.78492423	0.7886042	0.79263071	0.79603592	0.79870679	0.80065882
Oklahoma	0.73303914	0.73419641	0.73674772	0.73953719	0.74185946	0.74372121	0.74491131	0.74587865	0.74729897	0.74918342	0.75247452	0.75642876	0.76012639	0.76330169	0.76566904
Oregon	0.76900773	0.77109861	0.77422218	0.77739601	0.78026226	0.78311765	0.78606924	0.78901784	0.79214028	0.79560364	0.80004149	0.80454559	0.80836245	0.81147765	0.81383084
Pennsylvania	0.78129556	0.78439203	0.78826835	0.79214349	0.79583902	0.79930643	0.80237506	0.80498094	0.80745701	0.81021012	0.81375928	0.81734631	0.82040861	0.82292176	0.8249734
Rhode Island	0.7952584	0.79839014	0.8018554	0.80508021	0.80790447	0.81044214	0.81250645	0.81451332	0.81682289	0.81931888	0.82233186	0.82559926	0.8287903	0.83178457	0.83460619
South Carolina	0.73136318	0.73533459	0.74091512	0.7464315	0.75116365	0.75499085	0.75764895	0.75936351	0.76093716	0.76303162	0.76652614	0.77076555	0.77450325	0.77729273	0.77920729
South Dakota	0.75122048	0.75387806	0.75777338	0.76222079	0.76664309	0.77064971	0.77477888	0.77838801	0.78172463	0.78475267	0.78803856	0.79077123	0.79233892	0.79345023	0.79423175
Tennessee	0.73014798	0.73274443	0.7367844	0.74086534	0.74467146	0.74822327	0.75103912	0.7531744	0.75538506	0.75781867	0.76107311	0.76432077	0.76649212	0.76761658	0.76807324
Texas	0.72826604	0.72942493	0.73164293	0.73396299	0.73607469	0.73822846	0.74018774	0.74193449	0.74375871	0.74575093	0.74903484	0.75299572	0.75643618	0.75911811	0.76117221
Utah	0.74454779	0.74742352	0.75088115	0.75410314	0.75669059	0.75908549	0.7617242	0.76476951	0.76807019	0.77159088	0.77590353	0.78009663	0.78341518	0.78583595	0.78742259
Vermont	0.7956136	0.79905064	0.80286371	0.80640126	0.809688	0.81268188	0.81534396	0.81803065	0.82107534	0.82441159	0.82817222	0.83198085	0.83551304	0.83878558	0.84189602
Virginia	0.78555628	0.78829726	0.7917984	0.79522674	0.79841274	0.80128724	0.80361209	0.80540029	0.80699957	0.80900986	0.81219476	0.81590062	0.81933188	0.82250774	0.8253964
Washington	0.78110179	0.78319216	0.78644315	0.7898874	0.79322694	0.79646596	0.79962227	0.80278059	0.80627149	0.81010938	0.81463575	0.81898821	0.82260236	0.82537505	0.82758068
West Virginia	0.72593546	0.72805707	0.7316111	0.73551318	0.73930359	0.74282859	0.74590237	0.74852908	0.75099244	0.75346916	0.75672341	0.75994047	0.76229799	0.76373938	0.76447246
Wisconsin	0.78420739	0.78686075	0.79020711	0.79354171	0.79676478	0.79980013	0.80255339	0.80497178	0.80727955	0.80969679	0.81282335	0.81619951	0.81920468	0.82161328	0.8234171
Wyoming	0.75088074	0.75321573	0.75680018	0.76077982	0.76450119	0.76805433	0.77123415	0.77449507	0.77801494	0.78158339	0.78565335	0.789883	0.79369659	0.79716501	0.80001754
Southern Latin America	0.58730812	0.59211869	0.59807976	0.60358857	0.60939202	0.61437285	0.61926005	0.62475649	0.63034909	0.63574846	0.64123743	0.64604037	0.64982584	0.6525524	0.65571888
Argentina	0.58739728	0.59170754	0.59799632	0.60373931	0.60978733	0.61447371	0.61894262	0.62393441	0.62872344	0.63317513	0.63819447	0.6421757	0.64447251	0.64590658	0.64829683
Chile	0.5864951	0.59300658	0.59910608	0.60482851	0.61067393	0.61723468	0.62369446	0.63054008	0.63808276	0.64592808	0.65298903	0.66026551	0.66788841	0.67344593	0.67825821
Uruguay	0.58192186	0.58615612	0.58982236	0.59229503	0.59518842	0.59682541	0.5997751	0.60566505	0.61270082	0.61900927	0.62320829	0.62579607	0.6282858	0.63143777	0.63421691
Western Europe	0.74640048	0.75167003	0.75702193	0.76205678	0.76669311	0.77062172	0.77407985	0.77757769	0.78086689	0.7840192	0.78740001	0.7909044	0.79416518	0.79696151	0.79971572
Andorra	0.76146388	0.76470907	0.76718828	0.76895383	0.77002376	0.77098868	0.77240347	0.77458097	0.77697258	0.77962963	0.78197651	0.78541703	0.79015876	0.7972789	0.80473198
Austria	0.74985369	0.75165273	0.75437529	0.75880455	0.76470436	0.76982747	0.77444657	0.77923286	0.78352102	0.78745158	0.79184768	0.79577012	0.79915412	0.80204325	0.80488914
Belgium	0.73739066	0.74217307	0.7480123	0.75368509	0.75890364	0.7631704	0.76682608	0.77081395	0.77422827	0.77704104	0.78039633	0.78471304	0.78883605	0.79201438	0.79522494

Location	2005	2006	2007	2008	2009	2010	2011	2012	2013	2014	2015	2016	2017	2018	2019	2020	2021
Washington, DC	0.8638492	0.8658439	0.86874988	0.87235519	0.87629923	0.88019268	0.88368874	0.88667212	0.88931788	0.89176552	0.8942426	0.89684475	0.89943496	0.90160674	0.90346778	0.90487397	0.90596476
Florida	0.79950852	0.80016046	0.805009	0.81281767	0.82085509	0.82752137	0.83194251	0.83518066	0.83764876	0.83990453	0.84241877	0.84525239	0.84895038	0.85290197	0.85735427	0.86028435	0.86197928
Georgia	0.77995581	0.7817221	0.7858995	0.79204157	0.79908012	0.8058816	0.81150306	0.81598273	0.82006035	0.82373031	0.82736398	0.83101053	0.83485388	0.83852415	0.84223495	0.84459493	0.84656427
Hawaii	0.81489632	0.8141772	0.81594575	0.82045392	0.82543604	0.82968069	0.83403066	0.83817902	0.8425203	0.84641972	0.84950336	0.85219162	0.85544707	0.85915421	0.86422252	0.86787809	0.86954692
Idaho	0.77886097	0.77874014	0.78135431	0.7868806	0.79382761	0.80052621	0.80524672	0.80861984	0.81168413	0.8142482	0.81711009	0.82053129	0.8246588	0.82859735	0.83217305	0.83442742	0.83651792
Illinois	0.82363116	0.82380506	0.82643505	0.83132047	0.83697893	0.84250671	0.84664728	0.85013995	0.85340074	0.85624513	0.85927826	0.86237907	0.86593665	0.87003437	0.8746681	0.87758765	0.8791878
Indiana	0.78780042	0.78661414	0.78833222	0.79326555	0.79995392	0.80632191	0.8102658	0.81307024	0.81593646	0.81917592	0.8234687	0.82749875	0.83068857	0.83390058	0.83808164	0.84104285	0.84285983
Iowa	0.80994721	0.80908545	0.81119664	0.81593675	0.82209863	0.82805597	0.83220459	0.83512885	0.83795847	0.84120798	0.84602567	0.85118322	0.85520558	0.85820268	0.86106504	0.8627406	0.86405652
Kansas	0.79961479	0.79850218	0.79928582	0.80271151	0.80829952	0.81486303	0.82015747	0.82515781	0.82992241	0.8336819	0.83909081	0.84489567	0.84882384	0.85189158	0.85568546	0.85811325	0.8593838
Kentucky	0.76929998	0.76689502	0.76742391	0.77170995	0.77828183	0.78458599	0.78858841	0.79172061	0.79502482	0.79821007	0.80161768	0.80487866	0.80837217	0.81203222	0.81652405	0.81947102	0.82132538
Louisiana	0.77222979	0.77020122	0.77128654	0.77606806	0.78261679	0.78935779	0.79348297	0.79613755	0.79868449	0.801748	0.80655191	0.81153375	0.81566598	0.8189813	0.82202013	0.82386216	0.82534646
Maine	0.81857963	0.81895572	0.82131027	0.82551619	0.83054852	0.83562734	0.83953726	0.84241783	0.84487464	0.84723843	0.8494598	0.85206084	0.85539736	0.85908815	0.86254016	0.86429199	0.8657542
Maryland	0.84477433	0.84452601	0.84619351	0.85022895	0.85575781	0.86155986	0.86624088	0.87018057	0.87322171	0.8755142	0.87750082	0.87928748	0.88135015	0.883316	0.88590752	0.8881701	0.88922557
Massachusetts	0.87146087	0.87208267	0.87407476	0.87735192	0.88089729	0.88434169	0.88709265	0.88981969	0.89234999	0.89463298	0.89726832	0.89979076	0.90190062	0.90349056	0.90474149	0.90580568	0.90662136
Michigan	0.81680786	0.81702579	0.81890976	0.82218168	0.82572813	0.82932005	0.8321474	0.8345947	0.83717766	0.84036805	0.84464767	0.84921515	0.85350714	0.85719813	0.86039602	0.86229482	0.86397113
Minnesota	0.83803366	0.83795736	0.84012973	0.84460103	0.84998694	0.85501805	0.85837635	0.86116456	0.86410928	0.86735216	0.8711792	0.8749	0.8784915	0.88153238	0.88423098	0.88574722	0.88701394
Mississippi	0.74294979	0.73785966	0.73885629	0.74712518	0.758399	0.76796198	0.7745266	0.77966675	0.78326701	0.78670274	0.79102178	0.79498021	0.79840411	0.80154719	0.80562982	0.80865305	0.81047168
Missouri	0.79232988	0.79135331	0.79339483	0.79897713	0.80608923	0.8124277	0.81666516	0.82016489	0.82327026	0.8261325	0.82968185	0.83324877	0.8361164	0.83920406	0.84360312	0.84654037	0.84816914
Montana	0.80231763	0.80216067	0.80360098	0.80747296	0.81286301	0.81802596	0.821628	0.82410869	0.82682805	0.83079225	0.83698502	0.8438016	0.84935036	0.85362466	0.85640132	0.85767995	0.85910771
Nebraska	0.81049013	0.80975643	0.81121977	0.81465532	0.81995089	0.82627361	0.83120772	0.83431704	0.83701273	0.84065182	0.84676417	0.8524637	0.85630276	0.85938856	0.86223712	0.8639163	0.86512865
Nevada	0.7854999	0.7856779	0.7907889	0.79990851	0.80957506	0.81742273	0.82207684	0.82500747	0.82696897	0.82767345	0.82752292	0.8278382	0.83048291	0.83515676	0.84159686	0.84571734	0.84768278
New Hampshire	0.85516249	0.85570297	0.85740274	0.86065025	0.86473947	0.86873586	0.87242888	0.87575614	0.8785022	0.88140241	0.8852025	0.88880547	0.89179398	0.89440537	0.89650406	0.89742575	0.89826368
New Jersey	0.85330488	0.85375873	0.85558551	0.85896792	0.86301163	0.86666814	0.86978633	0.87315008	0.87628333	0.87902439	0.88159339	0.88383729	0.8855329	0.88704083	0.88916574	0.89095974	0.89192244
New Mexico	0.76140853	0.76245308	0.76599787	0.77194502	0.77932585	0.78677938	0.79288974	0.79800391	0.80278881	0.80720041	0.81139341	0.81550741	0.81964264	0.82352103	0.82749269	0.82994177	0.83171658
New York	0.84207842	0.84362318	0.84623957	0.84944106	0.85309236	0.85699376	0.86048824	0.86382948	0.86721026	0.87037887	0.87317968	0.87567647	0.87796616	0.88003638	0.88222699	0.88398769	0.88517687
North Carolina	0.78422871	0.78556983	0.78913744	0.79472081	0.80127194	0.80764867	0.8127426	0.81697851	0.82058114	0.82370885	0.82684578	0.83009444	0.83346043	0.83679007	0.84032264	0.84258991	0.84444531
North Dakota	0.8124327	0.81248085	0.81435885	0.81775702	0.82128937	0.82529299	0.82907567	0.83370259	0.83918961	0.84572032	0.85272723	0.85873152	0.86332878	0.86677772	0.86946411	0.8714634	0.87303178
Ohio	0.80130891	0.80190295	0.80408154	0.80777078	0.81238808	0.81708476	0.82086955	0.82400733	0.82714939	0.83015518	0.83327117	0.83655872	0.83998301	0.84319001	0.84650352	0.84864434	0.85051343
Oklahoma	0.76645306	0.7672088	0.76995141	0.77497592	0.78074761	0.78676629	0.79175017	0.79623219	0.80102825	0.80569204	0.80992102	0.81395989	0.81779762	0.82113423	0.82460971	0.82685731	0.82866324
Oregon	0.81497578	0.81617291	0.81890423	0.82313795	0.82804832	0.8328378	0.83671981	0.84001866	0.84333901	0.84688273	0.85094112	0.8552195	0.85929779	0.86294828	0.8663105	0.86836459	0.87017769
Pennsylvania	0.82604657	0.82706427	0.82941159	0.83286198	0.83694557	0.84125132	0.8449341	0.8482014	0.85128821	0.85408598	0.85693113	0.85982866	0.86281186	0.86577408	0.86888627	0.87079815	0.87241675
Rhode Island	0.83663359	0.83868594	0.84186213	0.84585046	0.85014421	0.85468085	0.85848081	0.86180964	0.8648276	0.86752102	0.87025444	0.87296488	0.87566678	0.87836046	0.88099217	0.88247115	0.88389348
South Carolina	0.77960739	0.78010407	0.78291056	0.78800914	0.79443254	0.80091042	0.80618206	0.81048096	0.81424638	0.8175081	0.82066857	0.82388592	0.82714201	0.83027907	0.83361293	0.83580625	0.83771865
South Dakota	0.79421848	0.79435773	0.79685798	0.80112718	0.80615925	0.81131194	0.8155374	0.81913629	0.82321513	0.82770429	0.83301613	0.83866908	0.84375463	0.84799196	0.85183974	0.85405383	0.85572087
Tennessee	0.76753847	0.76767102	0.77057345	0.77597665	0.78274085	0.7892576	0.79424059	0.79836996	0.80248224	0.80665125	0.81149604	0.81647233	0.82085239	0.82464378	0.8284145	0.83091173	0.83303324
Texas	0.76203418	0.7632655	0.76692595	0.77287204	0.77970558	0.78669844	0.79242585	0.79736383	0.80233401	0.80747158	0.81284725	0.81801207	0.8228579	0.82687354	0.83058669	0.83288269	0.83478328
Utah	0.78794973	0.7888842	0.79235853	0.79807673	0.80480725	0.81132807	0.81656466	0.82078805	0.82476785	0.82866223	0.83307259	0.83765989	0.84209526	0.84621962	0.8501301	0.85256905	0.85461679
Vermont	0.84401485	0.84594287	0.8486519	0.85197197	0.85531919	0.85872895	0.86189018	0.86485876	0.86798383	0.87125207	0.87481964	0.87833683	0.8815877	0.88452235	0.88722315	0.88867724	0.8899999
Virginia	0.82758542	0.82995382	0.83381356	0.83871469	0.84410621	0.84938871	0.85368094	0.85720844	0.86024216	0.86282264	0.8653471	0.86802926	0.87085374	0.87369732	0.87680399	0.8786606	0.8801199
Washington	0.8284537	0.82931957	0.83170398	0.83531685	0.83943885	0.84369776	0.84731836	0.85078179	0.85426781	0.85775008	0.8613466	0.86494843	0.86857319	0.87188454	0.87493693	0.87649116	0.87758484
West Virginia	0.76404071	0.76366599	0.76496691	0.76801167	0.77239549	0.77702527	0.78069254	0.78381686	0.78743297	0.79146958	0.79591759	0.80041998	0.80464921	0.80861081	0.81284408	0.81563762	0.81771351
Wisconsin	0.82428381	0.82515004	0.82724699	0.83049166	0.83452608	0.83875451	0.8424053	0.84566819	0.84895952	0.8522444	0.85567348	0.85909193	0.86235081	0.86537871	0.86843457	0.87023746	0.8717261
Wyoming	0.80182422	0.80402247	0.80785235	0.81331096	0.81874135	0.82428207	0.82878864	0.83251057	0.83601344	0.83964154	0.84343901	0.84726434	0.85105917	0.85469332	0.85833834	0.86052084	0.86214872
Southern Latin America	0.66051913	0.66438624	0.6673925	0.67068862	0.67415239	0.67876122	0.68413428	0.68842791	0.69171599	0.69603955	0.70328723	0.71078773	0.71754842	0.72495216	0.73113382	0.73396336	0.73598472
Argentina	0.65336883	0.6574459	0.66018758	0.66307924	0.66591194	0.66992588	0.6752427	0.67921824	0.68163321	0.68497451	0.69171013	0.69861803	0.70513553	0.71288869	0.71900284	0.7212943	0.72312297
Chile	0.68258919	0.68589275	0.68893032	0.69235979	0.69660214	0.7023047	0.70859794	0.71474348	0.72044878	0.72727593	0.73609808	0.74497222	0.75205808	0.75862571	0.76512032	0.76921368	0.77151472
Uruguay	0.63697577	0.6405907	0.64633171	0.65352552	0.66032113	0.66609914	0.66707973	0.66580219	0.66822836	0.67333426	0.68059551	0.68981673	0.69918946	0.70690493	0.71253659	0.71616977	0.71928344
Western Europe	0.80241404	0.80506685	0.80772333	0.81048438	0.81311022	0.81614217	0.81937524	0.82263373	0.8257869	0.82860951	0.8313218	0.83421602	0.83752217	0.84083355	0.84421211	0.84655036	0.84872632
Andorra	0.81221398	0.81978726	0.82661725	0.83182171	0.83592989	0.83948997	0.8427948	0.84581083	0.84832991	0.85101049	0.85359122	0.85625705	0.85947703	0.86258895	0.86562102	0.86744917	0.86944411
Austria	0.80841919	0.81206461	0.81548052	0.81879366	0.82154117	0.82450011	0.828002	0.83111331	0.83367978	0.83583858	0.8375799	0.83988379	0.84317333	0.84661447	0.84986251	0.85202039	0.853837
Belgium	0.79849724	0.80157276	0.80461266	0.80775794	0.81105583	0.81503021	0.81947036	0.82422602	0.82861983	0.83242505	0.8358784	0.83946294	0.84321204	0.84661763	0.84949839	0.85134665	0.85365402

Table S5. Socio-demographic Index values for all estimated GBD 2021 locations, 1990-2021

Location	1990	1991	1992	1993	1994	1995	1996	1997	1998	1999	2000	2001	2002	2003	2004	2005	2006	2007	2008	2009	2010	2011	2012	2013	2014	2015	2016	2017	2018	2019	2020	2021
Cyprus	0.64823087	0.65572617	0.66601185	0.67754748	0.68882936	0.6999239	0.70983995	0.71886424	0.72796438	0.73684613	0.7451993	0.75359472	0.76150247	0.76789622	0.77376477	0.77903136	0.78456096	0.79206838	0.79945409	0.80511209	0.80984019	0.81372613	0.81697853	0.8193806	0.8211843	0.82273285	0.82428362	0.82613988	0.82834324	0.83098073	0.83305952	0.83563055
Denmark	0.80115466	0.80454035	0.80816443	0.81158571	0.81513861	0.81910461	0.82337524	0.82767998	0.83168324	0.83538154	0.8391843	0.84317775	0.84708636	0.85044133	0.8533041	0.85569073	0.85803321	0.85978173	0.86178206	0.86394805	0.86676708	0.86977868	0.87251751	0.87493297	0.87681493	0.87854394	0.88058978	0.88368849	0.88752985	0.89162457	0.8943683	0.8964242
Finland	0.75622151	0.75841069	0.7608041	0.7635266	0.7671019	0.77116374	0.77480906	0.77881421	0.78230506	0.78577399	0.78998523	0.79445383	0.79877027	0.80232543	0.80569423	0.80893679	0.81208148	0.81559761	0.81887554	0.82145759	0.82484498	0.82827058	0.83143448	0.8344254	0.83743187	0.84087848	0.84462234	0.84858613	0.85220294	0.85536518	0.85765555	0.85983137
France	0.73074747	0.73645238	0.74287197	0.74858233	0.75317613	0.75712579	0.76089958	0.76465067	0.76785256	0.77030436	0.77279203	0.77622516	0.78008887	0.78317876	0.78608465	0.78904209	0.79232192	0.79555765	0.79817743	0.80050867	0.80324025	0.80637874	0.80961539	0.81298402	0.81655145	0.82023839	0.82373093	0.82709844	0.83044859	0.8337728	0.83604944	0.83836488
Germany	0.81707767	0.8233399	0.8283235	0.83228681	0.83556196	0.83764315	0.83918813	0.8413859	0.84385498	0.8462324	0.84892576	0.85207041	0.85506679	0.85767579	0.86024033	0.86289418	0.86587371	0.86914044	0.87255285	0.87508791	0.87794671	0.88102751	0.88365641	0.88592695	0.88765161	0.88886785	0.89089637	0.89405047	0.89697555	0.89970316	0.90143861	0.90295709
Greece	0.67418647	0.68031648	0.68705539	0.69332893	0.69915302	0.70486287	0.71013934	0.71568727	0.72159398	0.72752945	0.73271054	0.73715401	0.74194522	0.74718596	0.75241861	0.7563844	0.7602097	0.76392717	0.76752766	0.77124736	0.77515581	0.77852587	0.78078948	0.78194905	0.78244201	0.78248952	0.78279844	0.78410859	0.78599975	0.78813814	0.78963222	0.79185441
Iceland	0.76421252	0.76902739	0.77270641	0.77638942	0.77996796	0.78274006	0.78535642	0.78852834	0.79262637	0.79736881	0.80303033	0.80896579	0.81340039	0.81659707	0.81952809	0.82276654	0.82624474	0.83028989	0.83462268	0.83812101	0.84138464	0.84461993	0.84729944	0.85017395	0.8535587	0.85772498	0.86184447	0.86570289	0.86921982	0.87242558	0.87432315	0.87636168
Ireland	0.71989182	0.72519502	0.73191869	0.73838407	0.74401173	0.74892158	0.75401012	0.75997038	0.76648872	0.77353883	0.78019066	0.78635374	0.79301061	0.80000587	0.80685314	0.81171382	0.81450548	0.81693103	0.81993213	0.82392967	0.82810385	0.83180479	0.83532536	0.83864443	0.84208211	0.84743789	0.8526425	0.85781616	0.86392528	0.8694125	0.87195882	0.87375385
Israel	0.70917835	0.71337163	0.71789792	0.72225018	0.72665474	0.73112786	0.73471403	0.73801528	0.74192719	0.74544334	0.74918408	0.7528343	0.75598808	0.75887541	0.76221329	0.76554346	0.7687339	0.77075614	0.77201161	0.77330391	0.77525953	0.77764967	0.78040341	0.78326717	0.78615108	0.78887446	0.79177245	0.79517344	0.79912499	0.80323299	0.80635159	0.80901165
Italy	0.70625522	0.71145735	0.71664975	0.72172099	0.72687091	0.73164408	0.73605087	0.73997234	0.7431016	0.74607408	0.74967599	0.75355484	0.75719148	0.76009374	0.76278775	0.76568991	0.76840241	0.77069838	0.77306847	0.77549926	0.77810921	0.78070347	0.78309098	0.78535688	0.78753396	0.78990212	0.79247724	0.79531119	0.79836269	0.80153002	0.80363568	0.80577353
Abruzzo	0.71465076	0.72031447	0.72603665	0.73144186	0.73685327	0.74213579	0.74701372	0.75138555	0.75498585	0.75841477	0.76230951	0.76627143	0.76981078	0.77259129	0.7749208	0.77748367	0.78003411	0.7823351	0.78475363	0.7870674	0.78959427	0.79238875	0.79503352	0.79742888	0.79964069	0.80191169	0.8043285	0.80702798	0.8099339	0.81294168	0.81485374	0.81684436
Basilicata	0.6665074	0.67261149	0.67884641	0.68485708	0.69093163	0.69677391	0.70243778	0.70781557	0.71259737	0.71743942	0.72247405	0.72738238	0.7318562	0.73532332	0.73845318	0.74157218	0.74473821	0.74771626	0.75056069	0.75297114	0.75530103	0.75779697	0.76012226	0.76253445	0.76456645	0.7672315	0.76993871	0.77299233	0.77619881	0.77947954	0.78170773	0.78401643
Calabria	0.65686437	0.66331557	0.66992451	0.67656687	0.68326606	0.68945548	0.69513357	0.70034628	0.70467563	0.70898274	0.71376972	0.71876051	0.72334746	0.72711597	0.73064081	0.73425293	0.73768069	0.74069277	0.74375746	0.74681285	0.74979242	0.75258591	0.7550263	0.75710955	0.75894233	0.76088193	0.76314053	0.76578207	0.7686683	0.77172208	0.77366768	0.77571305
Campania	0.66170225	0.66752074	0.67347732	0.67943519	0.68549726	0.6909507	0.69580481	0.70011035	0.70334984	0.70642563	0.710384	0.71483251	0.71912469	0.72245743	0.72551192	0.72881216	0.73179638	0.73429793	0.73680287	0.73939939	0.7419047	0.74417748	0.74630984	0.74826609	0.75010838	0.75218558	0.75464023	0.75724465	0.76009759	0.76308919	0.76501158	0.76694337
Emilia-Romagna	0.72194887	0.72718414	0.73243719	0.7377063	0.7431804	0.74822337	0.75305153	0.75734066	0.76078139	0.76414084	0.76827105	0.77253873	0.77628209	0.77901961	0.78142918	0.78412452	0.7867322	0.78893364	0.79133854	0.7939658	0.79692883	0.8000847	0.80307826	0.8060609	0.80895656	0.81196621	0.81513592	0.81840568	0.82182386	0.82524279	0.82749918	0.82972471
Friuli-Venezia Giulia	0.72486612	0.72994147	0.7348731	0.73954377	0.74431686	0.74912879	0.75353693	0.75748016	0.76077176	0.76395572	0.7676116	0.77141897	0.77482504	0.7773821	0.77965972	0.78219666	0.78473396	0.78691235	0.78898627	0.79097991	0.7932738	0.79563655	0.79771559	0.7998653	0.80196795	0.80437676	0.80702286	0.80984508	0.81287264	0.8160362	0.81812693	0.82030206
Lazio	0.73762071	0.7425626	0.74747674	0.75217172	0.75688101	0.76125817	0.76520012	0.7688766	0.77210897	0.77514763	0.77856881	0.78217168	0.78561692	0.78842547	0.79113289	0.79392525	0.79643317	0.79854493	0.80055006	0.80261321	0.80480954	0.80696679	0.80882967	0.81055663	0.81216286	0.81395194	0.8159524	0.8182954	0.82089297	0.82365327	0.82544852	0.82733885
Liguria	0.73018464	0.73488403	0.73939642	0.7435783	0.74779961	0.75195344	0.75585102	0.75945647	0.76258474	0.76551459	0.76891022	0.77253027	0.77570885	0.77832143	0.78071815	0.78324859	0.7856375	0.78779409	0.79010402	0.79236739	0.79470308	0.79712919	0.79937259	0.80154651	0.80375766	0.80618761	0.80880576	0.81158151	0.81458299	0.81775469	0.81990495	0.82213339
Lombardia	0.74234795	0.74700898	0.75148731	0.75570077	0.75994766	0.76388011	0.76749434	0.77069954	0.77331619	0.77567219	0.77846409	0.78151123	0.78440318	0.78665847	0.7886867	0.7909113	0.79294652	0.7946035	0.79657114	0.79868819	0.801242	0.80384378	0.80622528	0.80849255	0.81074986	0.81319289	0.81583541	0.81875497	0.8218674	0.82509769	0.82724976	0.82942992
Marche	0.69707161	0.70242449	0.70784338	0.71322846	0.71874244	0.72381364	0.72864789	0.73305816	0.73674231	0.7404196	0.74471793	0.74912098	0.75326634	0.75668577	0.75982501	0.76309227	0.76622111	0.76899491	0.77171339	0.77445264	0.77720901	0.77984978	0.78222301	0.7844159	0.78654715	0.78879484	0.79118125	0.79372013	0.79655013	0.79950756	0.80146643	0.80347318
Molise	0.67659373	0.68266867	0.68890855	0.69499477	0.70121914	0.70720804	0.71289295	0.71841359	0.72326166	0.72777877	0.73262645	0.73749377	0.74196354	0.74561357	0.74901584	0.75235123	0.75582293	0.75910818	0.76205899	0.7647674	0.76724005	0.76957705	0.77155013	0.77292281	0.77407393	0.77544044	0.77727982	0.77940558	0.78188174	0.78460817	0.78636097	0.7883506
Piemonte	0.72036042	0.72492588	0.729438	0.73367533	0.73800274	0.74213374	0.74588248	0.74929939	0.75208487	0.75472891	0.75780246	0.76104798	0.76406478	0.76647121	0.76868012	0.77106543	0.77334562	0.77524675	0.77714958	0.77897951	0.78114853	0.78340101	0.78542205	0.78743869	0.78941068	0.79161836	0.79404043	0.79674922	0.79970669	0.8028134	0.80490063	0.80703906
Provincia autonoma di Bolzano	0.73240064	0.73785791	0.74316876	0.74834638	0.75362901	0.75885353	0.76380399	0.76795475	0.77138401	0.77445624	0.77819388	0.78213178	0.78557187	0.78819775	0.79059636	0.79319121	0.79577814	0.79798146	0.80049298	0.80329674	0.80635564	0.80950749	0.81268678	0.81577868	0.81868233	0.82180111	0.82508984	0.8283592	0.83180397	0.83522364	0.83753358	0.83971134
Provincia autonoma di Trento	0.73819977	0.74282506	0.74725347	0.75144544	0.75564618	0.7594878	0.76309036	0.76618372	0.76873235	0.77128382	0.77445619	0.7779654	0.78124091	0.78386558	0.78621061	0.78874659	0.79105893	0.79301487	0.79519578	0.79760891	0.80025814	0.80291827	0.80544577	0.80816631	0.8107183	0.81329985	0.81595375	0.81881509	0.82189684	0.82509021	0.82722537	0.82938492
Puglia	0.65735063	0.66335245	0.66940903	0.67529204	0.68124009	0.68676066	0.69182195	0.69621578	0.69972705	0.70320737	0.70743213	0.71187185	0.71597439	0.71924634	0.72219891	0.72525553	0.72809395	0.73048468	0.7327421	0.73502167	0.73741543	0.73975685	0.74204733	0.74412625	0.74611999	0.74836044	0.75072908	0.75359937	0.75666901	0.75981903	0.7618825	0.76398208
Sardegna	0.67992288	0.68512318	0.69029044	0.69524569	0.70019795	0.70478142	0.70896747	0.71296647	0.71629923	0.71954867	0.72321647	0.72709317	0.73064696	0.7337018	0.736569	0.73939613	0.74211495	0.74451652	0.74693411	0.74917971	0.7514295	0.75358639	0.75558526	0.75719746	0.75867488	0.76046161	0.76236154	0.76453972	0.76692095	0.76946266	0.77106593	0.77279343
Sicilia	0.65702584	0.66289764	0.66894624	0.67508263	0.68136789	0.68716076	0.69249562	0.69697706	0.70000867	0.70279709	0.70656829	0.71083671	0.71488561	0.71801036	0.72091595	0.72432638	0.72745396	0.72993636	0.73245307	0.73520871	0.73794983	0.74040256	0.74271317	0.74480941	0.74663498	0.74869127	0.75092322	0.75345291	0.75627166	0.75928364	0.76118761	0.76308681
Toscana	0.71322927	0.71811918	0.72296265	0.72758285	0.73217213	0.73651309	0.7405303	0.74421732	0.74738324	0.75040216	0.75387827	0.75750944	0.76092276	0.76374256	0.76638726	0.76919584	0.77191678	0.77429999	0.77680198	0.77942872	0.78219827	0.78497973	0.7875831	0.79006865	0.79250611	0.79511923	0.79791767	0.80082984	0.80395294	0.80719229	0.80940387	0.81165411
Umbria	0.70668672	0.71170329	0.71675097	0.72159059	0.7264223	0.7311043	0.73529354	0.73916301	0.7423024	0.74523288	0.74866176	0.75221567	0.75544479	0.75805393	0.76049987	0.76315973	0.76579504	0.76811451	0.77058336	0.77290828	0.77541458	0.77783289	0.77998695	0.7819531	0.78360069	0.78553629	0.78755754	0.78986584	0.79249864	0.7952744	0.79713307	0.79910132
Valle d'Aosta	0.72678467	0.73170528	0.73654815	0.74110259	0.74564019	0.75013773	0.75414282	0.75743204	0.75992974	0.76209512	0.76461864	0.76754572	0.77028118	0.77254796	0.77456816	0.77681635	0.77896987	0.78069425	0.78265594	0.78470077	0.78718271	0.78981185	0.79242449	0.79469401	0.79675235	0.79884438	0.80108053	0.80343824	0.80598988	0.80868573	0.81044976	0.81230429
Veneto	0.71497996	0.71999828	0.7248342	0.72942117	0.73395159	0.73813038	0.74205938	0.745613	0.74858881	0.75136182	0.75464995	0.75809357	0.76116472	0.76377475	0.76627952	0.76894777	0.77151022	0.77364916	0.77581791	0.77803542	0.78042181	0.78293418	0.78522309	0.78745176	0.78963029	0.79200151	0.79464011	0.79760854	0.80078415	0.80404827	0.80626835	0.80853444
Luxembourg	0.78105161	0.78596144	0.78905336	0.79284384	0.79782401	0.80268247	0.80651614	0.81023529	0.81388903	0.817571	0.82200657	0.82531282	0.82822349	0.83098395	0.83367257	0.83667526	0.84057445	0.84470701	0.84797888	0.85068873	0.85325828	0.85621683	0.85978903	0.86336879	0.86669356	0.87011294	0.87342088	0.87634768	0.8786379	0.8805939	0.88249532	0.88442896
Malta	0.65650458	0.66111269	0.66632465	0.67212026	0.67784731	0.6826177	0.68662326	0.69175641	0.69809529	0.70473455	0.71180525	0.71780304	0.72279027	0.72751113	0.73193385	0.73618875	0.74014018	0.74423781	0.7484271	0.75217106	0.7562505	0.76031055	0.76453788	0.76861614	0.77272874	0.77727691	0.78167341	0.78641018	0.79096562	0.79519785	0.79837517	0.80158503
Monaco	0.84549515	0.84830369	0.85086394	0.85326499	0.85562693	0.85798615	0.86024543	0.86244672	0.86471651	0.86684239	0.86897358	0.87109475	0.87318277	0.87519765	0.87723155	0.87928809	0.88127415	0.88326229	0.88533139	0.88732828	0.88926284	0.89123759	0.89307382	0.89486447	0.89654787	0.89835634	0.9000513	0.90175271	0.90338869	0.90503099	0.90668544	0.90826283
Netherlands	0.79461212	0.79928544	0.80396598	0.80827897	0.81253022	0.81666277	0.81998422	0.82288177	0.82588223	0.82869383	0.83191398	0.83551815	0.83867608	0.84163948	0.84480735	0.84793419	0.85079881	0.85359539	0.85636847	0.85860046	0.8612553	0.86424668	0.86695063	0.86940298	0.87184891	0.87440284	0.87678405	0.87930015	0.88190022	0.88464384	0.88655857	0.88846426
Norway	0.79588728	0.80046889	0.80543747	0.81056569	0.81541467	0.82022352	0.82570167	0.83146877	0.83656281	0.84113024	0.84647545	0.85211194	0.85675789	0.86030258	0.86346891	0.86621749	0.86858843	0.87092967	0.87374512	0.87658282	0.88037838	0.88435167	0.88816048	0.89198962	0.89549955	0.89891264	0.90258419	0.90604405	0.90926178	0.91227876	0.91452992	0.91613281
Agder	0.7839121	0.78833279	0.79314313	0.79817961	0.80289882	0.80757193	0.81314131	0.81916099	0.82450827	0.82942942	0.835206	0.84113718	0.84578887	0.84886127	0.85123076	0.85324717	0.8551314	0.85711177	0.85964656	0.863013	0.86777517	0.8728041	0.87766051	0.88240259	0.88646454	0.89023755	0.89391665	0.89749111	0.90098392	0.90436101	0.90618338	0.90748954
Innlandet	0.78002909	0.78484974	0.78992594	0.79504007	0.80001687	0.80487496	0.81013378	0.81549994	0.82012239	0.82414912	0.82887656	0.83407175	0.83855199	0.84213011	0.84545935	0.84817672	0.85025528	0.85198146	0.85389993	0.85647485	0.86025388	0.86429668	0.86850093	0.87274681	0.87668321	0.88069691	0.88472309	0.88869096	0.89239856	0.89598152	0.89808057	0.89975406
Møre og Romsdal	0.77915916	0.78437484	0.78992927	0.7956521	0.80096069	0.80598669	0.81159267	0.81741771	0.82255666	0.82721289	0.83271586	0.83838194	0.84312554	0.84689381	0.85036074	0.85329532	0.85573521	0.85788948	0.86034628	0.86376497	0.86864624	0.87373355	0.87786255	0.88199174	0.88583973	0.88949011	0.89335062	0.89693686	0.90026227	0.90339563	0.9057401	0.90742629
Nordland	0.76463467	0.76972526	0.77529284	0.78079124	0.78586567	0.79092471	0.79689245	0.80298912	0.80816006	0.81237422	0.81744423	0.82300433	0.82759556	0.83086046	0.83356025	0.83570012	0.83729213	0.8385198	0.84015423	0.84295699	0.84755707	0.85268038	0.85783537	0.86292616	0.8676087	0.87260291	0.87791292	0.8830385	0.88784245	0.8922541	0.89505198	0.89694228
Oslo	0.83944085	0.84271829	0.84645515	0.85040068	0.85438243	0.8586231	0.86369975	0.86948623	0.87488125	0.87998163	0.88602844	0.89230445	0.89618757	0.89909056	0.90174914	0.90422249	0.90644455	0.90866384	0.91121623	0.91441107	0.91810535	0.92160992	0.92485806	0.92795307	0.93076681	0.9334734	0.93625252	0.93878562	0.94101721	0.94300674	0.94451283	0.94569179
Rogaland	0.7902328	0.79510292	0.80085383	0.80684524	0.81233376	0.81748808	0.82310801	0.82901891	0.8343322	0.83912411	0.84475928	0.85055916	0.85498429	0.85824602	0.86126299	0.86390076	0.86634491	0.86740461	0.86848793	0.87109461	0.87519366	0.87975868	0.88423191	0.88871555	0.89270768	0.89646973	0.90046203	0.90422208	0.90784815	0.91113922	0.91350965	0.91508373
Troms og Finnmark	0.77581152	0.78026523	0.7853149	0.79070256	0.79601	0.80143653	0.80753328	0.81368337	0.81875846	0.82297383	0.82812721	0.83378226	0.83857985	0.84214319	0.84513613	0.84721552	0.84880157	0.85022512	0.85222337	0.8556188	0.8609487	0.8667806	0.87234976	0.87734784	0.88147376	0.8853693	0.88954817	0.89336986	0.89697248	0.90055047	0.90326508	0.9051632
Trøndelag	0.78494115	0.79003437	0.7954634	0.80088911	0.80591465	0.8107009	0.81622486	0.8221593	0.82740744	0.8320705	0.83751572	0.8434422	0.84849593	0.85243307	0.85574463	0.85854685	0.86096613	0.86338902	0.86648419	0.87047319	0.87559483	0.88072764	0.8855232	0.89017439	0.89398584	0.89755853	0.90132784	0.90493847	0.90832641	0.9117067	0.91423553	0.91597759
Vestfold og Telemark	0.79005183	0.79478011	0.79954847	0.80427272	0.80858043	0.81299535	0.81834269	0.82415252	0.82926935	0.83381208	0.83906515	0.8446608	0.84933615	0.85273725	0.85557901	0.85791226	0.85977761	0.86148978	0.8636156	0.86641602	0.87024156	0.87417044	0.87811767	0.88205685	0.8857214	0.88943605	0.89319456	0.8969139	0.90049229	0.90394682	0.90587119	0.90730742
Vestland	0.78979157	0.79490379	0.80037813	0.80603727	0.81138205	0.81649102	0.82216729	0.82788962	0.83291553	0.83734823	0.84276313	0.8486332	0.85347442	0.85716964	0.86051206	0.86339923	0.86592234	0.8684169	0.87168977	0.87581105	0.8800681	0.88446835	0.88859936	0.89264388	0.89625125	0.89984226	0.90370657	0.9073585	0.91064885	0.91367553	0.91595896	0.91759629
Viken	0.81190016	0.81635858	0.82107877	0.82592098	0.8304994	0.83501865	0.84003051	0.84517078	0.84968447	0.85377352	0.85847148	0.86327925	0.86714811	0.87006156	0.8726784	0.87484938	0.8764248	0.87673685	0.87764793	0.8798049	0.8831166	0.88654101	0.88986353	0.89322663	0.89630876	0.89929186	0.90253907	0.9056458	0.90854788	0.91122676	0.91323434	0.91469261
Portugal	0.59977776	0.60726945	0.61480808	0.62210118	0.62930639	0.63549876	0.64086453	0.6461929	0.65133599	0.65612067	0.66161085	0.66724715	0.67258394	0.67766341	0.68235293	0.6868871	0.6911588	0.69497485	0.6986046	0.7022234	0.70656355	0.71126289	0.71580408	0.71983699	0.72302128	0.72575622	0.72830085	0.73122553	0.73440295	0.73809048	0.74103738	0.74415185
San Marino	0.81324489	0.81828251	0.82278276	0.82738232	0.83197098	0.83676273	0.84138949	0.84586486	0.85047477	0.85533861	0.85969536	0.86374776	0.86568059	0.86752201	0.86928713	0.8709796	0.87265215	0.87430725	0.87599188	0.87770523	0.87944081	0.88119752	0.88292453	0.88459263	0.88535369	0.88469337	0.88447766	0.88455761	0.88513352	0.88620066	0.88686794	0.88800547
Spain	0.63667317	0.64413074	0.6515976	0.65863757	0.66532155	0.67157603	0.67708894	0.6824295	0.68749072	0.6922452	0.69705603	0.70172607	0.70630965	0.71067858	0.71488623	0.71871729	0.72197013	0.72521144	0.72941104	0.73395605	0.73835206	0.74209645	0.74518187	0.74765198	0.74980093	0.75226982	0.75475794	0.75766328	0.76065957	0.76394292	0.76650603	0.7692837
Sweden	0.78553579	0.79009644	0.79543146	0.80100952	0.80670026	0.81224465	0.81722703	0.82169406	0.82573199	0.8294871	0.83302811	0.8359564	0.83874153	0.84166314	0.84450688	0.84687634	0.84911587	0.85142085	0.85370585	0.85570275	0.85872794	0.86189238	0.86470667	0.86738377	0.86992333	0.87238851	0.87466165	0.87712061	0.87988891	0.88298412	0.88506201	0.8868803

Table S5. Socio-demographic Index values for all estimated GBD 2021 locations, 1990-2021

Location	1990	1991	1992	1993	1994	1995	1996	1997	1998	1999	2000	2001	2002	2003	2004	2005	2006	2007	2008	2009	2010	2011	2012	2013	2014	2015	2016	2017	2018	2019	2020	2021
Stockholm	0.82864929	0.83252503	0.83705641	0.84151191	0.84612539	0.85065475	0.85486826	0.85896218	0.86250724	0.86564484	0.86874746	0.87169989	0.87428166	0.87670657	0.8792564	0.88162691	0.88385143	0.88624949	0.88886385	0.89115289	0.8943043	0.89749399	0.90023896	0.9023454	0.90437172	0.90635999	0.90830839	0.91004256	0.9119315	0.91388968	0.91544818	0.91662271
Sweden except Stockholm	0.77412745	0.7788412	0.7843429	0.79015052	0.79604589	0.80177091	0.80686273	0.81133061	0.81539587	0.81921569	0.82278713	0.82565124	0.82844554	0.83145624	0.83433632	0.83666605	0.83885815	0.84106732	0.84317932	0.84498859	0.84785759	0.85089033	0.85358927	0.85616454	0.85858364	0.86086365	0.86292915	0.86530307	0.86803189	0.87118114	0.87330347	0.87515115
Switzerland	0.86276684	0.86479687	0.86773608	0.87086356	0.87314087	0.87483533	0.87664056	0.87848555	0.88003515	0.88204559	0.88524353	0.88844595	0.89090281	0.89278081	0.89490981	0.89727263	0.8999623	0.90291341	0.90577845	0.90805	0.91053052	0.91327657	0.91571489	0.91795501	0.91999418	0.92183299	0.9239306	0.92620229	0.92855059	0.9306821	0.93202764	0.93305911
UK	0.74433413	0.74914823	0.75455669	0.76015435	0.7651646	0.76865686	0.77157648	0.77505012	0.77905717	0.78403648	0.78921896	0.79351317	0.79665556	0.79918611	0.80195087	0.80448919	0.80681705	0.80935277	0.81203929	0.81479364	0.81803399	0.821719	0.82645375	0.83150867	0.83551664	0.83895192	0.84246023	0.84626362	0.84996979	0.85413057	0.85692029	0.85900018
England	0.74522081	0.7500107	0.75536208	0.76086151	0.76585217	0.76944555	0.77257153	0.77636961	0.7805758	0.78573764	0.79121817	0.79572874	0.79893122	0.80142212	0.80422916	0.80686705	0.80929741	0.81187828	0.8145103	0.81714915	0.82024807	0.82388806	0.82881473	0.83417598	0.83828785	0.8417623	0.84530113	0.84919211	0.8530158	0.85723951	0.85984675	0.86143731
East Midlands	0.72059559	0.72538219	0.73064677	0.73602802	0.7409704	0.74473198	0.74802908	0.75186528	0.75601731	0.76109291	0.76650495	0.77091617	0.77397647	0.7763029	0.77888155	0.78127626	0.78346778	0.78581163	0.78826944	0.79074877	0.79372244	0.79731194	0.80229019	0.8076191	0.81163509	0.81514094	0.8188665	0.82302766	0.82710738	0.83156984	0.83424814	0.83585605
Derby	0.72928646	0.73413347	0.74036291	0.74712417	0.75302847	0.75701379	0.75988357	0.76306436	0.76671602	0.77172695	0.77727355	0.78168517	0.78481718	0.78724388	0.78985701	0.79167985	0.79300453	0.79467539	0.79702261	0.80003206	0.8032148	0.80666595	0.81201513	0.81803361	0.82281919	0.82710299	0.83102614	0.83472001	0.83797194	0.84175987	0.84430589	0.84601068
Derbyshire	0.71332758	0.71762104	0.72273213	0.72795739	0.73260427	0.73597654	0.73890993	0.74231492	0.74595707	0.75073755	0.75576888	0.75958277	0.76190732	0.76326194	0.76493949	0.76673709	0.76862781	0.7704933	0.7720735	0.77373934	0.77647066	0.78049547	0.78658728	0.79317459	0.79811537	0.80233342	0.80655966	0.81107255	0.81535806	0.81982696	0.82255145	0.8242381
Leicester	0.70910048	0.71483792	0.72049984	0.72585692	0.73118958	0.73550854	0.73939507	0.74384626	0.74820112	0.75306515	0.75814382	0.76226968	0.76531609	0.76805804	0.77149691	0.77486092	0.77783426	0.78095923	0.78457021	0.78835353	0.79265757	0.79717113	0.80216128	0.80690263	0.81011027	0.81260416	0.81521784	0.81815553	0.8212361	0.82503059	0.82748417	0.8290671
Leicestershire	0.74960514	0.7544373	0.75936118	0.76424233	0.76853066	0.77194732	0.77510549	0.77859824	0.78222466	0.78664139	0.79142005	0.79524965	0.79788962	0.80013468	0.80299005	0.80592575	0.80876558	0.81154376	0.81400821	0.81584611	0.81780299	0.82019958	0.82408761	0.82853589	0.83164571	0.83422482	0.83734304	0.84095748	0.84444094	0.84821489	0.85046112	0.85188895
Lincolnshire	0.71327919	0.71737561	0.72171496	0.72645546	0.73105107	0.73448791	0.73750007	0.74106771	0.74496829	0.74981322	0.75489142	0.75899655	0.76164522	0.76338967	0.7651122	0.76660914	0.76770246	0.76860633	0.76969957	0.77149597	0.77416751	0.777765	0.78314061	0.78899952	0.79364411	0.79785562	0.80214629	0.80683445	0.81131396	0.81623843	0.81944532	0.82136376
Northamptonshire	0.73111547	0.73643078	0.74241386	0.74821903	0.75313512	0.75654172	0.75917524	0.76205477	0.76486547	0.76841545	0.77258434	0.77623332	0.77860274	0.78020194	0.78186061	0.78325157	0.78452215	0.78612642	0.78786248	0.78985085	0.79273203	0.79666712	0.8024164	0.80860972	0.81311018	0.81686085	0.82082775	0.82538893	0.82991887	0.83481513	0.83788898	0.83963536
Nottingham	0.73954936	0.74434723	0.74975203	0.75567582	0.76127025	0.76571848	0.76987001	0.77483316	0.78021001	0.78599733	0.79161737	0.79628495	0.79981123	0.80292014	0.80603745	0.80887743	0.81175174	0.8152604	0.81938004	0.82305858	0.82612397	0.82906958	0.83250605	0.83595844	0.83871921	0.84121217	0.84415762	0.8475535	0.85104215	0.85501249	0.85740749	0.85887136
Nottinghamshire	0.71531195	0.71968933	0.72460616	0.72961345	0.73429794	0.73777031	0.74078328	0.74447891	0.7485489	0.75345726	0.75850171	0.76236891	0.76487914	0.76661287	0.76879528	0.77084519	0.77256148	0.77435406	0.77595881	0.77744296	0.77962092	0.78274283	0.78752169	0.79283125	0.79688645	0.80049784	0.80445653	0.80905635	0.8136382	0.81843411	0.82131702	0.82300786
Rutland	0.76619252	0.76972339	0.77329705	0.77700464	0.78039162	0.78301858	0.78548548	0.78834767	0.79091409	0.79373654	0.79691224	0.79962497	0.80161127	0.8032843	0.80524775	0.80723545	0.80935123	0.81166631	0.81374273	0.8158297	0.81864541	0.82176025	0.82565923	0.82991478	0.83340509	0.83643048	0.83943789	0.84265611	0.84578287	0.84937926	0.8514898	0.85273375
East of England	0.74147823	0.74626637	0.75134567	0.75666071	0.76173949	0.76564098	0.76888928	0.77261216	0.77676624	0.78185797	0.78730323	0.79188721	0.79524007	0.79792144	0.80087581	0.80356092	0.80588557	0.80824658	0.81069862	0.8131753	0.81602737	0.81922047	0.8235436	0.82846015	0.83243345	0.83592015	0.83956349	0.84362321	0.8476727	0.85213271	0.85481968	0.85640792
Bedford	0.75154072	0.75643651	0.76112442	0.76602851	0.77067654	0.77422698	0.77774583	0.78174237	0.78578448	0.79042273	0.79524673	0.79924353	0.80228154	0.80485986	0.80756347	0.81013504	0.81235424	0.81404365	0.81552582	0.81756501	0.82027151	0.82355576	0.8279465	0.83263739	0.83637132	0.83976089	0.84320546	0.84698617	0.85060917	0.85442102	0.85666666	0.85809366
Cambridgeshire	0.7790098	0.78398518	0.78906669	0.79408017	0.79882102	0.80265243	0.8058381	0.80945881	0.81366605	0.81871415	0.82400557	0.82859093	0.83209721	0.83513843	0.83817458	0.84090443	0.84337313	0.84577472	0.84830744	0.85060155	0.85294263	0.85562805	0.85935958	0.86354669	0.86697755	0.87016403	0.87358348	0.87728827	0.88088434	0.88467525	0.88677226	0.88802758
Central Bedfordshire	0.74985197	0.75445555	0.75910647	0.76407086	0.76906202	0.77297579	0.77596436	0.77902984	0.78214723	0.78607818	0.79070657	0.79479039	0.7978689	0.80010626	0.80233225	0.80430673	0.80596054	0.80757382	0.80928668	0.81112697	0.81341284	0.81640747	0.82064807	0.82581232	0.83024547	0.83383725	0.8373315	0.84104542	0.84464343	0.84859672	0.85088571	0.85225452
Essex	0.73258243	0.73759316	0.74287631	0.74838448	0.75357441	0.75751889	0.76076077	0.76443018	0.76844495	0.77343901	0.77883546	0.78351507	0.78708095	0.79006216	0.79320559	0.79608436	0.7986483	0.80124219	0.80369954	0.80585803	0.80821737	0.81088406	0.81473743	0.81916366	0.82268804	0.82574806	0.82889356	0.8325995	0.83656553	0.8410991	0.84386727	0.84549145
Hertfordshire	0.781615	0.78606974	0.79094176	0.79610251	0.80106937	0.80495295	0.80819694	0.81189183	0.8160737	0.82114262	0.82676247	0.83186779	0.83583109	0.8389243	0.84205508	0.84460373	0.84659708	0.8483645	0.85003062	0.85160974	0.85379003	0.8564126	0.85998709	0.86411927	0.86754888	0.87067495	0.8739574	0.87745039	0.88076523	0.88429238	0.88629333	0.88751072
Luton	0.72045637	0.72555326	0.73073986	0.73636558	0.741806	0.74600248	0.74981492	0.75397631	0.75789874	0.76232555	0.76686617	0.7704984	0.77365374	0.77712462	0.78104911	0.7847005	0.78789807	0.79141464	0.79524194	0.79916157	0.80325852	0.80737134	0.81225534	0.81703972	0.82010678	0.82261018	0.8256479	0.82887828	0.83174543	0.83551944	0.83808561	0.83971713
Norfolk	0.72251977	0.72693238	0.73176345	0.73699042	0.7420267	0.74591973	0.74922801	0.75297926	0.75714266	0.76241538	0.76826306	0.77303409	0.77616366	0.7783889	0.78107126	0.78355972	0.78582226	0.78822315	0.79081153	0.79331269	0.79579735	0.79834074	0.80211798	0.80673339	0.81057724	0.81409921	0.81808582	0.82273758	0.82736907	0.83239679	0.83550271	0.83730968
Peterborough	0.72090611	0.72448672	0.72862923	0.73359124	0.7388356	0.74268802	0.74577596	0.74938571	0.75302914	0.75782605	0.76296228	0.76688204	0.76934652	0.7708413	0.77255259	0.77391286	0.77465406	0.77549251	0.7765108	0.7784663	0.78167115	0.7853315	0.79081991	0.79777602	0.80350336	0.8084507	0.81372439	0.81960737	0.82528317	0.83148331	0.83561547	0.83787447
Southend-on-Sea	0.71076864	0.71445575	0.71896386	0.72410162	0.72930133	0.73306595	0.7358702	0.73946743	0.74388285	0.74965635	0.75576757	0.76087273	0.76507208	0.76848403	0.7717417	0.77401582	0.77553406	0.77742574	0.77966746	0.78249494	0.78575933	0.78870666	0.79247893	0.79723967	0.8012837	0.80452588	0.80786245	0.8119344	0.81627964	0.82116516	0.82413703	0.82580139
Suffolk	0.72863521	0.73403869	0.73963033	0.74494139	0.74956172	0.7527291	0.75514542	0.7582659	0.76219203	0.76715892	0.7723517	0.77642029	0.77887141	0.78054771	0.78298291	0.78536666	0.7873079	0.78917872	0.79120836	0.79353145	0.79642961	0.79999049	0.80496748	0.81059607	0.81499207	0.8187038	0.82246061	0.82671827	0.83114002	0.83616767	0.83932516	0.84115103
Thurrock	0.71458309	0.71931345	0.72423301	0.72995803	0.7359403	0.74025707	0.74368404	0.7475592	0.75183529	0.75691649	0.7617984	0.76508055	0.76692578	0.76808568	0.76955836	0.77078143	0.77156193	0.77244608	0.77419036	0.77687307	0.78015505	0.78349176	0.78748853	0.79209418	0.79558483	0.79834589	0.80148926	0.80543057	0.80971971	0.81464239	0.81776351	0.81959772
Greater London	0.79481868	0.7998347	0.80520431	0.81057115	0.81546656	0.81912642	0.82245457	0.82638041	0.83046968	0.83521702	0.84030883	0.84470211	0.84808838	0.85094349	0.85408771	0.85702182	0.8598156	0.86280693	0.86555189	0.86835084	0.87160102	0.87543247	0.88013157	0.8848343	0.8881433	0.89057307	0.89296303	0.89553814	0.89802884	0.9008461	0.90328554	0.90486476
Barking and Dagenham	0.68400112	0.68851658	0.69405185	0.70052308	0.70701265	0.71171265	0.71545353	0.72007727	0.72505035	0.73052179	0.73572423	0.73971404	0.74214235	0.74335222	0.74435237	0.74487122	0.7453979	0.74677634	0.74881659	0.75143482	0.75443637	0.75814817	0.76424208	0.77143218	0.77682347	0.78108087	0.78561971	0.79100111	0.79622478	0.80168939	0.80525367	0.80732495
Barnet	0.78161285	0.78601238	0.79050668	0.79496134	0.79947041	0.80326453	0.80686318	0.81107866	0.81555993	0.82074288	0.82620045	0.83078806	0.83413865	0.83676581	0.83930163	0.84162286	0.84382766	0.84604543	0.84810945	0.85015497	0.85299744	0.85662641	0.86126516	0.86594832	0.86928202	0.8718356	0.87426116	0.87697559	0.87976099	0.88298601	0.8848172	0.88597606
Bexley	0.72575364	0.73122846	0.73681934	0.74231597	0.74726382	0.75091748	0.7540693	0.75763967	0.7613485	0.76601379	0.77138492	0.7762055	0.77995794	0.78293343	0.78604034	0.78878367	0.79118262	0.79371234	0.7962597	0.79854463	0.80093545	0.80412363	0.80903412	0.81463145	0.81944244	0.82388089	0.82819052	0.83243853	0.83639173	0.84046774	0.84293186	0.84454763
Brent	0.75459172	0.75873605	0.76343336	0.76877155	0.77390429	0.77770195	0.78124831	0.78559031	0.78987193	0.79478966	0.80006207	0.80431918	0.80731606	0.80980032	0.81252935	0.81474281	0.81656274	0.81819565	0.81939383	0.82072269	0.82331879	0.82693645	0.83211444	0.83775859	0.84167643	0.84433317	0.84667456	0.84928146	0.8521231	0.85596076	0.85856847	0.86016434
Bromley	0.76835343	0.77275209	0.77730136	0.78200834	0.7864666	0.78994873	0.79332161	0.79741178	0.80174169	0.80673101	0.81208574	0.81672076	0.82012981	0.82304021	0.82609268	0.82874628	0.83103164	0.8332419	0.83510496	0.83640616	0.8375565	0.83945508	0.84286939	0.8468273	0.8501393	0.85303116	0.85586052	0.85911733	0.86254855	0.8661867	0.86822346	0.86954533
Camden	0.86675758	0.87007384	0.87348791	0.87692945	0.88021241	0.88280208	0.88533046	0.88810116	0.89085206	0.89370705	0.89650609	0.89896685	0.90098518	0.90281368	0.90495797	0.90704259	0.90909842	0.91143591	0.91404634	0.91661255	0.91892516	0.92148464	0.92419541	0.92661573	0.92829456	0.92934758	0.93040276	0.9316126	0.93290834	0.93428657	0.93549264	0.93647483
Croydon	0.74915756	0.75447041	0.76013521	0.76560958	0.77045556	0.77391942	0.77681734	0.7804267	0.78439356	0.78928507	0.7948106	0.79944393	0.80265198	0.80493859	0.807574	0.80979112	0.81143552	0.81321221	0.81484408	0.8162574	0.81820851	0.82072449	0.82441292	0.82849933	0.83128042	0.83355712	0.83645535	0.84012104	0.84384323	0.84805561	0.85062101	0.85206634
Ealing	0.77644087	0.78078121	0.78568794	0.79092553	0.79588441	0.79991058	0.80373623	0.80804651	0.81250897	0.81758411	0.82289891	0.8273953	0.83078645	0.83324708	0.83559593	0.83738075	0.83869019	0.83989127	0.84093462	0.8419383	0.8437711	0.84693741	0.85188405	0.8574838	0.86198393	0.86574523	0.8692633	0.87281969	0.87611372	0.8795415	0.88149643	0.88272888
Enfield	0.73401506	0.7388007	0.74426686	0.74982052	0.7545119	0.75785849	0.76107907	0.76510467	0.76954459	0.7747313	0.78013717	0.78439149	0.78740562	0.78976075	0.79218572	0.79435668	0.79635243	0.79851456	0.80092507	0.80349802	0.80689946	0.8109965	0.81622114	0.82170996	0.8255965	0.82864724	0.83185869	0.8354004	0.83882577	0.84265274	0.84500303	0.846461
Greenwich	0.72479443	0.73056898	0.737057	0.74358501	0.74938775	0.75364686	0.75740294	0.76151525	0.76573266	0.77082124	0.77631967	0.78094286	0.78427594	0.78659955	0.78891732	0.79098356	0.79330042	0.79637723	0.79950247	0.80218477	0.8050603	0.80888263	0.81436808	0.82024314	0.8244898	0.82784916	0.83134099	0.83509882	0.83871121	0.84254074	0.84475097	0.84608019
Hackney	0.77285594	0.78014929	0.78804861	0.79484938	0.799757	0.80161743	0.8025406	0.80439112	0.80667073	0.81045905	0.81471258	0.8181534	0.82082866	0.82326581	0.82683393	0.83082455	0.83530327	0.84042971	0.84497184	0.84974099	0.85541438	0.86139314	0.86770897	0.87339639	0.87671631	0.87877768	0.88063149	0.88268841	0.8848806	0.88806219	0.89105735	0.89294628
Hammersmith and Fulham	0.84480445	0.84999613	0.85519526	0.86018324	0.86477984	0.86862595	0.8722719	0.87615072	0.88006375	0.88463422	0.88902354	0.89150181	0.89348866	0.89540412	0.89754032	0.89971064	0.90205138	0.90452785	0.90695206	0.90956889	0.91239215	0.91529824	0.9183679	0.92145253	0.92378413	0.92559759	0.92732396	0.92906332	0.93074204	0.93255592	0.93408915	0.93515317
Haringey	0.76203114	0.76673422	0.77166211	0.77638459	0.78046201	0.78331568	0.78609147	0.78970198	0.79342283	0.79774159	0.8026294	0.80732689	0.81104019	0.81404371	0.81731621	0.82032251	0.82282993	0.82525506	0.82736214	0.82926629	0.83224998	0.83617217	0.84079127	0.84556738	0.84921561	0.85238221	0.85596973	0.86014756	0.86423234	0.86845276	0.87078061	0.87208455
Harrow	0.76590342	0.77069264	0.77564635	0.78060053	0.7850939	0.7883951	0.79148835	0.79556512	0.80035525	0.80590543	0.81145076	0.81584836	0.81886292	0.82138838	0.82467462	0.82768907	0.83020534	0.83242872	0.83427321	0.83562057	0.83741689	0.83986909	0.84324814	0.84676932	0.84885397	0.84999922	0.85083322	0.85201227	0.85371187	0.85660861	0.8586377	0.86001527
Havering	0.7181706	0.72339777	0.72862237	0.73340787	0.73790641	0.74147993	0.74461862	0.74810964	0.75200598	0.75732907	0.76311541	0.76796775	0.77155728	0.77422303	0.77764239	0.78130919	0.78450011	0.78730519	0.79005801	0.79238045	0.79480725	0.79793974	0.80220709	0.8067285	0.81023208	0.81353735	0.81715744	0.82119701	0.82536884	0.82993939	0.83279668	0.83464387
Hillingdon	0.78360267	0.78858177	0.79412623	0.79947963	0.80434626	0.80797797	0.81116539	0.81509113	0.81976938	0.82537059	0.83113758	0.83593895	0.83958379	0.84203799	0.84417612	0.8459004	0.8473667	0.84901091	0.85104679	0.8538497	0.85691681	0.86043264	0.86496569	0.87020307	0.87423136	0.87692208	0.87948897	0.88235649	0.8852682	0.88848906	0.89126898	0.89313813
Hounslow	0.77774499	0.78277771	0.78797048	0.79308896	0.79776603	0.80104578	0.80372018	0.80741382	0.81198197	0.8178805	0.82420358	0.8291261	0.83268461	0.83526961	0.83797114	0.84048106	0.84263564	0.84484453	0.84698829	0.84887354	0.85138265	0.85509044	0.86064099	0.86695427	0.87206989	0.87641897	0.88066009	0.88493488	0.88885189	0.89300646	0.89553523	0.89699458
Islington	0.82973056	0.83453897	0.84007429	0.84599163	0.85134405	0.85520257	0.85840505	0.86142758	0.86428373	0.86761993	0.87150706	0.87508324	0.87763267	0.87984139	0.88237152	0.8849225	0.8876219	0.89057345	0.89372454	0.89718682	0.90071609	0.90419345	0.90750645	0.91031388	0.91232004	0.91404133	0.91575754	0.91763015	0.91961203	0.9217552	0.92352931	0.92474007
Kensington and Chelsea	0.87554948	0.87990911	0.88401687	0.88791932	0.89160978	0.89487695	0.89819944	0.90187835	0.90481233	0.90667107	0.90843189	0.91006559	0.91150955	0.91296531	0.9147092	0.91641701	0.91834677	0.9204801	0.92272821	0.92509751	0.92750215	0.93009406	0.93264645	0.93489989	0.93658587	0.93796648	0.93952173	0.94116257	0.94266851	0.94420575	0.94550614	0.9464767
Kingston upon Thames	0.81166499	0.81650631	0.82140929	0.82591313	0.83016335	0.83394691	0.83750788	0.84146924	0.84581094	0.85080176	0.85603353	0.86055739	0.86411828	0.86704239	0.87006145	0.87296388	0.87577009	0.8787043	0.88166979	0.88396299	0.88611596	0.88829931	0.89081257	0.893262	0.89490464	0.89636244	0.89828529	0.90080218	0.90347627	0.90637444	0.90780389	0.90869828
Lambeth	0.78759773	0.79358553	0.79955401	0.80524053	0.81038423	0.81426342	0.81784657	0.82227764	0.8268809	0.83201087	0.83752619	0.84236157	0.84614767	0.84958141	0.85362941	0.85770494	0.86183536	0.86616036	0.87025638	0.87409945	0.87835678	0.88282194	0.88775428	0.89275294	0.8967257	0.90014163	0.9034326	0.90685971	0.91016639	0.91356358	0.91535783	0.91638082
Lewisham	0.74150146	0.74663766	0.75223091	0.75791629	0.76303985	0.7669826	0.77034293	0.77421611	0.77846884	0.78354334	0.7892805	0.79422735	0.79790394	0.80066658	0.80332108	0.80553478	0.80769716	0.81006311	0.81249982	0.8149258	0.81793909	0.82158935	0.82660821	0.8321343	0.83639314	0.83983936	0.8432392	0.8468051	0.85023211	0.85395556	0.85607039	0.85730894
Merton	0.7741335	0.77926409	0.78451703	0.78993132	0.7950753	0.79920811	0.80298398	0.80759771	0.81252482	0.81785755	0.82330884	0.82786023	0.83118703	0.83348895	0.83576605	0.83767292	0.83947687	0.84182137	0.84448647	0.84673501	0.8492308	0.85250906	0.85734091	0.86259802	0.86664475	0.87005724	0.8733615	0.87673744	0.88013501	0.88413348	0.88650159	0.88773396

Table S5. Socio-demographic Index values for all estimated GBD 2021 locations, 1990-2021

Location	1990	1991	1992	1993	1994	1995	1996	1997	1998	1999	2000	2001	2002	2003	2004	2005
Newham	0.70179532	0.70643561	0.71172452	0.717249	0.72218856	0.72542304	0.72868579	0.7330763	0.73739294	0.74281997	0.74938282	0.75550038	0.76051169	0.7649267	0.76947164	0.77330625
Redbridge	0.7411182	0.74562564	0.75074485	0.7560224	0.76091812	0.76464643	0.7680517	0.772273	0.77680242	0.78183122	0.78723136	0.79189485	0.79547287	0.79841522	0.801404	0.80383152
Richmond upon Thames	0.83703281	0.84139175	0.84569853	0.84989642	0.85376138	0.85700329	0.8601784	0.86380732	0.86775948	0.87235908	0.87717294	0.88124833	0.88434753	0.88689502	0.88938374	0.89154436
Southwark	0.79790753	0.80392644	0.81013754	0.81609829	0.82154401	0.82584994	0.82976482	0.83445641	0.83964907	0.84554615	0.85185331	0.85723068	0.8612693	0.8647362	0.86753676	0.86990066
Sutton	0.74553969	0.75032286	0.75541399	0.76075214	0.76603767	0.77041068	0.7743114	0.77868675	0.78326047	0.78877511	0.79482141	0.79972559	0.80326486	0.80604838	0.80871934	0.81083275
Tower Hamlets	0.76510936	0.77171163	0.77896635	0.78615916	0.79257338	0.79732144	0.80206254	0.80783759	0.81367738	0.81971193	0.82560101	0.83049202	0.83477564	0.83914333	0.84429237	0.84934343
Waltham Forest	0.71666827	0.72145392	0.72705037	0.73312458	0.73892915	0.74340359	0.74738158	0.75179127	0.75626946	0.76142493	0.76692302	0.77158819	0.77497571	0.77763364	0.78052014	0.78297295
Wandsworth	0.8209162	0.82589432	0.83101038	0.83614442	0.84099764	0.84526143	0.84950543	0.85423898	0.85894473	0.86399287	0.86930362	0.87407677	0.87806733	0.88146394	0.88472489	0.88765211
Westminster	0.86095724	0.86466784	0.86896405	0.87316972	0.8769629	0.87997495	0.88294148	0.88594312	0.88857047	0.89119961	0.89395024	0.89655897	0.8986167	0.90046019	0.90251471	0.90464551
North East England	0.70561378	0.70969006	0.71510843	0.72115792	0.72662402	0.73056967	0.73413026	0.73851813	0.7433783	0.74916665	0.75517656	0.76000092	0.76344209	0.76610925	0.76900843	0.77181052
County Durham	0.69870267	0.70209493	0.70743793	0.71368685	0.71893279	0.72223749	0.72504864	0.72882138	0.73344258	0.73928988	0.74518594	0.74970548	0.75271731	0.75490689	0.75746944	0.76012681
Darlington	0.71374067	0.71720844	0.72252502	0.72866436	0.73414979	0.73770734	0.74034273	0.7433459	0.74677718	0.75201358	0.75775031	0.76164668	0.76418989	0.76635394	0.76952012	0.77273096
Gateshead	0.70759743	0.711675	0.71696653	0.72239918	0.72717075	0.73039026	0.73315504	0.73709505	0.74159011	0.74675429	0.75234321	0.75731376	0.76122134	0.76445465	0.76791895	0.77124027
Hartlepool	0.6761734	0.67952219	0.68397845	0.68891489	0.6937956	0.69792593	0.70171843	0.70538679	0.70878493	0.71319269	0.71845461	0.72282349	0.72562898	0.72730131	0.72916009	0.73082238
Middlesbrough	0.6864576	0.6905138	0.69509186	0.70022091	0.70530423	0.70908696	0.71234079	0.71640215	0.7205712	0.72577229	0.73196823	0.73688981	0.73963252	0.74149081	0.74415086	0.74749801
Newcastle upon Tyne	0.75197187	0.75685269	0.76215185	0.76752011	0.77259528	0.77690428	0.78115369	0.78608308	0.79124197	0.7965835	0.80202396	0.80722356	0.81229027	0.8171593	0.82195979	0.82625526
North Tyneside	0.71145049	0.71554531	0.72117566	0.72744509	0.73254419	0.73586823	0.73889513	0.7426041	0.74697149	0.75217304	0.757655	0.76226785	0.76571699	0.76863718	0.77204743	0.77565546
Northumberland	0.71178911	0.7149074	0.71927485	0.72458774	0.7296797	0.73325093	0.73626221	0.73982649	0.74382339	0.74912357	0.754331	0.7583374	0.76108912	0.76318611	0.76580619	0.76865151
Redcar and Cleveland	0.68303634	0.68580042	0.69039358	0.69621971	0.7018393	0.70576424	0.70909517	0.71321203	0.71757085	0.72275317	0.72772366	0.73125509	0.73339543	0.73446566	0.736414	0.73919644
South Tyneside	0.67454357	0.67854398	0.68371625	0.68935864	0.69439675	0.69802513	0.70147124	0.7057562	0.71040459	0.71604135	0.72199177	0.72691563	0.73052272	0.73358975	0.73708044	0.74023518
Stockton-on-Tees	0.71632095	0.72110951	0.72722835	0.7341021	0.74037253	0.74445209	0.74781541	0.75186257	0.75599085	0.7609368	0.76591902	0.76933421	0.77137674	0.77284083	0.77461364	0.77620877
Sunderland	0.69343691	0.69793136	0.70437117	0.71131964	0.71727147	0.72136011	0.72499526	0.72935751	0.7340164	0.73953845	0.74535827	0.74997041	0.75329407	0.75609381	0.75904144	0.76134892
North West England	0.72385302	0.72890951	0.73496005	0.74112905	0.74645965	0.74992915	0.7527917	0.75647748	0.76065051	0.76598297	0.7718241	0.77664303	0.77984929	0.78202154	0.7846043	0.78724829
Blackburn with Darwen	0.68238976	0.68573164	0.69000809	0.69500546	0.69969811	0.70263254	0.7053888	0.70887123	0.71278685	0.7180951	0.7238112	0.72831272	0.73147637	0.73371769	0.7364082	0.73904631
Blackpool	0.67525266	0.67992759	0.68518121	0.69017452	0.69459344	0.69710333	0.69907228	0.70250716	0.70682714	0.71250177	0.71846253	0.7224849	0.7237447	0.72343032	0.72460302	0.72670251
Bolton	0.70171733	0.70712331	0.71343732	0.71967045	0.72460435	0.72709563	0.7288027	0.73146441	0.73466247	0.73927543	0.74433186	0.74837426	0.75101369	0.75247887	0.75429027	0.7560104
Bury	0.71706448	0.72231565	0.72854172	0.73483739	0.74014542	0.74355079	0.74612714	0.74919134	0.75266293	0.75718499	0.76184501	0.76538935	0.7675506	0.76897756	0.77117702	0.77371005
Cheshire East	0.77167465	0.77617188	0.78136872	0.78659027	0.79132581	0.79480861	0.79763272	0.80089596	0.80432573	0.80897243	0.81418307	0.81855068	0.82158838	0.82352578	0.82576388	0.82778333
Cheshire West and Chester	0.75910231	0.76444865	0.77069595	0.77671578	0.78165317	0.78473812	0.78726768	0.79077266	0.79481591	0.79979384	0.80497692	0.80935167	0.81256748	0.81471177	0.8171595	0.81989868
Cumbria	0.72330224	0.72694566	0.73230522	0.73837222	0.74381555	0.7470524	0.74931814	0.75256176	0.75627073	0.76088571	0.76598984	0.77009799	0.77249706	0.77399634	0.77645186	0.77891592
Halton	0.70416202	0.70900064	0.71531612	0.72221838	0.7283098	0.73223908	0.73471428	0.73672702	0.73920701	0.74389495	0.7494757	0.75427543	0.757266	0.75848716	0.76002317	0.76228671
Knowsley	0.68322195	0.68799747	0.69378334	0.69889168	0.70311744	0.70617118	0.70947554	0.71368969	0.71749449	0.72218463	0.72871711	0.7351277	0.73911406	0.74111117	0.7432513	0.74633275
Lancashire	0.72795698	0.73359125	0.7401154	0.74654341	0.75195713	0.75532674	0.75792822	0.76131954	0.76507588	0.76985061	0.77491484	0.77909261	0.78159083	0.78300964	0.78514659	0.78768313
Liverpool	0.72546732	0.73147594	0.73760085	0.74330529	0.74805739	0.75138035	0.7549318	0.75970248	0.76517695	0.77130966	0.77769986	0.78344546	0.78812525	0.79246909	0.79693049	0.8010914
Manchester	0.74664761	0.75170562	0.75792519	0.76465168	0.77085982	0.77544199	0.77948319	0.7844125	0.79004386	0.79672932	0.80363785	0.80955397	0.81403795	0.81762344	0.82152632	0.82561957
Oldham	0.68065982	0.68488108	0.69047993	0.69653542	0.70102578	0.70310367	0.7046846	0.70700472	0.70939649	0.71317467	0.71780555	0.7214547	0.72403929	0.725877	0.72826509	0.73060863
Rochdale	0.68249515	0.686915	0.6927403	0.69886136	0.70449136	0.70786881	0.71010576	0.7131785	0.716891	0.72222794	0.72799553	0.73223349	0.73485662	0.73671113	0.73893372	0.74103171
Salford	0.70774013	0.71214562	0.71839073	0.72541104	0.73164119	0.73545503	0.737696	0.74074545	0.7456012	0.75253971	0.75973452	0.76548316	0.76987678	0.77320434	0.77643396	0.77950527
Sefton	0.71875043	0.72468521	0.73131607	0.73748007	0.74259591	0.74592269	0.74851159	0.75198556	0.75607325	0.76080362	0.76577516	0.769984	0.7730009	0.77556717	0.77886283	0.78178457
St Helens	0.69423368	0.69856037	0.70440511	0.71074052	0.71592096	0.71879406	0.72104531	0.72395752	0.72747365	0.73262009	0.73858007	0.74341145	0.74625448	0.74828049	0.75107331	0.75341307
Stockport	0.75162097	0.75700283	0.76316114	0.76904994	0.77389445	0.77700409	0.77942944	0.78265055	0.78649322	0.79141721	0.79690384	0.80148098	0.80477528	0.80704286	0.80934283	0.81152974
Tameside	0.69016982	0.69496595	0.70111759	0.70761022	0.71323014	0.71688948	0.72006188	0.72372026	0.72737693	0.73219299	0.73750282	0.74192775	0.74498667	0.7468947	0.74909784	0.75065637
Trafford	0.78197684	0.78671279	0.79218509	0.79785767	0.80283818	0.80630337	0.80907728	0.81221729	0.81584601	0.82081479	0.82635424	0.83102853	0.83470571	0.8377655	0.84100298	0.84381435
Warrington	0.75742522	0.76252441	0.76849276	0.77419985	0.77886459	0.78160568	0.78369463	0.78623013	0.78928744	0.79429724	0.80042762	0.80552801	0.80924165	0.81229377	0.81576474	0.81900113
Wigan	0.69385144	0.69826532	0.70397237	0.71003069	0.7151698	0.71830682	0.72096965	0.72421672	0.72723054	0.73126173	0.73656244	0.74105777	0.7435692	0.74472141	0.74655518	0.74906725
Wirral	0.70650643	0.71129916	0.71684603	0.7225839	0.72788925	0.73147166	0.73427898	0.73750223	0.74084384	0.74522038	0.75033765	0.75447106	0.75693217	0.75855582	0.76094516	0.76333047
South East England	0.7727906	0.77724496	0.78193906	0.78672868	0.7911516	0.79454658	0.79751112	0.80101641	0.80489727	0.80959197	0.81453592	0.81866875	0.82173457	0.82417183	0.82676548	0.82899217
Bracknell Forest	0.78835599	0.79237692	0.79636294	0.80020199	0.80372606	0.80649403	0.80923457	0.81280661	0.81708518	0.82244951	0.82807357	0.83272796	0.8361016	0.83862188	0.8412608	0.84347634
Brighton and Hove	0.79297918	0.79769068	0.80228916	0.80699432	0.8115921	0.81571898	0.81963282	0.82382299	0.82813446	0.83280619	0.83775655	0.8424023	0.84643095	0.85007394	0.85368981	0.8569527
Buckinghamshire	0.79566701	0.79972445	0.80415347	0.80856633	0.81271282	0.8160479	0.81914725	0.82274582	0.82637017	0.83064768	0.8352728	0.83909176	0.84187946	0.84408786	0.84643126	0.84829935
East Sussex	0.72956397	0.73423933	0.7390553	0.74394721	0.74875844	0.75252344	0.75539866	0.75871242	0.76253885	0.76734963	0.77251839	0.77674968	0.77982055	0.78224736	0.78489792	0.78706102

Location	2006	2007	2008	2009	2010	2011	2012	2013	2014	2015	2016	2017	2018	2019	2020	2021
Newham	0.77643043	0.7797725	0.78301783	0.78655411	0.79145262	0.79744619	0.80471522	0.81192357	0.81712044	0.82111356	0.82500948	0.82916684	0.83304735	0.83732911	0.84006801	0.84168813
Redbridge	0.80618249	0.80887257	0.81103299	0.81247961	0.81454633	0.81772521	0.82253196	0.82777521	0.83155791	0.83436611	0.8371229	0.84019408	0.84320787	0.84679322	0.84896208	0.85026508
Richmond upon Thames	0.89359517	0.89581427	0.89795278	0.89959898	0.90163792	0.9043754	0.90773199	0.91139663	0.91465004	0.91772744	0.92077201	0.9239145	0.92695264	0.92995653	0.93132447	0.93213255
Southwark	0.87252757	0.87568665	0.87926389	0.88306436	0.8870974	0.8916206	0.89642638	0.9007784	0.90380911	0.90606481	0.90835988	0.9109518	0.91345716	0.91605289	0.91816468	0.91950983
Sutton	0.8126601	0.81467805	0.8167627	0.81875456	0.82118182	0.82433217	0.82895974	0.83413145	0.83836008	0.84190393	0.84524533	0.84853633	0.85157254	0.85503627	0.85711221	0.85842158
Tower Hamlets	0.85398229	0.85841338	0.86236273	0.86667264	0.87145478	0.87624438	0.88124895	0.88603276	0.88944824	0.89195975	0.89427139	0.89666401	0.89879702	0.90105103	0.90308201	0.90448814
Waltham Forest	0.78490751	0.78678602	0.78857743	0.79042113	0.79305762	0.79682894	0.80251966	0.80910434	0.81450239	0.81900025	0.82330467	0.82778592	0.83204346	0.83644284	0.83902216	0.8405096
Wandsworth	0.89051156	0.89344408	0.89620639	0.8983738	0.90052498	0.90286992	0.90572514	0.9088197	0.91129499	0.91346733	0.9157198	0.91806224	0.92033556	0.922739	0.92370388	0.92424149
Westminster	0.90703524	0.90966241	0.91235581	0.91472829	0.9164292	0.91839146	0.92113793	0.92391496	0.92595501	0.92756193	0.92927966	0.93114166	0.93286599	0.93462227	0.93611797	0.93719863
North East England	0.77460176	0.77761649	0.78049179	0.78306961	0.78615205	0.79003667	0.79540193	0.80107439	0.80511943	0.80829669	0.81151855	0.81514928	0.8188021	0.82310774	0.82571259	0.82727303
County Durham	0.76250071	0.76518332	0.76809846	0.77067482	0.77361282	0.77744124	0.78260244	0.78797616	0.79160399	0.79445489	0.79755543	0.80101293	0.80429708	0.80820503	0.81071906	0.81227652
Darlington	0.77560942	0.77801142	0.77963178	0.78159551	0.7852023	0.79002344	0.79688458	0.80457829	0.8103757	0.81514191	0.81963678	0.82406911	0.82807476	0.83254584	0.83546751	0.83720409
Gateshead	0.77429699	0.77713292	0.77945133	0.78248103	0.78714876	0.79238245	0.79833724	0.80419905	0.80822169	0.8114731	0.81513915	0.8192299	0.82258115	0.82623236	0.82866343	0.83024418
Hartlepool	0.73313271	0.73627497	0.73904738	0.7413062	0.74413561	0.74873543	0.75654915	0.76493273	0.77058336	0.77476087	0.77898656	0.78363469	0.78841263	0.79367659	0.796959	0.79887777
Middlesbrough	0.75101106	0.754776	0.75789713	0.75984869	0.76201042	0.76535148	0.77078309	0.77641283	0.78004115	0.78249349	0.78498498	0.78812686	0.7915783	0.79618331	0.79943482	0.801471
Newcastle upon Tyne	0.8305273	0.835104	0.83961735	0.84325483	0.84616954	0.84904908	0.85248202	0.85585328	0.85836593	0.86022435	0.86188351	0.86398528	0.86641485	0.86947394	0.87130709	0.87252066
North Tyneside	0.77932008	0.78244648	0.7849722	0.78743786	0.79083703	0.79534912	0.80150615	0.80783385	0.81251038	0.8164165	0.82009684	0.82406908	0.82804409	0.83229832	0.83483439	0.8364006
Northumberland	0.7714102	0.77387572	0.77586278	0.77786207	0.78077744	0.78431666	0.78967859	0.79558811	0.79963404	0.80313107	0.8067701	0.81060907	0.8144867	0.81902426	0.82177669	0.82331701
Redcar and Cleveland	0.74231865	0.74511908	0.74720774	0.7486004	0.75059426	0.75444991	0.76139509	0.76923425	0.77451559	0.77772875	0.78075988	0.78427063	0.78810445	0.79310696	0.79644544	0.79836962
South Tyneside	0.74276091	0.74551789	0.7484208	0.75083025	0.75371171	0.75781144	0.76391028	0.77075899	0.77581579	0.77984432	0.78380594	0.78798301	0.79201587	0.79652902	0.79932541	0.80107303
Stockton-on-Tees	0.77816682	0.78018326	0.78180059	0.78396922	0.78735343	0.79176717	0.79783644	0.80411822	0.80850829	0.81170065	0.8149351	0.81842686	0.82187762	0.8263084	0.82931055	0.83098489
Sunderland	0.76372052	0.76661445	0.76961527	0.77241229	0.77550064	0.77947828	0.78497747	0.7907802	0.79506434	0.79839481	0.80167355	0.80548224	0.80953853	0.81426814	0.81730961	0.81916413
North West England	0.78980914	0.79258226	0.79540342	0.79826012	0.80162241	0.8055136	0.81076052	0.81647096	0.82080593	0.82440582	0.82800597	0.83195251	0.83582963	0.84018255	0.84284778	0.84444382
Blackburn with Darwen	0.74185751	0.74468719	0.74684093	0.74945207	0.75378134	0.75912251	0.76671182	0.77546956	0.78201727	0.78691731	0.79123695	0.79548674	0.80005974	0.80572532	0.80942581	0.81150374
Blackpool	0.72885299	0.73089932	0.73281453	0.73507899	0.73802993	0.74142686	0.74684695	0.75326273	0.75816527	0.76236711	0.76695876	0.7723135	0.77759512	0.78355735	0.78758585	0.78992589
Bolton	0.75773083	0.75934402	0.76056701	0.76243759	0.76605381	0.77079774	0.77736255	0.78433391	0.78911719	0.79270227	0.79629417	0.80056448	0.80471298	0.80930634	0.81234076	0.81417076
Bury	0.77584538	0.77782938	0.77943043	0.78091871	0.78349505	0.78759983	0.79381521	0.80071533	0.80604363	0.81029901	0.81420225	0.81815505	0.82186126	0.82601912	0.8285613	0.83008916
Cheshire East	0.82983807	0.83213362	0.83433343	0.83635945	0.83892111	0.84231857	0.8475738	0.85365725	0.85839506	0.86253848	0.86663733	0.87119795	0.87582037	0.88048343	0.88319117	0.88475463
Cheshire West and Chester	0.8227027	0.82579129	0.82895026	0.83141617	0.83380251	0.83677423	0.84138353	0.84658544	0.85069508	0.85413584	0.85743423	0.86079497	0.86387828	0.86750888	0.8697802	0.87117793
Cumbria	0.78125837	0.7838395	0.78657279	0.78952738	0.79311674	0.79736644	0.80368699	0.81079256	0.81632317	0.82112445	0.82576807	0.83045631	0.8347251	0.83908273	0.84177636	0.8434669
Halton	0.7652617	0.76862377	0.77195658	0.77531719	0.77925367	0.78347154	0.78922992	0.79624839	0.80225792	0.80746561	0.81277136	0.81855164	0.82414361	0.8302224	0.83409953	0.83618135
Knowsley	0.74965489	0.75322783	0.75728608	0.76104959	0.76431571	0.76828803	0.77423718	0.78060947	0.78545745	0.78948257	0.79340886	0.79768751	0.80211415	0.80747793	0.81132351	0.81367171
Lancashire	0.79007049	0.79255696	0.79530341	0.79841727	0.80204457	0.80607239	0.81109734	0.81618565	0.81983333	0.82299312	0.82627369	0.82984765	0.83332213	0.83721137	0.83961368	0.84112554
Liverpool	0.80546792	0.81013324	0.81468745	0.81830189	0.8210977	0.82391341	0.82742119	0.83094411	0.8333797	0.83509089	0.83692164	0.83912808	0.84144421	0.84478329	0.84703646	0.84843764
Manchester	0.82988392	0.83485281	0.84025763	0.8451014	0.84898639	0.85230152	0.85607922	0.86006397	0.86321152	0.86598442	0.86882049	0.87190682	0.87483461	0.8781406	0.88015764	0.88142914
Oldham	0.73277793	0.7350019	0.73623757	0.73819361	0.7428259	0.74898586	0.75669133	0.76466026	0.77025821	0.77412929	0.77767871	0.78214086	0.78694605	0.7925242	0.79625616	0.79840829
Rochdale	0.74251341	0.74411545	0.74570559	0.74777554	0.75157152	0.75679946	0.76405722	0.77178826	0.77720887	0.78100561	0.78440606	0.7881374	0.79199254	0.79681444	0.80011008	0.80206932
Salford	0.78257535	0.78569202	0.78908628	0.79257324	0.79591883	0.79940812	0.80447952	0.8101548	0.81395997	0.8169183	0.82054222	0.82478679	0.82891253	0.83367174	0.83684926	0.83869917
Sefton	0.78411247	0.7862983	0.78800487	0.78942336	0.79119608	0.79306048	0.79621548	0.80046564	0.80399812	0.80709585	0.81013867	0.81368371	0.81753595	0.82186386	0.82438642	0.8258419
St Helens	0.75551871	0.75813763	0.76087717	0.7639785	0.7676737	0.7717539	0.7772925	0.78357098	0.78804636	0.7911898	0.79421081	0.79810586	0.80250587	0.80740478	0.81042007	0.8122146
Stockport	0.8135672	0.81570169	0.81761666	0.81944177	0.82200484	0.82523717	0.83001618	0.83560134	0.83997996	0.84362522	0.84733211	0.85117696	0.85477669	0.85849273	0.86052225	0.86171971
Tameside	0.75143437	0.75236238	0.75311745	0.75440437	0.75689691	0.76046529	0.76633805	0.77303027	0.77791402	0.7815795	0.78478989	0.78811133	0.79136903	0.79573372	0.79874919	0.80054669
Trafford	0.84621897	0.84832994	0.84982075	0.85138938	0.85409001	0.85750281	0.86222785	0.86778532	0.87254436	0.87687609	0.88101739	0.88520163	0.88929246	0.89332238	0.89550613	0.89675378
Warrington	0.82121982	0.82299003	0.8246727	0.82680404	0.83044779	0.83511681	0.84105702	0.84739354	0.85249578	0.85701058	0.86150674	0.86594896	0.87009959	0.87459779	0.87725742	0.87875621
Wigan	0.75146393	0.75306437	0.75426927	0.75639054	0.76019588	0.76518481	0.77141251	0.77745707	0.78178146	0.78549911	0.78944013	0.79391114	0.79819132	0.80256961	0.80520008	0.80685214
Wirral	0.76548729	0.76751363	0.76941677	0.77131613	0.77392955	0.77743861	0.78305632	0.78956171	0.79458561	0.79886987	0.80294669	0.80722854	0.81120829	0.81532945	0.81778247	0.81931629
South East England	0.83094146	0.83305352	0.83517101	0.83719356	0.8396068	0.84249881	0.84653195	0.85107907	0.85477468	0.85812498	0.86158042	0.8652482	0.86877208	0.8725336	0.87472821	0.8760666
Bracknell Forest	0.8451873	0.84672589	0.84814235	0.84972147	0.8520067	0.85497518	0.85932558	0.86439815	0.8686428	0.87262318	0.87661109	0.88053256	0.8842129	0.88784011	0.88981581	0.89100266
Brighton and Hove	0.86005652	0.86318119	0.8662899	0.86900759	0.87172474	0.87446877	0.87750885	0.88060818	0.88320914	0.88556575	0.88804263	0.89074714	0.89337716	0.89605158	0.89732773	0.8982572
Buckinghamshire	0.84960667	0.85079262	0.85178781	0.85267416	0.85417882	0.85667491	0.86062679	0.86508993	0.86866959	0.87183366	0.87501242	0.87851956	0.88207819	0.88571458	0.88762548	0.88878524
East Sussex	0.78873477	0.79040214	0.79190036	0.79330811	0.79559561	0.79867364	0.80327563	0.80874454	0.81334036	0.81759223	0.82199993	0.82652316	0.83077296	0.83531134	0.83800984	0.83959479

Table S5. Socio-demographic Index values for all estimated GBD 2021 locations, 1990-2021

Location	1990	1991	1992	1993	1994	1995	1996	1997	1998	1999
Hampshire	0.77057515	0.77510157	0.77952905	0.78406633	0.78821479	0.79130303	0.79409376	0.79733912	0.80099523	0.80576507
Isle of Wight	0.70872398	0.71354308	0.71844799	0.72344772	0.72836756	0.73219927	0.73586797	0.74031316	0.7447326	0.74975351
Kent	0.73784225	0.74236173	0.7473738	0.75252731	0.75722404	0.76051329	0.76305068	0.76633313	0.77029105	0.77521338
Medway	0.71232182	0.71728112	0.72243867	0.72800876	0.73330279	0.73683364	0.7396607	0.74312831	0.74649024	0.75053659
Milton Keynes	0.77322075	0.7783318	0.78379735	0.7892451	0.79413698	0.79737607	0.79999672	0.80314766	0.80633484	0.81043299
Oxfordshire	0.79787948	0.80199145	0.80653596	0.81125977	0.81568051	0.81911236	0.82189667	0.82513394	0.82885011	0.83340747
Portsmouth	0.75983848	0.76459062	0.76968377	0.7751214	0.78022393	0.78449206	0.78862431	0.79332513	0.79795438	0.80271548
Reading	0.80819077	0.812818	0.81760133	0.82225255	0.82641374	0.82985922	0.83324972	0.83737308	0.84188345	0.84726899
Slough	0.77261453	0.77785295	0.78334851	0.78910534	0.79477329	0.79910723	0.80300987	0.80753105	0.81190104	0.81595741
Southampton	0.75913149	0.76418419	0.7695829	0.77535742	0.78077252	0.78522846	0.78950037	0.79443109	0.79948635	0.80482542
Surrey	0.80924177	0.8132188	0.81740927	0.82159771	0.82534758	0.82857438	0.83162533	0.83505683	0.83908377	0.84417746
West Berkshire	0.80555006	0.81045271	0.81574416	0.82092884	0.82532709	0.82837042	0.83079633	0.83374033	0.83724333	0.84179016
West Sussex	0.76207193	0.76674263	0.77167331	0.77648278	0.78072689	0.78381572	0.78640868	0.78959461	0.79335943	0.79806633
Windsor and Maidenhead	0.81814087	0.82161379	0.82533514	0.82930067	0.83310046	0.83615734	0.83881499	0.84205662	0.84576499	0.85032079
Wokingham	0.82484241	0.82902045	0.83302523	0.83697185	0.84070109	0.8438461	0.84679256	0.85028599	0.85408238	0.8584432
South West England	0.74794325	0.75283036	0.75783934	0.76293065	0.76765317	0.77118217	0.77424567	0.77803072	0.78243064	0.78787031
Bath and North East Somer	0.78741038	0.79167943	0.79606208	0.80068624	0.80515302	0.80885624	0.81209959	0.81597108	0.82046427	0.82548323
Bournemouth	0.75527341	0.76090478	0.76639109	0.77158988	0.77644777	0.78041867	0.78395412	0.78817566	0.79312978	0.7988344
Bristol, City of	0.78461085	0.78956354	0.794632	0.79989191	0.80492318	0.80900577	0.81269256	0.81700844	0.8216647	0.8269735
Cornwall	0.72335353	0.72880769	0.73448938	0.74031754	0.74528742	0.74856818	0.75117673	0.7545459	0.75868446	0.76419209
Devon	0.74413819	0.74903733	0.75394429	0.75887588	0.76328689	0.76630701	0.76895614	0.77250424	0.77676433	0.78217921
Dorset	0.74099453	0.74558531	0.75020683	0.75487434	0.75929161	0.76232776	0.76506023	0.76886593	0.77332645	0.77857711
Gloucestershire	0.76125096	0.76617735	0.77101677	0.77579515	0.78026287	0.78375104	0.78673673	0.79000974	0.79368743	0.79864308
North Somerset	0.74308392	0.74817698	0.75325752	0.75815137	0.76236807	0.76532543	0.76784105	0.77078733	0.77418301	0.77890552
Plymouth	0.73401315	0.73892965	0.74446706	0.7500998	0.75513172	0.75873207	0.76162238	0.76536625	0.76978392	0.77500881
Poole	0.75345223	0.75793914	0.76263313	0.76763787	0.77265346	0.77658766	0.77989236	0.78365508	0.78787276	0.7932336
Somerset	0.72981338	0.73466335	0.73987199	0.74526688	0.75023763	0.75366498	0.75629182	0.75935715	0.76301507	0.76791049
South Gloucestershire	0.77431501	0.77808284	0.78204776	0.78610998	0.78999421	0.7932856	0.79685437	0.80130492	0.80596664	0.81130214
Swindon	0.76333747	0.7689086	0.77445484	0.77979149	0.78451823	0.78746216	0.78949609	0.79224357	0.79562141	0.8002938
Torbay	0.7082129	0.71297099	0.71801047	0.72306183	0.72740408	0.73027304	0.73291066	0.73637527	0.74010756	0.74515311
Wiltshire	0.7511355	0.75545116	0.75988469	0.76442761	0.76889338	0.77224644	0.7748818	0.77814747	0.78204754	0.78723597
West Midlands	0.71222975	0.71729482	0.72299146	0.72877167	0.73384891	0.737083	0.73977625	0.74345791	0.74777658	0.75337682
Birmingham	0.71191805	0.71694157	0.72279039	0.72846113	0.73352236	0.73663593	0.73909568	0.74275126	0.74712647	0.75264774
Coventry	0.73083578	0.73669449	0.74263342	0.74870933	0.75436286	0.75822896	0.76168375	0.765997	0.77044098	0.77569627
Dudley	0.69868763	0.70269302	0.70743603	0.71248446	0.7167845	0.71963497	0.72233017	0.72589569	0.72971007	0.73441491
Herefordshire, County of	0.72286053	0.72801213	0.73390025	0.73995465	0.74529661	0.74891853	0.75192271	0.75559809	0.75991016	0.76562347
Sandwell	0.67477782	0.67888694	0.6842572	0.69025023	0.69529774	0.69806787	0.7004652	0.70399955	0.70780531	0.71287955
Shropshire	0.72909223	0.73397894	0.73932429	0.7444821	0.74905725	0.75192111	0.75420363	0.75758641	0.76175938	0.76723723
Solihull	0.75697799	0.76233615	0.76833255	0.77396156	0.77852488	0.78157575	0.7844943	0.78825403	0.79243076	0.7978239
Staffordshire	0.7235126	0.72890498	0.7349188	0.74098518	0.74579796	0.7485293	0.75086635	0.75409291	0.75817765	0.76361874
Stoke-on-Trent	0.68271787	0.68822388	0.6937868	0.69918133	0.70424865	0.70753277	0.71012029	0.71376595	0.71762583	0.72227484
Telford and Wrekin	0.71634804	0.72103108	0.72651593	0.73227261	0.73775608	0.74150181	0.74465784	0.74835689	0.75203452	0.75702405
Walsall	0.67868011	0.68177893	0.68645491	0.69220826	0.69683339	0.69884761	0.69953053	0.70106263	0.70401782	0.70898028
Warwickshire	0.75308534	0.75870583	0.76423581	0.76922466	0.77381449	0.77715759	0.78000855	0.78351569	0.78751949	0.7927207
Wolverhampton	0.69213366	0.69683426	0.70263252	0.70916594	0.71513495	0.71829962	0.72030557	0.7235514	0.72769262	0.73356159
Worcestershire	0.73175699	0.73646861	0.74149889	0.74666706	0.75116551	0.75415749	0.7568413	0.76053245	0.76511163	0.77122539
Yorkshire and the Humber	0.71744891	0.72194235	0.72722402	0.73271523	0.7376591	0.74109023	0.74421366	0.74803919	0.75224885	0.75756466
Barnsley	0.67440928	0.67729761	0.68214309	0.68770182	0.69211715	0.69445175	0.69706907	0.70110824	0.70552609	0.71064237
Bradford	0.69577717	0.69972324	0.70442799	0.70936894	0.71410483	0.7170709	0.71915714	0.72120309	0.72301763	0.72644198
Calderdale	0.71758382	0.72220176	0.72779437	0.73349803	0.73824296	0.74088839	0.74300182	0.74606725	0.74953405	0.75430504
Doncaster	0.67510562	0.67843429	0.68299625	0.68802227	0.69246382	0.69495632	0.69678601	0.69942385	0.70313663	0.70829183

Location	2000	2001	2002	2003	2004	2005	2006	2007	2008	2009	2010
Hampshire	0.81089733	0.81509293	0.81801107	0.82011123	0.82238382	0.82439782	0.8261282	0.82804709	0.82987976	0.83163608	0.83398175
Isle of Wight	0.7551791	0.75993144	0.76322741	0.76519787	0.76719031	0.76904815	0.77124374	0.77385158	0.77629632	0.77820529	0.78018829
Kent	0.78031289	0.7844356	0.78744966	0.78971696	0.79214864	0.79413323	0.79595689	0.7979821	0.79995811	0.80200709	0.80478083
Medway	0.75510823	0.75877207	0.76162445	0.76420479	0.76713692	0.76942453	0.77118967	0.77297525	0.77468939	0.7766228	0.77942113
Milton Keynes	0.8150882	0.81897639	0.8218732	0.82421027	0.82689155	0.8289722	0.83033809	0.83142131	0.83226545	0.83339458	0.83547402
Oxfordshire	0.83835575	0.84271664	0.84599586	0.84859294	0.8511909	0.8535381	0.85566033	0.85804485	0.86054188	0.8627231	0.8649828
Portsmouth	0.8076178	0.81199062	0.81542911	0.81822759	0.82090657	0.82322772	0.82545209	0.82805508	0.83115236	0.83416247	0.83671976
Reading	0.85293744	0.85784299	0.8618166	0.8650803	0.86823015	0.87057453	0.87185217	0.87366865	0.87611377	0.87822923	0.87904761
Slough	0.81900265	0.8214746	0.82330758	0.82451084	0.8257586	0.82621675	0.82598375	0.82585896	0.82615659	0.82804461	0.83135486
Southampton	0.81015023	0.81475129	0.81851083	0.82164415	0.82455951	0.82693701	0.82906817	0.83136861	0.83384975	0.83565154	0.83688952
Surrey	0.84950138	0.85398569	0.85728936	0.85994151	0.8624662	0.86445316	0.86607386	0.86788482	0.86970367	0.87132598	0.8732674
West Berkshire	0.84681987	0.85087726	0.85347602	0.85414895	0.85477897	0.85482124	0.85439969	0.85404108	0.85425028	0.85578691	0.85834418
West Sussex	0.80303899	0.80727905	0.81031956	0.81248303	0.81496096	0.81707191	0.81883466	0.82058725	0.82211726	0.82358414	0.82590089
Windsor and Maidenhead	0.85526134	0.85947554	0.86298159	0.8660002	0.86886598	0.87117198	0.87301075	0.87488871	0.87664683	0.87802149	0.87989865
Wokingham	0.86306258	0.86710861	0.8704076	0.87311457	0.87589429	0.87834707	0.88028486	0.88213974	0.88400656	0.88525218	0.88610988
South West England	0.79343697	0.79786834	0.80104634	0.80368179	0.80652576	0.80900425	0.8111514	0.81334646	0.81563201	0.81778862	0.82044909
Bath and North East Somer	0.83075085	0.83584502	0.84050575	0.84482246	0.8487305	0.85221763	0.85568901	0.85938525	0.86296555	0.86555203	0.86777709
Bournemouth	0.8048075	0.81011008	0.8145398	0.81852419	0.82241845	0.82560447	0.82841321	0.8313547	0.83434427	0.83682931	0.83898966
Bristol, City of	0.83222633	0.83658094	0.8398284	0.84242176	0.84513932	0.8478601	0.8506721	0.85393064	0.85767376	0.86085072	0.86339527
Cornwall	0.76994659	0.77429251	0.77761354	0.78045262	0.78339497	0.78573839	0.78780296	0.78975369	0.79148465	0.79322605	0.7954772
Devon	0.7877467	0.79236557	0.79589158	0.79892481	0.80224409	0.80541911	0.80820232	0.81077568	0.81318568	0.81530465	0.81780667
Dorset	0.7835336	0.78734836	0.79016348	0.79235565	0.79467044	0.79649652	0.79779012	0.79882781	0.79984405	0.80141766	0.8044968
Gloucestershire	0.80408717	0.80862767	0.81196667	0.8150688	0.81841569	0.82094651	0.82260538	0.8241468	0.82582094	0.82759457	0.83056038
North Somerset	0.78395343	0.78812337	0.7913713	0.79407923	0.79690982	0.79935442	0.8012024	0.80330976	0.80574388	0.80823865	0.81152651
Plymouth	0.78027017	0.78432143	0.78720446	0.78957262	0.7922817	0.79456638	0.79694807	0.80028357	0.80441718	0.80773762	0.81000155
Poole	0.79869131	0.80269517	0.80521256	0.80721052	0.80983995	0.81231308	0.81401556	0.81550983	0.81678664	0.81801996	0.82049631
Somerset	0.77303053	0.77698019	0.77940974	0.78145855	0.78400478	0.78625955	0.78807283	0.78949123	0.79052535	0.79190708	0.79472084
South Gloucestershire	0.81687563	0.82137858	0.82449811	0.82722374	0.83025257	0.83326002	0.8360384	0.83866626	0.84133347	0.84359673	0.84584925
Swindon	0.80526529	0.80913587	0.81160586	0.81326735	0.81487751	0.81593085	0.81649947	0.81731366	0.81855129	0.82002005	0.82294159
Torbay	0.75030851	0.75338146	0.75438811	0.754903	0.75646469	0.75815326	0.75962425	0.76116012	0.76302188	0.76474152	0.76640881
Wiltshire	0.79261431	0.79672143	0.79958959	0.80197267	0.8047506	0.80677253	0.8079798	0.80848882	0.80853481	0.80925605	0.81188872
West Midlands	0.75940199	0.76419493	0.76739212	0.76990253	0.77296591	0.77602218	0.77876534	0.78158843	0.78460552	0.78762594	0.79093321
Birmingham	0.75847432	0.76318415	0.7664411	0.76927616	0.77298803	0.7766293	0.77985239	0.78339913	0.78761596	0.79191728	0.79568782
Coventry	0.78134072	0.78585288	0.7890003	0.79146355	0.79441863	0.79685247	0.79863054	0.80071697	0.80362602	0.80689408	0.81012859
Dudley	0.73979363	0.74427842	0.74729128	0.74936771	0.75152197	0.75396139	0.75620269	0.7580014	0.75919646	0.76011798	0.76160445
Herefordshire, County of	0.77159358	0.77626011	0.77935754	0.78182235	0.78456624	0.78703024	0.78902465	0.79093782	0.792409	0.79360618	0.79585412
Sandwell	0.71857945	0.72283216	0.72545606	0.72756304	0.73041819	0.7333331	0.73538172	0.73642892	0.73729346	0.73898715	0.7418434
Shropshire	0.77301822	0.77746284	0.78066583	0.78391262	0.78757885	0.79078434	0.79328886	0.79522814	0.79663556	0.79833618	0.80141039
Solihull	0.80360557	0.80827892	0.81137497	0.81380002	0.81646701	0.81882621	0.82106282	0.82343308	0.82558809	0.82715904	0.82862908
Staffordshire	0.76907437	0.7732957	0.77582975	0.77754142	0.77990074	0.78247342	0.78465858	0.78686557	0.78936718	0.79166767	0.7942002
Stoke-on-Trent	0.72714976	0.73027224	0.73156998	0.73185777	0.73252905	0.73342029	0.73404156	0.73489991	0.7372284	0.74076156	0.74467652
Telford and Wrekin	0.76241506	0.76602649	0.7679446	0.76862517	0.76962126	0.77069061	0.77158447	0.77271811	0.77365602	0.77452161	0.77645308
Walsall	0.71460718	0.71886749	0.72126627	0.72305864	0.72548179	0.72792705	0.73059278	0.73319739	0.73508391	0.73755521	0.74138152
Warwickshire	0.79830751	0.80270591	0.80567369	0.80770336	0.81001716	0.81227052	0.81455719	0.81714308	0.81986034	0.82256539	0.82578936
Wolverhampton	0.73990862	0.74486558	0.74770409	0.74969265	0.75237235	0.7549415	0.75717898	0.75971752	0.76222535	0.7641851	0.76655327
Worcestershire	0.7776588	0.78260173	0.78565652	0.78788183	0.79043604	0.79306715	0.79546466	0.79761486	0.79925241	0.80033585	0.80217175
Yorkshire and the Humber	0.7632249	0.76782316	0.77092397	0.77316885	0.77585492	0.77852448	0.78108279	0.78377648	0.78647	0.78942334	0.793
Barnsley	0.71580215	0.71939295	0.7212779	0.72254548	0.72447191	0.72614909	0.72784122	0.72996402	0.73143156	0.7332235	0.73632621
Bradford	0.73074201	0.73449237	0.73735089	0.73968877	0.74245972	0.74526746	0.74804615	0.75042709	0.75214499	0.75484221	0.75935368
Calderdale	0.75936439	0.76307062	0.76503626	0.76611469	0.76814065	0.77065512	0.77299568	0.77493226	0.77632046	0.77826625	0.78181311
Doncaster	0.71366349	0.71796464	0.72074742	0.72231981	0.72469089	0.72730461	0.72996197	0.7320148	0.73296918	0.73508999	0.7403241

Location	2011	2012	2013	2014	2015	2016	2017	2018	2019	2020	2021
Hampshire	0.83709505	0.84157957	0.8465606	0.85056054	0.85437533	0.85834057	0.86227937	0.86579977	0.86946715	0.87155772	0.87285
Isle of Wight	0.78277279	0.78683895	0.79178496	0.79608654	0.80041738	0.80519924	0.81067578	0.81603557	0.82130478	0.82451604	0.82639963
Kent	0.80819853	0.81286395	0.8179832	0.82194814	0.82530204	0.8287368	0.83266675	0.83663029	0.84102153	0.84372411	0.84534539
Medway	0.78273483	0.78741044	0.79255667	0.79636819	0.79958832	0.8030539	0.8073482	0.81167293	0.81618517	0.81893955	0.82066784
Milton Keynes	0.83881144	0.84444555	0.85141317	0.85757335	0.86320655	0.86875047	0.87415256	0.87832676	0.88221577	0.88530394	0.88717934
Oxfordshire	0.86756895	0.87109535	0.87512378	0.87863759	0.88218828	0.88591533	0.88953937	0.89279149	0.89632717	0.89831888	0.89947593
Portsmouth	0.83909559	0.84233073	0.84606216	0.84911677	0.85179364	0.8544725	0.8573527	0.86001186	0.86292961	0.86467472	0.86587593
Reading	0.87957825	0.88140175	0.88456014	0.88765942	0.89049271	0.89330058	0.89621678	0.89897063	0.90190157	0.90431908	0.90587359
Slough	0.83583971	0.84213206	0.84874124	0.85385335	0.85803702	0.86202653	0.86594187	0.86947605	0.87328253	0.8765373	0.87855725
Southampton	0.83828507	0.84058256	0.84330949	0.84534903	0.84725151	0.84961047	0.85243428	0.85517765	0.85828484	0.86010019	0.86134908
Surrey	0.87548292	0.87877828	0.88260538	0.88588635	0.88904663	0.89228143	0.89553774	0.89874817	0.9020841	0.90382965	0.9048594
West Berkshire	0.86188037	0.86685645	0.87226468	0.87666497	0.88025107	0.88359419	0.88702536	0.89021858	0.89345061	0.89608614	0.89775701
West Sussex	0.82901353	0.83347447	0.83865143	0.84291021	0.84659433	0.8501431	0.85380912	0.85733381	0.86114164	0.86339007	0.86479215
Windsor and Maidenhead	0.88232926	0.88603994	0.89085791	0.89511708	0.89881061	0.90235087	0.9059027	0.90929175	0.91284889	0.91473804	0.91581805
Wokingham	0.88727031	0.88997221	0.8936533	0.89665973	0.89922274	0.90173139	0.90424761	0.90651357	0.90918875	0.91072748	0.91165875
South West England	0.82367897	0.82820951	0.83331304	0.83751707	0.84135103	0.8452322	0.84936962	0.85337881	0.85759435	0.86000988	0.86146702
Bath and North East Somer	0.87001823	0.8727443	0.87575204	0.87828145	0.88090449	0.88409246	0.88745563	0.89031457	0.8930694	0.89445098	0.89543536
Bournemouth	0.84123649	0.84459133	0.84861968	0.85211305	0.85519666	0.85822726	0.86143265	0.86447808	0.86761169	0.86931921	0.87046477
Bristol, City of	0.8658109	0.86912093	0.87306573	0.87672518	0.88024725	0.88375121	0.8873875	0.89072045	0.89406699	0.89587235	0.89704187
Cornwall	0.79843508	0.80332577	0.80902662	0.81370439	0.81775645	0.82192046	0.82638101	0.83073446	0.83539397	0.8381743	0.83976856
Devon	0.8208979	0.82539621	0.83043518	0.83442479	0.83778135	0.84089371	0.84426645	0.8477182	0.85162009	0.85396499	0.8553761
Dorset	0.808423	0.81357242	0.81921679	0.82387957	0.82816077	0.83261981	0.83748523	0.84228551	0.84711044	0.84993704	0.85161514
Gloucestershire	0.83437906	0.83915456	0.84429117	0.84859362	0.8526156	0.85648445	0.86041398	0.86417148	0.86810169	0.87037831	0.8718063
North Somerset	0.81562505	0.82109059	0.82696856	0.83172396	0.8360364	0.84056734	0.84552716	0.85013781	0.85473285	0.85742559	0.85901767
Plymouth	0.8120936	0.81574968	0.82019961	0.82356052	0.8264205	0.82930036	0.83241611	0.83570451	0.83954392	0.84182482	0.84330754
Poole	0.82436683	0.82995814	0.83599646	0.8408079	0.84482	0.84865368	0.85248022	0.85598662	0.859739	0.86203606	0.8634943
Somerset	0.79856494	0.8038229	0.80964748	0.81434725	0.81878724	0.82340885	0.82842183	0.83335523	0.83846511	0.84156352	0.84340431
South Gloucestershire	0.84861734	0.85315551	0.85859109	0.86302103	0.86698568	0.87066636	0.87417854	0.87753267	0.88130736	0.8836038	0.88498476
Swindon	0.8271647	0.8329984	0.83908782	0.84340154	0.84689113	0.85066236	0.85496726	0.85914119	0.8634704	0.86605834	0.86754591
Torbay	0.76847408	0.7725493	0.77785163	0.78236604	0.78666901	0.79130529	0.79652416	0.80181322	0.80739957	0.8109517	0.81303848
Wiltshire	0.81586941	0.8213935	0.82725146	0.83199469	0.83652255	0.84123202	0.84609662	0.85059802	0.85515896	0.85786894	0.85946624
West Midlands	0.79473515	0.80022585	0.80627967	0.81090038	0.81466979	0.81833681	0.82245982	0.82655635	0.83119289	0.8341226	0.83586514
Birmingham	0.79949509	0.80504462	0.81099026	0.81533317	0.81856957	0.82169385	0.82547098	0.82928301	0.83360171	0.8363727	0.8380669
Coventry	0.81365593	0.81832839	0.8231046	0.82667426	0.82985796	0.8332547	0.83682862	0.84027758	0.84430573	0.846954	0.84861807
Dudley	0.76384667	0.7688098	0.77512062	0.77979086	0.78316793	0.78637504	0.79038813	0.79458623	0.79924052	0.80229133	0.80416452
Herefordshire, County of	0.79905394	0.804075	0.81024118	0.81570086	0.82069788	0.82570314	0.83106166	0.83617974	0.84137324	0.84451711	0.84631047
Sandwell	0.74609279	0.75330892	0.76150626	0.76752487	0.77225644	0.77634958	0.78060815	0.78485671	0.78995172	0.79354727	0.7957223
Shropshire	0.80520777	0.81027226	0.81576026	0.81969129	0.82297794	0.82657257	0.83065305	0.83468107	0.83906667	0.84168606	0.84321876
Solihull	0.83099922	0.83534223	0.84059777	0.84515986	0.84933905	0.85328059	0.85760922	0.86220782	0.86719634	0.87026072	0.87207923
Staffordshire	0.79715621	0.80145485	0.80613642	0.80949591	0.81215811	0.81474739	0.81785817	0.82112974	0.82517526	0.82776957	0.82932024
Stoke-on-Trent	0.7489263	0.75520015	0.76256378	0.76850354	0.77337781	0.77786649	0.78239312	0.78680682	0.79227991	0.79608339	0.79824252
Telford and Wrekin	0.77990406	0.78621931	0.79337891	0.79871453	0.80263221	0.80638653	0.81091226	0.81586187	0.82163784	0.82535598	0.82734156
Walsall	0.745819	0.75194796	0.75862015	0.76366623	0.76777489	0.77161849	0.77601823	0.78061959	0.78627209	0.79021275	0.79252944
Warwickshire	0.82959047	0.83444076	0.83970743	0.84378855	0.84723101	0.85097732	0.85493102	0.85854707	0.86257368	0.86512987	0.86667266
Wolverhampton	0.76997031	0.77522982	0.78094124	0.78544814	0.78948146	0.79360241	0.79831587	0.80276611	0.80748807	0.81054331	0.81238984
Worcestershire	0.80494064	0.80937894	0.81463709	0.81890503	0.82268224	0.82643128	0.83070607	0.83489777	0.83932159	0.84201697	0.8436113
Yorkshire and the Humber	0.79715448	0.80273245	0.80873012	0.81300926	0.81641848	0.8199989	0.82412766	0.82823204	0.83292531	0.83578028	0.83745988
Barnsley	0.74085492	0.74768513	0.75503387	0.75979321	0.76331118	0.76738425	0.77228768	0.77724381	0.7831531	0.78704739	0.78926182
Bradford	0.7651499	0.77307222	0.78146074	0.78744802	0.79192952	0.79610027	0.80078437	0.80549308	0.81077249	0.8140866	0.8159859
Calderdale	0.78700259	0.79460396	0.8028525	0.80879226	0.81317984	0.81741625	0.82242643	0.82754697	0.83290612	0.8360443	0.83786393
Doncaster	0.74723035	0.75546636	0.7634393	0.7685089	0.77235445	0.77655094	0.78103803	0.78508206	0.79005164	0.7934974	0.79561402

Table S5. Socio-demographic Index values for all estimated GBD 2021 locations, 1990-2021

Location	1990	1991	1992	1993	1994	1995	1996	1997	1998	1999	2000	2001	2002	2003	2004	2005	2006	2007	2008	2009	2010	2011	2012	2013	2014	2015	2016	2017	2018	2019	2020	2021
East Riding of Yorkshire	0.72947959	0.7340582	0.73980128	0.74539872	0.75007542	0.75320415	0.7558504	0.75902701	0.76253921	0.76714029	0.77190792	0.77541984	0.77784142	0.77983664	0.78254281	0.78494296	0.78677659	0.78887476	0.7906523	0.79251856	0.79525519	0.79827886	0.802608	0.807537	0.81120261	0.81447687	0.81818708	0.82262412	0.82699617	0.8315954	0.83420933	0.83573194
Kingston upon Hull, City of	0.68194214	0.68630388	0.69136075	0.69685598	0.70205803	0.70543517	0.70884124	0.71368564	0.7193108	0.72586069	0.73235985	0.73746846	0.74056624	0.7423618	0.7446305	0.74708158	0.75004301	0.75388144	0.75809192	0.76192186	0.76485484	0.76791287	0.77243865	0.77748853	0.78028336	0.78180226	0.78360443	0.78627627	0.78943781	0.79430767	0.7978394	0.79993173
Kirklees	0.71098428	0.7159529	0.72172867	0.72729823	0.73149835	0.73376846	0.73592606	0.73900564	0.74254597	0.74727018	0.7518841	0.75527989	0.75751735	0.75945609	0.76230224	0.7649492	0.767068	0.76923034	0.77148322	0.77402084	0.77707879	0.78097817	0.78678323	0.79334374	0.79804513	0.8017518	0.80570369	0.81014066	0.81454694	0.81950305	0.82251833	0.82420765
Leeds	0.75334083	0.75870473	0.76446431	0.77012849	0.77521804	0.77927487	0.78321788	0.78771245	0.79245419	0.79795548	0.80360137	0.80853145	0.81256029	0.8160545	0.81962674	0.82311888	0.82664365	0.83033843	0.83416856	0.83744808	0.83988924	0.84211762	0.84523085	0.84877432	0.85134105	0.85342879	0.85566212	0.85836533	0.86129905	0.86502661	0.86733998	0.86876125
North East Lincolnshire	0.67951775	0.68501216	0.69126915	0.69718262	0.70238945	0.70550476	0.70773349	0.71049304	0.71345205	0.71788501	0.72312402	0.7267298	0.72790829	0.72769286	0.72856609	0.73075318	0.73366541	0.73679164	0.73916289	0.74188384	0.74587937	0.7508597	0.75815562	0.76636165	0.77223551	0.77695238	0.78176291	0.78697509	0.79201602	0.7981714	0.80242105	0.80483785
North Lincolnshire	0.72143198	0.72498602	0.72958877	0.7346742	0.7391942	0.74239997	0.74532825	0.74847834	0.7514857	0.75469626	0.75826371	0.76155487	0.7636934	0.76474285	0.76581921	0.76708404	0.76874501	0.77039975	0.77132587	0.77398941	0.77932018	0.78539781	0.79260005	0.79967865	0.80377813	0.80644455	0.80953287	0.813241	0.8169412	0.82141363	0.82424309	0.82590487
North Yorkshire	0.74608911	0.7503064	0.75507231	0.75988281	0.76392613	0.76651006	0.76891745	0.77233138	0.77626258	0.78108412	0.78635729	0.79060775	0.79354854	0.79585924	0.7987629	0.80141609	0.80357594	0.80507233	0.80621353	0.80807882	0.81118381	0.81539244	0.82126436	0.82717479	0.83104951	0.83419753	0.83777266	0.84240359	0.8471695	0.85190497	0.85461769	0.85619466
Rotherham	0.68404895	0.68800525	0.69307379	0.69868356	0.7038878	0.70681298	0.70906167	0.71208962	0.71538509	0.71998401	0.72528019	0.72943114	0.73208845	0.73415469	0.73697094	0.73948253	0.74122531	0.74298449	0.74503668	0.74759725	0.75118263	0.7552126	0.76091717	0.76809633	0.77342731	0.77770324	0.78242769	0.78782923	0.79301919	0.79862188	0.80215957	0.80422881
Sheffield	0.74087044	0.74493933	0.74959241	0.75468329	0.75974195	0.76373448	0.76714984	0.77074699	0.77481345	0.77996309	0.7856316	0.79071623	0.79450324	0.79751226	0.8011134	0.80470991	0.80794172	0.81160302	0.81580425	0.81959563	0.82283648	0.82613627	0.83025416	0.83426294	0.83707787	0.83945781	0.8421767	0.84527023	0.84819258	0.8515876	0.85360997	0.85489097
Wakefield	0.69243647	0.69653247	0.70182314	0.70780051	0.71315283	0.71636359	0.71906139	0.72236837	0.7256555	0.73027938	0.73579856	0.73988621	0.74183011	0.74291679	0.74498837	0.74700719	0.74842609	0.74943653	0.75002797	0.75165293	0.75497844	0.75973113	0.7670525	0.77524882	0.78087355	0.7846803	0.78832109	0.79254754	0.7965832	0.80144121	0.80474471	0.80681149
York	0.78715402	0.79237367	0.79758136	0.80225084	0.80640214	0.80992848	0.81335482	0.81728675	0.82146373	0.82626957	0.83125939	0.83585085	0.83970369	0.84293237	0.84627296	0.84947327	0.85252994	0.85559102	0.85859821	0.86126199	0.86379114	0.86637336	0.8694626	0.872595	0.87495675	0.87715855	0.87947393	0.8818522	0.88410163	0.8865909	0.88781128	0.88873893
Northern Ireland	0.72272326	0.72846976	0.7351616	0.74202178	0.74762993	0.75132092	0.7546225	0.75864347	0.76287217	0.76813194	0.77384799	0.77883952	0.78350445	0.78767388	0.79111671	0.79359254	0.79596234	0.79834743	0.80111937	0.80382392	0.80626793	0.80926674	0.81311377	0.81679016	0.82006248	0.82378622	0.82777576	0.83096932	0.83280699	0.8352507	0.83704932	0.83853194
Scotland	0.74397249	0.74822002	0.75341705	0.75954379	0.76480928	0.76863789	0.77139533	0.77465091	0.77953994	0.78525675	0.7904048	0.79483002	0.7984167	0.80117767	0.80371686	0.80595463	0.80752952	0.80918775	0.81205983	0.81565915	0.81936935	0.82244997	0.82485759	0.82758968	0.83094453	0.83410999	0.83743176	0.84114064	0.84476207	0.84863859	0.85078715	0.85206142
Wales	0.71014073	0.71547804	0.72140769	0.7275336	0.73310542	0.73695851	0.74048567	0.74477533	0.7493989	0.75503199	0.76099681	0.76584341	0.76943011	0.77209612	0.77506018	0.7777931	0.78019275	0.78269139	0.78514879	0.78762431	0.79065666	0.79419495	0.79911822	0.80492191	0.80941694	0.81315138	0.81681818	0.8205084	0.82461706	0.8297892	0.8327143	0.83427854
Latin America and Caribbean	0.49773809	0.5017305	0.50567483	0.50987588	0.51433915	0.5185465	0.52287732	0.527604	0.53255967	0.53750923	0.54272237	0.54788854	0.55297248	0.55781592	0.56284923	0.56790262	0.57305602	0.57835546	0.58376018	0.58847432	0.59355664	0.5990377	0.60455091	0.61016338	0.61554288	0.62047013	0.62510555	0.62980657	0.63453797	0.63917311	0.64298253	0.64654128
Andean Latin America	0.50001149	0.5016522	0.50365586	0.50638843	0.51030557	0.51489634	0.51937645	0.52403649	0.52844391	0.53274372	0.53756051	0.54257235	0.54791107	0.55310898	0.55823544	0.56309152	0.5679874	0.57299897	0.57856419	0.58399894	0.59020118	0.59694462	0.60383923	0.61070517	0.61705999	0.62276075	0.62816126	0.6335379	0.63886016	0.64392132	0.64780682	0.65160246
Bolivia	0.42391796	0.4293978	0.43483554	0.44060165	0.4468225	0.45347924	0.46022067	0.4669829	0.4737962	0.48019431	0.4863279	0.49203076	0.49734884	0.5023658	0.50727304	0.51216649	0.51714619	0.52194613	0.52694375	0.53179244	0.53700907	0.54268074	0.54861548	0.55478822	0.56093458	0.5670009	0.57308032	0.57924128	0.58518411	0.59069204	0.59485444	0.5990108
Ecuador	0.51843061	0.51767942	0.51837907	0.52070729	0.52490829	0.52938075	0.5326914	0.53534965	0.53719633	0.53903231	0.54304478	0.54855215	0.55489733	0.56095548	0.56629029	0.56992734	0.57251627	0.5747047	0.57812504	0.58248426	0.58876306	0.59672908	0.60545432	0.61408027	0.62202743	0.62854671	0.63428114	0.64001122	0.64588852	0.65178799	0.65671446	0.66101705
Peru	0.51041985	0.51216152	0.51382155	0.51598151	0.51930608	0.52364053	0.52831204	0.53357649	0.53872172	0.54385338	0.5489522	0.55377545	0.55885326	0.56387107	0.56906302	0.574517	0.58050336	0.58695849	0.59375081	0.59995499	0.60652093	0.61305122	0.61935526	0.62551902	0.63111435	0.63627827	0.64128528	0.64618085	0.65097732	0.65543373	0.65867224	0.66205404
Caribbean	0.51811138	0.52278859	0.52698419	0.53050679	0.53358431	0.53665059	0.53977593	0.54330289	0.54736507	0.55206017	0.55736894	0.56311049	0.56906859	0.57488862	0.58053331	0.58575799	0.59060234	0.59470961	0.59829691	0.60165483	0.60548008	0.60952505	0.61325472	0.61687685	0.62044265	0.62407467	0.62758207	0.63078807	0.63397217	0.63732123	0.63970853	0.64200305
Antigua and Barbuda	0.61210459	0.61881713	0.62469582	0.63007585	0.63476896	0.63800863	0.64164719	0.64589559	0.65061429	0.65553499	0.66081906	0.66562063	0.6703608	0.67531149	0.68041243	0.68548644	0.69082602	0.69636004	0.70217536	0.70712856	0.71143225	0.71548999	0.71950932	0.72286498	0.72558598	0.72837276	0.73159894	0.73496371	0.73887911	0.74305296	0.74634533	0.74988689
The Bahamas	0.69350927	0.68912678	0.68734384	0.6938165	0.70681989	0.71938161	0.72847884	0.73478121	0.73928129	0.74162285	0.74251795	0.74359288	0.74625032	0.74991671	0.75375964	0.75678266	0.75918594	0.761991	0.7655905	0.76956167	0.77411981	0.77828189	0.78183864	0.78462391	0.78725349	0.78995615	0.7927387	0.79534718	0.79817903	0.80103307	0.80294802	0.80502067
Barbados	0.65358252	0.65665407	0.66244664	0.66881501	0.67340937	0.67601225	0.67719305	0.67864483	0.67982717	0.68039846	0.6813042	0.68332939	0.68780758	0.69323596	0.6980297	0.70151005	0.70385619	0.70587165	0.7085145	0.71214336	0.71620855	0.72006069	0.72416246	0.72782617	0.73038329	0.73260299	0.73491253	0.73715726	0.73953144	0.74223931	0.74436665	0.74674876
Belize	0.42372699	0.43383439	0.44419851	0.45525858	0.46572931	0.47527851	0.48270216	0.48802013	0.49181934	0.4950702	0.49864373	0.50239943	0.50687166	0.51267946	0.51969733	0.52723106	0.53488962	0.54190069	0.54809322	0.55346587	0.55854689	0.56344036	0.56845624	0.57323646	0.57809133	0.58327909	0.58827591	0.59333729	0.59851201	0.6033516	0.60706005	0.610229
Bermuda	0.6964512	0.70027685	0.70398409	0.70767822	0.711069	0.71446883	0.71783167	0.72164281	0.72585353	0.73053982	0.7359965	0.74191891	0.7475445	0.75330516	0.75900888	0.7646909	0.77059677	0.77611426	0.78035833	0.78475911	0.78918147	0.79338052	0.79748487	0.8013882	0.80492341	0.80799879	0.81055084	0.81280723	0.8148928	0.81700034	0.81920342	0.82136542
Cuba	0.55801907	0.56341364	0.56616901	0.56544105	0.56318631	0.5607659	0.55934633	0.55892457	0.55936322	0.56182095	0.5666769	0.57275386	0.57996829	0.58764776	0.59434872	0.5998638	0.60497057	0.6076181	0.60940092	0.61177564	0.61526126	0.62038669	0.62615602	0.6318398	0.63730413	0.64281896	0.64790787	0.65230929	0.65685865	0.66167472	0.66521075	0.66872986
Dominica	0.56360259	0.56523784	0.56957068	0.57881181	0.59011915	0.60198693	0.61438205	0.62505735	0.63301799	0.63945392	0.64583094	0.65236857	0.65842355	0.66367887	0.66895213	0.67454908	0.68034248	0.68541592	0.69019752	0.69446187	0.69848919	0.70246326	0.70687061	0.71221048	0.71845556	0.72478554	0.7317608	0.73606247	0.73893595	0.74213207	0.74441994	0.74696718
Dominican Republic	0.44265408	0.44614458	0.44995308	0.4542293	0.45862061	0.46331387	0.46851694	0.47450439	0.48117094	0.48817854	0.49546859	0.50277542	0.51029802	0.51739557	0.52454327	0.53222865	0.54022269	0.54818702	0.55537131	0.56178375	0.56813058	0.57383481	0.57896638	0.58362583	0.58823674	0.59292416	0.59761961	0.60214029	0.60696084	0.61191302	0.61563563	0.6193882
Grenada	0.43673442	0.44588795	0.45591006	0.46599489	0.47641034	0.486559	0.49658981	0.5062176	0.51659002	0.52733341	0.53838812	0.54905897	0.55947313	0.56971804	0.57848746	0.58715888	0.59405452	0.60076634	0.60687928	0.61180526	0.616487	0.62097852	0.62497751	0.62904834	0.6337347	0.63893608	0.64433523	0.64983152	0.65537277	0.66080851	0.66508635	0.66899303
Guyana	0.46043013	0.46267707	0.46675399	0.47217423	0.47899292	0.48657346	0.49521604	0.50436278	0.5130689	0.52159598	0.52869399	0.53486981	0.54051427	0.54551647	0.55032637	0.5548448	0.55946561	0.56454114	0.56959521	0.57476993	0.58008391	0.58568827	0.59133658	0.59682629	0.60213651	0.60760549	0.61391436	0.62060261	0.62716704	0.63364035	0.64228464	0.65081234
Haiti	0.31033463	0.31545544	0.32038998	0.32489585	0.32839946	0.33299915	0.3382294	0.34388005	0.34969807	0.35598074	0.36257221	0.36864351	0.37447332	0.38006725	0.38506151	0.38984542	0.394322	0.39897344	0.40328587	0.40794442	0.41184011	0.41617719	0.42002153	0.42398555	0.4279409	0.43175676	0.43528872	0.438541	0.44167893	0.44432885	0.44639061	0.44827828
Jamaica	0.53478123	0.53959448	0.54508875	0.55121849	0.55764377	0.56431504	0.57094013	0.57721209	0.58304803	0.58884647	0.59459292	0.60019202	0.60559175	0.61087742	0.61583628	0.62040006	0.62501886	0.62954947	0.63396785	0.63811084	0.64212939	0.64639583	0.65058376	0.65457362	0.65850534	0.66246581	0.66646928	0.67029105	0.67407531	0.67777854	0.68056718	0.68326306
Puerto Rico	0.65875815	0.66326172	0.66727219	0.67102411	0.67545081	0.68067395	0.68586589	0.69127438	0.69704045	0.70285067	0.71003565	0.71895586	0.72595686	0.73047514	0.73421955	0.73774227	0.74162943	0.74588381	0.75092217	0.75636169	0.76176325	0.76678488	0.7723118	0.77846116	0.78512034	0.79180017	0.79908408	0.80664896	0.81290607	0.81884319	0.82291844	0.82552585
Saint Kitts and Nevis	0.58068588	0.59025495	0.59918719	0.60723589	0.61451466	0.6207165	0.62620741	0.63127474	0.63553999	0.63971374	0.64435338	0.64922665	0.65459341	0.65992531	0.66756813	0.67914546	0.69007867	0.69772763	0.70414149	0.7094995	0.71446042	0.71932438	0.72343615	0.72670659	0.72953022	0.73222067	0.73556614	0.73947068	0.74373646	0.74815207	0.75156841	0.75498705
Saint Lucia	0.49629657	0.50597555	0.51536826	0.52423495	0.53273108	0.54147979	0.55024241	0.55829346	0.5661387	0.57342856	0.5797889	0.58508756	0.59016758	0.59567515	0.60162548	0.60723987	0.6127553	0.61799471	0.623273	0.62816985	0.6327829	0.63743944	0.64156713	0.64515308	0.64874351	0.65215748	0.65565334	0.65934135	0.66308668	0.66684036	0.66972049	0.67250974
Saint Vincent and the Grenadines	0.47593019	0.48107566	0.48597787	0.49099879	0.49637913	0.50298609	0.50952716	0.51548262	0.52120968	0.52709955	0.53317329	0.53945571	0.54606362	0.55269372	0.55866354	0.56358812	0.56822175	0.57281905	0.57761114	0.58254911	0.58719038	0.5902591	0.59215391	0.59461407	0.59837867	0.60375413	0.61005921	0.61643847	0.62263494	0.62832599	0.6328609	0.63719596
Suriname	0.5020543	0.50665579	0.51095977	0.51441944	0.51765025	0.52100262	0.52505932	0.52975081	0.5345997	0.53893279	0.54366002	0.54868352	0.55387943	0.55941837	0.56510971	0.57046219	0.57564705	0.58065457	0.58564483	0.5905128	0.59546975	0.60035634	0.60474187	0.60891061	0.61270442	0.61609732	0.61886809	0.62168418	0.62470801	0.62789678	0.63068371	0.63366574
Trinidad and Tobago	0.62397015	0.62875892	0.63403955	0.63919191	0.64424291	0.64922006	0.65416946	0.65929363	0.66450771	0.67024457	0.67668384	0.68276349	0.68887227	0.69582834	0.70261081	0.70852098	0.71460021	0.72019642	0.7257207	0.72953889	0.73323708	0.7368024	0.73999907	0.74359396	0.74733896	0.75151068	0.75495335	0.75822699	0.76124199	0.7641696	0.76642208	0.76876325
Virgin Islands	0.65516086	0.66401866	0.67108187	0.67687274	0.68198222	0.68673021	0.6912477	0.6955982	0.69976869	0.70394934	0.70843656	0.71375111	0.72439827	0.73460985	0.7446136	0.75426756	0.7634475	0.77218059	0.78034262	0.78705515	0.7932197	0.7980717	0.80109285	0.80340579	0.80564138	0.80788925	0.81019904	0.81240517	0.81482544	0.81742901	0.81960171	0.82183085
Central Latin America	0.48578741	0.48955853	0.49411019	0.49941797	0.50506064	0.50987425	0.51469347	0.52017067	0.52613181	0.53180702	0.53730037	0.54243984	0.54713435	0.5514461	0.5560115	0.56074953	0.56564869	0.57070727	0.57583933	0.57994006	0.58425317	0.58930755	0.59482717	0.60073102	0.60650929	0.61181533	0.61708609	0.62244243	0.62776464	0.63284707	0.63699589	0.6406851
Colombia	0.48072005	0.48233989	0.48457236	0.48822937	0.49327818	0.49910276	0.50498697	0.5110795	0.5170225	0.52243594	0.52783909	0.5330765	0.53818934	0.54326513	0.54860183	0.55408919	0.56018273	0.56682264	0.57349465	0.57987647	0.58640811	0.59334048	0.60025424	0.60725064	0.61415192	0.62088923	0.6275545	0.63413036	0.64025507	0.64603885	0.65085535	0.65544291
Costa Rica	0.53412518	0.53934216	0.54462424	0.54994663	0.55517125	0.56054709	0.56518086	0.57012842	0.57606705	0.58222949	0.58824319	0.59488911	0.60089443	0.60576569	0.6103157	0.61441262	0.61785016	0.62120057	0.62570391	0.63129138	0.63659543	0.6412013	0.64657409	0.65194581	0.65689046	0.66203014	0.66743009	0.67362139	0.68129615	0.69004909	0.69619461	0.70034048
El Salvador	0.37305797	0.37485533	0.37707664	0.38030733	0.38514909	0.39178027	0.40017847	0.41045583	0.42142119	0.43273315	0.44396267	0.45417766	0.46296886	0.47039741	0.47653666	0.48204172	0.486826	0.49080975	0.49507774	0.49948971	0.50417134	0.50896924	0.51339687	0.51812191	0.5234689	0.52938695	0.53565027	0.54199896	0.54814317	0.55403165	0.55891989	0.56377519
Guatemala	0.31179246	0.31432948	0.31916548	0.32684082	0.3334691	0.3396421	0.34696121	0.35599596	0.36646119	0.37445532	0.3798773	0.38692769	0.39689964	0.40823416	0.41977706	0.43032753	0.43885638	0.44724596	0.45654187	0.46400308	0.47047736	0.47690968	0.48186285	0.48728669	0.49402719	0.50065561	0.50714979	0.51373568	0.52099547	0.52863047	0.53457141	0.53997242
Honduras	0.33204289	0.33703465	0.34230566	0.34783845	0.35316485	0.35878727	0.36432842	0.36998389	0.37595011	0.38175232	0.38798805	0.39417401	0.4003718	0.40666838	0.41306182	0.41961233	0.426353	0.43324031	0.43999816	0.44615105	0.45238223	0.45870033	0.46492515	0.47076683	0.4765878	0.48229418	0.48770593	0.49313069	0.49865723	0.50415651	0.50866925	0.51303725
Mexico	0.50499608	0.50951067	0.51463806	0.52049317	0.52674924	0.53155414	0.53653083	0.54198817	0.54784212	0.55381337	0.55953255	0.56477418	0.57011353	0.57569821	0.58098498	0.58522408	0.58854323	0.59144741	0.59445456	0.59668436	0.59952703	0.6036461	0.60891429	0.61508999	0.6218574	0.62889672	0.63592535	0.6426672	0.64905332	0.65509534	0.66011906	0.6645753
Aguascalientes	0.52464598	0.52908631	0.53339688	0.53811721	0.54326831	0.54745275	0.55219862	0.55740598	0.56313221	0.56946169	0.57602722	0.58238199	0.5889208	0.59528251	0.60098395	0.6052851	0.60866303	0.61151629	0.61424432	0.61621885	0.61901915	0.62293883	0.62772639	0.63339809	0.63976965	0.64638692	0.6529246	0.65932105	0.66550502	0.67148323	0.67656818	0.68115928
Baja California	0.5577769	0.5593657	0.56195761	0.56569212	0.5700044	0.57308653	0.57667421	0.58138937	0.58718167	0.59331803	0.59933828	0.60536455	0.61185852	0.61819065	0.6233876	0.62712069	0.6298734	0.6322404	0.63481885	0.63658599	0.63888763	0.64266689	0.64772087	0.65351265	0.65971276	0.66640432	0.67356449	0.68055944	0.68732717	0.69378576	0.69923592	0.70408197
Baja California Sur	0.54736547	0.55222823	0.55700866	0.5625506	0.56859309	0.57309075	0.5775911	0.58235863	0.58732678	0.59177257	0.59584449	0.60006956	0.60486462	0.61048615	0.61622124	0.62103964	0.62487862	0.62826975	0.6318338	0.63507846	0.63924246	0.64472575	0.65111728	0.65803001	0.66517874	0.67275445	0.68022975	0.68730215	0.6939065	0.70006349	0.70516978	0.70969428
Campeche	0.46780958	0.47300838	0.47904871	0.48646628	0.49468335	0.50170029	0.50918061	0.51699496	0.52510594	0.5331952	0.54081983	0.54766425	0.55399832	0.56010839	0.56568472	0.57021152	0.57369097	0.57695541	0.58046699	0.58358722	0.58795187	0.59396891	0.6012821	0.60945014	0.61820947	0.62666517	0.63461986	0.64212714	0.6490849	0.65552325	0.66079487	0.66536343
Chiapas	0.38012271	0.38508742	0.3916589	0.39966773	0.40841284	0.41503154	0.42085304	0.42670943	0.43257189	0.4385003	0.44413082	0.44941525	0.45517242	0.46203366	0.46912156	0.47529399	0.48064481	0.48513561	0.48943204	0.49313887	0.49767803	0.50360146	0.51055297	0.51827703	0.52633506	0.53433331	0.54174283	0.54860507	0.55484592	0.56063971	0.56553923	0.56994091

Table S5. Socio-demographic Index values for all estimated GBD 2021 locations, 1990-2021

Location	1990	1991	1992	1993	1994	1995	1996	1997	1998	1999	2000	2001	2002	2003	2004	2005	2006	2007	2008	2009	2010	2011	2012	2013	2014	2015	2016	2017	2018	2019	2020	2021
Chihuahua	0.52608557	0.52836974	0.53062121	0.53308826	0.53546396	0.53677045	0.53895721	0.54214119	0.54644911	0.55138163	0.55667892	0.56253594	0.56932625	0.57637622	0.5828403	0.58806159	0.59205091	0.59546385	0.59911245	0.60119468	0.60315739	0.60632246	0.61056631	0.6163539	0.62315116	0.63106406	0.63913046	0.64693782	0.65440955	0.66158134	0.66781309	0.67338657
Coahuila	0.52430357	0.52961776	0.53535695	0.54152311	0.54787387	0.55244184	0.55700854	0.56204904	0.56750608	0.57317433	0.57883433	0.5840259	0.5890641	0.5944839	0.59954588	0.60333064	0.60590202	0.60765195	0.60931691	0.60973611	0.61041472	0.61244547	0.61575958	0.62103424	0.62794617	0.63548626	0.64341066	0.6513131	0.65889094	0.66610472	0.67219156	0.67756288
Colima	0.54115293	0.54726373	0.55318114	0.55905337	0.56488775	0.56970981	0.57516713	0.58069234	0.58606007	0.59177315	0.59772777	0.60285289	0.60751331	0.61204098	0.61615131	0.61976899	0.62281415	0.62561546	0.62862318	0.63102798	0.63432309	0.63871612	0.64401238	0.65030157	0.65720227	0.66443733	0.6715459	0.67817972	0.68432488	0.69002339	0.69479277	0.69911677
Durango	0.47199486	0.4757531	0.48009595	0.48486755	0.48988234	0.4941355	0.49927379	0.50554468	0.51281359	0.51995516	0.52647474	0.53278507	0.53938985	0.54636552	0.55270206	0.55723541	0.5603506	0.56274673	0.5651476	0.56687741	0.56933197	0.57313736	0.57834545	0.58478399	0.59198057	0.59964337	0.60736431	0.61475571	0.62176943	0.62839404	0.63414276	0.63932484
Guanajuato	0.46311139	0.47018838	0.47736152	0.48458844	0.49185674	0.49813801	0.50509692	0.51239723	0.52000553	0.52749219	0.53458618	0.54100819	0.54720638	0.55323782	0.55873953	0.56315616	0.56669951	0.56968314	0.57274518	0.57518014	0.57837607	0.58297839	0.58883586	0.59514959	0.60177357	0.60879	0.61629616	0.62342171	0.63017593	0.63652515	0.64178294	0.64637431
Guerrero	0.40445724	0.40825547	0.4124704	0.41713034	0.42225453	0.42746483	0.43391589	0.44072225	0.44759717	0.45444156	0.46093254	0.46720119	0.47372739	0.48043549	0.48664537	0.49140142	0.49528328	0.49907592	0.50348019	0.50750852	0.51248156	0.5187392	0.52611801	0.53432833	0.54284332	0.55090221	0.55826047	0.56494497	0.57089293	0.57631346	0.58083578	0.58485047
Hidalgo	0.43377228	0.43986963	0.4466513	0.4541313	0.46210962	0.46901751	0.47618808	0.48374589	0.49146979	0.49898899	0.50586657	0.51211415	0.51828996	0.52481297	0.53118129	0.53641315	0.54068698	0.54463751	0.54886168	0.55265827	0.55741998	0.56348608	0.57072053	0.57876194	0.5872382	0.59585308	0.60387871	0.61131371	0.61810621	0.62427846	0.62919666	0.63347845
Jalisco	0.51972252	0.52512765	0.53088856	0.53702262	0.54334893	0.54828799	0.55351924	0.5592973	0.5653957	0.5714022	0.57701229	0.58202038	0.58694671	0.59202047	0.59691328	0.60072816	0.60388805	0.60674647	0.60984851	0.6119959	0.61466819	0.6184028	0.6231613	0.62878405	0.63501843	0.64149564	0.64800707	0.65435007	0.6604767	0.66641179	0.67142597	0.67599837
México	0.53064866	0.53512321	0.54058655	0.54705178	0.5538444	0.55805196	0.56144587	0.5648466	0.56839902	0.57259391	0.57691942	0.58106381	0.58582736	0.59120498	0.59686804	0.60177977	0.60567699	0.60910883	0.61242263	0.61495132	0.61801981	0.62217966	0.62735517	0.6333249	0.63982252	0.64641531	0.65315396	0.65977775	0.66614588	0.67220359	0.67717449	0.68158761
Mexico City	0.6183256	0.62283011	0.6280875	0.63454388	0.64174362	0.64699086	0.65211718	0.65780475	0.66381817	0.66972238	0.67514284	0.67961917	0.68392976	0.68818145	0.69202952	0.69492232	0.69665424	0.69788087	0.69910929	0.69977615	0.70105554	0.70359496	0.70757164	0.71272226	0.7187518	0.72509475	0.73171036	0.73834093	0.74480794	0.75091056	0.75573933	0.75984841
Michoacán de Ocampo	0.45429333	0.46131738	0.46851226	0.47542328	0.48190993	0.48712824	0.49248967	0.4982608	0.50433887	0.51045873	0.51653161	0.52210021	0.5274816	0.53296124	0.53789494	0.54188616	0.54540142	0.54837031	0.55118386	0.55309694	0.55560424	0.55941862	0.56425136	0.5697645	0.57574863	0.58200157	0.58799894	0.59374718	0.59921892	0.60446897	0.60894406	0.61308202
Morelos	0.52099149	0.5271591	0.53323135	0.53946774	0.54578695	0.5509336	0.55640727	0.56197648	0.56747134	0.57314982	0.57915846	0.58428072	0.58878902	0.59302338	0.5964867	0.59876495	0.60044167	0.60198986	0.60392155	0.60521237	0.60739619	0.61082863	0.61539009	0.62113935	0.6277245	0.63476951	0.64183492	0.64865498	0.65501762	0.66090471	0.66575519	0.67007405
Nayarit	0.47346391	0.47793638	0.48286679	0.48830129	0.49401284	0.49907927	0.50501084	0.51145693	0.51853219	0.52614717	0.53361802	0.54084317	0.54829926	0.55618327	0.56372443	0.56997449	0.57504674	0.57852923	0.58130463	0.58358488	0.58699834	0.5919435	0.59832169	0.60563177	0.61348113	0.62147646	0.62901613	0.63620787	0.6428643	0.64890895	0.65383828	0.65817891
Nuevo León	0.58172231	0.58573259	0.58994407	0.59423107	0.59848708	0.60140658	0.60454057	0.60833166	0.61298524	0.61816602	0.62340677	0.62844154	0.63362729	0.6385176	0.64273628	0.64574276	0.6479221	0.64969342	0.65138713	0.65225715	0.65370374	0.65651089	0.66056596	0.66545621	0.67097312	0.67685353	0.68283157	0.68869497	0.69450271	0.70033179	0.7054379	0.71014182
Oaxaca	0.41430202	0.41825538	0.42276546	0.42802641	0.433694	0.43843657	0.44378024	0.44923964	0.45449285	0.45971443	0.4647321	0.47001697	0.47596674	0.48289772	0.48994688	0.49571783	0.50049736	0.50547992	0.51104287	0.51590312	0.52153162	0.52772142	0.53427677	0.54121777	0.54829387	0.55548763	0.562415	0.56882615	0.57475406	0.58032331	0.5849127	0.5890419
Puebla	0.44952995	0.45626666	0.4635741	0.47106719	0.47834259	0.48355257	0.48820611	0.49319843	0.49853768	0.50406637	0.50946911	0.51476248	0.5204563	0.52662078	0.53246478	0.53734586	0.54150447	0.54515706	0.54877225	0.55173609	0.55548434	0.56038968	0.56637828	0.57313399	0.58022349	0.58729009	0.59416603	0.60068456	0.60680583	0.61261265	0.61754268	0.62203183
Querétaro	0.50732019	0.51218011	0.51746586	0.52387079	0.53088852	0.53682026	0.5430477	0.55003127	0.55746246	0.56582274	0.57471192	0.58262614	0.59020629	0.5969834	0.60286326	0.60763528	0.61150265	0.61493464	0.61829291	0.62065493	0.62337074	0.62740736	0.63267686	0.63847015	0.64455963	0.65067975	0.65690943	0.66285294	0.66847775	0.67385834	0.67840425	0.68255812
Quintana Roo	0.49122107	0.49965855	0.5088494	0.51896604	0.52927571	0.53809792	0.54733879	0.55607698	0.56403409	0.5712848	0.57777696	0.583471	0.58886607	0.59427902	0.59922215	0.60306565	0.60619519	0.60928534	0.6127802	0.61582787	0.6197026	0.62455861	0.63012531	0.63622861	0.64252622	0.64896632	0.65546411	0.66152672	0.6671612	0.67247292	0.6769749	0.68106867
San Luis Potosí	0.45014566	0.45517811	0.46086589	0.46788602	0.4759089	0.48281369	0.49026291	0.49800723	0.50598119	0.51378859	0.52106938	0.52781326	0.53446477	0.54137996	0.54808194	0.55416128	0.55964176	0.56486215	0.57023152	0.57481416	0.58010304	0.58599604	0.59232433	0.59920789	0.6064493	0.61351907	0.62014295	0.62633442	0.63214448	0.63767532	0.64234352	0.64665151
Sinaloa	0.51239837	0.51572454	0.51911006	0.52349329	0.52917168	0.53382454	0.53902124	0.54501796	0.55186449	0.55872934	0.56506574	0.5706942	0.57621237	0.58228089	0.58823541	0.59291479	0.59635723	0.59943278	0.60286843	0.60536385	0.60845589	0.61299726	0.61868781	0.6254509	0.63289691	0.64051841	0.64764677	0.65448631	0.66103819	0.66730947	0.67256473	0.67716497
Sonora	0.54686639	0.54932477	0.55217327	0.55580514	0.56014932	0.56353776	0.5673968	0.57224441	0.5781811	0.58479779	0.59178041	0.59785695	0.60344527	0.6092954	0.61458273	0.61897369	0.62255647	0.6253938	0.62814627	0.62963753	0.63155817	0.63550766	0.64130954	0.64865557	0.65705101	0.66606179	0.67505335	0.6836726	0.69186868	0.69948653	0.70560693	0.7106796
Tabasco	0.46341056	0.46983619	0.47681474	0.48391239	0.49092404	0.49713264	0.50413767	0.51162281	0.51927427	0.52682023	0.5338914	0.53980678	0.54523273	0.55086714	0.55619872	0.5609007	0.56483121	0.56845656	0.57224247	0.57543617	0.57954117	0.58503691	0.59166737	0.59880351	0.60636409	0.61399416	0.62114236	0.62785981	0.6341084	0.63985315	0.64460154	0.64872802
Tamaulipas	0.5389471	0.54463846	0.54981524	0.55530789	0.56093409	0.56610203	0.57255959	0.5787483	0.58448313	0.58890839	0.59220815	0.59502879	0.59778925	0.60077571	0.60345225	0.60547266	0.60721869	0.60929697	0.61200372	0.61373213	0.61576083	0.6193195	0.62410571	0.63000066	0.63670817	0.64390979	0.65124145	0.65831479	0.66513256	0.67169784	0.67723621	0.68208611
Tlaxcala	0.46792921	0.47561589	0.483462	0.49155613	0.49959339	0.50598163	0.51241288	0.51905729	0.52589066	0.53298182	0.54001133	0.54645412	0.55282779	0.55926087	0.56543135	0.57055635	0.57485642	0.57876288	0.58275381	0.58589762	0.58962131	0.59407332	0.59916092	0.604873	0.61091633	0.61710525	0.62316194	0.62889651	0.63427565	0.63937401	0.64374855	0.64782243
Veracruz de Ignacio de la Llave	0.45731063	0.46089962	0.46549269	0.47103541	0.4771233	0.48181168	0.48671671	0.49245068	0.49880852	0.50498357	0.51022218	0.51476074	0.51949377	0.52506646	0.53078548	0.5355783	0.53959946	0.54351481	0.54783522	0.55154502	0.55591504	0.56146529	0.56811892	0.5754009	0.58309252	0.5910758	0.59882257	0.60607728	0.61278797	0.61895227	0.62388476	0.62813696
Yucatán	0.46910116	0.47445196	0.48086408	0.48865262	0.49723525	0.50422376	0.51131875	0.51841807	0.52529203	0.5313903	0.53669085	0.54134838	0.54573208	0.55111079	0.55712125	0.56270542	0.56789218	0.57301505	0.57831159	0.58267134	0.58748259	0.59310568	0.59933071	0.60603672	0.61308507	0.62008059	0.62649344	0.63256604	0.63835718	0.64393674	0.64867162	0.65305081
Zacatecas	0.48204582	0.48758905	0.49321387	0.49888537	0.50454512	0.50895	0.51353475	0.51874579	0.52446871	0.53054276	0.53671219	0.54257064	0.54849739	0.55423957	0.55947384	0.56341252	0.56656109	0.56944972	0.57257197	0.57447126	0.57658921	0.57995006	0.58436328	0.5897267	0.59565048	0.6019342	0.60826097	0.61430989	0.62003687	0.6255434	0.63033879	0.63477366
Nicaragua	0.34603523	0.35174062	0.3577127	0.36356207	0.36972797	0.37662535	0.38408727	0.39177589	0.39980753	0.40808596	0.41610836	0.4237903	0.43081375	0.43724815	0.44327451	0.44884474	0.4539505	0.4590428	0.4638377	0.46796184	0.472172	0.47659347	0.4812969	0.48610547	0.49120735	0.49639166	0.50185334	0.50748202	0.51233823	0.51659705	0.52029267	0.52395847
Panama	0.54604812	0.55048422	0.55497275	0.55916867	0.56171453	0.56335302	0.56531007	0.56939594	0.57469732	0.58043284	0.58737839	0.5945357	0.60013868	0.60404336	0.60687703	0.60949412	0.61224971	0.61528542	0.61895891	0.62240281	0.62511632	0.62863394	0.63426344	0.64132435	0.64910817	0.65796205	0.66727676	0.6767835	0.68671992	0.69667068	0.70378849	0.70886483
Venezuela	0.51689122	0.5222723	0.52951799	0.53627998	0.54186606	0.54634823	0.54864923	0.55236444	0.55724312	0.560278	0.56350626	0.56545838	0.56260446	0.55521222	0.55097226	0.55394168	0.56333419	0.57536717	0.58618489	0.59328842	0.59912792	0.60482348	0.61018747	0.61444102	0.61468681	0.61067536	0.60699321	0.60516013	0.60406825	0.60231389	0.60003478	0.59651306
Tropical Latin America	0.49958779	0.50429048	0.50805683	0.51162222	0.51533011	0.51929088	0.52349854	0.52784148	0.53216492	0.53665855	0.54171565	0.54693151	0.55236462	0.55769144	0.56326866	0.56889732	0.57472031	0.58091535	0.58735364	0.59330312	0.599646	0.60595697	0.61178731	0.61740172	0.62264612	0.62739963	0.63153336	0.63576518	0.6401799	0.64469363	0.64860285	0.65244239
Brazil	0.50007051	0.5047848	0.50853994	0.51208179	0.51576084	0.51968596	0.52386333	0.52817789	0.53248117	0.53697203	0.5420515	0.54730443	0.55277603	0.55814571	0.56377291	0.56945812	0.57533877	0.58158876	0.58807782	0.5940733	0.60044585	0.60677729	0.61261934	0.61821824	0.62343381	0.6281498	0.63223511	0.63642219	0.64080297	0.64529801	0.64920157	0.65304389
Acre	0.37246017	0.37898024	0.38450439	0.38967272	0.39539421	0.40161963	0.40754837	0.41288988	0.4180948	0.42329498	0.42910086	0.4352422	0.44171553	0.44810708	0.45499678	0.4622089	0.46954104	0.47766215	0.48632357	0.49449839	0.50267316	0.51015135	0.51717238	0.52383613	0.53006515	0.5356433	0.54055077	0.54550663	0.55066535	0.55603086	0.56087343	0.56585153
Alagoas	0.37534582	0.38023745	0.38402716	0.38695162	0.38989601	0.39264695	0.39559337	0.39921432	0.40327678	0.40730825	0.4119514	0.41679849	0.42219061	0.42769309	0.43363762	0.43978004	0.44625282	0.45313153	0.46010413	0.46685006	0.47413253	0.48187333	0.48887141	0.49558388	0.50181441	0.50753087	0.51247415	0.51742963	0.52241993	0.52738526	0.5315926	0.53565998
Amapá	0.46648997	0.47304644	0.47814556	0.48218248	0.48682933	0.49274083	0.49860984	0.50414024	0.50893105	0.51336831	0.51824111	0.52356672	0.52916824	0.53394388	0.53867638	0.54376686	0.54992141	0.5567305	0.56379686	0.5703049	0.57661695	0.58263976	0.58882984	0.59478011	0.60037187	0.60532117	0.60951412	0.61382742	0.61842977	0.62329167	0.62765185	0.63217964
Amazonas	0.48441458	0.49017192	0.4941568	0.49817121	0.5008659	0.50203469	0.50282375	0.50301774	0.50336985	0.5041176	0.50606222	0.5083553	0.51161263	0.51529552	0.52014233	0.52558709	0.53183699	0.5382475	0.54462383	0.55048988	0.55676855	0.56268609	0.56758545	0.57235744	0.57702076	0.58139865	0.58537281	0.589701	0.59452521	0.59967702	0.60452735	0.60948447
Bahia	0.41801382	0.42390082	0.42864482	0.43292135	0.43699494	0.44027221	0.44380753	0.44752948	0.4510641	0.45485337	0.45926884	0.46395652	0.46935003	0.47474591	0.48082891	0.48749493	0.49424201	0.50147269	0.50889675	0.51622681	0.52363228	0.53042636	0.53657321	0.54246878	0.5479409	0.55278909	0.55691537	0.56103638	0.56529991	0.56967424	0.57350195	0.57739425
Ceará	0.39835372	0.40427565	0.40929496	0.41403284	0.41908863	0.42444566	0.43008083	0.43524836	0.43991082	0.44436072	0.44905546	0.45376096	0.45907005	0.46430322	0.46989832	0.47575904	0.48218972	0.48899228	0.49650801	0.50377829	0.51166156	0.51960503	0.52649963	0.5329569	0.53883263	0.54403997	0.54843112	0.5527994	0.55713287	0.56146657	0.56509036	0.5686657
Distrito Federal	0.58373019	0.59108295	0.59589769	0.59987668	0.60481995	0.61800674	0.63029083	0.64061308	0.64963242	0.65748718	0.66441614	0.67097978	0.6771953	0.68278788	0.68818668	0.6934783	0.69910631	0.70523097	0.71186188	0.71828101	0.72484482	0.73120969	0.73692571	0.74230807	0.74721747	0.7515915	0.75540551	0.75937914	0.76355805	0.76790887	0.7719088	0.77601664
Espírito Santo	0.48980083	0.49533266	0.50016147	0.50465497	0.50978021	0.51548006	0.52084499	0.52608135	0.53127897	0.5368475	0.54356148	0.55028861	0.55685332	0.56311935	0.5703109	0.57792774	0.5856401	0.59346908	0.60121699	0.60738254	0.61422973	0.62154727	0.62832847	0.63451315	0.63999595	0.64483223	0.64898961	0.65318995	0.65761295	0.66217932	0.66617627	0.67019259
Goiás	0.45343815	0.46017901	0.46509712	0.46976741	0.47463189	0.47985886	0.48553627	0.49147289	0.49770774	0.50409262	0.51143262	0.51912453	0.52749671	0.53541233	0.54316138	0.55036251	0.55758046	0.56505097	0.57259058	0.57975964	0.58687107	0.59396246	0.60064445	0.60670179	0.61209319	0.61682493	0.62077903	0.62478839	0.6289701	0.63327306	0.63708137	0.64093387
Maranhão	0.31302354	0.31922436	0.32415762	0.32866124	0.33363052	0.339376	0.34566957	0.35106659	0.35527089	0.35916684	0.36358774	0.36818221	0.37328639	0.37876805	0.38490452	0.39180998	0.3992711	0.40732112	0.4162305	0.42420826	0.4322344	0.44044871	0.44837228	0.45559983	0.46205531	0.4678107	0.47286503	0.47798381	0.48330632	0.48883121	0.4938582	0.49892987
Mato Grosso	0.45731269	0.46396776	0.4697163	0.47556961	0.48130407	0.48636848	0.49149522	0.49715891	0.50279996	0.50900163	0.51622992	0.52354486	0.53205088	0.5415599	0.55233966	0.56168368	0.56946023	0.57784383	0.58689801	0.59482991	0.60169954	0.60844833	0.61456396	0.6198616	0.62450143	0.62859601	0.63205244	0.63562973	0.63947142	0.64361388	0.64739224	0.65118576
Mato Grosso do Sul	0.48088635	0.4853938	0.48863006	0.49189174	0.49586395	0.49877929	0.50201289	0.50586417	0.51042577	0.51558516	0.5214238	0.52776671	0.5343377	0.5412101	0.54783577	0.55405041	0.56047904	0.56723467	0.57417317	0.58067343	0.58769634	0.59473936	0.60139531	0.60765836	0.61356757	0.61898809	0.62369262	0.62852876	0.63358374	0.63874298	0.64321738	0.64753332
Minas Gerais	0.49423104	0.49974703	0.50436229	0.50853184	0.51311003	0.51692601	0.52085725	0.52482581	0.52860466	0.5324724	0.5371023	0.54220372	0.54796245	0.55392298	0.5604885	0.56697721	0.57366948	0.58057011	0.5876665	0.59364851	0.60026782	0.60687262	0.61287949	0.61862861	0.62396609	0.62876546	0.63279528	0.63682282	0.64098257	0.64523321	0.64878904	0.65229325
Pará	0.43318856	0.43978241	0.44391405	0.4493909	0.45441657	0.4561978	0.45738086	0.4580976	0.45867518	0.45933038	0.46088399	0.46326273	0.46652025	0.47027497	0.47537039	0.48131073	0.4880692	0.49540936	0.50332024	0.51029576	0.51874817	0.5272334	0.53455948	0.54135179	0.54753813	0.55306489	0.55785553	0.56272867	0.56781093	0.57302851	0.57757771	0.5821344
Paraíba	0.39684631	0.40384955	0.40889901	0.41379963	0.41911529	0.42348025	0.42785255	0.43197639	0.43578099	0.43945325	0.44356385	0.44828987	0.45357463	0.45878929	0.46396904	0.4696234	0.47627149	0.48337298	0.49096978	0.49830088	0.50571183	0.5131686	0.52023424	0.52685659	0.53296335	0.53834627	0.54285845	0.54731419	0.55182047	0.55632748	0.56013944	0.56386815
Paraná	0.50965088	0.51354421	0.51666547	0.5199408	0.5240295	0.52844239	0.5334661	0.53891865	0.54482851	0.55106159	0.55764124	0.56430073	0.57125354	0.57848667	0.58556012	0.59191819	0.59816094	0.60494367	0.61151613	0.61731326	0.62318032	0.62898059	0.63439992	0.63969304	0.64480177	0.64956588	0.6537573	0.65808635	0.66266957	0.6673212	0.67118223	0.67485653
Pernambuco	0.42521206	0.43119677	0.43529459	0.43862526	0.44179538	0.444271	0.44706538	0.4500632	0.45326235	0.45655056	0.46036538	0.46463093	0.46970161	0.47484008	0.48033601	0.48638652	0.49287223	0.49985444	0.50714301	0.51431826	0.52227143	0.53013497	0.53780983	0.54507415	0.55178047	0.55775813	0.56292157	0.56802278	0.57314208	0.5781638	0.58251064	0.58676422
Piauí	0.34403412	0.3504907	0.35514493	0.36030186	0.36606124	0.37274327	0.3792832	0.38493097	0.38973714	0.39411099	0.39882112	0.4033661	0.40839295	0.41363987	0.41928292	0.42540848	0.43225262	0.43962404	0.44780789	0.45596594	0.46446946	0.47285306	0.48032714	0.48729461	0.49362619	0.49922648	0.50392286	0.50851113	0.51309203	0.51764091	0.52147543	0.52524312
Rio de Janeiro	0.57716593	0.58073292	0.58356901	0.58618393	0.58873055	0.59157667	0.59498969	0.5987292	0.60298357	0.60769205	0.61300501	0.61835324	0.62369883	0.62844558	0.63356798	0.63859363	0.64363218	0.64869694	0.65412966	0.65890302	0.6640695	0.66959267	0.6749798	0.68021835	0.68514694	0.68975329	0.69393831	0.69841903	0.70321155	0.70811781	0.71223305	0.71609384

Table S5. Socio-demographic Index values for all estimated GBD 2021 locations, 1990-2021

Location	1990	1991	1992	1993	1994	1995	1996	1997	1998	1999	2000	2001	2002	2003	2004	2005	2006	2007	2008	2009	2010	2011	2012	2013	2014	2015	2016	2017	2018	2019	2020	2021
Rio Grande do Norte	0.41829153	0.42351775	0.42699588	0.43064853	0.43408436	0.43725132	0.44117713	0.44535534	0.44935527	0.45364305	0.45873082	0.46428238	0.47050124	0.47640276	0.48275443	0.48958579	0.49701859	0.50477026	0.51260986	0.52007218	0.52790584	0.53575974	0.54318056	0.55013798	0.55654885	0.56226331	0.56716165	0.57201278	0.57691059	0.58176686	0.58587668	0.58985482
Rio Grande do Sul	0.55690185	0.56058552	0.56412969	0.56785538	0.57143399	0.57367372	0.57617181	0.57912416	0.58257712	0.5867374	0.59177442	0.59721093	0.60286137	0.60844358	0.61397434	0.61918651	0.62451543	0.63026995	0.63616683	0.64153856	0.64721481	0.65241174	0.65712977	0.66170394	0.66604768	0.67015455	0.67381145	0.67766695	0.68176946	0.68604738	0.68972093	0.69333753
Rondônia	0.44984365	0.4540549	0.45657169	0.45884913	0.46078584	0.46287132	0.46566814	0.46933387	0.4735258	0.47839879	0.48414852	0.49035524	0.4974075	0.50509925	0.51331333	0.52168498	0.52935747	0.53721999	0.54554468	0.55363802	0.56199134	0.57060824	0.57832751	0.58536475	0.59173397	0.59738986	0.60224486	0.60706357	0.6119503	0.6167603	0.62087528	0.62485467
Roraima	0.47622098	0.48107887	0.48353106	0.4838484	0.48391868	0.48935587	0.49509622	0.50023842	0.50506233	0.51035871	0.51631806	0.5226118	0.5289284	0.53490412	0.54009252	0.54515053	0.55076404	0.55686254	0.56331634	0.56946522	0.57550591	0.58117384	0.58602361	0.59065211	0.59495541	0.59875076	0.60192626	0.60532266	0.60900961	0.61316143	0.61700942	0.62100557
Santa Catarina	0.54767163	0.55245081	0.55665665	0.5601993	0.56401257	0.5677413	0.57195993	0.57643937	0.5809412	0.58580791	0.59174644	0.59792221	0.60428927	0.61066548	0.61721484	0.6234999	0.62967057	0.63612891	0.64293118	0.64899146	0.65541699	0.66170105	0.66721635	0.67226091	0.67671186	0.68056338	0.68374263	0.68712054	0.69078787	0.69466464	0.69801177	0.7013407
São Paulo	0.56475845	0.56884995	0.5721516	0.57533428	0.57893532	0.58374583	0.58896242	0.59449103	0.5998756	0.60539284	0.61146726	0.61742224	0.62305869	0.62826422	0.63342469	0.63856599	0.64372872	0.64914092	0.6545714	0.65967818	0.66517002	0.67062106	0.67567899	0.68071911	0.68550619	0.68983514	0.69359848	0.69753702	0.70174714	0.70614456	0.70995406	0.7137276
Sergipe	0.44464477	0.45032769	0.45454991	0.45810966	0.46063027	0.46269499	0.46547878	0.46896629	0.47253387	0.47613094	0.48022488	0.48525471	0.49100615	0.49676668	0.50282255	0.50910864	0.51578591	0.52283265	0.53017827	0.53658309	0.54358193	0.55045065	0.55674883	0.56264961	0.56800955	0.57273835	0.57671048	0.5807074	0.58481942	0.58899082	0.59243863	0.59584018
Tocantins	0.38598803	0.38829043	0.38925325	0.39052515	0.39298922	0.39873167	0.4047504	0.41102044	0.41767479	0.42437851	0.43220813	0.4414859	0.45121971	0.46155584	0.4718882	0.48187005	0.49162077	0.50186296	0.51250914	0.522696	0.53302898	0.54252897	0.55132071	0.55961728	0.56732128	0.57440259	0.58065846	0.58681648	0.59287864	0.5988406	0.60386448	0.60858768
Paraguay	0.46952779	0.47435229	0.47931417	0.4846142	0.49022693	0.49633688	0.5023228	0.50828695	0.5138199	0.51872058	0.52300779	0.52691676	0.53117736	0.53536013	0.53955666	0.5436227	0.5479433	0.55275868	0.5580169	0.56290006	0.56884656	0.57495446	0.58065019	0.58724704	0.59395315	0.60048413	0.60701814	0.61358159	0.62003236	0.62607014	0.63105769	0.6357181
North Africa and Middle East	0.43742067	0.44592093	0.45397143	0.46210216	0.47039382	0.4785534	0.48646286	0.49398005	0.50144505	0.50895578	0.51682421	0.52409127	0.53114945	0.53829213	0.54567816	0.55304739	0.56008896	0.5665161	0.57233926	0.57706216	0.58184904	0.58759308	0.5941117	0.60112707	0.60841869	0.61577742	0.62314232	0.63056077	0.63791791	0.64509479	0.65167349	0.65822472
North Africa and Middle East	0.43742067	0.44592093	0.45397143	0.46210216	0.47039382	0.4785534	0.48646286	0.49398005	0.50144505	0.50895578	0.51682421	0.52409127	0.53114945	0.53829213	0.54567816	0.55304739	0.56008896	0.5665161	0.57233926	0.57706216	0.58184904	0.58759308	0.5941117	0.60112707	0.60841869	0.61577742	0.62314232	0.63056077	0.63791791	0.64509479	0.65167349	0.65822472
Afghanistan	0.17383217	0.17647255	0.17963372	0.18018371	0.17851866	0.17827913	0.1780854	0.17788412	0.1775134	0.17707524	0.17702577	0.17777314	0.18380843	0.19051899	0.19690354	0.20392813	0.21107419	0.21962761	0.22808917	0.23790082	0.24775995	0.25704168	0.26648441	0.27563656	0.28403035	0.2918495	0.2996307	0.30742462	0.31486609	0.32245405	0.32983007	0.3372
Algeria	0.46048691	0.4683192	0.47595094	0.48325559	0.49045066	0.49795754	0.50599097	0.51405657	0.52238242	0.53078558	0.53948151	0.54752928	0.55517222	0.56253696	0.56935285	0.57600196	0.58212636	0.58779054	0.59319097	0.59787038	0.60282353	0.60782553	0.61270353	0.6174222	0.62208729	0.62674557	0.63171074	0.63697318	0.64250004	0.64821078	0.65365147	0.65950092
Bahrain	0.58457885	0.5905969	0.59604023	0.60216562	0.60813372	0.61385072	0.61948538	0.62459606	0.6307383	0.63808319	0.6467507	0.65719606	0.6649012	0.66956411	0.67414778	0.6797906	0.68614429	0.69304515	0.69980639	0.70488584	0.70790262	0.70895874	0.71036242	0.71334316	0.71676806	0.7199266	0.7236113	0.7293231	0.73619258	0.7428478	0.74810308	0.7530432
Egypt	0.41718274	0.42690945	0.43774456	0.44849005	0.458727	0.46803537	0.47611663	0.48297089	0.48918407	0.49550262	0.50216989	0.50871682	0.51503813	0.5206234	0.52518168	0.52866548	0.53039581	0.52945759	0.52474383	0.51625583	0.50771548	0.50459404	0.50915707	0.51933446	0.53174981	0.54437015	0.55601247	0.56691463	0.57744279	0.58773688	0.59736334	0.60678709
Iran	0.45379994	0.46898286	0.4806267	0.49227703	0.50507286	0.51753149	0.52860072	0.53772778	0.54636602	0.55562433	0.56547456	0.57461821	0.58409727	0.59396301	0.60371745	0.61329106	0.6212681	0.62774944	0.63286223	0.63739838	0.64255917	0.64789909	0.65150059	0.65447529	0.6579615	0.66205799	0.66765245	0.67434346	0.68090962	0.68674166	0.69191876	0.6972074
Alborz	0.54040869	0.55244616	0.56172076	0.57136041	0.58199185	0.59235619	0.6019195	0.60979177	0.61716027	0.62475409	0.63279463	0.64006476	0.64754683	0.65527469	0.66295954	0.67055544	0.6770396	0.6825559	0.68702035	0.6910409	0.69581766	0.70090292	0.70454243	0.70798813	0.71207689	0.71648139	0.72214212	0.72849718	0.73437934	0.73937839	0.74376241	0.74830202
Ardebil	0.36072637	0.37879938	0.39300024	0.40775365	0.42454399	0.44117041	0.45601447	0.46850021	0.48059427	0.49381	0.50770823	0.52056048	0.53345248	0.54654539	0.55914828	0.57122281	0.58096928	0.58852021	0.59446442	0.59981496	0.60585685	0.61209234	0.61637548	0.61966722	0.62313981	0.6268815	0.63178915	0.63780159	0.64382993	0.64916853	0.65381615	0.65863977
Bushehr	0.4538396	0.46944879	0.48166242	0.49424656	0.50801352	0.52138512	0.53389066	0.5443784	0.55406983	0.56393067	0.57396415	0.58308205	0.59244592	0.60232832	0.61204096	0.62173823	0.63002304	0.63680753	0.64211431	0.64697555	0.65255811	0.65844479	0.66239921	0.66555968	0.66914772	0.67329909	0.6789001	0.68568407	0.69235058	0.69821121	0.70330496	0.70841871
Chahar Mahaal and Bakhtiari	0.39617743	0.41375284	0.42804194	0.4429338	0.45940876	0.47550171	0.49024461	0.50269138	0.51447497	0.52641467	0.53854403	0.54949036	0.56030347	0.57137699	0.58221791	0.59270157	0.60137153	0.60831844	0.61355128	0.61809149	0.62319591	0.62849679	0.63172724	0.63414164	0.63714608	0.64097387	0.64662885	0.65353781	0.66048041	0.66674984	0.67234977	0.6779886
East Azarbayejan	0.41107167	0.42856579	0.4413805	0.45343279	0.46643214	0.47887716	0.48960814	0.49839477	0.50690652	0.51633452	0.52643573	0.53582323	0.54556048	0.55555939	0.56532694	0.57470787	0.58229499	0.58822174	0.59281479	0.59702743	0.6020572	0.60751365	0.61166103	0.61544217	0.620065	0.62567214	0.63288757	0.64099987	0.64881945	0.65576851	0.6619758	0.66819323
Fars	0.45809653	0.47369369	0.48643907	0.49944313	0.51335231	0.52675456	0.53883797	0.5495049	0.55979745	0.57048609	0.58115914	0.59118372	0.60153883	0.61180187	0.62155638	0.63081941	0.63833705	0.64498069	0.65053186	0.65562818	0.66152584	0.66779732	0.67239882	0.67613073	0.68008035	0.68425814	0.6895716	0.69564725	0.70134214	0.70620755	0.71040186	0.71483077
Gilan	0.48712664	0.49946022	0.50870015	0.51805687	0.5280929	0.5378675	0.54686668	0.55462934	0.56217551	0.57020549	0.57887202	0.58701381	0.59569803	0.60481486	0.61396167	0.62314844	0.63152725	0.63926951	0.64585756	0.65180483	0.65813963	0.6643904	0.66883051	0.67267945	0.6768535	0.68124253	0.68686625	0.69308583	0.69878458	0.70363634	0.70788343	0.71231945
Golestan	0.40735274	0.42692675	0.44105996	0.45428653	0.4685542	0.48208155	0.49346702	0.50253299	0.51124636	0.52075827	0.53108678	0.5406348	0.55058163	0.56092596	0.57104193	0.58110741	0.58933407	0.59519379	0.59936676	0.6029337	0.60729219	0.61191122	0.61449407	0.61605468	0.61810864	0.62100968	0.62574684	0.63212231	0.63885318	0.64501391	0.65048709	0.65607753
Hamadan	0.39303783	0.41022029	0.42391272	0.4378005	0.45293889	0.46759097	0.48058656	0.49169276	0.5021933	0.51331166	0.52490645	0.5357609	0.54697637	0.55872245	0.57020542	0.58137325	0.59086598	0.59886027	0.60532807	0.61110702	0.61713502	0.62300822	0.62675037	0.62948069	0.63253792	0.63593216	0.64069585	0.64657251	0.6523723	0.65746758	0.66197252	0.66673482
Hormozgan	0.38090987	0.39854874	0.41252512	0.42686088	0.44282125	0.45852999	0.47260337	0.48461954	0.49624277	0.50874629	0.52171885	0.53348297	0.54516827	0.5572524	0.56951993	0.58145571	0.59111459	0.5982347	0.6032555	0.60744938	0.612072	0.61714823	0.62047072	0.62305076	0.62649556	0.63092541	0.63691823	0.64425644	0.65167453	0.6584113	0.66439043	0.67033137
Ilam	0.4096367	0.42851454	0.44390619	0.45971596	0.47669329	0.49295697	0.50781905	0.52057502	0.53269307	0.54578352	0.55953875	0.57259365	0.58594631	0.59960845	0.61244793	0.6244424	0.63492378	0.64446234	0.65269259	0.66003048	0.66714449	0.67356111	0.67740557	0.68004545	0.68245549	0.68473158	0.68837089	0.69282091	0.69689833	0.70022852	0.70302049	0.70619493
Isfahan	0.4879595	0.50249911	0.51375956	0.52465424	0.53593669	0.54664457	0.5563287	0.56452704	0.57231449	0.58050582	0.58908825	0.59704574	0.60532413	0.61395121	0.62256599	0.63124642	0.63909362	0.64623614	0.65227017	0.65777781	0.66359148	0.66930034	0.67319066	0.67640926	0.67978134	0.68315896	0.68767327	0.69295059	0.69786856	0.70206375	0.70572271	0.70971684
Kerman	0.43023124	0.44666959	0.45961309	0.47293905	0.48755725	0.50177046	0.51455066	0.5252289	0.53508084	0.54540598	0.55617564	0.56588313	0.57569564	0.58614764	0.59604588	0.60509009	0.61200228	0.61701861	0.6203251	0.62293311	0.62613935	0.62968143	0.63148626	0.63263676	0.6344102	0.63701352	0.64126343	0.64698714	0.6529345	0.65836188	0.66327078	0.66843927
Kermanshah	0.40406333	0.41894466	0.43067778	0.44296946	0.45667165	0.47016593	0.48291024	0.49331954	0.50287711	0.51276466	0.52332682	0.53336772	0.54406293	0.55564768	0.56743563	0.5790679	0.58910471	0.59778718	0.60498501	0.61158977	0.61865164	0.6257156	0.63073523	0.63475457	0.63877166	0.64288124	0.64803394	0.65412878	0.66001774	0.66516019	0.66970802	0.67446647
Khorasan-e-Razavi	0.40318232	0.41938805	0.4313797	0.44316534	0.45626454	0.46899144	0.48000785	0.4886858	0.49668773	0.50542073	0.51490729	0.5238786	0.5336534	0.54438878	0.55547648	0.56681338	0.57643632	0.58438061	0.59086725	0.5966024	0.60302374	0.6095685	0.6140323	0.61774605	0.62207644	0.62715662	0.63377622	0.64167964	0.64959811	0.65681776	0.66334337	0.66982664
Khuzestan	0.4117171	0.43032942	0.4440924	0.45705126	0.47101434	0.48425984	0.49504135	0.50351168	0.51158507	0.52077289	0.53104134	0.540748	0.55094375	0.56138294	0.57169178	0.58179572	0.58968845	0.59541384	0.59979113	0.60378766	0.60865116	0.61396507	0.61764073	0.6207112	0.62442376	0.62905901	0.63524247	0.64272055	0.65029543	0.65719997	0.66339721	0.66958791
Kohgiluyeh and Boyer-Ahmad	0.38544463	0.4030339	0.41664253	0.43071714	0.44654789	0.46222947	0.47612742	0.48776393	0.49903621	0.51107238	0.52355661	0.5353544	0.54776883	0.56086145	0.57385919	0.58642175	0.5966095	0.60489609	0.61149933	0.61765013	0.624879	0.63251482	0.63842728	0.64381055	0.64959119	0.65560125	0.66262008	0.6703172	0.67747087	0.68356697	0.68865258	0.6937062
Kurdistan	0.34632778	0.36244794	0.37544031	0.38862839	0.40296388	0.41696241	0.42968521	0.44047445	0.45090565	0.46191712	0.47371049	0.48522519	0.49764178	0.51107844	0.52453113	0.53764009	0.54886023	0.55845825	0.56649055	0.57375338	0.58125951	0.58865669	0.59407374	0.59857926	0.60324242	0.60802492	0.61391165	0.62056984	0.6269015	0.63241361	0.63723346	0.64226169
Lorestan	0.37269265	0.39183502	0.40713361	0.42251958	0.43932289	0.45564923	0.47010403	0.48200047	0.49341582	0.50566577	0.51879755	0.53142939	0.54443517	0.55766933	0.57051034	0.58266223	0.59258997	0.60040075	0.60660969	0.61209201	0.61775946	0.62330088	0.62683319	0.62950842	0.63256128	0.63628853	0.64145836	0.64770928	0.65385361	0.659311	0.664169	0.66911829
Markazi	0.43112644	0.44631843	0.45866664	0.47125167	0.48463155	0.49757988	0.5096906	0.5202388	0.53042509	0.54091661	0.55165623	0.56158775	0.57175965	0.58231232	0.59263399	0.60243907	0.61079517	0.61792865	0.62362005	0.62872574	0.63421755	0.63974771	0.64343683	0.64636712	0.64958868	0.65315602	0.6580912	0.66391587	0.66941126	0.6741577	0.67837742	0.68284382
Mazandaran	0.50422733	0.52030713	0.53216275	0.54317226	0.55443878	0.56498536	0.57415348	0.58140107	0.58772236	0.59430813	0.60136911	0.60818109	0.61615703	0.62565229	0.63572296	0.64607724	0.6553376	0.66354985	0.67026376	0.67607989	0.68194593	0.68754549	0.6912232	0.69416052	0.69731276	0.70078164	0.70577577	0.71153246	0.71691625	0.72153492	0.72560047	0.7299299
North Khorasan	0.34784205	0.36586324	0.37902068	0.39191539	0.40644773	0.42075366	0.43325316	0.4435523	0.45344414	0.46444135	0.47646473	0.48808215	0.50063279	0.5141521	0.52795859	0.54177227	0.55362013	0.56325539	0.57115498	0.57803034	0.58535378	0.59267629	0.59772944	0.60177242	0.60606771	0.61091752	0.61724259	0.62480784	0.63240584	0.63931158	0.64549311	0.6516102
Qazvin	0.41337461	0.42875875	0.43991072	0.45024635	0.46109912	0.47135693	0.48063107	0.48881906	0.49744318	0.50768098	0.5195944	0.53207715	0.54603257	0.56101473	0.57585077	0.59022113	0.60256638	0.61284177	0.62093951	0.62778342	0.63474628	0.64134344	0.64578637	0.64917743	0.65259076	0.65626529	0.661262	0.6672753	0.67316865	0.67833935	0.6829032	0.6876536
Qom	0.45262891	0.46572204	0.47580291	0.48642548	0.49844008	0.5103195	0.52109603	0.53002684	0.53828674	0.54697124	0.5560084	0.56442097	0.573137	0.5822297	0.59142963	0.60085109	0.60906901	0.61559525	0.62091434	0.62596655	0.63193756	0.63831677	0.64310698	0.64734331	0.6520977	0.65700564	0.66322942	0.67039789	0.67729151	0.6833538	0.68869247	0.69407591
Semnan	0.49608945	0.50557948	0.51270333	0.52076963	0.53021532	0.53984031	0.54941265	0.55799461	0.56663775	0.57572781	0.58526817	0.59426707	0.60359812	0.61339484	0.6230342	0.63250122	0.64080173	0.64805461	0.65418495	0.65985729	0.66614538	0.67269035	0.67749802	0.68199998	0.68687585	0.69177677	0.6977284	0.70432963	0.71030264	0.71528784	0.71953728	0.72392638
Sistan and Baluchistan	0.30884073	0.32607869	0.33889932	0.35173246	0.36658488	0.38115001	0.39305591	0.40195476	0.41001621	0.41950553	0.4298579	0.43874689	0.44740568	0.45628781	0.4653299	0.47454565	0.48059877	0.4829184	0.48320659	0.48305692	0.48456532	0.48722436	0.48829234	0.48878132	0.49069476	0.49500313	0.50203843	0.51167765	0.52247451	0.53282763	0.54220697	0.55130598
South Khorasan	0.35044613	0.36744059	0.38062956	0.39400221	0.4088528	0.42348866	0.43655345	0.44731287	0.45727079	0.46779298	0.47900871	0.48962953	0.5010653	0.51342883	0.52606123	0.5388346	0.54973374	0.55879773	0.56611706	0.57261367	0.57981909	0.58729861	0.59279322	0.59761989	0.60304895	0.60898831	0.61634647	0.62476791	0.63297067	0.64027843	0.64672361	0.65312538
Tehran	0.58761268	0.5974056	0.60500043	0.61307399	0.62213867	0.63111852	0.63950695	0.64642594	0.65297988	0.6598799	0.66735854	0.67430059	0.68149363	0.68901755	0.69649539	0.70389952	0.7103273	0.71589513	0.72041864	0.72443014	0.72898465	0.7336284	0.73690465	0.73985661	0.7432248	0.74690488	0.7518639	0.75762071	0.76300458	0.76764971	0.77174329	0.77603245
West Azarbayejan	0.37997815	0.39490202	0.40617076	0.41740854	0.42990115	0.44205872	0.45258118	0.46098646	0.46915774	0.47852181	0.48878611	0.49836255	0.50842601	0.51912335	0.53000796	0.54087308	0.54972823	0.55624435	0.56090299	0.56483796	0.56947461	0.57442046	0.57758425	0.58009813	0.58325168	0.58733977	0.59304459	0.60020635	0.60762044	0.61448487	0.62073248	0.62711246
Yazd	0.45422759	0.46880977	0.47987015	0.49157595	0.50502601	0.51851044	0.53070408	0.54095931	0.55073595	0.56128263	0.57230228	0.5821624	0.59202505	0.60198352	0.61180759	0.62171034	0.63017657	0.63719027	0.64266609	0.64757973	0.65319099	0.65912493	0.66313631	0.66646806	0.67028166	0.67485775	0.68107005	0.68846204	0.6956733	0.70205677	0.7076976	0.71334428
Zanjan	0.39131609	0.4088901	0.42093784	0.43209964	0.4443469	0.45610739	0.46585059	0.47326233	0.47968483	0.48694798	0.49513564	0.50343129	0.51348507	0.52535976	0.53835734	0.55220776	0.56492451	0.57577594	0.58434419	0.59152648	0.59889183	0.60624799	0.61159146	0.61603449	0.62063635	0.62547478	0.63161215	0.63866159	0.6454688	0.65140858	0.65653951	0.66172913
Iraq	0.41204417	0.41581412	0.42035359	0.42483493	0.42875173	0.43207555	0.43681848	0.44310403	0.45238409	0.46273644	0.4723764	0.48145832	0.48866474	0.49217444	0.49893388	0.50471943	0.51091805	0.51769897	0.52524291	0.53320091	0.54234328	0.55304856	0.56474547	0.576969	0.58831765	0.59949401	0.61150865	0.62267453	0.63323211	0.64363532	0.65330038	0.66262623
Jordan	0.53914747	0.54281941	0.54745885	0.55218235	0.55725433	0.56257154	0.56752966	0.57258151	0.57764357	0.5827592	0.58790939	0.5931828	0.59900038	0.60494595	0.6119344	0.61990923	0.62780381	0.63567028	0.64389269	0.65219632	0.65994949	0.66739668	0.67456892	0.68111996	0.68713404	0.69295203	0.6984733	0.70392383	0.70941288	0.71499895	0.72006824	0.72530723
Kuwait	0.6645179	0.66880962	0.67015911	0.67003625	0.67139321	0.67721552	0.6860933	0.69578939	0.70438374	0.71184133	0.71835756	0.72284063	0.72681698	0.73177868	0.73815371	0.74638491	0.75492849	0.76241148	0.77000261	0.77754655	0.78487393	0.79181207	0.79835699	0.80458383	0.81074323	0.81699841	0.82302653	0.82888699	0.83437146	0.83968831	0.84345981	0.84665105
Lebanon	0.53671897	0.53919749	0.54179352	0.54458131	0.54768403	0.55147172	0.55643616	0.56212082	0.56905907	0.57607853	0.58269828	0.5891855	0.59597879	0.60296724	0.61084576	0.61939136	0.62779122	0.63693823	0.64666766	0.65743319	0.66880452	0.68092062	0.69391098	0.704811	0.71242387	0.71869729	0.72458335	0.72996329	0.73474624	0.73864401	0.74200972	0.74474635

Table S5. Socio-demographic Index values for all estimated GBD 2021 locations, 1990-2021

Location	1990	1991	1992	1993	1994	1995	1996	1997	1998	1999	2000	2001	2002	2003	2004	2005	2006	2007	2008	2009	2010	2011	2012	2013	2014	2015	2016	2017	2018	2019	2020	2021
Libya	0.52799817	0.5414324	0.55446823	0.56679842	0.57865142	0.58957863	0.60035004	0.61062032	0.61997398	0.6286006	0.63704793	0.64468513	0.65166915	0.65934512	0.66691393	0.67532227	0.683702	0.69185513	0.69887325	0.70499847	0.7113625	0.71146666	0.71670069	0.71766525	0.71604221	0.71365137	0.71076014	0.71066626	0.71272044	0.71615724	0.72027039	0.7257714
Morocco	0.35807287	0.36498463	0.37118719	0.37677898	0.38284945	0.38816411	0.3941501	0.39950401	0.40503879	0.41030319	0.41534403	0.42059513	0.42577575	0.43121781	0.43682235	0.44253134	0.44867616	0.45503632	0.46180355	0.46892989	0.4762698	0.48392215	0.49160339	0.49952861	0.50744327	0.51558289	0.52351859	0.53161086	0.53972967	0.54783744	0.55524607	0.5626983
Oman	0.42927095	0.44226812	0.45773797	0.47499903	0.49351311	0.51319436	0.53324639	0.55431554	0.5744749	0.59239847	0.60842154	0.62216063	0.63516213	0.64724909	0.65762612	0.66640123	0.67535389	0.68505558	0.69495226	0.70309857	0.71068869	0.71826453	0.72645007	0.73343249	0.73874377	0.74359718	0.74879719	0.75378606	0.75917951	0.76452786	0.76885422	0.7733916
Palestine	0.40179221	0.40572715	0.41114687	0.41677965	0.42313667	0.42930703	0.43473137	0.44090178	0.44778242	0.45490969	0.4606815	0.4652991	0.46860026	0.47288173	0.47852732	0.48532059	0.49189041	0.49897843	0.50684948	0.51581584	0.52571191	0.53677763	0.54829543	0.55916348	0.56923988	0.57920426	0.58949277	0.59915719	0.60812486	0.61646684	0.62381002	0.63101167
Qatar	0.65120838	0.65533877	0.66067376	0.6661944	0.67202538	0.67832034	0.68483592	0.69310965	0.70140906	0.70888098	0.71483161	0.72083106	0.72711217	0.7335366	0.74022321	0.74718936	0.75412303	0.76096013	0.76765923	0.77427685	0.78080707	0.78721245	0.7934949	0.79961155	0.80572571	0.81187285	0.81797984	0.82401687	0.82996213	0.83580528	0.8414674	0.84686058
Saudi Arabia	0.53895452	0.54990772	0.56114427	0.57218691	0.58287887	0.59325142	0.60364842	0.61374912	0.62351821	0.63279645	0.64231177	0.65150209	0.66035686	0.66983958	0.67991786	0.69052085	0.70115261	0.71144214	0.7219602	0.73115849	0.74030067	0.74995945	0.75905494	0.76738786	0.77517813	0.7823008	0.78886788	0.79488629	0.80065698	0.80608784	0.81054186	0.81514349
Sudan	0.29217864	0.29647345	0.30086368	0.30540933	0.31009643	0.31520826	0.32068023	0.32676146	0.33308776	0.33975488	0.34687581	0.35421551	0.36172824	0.36969365	0.37792066	0.38668571	0.39615137	0.40607999	0.4160876	0.42597592	0.4361711	0.44723944	0.45679026	0.466436	0.47636213	0.48654944	0.49684822	0.50688353	0.51621409	0.52500912	0.53345542	0.54194974
Syria	0.43049264	0.43790749	0.44590424	0.45414786	0.4624373	0.47069204	0.47874309	0.48623235	0.49370799	0.50047487	0.50714369	0.51385011	0.52130974	0.52893576	0.53808447	0.54975045	0.56048476	0.57002812	0.57899049	0.58778912	0.59596776	0.60152446	0.6028726	0.60241013	0.60223576	0.60228291	0.60291118	0.60489637	0.60850716	0.61333684	0.61772759	0.62300407
Tunisia	0.47113852	0.47954306	0.48828528	0.49685204	0.50556488	0.51419694	0.52329427	0.53206404	0.54049816	0.54872456	0.55667265	0.56442101	0.57171953	0.57888267	0.58603259	0.59293776	0.59975851	0.60669678	0.61352932	0.62010683	0.62643277	0.63201984	0.63757834	0.64300851	0.6482974	0.65341354	0.65835352	0.66322285	0.668219	0.67317055	0.67761515	0.68243222
Türkiye	0.46160698	0.46924407	0.47681457	0.48465697	0.49161995	0.49882484	0.50623855	0.51393336	0.5215099	0.52851242	0.53586633	0.54235913	0.54902364	0.55610253	0.564024	0.57249589	0.58138318	0.59044239	0.59921206	0.60710462	0.61559723	0.62478721	0.63409709	0.64372382	0.65338883	0.66297174	0.67194815	0.68109627	0.68989587	0.69826377	0.70579974	0.71269267
United Arab Emirates	0.64441227	0.6607399	0.67592621	0.6894638	0.70165679	0.71313829	0.72418198	0.73472318	0.74447662	0.75325392	0.76096909	0.76785879	0.77434642	0.78114399	0.78852595	0.79642809	0.80553702	0.81487088	0.82279344	0.82830417	0.83128425	0.8329128	0.83358611	0.83380382	0.83385873	0.8341755	0.83522071	0.83721355	0.83990014	0.84298533	0.84615198	0.84931773
Yemen	0.21566459	0.22288541	0.23041505	0.23802525	0.24587943	0.25396257	0.26227861	0.27078254	0.27950051	0.2882613	0.29714516	0.30611242	0.31506502	0.32408169	0.33337129	0.34346256	0.35347295	0.36330122	0.37314029	0.38276225	0.39293664	0.4014718	0.40956135	0.4176424	0.42518967	0.43019779	0.43423298	0.43754916	0.44072799	0.4440014	0.44689307	0.45037638
South Asia	0.31979652	0.32585413	0.33194096	0.33801338	0.3443229	0.35072645	0.35728657	0.36371922	0.37028758	0.37697932	0.38338846	0.38953334	0.39519407	0.40098342	0.40718055	0.41398666	0.42136736	0.42926185	0.43701186	0.44519735	0.45407579	0.46333927	0.47308056	0.48343427	0.49408436	0.50489146	0.515311	0.52512844	0.53447741	0.5432457	0.55058557	0.55786466
South Asia	0.31979652	0.32585413	0.33194096	0.33801338	0.3443229	0.35072645	0.35728657	0.36371922	0.37028758	0.37697932	0.38338846	0.38953334	0.39519407	0.40098342	0.40718055	0.41398666	0.42136736	0.42926185	0.43701186	0.44519735	0.45407579	0.46333927	0.47308056	0.48343427	0.49408436	0.50489146	0.515311	0.52512844	0.53447741	0.5432457	0.55058557	0.55786466
Bangladesh	0.22854893	0.23720741	0.24524796	0.25209072	0.25899729	0.26529392	0.27117778	0.27777328	0.28451594	0.29095143	0.29725198	0.30318855	0.30864009	0.31436004	0.32092285	0.32809051	0.3356906	0.34393255	0.35252003	0.36148151	0.37059896	0.38012489	0.39062355	0.40170648	0.41326317	0.42548212	0.43770991	0.44975241	0.46171775	0.473265	0.48307917	0.49242088
Bhutan	0.21503985	0.22131244	0.22814827	0.23600469	0.24433125	0.25329196	0.26236275	0.27148742	0.28044038	0.28951049	0.29880487	0.30820065	0.31794354	0.32767042	0.33728649	0.34699858	0.35682421	0.3675605	0.37823354	0.38897716	0.39975194	0.41014925	0.41944322	0.42779067	0.43496927	0.44181075	0.44840185	0.4542805	0.45962471	0.46463335	0.46871448	0.47306238
India	0.3325936	0.33859178	0.34459173	0.3506854	0.35699603	0.3634185	0.37001864	0.37635876	0.38281785	0.38945389	0.39575233	0.40183747	0.40741141	0.41311578	0.41918496	0.42591306	0.43332156	0.44135146	0.44923585	0.45767233	0.467019	0.47683291	0.48721088	0.49831798	0.5097221	0.52124272	0.53222002	0.54238851	0.55193033	0.56080993	0.56813815	0.57540165
Nepal	0.19956065	0.20568744	0.21211309	0.21884343	0.22607306	0.23325315	0.24070062	0.2483828	0.25622718	0.26435918	0.27271911	0.28128747	0.28951361	0.2977726	0.30619653	0.3146618	0.32305635	0.33144561	0.34015071	0.34870189	0.3568681	0.3646426	0.37243759	0.38002233	0.38768909	0.39502115	0.40162878	0.40846387	0.41523668	0.42200725	0.42741709	0.43317463
Pakistan	0.31046762	0.31626567	0.32224805	0.32821925	0.33443313	0.3409263	0.3476562	0.35421863	0.36087534	0.3676414	0.37431913	0.38059393	0.38662999	0.39271926	0.39908417	0.40566744	0.41223483	0.41851976	0.4243482	0.43001983	0.43564066	0.44145559	0.4470966	0.4528231	0.45878142	0.46479267	0.47102217	0.47754414	0.48432613	0.49102298	0.49736441	0.50402869
Azad Jammu & Kashmir	0.3258571	0.33307721	0.34042907	0.34770421	0.3551887	0.36290103	0.37081185	0.37850927	0.38622689	0.39394301	0.40149838	0.40860326	0.41545557	0.42235537	0.42958201	0.43704644	0.44466119	0.45231842	0.45978972	0.46714764	0.47426164	0.48132564	0.48792453	0.49423462	0.50053617	0.50665208	0.51270136	0.51879497	0.52499004	0.53093598	0.53639063	0.54211877
Balochistan	0.24883029	0.25386723	0.25913746	0.26444552	0.27003226	0.27580896	0.28165795	0.28728496	0.29293677	0.29861592	0.3041235	0.30936329	0.31448679	0.31976754	0.32540818	0.33135828	0.33731296	0.34294716	0.34816028	0.35325682	0.35839244	0.36376712	0.36895028	0.37414089	0.37949329	0.38469183	0.38988999	0.39534393	0.4010568	0.40675045	0.41216125	0.4179715
Gilgit-Baltistan	0.22210367	0.22764097	0.23326191	0.23877023	0.24441432	0.25016309	0.25632411	0.2623317	0.268435	0.27461903	0.2806372	0.28644967	0.29209912	0.29789534	0.30407231	0.31063787	0.31745107	0.32422086	0.33074442	0.33714888	0.34333231	0.34956674	0.35526468	0.3605803	0.36562052	0.37025268	0.37486591	0.37949799	0.38426318	0.38884361	0.39301978	0.39754999
Islamabad Capital Territory	0.48715087	0.49411139	0.5010706	0.50780845	0.51467635	0.52176153	0.52911339	0.53621266	0.54330233	0.55040588	0.55742471	0.56408607	0.5705217	0.57701613	0.58382639	0.59105605	0.5985292	0.60613854	0.61359788	0.62095494	0.62811281	0.63527361	0.64200777	0.64843124	0.65479537	0.66093533	0.6670449	0.67316109	0.67934485	0.68524364	0.69059481	0.69619537
Khyber Pakhtunkhwa	0.26790174	0.27252131	0.27727892	0.28209586	0.28720818	0.29266462	0.29836347	0.30393949	0.30961095	0.31538685	0.32101648	0.32620678	0.33115461	0.336239	0.34175944	0.34772621	0.35399984	0.36051255	0.36699385	0.37353807	0.3799994	0.38656624	0.39283876	0.39899187	0.40528866	0.41149302	0.41772104	0.42407868	0.43070554	0.43734857	0.44373658	0.45058115
Punjab	0.31604994	0.32222957	0.32863199	0.33504202	0.34172676	0.34874026	0.35603949	0.36311293	0.37027953	0.3775739	0.38480276	0.39161443	0.39816643	0.40472799	0.41152918	0.41853623	0.42554756	0.43229033	0.43853633	0.44455107	0.45044463	0.45646969	0.46227533	0.46818561	0.47427629	0.48043288	0.48684477	0.49355939	0.50047832	0.50724092	0.51358412	0.52020049
Sindh	0.32706105	0.33255951	0.33817075	0.34371207	0.34942375	0.35531416	0.36135517	0.36734198	0.37344264	0.3796483	0.38578503	0.39154363	0.3970977	0.40275713	0.40870602	0.41484554	0.42084535	0.42634357	0.43126977	0.43606369	0.44097512	0.44624365	0.4515419	0.45706338	0.46304727	0.4692352	0.47578641	0.48279361	0.49024318	0.49775152	0.50498481	0.51257496
Southeast Asia, east Asia, and Oceania	0.47103694	0.47931819	0.48734562	0.49528092	0.50297128	0.51116746	0.52051322	0.52960263	0.53725808	0.54471189	0.55128675	0.55646847	0.56247943	0.56954356	0.57706574	0.58501175	0.59352872	0.60222627	0.61040808	0.61839042	0.62814353	0.63636052	0.64172537	0.64735119	0.6527504	0.65681903	0.6616056	0.66891988	0.67660333	0.68470468	0.69221505	0.6988499
East Asia	0.47117916	0.47993558	0.4884314	0.49685947	0.50488588	0.51353027	0.5237577	0.53381551	0.54265255	0.55136703	0.55881567	0.56423475	0.57079838	0.57890473	0.58768267	0.59709617	0.60727342	0.61752384	0.6269558	0.63627115	0.64798501	0.65750207	0.66292987	0.66868191	0.67406275	0.67730686	0.68152833	0.68974757	0.69862583	0.70832781	0.71770931	0.7257049
China	0.45866894	0.46730197	0.47578403	0.48435383	0.49257864	0.50150091	0.512034	0.52232687	0.53137948	0.54033369	0.54798919	0.55368037	0.56054294	0.56901883	0.57817809	0.58805219	0.59875817	0.60952025	0.61945483	0.62930773	0.64152144	0.65144798	0.65714429	0.66310301	0.66864085	0.67194011	0.67621844	0.68461966	0.693718	0.70368682	0.71336458	0.72162976
North Korea	0.49778013	0.49912051	0.49962984	0.49970065	0.49953134	0.49908053	0.49842901	0.49713687	0.49615921	0.49648475	0.49746199	0.49967404	0.50263357	0.50616524	0.51022913	0.51509776	0.51974144	0.52406697	0.5288135	0.53311441	0.53719292	0.54121227	0.54533171	0.5494833	0.55360356	0.55732127	0.56074356	0.56338829	0.56516148	0.56676571	0.56818855	0.56985463
Taiwan (province of China)	0.66763385	0.67653724	0.68480364	0.69292413	0.70107875	0.70962682	0.71912842	0.7291455	0.73812579	0.7453384	0.75292684	0.76082981	0.76900898	0.7769558	0.78467798	0.79244513	0.80017467	0.80813769	0.81540069	0.82143228	0.82722675	0.83206407	0.83667766	0.84128046	0.84569556	0.85005527	0.85446604	0.85888802	0.86315836	0.86741185	0.87124795	0.87474705
Oceania	0.39119508	0.39441882	0.39757226	0.4011131	0.40489236	0.40850725	0.41243707	0.41589313	0.41922484	0.42252053	0.42506966	0.42700403	0.42853491	0.43005967	0.43158133	0.43315184	0.4345841	0.43610175	0.43739244	0.43880798	0.44075187	0.44261777	0.4445244	0.44659351	0.44936352	0.45239726	0.45545381	0.45837779	0.4608181	0.46333393	0.46553464	0.46744513
American Samoa	0.61363392	0.61584761	0.61858365	0.6220786	0.62605324	0.62981405	0.63270698	0.63492669	0.63659798	0.63832219	0.64060955	0.64321442	0.64600206	0.64895196	0.65192114	0.65506101	0.65772572	0.66078118	0.66402576	0.66730053	0.67124892	0.67597929	0.68086154	0.68583306	0.69134314	0.69716291	0.7027264	0.70764791	0.71280059	0.71730813	0.7208385	0.72372753
Cook Islands	0.56451485	0.57062278	0.57712347	0.58405918	0.59154087	0.59878997	0.60641415	0.61399156	0.62120223	0.62802903	0.63536561	0.64442144	0.65279049	0.66146504	0.66876968	0.6740041	0.680644	0.68750519	0.69559207	0.70377698	0.71186161	0.71944467	0.72696339	0.73331702	0.7395957	0.7465503	0.75235309	0.75823357	0.76404118	0.76979591	0.77470058	0.77910995
Fiji	0.53464891	0.53877826	0.54318855	0.54777647	0.55284902	0.55853097	0.56504188	0.57118775	0.57717235	0.58357394	0.58919663	0.59449742	0.59932203	0.60348962	0.60754489	0.61081294	0.61393277	0.61658292	0.61895881	0.62088347	0.62330879	0.62643825	0.62988133	0.63427811	0.63939007	0.64502635	0.65074268	0.65646401	0.66199145	0.66706098	0.67143149	0.67505163
Guam	0.67622031	0.66997429	0.66635634	0.66731818	0.67211171	0.67812022	0.68417373	0.69168487	0.70181929	0.71364159	0.72480236	0.73426738	0.74128425	0.74591153	0.74901652	0.75086587	0.75117625	0.75262547	0.75515241	0.7582523	0.76148432	0.76398739	0.76684598	0.77022496	0.77344168	0.77672361	0.78051164	0.78555492	0.79129477	0.79677545	0.80096947	0.8039822
Kiribati	0.41038982	0.4128842	0.41557761	0.41848166	0.42171622	0.42502435	0.42830068	0.43164194	0.4357419	0.43969292	0.44443634	0.4487823	0.4534623	0.45820837	0.46233621	0.46672228	0.4705572	0.47422745	0.47720193	0.4800356	0.48244769	0.48502821	0.48817586	0.49188077	0.49531724	0.5000597	0.50520784	0.51001924	0.51481038	0.51944132	0.52345411	0.52718658
Marshall Islands	0.43083929	0.43557141	0.44049102	0.44547908	0.45084822	0.45685967	0.46120943	0.46475273	0.46813303	0.47129273	0.47456254	0.47858832	0.48277626	0.48624862	0.48957308	0.49327139	0.49745617	0.50225004	0.50635959	0.51079454	0.51608514	0.52117709	0.52604202	0.5314463	0.53664013	0.54179317	0.54678294	0.55206515	0.55759928	0.56342875	0.56883751	0.57409113
Federated States of Micronesia	0.46251162	0.46814483	0.47379294	0.4799396	0.48530001	0.49101519	0.49557031	0.49911011	0.50286156	0.50666343	0.51100423	0.51531681	0.51954567	0.5238296	0.52761096	0.53170332	0.53571868	0.53943146	0.54276635	0.5462755	0.55012255	0.55424041	0.55796585	0.56108282	0.56384003	0.56715134	0.5704581	0.57403983	0.57755508	0.58119525	0.58452723	0.58753497
Nauru	0.53914598	0.53843356	0.53703889	0.53505974	0.5330011	0.53089093	0.52854447	0.52624159	0.5238853	0.52189432	0.52025489	0.51923542	0.51843871	0.51845999	0.51880954	0.51989286	0.52267318	0.52320135	0.5260841	0.53022109	0.53568999	0.54234274	0.54989062	0.56011291	0.57142436	0.58128323	0.59010299	0.59761908	0.60531188	0.61269725	0.61933008	0.62517783
Niue	0.58753298	0.59388206	0.59988124	0.60551478	0.61065018	0.61537461	0.61973455	0.62391385	0.62757893	0.63085717	0.63406895	0.63736663	0.64112202	0.64577954	0.65061483	0.65728126	0.66416298	0.6701993	0.67639686	0.68257917	0.68628063	0.69031558	0.69415095	0.69722108	0.70054592	0.70423298	0.7074761	0.711221	0.71510823	0.71907889	0.72279025	0.72622205
Northern Mariana Islands	0.70859384	0.71222364	0.71578761	0.7191794	0.72251049	0.72621905	0.73007409	0.73379537	0.73742313	0.74093482	0.74449287	0.74717061	0.74875519	0.75017153	0.75152884	0.75182669	0.75179781	0.75160842	0.75080719	0.74859717	0.74661726	0.74441191	0.74293735	0.74228436	0.74260545	0.7437974	0.74845547	0.75649548	0.7612554	0.76577065	0.76942766	0.77153521
Palau	0.66290951	0.66823073	0.67330246	0.67664872	0.68019577	0.68483214	0.69009567	0.69512212	0.69965291	0.70311532	0.70519694	0.70740239	0.70958764	0.71100022	0.71254381	0.71433983	0.71635343	0.71880954	0.72080662	0.72228827	0.72406596	0.72673345	0.7296129	0.73195347	0.7347683	0.73816643	0.74136191	0.74422477	0.74776125	0.75071531	0.75278093	0.75404693
Papua New Guinea	0.31066863	0.31496731	0.31953026	0.32500598	0.33066447	0.33542129	0.3403487	0.34425832	0.34780141	0.35125062	0.35413579	0.35658899	0.35857209	0.36073682	0.36282754	0.36529496	0.36782676	0.37079415	0.37342621	0.37652828	0.38028678	0.38358754	0.38678103	0.38992644	0.39400457	0.39828586	0.40240727	0.40615344	0.40916508	0.41231115	0.41518688	0.41779744
Samoa	0.48749143	0.49098715	0.49402745	0.49707926	0.49948484	0.50236973	0.50577803	0.50909638	0.51236757	0.51540905	0.51890233	0.52297163	0.52724267	0.53160374	0.53600328	0.54055791	0.54481207	0.54911075	0.5531646	0.55647402	0.55962291	0.56289157	0.56532039	0.56744854	0.56949366	0.57199898	0.57540205	0.57892544	0.58232066	0.5862567	0.5900753	0.59339277
Solomon Islands	0.30121717	0.30583067	0.3114317	0.31715109	0.32330025	0.32996856	0.33620805	0.34183563	0.34713506	0.35186719	0.35479914	0.35662675	0.35791012	0.35942436	0.36129056	0.36358002	0.3664437	0.36982795	0.37376488	0.37782103	0.38271759	0.38805534	0.39300464	0.39790625	0.40224474	0.4062339	0.41040696	0.41467639	0.41888922	0.42273639	0.42611243	0.42936032
Tokelau	0.52194239	0.52662847	0.53130785	0.53593501	0.54052366	0.54529563	0.55028265	0.55563133	0.56104761	0.56641541	0.57181437	0.5769156	0.58185832	0.58685435	0.59234244	0.59817612	0.60442939	0.61116324	0.61794025	0.62407008	0.63017554	0.63601652	0.64161199	0.64796615	0.653232	0.65879899	0.66397432	0.66905413	0.67417951	0.67924784	0.68293738	0.68642562
Tonga	0.49180684	0.49816621	0.50365985	0.50890445	0.51420783	0.52021502	0.52580814	0.53100909	0.53617231	0.54131702	0.5463032	0.55105425	0.55600835	0.56073607	0.56480638	0.56844917	0.57161987	0.57435151	0.57730542	0.57917075	0.5806573	0.58265672	0.58477088	0.58735114	0.59082685	0.59496575	0.60034367	0.60624359	0.61195394	0.61732406	0.62210446	0.62634994
Tuvalu	0.40624757	0.41481994	0.42290198	0.43051826	0.43856305	0.44496224	0.44980498	0.45536843	0.46245468	0.4688458	0.47495682	0.48085089	0.48725225	0.49275471	0.49769215	0.50185131	0.50597541	0.5105043	0.5156116	0.51987057	0.52345551	0.52774424	0.53120323	0.53516623	0.53912224	0.54404788	0.54917393	0.55446648	0.5598594	0.56604549	0.5716661	0.57662053
Vanuatu	0.35310025	0.35700538	0.3608933	0.36444416	0.368501	0.37247913	0.37646088	0.38066717	0.3846902	0.38830053	0.39215835	0.39532506	0.39772378	0.40039646	0.4032261	0.40648857	0.41050173	0.41491558	0.41985324	0.42485465	0.42965805	0.43428304	0.438662	0.44272018	0.4466873	0.45032986	0.45407276	0.45801683	0.46208022	0.46627616	0.47003839	0.47310071
Southeast Asia	0.46410364	0.47174987	0.47949041	0.48734942	0.49534811	0.50335114	0.51123253	0.51866415	0.52443938	0.52966778	0.53456661	0.53901043	0.54347855	0.54805101	0.55270742	0.5573913	0.56230217	0.56765426	0.57320839	0.57849739	0.58426847	0.59041462	0.59678029	0.60314492	0.60943868	0.61568907	0.62183986	0.62797813	0.63409588	0.64010676	0.64506966	0.6497773

Table S5. Socio-demographic Index values for all estimated GBD 2021 locations, 1990-2021

Location	1990	1991	1992	1993	1994	1995	1996	1997	1998	1999	2000	2001	2002	2003	2004	2005
Cambodia	0.28907506	0.2924792	0.29690095	0.3016272	0.30572259	0.31067888	0.31563097	0.32050375	0.3249743	0.33022425	0.33607503	0.34250791	0.34946571	0.35692966	0.36485464	0.37333181
Indonesia	0.45713495	0.46650558	0.47575316	0.48477524	0.4936071	0.50200838	0.51010815	0.51810219	0.5240456	0.52917633	0.53378959	0.53797798	0.54224568	0.54655026	0.55090231	0.55542491
Aceh	0.48267308	0.49350426	0.50372524	0.51329353	0.52219444	0.53024485	0.53778589	0.54526999	0.55097646	0.55586759	0.56026794	0.56438388	0.56872189	0.57307457	0.57772252	0.58230388
Bali	0.45865501	0.46884804	0.47884452	0.48870046	0.49823275	0.50706076	0.51523109	0.52299363	0.52835559	0.53262285	0.53616426	0.53927741	0.54257347	0.54593923	0.54936586	0.55303279
Bangka-Belitung Islands	0.45264677	0.46259192	0.47239148	0.48188828	0.49104859	0.49948181	0.5073679	0.51494633	0.52054382	0.5252606	0.52892114	0.53212382	0.53552897	0.53917523	0.54298703	0.54698955
Banten	0.45096593	0.46078495	0.47063749	0.48025382	0.48959495	0.49835638	0.50682149	0.51493441	0.5205915	0.52508329	0.52859153	0.53158023	0.53463584	0.53775383	0.54095116	0.54436088
Bengkulu	0.41061859	0.42133881	0.43188088	0.44194522	0.45177497	0.4609698	0.46969509	0.47820296	0.48429071	0.48926872	0.49336859	0.49684776	0.50031399	0.50391788	0.50767793	0.51176383
Gorontalo	0.38988813	0.39714096	0.40452793	0.411806	0.41894345	0.4256583	0.43220747	0.43848086	0.44209189	0.44455879	0.44602471	0.44716892	0.44876276	0.45080182	0.45329135	0.45642028
Jakarta	0.60333738	0.61230558	0.62148036	0.63101074	0.64050786	0.64953473	0.65824441	0.66673238	0.67297262	0.67846727	0.68357742	0.68836419	0.6932305	0.69805222	0.70277371	0.70751587
Jambi	0.42398626	0.43408631	0.44383582	0.45305108	0.46198594	0.47055152	0.47894171	0.48736579	0.49358512	0.49887756	0.50341566	0.50758427	0.51194615	0.51647752	0.52114362	0.5258922
West Java	0.45336233	0.46290042	0.47222742	0.4812603	0.49007793	0.49842083	0.50635713	0.51402432	0.51946385	0.52382959	0.52755512	0.53072386	0.5339323	0.53721964	0.54068547	0.54455824
Central Java	0.41842687	0.42727032	0.43605899	0.44452502	0.45277668	0.46075536	0.46848922	0.47604762	0.48139323	0.48599492	0.49003761	0.49375055	0.49769922	0.50190521	0.50636632	0.51119198
East Java	0.43934538	0.44800253	0.45656865	0.4649814	0.47336271	0.48155471	0.48962517	0.49773415	0.50389921	0.50946165	0.51482937	0.51994422	0.52515545	0.53039257	0.53563368	0.54100965
West Kalimantan	0.39242094	0.40354693	0.41455971	0.42518101	0.43559683	0.44559281	0.45513546	0.46436313	0.47128992	0.47697509	0.48143807	0.48499739	0.48839009	0.49165766	0.49474646	0.4975574
South Kalimantan	0.4410948	0.45065933	0.46018627	0.46958769	0.47871541	0.48726002	0.49536063	0.50319087	0.50876176	0.51339747	0.51724759	0.5204995	0.52380167	0.52709316	0.53026868	0.53339914
Central Kalimantan	0.45881647	0.46869481	0.47885225	0.48908016	0.49915978	0.50878541	0.51785993	0.52616073	0.53164278	0.53561783	0.53838456	0.54053222	0.5429412	0.5454602	0.54791457	0.55040024
East Kalimantan	0.57046405	0.57970907	0.58815567	0.59575493	0.60314455	0.61001293	0.61667543	0.62354796	0.62922239	0.63426767	0.63879647	0.64279042	0.64685469	0.6511169	0.65555905	0.66020366
North Kalimantan	0.55530819	0.56344465	0.57133525	0.57892582	0.58639615	0.59339167	0.60026322	0.60735223	0.61341745	0.61905216	0.62429697	0.6292073	0.63436734	0.63974222	0.64531857	0.65115801
Riau Islands	0.57319537	0.58260415	0.59169803	0.60039852	0.60890987	0.61700569	0.62486876	0.63252718	0.63822439	0.6429552	0.64675899	0.64991663	0.65299036	0.65606106	0.65911176	0.66231984
Lampung	0.39163143	0.40189365	0.41222917	0.4223729	0.4324207	0.44226627	0.45188518	0.46129907	0.46832546	0.47438466	0.47950514	0.4838987	0.48810632	0.49217245	0.49620385	0.50041727
Maluku	0.47640259	0.48498838	0.49348521	0.50141601	0.50877279	0.51504198	0.52042485	0.52492807	0.52658782	0.52595923	0.52298834	0.51852653	0.51349154	0.50800674	0.5022141	0.49670039
North Maluku	0.37296561	0.3821621	0.39183111	0.40169747	0.41183439	0.42175935	0.4314559	0.44083814	0.44715694	0.45223301	0.45598959	0.45832082	0.45989066	0.46091262	0.46163489	0.46238837
West Nusa Tenggara	0.33727977	0.34859644	0.36028142	0.3721195	0.3838879	0.39520338	0.40617395	0.41698739	0.42546344	0.43319311	0.44070553	0.44798776	0.4552512	0.46240815	0.46962199	0.47675744
East Nusa Tenggara	0.36558804	0.37339094	0.38136121	0.38919136	0.39697485	0.40450521	0.41180164	0.41880041	0.42270241	0.4257347	0.42845105	0.43095467	0.4338165	0.43696531	0.44029416	0.44383243
Papua	0.46213856	0.46988952	0.47772002	0.48572292	0.49373091	0.50155954	0.50924694	0.51689711	0.52306207	0.52822232	0.53252213	0.53609896	0.53928483	0.5420573	0.54444202	0.54693318
West Papua	0.45730664	0.46623393	0.47529576	0.48422116	0.49299674	0.50106979	0.50870087	0.51604117	0.52131663	0.52556815	0.52885419	0.53153173	0.53433132	0.53743263	0.54079912	0.54472282
Riau	0.52781292	0.53900316	0.54937091	0.55866629	0.56722407	0.57476045	0.58177547	0.58895294	0.59478333	0.60004246	0.60519772	0.6100163	0.61472702	0.61896732	0.62276314	0.62626858
West Sulawesi	0.38816917	0.39645614	0.40460363	0.41233286	0.41983909	0.42688334	0.43368485	0.44045657	0.44497837	0.44842902	0.45086369	0.45264887	0.4545774	0.45667968	0.45898469	0.46174285
South Sulawesi	0.41327701	0.42271782	0.43209604	0.44123092	0.45020513	0.4587767	0.46711216	0.47542219	0.48160197	0.48690734	0.49163905	0.49594103	0.50036845	0.50482569	0.50923435	0.51364434
Central Sulawesi	0.4118575	0.42082313	0.42982927	0.43882523	0.44787772	0.4563719	0.4643591	0.47224867	0.47801563	0.48302239	0.48736392	0.49125871	0.49536197	0.49950147	0.50362144	0.50768352
Southeast Sulawesi	0.38693152	0.39743305	0.40767444	0.41753534	0.42734876	0.43685672	0.44611154	0.45527829	0.46221734	0.46803577	0.47301416	0.47752407	0.4823181	0.48736429	0.49261118	0.49832063
North Sulawesi	0.47969291	0.48708994	0.49460044	0.50230088	0.51008862	0.51771279	0.52535874	0.53278829	0.53761274	0.54107977	0.54363949	0.54556523	0.54751292	0.54952838	0.55169202	0.55419005
West Sumatra	0.46521023	0.47432421	0.48333304	0.49227772	0.50105818	0.509331	0.51729881	0.52517652	0.5308862	0.53573501	0.53988665	0.54357323	0.54745489	0.55149686	0.55570819	0.56023794
South Sumatra	0.45624876	0.46582972	0.47479025	0.48295831	0.49069695	0.49794121	0.50502407	0.51230621	0.51787534	0.52247358	0.526408	0.52982247	0.53328289	0.53690512	0.54067667	0.54476334
North Sumatra	0.46994797	0.47915991	0.48843197	0.49751008	0.50639027	0.51446984	0.52210997	0.52981071	0.53543833	0.54025256	0.5441669	0.54752018	0.55114495	0.55502277	0.55907586	0.56327535
Yogyakarta	0.46358844	0.47368078	0.48426299	0.49551371	0.50692616	0.51791318	0.52817551	0.53764889	0.5440565	0.54939504	0.55391417	0.55777964	0.56164756	0.56558187	0.56967347	0.57403804
Laos	0.26428316	0.26878762	0.27343531	0.27814042	0.28327023	0.28879534	0.29483837	0.30147797	0.30840254	0.31597262	0.32394736	0.33258041	0.34166727	0.35065818	0.35968176	0.36872868
Malaysia	0.54579941	0.55168355	0.55818675	0.56584566	0.57438045	0.5834411	0.59315214	0.60341811	0.61264541	0.6217249	0.63052354	0.63825748	0.64504425	0.65088568	0.65624333	0.66124224
Maldives	0.33160154	0.34439861	0.35825234	0.372807	0.3880816	0.40394558	0.42051913	0.43743603	0.45397336	0.46933807	0.48349567	0.4959237	0.50740486	0.51892686	0.52991019	0.53825333
Mauritius	0.54458653	0.54820912	0.55345463	0.56068864	0.56909217	0.57731939	0.58479706	0.59149773	0.59736304	0.60203423	0.6070879	0.61224082	0.61719001	0.62276453	0.62903019	0.63476658
Myanmar	0.31921972	0.32203472	0.32562888	0.32970743	0.33429012	0.33959098	0.34519164	0.35089288	0.3566969	0.36303773	0.37036002	0.37817198	0.38634363	0.39521877	0.40469236	0.41466208
Philippines	0.5100118	0.51370718	0.5171095	0.52060038	0.52461361	0.52882791	0.53293885	0.53658987	0.53949647	0.54225797	0.54499772	0.54740943	0.54952115	0.55156797	0.55370429	0.5556641
Abra	0.49311416	0.49824011	0.50287753	0.50746804	0.51224072	0.51691409	0.52114351	0.52462794	0.52718849	0.52935087	0.53128071	0.53273718	0.53376801	0.53478051	0.53592088	0.53697419
Agusan Del Norte	0.48847885	0.49175867	0.49459271	0.497382	0.50052054	0.50369509	0.50664144	0.50897915	0.51038441	0.51155749	0.51259648	0.51320465	0.5134108	0.51351357	0.51379208	0.51394635
Agusan Del Sur	0.39435843	0.3989436	0.40309912	0.40725679	0.41180822	0.41635105	0.42060562	0.42413312	0.426757	0.42915898	0.43140784	0.43320888	0.43462048	0.43597421	0.43742142	0.43874005
Aklan	0.50014398	0.50300517	0.5056726	0.5085199	0.51197749	0.51577454	0.51959261	0.52299977	0.52553172	0.52790303	0.53022348	0.53217388	0.53385301	0.53563188	0.53776595	0.53993197
Albay	0.48867363	0.49157791	0.49429255	0.49716773	0.50076741	0.50478604	0.50882488	0.5124535	0.51536435	0.51814019	0.52093014	0.52339654	0.52560853	0.52788142	0.53042267	0.53292506
Antique	0.4017761	0.4080367	0.41374071	0.41936131	0.42536116	0.43133549	0.43681794	0.44154427	0.44529913	0.44869387	0.45178835	0.45429256	0.45623893	0.45810206	0.45999414	0.46163724
Apayao	0.43566095	0.44053422	0.44499167	0.44948029	0.45449777	0.45973585	0.46488663	0.46958684	0.47345454	0.47718474	0.48086003	0.48416371	0.48716531	0.49018527	0.4935853	0.4970595
Aurora	0.45173852	0.45695075	0.46184658	0.46678198	0.47225426	0.47790609	0.48333857	0.48814952	0.49201394	0.49558613	0.49900479	0.50198737	0.50456618	0.50710023	0.50968107	0.5120625
Basilan	0.35025426	0.35703936	0.36332239	0.36954341	0.37621656	0.38300806	0.38952145	0.3954422	0.40060043	0.40556331	0.41037568	0.41474166	0.41870216	0.42251806	0.42622531	0.42958818

Location	2006	2007	2008	2009	2010	2011	2012	2013	2014	2015	2016	2017	2018	2019	2020	2021
Cambodia	0.38169817	0.38987623	0.39767952	0.40422093	0.41021112	0.41596441	0.42176975	0.42771506	0.43359775	0.43948736	0.44535817	0.45122174	0.45734149	0.46364343	0.46887605	0.47362149
Indonesia	0.56026592	0.5658119	0.57222527	0.5788669	0.58598242	0.59365298	0.6011857	0.6085099	0.61549989	0.62222247	0.62866662	0.63489574	0.64094875	0.64686546	0.65192665	0.65686834
Aceh	0.58672602	0.59150689	0.59674327	0.60181305	0.60705953	0.61268272	0.61854653	0.62454873	0.63050526	0.63648236	0.642395	0.6482508	0.65405877	0.65986518	0.66475876	0.66955164
Bali	0.55703291	0.56190502	0.56780069	0.57417724	0.58114054	0.58857033	0.59582696	0.60287149	0.60965942	0.61620184	0.62245407	0.62847275	0.63430388	0.64000539	0.64485235	0.64958588
Bangka-Belitung Islands	0.5512765	0.55631699	0.56225432	0.56835578	0.5747897	0.58178671	0.58868338	0.59537815	0.60187021	0.60823522	0.61460851	0.62102515	0.62738145	0.63359559	0.638989	0.64425964
Banten	0.54818718	0.55279114	0.55837624	0.56437318	0.57102114	0.57857249	0.5859379	0.59310912	0.59998839	0.60662567	0.61303665	0.61918089	0.62514768	0.63096052	0.63587866	0.64071299
Bengkulu	0.51629332	0.52173677	0.52817261	0.53479748	0.54181862	0.54940364	0.55681278	0.56409049	0.57103184	0.5777767	0.5843805	0.59086907	0.5972185	0.60341936	0.60863108	0.61367065
Gorontalo	0.4604025	0.46566364	0.4724715	0.48008009	0.48850748	0.49763464	0.50656442	0.51519052	0.52338898	0.53119496	0.53859668	0.5456919	0.55250868	0.55910806	0.56463912	0.56999021
Jakarta	0.71242858	0.71786297	0.72395734	0.73023891	0.73689221	0.74393246	0.75070975	0.75717729	0.76334451	0.76925722	0.77492035	0.78034984	0.78558506	0.79069715	0.79508951	0.79940944
Jambi	0.53111719	0.5374601	0.54511489	0.55317563	0.56192132	0.57129382	0.58031825	0.58885735	0.59684259	0.60427083	0.61117963	0.61763925	0.62378203	0.62971761	0.63470231	0.63956368
West Java	0.54898783	0.55426903	0.56051638	0.56700756	0.57393897	0.58144324	0.58878356	0.5959741	0.60283135	0.6094301	0.61576965	0.62195319	0.62798418	0.6338634	0.63884136	0.64367663
Central Java	0.51647028	0.52242419	0.52913658	0.53597076	0.54318954	0.5508159	0.55824886	0.56549478	0.57238966	0.57903147	0.58540672	0.5915727	0.59758761	0.60349025	0.60850881	0.61338882
East Java	0.54661489	0.55280696	0.55971581	0.5667625	0.57426857	0.58226911	0.59008313	0.59756583	0.60465166	0.61140792	0.61783986	0.62401929	0.62998262	0.63578677	0.64072987	0.64551738
West Kalimantan	0.5004363	0.50407497	0.50861467	0.5136176	0.5193251	0.52577988	0.53252363	0.53933342	0.54594146	0.55237456	0.55860712	0.56466654	0.57058178	0.57636235	0.58128935	0.58612167
South Kalimantan	0.5366955	0.5406522	0.54545082	0.55062024	0.55642207	0.56299804	0.56966811	0.57633518	0.5828793	0.58924978	0.59547153	0.6015648	0.60751384	0.61334189	0.61831046	0.62320608
Central Kalimantan	0.55309563	0.55639235	0.56077621	0.56569527	0.57137421	0.57788061	0.58453086	0.59117447	0.59765603	0.60395753	0.61019142	0.61640037	0.62255626	0.62864572	0.63386662	0.63899383
East Kalimantan	0.66508645	0.67062866	0.67711686	0.68365906	0.69066649	0.6984318	0.70607231	0.71336138	0.72020158	0.72672101	0.73287396	0.73872114	0.74436852	0.74989106	0.75463949	0.75929387
North Kalimantan	0.65717162	0.66355161	0.67050898	0.6771463	0.68391938	0.69114793	0.69826096	0.70517295	0.71188818	0.71842697	0.7247458	0.73089841	0.73687353	0.74271922	0.74790202	0.75298676
Riau Islands	0.6659116	0.6702922	0.6755603	0.68112742	0.68712948	0.69358947	0.69999368	0.70623153	0.7121994	0.71794241	0.72348517	0.72885742	0.73407381	0.73915486	0.7434696	0.74775431
Lampung	0.50517671	0.51099373	0.51802019	0.52564643	0.53407416	0.54289371	0.5513703	0.55939671	0.56688711	0.57397078	0.58064169	0.58699908	0.59308111	0.598979	0.60397338	0.60887136
Maluku	0.49251133	0.49083345	0.49229133	0.49595364	0.50170832	0.50914016	0.51704502	0.52509196	0.5329444	0.54067919	0.54813291	0.55534118	0.56233884	0.56918695	0.57482534	0.58032188
North Maluku	0.46358432	0.46605875	0.47046924	0.47616491	0.48333748	0.49176511	0.50026361	0.50862948	0.51668894	0.524421	0.5318067	0.53888887	0.5457041	0.55229142	0.55764156	0.56291087
West Nusa Tenggara	0.48383361	0.491206	0.49889746	0.50653697	0.51404228	0.52193726	0.52966433	0.53717467	0.54441446	0.5513934	0.55809279	0.56453903	0.57078447	0.5768659	0.5820152	0.58703462
East Nusa Tenggara	0.44776538	0.4525351	0.45847722	0.46489578	0.47209961	0.48008645	0.48801472	0.49578864	0.50327052	0.51053667	0.51761217	0.52461615	0.53149789	0.53824536	0.54391325	0.54945119
Papua	0.54958513	0.55306955	0.55766566	0.56313014	0.56958178	0.57705575	0.58465497	0.59219642	0.59954624	0.60674908	0.61376989	0.62068688	0.62747226	0.63412061	0.64001264	0.64580288
West Papua	0.54952541	0.55578751	0.56394076	0.57329506	0.58421778	0.59648716	0.60772107	0.61803843	0.62740989	0.6359972	0.64387731	0.65115933	0.65795906	0.6644003	0.66982939	0.67512564
Riau	0.62992535	0.63440792	0.64000295	0.6459983	0.65260316	0.65996766	0.66750217	0.67490295	0.68198684	0.68878372	0.69518683	0.70131759	0.70725774	0.7130141	0.71796346	0.72278452
West Sulawesi	0.46529875	0.47021593	0.47685496	0.48430645	0.49271753	0.50194275	0.51109162	0.51991839	0.5283045	0.53627554	0.54378197	0.55095746	0.55786177	0.56453058	0.57018471	0.57565685
South Sulawesi	0.51836497	0.52395152	0.53070743	0.5380618	0.5461011	0.55461525	0.56283476	0.57071405	0.57822434	0.58540084	0.59222524	0.59873435	0.60497768	0.61102327	0.61614532	0.62113541
Central Sulawesi	0.51201061	0.51720467	0.52358948	0.53057415	0.53837511	0.54702868	0.55550698	0.56367404	0.57144215	0.5788657	0.58590289	0.59265171	0.59916342	0.60551197	0.61094788	0.61625384
Southeast Sulawesi	0.50458344	0.51183548	0.52030102	0.52903955	0.53805543	0.54740529	0.55647391	0.56510272	0.57318621	0.58073303	0.5875894	0.59394341	0.59997204	0.60579632	0.61061776	0.61532587
North Sulawesi	0.5571767	0.56115905	0.56642855	0.57237058	0.57907107	0.58648729	0.59387226	0.60110657	0.60806176	0.61478816	0.62130855	0.62767902	0.63391322	0.64002776	0.64529661	0.65046831
West Sumatra	0.56525197	0.5710871	0.57780879	0.5847555	0.59220161	0.60018689	0.60806795	0.6157333	0.62300956	0.63003218	0.63670136	0.64314623	0.64936973	0.65548106	0.66065894	0.66576366
South Sumatra	0.549304	0.55464109	0.56085431	0.56721212	0.57404763	0.58148689	0.58895007	0.596305	0.6033241	0.61015675	0.61668165	0.62300434	0.629152	0.63521332	0.64041033	0.64553676
North Sumatra	0.56783008	0.57316613	0.5794509	0.58607463	0.59332584	0.60125332	0.60901473	0.61657934	0.62379014	0.63068585	0.63739153	0.64387346	0.65020385	0.65640729	0.66174552	0.66702565
Yogyakarta	0.57888155	0.58456988	0.59111386	0.59792884	0.60523456	0.61307587	0.62074417	0.62816785	0.63521167	0.64195219	0.64831678	0.65444207	0.66036427	0.66613281	0.67086108	0.67542992
Laos	0.37812228	0.38772365	0.3970113	0.40613902	0.41486557	0.42335452	0.43114936	0.43869104	0.44586942	0.4527496	0.459584	0.46616889	0.4726181	0.47881137	0.48419296	0.48913609
Malaysia	0.66595629	0.67084373	0.67639991	0.68207065	0.68825862	0.6940834	0.69966473	0.70493072	0.70995376	0.71465987	0.71922342	0.72423541	0.72938808	0.73458775	0.73874399	0.74252383
Maldives	0.54795041	0.55752925	0.56683963	0.57444206	0.58198099	0.58968446	0.59694852	0.60423852	0.61157807	0.61845439	0.62487595	0.63108329	0.63720566	0.64308279	0.64760859	0.65088663
Mauritius	0.63954518	0.64435866	0.64963085	0.6550003	0.6604698	0.66649763	0.67312765	0.68011402	0.6866165	0.69223973	0.69702056	0.70161814	0.70626665	0.71093504	0.7146298	0.71826045
Myanmar	0.42470507	0.43453693	0.44359553	0.45195488	0.46018043	0.46785087	0.47533642	0.48282186	0.49025515	0.49737416	0.50411598	0.51082343	0.51755747	0.52410897	0.5294347	0.53390084
Philippines	0.55797627	0.56082202	0.56397472	0.56735582	0.57176589	0.57724028	0.58376763	0.59083755	0.59838452	0.60621562	0.61422496	0.62231503	0.63046428	0.63854738	0.64486723	0.65121933
Abra	0.53856713	0.54110627	0.54455748	0.5487971	0.55428983	0.56096209	0.56878773	0.57723633	0.58624882	0.59554948	0.60492779	0.61425622	0.62341822	0.63236923	0.63953061	0.64654691
Agusan Del Norte	0.51462695	0.51615474	0.51849637	0.52144351	0.52559722	0.53097806	0.53761675	0.54493819	0.55285983	0.56118542	0.56979452	0.57849767	0.58715901	0.59578107	0.60257288	0.60936062
Agusan Del Sur	0.4405241	0.44308084	0.44631035	0.45008245	0.45502525	0.4610141	0.46809602	0.47573261	0.48389936	0.49238263	0.50112963	0.50998689	0.51893209	0.52794931	0.53518578	0.54241367
Aklan	0.54263086	0.54623701	0.55059763	0.55522387	0.5607583	0.56692079	0.57386867	0.5812155	0.58886003	0.59662837	0.60450352	0.61235292	0.62016064	0.6279543	0.63393133	0.64007481
Albay	0.5359961	0.53986962	0.5444071	0.54930352	0.55513605	0.56166922	0.56904064	0.57679863	0.58491181	0.59324439	0.60166376	0.61006834	0.61845767	0.62675705	0.63327635	0.6398634
Antique	0.4636722	0.46651766	0.47020268	0.47458869	0.48023859	0.48688442	0.49467046	0.50309332	0.51202966	0.52134594	0.53080936	0.54032422	0.54978241	0.55918367	0.56678776	0.57433252
Apayao	0.5010587	0.50583892	0.51126283	0.51696672	0.52362411	0.53091238	0.53899792	0.54752406	0.55619904	0.56486221	0.57345629	0.58194008	0.59027874	0.59844973	0.60475473	0.61105972
Aurora	0.51477039	0.51816251	0.5220613	0.52633573	0.53163879	0.53772448	0.54485065	0.55266036	0.56078751	0.56896931	0.57717183	0.58537656	0.5935932	0.60179478	0.60814536	0.61450607
Basilan	0.43327269	0.43752808	0.44231082	0.44751166	0.4537452	0.46091357	0.4689508	0.47743764	0.48631662	0.49541035	0.50464591	0.51393515	0.52320306	0.53235515	0.53964471	0.54682572

Table S5. Socio-demographic Index values for all estimated GBD 2021 locations, 1990-2021

Location	1990	1991	1992	1993	1994	1995	1996	1997	1998	1999	2000	2001	2002	2003	2004	2005	2006	2007	2008	2009	2010	2011	2012	2013	2014	2015	2016	2017	2018	2019	2020	2021
Bataan	0.51730903	0.52073572	0.52392824	0.5272315	0.53117075	0.53538361	0.53949478	0.54317543	0.54614722	0.54905244	0.5519477	0.55448765	0.55672351	0.55889357	0.56108759	0.56305373	0.56533508	0.56820748	0.57158244	0.57517505	0.57969749	0.58498552	0.59112679	0.59780381	0.60497966	0.61250585	0.62027546	0.62816166	0.63619725	0.64426059	0.65061044	0.65690151
Batanes	0.52804963	0.53193989	0.53558574	0.5393391	0.54393731	0.54910787	0.55456262	0.55959485	0.5634099	0.56694633	0.57059109	0.5740181	0.57728226	0.58051613	0.58398298	0.58749535	0.59165252	0.59664492	0.60211166	0.60739106	0.6131907	0.61895739	0.6251476	0.63171027	0.63845433	0.64519718	0.65190275	0.65863143	0.66539208	0.67219288	0.67707236	0.68230486
Batangas	0.55242986	0.55571767	0.55872984	0.56179959	0.56536359	0.56913432	0.57282015	0.57606201	0.57853729	0.58078085	0.58297807	0.58484085	0.58645468	0.58810538	0.58989451	0.59153416	0.59355417	0.59622005	0.59943515	0.60302307	0.60760594	0.61299383	0.61927929	0.62608892	0.63337662	0.64097125	0.64874973	0.65658235	0.66444634	0.67220937	0.67827952	0.68441127
Benguet	0.58446435	0.58722893	0.58973213	0.59234214	0.59546542	0.59880434	0.60210487	0.60493937	0.6069642	0.60889627	0.61088718	0.61265109	0.6142778	0.6160018	0.61799382	0.61996378	0.62242933	0.62566677	0.62953794	0.63371516	0.63876873	0.64445207	0.65085035	0.657592	0.66465272	0.67201387	0.67962665	0.68728025	0.69483144	0.70223486	0.70783292	0.71350365
Biliran	0.48895728	0.49398281	0.49863276	0.50330626	0.50846065	0.51375464	0.51891805	0.52355834	0.52737613	0.53093711	0.53436303	0.53739152	0.54004356	0.54268936	0.54542497	0.54798481	0.5508268	0.55430762	0.5584497	0.56277987	0.5677837	0.57325657	0.57939771	0.58595626	0.5929906	0.60035125	0.60789209	0.61555329	0.62337909	0.63130052	0.63764281	0.64405335
Bohol	0.44390387	0.44830252	0.45234869	0.45643874	0.4611379	0.46614676	0.4711269	0.47556494	0.47910772	0.48241894	0.48559586	0.4882872	0.49060598	0.49281291	0.49518294	0.49745455	0.50017767	0.50369931	0.50794585	0.51261214	0.51825989	0.52473747	0.532207	0.5402093	0.54850296	0.55694141	0.56546884	0.57392992	0.58229391	0.59059041	0.5971473	0.60378059
Bukidnon	0.39938042	0.40402311	0.40827593	0.41258865	0.41742549	0.42236631	0.42703538	0.43108439	0.4343232	0.43729774	0.44007909	0.44233074	0.44411197	0.44578894	0.44744354	0.44882422	0.45049022	0.45275917	0.45564395	0.45905516	0.4636238	0.46930827	0.47618836	0.48368451	0.49175506	0.50024768	0.50900102	0.51791313	0.5269522	0.53596388	0.5432682	0.55051685
Bulacan	0.56284007	0.56642846	0.56976605	0.57318035	0.57709438	0.581283	0.5855106	0.58943645	0.59269319	0.59575617	0.59879603	0.60152106	0.6040156	0.60653658	0.60925856	0.61188916	0.61489421	0.61850779	0.62258289	0.62685917	0.63197556	0.63774941	0.64430353	0.65124647	0.65846391	0.66586807	0.67339665	0.68093464	0.68845606	0.69588286	0.70165408	0.70752202
Cagayan	0.47652386	0.48089784	0.4849639	0.48911719	0.49392694	0.49897151	0.50394762	0.50841483	0.51200176	0.51532124	0.51849835	0.52120883	0.52350087	0.52569649	0.52794402	0.53001031	0.53246614	0.53570295	0.53962958	0.54397672	0.54938061	0.5556774	0.56297305	0.57088411	0.57924451	0.58780075	0.59650305	0.6051487	0.61370978	0.6220833	0.62860478	0.63506809
Camarines Norte	0.45438976	0.45784047	0.46106358	0.46446262	0.46842878	0.47258349	0.47653263	0.47984856	0.48221663	0.48431289	0.48628166	0.48777093	0.48886074	0.48987763	0.49124432	0.49261278	0.49456887	0.49740224	0.50092203	0.50481171	0.50969936	0.51547939	0.52234025	0.52981666	0.53781991	0.54612331	0.55462317	0.56323271	0.57186191	0.58049559	0.58738351	0.59428649
Camarines Sur	0.48516488	0.48936142	0.49320097	0.49705719	0.50150615	0.50614349	0.51073367	0.51485311	0.51806387	0.52104005	0.52388328	0.52629929	0.52836034	0.53035073	0.5323433	0.53412759	0.53627212	0.53913326	0.54268436	0.54657535	0.55142964	0.5570564	0.56363917	0.57079921	0.57846816	0.58641965	0.59457897	0.60282115	0.61107904	0.61929616	0.62586626	0.63255464
Camiguin	0.49530582	0.49820694	0.50095142	0.503858	0.50718119	0.51070271	0.51417361	0.51712964	0.51911276	0.5208524	0.52257327	0.5241482	0.52556588	0.52698641	0.52906275	0.53142481	0.5345077	0.53852345	0.54321424	0.54787944	0.55338061	0.55942911	0.56627343	0.57367671	0.581441	0.58933593	0.59733393	0.60531239	0.61318479	0.62090381	0.62657239	0.6324379
Capiz	0.42655316	0.43079642	0.43456981	0.43833829	0.44258124	0.44686682	0.45086895	0.45424239	0.45673728	0.4589745	0.46102131	0.46249156	0.46348009	0.4644371	0.46564337	0.46669233	0.46822757	0.47060478	0.47382466	0.47749504	0.4822294	0.48802417	0.49494841	0.50238271	0.51040366	0.51882531	0.52752021	0.53634523	0.54517963	0.55396279	0.56102037	0.56808796
Catanduanes	0.46255151	0.46703283	0.47110294	0.47520572	0.47981855	0.48452105	0.48900294	0.49280534	0.49576623	0.49844094	0.50091321	0.50289905	0.50440134	0.50578591	0.50712867	0.50809942	0.50927829	0.51096617	0.51320001	0.51608385	0.52020463	0.525721	0.53265367	0.54034958	0.54878823	0.55761777	0.56667033	0.57585695	0.58513414	0.59443609	0.60201223	0.60947869
Cavite	0.61021183	0.6124665	0.61443187	0.61645647	0.61904967	0.62193706	0.6248783	0.62747624	0.62937326	0.63114898	0.63294777	0.63448097	0.63585756	0.63735987	0.63907192	0.64077745	0.6429148	0.64573383	0.64908738	0.65266111	0.65713881	0.66226784	0.66809263	0.67431285	0.68087015	0.68761723	0.69457625	0.70160767	0.70870624	0.71579043	0.72123057	0.72678437
Cebu	0.5327355	0.5351841	0.53740934	0.53969607	0.54249573	0.54554257	0.54856922	0.55119372	0.55306328	0.55474353	0.55642898	0.55783064	0.55904088	0.56034274	0.56183098	0.56327943	0.56518138	0.56786317	0.57124477	0.57495432	0.57963133	0.58504329	0.59131123	0.59806098	0.60527238	0.61281872	0.62062354	0.62851333	0.63637842	0.64408431	0.65001485	0.65607636
Cotabato (North Cotabato)	0.42436313	0.42818722	0.43160143	0.43502575	0.43895756	0.44301191	0.4468479	0.45002744	0.45227877	0.45430044	0.45617086	0.4575233	0.45841112	0.45918173	0.46004843	0.46065059	0.46165937	0.46333898	0.46559733	0.46834209	0.47224272	0.47727292	0.48351726	0.49046199	0.49798351	0.50597266	0.51424832	0.52273164	0.53134207	0.53999565	0.54690644	0.55377082
Davao de Oro	0.39418893	0.39847572	0.40227264	0.40603481	0.41026461	0.41461367	0.41869716	0.42218896	0.42477907	0.42707944	0.42921786	0.43083905	0.43201518	0.4330585	0.43417484	0.43514979	0.43663131	0.43889782	0.44175208	0.44512159	0.4496941	0.45536184	0.46218962	0.46966269	0.47761347	0.48584193	0.49431108	0.50298274	0.51174337	0.52052061	0.52746244	0.53441776
Davao Del Norte	0.47725283	0.48225795	0.48690984	0.49153825	0.4966396	0.50195836	0.50711014	0.51174264	0.51544404	0.51883727	0.52212084	0.52505647	0.52769764	0.5303122	0.53308924	0.53575812	0.53881707	0.54248804	0.54659841	0.55097192	0.55618365	0.56207274	0.56883134	0.57603195	0.58364044	0.5915541	0.59976688	0.60810743	0.61643135	0.62474035	0.63133764	0.63810495
Davao Del Sur	0.50911851	0.51301609	0.51657149	0.52016433	0.52417087	0.52832122	0.53231932	0.53582232	0.53855714	0.54111292	0.54363175	0.5457578	0.54757006	0.54942521	0.55143046	0.55334759	0.55572699	0.55888083	0.56266338	0.56688136	0.57218286	0.57839167	0.58562196	0.59348122	0.60187632	0.61064279	0.61964858	0.62867747	0.63761483	0.64631332	0.65316974	0.66000147
Davao Occidental	0.38892382	0.39453863	0.39978894	0.40509033	0.41083885	0.41669294	0.42225812	0.42732412	0.4319013	0.43642041	0.44086023	0.44490754	0.44856808	0.45211209	0.45553339	0.4586042	0.46190319	0.46566822	0.4699581	0.47488221	0.48087414	0.48819166	0.49678071	0.50589737	0.51561995	0.52561758	0.53566598	0.54566161	0.55547244	0.5651286	0.57338474	0.5814625
Davao Oriental	0.38524584	0.38959462	0.39358236	0.39764345	0.40250844	0.40775242	0.41302433	0.41785172	0.42187172	0.42570912	0.42947631	0.432804	0.43568961	0.43849756	0.4412418	0.44359074	0.44616016	0.44930632	0.45292752	0.45694815	0.46194415	0.46785605	0.47478946	0.48230605	0.49033515	0.49867918	0.50717729	0.51575039	0.52437516	0.53298609	0.53985027	0.54667344
Dinagat Islands	0.45740369	0.46302562	0.46819699	0.47334562	0.47895309	0.48467229	0.49010923	0.49499197	0.49919666	0.50324373	0.50715463	0.51070535	0.51380372	0.51672098	0.51959612	0.52218647	0.52500842	0.52826576	0.53176201	0.53548164	0.54012138	0.54571947	0.5523491	0.55945463	0.56702533	0.57490673	0.58298046	0.59118251	0.59934323	0.60750915	0.61416855	0.62078136
Eastern Samar	0.35878738	0.36287804	0.36639932	0.36992462	0.37396516	0.37800492	0.38158973	0.38433124	0.38619929	0.38775801	0.38903916	0.38967202	0.38964507	0.38937881	0.38922443	0.3887952	0.38886177	0.38983075	0.39169623	0.39439736	0.39851924	0.40441011	0.41208072	0.42047546	0.42967434	0.43935852	0.44929564	0.45942445	0.46963707	0.47979713	0.48796383	0.49567523
Guimaras	0.44841468	0.45316568	0.45753379	0.46197678	0.46696004	0.47214378	0.47713384	0.48152395	0.48495762	0.48811492	0.49114348	0.49370461	0.49585227	0.49798168	0.50056942	0.50323377	0.50645176	0.51053953	0.51534873	0.52028262	0.52601927	0.53240545	0.53963773	0.54731477	0.55528574	0.56341961	0.57161359	0.57977664	0.58797511	0.59620624	0.60268828	0.60924067
Ifugao	0.44254822	0.44799527	0.45305081	0.45813379	0.46360023	0.46907812	0.4742085	0.47866469	0.48223806	0.48557833	0.48872139	0.4913429	0.49345894	0.49540788	0.49742238	0.49921543	0.50131215	0.50400927	0.50719384	0.5107684	0.51541552	0.52116326	0.52797046	0.53524472	0.54299618	0.55103808	0.55922049	0.56752477	0.57579771	0.58406663	0.59062678	0.59712822
Ilocos Norte	0.55620797	0.55941142	0.5624026	0.56546019	0.56892279	0.57254755	0.57609089	0.57920008	0.58153885	0.58374293	0.58592588	0.58778312	0.5893994	0.59100477	0.59275388	0.59441944	0.59655341	0.59943024	0.602955	0.60683779	0.61166485	0.61727603	0.62372521	0.63059125	0.6377458	0.64510911	0.65258588	0.66009901	0.66761516	0.67507917	0.68093679	0.68692159
Ilocos Sur	0.52445264	0.52877526	0.53283102	0.53697683	0.54152727	0.54616298	0.55055354	0.55436649	0.5572827	0.55995661	0.56252015	0.56464872	0.56645519	0.56822488	0.57022958	0.57222931	0.57470133	0.57793142	0.58183559	0.58606239	0.59125395	0.59725136	0.6041791	0.61156802	0.61934029	0.62730858	0.63543429	0.64352496	0.65156554	0.65954628	0.66589429	0.67227776
Iloilo	0.5274337	0.53164338	0.53553417	0.53944869	0.54368283	0.54806212	0.5522409	0.5558445	0.55851279	0.56095575	0.56335959	0.5653781	0.5671425	0.56892978	0.57108015	0.57327554	0.57599799	0.57963491	0.58399969	0.58863906	0.59420191	0.60035015	0.60725643	0.61459278	0.62225255	0.63002535	0.63791008	0.64576382	0.65349013	0.66111503	0.66693733	0.67291782
Isabela	0.48699406	0.49145654	0.49562262	0.49979648	0.50443154	0.50927208	0.51395891	0.51808191	0.52135996	0.52433769	0.52716772	0.52954407	0.53154412	0.53351323	0.53560178	0.53752363	0.53984775	0.54286959	0.5464968	0.55053601	0.55560088	0.56153599	0.5684623	0.57594551	0.58384658	0.59196152	0.60022421	0.60847328	0.61669159	0.62477231	0.63104261	0.63731965
Kalinga	0.39658691	0.40225292	0.40761113	0.41302918	0.41895949	0.425071	0.43105406	0.43650476	0.44105031	0.44531703	0.44945356	0.45307383	0.45628207	0.45949121	0.4630008	0.46646004	0.470394	0.47513579	0.48055517	0.4863254	0.49299647	0.50017572	0.50808955	0.5163918	0.52481059	0.53317686	0.54148945	0.54972099	0.55786137	0.56590304	0.57200029	0.57819828
La Union	0.53314864	0.53607132	0.53867507	0.5413428	0.54451687	0.54789454	0.55114507	0.55390737	0.55590549	0.55770902	0.55944715	0.56079972	0.56181975	0.56281121	0.56390595	0.56486544	0.56624375	0.56840593	0.57124369	0.57457037	0.57905343	0.58454445	0.59117864	0.59849276	0.60641322	0.61462279	0.62302889	0.63144707	0.63982786	0.64806169	0.65451761	0.66096366
Laguna	0.58311235	0.5857236	0.58796255	0.5902118	0.59291968	0.59582943	0.59865823	0.6010914	0.60282229	0.60438111	0.60593034	0.60716869	0.60818691	0.60927961	0.610613	0.61188147	0.61362688	0.61611386	0.61922123	0.62267607	0.62702988	0.63215151	0.63814732	0.64460403	0.6515515	0.65882266	0.66633926	0.67391037	0.68153834	0.68909832	0.69499712	0.70092466
Lanao Del Norte	0.46393371	0.46730205	0.47024729	0.47322799	0.47669525	0.48023801	0.48352204	0.48614458	0.48790355	0.48943335	0.49081105	0.49166191	0.49199088	0.49216804	0.49235395	0.49223712	0.49246926	0.49335385	0.49493157	0.49709516	0.50042609	0.50507825	0.5110446	0.5177011	0.52514132	0.5331415	0.54157525	0.55027829	0.55915655	0.56804898	0.57530936	0.58250552
Lanao Del Sur	0.39307171	0.39783336	0.4021185	0.40640945	0.41086585	0.41514232	0.41888532	0.42184144	0.42389968	0.42561216	0.4270541	0.42796934	0.42834406	0.42863386	0.42914705	0.42953723	0.43047656	0.43224423	0.43479719	0.4380233	0.44249021	0.44817248	0.45507745	0.46267006	0.4709311	0.47958677	0.48849782	0.49755357	0.50672528	0.51590961	0.52329783	0.53070277
Leyte	0.45275446	0.45725566	0.46133877	0.46545871	0.47003179	0.47474528	0.47934116	0.48339813	0.48653287	0.48941963	0.49216187	0.49451497	0.49651534	0.49847772	0.50066122	0.50277523	0.5053147	0.50858904	0.51252135	0.51695936	0.52254593	0.52905307	0.53666957	0.54494778	0.55365192	0.56248618	0.57140471	0.58023973	0.58889864	0.5974381	0.60416111	0.61094256
Maguindanao	0.33805456	0.34389615	0.34930155	0.35472195	0.3605382	0.3663649	0.37191177	0.37685492	0.38088845	0.38457893	0.3880739	0.39102055	0.39347961	0.39579569	0.39824316	0.40062091	0.40344862	0.40702858	0.41118786	0.41568944	0.42128118	0.42786739	0.43549019	0.44362905	0.45217335	0.46098824	0.46993436	0.47895129	0.48799597	0.49699725	0.5041702	0.51129551
Marinduque	0.41590204	0.41991423	0.42354926	0.42723811	0.43134064	0.43546967	0.43925705	0.44229308	0.44439647	0.44622425	0.44780766	0.44883518	0.44938912	0.44984263	0.45049874	0.45092478	0.45178308	0.45340269	0.45583086	0.45897958	0.46338761	0.46916505	0.47638621	0.4842276	0.4927244	0.50158883	0.51051274	0.51945399	0.5284135	0.53740949	0.54465332	0.55186124
Masbate	0.35722893	0.3601303	0.36258151	0.3650843	0.36797611	0.37085019	0.37327649	0.37485854	0.37548957	0.37583933	0.37592122	0.37541368	0.37428736	0.37296442	0.37150644	0.36959719	0.36788461	0.36683888	0.36655854	0.36726094	0.36942342	0.37347963	0.37934986	0.38599463	0.39366559	0.40206283	0.41091618	0.42009045	0.42946856	0.43896097	0.44664412	0.45410809
Misamis Occidental	0.47913777	0.48213092	0.48475561	0.48737597	0.49022803	0.49300532	0.49542158	0.49717032	0.49795515	0.49845125	0.49877893	0.49865014	0.49814921	0.49755264	0.49727484	0.49699739	0.49728091	0.4983823	0.50021534	0.50260102	0.50617145	0.51100583	0.51714556	0.5239952	0.5314557	0.53933911	0.54751223	0.55582144	0.56412563	0.57247424	0.57908862	0.5857211
Misamis Oriental	0.54614833	0.54896294	0.55146648	0.55399853	0.55705062	0.5603096	0.56349272	0.56616435	0.56796238	0.56956938	0.57111426	0.57223796	0.5730367	0.57382276	0.57471239	0.5754302	0.57656557	0.57831873	0.58056242	0.5831553	0.58674428	0.59127318	0.59683424	0.60302059	0.60979533	0.61704272	0.62460115	0.63232768	0.64012107	0.64791731	0.65403382	0.66020852
Mountain Province	0.38414298	0.389891	0.39498319	0.39982522	0.40488079	0.4097621	0.41407016	0.41748634	0.41995837	0.42196752	0.42357614	0.42441576	0.42454524	0.42438025	0.42406488	0.42328321	0.4227177	0.42292593	0.42413562	0.42630628	0.42974588	0.4345494	0.4407264	0.44768079	0.4557423	0.46462027	0.47396115	0.48380356	0.49393712	0.50417424	0.51254507	0.52052229
National Capital Region	0.63160408	0.63385386	0.63586242	0.63794006	0.64054516	0.6433943	0.64624143	0.64879458	0.65070901	0.65261863	0.65460432	0.65633226	0.6579052	0.65951263	0.66133099	0.66314196	0.66546212	0.66855547	0.67221608	0.6761265	0.68094521	0.68641653	0.69257274	0.69909269	0.70589905	0.71288225	0.72002705	0.72718223	0.73426032	0.74120819	0.74642606	0.75174222
Negros Occidental	0.49213542	0.49510811	0.49776751	0.50045048	0.50352918	0.50670884	0.50975214	0.51220567	0.51372528	0.51499499	0.51616173	0.51691183	0.51731886	0.51766022	0.51814239	0.51848851	0.51929894	0.52083332	0.52300414	0.52562109	0.52929347	0.53391943	0.53958533	0.54585822	0.55269499	0.55997196	0.56754244	0.57525008	0.58299961	0.59077908	0.59691778	0.60317817
Negros Oriental	0.43817326	0.44179132	0.4451439	0.44855606	0.45249659	0.45666524	0.46074033	0.46435162	0.46715082	0.46971487	0.47216998	0.47422025	0.47593512	0.47761205	0.47929826	0.48076246	0.48254915	0.48498773	0.48805853	0.49160108	0.49618221	0.50156702	0.50789908	0.51488165	0.52241419	0.53026019	0.53836243	0.54654158	0.55479699	0.56301219	0.5695307	0.57608402
Northern Samar	0.37764913	0.38244778	0.38681468	0.39120347	0.39597642	0.40071292	0.40510178	0.40875011	0.41141009	0.41374623	0.41583515	0.41730969	0.41822878	0.41897252	0.41992488	0.42082439	0.42226388	0.42458261	0.42771499	0.43129026	0.43588865	0.44151493	0.44827083	0.45562729	0.46356622	0.4719156	0.48057928	0.4894836	0.49855788	0.50769037	0.51501261	0.52220911
Nueva Ecija	0.53138136	0.53378625	0.53589821	0.53811488	0.54086317	0.54385148	0.54671669	0.54910359	0.55074059	0.55222002	0.55366637	0.55480685	0.55570562	0.55666473	0.55780036	0.55887801	0.56040271	0.5626609	0.56559324	0.56893863	0.5732195	0.57829571	0.5842918	0.59086093	0.59799809	0.6055098	0.61332977	0.62127825	0.62931526	0.63734588	0.6437457	0.6501666
Nueva Vizcaya	0.48217618	0.48687121	0.49090801	0.49480748	0.4990883	0.50326989	0.5069903	0.50997028	0.5120849	0.51390103	0.51549017	0.51651509	0.51713657	0.51763804	0.5182423	0.51866244	0.51945415	0.52090488	0.52309082	0.52603599	0.53017195	0.53563317	0.54236745	0.54962416	0.55755534	0.56595879	0.57467543	0.58359332	0.59262937	0.60170588	0.60904706	0.61619295
Occidental Mindoro	0.33903764	0.34496705	0.35004917	0.35491804	0.35998364	0.36463575	0.36837777	0.3709589	0.37274986	0.37417682	0.37516621	0.37535431	0.37464914	0.37351343	0.37183662	0.36944013	0.36714958	0.36547909	0.36472746	0.36531598	0.36746442	0.37175191	0.37796597	0.38495383	0.39349826	0.40317466	0.41360415	0.42464139	0.43608394	0.44773088	0.45755516	0.46683504
Oriental Mindoro	0.43805419	0.44343691	0.44835161	0.45324727	0.45848592	0.46369684	0.46858479	0.47282111	0.4761544	0.47923812	0.48214392	0.48463363	0.48675639	0.48881242	0.49099868	0.49304322	0.49556269	0.49889006	0.50300885	0.5076352	0.51339077	0.5200893	0.5277632	0.53591926	0.54453945	0.55339106	0.56237847	0.57133617	0.58022105	0.58907426	0.59616333	0.60323574
Palawan	0.39112553	0.39534837	0.39904474	0.40265734	0.40685734	0.41116274	0.41520016	0.41852935	0.42092942	0.42308291	0.42505142	0.42646715	0.42737038	0.42812927	0.42886414	0.42925955	0.43002779	0.43149819	0.43370491	0.43654124	0.44065406	0.44615281	0.45303761	0.46060718	0.4688514	0.47756987	0.48667678	0.4960264	0.5054682	0.5149248	0.52253468	0.53000513

Table S5. Socio-demographic Index values for all estimated GBD 2021 locations, 1990-2021

Location	1990	1991	1992	1993	1994	1995	1996	1997	1998	1999	2000	2001	2002	2003	2004	2005	2006	2007	2008	2009	2010	2011	2012	2013	2014	2015	2016	2017	2018	2019	2020	2021
Pampanga	0.55774643	0.56147988	0.56484903	0.56814459	0.57190275	0.5759152	0.57986541	0.58340056	0.58624531	0.58892518	0.59161387	0.59400007	0.59614123	0.59830123	0.60050269	0.60247068	0.60475493	0.60760769	0.61094089	0.61458145	0.61917099	0.62455238	0.63084502	0.63765863	0.64493023	0.65251482	0.66029655	0.66811679	0.6759948	0.68377512	0.68986456	0.69599928
Pangasinan	0.53558358	0.53872646	0.54160505	0.54459925	0.54812259	0.55178706	0.55531079	0.55828438	0.56042102	0.56235704	0.5642316	0.5657424	0.56694724	0.56815525	0.56952375	0.57082225	0.57258064	0.57508422	0.57826383	0.58187691	0.58654922	0.5921009	0.59862753	0.60576604	0.61336556	0.62119627	0.6292134	0.63720241	0.64519851	0.65310651	0.65929418	0.66557535
Quezon	0.49475598	0.49790492	0.50070895	0.50354903	0.50677801	0.51003994	0.5130687	0.51554354	0.51720375	0.51865252	0.52004659	0.52118402	0.52212822	0.52315832	0.52445697	0.52578499	0.52766585	0.53041695	0.53395268	0.53808912	0.5433584	0.5494748	0.55657661	0.564322	0.57258105	0.58107409	0.58972957	0.59837124	0.60695527	0.61537239	0.62199063	0.62856238
Quirino	0.4177167	0.42303817	0.42800728	0.43308111	0.43868367	0.44439393	0.44986361	0.45469782	0.45860044	0.46221869	0.46556972	0.46836819	0.47061397	0.47264702	0.47463839	0.47629671	0.47826004	0.48083002	0.48397154	0.48742756	0.49180455	0.49717358	0.50362741	0.51057238	0.51802951	0.5257954	0.53383301	0.54194224	0.55015005	0.55837508	0.56494078	0.57149884
Rizal	0.5565378	0.55997965	0.56369138	0.56788435	0.57302792	0.57871459	0.58460329	0.5902104	0.59504784	0.59961319	0.60397361	0.60779266	0.61114182	0.61433544	0.61742208	0.62012651	0.62297682	0.62624142	0.62979738	0.6334617	0.63794313	0.64300619	0.64879787	0.65501257	0.6616108	0.66848546	0.6756337	0.6829413	0.69032797	0.69772185	0.70349531	0.7094079
Romblon	0.37349168	0.37796991	0.38199002	0.38605967	0.39077332	0.39568969	0.40040255	0.4043873	0.40742765	0.41025044	0.41286539	0.41488296	0.41639351	0.41781538	0.41933921	0.42054717	0.42210838	0.4244441	0.42765163	0.43161897	0.43687056	0.44355362	0.45172336	0.46052892	0.46986101	0.47937041	0.48892931	0.49842319	0.5078754	0.5173138	0.52489829	0.53239065
Samar (Western Samar)	0.40212297	0.40606027	0.40956094	0.41303879	0.41684793	0.42065389	0.42412812	0.42691015	0.42875093	0.4302514	0.43153768	0.43227449	0.43252622	0.43266752	0.43314898	0.4336472	0.43471247	0.43662302	0.43927735	0.44229819	0.44633676	0.45145341	0.45773953	0.46464227	0.47205886	0.47978456	0.48775288	0.49588352	0.50416108	0.51245123	0.51901364	0.5255082
Sarangani	0.4132386	0.41756627	0.42167619	0.42592459	0.43072749	0.43584704	0.44100487	0.44584224	0.44986161	0.45370555	0.45758851	0.46112008	0.46443457	0.4678022	0.47139606	0.47491678	0.47884591	0.48344688	0.48840828	0.49361508	0.49983808	0.50663018	0.51420378	0.52230177	0.53047681	0.53864512	0.54684426	0.55502382	0.56316235	0.57121446	0.5774474	0.58386943
Siquijor	0.43657916	0.4412208	0.44563819	0.45017277	0.45537821	0.46094142	0.46665048	0.47197804	0.47636038	0.480492	0.48456544	0.48836387	0.49190731	0.49549428	0.498892	0.50182594	0.50494068	0.5085996	0.51265358	0.51698715	0.52235974	0.52834551	0.53514931	0.54251028	0.55013087	0.55782956	0.56563678	0.57346837	0.58125928	0.5889612	0.59472782	0.6007397
Sorsogon	0.45118656	0.45434962	0.45726848	0.46032926	0.46404047	0.46806262	0.47202674	0.47546852	0.47805993	0.4804133	0.48269207	0.48461039	0.4862521	0.487954	0.48995273	0.49193837	0.49446113	0.49789712	0.50217657	0.50683034	0.51250641	0.51902351	0.52648866	0.53441031	0.54272952	0.55128515	0.56002191	0.56882099	0.57764814	0.58640728	0.59330996	0.60025457
South Cotabato	0.49330467	0.49706363	0.50042696	0.50381786	0.50770815	0.51172594	0.51558197	0.51890343	0.52139642	0.52366208	0.5258649	0.52764294	0.52902999	0.5304012	0.53192992	0.53328942	0.5350743	0.53759213	0.54068989	0.54424253	0.5489041	0.5545683	0.56135752	0.56877584	0.57673543	0.58503259	0.5935306	0.60208225	0.61056141	0.61893074	0.62549704	0.63208137
Southern Leyte	0.44465998	0.44898268	0.45295687	0.45698272	0.46147338	0.46602647	0.47033965	0.47400583	0.47678152	0.47932229	0.48171349	0.48372561	0.48541075	0.48713178	0.4891427	0.49104828	0.49350235	0.49676617	0.50064954	0.505049	0.5105676	0.51719274	0.52494954	0.53333255	0.54211491	0.55103816	0.56003126	0.56894778	0.57775058	0.58646596	0.59333487	0.60021125
Sultan Kudarat	0.38977251	0.39478584	0.39921778	0.40355312	0.40822894	0.4127868	0.41688386	0.42011876	0.42240267	0.42435751	0.42603049	0.42710189	0.42755292	0.42774562	0.42787117	0.42760143	0.42767816	0.42841617	0.42984594	0.43192742	0.43524464	0.43998786	0.44614033	0.45297282	0.46062176	0.46884483	0.47754768	0.48662115	0.49596074	0.50539423	0.513117	0.52067608
Sulu	0.29102567	0.29649175	0.30189253	0.30759409	0.3137567	0.32001469	0.32606595	0.33159151	0.33616669	0.3404493	0.34463609	0.34847542	0.35204626	0.3556816	0.35944538	0.36301171	0.366933	0.37151761	0.37654503	0.38202734	0.38877923	0.3964133	0.40501488	0.41425509	0.42366848	0.43307547	0.44246982	0.45174437	0.46089166	0.46985252	0.47661869	0.48353585
Surigao Del Norte	0.46896328	0.47288464	0.47654864	0.48035605	0.48489205	0.48980192	0.49473474	0.49930364	0.50312382	0.50679381	0.51047132	0.51378696	0.51683805	0.51991578	0.52303817	0.52596493	0.52927105	0.53315197	0.53740327	0.54182556	0.54712347	0.55304533	0.55981052	0.5670046	0.57460873	0.58242857	0.59042628	0.59850541	0.60659591	0.61465793	0.62097748	0.6274452
Surigao Del Sur	0.44007541	0.44520702	0.44996192	0.45474039	0.45989772	0.46504644	0.46985206	0.4739868	0.47723574	0.48026483	0.48312167	0.48546311	0.48735736	0.48915469	0.49099669	0.49265909	0.49469545	0.49741893	0.50071438	0.50435405	0.5090011	0.514462	0.520834	0.52769563	0.53506364	0.5427527	0.55072028	0.558849	0.56709674	0.57547677	0.58210371	0.58874071
Tarlac	0.51657719	0.5199222	0.52311017	0.52645056	0.53036813	0.53455624	0.53859097	0.54208588	0.54480916	0.54727708	0.54963501	0.55154068	0.55307732	0.55456012	0.55611742	0.55744202	0.55906925	0.56130022	0.5640823	0.56729701	0.5715885	0.57688769	0.58322587	0.5900956	0.59749217	0.60527788	0.61332015	0.62144073	0.62961204	0.6377408	0.6441904	0.65064553
Tawi-Tawi	0.36226329	0.36809861	0.37355857	0.37901949	0.38474841	0.3903613	0.39553529	0.39997237	0.40341122	0.40646638	0.40929984	0.41157424	0.41341358	0.41516651	0.41741326	0.41987784	0.42311573	0.42740368	0.43242302	0.43773004	0.44409331	0.45122105	0.45920788	0.46764062	0.47641084	0.48541296	0.49452887	0.503662	0.51277203	0.5218073	0.52879969	0.53581204
Zambales	0.53965418	0.54144611	0.54300487	0.54468964	0.54687247	0.5492271	0.55146769	0.55324069	0.55423059	0.55510224	0.55597225	0.5565431	0.55686696	0.55729537	0.55793563	0.55849005	0.55952704	0.56136711	0.5638859	0.56693501	0.57114626	0.57629563	0.5824457	0.58924208	0.59668824	0.60453675	0.61268794	0.62098829	0.62934206	0.63767085	0.64426807	0.6508406
Zamboanga Del Norte	0.39846476	0.40212783	0.40541258	0.40871726	0.4125698	0.41659396	0.42046487	0.42380208	0.42619496	0.42836274	0.43045888	0.43208335	0.43328012	0.43439178	0.43571821	0.4369501	0.43863676	0.44107476	0.44419401	0.44776552	0.45248399	0.45824558	0.46512348	0.47261233	0.48056852	0.48883426	0.49731943	0.5059329	0.51465801	0.52334977	0.53029097	0.53718454
Zamboanga Del Sur	0.47517651	0.47954149	0.48350175	0.48753037	0.4920596	0.49676932	0.50135883	0.50547606	0.50877701	0.51186457	0.51488974	0.51750465	0.51983278	0.52216686	0.52474886	0.52730215	0.53031595	0.53407927	0.53841831	0.54300328	0.54853417	0.55477975	0.56181956	0.56936313	0.5772568	0.58539659	0.59366976	0.60194062	0.61015045	0.61826857	0.62455206	0.63090878
Zamboanga Sibugay	0.38705559	0.39174472	0.39601762	0.40033669	0.40521062	0.41031649	0.41527972	0.41961087	0.42300755	0.42610139	0.429073	0.431565	0.43365052	0.43562788	0.43789889	0.44008967	0.44280836	0.44640623	0.45067228	0.45528606	0.46094767	0.46752674	0.47518019	0.48339369	0.49195798	0.50070771	0.5095203	0.51831286	0.52707055	0.53581192	0.54274036	0.54969602
Seychelles	0.5755265	0.58269833	0.59005292	0.59804419	0.60604127	0.61385328	0.62213517	0.63069773	0.63843888	0.64533967	0.65155199	0.65649067	0.66090779	0.66416203	0.66646135	0.66926474	0.67214991	0.67507744	0.67659095	0.67784703	0.68016364	0.68368149	0.68762939	0.69252573	0.69711459	0.70172282	0.70618362	0.71102728	0.71640782	0.72207368	0.72666377	0.73015077
Sri Lanka	0.52262255	0.5282392	0.53334554	0.5388911	0.54512455	0.55174406	0.55893889	0.56612842	0.57232619	0.57763005	0.58294264	0.5875566	0.59235913	0.59741028	0.60225006	0.60690666	0.61180708	0.61697927	0.62241432	0.62814963	0.63426312	0.64079957	0.64804982	0.65543584	0.66278243	0.66976029	0.67627744	0.68240527	0.68812586	0.69351606	0.69764854	0.70153494
Thailand	0.50664486	0.51591147	0.52516187	0.53430371	0.54332555	0.55245221	0.56130249	0.56844949	0.5738179	0.57885417	0.58350092	0.58766156	0.5917591	0.59604892	0.60052067	0.60504078	0.61035668	0.61636859	0.62106754	0.62432907	0.62858944	0.63322673	0.63841097	0.64367292	0.64877206	0.65401562	0.6593693	0.66480413	0.67022559	0.6752739	0.67911999	0.68254793
Timor-Leste	0.26246808	0.27094335	0.27991676	0.28926663	0.29876319	0.30801868	0.31758875	0.32670537	0.3348945	0.33925214	0.34379365	0.34949032	0.35416202	0.35844003	0.362688	0.36691358	0.37065409	0.37539077	0.38122753	0.38794482	0.39488702	0.40187236	0.4089103	0.41521788	0.42115554	0.42638362	0.43120727	0.43451973	0.43701866	0.44019585	0.44234934	0.44466762
Viet Nam	0.40763005	0.41321725	0.41978166	0.42707218	0.43510627	0.44385879	0.45310241	0.46285352	0.47247889	0.48144577	0.48980205	0.49763035	0.50545345	0.51336751	0.52122939	0.52895633	0.53630065	0.54334265	0.55012712	0.55645594	0.56276857	0.56894546	0.57507867	0.58102921	0.58691381	0.59284957	0.59884949	0.60501942	0.61135141	0.61773382	0.62309032	0.62793372
Sub-Saharan Africa	0.29714797	0.30063245	0.30387388	0.30684892	0.30970504	0.31272868	0.31613923	0.31969911	0.32330997	0.32702857	0.33090196	0.33496328	0.33935347	0.34403438	0.34924842	0.35502181	0.36107474	0.36756161	0.37410063	0.38055519	0.38731002	0.39412176	0.40091964	0.40775813	0.41467049	0.42143301	0.42788695	0.43425414	0.4406728	0.44717187	0.45311872	0.4585873
Central sub-Saharan Africa	0.30237495	0.30522059	0.30780357	0.30908204	0.30989798	0.31105925	0.3126744	0.31450168	0.31650628	0.318569	0.32084148	0.32374919	0.3275669	0.33193465	0.33742002	0.34409927	0.35145974	0.35956551	0.36848818	0.37666929	0.38538095	0.39469176	0.40423388	0.41352221	0.42257865	0.43098753	0.43874592	0.44617858	0.45324977	0.46015956	0.46645637	0.47225565
Angola	0.27073694	0.27516062	0.2792315	0.2818677	0.28448759	0.28744781	0.29142128	0.29543399	0.29955335	0.30372004	0.30760331	0.31169753	0.31670657	0.32177736	0.32739527	0.33378519	0.34101262	0.34884684	0.35708201	0.36440774	0.37203354	0.38000661	0.38789186	0.39579633	0.40372009	0.41137203	0.41882206	0.42617708	0.43335501	0.4404546	0.44728399	0.45372195
Central African Republic	0.21682519	0.22019166	0.22300754	0.22545113	0.22848772	0.23169679	0.23398959	0.236672	0.23974353	0.24298853	0.24602113	0.24924674	0.25252437	0.25510152	0.25777878	0.26045192	0.26371245	0.26733219	0.27115812	0.27533852	0.27998138	0.28509725	0.29046002	0.29010493	0.2899995	0.29082787	0.2924304	0.29475224	0.29801122	0.30195306	0.3054263	0.30916769
Congo (Brazzaville)	0.42065466	0.42673437	0.43198766	0.43660659	0.44016835	0.44374413	0.44756664	0.45102696	0.45444505	0.45717654	0.46076481	0.46375168	0.46703727	0.47013437	0.47361244	0.47813763	0.48335582	0.48764074	0.49282285	0.49829997	0.50554285	0.51381578	0.52292828	0.53155168	0.54059449	0.54841403	0.55510861	0.5618341	0.56779397	0.57337367	0.57845155	0.58307524
DR Congo	0.28984321	0.29043157	0.29095924	0.28962879	0.28685915	0.28396753	0.28041316	0.2763365	0.27181382	0.26736766	0.26245675	0.2578575	0.25413636	0.25233388	0.25247092	0.25416102	0.25735512	0.26227227	0.26810387	0.27436114	0.28222375	0.29087295	0.30045478	0.31083126	0.32162293	0.33242131	0.34198879	0.35110204	0.36028768	0.36934068	0.37671227	0.38317985
Equatorial Guinea	0.26878363	0.27529699	0.28348776	0.29223603	0.30068634	0.31044355	0.32460122	0.35055096	0.37123716	0.39112802	0.41138345	0.43359006	0.451939	0.46784683	0.48387961	0.49868372	0.51157983	0.52407646	0.53701899	0.54851468	0.55910938	0.57027742	0.5818339	0.59280061	0.60347317	0.61313462	0.62185816	0.63023454	0.63802282	0.64530309	0.6521249	0.65785746
Gabon	0.45542119	0.4615631	0.46702405	0.47239446	0.47806195	0.48382622	0.48946146	0.49507416	0.50037255	0.50480635	0.50913309	0.51304571	0.51691762	0.52105141	0.52547907	0.53048857	0.53479772	0.53944142	0.54411089	0.54859132	0.55402271	0.56023208	0.56684296	0.57380813	0.58091566	0.588365	0.5960623	0.60381625	0.61172438	0.61990473	0.62760965	0.63469139
Eastern sub-Saharan Africa	0.23362212	0.23634046	0.23861529	0.24081697	0.24290181	0.24542354	0.24858486	0.25214454	0.25589051	0.25995712	0.26416531	0.26877226	0.27348445	0.27843367	0.28393661	0.28997039	0.29645526	0.30357591	0.3109185	0.31845677	0.32629597	0.3342534	0.34162808	0.3492073	0.35700952	0.36490704	0.37275883	0.38049999	0.38835511	0.39634466	0.40348021	0.40972098
Burundi	0.20586736	0.20849572	0.21086462	0.21324939	0.21522297	0.21563168	0.21552633	0.21576182	0.2164026	0.21685577	0.21686986	0.21698873	0.21778248	0.21896154	0.22091871	0.22308437	0.22582189	0.22899682	0.23285355	0.23694304	0.24144779	0.24627728	0.2515723	0.25719136	0.26279472	0.26732794	0.27158785	0.2758405	0.27973329	0.28344251	0.286496	0.28937436
Comoros	0.27004812	0.27914565	0.28850627	0.29760742	0.30570659	0.31422034	0.32224907	0.33014812	0.33751791	0.34477469	0.35187551	0.359212	0.36648613	0.37341057	0.38003598	0.38689184	0.39352143	0.39905109	0.40464976	0.41025358	0.41554338	0.42109297	0.42672996	0.43278993	0.43884451	0.44471239	0.45050698	0.45628084	0.46195134	0.46738801	0.47200313	0.47597869
Djibouti	0.33778179	0.33877001	0.34098606	0.34490439	0.3479814	0.35124563	0.35438563	0.3580964	0.36159676	0.36530521	0.36901509	0.37285316	0.37665	0.38068662	0.38498771	0.38956871	0.39503747	0.40061855	0.4066583	0.41196933	0.41699935	0.42231614	0.42771144	0.43336044	0.43945572	0.44585528	0.45344285	0.46044015	0.46770567	0.47526324	0.48187995	0.48795837
Eritrea	0.21602824	0.22257474	0.23029607	0.23938504	0.25075254	0.26149848	0.27252705	0.28355172	0.29376483	0.30314234	0.31024816	0.31724799	0.32356658	0.32859358	0.33287938	0.33668251	0.33983582	0.34284317	0.3434278	0.34514774	0.34814618	0.35416315	0.36011275	0.36434365	0.37252759	0.37693516	0.38217167	0.38593603	0.39115488	0.39633056	0.40045941	0.40386394
Ethiopia	0.14803388	0.14959971	0.15017439	0.15158236	0.15298264	0.1550856	0.15812723	0.16187474	0.16507771	0.16890355	0.17341853	0.17897297	0.18413801	0.18869481	0.1950307	0.20286824	0.21152423	0.22181993	0.23287219	0.24426582	0.25608146	0.26788086	0.27900696	0.28988527	0.30048455	0.31073642	0.32043187	0.32946924	0.33805007	0.34642266	0.35321635	0.3588233
Addis Ababa	0.48706004	0.49570029	0.50205738	0.50860198	0.51436979	0.52029968	0.52668361	0.53318706	0.53839075	0.54373878	0.54939268	0.55542161	0.56043045	0.56431784	0.56943151	0.5755078	0.58179341	0.58915693	0.59692114	0.60488966	0.61329088	0.62176334	0.62952179	0.63741593	0.64555244	0.65380755	0.66176964	0.66940403	0.67681666	0.68417585	0.68996669	0.6947296
Afar	0.10250007	0.10292383	0.10230489	0.10242463	0.10249155	0.10331965	0.10512803	0.10761301	0.1093854	0.1117485	0.11480189	0.11905238	0.12278243	0.12576246	0.13060772	0.13697002	0.14420382	0.15310516	0.16278367	0.17290444	0.18356445	0.19430056	0.2046402	0.21494064	0.22521527	0.23547254	0.24550483	0.25517861	0.26462211	0.27398706	0.28194644	0.28877341
Amhara	0.09634946	0.09732577	0.0972893	0.0981463	0.09899303	0.10068193	0.10354246	0.10732105	0.11052187	0.11448427	0.11932173	0.12553943	0.13127708	0.13619058	0.14324911	0.15213541	0.16202923	0.17382063	0.18639961	0.19927525	0.2126035	0.22578478	0.23798506	0.24961776	0.26090417	0.27172065	0.28187013	0.29117117	0.29993476	0.30840144	0.31505898	0.32045319
Benishangul-Gumuz	0.0929276	0.0932423	0.0923732	0.09244251	0.0925012	0.09349795	0.09569905	0.09878089	0.10108727	0.10417273	0.10818158	0.11363627	0.11849202	0.12246567	0.12882057	0.137216	0.14690757	0.15884862	0.17184181	0.18538265	0.19953234	0.21347031	0.22643598	0.23921067	0.25166825	0.26378196	0.27530219	0.28602094	0.29611152	0.30580875	0.31370507	0.32022848
Dire Dawa	0.31220593	0.31740891	0.32136416	0.32596991	0.33041608	0.33529205	0.34074893	0.34648945	0.35142468	0.35667691	0.36228323	0.3682846	0.37345748	0.3777916	0.38340132	0.39013703	0.39752568	0.40621972	0.41557533	0.42532002	0.43556487	0.4458478	0.45562	0.46568671	0.47595852	0.48634401	0.49651842	0.50638749	0.51601433	0.52556768	0.53366763	0.54066865
Gambella	0.16861025	0.17373969	0.17753899	0.18211209	0.18635983	0.19095669	0.19612872	0.2017351	0.20636015	0.2115266	0.21745483	0.22461352	0.23119634	0.23711931	0.24538736	0.2555678	0.26698988	0.28069613	0.2955801	0.31103477	0.32707721	0.34285349	0.35748561	0.37176026	0.38576422	0.39934786	0.41203614	0.4234045	0.43390134	0.44395416	0.4518667	0.45822008
Harari	0.32284208	0.32743962	0.33035693	0.33374096	0.33684334	0.3404204	0.34467209	0.34911529	0.35260515	0.3564544	0.36060537	0.36532529	0.36946047	0.3729444	0.37798011	0.3843402	0.39140154	0.39987955	0.40909223	0.41866488	0.42869506	0.43878276	0.44832266	0.45822213	0.46856024	0.47925971	0.49003538	0.50059889	0.51105614	0.5214891	0.53044876	0.53816876
Oromia	0.12554488	0.12650957	0.12644179	0.12724957	0.12802516	0.12953496	0.1320164	0.13518952	0.13768975	0.14086015	0.14476873	0.14986609	0.15453324	0.15856473	0.16460966	0.17231514	0.18092932	0.19136077	0.20263326	0.21427913	0.22638323	0.23858833	0.25029045	0.26190747	0.27326951	0.28418588	0.29455197	0.30422946	0.313416	0.32234769	0.32969397	0.33581502
Somali	0.09359008	0.09411888	0.09375881	0.09410837	0.09438087	0.09526005	0.09693018	0.09912006	0.10059664	0.10256726	0.10517341	0.10876118	0.1119455	0.11450819	0.1187302	0.12439919	0.13091725	0.1390476	0.14804035	0.15757025	0.1676664	0.17788091	0.18780065	0.19775066	0.20767007	0.21752944	0.2270536	0.23620899	0.24514346	0.25402577	0.26165225	0.26825381
Southern Nations, Nationalities, and Pe	0.1324263	0.13344635	0.13314474	0.13385128	0.1345222	0.13610573	0.13892543	0.14251578	0.14516324	0.14852663	0.15273481	0.15831281	0.16324994	0.16728194	0.17364111	0.1819693	0.19136959	0.20285632	0.21523697	0.22804416	0.24140239	0.25472511	0.2672688	0.27940218	0.29102727	0.30235607	0.31306423	0.32310623	0.33260584	0.3418741	0.34933889	0.35552153

Table S5. Socio-demographic Index values for all estimated GBD 2021 locations, 1990-2021

Location	1990	1991	1992	1993	1994	1995	1996	1997	1998	1999	2000	2001	2002	2003	2004	2005	2006	2007	2008	2009	2010	2011	2012	2013	2014	2015	2016	2017	2018	2019	2020	2021
Tigray	0.13277103	0.13468207	0.13595766	0.13824969	0.14070419	0.14395787	0.14814146	0.15304046	0.157344	0.16222444	0.16787049	0.17469034	0.18138198	0.18763219	0.19577268	0.20558104	0.2164032	0.22904742	0.24256363	0.25637622	0.27021671	0.28342429	0.295752	0.3077623	0.31943002	0.33072634	0.34133269	0.3511351	0.36033055	0.36923235	0.37634669	0.38218351
Kenya	0.33385029	0.34047622	0.34629864	0.35161177	0.35677919	0.36153581	0.36620308	0.37043646	0.3745814	0.37863001	0.38239562	0.38618841	0.38988626	0.39368048	0.39774365	0.40244401	0.40774576	0.41373623	0.41973815	0.42612389	0.43330584	0.44089383	0.4486664	0.45666694	0.4647243	0.47308405	0.48170907	0.49029662	0.49910127	0.50800421	0.51613657	0.52376808
Baringo	0.27733209	0.28687801	0.29582204	0.30437222	0.31297431	0.32120033	0.32941504	0.33729216	0.34507728	0.35274271	0.36026177	0.3677072	0.37496099	0.38210732	0.38895144	0.39587713	0.40284885	0.40994404	0.41653543	0.42322585	0.43042939	0.43777825	0.44523747	0.45286559	0.46046064	0.46832563	0.47637004	0.48432326	0.49242459	0.50052297	0.50783667	0.51488321
Bomet	0.28194548	0.2889782	0.29529378	0.30134503	0.30755692	0.31362503	0.31969209	0.32547898	0.33127063	0.33719312	0.34323324	0.34934892	0.35546306	0.36177148	0.36834362	0.37558909	0.38328393	0.3915904	0.40000859	0.4088541	0.41837562	0.42814142	0.43815825	0.44836723	0.45863924	0.46914511	0.47968712	0.49000683	0.50038282	0.51072064	0.52036646	0.52936785
Bungoma	0.28928161	0.29668768	0.30324503	0.30916962	0.31480656	0.31990212	0.32482257	0.32933665	0.33375521	0.33806407	0.34201606	0.34596829	0.34983724	0.35388879	0.35830797	0.3635242	0.3694102	0.37593137	0.38237783	0.38928534	0.39713017	0.40544736	0.41394024	0.4227508	0.43167406	0.44081965	0.45027186	0.45978388	0.46954151	0.47933502	0.48823412	0.49672518
Busia	0.22949336	0.23750154	0.24528357	0.25302209	0.26076432	0.26829662	0.27567797	0.28265069	0.28951793	0.29635833	0.30278151	0.30904917	0.31520972	0.32128512	0.32740101	0.33406055	0.34113386	0.34876109	0.35623582	0.36404012	0.37258396	0.38140168	0.39034518	0.39962196	0.40893397	0.41852651	0.42842547	0.43836816	0.4485658	0.4588495	0.46846162	0.47766502
Elgeyo Marakwet	0.27279867	0.28222815	0.29088151	0.2990872	0.30722266	0.31502021	0.32270015	0.33003305	0.33735688	0.344706	0.35191274	0.35903312	0.36593207	0.37276681	0.3797114	0.38719587	0.39510572	0.40358442	0.41184969	0.42018957	0.42915307	0.43829199	0.44745659	0.45665741	0.46575298	0.47495817	0.48413876	0.49304217	0.50192682	0.51078433	0.51880107	0.52629704
Embu	0.3725255	0.37806986	0.38285962	0.38735561	0.39192078	0.39623837	0.40048957	0.40437428	0.40816546	0.41184617	0.41528903	0.41884967	0.42235551	0.42595306	0.42982265	0.43422769	0.4391254	0.44462982	0.45009413	0.45605514	0.46276491	0.46990422	0.47713466	0.48460733	0.49219877	0.50006134	0.50815116	0.51615204	0.5243762	0.53268266	0.54029862	0.5475554
Garissa	0.15861368	0.16207562	0.16544249	0.16880049	0.17238321	0.17603629	0.17987137	0.18367978	0.18755351	0.1914946	0.19529926	0.19935953	0.20350887	0.2078281	0.212455	0.21753118	0.22314372	0.22929606	0.2354951	0.24187021	0.24873366	0.25582027	0.26289237	0.26980041	0.27650687	0.28318003	0.28979082	0.29619717	0.30269397	0.30923082	0.31529842	0.32106827
Homa Bay	0.23103542	0.23820869	0.24496969	0.25161778	0.25793984	0.26391181	0.26961685	0.27481863	0.28023426	0.28554955	0.29059477	0.29554062	0.30017308	0.3049778	0.31024252	0.31645244	0.32342622	0.33135032	0.33957262	0.34864525	0.35879107	0.36957989	0.38094713	0.3929373	0.40514106	0.41777086	0.43074586	0.44375587	0.45697261	0.47018344	0.48259887	0.49387181
Isiolo	0.23378919	0.24013692	0.24602939	0.25170039	0.25751027	0.26308584	0.26876742	0.27424321	0.27968529	0.28506706	0.2902204	0.29538253	0.30044752	0.30553593	0.31049802	0.31571355	0.32120826	0.32686212	0.33216512	0.33791517	0.34450006	0.35170062	0.35906996	0.36685988	0.37473934	0.38296336	0.39158026	0.4001813	0.40898882	0.4179019	0.42612666	0.43396683
Kajiado	0.29932777	0.30629963	0.31229909	0.31776775	0.32318561	0.32812722	0.33300738	0.33743442	0.34169863	0.34578703	0.34982514	0.35388005	0.35791287	0.36201337	0.36634563	0.37149759	0.37725651	0.38376645	0.39051628	0.39771444	0.40562934	0.41384177	0.42223328	0.43102159	0.43998136	0.44933957	0.45898524	0.46861712	0.47843969	0.48840649	0.49800022	0.50696971
Kakamega	0.30916649	0.3160822	0.32214203	0.32753564	0.33254938	0.3369821	0.34124881	0.34513501	0.34903707	0.35283782	0.35637908	0.35982513	0.36310493	0.36643328	0.37019984	0.37474499	0.3800939	0.38640671	0.392941	0.40007452	0.40821719	0.41688159	0.42572593	0.4349214	0.4442112	0.4536624	0.46330091	0.47283502	0.48247028	0.49208871	0.50101962	0.509529
Kericho	0.30207479	0.30886448	0.31469895	0.31993667	0.32505265	0.32978184	0.33469019	0.33936091	0.34421904	0.34934019	0.35459767	0.36015215	0.36587346	0.37178928	0.37784211	0.38445833	0.39139443	0.39886043	0.4063073	0.41412668	0.42268709	0.43151634	0.44047099	0.44953552	0.45872171	0.46813571	0.47767996	0.48705026	0.49650186	0.5060331	0.51507995	0.52357336
Kiambu	0.41046936	0.41732057	0.42291446	0.42754295	0.43178245	0.43548962	0.43914341	0.44242197	0.44577181	0.449176	0.4524213	0.45573397	0.45902724	0.46260647	0.46667551	0.47155409	0.47715126	0.48352829	0.48998317	0.49674967	0.50424438	0.51206453	0.51986702	0.52781749	0.53571055	0.543913	0.55237716	0.56074853	0.56934094	0.57798717	0.58578667	0.59311015
Kilifi	0.25806592	0.26463814	0.2706432	0.27612922	0.28136699	0.28626767	0.29116174	0.29567814	0.2999119	0.30396034	0.30776104	0.31144386	0.31512961	0.31879326	0.32260586	0.32721863	0.33272644	0.33929043	0.34646581	0.35427583	0.363203	0.3728217	0.38298144	0.39355055	0.40442284	0.41584185	0.42760697	0.43943563	0.45138702	0.46343468	0.47510409	0.48600916
Kirinyaga	0.40648097	0.41213527	0.41648633	0.41992011	0.42311659	0.42583744	0.42835934	0.43041381	0.43245187	0.4345157	0.43636437	0.43832667	0.44020221	0.44220373	0.44435849	0.44702609	0.45030034	0.45447253	0.45866836	0.46331757	0.46882596	0.47469599	0.48066269	0.48689365	0.49323641	0.50005561	0.50722261	0.51438214	0.52178596	0.52935903	0.53631716	0.54301445
Kisii	0.3250303	0.33361211	0.34117525	0.34792826	0.35402016	0.35927805	0.36421181	0.36844775	0.3726328	0.3766571	0.38037322	0.38425154	0.38803172	0.39191847	0.39630802	0.4015104	0.40755891	0.41450806	0.42167082	0.42947671	0.43834573	0.44780142	0.4575415	0.46745597	0.47747863	0.48752835	0.49770319	0.50760074	0.51753212	0.52732535	0.53629584	0.54460484
Kisumu	0.30649772	0.31641633	0.32543617	0.33373587	0.34139611	0.34841067	0.35485575	0.36055684	0.36605227	0.37122032	0.37590796	0.38034198	0.38454179	0.38873661	0.39336326	0.39883663	0.4051727	0.41255596	0.42026899	0.42845044	0.43746431	0.44687155	0.45645513	0.46614566	0.47583221	0.48576018	0.49593723	0.50598977	0.51617172	0.52638188	0.5357574	0.54440932
Kitui	0.28150598	0.28787019	0.29342906	0.29857888	0.30363472	0.30832271	0.3128156	0.31699521	0.32113896	0.32515171	0.32895175	0.33260177	0.33629652	0.34014204	0.34433206	0.34927059	0.35472603	0.3607764	0.36675813	0.37324773	0.38067412	0.38858268	0.39666831	0.40510681	0.41365085	0.4224483	0.43142387	0.44027473	0.4492419	0.45827618	0.46673185	0.47474946
Kwale	0.27463023	0.28113916	0.28704066	0.29245678	0.29764394	0.3023206	0.30684919	0.31086504	0.31463621	0.31818574	0.32134295	0.32435135	0.32718985	0.33006997	0.33310789	0.3366711	0.34102117	0.34636993	0.3521558	0.35854506	0.36598907	0.37418784	0.38300052	0.39234386	0.40200224	0.41220808	0.42276399	0.43337097	0.44402927	0.45478114	0.46520963	0.47513821
Laikipia	0.37531182	0.38028994	0.38435457	0.38792161	0.39147231	0.39476489	0.39812225	0.40131984	0.40463517	0.40818291	0.41189389	0.41603417	0.42050785	0.42545425	0.43087243	0.43714841	0.44411975	0.45185932	0.45983744	0.46796876	0.47655284	0.4851985	0.49394184	0.50279173	0.51165431	0.52079312	0.53015216	0.53939312	0.54878653	0.55827591	0.56720183	0.57571177
Lamu	0.30868886	0.31585899	0.32230194	0.32826803	0.33414991	0.33973143	0.34516542	0.35011336	0.35477451	0.35923542	0.36335844	0.36741006	0.37141826	0.37538164	0.37941555	0.38391899	0.38905098	0.39479026	0.40051306	0.40653145	0.41333928	0.42057444	0.42808183	0.43582573	0.44361782	0.45191862	0.46054325	0.46921016	0.47816096	0.48724627	0.49569401	0.50378649
Machakos	0.36059665	0.36662955	0.3716981	0.37612379	0.38026096	0.38395835	0.38767449	0.39111104	0.39466023	0.3982618	0.40165728	0.40531003	0.40908603	0.41314001	0.41753381	0.42254273	0.42828797	0.4347004	0.44101769	0.4477737	0.45529974	0.46328831	0.47138925	0.47967766	0.48792392	0.4964443	0.50517693	0.51387265	0.52278938	0.53179865	0.54009577	0.54803579
Makueni	0.29531168	0.30240758	0.30879212	0.31480265	0.32078657	0.32634615	0.33179483	0.33694906	0.34208013	0.34708515	0.35185986	0.35656801	0.36123336	0.36607408	0.37123528	0.37717237	0.38371751	0.39092963	0.39804248	0.40557284	0.41400964	0.42282974	0.43165202	0.44070173	0.44967929	0.4588639	0.46819627	0.47735896	0.48666166	0.49599583	0.50447246	0.51256894
Mandera	0.11279696	0.11471049	0.1164992	0.11820603	0.12008136	0.12206423	0.1242503	0.1265717	0.12915355	0.13187903	0.13460548	0.1375094	0.14070298	0.1441618	0.14793749	0.15200491	0.1565108	0.16153015	0.16669031	0.17220618	0.1781146	0.18412552	0.19002917	0.19582674	0.20162299	0.20734981	0.21288621	0.2181795	0.22341598	0.2285393	0.23343644	0.23826383
Marsabit	0.20416759	0.20918192	0.21386183	0.21839261	0.22307763	0.22765943	0.23242213	0.23707352	0.24181788	0.2465365	0.25106416	0.25558303	0.26014092	0.26479248	0.26959364	0.27487534	0.28060571	0.28673229	0.29279861	0.2993658	0.30671286	0.31457732	0.32255832	0.33086408	0.33922532	0.34785963	0.35677731	0.36561218	0.3745712	0.38360518	0.39207979	0.40018158
Meru	0.31388171	0.32028248	0.32585505	0.33085257	0.33571744	0.34026206	0.34467498	0.34859634	0.35240216	0.35610458	0.35959025	0.36316546	0.36672433	0.37051689	0.37474811	0.3796408	0.38518795	0.39147474	0.39781287	0.40455772	0.41207553	0.42002137	0.42812692	0.43645421	0.44493826	0.4538223	0.46297858	0.47206584	0.48136409	0.49080099	0.49971654	0.50821615
Migori	0.19986554	0.20588387	0.21199316	0.21843986	0.22488455	0.23135671	0.2378687	0.24406471	0.25054333	0.2571417	0.2635732	0.26982946	0.27590578	0.28214766	0.28865359	0.29585831	0.30346298	0.31174422	0.32031054	0.32946746	0.33942096	0.3498345	0.36078118	0.37213835	0.38367253	0.39571105	0.40839196	0.42127645	0.43443386	0.44776645	0.46078865	0.47280301
Mombasa	0.40787314	0.41395275	0.4192163	0.42393569	0.42856749	0.43261654	0.4369127	0.44074957	0.4443741	0.44787693	0.45100524	0.45442917	0.45770631	0.46099166	0.46472932	0.46926463	0.47486824	0.48158893	0.48845504	0.49565237	0.50364394	0.51213761	0.52080746	0.52964475	0.53831094	0.54705859	0.55588246	0.56440592	0.57302215	0.58157873	0.58916859	0.59638778
Murang'a	0.36781457	0.37465438	0.38042455	0.38563214	0.39071109	0.39541158	0.3999398	0.40405497	0.40821041	0.4124021	0.41638265	0.4203242	0.42420926	0.42818837	0.43238445	0.43706301	0.44220975	0.44799978	0.45371495	0.45978679	0.46661773	0.47378175	0.48096035	0.48830174	0.49563739	0.50333779	0.51132457	0.51930252	0.52756449	0.53594549	0.54357788	0.55086949
Nairobi	0.52277872	0.52729583	0.53093629	0.53405764	0.53729997	0.54033999	0.54362148	0.54666433	0.54982857	0.55330665	0.55674507	0.56066175	0.56458962	0.56869147	0.57302695	0.57790573	0.58340574	0.58956907	0.59554139	0.60163438	0.60837269	0.61533231	0.62218961	0.62896797	0.6355324	0.64233846	0.64942335	0.65644035	0.66370308	0.67099893	0.67750179	0.68372244
Nakuru	0.35583072	0.36297868	0.36906454	0.37453583	0.37991466	0.3847881	0.38972562	0.3942518	0.39872702	0.40322297	0.40762374	0.41232169	0.41693325	0.42163269	0.42699353	0.43325419	0.44054664	0.44879448	0.45699148	0.46538676	0.47440038	0.48366594	0.49293477	0.50219343	0.51125128	0.52028537	0.52929916	0.53792534	0.54658946	0.55516073	0.56279661	0.5700454
Nandi	0.28995178	0.29853213	0.30628018	0.31351369	0.32051249	0.3270702	0.33328517	0.33899673	0.34442121	0.34967013	0.35473974	0.35965211	0.36450596	0.36924546	0.37408417	0.37943795	0.38518209	0.3917347	0.39854001	0.40581572	0.41379656	0.42209894	0.43072787	0.43971352	0.44895729	0.45857811	0.46833688	0.47796967	0.48774354	0.49768713	0.50725822	0.51627971
Narok	0.21300106	0.21983286	0.22626394	0.23261557	0.23909751	0.24539736	0.2516631	0.25763425	0.26361473	0.26965379	0.27584801	0.28205725	0.28840901	0.29478531	0.30108875	0.30787153	0.31485739	0.32228601	0.32995789	0.33814823	0.34707429	0.35633481	0.36586487	0.37565561	0.38574137	0.39618848	0.40679733	0.4173734	0.42805452	0.43882838	0.44929615	0.45910307
Nyamira	0.3538386	0.36193387	0.36932022	0.37613247	0.38274759	0.38888831	0.39471945	0.39992248	0.40508713	0.41023468	0.41530409	0.42038402	0.42531827	0.43048923	0.43613874	0.44249102	0.44933411	0.4569359	0.46445611	0.47263268	0.48183352	0.4914672	0.50138743	0.51158593	0.52189969	0.53227555	0.54259666	0.55257876	0.56251727	0.57232096	0.58118467	0.58926708
Nyandarua	0.37402032	0.38002996	0.38496514	0.38929406	0.3936867	0.39790393	0.40234727	0.40683852	0.41164302	0.41667614	0.42171721	0.4270748	0.43250056	0.43811961	0.4438892	0.45013407	0.45703348	0.46449859	0.47175062	0.47923949	0.48725052	0.49545696	0.50355318	0.51173563	0.51983166	0.52815573	0.53657032	0.54478966	0.55310811	0.56141338	0.56890929	0.57610986
Nyeri	0.40915722	0.41528834	0.4202853	0.42467117	0.42890928	0.43282131	0.43657565	0.43991807	0.44321536	0.44640634	0.44935217	0.45246709	0.45554338	0.45871321	0.46239312	0.46694089	0.4722988	0.47860757	0.48490753	0.49146844	0.49873753	0.50623899	0.51363901	0.52107548	0.52842088	0.53589522	0.54344866	0.55077546	0.5582799	0.5658029	0.57243095	0.57878748
Samburu	0.20384115	0.20847203	0.21241941	0.2160685	0.21978518	0.2232594	0.22682888	0.23024927	0.23374577	0.23722832	0.24070063	0.24433472	0.24798385	0.25169334	0.2555988	0.26005399	0.26492789	0.27031853	0.2757737	0.28167267	0.28823706	0.29515973	0.30237877	0.30988598	0.31759035	0.32583984	0.3344649	0.3430872	0.35205085	0.3612377	0.37000448	0.3783124
Siaya	0.22980971	0.23693969	0.24360731	0.25012	0.25651642	0.26258297	0.26846617	0.27375566	0.27908493	0.28434942	0.28932821	0.29424968	0.29897473	0.30383257	0.30918423	0.31546086	0.32247835	0.33029095	0.3382463	0.34683951	0.35642434	0.36663269	0.37725414	0.38813675	0.39915091	0.41029717	0.42171914	0.43306226	0.44451682	0.45591235	0.46663229	0.4765244
Taita Taveta	0.3436025	0.34973319	0.35508382	0.35999918	0.36484054	0.36937882	0.37401362	0.37826783	0.38233852	0.38623138	0.38979897	0.3935252	0.39721597	0.40092075	0.40492704	0.40959221	0.41515821	0.42170599	0.42840066	0.43544198	0.44333336	0.45173013	0.4603975	0.46926755	0.47814447	0.48726442	0.49656209	0.50571664	0.51498696	0.52426924	0.53276871	0.54098098
Tana River	0.23178974	0.23630482	0.24005518	0.24317251	0.24606803	0.24847263	0.25066338	0.2523352	0.25367282	0.25482098	0.25568915	0.25649831	0.25728435	0.25805622	0.25922065	0.26126131	0.26432605	0.26838592	0.27277766	0.27783552	0.28408346	0.2910988	0.29868733	0.3069085	0.3154006	0.32450322	0.33402448	0.34367519	0.35352843	0.36355944	0.37345301	0.38284535
Tharaka Nithi	0.31129438	0.31816152	0.32440645	0.33022076	0.33601021	0.34148973	0.34683487	0.35198098	0.35707821	0.36209336	0.36689183	0.37162648	0.37651194	0.38163932	0.38708279	0.39324083	0.40003186	0.4074344	0.41482035	0.42269232	0.4314317	0.44045676	0.44955571	0.45878203	0.46793069	0.47715169	0.48638513	0.4953951	0.50444461	0.51349274	0.52178997	0.52968011
Trans Nzoia	0.32615715	0.33404023	0.34103614	0.34735654	0.35349731	0.35901831	0.36447708	0.36946461	0.37446073	0.3794964	0.3844205	0.38957093	0.39475898	0.4002202	0.40589697	0.41198674	0.41846072	0.42554918	0.43267935	0.44011034	0.44818471	0.45662743	0.46527019	0.47408703	0.48301679	0.49231628	0.50189977	0.51136886	0.52104663	0.53079554	0.53986969	0.54850455
Turkana	0.17963154	0.1845953	0.18932007	0.19401223	0.19891183	0.20368939	0.20862563	0.21344457	0.21835903	0.22321956	0.22786553	0.23258178	0.23728846	0.24207306	0.24684801	0.2519513	0.25732239	0.26312162	0.26895789	0.27534796	0.28253537	0.2901888	0.29809817	0.30628331	0.31471543	0.32341465	0.33228026	0.34097037	0.34958168	0.35797862	0.36591471	0.37343424
Uasin Gishu	0.35112137	0.35898871	0.36583811	0.37197651	0.37801782	0.38359007	0.3891049	0.39428132	0.39937779	0.40455488	0.40964379	0.41485127	0.4201437	0.4256286	0.43129544	0.43744848	0.44400576	0.4510338	0.45793381	0.46514626	0.47296452	0.48106746	0.48936919	0.49784053	0.50638642	0.51517421	0.52411778	0.53296834	0.54195286	0.55096959	0.55934577	0.56734559
Vihiga	0.29284767	0.3008052	0.30818241	0.31538513	0.32261647	0.32954091	0.33634874	0.34266026	0.34890136	0.35516175	0.3611816	0.36723014	0.37308259	0.3788441	0.38468248	0.39112676	0.39806668	0.40559811	0.4128218	0.42027675	0.42848862	0.43693298	0.44545206	0.45414737	0.46268723	0.47145079	0.48042863	0.48925912	0.49820909	0.50720557	0.51532506	0.52308979
Wajir	0.11463278	0.11733	0.12008756	0.12283221	0.12575899	0.12868182	0.13174747	0.13486616	0.13804187	0.14122878	0.14435906	0.14763667	0.15106587	0.15465826	0.15847489	0.16259706	0.16714514	0.17210837	0.17713011	0.18244053	0.18821	0.19427243	0.20030946	0.20634144	0.21229647	0.21821194	0.2240721	0.22976103	0.23551673	0.24129824	0.24676802	0.25205011
West Pokot	0.21564318	0.22239318	0.22865749	0.23462591	0.24055679	0.24610384	0.25164162	0.25679919	0.26198374	0.26722024	0.27246151	0.27780292	0.28321097	0.28880577	0.29463593	0.30113574	0.30798146	0.31536315	0.32274818	0.3305135	0.33896688	0.34782901	0.35706252	0.36657439	0.37624551	0.3861947	0.39625639	0.40613952	0.41605866	0.42600268	0.43532573	0.4441209
Madagascar	0.27988946	0.28042959	0.28050128	0.2802043	0.27961515	0.279164	0.27906649	0.27942619	0.28031767	0.28157107	0.28319072	0.28566818	0.28715315	0.28967546	0.29262988	0.29591933	0.29972339	0.30402624	0.30899026	0.31345089	0.31835318	0.32380104	0.32994754	0.33651923	0.34335261	0.3504436	0.35802634	0.36623559	0.37496964	0.3842092	0.39277871	0.40024694
Malawi	0.20401024	0.20604379	0.20673236	0.2080912	0.20914457	0.21175623	0.21526447	0.21941625	0.22312233	0.22706837	0.23090081	0.23402051	0.23760216	0.24155923	0.24643454	0.25167232	0.25759565	0.26522693	0.27368986	0.28229651	0.29117962	0.30044851	0.30919762	0.31793621	0.32704243	0.33614014	0.34473154	0.35312107	0.36147041	0.37011461	0.37787902	0.38455363

Table S5. Socio-demographic Index values for all estimated GBD 2021 locations, 1990-2021

Location	1990	1991	1992	1993	1994	1995	1996	1997	1998	1999	2000	2001	2002	2003	2004
Mozambique	0.17306472	0.17555044	0.17728519	0.17985181	0.18223686	0.18342798	0.18570587	0.18865044	0.19199863	0.19543529	0.1985344	0.20224192	0.20639006	0.21053449	0.21497246
Rwanda	0.27509719	0.2761947	0.27717817	0.27698013	0.27233126	0.27110223	0.27109792	0.27245586	0.27458255	0.27701439	0.28098345	0.28608205	0.29295543	0.29967692	0.30694392
Somalia	0.04884856	0.04942238	0.05011553	0.050798	0.0514717	0.05214861	0.05284055	0.05357097	0.05432153	0.05507184	0.05584206	0.05664316	0.05745761	0.05827215	0.05907695
South Sudan	0.2066565	0.20893272	0.2111994	0.21341647	0.21561209	0.21792046	0.22039944	0.22303717	0.22578887	0.22864859	0.23173034	0.23501413	0.23834119	0.24179719	0.2454625
Uganda	0.1870011	0.18892416	0.19100343	0.19355746	0.19729319	0.20183542	0.20708073	0.2130917	0.21971244	0.22691694	0.23432323	0.24218639	0.25055239	0.25916488	0.26800461
Tanzania	0.25930607	0.26241624	0.26498945	0.26725492	0.2694172	0.2720782	0.27550791	0.27925918	0.28315261	0.28738168	0.29182726	0.29671565	0.301984	0.30761549	0.31377298
Zambia	0.30400855	0.30584236	0.30731628	0.30878021	0.30922345	0.30999581	0.31132669	0.31308733	0.31492643	0.31739644	0.32062532	0.32440137	0.32868012	0.3337214	0.33948892
Southern sub-Saharan Africa	0.50694663	0.51237779	0.51756947	0.5227725	0.52806332	0.53337978	0.5387804	0.54413428	0.54914167	0.55388466	0.55849062	0.56251127	0.56604984	0.56921519	0.5723897
Botswana	0.41807775	0.42975229	0.44049084	0.45052361	0.45995497	0.46926651	0.47858835	0.48776786	0.49619362	0.50497883	0.51349822	0.52137018	0.52925296	0.53700018	0.54434922
Eswatini	0.39942095	0.40826997	0.41668338	0.42480299	0.43250347	0.44012705	0.44739924	0.45408985	0.46013698	0.46599085	0.47185284	0.47723337	0.48247442	0.48760883	0.49266916
Lesotho	0.33915513	0.34549657	0.35216963	0.35878046	0.3653108	0.37126532	0.3772212	0.38309665	0.38840648	0.393495	0.3986358	0.40399013	0.40902166	0.41398257	0.41867092
Namibia	0.45004023	0.45453444	0.46002131	0.46573413	0.47187322	0.47779433	0.48311638	0.48801294	0.49281255	0.49761386	0.50240696	0.50671903	0.51081827	0.51457465	0.5191036
South Africa	0.54157144	0.54692114	0.5520669	0.55719844	0.56244864	0.5679053	0.57345399	0.57909928	0.58443731	0.58940735	0.59409368	0.59788417	0.60092168	0.6035156	0.60626251
Eastern Cape	0.48715663	0.49194577	0.49655875	0.50122723	0.5059891	0.51093299	0.51604844	0.52136192	0.52646737	0.53126789	0.53587488	0.53946484	0.54227296	0.5448034	0.54760921
Free State	0.54746237	0.55288382	0.55782977	0.56261717	0.56735304	0.57204963	0.57661477	0.58112693	0.58530681	0.58910135	0.59266132	0.59559149	0.59809989	0.60023778	0.60247516
Gauteng	0.63485054	0.63835467	0.64181829	0.64537204	0.64914906	0.65314495	0.6572921	0.66143214	0.6651077	0.66825071	0.67101892	0.67293906	0.67416507	0.6749922	0.67604683
KwaZulu-Natal	0.51359711	0.51988313	0.52587253	0.53168507	0.53742918	0.54332559	0.54924773	0.55518982	0.56085484	0.56617793	0.57121523	0.5753755	0.57878692	0.58173547	0.58480061
Limpopo	0.44506187	0.45261151	0.45985604	0.4671972	0.47478303	0.4825359	0.49038907	0.49845894	0.50623447	0.51369754	0.52094561	0.52718196	0.53241909	0.53689973	0.5412736
Mpumalanga	0.48855668	0.49703714	0.50542614	0.5136843	0.52197561	0.53017023	0.53775456	0.54482196	0.551184	0.55696781	0.56228324	0.56662508	0.56993159	0.57236109	0.57467005
North West	0.51158202	0.51651919	0.52113466	0.52546258	0.52959693	0.5337387	0.53798864	0.54250056	0.54690915	0.55107198	0.5550737	0.55826226	0.56072338	0.56280564	0.56517049
Northern Cape	0.5281267	0.53220469	0.535917	0.53958431	0.54347987	0.54788172	0.55271327	0.55806787	0.56348988	0.56881043	0.57398248	0.57837268	0.58197434	0.58498589	0.58780972
Western Cape	0.63062179	0.63312488	0.63513785	0.63689241	0.63871643	0.6408907	0.64345619	0.64636603	0.64910933	0.65168892	0.6541842	0.65612973	0.65774184	0.65929781	0.66126574
Zimbabwe	0.39855934	0.40605288	0.41208383	0.4179742	0.42405156	0.42892904	0.43418719	0.43814199	0.44109313	0.4432466	0.4448414	0.44635468	0.4469709	0.44600218	0.4436964
Western sub-Saharan Africa	0.2737	0.27741073	0.28102468	0.28443172	0.28769958	0.2909832	0.29461847	0.29830244	0.3020258	0.30580978	0.30978096	0.31402809	0.318881	0.32423289	0.33022827
Benin	0.21890715	0.22253902	0.22632347	0.23031259	0.23403204	0.2380097	0.24189532	0.24573153	0.24937645	0.25321534	0.25738068	0.26138801	0.26541746	0.26954428	0.27394114
Burkina Faso	0.12969562	0.13329913	0.1365948	0.13982546	0.14284897	0.14611567	0.15004169	0.1542284	0.15870705	0.16346807	0.16808434	0.1729964	0.17798199	0.18325354	0.188565
Cabo Verde	0.27672337	0.28293172	0.28846037	0.29451729	0.30221679	0.31067563	0.31973623	0.32904959	0.33878607	0.34929692	0.35989902	0.37007028	0.37984667	0.38941979	0.39883873
Cameroon	0.30305533	0.30962346	0.31560127	0.32083165	0.32541012	0.32953932	0.33342598	0.337346	0.34119709	0.34495536	0.34881479	0.35263187	0.35651182	0.36066858	0.36510003
Chad	0.11463883	0.11795463	0.12113283	0.12360007	0.12608057	0.12828949	0.13016948	0.13213602	0.1340903	0.13582526	0.13741237	0.13966369	0.1420417	0.14534805	0.15091087
Côte d'Ivoire	0.27932035	0.28649268	0.29345129	0.29974322	0.30521065	0.31010551	0.31496539	0.31984398	0.32471287	0.32920101	0.33301539	0.33654551	0.33997764	0.34334893	0.34637541
The Gambia	0.23871485	0.24501016	0.25108786	0.25699614	0.26242479	0.26769651	0.27295019	0.27827412	0.28394606	0.29007062	0.29635428	0.30254326	0.30771293	0.31293092	0.31861503
Ghana	0.373112	0.37923926	0.38485677	0.38996849	0.39495366	0.3999302	0.40500305	0.41003807	0.41488853	0.4197555	0.42472521	0.42941185	0.43410413	0.43911821	0.44423698
Guinea	0.17829542	0.18145939	0.18448502	0.18768624	0.19112884	0.19457324	0.19863376	0.20328045	0.20800338	0.21241271	0.21664979	0.22109094	0.22589878	0.23052259	0.23513497
Guinea-Bissau	0.20761484	0.21250598	0.21702609	0.22167898	0.22643493	0.23122353	0.23610637	0.24136058	0.2443718	0.24821732	0.25202652	0.25578677	0.25932182	0.26274772	0.26627174
Liberia	0.23529685	0.23821222	0.23828331	0.23598441	0.23265677	0.22842448	0.22268259	0.21967276	0.21905385	0.22221287	0.23117336	0.23959439	0.24831197	0.25159546	0.25517961
Mali	0.12652643	0.12912815	0.13153745	0.13417705	0.13693783	0.13986623	0.14307892	0.14641433	0.14998673	0.15373734	0.15719003	0.16134445	0.1654207	0.1697335	0.17377955
Mauritania	0.33578094	0.34113517	0.34653129	0.35217657	0.35758591	0.3630377	0.36850833	0.37302134	0.37708025	0.3811123	0.38420538	0.38648742	0.38856448	0.39110488	0.39401293
Niger	0.08086848	0.08284821	0.08477158	0.08669649	0.08860873	0.09047371	0.09234636	0.09421911	0.09654515	0.09881369	0.10088695	0.10327178	0.10574111	0.10832289	0.1107645
Nigeria	0.30586805	0.30848923	0.31123405	0.31398306	0.31674745	0.31963744	0.32313768	0.32669055	0.33026385	0.3339365	0.33820174	0.34302975	0.34909253	0.35591139	0.36378081
São Tomé and Príncipe	0.30954285	0.31060103	0.31151545	0.31274356	0.31426109	0.31584392	0.31772929	0.31979567	0.32251922	0.32565192	0.32946011	0.33448479	0.34030284	0.34685198	0.35372238
Senegal	0.23804761	0.24448218	0.25041653	0.25576796	0.26038	0.26443877	0.26831896	0.27207288	0.27599566	0.28011192	0.2837849	0.2874519	0.29104629	0.29488717	0.29898452
Sierra Leone	0.21156934	0.21301357	0.21422304	0.2160892	0.21827741	0.2198226	0.21996927	0.21908979	0.21858431	0.21794304	0.21849284	0.22039123	0.22458583	0.2292359	0.23413167
Togo	0.26969227	0.27453057	0.27878931	0.28122463	0.28465339	0.28853669	0.29260668	0.29694784	0.30002233	0.30302023	0.30547296	0.30782826	0.31046991	0.31379206	0.31668794

Location	2005	2006	2007	2008	2009	2010	2011	2012	2013	2014	2015	2016	2017	2018	2019	2020	2021
Mozambique	0.21969237	0.22475809	0.23015823	0.23579017	0.24136066	0.2467515	0.25223247	0.25831556	0.26528046	0.27289281	0.28103269	0.28915242	0.29716477	0.30522677	0.31317451	0.32029118	0.32646261
Rwanda	0.31421958	0.32199852	0.32961659	0.33703923	0.34449463	0.35165942	0.35894034	0.36641182	0.37376968	0.38153318	0.38989541	0.39828796	0.40641147	0.41465791	0.42308673	0.4298966	0.43558871
Somalia	0.05989854	0.06074148	0.06159815	0.06247539	0.06338223	0.06432213	0.06530681	0.06635488	0.06745949	0.0686093	0.06981152	0.07106405	0.07233478	0.07364225	0.07497981	0.07636337	0.07768811
South Sudan	0.2492809	0.25330051	0.25749176	0.26174046	0.26617923	0.27057262	0.27471914	0.27506614	0.27581394	0.27631146	0.27583363	0.27445549	0.27438786	0.27498785	0.2757062	0.27678792	0.27837113
Uganda	0.27736919	0.28703058	0.29674042	0.30674884	0.31673115	0.32654366	0.33621772	0.34536452	0.35438162	0.3634309	0.37253799	0.38131223	0.39008226	0.39911778	0.40843156	0.41684608	0.42326118
Tanzania	0.32043734	0.327234	0.33431324	0.34135263	0.34837803	0.35556756	0.36303632	0.37041249	0.37796317	0.38597458	0.39427776	0.40287931	0.41182497	0.42098536	0.43022538	0.43874724	0.44656827
Zambia	0.34604886	0.35354071	0.36245097	0.3716885	0.38166712	0.39247099	0.40347563	0.4145745	0.4254923	0.43624401	0.44674472	0.4571226	0.46753263	0.47788771	0.48791152	0.49748556	0.50594895
Southern sub-Saharan Africa	0.57611569	0.58068483	0.585673	0.59040532	0.59458507	0.59883555	0.60334685	0.60803777	0.61284381	0.61745259	0.62183758	0.62591906	0.6297615	0.63341066	0.63694019	0.63988183	0.64220028
Botswana	0.55170697	0.55922162	0.5667025	0.57388381	0.57969641	0.58582415	0.59198546	0.59773955	0.60406473	0.61008605	0.61541112	0.62079757	0.62570495	0.63060684	0.63532409	0.6392753	0.64272163
Eswatini	0.49804385	0.50365829	0.50926022	0.5147309	0.5204274	0.5262458	0.53191391	0.53779802	0.54383642	0.54961385	0.55537374	0.56106119	0.56669849	0.57215481	0.57720631	0.58165307	0.58545971
Lesotho	0.42321123	0.42780673	0.43274504	0.43834057	0.44358013	0.44896648	0.4545301	0.46041969	0.46669226	0.47318816	0.47975446	0.48614483	0.49197815	0.49738179	0.50245732	0.50674665	0.51039307
Namibia	0.52388174	0.52935012	0.53506213	0.54089699	0.54651648	0.55274157	0.5596189	0.5669787	0.57459034	0.58232142	0.58982104	0.59628306	0.60175464	0.6066184	0.61092109	0.61443589	0.61756487
South Africa	0.60998998	0.61501469	0.62060815	0.62594803	0.63064563	0.63526854	0.63999172	0.64471566	0.64943055	0.65396449	0.65841776	0.66264249	0.66662261	0.67033537	0.67404104	0.6771661	0.6796266
Eastern Cape	0.55139461	0.55641342	0.56194656	0.56694482	0.57110398	0.57515226	0.5792332	0.58339892	0.58767183	0.59183366	0.59599022	0.59997241	0.60386294	0.60756497	0.61135182	0.61463222	0.6172047
Free State	0.60569103	0.61041265	0.61594219	0.62140353	0.62640535	0.63153084	0.63683378	0.64221275	0.647592	0.65273241	0.65756921	0.66189221	0.66580999	0.66939803	0.67288493	0.6757477	0.6779086
Gauteng	0.67807479	0.68139232	0.68528898	0.6890725	0.69246862	0.69595935	0.69969533	0.70354202	0.7075252	0.7114802	0.71551531	0.71941139	0.72314472	0.72667679	0.73020676	0.73314709	0.73542663
KwaZulu-Natal	0.58896077	0.59451299	0.60066207	0.60656677	0.61163366	0.61642504	0.62123685	0.62600658	0.63069576	0.63517323	0.63958765	0.64377135	0.64773975	0.65141775	0.6550703	0.65812815	0.6604837
Limpopo	0.54631837	0.55238608	0.5589643	0.56522629	0.57071318	0.57600195	0.58110199	0.58591085	0.59050406	0.59461867	0.59829389	0.60148795	0.60413435	0.60646107	0.60889869	0.61084494	0.61228784
Mpumalanga	0.578043	0.58284765	0.58837325	0.59384114	0.59889605	0.60391027	0.60897037	0.61382709	0.61847148	0.62285777	0.62709222	0.63111764	0.63480553	0.63810419	0.64128793	0.64395427	0.64605747
North West	0.5688307	0.57418797	0.58047243	0.58670005	0.59231345	0.59794054	0.60388036	0.60983482	0.61576094	0.62148756	0.62720277	0.63267377	0.63758414	0.64194456	0.64616033	0.64971093	0.65247643
Northern Cape	0.59119745	0.59557452	0.60024316	0.60447754	0.60794924	0.61148962	0.61557621	0.62031935	0.625579	0.63103997	0.63666516	0.64221084	0.64748292	0.65240921	0.65714213	0.66107246	0.66414062
Western Cape	0.6640782	0.66788147	0.67202766	0.67591549	0.67938032	0.68294892	0.68670705	0.69046072	0.69416794	0.69767622	0.70107381	0.7043196	0.70754415	0.7106979	0.713881	0.71651464	0.7185329
Zimbabwe	0.43965166	0.43440576	0.42916514	0.42244897	0.41833061	0.41730536	0.41972969	0.42574852	0.43288189	0.43999723	0.44655287	0.45247304	0.45842498	0.46437926	0.46838444	0.47157317	0.47381949
Western sub-Saharan Africa	0.33677971	0.34324817	0.35004017	0.35671646	0.36349154	0.37060875	0.37760914	0.38475339	0.39189044	0.39916209	0.4062382	0.41292729	0.41961502	0.42643735	0.43338389	0.43982233	0.44602298
Benin	0.27840675	0.28293394	0.28767305	0.29262745	0.29770223	0.30280901	0.30792554	0.31337359	0.31931869	0.32572727	0.33221635	0.33876392	0.34560676	0.3526527	0.36003116	0.36696449	0.37348657
Burkina Faso	0.19408613	0.19957195	0.20489109	0.21022329	0.21542337	0.22100103	0.22669663	0.23258913	0.23849749	0.2443789	0.25020826	0.25608177	0.26203301	0.26810207	0.27428709	0.27987132	0.2851184
Cabo Verde	0.40800922	0.41743379	0.42737979	0.43737213	0.44669151	0.45564757	0.46447397	0.47285777	0.48079648	0.4882599	0.49522949	0.50185542	0.50843579	0.51528589	0.52248801	0.52846159	0.53353454
Cameroon	0.36954576	0.37435588	0.37949035	0.3848383	0.39028864	0.39606715	0.4020928	0.40858012	0.41560796	0.42303356	0.43083028	0.43887895	0.4471851	0.45568785	0.46424647	0.47228585	0.47969122
Chad	0.15703304	0.1625764	0.16816106	0.17339678	0.17833536	0.18411062	0.18959161	0.19526275	0.20095288	0.20665884	0.2124287	0.21753218	0.22220695	0.22691388	0.23170475	0.23610308	0.24043602
Côte d'Ivoire	0.34872791	0.35071594	0.35272748	0.35508764	0.35793875	0.36120519	0.36408365	0.36783169	0.37235379	0.37757544	0.38349138	0.38982519	0.39669397	0.40409193	0.41172558	0.4190401	0.42594188
The Gambia	0.32356835	0.32815143	0.33264015	0.33731525	0.34238961	0.34799783	0.35286924	0.35837365	0.36425588	0.3699013	0.37584048	0.38156922	0.38727364	0.39315568	0.39918978	0.40452419	0.40971416
Ghana	0.44936002	0.45469274	0.46060625	0.46725907	0.47384805	0.48077789	0.48902097	0.49751393	0.50600465	0.51411906	0.5215392	0.52862959	0.53616994	0.54384054	0.55163203	0.55846217	0.56493039
Guinea	0.23969185	0.24401013	0.24847169	0.25316769	0.25755279	0.26240595	0.2676246	0.27342832	0.27960044	0.28596126	0.29236361	0.29911241	0.30688146	0.31483959	0.32283985	0.329878	0.33640129
Guinea-Bissau	0.27005613	0.27380399	0.27780502	0.2821515	0.28642335	0.29114164	0.29668297	0.30156234	0.30660201	0.31160291	0.31709001	0.32297967	0.32932649	0.33566783	0.34215611	0.347799	0.35310962
Liberia	0.25896799	0.26289726	0.26723957	0.2718536	0.27680411	0.28223575	0.28801514	0.29485432	0.30298566	0.31095544	0.31848517	0.32508517	0.33152779	0.33778458	0.34340326	0.34813389	0.35244245
Mali	0.17802984	0.18245247	0.18691299	0.19153675	0.19653765	0.20180354	0.20709722	0.21246506	0.21799387	0.22372998	0.22982626	0.23618623	0.24272613	0.24941973	0.25625075	0.26262922	0.26857994
Mauritania	0.3974991	0.40315947	0.40837712	0.41323362	0.41791061	0.42320244	0.42886729	0.43472438	0.44106891	0.44765388	0.45452349	0.46126954	0.46845237	0.47583951	0.48376774	0.4913656	0.4989451
Niger	0.11345202	0.11624707	0.11897163	0.12195779	0.12482938	0.127953	0.13095807	0.13446972	0.13797817	0.14173174	0.1454405	0.14920011	0.15295917	0.1568752	0.16088948	0.16453926	0.16807277
Nigeria	0.37258355	0.38102604	0.38977965	0.39803771	0.40635331	0.41499775	0.42335191	0.43178038	0.44012466	0.44876682	0.45724099	0.46518378	0.47297287	0.48084441	0.48878736	0.49620474	0.50339083
São Tomé and Príncipe	0.36110588	0.36941301	0.37781352	0.38668894	0.39582124	0.40553067	0.41578373	0.42572131	0.43559246	0.44514624	0.45405147	0.46323008	0.47217093	0.4810982	0.48983823	0.49786141	0.50541375
Senegal	0.30325721	0.30763391	0.3125703	0.31789409	0.32365773	0.32980407	0.3360599	0.34297711	0.350134	0.35718157	0.3641445	0.37111453	0.37835148	0.38607967	0.39401232	0.40119752	0.40805419
Sierra Leone	0.23954994	0.24564789	0.25252308	0.25978156	0.26705838	0.27427027	0.28167357	0.29033851	0.30073669	0.31034887	0.31699865	0.32409156	0.33121343	0.33834156	0.34577002	0.35241202	0.35866588
Togo	0.31902558	0.32165939	0.3243825	0.32774314	0.33155854	0.33589958	0.34077726	0.3463205	0.3522786	0.35866144	0.36560017	0.37291003	0.38033623	0.38792123	0.39566727	0.40235692	0.4085337

Table S6. Mediation factor matrix			
**Distal Name**	**Mediator Name**	**Cause Name**	**Mediation Factor**
Smoking	High fasting plasma glucose	Lower extremity peripheral arterial disease	0.087 (0.061 to 0.121)
Smoking	High fasting plasma glucose	Diabetes mellitus type 2	1.000 (1.000 to 1.000)
High fasting plasma glucose	High LDL cholesterol	Ischemic heart disease	0.035 (0.024 to 0.049)
High fasting plasma glucose	High LDL cholesterol	Ischemic stroke	0.042 (0.028 to 0.062)
Diet low in fruits	High fasting plasma glucose	Ischemic stroke	0.050 (0.040 to 0.061)
Diet low in fruits	High fasting plasma glucose	Lower extremity peripheral arterial disease	1.000 (1.000 to 1.000)
Diet low in fruits	High fasting plasma glucose	Chronic kidney disease	0.151 (0.021 to 0.313)
Diet low in fruits	High fasting plasma glucose	Drug-susceptible tuberculosis	1.000 (1.000 to 1.000)
Diet low in fruits	High fasting plasma glucose	Multidrug-resistant tuberculosis without extensive drug resistance	1.000 (1.000 to 1.000)
Diet low in fruits	High fasting plasma glucose	Extensively drug-resistant tuberculosis	1.000 (1.000 to 1.000)
Diet low in fruits	High fasting plasma glucose	Diabetes mellitus type 2	1.000 (1.000 to 1.000)
Diet low in fruits	High systolic blood pressure	Ischemic heart disease	0.065 (0.052 to 0.080)
Diet low in fruits	High systolic blood pressure	Ischemic stroke	0.050 (0.040 to 0.061)
Diet low in fruits	High systolic blood pressure	Intracerebral hemorrhage	0.024 (0.019 to 0.029)
Diet low in fruits	High systolic blood pressure	Subarachnoid hemorrhage	0.024 (0.019 to 0.029)
Diet low in fruits	High systolic blood pressure	Hypertensive heart disease	1.000 (1.000 to 1.000)
Diet low in fruits	High systolic blood pressure	Aortic aneurysm	1.000 (1.000 to 1.000)
Diet low in fruits	High systolic blood pressure	Chronic kidney disease	0.849 (0.687 to 0.979)
Diet low in fruits	High LDL cholesterol	Ischemic heart disease	0.065 (0.052 to 0.080)
Diet low in fruits	High LDL cholesterol	Ischemic stroke	0.050 (0.040 to 0.061)
Diet low in vegetables	High fasting plasma glucose	Ischemic heart disease	0.063 (0.013 to 0.199)

Table S6. Mediation factor matrix			
**Distal Name**	**Mediator Name**	**Cause Name**	**Mediation Factor**
Diet low in vegetables	High fasting plasma glucose	Ischemic stroke	0.083 (0.039 to 0.160)
Diet low in vegetables	High fasting plasma glucose	Intracerebral hemorrhage	0.083 (0.039 to 0.160)
Diet low in vegetables	High fasting plasma glucose	Lower extremity peripheral arterial disease	1.000 (1.000 to 1.000)
Diet low in vegetables	High fasting plasma glucose	Chronic kidney disease	0.055 (-0.014 to 0.140)
Diet low in vegetables	High fasting plasma glucose	Drug-susceptible tuberculosis	1.000 (1.000 to 1.000)
Diet low in vegetables	High fasting plasma glucose	Multidrug-resistant tuberculosis without extensive drug resistance	1.000 (1.000 to 1.000)
Diet low in vegetables	High fasting plasma glucose	Extensively drug-resistant tuberculosis	1.000 (1.000 to 1.000)
Diet low in vegetables	High fasting plasma glucose	Diabetes mellitus type 2	1.000 (1.000 to 1.000)
Diet low in vegetables	High systolic blood pressure	Ischemic heart disease	0.041 (0.025 to 0.054)
Diet low in vegetables	High systolic blood pressure	Ischemic stroke	0.027 (0.016 to 0.038)
Diet low in vegetables	High systolic blood pressure	Intracerebral hemorrhage	0.036 (0.022 to 0.052)
Diet low in vegetables	High systolic blood pressure	Subarachnoid hemorrhage	0.036 (0.022 to 0.052)
Diet low in vegetables	High systolic blood pressure	Hypertensive heart disease	1.000 (1.000 to 1.000)
Diet low in vegetables	High systolic blood pressure	Aortic aneurysm	1.000 (1.000 to 1.000)
Diet low in vegetables	High systolic blood pressure	Chronic kidney disease	0.945 (0.860 to 1.014)
Diet low in vegetables	High LDL cholesterol	Ischemic heart disease	0.041 (0.025 to 0.054)
Diet low in vegetables	High LDL cholesterol	Ischemic stroke	0.087 (0.042 to 0.156)
Diet low in whole grains	High fasting plasma glucose	Lower extremity peripheral arterial disease	1.000 (1.000 to 1.000)
Diet low in whole grains	High fasting plasma glucose	Chronic kidney disease	1.000 (1.000 to 1.000)
Diet low in whole grains	High fasting plasma glucose	Drug-susceptible tuberculosis	1.000 (1.000 to 1.000)
Diet low in whole grains	High fasting plasma glucose	Multidrug-resistant tuberculosis without extensive drug resistance	1.000 (1.000 to 1.000)

Table S6. Mediation factor matrix			
**Distal Name**	**Mediator Name**	**Cause Name**	**Mediation Factor**
Diet low in whole grains	High fasting plasma glucose	Extensively drug-resistant tuberculosis	1.000<br>(1.000 to 1.000)
Diet low in whole grains	High fasting plasma glucose	Diabetes mellitus type 2	1.000<br>(1.000 to 1.000)
Diet low in whole grains	High LDL cholesterol	Ischemic heart disease	0.385<br>(0.169 to 0.540)
Diet low in whole grains	High LDL cholesterol	Ischemic stroke	0.165<br>(0.054 to 0.371)
Diet low in nuts and seeds	High LDL cholesterol	Ischemic heart disease	0.196<br>(0.011 to 0.759)
Diet low in milk	Diet low in calcium	Colon and rectum cancer	1.000<br>(1.000 to 1.000)
Diet high in red meat	High fasting plasma glucose	Lower extremity peripheral arterial disease	1.000<br>(1.000 to 1.000)
Diet high in red meat	High fasting plasma glucose	Chronic kidney disease	1.000<br>(1.000 to 1.000)
Diet high in red meat	High fasting plasma glucose	Drug-susceptible tuberculosis	1.000<br>(1.000 to 1.000)
Diet high in red meat	High fasting plasma glucose	Multidrug-resistant tuberculosis without extensive drug resistance	1.000<br>(1.000 to 1.000)
Diet high in red meat	High fasting plasma glucose	Extensively drug-resistant tuberculosis	1.000<br>(1.000 to 1.000)
Diet high in red meat	High fasting plasma glucose	Diabetes mellitus type 2	1.000<br>(1.000 to 1.000)
Diet high in processed meat	High fasting plasma glucose	Ischemic heart disease	0.010<br>(0.006 to 0.016)
Diet high in processed meat	High fasting plasma glucose	Ischemic stroke	1.000<br>(1.000 to 1.000)
Diet high in processed meat	High fasting plasma glucose	Lower extremity peripheral arterial disease	1.000<br>(1.000 to 1.000)
Diet high in processed meat	High fasting plasma glucose	Chronic kidney disease	1.000<br>(1.000 to 1.000)
Diet high in processed meat	High fasting plasma glucose	Drug-susceptible tuberculosis	1.000<br>(1.000 to 1.000)
Diet high in processed meat	High fasting plasma glucose	Multidrug-resistant tuberculosis without extensive drug resistance	1.000<br>(1.000 to 1.000)
Diet high in processed meat	High fasting plasma glucose	Extensively drug-resistant tuberculosis	1.000<br>(1.000 to 1.000)
Diet high in processed meat	High fasting plasma glucose	Diabetes mellitus type 2	1.000<br>(1.000 to 1.000)
Diet high in sugar-sweetened beverages	High fasting plasma glucose	Ischemic stroke	1.000<br>(1.000 to 1.000)

Table S6. Mediation factor matrix

Distal Name	Mediator Name	Cause Name	Mediation Factor
Diet high in sugar-sweetened beverages	High fasting plasma glucose	Lower extremity peripheral arterial disease	1.000 (1.000 to 1.000)
Diet high in sugar-sweetened beverages	High fasting plasma glucose	Chronic kidney disease	1.000 (1.000 to 1.000)
Diet high in sugar-sweetened beverages	High fasting plasma glucose	Drug-susceptible tuberculosis	1.000 (1.000 to 1.000)
Diet high in sugar-sweetened beverages	High fasting plasma glucose	Multidrug-resistant tuberculosis without extensive drug resistance	1.000 (1.000 to 1.000)
Diet high in sugar-sweetened beverages	High fasting plasma glucose	Extensively drug-resistant tuberculosis	1.000 (1.000 to 1.000)
Diet high in sugar-sweetened beverages	High fasting plasma glucose	Diabetes mellitus type 2	1.000 (1.000 to 1.000)
Diet low in polyunsaturated fatty acids	High LDL cholesterol	Ischemic heart disease	0.002 (-0.030 to 0.036)
Diet low in polyunsaturated fatty acids	High LDL cholesterol	Ischemic stroke	1.000 (1.000 to 1.000)
Diet high in trans fatty acids	High LDL cholesterol	Ischemic heart disease	0.150 (0.024 to 0.240)
Diet high in sodium	High systolic blood pressure	Ischemic heart disease	1.000 (1.000 to 1.000)
Diet high in sodium	High systolic blood pressure	Ischemic stroke	1.000 (1.000 to 1.000)
Diet high in sodium	High systolic blood pressure	Intracerebral hemorrhage	1.000 (1.000 to 1.000)
Diet high in sodium	High systolic blood pressure	Subarachnoid hemorrhage	1.000 (1.000 to 1.000)
Diet high in sodium	High systolic blood pressure	Hypertensive heart disease	1.000 (1.000 to 1.000)
Diet high in sodium	High systolic blood pressure	Atrial fibrillation and flutter	1.000 (1.000 to 1.000)
Diet high in sodium	High systolic blood pressure	Aortic aneurysm	1.000 (1.000 to 1.000)
Diet high in sodium	High systolic blood pressure	Lower extremity peripheral arterial disease	1.000 (1.000 to 1.000)
Diet high in sodium	High systolic blood pressure	Chronic kidney disease	1.000 (1.000 to 1.000)
Low physical activity	High fasting plasma glucose	Ischemic heart disease	0.144 (0.115 to 0.176)
Low physical activity	High fasting plasma glucose	Ischemic stroke	0.078 (0.032 to 0.138)
Low physical activity	High fasting plasma glucose	Lower extremity peripheral arterial disease	1.000 (1.000 to 1.000)

**Table S6. Mediation factor matrix**			
**Distal Name**	**Mediator Name**	**Cause Name**	**Mediation Factor**
Low physical activity	High fasting plasma glucose	Chronic kidney disease	1.000 (1.000 to 1.000)
Low physical activity	High fasting plasma glucose	Drug-susceptible tuberculosis	1.000 (1.000 to 1.000)
Low physical activity	High fasting plasma glucose	Multidrug-resistant tuberculosis without extensive drug resistance	1.000 (1.000 to 1.000)
Low physical activity	High fasting plasma glucose	Extensively drug-resistant tuberculosis	1.000 (1.000 to 1.000)
Low physical activity	High fasting plasma glucose	Diabetes mellitus type 2	1.000 (1.000 to 1.000)
Lead exposure in bone	High systolic blood pressure	Ischemic heart disease	1.000 (1.000 to 1.000)
Lead exposure in bone	High systolic blood pressure	Ischemic stroke	1.000 (1.000 to 1.000)
Lead exposure in bone	High systolic blood pressure	Intracerebral hemorrhage	1.000 (1.000 to 1.000)
Lead exposure in bone	High systolic blood pressure	Subarachnoid hemorrhage	1.000 (1.000 to 1.000)
Lead exposure in bone	High systolic blood pressure	Hypertensive heart disease	1.000 (1.000 to 1.000)
Lead exposure in bone	High systolic blood pressure	Atrial fibrillation and flutter	1.000 (1.000 to 1.000)
Lead exposure in bone	High systolic blood pressure	Aortic aneurysm	1.000 (1.000 to 1.000)
Lead exposure in bone	High systolic blood pressure	Lower extremity peripheral arterial disease	1.000 (1.000 to 1.000)
Lead exposure in bone	High systolic blood pressure	Chronic kidney disease	1.000 (1.000 to 1.000)
Childhood sexual abuse against females	High alcohol use	Alcohol use disorders	1.000 (1.000 to 1.000)
Childhood sexual abuse against males	High alcohol use	Alcohol use disorders	1.000 (1.000 to 1.000)
High body-mass index in adults	High fasting plasma glucose	Ischemic heart disease	0.149 (0.098 to 0.204)
High body-mass index in adults	High fasting plasma glucose	Ischemic stroke	0.217 (0.122 to 0.306)
High body-mass index in adults	High fasting plasma glucose	Intracerebral hemorrhage	0.222 (0.125 to 0.325)
High body-mass index in adults	High fasting plasma glucose	Lower extremity peripheral arterial disease	1.000 (1.000 to 1.000)
High body-mass index in adults	High fasting plasma glucose	Chronic kidney disease	0.496 (0.389 to 0.612)

Table S6. Mediation factor matrix			
**Distal Name**	**Mediator Name**	**Cause Name**	**Mediation Factor**
High body-mass index in adults	High fasting plasma glucose	Drug-susceptible tuberculosis	1.000 (1.000 to 1.000)
High body-mass index in adults	High fasting plasma glucose	Multidrug-resistant tuberculosis without extensive drug resistance	1.000 (1.000 to 1.000)
High body-mass index in adults	High fasting plasma glucose	Extensively drug-resistant tuberculosis	1.000 (1.000 to 1.000)
High body-mass index in adults	High fasting plasma glucose	Diabetes mellitus type 2	1.000 (1.000 to 1.000)
High body-mass index in adults	High systolic blood pressure	Ischemic heart disease	0.312 (0.281 to 0.344)
High body-mass index in adults	High systolic blood pressure	Ischemic stroke	0.647 (0.566 to 0.724)
High body-mass index in adults	High systolic blood pressure	Intracerebral hemorrhage	0.651 (0.575 to 0.727)
High body-mass index in adults	High systolic blood pressure	Subarachnoid hemorrhage	0.651 (0.575 to 0.727)
High body-mass index in adults	High systolic blood pressure	Hypertensive heart disease	1.000 (1.000 to 1.000)
High body-mass index in adults	High systolic blood pressure	Atrial fibrillation and flutter	0.310 (0.281 to 0.339)
High body-mass index in adults	High systolic blood pressure	Aortic aneurysm	1.000 (1.000 to 1.000)
High body-mass index in adults	High systolic blood pressure	Chronic kidney disease	0.504 (0.388 to 0.611)
High body-mass index in adults	High LDL cholesterol	Ischemic heart disease	0.100 (0.053 to 0.149)
High body-mass index in adults	High LDL cholesterol	Ischemic stroke	0.034 (0.002 to 0.078)

Table S7. Status of risk–outcome pairs considered for inclusion in GBD 2021: included in both GBD 2019 and GBD 2021, added in GBD 2021, or removed in GBD 2021

Risk Name	Outcome Name	Status
Unsafe water source	Diarrheal diseases	Used in GBD 2019 and GBD 2021
Unsafe sanitation	Diarrheal diseases	Used in GBD 2019 and GBD 2021
No access to handwashing facility	Lower respiratory infections	Used in GBD 2019 and GBD 2021
No access to handwashing facility	Diarrheal diseases	Used in GBD 2019 and GBD 2021
Ambient particulate matter pollution	Lower respiratory infections	Used in GBD 2019 and GBD 2021
Ambient particulate matter pollution	Upper respiratory infections	Used in GBD 2019 and GBD 2021
Ambient particulate matter pollution	Otitis media	Used in GBD 2019 and GBD 2021
Ambient particulate matter pollution	Diarrheal diseases	Used in GBD 2019 and GBD 2021
Ambient particulate matter pollution	Meningitis	Used in GBD 2019 and GBD 2021
Ambient particulate matter pollution	Encephalitis	Used in GBD 2019 and GBD 2021
Ambient particulate matter pollution	Neonatal preterm birth	Used in GBD 2019 and GBD 2021
Ambient particulate matter pollution	Neonatal encephalopathy due to birth asphyxia and trauma	Used in GBD 2019 and GBD 2021
Ambient particulate matter pollution	Neonatal sepsis and other neonatal infections	Used in GBD 2019 and GBD 2021
Ambient particulate matter pollution	Hemolytic disease and other neonatal jaundice	Used in GBD 2019 and GBD 2021
Ambient particulate matter pollution	Other neonatal disorders	Used in GBD 2019 and GBD 2021
Ambient particulate matter pollution	Tracheal, bronchus, and lung cancer	Used in GBD 2019 and GBD 2021
Ambient particulate matter pollution	Ischemic heart disease	Used in GBD 2019 and GBD 2021
Ambient particulate matter pollution	Ischemic stroke	Used in GBD 2019 and GBD 2021
Ambient particulate matter pollution	Intracerebral hemorrhage	Used in GBD 2019 and GBD 2021
Ambient particulate matter pollution	Subarachnoid hemorrhage	Used in GBD 2019 and GBD 2021
Ambient particulate matter pollution	Chronic obstructive pulmonary disease	Used in GBD 2019 and GBD 2021
Ambient particulate matter pollution	Diabetes mellitus type 2	Used in GBD 2019 and GBD 2021
Ambient particulate matter pollution	Sudden infant death syndrome	Used in GBD 2019 and GBD 2021
Household air pollution from solid fuels	Lower respiratory infections	Used in GBD 2019 and GBD 2021
Household air pollution from solid fuels	Upper respiratory infections	Used in GBD 2019 and GBD 2021
Household air pollution from solid fuels	Otitis media	Used in GBD 2019 and GBD 2021
Household air pollution from solid fuels	Diarrheal diseases	Used in GBD 2019 and GBD 2021
Household air pollution from solid fuels	Meningitis	Used in GBD 2019 and GBD 2021
Household air pollution from solid fuels	Encephalitis	Used in GBD 2019 and GBD 2021
Household air pollution from solid fuels	Neonatal preterm birth	Used in GBD 2019 and GBD 2021
Household air pollution from solid fuels	Neonatal encephalopathy due to birth asphyxia and trauma	Used in GBD 2019 and GBD 2021

Table S7. Status of risk–outcome pairs considered for inclusion in GBD 2021: included in both GBD 2019 and GBD 2021, added in GBD 2021, or removed in GBD 2021

Risk Name	Outcome Name	Status
Household air pollution from solid fuels	Neonatal sepsis and other neonatal infections	Used in GBD 2019 and GBD 2021
Household air pollution from solid fuels	Hemolytic disease and other neonatal jaundice	Used in GBD 2019 and GBD 2021
Household air pollution from solid fuels	Other neonatal disorders	Used in GBD 2019 and GBD 2021
Household air pollution from solid fuels	Tracheal, bronchus, and lung cancer	Used in GBD 2019 and GBD 2021
Household air pollution from solid fuels	Ischemic heart disease	Used in GBD 2019 and GBD 2021
Household air pollution from solid fuels	Ischemic stroke	Used in GBD 2019 and GBD 2021
Household air pollution from solid fuels	Intracerebral hemorrhage	Used in GBD 2019 and GBD 2021
Household air pollution from solid fuels	Subarachnoid hemorrhage	Used in GBD 2019 and GBD 2021
Household air pollution from solid fuels	Chronic obstructive pulmonary disease	Used in GBD 2019 and GBD 2021
Household air pollution from solid fuels	Diabetes mellitus type 2	Used in GBD 2019 and GBD 2021
Household air pollution from solid fuels	Cataract	Used in GBD 2019 and GBD 2021
Household air pollution from solid fuels	Sudden infant death syndrome	Used in GBD 2019 and GBD 2021
Ambient ozone pollution	Chronic obstructive pulmonary disease	Used in GBD 2019 and GBD 2021
High temperature	Lower respiratory infections	Used in GBD 2019 and GBD 2021
High temperature	Ischemic heart disease	Used in GBD 2019 and GBD 2021
High temperature	Ischemic stroke	Used in GBD 2019 and GBD 2021
High temperature	Intracerebral hemorrhage	Used in GBD 2019 and GBD 2021
High temperature	Subarachnoid hemorrhage	Used in GBD 2019 and GBD 2021
High temperature	Hypertensive heart disease	Used in GBD 2019 and GBD 2021
High temperature	Chronic obstructive pulmonary disease	Used in GBD 2019 and GBD 2021
High temperature	Diabetes mellitus type 1	Used in GBD 2019 and GBD 2021
High temperature	Diabetes mellitus type 2	Used in GBD 2019 and GBD 2021
High temperature	Chronic kidney disease due to diabetes mellitus type 1	Used in GBD 2019 and GBD 2021
High temperature	Chronic kidney disease due to diabetes mellitus type 2	Used in GBD 2019 and GBD 2021
High temperature	Chronic kidney disease due to hypertension	Used in GBD 2019 and GBD 2021
High temperature	Chronic kidney disease due to glomerulonephritis	Used in GBD 2019 and GBD 2021
High temperature	Chronic kidney disease due to other and unspecified causes	Used in GBD 2019 and GBD 2021
High temperature	Pedestrian road injuries	Used in GBD 2019 and GBD 2021
High temperature	Cyclist road injuries	Used in GBD 2019 and GBD 2021
High temperature	Motorcyclist road injuries	Used in GBD 2019 and GBD 2021
High temperature	Motor vehicle road injuries	Used in GBD 2019 and GBD 2021

Table S7. Status of risk–outcome pairs considered for inclusion in GBD 2021: included in both GBD 2019 and GBD 2021, added in GBD 2021, or removed in GBD 2021

Risk Name	Outcome Name	Status
High temperature	Other road injuries	Used in GBD 2019 and GBD 2021
High temperature	Drowning	Used in GBD 2019 and GBD 2021
High temperature	Unintentional firearm injuries	Used in GBD 2019 and GBD 2021
High temperature	Other exposure to mechanical forces	Used in GBD 2019 and GBD 2021
High temperature	Self-harm by firearm	Used in GBD 2019 and GBD 2021
High temperature	Self-harm by other specified means	Used in GBD 2019 and GBD 2021
High temperature	Physical violence by firearm	Used in GBD 2019 and GBD 2021
High temperature	Physical violence by sharp object	Used in GBD 2019 and GBD 2021
High temperature	Physical violence by other means	Used in GBD 2019 and GBD 2021
Low temperature	Lower respiratory infections	Used in GBD 2019 and GBD 2021
Low temperature	Ischemic heart disease	Used in GBD 2019 and GBD 2021
Low temperature	Ischemic stroke	Used in GBD 2019 and GBD 2021
Low temperature	Intracerebral hemorrhage	Used in GBD 2019 and GBD 2021
Low temperature	Subarachnoid hemorrhage	Used in GBD 2019 and GBD 2021
Low temperature	Hypertensive heart disease	Used in GBD 2019 and GBD 2021
Low temperature	Chronic obstructive pulmonary disease	Used in GBD 2019 and GBD 2021
Low temperature	Diabetes mellitus type 1	Used in GBD 2019 and GBD 2021
Low temperature	Diabetes mellitus type 2	Used in GBD 2019 and GBD 2021
Low temperature	Chronic kidney disease due to diabetes mellitus type 1	Used in GBD 2019 and GBD 2021
Low temperature	Chronic kidney disease due to diabetes mellitus type 2	Used in GBD 2019 and GBD 2021
Low temperature	Chronic kidney disease due to hypertension	Used in GBD 2019 and GBD 2021
Low temperature	Chronic kidney disease due to glomerulonephritis	Used in GBD 2019 and GBD 2021
Low temperature	Chronic kidney disease due to other and unspecified causes	Used in GBD 2019 and GBD 2021
Low temperature	Pedestrian road injuries	Used in GBD 2019 and GBD 2021
Low temperature	Cyclist road injuries	Used in GBD 2019 and GBD 2021
Low temperature	Motorcyclist road injuries	Used in GBD 2019 and GBD 2021
Low temperature	Motor vehicle road injuries	Used in GBD 2019 and GBD 2021
Low temperature	Other road injuries	Used in GBD 2019 and GBD 2021
Low temperature	Drowning	Used in GBD 2019 and GBD 2021
Low temperature	Unintentional firearm injuries	Used in GBD 2019 and GBD 2021
Low temperature	Other exposure to mechanical forces	Used in GBD 2019 and GBD 2021

Table S7. Status of risk–outcome pairs considered for inclusion in GBD 2021: included in both GBD 2019 and GBD 2021, added in GBD 2021, or removed in GBD 2021		
**Risk Name**	**Outcome Name**	**Status**
Low temperature	Self-harm by firearm	Used in GBD 2019 and GBD 2021
Low temperature	Self-harm by other specified means	Used in GBD 2019 and GBD 2021
Low temperature	Physical violence by firearm	Used in GBD 2019 and GBD 2021
Low temperature	Physical violence by sharp object	Used in GBD 2019 and GBD 2021
Low temperature	Physical violence by other means	Used in GBD 2019 and GBD 2021
Residential radon	Tracheal, bronchus, and lung cancer	Used in GBD 2019 and GBD 2021
Lead exposure in blood	Idiopathic developmental intellectual disability	Used in GBD 2019 and GBD 2021
Lead exposure in bone	Ischemic heart disease	Used in GBD 2019 and GBD 2021
Lead exposure in bone	Ischemic stroke	Used in GBD 2019 and GBD 2021
Lead exposure in bone	Intracerebral hemorrhage	Used in GBD 2019 and GBD 2021
Lead exposure in bone	Subarachnoid hemorrhage	Used in GBD 2019 and GBD 2021
Lead exposure in bone	Hypertensive heart disease	Used in GBD 2019 and GBD 2021
Lead exposure in bone	Atrial fibrillation and flutter	Used in GBD 2019 and GBD 2021
Lead exposure in bone	Aortic aneurysm	Used in GBD 2019 and GBD 2021
Lead exposure in bone	Peripheral artery disease	Used in GBD 2019 and GBD 2021
Lead exposure in bone	Chronic kidney disease due to diabetes mellitus type 2	Used in GBD 2019 and GBD 2021
Lead exposure in bone	Chronic kidney disease due to hypertension	Used in GBD 2019 and GBD 2021
Lead exposure in bone	Chronic kidney disease due to glomerulonephritis	Used in GBD 2019 and GBD 2021
Lead exposure in bone	Chronic kidney disease due to other and unspecified causes	Used in GBD 2019 and GBD 2021
Occupational exposure to asbestos	Larynx cancer	Used in GBD 2019 and GBD 2021
Occupational exposure to asbestos	Tracheal, bronchus, and lung cancer	Used in GBD 2019 and GBD 2021
Occupational exposure to asbestos	Ovarian cancer	Used in GBD 2019 and GBD 2021
Occupational exposure to asbestos	Mesothelioma	Used in GBD 2019 and GBD 2021
Occupational exposure to asbestos	Asbestosis	Used in GBD 2019 and GBD 2021
Occupational exposure to arsenic	Tracheal, bronchus, and lung cancer	Used in GBD 2019 and GBD 2021
Occupational exposure to benzene	Acute lymphoid leukemia	Used in GBD 2019 and GBD 2021
Occupational exposure to benzene	Chronic lymphoid leukemia	Used in GBD 2019 and GBD 2021
Occupational exposure to benzene	Acute myeloid leukemia	Used in GBD 2019 and GBD 2021
Occupational exposure to benzene	Chronic myeloid leukemia	Used in GBD 2019 and GBD 2021
Occupational exposure to benzene	Other leukemia	Used in GBD 2019 and GBD 2021
Occupational exposure to beryllium	Tracheal, bronchus, and lung cancer	Used in GBD 2019 and GBD 2021

Table S7. Status of risk–outcome pairs considered for inclusion in GBD 2021: included in both GBD 2019 and GBD 2021, added in GBD 2021, or removed in GBD 2021		
**Risk Name**	**Outcome Name**	**Status**
Occupational exposure to cadmium	Tracheal, bronchus, and lung cancer	Used in GBD 2019 and GBD 2021
Occupational exposure to chromium	Tracheal, bronchus, and lung cancer	Used in GBD 2019 and GBD 2021
Occupational exposure to diesel engine exhaust	Tracheal, bronchus, and lung cancer	Used in GBD 2019 and GBD 2021
Occupational exposure to formaldehyde	Nasopharynx cancer	Used in GBD 2019 and GBD 2021
Occupational exposure to formaldehyde	Acute lymphoid leukemia	Used in GBD 2019 and GBD 2021
Occupational exposure to formaldehyde	Chronic lymphoid leukemia	Used in GBD 2019 and GBD 2021
Occupational exposure to formaldehyde	Acute myeloid leukemia	Used in GBD 2019 and GBD 2021
Occupational exposure to formaldehyde	Chronic myeloid leukemia	Used in GBD 2019 and GBD 2021
Occupational exposure to formaldehyde	Other leukemia	Used in GBD 2019 and GBD 2021
Occupational exposure to nickel	Tracheal, bronchus, and lung cancer	Used in GBD 2019 and GBD 2021
Occupational exposure to polycyclic aromatic hydrocarbons	Tracheal, bronchus, and lung cancer	Used in GBD 2019 and GBD 2021
Occupational exposure to silica	Tracheal, bronchus, and lung cancer	Used in GBD 2019 and GBD 2021
Occupational exposure to silica	Silicosis	Used in GBD 2019 and GBD 2021
Occupational exposure to sulfuric acid	Larynx cancer	Used in GBD 2019 and GBD 2021
Occupational exposure to trichloroethylene	Kidney cancer	Used in GBD 2019 and GBD 2021
Occupational asthmagens	Asthma	Used in GBD 2019 and GBD 2021
Occupational particulate matter, gases, and fumes	Chronic obstructive pulmonary disease	Used in GBD 2019 and GBD 2021
Occupational particulate matter, gases, and fumes	Coal workers pneumoconiosis	Used in GBD 2019 and GBD 2021
Occupational particulate matter, gases, and fumes	Other pneumoconiosis	Used in GBD 2019 and GBD 2021
Occupational noise	Age-related and other hearing loss	Used in GBD 2019 and GBD 2021
Occupational injuries	Pedestrian road injuries	Used in GBD 2019 and GBD 2021
Occupational injuries	Cyclist road injuries	Used in GBD 2019 and GBD 2021
Occupational injuries	Motorcyclist road injuries	Used in GBD 2019 and GBD 2021
Occupational injuries	Motor vehicle road injuries	Used in GBD 2019 and GBD 2021
Occupational injuries	Other road injuries	Used in GBD 2019 and GBD 2021
Occupational injuries	Other transport injuries	Used in GBD 2019 and GBD 2021
Occupational injuries	Falls	Used in GBD 2019 and GBD 2021
Occupational injuries	Drowning	Used in GBD 2019 and GBD 2021
Occupational injuries	Fire, heat, and hot substances	Used in GBD 2019 and GBD 2021
Occupational injuries	Poisoning by carbon monoxide	Used in GBD 2019 and GBD 2021
Occupational injuries	Poisoning by other means	Used in GBD 2019 and GBD 2021

Table S7. Status of risk–outcome pairs considered for inclusion in GBD 2021: included in both GBD 2019 and GBD 2021, added in GBD 2021, or removed in GBD 2021

Risk Name	Outcome Name	Status
Occupational injuries	Unintentional firearm injuries	Used in GBD 2019 and GBD 2021
Occupational injuries	Other exposure to mechanical forces	Used in GBD 2019 and GBD 2021
Occupational injuries	Venomous animal contact	Used in GBD 2019 and GBD 2021
Occupational injuries	Non-venomous animal contact	Used in GBD 2019 and GBD 2021
Occupational injuries	Pulmonary aspiration and foreign body in airway	Used in GBD 2019 and GBD 2021
Occupational injuries	Foreign body in other body part	Used in GBD 2019 and GBD 2021
Occupational injuries	Other unintentional injuries	Used in GBD 2019 and GBD 2021
Occupational ergonomic factors	Low back pain	Used in GBD 2019 and GBD 2021
Non-exclusive breastfeeding	Lower respiratory infections	Used in GBD 2019 and GBD 2021
Non-exclusive breastfeeding	Diarrheal diseases	Used in GBD 2019 and GBD 2021
Discontinued breastfeeding	Diarrheal diseases	Used in GBD 2019 and GBD 2021
Child underweight	Lower respiratory infections	Used in GBD 2019 and GBD 2021
Child underweight	Diarrheal diseases	Used in GBD 2019 and GBD 2021
Child underweight	Measles	Used in GBD 2019 and GBD 2021
Child underweight	Protein-energy malnutrition	Used in GBD 2019 and GBD 2021
Child wasting	Lower respiratory infections	Used in GBD 2019 and GBD 2021
Child wasting	Diarrheal diseases	Used in GBD 2019 and GBD 2021
Child wasting	Measles	Used in GBD 2019 and GBD 2021
Child wasting	Protein-energy malnutrition	Used in GBD 2019 and GBD 2021
Child stunting	Lower respiratory infections	Used in GBD 2019 and GBD 2021
Child stunting	Diarrheal diseases	Used in GBD 2019 and GBD 2021
Child stunting	Measles	Used in GBD 2019 and GBD 2021
Short gestation	Lower respiratory infections	Used in GBD 2019 and GBD 2021
Short gestation	Upper respiratory infections	Used in GBD 2019 and GBD 2021
Short gestation	Otitis media	Used in GBD 2019 and GBD 2021
Short gestation	Diarrheal diseases	Used in GBD 2019 and GBD 2021
Short gestation	Meningitis	Used in GBD 2019 and GBD 2021
Short gestation	Encephalitis	Used in GBD 2019 and GBD 2021
Short gestation	Neonatal preterm birth	Used in GBD 2019 and GBD 2021
Short gestation	Neonatal encephalopathy due to birth asphyxia and trauma	Used in GBD 2019 and GBD 2021
Short gestation	Neonatal sepsis and other neonatal infections	Used in GBD 2019 and GBD 2021

Table S7. Status of risk–outcome pairs considered for inclusion in GBD 2021: included in both GBD 2019 and GBD 2021, added in GBD 2021, or removed in GBD 2021		
**Risk Name**	**Outcome Name**	**Status**
Short gestation	Hemolytic disease and other neonatal jaundice	Used in GBD 2019 and GBD 2021
Short gestation	Other neonatal disorders	Used in GBD 2019 and GBD 2021
Short gestation	Sudden infant death syndrome	Used in GBD 2019 and GBD 2021
Low birth weight	Lower respiratory infections	Used in GBD 2019 and GBD 2021
Low birth weight	Upper respiratory infections	Used in GBD 2019 and GBD 2021
Low birth weight	Otitis media	Used in GBD 2019 and GBD 2021
Low birth weight	Diarrheal diseases	Used in GBD 2019 and GBD 2021
Low birth weight	Meningitis	Used in GBD 2019 and GBD 2021
Low birth weight	Encephalitis	Used in GBD 2019 and GBD 2021
Low birth weight	Neonatal preterm birth	Used in GBD 2019 and GBD 2021
Low birth weight	Neonatal encephalopathy due to birth asphyxia and trauma	Used in GBD 2019 and GBD 2021
Low birth weight	Neonatal sepsis and other neonatal infections	Used in GBD 2019 and GBD 2021
Low birth weight	Hemolytic disease and other neonatal jaundice	Used in GBD 2019 and GBD 2021
Low birth weight	Other neonatal disorders	Used in GBD 2019 and GBD 2021
Low birth weight	Sudden infant death syndrome	Used in GBD 2019 and GBD 2021
Iron deficiency	Maternal hemorrhage	Used in GBD 2019 and GBD 2021
Iron deficiency	Maternal sepsis and other maternal infections	Used in GBD 2019 and GBD 2021
Iron deficiency	Maternal hypertensive disorders	Used in GBD 2019 and GBD 2021
Iron deficiency	Maternal obstructed labor and uterine rupture	Used in GBD 2019 and GBD 2021
Iron deficiency	Maternal abortion and miscarriage	Used in GBD 2019 and GBD 2021
Iron deficiency	Ectopic pregnancy	Used in GBD 2019 and GBD 2021
Iron deficiency	Indirect maternal deaths	Used in GBD 2019 and GBD 2021
Iron deficiency	Late maternal deaths	Used in GBD 2019 and GBD 2021
Iron deficiency	Maternal deaths aggravated by HIV/AIDS	Used in GBD 2019 and GBD 2021
Iron deficiency	Other maternal disorders	Used in GBD 2019 and GBD 2021
Iron deficiency	Dietary iron deficiency	Used in GBD 2019 and GBD 2021
Vitamin A deficiency	Diarrheal diseases	Used in GBD 2019 and GBD 2021
Vitamin A deficiency	Measles	Used in GBD 2019 and GBD 2021
Vitamin A deficiency	Vitamin A deficiency	Used in GBD 2019 and GBD 2021
Zinc deficiency	Diarrheal diseases	Used in GBD 2019 and GBD 2021
Smoking	Latent tuberculosis infection	Used in GBD 2019 and GBD 2021

Table S7. Status of risk–outcome pairs considered for inclusion in GBD 2021: included in both GBD 2019 and GBD 2021, added in GBD 2021, or removed in GBD 2021

Risk Name	Outcome Name	Status
Smoking	Drug-susceptible tuberculosis	Used in GBD 2019 and GBD 2021
Smoking	Multidrug-resistant tuberculosis without extensive drug resistance	Used in GBD 2019 and GBD 2021
Smoking	Extensively drug-resistant tuberculosis	Used in GBD 2019 and GBD 2021
Smoking	Lower respiratory infections	Used in GBD 2019 and GBD 2021
Smoking	Lip and oral cavity cancer	Used in GBD 2019 and GBD 2021
Smoking	Nasopharynx cancer	Used in GBD 2019 and GBD 2021
Smoking	Other pharynx cancer	Used in GBD 2019 and GBD 2021
Smoking	Esophageal cancer	Used in GBD 2019 and GBD 2021
Smoking	Stomach cancer	Used in GBD 2019 and GBD 2021
Smoking	Colon and rectum cancer	Used in GBD 2019 and GBD 2021
Smoking	Liver cancer due to hepatitis B	Used in GBD 2019 and GBD 2021
Smoking	Liver cancer due to hepatitis C	Used in GBD 2019 and GBD 2021
Smoking	Liver cancer due to alcohol use	Used in GBD 2019 and GBD 2021
Smoking	Liver cancer due to NASH	Used in GBD 2019 and GBD 2021
Smoking	Liver cancer due to other causes (internal)	Used in GBD 2019 and GBD 2021
Smoking	Pancreatic cancer	Used in GBD 2019 and GBD 2021
Smoking	Larynx cancer	Used in GBD 2019 and GBD 2021
Smoking	Tracheal, bronchus, and lung cancer	Used in GBD 2019 and GBD 2021
Smoking	Breast cancer	Used in GBD 2019 and GBD 2021
Smoking	Cervical cancer	Used in GBD 2019 and GBD 2021
Smoking	Prostate cancer	Used in GBD 2019 and GBD 2021
Smoking	Kidney cancer	Used in GBD 2019 and GBD 2021
Smoking	Bladder cancer	Used in GBD 2019 and GBD 2021
Smoking	Acute lymphoid leukemia	Used in GBD 2019 and GBD 2021
Smoking	Chronic lymphoid leukemia	Used in GBD 2019 and GBD 2021
Smoking	Acute myeloid leukemia	Used in GBD 2019 and GBD 2021
Smoking	Chronic myeloid leukemia	Used in GBD 2019 and GBD 2021
Smoking	Other leukemia	Used in GBD 2019 and GBD 2021
Smoking	Ischemic heart disease	Used in GBD 2019 and GBD 2021
Smoking	Ischemic stroke	Used in GBD 2019 and GBD 2021
Smoking	Intracerebral hemorrhage	Used in GBD 2019 and GBD 2021

Table S7. Status of risk–outcome pairs considered for inclusion in GBD 2021: included in both GBD 2019 and GBD 2021, added in GBD 2021, or removed in GBD 2021

Risk Name	Outcome Name	Status
Smoking	Subarachnoid hemorrhage	Used in GBD 2019 and GBD 2021
Smoking	Atrial fibrillation and flutter	Used in GBD 2019 and GBD 2021
Smoking	Aortic aneurysm	Used in GBD 2019 and GBD 2021
Smoking	Peripheral artery disease	Used in GBD 2019 and GBD 2021
Smoking	Chronic obstructive pulmonary disease	Used in GBD 2019 and GBD 2021
Smoking	Asthma	Used in GBD 2019 and GBD 2021
Smoking	Peptic ulcer disease	Used in GBD 2019 and GBD 2021
Smoking	Gallbladder and biliary diseases	Used in GBD 2019 and GBD 2021
Smoking	Alzheimer's disease and other dementias	Used in GBD 2019 and GBD 2021
Smoking	Parkinson's disease	Used in GBD 2019 and GBD 2021
Smoking	Multiple sclerosis	Used in GBD 2019 and GBD 2021
Smoking	Diabetes mellitus type 2	Used in GBD 2019 and GBD 2021
Smoking	Cataract	Used in GBD 2019 and GBD 2021
Smoking	Age-related macular degeneration	Used in GBD 2019 and GBD 2021
Smoking	Rheumatoid arthritis	Used in GBD 2019 and GBD 2021
Smoking	Low back pain	Used in GBD 2019 and GBD 2021
Smoking	Pedestrian road injuries	Used in GBD 2019 and GBD 2021
Smoking	Cyclist road injuries	Used in GBD 2019 and GBD 2021
Smoking	Motorcyclist road injuries	Used in GBD 2019 and GBD 2021
Smoking	Motor vehicle road injuries	Used in GBD 2019 and GBD 2021
Smoking	Other road injuries	Used in GBD 2019 and GBD 2021
Smoking	Other transport injuries	Used in GBD 2019 and GBD 2021
Smoking	Falls	Used in GBD 2019 and GBD 2021
Smoking	Other exposure to mechanical forces	Used in GBD 2019 and GBD 2021
Smoking	Non-venomous animal contact	Used in GBD 2019 and GBD 2021
Smoking	Physical violence by other means	Used in GBD 2019 and GBD 2021
Chewing tobacco	Lip and oral cavity cancer	Used in GBD 2019 and GBD 2021
Chewing tobacco	Esophageal cancer	Used in GBD 2019 and GBD 2021
Secondhand smoke	Lower respiratory infections	Used in GBD 2019 and GBD 2021
Secondhand smoke	Otitis media	Used in GBD 2019 and GBD 2021
Secondhand smoke	Tracheal, bronchus, and lung cancer	Used in GBD 2019 and GBD 2021

Table S7. Status of risk–outcome pairs considered for inclusion in GBD 2021: included in both GBD 2019 and GBD 2021, added in GBD 2021, or removed in GBD 2021

Risk Name	Outcome Name	Status
Secondhand smoke	Breast cancer	Used in GBD 2019 and GBD 2021
Secondhand smoke	Ischemic heart disease	Used in GBD 2019 and GBD 2021
Secondhand smoke	Ischemic stroke	Used in GBD 2019 and GBD 2021
Secondhand smoke	Intracerebral hemorrhage	Used in GBD 2019 and GBD 2021
Secondhand smoke	Subarachnoid hemorrhage	Used in GBD 2019 and GBD 2021
Secondhand smoke	Chronic obstructive pulmonary disease	Used in GBD 2019 and GBD 2021
Secondhand smoke	Diabetes mellitus type 2	Used in GBD 2019 and GBD 2021
Alcohol use	Latent tuberculosis infection	Used in GBD 2019 and GBD 2021
Alcohol use	Drug-susceptible tuberculosis	Used in GBD 2019 and GBD 2021
Alcohol use	Multidrug-resistant tuberculosis without extensive drug resistance	Used in GBD 2019 and GBD 2021
Alcohol use	Extensively drug-resistant tuberculosis	Used in GBD 2019 and GBD 2021
Alcohol use	Lip and oral cavity cancer	Used in GBD 2019 and GBD 2021
Alcohol use	Nasopharynx cancer	Used in GBD 2019 and GBD 2021
Alcohol use	Other pharynx cancer	Used in GBD 2019 and GBD 2021
Alcohol use	Esophageal cancer	Used in GBD 2019 and GBD 2021
Alcohol use	Colon and rectum cancer	Used in GBD 2019 and GBD 2021
Alcohol use	Liver cancer due to hepatitis B	Used in GBD 2019 and GBD 2021
Alcohol use	Liver cancer due to hepatitis C	Used in GBD 2019 and GBD 2021
Alcohol use	Liver cancer due to alcohol use	Used in GBD 2019 and GBD 2021
Alcohol use	Liver cancer due to NASH	Used in GBD 2019 and GBD 2021
Alcohol use	Larynx cancer	Used in GBD 2019 and GBD 2021
Alcohol use	Breast cancer	Used in GBD 2019 and GBD 2021
Alcohol use	Ischemic heart disease	Used in GBD 2019 and GBD 2021
Alcohol use	Ischemic stroke	Used in GBD 2019 and GBD 2021
Alcohol use	Intracerebral hemorrhage	Used in GBD 2019 and GBD 2021
Alcohol use	Hypertensive heart disease	Used in GBD 2019 and GBD 2021
Alcohol use	Alcoholic cardiomyopathy	Used in GBD 2019 and GBD 2021
Alcohol use	Atrial fibrillation and flutter	Used in GBD 2019 and GBD 2021
Alcohol use	Cirrhosis and other chronic liver diseases due to hepatitis B	Used in GBD 2019 and GBD 2021
Alcohol use	Cirrhosis and other chronic liver diseases due to hepatitis C	Used in GBD 2019 and GBD 2021
Alcohol use	Cirrhosis and other chronic liver diseases due to alcohol use	Used in GBD 2019 and GBD 2021

Table S7. Status of risk–outcome pairs considered for inclusion in GBD 2021: included in both GBD 2019 and GBD 2021, added in GBD 2021, or removed in GBD 2021

Risk Name	Outcome Name	Status
Alcohol use	Cirrhosis and other chronic liver diseases due to NAFLD	Used in GBD 2019 and GBD 2021
Alcohol use	Cirrhosis and other chronic liver diseases due to other causes	Used in GBD 2019 and GBD 2021
Alcohol use	Pancreatitis	Used in GBD 2019 and GBD 2021
Alcohol use	Idiopathic epilepsy	Used in GBD 2019 and GBD 2021
Alcohol use	Alcohol use disorders	Used in GBD 2019 and GBD 2021
Alcohol use	Diabetes mellitus type 2	Used in GBD 2019 and GBD 2021
Alcohol use	Pedestrian road injuries	Used in GBD 2019 and GBD 2021
Alcohol use	Cyclist road injuries	Used in GBD 2019 and GBD 2021
Alcohol use	Motorcyclist road injuries	Used in GBD 2019 and GBD 2021
Alcohol use	Motor vehicle road injuries	Used in GBD 2019 and GBD 2021
Alcohol use	Other road injuries	Used in GBD 2019 and GBD 2021
Alcohol use	Other transport injuries	Used in GBD 2019 and GBD 2021
Alcohol use	Falls	Used in GBD 2019 and GBD 2021
Alcohol use	Drowning	Used in GBD 2019 and GBD 2021
Alcohol use	Fire, heat, and hot substances	Used in GBD 2019 and GBD 2021
Alcohol use	Poisoning by carbon monoxide	Used in GBD 2019 and GBD 2021
Alcohol use	Poisoning by other means	Used in GBD 2019 and GBD 2021
Alcohol use	Unintentional firearm injuries	Used in GBD 2019 and GBD 2021
Alcohol use	Venomous animal contact	Used in GBD 2019 and GBD 2021
Alcohol use	Non-venomous animal contact	Used in GBD 2019 and GBD 2021
Alcohol use	Environmental heat and cold exposure	Used in GBD 2019 and GBD 2021
Alcohol use	Other unintentional injuries	Used in GBD 2019 and GBD 2021
Alcohol use	Self-harm by firearm	Used in GBD 2019 and GBD 2021
Alcohol use	Self-harm by other specified means	Used in GBD 2019 and GBD 2021
Alcohol use	Physical violence by firearm	Used in GBD 2019 and GBD 2021
Alcohol use	Physical violence by sharp object	Used in GBD 2019 and GBD 2021
Alcohol use	Sexual violence	Used in GBD 2019 and GBD 2021
Alcohol use	Physical violence by other means	Used in GBD 2019 and GBD 2021
Drug use dependence and blood borne viruses	HIV/AIDS - Drug-susceptible Tuberculosis	Used in GBD 2019 and GBD 2021
Drug use dependence and blood borne viruses	HIV/AIDS - Multidrug-resistant Tuberculosis without extensive drug resistance	Used in GBD 2019 and GBD 2021
Drug use dependence and blood borne viruses	HIV/AIDS - Extensively drug-resistant Tuberculosis	Used in GBD 2019 and GBD 2021

Table S7. Status of risk–outcome pairs considered for inclusion in GBD 2021: included in both GBD 2019 and GBD 2021, added in GBD 2021, or removed in GBD 2021

Risk Name	Outcome Name	Status
Drug use dependence and blood borne viruses	HIV/AIDS resulting in other diseases	Used in GBD 2019 and GBD 2021
Drug use dependence and blood borne viruses	Acute hepatitis B	Used in GBD 2019 and GBD 2021
Drug use dependence and blood borne viruses	Acute hepatitis C	Used in GBD 2019 and GBD 2021
Drug use dependence and blood borne viruses	Liver cancer due to hepatitis B	Used in GBD 2019 and GBD 2021
Drug use dependence and blood borne viruses	Liver cancer due to hepatitis C	Used in GBD 2019 and GBD 2021
Drug use dependence and blood borne viruses	Cirrhosis and other chronic liver diseases due to hepatitis B	Used in GBD 2019 and GBD 2021
Drug use dependence and blood borne viruses	Cirrhosis and other chronic liver diseases due to hepatitis C	Used in GBD 2019 and GBD 2021
Drug use dependence and blood borne viruses	Opioid use disorders	Used in GBD 2019 and GBD 2021
Drug use dependence and blood borne viruses	Cocaine use disorders	Used in GBD 2019 and GBD 2021
Drug use dependence and blood borne viruses	Amphetamine use disorders	Used in GBD 2019 and GBD 2021
Drug use dependence and blood borne viruses	Cannabis use disorders	Used in GBD 2019 and GBD 2021
Drug use dependence and blood borne viruses	Other drug use disorders	Used in GBD 2019 and GBD 2021
Suicide due to drug use disorders	Self-harm by firearm	Used in GBD 2019 and GBD 2021
Suicide due to drug use disorders	Self-harm by other specified means	Used in GBD 2019 and GBD 2021
Diet low in fruits	Tracheal, bronchus, and lung cancer	Used in GBD 2019 and GBD 2021
Diet low in fruits	Ischemic heart disease	Used in GBD 2019 and GBD 2021
Diet low in fruits	Ischemic stroke	Used in GBD 2019 and GBD 2021
Diet low in fruits	Intracerebral hemorrhage	Used in GBD 2019 and GBD 2021
Diet low in fruits	Subarachnoid hemorrhage	Used in GBD 2019 and GBD 2021
Diet low in fruits	Diabetes mellitus type 2	Used in GBD 2019 and GBD 2021
Diet low in vegetables	Esophageal cancer	Used in GBD 2019 and GBD 2021
Diet low in vegetables	Ischemic heart disease	Used in GBD 2019 and GBD 2021
Diet low in vegetables	Ischemic stroke	Used in GBD 2019 and GBD 2021
Diet low in vegetables	Intracerebral hemorrhage	Used in GBD 2019 and GBD 2021
Diet low in vegetables	Subarachnoid hemorrhage	Used in GBD 2019 and GBD 2021
Diet low in legumes	Ischemic heart disease	Used in GBD 2019 and GBD 2021
Diet low in whole grains	Colon and rectum cancer	Used in GBD 2019 and GBD 2021
Diet low in whole grains	Ischemic heart disease	Used in GBD 2019 and GBD 2021
Diet low in whole grains	Ischemic stroke	Used in GBD 2019 and GBD 2021
Diet low in whole grains	Diabetes mellitus type 2	Used in GBD 2019 and GBD 2021
Diet low in nuts and seeds	Ischemic heart disease	Used in GBD 2019 and GBD 2021

Table S7. Status of risk–outcome pairs considered for inclusion in GBD 2021: included in both GBD 2019 and GBD 2021, added in GBD 2021, or removed in GBD 2021

Risk Name	Outcome Name	Status
Diet low in milk	Colon and rectum cancer	Used in GBD 2019 and GBD 2021
Diet high in red meat	Colon and rectum cancer	Used in GBD 2019 and GBD 2021
Diet high in red meat	Breast cancer	Used in GBD 2019 and GBD 2021
Diet high in red meat	Ischemic heart disease	Used in GBD 2019 and GBD 2021
Diet high in red meat	Ischemic stroke	Used in GBD 2019 and GBD 2021
Diet high in red meat	Intracerebral hemorrhage	Used in GBD 2019 and GBD 2021
Diet high in red meat	Subarachnoid hemorrhage	Used in GBD 2019 and GBD 2021
Diet high in red meat	Diabetes mellitus type 2	Used in GBD 2019 and GBD 2021
Diet high in processed meat	Colon and rectum cancer	Used in GBD 2019 and GBD 2021
Diet high in processed meat	Ischemic heart disease	Used in GBD 2019 and GBD 2021
Diet high in processed meat	Diabetes mellitus type 2	Used in GBD 2019 and GBD 2021
Diet high in sugar-sweetened beverages	Ischemic heart disease	Used in GBD 2019 and GBD 2021
Diet high in sugar-sweetened beverages	Diabetes mellitus type 2	Used in GBD 2019 and GBD 2021
Diet low in fiber	Colon and rectum cancer	Used in GBD 2019 and GBD 2021
Diet low in fiber	Ischemic heart disease	Used in GBD 2019 and GBD 2021
Diet low in fiber	Ischemic stroke	Used in GBD 2019 and GBD 2021
Diet low in fiber	Intracerebral hemorrhage	Used in GBD 2019 and GBD 2021
Diet low in fiber	Subarachnoid hemorrhage	Used in GBD 2019 and GBD 2021
Diet low in fiber	Diabetes mellitus type 2	Used in GBD 2019 and GBD 2021
Diet low in calcium	Colon and rectum cancer	Used in GBD 2019 and GBD 2021
Diet low in seafood omega-3 fatty acids	Ischemic heart disease	Used in GBD 2019 and GBD 2021
Diet low in polyunsaturated fatty acids	Ischemic heart disease	Used in GBD 2019 and GBD 2021
Diet high in trans fatty acids	Ischemic heart disease	Used in GBD 2019 and GBD 2021
Diet high in sodium	Stomach cancer	Used in GBD 2019 and GBD 2021
Diet high in sodium	Ischemic heart disease	Used in GBD 2019 and GBD 2021
Diet high in sodium	Ischemic stroke	Used in GBD 2019 and GBD 2021
Diet high in sodium	Intracerebral hemorrhage	Used in GBD 2019 and GBD 2021
Diet high in sodium	Subarachnoid hemorrhage	Used in GBD 2019 and GBD 2021
Diet high in sodium	Hypertensive heart disease	Used in GBD 2019 and GBD 2021
Diet high in sodium	Atrial fibrillation and flutter	Used in GBD 2019 and GBD 2021
Diet high in sodium	Aortic aneurysm	Used in GBD 2019 and GBD 2021

Table S7. Status of risk–outcome pairs considered for inclusion in GBD 2021: included in both GBD 2019 and GBD 2021, added in GBD 2021, or removed in GBD 2021

Risk Name	Outcome Name	Status
Diet high in sodium	Peripheral artery disease	Used in GBD 2019 and GBD 2021
Diet high in sodium	Chronic kidney disease due to diabetes mellitus type 2	Used in GBD 2019 and GBD 2021
Diet high in sodium	Chronic kidney disease due to hypertension	Used in GBD 2019 and GBD 2021
Diet high in sodium	Chronic kidney disease due to glomerulonephritis	Used in GBD 2019 and GBD 2021
Diet high in sodium	Chronic kidney disease due to other and unspecified causes	Used in GBD 2019 and GBD 2021
Intimate partner violence (HIV PAF approach)	HIV/AIDS - Drug-susceptible Tuberculosis	Used in GBD 2019 and GBD 2021
Intimate partner violence (HIV PAF approach)	HIV/AIDS - Multidrug-resistant Tuberculosis without extensive drug resistance	Used in GBD 2019 and GBD 2021
Intimate partner violence (HIV PAF approach)	HIV/AIDS - Extensively drug-resistant Tuberculosis	Used in GBD 2019 and GBD 2021
Intimate partner violence (HIV PAF approach)	HIV/AIDS resulting in other diseases	Used in GBD 2019 and GBD 2021
Intimate partner violence (exposure approach)	Major depressive disorder	Used in GBD 2019 and GBD 2021
Intimate partner violence (direct PAF approach)	Physical violence by firearm	Used in GBD 2019 and GBD 2021
Intimate partner violence (direct PAF approach)	Physical violence by sharp object	Used in GBD 2019 and GBD 2021
Intimate partner violence (direct PAF approach)	Sexual violence	Used in GBD 2019 and GBD 2021
Intimate partner violence (direct PAF approach)	Physical violence by other means	Used in GBD 2019 and GBD 2021
Childhood sexual abuse against females	Major depressive disorder	Used in GBD 2019 and GBD 2021
Childhood sexual abuse against females	Alcohol use disorders	Used in GBD 2019 and GBD 2021
Childhood sexual abuse against males	Major depressive disorder	Used in GBD 2019 and GBD 2021
Childhood sexual abuse against males	Alcohol use disorders	Used in GBD 2019 and GBD 2021
Bullying victimization	Major depressive disorder	Used in GBD 2019 and GBD 2021
Bullying victimization	Anxiety disorders	Used in GBD 2019 and GBD 2021
Unsafe sex	HIV/AIDS - Drug-susceptible Tuberculosis	Used in GBD 2019 and GBD 2021
Unsafe sex	HIV/AIDS - Multidrug-resistant Tuberculosis without extensive drug resistance	Used in GBD 2019 and GBD 2021
Unsafe sex	HIV/AIDS - Extensively drug-resistant Tuberculosis	Used in GBD 2019 and GBD 2021
Unsafe sex	HIV/AIDS resulting in other diseases	Used in GBD 2019 and GBD 2021
Unsafe sex	Syphilis	Used in GBD 2019 and GBD 2021
Unsafe sex	Chlamydial infection	Used in GBD 2019 and GBD 2021
Unsafe sex	Gonococcal infection	Used in GBD 2019 and GBD 2021
Unsafe sex	Trichomoniasis	Used in GBD 2019 and GBD 2021
Unsafe sex	Genital herpes	Used in GBD 2019 and GBD 2021
Unsafe sex	Other sexually transmitted infections	Used in GBD 2019 and GBD 2021

Table S7. Status of risk–outcome pairs considered for inclusion in GBD 2021: included in both GBD 2019 and GBD 2021, added in GBD 2021, or removed in GBD 2021

Risk Name	Outcome Name	Status
Unsafe sex	Cervical cancer	Used in GBD 2019 and GBD 2021
Low physical activity	Colon and rectum cancer	Used in GBD 2019 and GBD 2021
Low physical activity	Breast cancer	Used in GBD 2019 and GBD 2021
Low physical activity	Ischemic heart disease	Used in GBD 2019 and GBD 2021
Low physical activity	Ischemic stroke	Used in GBD 2019 and GBD 2021
Low physical activity	Diabetes mellitus type 2	Used in GBD 2019 and GBD 2021
High LDL cholesterol	Ischemic heart disease	Used in GBD 2019 and GBD 2021
High LDL cholesterol	Ischemic stroke	Used in GBD 2019 and GBD 2021
High systolic blood pressure	Ischemic heart disease	Used in GBD 2019 and GBD 2021
High systolic blood pressure	Ischemic stroke	Used in GBD 2019 and GBD 2021
High systolic blood pressure	Intracerebral hemorrhage	Used in GBD 2019 and GBD 2021
High systolic blood pressure	Subarachnoid hemorrhage	Used in GBD 2019 and GBD 2021
High systolic blood pressure	Hypertensive heart disease	Used in GBD 2019 and GBD 2021
High systolic blood pressure	Atrial fibrillation and flutter	Used in GBD 2019 and GBD 2021
High systolic blood pressure	Aortic aneurysm	Used in GBD 2019 and GBD 2021
High systolic blood pressure	Peripheral artery disease	Used in GBD 2019 and GBD 2021
High systolic blood pressure	Chronic kidney disease due to diabetes mellitus type 2	Used in GBD 2019 and GBD 2021
High systolic blood pressure	Chronic kidney disease due to hypertension	Used in GBD 2019 and GBD 2021
High systolic blood pressure	Chronic kidney disease due to glomerulonephritis	Used in GBD 2019 and GBD 2021
High systolic blood pressure	Chronic kidney disease due to other and unspecified causes	Used in GBD 2019 and GBD 2021
High body-mass index in adults	Colon and rectum cancer	Used in GBD 2019 and GBD 2021
High body-mass index in adults	Liver cancer due to hepatitis B	Used in GBD 2019 and GBD 2021
High body-mass index in adults	Liver cancer due to hepatitis C	Used in GBD 2019 and GBD 2021
High body-mass index in adults	Liver cancer due to alcohol use	Used in GBD 2019 and GBD 2021
High body-mass index in adults	Liver cancer due to other causes (internal)	Used in GBD 2019 and GBD 2021
High body-mass index in adults	Gallbladder and biliary tract cancer	Used in GBD 2019 and GBD 2021
High body-mass index in adults	Pancreatic cancer	Used in GBD 2019 and GBD 2021
High body-mass index in adults	Breast cancer	Used in GBD 2019 and GBD 2021
High body-mass index in adults	Uterine cancer	Used in GBD 2019 and GBD 2021
High body-mass index in adults	Ovarian cancer	Used in GBD 2019 and GBD 2021
High body-mass index in adults	Kidney cancer	Used in GBD 2019 and GBD 2021

Table S7. Status of risk–outcome pairs considered for inclusion in GBD 2021: included in both GBD 2019 and GBD 2021, added in GBD 2021, or removed in GBD 2021

Risk Name	Outcome Name	Status
High body-mass index in adults	Thyroid cancer	Used in GBD 2019 and GBD 2021
High body-mass index in adults	Burkitt lymphoma	Used in GBD 2019 and GBD 2021
High body-mass index in adults	Other non-Hodgkin lymphoma	Used in GBD 2019 and GBD 2021
High body-mass index in adults	Multiple myeloma	Used in GBD 2019 and GBD 2021
High body-mass index in adults	Acute lymphoid leukemia	Used in GBD 2019 and GBD 2021
High body-mass index in adults	Chronic lymphoid leukemia	Used in GBD 2019 and GBD 2021
High body-mass index in adults	Acute myeloid leukemia	Used in GBD 2019 and GBD 2021
High body-mass index in adults	Chronic myeloid leukemia	Used in GBD 2019 and GBD 2021
High body-mass index in adults	Other leukemia	Used in GBD 2019 and GBD 2021
High body-mass index in adults	Ischemic heart disease	Used in GBD 2019 and GBD 2021
High body-mass index in adults	Ischemic stroke	Used in GBD 2019 and GBD 2021
High body-mass index in adults	Intracerebral hemorrhage	Used in GBD 2019 and GBD 2021
High body-mass index in adults	Subarachnoid hemorrhage	Used in GBD 2019 and GBD 2021
High body-mass index in adults	Hypertensive heart disease	Used in GBD 2019 and GBD 2021
High body-mass index in adults	Atrial fibrillation and flutter	Used in GBD 2019 and GBD 2021
High body-mass index in adults	Asthma	Used in GBD 2019 and GBD 2021
High body-mass index in adults	Gallbladder and biliary diseases	Used in GBD 2019 and GBD 2021
High body-mass index in adults	Alzheimer's disease and other dementias	Used in GBD 2019 and GBD 2021
High body-mass index in adults	Diabetes mellitus type 2	Used in GBD 2019 and GBD 2021
High body-mass index in adults	Chronic kidney disease due to diabetes mellitus type 2	Used in GBD 2019 and GBD 2021
High body-mass index in adults	Chronic kidney disease due to hypertension	Used in GBD 2019 and GBD 2021
High body-mass index in adults	Chronic kidney disease due to glomerulonephritis	Used in GBD 2019 and GBD 2021
High body-mass index in adults	Chronic kidney disease due to other and unspecified causes	Used in GBD 2019 and GBD 2021
High body-mass index in adults	Cataract	Used in GBD 2019 and GBD 2021
High body-mass index in adults	Osteoarthritis hip	Used in GBD 2019 and GBD 2021
High body-mass index in adults	Osteoarthritis knee	Used in GBD 2019 and GBD 2021
High body-mass index in adults	Low back pain	Used in GBD 2019 and GBD 2021
High body-mass index in adults	Gout	Used in GBD 2019 and GBD 2021
High body-mass index in children	Asthma	Used in GBD 2019 and GBD 2021
Low bone mineral density	Pedestrian road injuries	Used in GBD 2019 and GBD 2021
Low bone mineral density	Cyclist road injuries	Used in GBD 2019 and GBD 2021

Table S7. Status of risk–outcome pairs considered for inclusion in GBD 2021: included in both GBD 2019 and GBD 2021, added in GBD 2021, or removed in GBD 2021

Risk Name	Outcome Name	Status
Low bone mineral density	Motorcyclist road injuries	Used in GBD 2019 and GBD 2021
Low bone mineral density	Motor vehicle road injuries	Used in GBD 2019 and GBD 2021
Low bone mineral density	Other road injuries	Used in GBD 2019 and GBD 2021
Low bone mineral density	Other transport injuries	Used in GBD 2019 and GBD 2021
Low bone mineral density	Falls	Used in GBD 2019 and GBD 2021
Low bone mineral density	Other exposure to mechanical forces	Used in GBD 2019 and GBD 2021
Low bone mineral density	Non-venomous animal contact	Used in GBD 2019 and GBD 2021
Low bone mineral density	Physical violence by other means	Used in GBD 2019 and GBD 2021
Kidney dysfunction	Ischemic heart disease	Used in GBD 2019 and GBD 2021
Kidney dysfunction	Ischemic stroke	Used in GBD 2019 and GBD 2021
Kidney dysfunction	Intracerebral hemorrhage	Used in GBD 2019 and GBD 2021
Kidney dysfunction	Peripheral artery disease	Used in GBD 2019 and GBD 2021
Kidney dysfunction	Chronic kidney disease due to diabetes mellitus type 1	Used in GBD 2019 and GBD 2021
Kidney dysfunction	Chronic kidney disease due to diabetes mellitus type 2	Used in GBD 2019 and GBD 2021
Kidney dysfunction	Chronic kidney disease due to hypertension	Used in GBD 2019 and GBD 2021
Kidney dysfunction	Chronic kidney disease due to glomerulonephritis	Used in GBD 2019 and GBD 2021
Kidney dysfunction	Chronic kidney disease due to other and unspecified causes	Used in GBD 2019 and GBD 2021
Kidney dysfunction	Gout	Used in GBD 2019 and GBD 2021
Nitrogen dioxide pollution	Asthma	Added in GBD 2021
High temperature	Myocarditis	Added in GBD 2021
High temperature	Alcoholic cardiomyopathy	Added in GBD 2021
High temperature	Other cardiomyopathy	Added in GBD 2021
High temperature	Other transport injuries	Added in GBD 2021
High temperature	Venomous animal contact	Added in GBD 2021
High temperature	Non-venomous animal contact	Added in GBD 2021
High temperature	Exposure to forces of nature	Added in GBD 2021
High temperature	Other unintentional injuries (internal)	Added in GBD 2021
Low temperature	Myocarditis	Added in GBD 2021
Low temperature	Alcoholic cardiomyopathy	Added in GBD 2021
Low temperature	Other cardiomyopathy	Added in GBD 2021
Low temperature	Other transport injuries	Added in GBD 2021

Table S7. Status of risk–outcome pairs considered for inclusion in GBD 2021: included in both GBD 2019 and GBD 2021, added in GBD 2021, or removed in GBD 2021		
**Risk Name**	**Outcome Name**	**Status**
Low temperature	Venomous animal contact	Added in GBD 2021
Low temperature	Non-venomous animal contact	Added in GBD 2021
Low temperature	Exposure to forces of nature	Added in GBD 2021
Low temperature	Other unintentional injuries (internal)	Added in GBD 2021
Child underweight	Malaria	Added in GBD 2021
Child wasting	Malaria	Added in GBD 2021
Child stunting	Malaria	Added in GBD 2021
High alcohol use	Liver cancer due to other causes	Added in GBD 2021
High alcohol use	Other exposure to mechanical forces	Added in GBD 2021
Diet low in fruits	Drug-susceptible tuberculosis	Added in GBD 2021
Diet low in fruits	Multidrug-resistant tuberculosis without extensive drug resistance	Added in GBD 2021
Diet low in fruits	Extensively drug-resistant tuberculosis	Added in GBD 2021
Diet low in fruits	Hypertensive heart disease	Added in GBD 2021
Diet low in fruits	Aortic aneurysm	Added in GBD 2021
Diet low in fruits	Lower extremity peripheral arterial disease	Added in GBD 2021
Diet low in fruits	Chronic kidney disease due to diabetes mellitus type 2	Added in GBD 2021
Diet low in fruits	Chronic kidney disease due to hypertension	Added in GBD 2021
Diet low in fruits	Chronic kidney disease due to glomerulonephritis	Added in GBD 2021
Diet low in fruits	Chronic kidney disease due to other and unspecified causes	Added in GBD 2021
Diet low in vegetables	Drug-susceptible tuberculosis	Added in GBD 2021
Diet low in vegetables	Multidrug-resistant tuberculosis without extensive drug resistance	Added in GBD 2021
Diet low in vegetables	Extensively drug-resistant tuberculosis	Added in GBD 2021
Diet low in vegetables	Hypertensive heart disease	Added in GBD 2021
Diet low in vegetables	Aortic aneurysm	Added in GBD 2021
Diet low in vegetables	Lower extremity peripheral arterial disease	Added in GBD 2021
Diet low in vegetables	Diabetes mellitus type 2	Added in GBD 2021
Diet low in vegetables	Chronic kidney disease due to diabetes mellitus type 2	Added in GBD 2021
Diet low in vegetables	Chronic kidney disease due to hypertension	Added in GBD 2021
Diet low in vegetables	Chronic kidney disease due to glomerulonephritis	Added in GBD 2021
Diet low in vegetables	Chronic kidney disease due to other and unspecified causes	Added in GBD 2021
Diet low in whole grains	Drug-susceptible tuberculosis	Added in GBD 2021

Table S7. Status of risk–outcome pairs considered for inclusion in GBD 2021: included in both GBD 2019 and GBD 2021, added in GBD 2021, or removed in GBD 2021

Risk Name	Outcome Name	Status
Diet low in whole grains	Multidrug-resistant tuberculosis without extensive drug resistance	Added in GBD 2021
Diet low in whole grains	Extensively drug-resistant tuberculosis	Added in GBD 2021
Diet low in whole grains	Lower extremity peripheral arterial disease	Added in GBD 2021
Diet low in whole grains	Chronic kidney disease due to diabetes mellitus type 2	Added in GBD 2021
Diet low in whole grains	Chronic kidney disease due to hypertension	Added in GBD 2021
Diet low in whole grains	Chronic kidney disease due to glomerulonephritis	Added in GBD 2021
Diet low in whole grains	Chronic kidney disease due to other and unspecified causes	Added in GBD 2021
Diet low in milk	Prostate cancer	Added in GBD 2021
Diet high in red meat	Drug-susceptible tuberculosis	Added in GBD 2021
Diet high in red meat	Multidrug-resistant tuberculosis without extensive drug resistance	Added in GBD 2021
Diet high in red meat	Extensively drug-resistant tuberculosis	Added in GBD 2021
Diet high in red meat	Lower extremity peripheral arterial disease	Added in GBD 2021
Diet high in red meat	Chronic kidney disease due to diabetes mellitus type 2	Added in GBD 2021
Diet high in red meat	Chronic kidney disease due to hypertension	Added in GBD 2021
Diet high in red meat	Chronic kidney disease due to glomerulonephritis	Added in GBD 2021
Diet high in red meat	Chronic kidney disease due to other and unspecified causes	Added in GBD 2021
Diet high in processed meat	Drug-susceptible tuberculosis	Added in GBD 2021
Diet high in processed meat	Multidrug-resistant tuberculosis without extensive drug resistance	Added in GBD 2021
Diet high in processed meat	Extensively drug-resistant tuberculosis	Added in GBD 2021
Diet high in processed meat	Ischemic stroke	Added in GBD 2021
Diet high in processed meat	Lower extremity peripheral arterial disease	Added in GBD 2021
Diet high in processed meat	Chronic kidney disease due to diabetes mellitus type 2	Added in GBD 2021
Diet high in processed meat	Chronic kidney disease due to hypertension	Added in GBD 2021
Diet high in processed meat	Chronic kidney disease due to glomerulonephritis	Added in GBD 2021
Diet high in processed meat	Chronic kidney disease due to other and unspecified causes	Added in GBD 2021
Diet high in sugar-sweetened beverages	Drug-susceptible tuberculosis	Added in GBD 2021
Diet high in sugar-sweetened beverages	Multidrug-resistant tuberculosis without extensive drug resistance	Added in GBD 2021
Diet high in sugar-sweetened beverages	Extensively drug-resistant tuberculosis	Added in GBD 2021
Diet high in sugar-sweetened beverages	Ischemic stroke	Added in GBD 2021
Diet high in sugar-sweetened beverages	Lower extremity peripheral arterial disease	Added in GBD 2021
Diet high in sugar-sweetened beverages	Chronic kidney disease due to diabetes mellitus type 2	Added in GBD 2021

Table S7. Status of risk–outcome pairs considered for inclusion in GBD 2021: included in both GBD 2019 and GBD 2021, added in GBD 2021, or removed in GBD 2021

Risk Name	Outcome Name	Status
Diet high in sugar-sweetened beverages	Chronic kidney disease due to hypertension	Added in GBD 2021
Diet high in sugar-sweetened beverages	Chronic kidney disease due to glomerulonephritis	Added in GBD 2021
Diet high in sugar-sweetened beverages	Chronic kidney disease due to other and unspecified causes	Added in GBD 2021
Diet low in calcium	Prostate cancer	Added in GBD 2021
Diet low in omega-6 polyunsaturated fatty acids	Ischemic stroke	Added in GBD 2021
Low physical activity	Drug-susceptible tuberculosis	Added in GBD 2021
Low physical activity	Multidrug-resistant tuberculosis without extensive drug resistance	Added in GBD 2021
Low physical activity	Extensively drug-resistant tuberculosis	Added in GBD 2021
Low physical activity	Lower extremity peripheral arterial disease	Added in GBD 2021
Low physical activity	Chronic kidney disease due to diabetes mellitus type 2	Added in GBD 2021
Low physical activity	Chronic kidney disease due to hypertension	Added in GBD 2021
Low physical activity	Chronic kidney disease due to glomerulonephritis	Added in GBD 2021
Low physical activity	Chronic kidney disease due to other and unspecified causes	Added in GBD 2021
High fasting plasma glucose	Latent tuberculosis infection	Added in GBD 2021
High fasting plasma glucose	Drug-susceptible tuberculosis	Added in GBD 2021
High fasting plasma glucose	Multidrug-resistant tuberculosis without extensive drug resistance	Added in GBD 2021
High fasting plasma glucose	Extensively drug-resistant tuberculosis	Added in GBD 2021
High fasting plasma glucose	Colon and rectum cancer	Added in GBD 2021
High fasting plasma glucose	Liver cancer due to NASH	Added in GBD 2021
High fasting plasma glucose	Liver cancer due to other causes	Added in GBD 2021
High fasting plasma glucose	Pancreatic cancer	Added in GBD 2021
High fasting plasma glucose	Tracheal, bronchus, and lung cancer	Added in GBD 2021
High fasting plasma glucose	Breast cancer	Added in GBD 2021
High fasting plasma glucose	Bladder cancer	Added in GBD 2021
High fasting plasma glucose	Ischemic heart disease	Added in GBD 2021
High fasting plasma glucose	Ischemic stroke	Added in GBD 2021
High fasting plasma glucose	Intracerebral hemorrhage	Added in GBD 2021
High fasting plasma glucose	Lower extremity peripheral arterial disease	Added in GBD 2021
High fasting plasma glucose	Alzheimer's disease and other dementias	Added in GBD 2021
High fasting plasma glucose	Diabetes mellitus type 1	Added in GBD 2021
High fasting plasma glucose	Diabetes mellitus type 2	Added in GBD 2021

Table S7. Status of risk–outcome pairs considered for inclusion in GBD 2021: included in both GBD 2019 and GBD 2021, added in GBD 2021, or removed in GBD 2021

Risk Name	Outcome Name	Status
High fasting plasma glucose	Chronic kidney disease due to diabetes mellitus type 1	Added in GBD 2021
High fasting plasma glucose	Chronic kidney disease due to diabetes mellitus type 2	Added in GBD 2021
High fasting plasma glucose	Chronic kidney disease due to hypertension	Added in GBD 2021
High fasting plasma glucose	Chronic kidney disease due to glomerulonephritis	Added in GBD 2021
High fasting plasma glucose	Chronic kidney disease due to other and unspecified causes	Added in GBD 2021
High fasting plasma glucose	Glaucoma	Added in GBD 2021
High fasting plasma glucose	Cataract	Added in GBD 2021
High body-mass index in adults	Drug-susceptible tuberculosis	Added in GBD 2021
High body-mass index in adults	Multidrug-resistant tuberculosis without extensive drug resistance	Added in GBD 2021
High body-mass index in adults	Extensively drug-resistant tuberculosis	Added in GBD 2021
High body-mass index in adults	Aortic aneurysm	Added in GBD 2021
High body-mass index in adults	Lower extremity peripheral arterial disease	Added in GBD 2021
Alcohol use	Lower respiratory infections	Removed from GBD 2021
Alcohol use	Exposure to forces of nature	Removed from GBD 2021
High systolic blood pressure	Rheumatic heart disease	Removed from GBD 2021
High systolic blood pressure	Endocarditis	Removed from GBD 2021
High systolic blood pressure	Other cardiovascular and circulatory diseases	Removed from GBD 2021
High systolic blood pressure	Other cardiomyopathy	Removed from GBD 2021
High systolic blood pressure	Non-rheumatic calcific aortic valve disease	Removed from GBD 2021
High systolic blood pressure	Chronic kidney disease due to diabetes mellitus type 1	Removed from GBD 2021
Diet low in fruits	Esophageal cancer	Removed from GBD 2021
Diet low in nuts and seeds	Diabetes mellitus type 2	Removed from GBD 2021
Diet high in sodium	Rheumatic heart disease	Removed from GBD 2021
Diet high in sodium	Endocarditis	Removed from GBD 2021
Diet high in sodium	Other cardiovascular and circulatory diseases	Removed from GBD 2021
Diet high in sodium	Other cardiomyopathy	Removed from GBD 2021
Diet high in sodium	Non-rheumatic calcific aortic valve disease	Removed from GBD 2021
Diet high in sodium	Chronic kidney disease due to diabetes mellitus type 1	Removed from GBD 2021
High fasting plasma glucose	Ovarian cancer	Removed from GBD 2021
High fasting plasma glucose	Subarachnoid hemorrhage	Removed from GBD 2021
Lead exposure in bone	Chronic kidney disease due to diabetes mellitus type 1	Removed from GBD 2021

Table S7. Status of risk–outcome pairs considered for inclusion in GBD 2021: included in both GBD 2019 and GBD 2021, added in GBD 2021, or removed in GBD 2021

Risk Name	Outcome Name	Status
Lead exposure in bone	Endocarditis	Removed from GBD 2021
Lead exposure in bone	Non-rheumatic calcific aortic valve disease	Removed from GBD 2021
Lead exposure in bone	Other cardiomyopathy	Removed from GBD 2021
Lead exposure in bone	Other cardiovascular and circulatory diseases	Removed from GBD 2021
Lead exposure in bone	Rheumatic heart disease	Removed from GBD 2021
High body-mass index in adults	Esophageal cancer	Removed from GBD 2021

Table S8. Status of mediated risk–outcome pairs considered for inclusion in GBD 2021: included in both GBD 2019 and GBD 2021, added in GBD 2021, or removed in GBD 2021

Risk Name	Mediator Name	Outcome Name	Status
Childhood sexual abuse against females	High alcohol use	Alcohol use disorders	Used in GBD 2019 and GBD 2021
Childhood sexual abuse against males	High alcohol use	Alcohol use disorders	Used in GBD 2019 and GBD 2021
Diet high in processed meat	High fasting plasma glucose	Diabetes mellitus type 2	Used in GBD 2019 and GBD 2021
Diet high in processed meat	High fasting plasma glucose	Ischemic heart disease	Used in GBD 2019 and GBD 2021
Diet high in red meat	High fasting plasma glucose	Diabetes mellitus type 2	Used in GBD 2019 and GBD 2021
Diet high in sodium	High systolic blood pressure	Aortic aneurysm	Used in GBD 2019 and GBD 2021
Diet high in sodium	High systolic blood pressure	Atrial fibrillation and flutter	Used in GBD 2019 and GBD 2021
Diet high in sodium	High systolic blood pressure	Hypertensive heart disease	Used in GBD 2019 and GBD 2021
Diet high in sodium	High systolic blood pressure	Intracerebral hemorrhage	Used in GBD 2019 and GBD 2021
Diet high in sodium	High systolic blood pressure	Ischemic heart disease	Used in GBD 2019 and GBD 2021
Diet high in sodium	High systolic blood pressure	Ischemic stroke	Used in GBD 2019 and GBD 2021
Diet high in sodium	High systolic blood pressure	Lower extremity peripheral arterial disease	Used in GBD 2019 and GBD 2021
Diet high in sodium	High systolic blood pressure	Subarachnoid hemorrhage	Used in GBD 2019 and GBD 2021
Diet high in sodium	High systolic blood pressure	Chronic kidney disease due to diabetes mellitus type 2	Used in GBD 2019 and GBD 2021
Diet high in sodium	High systolic blood pressure	Chronic kidney disease due to glomerulonephritis	Used in GBD 2019 and GBD 2021
Diet high in sodium	High systolic blood pressure	Chronic kidney disease due to hypertension	Used in GBD 2019 and GBD 2021
Diet high in sodium	High systolic blood pressure	Chronic kidney disease due to other and unspecified causes	Used in GBD 2019 and GBD 2021
Diet high in sugar-sweetened beverages	High fasting plasma glucose	Diabetes mellitus type 2	Used in GBD 2019 and GBD 2021
Diet high in trans fatty acids	High LDL cholesterol	Ischemic heart disease	Used in GBD 2019 and GBD 2021
Diet low in fruits	High fasting plasma glucose	Diabetes mellitus type 2	Used in GBD 2019 and GBD 2021
Diet low in fruits	High fasting plasma glucose	Ischemic stroke	Used in GBD 2019 and GBD 2021
Diet low in fruits	High LDL cholesterol	Ischemic heart disease	Used in GBD 2019 and GBD 2021
Diet low in fruits	High LDL cholesterol	Ischemic stroke	Used in GBD 2019 and GBD 2021
Diet low in fruits	High systolic blood pressure	Intracerebral hemorrhage	Used in GBD 2019 and GBD 2021
Diet low in fruits	High systolic blood pressure	Ischemic heart disease	Used in GBD 2019 and GBD 2021
Diet low in fruits	High systolic blood pressure	Ischemic stroke	Used in GBD 2019 and GBD 2021
Diet low in fruits	High systolic blood pressure	Subarachnoid hemorrhage	Used in GBD 2019 and GBD 2021
Diet low in milk	Diet low in calcium	Colon and rectum cancer	Used in GBD 2019 and GBD 2021
Diet low in nuts and seeds	High LDL cholesterol	Ischemic heart disease	Used in GBD 2019 and GBD 2021
Diet low in vegetables	High fasting plasma glucose	Intracerebral hemorrhage	Used in GBD 2019 and GBD 2021
Diet low in vegetables	High fasting plasma glucose	Ischemic heart disease	Used in GBD 2019 and GBD 2021

Table S8. Status of mediated risk–outcome pairs considered for inclusion in GBD 2021: included in both GBD 2019 and GBD 2021, added in GBD 2021, or removed in GBD 2021

Risk Name	Mediator Name	Outcome Name	Status
Diet low in vegetables	High fasting plasma glucose	Ischemic stroke	Used in GBD 2019 and GBD 2021
Diet low in vegetables	High LDL cholesterol	Ischemic heart disease	Used in GBD 2019 and GBD 2021
Diet low in vegetables	High LDL cholesterol	Ischemic stroke	Used in GBD 2019 and GBD 2021
Diet low in vegetables	High systolic blood pressure	Intracerebral hemorrhage	Used in GBD 2019 and GBD 2021
Diet low in vegetables	High systolic blood pressure	Ischemic heart disease	Used in GBD 2019 and GBD 2021
Diet low in vegetables	High systolic blood pressure	Ischemic stroke	Used in GBD 2019 and GBD 2021
Diet low in vegetables	High systolic blood pressure	Subarachnoid hemorrhage	Used in GBD 2019 and GBD 2021
Diet low in whole grains	High fasting plasma glucose	Diabetes mellitus type 2	Used in GBD 2019 and GBD 2021
Diet low in whole grains	High LDL cholesterol	Ischemic heart disease	Used in GBD 2019 and GBD 2021
Diet low in whole grains	High LDL cholesterol	Ischemic stroke	Used in GBD 2019 and GBD 2021
High body-mass index in adults	High fasting plasma glucose	Diabetes mellitus type 2	Used in GBD 2019 and GBD 2021
High body-mass index in adults	High fasting plasma glucose	Intracerebral hemorrhage	Used in GBD 2019 and GBD 2021
High body-mass index in adults	High fasting plasma glucose	Ischemic heart disease	Used in GBD 2019 and GBD 2021
High body-mass index in adults	High fasting plasma glucose	Ischemic stroke	Used in GBD 2019 and GBD 2021
High body-mass index in adults	High LDL cholesterol	Ischemic heart disease	Used in GBD 2019 and GBD 2021
High body-mass index in adults	High LDL cholesterol	Ischemic stroke	Used in GBD 2019 and GBD 2021
High body-mass index in adults	High systolic blood pressure	Atrial fibrillation and flutter	Used in GBD 2019 and GBD 2021
High body-mass index in adults	High systolic blood pressure	Hypertensive heart disease	Used in GBD 2019 and GBD 2021
High body-mass index in adults	High systolic blood pressure	Intracerebral hemorrhage	Used in GBD 2019 and GBD 2021
High body-mass index in adults	High systolic blood pressure	Ischemic heart disease	Used in GBD 2019 and GBD 2021
High body-mass index in adults	High systolic blood pressure	Ischemic stroke	Used in GBD 2019 and GBD 2021
High body-mass index in adults	High systolic blood pressure	Subarachnoid hemorrhage	Used in GBD 2019 and GBD 2021
High fasting plasma glucose	High LDL cholesterol	Ischemic heart disease	Used in GBD 2019 and GBD 2021
High fasting plasma glucose	High LDL cholesterol	Ischemic stroke	Used in GBD 2019 and GBD 2021
Lead exposure in bone	High systolic blood pressure	Aortic aneurysm	Used in GBD 2019 and GBD 2021
Lead exposure in bone	High systolic blood pressure	Atrial fibrillation and flutter	Used in GBD 2019 and GBD 2021
Lead exposure in bone	High systolic blood pressure	Hypertensive heart disease	Used in GBD 2019 and GBD 2021
Lead exposure in bone	High systolic blood pressure	Intracerebral hemorrhage	Used in GBD 2019 and GBD 2021
Lead exposure in bone	High systolic blood pressure	Ischemic heart disease	Used in GBD 2019 and GBD 2021
Lead exposure in bone	High systolic blood pressure	Ischemic stroke	Used in GBD 2019 and GBD 2021
Lead exposure in bone	High systolic blood pressure	Lower extremity peripheral arterial disease	Used in GBD 2019 and GBD 2021

Table S8. Status of mediated risk–outcome pairs considered for inclusion in GBD 2021: included in both GBD 2019 and GBD 2021, added in GBD 2021, or removed in GBD 2021

Risk Name	Mediator Name	Outcome Name	Status
Lead exposure in bone	High systolic blood pressure	Subarachnoid hemorrhage	Used in GBD 2019 and GBD 2021
Lead exposure in bone	High systolic blood pressure	Chronic kidney disease due to diabetes mellitus type 2	Used in GBD 2019 and GBD 2021
Lead exposure in bone	High systolic blood pressure	Chronic kidney disease due to glomerulonephritis	Used in GBD 2019 and GBD 2021
Lead exposure in bone	High systolic blood pressure	Chronic kidney disease due to hypertension	Used in GBD 2019 and GBD 2021
Lead exposure in bone	High systolic blood pressure	Chronic kidney disease due to other and unspecified causes	Used in GBD 2019 and GBD 2021
Low physical activity	High fasting plasma glucose	Diabetes mellitus type 2	Used in GBD 2019 and GBD 2021
Low physical activity	High fasting plasma glucose	Ischemic heart disease	Used in GBD 2019 and GBD 2021
Low physical activity	High fasting plasma glucose	Ischemic stroke	Used in GBD 2019 and GBD 2021
Smoking	High fasting plasma glucose	Diabetes mellitus type 2	Used in GBD 2019 and GBD 2021
Diet high in processed meat	High fasting plasma glucose	Ischemic Stroke	Added in GBD 2021
Diet high in processed meat	High fasting plasma glucose	Lower extremity peripheral arterial disease	Added in GBD 2021
Diet high in processed meat	High fasting plasma glucose	Drug-susceptible tuberculosis	Added in GBD 2021
Diet high in processed meat	High fasting plasma glucose	Multidrug-resistant tuberculosis without extensive drug resistance	Added in GBD 2021
Diet high in processed meat	High fasting plasma glucose	Extensively drug-resistant tuberculosis	Added in GBD 2021
Diet high in processed meat	High fasting plasma glucose	Chronic kidney disease due to diabetes mellitus type 2	Added in GBD 2021
Diet high in processed meat	High fasting plasma glucose	Chronic kidney disease due to glomerulonephritis	Added in GBD 2021
Diet high in processed meat	High fasting plasma glucose	Chronic kidney disease due to hypertension	Added in GBD 2021
Diet high in processed meat	High fasting plasma glucose	Chronic kidney disease due to other and unspecified causes	Added in GBD 2021
Diet high in red meat	High fasting plasma glucose	Lower extremity peripheral arterial disease	Added in GBD 2021
Diet high in red meat	High fasting plasma glucose	Drug-susceptible tuberculosis	Added in GBD 2021
Diet high in red meat	High fasting plasma glucose	Multidrug-resistant tuberculosis without extensive drug resistance	Added in GBD 2021
Diet high in red meat	High fasting plasma glucose	Extensively drug-resistant tuberculosis	Added in GBD 2021
Diet high in red meat	High fasting plasma glucose	Chronic kidney disease due to diabetes mellitus type 2	Added in GBD 2021
Diet high in red meat	High fasting plasma glucose	Chronic kidney disease due to glomerulonephritis	Added in GBD 2021
Diet high in red meat	High fasting plasma glucose	Chronic kidney disease due to hypertension	Added in GBD 2021
Diet high in red meat	High fasting plasma glucose	Chronic kidney disease due to other and unspecified causes	Added in GBD 2021
Diet high in sugar-sweetened beverages	High fasting plasma glucose	Ischemic Stroke	Added in GBD 2021
Diet high in sugar-sweetened beverages	High fasting plasma glucose	Lower extremity peripheral arterial disease	Added in GBD 2021
Diet high in sugar-sweetened beverages	High fasting plasma glucose	Drug-susceptible tuberculosis	Added in GBD 2021
Diet high in sugar-sweetened beverages	High fasting plasma glucose	Multidrug-resistant tuberculosis without extensive drug resistance	Added in GBD 2021
Diet high in sugar-sweetened beverages	High fasting plasma glucose	Extensively drug-resistant tuberculosis	Added in GBD 2021

Table S8. Status of mediated risk–outcome pairs considered for inclusion in GBD 2021: included in both GBD 2019 and GBD 2021, added in GBD 2021, or removed in GBD 2021			
**Risk Name**	**Mediator Name**	**Outcome Name**	**Status**
Diet high in sugar-sweetened beverages	High fasting plasma glucose	Chronic kidney disease due to diabetes mellitus type 2	Added in GBD 2021
Diet high in sugar-sweetened beverages	High fasting plasma glucose	Chronic kidney disease due to glomerulonephritis	Added in GBD 2021
Diet high in sugar-sweetened beverages	High fasting plasma glucose	Chronic kidney disease due to hypertension	Added in GBD 2021
Diet high in sugar-sweetened beverages	High fasting plasma glucose	Chronic kidney disease due to other and unspecified causes	Added in GBD 2021
Diet low in fruits	High fasting plasma glucose	Lower extremity peripheral arterial disease	Added in GBD 2021
Diet low in fruits	High fasting plasma glucose	Drug-susceptible tuberculosis	Added in GBD 2021
Diet low in fruits	High fasting plasma glucose	Multidrug-resistant tuberculosis without extensive drug resistance	Added in GBD 2021
Diet low in fruits	High fasting plasma glucose	Extensively drug-resistant tuberculosis	Added in GBD 2021
Diet low in fruits	High systolic blood pressure	Hypertensive heart disease	Added in GBD 2021
Diet low in fruits	High systolic blood pressure	Aortic Aneurysm	Added in GBD 2021
Diet low in fruits	High fasting plasma glucose	Chronic kidney disease due to diabetes mellitus type 2	Added in GBD 2021
Diet low in fruits	High fasting plasma glucose	Chronic kidney disease due to glomerulonephritis	Added in GBD 2021
Diet low in fruits	High fasting plasma glucose	Chronic kidney disease due to hypertension	Added in GBD 2021
Diet low in fruits	High fasting plasma glucose	Chronic kidney disease due to other and unspecified causes	Added in GBD 2021
Diet low in fruits	High systolic blood pressure	Chronic kidney disease due to diabetes mellitus type 2	Added in GBD 2021
Diet low in fruits	High systolic blood pressure	Chronic kidney disease due to glomerulonephritis	Added in GBD 2021
Diet low in fruits	High systolic blood pressure	Chronic kidney disease due to hypertension	Added in GBD 2021
Diet low in fruits	High systolic blood pressure	Chronic kidney disease due to other and unspecified causes	Added in GBD 2021
Diet low in polyunsaturated fatty acids	High LDL cholesterol	Ischemic heart disease	Added in GBD 2021
Diet low in polyunsaturated fatty acids	High LDL cholesterol	Ischemic stroke	Added in GBD 2021
Diet low in vegetables	High fasting plasma glucose	Lower extremity peripheral arterial disease	Added in GBD 2021
Diet low in vegetables	High fasting plasma glucose	Drug-susceptible tuberculosis	Added in GBD 2021
Diet low in vegetables	High fasting plasma glucose	Multidrug-resistant tuberculosis without extensive drug resistance	Added in GBD 2021
Diet low in vegetables	High fasting plasma glucose	Extensively drug-resistant tuberculosis	Added in GBD 2021
Diet low in vegetables	High fasting plasma glucose	Diabetes mellitus type 2	Added in GBD 2021
Diet low in vegetables	High systolic blood pressure	Hypertensive heart disease	Added in GBD 2021
Diet low in vegetables	High systolic blood pressure	Aortic Aneurysm	Added in GBD 2021
Diet low in vegetables	High fasting plasma glucose	Chronic kidney disease due to diabetes mellitus type 2	Added in GBD 2021
Diet low in vegetables	High fasting plasma glucose	Chronic kidney disease due to glomerulonephritis	Added in GBD 2021
Diet low in vegetables	High fasting plasma glucose	Chronic kidney disease due to hypertension	Added in GBD 2021
Diet low in vegetables	High fasting plasma glucose	Chronic kidney disease due to other and unspecified causes	Added in GBD 2021

Table S8. Status of mediated risk–outcome pairs considered for inclusion in GBD 2021: included in both GBD 2019 and GBD 2021, added in GBD 2021, or removed in GBD 2021			
**Risk Name**	**Mediator Name**	**Outcome Name**	**Status**
Diet low in vegetables	High systolic blood pressure	Chronic kidney disease due to diabetes mellitus type 2	Added in GBD 2021
Diet low in vegetables	High systolic blood pressure	Chronic kidney disease due to glomerulonephritis	Added in GBD 2021
Diet low in vegetables	High systolic blood pressure	Chronic kidney disease due to hypertension	Added in GBD 2021
Diet low in vegetables	High systolic blood pressure	Chronic kidney disease due to other and unspecified causes	Added in GBD 2021
Diet low in whole grains	High fasting plasma glucose	Lower extremity peripheral arterial disease	Added in GBD 2021
Diet low in whole grains	High fasting plasma glucose	Drug-susceptible tuberculosis	Added in GBD 2021
Diet low in whole grains	High fasting plasma glucose	Multidrug-resistant tuberculosis without extensive drug resistance	Added in GBD 2021
Diet low in whole grains	High fasting plasma glucose	Extensively drug-resistant tuberculosis	Added in GBD 2021
Diet low in whole grains	High fasting plasma glucose	Chronic kidney disease due to diabetes mellitus type 2	Added in GBD 2021
Diet low in whole grains	High fasting plasma glucose	Chronic kidney disease due to glomerulonephritis	Added in GBD 2021
Diet low in whole grains	High fasting plasma glucose	Chronic kidney disease due to hypertension	Added in GBD 2021
Diet low in whole grains	High fasting plasma glucose	Chronic kidney disease due to other and unspecified causes	Added in GBD 2021
High body-mass index in adults	High fasting plasma glucose	Lower extremity peripheral arterial disease	Added in GBD 2021
High body-mass index in adults	High fasting plasma glucose	Drug-susceptible tuberculosis	Added in GBD 2021
High body-mass index in adults	High fasting plasma glucose	Multidrug-resistant tuberculosis without extensive drug resistance	Added in GBD 2021
High body-mass index in adults	High fasting plasma glucose	Extensively drug-resistant tuberculosis	Added in GBD 2021
High body-mass index in adults	High systolic blood pressure	Aortic Aneurysm	Added in GBD 2021
High body-mass index in adults	High fasting plasma glucose	Chronic kidney disease due to diabetes mellitus type 2	Added in GBD 2021
High body-mass index in adults	High fasting plasma glucose	Chronic kidney disease due to glomerulonephritis	Added in GBD 2021
High body-mass index in adults	High fasting plasma glucose	Chronic kidney disease due to hypertension	Added in GBD 2021
High body-mass index in adults	High fasting plasma glucose	Chronic kidney disease due to other and unspecified causes	Added in GBD 2021
High body-mass index in adults	High systolic blood pressure	Chronic kidney disease due to diabetes mellitus type 2	Added in GBD 2021
High body-mass index in adults	High systolic blood pressure	Chronic kidney disease due to glomerulonephritis	Added in GBD 2021
High body-mass index in adults	High systolic blood pressure	Chronic kidney disease due to hypertension	Added in GBD 2021
High body-mass index in adults	High systolic blood pressure	Chronic kidney disease due to other and unspecified causes	Added in GBD 2021
Low physical activity	High fasting plasma glucose	Lower extremity peripheral arterial disease	Added in GBD 2021
Low physical activity	High fasting plasma glucose	Drug-susceptible tuberculosis	Added in GBD 2021
Low physical activity	High fasting plasma glucose	Multidrug-resistant tuberculosis without extensive drug resistance	Added in GBD 2021
Low physical activity	High fasting plasma glucose	Extensively drug-resistant tuberculosis	Added in GBD 2021
Low physical activity	High fasting plasma glucose	Chronic kidney disease due to diabetes mellitus type 2	Added in GBD 2021
Low physical activity	High fasting plasma glucose	Chronic kidney disease due to glomerulonephritis	Added in GBD 2021

Table S8. Status of mediated risk–outcome pairs considered for inclusion in GBD 2021: included in both GBD 2019 and GBD 2021, added in GBD 2021, or removed in GBD 2021

Risk Name	Mediator Name	Outcome Name	Status
Low physical activity	High fasting plasma glucose	Chronic kidney disease due to hypertension	Added in GBD 2021
Low physical activity	High fasting plasma glucose	Chronic kidney disease due to other and unspecified causes	Added in GBD 2021
Smoking	High fasting plasma glucose	Lower extremity peripheral arterial disease	Added in GBD 2021
Diet high in sodium	High systolic blood pressure	Chronic kidney disease due to diabetes mellitus type 1	Dropped in GBD 2021
Diet high in sodium	High systolic blood pressure	Other cardiomyopathy	Dropped in GBD 2021
Diet high in sodium	High systolic blood pressure	Rheumatic heart disease	Dropped in GBD 2021
Diet high in sodium	Kidney dysfunction	Chronic kidney disease due to diabetes mellitus type 1	Dropped in GBD 2021
Diet high in sodium	Kidney dysfunction	Chronic kidney disease due to diabetes mellitus type 2	Dropped in GBD 2021
Diet high in sodium	Kidney dysfunction	Chronic kidney disease due to glomerulonephritis	Dropped in GBD 2021
Diet high in sodium	Kidney dysfunction	Chronic kidney disease due to hypertension	Dropped in GBD 2021
Diet high in sodium	Kidney dysfunction	Chronic kidney disease due to other and unspecified causes	Dropped in GBD 2021
Diet high in sugar-sweetened beverages	High systolic blood pressure	Ischemic heart disease	Dropped in GBD 2021
Diet high in sugar-sweetened beverages	High fasting plasma glucose	Ischemic heart disease	Dropped in GBD 2021
Diet high in sugar-sweetened beverages	High LDL cholesterol	Ischemic heart disease	Dropped in GBD 2021
Diet high in sugar-sweetened beverages	High body-mass index in adults	Diabetes mellitus type 2	Dropped in GBD 2021
Diet high in sugar-sweetened beverages	High body-mass index in adults	Ischemic heart disease	Dropped in GBD 2021
Diet high in trans fatty acids	High systolic blood pressure	Ischemic heart disease	Dropped in GBD 2021
Diet low in fiber	Diet low in fruits	Ischemic heart disease	Dropped in GBD 2021
Diet low in fiber	Diet low in vegetables	Ischemic heart disease	Dropped in GBD 2021
Diet low in fiber	Diet low in whole grains	Ischemic heart disease	Dropped in GBD 2021
Diet low in nuts and seeds	High systolic blood pressure	Ischemic heart disease	Dropped in GBD 2021
Diet low in nuts and seeds	High fasting plasma glucose	Diabetes mellitus type 2	Dropped in GBD 2021
Diet low in nuts and seeds	High fasting plasma glucose	Ischemic heart disease	Dropped in GBD 2021
Diet low in polyunsaturated fatty acids	High systolic blood pressure	Ischemic heart disease	Dropped in GBD 2021
Diet low in polyunsaturated fatty acids	High fasting plasma glucose	Ischemic heart disease	Dropped in GBD 2021
Diet low in seafood omega-3 fatty acids	High systolic blood pressure	Ischemic heart disease	Dropped in GBD 2021
Diet low in vegetables	High fasting plasma glucose	Subarachnoid hemorrhage	Dropped in GBD 2021
High body-mass index in adults	High fasting plasma glucose	Subarachnoid hemorrhage	Dropped in GBD 2021
High body-mass index in adults	Kidney dysfunction	Chronic kidney disease due to diabetes mellitus type 2	Dropped in GBD 2021
High body-mass index in adults	Kidney dysfunction	Chronic kidney disease due to glomerulonephritis	Dropped in GBD 2021
High body-mass index in adults	Kidney dysfunction	Chronic kidney disease due to hypertension	Dropped in GBD 2021

Table S8. Status of mediated risk–outcome pairs considered for inclusion in GBD 2021: included in both GBD 2019 and GBD 2021, added in GBD 2021, or removed in GBD 2021

Risk Name	Mediator Name	Outcome Name	Status
High body-mass index in adults	Kidney dysfunction	Chronic kidney disease due to other and unspecified causes	Dropped in GBD 2021
High body-mass index in adults	Kidney dysfunction	Chronic kidney disease due to hypertension	Dropped in GBD 2021
High body-mass index in adults	Kidney dysfunction	Chronic kidney disease due to other and unspecified causes	Dropped in GBD 2021
High fasting plasma glucose	High systolic blood pressure	Intracerebral hemorrhage	Dropped in GBD 2021
High fasting plasma glucose	High systolic blood pressure	Ischemic heart disease	Dropped in GBD 2021
High fasting plasma glucose	High systolic blood pressure	Ischemic stroke	Dropped in GBD 2021
High fasting plasma glucose	High systolic blood pressure	Subarachnoid hemorrhage	Dropped in GBD 2021
High fasting plasma glucose	Kidney dysfunction	Chronic kidney disease due to diabetes mellitus type 1	Dropped in GBD 2021
High fasting plasma glucose	Kidney dysfunction	Chronic kidney disease due to diabetes mellitus type 2	Dropped in GBD 2021
High fasting plasma glucose	Kidney dysfunction	Chronic kidney disease due to glomerulonephritis	Dropped in GBD 2021
High fasting plasma glucose	Kidney dysfunction	Chronic kidney disease due to hypertension	Dropped in GBD 2021
High fasting plasma glucose	Kidney dysfunction	Chronic kidney disease due to other and unspecified causes	Dropped in GBD 2021
High LDL cholesterol	High systolic blood pressure	Ischemic heart disease	Dropped in GBD 2021
High LDL cholesterol	High systolic blood pressure	Ischemic stroke	Dropped in GBD 2021
High systolic blood pressure	Kidney dysfunction	Chronic kidney disease due to diabetes mellitus type 1	Dropped in GBD 2021
High systolic blood pressure	Kidney dysfunction	Chronic kidney disease due to diabetes mellitus type 2	Dropped in GBD 2021
High systolic blood pressure	Kidney dysfunction	Chronic kidney disease due to glomerulonephritis	Dropped in GBD 2021
High systolic blood pressure	Kidney dysfunction	Chronic kidney disease due to hypertension	Dropped in GBD 2021
High systolic blood pressure	Kidney dysfunction	Chronic kidney disease due to other and unspecified causes	Dropped in GBD 2021
Lead exposure in bone	High systolic blood pressure	Chronic kidney disease due to diabetes mellitus type 1	Dropped in GBD 2021
Lead exposure in bone	High systolic blood pressure	Other cardiomyopathy	Dropped in GBD 2021
Lead exposure in bone	High systolic blood pressure	Rheumatic heart disease	Dropped in GBD 2021
Lead exposure in bone	Kidney dysfunction	Chronic kidney disease due to diabetes mellitus type 1	Dropped in GBD 2021
Lead exposure in bone	Kidney dysfunction	Chronic kidney disease due to diabetes mellitus type 2	Dropped in GBD 2021
Lead exposure in bone	Kidney dysfunction	Chronic kidney disease due to glomerulonephritis	Dropped in GBD 2021
Lead exposure in bone	Kidney dysfunction	Chronic kidney disease due to hypertension	Dropped in GBD 2021
Lead exposure in bone	Kidney dysfunction	Chronic kidney disease due to other and unspecified causes	Dropped in GBD 2021
Smoking	Low bone mineral density	Cyclist road injuries	Dropped in GBD 2021
Smoking	Low bone mineral density	Falls	Dropped in GBD 2021
Smoking	Low bone mineral density	Motor vehicle road injuries	Dropped in GBD 2021
Smoking	Low bone mineral density	Non-venomous animal contact	Dropped in GBD 2021

Table S8. Status of mediated risk–outcome pairs considered for inclusion in GBD 2021: included in both GBD 2019 and GBD 2021, added in GBD 2021, or removed in GBD 2021

Risk Name	Mediator Name	Outcome Name	Status
Smoking	Low bone mineral density	Other exposure to mechanical forces	Dropped in GBD 2021
Smoking	Low bone mineral density	Other road injuries	Dropped in GBD 2021
Smoking	Low bone mineral density	Other transport injuries	Dropped in GBD 2021
Smoking	Low bone mineral density	Pedestrian road injuries	Dropped in GBD 2021
Smoking	Low bone mineral density	Physical violence by other means	Dropped in GBD 2021

Table S9. TMREL changes compared to GBD 2019.

Risk Name	2019 TMREL range	2021 TMREL range
Ambient ozone pollution	29.1–35.7 ppb	29.1–35.7 ppb
Ambient nitrogen dioxide pollution	---	4.6–6.2 ppb
Ambient particulate matter pollution	2.4–5.9 µg/m3	2.4–5.9 µg/m3
Household air pollution	2.4–5.9 µg/m3	2.4–5.9 µg/m3
Residential radon	0–0 Bq/m3	0–0 Bq/m3
High fasting plasma glucose	4.88–5.30 mmol/L	4.88–5.30 mmol/L
High systolic blood pressure	110–115 mmHg	105–115 mmHg
Low bone mineral density	1–1.3 g/cm2	1–1.3 g/cm2
Diet low in fruits	310–340 g/day	340–350 g/day
Diet low in vegetables	280–320 g/day	306–372 g/day
Diet low in whole grains	140–160 g/day	160–210 g/day
Diet low in nuts and seeds	10–19 g/day	19–24 g/day
Diet low in milk	360–500 g/day	280–340 g/day (males)<br>500–610 g/day (females)
Diet high in red meat	0 g/day	0–200 g/day
Diet high in processed meat	0 g/day	0 g/day
Diet high in sugar–sweetened beverages	0 g/day	0 g/day
Diet low in fiber	21–22 g/day	22–25 g/day
Diet low in seafood omega–3 fatty acids	430–470 mg/day	470–660 mg/day
Diet low in omega–6 polyunsaturated fatty acids	7–9% of total daily energy	9–10% of total daily energy
Diet high in trans fatty acids	0 % of daily energy	0–1.1 % of total daily energy
Diet high in sodium	1–5 g/day	1–5 g/day
Low physical activity	3000–4500 METs	3600–4400 METs
Diet low in calcium	1.06–1.1 g/d	0.72–0.86 g/day (males)<br>1.06–1.2 g/day (females)
Bone Mineral Density	Age group and µg/g (95% UI)<br>25–29: 0.022 (0.020–0.024)<br>30–34: 0.026 (0.024–0.028)<br>35–39: 0.030 (0.027–0.033)<br>40–44: 0.034 (0.031–0.037)<br>45–49: 0.038 (0.035–0.041)<br>50–54: 0.042 (0.038–0.046)<br>55–59: 0.046 (0.042–0.050)<br>60–64: 0.050 (0.045–0.054)<br>65–69: 0.054 (0.049–0.059)<br>70–74: 0.058 (0.053–0.063)<br>75–79: 0.062 (0.056–0.067)<br>80–84: 0.066 (0.060–0.072)<br>85–89: 0.070 (0.064–0.076)<br>90–94: 0.074 (0.067–0.080)	Age group and µg/g (95% UI)<br>25–29: 0.022 (0.020–0.024)<br>30–34: 0.026 (0.024–0.028)<br>35–39: 0.030 (0.027–0.033)<br>40–44: 0.034 (0.031–0.037)<br>45–49: 0.038 (0.035–0.041)<br>50–54: 0.042 (0.038–0.046)<br>55–59: 0.046 (0.042–0.050)<br>60–64: 0.050 (0.045–0.054)<br>65–69: 0.054 (0.049–0.059)<br>70–74: 0.058 (0.053–0.063)<br>75–79: 0.062 (0.056–0.067)<br>80–84: 0.066 (0.060–0.072)<br>85–89: 0.070 (0.064–0.076)<br>90–94: 0.074 (0.067–0.080)

Table S9. TMREL changes compared to GBD 2019.

Risk Name	2019 TMREL range	2021 TMREL range
Lead exposure in bone	2 µg/dL	Age group and µg/g (95% UI)<br>25–29:0.022 (0.020–0.024)<br>30–34:0.026 (0.024–0.028)<br>35–39:0.030 (0.027–0.033)<br>40–44:0.034 (0.031¬0.037)<br>45–49:0.038 (0.035–0.041)<br>50–54:0.042 (0.038¬0.046)<br>55–59:0.046 (0.042–0.050)<br>60–64:0.050 (0.045–0.054)<br>65–69:0.054 (0.049¬0.059)<br>70–74:0.058 (0.053–0.063)<br>75–79:0.062 (0.056–0.067)<br>80–84:0.066 (0.060–0.072)<br>85–89:0.070 (0.064–0.076)<br>90–94:0.074 (0.067–0.080)<br>95+:0.078 (0.071–0.085)
Lead exposure in blood	2 µg/dL	0.016 µg/dL
Diet low in legumes	90–100 g/day	100–110 g/day
High LDL cholesterol	0.7–1.3 mmol/L	0.9–1.4 mmol/L
High body–mass index in adults	20–25 kg/m2	20–21 kg/m2
High body–mass index	20–25 kg/m2	---
Iron deficiency	implied mean haemoglobin in absence of iron deficiency	implied mean haemoglobin in absence of iron deficiency
Kidney Dysfunction	ACR 30 mg/g or less and eGFR greater than 60ml/min/1.73m2	ACR 30 mg/g or less and eGFR greater than 60ml/min/1.73m2
Low birthweight and short gestation	38–40 weeks of gestation and 3500–4000 grams<br>38–40 weeks of gestation and 4000–4500 grams<br>40–42 weeks of gestation and 4000–4500 grams	38–40 weeks of gestation and 3500–4000 grams<br>38–40 weeks of gestation and 4000–4500 grams<br>40–42 weeks of gestation and 4000–4500 grams
Non–exclusive breastfeeding	low risk for children who recieved no nourishment	low risk for children who recieved no nourishment
Discontinued breastfeeding	lowest risk for children between 6–23 months	lowest risk for children between 6–23 months
No access to handwashing facility	having access to handwashing facility after any contact with excreta, including children's excreta	having access to a handwashing facility with soap (bar, liquid, or powder/detergent), water, and wash station (either permanent or mobile)
Unsafe sanitation	having access to a sanitation facility with sewer connection or septic tank	having access to a sanitation facility with sewer connection or septic tank
Unsafe water	having access to high–quality piped water	having access to high–quality piped water
Non–optimal temperature	temparture associated with the lowest mortality for all included causes	temparture associated with the lowest mortality for all included causes
Occupational risk factors	zero exposure	zero exposure
Secondhand smoke	zero exposure	zero exposure
Smoking	zero exposure	zero exposure
Unsafe sex	absence of disease transmission due to sexual contact	absence of disease transmission due to sexual contact
Vitamin A deficiency	zero exposure	zero exposure
Zinc deficency	zero exposure	zero exposure
Bullying victimisation	zero exposure	zero exposure
Chewing tobacco	everyone in population is a lifelong non–user	everyone in population is a lifelong non–user
Child growth failure	>–1 SD	>–1 SD
Childhood sexual abuse	zero exposure	zero exposure
Drug use	zero exposure	zero exposure

Table S9. TMREL changes compared to GBD 2019.		
**Risk Name**	**2019 TMREL range**	**2021 TMREL range**
High alcohol use	exposure that minimises your risk of suffering burden from any given cause related to alcohol	exposure that minimises your risk of suffering burden from any given cause related to alcohol
Intimate Partner Violence	zero exposure	zero exposure
