# Supplementary material for: Global burden and strength of evidence for 88 risk factors in 204 countries and 811 subnational locations, 1990–2021: a systematic analysis for the Global Burden of Disease Study 2021
Source: Lancet. 2024 May 18;403(10440):2162–203. doi: 10.1016/S0140-6736(24)00933-4 (PMC11120204; doi:10.1016/S0140-6736(24)00933-4)

THE LANCET

Supplementary appendix 2

This appendix formed part of the original submission and has been peer reviewed. We post it as supplied by the authors.

Supplement to: GBD 2021 Risk Factors Collaborators. Global burden and strength of evidence for 88 risk factors in 204 countries and 811 subnational locations, 1990–2021: a systematic analysis for the Global Burden of Disease Study 2021. *Lancet* 2024; **403:** 2162–203.

Appendix 2: supplementary results appendix to “Global burden and strength of evidence for 88 risk factors in 204 countries and 811 subnational locations, 1990–2021: a systematic analysis for the Global Burden of Disease Study 2021”

This supplement provides additional figures and tables containing more detailed results for “Global burden and strength of evidence for 88 risk factors in 204 countries and 811 subnational locations, 1990–2021: a systematic analysis for the Global Burden of Disease Study 2021”

The results are available in an interactive data downloading tool in the Global Health Data Exchange (GHDx): vizhub.healthdata.org/gbd-results . The tool contains the complete set of results from all GBD 2021 papers. Specialised tables from the papers will be available as separate entries in the GHDx,

List of supplementary results tables and figures

Tables

Supplementary results tables can be accessed at https://ghdx.healthdata.org/record/ihme-data/gbd-2021-burden-by-risk-1990-2021

Table S1. Death PAFs, all-age death counts, all-age death rates (per 100 000), death, age-standardised death rate (per 100 000), DALY PAFs, all-age DALYs rate (per 100 000) age-standardised DALY rate (per 100 000) for each risk factor and outcome for both sexes combined in 1990, 2000, 2010, and 2021, by geography. Percentage change for 1990-2021 and 2000-2021. Data in parenthesis are 95% uncertainty intervals. PAFs=population attributable fractions. DALYs=disability-adjusted life-years.

Table S2. Death PAFs, all-age death counts, all-age death rates (per 100 000), death, age-standardised death rate (per 100 000), DALY PAFs, all-age DALYs rate (per 100 000) age-standardised DALY rate (per 100 000) for each risk factor and outcome for both sexes combined in 1990, 2000, 2010, and 2021, excluding one- and two-star risk-outcome pairs, by geography. Percentage change for 1990-2021 and 2000-2021. Data in parenthesis are 95% uncertainty intervals. PAFs=population attributable fractions. DALYs=disability-adjusted life-years.

Table S3. Age-standardised Summary Exposure Values (SEVs) for risk factors and percentage change, by sex, 1990, 2000, 2010 and 2021, and geography. Data in parentheses are 95% uncertainty intervals.

Table S4. All-age PAFs attributable to the joint distribution of all risk factors, both sexes combined for each GBD Level 1, Level 2, and Level 3 cause and all causes in 2021, by geography.

Table S5. Relative risks used by age and sex for each outcome for unsafe water, unsafe sanitation, iron deficiency, high alcohol use, low bone mineral density, occupational risks, non-exclusive breastfeeding, discontinued breastfeeding, intimate partner violence, childhood sexual abuse, chewing tobacco, low birth weight and short gestation, particulate matter pollution, lead exposure in blood. Table only includes relative risks not available in the Burden of Proof visualization tool (https://vizhub.healthdata.org/burden-of-proof/).

Table S6. Risk-outcome scores and global DALY counts for all risk-outcome pairs submitted to the burden of proof analysis, 2021. Data in parenthesis are 95% uncertainty intervals. DALYs=disability-adjusted life-years.

Figures

Figure S1a-b. Leading 25 Level 3 risk factors by sex by attributable DALYs, percentage of total DALYs (2000 and 2021), and percentage change in attributable DALY counts and age-standardised DALY rates from 2000 to 2021. Each column displays the top 25 risks in descending order for the specified year. Risk factors are connected by lines between time periods; solid lines represent an increase or lateral shift in ranking, dashed lines represented a decrease in rank. DALY=disability-adjusted life-year....3

Figure S2a-e. Leading 25 Level 3 risk factors by age by attributable DALYs, percentage of total DALYs (2000 and 2021), and percentage change in attributable DALY counts and age-standardised DALY rates from 2000 to 2021. Each column displays the top 25 risks in descending order for the specified year. Risk factors are connected by lines between time periods; solid lines represent an increase or lateral shift in ranking, dashed lines represented a decrease in rank. DALY=disability-adjusted life-year....5

Figure S3. Age-standardised DALY rate (per 100 000) for all-causes attributable to (A) all GBD risk-outcome pairs (A) and GBD risk-outcome pairs, excluding one- and two-star associations (B), 2021. One-star and two-star associations are those that are either or both weakly associated and lacking strong evidence, based on burden risk function of proof analysis. DALY=disability-adjusted life year....10

Figure S4. Global DALY counts attributable to Level 1 risk factors for all GBD risk–outcome pairs (A) versus GBD risk–outcome pairs excluding one- and two-star associations (B), 2021. One-star and two-star associations are those that are either or both weakly associated and lacking strong evidence, based on burden of proof risk function analysis. DALY=disability-adjusted life year....11

Figure S1a. Leading 25 Level 3 risk factors for females by attributable DALYs, percentage of total DALYs (2000 and 2021), and percentage change in attributable DALY counts and age-standardised DALY rates from 2000 to 2021. DALY = disability-adjusted life-year. DALY = disability-adjusted life-year.

Leading risks 2000	Percent DALYs 2000
1 Particulate matter pollution	10.0 (7.8 to 11.8)
2 Child growth failure	9.6 (6.5 to 11.6)
3 Low birth weight and short gestation	8.1 (7.5 to 8.8)
4 High systolic blood pressure	6.3 (5.2 to 7.4)
5 Unsafe water source	4.1 (2.3 to 5.5)
6 Unsafe sex	3.5 (2.8 to 4.3)
7 Unsafe sanitation	3.4 (2.7 to 4.2)
8 High fasting plasma glucose	3.2 (2.8 to 3.6)
9 High body-mass index	2.8 (1.2 to 4.5)
10 No access to handwashing facility	2.3 (-0.5 to 5.0)
11 High LDL cholesterol	2.3 (1.4 to 3.2)
12 Kidney dysfunction	2.2 (1.9 to 2.4)
13 Smoking	2.0 (1.5 to 2.4)
14 Iron deficiency	1.9 (1.4 to 2.4)
15 Secondhand smoke	1.7 (0.9 to 2.6)
16 Diet low in fruits	1.2 (0.5 to 1.8)
17 Suboptimal breastfeeding	1.2 (0.9 to 1.5)
18 Diet low in whole grains	1.0 (0.4 to 1.5)
19 Low temperature	1.0 (0.8 to 1.1)
20 Diet high in sodium	1.0 (0.2 to 2.3)
21 Lead exposure	0.9 (0.0 to 1.7)
22 High alcohol use	0.9 (0.7 to 1.1)
23 Intimate partner violence	0.7 (0.5 to 1.0)
24 Diet low in vegetables	0.6 (0.4 to 0.8)
25 Diet low in fiber	0.6 (0.2 to 0.9)
26 Drug use	0.5 (0.4 to 0.6)
30 Low physical activity	0.5 (0.2 to 0.7)
32 Low bone mineral density	0.5 (0.4 to 0.5)

Leading risks 2021	Percent DALYs 2021	Percent change number of DALYs 2000-2021	Percent change age-standardised rate of DALYs 2000-2021
1 High systolic blood pressure	7.6 (6.1 to 8.9)	28.4 (19.2 to 37.1)	-27.8 (-33.1 to -22.9)
2 Particulate matter pollution	7.5 (6.1 to 8.8)	-20.1 (-29.4 to -7.5)	-44.0 (-49.9 to -36.1)
3 High fasting plasma glucose	5.5 (4.8 to 6.2)	86.5 (77.4 to 96.0)	7.8 (2.5 to 13.6)
4 Low birth weight and short gestation	5.1 (4.4 to 5.8)	-32.5 (-40.8 to -23.1)	-33.5 (-41.6 to -24.1)
5 High body-mass index	5.1 (2.1 to 7.8)	93.2 (82.8 to 103.5)	13.7 (7.4 to 20.2)
6 Kidney dysfunction	2.9 (2.6 to 3.3)	45.7 (37.0 to 54.9)	-14.8 (-19.9 to -9.3)
7 High LDL cholesterol	2.6 (1.5 to 3.7)	23.1 (15.2 to 30.6)	-29.2 (-33.6 to -24.8)
8 Child growth failure	2.6 (1.4 to 3.6)	-71.0 (-78.9 to -64.1)	-72.8 (-80.2 to -66.1)
9 Unsafe sex	2.2 (2.0 to 2.4)	-33.3 (-43.2 to -18.8)	-51.5 (-58.1 to -41.8)
10 Smoking	1.9 (1.5 to 2.4)	2.9 (-4.4 to 10.7)	-40.3 (-44.6 to -35.7)
11 Iron deficiency	1.9 (1.4 to 2.4)	7.5 (2.5 to 12.3)	-14.0 (-17.9 to -10.1)
12 Unsafe water source	1.5 (0.7 to 2.2)	-60.2 (-69.1 to -52.4)	-66.8 (-73.7 to -60.3)
13 Diet low in fruits	1.4 (0.6 to 2.1)	23.3 (13.7 to 39.8)	-27.1 (-32.7 to -18.3)
14 Secondhand smoke	1.3 (0.7 to 2.0)	-18.9 (-26.6 to -8.6)	-48.3 (-52.8 to -42.8)
15 Diet low in whole grains	1.2 (0.5 to 1.8)	26.8 (19.3 to 35.1)	-26.0 (-30.4 to -21.2)
16 Unsafe sanitation	1.1 (0.8 to 1.5)	-64.1 (-71.7 to -56.9)	-69.8 (-75.8 to -63.3)
17 Diet high in sodium	1.1 (0.2 to 2.7)	20.2 (-15.0 to 35.8)	-31.6 (-51.0 to -22.9)
18 Lead exposure	1.0 (-0.0 to 2.1)	29.2 (2.2 to 42.0)	-24.0 (-30.4 to -17.0)
19 Low temperature	0.9 (0.8 to 1.0)	-1.8 (-13.0 to 9.7)	-44.1 (-49.4 to -38.7)
20 High alcohol use	0.8 (0.7 to 1.0)	4.9 (-2.1 to 12.5)	-32.3 (-36.8 to -27.3)
21 No access to handwashing facility	0.8 (-0.2 to 1.8)	-61.6 (-70.4 to -52.5)	-66.9 (-74.4 to -58.7)
22 Low physical activity	0.7 (0.3 to 1.1)	61.3 (47.6 to 77.6)	-9.0 (-17.0 to 1.1)
23 Diet low in vegetables	0.7 (0.4 to 1.0)	26.8 (16.3 to 42.0)	-26.1 (-32.4 to -17.6)
24 Low bone mineral density	0.7 (0.6 to 0.8)	61.5 (55.5 to 68.0)	-9.6 (-12.9 to -6.2)
25 Drug use	0.7 (0.6 to 0.8)	39.5 (29.5 to 49.2)	2.1 (-5.1 to 9.4)
27 Intimate partner violence	0.5 (0.3 to 0.8)	-21.7 (-44.0 to -2.2)	-41.6 (-57.5 to -27.4)
30 Diet low in fiber	0.5 (0.2 to 0.8)	-4.5 (-13.1 to 8.2)	-42.8 (-48.1 to -35.8)
35 Suboptimal breastfeeding	0.3 (0.2 to 0.4)	-71.5 (-76.7 to -65.5)	-71.7 (-76.9 to -65.8)

Legend:
Environmental/occupational risks
Behavioural risks
Metabolic risks

Figure S1b. Leading 25 Level 3 risk factors for males by attributable DALYs, percentage of total DALYs (2000 and 2021), and percentage change in attributable DALY counts and age-standardised DALY rates from 2000 to 2021. DALY = disability-adjusted life-year. DALY = disability-adjusted life-year.

Leading risks 2000	Percent DALYs 2000
1 Particulate matter pollution	11.0 (9.0 to 12.7)
2 Low birth weight and short gestation	9.7 (9.0 to 10.3)
3 Child growth failure	9.0 (6.2 to 10.8)
4 Smoking	8.8 (7.5 to 10.1)
5 High systolic blood pressure	6.4 (5.2 to 7.4)
6 Unsafe water source	3.9 (2.1 to 5.2)
7 High alcohol use	3.8 (3.0 to 4.8)
8 Unsafe sanitation	3.3 (2.5 to 4.1)
9 High fasting plasma glucose	3.1 (2.7 to 3.4)
10 High LDL cholesterol	2.9 (1.9 to 3.9)
11 Occupational injuries	2.6 (2.4 to 2.8)
12 No access to handwashing facility	2.3 (-0.5 to 4.8)
13 Kidney dysfunction	2.2 (2.0 to 2.5)
14 High body-mass index	2.2 (1.0 to 3.4)
15 Unsafe sex	1.7 (1.4 to 2.2)
16 Diet low in fruits	1.5 (0.5 to 2.2)
17 Diet high in sodium	1.4 (0.4 to 3.1)
18 Secondhand smoke	1.4 (0.7 to 2.2)
19 Diet low in whole grains	1.3 (0.6 to 2.0)
20 Suboptimal breastfeeding	1.2 (0.9 to 1.6)
21 Lead exposure	1.1 (-0.0 to 2.2)
22 Drug use	1.0 (0.9 to 1.2)
23 Low temperature	0.8 (0.7 to 0.9)
24 Iron deficiency	0.7 (0.5 to 1.0)
25 Diet low in fiber	0.7 (0.2 to 1.1)
26 Diet low in vegetables	0.7 (0.4 to 1.0)
27 Diet low in nuts and seeds	0.7 (0.2 to 1.0)
29 Diet low in omega-6 polyunsaturated fatty acids	0.6 (-2.0 to 2.2)

Leading risks 2021	Percent DALYs 2021	Percent change number of DALYs 2000-2021	Percent change age-standardised rate of DALYs 2000-2021
1 Smoking	9.0 (7.6 to 10.6)	12.4 (3.3 to 23.0)	-34.0 (-39.3 to -27.9)
2 Particulate matter pollution	8.5 (7.1 to 9.9)	-14.9 (-23.7 to -4.1)	-40.4 (-46.0 to -33.9)
3 High systolic blood pressure	8.1 (6.7 to 9.4)	39.4 (29.5 to 50.6)	-21.0 (-26.5 to -15.0)
4 Low birth weight and short gestation	5.9 (5.2 to 6.7)	-32.4 (-41.4 to -21.6)	-32.6 (-41.7 to -21.7)
5 High fasting plasma glucose	5.3 (4.7 to 5.9)	89.6 (79.3 to 100.0)	7.6 (2.0 to 13.7)
6 High body-mass index	3.9 (1.8 to 6.0)	100.2 (89.6 to 111.9)	18.8 (12.2 to 25.9)
7 High alcohol use	3.9 (3.2 to 4.8)	13.9 (3.0 to 23.5)	-24.4 (-31.4 to -18.0)
8 High LDL cholesterol	3.4 (2.2 to 4.7)	29.7 (21.7 to 39.2)	-23.5 (-27.9 to -18.4)
9 Kidney dysfunction	3.0 (2.7 to 3.4)	52.8 (44.2 to 62.8)	-10.3 (-15.3 to -4.7)
10 Child growth failure	2.6 (1.4 to 3.5)	-68.6 (-76.8 to -60.4)	-70.3 (-78.0 to -62.5)
11 Occupational injuries	1.8 (1.6 to 1.9)	-24.5 (-30.5 to -18.9)	-43.1 (-47.7 to -38.9)
12 Diet high in sodium	1.7 (0.4 to 3.7)	31.9 (6.1 to 50.3)	-24.3 (-38.4 to -13.5)
13 Diet low in fruits	1.6 (0.6 to 2.5)	22.0 (13.5 to 33.4)	-26.3 (-31.3 to -20.2)
14 Diet low in whole grains	1.6 (0.7 to 2.4)	32.1 (24.1 to 40.7)	-21.4 (-25.7 to -16.2)
15 Unsafe water source	1.4 (0.7 to 2.0)	-60.1 (-68.0 to -51.3)	-66.0 (-72.8 to -58.3)
16 Lead exposure	1.3 (-0.1 to 2.6)	28.5 (7.7 to 45.0)	-23.8 (-29.8 to -16.3)
17 Drug use	1.2 (1.1 to 1.3)	27.5 (20.0 to 35.0)	-7.4 (-12.8 to -2.1)
18 Secondhand smoke	1.1 (0.6 to 1.7)	-12.9 (-20.5 to -1.6)	-41.7 (-46.3 to -35.7)
19 Unsafe sanitation	1.1 (0.8 to 1.5)	-63.6 (-70.5 to -55.5)	-68.9 (-74.8 to -61.7)
20 Unsafe sex	1.0 (0.9 to 1.1)	-38.0 (-47.5 to -22.6)	-54.1 (-60.8 to -43.2)
21 Low temperature	0.9 (0.7 to 1.0)	22.2 (8.4 to 39.7)	-35.3 (-41.4 to -28.3)
22 No access to handwashing facility	0.8 (-0.2 to 1.8)	-59.4 (-69.2 to -50.7)	-64.7 (-73.4 to -56.7)
23 Diet low in vegetables	0.7 (0.4 to 1.0)	18.0 (8.9 to 32.2)	-30.6 (-35.8 to -23.5)
24 Diet low in omega-6 polyunsaturated fatty acids	0.7 (-2.4 to 2.7)	34.8 (22.8 to 43.4)	-19.1 (-24.9 to -13.1)
25 Diet low in nuts and seeds	0.7 (0.2 to 1.1)	12.1 (3.9 to 20.9)	-32.1 (-37.0 to -27.0)
26 Diet low in fiber	0.7 (0.2 to 1.0)	1.8 (-6.9 to 12.8)	-37.5 (-42.8 to -30.9)
27 Iron deficiency	0.6 (0.4 to 0.8)	-11.4 (-16.8 to -6.0)	-27.1 (-31.3 to -22.7)
38 Suboptimal breastfeeding	0.3 (0.2 to 0.4)	-71.1 (-76.4 to -65.0)	-71.2 (-76.5 to -65.2)

Legend:

Environmental/occupational risks
Behavioural risks
Metabolic risks

Figure S2a. Leading 25* Level 3 risk factors for ages under 5 by attributable DALYs, percentage of total DALYs (2000 and 2021), and percentage change in attributable DALY counts and age-standardised DALY rates from 2000 to 2021.

DALY = disability-adjusted life-year.

Leading risks 2000	Percent DALYs 2000	Leading risks 2021	Percent DALYs 2021	Percent change number of DALYs 2000-2021	Percent change age-standardised rate of DALYs 2000-2021
1 Child growth failure	28.2 (19.7 to 33.1)	1 Low birth weight and short gestation	33.5 (31.7 to 34.8)	-35.5 (-44.3 to -25.1)	-33.0 (-41.6 to -22.8)
2 Low birth weight and short gestation	27.0 (25.9 to 28.0)	2 Child growth failure	15.9 (8.3 to 21.6)	-70.7 (-78.6 to -63.1)	-71.5 (-78.8 to -64.4)
3 Particulate matter pollution	14.7 (10.1 to 18.5)	3 Particulate matter pollution	14.4 (11.3 to 17.1)	-49.2 (-57.4 to -36.2)	-41.9 (-47.2 to -35.6)
4 Unsafe water source	9.0 (5.3 to 11.8)	4 Unsafe water source	5.2 (2.8 to 7.2)	-70.2 (-77.1 to -61.8)	-66.3 (-72.0 to -60.2)
5 Unsafe sanitation	7.6 (6.1 to 9.0)	5 Unsafe sanitation	4.1 (3.1 to 5.3)	-71.9 (-78.4 to -64.0)	-69.2 (-74.4 to -63.2)
6 No access to handwashing facility	5.6 (-1.4 to 11.9)	6 No access to handwashing facility	3.4 (-1.0 to 7.3)	-68.3 (-76.3 to -59.2)	-65.7 (-73.4 to -57.8)
7 Suboptimal breastfeeding	3.8 (2.8 to 4.6)	7 Suboptimal breastfeeding	2.1 (1.6 to 2.6)	-71.3 (-75.7 to -66.2)	-71.4 (-75.8 to -66.4)
8 Secondhand smoke	1.6 (0.5 to 2.7)	8 Iron deficiency	1.3 (0.8 to 1.9)	-11.4 (-14.9 to -8.1)	-18.1 (-21.2 to -15.2)
9 Vitamin A deficiency	1.5 (-3.7 to 5.6)	9 Secondhand smoke	0.8 (0.3 to 1.3)	-74.4 (-78.6 to -69.7)	-45.3 (-48.9 to -40.3)
10 Iron deficiency	0.8 (0.5 to 1.1)	10 High temperature	0.6 (0.2 to 1.0)	-51.4 (-61.0 to -40.4)	-19.8 (-29.5 to -7.9)
11 High temperature	0.7 (0.3 to 1.1)	11 Vitamin A deficiency	0.5 (-1.0 to 1.6)	-83.6 (-93.9 to -63.0)	-81.6 (-112.3 to -45.8)
12 Low temperature	0.5 (0.3 to 0.8)	12 Low temperature	0.2 (0.1 to 0.4)	-76.7 (-84.9 to -71.2)	-39.5 (-44.2 to -34.5)
13 Kidney dysfunction	0.1 (0.1 to 0.2)	13 Kidney dysfunction	0.2 (0.1 to 0.2)	-32.3 (-44.5 to -10.7)	-12.4 (-16.5 to -7.9)
14 Zinc deficiency	0.1 (-0.2 to 0.5)	14 Lead exposure	0.1 (0.0 to 0.1)	-34.3 (-39.9 to -30.3)	-23.9 (-28.9 to -18.4)
15 Lead exposure	0.0 (0.0 to 0.1)	15 High fasting plasma glucose	0.0 (0.0 to 0.0)	-20.9 (-39.5 to 1.2)	7.9 (3.3 to 12.9)
16 High fasting plasma glucose	0.0 (0.0 to 0.0)	16 Zinc deficiency	0.0 (-0.1 to 0.2)	-84.9 (-92.4 to -78.1)	-86.0 (-93.0 to -79.8)
17 High body-mass index	0.0 (0.0 to 0.0)	17 High body-mass index	0.0 (0.0 to 0.0)	14.6 (-2.3 to 31.0)	15.7 (9.9 to 21.7)
18 Nitrogen dioxide pollution	0.0 (0.0 to 0.0)	18 Nitrogen dioxide pollution	0.0 (0.0 to 0.0)	-27.3 (-56.4 to -14.4)	-30.4 (-60.9 to -18.8)
19 High alcohol use	0.0 (0.0 to 0.0)	19 High alcohol use	0.0 (0.0 to 0.0)	1.0 (-8.2 to 10.3)	-25.8 (-32.0 to -20.4)

*For ages under 5, GBD does not estimate 25 level 3 risk factors.

Legend:

Environmental/occupational risks
Behavioural risks
Metabolic risks

Figure S2b. Leading 25* Level 3 risk factors for ages 5-14 years by attributable DALYs, percentage of total DALYs (2000 and 2021), and percentage change in attributable DALY counts and age-standardised DALY rates from 2000 to 2021.
DALY = disability-adjusted life-year.

Leading risks 2000	Percent DALYs 2000	Leading risks 2021	Percent DALYs 2021	Percent change number of DALYs 2000-2021	Percent change age-standardised rate of DALYs 2000-2021
1 Iron deficiency	4.5 (3.4 to 5.8)	1 Iron deficiency	5.8 (4.4 to 7.4)	-0.1 (-8.2 to 9.2)	-18.1 (-21.2 to -15.2)
2 Unsafe water source	4.1 (2.0 to 6.1)	2 Unsafe water source	2.9 (1.5 to 4.3)	-45.5 (-58.4 to -31.1)	-66.3 (-72.0 to -60.2)
3 Unsafe sanitation	3.5 (2.3 to 4.8)	3 Low birth weight and short gestation	2.7 (2.1 to 3.2)	27.9 (21.9 to 33.7)	-33.0 (-41.6 to -22.8)
4 No access to handwashing facility	2.0 (-0.3 to 4.1)	4 Unsafe sanitation	2.2 (1.6 to 3.0)	-51.3 (-62.9 to -39.4)	-69.2 (-74.4 to -63.2)
5 Particulate matter pollution	1.8 (0.3 to 2.9)	5 No access to handwashing facility	1.3 (-0.2 to 2.7)	-48.2 (-60.4 to -36.0)	-65.7 (-73.4 to -57.8)
6 Low birth weight and short gestation	1.6 (1.3 to 2.0)	6 Particulate matter pollution	1.1 (0.2 to 1.9)	-50.9 (-57.8 to -43.4)	-41.9 (-47.2 to -35.6)
7 Child growth failure	1.1 (0.9 to 1.4)	7 Bullying victimization	0.7 (0.3 to 1.3)	14.7 (9.0 to 20.5)	12.9 (7.2 to 20.4)
8 High temperature	0.6 (0.4 to 0.8)	8 Kidney dysfunction	0.6 (0.5 to 0.7)	-12.7 (-21.4 to 2.1)	-12.4 (-16.5 to -7.9)
9 Kidney dysfunction	0.5 (0.4 to 0.6)	9 Child growth failure	0.6 (0.5 to 0.7)	-61.9 (-69.2 to -54.1)	-71.5 (-78.8 to -64.4)
10 Secondhand smoke	0.5 (0.2 to 0.9)	10 High temperature	0.5 (0.3 to 0.7)	-35.6 (-43.9 to -26.3)	-19.8 (-29.5 to -7.9)
11 Vitamin A deficiency	0.5 (0.3 to 0.6)	11 Lead exposure	0.4 (0.2 to 0.8)	-23.9 (-30.0 to -19.2)	-23.9 (-28.9 to -18.4)
12 Bullying victimization	0.5 (0.2 to 0.8)	12 Vitamin A deficiency	0.4 (0.3 to 0.5)	-37.2 (-45.7 to -25.9)	-81.6 (-112.3 to -45.8)
13 Lead exposure	0.5 (0.2 to 0.8)	13 Unsafe sex	0.3 (0.3 to 0.4)	41.1 (22.1 to 63.4)	-52.4 (-58.9 to -42.3)
14 High fasting plasma glucose	0.2 (0.2 to 0.2)	14 Secondhand smoke	0.3 (0.1 to 0.5)	-57.3 (-61.8 to -52.1)	-45.3 (-48.9 to -40.3)
15 Unsafe sex	0.2 (0.2 to 0.2)	15 High fasting plasma glucose	0.2 (0.2 to 0.3)	-10.3 (-22.2 to 0.3)	7.9 (3.3 to 12.9)
16 High alcohol use	0.1 (0.1 to 0.2)	16 High body-mass index	0.2 (0.1 to 0.4)	52.9 (41.6 to 64.7)	15.7 (9.9 to 21.7)
17 High body-mass index	0.1 (0.0 to 0.2)	17 High alcohol use	0.1 (0.1 to 0.2)	-3.5 (-12.3 to 5.9)	-25.8 (-32.0 to -20.4)
18 Nitrogen dioxide pollution	0.1 (-0.1 to 0.3)	18 Nitrogen dioxide pollution	0.1 (-0.1 to 0.4)	-18.8 (-54.5 to -5.6)	-30.4 (-60.9 to -18.8)
19 Childhood sexual abuse	0.0 (0.0 to 0.0)	19 Childhood sexual abuse	0.0 (0.0 to 0.1)	55.9 (29.8 to 80.3)	-16.0 (-23.4 to -7.1)
20 Drug use	0.0 (0.0 to 0.0)	20 Drug use	0.0 (0.0 to 0.0)	-2.5 (-12.1 to 3.7)	-4.6 (-10.1 to 0.8)
21 Low temperature	-0.8 (-1.0 to -0.6)	21 Low temperature	-0.4 (-0.5 to -0.3)	-62.4 (-66.1 to -58.3)	-39.5 (-44.2 to -34.5)

*For ages 5-14 years, GBD does not estimate 25 level 3 risk factors.

Legend:
Environmental/occupational risks
Behavioural risks
Metabolic risks

Figure S2c. Leading 25 Level 3 risk factors for ages 15-49 years by attributable DALYs, percentage of total DALYs (2000 and 2021), and percentage change in attributable DALY counts and age-standardised DALY rates from 2000 to 2021.
DALY = disability-adjusted life-year.

Leading risks 2000	Percent DALYs 2000	Leading risks 2021	Percent DALYs 2021	Percent change number of DALYs 2000-2021	Percent change age-standardised rate of DALYs 2000-2021
1 Unsafe sex	7.2 (5.6 to 9.2)	1 High alcohol use	3.8 (3.0 to 4.6)	-6.7 (-13.0 to -0.6)	-25.8 (-32.0 to -20.4)
2 Occupational injuries	4.6 (4.2 to 5.0)	2 Unsafe sex	3.6 (3.1 to 4.2)	-44.3 (-52.4 to -31.3)	-52.4 (-58.9 to -42.3)
3 High alcohol use	4.5 (3.5 to 5.9)	3 High body-mass index	3.2 (1.4 to 4.9)	75.2 (64.1 to 86.9)	15.7 (9.9 to 21.7)
4 Smoking	4.4 (3.5 to 5.2)	4 Smoking	3.1 (2.6 to 3.8)	-19.3 (-24.7 to -13.9)	-34.8 (-39.2 to -29.7)
5 Particulate matter pollution	3.3 (2.7 to 3.9)	5 Occupational injuries	2.9 (2.7 to 3.2)	-28.9 (-33.7 to -24.2)	-43.6 (-47.5 to -39.8)
6 High systolic blood pressure	2.7 (2.1 to 3.4)	6 High systolic blood pressure	2.8 (2.2 to 3.5)	14.6 (5.9 to 25.0)	-24.3 (-28.4 to -20.0)
7 Drug use	2.1 (1.9 to 2.4)	7 Particulate matter pollution	2.8 (2.2 to 3.3)	-6.6 (-13.7 to 1.1)	-41.9 (-47.2 to -35.6)
8 High LDL cholesterol	2.1 (1.5 to 2.6)	8 High fasting plasma glucose	2.6 (2.4 to 2.9)	67.1 (57.5 to 76.4)	7.9 (3.3 to 12.9)
9 Iron deficiency	2.1 (1.6 to 2.6)	9 Drug use	2.2 (1.9 to 2.5)	16.7 (10.3 to 23.0)	-4.6 (-10.1 to 0.8)
10 High body-mass index	2.0 (0.9 to 3.2)	10 High LDL cholesterol	2.1 (1.5 to 2.6)	10.7 (3.9 to 17.7)	-26.1 (-29.6 to -22.4)
11 High fasting plasma glucose	1.8 (1.6 to 1.9)	11 Kidney dysfunction	1.8 (1.6 to 2.0)	24.9 (17.0 to 32.9)	-12.4 (-16.5 to -7.9)
12 Kidney dysfunction	1.6 (1.4 to 1.8)	12 Iron deficiency	1.8 (1.4 to 2.2)	-3.9 (-9.6 to 1.4)	-18.1 (-21.2 to -15.2)
13 Diet low in fruits	1.3 (0.4 to 2.0)	13 Diet low in fruits	1.2 (0.4 to 1.9)	3.8 (-2.5 to 12.1)	-26.6 (-30.9 to -20.5)
14 Unsafe water source	1.1 (0.6 to 1.7)	14 Occupational ergonomic factors	1.0 (0.8 to 1.3)	12.5 (9.1 to 16.1)	-12.9 (-15.7 to -10.0)
15 Occupational ergonomic factors	1.0 (0.8 to 1.3)	15 Diet low in whole grains	1.0 (0.5 to 1.5)	12.3 (4.9 to 19.0)	-23.3 (-26.9 to -19.5)
16 Diet low in whole grains	1.0 (0.5 to 1.4)	16 Low birth weight and short gestation	0.8 (0.7 to 1.0)	63.2 (55.9 to 70.6)	-33.0 (-41.6 to -22.8)
17 Intimate partner violence	1.0 (0.6 to 1.3)	17 Unsafe water source	0.7 (0.4 to 1.1)	-29.1 (-41.9 to -14.3)	-66.3 (-72.0 to -60.2)
18 Unsafe sanitation	1.0 (0.6 to 1.5)	18 Secondhand smoke	0.7 (0.4 to 1.0)	-9.3 (-16.1 to -3.1)	-45.3 (-48.9 to -40.3)
19 Secondhand smoke	0.8 (0.5 to 1.2)	19 Intimate partner violence	0.6 (0.4 to 0.9)	-29.8 (-48.5 to -11.8)	-41.5 (-57.5 to -27.4)
20 Diet low in fiber	0.8 (0.2 to 1.2)	20 Bullying victimization	0.6 (0.3 to 1.1)	35.6 (28.2 to 45.8)	12.9 (7.2 to 20.4)
21 Lead exposure	0.6 (0.1 to 1.2)	21 Diet low in fiber	0.6 (0.2 to 0.9)	-13.7 (-21.3 to -3.0)	-39.8 (-43.9 to -34.3)
22 Diet high in sodium	0.6 (0.2 to 1.5)	22 Unsafe sanitation	0.5 (0.4 to 0.8)	-37.3 (-48.3 to -24.3)	-69.2 (-74.4 to -63.2)
23 Diet low in seafood omega-3 fatty acids	0.6 (0.1 to 0.9)	23 Diet low in vegetables	0.5 (0.3 to 0.7)	9.0 (0.4 to 20.5)	-28.5 (-33.4 to -21.3)
24 Low birth weight and short gestation	0.6 (0.5 to 0.7)	24 Diet low in nuts and seeds	0.5 (0.2 to 0.8)	-1.8 (-9.6 to 5.4)	-34.0 (-37.9 to -30.2)
25 Diet low in nuts and seeds	0.6 (0.2 to 0.9)	25 Diet high in sodium	0.5 (0.1 to 1.3)	-7.8 (-29.7 to 2.9)	-26.8 (-40.9 to -19.1)
26 Diet low in vegetables	0.5 (0.3 to 0.8)	26 Lead exposure	0.5 (0.1 to 0.9)	-13.1 (-19.9 to 13.2)	-23.9 (-28.9 to -18.4)
28 Bullying victimization	0.5 (0.2 to 1.0)	28 Diet low in seafood omega-3 fatty acids	0.5 (0.1 to 0.8)	-9.4 (-17.3 to -2.5)	-39.0 (-42.7 to -35.2)

Legend:
Environmental/occupational risks
Behavioural risks
Metabolic risks

Figure S2d. Leading 25 Level 3 risk factors for ages 50-69 years by attributable DALYs, percentage of total DALYs (2000 and 2021), and percentage change in attributable DALY counts and age-standardised DALY rates from 2000 to 2021.

DALY = disability-adjusted life-year.

Leading risks 2000	Percent DALYs 2000
1 Smoking	14.5 (12.3 to 16.7)
2 High systolic blood pressure	14.3 (11.8 to 16.6)
3 Particulate matter pollution	12.9 (11.3 to 14.6)
4 High fasting plasma glucose	7.0 (6.3 to 7.7)
5 High LDL cholesterol	6.1 (3.9 to 8.3)
6 High body-mass index	6.0 (2.6 to 9.5)
7 High alcohol use	4.3 (3.4 to 5.4)
8 Kidney dysfunction	4.2 (3.8 to 4.8)
9 Diet high in sodium	3.2 (0.9 to 6.7)
10 Diet low in fruits	3.2 (1.1 to 4.9)
11 Diet low in whole grains	2.8 (1.2 to 4.2)
12 Secondhand smoke	2.3 (1.3 to 3.4)
13 Lead exposure	2.2 (-0.2 to 4.5)
14 Unsafe sex	1.9 (1.7 to 2.1)
15 Low temperature	1.8 (1.6 to 2.0)
16 Diet low in vegetables	1.5 (0.8 to 2.2)
17 Diet low in fiber	1.4 (0.4 to 2.2)
18 Unsafe water source	1.3 (0.6 to 2.2)
19 Diet low in seafood omega-3 fatty acids	1.3 (0.3 to 2.2)
20 Diet low in nuts and seeds	1.3 (0.4 to 2.0)
21 Diet low in omega-6 polyunsaturated fatty acids	1.2 (-3.9 to 4.5)
22 Occupational injuries	1.2 (1.1 to 1.3)
23 Unsafe sanitation	1.1 (0.7 to 1.8)
24 Occupational particulate matter, gases, and fumes	0.9 (0.7 to 1.1)
25 Low bone mineral density	0.9 (0.7 to 1.0)
27 Low physical activity	0.8 (0.3 to 1.2)
28 Drug use	0.7 (0.6 to 0.9)
29 Occupational ergonomic factors	0.7 (0.5 to 1.0)

Leading risks 2021	Percent DALYs 2021	Percent change number of DALYs 2000-2021	Percent change age-standardised rate of DALYs 2000-2021
1 High systolic blood pressure	11.6 (9.6 to 13.5)	33.6 (25.1 to 42.8)	-24.3 (-28.4 to -20.0)
2 Smoking	10.2 (8.5 to 11.9)	15.6 (6.9 to 25.8)	-34.8 (-39.2 to -29.7)
3 Particulate matter pollution	8.8 (7.3 to 10.2)	10.9 (2.6 to 21.2)	-41.9 (-47.2 to -35.6)
4 High fasting plasma glucose	8.4 (7.5 to 9.3)	96.1 (86.5 to 106.4)	7.9 (3.3 to 12.9)
5 High body-mass index	7.6 (3.3 to 11.5)	105.6 (94.8 to 116.7)	15.7 (9.9 to 21.7)
6 High LDL cholesterol	5.0 (3.2 to 6.7)	32.6 (25.7 to 40.0)	-26.1 (-29.6 to -22.4)
7 Kidney dysfunction	4.1 (3.6 to 4.6)	57.9 (50.1 to 67.2)	-12.4 (-16.5 to -7.9)
8 High alcohol use	3.5 (2.9 to 4.2)	32.6 (19.7 to 45.0)	-25.8 (-32.0 to -20.4)
9 Diet high in sodium	2.4 (0.6 to 5.3)	26.0 (2.2 to 41.0)	-26.8 (-40.9 to -19.1)
10 Diet low in fruits	2.4 (0.9 to 3.7)	24.8 (17.4 to 34.9)	-26.6 (-30.9 to -20.5)
11 Diet low in whole grains	2.3 (1.0 to 3.5)	35.3 (28.3 to 43.0)	-23.3 (-26.9 to -19.5)
12 Secondhand smoke	1.7 (0.9 to 2.4)	17.8 (9.0 to 26.9)	-45.3 (-48.9 to -40.3)
13 Lead exposure	1.7 (-0.2 to 3.4)	23.4 (14.6 to 33.3)	-23.9 (-28.9 to -18.4)
14 Unsafe sex	1.3 (1.1 to 1.4)	10.0 (-3.5 to 27.6)	-52.4 (-58.9 to -42.3)
15 Low temperature	1.2 (1.1 to 1.4)	9.5 (1.8 to 17.8)	-39.5 (-44.2 to -34.5)
16 Diet low in vegetables	1.1 (0.6 to 1.6)	21.4 (12.8 to 36.6)	-28.5 (-33.4 to -21.3)
17 Diet low in omega-6 polyunsaturated fatty acids	1.0 (-3.2 to 3.7)	37.9 (29.0 to 45.7)	-21.3 (-25.7 to -17.0)
18 Diet low in nuts and seeds	0.9 (0.3 to 1.4)	14.5 (6.7 to 22.3)	-34.0 (-37.9 to -30.2)
19 Diet low in fiber	0.9 (0.3 to 1.4)	5.4 (-4.3 to 18.3)	-39.8 (-43.9 to -34.3)
20 Diet low in seafood omega-3 fatty acids	0.8 (0.2 to 1.4)	6.8 (-1.6 to 14.5)	-39.0 (-42.7 to -35.2)
21 Drug use	0.8 (0.7 to 0.9)	82.9 (66.6 to 99.6)	-4.6 (-10.1 to 0.8)
22 Low physical activity	0.8 (0.3 to 1.2)	67.0 (49.5 to 84.8)	-7.9 (-14.7 to 1.0)
23 Low bone mineral density	0.8 (0.7 to 0.9)	47.3 (41.8 to 52.7)	-11.9 (-14.9 to -8.9)
24 Occupational injuries	0.7 (0.6 to 0.8)	-3.1 (-12.0 to 5.1)	-43.6 (-47.5 to -39.8)
25 Occupational ergonomic factors	0.7 (0.5 to 0.9)	57.1 (49.9 to 64.3)	-12.9 (-15.7 to -10.0)
27 Occupational particulate matter, gases, and fumes	0.6 (0.5 to 0.7)	9.7 (0.2 to 20.7)	-33.3 (-39.2 to -27.3)
29 Unsafe water source	0.5 (0.2 to 0.8)	-35.4 (-45.9 to -22.8)	-66.3 (-72.0 to -60.2)
36 Unsafe sanitation	0.4 (0.2 to 0.6)	-45.2 (-52.8 to -35.2)	-69.2 (-74.4 to -63.2)

Legend:

Environmental/occupational risks
Behavioural risks
Metabolic risks

Figure S2e. Leading 25 Level 3 risk factors for ages 70+ years by attributable DALYs, percentage of total DALYs (2000 and 2021), and percentage change in attributable DALY counts and age-standardised DALY rates from 2000 to 2021.

DALY = disability-adjusted life-year.

Leading risks 2000	Percent DALYs 2000
1 High systolic blood pressure	21.2 (17.7 to 24.5)
2 Particulate matter pollution	17.1 (14.7 to 19.3)
3 Smoking	11.9 (9.8 to 13.9)
4 High fasting plasma glucose	9.4 (8.0 to 10.8)
5 High LDL cholesterol	6.2 (3.1 to 9.5)
6 Kidney dysfunction	6.1 (5.1 to 7.1)
7 High body-mass index	5.4 (2.3 to 8.6)
8 Low temperature	3.4 (3.1 to 3.7)
9 Diet high in sodium	3.3 (0.7 to 7.8)
10 Diet low in fruits	2.6 (1.2 to 3.9)
11 Lead exposure	2.6 (-0.3 to 5.3)
12 Diet low in whole grains	2.6 (1.1 to 4.1)
13 Secondhand smoke	2.5 (1.3 to 3.7)
14 High alcohol use	2.0 (1.4 to 2.8)
15 Unsafe water source	1.7 (0.8 to 2.7)
16 Occupational particulate matter, gases, and fumes	1.6 (1.2 to 1.9)
17 Diet low in vegetables	1.5 (0.9 to 2.1)
18 Unsafe sanitation	1.5 (0.9 to 2.3)
19 Low physical activity	1.3 (0.5 to 2.2)
20 Low bone mineral density	1.3 (1.1 to 1.5)
21 Diet low in seafood omega-3 fatty acids	1.2 (0.2 to 2.1)
22 Diet low in fiber	1.1 (0.4 to 1.9)
23 Diet low in nuts and seeds	1.1 (0.3 to 1.9)
24 Diet low in omega-6 polyunsaturated fatty acids	1.0 (-2.8 to 4.1)
25 Ambient ozone pollution	0.9 (0.2 to 1.6)
26 Diet high in processed meat	0.8 (0.2 to 1.3)

Leading risks 2021	Percent DALYs 2021	Percent change number of DALYs 2000-2021	Percent change age-standardised rate of DALYs 2000-2021
1 High systolic blood pressure	17.1 (14.4 to 19.7)	40.6 (31.8 to 49.8)	-24.3 (-28.4 to -20.0)
2 Particulate matter pollution	11.4 (9.4 to 13.5)	16.0 (5.8 to 26.9)	-41.9 (-47.2 to -35.6)
3 High fasting plasma glucose	10.2 (8.7 to 11.9)	90.2 (82.4 to 97.4)	7.9 (3.3 to 12.9)
4 Smoking	8.7 (7.1 to 10.2)	27.2 (17.4 to 38.2)	-34.8 (-39.2 to -29.7)
5 High body-mass index	6.2 (2.7 to 9.7)	100.6 (90.7 to 110.5)	15.7 (9.9 to 21.7)
6 Kidney dysfunction	5.7 (4.9 to 6.5)	62.4 (54.9 to 71.0)	-12.4 (-16.5 to -7.9)
7 High LDL cholesterol	4.7 (2.3 to 7.3)	31.3 (25.1 to 37.5)	-26.1 (-29.6 to -22.4)
8 Diet high in sodium	2.8 (0.5 to 6.5)	43.7 (13.6 to 61.4)	-26.8 (-40.9 to -19.1)
9 Lead exposure	2.5 (-0.3 to 5.0)	64.2 (52.4 to 77.5)	-23.9 (-28.9 to -18.4)
10 Low temperature	2.4 (2.1 to 2.6)	21.7 (13.9 to 29.8)	-39.5 (-44.2 to -34.5)
11 Diet low in fruits	2.1 (1.1 to 3.1)	39.8 (29.2 to 56.5)	-26.6 (-30.9 to -20.5)
12 Diet low in whole grains	2.0 (0.9 to 3.3)	37.6 (31.8 to 44.6)	-23.3 (-26.9 to -19.5)
13 Secondhand smoke	1.9 (1.0 to 2.8)	29.0 (18.9 to 39.7)	-45.3 (-48.9 to -40.3)
14 High alcohol use	1.7 (1.2 to 2.3)	47.7 (33.1 to 62.8)	-25.8 (-32.0 to -20.4)
15 Low bone mineral density	1.4 (1.2 to 1.6)	86.3 (80.5 to 93.1)	-11.9 (-14.9 to -8.9)
16 Low physical activity	1.3 (0.5 to 2.1)	67.7 (52.1 to 87.0)	-7.9 (-14.7 to 1.0)
17 Occupational particulate matter, gases, and fumes	1.2 (0.9 to 1.5)	29.1 (15.7 to 42.6)	-33.3 (-39.2 to -27.3)
18 Diet low in vegetables	1.1 (0.8 to 1.6)	32.6 (22.8 to 46.6)	-28.5 (-33.4 to -21.3)
19 Diet low in omega-6 polyunsaturated fatty acids	0.9 (-2.3 to 3.3)	45.3 (37.0 to 52.3)	-21.3 (-25.7 to -17.0)
20 Ambient ozone pollution	0.8 (0.2 to 1.4)	56.5 (43.3 to 74.5)	-18.8 (-25.9 to -8.8)
21 Unsafe water source	0.8 (0.3 to 1.3)	-22.7 (-39.1 to -1.6)	-66.3 (-72.0 to -60.2)
22 Diet low in nuts and seeds	0.8 (0.2 to 1.3)	18.4 (10.5 to 25.2)	-34.0 (-37.9 to -30.2)
23 Diet low in seafood omega-3 fatty acids	0.7 (0.1 to 1.3)	9.1 (2.5 to 16.0)	-39.0 (-42.7 to -35.2)
24 Diet low in fiber	0.7 (0.3 to 1.1)	8.6 (0.8 to 19.9)	-39.8 (-43.9 to -34.3)
25 Diet high in processed meat	0.6 (0.1 to 1.0)	24.0 (7.6 to 32.4)	-30.4 (-41.3 to -24.8)
27 Unsafe sanitation	0.5 (0.3 to 0.9)	-35.5 (-47.5 to -19.4)	-69.2 (-74.4 to -63.2)

Legend:

Environmental/occupational risks
Behavioural risks
Metabolic risks

Figure S3. Age−standardised DALY rate (per 100 000) for all−causes attributable to (A) all GBD risk−outcome pairs, and (B) GBD risk−outcome pairs, excluding one− and two−star associations, 2021.

(A) All GBD risk−outcome pairs, 2021

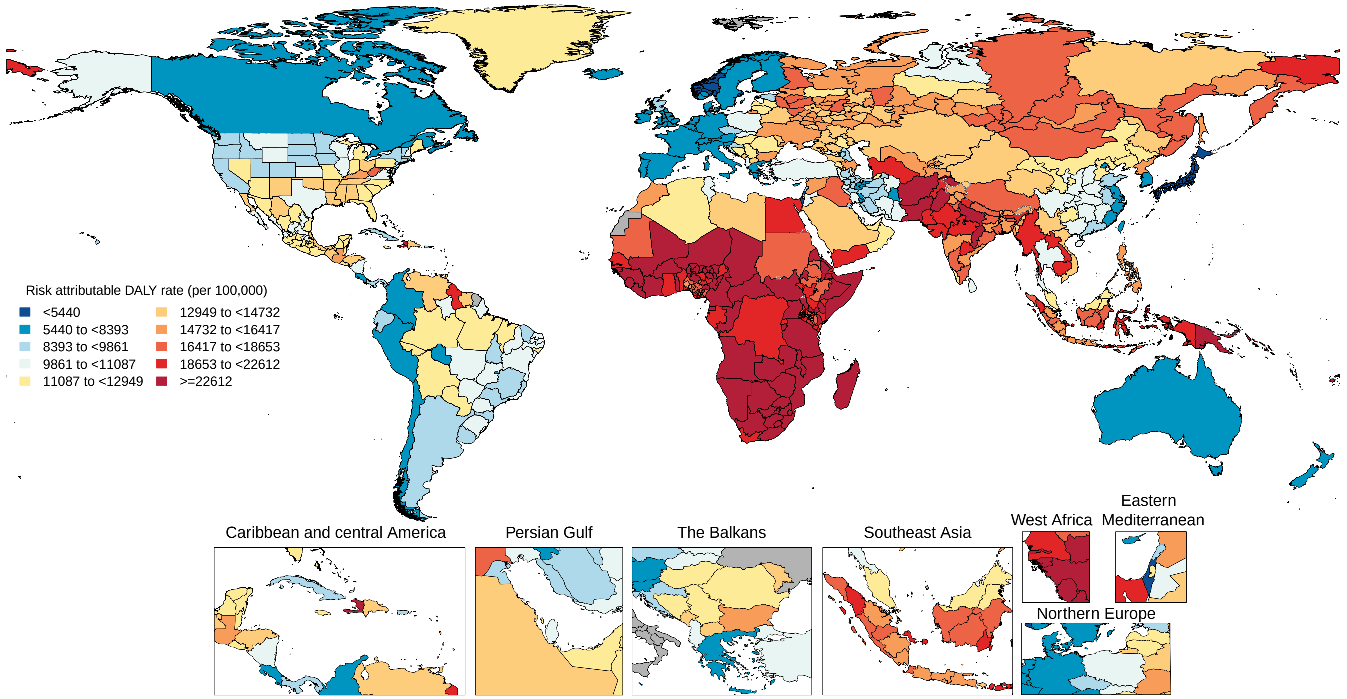

(B) GBD risk−outcome pairs, excluding one− and two−star associations, 2021

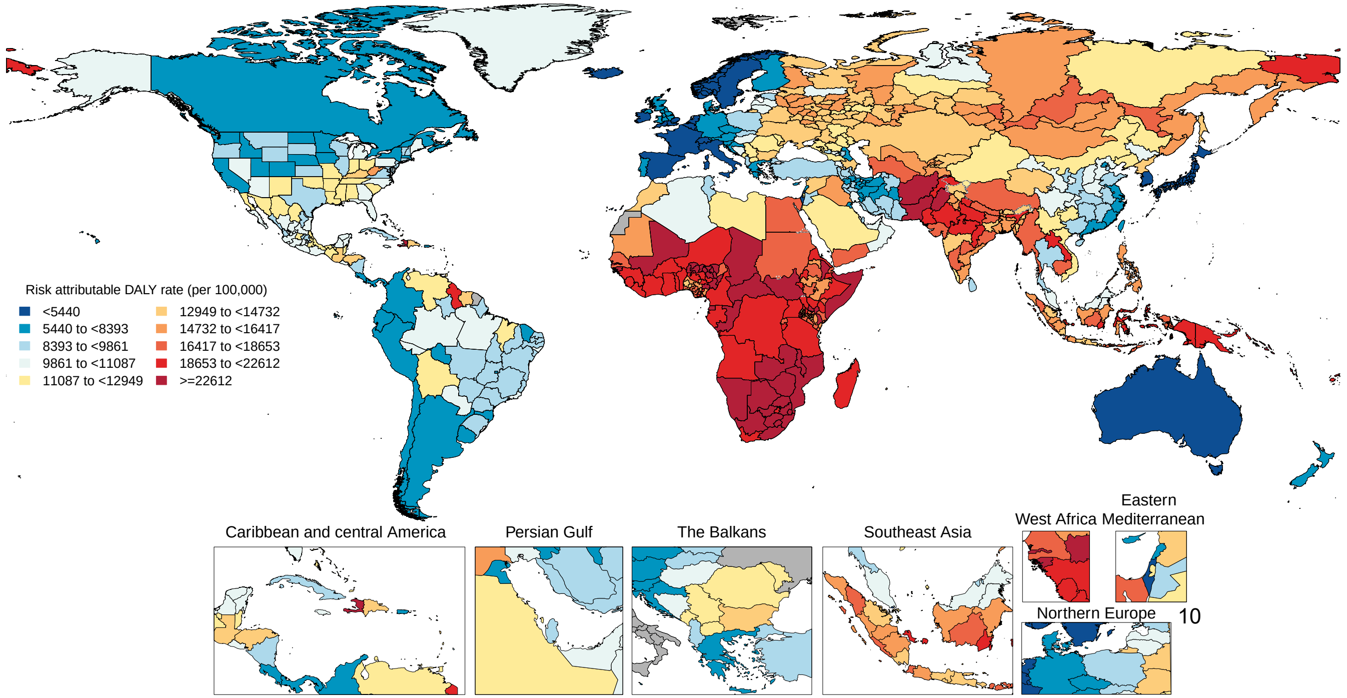

Figure S4. Global DALY counts attributable to risk factors for all GBD risk–outcome pairs (A) versus GBD risk–outcome pairs excluding one- and two-star associations (B), 2021.

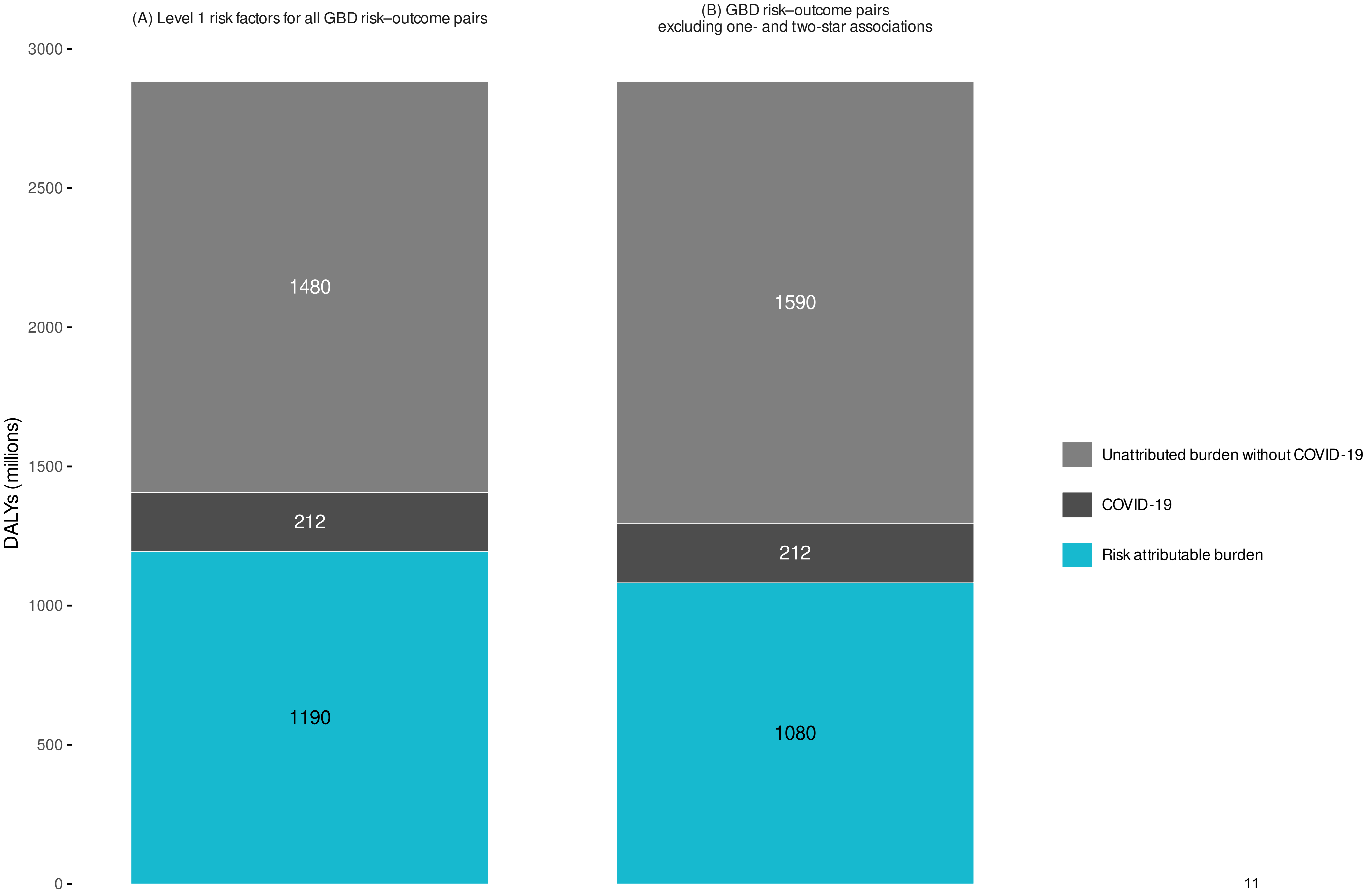

Supplement: Supplementary appendix 2 [file mmc2.pdf]
