# Supplementary material for: Global burden and strength of evidence for 88 risk factors in 204 countries and 811 subnational locations, 1990–2021: a systematic analysis for the Global Burden of Disease Study 2021
Source: Lancet. 2024 May 18;403(10440):2162–203. doi: 10.1016/S0140-6736(24)00933-4 (PMC11120204; doi:10.1016/S0140-6736(24)00933-4)
Supplement: Supplementary appendix 3 [file mmc3.pdf]

THE LANCET

Supplementary appendix 3

This appendix formed part of the original submission and has been peer reviewed. We post it as supplied by the authors.

Supplement to: GBD 2021 Risk Factors Collaborators. Global burden and strength of evidence for 88 risk factors in 204 countries and 811 subnational locations, 1990–2021: a systematic analysis for the Global Burden of Disease Study 2021. *Lancet* 2024; **403:** 2162–203.

Appendix 3: Authorship appendix to "Global burden and strength of evidence for 88 risk factors in 204 countries and 811 subnational locations, 1990–2021: a systematic analysis for the Global Burden of Disease Study 2021"

This appendix provides further authorship detail for "Global burden and strength of evidence for 88 risk factors in 204 countries and 811 subnational locations, 1990–2021: a systematic analysis for the Global Burden of Disease Study 2021."

Table of Contents

GBD 2021 Risk Factor Capstone Collaborators....2

Affiliations....10

Authors' Contributions....50

- Managing the overall research enterprise....50
- Writing the first draft of the manuscript....50
- Primary responsibility for applying analytical methods to produce estimates....51
- Primary responsibility for seeking, cataloguing, extracting, or cleaning data; designing or coding figures and tables....51
- Providing data or critical feedback on data sources....51
- Developing methods or computational machinery....54
- Providing critical feedback on methods or results....55
- Drafting the work or revising it critically for important intellectual content....61
- Managing the estimation or publications process....67

GBD 2021 Risk Factor Capstone Collaborators

Michael Brauer*, Gregory A Roth*, Aleksandr Y Aravkin, Peng Zheng, Kalkidan Hassen Abate, Yohannes Habtegiorgis Abate, Cristiana Abbafati, Rouzbeh Abbasgholizadeh, Madineh Akram Abbasi, Mohammadreza Abbasian, Mitra Abbasifard, Mohsen Abbasi-Kangevari, Samar Abd ElHafeez, Sherief Abd-Elsalam, Parsa Abdi, Mohammad Abdollahi, Meriem Abdoun, Deldar Morad Abdulah, Auwal Abdullahi, Mesfin Abebe, Aidin Abedi, Armita Abedi, Tadesse M Abegaz, Roberto Ariel Abeldaño Zuñiga, Olumide Abiodun, Temesgen Lera Abiso, Richard Gyan Aboagye, Hassan Abolhassani, Mohamed Abouzid, Girma Beressa Aboye, Lucas Guimarães Abreu, Hasan Abualruz, Bilyaminu Abubakar, Eman Abu-Gharbieh, Hana Jihad Jihad Abukhadijah, Salahdein Aburuz, Ahmed Abu-Zaid, Mesafint Molla Adane, Isaac Yeboah Addo, Giovanni Addolorato, Rufus Adesoji Adedoyin, Victor Adekanmbi, Bashir Aden, Juliana Bunmi Adetunji, Temitayo Esther Adeyeoluwa, Rishan Adha, Amin Adibi, Qorinah Estiningtyas Sakilah Adnani, Leticia Akua Adzigbli, Aanuoluwapo Adeyimika Afolabi, Rotimi Felix Afolabi, Ashkan Afshin, Shadi Afyouni, Muhammad Sohail Afzal, Saira Afzal, Suneth Buddhika Agampodi, Faith Agbozo, Shahin Aghamiri, Antonella Agodi, Anurag Agrawal, Williams Agyemang-Duah, Bright Opoku Ahinkorah, Aqeel Ahmad, Danish Ahmad, Firdos Ahmad, Noah Ahmad, Shahzaib Ahmad, Tauseef Ahmad, Ali Ahmed, Anisuddin Ahmed, Ayman Ahmed, Luai A Ahmed, Muktar Beshir Ahmed, Safoora Ahmed, Syed Anees Ahmed, Marjan Ajami, Gizachew Taddesse Akalu, Essona Matatom Akara, Hossein Akbarialiabad, Shiva Akhlaghi, Karolina Akinosoglou, Tomi Akinyemiju, Mohammed Ahmed Akkaif, Sreelatha Akkala, Blessing Akombi-Inyang, Salah Al Awaidy, Syed Mahfuz Al Hasan, Fares Alahdab, Tareq Mohammed Ali AL-Ahdal, Samer O Alalalmeh, Tariq A Alalwan, Ziyad Al-Aly, Khurshid Alam, Nazmul Alam, Fahad Mashhour Alanezi, Turki M Alanzi, Almaza Albakri, Mohammad T AlBataineh, Wafa A Aldhaleei, Robert W Aldridge, Mulubirhan Assefa Alemayohu, Yihun Mulugeta Alemu, Bassam Al-Fatly, Adel Ali Saeed Al-Gheethi, Khairat Al-Habbal, Khalid F Alhabib, Robert Kaba Alhassan, Abid Ali, Amjad Ali, Beriwan Abdulqadir Ali, Iman Ali, Liaqat Ali, Mohammed Usman Ali, Rafat Ali, Syed Shujait Shujait Ali, Waad Ali, Gianfranco Alicandro, Sheikh Mohammad Alif, Syed Mohamed Aljunid, François Alla, Sabah Al-Marwani, Hesham M Al-Mekhlafi, Sami Almustanyir, Mahmoud A Alomari, Jordi Alonso, Jaber S Alqahtani, Ahmed Yaseen Alqutaibi, Rajaa M Al-Raddadi, Ahmad Alrawashdeh, Rami Hani Al-Rifai, Sahel Majed Alrousan, Salman Khalifah Al-Sabah, Najim Z Alshahrani, Zaid Altaany, Awais Altaf, Jaffar A Al-Tawfiq, Khalid A Altirkawi, Deborah Oyine Aluh, Nelson Alvis-Guzman, Nelson J Alvis-Zakzuk, Hassan Alwafi, Mohammad Sami Al-Wardat, Yaser Mohammed Al-Worafi, Hany Aly, Safwat Aly, Karem H Alzoubi, Walid Al-Zyoud, Uchenna Anderson Amaechi, Masous Aman Mohammadi, Reza Amani, Sohrab Amiri, Mohammad Hosein Amirzade-Iranaq, Enrico Ammirati, Hubert Amu, Dickson A Amugsi, Ganiyu Adeniyi Amusa, Robert Ancuceanu, Deanna Anderlini, Jason A Anderson, Pedro Prata Andrade, Catalina Liliana Andrei, Tudorel Andrei, Susan C Anenberg, Dhanalakshmi Angappan, Colin Angus, Abhishek Anil, Sneha Anil, Afifa Anjum, Amir Anoushiravani, Ippazio Cosimo Antonazzo, Catherine M Antony, Ernoiz Antriyandarti, Boluwatife Stephen Anuoluwa, Davood Anvari, Saeid Anvari, Saleha Anwar, Sumadi Lukman Anwar, Razique Anwer, Ekenedilichukwu Emmanuel Anyabolo, Anayochukwu Edward Anyasodor, Geminn Louis Carace Apostol, Jalal Arabloo, Razman Arabzadeh Bahri, Mosab Arafat, Demelash Areda, Brhane Berhe Aregawi, Abdulfatai Aremu, Benedetta Armocida, Michael Benjamin Arndt, Johan Ärnlöv, Mahwish Arooj, Anton A Artamonov, Kurnia Dwi Artanti, Idowu Thomas Aruleba, Ashokan Arumugam, Akram M Asbeutah, Saeed Asgary, Akeza Awealom Asgedom, Charlie Ashbaugh, Mubarek Yesse Ashemo, Tahira Ashraf, Amir Askarinejad, Michael Assmus, Thomas Astell-Burt, Mohammad Athar, Seyyed Shamsadin Athari, Prince Atorkey, Alok Atreya, Avinash Aujayeb, Marcel Ausloos, Leticia Avila-Burgos, Andargie Abate Awoke, Beatriz Paulina Ayala Quintanilla, Haleh Ayatollahi,

Carlos Ayestas Portugal, Jose L Ayuso-Mateos, Sina Azadnajafabad, Rui M S Azevedo, Gulrez Shah Azhar, Hosein Azizi, Ahmed Y Azzam, Insa Linnea Backhaus, Muhammad Badar, Ashish D Badiye, Arvind Bagga, Soroush Baghdadi, Nasser Bagheri, Sara Bagherieh, Pegah Bahrami Taghanaki, Ruhai Bai, Atif Amin Baig, Jennifer L Baker, Shankar M Bakkannavar, Madhan Balasubramanian, Ovidiu Constantin Baltatu, Kiran Bam, Soham Bandyopadhyay, Biswajit Banik, Palash Chandra Banik, Aduragbemi Banke-Thomas, Hansi Bansal, Martina Barchitta, Mainak Bardhan, Erfan Bardideh, Suzanne Lyn Barker-Collo, Till Winfried Bärnighausen, Francesco Barone-Adesi, Hiba Jawdat Barqawi, Lope H Barrero, Amadou Barrow, Sandra Barteit, Zarrin Basharat, Afisu Basiru, João Diogo Basso, Mohammad-Mahdi Bastan, Sanjay Basu, Sai Batchu, Kavita Batra, Ravi Batra, Bernhard T Baune, Mohsen Bayati, Nebiyou Simegnew Bayileyegn, Thomas Beaney, Amir Hossein Behnoush, Maryam Beiranvand, Yannick Béjot, Alehegn Bekele, Uzma Iqbal Belgaumi, Arielle Wilder Bell, Michelle L Bell, Muhammad Bashir Bello, Olorunjuwon Omolaja Bello, Luis Belo, Apostolos Beloukas, Salaheddine Bendak, Derrick A Bennett, Fiona B Bennitt, Isabela M Bensenor, Habib Benzian, Azizullah Beran, Zombor Berezvai, Eduardo Bernabe, Robert S Bernstein, Paulo J G Bettencourt, Akshaya Srikanth Bhagavathula, Neeraj Bhala, Dinesh Bhandari, Nikha Bhardwaj, Pankaj Bhardwaj, Sonu Bhaskar, Ajay Nagesh Bhat, Vivek Bhat, Gurjit Kaur Bhatti, Jasvinder Singh Bhatti, Manpreet S Bhatti, Rajbir Bhatti, Mohiuddin Ahmed Bhuiyan, Zulfiqar A Bhutta, Boris Bikbov, Jessica Devin Bishai, Catherine Bisignano, Atanu Biswas, Bijit Biswas, Raaj Kishore Biswas, Tone Bjørge, Micheal Kofi Boachie, Hosea Boakye, Moses John Bockarie, Virginia Bodolica, Aadam Olalekan Bodunrin, Eyob Ketema Bogale, Srinivasa Rao Bolla, Archith Boloor, Milad Bonakdar Hashemi, Sri Harsha Boppana, Berrak Bora Basara, Hamed Borhany, Alejandro Botero Carvajal, Souad Bouaoud, Soufiane Boufous, Rupert Bourne, Christopher Boxe, Dejana Braithwaite, Luisa C Brant, Amanpreet Brar, Nicholas J K Breitborde, Susanne Breitner, Hermann Brenner, Andrey Nikolaevich Briko, Gabrielle Britton, Colin Stewart Brown, Annie J Browne, Andre R Brunoni, Dana Bryazka, Norma B Bulamu, Lemma N Bulto, Danilo Buonsenso, Katrin Burkart, Richard A Burns, Reinhard Busse, Yasser Bustanji, Nadeem Shafique Butt, Zahid A Butt, Florentino Luciano Caetano dos Santos, Jack Cagney, Lucero Cahuana-Hurtado, Daniela Calina, Luis Alberto Cámera, Luciana Aparecida Campos, Ismael R Campos-Nonato, Chao Cao, Fan Cao, Yubin Cao, Angelo Capodici, Rosario Cárdenas, Sinclair Carr, Giulia Carreras, Juan J Carrero, Andrea Carugno, Felix Carvalho, Márcia Carvalho, Joao Mauricio Castaldelli-Maia, Carlos A Castañeda-Orjuela, Giulio Castelpietra, Ferrán Catalá-López, Alberico L Catapano, Maria Sofia Cattaruzza, Arthur Caye, Christopher R Cederroth, Luca Cegolon, Muthia Cenderadewi, Kelly M Cercy, Ester Cerin, Joshua Chadwick, Chiranjib Chakraborty, Promit Ananyo Chakraborty, Sandip Chakraborty, Jeffrey Shi Kai Chan, Raymond N C Chan, Joht Singh Chandan, Rama Mohan Chandika, Pankaj Chaturvedi, An-Tian Chen, Catherine S Chen, Haowei Chen, Meng Xuan Chen, Mingling Chen, Simiao Chen, Ching-Yu Cheng, Esther T W Cheng, Nicolas Cherbuin, Gerald Chi, Fatemeh Chichagi, Odgerel Chimed-Ochir, Ritesh Chimoriya, Patrick R Ching, Jesus Lorenzo Chirinos-Caceres, Abdulaal Chitheer, William C S Cho, Bryan Chong, Hitesh Chopra, Rajiv Chowdhury, Devasahayam J Christopher, Dinh-Toi Chu, Isaac Sunday Chukwu, Eric Chung, Sheng-Chia Chung, Muhammad Chutiyami, Iolanda Cioffi, Rebecca M Cogen, Aaron J Cohen, Alyssa Columbus, Joao Conde, Alexandru Corlateanu, Samuele Cortese, Paolo Angelo Cortesi, Vera Marisa Costa, Simona Costanzo, Michael H Criqui, Jessica A Cruz, Natália Cruz-Martins, Garland T Culbreth, Alanna Gomes da Silva, Omid Dadras, Xiaochen Dai, Zhaoli Dai, Patience Unekwuojo Daikwo, Lachlan L Dalli, Giovanni Damiani, Emanuele D'Amico, Lucio D'Anna, Aso Mohammad Darwesh, Jai K Das, Subasish Das, Nihar Ranjan Dash, Mohsen Dashti, Claudio Alberto Dávila-Cervantes, Nicole Davis Weaver, Dragos Virgil Davitoiu, Fernando Pio De la Hoz, Alejandro de la Torre-Luque, Diego De Leo, Shayom Debopadhaya, Louisa Degenhardt, Cristian Del Bo', Ivan Delgado-Enciso, Juana Maria Delgado-Saborit,

Chalachew Kassaw Demoze, Edgar Denova-Gutiérrez, Nikolaos Dervenis, Emina Dervišević, Hardik Dineshbhai Desai, Rupak Desai, Vinoth Gnana Chellaiyan Devanbu, Syed Masudur Rahman Dewan, Arkadeep Dhali, Kuldeep Dhama, Amol S Dhane, Mandira Lamichhane Dhimal, Meghnath Dhimal, Sameer Dhingra, Vishal R Dhulipala, Raja Ram Dhungana, Diana Dias da Silva, Daniel Diaz, Luis Antonio Diaz, Michael J Diaz, Adriana Dima, Delaney D Ding, Monica Dinu, Shirin Djalalinia, Thanh Chi Do, Thao Huynh Phuong Do, Camila Bruneli do Prado, Masoud Dodangeh, Sushil Dohare, Klara Georgieva Dokova, Wanyue Dong, Deepa Dongarwar, Mario D'Oria, Fariba Dorostkar, E Ray Dorsey, Rajkumar Doshi, Leila Doshmangir, Robert Kokou Dowou, Tim Robert Driscoll, Ashel Chelsea Dsouza, Haneil Larson Dsouza, Samuel C Dumith, Bruce B Duncan, Andre Rodrigues Duraes, Senbagam Duraisamy, Anar Dushpanova, Paulina Agnieszka Dzianach, Arkadiusz Marian Dziedzic, Alireza Ebrahimi, Chidiebere Peter Echieh, Abdelaziz Ed-Dra, Hisham Atan Edinur, David Edvardsson, Kristina Edvardsson, Ferry Efendi, Aziz Eftekharimehrabad, Ebrahim Eini, Michael Ekholuenetale, Temitope Cyrus Ekundayo, Rabie Adel El Arab, Maysaa El Sayed Zaki, Faris El-Dahiyat, Noha Mousaad Elemam, Frank J Elgar, Ghada Metwally Tawfik ElGohary, Hala Rashad Elhabashy, Muhammed Elhadi, Ahmed O Elmehrath, Omar Abdelsadek Abdou Elmeligy, Mohammed Elshaer, Ibrahim Elsohaby, Theophilus I Emeto, Negin Esfandiari, Babak Eshrati, Majid Eslami, Sayed Vahid Esmaeili, Kara Estep, Farshid Etaee, Natalia Fabin, Adeniyi Francis Fagbamigbe, Omotayo Francis Fagbule, Saman Fahimi, Luca Falzone, Mohammad Fareed, Carla Sofia e Sá Farinha, MoezAlIslam Ezzat Mahmoud Faris, Pawan Sirwan Faris, Andre Faro, Folorunso Oludayo Fasina, Ali Fatehizadeh, Nelsensius Klau Fauk, Timur Fazylov, Valery L Feigin, Xiaoqi Feng, Seyed-Mohammad Fereshtehnejad, Abdullah Hamid Feroze, Pietro Ferrara, Alize J Ferrari, Nuno Ferreira, Getahun Fetensa, Bikila Regassa Feyisa, Irina Filip, Florian Fischer, Ida Fitriana, Joanne Flavel, Carsten Flohr, David Flood, Luisa S Flor, Nataliya A Foigt, Morenike Oluwatoyin Folayan, Lisa M Force, Daniela Fortuna, Matteo Foschi, Richard Charles Franklin, Alberto Freitas, Sara D Friedman, Blima Fux, Sridevi G, Peter Andras Gaal, Santosh Gaihre, Márió Gajdács, Yaseen Galali, Silvano Gallus, Aravind P Gandhi, Balasankar Ganesan, Mohammad Arfat Ganiyani, Vanessa Garcia, William M Gardner, Ravindra K Garg, Rupesh K Gautam, Tilaye Gebru Gebi, Miglas W Gebregergis, Mesfin Gebrehiwot, Tesfay B B Gebremariam, Teferi Gebru Gebremeskel, Urge Gerema, Lemma Getacher, Genanew K a Getahun, Molla Getie, Fataneh Ghadirian, Sadegh Ghafarian, Amir Ghaffari Jolfayi, Khalid Yaser Ghailan, Alireza Ghajar, MohammadReza Ghasemi, Ghazal Ghasempour Dabaghi, Afsaneh Ghasemzadeh, Fariba Ghassemi, Ramy Mohamed Ghazy, Ali Gholami, Ali Gholamrezanezhad, Nasim Gholizadeh, Mahsa Ghorbani, Artyom Urievich Gil, Gabriela Fernanda Gil, Nora M Gilbertson, Paramjit Singh Gill, Tiffany K Gill, Ebisa Zerihun Gindaba, Alem Girmay, James C Glasbey, Elena V Gnedovskaya, Laszlo Göbölös, Myron Anthony Godinho, Amit Goel, Mahaveer Golechha, Pouya Goleij, Davide Golinelli, Nelson G M Gomes, Sameer Vali Gopalani, Giuseppe Gorini, Houman Goudarzi, Alessandra C Goulart, Mahdi Gouravani, Anmol Goyal, Simon Matthew Graham, Michal Grivna, Giuseppe Grosso, Shi-Yang Guan, Giovanni Guarducci, Mohammed Ibrahim Mohialdeen Gubari, Avirup Guha, Stefano Guicciardi, Snigdha Gulati, David Gulisashvili, Damitha Asanga Gunawardane, Cui Guo, Anish Kumar Gupta, Bhawna Gupta, Mohak Gupta, Rahul Gupta, Rajat Das Gupta, Rajeev Gupta, Sapna Gupta, Veer Bala Gupta, Vijai Kumar Gupta, Vivek Kumar Gupta, Farrokh Habibzadeh, Parham Habibzadeh, Tesfahun Simon Hadaro, Zahra Hadian, Nils Haep, Hamed Haghi-Aminjan, Dariush Haghmorad, Hailey Hagins, Demewoz Haile, Alemayehu Hailu, Adel Hajj Ali, Esam S Halboub, Aram Halimi, Brian J Hall, Sebastian Haller, Rabih Halwani, Randah R Hamadeh, Nadia M Hamdy, Sajid Hameed, Samer Hamidi, Ahmad Hammoud, Asif Hanif, Nasrin Hanifi, Zaim Anan Haq, Md Rabiul Haque, Harapan Harapan, Arief Hargono, Josep Maria Haro, Ahmed I Hasaballah, Ikramul Hasan, Mohammad Jahid Hasan, S M Mahmudul Hasan, Hamidreza Hasani,

Mohammad Hasanian, Nadim Hashmeh, Md Saquib Hasnain, Amr Hassan, Ikrama Hassan, Mahgol Sadat Hassan Zadeh Tabatabaei, Shokoufeh Hassani, Soheil Hassanipour, Hadi Hassankhani, Johannes Haubold, Rasmus J Havmoeller, Simon I Hay, Jeffrey J Hebert, Omar E Hegazi, Tadele Yohannes Hegena, Golnaz Heidari, Mohammad Heidari, Bartosz Helfer, Mehdi Hemmati, Claire A Henson, Molly E Herbert, Claudiu Herteliu, Austin Heuer, Kamal Hezam, Thomas Kwadwo Hinneh, Yuta Hiraike, Nguyen Quoc Hoan, Ramesh Holla, Julia Hon, Mohammad Enamul Hoque, Nobuyuki Horita, Sahadat Hossain, Seyed Ehsan Hosseini, Hassan Hosseinzadeh, Mehdi Hosseinzadeh, Mihaela Hostiuc, Sorin Hostiuc, Hanno Hoven, Mohamed Hsairi, Johnathan M Hsu, Chengxi Hu, Junjie Huang, Md Nazmul Huda, Erin N Hulland, Michael Hultström, Kiavash Hushmandi, Javid Hussain, Nawfal R Hussein, Chantal K Huynh, Hong-Han Huynh, Segun Emmanuel Ibitoye, Oluwatope Olaniyi Idowu, Audrey L Ihler, Nayu Ikeda, Kevin S Ikuta, Olayinka Stephen Ilesanmi, Irena M Ilic, Milena D Ilic, Mohammad Tarique Imam, Mustapha Immurana, Leeberk Raja Inbaraj, Lalu Muhammad Irham, Mustafa Alhaji Isa, Md Rabiul Islam, Faisal Ismail, Nahlah Elkudssiah Ismail, Hiroyasu Iso, Gaetano Isola, Masao Iwagami, Chidozie C D Iwu, Chinwe Juliana Iwu-Jaja, Vinothini J, Jalil Jaafari, Louis Jacob, Kathryn H Jacobsen, Farhad Jadidi-Niaragh, Kasra Jahankhani, Nader Jahanmehr, Haitham Jahrami, Akhil Jain, Nityanand Jain, Ammar Abdulrahman Jairoun, Abhishek Jaiswal, Mihajlo Jakovljevic, Reza Jalilzadeh Yengejeh, Roland Dominic G Jamora, Abubakar Ibrahim Jatau, Sabzali Javadov, Tahereh Javaheri, Shubha Jayaram, Jayakumar Jeganathan, Bijay Mukesh Jeswani, Heng Jiang, Catherine O Johnson, Mohammad Jokar, Nabi Jomehzadeh, Jost B Jonas, Tamas Joo, Abel Joseph, Nitin Joseph, Vivek Joshi, Charity Ehimwenma Joshua, Jacek Jerzy Jozwiak, Mikk Jürisson, Billingsley Kaambwa, Ali Kabir, Zubair Kabir, Vidya Kadashetti, Ethan M Kahn, Rizwan Kalani, Feroze Kaliyadan, Sanjay Kalra, Rajesh Kamath, Thanigaivelan Kanagasabai, Tanuj Kanchan, Himal Kandel, Edmund Wedam Kanmiki, Kehinde Kazeem Kanmodi, Sushil Kumar Kansal, Daniel John Kapner, Neeti Kapoor, Efstratios Karagiannidis, Mehrdad Karajizadeh, Paschalis Karakasis, Shama D Karanth, Ibraheem M Karaye, André Karch, Asima Karim, Hanie Karimi, Shilpi Karmakar, Faizan Zaffar Kashoo, Hengameh Kasraei, Woldeteklehaymanot Dagne Kassahun, Nicholas J Kassebaum, Molly B Kassel, Srinivasa Vittal Katikireddi, Joonas H Kauppila, Norito Kawakami, Neda Kaydi, Gbenga A Kayode, Foad Kazemi, Peter Njenga Keiyoro, Laura Kemmer, John H Kempen, Jessica A Kerr, Emmanuelle Kesse-Guyot, Yousef Saleh Khader, Morteza Abdullatif Khafaie, Himanshu Khajuria, Amirmohammad Khalaji, Mariam Khalil, Alireza Khalilian, Faham Khamesipour, Asaduzzaman Khan, M Nuruzzaman Khan, Maseer Khan, Mohammad Jobair Khan, Moien AB Khan, Shaghayegh Khanmohammadi, Khaled Khatab, Haitham Khatatbeh, Moawiah Mohammad Khatatbeh, Mahalaqua Nazli Khatib, Armin Khavandegar, Hamid Reza Khayat Kashani, Feriha Fatima Khidri, Elaheh Khodadoust, Moein Khormali, Zahra Khorrami, Atulya Aman Khosla, Mahmood Khosrowjerdi, Haneen Khreis, Helda Khusun, Zemene Demelash Kifle, Kwanghyun Kim, Min Seo Kim, Yun Jin Kim, Ruth W Kimokoti, Adnan Kisa, Sezer Kisa, Luke D Knibbs, Ann Kristin Skrindo Knudsen, David S Q Koh, Ali-Asghar Kolahi, Farzad Kompani, Jianqiu Kong, Gerbrand Koren, Miikka Korja, Vladimir Andreevich Korshunov, Oleksii Korzh, Soewarta Kosen, Nikhil Kothari, Parvaiz A Koul, Sindhura Lakshmi Koulmane Laxminarayana, Kewal Krishan, Vijay Krishnamoorthy, Yuvaraj Krishnamoorthy, Bindu Krishnan, Kris J Krohn, Barthelemy Kuate Defo, Burcu Kucuk Bicer, Md Abdul Kuddus, Mohammed Kuddus, Nuworza Kugbey, Ilari Kuitunen, Mukhtar Kulimbet, Vishnutheertha Kulkarni, Ashish Kumar, Nithin Kumar, Vijay Kumar, Satyajit Kundu, Om P Kurmi, Asep Kusnali, Dian Kusuma, Tezer Kutluk, Carlo La Vecchia, Muhammad Awwal Ladan, Lucie Laflamme, Chandrakant Lahariya, Daphne Teck Ching Lai, Dharmesh Kumar Lal, Tea Lallukka, Judit Lám, Qing Lan, Tuo Lan, Iván Landires, Francesco Lanfranchi, Berthold Langguth, Van Charles Lansingh, Ariane Laplante-Lévesque, Bagher Larijani, Anders O Larsson, Savita Lasrado, Paolo Lauriola, Huu-Hoai Le, Long Khanh Dao Le, Nhi

Huu Hanh Le, Thao Thi Thu Le, Janet L Leasher, Caterina Ledda, Munjae Lee, Paul H Lee, Seung Won Lee, Shaun Wen Huey Lee, Yo Han Lee, Kate E LeGrand, James Leigh, Elvynna Leong, Temesgen L Lerango, Haley Lescinsky, Janni Leung, Ming-Chieh Li, Wang-Zhong Li, Wei Li, Yichong Li, Zhihui Li, Virendra S Ligade, Lee-Ling Lim, Stephen S Lim, Ro-Ting Lin, Shuzhi Lin, Chaojie Liu, Gang Liu, Jinli Liu, Jue Liu, Richard T Liu, Shiwei Liu, Wei Liu, Xiaofeng Liu, Xuefeng Liu, Katherine M Livingstone, Erand Llanaj, Ayush Lohiya, Rubén López-Bueno, Platon D Lopukhov, Stefan Lorkowski, Paulo A Lotufo, Rafael Lozano, Jailos Lubinda, Giancarlo Lucchetti, Lisha Luo, Hengliang lv, Hawraz Ibrahim M Amin, Zheng Feei Ma, Kelsey Lynn Maass, Mahmoud Mabrok, Nikolaos Machairas, Monika Machoy, Asma Mafhoumi, Mohammed Magdy Abd El Razek, Azzam A Maghazachi, D R Mahadeshwara Prasad, Sandeep B Maharaj, Mansour Adam Mahmoud, Elham Mahmoudi, Azeem Majeed, Omar Mohamed Makram, Konstantinos Christos Makris, Satyaveni Malasala, Venkatesh Maled, Kashish Malhotra, Ahmad Azam Malik, Iram Malik, Lesibana Anthony Malinga, Deborah Carvalho Malta, Abdullah A Mamun, Ana Laura Manda, Yosef Manla, Ali Mansour, Borhan Mansouri, Pejman Mansouri, Marjan Mansourian, Mohammad Ali Mansournia, Lorenzo Giovanni Mantovani, Emmanuel Manu, Hamid Reza Marateb, Joemer C Maravilla, Elizabeth Marsh, Gabriel Martinez, Ramon Martinez-Piedra, Santi Martini, Francisco Rogerlândio Martins-Melo, Miquel Martorell, Wolfgang Marx, Sharmeen Maryam, Yasith Mathangasinghe, Alexander G Mathioudakis, Fernanda Penido Matozinhos, Jishanth Mattumpuram, Andrea Maugeri, Pallab K Maulik, Mahsa Mayeli, Mohsen Mazidi, Antonio Mazzotti, John J McGrath, Martin McKee, Anna Laura W McKowen, Susan A McLaughlin, Michael A McPhail, Steven M McPhail, Enkeleint A Mechili, Asim Mehmood, Khalid Mehmood, Kamran Mehrabani-Zeinabad, Entezar Mehrabi Nasab, Toni Meier, Fabiola Mejia-Rodriguez, Tesfahun Mekene Meto, Birye Dessalegn Mekonnen, Ritesh G Menezes, Belayneh Mengist, George A Mensah, Laverne G Mensah, Alexios-Fotios A Mentis, Sultan Ayoub Meo, Atte Meretoja, Tuomo J Meretoja, Abera M Mersha, Bezawit Afework Mesfin, Tomislav Mestrovic, Kukulege Chamila Dinushi Mettananda, Sachith Mettananda, Tomasz Miazgowski, Georgia Micha, Irmina Maria Michalek, Ana Carolina Micheletti Gomide Nogueira de Sá, Ted R Miller, Mojde Mirarefin, Mojgan Mirghafourvand, Andreea Mirica, Antonio Mirijello, Erkin M Mirrakhimov, Arvin Mirshahi, Maryam Mirzaei, Ajay Kumar Mishra, Vinaytosh Mishra, Philip B Mitchell, Prasanna Mithra, Chaitanya Mittal, Babak Moazen, Madeline E Moberg, Gabriele Mocciaro, Ashraf Mohamadkhani, Abdalla Z Mohamed, Ahmed Ismail Mohamed, Jama Mohamed, Mouhand F H Mohamed, Nouh Saad Mohamed, Esmaeil Mohammadi, Saeed Mohammadi, Abdollah Mohammadian-Hafshejani, Noushin Mohammadifard, Hussen Mohammed, Mustapha Mohammed, Salahuddin Mohammed, Shafiu Mohammed, Ali H Mokdad, Lorenzo Monasta, Stefania Mondello, Mohammad Ali Moni, AmirAli Moodi Ghalibaf, Catrin E Moore, Maryam Moradi, Yousef Moradi, Paula Moraga, Lidia Morawska, Rafael Silveira Moreira, Negar Morovatdar, Shane Douglas Morrison, Jakub Morze, Reza Mosaddeghi Heris, Elias Mossialos, Rohith Motappa, Vincent Mougin, Parsa Mousavi, Ahmed Msherghi, Sumaira Mubarik, Lorenzo Muccioli, Ulrich Otto Mueller, Francesk Mulita, Erin C Mullany, Kavita Munjal, Efrén Murillo-Zamora, BV Murlimanju, Ana-Maria Musina, Ghulam Mustafa, Sathish Muthu, Saravanan Muthupandian, Raman Muthusamy, Muhammad Muzaffar, Woojae Myung, Ayoub Nafei, Ahamarshan Jayaraman Nagarajan, Shankar Prasad Nagaraju, Gabriele Nagel, Mohsen Naghavi, Pirouz Naghavi, Ganesh R Naik, Gurudatta Naik, Firzan Nainu, Tapas Sadasivan Nair, Soroush Najdaghi, Noureddin Nakhostin Ansari, Dhairya P Nanavaty, Vinay Nangia, Sreenivas Narasimha Swamy, Delaram Narimani Davani, Bruno Ramos Nascimento, Gustavo G Nascimento, Abdulqadir J Nashwan, Zuhair S Natto, Javaid Nauman, Samidi N K Navaratna, Muhammad Naveed, Biswa Prakash Nayak, Vinod C Nayak, Rawlance Ndejjo, Sabina Onyinye Nduaguba, Hadush Negash, Ionut Negoi, Ruxandra Irina Negoi, Seyed Aria Nejadghaderi, Chakib Nejjari, Mohammad Hadi

Nematollahi, Samata Nepal, Subas Neupane, Marie Ng, Georges Nguefack-Tsague, Josephine W Ngunjiri, Dang H Nguyen, Nhien Ngoc Y Nguyen, Phat Tuan Nguyen, Phuong The Nguyen, Van Thanh Nguyen, Duc Nguyen Tran Minh, Robina Khan Niazi, Sneha Ingle Nicholson, Jing Nie, Ali Nikoobar, Amin Reza Nikpoor, Dina Nur Anggraini Ningrum, Chukwudi A Nnaji, Efaq Ali Noman, Shuhei Nomura, Nafise Noroozi, Bo Norrving, Jean Jacques Noubiap, Chisom Adaobi Nri-Ezedi, George Ntaios, Mpiko Ntsekhe, Mengistu H Nunemo, Dieta Nurrika, Jerry John Nutor, Bogdan Oancea, Erin M O'Connell, Ismail A Odetokun, Martin James O'Donnell, Michael Safo Oduro, Adesola Adenike Ogunfowokan, Abiola Ogunkoya, In-Hwan Oh, Hassan Okati-Aliabad, Sylvester Reuben Okeke, Akinkunmi Paul Okekunle, Osaretin Christabel Okonji, Andrew T Olagunju, Omotola O Olasupo, Matthew Idowu Olatubi, Arão Belitardo Oliveira, Gláucia Maria Moraes Oliveira, Abdulhakeem Abayomi Olorukooba, Isaac Iyinoluwa Olufadewa, Bolajoko Olubukunola Olusanya, Jacob Olusegun Olusanya, Yinka Doris Oluwafemi, Hany A Omar, Ahmed Omar Bali, Goran Latif Omer, Kanyin Liane Ong, Sokking Ong, Obinna E Onwujekwe, Kenneth Ikenna Onyedibe, Anita Frimpomaa Oppong, Michal Ordak, Verner N Orish, Raffaele Ornello, Heather M Orpana, Alberto Ortiz, Esteban Ortiz-Prado, Wael M S Osman, Samuel M Ostroff, Uchechukwu Levi Osuagwu, Adrian Otoiu, Nikita Otstavnov, Stanislav S Otstavnov, Amel Ouyahia, Mayowa O Owolabi, Ifeoluwa Temitayo Oyeyemi, Oyetunde T Oyeyemi, Mahesh Padukudru P A, Kevin Pacheco-Barrios, Alicia Padron-Monedero, Jagadish Rao Padubidri, Pramod Kumar Pal, Tamás Palicz, Feng Pan, Hai-Feng Pan, Adrian Pana, Sujogya K Panda, Songhomitra Panda-Jonas, Ashok Pandey, Seithikurippu R Pandi-Perumal, Helena Ullyartha Pangaribuan, Ioannis Pantazopoulos, Anca Mihaela Pantea Stoian, Paraskevi Papadopoulou, Marie C Parent, Pragyan Paramita Parija, Romil R Parikh, Seoyeon Park, Sungchul Park, Nicholas Parsons, Ava Pashaei, Maja Pasovic, Roberto Passera, Shankargouda Patil, Dimitrios Patoulias, Venkata Suresh Patthipati, Uttam Paudel, Shrikant Pawar, Hamidreza Pazoki Toroudi, Amy E Peden, Paolo Pedersini, Minjin Peng, Umberto Pensato, Veincent Christian Filipino Pepito, Emmanuel K Peprah, Prince Peprah, Mario F P Peres, Arokiasamy Perianayagam, Norberto Perico, Simone Perna, Konrad Pesudovs, Ionela-Roxana Petcu, Fanny Emily Petermann-Rocha, Hoang Tran Pham, Anil K Philip, Michael R Phillips, Brandon V Pickering, Daniela Pierannunzio, Manon Pigeolet, David M Pigott, Zahra Zahid Piracha, Michael A Piradov, Enrico Pisoni, Mapa Prabhath Piyasena, Dietrich Plass, Evgenii Plotnikov, Dimitri Poddighe, Kevan R Polkinghorne, Ramesh Poluru, Constance Dimity Pond, Djordje S Popovic, Fabio Porru, Maarten J Postma, Govinda Raj Poudel, Ahmad Pour-Rashidi, Akram Pourshams, Naeimeh Pourtaheri, Disha Prabhu, Sergio I Prada, Jalandhar Pradhan, Pranil Man Singh Pradhan, Manya Prasad, Elton Junio Sady Prates, Hery Purnobasuki, Bharathi M Purohit, Jagadeesh Puvvula, Nameer Hashim Qasim, Ibrahim Qattea, Asma Saleem Qazi, Gangzhen Qian, Suli Qiu, Mehrdad Rabiee Rad, Amir Radfar, Raghu Anekal Radhakrishnan, Venkatraman Radhakrishnan, Hadi Raeisi Shahraki, Quinn Rafferty, Alireza Rafiei, Alberto Raggi, Pankaja Raghav Raghav, Nasiru Raheem, Fakher Rahim, Md Jillur Rahim, Mahban Rahimifard, Vafa Rahimi-Movaghar, Md Obaidur Rahman, Muhammad Aziz Rahman, Amir Masoud Rahmani, Bita Rahmani, Mohammad Rahmanian, Nazanin Rahmanian, Vahid Rahmanian, Masoud Rahmati, Setyaningrum Rahmawaty, Diego Raimondo, Sathish Rajaa, Vinoth Rajendran, Prashant Rajput, Mahmoud Mohammed Ramadan, Shakthi Kumaran Ramasamy, Premkumar Ramasubramani, Sheena Ramazanu, Pramod W Ramteke, Juwel Rana, Kritika Rana, Chhabi Lal Ranabhat, Amey Rane, Usha Rani, Annemarei Ranta, Chythra R Rao, Mithun Rao, Puja C Rao, Sowmya J Rao, Davide Rasella, Sina Rashedi, Vahid Rashedi, Mahsa Rashidi, Mohammad-Mahdi Rashidi, Ashkan Rasouli-Saravani, Zubair Ahmed Ratan, Giridhara Rathnaiah Babu, Santosh Kumar Rauniyar, Ilari Rautalin, David Laith Rawaf, Salman Rawaf, Reza Rawassizadeh, Christian Razo, Zinabu Ferede Ferede Reda, Murali Mohan Rama Krishna Reddy, Elrashdy Moustafa Mohamed Redwan, Lennart Reifels, Marissa B Reitsma, Giuseppe Remuzzi,

Bhageerathy Reshmi, Serge Resnikoff, Stefano Restaino, Luis Felipe Reyes, Maryam Rezaei, Nazila Rezaei, Negar Rezaei, Mohsen Rezaeian, Taeho Gregory Rhee, Mavra A Riaz, Antonio Luiz P Ribeiro, Jennifer Rickard, Hannah Elizabeth Robinson-Oden, Célia Fortuna Rodrigues, Mónica Rodrigues, Jefferson Antonio Buendia Rodriguez, Leonardo Roever, Debby Syahru Romadlon, Luca Ronfani, Jennifer Jacqueline Rosauer, Gholamreza Roshandel, Morteza Rostamian, Kunle Rotimi, Himanshu Sekhar Rout, Bedanta Roy, Nitai Roy, Enrico Rubagotti, Guilherme de Andrade Ruela, Susan Fred Rumisha, Tilleye Runghien, Michele Russo, Sacha Walde Ruzzante, Chandan S N, Aly M A Saad, Korosh Saber, Maha Mohamed Saber-Ayad, Siamak Sabour, Simona Sacco, Perminder S Sachdev, Rajesh Sachdeva, Basema Saddik, Adam Saddler, Bashdar Abuzed Sadee, Ehsan Sadeghi, Masoumeh Sadeghi, Elham Sadeghi Majd, Mohammad Reza Saeb, Umar Saeed, Mehdi Safari, Sare Safi, Sher Zaman Safi, Rajesh Sagar, Dominic Sagoe, Fatemeh Saheb Sharif-Askari, Narjes Saheb Sharif-Askari, Amirhossein Sahebkar, Soumya Swaroop Sahoo, Monalisha Sahu, Zahra Saif, Mirza Rizwan Sajid, Joseph W Sakshaug, Nasir Salam, Payman Salamati, Afeez Abolarinwa Salami, Luciane B Salaroli, Leili Salehi, Sana Salehi, Marwa Rashad Salem, Mohammed Z Y Salem, Dauda Salihu, Sohrab Salimi, Giovanni A Salum, Hossein Samadi Kafil, Sara Samadzadeh, Yoseph Leonardo Samodra, Vijaya Paul Samuel, Abdallah M Samy, Juan Sanabria, Rama Krishna Sanjeev, Francesca Sanna, Damian Francesco Santomauro, Milena M Santric-Milicevic, Made Ary Sarasmita, Sivan Yegnanarayana Iyer Saraswathy, Aswini Saravanan, Babak Saravi, Yaser Sarikhani, Rodrigo Sarmiento-Suárez, Gargi Sachin Sarode, Sachin C Sarode, Benn Sartorius, Arash Sarveazad, Brijesh Sathian, Davide Sattin, Monika Sawhney, Ganesh Kumar Saya, Abu Sayeed, Md Abu Sayeed, Mehdi Sayyah, Christophe Schinckus, Maria Inês Schmidt, Art Schuermans, Austin E Schumacher, Aletta Elisabeth Schutte, Michaël Schwarzinger, David C Schwebel, Falk Schwendicke, Siddharthan Selvaraj, Mohammad H Semreen, Subramanian Senthilkumaran, Dragos Serban, Marc L Serre, Yashendra Sethi, Mahan Shafie, Humaira Shah, Nilay S Shah, Pritik A Shah, Syed Mahboob Shah, Ataollah Shahbandi, Amira A Shaheen, Samiah Shahid, Wajeehah Shahid, Hamid R Shahsavari, Moyad Jamal Shahwan, Masood Ali Shaikh, Summaiya Zareen Shaikh, Ali S Shalash, Sunder Sham, Muhammad Aaqib Shamim, Mehran Shams-Beyranvand, Mohammad Ali Shamshirgaran, Mohammad Anas Shamsi, Mohd Shanawaz, Abhishek Shankar, Sadaf Sharfaei, Amin Sharifan, Javad Sharifi-Rad, Manoj Sharma, Ujjawal Sharma, Vishal Sharma, Rajesh P Shastry, Amin Shavandi, Amr Mohamed Elsayed Shehabeldine, Somia Shehzadi, Aziz Sheikh, Jiabin Shen, Adithi Shetty, B Suresh Kumar Shetty, Pavanchand H Shetty, Amir Shiani, Desalegn Shiferaw, Mika Shigematsu, Min-Jeong Shin, Rahman Shiri, Aminu Shittu, Ivy Shiue, K M Shivakumar, Velizar Shivarov, Sina Shool, Seyed Afshin Shorofi, Rajan Shrestha, Sunil Shrestha, Kanwar Hamza Shuja, Kerem Shuval, Yafei Si, Emmanuel Edwar Siddig, Diego Augusto Santos Silva, Luís Manuel Lopes Rodrigues Silva, Soraia Silva, Thales Philipe R Silva, Colin R Simpson, Abhinav Singh, Balbir Bagicha Singh, Baljinder Singh, Garima Singh, Harmanjit Singh, Jasvinder A Singh, Mahendra Singh, Narinder Pal Singh, Paramdeep Singh, Surjit Singh, Robert Sinto, Shravan Sivakumar, Samarjeet Singh Siwal, Natia Skhvitaridze, Søren T Skou, David A Sleet, Farrukh Sobia, Matiwos Soboka, Bogdan Socea, Shahabaddin Solaimanian, Ranjan Solanki, Shipra Solanki, Sameh S M Soliman, Ranjani Somayaji, Yi Song, Reed J D Sorensen, Joan B Soriano, Ireneous N Soyiri, Michael Spartalis, Sandra Spearman, Cory N Spencer, Chandrashekhar T Sreeramareddy, Panagiotis Stachteas, Lauryn K Stafford, Jeffrey D Stanaway, Muhammad Haroon Stanikzai, Caroline Stein, Dan J Stein, Fridolin Steinbeis, Caitlyn Steiner, Sabine Steinke, Paschalis Steiropoulos, Leo Stockfelt, Mark A Stokes, Kurt Straif, Saverio Stranges, Narayan Subedi, Vetriselvan Subramaniyan, Muhammad Suleman, Rizwan Suliankatchi Abdulkader, Johan Sundström, David Sunkersing, Katharina S Sunnerhagen, Vinay Suresh, Chandan Kumar Swain, Lukasz Szarpak, Mindy D Szeto, Payam Tabaee Damavandi, Rafael Tabarés-Seisdedos, Seyyed Mohammad

Tabatabaei, Ozra Tabatabaei Malazy, Seyed-Amir Tabatabaeizadeh, Shima Tabatabai, Celine Tabche, Mohammad Tabish, Santosh Kumar Tadakamadla, Yasaman Taheri Abkenar, Moslem Taheri Soodejani, Amir Taherkhani, Jabeen Taiba, Ken Takahashi, Iman M Talaat, Jacques Lukenze Tamuzi, Ker-Kan Tan, Haosu Tang, Nathan Y Tat, Nuno Taveira, Yibekal Manaye Tefera, Arash Tehrani-Banihashemi, Worku Animaw Temesgen, Mohamad-Hani Temsah, Masayuki Teramoto, Dufera Rikitu Terefa, Enoch Teye-Kwadjo, Ramna Thakur, Pugazhenthan Thangaraju, Kavumpurathu Raman Thankappan, Rekha Thapar, Rasiah Thayakaran, Sathish Thirunavukkarasu, Nihal Thomas, Nikhil Kenny Thomas, Jing Tian, Ales Tichopad, Jansje Henny Vera Ticoalu, Tenaw Yimer Tiruye, Ruoyan Tobe-Gai, Musliu Adetola Tolani, Tadesse Tolossa, Marcello Tonelli, Roman Topor-Madry, Fotis Topouzis, Mathilde Touvier, Marcos Roberto Tovani-Palone, Khaled Trabelsi, Jasmine T Tran, Mai Thi Ngoc Tran, Nghia Minh Tran, Domenico Trico, Indang Trihandini, Christopher E Troeger, Samuel Joseph Tromans, Thien Tan Tri Tai Truyen, Aristidis Tsatsakis, Evangelia Eirini Tsermpini, Munkhtuya Tumurkhuu, Aniefiok John Udoakang, Arit Udoh, Atta Ullah, Saeed Ullah, Sana Ullah, Muhammad Umair, Srikanth Umakanthan, Brigid Unim, Bhaskaran Unnikrishnan, Era Upadhyay, Daniele Urso, Jibrin Sammani Usman, Asokan Govindaraj Vaithinathan, Omid Vakili, Mario Valenti, Rohollah Valizadeh, Jef Van den Eynde, Aaron van Donkelaar, Orsolya Varga, Priya Vart, Shoban Babu Varthya, Tommi Juhani Vasankari, Milena Vasic, Siavash Vaziri, Narayanaswamy Venketasubramanian, Nicholas Alexander Verghese, Madhur Verma, Massimiliano Veroux, Georgios-Ioannis Verras, Dominique Vervoort, Jorge Hugo Villafañe, Victor E Villalobos-Daniel, Leonardo Villani, Gabriela Ines Villanueva, Manish Vinayak, Francesco S Violante, Vasily Vlassov, Bay Vo, Stein Emil Vollset, Simona Ruxandra Volovat, Theo Vos, Isidora S Vujcic, Yasir Waheed, Cong Wang, Fang Wang, Shu Wang, Yanzhong Wang, Yuan-Pang Wang, Mary Njeri Wanjau, Muhammad Waqas, Paul Ward, Abdul Waris, Emebet Gashaw Wassie, Kosala Gayan Weerakoon, Robert G Weintraub, Daniel J Weiss, Eli J Weiss, Haftom Legese Legese Weldetinsaa, Katherine M Wells, Yi Feng Wen, Taweewat Wiangkham, Nuwan Darshana Wickramasinghe, Caroline Wilkerson, Peter Willeit, Shadrach Wilson, Yen Jun Wong, Utoomporn Wongsin, Sarah Wozniak, Chenkai Wu, Dongze Wu, Felicia Wu, Zenghong Wu, Juan Xia, Hong Xiao, Suowen Xu, Xiaoyue Xu, Yvonne Yiru Xu, Mukesh Kumar Yadav, Sajad Yaghoubi, Kazumasa Yamagishi, Lin Yang, Yuichiro Yano, Habib Yaribeygi, Yuichi Yasufuku, Pengpeng Ye, Renjulal Yesodharan, Subah Abderehim Yesuf, Saber Yezli, Siyan Yi, Arzu Yiğit, Zeamanuel Anteneh Yigzaw, Dehui Yin, Paul Yip, Malede Berihun Yismaw, Dong Keon Yon, Naohiro Yonemoto, Yuyi You, Mustafa Z Younis, Zabihollah Yousefi, Chuanhua Yu, Yong Yu, Siddhesh Zadey, Vesna Zadnik, Fathiah Zakham, Nazar Zaki, Josefina Zakzuk, Giulia Zamagni, Sojib Bin Zaman, Ghazal G Z Zandieh, Aurora Zanghì, Heather J Zar, Iman Zare, Fatemeh Zarimeidani, Mikhail Sergeevich Zastrozhin, Youjie Zeng, Chunxia Zhai, Anthony Lin Zhang, Haijun Zhang, Liqun Zhang, Meixin Zhang, Yunquan Zhang, Zhenyu Zhang, Zhi-Jiang Zhang, Hanqing Zhao, Jeff T Zhao, Xiu-Ju George Zhao, Yang Zhao, Yong Zhao, Chenwen Zhong, Jingjing Zhou, Juexiao Zhou, Shangcheng Zhou, Bin Zhu, Lei Zhu, Zhaohua Zhu, Boback Ziaeian, Makan Ziafati, Magdalena Zielińska, Stephanie R M Zimsen, Ghazal Zoghi, Thomas Zoller, Alimuddin Zumla, Sa'ed H Zyoud, Samer H Zyoud, Christopher J L Murray**, Emmanuela Gakidou**, *Joint first authors

**Joint senior authors

Affiliations

Institute for Health Metrics and Evaluation (Prof M Brauer DSc, G A Roth MD, A Y Aravkin PhD, P Zheng PhD, A Afshin MD, N Ahmad BS, J A Anderson BS, C M Antony MA, M B Arndt PhD, C Ashbaugh MA, M Assmus BA, G S Azhar PhD, F B Bennitt BA, J D Bishai BA, C Bisignano MPH, D Bryazka BA, K Burkart PhD, J Cagney MSc, K M Cercy BS, C S Chen BA, R M Cogen BA, A J Cohen DSc, J A Cruz BS, G T Culbreth PhD, X Dai PhD, N Davis Weaver MPH, Prof L Degenhardt PhD, K Estep MPA, Prof V L Feigin PhD, A J Ferrari PhD, L S Flor MPH, L M Force MD, V Garcia BS, W M Gardner MPH, G F Gil MPH, N M Gilbertson PhD, H Hagins MSPH, D Haile PhD, N Hashmeh MSc, Prof S I Hay FMedSci, C A Henson MPH, M E Herbert MSc, A Heuer MSc, J Hon MLS, J M Hsu BA, E N Hulland PhD, C K Huynh BA, A L Ihler PSM, K S Ikuta MD, C O Johnson PhD, E M Kahn MPH, D J Kapner PhD, N J Kassebaum MD, M B Kassel BA, L Kemmer PhD, K J Krohn MPH, K E LeGrand MPH, H Lescinsky BA, Prof S S Lim PhD, Prof R Lozano MD, K L Maass PhD, E Marsh MS, A W McKowen MA, S A McLaughlin PhD, T Mestrovic PhD, M E Moberg MS, A H Mokdad PhD, V Mougin BA, E C Mullany BA, Prof M Naghavi PhD, S I Nicholson MSc, E M O'Connell BA, K L Ong PhD, S M Ostroff PhD, M C Parent MPP, M Pasovic MEd, B V Pickering BS, D M Pigott PhD, Q Rafferty BA, P C Rao MPH, C Razo PhD, M B Reitsma BS, H E Robinson-Oden MLIS, J J Rosauer BA, T Runghien MSc, D F Santomauro PhD, A E Schumacher PhD, R J D Sorensen PhD, S Spearman MS, C N Spencer BA, L K Stafford MS, J D Stanaway PhD, C Stein PhD, C Steiner MPH, C E Troeger MPH, N A Verghese BA, Prof S Vollset DrPH, Prof T Vos PhD, E J Weiss BS, K M Wells BA, C Wilkerson MPH, S Wilson BS, S Wozniak MPH, Y Xu MPH, M Zhang MS, J T Zhao MS, S R M Zimsen MA, Prof C J L Murray DPhil, Prof E Gakidou PhD), Division of Cardiology (G A Roth MD), Department of Health Metrics Sciences, School of Medicine (G A Roth MD, A Y Aravkin PhD, P Zheng PhD, A Afshin MD, K Burkart PhD, X Dai PhD, L S Flor MPH, L M Force MD, N M Gilbertson PhD, Prof S I Hay FMedSci, N J Kassebaum MD, Prof S S Lim PhD, Prof R Lozano MD, A H Mokdad PhD, Prof M Naghavi PhD, M C Parent MPP, D M Pigott PhD, B Sartorius PhD, J D Stanaway PhD, C Stein PhD, Prof S Vollset DrPH, Prof T Vos PhD, Prof C J L Murray DPhil, Prof E Gakidou PhD), Department of Applied Mathematics (A Y Aravkin PhD), Department of Global Health (M B Arndt PhD, M Khalil BA, R J D Sorensen PhD), Division of Pediatric Hematology-Oncology (L M Force MD), Department of Neurology (R Kalani MD), Department of Anesthesiology & Pain Medicine (N J Kassebaum MD, V Krishnamoorthy MD), Division of Plastic and Reconstructive Surgery (S D Morrison MD), Henry M Jackson School of International Studies (S M Ostroff PhD), Department of Pharmacy (S Solaimanian BSN), Department of Medicine (R Somayaji MD), University of Washington, Seattle, WA, USA; School of Population and Public Health (Prof M Brauer DSc, P A Chakraborty MPH), Department of Pharmaceutical Sciences (A Adibi MSc), School of Nursing (A Pashaei MSc), University of British Columbia, Vancouver, BC, Canada; Department of Nutrition and Dietetics (K H Abate PhD), Department of Epidemiology (M B Ahmed MPH, D Shiferaw MPH), Department of Public Health (M Y Ashemo PhD, U Gerema MSc), Department of Surgery (N S Bayileyegn MD), Institute of Health Science (A I Mohamed MSc), Department of Psychiatry (M Soboka MSc), Jimma University, Jimma, Ethiopia; Department of Clinical Governance and Quality Improvement (Y H Abate MSc), Aleta Wondo Hospital, Aleta Wondo, Ethiopia; Department of Juridical and Economic Studies (C Abbafati PhD), Department of Public Health and Infectious Diseases (M S Cattaruzza PhD), La Sapienza University, Rome, Italy; Doheny Eye Institute (R Abbasgholizadeh MD), David Geffen School of Medicine (B Ziaeian MD), University of California Los Angeles, Los Angeles, CA, USA; Infectious and Tropical Research Center (M A Abbasi PhD), Research Center of Psychiatry and

Behavioral Sciences (H Azizi PhD), Department of Radiology (M Dashti MD, A Ghasemzadeh MD), Department of Health Policy and Management (L Doshmangir PhD), School of Nursing and Midwifery (H Hassankhani PhD), Department of Immunology (F Jadidi-Niaragh PhD), Faculty of Nursing and Midwifery (Prof M Mirghafourvand PhD), Neurosciences Research Center (NSRC) (R Mosaddeghi Heris MD), Student Research Committee (R Mosaddeghi Heris MD), Drug Applied Research Center (H Samadi Kafil PhD), Tabriz University of Medical Sciences, Tabriz, Iran; Department of Orthopedic Surgery (M Abbasian MD), Department of Pediatrics (S Aly MD), TH Chan School of Public Health (Prof T W Bärnighausen MD, P M S Pradhan MD), Center for Primary Care (S Basu PhD), Department of Global Health and Social Medicine (A W Bell MSW, M Pigeolet MD), Harvard Business School (F Caetano dos Santos PhD), Department of Epidemiology (S Carr MS), Division of Cardiovascular Medicine (G Chi MD), Harvard Medical School (A O Elmehrath PGCert), Department of Neurological Surgery (A H Feroze MD), Department of Ophthalmology (Prof J H Kempen MD), Department of Global Health and Population (Z Li PhD), Department of Health Policy and Oral Epidemiology (Z S Natto DrPH), Department of Physical Medicine and Rehabilitation (K Pacheco-Barrios MD), Beth Israel Deaconess Medical Center (S Sharfaei MD), Division of General Internal Medicine (Prof A Sheikh MD), Harvard University, Boston, MA, USA; Department of Orthopaedic Surgery (M Abbasian MD), Department of Biotechnology (S Aghamiri PhD), National Nutrition and Food Technology Research Institute (M Ajami PhD), Research Institute of Dental Sciences (Prof S Asgary MSc), Urology Department (M Bonakdar Hashemi MD), Internal Medicine Department (H Borhany MD), Department of Occupational Health and Safety Engineering (S Esmaeili PhD), Psychiatric Nursing and Management Department (F Ghadirian PhD), Faculty of Medicine (A Ghaffari Jolfayi MD), Department of Medical Genetics (M Ghasemi PhD), Center for Comprehensive Genetic Services (M Ghasemi PhD), Food Technology Research (Z Hadian PharmD), Research Institute for Endocrine Sciences (A Halimi BSc), Department of Immunology (K Jahankhani MSc, A Rasouli-Saravani PhD), Department of Health Policy and Management (N Jahanmehr PhD), Safety Promotion and Injury Prevention Research Center (N Jahanmehr PhD), Department of Neurosurgery (H Khayat Kashani MD), Ophthalmic Research Center (Z Khorrami PhD, S Safi PhD), Social Determinants of Health Research Center (A Kolahi MD, A Nikoobar DipSc, M Rashidi MD), School of Medicine (S Nejadghaderi MD), Student Research Committee (M Rahmanian MD), Department of Epidemiology (S Sabour PhD), Department of Health (M Safari PhD), Department of Anesthesiology (S Salimi MD), Emergency Department (S Shool MD), Department of Medical Education (S Tabatabai PhD), Shahid Beheshti University of Medical Sciences, Tehran, Iran; Department of Internal Medicine (M Abbasifard MD), Clinical Research Development Unit (M Abbasifard MD), Department of Epidemiology and Biostatistics (Prof M Rezaeian PhD), Rafsanjan University of Medical Sciences, Rafsanjan, Iran; Non-communicable Diseases Research Center (M Abbasi-Kangevari MD, S Azadnajafabad MD, M Bastan MD, P Mousavi MD, M Rashidi MD, N Rezaei MD, N Rezaei PhD), The Institute of Pharmaceutical Sciences (TIPS) (Prof M Abdollahi PhD, S Hassani PhD), School of Pharmacy (Prof M Abdollahi PhD), Research Center for Immunodeficiencies (H Abolhassani PhD), Universal Scientific Education and Research Network (USERN) (M Amirzade-Iranaq DDS), Digestive Diseases Research Institute (A Anoushiravani MD, S Fahimi MD, A Mohamadkhani PhD, Prof A Pourshams MD), Urology Research Center (R Arabzadeh Bahri MD), Department of Epidemiology and Biostatistics (H Azizi PhD, M Mansournia PhD), School of Medicine (A Behnoush BS, M Gouravani MD, H Karimi MD, A Khalaji BS, S Khanmohammadi MD, M Mayeli MD), Department of Scientific Research (F Chichagi MD), Iranian Research Center for HIV/AIDS (O Dadras DrPH), Department of Ophthalmology (S Ghafarian MD, Prof F Ghassemi MD), Sina Trauma and Surgery Research Center (M Hassan Zadeh Tabatabaei MD, A Khavandegar MD, M Khormali MD, Prof V Rahimi-

Movaghar MD, Prof P Salamati MD, S Shool MD), Center for Research and Training in Skin Diseases and Leprosy (F Khamesipour PhD), Children's Medical Center (F Kompani MD), Endocrinology and Metabolism Research Institute (Prof B Larijani FACE, N Rezaei PhD, O Tabatabaei Malazy PhD), Orthopedic Department (A Mafhoumi MD), Department of Cardiology (E Mahmoudi MD, P Mansouri MD, S Rashedi MD), Tehran Heart Center (E Mehrabi Nasab MD), Faculty of Nursing and Midwifery (A Mirshahi MSc), Faculty of Medicine (E Mohammadi MD), Department of Physiotherapy (Prof N Nakhostin Ansari PhD), Research Center for War-affected People (Prof N Nakhostin Ansari PhD), Department of Pharmacology (N Noroozi DVM, S Solaimanian BSN), Department of Neurosurgery (A Pour-Rashidi MD), Pharmaceutical Sciences Research Center (PSRC) (M Rahimifard PhD), Department of Neurology (M Shafie MD), Department of Medicine (A Shahbandi MD), Department of Pharmaceutical Care (A Sharifan PharmD), Research Center for Rational Use of Drugs (A Sharifan PharmD), Tehran University of Medical Sciences, Tehran, Iran (E Mohammadi MD); Department of Epidemiology (S Abd ElHafeez DrPH), Pediatric Dentistry and Dental Public Health Department (Prof O A A Elmeligy PhD), Department of Tropical Health and Parasitology (R M Ghazy PhD), Department of Pathology (Prof I M Talaat PhD), Alexandria University, Alexandria, Egypt; Department of Tropical Medicine and Infectious Diseases (S Abd-Elsalam PhD), Tanta University, Tanta, Egypt; Department of Medicine (P Abdi Beng), Memorial University, St John's, NL, Canada; Department of Medicine (Prof M Abdoun BMedSc), University of Setif Algeria, Sétif, Algeria; Community and Maternity Nursing Unit (D M Abdulah MPH), University of Duhok, Duhok, Iraq; Department of Physiotherapy (A Abdullahi PhD, J S Usman PhD), Department of Nursing Science (M Ladan PhD), Bayero University Kano, Kano, Nigeria; Department of Rehabilitation Sciences (A Abdullahi PhD, M U Ali MSc, M Khan MPH, J S Usman PhD), Hong Kong Polytechnic University, Hong Kong, China; Department of Midwifery (M Abebe MSc), Department of Psychiatry (C K Demoze MS), Department of Public Health (T L Lerango MPH), Dilla University, Dilla, Ethiopia; Department of Neurosurgery (A Abedi MD), Keck School of Medicine (A Abedi MD), Department of Radiology (A Gholamrezanezhad MD), Mark and Mary Stevens Neuroimaging and Informatics Institute (S Salehi MD), University of Southern California, Los Angeles, CA, USA; Department of Emergency Medicine (A Abedi MD), Department of Immunology (S Athari PhD), Department of Critical Care and Emergency Nursing (N Hanifi PhD), Zanjan University of Medical Sciences, Zanjan, Iran; Department of Clinical Pharmacy (T M Abegaz MS), Department of Pharmacology (Z D Kifle MSc), University of Gondar, Gondar, Ethiopia; College of Pharmacy and Pharmaceutical Sciences (T M Abegaz MS), Florida Agricultural and Mechanical University, Tallahassee, FL, USA; Postgraduate Department (Prof R A Abeldaño Zuñiga PhD), University of Sierra Sur, Miahuatlan de Porfirio Diaz, Mexico; National Research Council of Mexico, Mexico City, Mexico (Prof R A Abeldaño Zuñiga PhD); Department of Community Medicine (O Abiodun MPH), Babcock University, Ilishan-Remo, Nigeria; School of Public Health (T L Abiso MPH), Wolaita Sodo University, Wolaita Sodo, Ethiopia; Department of Family and Community Health (R G Aboagye MPH, F Agbozo PhD), Department of Epidemiology and Biostatistics (L A Adzigbli BSc, R K Dowou MPhil), Institute of Health Research (R K Alhassan PhD, M Immurana PhD), Department of Population and Behavioural Sciences (H Amu PhD, E Manu PhD), Department of Health Policy Planning and Management (M K Boachie PhD), Department of Microbiology and Immunology (V N Orish PhD), University of Health and Allied Sciences, Ho, Ghana; Department of Medical Biochemistry and Biophysics (H Abolhassani PhD), Department of Neurobiology, Care Sciences and Society (Prof J Ärnlöv PhD), Department of Medical Epidemiology and Biostatistics (Prof J J Carrero PhD), Department of Physiology and Pharmacology (C R Cederroth PhD), Department of Neurobiology, Care Sciences, and Society (S Fereshtehnejad PhD), Department of Molecular Medicine and Surgery (Prof J H Kauppila MD),

Department of Global Public Health (Prof L Laflamme PhD), Karolinska Institute, Stockholm, Sweden; Department of Physical Pharmacy and Pharmacokinetics (M Abouzid PharmD), Poznan University of Medical Sciences, Poznan, Poland; Department of Public Health (G B Aboye MSc), Madda Walabu University, Addis Ababa, Ethiopia; Department of Nutrition and Dietetics (G B Aboye MSc), Jimma University, Addis Ababa, Ethiopia; Department of Pediatric Dentistry (Prof L G Abreu PhD), Department of Internal Medicine (Prof L C Brant PhD, Prof A P Ribeiro MD), School of Nursing (A G da Silva PhD), Department of Maternal-Child Nursing and Public Health (Prof D C Malta PhD, Prof F P Matozinhos PhD, Prof A C Micheletti Gomide Nogueira de Sá MSc, E J S Prates BS), Department of Clinical Medicine (Prof B R Nascimento PhD), Clinical Hospital (Prof B R Nascimento PhD), Centre of Telehealth (Prof A P Ribeiro MD), Vaccination Research Observatory (T R Silva PhD), Federal University of Minas Gerais, Belo Horizonte, Brazil; Department of Nursing (H Abualruz PhD), Al Zaytoonah University of Jordan, Amman, Jordan; Department of Pharmacology and Toxicology (B Abubakar PhD), Department of Veterinary Microbiology (M B Bello PhD), Department of Veterinary Public Health and Preventive Medicine (A Shittu MSc), Usmanu Danfodiyo University, Sokoto, Sokoto, Nigeria; Clinical Science Department (Prof M O Folayan FWACS), Nigerian Institute of Medical Research, Lagos, Nigeria (B Abubakar PhD); Clinical Sciences Department (E Abu-Gharbieh PhD, H J Barqawi MPhil, N R Dash MD, Prof R Halwani PhD, Prof A A Maghazachi PhD, M M Saber-Ayad MD, N Saheb Sharif-Askari PhD, Prof I M Talaat PhD), College of Medicine (F Ahmad PhD, Prof R Halwani PhD, Prof B Saddik PhD), Department of Pharmacy Practice and Pharmacotherapeutics (Prof K H Alzoubi PhD, Prof H A Omar PhD), Department of Physiotherapy (A Arumugam PhD), Department of Basic Biomedical Sciences (Y Bustanji PhD), Sharjah Institute for Medical Research (N M Elemam PhD), Department of Clinical Nutrition and Dietetics (M E M Faris PhD), Department of Basic Medical Sciences (A Karim PhD), Department of Clinical Science (Prof M M Ramadan PhD), Sharjah Institute of Medical Sciences (F Saheb Sharif-Askari PhD), College of Pharmacy (Prof M H Semreen PhD), Research Institute of Medical & Health Sciences (Prof M H Semreen PhD), Department of Medicinal Chemistry (S S M Soliman PhD), University of Sharjah, Sharjah, United Arab Emirates (K A Altirkawi MD); Research Department (H J J Abukhadijah MPH), Department of Nursing Education & Research (A J Nashwan MSc), Department of Geriatric and Long Term Care (B Sathian PhD), Hamad Medical Corporation, Doha, Qatar; Department of Therapeutics (Prof S Aburuz PhD), Institute of Public Health (L A Ahmed PhD, R H Al-Rifai PhD, Prof S M Shah PhD), College of Medicine and Health Sciences (Prof M Grivna PhD, J Nauman PhD), Family Medicine Department (M A Khan MSc), Department of Computer Science and Software Engineering (Prof N Zaki PhD), United Arab Emirates University, Al Ain, United Arab Emirates; College of Pharmacy (Prof S Aburuz PhD), University of Jordan, Amman, Jordan; Department of Surgery (A Abu-Zaid MD), College of Medicine (S Almustanyir MD), Alfaisal University, Riyadh, Saudi Arabia; College of Graduate Health Sciences (A Abu-Zaid MD), University of Tennessee, Memphis, TN, USA; College of Medicine and Health Sciences (M M Adane PhD, A A Awoke MSc), Department of Epidemiology and Biostatistics (Y Alemu MPH), Department of Health Promotion and Behavioural Science (E K Bogale MPH), Department of Nursing (W A Temesgen PhD), Department of Health Promotion and Behavioral Science (Z A Yigzaw MPH), Department of Pharmacy (M Yismaw MSc), Bahir Dar University, Bahir Dar, Ethiopia; Centre for Social Research in Health (I Y Addo PhD, S R Okeke PhD), St George and Sutherland Clinical School (H Akbarialiabad MD), School of Public Health and Community Medicine (B Akombi-Inyang PhD, A E Peden PhD, Prof A E Schutte PhD), Transport and Road Safety (TARS) Research Centre (S Boufous PhD), School of Population Health (Z Dai PhD, X Feng PhD, X Xu PhD), National Drug and Alcohol Research Centre (Prof L Degenhardt PhD), School of Clinical Medicine (M Huda PhD), School of Medicine (P K Maulik PhD), School of Psychiatry (Prof P B

Mitchell MD, Prof P S Sachdev MD), Centre for Primary Health Care and Equity (CPHCE) (P Peprah MSc), School of Optometry and Vision Science (Prof K Pesudovs PhD, Prof S Resnikoff MD), School of Risk and Actuarial Studies (Y Si PhD), The George Institute for Global Health (P Ye MPH), University of New South Wales, Sydney, NSW, Australia; Quality and Systems Performance Unit (I Y Addo PhD), Cancer Institute NSW, Sydney, NSW, Australia; Internal Medicine and Alcohol Related Disease Unit (Prof G Addolorato MD), Department of Woman and Child Health and Public Health (D Buonsenso MD), Fondazione Policlinico Universitario A Gemelli IRCCS (Agostino Gemelli University Polyclinic IRCCS), Rome, Italy; Department of Medical and Surgical Sciences (Prof G Addolorato MD), Università Cattolica di Roma (Catholic University of Rome), Rome, Italy; Department of Medical Rehabilitation (Prof R A Adedoyin PhD), Department of Child Dental Health (Prof M O Folayan FWACS), Obafemi Awolowo University, Ile-Ife, Nigeria; Department of Obstetrics and Gynecology (V Adekanmbi PhD), University of Texas Medical Branch, Galveston, TX, USA; College of Medicine and Health Sciences (K Al-Habbal MD), Department of Biology (W M S Osman PhD), Khalifa University, Abu Dhabi, United Arab Emirates (B Aden PhD); Institute of Public Health (B Aden PhD), Walden University, Al Ain, United Arab Emirates; Department of Biochemistry (J B Adetunji PhD), Osun State University, Osogbo, Nigeria; Department of Biosciences and Biotechnology (T E Adeyeoluwa PhD, I T Oyeyemi PhD, O T Oyeyemi PhD, A J Udoakang PhD), Department of Environmental and Occupational Health (B S Anuoluwa MPH, P U Daikwo MSc), Department of Microbiology (O O Bello PhD, Y D Oluwafemi PhD), Department of Biological Sciences (T C Ekundayo PhD), Department of Chemistry (O O Idowu MSc), University of Medical Sciences, Ondo, Ondo, Nigeria; Department of Veterinary Medicine (T E Adeyeoluwa PhD), Department of Community Medicine (A A Afolabi MPH, O S Ilesanmi PhD), Department of Epidemiology and Medical Statistics (R F Afolabi PhD, A F Fagbamigbe PhD), Department of Periodontology and Community Dentistry (O F Fagbule FWACS), Department of Health Promotion and Education (S E Ibitoye MPH, A Ogunkoya MPH), College of Medicine (A P Okekunle PhD), Faculty of Public Health (I I Olufadewa MHS), Department of Medicine (Prof M O Owolabi DrM), University of Ibadan, Ibadan, Nigeria; Department of Business Administration (R Adha PhD), Muhammadiyah University of Mataram, Mataram, Indonesia; Faculty of Medicine (Q E S Adnani PhD), Center of Excellence in Higher Education for Pharmaceutical Care Innovation (Prof M J Postma PhD), Universitas Padjadjaran (Padjadjaran University), Bandung, Indonesia; Department of Radiology (S Afyouni PhD, G G Z Zandieh MD), School of Medicine (I Ali BS), Anesthesia and Critical Care Medicine (S Boppana MD), Department of Biostatistics (A Columbus MS), School of Nursing (T K Hinneh MSc), Department of Neurosurgery (F Kazemi MD), Department of Medicine (E Sadeghi Majd MD), Department of Health Policy and Management (D Vervoort MD), Department of International Health (H Zhang MS), Johns Hopkins University, Baltimore, MD, USA; Department of Life Sciences (M S Afzal PhD, M Umair PhD), University of Management and Technology, Lahore, Pakistan; Department of Community Medicine (Prof S Afzal PhD), King Edward Memorial Hospital, Lahore, Pakistan; Department of Public Health (Prof S Afzal PhD), Public Health Institute, Lahore, Pakistan; Department of Community Medicine (Prof S B Agampodi MD, N D Wickramasinghe MD), Department of Parasitology (Prof K G Weerakoon PhD), Rajarata University of Sri Lanka, Anuradhapura, Sri Lanka; New Initiatives (Prof S B Agampodi MD), International Vaccine Institute, Seoul, South Korea; Department of Global and Public Health (F Agbozo PhD), School of Population and Global Health (Prof F J Elgar PhD), Department of Epidemiology, Biostatistics and Occupational Health (J Rana MPH), McGill University, Montreal, QC, Canada; Department of Medical and Surgical Sciences and Advanced Technologies "GF Ingrassia" (Prof A Agodi PhD, M Barchitta PhD, E D'Amico MD, A Maugeri PhD, Prof M Veroux PhD), Department of Biomedical and Biotechnological Sciences (L Falzone PhD, G Grosso PhD), Department of

General Surgery and Medical-Surgical Specialties (Prof G Isola PhD), Department of Clinical and Experimental Medicine (C Ledda PhD), University of Catania, Catania, Italy; Trivedi School of Biosciences (Prof A Agrawal PhD), Ashoka University, Sonipat, India; Section of General Internal Medicine (Prof A Agrawal PhD), Baylor College of Medicine, Houston, TX, USA; Department of Geography and Planning (W Agyemang-Duah MSc), Department of Biomedical and Molecular Sciences (A Nikpoor PhD), Queen's University, Kingston, ON, Canada; School of Public Health (B O Ahinkorah MPhil), School of Nursing and Midwifery (M Chutiyami PhD), School of Life Sciences (G Liu PhD), University of Technology Sydney, Sydney, NSW, Australia; Department of Medical Biochemistry (A Ahmad PhD), Department of Pediatrics (Prof G Mustafa MD), Department of Pharmacology (M Tabish MPharm), Shaqra University, Shaqra, Saudi Arabia; School of Medicine and Psychology (D Ahmad PhD), National Centre for Epidemiology and Population Health (Y Alemu MPH), Research School of Population Health (N Bagheri PhD, R A Burns PhD, Prof N Cherbuin PhD), Australian National University, Canberra, ACT, Australia; Public Health Foundation of India, Gandhinagar, India (D Ahmad PhD); Department of Medical Oncology (S Ahmad MD, A A Khosla MD), Department of Medicine (M Ganiyani MD), Miami Cancer Institute, Miami, FL, USA; Department of Community Medicine and Preventive Health (S Ahmad MD), King Edward Medical University Lahore, Lahore, Pakistan; Department of Epidemiology and Health Statistics (T Ahmad MS), Southeast University, Nanjing, China; Department of Pharmacy Practice (A Ahmed PhD), Riphah Institute of Pharmaceutical Sciences, Islamabad, Pakistan; Division of Infectious Diseases and Global Public Health (IDGPH) (A Ahmed PhD), University of California San Diego, San Diego, CA, USA; Maternal and Child Health Division (A Ahmed MS, A Sayeed MSc), International Centre for Diarrhoeal Disease Research, Bangladesh, Dhaka, Bangladesh; Department of Women's and Children's Health (A Ahmed MS), Department of Surgical Sciences (M Hultström PhD), Department of Medical Cell Biology (M Hultström PhD), Department of Medical Sciences (Prof A O Larsson PhD, Prof J Sundström PhD), Uppsala University, Uppsala, Sweden; Institute of Endemic Diseases (A Ahmed MSc), Unit of Basic Medical Sciences (E E Siddig MD), University of Khartoum, Khartoum, Sudan; Swiss Tropical and Public Health Institute (A Ahmed MSc), University of Basel, Basel, Switzerland; Australian Center for Precision Health (M B Ahmed MPH), Department of Allied Health and Human Performance (T Y Tiruye PhD), University of South Australia, Adelaide, SA, Australia; Department of Biochemistry (S Ahmed BSc), Jamia Hamdard, Delhi, India; Brody School of Medicine (S Ahmed PhD), Department of Computer Science (A O Bodunrin MSc), Department of Cardiovascular Sciences (A Ghajar MD), Department of Physiology (M Tumurkhuu PhD), East Carolina University, Greenville, NC, USA; Department of Food and Nutrition Policy and Planning Research (M Ajami PhD), Department of Food Science and Nutrition (M Aman Mohammadi PhD), National Nutrition and Food Technology Research Institute, Tehran, Iran; Department of Microbiology, Immunology and Parasitology (G T Akalu MSc), St Paul's Hospital Millennium Medical College, Addis Ababa, Ethiopia; Department of Microbial, Cellular, and Molecular Biology (G T Akalu MSc), Department of Medical Laboratory Science (M Getie MSc), Addis Ababa University, Addis Ababa, Ethiopia; Moyen Mono Health District (E Akara MD), Ministry of Health, Tohoun, Togo; Toxicology Research Center (S Akhlaghi PhD), Department of Orthodontics (E Eini DDS), Environmental Technologies Research Center (N Kaydi PhD), Department of Public Health (M A Khafaie PhD), Education Development Center (M Sayyah MD), Ahvaz Jundishapur University of Medical Sciences, Ahvaz, Iran; Department of Internal Medicine (K Akinosoglou PhD), University of Patras, Patras, Greece; Department of Internal Medicine and Infectious Diseases (K Akinosoglou PhD), University General Hospital of Patras, Patras, Greece; Department of Population Health Sciences (T Akinyemiju PhD), Duke Global Health Institute (T Akinyemiju PhD, C Wu PhD), Department of Anesthesiology (V Krishnamoorthy MD), Duke

University, Durham, NC, USA; Department of Cardiology (M A Akkaif PhD), Fudan University, Shanghai, China; Department of Management, Policy, and Community Health (S Akkala MPH), Health Science Center (D Dongarwar MS), University of Texas, Houston, TX, USA; Department of Communicable Diseases (S Al Awaidy MSc), Ministry of Health, Muscat, Oman; Middle East, Eurasia, and Africa Influenza Stakeholders Network, Muscat, Oman (S Al Awaidy MSc); Division of Public Health Sciences (S Al Hasan PhD), Washington University School of Medicine, St Louis, MO, USA; McWilliams School of Biomedical Informatics (F Alahdab MD), UTHealth, Houston, TX, USA; Department of Biomedical Informatics, Biostatistics, and Epidemiology (F Alahdab MD), University of Missouri, Columbia, MO, USA; Heidelberg Institute of Global Health (HIGH) (T M A AL-Ahdal MPH, Prof T W Bärnighausen MD, S Barteit PhD, S Chen DSc, B Moazen MSc), Heidelberg University, Heidelberg, Germany; Department of Clinical Sciences (S O Alalalmeh BPharm, O E Hegazi BPharm), Center for Medical and Bio-Allied Health Sciences Research (Prof M J Shahwan PhD, M A Shamsi PhD, S H Zyoud PhD), Ajman University, Ajman, United Arab Emirates; Department of Biology (T A Alalwan PhD, Prof S Perna PhD), College of Health and Sport Sciences (A G Vaithinathan MSc), University of Bahrain, Zallaq, Bahrain; John T Milliken Department of Internal Medicine (Z Al-Aly MD), Department of Surgery (T Lan PhD, C Wang MPH), Department of Energy, Environmental, and Chemical Engineering (A van Donkelaar PhD), Brown School (C Wang MPH), Washington University in St Louis, St Louis, MO, USA; Clinical Epidemiology Center (Z Al-Aly MD), US Department of Veterans Affairs (VA), St Louis, MO, USA; Murdoch Business School (K Alam PhD), Murdoch University, Perth, WA, Australia; Department of Public Health (Prof N Alam DrPH), Asian University for Women, Chittagong, Bangladesh; Asian University for Women (Prof N Alam DrPH), McGill University, Chittagong, Bangladesh; Department of Health Information Management and Technology (T M Alanzi PhD), Division of Forensic Medicine (Prof R G Menezes MD), Imam Abdulrahman Bin Faisal University, Dammam, Saudi Arabia (F M Alanezi PhD); Department of Medicine (A Albakri MD), Royal Jordanian Medical Services, Amman, Jordan; Faculty of Medicine (Prof M T AlBataineh PhD), Department of Basic Sciences (Z Altaany PhD), Department of Basic Medical Sciences (M M Khatatbeh PhD), Yarmouk University, Irbid, Jordan; Division of Gastroenterology and Hepatology (W A Aldhaleei MD), Mayo Clinic, Jacksonville, FL, USA; Institute of Health Informatics (R W Aldridge PhD), Department of Health Informatics (S Chung PhD), Department of Behavioural Science and Health (S Hossain MS), Department of Population Health Sciences (D Sunkersing PhD), Department of Infection (Prof A Zumla PhD), University College London, London, UK; Department of Epidemiology (M Alemayohu MPH), Department of Environmental Health (A A Asgedom PhD), Mekelle University, Mekelle, Ethiopia; Unit of Epidemiology & Medical Statistics (M Alemayohu MPH), University of Verona, Verona, Italy; Department of Neurology (B Al-Fatly MSc, S Samadzadeh MD), Institute of Public Health (F Fischer PhD), Department of Surgery (N Haep MD), Department of Infectious Diseases and Respiratory Medicine (F Steinbeis MD, T Zoller DrHabil), Charité Universitätsmedizin Berlin (Charité Medical University Berlin), Berlin, Germany; Global Centre for Environmental Remediation (A A S Al-Gheethi PhD), School of Medicine and Public Health (P Atorkey MPhil), University of Newcastle, Newcastle, NSW, Australia; Cooperative Research Centre for Contamination Assessment and Remediation of the Environment, Newcastle, NSW, Australia (A A S Al-Gheethi PhD); Department of Cardiac Sciences (Prof K F Alhabib MD), Section of Adult Hematology (Prof G M T ElGohary MD), Department of Physiology (Prof S A Meo PhD), Pediatric Intensive Care Unit (M Temsah MD), King Saud University, Riyadh, Saudi Arabia; Department of Zoology (A Ali PhD), Abdul Wali Khan University Mardan, Mardan, Pakistan; Department of Biotechnology and Genetic Engineering (A Ali PhD, M Waqas PhD), Hazara University Mansehra, Mansehra, Pakistan; Erbil Technical Health College (B A Ali PhD), Erbil Polytechnic University, Erbil, Iraq; School of Pharmacy (B A

Ali PhD), Tishk International University, Erbil, Iraq; Department of Biological Sciences (L Ali PhD, A S Qazi PhD), National University of Medical Sciences (NUMS), Rawalpindi, Pakistan; Department of Medical Rehabilitation (Physiotherapy) (M U Ali MSc), Department of Microbiology (M A Isa PhD), University of Maiduguri, Maiduguri, Nigeria; Department of Biosciences (R Ali MPhil, N Salam PhD), Centre for Interdisciplinary Research in Basic Sciences (CIRBSc) (S Anwar PhD), Centre For Interdisciplinary Research In Basic Sciences (CIRBSc) (M A Shamsi PhD), Jamia Millia Islamia, New Delhi, India; Center for Biotechnology and Microbiology (S S Ali PhD, M Suleman PhD), University of Swat, Swat, Pakistan; Department of Geography (W Ali PhD), Sulltan Qaboos University, Muscat, Oman; Department of Pathophysiology and Transplantation (G Alicandro PhD), Department of Food, Environmental and Nutritional Sciences (C Del Bo' PhD), Università degli Studi di Milano (University of Milan), Milan, Italy; Cystic Fibrosis Center (G Alicandro PhD), Fondazione IRCCS Ospedale Maggiore Policlinico (IRCCS Ca' Granda Maggiore Polyclinic Hospital Foundation), Milan, Italy; School of Public Health and Preventive Medicine (S M Alif PhD), Department of Medical Radiation Sciences (Prof A M Asbeutah PhD), School of Psychological Sciences (N Parsons PhD), Department of Medicine (Prof K R Polkinghorne PhD), Monash University, Melbourne, VIC, Australia; Department of Health Policy and Management (Prof S M Aljunid PhD), Department of Surgery (S K Al-Sabah MD), Department of Radiologic Sciences (Prof A M Asbeutah PhD), Kuwait University, Kuwait, Kuwait; International Centre for Casemix and Clinical Coding (Prof S M Aljunid PhD), National University of Malaysia, Bandar Tun Razak, Malaysia; Bordeaux School of Public Health (Prof F Alla PhD), University of Bordeaux, Bordeaux, France; Department of Dentistry (S Al-Marwani MSc), Independent Consultant, Sana'a, Yemen; Public Health and Community Medicine (S Al-Marwani MSc), Independent Consultant, Irbid, Jordan; Department of Parasitology (Prof H M Al-Mekhlafi PhD), Department of Medicine (L Lim MRCP), Faculty of Medicine (H Shah MS), University of Malaya, Kuala Lumpur, Malaysia; Department of Parasitology (Prof H M Al-Mekhlafi PhD), Sana'a University, Sana'a, Yemen; Ministry of Health, Riyadh, Saudi Arabia (S Almustanyir MD); Department of Physical Education (Prof M A Alomari PhD), QU Health (M Mohammed PhD), Social and Economic Survey Research Institute (Prof A Perianayagam PhD), Department of Population Medicine (Prof G Rathnaiah Babu PhD), Qatar University, Doha, Qatar; Department of Rehabilitation Sciences and Physical Therapy (Prof M A Alomari PhD), Department of Allied Medical Sciences (A Alrawashdeh PhD), Department of Rehabilitation Sciences (M S Al-Wardat PhD), Department of Clinical Pharmacy (Prof K H Alzoubi PhD), Department of Public Health (Prof Y S Khader PhD), Jordan University of Science and Technology, Irbid, Jordan; Research Program of Epidemiology and Public Health (J Alonso MD), Pompeu Fabra University, Barcelona, Spain; Department of Experimental and Health Sciences (J Alonso MD), Biomedical Research Networking Center in Epidemiology and Public Health (CiberESP), Madrid, Spain; Department of Respiratory Care (J S Alqahtani PhD), Prince Sultan Military College of Health Sciences, Dammam, Saudi Arabia; Department of Prosthodontics and Implant Dentistry (A Alqutaibi PhD), Taibah University, Medinah, Saudi Arabia; Department of Prosthodontics (A Alqutaibi PhD), Ibb University, Ibb, Yemen; Department of Community Medicine (R M Al-Raddadi PhD), Department of Family and Community Medicine (N S Butt PhD), Department of Pediatric Dentistry (Prof O A A Elmeligy PhD), Rabigh Faculty of Medicine (A A Malik PhD), Department of Dental Public Health (Z S Natto DrPH), King Abdulaziz University, Jeddah, Saudi Arabia; Macro-Fiscal Policy Department (S M Alrousan PhD), Ministry of Finance, Dubai, United Arab Emirates; Jaber Al Ahmad Al Sabah Hospital (S K Al-Sabah MD), Ministry of Health, Kuwait, Kuwait; Department of Family and Community Medicine (N Z Alshahrani MD), University of Jeddah, Jeddah, Saudi Arabia; Institute of Molecular Biology and Biotechnology (A Altaf PhD, S Shahid PhD), University College of Medicine & Dentistry (Prof M Arooj PhD), University Institute of Radiological

Sciences and Medical Imaging Technology (T Ashraf MS), University Institute of Public Health (S Hameed MPH, A Hanif PhD, A A Malik PhD), Department of Technology (M Muzaffar MBA), Research Centre for Health Sciences (RCHS) (M Muzaffar MBA, S Shahid PhD), Department of Physics (W Shahid PhD), University Institute of Medical Lab Technology (S Shehzadi PhD), The University of Lahore, Lahore, Pakistan (M A Riaz MCom); Department of Specialty Internal Medicine (Prof J A Al-Tawfiq MD), Johns Hopkins Aramco Healthcare, Dhahran, Saudi Arabia; Department of Medicine (Prof J A Al-Tawfiq MD), Indiana University School of Medicine, Indianapolis, IN, USA; Lisbon Institute of Global Mental Health (D O Aluh MSc), Nova University of Lisbon, Lisbon, Nigeria; Clinical Pharmacy and Pharmacy Management (D O Aluh MSc), University of Nigeria Nsukka, Nsukka, Nigeria; Research Group in Hospital Management and Health Policies (Prof N Alvis-Guzman PhD), Department of Economic Sciences (N J Alvis-Zakzuk MSc), Universidad de la Costa (University of the Coast), Barranquilla, Colombia; Research Group in Health Economics (Prof N Alvis-Guzman PhD), Institute for Immunological Research (Prof J Zakzuk PhD), University of Cartagena, Cartagena, Colombia; National Health Observatory (N J Alvis-Zakzuk MSc), Colombian National Health Observatory (C A Castañeda-Orjuela MD), National Institute of Health, Bogota, Colombia; Department of Clinical Pharmacology and Toxicology (H Alwafi PhD), Department of Medical Genetics (M Athar PhD), Science and Technology Unit (M Athar PhD), Umm Al-Qura University, Makkah, Saudi Arabia; Department of Medical Sciences (Prof Y M Al-Worafi PhD), Azal University for Human Development, Sana'a, Yemen; Department of Clinical Sciences (Prof Y M Al-Worafi PhD), University of Science and Technology of Fujairah, Fujairah, United Arab Emirates; Department of Pediatrics (Prof H Aly MD), Department of Internal Medicine (M Gupta MD), Heart, Vascular, and Thoracic Institute (A Hajj Ali MD), Lerner Research Institute (X Liu PhD), Cleveland Clinic, Cleveland, OH, USA; Department of Pediatric Cardiology (S Aly MD), Boston Children's Hospital, Boston, MA, USA; Department of Biomedical Engineering (W Al-Zyoud PhD), German Jordanian University, Amman, Jordan; Evaluation Unit (U A Amaechi BDS), Global Alliance for Vaccines and Immunisations, Geneva, Switzerland; Interdisciplinary Graduate Program in Human Toxicology (R Amani DVM), University of Iowa, Iowa City, IA, USA; Health Policy Research Center (R Amani DVM, H Kasraei MD, Y Sarikhani PhD), Health Human Resources Research Center (M Bayati PhD), Trauma Research Center (M Karajizadeh PhD), Department of Epidemiology and Biostatistics (H Raeisi Shahraki PhD), Autophagy Research Center (O Vakili PhD), Shiraz University of Medical Sciences, Shiraz, Iran; Medicine, Quran and Hadith Research Center (S Amiri PhD), Nephrology and Urology Research Center (K Hushmandi PhD), Baqiyatallah University of Medical Sciences, Tehran, Iran; De Gasperis Cardio Center (E Ammirati MD), Niguarda Hospital, Milano, Italy; Department of Maternal and Child Wellbeing (D A Amugsi PhD), African Population and Health Research Center, Nairobi, Kenya; Department of Medicine (G A Amusa MD), University of Jos, Jos, Nigeria; Department of Internal Medicine (G A Amusa MD), Jos University Teaching Hospital, Jos, Nigeria; Faculty of Pharmacy (Prof R Ancuceanu PhD), Department of Cardiology (C Andrei PhD), Department of General Surgery (D V Davitoiu PhD, A Manda MD, I Negoi PhD, D Serban PhD, B Socea PhD), Department of Internal Medicine (M Hostiuc PhD), Department of Legal Medicine and Bioethics (S Hostiuc PhD), Department of Anatomy and Embryology (R I Negoi PhD), Department of Diabetes, Nutrition and Metabolic Diseases (A Pantea Stoian PhD), Carol Davila University of Medicine and Pharmacy, Bucharest, Romania; Centre for Sensorimotor Performance (D Anderlini MD), Department of Urology (Prof E Chung MD), School of Public Health (A J Ferrari PhD, D F Santomauro PhD), Institute for Social Science Research (E Kanmiki MPH, A A Mamun PhD, J C Maravilla PhD), School of Health and Rehabilitation Sciences (A Khan PhD, M Moni PhD), Queensland Brain Institute (Prof J J McGrath MD), Faculty of Medicine (B Sartorius PhD), The University of Queensland, Brisbane, QLD,

Australia; Neurology Department (D Anderlini MD), Royal Brisbane and Women's Hospital, Brisbane, QLD, Australia; Department of Health Care Management (P P Andrade MD, Prof R Busse PhD, S Mohammed PhD), Technical University of Berlin, Berlin, Germany; European University, Lisbon, Portugal (P P Andrade MD); Department of Statistics and Econometrics (Prof T Andrei PhD, Prof M Ausloos PhD, Prof C Herteliu PhD, A Mirica PhD, A Otoiu PhD, I Petcu PhD), Faculty of Management (A Dima PhD), Bucharest University of Economic Studies, Bucharest, Romania; Department of Environmental and Occupational Health (S C Anenberg PhD), George Washington University, Washington, DC, USA; Department of Child Neurology (D Angappan MD), Oregon Health and Science University, Portland, OR, USA; School of Health and Related Research (C Angus MSc), University of Sheffield, Sheffield, UK; Department of Pharmacology (A Anil MD, A Saravanan MD, M Shamim MBBS, S Singh MD, S B Varthya MD), Department of Anatomy (Prof N Bhardwaj MD), Department of Community Medicine and Family Medicine (P Bhardwaj MD, Prof P R Raghav MD), School of Public Health (P Bhardwaj MD), Department of Forensic Medicine and Toxicology (T Kanchan MD), Burns and Plastic Surgery (S Karmakar MCh), Department of Anaesthesiology and Critical Care (N Kothari PhD), Department of Community Medicine (G Singh MD), Department of Urology and Kidney Transplant (M Singh MCh Urology), All India Institute of Medical Sciences, Jodhpur, India; All India Institute of Medical Sciences, Bhubaneswar, India (A Anil MD); Department of Obstetrics and Gynecology (S Anil MBBS), Ernakulam Medical Centre, Kochi, India; Department of Psychiatry (A Anjum BHlthSci), MRC Epidemiology Unit (H Khreis PhD), Department of Public Health and Primary Care (Prof P Willeit PhD), University of Cambridge, Cambridge, UK; Research Center on Public Health (I Antonazzo PhD, P Ferrara MD), School of Medicine and Surgery (P A Cortesi PhD, Prof L G Mantovani DSc), University of Milan Bicocca, Monza, Italy; Agribusiness Study Program (E Antriyandarti DrAgrSc), Sebelas Maret University, Surakarta, Indonesia; Department of Parasitology (D Anvari PhD), Department of Dermatology (N Gholizadeh MD), Department of Biostatistics (Prof A Khalilian PhD), Department of Immunology (Prof A Rafiei PhD), Molecular and Cell Biology Research Center (Prof A Rafiei PhD), Department of Medical-Surgical Nursing (S Shorofi PhD), Department of Environmental Health (Prof Z Yousefi PhD), Mazandaran University of Medical Sciences, Sari, Iran; Department of Parasitology (D Anvari PhD), Iranshahr University of Medical Sciences, Iranshahr, Iran; Regenerative Medicine, Organ Procurement and Transplantation Multi-disciplinary Center (S Anvari MD), Gastrointestinal and Liver Diseases Research Center (S Hassanipour PhD), Caspian Digestive Disease Research Center (S Hassanipour PhD), Department of Environmental Health Engineering (J Jaafari PhD), Inflammatory Lung Diseases Research Center (N Rahmanian PhD), Guilan University of Medical Sciences, Rasht, Iran; School of Chemical and Life Sciences (SCLS) (S Anwar PhD), Jamia Hamdard, New Delhi, India; Department of Surgery (S Anwar PhD), Department of Pharmacology (I Fitriana PhD), Gadjah Mada University, Yogyakarta, Indonesia; Department of Pathology (R Anwer PhD), Imam Mohammad Ibn Saud Islamic University, Riyadh, Saudi Arabia; Department of Medical Laboratory Sciences (E E Anyabolo BMLS), Department of Pharmacology and Therapeutics (Prof O E Onwujekwe PhD), University of Nigeria Nsukka, Enugu, Nigeria; Operations Department (E E Anyabolo BMLS), Breast Without Spot, Enugu, Nigeria; School of Dentistry and Medical Sciences (A E Anyasodor PhD), Charles Sturt University, Orange, NSW, Australia; School of Medicine and Public Health (G C Apostol MD), Center for Research and Innovation (V F Pepito MSc), Ateneo De Manila University, Pasig City, Philippines; Inter-Agency Committee on Environmental Health (G C Apostol MD), Department of Health Philippines, Manila, Philippines; Health Management and Economics Research Center (J Arabloo PhD, H Ayatollahi PhD), Rajaie Cardiovascular Research Center (A Askarinejad MD), Department of Health Information Management (H Ayatollahi PhD), School of Medicine (M Bastan MD), Department of Medical Laboratory

Sciences (F Dorostkar PhD), Preventive Medicine and Public Health Research Center (B Eshrati PhD, A Tehrani-Banihashemi PhD), Department of Cardiology (A Ghaffari Jolfayi MD), Department of Ophthalmology (H Hasani MD, M Ziafati MD), Minimally Invasive Surgery Research Center (A Kabir MD), Eye Research Center (H Kasraei MD), Educational Development Center (E Khodadoust MD), Department of Physiology (H Pazoki Toroudi PhD), Physiology Research Center (H Pazoki Toroudi PhD), Colorectal Research Center (A Sarveazad PhD), Department of Community and Family Medicine (A Tehrani-Banihashemi PhD), Iran University of Medical Sciences, Tehran, Iran (M Moradi MD); College of Pharmacy (M Arafat PhD), AAU Health and Biomedical Research Center (Prof F El-Dahiyat PhD), Al Ain University, Abu Dhabi, United Arab Emirates; College of Art and Science (D Areda PhD), Ottawa University, Surprise, AZ, USA; School of Life Sciences (D Areda PhD), Arizona State University, Tempe, AZ, USA; College of Medicine and Health Sciences (B B Aregawi PhD), Department of Midwifery (M W Gebregergis MSc), Department of Medical Laboratory Sciences (H Negash MSc, H L L Weldetinsaa MSc), Adigrat University, Adigrat, Ethiopia; Department of Veterinary Pharmacology and Toxicology (A Aremu PhD), Department of Veterinary Physiology and Biochemistry (A Basiru PhD), Department of Veterinary Public Health and Preventive Medicine (I A Odetokun PhD), University of Ilorin, Ilorin, Nigeria; Department of Cardiovascular, Endocrine-Metabolic Diseases and Aging (B Armocida MSc), Istituto Superiore di Sanità, Rome, Italy; School of Health and Social Studies (Prof J Ärnlöv PhD), Dalarna University, Falun, Sweden; Department of Biophysics (A A Artamonov PhD), Russian Academy of Sciences, Moscow, Russia; Department of Epidemiology (K D Artanti MSc, A Hargono DMD), Department of Community Health Nursing (F Efendi PhD), Faculty of Public Health (S Martini PhD), Department of Biology (Prof H Purnobasuki PhD), Universitas Airlangga (Airlangga University), Surabaya, Indonesia; Department of Electrical and Electronics Engineering Science (I T Aruleba MSc), University of Johannesburg, Johannesburg, South Africa; Department of Community Medicine and Rehabilitation (A Arumugam PhD), Department of Nursing (Prof D Edvardsson PhD), Umeå University, Umea, Sweden; National Agency for Strategic Research in Medical Education (NASRME) (Prof S Asgary MSc), Development of Research and Technology Center (S Djalalinia PhD), Ministry of Health and Medical Education, Tehran, Iran; Department of Public Health (M Y Ashemo PhD, T Y Hegena MPH, M H Nunemo MPH), Wachemo University, Hossana, Ethiopia; School of Architecture, Design, and Planning (Prof T Astell-Burt PhD), Menzies Centre for Health Policy and Economics (M Balasubramanian PhD), Charles Perkins Centre (R Biswas PhD), Westmead Clinical School (R Chimoriya PhD), School of Pharmacy and Charles Perkins Centre (Z Dai PhD), School of Public Health (Prof T R Driscoll PhD, L D Knibbs PhD), Save Sight Institute (H Kandel PhD, Y You PhD), Department of Public Health (M Khan PhD), Asbestos Diseases Research Institute (J Leigh MD), School of Chemical & Biomolecular Engineering (E A Noman PhD), School of Veterinary Science (B B Singh PhD), University of Sydney, Sydney, NSW, Australia; Hunter New England Population Health, Wallsend, NSW, Australia (P Atorkey MPhil); Department of Forensic Medicine (A Atreya MD), Lumbini Medical College, Palpa, Nepal; Northumbria HealthCare NHS Foundation Trust, Newcastle upon Tyne, UK (A Aujayeb MBBS); School of Business (Prof M Ausloos PhD), Department of Health Sciences (P H Lee PhD, S J Tromans PhD), University of Leicester, Leicester, UK; Center for Health Systems Research (L Avila-Burgos ScD), Center for Nutrition and Health Research (I R Campos-Nonato PhD, E Denova-Gutiérrez DSc), Research in Nutrition and Health (F Mejia-Rodriguez MSc), National Institute of Public Health, Cuernavaca, Mexico; The Judith Lumley Centre (B Ayala Quintanilla PhD), School of Nursing and Midwifery (Prof D Edvardsson PhD, F Efendi PhD, M Rahman PhD), Department of Public Health (H Jiang PhD, Prof C Liu PhD), La Trobe University, Melbourne, VIC, Australia; San Martin de Porres University, Lima, Peru (B Ayala Quintanilla PhD); Health Insurance

Directorate (C Ayestas Portugal MPH), Ministry of Health, Lima, Peru; Department of Psychiatry (Prof J L Ayuso-Mateos PhD), Department of Medicine (Prof A Ortiz MD), Hospital Universitario de La Princesa (Princess University Hospital) (Prof J B Soriano MD), Universidad Autónoma de Madrid (Autonomous University of Madrid), Madrid, Spain; Biomedical Research Networking Center for Mental Health Network (CiberSAM) (Prof J L Ayuso-Mateos PhD), National School of Public Health (Prof R Sarmiento-Suárez MPH), Carlos III Health Institute, Madrid, Spain; Department of Sciences (Prof R M S Azevedo PhD), Therapeutic and Diagnostic Technologies Department (Prof N Cruz-Martins PhD), Toxicology Research Unit (TOXRUN) (Prof D Dias da Silva PhD), Cooperativa de Ensino Superior Politécnico e Universitário (Polytechnic and University Higher Education Cooperative), Gandra, Portugal; Badan Pusat Statistik (BPS) (Central Bureau of Statistics) (G S Azhar PhD), The RAND Corporation, Santa Monica, CA, USA; Department of Neurovascular Research (A Y Azzam MBBCh), Nested Knowledge Inc, Saint Paul, MN, USA; Faculty of Medicine (A Y Azzam MBBCh), Department of Cardiology (O M Makram MD), October 6 University, 6th of October City, Egypt; Centre for Global Health Inequalities Research (CHAIN) (I L Backhaus PhD, H Hoven PhD), Department of Circulation and Medical Imaging (J Nauman PhD), Norwegian University of Science and Technology, Trondheim, Norway; Gomal Center of Biochemistry and Biotechnology (M Badar PhD), Gomal University, Dera Ismail Khan, Pakistan; Department of Forensic Science (A D Badiye PhD, H Bansal MSc, N Kapoor PhD), Government Institute of Forensic Science, Nagpur, India; Department of Pediatrics (Prof A Bagga MD), Centre for Community Medicine (A Jaiswal MD), Centre for Dental Education and Research (B M Purohit MDS), Department of Psychiatry (Prof R Sagar MD), Department of Radiation Oncology (A Shankar MD), All India Institute of Medical Sciences, New Delhi, India; Division of Orthopaedics (S Baghdadi MD), Children's Hospital of Philadelphia, Philadelphia, PA, USA; Health Research Institute (N Bagheri PhD), University of Canberra, Canberra, ACT, Australia; School of Medicine (S Bagherieh BSc, G Ghasempour Dabaghi MD, M Rabiee Rad MD), Department of Environmental Health Engineering (A Fatehizadeh PhD), Department of Epidemiology and Biostatistics (Prof M Mansourian PhD), Cardiac Rehabilitation Research Center (K Mehrabani-Zeinabad PhD, Prof M Sadeghi MD), Isfahan Cardiovascular Research Institute (N Mohammadifard PhD), Heart Failure Research Center (S Najdaghi MD, D Narimani Davani MD), Neuroscience Research Center (S Najdaghi MD), Department of Medical Physics (K Saber PhD), Department of Clinical Biochemistry (O Vakili PhD), Isfahan University of Medical Sciences, Isfahan, Iran; Department of Surgery (P Bahrami Taghanaki MD), Dental Research Center (E Bardideh DDS), Orthodontics Department (M Ghorbani DDS), Clinical Research Development Unit (N Morovatdar MD), Applied Biomedical Research Center (A Sahebkar PhD), Biotechnology Research Center (A Sahebkar PhD), Department of Medical Informatics (S Tabatabaei PhD), Clinial Research Development Unit (S Tabatabaei PhD), Mashhad University of Medical Sciences, Mashhad, Iran; School of Public Affairs (R Bai MD), School of Atmospheric Physics (K Mehmood PhD), Nanjing University of Information Science and Technology, Nanjing, China; International Medical School (A A Baig PhD), Management and Science University, Alam, Malaysia; Center for Clinical Research and Prevention (J L Baker PhD), Bispebjerg University Hospital, Frederiksberg, Denmark; Department of Forensic Medicine and Toxicology (S M Bakkannavar MD, Prof V C Nayak MD), Kasturba Medical College, Mangalore (R Holla MD, M Rao MD), Department of Biochemistry and Genetics (V Joshi MD), Prasanna School of Public health (R Kamath MHA), Department of Pharmacy Management (V S Ligade PhD), Department of Nephrology (Prof S Nagaraju DM), Manipal College of Dental Sciences (Prof R A Radhakrishnan PhD), Department of Community Medicine (C R Rao MD), Department of Health Information Management (B Reshmi PhD), Manipal Academy of Higher Education, Manipal, India; Health Care Management Department (M Balasubramanian PhD), Flinders Health and Medical Research

Institute (N B Bulamu PhD), College of Nursing and Health Sciences (L N Bulto PhD), College of Medicine and Public Health (T G Gebremeskel PhD, B Kaambwa PhD, G R Naik PhD), Health Economics Unit (B Kaambwa PhD), Department of Nursing and Health Sciences (S Shorofi PhD), Flinders University, Adelaide, SA, Australia; Center of Innovation, Technology and Education (CITE) (Prof O C Baltatu PhD), Institute of Biomedical Engineering (Prof L A Campos PhD), Anhembi Morumbi University, Sao Jose dos Campos, Brazil; Department of Medicine (K Bam MPH), School of Nursing and Midwifery (D Bhandari PhD), Stroke and Ageing Research Group (L L Dalli PhD), Australian Regenerative Medicine Institute (Y Mathangasinghe MD), Monash University, Clayton, VIC, Australia; Nuffield Department of Surgical Sciences (S Bandyopadhyay BA), Nuffield Department of Population Health (D A Bennett PhD), Big Data Institute (A J Browne MPH), Nuffield Department of Orthopaedics, Rheumatology, and Musculoskeletal Sciences (S M Graham PhD), Health Economics Research Centre (Prof J A B Rodriguez PhD), Nuffield Department of Medicine (T Runghien MSc, B Sartorius PhD), University of Oxford, Oxford, UK; Department of Neurosurgery (S Bandyopadhyay BA), School of Psychology (Prof S Cortese PhD), Faculty of Medicine (R Thayakaran PhD), University of Southampton, Southampton, UK; Institute of Health and Wellbeing (B Banik PhD), Federation University Australia, Melbourne, VIC, Australia; Manna Institute (B Banik PhD), University of New England, Armidale, NSW, Australia; Department of Non-communicable Diseases (P C Banik MPhil), Bangladesh University of Health Sciences, Dhaka, Bangladesh; Infectious Disease Epidemiology (A Banke-Thomas PhD), Department of Non-Communicable Disease Epidemiology (M Iwagami PhD), Department of Health Services Research and Policy (Prof M McKee DSc), London School of Hygiene & Tropical Medicine, London, UK; Department of Human Sciences (A Banke-Thomas PhD), University of Greenwich, London, UK; Miami Cancer Institute (M Bardhan MD), Baptist Health South Florida, Miami, FL, USA; School of Psychology (Prof S L Barker-Collo PhD), University of Auckland, Auckland, New Zealand; Department of Translational Medicine (F Barone-Adesi PhD), University of Eastern Piedmont, Novara, Italy; Department of Industrial Engineering (Prof L H Barrero DSc), Pontifical Javeriana University, Bogota, Colombia; Department of Epidemiology (A Barrow MPH, D Braithwaite PhD, D D Ding BS), Division of Pulmonary, Critical Care, and Sleep (M Beiranvand PhD), College of Medicine (M J Diaz BS), UF Health Cancer Center (S D Karanth PhD), Department of Computer and Information Science and Engineering (P Naghavi MSc), University of Florida, Gainesville, FL, USA; Department of Public & Environmental Health (A Barrow MPH), University of The Gambia, Brikama, The Gambia; Alpha Genomics, Islamabad, Pakistan (Z Basharat PhD); Faculty of Pharmacy (J D Basso PharmD, S Silva MSc), Coimbra Chemistry Centre (J D Basso PharmD), Department of Geography and Demography (M Rodrigues PhD), Coimbra Institute for Biomedical Imaging and Translational Research (S Silva MSc), University of Coimbra, Coimbra, Portugal; School of Public Health (S Basu PhD), Department of Primary Care and Public Health (T Beaney MSc, Prof A Majeed MD, Prof S Rawaf MD, C Tabche MSc), Department of Infection (C S Brown MD), Department of Brain Sciences (L D'Anna PhD), Department of Surgery and Cancer (Prof E Mossialos PhD), WHO Collaborating Centre for Public Health Education and Training (D L Rawaf MRCS), Imperial College London, London, UK; Cooper Medical School (S Batchu BS), Cooper Medical School of Rowan University, Camden, NJ, USA; Department of Medical Education (K Batra PhD), School of Public Health (R Batra MS), Department of Social and Behavioral Health (Prof M Sharma PhD), University of Nevada Las Vegas, Las Vegas, NV, USA; IT Department (R Batra MS), Coforge, Georgia, GA, USA; Department of Psychiatry (Prof B T Baune PhD), Institute for Epidemiology and Social Medicine (A Karch MD), University of Münster, Münster, Germany; Department of Psychiatry (Prof B T Baune PhD), Melbourne Medical School, Melbourne, VIC, Australia; Endocrinology and Metabolism Research Institute (A Khalaji BS), Department of Epidemiology (S Khanmohammadi MD, S Nejadghaderi

MD, S Rashedi MD), Non-Communicable Diseases Research Center (NCDRC), Tehran, Iran (A Behnoush BS); Department of Neurology (Prof Y Béjot PhD), University Hospital of Dijon, Dijon, France; Dijon Stroke Registry (Prof Y Béjot PhD), University of Burgundy, Dijon, France; Department of Medical Anatomy (A Bekele MSc), Department of Midwifery (T S Hadaro MSc), Department of Public Health (T Mekene Meto MPH), Department of Nursing (A M Mersha MSc), Department of Clinical Midwifery (B A Mesfin BMedSc), Arba Minch University, Arba Minch, Ethiopia; Department of Oral Pathology and Microbiology (U I Belgaumi MD), Department of Oral and Maxillofacial Pathology (V Kadashetti MDS), Department of Public Health Dentistry (Prof K M Shivakumar PhD), Krishna Vishwa Vidyapeeth (Deemed to be University), Karad, India; Department of Social Services (A W Bell MSW), Tufts Medical Center, Boston, MA, USA; School of the Environment (Prof M L Bell PhD), Department of Internal Medicine (F Etaee MD), Department of Psychiatry (W Li PhD, T G Rhee PhD), Department of Radiology and Biomedical Imaging (X Liu PhD), Department of Genetics (S Pawar PhD), Yale University, New Haven, CT, USA; Infectious Disease Research Department (M B Bello PhD), Medical Genomics Research Department (M Umair PhD), King Abdullah International Medical Research Center, Riyadh, Saudi Arabia; Department of Biological Sciences (L Belo PhD), Research Unit on Applied Molecular Biosciences (UCIBIO) (L Belo PhD, Prof F Carvalho PhD, V M Costa PhD, Prof D Dias da Silva PhD), Associated Laboratory for Green Chemistry (LAQV) (M Carvalho PhD, N G M Gomes PhD), Institute for Research and Innovation in Health (Prof N Cruz-Martins PhD), Department of Community Medicine, Information and Health Decision Sciences (A Freitas PhD), Department of Chemistry (N G M Gomes PhD), Department of Chemical Engineering (Prof C F Rodrigues PhD), University of Porto, Porto, Portugal; Department of Biomedical Sciences (Prof A Beloukas PhD), University of West Attica, Athens, Greece; Institute of Infection and Global Health (Prof A Beloukas PhD), Liverpool Orthopaedic and Trauma Service (S M Graham PhD), University of Liverpool, Liverpool, UK; Department of Industrial Engineering (S Bendak PhD), Haliç University, Istanbul, Turkiye; Department of Internal Medicine (I M Bensenor PhD, Prof A R Brunoni PhD), Department of Psychiatry (Prof A R Brunoni PhD, Prof J Castaldelli-Maia PhD, Prof M F P Peres MD, Y Wang PhD), Department of Epidemiology (Prof A C Goulart PhD), Department of Medicine (Prof P A Lotufo DrPH), Center for Clinical and Epidemiological Research (A B Oliveira PhD), University of São Paulo, São Paulo, Brazil; Department of Epidemiology and Health Promotion (Prof H Benzian PhD), Department of Child and Adolescent Psychiatry (Prof S Cortese PhD), School of Global Public Health (S D Friedman BA, E K Peprah PhD), New York University, New York, NY, USA; School of Medicine (A Beran MD, J T Tran BS), Indiana University, Indianapolis, IN, USA; Institute of Marketing (Z Berezvai PhD), Corvinus University of Budapest, Budapest, Hungary; Competition Economics and Market Research Section (Z Berezvai PhD), Hungarian Competition Authority, Budapest, Hungary; Faculty of Dentistry, Oral & Craniofacial Sciences (E Bernabe PhD), Unit for Population-Based Dermatology Research (Prof C Flohr PhD), Department of Twin Research and Genetic Epidemiology (M Mazidi PhD), Institute of Psychiatry, Psychology & Neuroscience (D Urso MD), School of Population Health and Environmental Sciences (Y Wang PhD), King's College London, London, UK; Hubert Department of Global Health (R S Bernstein MD), Rollins School of Public Health (Prof D A Sleet PhD), Department of Family and Preventive Medicine (S Thirunavukkarasu PhD), Emory University, Atlanta, GA, USA; Butte County Department of Public Health, Chico, CA, USA (R S Bernstein MD); Faculty of Medicine (P J G Bettencourt PhD), Catholic University of Portugal, Rio de Mouro, Portugal; Department of Public Health (A S Bhagavathula PhD), North Dakota State University, Fargo, ND, USA; Institutes of Applied Health Research and Translational Medicine (N Bhala PhD), Queen Elizabeth Hospital Birmingham, Birmingham, UK; Institute of Applied Health Research (N Bhala PhD, J S Chandan PhD, K Malhotra MBBS, R

Thayakaran PhD), NIHR Global Health Research Unit on Global Surgery (J C Glasbey MSc), University of Birmingham, Birmingham, UK; Public Health Research Laboratory (D Bhandari PhD), Faculty of Humanities and Social Sciences (U Paudel PhD), Department of Community Medicine (P M S Pradhan MD), Central Department of Public Health (N Subedi MPH), Tribhuvan University, Kathmandu, Nepal; Global Health Neurology Lab (S Bhaskar PhD), NSW Brain Clot Bank, Sydney, NSW, Australia; Department of Neurology and Neurophysiology (S Bhaskar PhD), South West Sydney Local Heath District and Liverpool Hospital, Sydney, NSW, Australia; Department of General Medicine (A N Bhat MD, J Jeganathan MD), Department of Internal Medicine (A Boloor MD, M M R Reddy MD), Department of Forensic Medicine and Toxicology (H L Dsouza MD, Prof B K Shetty MD, P H Shetty MD), Department of Community Medicine (N Joseph MD, N Kumar MD, P Mithra MD, R Motappa MD, R Thapar MD), Department of Anatomy (B Murlimanju MD), Department of Obstetrics and Gynaecology (A Shetty MS), Kasturba Medical College, Mangalore (Prof B Unnikrishnan MD), Manipal Academy of Higher Education, Mangalore, India; Department of Internal Medicine (V Bhat MBBS), St John's National Academy of Health Sciences, Bangalore, India; Medical Lab Technology (G K Bhatti PhD), University Centre for Research and Development (S Kalra DM), Chandigarh University, Mohali, India; Department of Human Genetics and Molecular Medicine (Prof J S Bhatti PhD, U Sharma PhD), Department of Microbiology (M K Yadav PhD), Central University of Punjab, Bathinda, India; Department of Botanical and Environmental Sciences (Prof M S Bhatti PhD), Department of Pharmaceutical Sciences (R Bhatti PhD), Guru Nanak Dev University, Amritsar, India; Department of Pharmacy (Prof M A Bhuiyan PhD, S Dewan PhD), University of Asia Pacific, Dhaka, Bangladesh; Centre for Global Child Health (Prof Z A Bhutta PhD), Department of Surgery (A Brar MD), Division of Neurology (S Fereshtehnejad PhD), University of Toronto, Toronto, ON, Canada; Centre of Excellence in Women & Child Health (Prof Z A Bhutta PhD), Division of Women and Child Health (J K Das MD), Department of Family Medicine (Prof S M Shah PhD), Aga Khan University, Karachi, Pakistan; Scientific-Tools.Org, Bergamo, Italy (B Bikbov MD); Department of Neurology (Prof A Biswas DM), Department of GI Surgery (A Dhali MBBS), Institute of Post-Graduate Medical Education and Research and Seth Sukhlal Karnani Memorial Hospital, Kolkata, India; Department of Community Medicine and Family Medicine (B Biswas MD), All India Institute of Medical Sciences, Deoghar, India; Clinical Research Centre (R Biswas PhD), Sydney Local Health District, Sydney, NSW, Australia; Department of Global Public Health and Primary Care (Prof T Bjørge PhD, A Hailu PhD), Department of Psychosocial Science (D Sagoe PhD), University of Bergen, Bergen, Norway; Cancer Registry of Norway, Oslo, Norway (Prof T Bjørge PhD); SAMRC Centre for Health Economics and Decision Science (M K Boachie PhD), University of the Witwatersrand, Johannesburg, South Africa; Sargent College of Health and Rehabilitation Science (H Boakye MPH), Health Informatic Lab (T Javaheri PhD), Department of Computer Science (R Rawassizadeh PhD), Boston University, Boston, MA, USA; European & Developing Countries Clinical Trials Partnership, Cape Town, South Africa (Prof M J Bockarie MSc); Department of Medicine (Prof M J Bockarie MSc, G A Mensah MD), School of Public Health and Family Medicine (C A Nnaji MPH), Division of Cardiology (Prof M Ntsekhe PhD), Department of Paediatrics and Child Health (Prof H J Zar PhD), University of Cape Town, Cape Town, South Africa; School of Business Administration (Prof V Bodolica PhD), American University of Sharjah, Sharjah, United Arab Emirates; Department of Biomedical Sciences (S Bolla PhD), Department of Medicine (Prof D Poddighe PhD), Nazarbayev University, Astana, Kazakhstan; General Directorate of Health Information Systems (B Bora Basara PhD), Ministry of Health, Ankara, Turkiye; Facultad de Salud (Faculty of Health) (Prof A Botero Carvajal MSc), Universidad Santiago de Cali (Santiago de Cali University), Cali, Colombia; Department of Medicine (Prof S Bouaoud MD), Faculty of Medicine (Prof A Ouyahia PhD), University Ferhat Abbas of Setif, Setif,

Algeria; Department of Epidemiology and Preventive Medicine (Prof S Bouaoud MD), University Hospital Saadna Abdenour, Setif, Algeria; Vision and Eye Research Institute (Prof R Bourne MD), Anglia Ruskin University, Cambridge, UK; Department of Earth, Environment, and Equity (C Boxe PhD), Howard University, Washington, DC, USA; Cancer Population Sciences Program (D Braithwaite PhD), University of Florida Health Cancer Center, Gainesville, FL, USA; Department of Psychiatry and Behavioral Health (Prof N J K Breitborde PhD), Department of Psychology (Prof N J K Breitborde PhD), Division of Cardiovascular Medicine (A Guha MD), Ohio State University, Columbus, OH, USA; Institute for Medical Information Processing, Biometry, and Epidemiology (S Breitner DSc), Ludwig Maximilian University of Munich, Munich, Germany; Institute of Epidemiology (S Breitner DSc), Helmholtz Zentrum München German Research Center for Environmental Health, Neuherberg, Germany; Division of Clinical Epidemiology and Aging Research (Prof H Brenner MD), German Cancer Research Center, Heidelberg, Germany; Biomedical Department (A N Briko PhD), Department of Medical and Technical Information Technology (A Hammoud MSc), Bauman Moscow State Technical University, Moscow, Russia; Department of Neuroscience (G Britton PhD), University of Panama, Ancon, Panama; Infectious Diseases Department (G Britton PhD), Gorgas Memorial Institute for Health Studies, Panama City, Panama; HCAI, Fungal, AMR, AMU, & Sepsis Division (C S Brown MD), United Kingdom Health Security Agency, London, UK; Global Health Research Institute (D Buonsenso MD), Department of Health Science and Public Health (L Villani DrPH), Università Cattolica del Sacro Cuore (Catholic University of Sacred Heart), Rome, Italy; Department of Biopharmaceutics and Clinical Pharmacy (Y Bustanji PhD), The University of Jordan, Amman, Jordan; School of Public Health Sciences (Z A Butt PhD), University of Waterloo, Waterloo, ON, Canada; Al Shifa School of Public Health (Z A Butt PhD), Al Shifa Trust Eye Hospital, Rawalpindi, Pakistan; School of Public Health and Administration (L Cahuana-Hurtado PhD), Peruvian University Cayetano Heredia, Lima, Peru; Department of Clinical Pharmacy (Prof D Calina PhD), University of Medicine and Pharmacy of Craiova, Craiova, Romania; Department of Internal Medicine (Prof L A Cámera MD), Hospital Italiano de Buenos Aires (Italian Hospital of Buenos Aires), Buenos Aires, Argentina; Board of Directors (Prof L A Cámera MD), Argentine Society of Medicine, Buenos Aires, Argentina; Dana-Farber Cancer Institute, Boston, MA, USA (C Cao MPH); Department of Ophthalmology (F Cao MD), Beijing Institute of Ophthalmology, Beijing, China; Department of Oral and Maxillofacial Surgery (Y Cao PhD), Sichuan University, Chengdu, China; Department of Biomedical and Neuromotor Sciences (A Capodici MD, S Guicciardi MD, A Mazzotti PhD, L Muccioli MD), Department of Medical and Surgical Sciences (Prof F S Violante MD), University of Bologna, Bologna, Italy; Department of Management and Healthcare (EMbeDS) (A Capodici MD), Sant'Anna School of Advanced Studies, Pisa, Italy; Department of Health Care (Prof R Cárdenas DSc), Metropolitan Autonomous University, Mexico City, Mexico; Oncological Network, Prevention and Research Institute (G Gorini MD), Institute for Cancer Research, Prevention and Clinical Network, Florence, Italy (G Carreras PhD); Dermatology Unit (A Carugno MD), Azienda Socio Sanitaria Territoriale Papa Giovanni XXIII (Territorial Healthcare Company Pope John XXIII), Bergamo, Italy; Instituto de Investigação, Inovação e Desenvolvimento (Institute of Research Innovation and Development) (M Carvalho PhD), University Fernando Pessoa, Porto, Portugal; Epidemiology and Public Health Evaluation Group (C A Castañeda-Orjuela MD), Department of Public Health (Prof F P De la Hoz PhD), National University of Colombia, Bogota, Colombia; Department of Medicine (G Castelpietra PhD), University of Udine, Udine, Italy; Department of Mental Health (G Castelpietra PhD), Healthcare Agency "Friuli Occidentale", Pordenone, Italy; National School of Public Health (F Catalá-López PhD, A Padron-Monedero PhD), Institute of Health Carlos III, Madrid, Spain; Clinical Epidemiology Program (F Catalá-López PhD), Ottawa Hospital Research Institute, Ottawa, ON,

Canada; Department of Pharmacological and Biomolecular Sciences (Prof A L Catapano PhD), Department of Food, Environmental and Nutritional Sciences (I Cioffi PhD), IRCCS Istituto Ortopedico Galeazzi (G Damiani MD), Department of Clinical Sciences and Community Health (Prof C La Vecchia MD), University of Milan, Milan, Italy; MultiMedica Sesto San Giovanni IRCCS, Sesto San Giovanni, Italy (Prof A L Catapano PhD); Department of Psychiatry (A Caye PhD), Postgraduate Program in Epidemiology (Prof B B Duncan MD, Prof M I Schmidt MD), Department of Psychiatry and Legal Medicine (Prof G A Salum PhD), Federal University of Rio Grande do Sul, Porto Alegre, Brazil; Department of Otolaryngology, Head and Neck Surgery (C R Cederroth PhD), University of Tübingen, Tübingen, Germany; Department of Medical, Surgical, and Health Sciences (L Cegolon PhD, Prof M D'Oria MD), University of Trieste, Trieste, Italy; Public Health Unit (L Cegolon PhD), University Health Agency Giuliano-Isontina (ASUGI), Trieste, Italy; College of Public Health, Medical, and Veterinary Sciences (M Cenderadewi MPHTM, Prof R C Franklin PhD, A E Peden PhD), Department of Public Health and Tropical Medicine (T I Emeto PhD), James Cook University, Townsville, QLD, Australia; Department of Public Health (M Cenderadewi MPHTM), University of Mataram, Mataram, Indonesia; Mary MacKillop Institute for Health Research (Prof E Cerin PhD), Faculty of Health Sciences (G R Poudel PhD), Australian Catholic University, Melbourne, VIC, Australia; School of Public Health (Prof E Cerin PhD), Department of Urban Planning and Design (C Guo PhD), Centre for Suicide Research and Prevention (Prof P Yip PhD), Department of Social Work and Social Administration (Prof P Yip PhD), University of Hong Kong, Hong Kong, China; ICMR School of Public Health (J Chadwick MD), National Institute of Epidemiology, Chennai, India; Department of Biotechnology (Prof C Chakraborty PhD), Adamas University, Kolkata, India; Institute for Skeletal Aging & Orthopedic Surgery (Prof C Chakraborty PhD), Hallym University, Chuncheon, South Korea; State Disease Investigation Laboratory (S Chakraborty MVSc), Animal Resources Development Department, Agartala, India; Heart Failure and Structural Heart Disease Unit (J Chan MBChB), Cardiovascular Analytics Group, Hong Kong, China; Department of Medicine and Therapeutic (R N C Chan MBChB), Prince of Wales Hospital, Hong Kong, China; Department of Clinical Nutrition (R M Chandika PhD), Department of Epidemiology (S Dohare MD, K Y Ghailan PhD, M Khan MD), Department of Maxillofacial Surgery and Diagnostic Sciences (E S Halboub PhD), Department of Health Informatics (A Mehmood PhD), Department of Health Education and Promotion (M Shanawaz MD, F Sobia PhD), Jazan University, Jazan, Saudi Arabia; Center for Cancer Epidemiology (Prof P Chaturvedi MD), Homi Bhabha National Institute (HBNI), Mumbai, India; Fuwai Hospital (A Chen PhD), Chinese Academy of Medical Sciences & Peking Union Medical College, Beijing, China; Department of Computer Science (A Chen PhD), University of Texas Austin, Austin, TX, USA; Clinical Research Center (H Chen MB), Zhujiang Hospital (Z Zhu PhD), Southern Medical University, Guangzhou, China; Department of Internal Medicine (D Flood MD), University of Michigan, Ann Arbor, MI, USA (M Chen BDS); Department of Endocrine and Metabolic Diseases (M Chen PhD), Shanghai Mental Health Center (Prof M R Phillips MD), Shanghai Jiao Tong University, Shanghai, China; Ocular Epidemiology Research Group (Prof C Cheng MD), Singapore Eye Research Institute, Singapore, Singapore; Ophthalmology & Visual Sciences Academic Clinical Program (Prof C Cheng MD), National Dental Research Institute Singapore (G G Nascimento PhD), Duke-NUS Medical School, Singapore, Singapore; Department of Paediatrics (E T W Cheng MBChB), Jockey Club School of Public Health and Primary Care (J Huang MD, C Zhong MD), Department of Medicine and Therapeutics (L Lim MRCP), The Chinese University of Hong Kong, Hong Kong, China; Department of Public Health and Health Policy (O Chimed-Ochir PhD), Hiroshima University, Hiroshima, Japan; Translational Health Research Institute (R Chimoriya PhD, K Rana PhD), Department of Engineering (G R Naik PhD), School of Medicine (U L Osuagwu PhD), Western Sydney

University, Sydney, NSW, Australia; Division of Infectious Diseases (P R Ching MD), Virginia Commonwealth University, Richmond, VA, USA; Department of Public Health, Administration, and Social Sciences (J L Chirinos-Caceres DrPH), Cayetano Heredia University, Lima, Peru; Iraq Field Epidemiology Training Program (I-FETP) (A Chitheer MD), Ministry of Health, Baghdad, Iraq; Department of Clinical Oncology (W C S Cho PhD), Queen Elizabeth Hospital, Hong Kong, China; Department of Medicine (B Chong MBBS), Saw Swee Hock School of Public Health (Prof D S Q Koh PhD, Prof D S Q Koh PhD, S Yi PhD), School of Medicine (M Ng PhD), Leadership Institute for Global Health Transformation (LIGHT) (S Ramazanu PhD), Department of Surgery (K Tan PhD), Yong Loo Lin School of Medicine (Prof N Venketasubramanian MBBS), National University of Singapore, Singapore, Singapore; Department of Biosciences (H Chopra PhD), Center for Global Health Research (M Fareed PhD, S Muthupandian PhD), Saveetha Dental College and Hospitals (M R Tovani-Palone PhD), Saveetha Institute of Medical and Technical Sciences (SIMATS), Chennai, India; Florida International University, Miami, FL, USA (Prof R Chowdhury PhD); Department of Epidemiology (Prof R Chowdhury PhD), Department of Emergency Medicine (I Pantazopoulos PhD), University of Bern, Bern, Switzerland; Department of Pulmonary Medicine (Prof D J Christopher MD), Department of Endocrinology, Diabetes and Metabolism (Prof N Thomas PhD), Christian Medical College and Hospital (CMC), Vellore, India; Center for Biomedicine and Community Health (D Chu PhD), Viet Nam National University-International School, Hanoi, Viet Nam; Department of Paediatric Surgery (I S Chukwu BMedSc), Federal Medical Centre, Umuahia, Nigeria; Department of AndroUrology (Prof E Chung MD), AndroUrology Centre, Brisbane, QLD, Australia; Health Data Research UK, London, UK (S Chung PhD); Health Effects Institute, Boston, MA, USA (A J Cohen DSc); Nova Medical School (J Conde PhD), Nova University of Lisbon, Lisbon, Portugal; Department of Respiratory Medicine and Allergology (Prof A Corlateanu PhD), Nicolae Testemitanu State University of Medicine and Pharmacy, Chisinau, Moldova; Department of Epidemiology and Prevention (S Costanzo PhD), IRCCS Neuromed, Pozzilli, Italy; Department of Family Medicine and Public Health (Prof M H Criqui MD), University of California San Diego, La Jolla, CA, USA; Department of Dermatology (G Damiani MD), Lerner College of Medicine (L Göbölös PhD), Harrington Heart and Vascular Institute (A Guha MD), Department of Quantitative Health Science (X Liu PhD), Department of Neonatology (I Qattea MD), Department of Nutrition and Preventive Medicine (Prof J Sanabria MD), Case Western Reserve University, Cleveland, OH, USA; Department of Information Technology (A M Darwesh PhD), Department of Computer Science (Prof M Hosseinzadeh PhD), Diplomacy and Public Relations Department (A Omar Bali PhD), University of Human Development, Sulaymaniyah, Iraq; Ingram School of Engineering (S Das PhD), Texas State University, San Marcos, TX, USA; Department of Population and Development (C A Dávila-Cervantes PhD), Latin American Faculty of Social Sciences Mexico, Mexico City, Mexico; Department of Surgery (D V Davitoiu PhD, B Socea PhD), "Sf Pantelimon" Emergency Clinical Hospital Bucharest, Bucharest, Romania; Department of Legal Medicine, Psychiatry and Pathology (A de la Torre-Luque PhD), Universidad Complutense de Madrid (Complutense University of Madrid), Madrid, Spain; Australian Institute for Suicide Research and Prevention (Prof D De Leo DSc), Griffith University, Mount Gravatt, QLD, Australia; Medical College (S Debopadhaya BS), Albany Medical College, Albany, NY, USA; School of Medicine (I Delgado-Enciso DSc), University of Colima, Colima, Mexico; Department of Research (I Delgado-Enciso DSc), Colima State Health Services, Colima, Mexico; Department of Medicine (J Delgado-Saborit PhD), Universitat Jaume I, Castellon, Spain; St Paul's Eye Unit (N Dervenis MD), Royal Liverpool University Hospital, Liverpool, UK; Department of Ophthalmology (N Dervenis MD), 2nd Department of Cardiology (E Karagiannidis PhD, P Karakasis MSc, D Patoulias PhD, P Stachteas MSc), 1st Department of Ophthalmology (Prof F Topouzis PhD), Aristotle University of Thessaloniki,

Thessaloniki, Greece; Department of Forensic Medicine (E Dervišević PhD), University of Sarajevo, Sarajevo, Bosnia and Herzegovina; Graduate Medical Education (H D Desai MD), Gujarat Adani Institute of Medical Sciences, Bhuj, India; Division of Cardiology (R Desai MBBS), Atlanta Veterans Affairs Medical Center, Decatur, GA, USA; Department of Community Medicine (V G C Devanbu MD), Chettinad Academy of Research and Education, Chennai, India; Pharmacology Department (S Dewan PhD), Center for Life Sciences Research Bangladesh, Dhaka, Bangladesh; Division of Pathology (K Dhama PhD), ICAR-Indian Veterinary Research Institute, Bareilly, India; Research and Development Cell (A S Dhane MBA), Department of Oral Pathology and Microbiology (Prof G S Sarode PhD, Prof S C Sarode PhD), Dr D Y Patil University, Pune, India; Planetary Health Research Centre, Kathmandu, Nepal (M L Dhimal PhD); Institute of Occupational, Social and Environmental Medicine (M L Dhimal PhD, M Dhimal PhD), Goethe University Frankfurt, Frankfurt am Main, Germany; Research Department (M Dhimal PhD, A Pandey MPH, U Paudel PhD), Nepal Health Research Council, Kathmandu, Nepal; Department of Pharmacy Practice (S Dhingra PhD), National Institute of Pharmaceutical Education and Research, Hajipur, India; The Zena and Michael A Wiener Cardiovascular Institute (V R Dhulipala MD), Department of Cardiology (M Vinayak MD), Icahn School of Medicine at Mount Sinai, New York, NY, USA; Institute for Health and Sport (R Dhungana MPhil), Victoria University, Footscray, VIC, Australia; Faculty of Science (Prof D Diaz PhD), School of Medicine (Prof R Lozano MD), National Autonomous University of Mexico, Mexico City, Mexico; Department of Gastroenterology (L A Diaz MD), Pontifical Catholic University of Chile, Santiago, Chile; Department of Experimental and Clinical Medicine (M Dinu PhD), University of Florence, Florence, Italy; Department of Medicine (T C Do MD), Faculty of Medicine (N N Nguyen MD), School of Medicine (H Pham MD), Pham Ngoc Thach University of Medicine, Ho Chi Minh City, Viet Nam; Department of Medicine (T H Do MD), Can Tho University of Medicine and Pharmacy, Can Tho, Viet Nam; Center for Health Sciences (C B do Prado MSc), Federal University of Espírito Santo, Vitória, Brazil; Department of Biostatistics (M Dodangeh Mcom), Independent Consultant, Tehran, Iran; Department of Social Medicine and Health Care Organisation (K G Dokova PhD), Medical University "Prof Dr Paraskev Stoyanov", Varna, Bulgaria; School of Elderly Care Services and Management (W Dong MD), Nanjing University of Chinese Medicine, Nanjing, China; Cardio-Thoraco-Vascular Department (Prof M D'Oria MD), Azienda Sanitaria Universitaria Giuliano Isontina, Trieste, Italy; School of Medicine (Prof S Xu PhD), University of Rochester, Rochester, NY, USA (E Dorsey MD); Department of Cardiology (R Doshi MD), St Joseph's University Medical Center, Paterson, NJ, USA; Department of Medicine (A C Dsouza MBBS), Bangalore Medical College and Research Institute, Bangalore, India; Department of Forensic Medicine and Toxicology (H L Dsouza MD), Kasturba Medical College Mangalore, Mangalore, India; Postgraduate Program in Health Sciences (S C Dumith PhD), Federal University of Rio Grande, Rio Grande, Brazil; School of Medicine (Prof A R Duraes PhD), Institute of Collective Health (Prof D Rasella PhD), Federal University of Bahia, Salvador, Brazil; Department of Internal Medicine (Prof A R Duraes PhD), Escola Bahiana de Medicina e Saúde Pública (Bahiana School of Medicine and Public Health), Salvador, Brazil; Department of Biotechnology (S Duraisamy PhD), SRM Institute of Science and Technology, Kattankulathur, India; Health Research Institute (A Dushpanova PhD), Al Farabi Kazakh National University, Almaty, Kazakhstan; Child Health Analytics Research Program (P A Dzianach PhD, F Sanna PhD, D J Weiss PhD), Geospatial Health and Development Team (J Lubinda PhD, A Saddler PhD), The Malaria Atlas Project (M A McPhail PhD, S F Rumisha PhD), Telethon Kids Institute, Perth, WA, Australia; Department of Conservative Dentistry with Endodontics (A M Dziedzic DSc), Medical University of Silesia, Katowice, Poland; Department of Orthopaedic Surgery (A Ebrahimi MD), Department of Psychiatry (R T Liu PhD), Department of Radiology (X Liu PhD), Division of Cardiology (D H Nguyen BS),

Cardiovascular Research Center (A Schuermans BSc), Massachusetts General Hospital, Boston, MA, USA; Division of Cardiothoracic Vascular Surgery (C P Echieh FWACS), University of Calabar, Calabar, Nigeria; Division of Cardiothoracic Surgery (C P Echieh FWACS), University of Arizona, Tucson, AZ, USA; Higher School of Technology (Prof A Ed-Dra PhD), Sultan Moulay Slimane University, Beni Mellal, Morocco; School of Health Sciences (H A Edinur PhD), Universiti Sains Malaysia (University of Science Malaysia), Kubang Kerian, Malaysia; College of Science, Health and Engineering (K Edvardsson PhD), La Trobe University, Bundoora, VIC, Australia; Department of Biochemistry (A Eftekharimehrabad PhD), Ege University, Izmir, Turkiye; Azerbaijan State University of Economics (UNEC), Baku, Azerbaijan (A Eftekharimehrabad PhD); Faculty of Science and Health (M Ekholuenetale PhD), University of Portsmouth, Hampshire, UK; AlMoosa College of Health Science, Al Ahsa, Saudi Arabia (R A El Arab MSc); Department of Clinical Pathology (Prof M El Sayed Zaki PhD, M Elshaer MD), Department of Cardiology (Prof M M Ramadan PhD), Mansoura University, Mansoura, Egypt; Clinical Pharmacy Program (Prof F El-Dahiyat PhD), Al Ain University, Al Ain, United Arab Emirates; Department of Internal Medicine (Prof G M T ElGohary MD), Biochemistry Department (Prof N M Hamdy PhD), Department of Entomology (A M Samy PhD), Medical Ain Shams Research Institute (MASRI) (A M Samy PhD), Neurology Department (Prof A S Shalash PhD), Ain Shams University, Cairo, Egypt; Department of Neurophysiology (Prof H R Elhabashy MD), Faculty of Medicine (A O Elmehrath PGCert), Department of Neurology (A Hassan MD), Cairo University, Cairo, Egypt; Faculty of Medicine (M Elhadi MD), Department of Radiology (A Msherghi MD), University of Tripoli, Tripoli, Libya; Department of Infectious Diseases and Public Health (I Elsohaby PhD), Department of Biomedical Sciences (A Waris MS), City University of Hong Kong, Hong Kong, China; Department of Animal Medicine (I Elsohaby PhD), Cardiovascular Department (Prof A M A Saad MD), Zagazig University, Zagazig, Egypt; Division of Epidemiology (N Esfandiari DVM), University of Tehran, Tehran, Iran; Department of Bacteriology and Virology (M Eslami PhD), Department of Immunology (D Haghmorad PhD), Cancer Research Center (D Haghmorad PhD), Research Center of Physiology (H Yaribeygi PhD), Semnan University of Medical Sciences, Semnan, Iran; Cancer Research Center (M Eslami PhD), Semnan University of Medical Scinces, Semnan, Iran; Independent Consultant, Bologna, Italy (N Fabin MD); Research Centre for Healthcare and Community (A F Fagbamigbe PhD), Faculty of Health and Life Sciences (O P Kurmi PhD), Coventry University, Coventry, UK; Department of Periodontology and Community Dentistry (O F Fagbule FWACS), Department of Community Medicine (O S Ilesanmi PhD), Department of Medicine (Prof M O Owolabi DrM), Department of Oral and Maxillofacial Surgery (A A Salami BDS), University College Hospital, Ibadan, Ibadan, Nigeria; Epidemiology and Biostatistics Unit (L Falzone PhD), IRCCS Pascale, Naples, Italy; Dissemination Division (C S e Farinha MSc), National Institute of Statistics, Lisbon, Portugal; Activity Planning and Control Unit (C S e Farinha MSc), Directorate-General of Health (DGS), Lisbon, Portugal; Department of Biology (P S Faris PhD), Department of Food Technology (Y Galali ResM, B A Sadee PhD), Department of Chemistry (H I M Amin PhD), Salahaddin University-Erbil, Erbil, Iraq; Department of Biology (P S Faris PhD), Department of Nutrition and Dietetics (Y Galali ResM, B A Sadee PhD), Department of Medical Biochemical Analysis (H I M Amin PhD), Cihan University-Erbil, Erbil, Iraq; Department of Psychology (Prof A Faro PhD), Federal University of Sergipe, São Cristóvão, Brazil; Department of Veterinary Tropical Diseases (Prof F O Fasina PhD), School of Health Systems and Public Health (C C D Iwu MPH), Department of Medical Microbiology (L A Malinga PhD), University of Pretoria, Pretoria, South Africa; Animal Production and Health Division (EMPRES) (Prof F O Fasina PhD), Food and Agriculture Organization of the United Nations, Rome, Italy; Centre for Health Policy Research (Prof P Ward PhD), Torrens University Australia, Adelaide, SA, Australia (N K Fauk MSc); Institute of Resource Governance and Social Change, Kupang, Indonesia (N K Fauk MSc); Laboratory of

experimental Medicine (T Fazylov MD), Department of Health Research (M Kulimbet MSc), Atchabarov Scientific Research Institute of Fundamental and Applied Medicine (M Kulimbet MSc), Kazakh National Medical University, Almaty, Kazakhstan; National Institute for Stroke and Applied Neurosciences (Prof V L Feigin PhD), The National Institute for Stroke and Applied Neurosciences (I Rautalin PhD), Auckland University of Technology, Auckland, New Zealand; Third Department of Neurology (E V Gnedovskaya PhD), Research Center of Neurology, Moscow, Russia (Prof V L Feigin PhD, Prof M A Piradov DSc); National Institute of Environmental Health (X Feng PhD), National Center for Chronic and Noncommunicable Disease Control and Prevention (P Ye MPH), Chinese Center for Disease Control and Prevention, Beijing, China (Prof S Liu PhD); Department of Social Sciences (Prof N Ferreira PhD), University of Nicosia, Nicosia, Cyprus; Department of Nursing (G Fetensa MSc), Institute of Health Sciences (B R Feyisa MPH), Department of Public Health (D R Terefa MSc, T Tolossa MPH), Wollega University, Nekemte, Ethiopia; Department of Psychiatry (I Filip MD), Kaiser Permanente, Fontana, CA, USA; School of Health Sciences (I Filip MD), AT Still University, Mesa, AZ, USA; School of Social Sciences (J Flavel PhD), Stretton Health Equity, Adelaide, SA, Australia; Center for Research in Indigenous Health (D Flood MD), Maya Health Alliance, Tecpán, Guatemala; Institute of Gerontology (N A Foigt PhD), National Academy of Medical Sciences of Ukraine, Kyiv, Ukraine; Healthcare Innovation Department (D Fortuna MSc), Regional Agency for Health and Social Care of Emilia-Romagna, Bologna, Italy; Department of Biotechnological and Applied Clinical Sciences (DISCAB) (M Foschi MD), Department of Biotechnological and Applied Clinical Sciences (R Ornello PhD), Department of Neurology (Prof S Sacco MD), University of L'Aquila, L'Aquila, Italy; Department of Neuroscience (M Foschi MD), Hospital Santa Maria delle Croci, Ravenna, Italy; Center for Health Technology and Services Research (CINTESIS), Porto, Portugal (A Freitas PhD); Department of Pathology (Prof B Fux PhD), Department of Integrated Health Education (Prof L B Salaroli PhD), Federal University of Espirito Santo, Vitória, Brazil; Department of Community Medicine and Family Medicine (S G MD, V J MD, V Rajendran MD), All India Institute of Medical Sciences, Gorakhpur, India; Health Services Management Training Centre (P A Gaal PhD, T Joo PhD, J Lám PhD, T Palicz MD), Semmelweis University, Budapest, Hungary; Department of Applied Social Sciences (P A Gaal PhD), Sapientia Hungarian University of Transylvania, Târgu-Mureş, Romania; Institute of Applied Health Sciences (S Gaihre PhD), University of Aberdeen, Aberdeen, UK; Department of Oral Biology and Experimental Dental Research (M Gajdács PhD), University of Szeged, Szeged, Hungary; Department of Environmental Health Sciences (S Gallus DSc), Mario Negri Institute for Pharmacological Research, Milan, Italy; Department of Community Medicine and Family Medicine (A P Gandhi MD), All India Institute of Medical Sciences, Nagpur, India; Institute of Health and Wellbeing (B Ganesan PhD), Federation University Australia, Churchill, VIC, Australia; Department of General Medicine (M Ganiyani MD), Grant Medical College & Sir JJ Group of Hospitals, Mumbai, India; Department of Neurology (Prof R K Garg MD, V Suresh MBBS(c)), King George's Medical University, Lucknow, India; Department of Pharmacology (Prof R K Gautam PhD), Indore Institute of Pharmacy, Indore, India; Health Sciences Department of Oncology Nursing (T G Gebi MSc), Haramaya University, Harar, Ethiopia; Department of Environmental Health (M Gebrehiwot DSc), Wollo University, Dessie, Ethiopia; Department of Public Health Nutrition (T B B Gebremariam MPH), Department of Nursing (A Girmay MSc), Aksum University, Aksum, Ethiopia; Department of Reproductive and Family Health (T G Gebremeskel PhD), Axum College of Health Science, Axum, Ethiopia; Department of Public Health (L Getacher MPH), Debre Berhan University, Debre Berhan, Ethiopia; Department of Public Health (G K a Getahun MPH), Menelik II Medical and Health Science College, Addis Ababa, Ethiopia; Center of Health Management (K Y Ghailan PhD), Aden University, Aden, Yemen; Department of Epidemiology and Biostatistics (A Gholami PhD),

Non-Communicable Diseases Research Center (A Gholami PhD), Department of Basic Medical Sciences (S Yaghoubi PhD), Neyshabur University of Medical Sciences, Neyshabur, Iran; NCD Surveillance Unit (A U Gil PhD), World Health Organization (WHO), Moscow, Russia; Institute for Leadership and Health Management (A U Gil PhD), Moscow Medical Academy, Moscow, Russia; Warwick Medical School (Prof P S Gill DM), University of Warwick, Coventry, UK (J W Sakshaug PhD); Adelaide Medical School (T K Gill PhD), Centre for Heart Rhythm Disorders (J Noubiap MD), University of Adelaide, Adelaide, SA, Australia; Department of Nursing (E Z Gindaba MSc), Ethiopian Public Health Institute, Chiro, Ethiopia; Department of Cardiac Surgery (L Göbölös PhD), Cleveland Clinic Abu Dhabi, Abu Dhabi, United Arab Emirates; Westmead Applied Research Centre (M A Godinho MBBS), University of Sydney, Westmead, NSW, Australia; Department of Hepatology (Prof A Goel DM), Sanjay Gandhi Postgraduate Institute of Medical Sciences, Lucknow, India; Department of Health Systems and Policy Research (M Golechha PhD), Indian Institute of Public Health, Gandhinagar, India; Department of Genetics (P Goleij MSc), Sana Institute of Higher Education, Sari, Iran; Universal Scientific Education and Research Network (USERN) (P Goleij MSc), Substance Abuse Prevention Research Center (B Mansouri PhD), Department of Rehabilitation and Sports Medicine (M Mirzaei MSc), Research Center for Environmental Determinants of Health (Prof E Sadeghi PhD), Department of Speech Therapy (A Shiani PhD), Department of Infectious Disease (Prof S Vaziri MD), Kermanshah University of Medical Sciences, Kermanshah, Iran; Department of Life Sciences (D Golinelli MD), Link Campus University, Rome, Italy; Hudson College of Public Health (S V Gopalani MPH), University of Oklahoma Health Sciences Center, Oklahoma City, OK, USA; Department of Health and Social Affairs (S V Gopalani MPH), Government of the Federated States of Micronesia, Palikir, Federated States of Micronesia; Department of Respiratory Medicine (H Goudarzi PhD), Center for Environmental and Health Sciences (H Goudarzi PhD), Hokkaido University, Sapporo, Japan; Blood and Marrow Transplantation and Cellular Therapy Program (A Goyal MD), Department of Gastroenterology and Hepatology (A Joseph MD), Department of Biomedical Data Science (S Park MD), Department of Radiology (S Ramasamy MD), Stanford University, Stanford, CA, USA; Department of Public Health and Preventive Medicine (Prof M Grivna PhD), Charles University, Prague, Czech Republic; Department of Epidemiology and Biostatistics (S Guan MD, Prof H Pan PhD, C Zhai MD), Anhui Medical University, Hefei, China; Post Graduate School of Public Health (G Guarducci MD), University of Siena, Siena, Italy; Department of Family and Community Medicine (M I M Gubari PhD), University Of Sulaimani, Sulaimani, Iraq; Health Directorate (S Guicciardi MD), Local Health Authority of Bologna, Bologna, Italy; Department of General Surgery (S Gulati MD), Dignity Health, Phoenix, AZ, USA; Diagnostic Radiology and Nuclear Medicine (D Gulisashvili MD), School of Medicine (P Habibzadeh MD), University of Maryland, Baltimore, MD, USA; Department of Community Medicine (D A Gunawardane MD, S N K Navaratna MD), University of Peradeniya, Kandy, Sri Lanka; Department of Internal Medicine (A K Gupta PharmD), Faculty of Medicine and Health Sciences (Prof N P Singh MD), Shree Guru Gobind Singh Tricentenary University, Gurugram, India; Non-communicable Division (NCD) (A K Gupta PharmD), Indian Council of Medical Research, Delhi, India; Department of Public Health (B Gupta PhD), Torrens University Australia, Melbourne, VIC, Australia; Department of Cardiology (R Gupta MD), Lehigh Valley Health Network, Allentown, PA, USA; Department of Epidemiology and Biostatistics (R Gupta MPH), Department of Chemistry and Biochemistry (S Malasala PhD), University of South Carolina, Columbia, SC, USA; Centre for Noncommunicable Diseases and Nutrition (R Gupta MPH), School of Pharmacy (M R Islam PhD), BRAC University, Dhaka, Bangladesh; Department of Preventive Cardiology (Prof R Gupta MD), Eternal Heart Care Centre & Research Institute, Jaipur, India; Department of Medicine (Prof R Gupta MD), Mahatma Gandhi University Medical Sciences, Jaipur, India; Department of Toxicology (S Gupta MSc),

Shriram Institute for Industrial Research, Delhi, India; School of Medicine (V Gupta PhD), Institute for Mental and Physical Health and Clinical Translation (IMPACT) (W Marx PhD), Deakin University, Geelong, VIC, Australia; School of Biotechnology (V Gupta PhD), Dublin City University, Dublin, Ireland; Faculty of Medicine Health and Human Sciences (Prof V K Gupta PhD), Macquarie Medical School (Y You PhD), Macquarie University, Sydney, NSW, Australia; Global Virus Network, Middle East Region, Shiraz, Iran (F Habibzadeh MD); Food Technology Research (Z Hadian PharmD), Food and Nutrition Research Institute, Tehran, Iran; Clinician Scientist Program (N Haep MD), Berlin Institute of Health, Berlin, Germany; Pharmaceutical Sciences Research Center (H Haghi-Aminjan PhD), Ardabil University of Medical Science, Ardabil, Iran; NYU Shanghai, Shanghai, China (B J Hall PhD); Department of Infectious Disease Epidemiology (S Haller MD), Robert Koch Institute, Berlin, Germany; Department of Public Health (S Haller MD), Charité Institute of Public Health, Berlin, Germany; Department of Family and Community Medicine (Prof R R Hamadeh PhD), College of Medicine and Medical Sciences (H Jahrami PhD), Arabian Gulf University, Manama, Bahrain; School of Health and Environmental Studies (Prof S Hamidi DrPH), Hamdan Bin Mohammed Smart University, Dubai, United Arab Emirates; The Warren Alpert Medical School (Z A Haq BA), Department of Internal Medicine (M F H Mohamed MSc), Brown University, Providence, RI, USA; Department of Population Sciences (Prof M Haque PhD), Department of Pharmaceutical Technology (I Hasan MPharm), University of Dhaka, Dhaka, Bangladesh; Medical Research Unit (H Harapan PhD), Universitas Syiah Kuala (Syiah Kuala University), Banda Aceh, Indonesia; Research Unit (J M Haro MD), University of Barcelona, Barcelona, Spain; Biomedical Research Networking Center for Mental Health Network (CiberSAM), Barcelona, Spain (J M Haro MD); Department of Zoology and Entomology (A I Hasaballah PhD), Botany and Microbiology Department (A M E Shehabeldine PhD), Al-Azhar University, Cairo, Egypt; Department of Public Health (M Hasan MPH), Tropical Disease and Health Research Center, Bangladesh, Dhaka, Bangladesh; Department of Biomedical Engineering and Public Health (S Hasan PhD), World University of Bangladesh, Dhaka, Bangladesh; Department of Radiology (M Hasanian MD), Arak University of Medical Sciences, Arak, Iran; Department of Pharmacy (Prof M S Hasnain PhD), Palamau Institute of Pharmacy, Daltonganj, India; Public Health Department (I Hassan MPH), Dalhatu Araf Specialist Hospital, Lafia, Nigeria; Department of Public Health (I Hassan MPH), Federal University of Lafia, Lafia, Nigeria; Independent Consultant, Tabriz, Iran (H Hassankhani PhD); Department of Diagnostic and Interventional Radiology and Neuroradiology (J Haubold MD), Institute of Artificial Intelligence in Medicine (J Haubold MD), University Hospital Essen, Essen, Germany; Skaane University Hospital (R J Havmoeller PhD), Skaane County Council, Malmö, Sweden; Faculty of Kinesiology (Prof J J Hebert PhD), University of New Brunswick, Fredericton, NB, Canada; School of Allied Health (Prof J J Hebert PhD), Murdoch University, Murdoch, WA, Australia; Independent Consultant, Santa Clara, CA, USA (G Heidari MD); Community-Oriented Nursing Midwifery Research Center (M Heidari PhD), Department of Epidemiology and Biostatistics (A Mohammadian-Hafshejani PhD), Students Research Committee (F Zarimeidani Dipl), Shahrekord University of Medical Sciences, Shahrekord, Iran; Institute of Psychology (B Helfer PhD), University of Wroclaw, Wroclaw, Poland; Meta Research Centre (B Helfer PhD), University of Wrocław, Wroclaw, Poland; Department of Medicine (M Hemmati MD), MedStar Health, Columbia, MD, USA; Department of Medicine (M Hemmati MD), Georgetown University, Washington DC, DC, USA; School of Business (Prof C Herteliu PhD), London South Bank University, London, UK; Department of Microbiology (K Hezam PhD), Taiz University, Taiz, Yemen; School of Medicine (K Hezam PhD), Nankai University, Tianjin, China; Division for Health Service Promotion (Y Hiraike PhD), Department of Mental Health (Prof N Kawakami PhD), Department of Global Health Policy (S Nomura PhD, S K Rauniyar PhD), University of Tokyo, Tokyo, Japan; School of Dentistry

(N Q Hoan DDS), Hanoi Medical University, Hanoi, Viet Nam; University of Western Australia, Perth, NSW, Australia (M E Hoque PhD, Prof K Takahashi PhD); Department of Pulmonology (N Horita PhD), Yokohama City University, Yokohama, Japan; National Human Genome Research Institute (NHGRI) (N Horita PhD), Center for Translation Research and Implementation Science (G A Mensah MD), National Institutes of Health, Bethesda, MD, USA; Department of Public Health and Informatics (S Hossain MS), Jahangirnagar University, Dhaka, Bangladesh; Department of Mechanical Engineering (S Hosseini PhD), Arkansas Tech University, Russellville, AR, USA; School of Health and Society (H Hosseinzadeh PhD, Z Ratan MSc), University of Wollongong, Wollongong, NSW, Australia; Institute of Research and Development (Prof M Hosseinzadeh PhD), Duy Tan University, Da Nang, Viet Nam; Department of Clinical Legal Medicine (S Hostiuc PhD), National Institute of Legal Medicine Mina Minovici, Bucharest, Romania; Institute for Occupational and Maritime Medicine (ZfAM) (H Hoven PhD), University Medical Center Hamburg-Eppendorf (UKE), Hamburg, Germany; Faculty of Medicine of Tunis (Prof M Hsairi MPH), University Tunis El Manar, Tunis, Tunisia; Department of Psychology (C Hu PhD), Tsinghua Vanke School of Public Health (Z Li PhD), Tsinghua University, Beijing, China; Research Division (M Huda PhD), ARCED Foundation, Dhaka, Bangladesh; Harvard Medical School (E N Hulland PhD), Harvard University, Boston, USA; Department of Biological Sciences and Chemistry (Prof J Hussain PhD), Natural and Medical Sciences Research Center (S Mohammadi PhD, A Ullah MS, S Ullah MSc, M Waqas PhD), School of Pharmacy (A K Philip PhD), University of Nizwa, Nizwa, Oman; Department of Biomolecular Sciences (N R Hussein PhD), University of Zakho, Zakho, Iraq; International Master Program for Translational Science (H Huynh BS), Graduate Institute of Biomedical Informatics (D N A Ningrum MPH), School of Public Health (Y L Samodra MPH, Y L Samodra MPH), Department of Clinical Pharmacy (M A Sarasmita PharmD), Global Health and Health Security Department (U Wongsin PhD), Taipei Medical University, Taipei, Taiwan; International Center for Nutrition and Information (N Ikeda PhD), National Institutes of Biomedical Innovation, Health and Nutrition, Tokyo, Japan; Division of Infectious Diseases (K S Ikuta MD), Veterans Affairs Greater Los Angeles, Los Angeles, CA, USA; Faculty of Medicine (I M Ilic PhD, Prof M M Santric-Milicevic PhD, I S Vujcic PhD), School of Public Health and Health Management (Prof M M Santric-Milicevic PhD), University of Belgrade, Belgrade, Serbia; Department of Epidemiology (Prof M D Ilic PhD), University of Kragujevac, Kragujevac, Serbia; College of Pharmacy (M Imam PhD), Department of Electrical Engineering (I Malik PhD), Prince Sattam bin Abdulaziz University, Al Kharj, Saudi Arabia; Department of Health Research (L R Inbaraj MD), ICMR National Institute for Research in Tuberculosis, Chennai, India; Faculty of Pharmacy (L M Irham BPharm), University of Ahmad Dahlan, Yogyakarta, Indonesia; Department of Biotechnology (M A Isa PhD), Sharda University, Greater Noida, India; Clinical Laboratory Department (F Ismail PhD), Tobruk University, Tobruk, Libya; Department of Blood Transmitted Diseases (F Ismail PhD), National Center for Disease Control, Tobruk, Libya; Department of Clinical Pharmacy & Pharmacy Practice (Prof N Ismail PhD), Faculty of Dentistry (S Selvaraj PhD), Asian Institute of Medicine, Science and Technology University, Bedong, Malaysia; Malaysian Academy of Pharmacy, Puchong, Malaysia (Prof N Ismail PhD); Public Health Department of Social Medicine (Prof H Iso MD), Graduate School of Medicine (Prof K Yamagishi MD), Department of Biostatistics and Data Science (Y Yasufuku MSc), Osaka University, Suita, Japan; Department of Health Services Research (M Iwagami PhD), Research and Development Center for Health Services (Prof K Yamagishi MD), University of Tsukuba, Tsukuba, Japan; Department of Global Health (C J Iwu-Jaja PhD), Risk and Resilience in Mental Disorders Unit (Prof D J Stein MD), South African Medical Research Council, Cape Town, South Africa (C A Nnaji MPH); Department of Global Health (C J Iwu-Jaja PhD), Department of Epidemiology (J L Tamuzi MSc), Department of Industrial Psychology (E Teye-Kwadjo PhD), Stellenbosch University, Cape

Town, South Africa; Research and Development Unit (L Jacob MD), Biomedical Research Networking Center for Mental Health Network (CiberSAM), Sant Boi de Llobregat, Spain; Faculty of Medicine (L Jacob MD), University of Versailles Saint-Quentin-en-Yvelines, Montigny-le-Bretonneux, France; Department of Health Studies (K H Jacobsen PhD), University of Richmond, Richmond, VA, USA; Department of Psychiatry (Z Saif MBA), Ministry of Health, Manama, Bahrain (H Jahrami PhD); Department of Leukemia (A Jain MD), The University of MD Anderson Cancer Center, Houston, TX, USA; Statistics Unit (N Jain MD), Riga Stradins University, Riga, Latvia; Department of Health and Safety (A A Jairoun PhD), Dubai Municipality, Dubai, United Arab Emirates; The World Academy of Sciences UNESCO, Trieste, Italy (Prof M Jakovljevic PhD); Shaanxi University of Technology, Hanzhong, China (Prof M Jakovljevic PhD); Department of Environmental Health Engineering (R Jalilzadeh Yengejeh PhD), Islamic Azad University, Ahvaz, Iran; Department of Neurosciences (Prof R G Jamora PhD), University of the Philippines Manila, Manila, Philippines; Institute for Neurosciences (Prof R G Jamora PhD), St Luke's Medical Center, Bonifacio Global City, Philippines; School of Pharmacy and Pharmacology (A Jatau PhD), Menzies Institute for Medical Research (F Pan PhD, J Tian PhD), University of Tasmania, Hobart, TAS, Australia; Department of Physiology (Prof S Javadov PhD), University of Puerto Rico Medical Sciences Campus, San Juan, Puerto Rico; Department of Biochemistry (Prof S Jayaram MD), Government Medical College, Mysuru, India; Department of Internal Medicine (B M Jeswani MBBS), GCS Medical College, Hospital & Research Centre, Ahmedabad, India; Melbourne School of Population and Global Health (H Jiang PhD, L Reifels PhD), School of Health Sciences (A Meretoja MD), University of Melbourne, Melbourne, VIC, Australia; Zoonoses Research Center (M Jokar DVM), Islamic Azad University, Karaj, Iran; Department of Clinical Sciences (M Jokar DVM), Department of Public Health (Y Sarikhani PhD), Jahrom University of Medical Sciences, Jahrom, Iran; Department of Microbiology (N Jomehzadeh PhD), Abadan School of Medical Sciences, Abadan, Iran; Institute of Molecular and Clinical Ophthalmology Basel, Basel, Switzerland (Prof J B Jonas MD); Department of Ophthalmology (Prof J B Jonas MD), Heidelberg University, Mannheim, Germany; Hungarian Health Management Association, Budapest, Hungary (T Joo PhD, T Palicz MD); Department of Biochemistry and Molecular Biology (V Joshi MD), Drexel University, West Reading, PA, USA; Department of Economics (C E Joshua BSc), National Open University, Benin City, Nigeria; Department of Family Medicine and Public Health (J J Jozwiak PhD), University of Opole, Opole, Poland; Institute of Family Medicine and Public Health (M Jürisson PhD), University of Tartu, Tartu, Estonia; School of Public Health (Z Kabir PhD), University College Cork, Cork, Ireland; Department of Dermatology (F Kaliyadan MD), King Faisal University, Hofuf, Saudi Arabia; Department of Endocrinology (S Kalra DM), Bharti Hospital Karnal, Karnal, India; Care and Public Health Research Institute (CAPHRI) (R Kamath MHA), Maastricht University, Maastricht, Netherlands; School of Graduate Studies (T Kanagasabai PhD), Meharry Medical College, Nashville, TN, USA; Sydney Eye Hospital (H Kandel PhD), South Eastern Sydney Local Health District, Sydney, NSW, Australia; Regional Institute for Population Studies (E Kanmiki MPH), University of Ghana, Accra, Ghana; Faculty of Dentistry (K K Kanmodi MPH), University of Puthisastra, Phnom Penh, Cambodia; Office of the Executive Director (K K Kanmodi MPH), Campaign for Health and Neck Cancer Education (CHANCE) Programme (A A Salami BDS), Cephas Health Research Initiative Inc, Ibadan, Nigeria; Dr S S Bhatnagar University Institute of Chemical Engineering & Technology (Prof S K Kansal PhD), Department of Anthropology (Prof K Krishan PhD), Institute of Forensic Science & Criminology (V Sharma PhD), Panjab University, Chandigarh, India; School of Health Professions and Human Services (I M Karaye MD), Hofstra University, Hempstead, NY, USA; Department of Anesthesiology (I M Karaye MD), Montefiore Medical Center, Bronx, NY, USA; Department of Physical Therapy and Health Rehabilitation (F Z Kashoo MSc), Majmaah University,

Majmaah, Saudi Arabia; Department of Medical Laboratory Sciences (W D Kassahun MSc), Woldia University, Woldia, Ethiopia; MRC/CSO Social and Public Health Sciences Unit (S V Katikireddi PhD), School of Cardiovascular and Metabolic Health (F E Petermann-Rocha PhD), University of Glasgow, Glasgow, UK; Surgery Research Unit (Prof J H Kauppila MD), Center for Environmental and Respiratory Health Research (I Shiue PhD), Martti Ahtisaari Institute (I Shiue PhD), University of Oulu, Oulu, Finland; International Research Center of Excellence (G A Kayode PhD), Institute of Human Virology Nigeria, Abuja, Nigeria; Julius Centre for Health Sciences and Primary Care (G A Kayode PhD), Copernicus Institute of Sustainable Development (G Koren PhD), Utrecht University, Utrecht, Netherlands; Open, Distance and eLearning Campus (Prof P N Keiyoro PhD), School of Nursing Sciences (M N Wanjau MA), University of Nairobi, Nairobi, Kenya; Eye Unit (Prof J H Kempen MD), MyungSung Medical College, Addis Ababa, Ethiopia; Centre for Adolescent Health (J A Kerr PhD), Department of Critical Care and Neurosciences (Prof R G Weintraub MB), Murdoch Childrens Research Institute, Parkville, VIC, Australia; Department of Psychological Medicine (J A Kerr PhD), University of Otago, Christchurch, New Zealand; Department of Human Nutrition (E Kesse-Guyot PhD), National Research Institute for Agriculture, Food and Environment, Jouy-en-Josas, France; Department of Health, Medicine and Human Biology (M Touvier PhD), Sorbonne Paris Nord University, Bobigny, France (E Kesse-Guyot PhD); Amity Institute of Forensic Sciences (H Khajuria PhD, B P Nayak PhD), Amity Institute of Pharmacy (K Munjal PhD), Amity University, Noida, India; Research Center for Hydatid Disease in Iran (F Khamesipour PhD), Department of Clinical Biochemistry (M Nematollahi PhD), Kerman University of Medical Sciences, Kerman, Iran; Population Science Department (M Khan PhD), Jatiya Kabi Kazi Nazrul Islam University, Mymensingh, Bangladesh; Primary Care Department (M A Khan MSc), NHS North West London, London, UK; College of Health, Wellbeing and Life Sciences (Prof K Khatab PhD), Sheffield Hallam University, Sheffield, UK; College of Arts and Sciences (Prof K Khatab PhD), Ohio University, Zanesville, OH, USA; Faculty of Nursing (H Khatatbeh PhD), Jerash University, Jerash, Jordan; Global Consortium for Public Health Research (Prof M Khatib PhD), Datta Meghe Institute of Higher Education and Research, Wardha, India; Department of Biochemistry (F Khidri PhD), Liaquat University Of Medical and Health Sciences, Jamshoro, Pakistan; Department of Internal Medicine (A A Khosla MD), Corewell Health East William Beaumont University Hospital, Royal Oak, MI, USA; Research Department (M Khosrowjerdi PhD), Inland Norway University of Applied Sciences, Elverum, Norway; Texas A&M Transportation Institute (H Khreis PhD), Texas A&M University, College Station, TX, USA; Faculty of Health Sciences (H Khusun PhD), University of Muhammadiyah Prof Dr Hamka, Jakarta, Indonesia; Program Division (H Khusun PhD), SEAMEO Regional Center for Food and Nutrition, Jakarta, Indonesia; Department of Preventive Medicine (K Kim MD), Yonsei University, Seoul, South Korea; Cardiovascular Disease Initiative (M Kim MD), Broad Institute of MIT and Harvard, Cambridge, MA, USA; School of Traditional Chinese Medicine (Y Kim PhD), Xiamen University Malaysia, Sepang, Malaysia; Millennium Prevention, Westwood, MA, USA (R W Kimokoti MD); School of Health Sciences (Prof A Kisa PhD), Kristiania University College, Oslo, Norway; Department of International Health and Sustainable Development (Prof A Kisa PhD), Tulane University, New Orleans, LA, USA; Department of Nursing and Health Promotion (S Kisa PhD), Oslo Metropolitan University, Oslo, Norway; Department of Disease Burden (A S Knudsen PhD), GBD Collaborating Unit (Prof S Vollset DrPH), Norwegian Institute of Public Health, Bergen, Norway; Department of Urology (J Kong MD), The Department of Gynecology (S Qiu MD), Sun Yat-sen University, Guangzhou, China; Department of Neurosurgery (M Korja PhD, I Rautalin PhD), Neurology Unit (A Meretoja MD), Breast Surgery Unit (T J Meretoja MD), Helsinki University Hospital, Helsinki, Finland; Department of Epidemiology and Evidence-Based Medicine (V A Korshunov PhD, P D Lopukhov PhD), IM Sechenov First

Moscow State Medical University, Moscow, Russia; Department of General Practice – Family Medicine (Prof O Korzh DSc), Kharkiv National Medical University, Kharkiv, Ukraine; Independent Consultant, Jakarta, Indonesia (S Kosen MD); Department of Internal and Pulmonary Medicine (Prof P A Koul MD), Sheri Kashmir Institute of Medical Sciences, Srinagar, India; Kasturba Medical College, Mangalore (S Koulmane Laxminarayana MD), Department of Health Innovation (U Rani PhD), Manipal College of Nursing (R Yesodharan MSc), Manipal Academy of Higher Education, Udupi, India; Evidence Synthesis Unit (Y Krishnamoorthy MD), Partnership for Research, Opportunity, Planning, Upskilling, and Leadership (PROPUL) Evidence, Chennai, India; Department of Physiology (B Krishnan MD), Pravara Institute of Medical Sciences (Deemed to be University), Loni, India; Department of Demography (Prof B Kuate Defo PhD), Department of Social and Preventive Medicine (Prof B Kuate Defo PhD), University of Montreal, Montreal, QC, Canada; Faculty of Medicine (B Kucuk Bicer PhD), Gazi University, Ankara, Turkiye; Department of Mathematics (M Kuddus PhD), University of Rajshahi, Rajshahi, Bangladesh; Department of Biochemistry (Prof M Kuddus PhD), University of Hail, Hail, Saudi Arabia; University of Environment and Sustainable Development, Somanya, Ghana (N Kugbey PhD); Department of Pediatrics (I Kuitunen PhD), Kuopio University Hospital, Kuopio, Finland; Institute of Clinical Medicine (I Kuitunen PhD), University of Eastern Finland, Kuopio, Finland; Department of Medicine (V Kulkarni MS), Digital Health and Informatics Directorate (Prof S M McPhail PhD), Queensland Health, Brisbane, QLD, Australia; Department of Internal Medicine (A Kumar MD), Cabrini Institute, Akron, OH, USA; Department of Economics (V Kumar PhD), Parul University, Vadodara, India; Centre for Studies in Economics and Planning (V Kumar PhD), Central University of Gujarat, Gandhinagar, India; Global Health Institute (S Kundu MPH), North South University, Dhaka, Bangladesh; Department of Nutrition and Food Science (S Kundu MPH), Department of Biochemistry and Food Analysis (N Roy PhD), Department of Post-Harvest Technology and Marketing (A Sayeed MSc), Patuakhali Science and Technology University, Patuakhali, Bangladesh; Department of Medicine (O P Kurmi PhD), Department of Psychiatry and Behavioural Neurosciences (A T Olagunju MD), Health Information Research Unit (O O Olasupo PhD), McMaster University, Hamilton, ON, Canada; National Research and Innovation Agency Republic of Indonesia (BRIN), Jakarta, Indonesia (A Kusnali LLB, H U Pangaribuan MSc); Department of Health Services Research and Management (D Kusuma DSc), City University of London, London, UK; Faculty of Public Health (D Kusuma DSc, Prof I Trihandini PhD), University of Indonesia, Depok, Indonesia; Department of Pediatric Oncology (Prof T Kutluk MD), Hacettepe University, Ankara, Turkiye; Institute for Social and Health Sciences (Prof L Laflamme PhD), University of South Africa, Pretoria, South Africa; Department of Health Policy and Strategy (Prof C Lahariya MD), Foundation for People-centric Health Systems, New Delhi, India; SD Gupta School of Public Health (Prof C Lahariya MD), Indian Institute of Health Management Research University, Jaipur, India; School of Digital Science (D T C Lai PhD), Institute of Applied Data Analytics (D T C Lai PhD), Faculty of Science (E Leong PhD), Universiti Brunei Darussalam (University of Brunei Darussalam), Bandar Seri Begawan, Brunei; Indian Council of Medical Research, New Delhi, India (D K Lal MD); Department of Public Health (Prof T Lallukka PhD), Department of Virology (F Zakham PhD), University of Helsinki, Helsinki, Finland (T J Meretoja MD); NEVES Society for Patient Safety, Budapest, Hungary (J Lám PhD); Division of Cancer Epidemiology and Genetics (Q Lan PhD), National Cancer Institute, Rockville, MD, USA; Unit of Genetics and Public Health (Prof I Landires MD), Institute of Medical Sciences, Las Tablas, Panama; Ministry of Health, Herrera, Panama (Prof I Landires MD); Department of Health Sciences (DISSAL) (F Lanfranchi MD), University of Genoa, Genoa, Italy; Department of Psychiatry and Psychotherapy (B Langguth PhD), University of Regensburg, Regensburg, Germany; Chief Medical Office (Prof V C Lansingh PhD), HelpMeSee, New York, NY, USA;

Mexican Institute of Ophthalmology, Queretaro, Mexico (Prof V C Lansingh PhD); Department of Behavioural Sciences and Learning (A Laplante-Lévesque PhD), Linköping University, Linköping, Sweden; Department of Clinical Chemistry and Pharmacology (Prof A O Larsson PhD), Uppsala University Hospital, Uppsala, Sweden; Department of Otorhinolaryngology (S Lasrado MS), Father Muller Medical College, Mangalore, India; International Society Doctors for the Environment, Arezzo, Italy (P Lauriola MD); Faculty of Medicine (H Le MD, N Le MD), Department of General Medicine (V T Nguyen MD), University of Medicine and Pharmacy at Ho Chi Minh City, Ho Chi Minh City, Viet Nam (T T Le MD); Department of Cardiovascular Research (H Le MD, N Le MD), Methodist Hospital, Merrillville, IN, USA; Health Economics Division (L K D Le PhD), Monash University, Burwood, VIC, Australia; College of Optometry (J L Leasher OD), Nova Southeastern University, Fort Lauderdale, FL, USA; Department of Medical Science (M Lee PhD), Ajou University School of Medicine, Suwon, South Korea; Department of Precision Medicine (Prof S W Lee MD), Sungkyunkwan University, Suwon-si, South Korea; School of Pharmacy (S W H Lee PhD, S Shrestha PharmD, Y Wong PhD), School of Medicine (V Subramaniyan PhD), Monash University, Subang Jaya, Malaysia; School of Pharmacy (S W H Lee PhD), Taylor's University Lakeside Campus, Subang Jaya, Malaysia; Department of Preventive Medicine (Prof Y Lee PhD), Department of Health Policy and Management (S Park PhD), Korea University, Seoul, South Korea (Prof M Shin PhD); National Centre for Youth Substance Abuse Research (J Leung PhD), The University of Queensland, St Lucia, QLD, Australia; Department of Health Promotion and Health Education (M Li PhD), National Taiwan Normal University, Taipei, Taiwan; The First Affiliated Hospital of Guangzhou Medical University (W Li MD), Guangzhou Medical University, Guangzhou, China; National Clinical Research Center for Cardiovascular Diseases (Y Li PhD), Chinese Academy of Medical Sciences, Shenzhen, China; College of Public Health (R Lin PhD), China Medical University, Taichung, Taiwan; Asbestos Diseases Research Institute, Concord, NSW, Australia (R Lin PhD); The Center for Drug Safety and Policy Research (S Lin MS), Department of Sociology (J Nie PhD), Xi'an Jiaotong University, Xi'an, China; Med-X Institute (J Liu PhD), The First Affiliated Hospital of Xi'an Jiaotong University, Xi'an, China; School of Public Health (J Liu PhD), Xi'an Jiaotong University Health Science Center, Xi'an, China; Department of Epidemiology and Biostatistics (Prof J Liu PhD), Institute of Child and Adolescent Health (Y Song PhD), School of Public Health (H Zhang MS), Department of Global Health (Prof Z Zhang PhD), Peking University, Beijing, China; Department of Psychiatry (R T Liu PhD), Harvard Medical School, Boston, MA, USA; Institute for Health and Environment (W Liu PhD), Chongqing University of Science and Technology, Chongqing, China; Institute for Physical Activity and Nutrition (K M Livingstone PhD), Department of Psychology (M A Stokes PhD), Deakin University, Burwood, VIC, Australia; Department of Molecular Epidemiology (E Llanaj PhD), German Institute of Human Nutrition Potsdam-Rehbrücke, Potsdam, Germany; German Center for Diabetes Research (DZD), München-Neuherberg, Germany (E Llanaj PhD); Department of Public Health (A Lohiya MD), Kalyan Singh Super Specialty Cancer Institute, Lucknow, India; Department of Physical Medicine and Nursing (R López-Bueno PhD), University of Zaragoza, Zaragoza, Spain; Department of Musculoskeletal Disorders (R López-Bueno PhD), National Research Centre for the Working Environment, Copenhagen, Denmark; Institute of Nutritional Sciences (Prof S Lorkowski PhD), Friedrich Schiller University Jena, Jena, Germany; Competence Cluster for Nutrition and Cardiovascular Health (nutriCARD), Jena, Germany (Prof S Lorkowski PhD); School of Medicine (Prof G Lucchetti PhD), Federal University of Juiz de Fora, Juiz de Fora, Brazil; Center for Evidence-Based and Translational Medicine (L Luo MPH), Department of Epidemiology and Biostatistics (Prof C Yu PhD), School of Medicine (Z Zhang PhD), School of Health Sciences (X G Zhao PhD), Wuhan University, Wuhan, China; Department of Epidemiology (H lv BA), Chinese Center for Disease Control and Prevention, Shenyang, China; Centre for

Public Health and Wellbeing (Z Ma PhD), University of the West of England, Bristol, UK; Faculty of Veterinary Medicine (M Mabrok PhD), Suez Canal University, Ismailia, Egypt; Department of Microbiology and Parasitology (M Mabrok PhD), King Salman International University, South of Sinai, Egypt; 2nd Department of Propaedeutic Surgery (N Machairas PhD), Department of Biophysics (Prof P Papadopoulou PhD), 3rd Department of Cardiology (M Spartalis PhD), University of Athens, Athens, Greece; Periodontal Department (Prof M Machoy PhD), Department of Propedeutics of Internal Diseases & Arterial Hypertension (Prof T Miazgowski MD), Pomeranian Medical University, Szczecin, Poland; Ophthalmology Department (M Magdy Abd El Razek MSc), Ministry of Health & Population, Aswan, Egypt; Department of Forensic Medicine & Toxicology (D Mahadeshwara Prasad MD), Mysore Medical College & Research Institute, Mysooru, India; Department of Health & Family Welfare (D Mahadeshwara Prasad MD), Government of Karnataka, Bangalore, India; School of Pharmacy (S B Maharaj DBA), Department of Paraclinical Sciences (S Umakanthan MD), University of the West Indies, St Augustine, Trinidad and Tobago; Planetary Health Alliance, Boston, MA, USA (S B Maharaj DBA); Department of Clinical and Hospital Pharmacy (M A Mahmoud PhD), Taibah University, Al-Madinah Al-Munawarrah, Saudi Arabia; Center for Health & Nature (O M Makram MD), Houston Methodist Hospital, Houston, TX, USA; Cyprus International Institute for Environmental and Public Health (K C Makris PhD), Cyprus University of Technology, Limassol, Cyprus; Department of Forensic Medicine (Prof V Maled MD), Shri Dharmasthala Manjunatheshwara University, Dharwad, India; Department of Forensic Medicine (Prof V Maled MD), Department of Infectious Diseases and Microbiology (P A Shah MBBS), Rajiv Gandhi University of Health Sciences, Bangalore, India; Rama Medical College Hospital and Research Centre, Uttar Pradesh, India (K Malhotra MBBS); Department of Health Research (L A Malinga PhD), Ministry of Health, Pretoria, South Africa; General Surgery Department I (A Manda MD), Department of General Surgery (I Negoi PhD), Fourth Department of General Surgery (D Serban PhD), Emergency University Hospital Bucharest, Bucharest, Romania; Smidt Heart Institute (Y Manla MD), Cedars-Sinai Medical Center, Los Angeles, CA, USA; Information and Communication Technology Research Pole (Lab-STICC) (Prof A Mansour PhD), ENSTA Bretagne, Brest, France; Laboratory of Public Health (Prof L G Mantovani DSc), Instituto Auxologico Italiano IRCCS (Italian Auxological Institute), Milan, Italy; Biomedical Engineering Research Center (CREB) (H Marateb PhD), Universitat Politècnica de Catalunya (Barcelona Tech - UPC), Barcelona, Spain; Department of Artificial Intelligence (H Marateb PhD), Smart University of Medical Sciences, Tehran, Iran; Department of Economics (Prof G Martinez PhD), Autonomous Technology Institute of Mexico, Mexico City, Mexico; Non-communicable Diseases and Mental Health Department (R Martinez-Piedra BSc), Pan American Health Organization, Washington, DC, USA; Indonesian Public Health Association, Surabaya, Indonesia (S Martini PhD); Campus Fortaleza (F R Martins-Melo PhD), Federal Institute of Education, Science and Technology of Ceará, Fortaleza, Brazil; Department of Nutrition and Dietetics (M Martorell PhD), University of Concepcion, Concepción, Chile; Centre for Healthy Living (M Martorell PhD), University of Concepción, Concepción, Chile; Department of Pharmacy (S Maryam PharmD), Bahauddin Zakariya University, Multan, Pakistan; Department of Anatomy, Genetics and Biomedical Informatics (Y Mathangasinghe MD), Postgraduate Institute of Medicine (S N K Navaratna MD), University of Colombo, Colombo, Sri Lanka; Division of Immunology, Immunity to Infection and Respiratory Medicine (A G Mathioudakis PhD), University of Manchester, Manchester, UK; North West Lung Centre (A G Mathioudakis PhD), Manchester University NHS Foundation Trust, Manchester, UK; Department of Medicine (J Mattumpuram MD), University of Louisville, Louisville, KY, USA; Research Division (P K Maulik PhD), The George Institute for Global Health, New Delhi, India; Orthopedic Trauma Pathology Department (A Mazzotti PhD), IRCCS, Bologna, Italy;

National Centre for Register-based Research (Prof J J McGrath MD), Research Unit for Global Health (R Shrestha MPH), Aarhus University, Aarhus, Denmark; Australian Centre for Health Services Innovation (Prof S M McPhail PhD), Queensland University of Technology, Kelvin Grove, QLD, Australia; Department of Healthcare (E A Mechili PhD), University of Vlora, Vlora City, Albania; Clinic of Social and Family Medicine (E A Mechili PhD), Department of Medicine (Prof A Tsatsakis DSc), University of Crete, Heraklion, Greece; Institute for Agricultural and Nutritional Sciences (T Meier PhD), Martin Luther University Halle-Wittenberg, Halle, Germany; Office of Innovation (T Meier PhD), Competence Cluster for Nutrition and Cardiovascular Health (nutriCARD), Halle, Germany; School of Nursing and Midwifery (B Mekonnen MPH), School of Exercise and Nutrition Sciences (N Subedi MPH), Department of Health Economics (T Tolossa MPH), Deakin University, Melbourne, VIC, Australia; Department of Public Health (B Mengist MPH), Public Health Department (T Y Tiruye PhD), Department of Human Nutrition and Food Sciences (E G Wassie MSc), Debre Markos University, Debre Markos, Ethiopia; Eunice Kennedy Shriver National Institute of Child Health and Human Development (L G Mensah MD), National Institute of Health, Bethesda, MD, USA; International Dx Department (A A Mentis MD), BGI Genomics, Copenhagen, Denmark; University Centre Varazdin (T Mestrovic PhD), University North, Varazdin, Croatia; Department of Pharmacology (Prof K D Mettananda PhD), Department of Paediatrics (Prof S Mettananda DPhil), University of Kelaniya, Ragama, Sri Lanka; Clinical Medicine Department (Prof K D Mettananda PhD), University Paediatrics Unit (Prof S Mettananda DPhil), Colombo North Teaching Hospital, Ragama, Sri Lanka; Anaesthesiology Department (G Micha PhD), "Helena Venizelou" General and Maternity Hospital, Athens, Greece; Department of Epidemiology (I Michalek PhD), National Cancer Registry (I Michalek PhD), Maria Sklodowska-Curie National Research Institute of Oncology, Warsaw, Poland; Pacific Institute for Research & Evaluation, Calverton, MD, USA (T R Miller PhD); School of Public Health (T R Miller PhD), School of Population Health (D J Weiss PhD), Curtin University, Perth, WA, Australia; County of Cook (M Mirarefin MPH), Office of The Medical Examiner, Chicago, NV, USA; Department of Medical Sciences (A Mirijello MD), IRCCS Casa Sollievo della Sofferenza General Hospital, San Giovanni Rotondo, Italy; Internal Medicine Programme (Prof E M Mirrakhimov PhD), Kyrgyz State Medical Academy, Bishkek, Kyrgyzstan; Department of Atherosclerosis and Coronary Heart Disease (Prof E M Mirrakhimov PhD), National Center of Cardiology and Internal Disease, Bishkek, Kyrgyzstan; Center for Palliative and Supportive Care (A Mirshahi MSc), Comprehensive Cancer Center (G Naik MPH), Department of Health Policy & Organization (M Rahim MA), Department of Health Services Administration (M Rahim MA), Department of Psychology (D C Schwebel PhD), School of Medicine (Prof J A Singh MD), University of Alabama at Birmingham, Birmingham, AL, USA; Division of Cardiology (A K Mishra MD), St Vincent College, Worcester, MA, USA; College of Healthcare Management and Economics (V Mishra PhD), Gulf Medical University, Ajman, United Arab Emirates; Research and Development (V Mishra PhD), Panacea Institute of Interdisciplinary Research and Education, Varanasi, India; Department of Forensic Medicine and Toxicology (C Mittal MD), Dr B C Roy Multi-Specialty Medical Research Centre, Kharagpur, India; Institute of Addiction Research (ISFF) (B Moazen MSc), Frankfurt University of Applied Sciences, Frankfurt am Main, Germany; Foundation for Liver Research (G Mocciaro PhD), Foundation for Liver Research, London, UK; New York University Abu Dhabi, Abu Dhabi, United Arab Emirates (A Z Mohamed PhD); College of Health Science (A I Mohamed MSc), College of Applied and Natural Science (J Mohamed MSc), University of Hargeisa, Hargeisa, Somalia; Molecular Biology Unit (N S Mohamed MSc), Bio-Statistical and Molecular Biology Department (N S Mohamed MSc), Sirius Training and Research Centre, Khartoum, Sudan; Department of Public Health (H Mohammed MPH, Y M Tefera MPH), Dire Dawa University, Dire Dawa, Ethiopia; Department of

Pharmaceutical Sciences (S Mohammed PhD), Notre Dame of Maryland University, Baltimore, MD, USA; Department of Pharmacy (S Mohammed PhD), Mizan-Tepi University, Mizan, Ethiopia; Health Systems and Policy Research Unit (S Mohammed PhD), Department of Community Medicine (A A Olorukooba MSc), Department of Surgery (M A Tolani FWACS), Ahmadu Bello University, Zaria, Nigeria; Clinical Epidemiology and Public Health Research Unit (L Monasta DSc, L Ronfani PhD, G Zamagni MSc), Burlo Garofolo Institute for Maternal and Child Health, Trieste, Italy; Department of Biomedical and Dental Sciences and Morphofunctional Imaging (Prof S Mondello MD), Messina University, Messina, Italy; Faculty of Medicine (A Moodi Ghalibaf MD), Medical Toxicology & Drug Abuse Research Center (M Rezaei MD), Birjand University of Medical Sciences, Birjand, Iran; Centre for Neonatal and Paediatric Infection (C E Moore PhD), St George's University of London, London, UK; Department of Epidemiology and Biostatistics (Y Moradi PhD), Kurdistan University of Medical Sciences, Sanandaj, Iran; Computer, Electrical, and Mathematical Sciences and Engineering Division (P Moraga PhD), King Abdullah University of Science and Technology, Thuwal, Saudi Arabia; International Laboratory for Air Quality and Health (Prof L Morawska PhD), School of Public Health and Social Work (M T N Tran PhD), Queensland University of Technology, Brisbane, QLD, Australia; Department of Public Health (Prof R S Moreira PhD), Oswaldo Cruz Foundation, Recife, Brazil; Department of Public Health (Prof R S Moreira PhD), Federal University of Pernambuco, Recife, Brazil; Department of Biology and Biological Engineering (J Morze PhD), Chalmers University of Technology, Gothenburg, Sweden; College of Medical Sciences (J Morze PhD), SGMK Copernicus University, Warsaw, Poland; Department of Health Policy (Prof E Mossialos PhD), London School of Economics and Political Science, London, UK; Unit of Pharmacotherapy, Epidemiology and Economy (S Mubarik PhD), University Medical Center Groningen (Prof M J Postma PhD), Department of Internal Medicine (P Vart PhD), University of Groningen, Groningen, Netherlands; Federal Institute for Population Research, Wiesbaden, Germany (Prof U O Mueller MD); Center for Population and Health, Wiesbaden, Germany (Prof U O Mueller MD); Department of Surgery (F Mulita PhD, G Verras MD), General University Hospital of Patras, Patras, Greece; Faculty of Medicine (F Mulita PhD), Department of Internal Medicine (G Ntaios PhD), Department of Emergency Medicine (I Pantazopoulos PhD), University of Thessaly, Larissa, Greece; Clinical Epidemiology Research Unit (E Murillo-Zamora PhD), Mexican Institute of Social Security, Villa de Alvarez, Mexico; Postgraduate in Medical Sciences (E Murillo-Zamora PhD), Universidad de Colima, Colima, Mexico; Surgery Department (A Musina MD), Department of Medical Oncology (S R Volovat PhD), University of Medicine and Pharmacy "Grigore T Popa" Iasi, Iaşi, Romania; Second Surgical Unit (A Musina MD), Department of Medical Oncology (S R Volovat PhD), Regional Institute of Oncology, Iaşi, Romania; Department of Pediatrics & Pediatric Pulmonology (Prof G Mustafa MD), Institute of Mother & Child Care, Multan, Pakistan; Department of Research Methodology (S Muthu MS), Orthopaedic Research Group, Coimbatore, India; Department of Biotechnology (S Muthu MS), Karpagam Academy of Higher Education (Deemed to be University), Coimbatore, India; Prince Fahad bin Sultan Chair for Biomedical Research (S Muthupandian PhD), University of Tabuk, Tabuk, Saudi Arabia; Center for Global Health Research (Prof R Muthusamy PhD), Saveetha Medical College and Hospitals (S R Pandi-Perumal MSc), Centre of Molecular Medicine and Diagnostics (COMManD) (Prof S Patil PhD), Saveetha University, Chennai, India; Department of Neuropsychiatry (W Myung PhD), Department of Food and Nutrition (A P Okekunle PhD), Seoul National University, Seoul, South Korea; Department of Neuropsychiatry (W Myung PhD), Seoul National University Bundang Hospital, Seongnam, South Korea; Elderly Health Research Center (A Nafei PhD), Research and Academic Institution, Tehran, Iran; Research and Analytics Department (A J Nagarajan MTech), Initiative for Financing Health and Human Development, Chennai, India; Department

of Research and Analytics (A J Nagarajan MTech), Bioinsilico Technologies, Chennai, India; Institute of Epidemiology and Medical Biometry (Prof G Nagel PhD), Ulm University, Ulm, Germany; Faculty of Pharmacy (F Nainu PhD), Hasanuddin University, Makassar, Indonesia; Department of Community Medicine (T S Nair MD), MOSC Medical College, Kolenchery, India; Internal Medicine (D P Nanavaty MD), The Brooklyn Hospital Center, Brooklyn, NY, USA; Suraj Eye Institute, Nagpur, India (V Nangia MD); Mysore Medical College and Research Institute (Prof S Narasimha Swamy MD), Government Medical College, Mysore, India; Department of Biotechnology (M Naveed PhD), University of Central Punjab, Lahore, Pakistan; Department of Disease Control and Environmental Health (R Ndejjo MSc), Makerere University, Kampala, Uganda; School of Pharmacy (S O Nduaguba PhD), West Virginia University, Morgantown, WV, USA; Department of Cardiology (R I Negoi PhD), Cardio-Aid, Bucharest, Romania; Faculty of Medicine (Prof C Nejjari PhD), Euromed University of Fes, Fez, Morocco; Faculty of Medicine (Prof C Nejjari PhD), University Sidi Mohammed Ben Abdellah, Fez, Morocco; Department of Community Medicine (S Nepal MD), Kathmandu University, Palpa, Nepal; Department of Health Sciences (S Neupane PhD), University of Tampere, Tampere, Finland; Department of Public Health (G Nguefack-Tsague PhD), University of Yaoundé I, Yaoundé, Cameroon; Department of Biological Sciences (J W Ngunjiri DrPH), University of Embu, Embu, Kenya; Department of Medical Engineering (D H Nguyen BS), University of South Florida, Tampa, FL, USA; Department of Pediatrics (N N Nguyen MD), Children's Hospital 2, Ho Chi Minh City, Viet Nam; Department of Surgery (P T Nguyen MD), Danang Family Hospital, Danang, Viet Nam; Institute for Cancer Control (P T Nguyen MPH), National Cancer Center, Tokyo, Japan; Graduate School of Public Health (P T Nguyen MPH), St Luke's International University, Chuo-ku, Japan; Molecular Neuroscience Research Center (D Nguyen Tran Minh MD), Shiga University of Medical Science, Shiga, Japan; International Islamic University Islamabad, Islamabad, Pakistan (R K Niazi PhD); Public Health Department (D N A Ningrum MPH), Universitas Negeri Semarang (State University of Semarang), Kota Semarang, Indonesia; Faculty of Applied Sciences and Technology (E A Noman PhD), Universiti Tun Hussein Onn Malaysia, Johor, Malaysia; Department of Health Policy and Management (S Nomura PhD), Keio University, Tokyo, Japan; Department of Clinical Sciences (Prof B Norrving PhD), Lund University, Lund, Sweden; Department of Paediatrics (C A Nri-Ezedi MD), Nnamdi Azikiwe University, Awka, Nigeria; The Cardiac Clinic (Prof M Ntsekhe PhD), Groote Schuur Hospital, Cape Town, South Africa; Department of Public Health (D Nurrika PhD), Banten School of Health Science, South Tangerang, Indonesia; Ministry of Research, Technology and Higher Education (D Nurrika PhD), Higher Education Service Institutions (LL-DIKTI) Region IV, Bandung, Indonesia; School of Nursing (J Nutor PhD), Department of Epidemiology and Biostatistics (M Teramoto MD), Department of Bioengineering and Therapeutic Sciences (Prof M S Zastrozhin PhD), University of California San Francisco, San Francisco, CA, USA; Department of Applied Economics and Quantitative Analysis (Prof B Oancea PhD), University of Bucharest, Bucharest, Romania; Department of Medicine (M J O'Donnell PhD), National University of Ireland - Galway, Galway, Ireland; PSSM Data Sciences (M Oduro PhD), Pfizer Inc, Groton, CT, USA; Department of Nursing Science (Prof A A Ogunfowokan PhD), Obafemi Awolowo University, Ile Ife, Nigeria; Department of Preventive Medicine (I Oh PhD), Department of Pediatrics (Prof D Yon MD), Kyung Hee University, Seoul, South Korea; Health Promotion Research Center (H Okati-Aliabad PhD), Zahedan University of Medical Sciences, Zahedan, Iran; Independent Consultant, Sydney, NSW, Australia (S R Okeke PhD); School of Pharmacy (O C Okonji MSc), University of the Western Cape, Cape Town, South Africa; Department of Psychiatry (A T Olagunju MD), University of Lagos, Lagos, Nigeria; Department of Nursing Science (M I Olatubi PhD), Bowen University, Iwo, Nigeria; Associação Brasileira de Cefaleia em Salvas e Enxaqueca (A B Oliveira PhD), ABRACES, São Paulo, Brazil; Cardiology Department (G M M Oliveira PhD), Federal University of Rio de

Janeiro, Rio de Janeiro, Brazil; Slum and Rural Health Initiative Research Academy (I I Olufadewa MHS), Slum and Rural Health Initiative, Ibadan, Nigeria; Centre for Healthy Start Initiative, Lagos, Nigeria (B O Olusanya PhD, J O Olusanya MBA); Department of Pharmacology and Toxicology (Prof H A Omar PhD), Beni-Suef University, Beni-Suef, Egypt; Surgery Department (G L Omer MD), Sulaimani University, Sulaimani, Iraq; ENT Department (G L Omer MD), Tor Vergata University of Rome, Rome, Italy; Non-communicable Disease Prevention Unit (S Ong FAMS), Ministry of Health, Bandar Seri Begawan, Brunei; Early Detection & Cancer Prevention Services (S Ong FAMS), Pantai Jerudong Specialist Centre, Bandar Seri Begawan, Brunei; Department of Biomedical Sciences (K I Onyedibe PhD), Mercer University School of Medicine, Macon, GA, USA; School of Nursing (A F Oppong MSc), University of Connecticut, Storrs, CT, USA; Department of Pharmacotherapy and Pharmaceutical Care (M Ordak PhD), Department of Biochemistry and Pharmacogenomics (M Zielińska MPharm), Medical University of Warsaw, Warsaw, Poland; Sickle Cell Unit (V N Orish PhD), Ho Teaching Hospital, Ho, Ghana; Department of Neurology (R Ornello PhD), ASL Avezzano-Sulmona-L'Aquila, L'Aquila, Italy; Substance Related Harms Division (H M Orpana PhD), Public Health Agency of Canada, Ottawa, ON, Canada; School of Epidemiology and Public Health (H M Orpana PhD), University of Ottawa, Ottawa, ON, Canada; Department of Nephrology and Hypertension (Prof A Ortiz MD), The Institute for Health Research Foundation Jiménez Díaz University Hospital, Madrid, Spain; One Health Global Research Group (Prof E Ortiz-Prado PhD), Universidad de las Americas (University of the Americas), Quito, Ecuador; Department of Optometry and Vision Science (U L Osuagwu PhD), University of KwaZulu-Natal, KwaZulu-Natal, South Africa; Laboratory of Public Health Indicators Analysis and Health Digitalization (N Otstavnov BA, S S Otstavnov PhD), Moscow Institute of Physics and Technology, Dolgoprudny, Russia; Department of Project Management (S S Otstavnov PhD), Department of Health Care Administration and Economics (Prof V Vlassov MD), National Research University Higher School of Economics, Moscow, Russia; Division of Infectious Diseases (Prof A Ouyahia PhD), University Hospital of Setif, Setif, Algeria; Department of Respiratory Medicine (Prof M P P A DNB), Department of Oral and Maxillofacial Surgery (C S N PhD), Jagadguru Sri Shivarathreeswara University, Mysore, India; Vicerrectorado de Investigacion (K Pacheco-Barrios MD), Universidad San Ignacio de Loyola, Lima, Peru; Department of Forensic Medicine and Toxicology (J Padubidri MD), Kasturba Medical College, Mangalore, Mangalore, India; Department of Neurology (Prof P K Pal DM), National Institute of Mental Health and Neurosciences, Bangalore, India; Department of Public Health (A Pana PhD), Babes Bolyai University, Cluj Napoca, Romania; Department of Health Metrics (A Pana PhD), Center for Health Outcomes & Evaluation, Bucharest, Romania; Department of Zoology (S K Panda PhD), Department of Analytical and Applied Economics (Prof H S Rout PhD, C K Swain MPhil), Utkal University, Bhubaneswar, India; Department of Animal Physiology and Neurobiology (S K Panda PhD), Department of Cardiovascular Sciences (A Schuermans BSc, J Van den Eynde BSc), Katholieke Universiteit Leuven, Leuven, Belgium; Privatpraxis, Heidelberg, Germany (S Panda-Jonas MD); Research Department (A Pandey MPH), Public Health Research Society Nepal, Kathmandu, Nepal; Division of Research and Development (S R Pandi-Perumal MSc), Lovely Professional University, Phagwara, India; Department of Science and Mathematics (Prof P Papadopoulou PhD), Deree-The American College of Greece, Athens, Greece; Department of Community Medicine (P P Parija MD), All India Institute of Medical Sciences, Jammu, India; Department of Epidemiology and Community Health (R R Parikh MD), Department of Surgery (J Rickard MD), University of Minnesota, Minneapolis, MN, USA; Department of Medical Sciences (R Passera PhD), University of Torino, Torino, Italy; Department of Imaging (R Passera PhD), AOU Città della Salute e della Scienza di Torino, Torino, Italy; College of Dental Medicine (Prof S Patil PhD), Roseman University of Health Sciences, South Jordan, UT, USA; 2nd Department of Internal

Medicine (D Patoulias PhD), European Interbalkan Medical Center, Thessaloniki, Greece; Department of Internal Medicine (V Patthipati MD), Advent Health, Palm Coast, FL, USA; Department of Hospital Medicine (V Patthipati MD), Sound Physicians, Palm Coast, FL, USA; Clinical Research Department (P Pedersini MSc), IRCCS Fondazione Don Carlo Gnocchi, Milan, Italy; Outpatient Department (M Peng MPH), Taihe Hospital, Shiyan, China; The First Clinical College (M Peng MPH), School of Public Health and Management (Y Yu MS), Hubei University of Medicine, Shiyan, China; Department of Neurology (U Pensato MD), Dermatology Unit (M Valenti MD), IRCCS Humanitas Research Hospital, Milan, Italy; International Institute for Educational Planning (IIEP) (Prof M F P Peres MD), Albert Einstein Hospital, São Paulo, Brazil; Mario Negri Institute for Pharmacological Research, Bergamo, Italy (N Perico MD, Prof G Remuzzi MD); Facultad de Medicina (F E Petermann-Rocha PhD), Universidad Diego Portales (Diego Portales University), Santiago, Chile; Department of Psychiatry (Prof M R Phillips MD), Columbia University, New York, NY, USA; National Centre for Disease Prevention and Health Promotion (D Pierannunzio PhD), National Institute of Health, Roma, Italy; Department of Pediatric Orthopedic Surgery (M Pigeolet MD), Hôpital Necker - Enfants Malades, Paris, France; International Center of Medical Sciences Research, Islamabad, Pakistan (Z Z Piracha PhD); Air and Climate Unit (E Pisoni PhD), European Commission, Ispra, Italy; Centre for Public Health (M Piyasena PhD), Queen's University Belfast, Belfast, UK; Directorate of Policy Analysis and Development (M Piyasena PhD), Ministry of Health Sri Lanka, Colombo, Sri Lanka; Department of Environmental Hygiene (D Plass DrPH), German Environment Agency, Dessau-Roßlau, Germany; Research School of Chemistry and Applied Biomedical Sciences (E Plotnikov PhD), Tomsk Polytechnic University, Tomsk, Russia; Mental Health Research Institute (E Plotnikov PhD), Tomsk National Research Medical Center of the Russian Academy of Sciences, Tomsk, Russia; Clinical Academic Department of Pediatrics (Prof D Poddighe PhD), University Medical Center (UMC), Astana, Kazakhstan; Department of Data Management and Analysis (R Poluru PhD), The INCLEN Trust International, New Delhi, India; Discipline of General Practice (Prof C D Pond PhD), University of Newcastle, Callaghan, NSW, Australia; Department of Internal Medicine (D S Popovic PhD), University of Novi Sad, Novi Sad, Serbia; Clinic for Endocrinology, Diabetes and Metabolic Disorders (D S Popovic PhD), Clinical Center of Vojvodina, Novi Sad, Serbia; Department of Public Health (F Porru MD), Erasmus University Medical Center, Rotterdam, Netherlands; Non-communicable Diseases Research Center (N Pourtaheri PhD), Bam University of Medical Sciences, Bam, Iran; Independent Consultant, San Diego, CA, USA (D Prabhu PhD); Centro de Investigaciones Clinicas (Clinical Research Center) (S I Prada PhD), Fundación Valle del Lili (Valle del Lili Foundation), Cali, Colombia; Humanities and Social Sciences (Prof J Pradhan PhD), National Institute of Technology Rourkela, Rourkela, India; Department of Clinical Research and Epidemiology (M Prasad MD), Institute of Liver and Biliary Sciences, New Delhi, India; Department of Biostatistics Epidemiology and Informatics (J Puvvula PhD), University of Pennsylvania, Philadelphia, PA, USA; Cihan University Sulaimaniya Research Center (N H Qasim PhD), Cihan University Sulaimaniya, Sulaimaniya, Iraq; Department of Cardiology (G Qian MS), Third Military Medical University, Chongqing, China; Cardiovascular Research Center (M Rabiee Rad MD), Isfahan Cariovascular Research Institute, Isfahan, Iran; College of Medicine (A Radfar MD), University of Central Florida, Orlando, FL, USA; Department of Medical Oncology (Prof V Radhakrishnan MD), Cancer Institute (WIA), Chennai, India; UO Neurologia, Salute Pubblica e Disabilità (A Raggi PhD), Fondazione IRCCS Istituto Neurologico Carlo Besta (IRCCS Foundation Carlo Besta Neurological Institute), Milan, Italy; Pathology Department (N Raheem FMCPath), Mobiddo Adama University Teaching Hospital -Yola, Yola, Nigeria; Department of Health Sciences (Prof F Rahim PhD), Cihan University-Sulaymaniyah, Sulaymaniyah, Iraq; Cihan University Sulaimaniya Research Center (CUSRC), Sulaymaniyah, Iraq (Prof F

Rahim PhD); National Institute of Infectious Diseases (M Rahman PhD), Center for Surveillance, Immunization, and Epidemiologic Research, Tokyo, Japan; Center for Evidence-Based Medicine and Clinical Research, Dhaka, Bangladesh (M Rahman PhD); Institute of Health and Wellbeing (M Rahman PhD), Federation University Australia, Berwick, VIC, Australia; Future Technology Research Center (A Rahmani PhD), National Yunlin University of Science and Technology, Yunlin, Taiwan; Department of Nutrition (B Rahmani MSc), Qazvin University of Medical Sciences, Qazvin, Iran; Student Research Committee (N Rahmanian PhD), Student Research Committee, Rasht, Iran; Department of Public Health (V Rahmanian PhD), Torbat Jam Faculty of Medical Sciences, Torbat Jam, Iran; Department of Physical Education and Sport Sciences (Prof M Rahmati PhD), Lorestan University, Khoramabad, Iran; Department of Nutrition Science (S Rahmawaty PhD), Muhammadiyah University of Surakarta, Surakarta, Indonesia; Division of Gynecology and Obstetrics (D Raimondo PhD), Occupational Health Unit (Prof F S Violante MD), Sant'Orsola Malpighi Hospital, Bologna, Italy; Department of Community Medicine (S Rajaa MD), Employees' State Insurance Model Hospital, Chennai, India; Centre for Chronic Disease Control, New Delhi, India (P Rajput PhD); Department of Community Medicine (P Ramasubramani MD), Mahatma Gandhi Medical College and Research Institute, Puducherry, India; Laboratory Department (Prof P W Ramteke PhD), Mure Memorial Hospital, Nagpur, India; Department of Molecular Biology & Genetic Engineering (Prof P W Ramteke PhD), RTM Nagpur University, Nagpur, India; Research and Innovation Division (J Rana MPH), South Asian Institute for Social Transformation (SAIST), Dhaka, Bangladesh; Research Department (C L Ranabhat PhD), Policy Research Institute, Kathmandu, Nepal; Health and Public Policy Department (C L Ranabhat PhD), Global Center for Research and Development, Kathmandu, Nepal; Health Economics and Outcomes Research Department (A Rane MS), Agios Pharmaceuticals, Cambridge, MA, USA; Department of Pharmaceutical Economics and Policy (A Rane MS), Massachusetts College of Pharmacy and Health Sciences, Boston, MA, USA; Department of Medicine (Prof A Ranta PhD), University of Otago, Wellington, New Zealand; Department of Neurology (Prof A Ranta PhD), Capital & Coast District Health Board, Wellington, New Zealand; Department of Oral Pathology (S Rao MDS), Sharavathi Dental College and Hospital, Shimogga, India; University of Social Welfare and Rehabilitation Sciences, Tehran, Iran (V Rashedi PhD); Department of Clinical Science (M Rashidi DVM), Islamic Azad University, Garmsar, Iran; Department of Biomedical Engineering (Z Ratan MSc), Khulna University of Engineering and Technology, Khulna, Bangladesh; Inovus Medical, St Helens, UK (D L Rawaf MRCS); Academic Public Health England (Prof S Rawaf MD), Public Health England, London, UK; National Data Management and Analytics Center (Z F F Reda MPH), Ethiopian Public Health Institute, Addis Ababa, Ethiopia; Department of Biological Sciences (Prof E M M Redwan PhD), King Abdulaziz University, Jeddah, Egypt; Department of Protein Research (Prof E M M Redwan PhD), Research and Academic Institution, Alexandria, Egypt; Brien Holden Vision Institute, Sydney, NSW, Australia (Prof S Resnikoff MD); Department of Obstetrics and Gynecology (S Restaino MD), Azienda Sanitaria Universitaria Friuli Centrale, Udine, Italy; Unisabana Center for Translational Science (L F Reyes PhD), Universidad de La Sabana (Savannah University), Chia, Colombia; Critical Care Department (L F Reyes PhD), Clinica Universidad De La Sabana (Savannah University Clinic), Chia, Colombia; Department of Public Health Sciences (T G Rhee PhD), University of Connecticut, Farmington, CT, USA; Department of Surgery (J Rickard MD), University Teaching Hospital of Kigali, Kigali, Rwanda; One Health Toxicology Research Unit (1H-TOXRUN) (Prof C F Rodrigues PhD), Instituto Universitário de Ciências da Saúde (CESPU), Paredes, Portugal; Departamento de Farmacologia y Toxicologia (Prof J A B Rodriguez PhD), Universidad de Antioquia (University of Antioquia), Medellin, Colombia; Department of Clinical Research (L Roever PhD), Federal University of Uberlândia, Uberlândia, Brazil; Gilbert and Rose-Marie Chagoury

School of Medicine (L Roever PhD), Lebanese American University, Beirut, Lebanon; Faculty of Nursing (D S Romadlon PhD), Chulalongkorn University, Bangkok, Thailand; Golestan Research Center of Gastroenterology and Hepatology (G Roshandel PhD), Golestan University of Medical Sciences, Gorgan, Iran; School of Medicine (M Rostamian PhD), Gonabad University of Medical Sciences, Gonabad, Iran; Technical Department (K Rotimi MSc), Malaria Consortium, Abuja, Nigeria; Department of Public Health Pharmacy (K Rotimi MSc), West African Postgraduate College of Pharmacists, Lagos, Nigeria; Faculty of Medicine (B Roy PhD), Quest International University Perak, Ipoh, Malaysia; Centro de Investigación Palmira (Palmira Research Center) (E Rubagotti PhD), Corporación Colombiana de Investigación Agropecuaria AGROSAVIA (Colombian Agricultural Research Corporation), Bogota, Colombia; Advanced Campus Governador Valadares (Prof G d Ruela MSc), Juiz de For a Federal University, Governador Valadares, Brazil; Nursing Department (Prof G d Ruela MSc), Universidade Presidente Antônio Carlos (President Antônio Carlos University), Governador Valadares, Brazil; Department of Health Statistics (S F Rumisha PhD), National Institute for Medical Research, Dar es Salaam, Tanzania; Department of Cardiology (M Russo PhD), S Maria dei Battuti Hospital, Conegliano, Italy; Department of Civil Engineering (S W Ruzzante MSc), University of Victoria, Victoria, BC, Canada; Department of Medical Pharmacology (M M Saber-Ayad MD), Public Health and Community Medicine Department (M R Salem MD), Cairo University, Giza, Egypt; Neuropsychiatric Institute (Prof P S Sachdev MD), Prince of Wales Hospital, Randwick, NSW, Australia; Department of Cardiology (R Sachdeva MD), Department of Veterans Affairs, Decatur, GA, USA; Department of Cardiology (R Sachdeva MD), Medical College of Georgia at Augusta University, Augusta, GA, USA; Department of Pharmaceutical Chemistry (Prof M R Saeb PhD), Medical University of Gdańsk, Gdańsk, Poland; Multidisciplinary Laboratory (Prof U Saeed PhD), Foundation University, Islamabad, Pakistan; International Center of Medical Sciences Research (ICMSR), Islamabad, Pakistan (Prof U Saeed PhD); Faculty of Medicine, Bioscience and Nursing (S Z Safi PhD), MAHSA University, Selangor, Malaysia; Interdisciplinary Research Centre in Biomedical Materials (IRCBM) (S Z Safi PhD), COMSATS Institute of Information Technology, Lahore, Pakistan; Department of Community Medicine and Family Medicine (S S Sahoo MD, M Verma MD), Department of Radiodiagnosis (P Singh MD), All India Institute of Medical Sciences, Bathinda, India; Department of Preventive & Social Medicine (M Sahu MD), All India Institute of Hygiene & Public Health, Kolkata, India; Department of Statistics (M R Sajid PhD), University of Gujrat, Gujrat, Pakistan; Institute for Employment Research, Nuremberg, Germany (J W Sakshaug PhD); Department of Health Education & Promotion (Prof L Salehi PhD), ACS Medical College and Hospital, Karaj, Iran; Research Center for Health, Safety and Environment (Prof L Salehi PhD), School of Medicine (M Shams-Beyranvand MSc), Alborz University of Medical Sciences, Karaj, Iran; Technology Management Department (Prof M Z Y Salem PhD), University College of Applied Sciences, Gaza, Palestine; School of Economics and Management (Prof M Z Y Salem PhD), University of Kassel, Kassel, Germany; College of Nursing (D Salihu PhD), Jouf University, Jouf University, Saudi Arabia; Global Initiatives (Prof G A Salum PhD), Child Mind Institute, New York, NY, USA; Department of Neurology (S Samadzadeh MD), Department of Sports Science and Clinical Biomechanics (Prof S T Skou PhD), University of Southern Denmark, Odense, Denmark; Department of Anatomy (Prof V P Samuel PhD), Ras Al Khaimah Medical and Health Sciences University, Ras Al Khaimah, United Arab Emirates; Department of Surgery (Prof J Sanabria MD), Marshall University, Huntington, WV, USA; Department of Pediatrics (R K Sanjeev MD), Pravara Institute of Medical Sciences, Loni, India; Policy and Epidemiology Group (D F Santomauro PhD), Queensland Centre for Mental Health Research, Wacol, QLD, Australia; Pharmacy Study Program (M A Sarasmita PharmD), Udayana University, Badung, Indonesia; Independent Consultant, Thiruvananthapuram, India (S Y Saraswathy PhD); Indira Gandhi

Medical College and Research Institute, Puducherry, India (A Saravanan MD); Department of Orthopaedics and Trauma Surgery (B Saravi PhD), University of Freiburg, Freiburg, Germany; Department of Orthopaedics (B Saravi PhD), Loretto Hospital Freiburg, Freiburg, Germany; Department of Health and Society (Prof R Sarmiento-Suárez MPH), University of Applied and Environmental Sciences, Bogota, Colombia; Faculty of Health & Social Sciences (B Sathian PhD), Bournemouth University, Bournemouth, UK; IRCCS Istituti Clinici Scientifici Maugeri (IRCCS Maugeri Scientific Clinical Institute), Milan, Italy (D Sattin PsyD); Department of Public Health Sciences (M Sawhney PhD), University of North Carolina at Charlotte, Charlotte, NC, USA; Department of Preventive and Social Medicine (G Saya MD), Jawaharlal Institute of Postgraduate Medical Education and Research, Puducherry, India; National Centre for Epidemiology and Population Health (M Sayeed MS), Australian National University, Acton, ACT, Australia; Faculty of Business and Computing (Prof C Schinckus PhD), University of the Fraser Valley, Abbotsford, BC, Canada; Department of Finance (Prof C Schinckus PhD), International School of Management, Paris, France; Cardiovascular Program (X Xu PhD), The George Institute for Global Health, Sydney, NSW, Australia (Prof A E Schutte PhD, Prof J Sundström PhD); Department of Methodology and Innovation in Prevention (M Schwarzinger MD), University Hospital of Bordeaux, France, Bordeaux, France; University of Bordeaux (M Schwarzinger MD), The National Institute of Health and Medical Research (Inserm), Bordeaux, France; Clinic for Conservative Dentistry and Periodontology (Prof F Schwendicke PhD), University Hospital of the Ludwig-Maximilians-University Munich, Munich, Germany; Emergency Department (S Senthilkumaran MD), Manian Medical Centre, Erode, India; Department of Environmental Sciences and Engineering (M L Serre PhD), University of North Carolina Chapel Hill, Chapel Hill, NC, USA; Department of Medicine and Surgery (Y Sethi MBBS), Government Doon Medical College, Dehradun, India; Department of Medicine (N S Shah MD), Northwestern University, Chicago, IL, USA; HepatoPancreatoBiliary Surgery and Liver Transplant Department (P A Shah MBBS), Healthcare Global Limited Cancer Care Hospital, Bangalore, India; Public Health Division (A A Shaheen PhD), Department of Clinical and Community Pharmacy (Prof S H Zyoud PhD), Clinical Research Centre (Prof S H Zyoud PhD), An-Najah National University, Nablus, Palestine; Department of Chemistry (H Shahsavari PhD), Institute for Advanced Studies in Basic Sciences (IASBS), Zanjan, Iran; Independent Consultant, Karachi, Pakistan (M A Shaikh MD); Department of Neuro-Physiotherapy (S Z Shaikh PhD), Independent Consultant, Thane, India; Department of Pathology and Laboratory Medicine (S Sham MD), Northwell Health, New York, NY, USA; Department of Pathobiology (M Shamshirgaran PhD), Shahid Bahonar University of Kerman, Kerman, Iran; Department of Clinical Review and Safety (S Sharfaei MD), Baim Institute for Clinical Research, Boston, MA, USA; Facultad de Medicina (Faculty of Medicine) (J Sharifi-Rad PhD), Universidad del Azuay (University of Azuay), Cuenca, Ecuador; Division of Microbiology and Biotechnology (R P Shastry PhD), Yenepoya Research Center, Mangalore, India; Department of Engineering (A Shavandi PhD), Free University of Brussels, Brussels, Belgium; Centre for Medical Informatics (Prof A Sheikh MD), Usher Institute (Prof C R Simpson PhD), College of Medicine and Veterinary Medicine (G Verras MD), University of Edinburgh, Edinburgh, UK; Psychology Department (J Shen PhD), University of Massachusetts Lowell, Boston, MA, USA; Department of Public Health (D Shiferaw MPH), Dambi Dollo University, Dembi Dollo, Ethiopia; National Institute of Infectious Diseases, Tokyo, Japan (M Shigematsu PhD); Finnish Institute of Occupational Health, Helsinki, Finland (R Shiri PhD); Department of Clinical Immunology and Hematology (V Shivarov PhD), Sofiamed University Hospital, Sofia, Bulgaria; Department of Genetics (V Shivarov PhD), Sofia University "St Kliment Ohridiski", Sofia, Bulgaria; Department of Public Health (R Shrestha MPH), Nepal Development Society, Pokhara, Nepal; National Institute of Psychology (K Shuja MS), Quaid-i-Azam University, Islamabad,

Pakistan; The Cooper Institute, Dallas, TX, USA (K Shuval PhD); Department of Medical Microbiology and Infectious Diseases (E E Siddig MD), Erasmus University, Rotterdam, Netherlands; Department of Physical Education (Prof D A S Silva PhD), Federal University of Santa Catarina, Florianópolis, Brazil; Center of Potential and Innovation of Natural Resources (Prof L M R Silva PhD), Polytechnic Institute of Guarda, Guarda, Portugal; Health Sciences Research Centre (Prof L M R Silva PhD), University of Beira Interior, Covilhã, Portugal; Department of Nursing in Women's Health (T R Silva PhD), Federal University of São Paulo, São Paulo, Brazil; School of Health (Prof C R Simpson PhD), Victoria University of Wellington, Wellington, New Zealand; Department of Dentistry (A Singh MD), All India Institute of Medical Sciences, Bhopal, India; School of Public Health & Zoonoses (B B Singh PhD), Guru Angad Dev Veterinary & Animal Sciences University, Ludhiana, India; Department of Biochemistry (B Singh PhD), Central University of Punbjab, Bathinda, India; Department of Community Medicine (G Singh MD), Lady Hardinge Medical College, New Delhi, India; Department of Pharmacology (H Singh DM Clinical Pharmacology), Government Medical College and Hospital, Chandigarh, India; Medicine Service (Prof J A Singh MD), US Department of Veterans Affairs (VA), Birmingham, AL, USA; Department of Internal Medicine (R Sinto MD), University of Indonesia, Jakarta Pusat, Indonesia; Department of Internal Medicine (R Sinto MD), Dr Cipto Mangunkusumo National Hospital, Jakarta Pusat, Indonesia; Department of Neurology (S Sivakumar MD), University of Massachusetts Medical School, Worcester, MA, USA; Department of Chemistry (S S Siwal PhD), Maharishi Markandeshwar (Deemed to be University), Mullana, India; Medical Statistics Department (N Skhvitaridze MBA), National Center for Disease Control and Public Health, Tbilisi, Georgia; School of Health Sciences (N Skhvitaridze MBA), The University of Georgia, Tbilisi, Georgia; Department of Physiotherapy and Occupational Therapy (Prof S T Skou PhD), Næstved-Slagelse-Ringsted Hospitals, Slagelse, Denmark; Division of Injury Prevention (Prof D A Sleet PhD), The Bizzell Group, Atlanta, GA, USA; Department of Systemic Pathology (R Solanki MD), Touro College of Osteopathic Medicine, Middletown, NY, USA; Department of Pathology (R Solanki MD), American University of the Caribbean School of Medicine, Cupecoy, Saint Martin; Department of Biochemistry (S Solanki MD), American University of Integrative Sciences, Bridgetown, Barbados; Department of Medicine (R Somayaji MD, Prof M Tonelli MD), Department of Oncology (L Yang PhD), University of Calgary, Calgary, AB, Canada; Centro de Investigación Biomédica en Red Enfermedades Respiratorias (CIBERES) (Center for Biomedical Research in Respiratory Diseases Network), Madrid, Spain (Prof J B Soriano MD); Hull York Medical School (I N Soyiri PhD), University of Hull, Hull City, UK; Division of Community Medicine (C T Sreeramareddy MD), International Medical University, Kuala Lumpur, Malaysia; Public Health Department (M Stanikzai MPH), Kandahar University, Kandahar, Afghanistan; Department of Dermatology (S Steinke MD), University Hospital Muenster, Münster, Germany; Department of Medicine (P Steiropoulos MD), Democritus University of Thrace, Alexandroupolis, Greece; Occupational and Environmental Medicine Department (L Stockfelt PhD), Institute of Neuroscience and Physiology (Prof K S Sunnerhagen PhD), University of Gothenburg, Gothenburg, Sweden; Schiller Institute (Prof K Straif PhD), Boston College, Boston, MA, USA; Barcelona Institute for Global Health, Barcelona, Spain (Prof K Straif PhD); Department of Epidemiology & Biostatistics (Prof S Stranges MD), The University of Western Ontario, London, ON, Canada; Department of Population Health (Prof S Stranges MD), Luxembourg Institute of Health, Strassen, Luxembourg; School of Life Sciences (M Suleman PhD), Xiamen University, China, Xiamen, China; National Institute of Epidemiology (R Suliankatchi Abdulkader MD), Indian Council of Medical Research, Chennai, India; Department of Neurocare (Prof K S Sunnerhagen PhD), Sabzevar University of Medical Sciences, Gothenburg, Sweden; Department of Clinical Outcomes (Prof L Szarpak PhD), Maria Sklodowska-Curie

Medical Academy, Warsaw, Poland; Department of Clinical Research and Development (Prof L Szarpak PhD), LUXMED Group, Warsaw, Poland; Department of Dermatology (M D Szeto BS), University of Colorado, Aurora, CO, USA; Department of Neurology (P Tabaee Damavandi MD), Neurocenter of Southern Switzerland (NSI), Lugano, Switzerland; Department of Medicine (Prof R Tabarés-Seisdedos PhD), University of Valencia, Valencia, Spain; Carlos III Health Institute (Prof R Tabarés-Seisdedos PhD), Biomedical Research Networking Center for Mental Health Network (CiberSAM), Madrid, Spain; Department of Basic Medical Sciences (S Tabatabaeizadeh PhD), Department of Internal Medicine (S Tabatabaeizadeh PhD), Islamic Azad University, Mashhad, Iran; School of Dentistry and Oral Health (S K Tadakamadla PhD), School of Medicine and Dentistry (M N Wanjau MA), Griffith University, Gold Coast, QLD, Australia; Living Systems Institute (Y Taheri Abkenar PharmD), Department of Health and Community Sciences (A Udoh PhD), University of Exeter, Exeter, UK; Department of Biostatistics and Epidemiology (M Taheri Soodejani PhD), Shahid Sadoughi University of Medical Sciences, Yazd, Iran; Research Center for Molecular Medicine (A Taherkhani PhD), Hamadan University of Medical Sciences, Hamadan, Iran; Department of Environmental, Agricultural and Occupational Health (J Taiba MPH), University of Nebraska Medical Center, Omaha, NE, USA; University of Occupational and Environmental Health, Kitakyushu, Japan (Prof K Takahashi PhD); Department of Medicine (J L Tamuzi MSc), Northlands Medical Group, Omuthiya, Namibia; State Key Laboratory of Numerical Modeling for Atmospheric Sciences and Geophysical Fluid Dynamics (LASG) (H Tang PhD), Chinese Academy of Sciences, Beijing, China; Department of Economics (N Y Tat MS), Rice University, Houston, TX, USA; Department of Research and Innovation (N Y Tat MS), Enventure Medical Innovation, Houston, TX, USA; University Institute "Egas Moniz", Monte da Caparica, Portugal (Prof N Taveira PhD); Research Institute for Medicines (Prof N Taveira PhD), Universidade de Lisboa (University of Lisbon), Lisbon, Portugal; Outpatient Department (D R Terefa MSc), Wollega University, Bedele town, Ethiopia; Department of Psychology (E Teye-Kwadjo PhD), University of Ghana, Legon, Ghana; School of Humanities and Social Sciences (R Thakur PhD), Indian Institute of Technology Mandi, Mandi, India; Department of Pharmacology (P Thangaraju MD), All India Institute of Medical Sciences, Raipur, India; Public Health Department (Prof K R Thankappan MD), Amrita Institute of Medical Sciences, Kochi, India; Department of Gastroenterology (N K Thomas MD), PSG Institute of Medical Sciences and Research, Coimbatore, India; Faculty of Biomedical Engineering (A Tichopad PhD), Czech Technical University, Prague, Czech Republic; Faculty of Public Health (J H V Ticoalu MPH), Universitas Sam Ratulangi (Sam Ratulangi University), Manado, Indonesia; Office of Global Relations (Prof R Tobe-Gai PhD), Nagasaki University, Nagasaki, Japan; Institute of Public Health (R Topor-Madry PhD), Jagiellonian University Medical College, Kraków, Poland; Agency for Health Technology Assessment and Tariff System, Warsaw, Poland (R Topor-Madry PhD); Nutritional Epidemiology Research Team (EREN) (M Touvier PhD), National Institute for Health and Medical Research (INSERM), Paris, France; SRM College of Pharmacy (M R Tovani-Palone PhD), SRM Institute of Science and Technology (SRMIST), Chennai, India; High Institute of Sport and Physical Education of Sfax (K Trabelsi PhD), University of Sfax, Sfax, Tunisia; Health Informatics Department (M T N Tran PhD), Hanoi Medical University, Ha Noi, Viet Nam; Department of Health (N M Tran MD), Children's Hospital 1, Ho Chi Minh City, Viet Nam; Department of Surgical, Medical, Molecular Pathology and Critical Care Medicine (D Trico MD), University of Pisa, Pisa, Italy; Adult Learning Disability Service (S J Tromans PhD), Leicestershire Partnership National Health Service Trust, Leicester, UK; School of Medicine (T T Truyen MD), Nam Can Tho University, Can Tho, Viet Nam; Department of Psychiatry (E Tsermpini PhD), Dalhousie University, Halifax, NS, Canada; International Center for Chemical and Biological Sciences (S Ullah MSc), University of Karachi, Karachi, Pakistan; Department of Zoology (S

Ullah PhD), Division of Science and Technology (S Ullah PhD), University of Education, Lahore, Lahore, Pakistan; Department of Cardiovascular, Endocrine-metabolic Diseases and Aging (B Unim PhD), National Institute of Health, Rome, Italy; Amity Institute of Biotechnology (E Upadhyay PhD), Amity University Rajasthan, Jaipur, India; Center for Neurodegenerative Diseases and the Aging Brain (D Urso MD), University of Bari, Tricase, Italy; Department of Biomedical Sciences (M Valenti MD), Humanitas University, Milan, Italy; Urmia University of Medical Sciences, Urmia, Iran (R Valizadeh PhD); Department of Public Health and Epidemiology (O Varga PhD), University of Debrecen, Debrecen, Hungary; UKK Institute, Tampere, Finland (Prof T J Vasankari MD); Faculty of Medicine and Health Technology (Prof T J Vasankari MD), Tampere University, Tampere, Finland; Institute of Public Health of Serbia, Belgrade, Serbia (M Vasic PhD); Raffles Neuroscience Centre (Prof N Venketasubramanian MBBS), Raffles Hospital, Singapore, Singapore; Department of Physiotherapy (J H Villafañe PhD), Universidad Europea de Madrid (European University of Madrid), Villaviciosa de Odón, Spain; Center for Disease and Control Programs (Prof V E Villalobos-Daniel PhD), Ministry of Health, Mexico City, Mexico; Health Initiative of the Americas (Prof V E Villalobos-Daniel PhD), University of California Berkeley, Berkeley, CA, USA; Department Pediatric Hematology and Oncology (G I Villanueva MD), Hospital de Clinicas Jose de San Martin (Jose de San Martín Clinical Hospital), Buenos Aires, Argentina; Faculty of Information Technology (B Vo PhD), HUTECH University, Ho Chi Minh City, Viet Nam; Office of Research, Innovation, and Commercialization (ORIC) (Prof Y Waheed PhD), Shaheed Zulfiqar Ali Bhutto Medical University (SZABMU), Islamabad, Pakistan; Gilbert and Rose-Marie Chagoury School of Medicine (Prof Y Waheed PhD), Lebanese American University, Byblos, Lebanon; School of Public Health (F Wang PhD), Department of Epidemiology (D Yin DrPH), Xuzhou Medical University, Xuzhou, China; Department of Neurosurgery (S Wang MD), School of Public Health (J Xia PhD), Capital Medical University, Beijing, China; Department of Neurosurgery (S Wang MD), Beijing Tiantan Hospital, Beijing, China; Cardiology Department (Prof R G Weintraub MB), Royal Children's Hospital, Melbourne, VIC, Australia; Key Laboratory of Shaanxi Province for Craniofacial Precision Medicine Research (Y Wen PhD), Stomatological Hospital (College) of Xi'an Jiaotong University, Xi'an, China; Department of Physical Therapy (T Wiangkham PhD), Naresuan University, Phitsanulok, Thailand; Department of Medical Statistics, Informatics and Health Economics (Prof P Willeit PhD), Medical University Innsbruck, Innsbruck, Austria; Global Health Research Center (C Wu PhD), Duke Kunshan University, Kunshan, China; Department of Rheumatology and Immunology (D Wu PhD), Sichuan Provincial People's Hospital, Chengdu, China; Department of Food Science and Human Nutrition (Prof F Wu PhD), Michigan State University, East Lansing, MI, USA; Division of Gastroenterology (Prof Z Wu PhD), Huazhong University of Science and Technology, Wuhan, China; School of Public Health (H Xiao PhD), Zhejiang University, Zhejiang, China; Department of Public Health Science (H Xiao PhD), Fred Hutchinson Cancer Research Center, Seattle, WA, USA; Department of Endocrinology (Prof S Xu PhD), University of Science and Technology of China, Hefei, China; Department of Cancer Epidemiology and Prevention Research (L Yang PhD), Alberta Health Services, Calgary, AB, Canada; Faculty of Medicine (Y Yano MD), Department of Public Health (Prof N Yonemoto PhD), Juntendo University, Tokyo, Japan; Department of Family Medicine (S A Yesuf MSc), St Peter's Specialized Hospital, Addis Ababa, Ethiopia; Independent Consultant, Addis Ababa, Ethiopia (S A Yesuf MSc); Biostatics, Epidemiology, and Science Computing Department (S Yezli PhD), King Faisal Specialist Hospital & Research Center, Riyadh, Saudi Arabia; KHANA Center for Population Health Research, Phnom Penh, Cambodia (S Yi PhD); Department of Health Management (A Yiğit PhD), Süleyman Demirel Üniversitesi (Süleyman Demirel University), Isparta, Turkiye; Department of Biostatistics (Prof N Yonemoto PhD), University of Toyama School of Medicine,

Toyama, Japan; Department of Health Policy and Management (Prof M Z Younis PhD), Jackson State University, Jackson, MS, USA; School of Business & Economics (Prof M Z Younis PhD), Universiti Putra Malaysia (University of Putra Malaysia), Kuala Lumpur, Malaysia; Association for Socially Applicable Research (ASAR), Pune, India (S Zadey MS); Department of Emergency Medicine (S Zadey MS), Global Emergency Medicine Innovation and Implementation (GEMINI) Research Center, Durham, NC, USA; Epidemiology and Cancer Registry Sector (Prof V Zadnik PhD), Institute of Oncology Ljubljana, Ljubljana, Slovenia; Faculty of Medicine and Health Sciences (F Zakham PhD), Hodeidah University, Hodeidah, Yemen; Department of Health Sciences (S Zaman MSc), James Madison University, Harrisonburg, VA, USA; Sant'Elia Hospital (A Zanghì MD), University of Catania, Caltanissetta, Italy; Unit on Child & Adolescent Health (Prof H J Zar PhD), Medical Research Council South Africa, Cape Town, South Africa; Research and Development Department (I Zare BSc), Sina Medical Biochemistry Technologies, Shiraz, Iran; Addictology Department (Prof M S Zastrozhin PhD), Russian Medical Academy of Continuous Professional Education, Moscow, Russia; Department of Anesthesiology (Y Zeng MD), Third Xiangya Hospital of Central South University, Changsha, China; School of Health and Biomedical Sciences (A L Zhang PhD), Royal Melbourne Institute of Technology University, Melbourne, VIC, Australia; Medical Oncology Department of Gastrointestinal Cancer (L Zhang MS), Cancer Hospital of Dalian University of Technology, Shenyang, China; School of Biomedical Engineering (L Zhang MS), Dalian University of Technology, Dalian, China; School of Public Health (Y Zhang PhD), Hubei Province Key Laboratory of Occupational Hazard Identification and Control (Y Zhang PhD), Wuhan University of Science and Technology, Wuhan, China; College of Traditional Chinese Medicine (H Zhao MD), Hebei University, Baoding, China; School of Biology and Pharmaceutical Engineering (X G Zhao PhD), Wuhan Polytechnic University, Wuhan, China; Department of Basic Medicine (Y Zhao BS), Army Medical University, Chongqing, China; School of Public Health and Management (Prof Y Zhao MSc), Chongqing Medical University, Chongqing, China; School of Public Health and Management (J Zhou MD), Guangzhou University of Chinese Medicine, Guangzhou, China; Computational Bioscience Research Center (J Zhou PhD), King Abdullah University of Science and Technology, Jeddah, Saudi Arabia; School of Humanities and Management (Prof S Zhou PhD), Zhejiang Chinese Medical University, Hangzhou, China; School of Public Health and Emergency Management (B Zhu PhD), Southern University of Science and Technology, Shenzhen, China; School of Life Sciences (L Zhu PhD), Yunnan University, Kunming, China; Endocrinology and Metabolism Research Center (G Zoghi MD), Hormozgan University of Medical Sciences, Bandar Abbas, Iran; NIHR-Biomedical Research Centre (NIHR-BRC) (Prof A Zumla PhD), University College London Hospitals, London, UK; School of Physics (S H Zyoud PhD), Universiti Sains Malaysia (University of Science Malaysia), Penang, Malaysia

Authors' Contributions

Managing the overall research enterprise

Charlie Ashbaugh, Michael Brauer, Simon I Hay, Christopher J L Murray, Puja C Rao, and Gregory A Roth

Writing the first draft of the manuscript

Aleksandr Y Aravkin, Charlie Ashbaugh, Catherine Bisignano, Michael Brauer, Susan A McLaughlin, Samuel M Ostroff, and Puja C Rao.

Primary responsibility for applying analytical methods to produce estimates

Ashkan Afshin, Aleksandr Y Aravkin, Charlie Ashbaugh, Jessica Devin Bishai, Michael Brauer, Katrin G Burkart, Jack Cagney, Rebecca M Cogen, Garland T Culbreth, Xiaochen Dai, Kara Estep, Alize J Ferrari, Luisa S Flor, Emmanuela Gakidou, Gabriela Fernanda Gil, Hailey Hagins, Demewoz Haile, Austin Heuer, Catherine O Johnson, Nicholas J Kassebaum, Kate E LeGrand, Haley Lescinsky, Stephen L Lim, Elizabeth Marsh, Erin C Mullany, Sneha Ingle Nicholson, Erin M O'Connell, Kanyin Liane Ong, Marie C Parent, Quinn Rafferty, Puja C Rao, Christian Razo, Gregory A Roth, Damian Francesco Santomauro, Sandra Spearman, Cory N Spencer, Lauryn K Stafford, Jeffrey D Stanaway, Theo Vos, Sarah Wozniak, Jeff T Zhao, and Peng Zheng.

Primary responsibility for seeking, cataloguing, extracting, or cleaning data; designing or coding figures and tables

Vanessa Garcia, Nadim Hashmeh, Johnathan M Hsu, Audrey L Ihler, and Sandra Spearman.

Providing data or critical feedback on data sources

Kalkidan Hassen Abate, Yohannes Habtegiorgis Abate, Cristiana Abbafati, Madineh Akram Abbasi, Samar Abd ElHafeez, Auwal Abdullahi, Mesfin Abebe, Aidin Abedi, Armita Abedi, Temesgen Lera Abiso, Richard Gyan Aboagye, Hassan Abolhassani, Girma Beressa Aboye, Lucas Guimarães Abreu, Salahdein Aburuz, Ahmed Abu-Zaid, Mesafint Molla Adane, Victor Adekanmbi, Rishan Adha, Qorinah Estiningtyas Sakilah Adnani, Leticia Akua Adzigbli, Muhammad Sohail Afzal, Saira Afzal, Antonella Agodi, Bright Opoku Ahinkorah, Noah Ahmad, Tauseef Ahmad, Ali Ahmed, Ayman Ahmed, Muktar Beshir Ahmed, Safoora Ahmed, Hossein Akbarialiabad, Sreelatha Akkala, Salah Al Awaidy, Fares Alahdab, Fahad Mashhour Alanezi, Turki M Alanzi, Almaza Albakri, Mohammad T AlBataineh, Robert W Aldridge, Mulubirhan Assefa Alemayohu, Khalid F Alhabib, Abid Ali, Amjad Ali, Beriwan Abdulqadir Ali, Liaqat Ali, Syed Shujait Shujait Ali, Sheikh Mohammad Alif, Syed Mohamed Aljunid, François Alla, Sami Almustanyir, Jaber S Alqahtani, Rami Hani Al-Rifai, Najim Z Alshahrani, Awais Altaf, Nelson Alvis-Guzman, Nelson J Alvis-Zakzuk, Hassan Alwafi, Hany Aly, Uchenna Anderson Amaechi, Hubert Amu, Ganiyu Adeniyi Amusa, Deanna Anderlini, Jason A Anderson, Pedro Prata Andrade, Catalina Liliana Andrei, Tudorel Andrei, Susan C Anenberg, Dhanalakshmi Angappan, Amir Anoushiravani, Davood Anvari, Saeid Anvari, Saleha Anwar, Razique Anwer, Ekenedilichukwu Emmanuel Anyabolo, Geminn Louis Carace Apostol, Jalal Arabloo, Benedetta Armocida, Johan Ärnlöv, Anton A Artamonov, Akram M Asbeutah, Akeza Awealom Asgedom, Tahira Ashraf, Seyyed Shamsadin Athari, Prince Atorkey, Alok Atreya, Marcel Ausloos, Beatriz Paulina Ayala Quintanilla, Jose L Ayuso-Mateos, Ahmed Y Azzam, Ashish D Badiye, Arvind Bagga, Soroush Baghdadi, Sara Bagherieh, Pegah Bahrami Taghanaki, Atif Amin Baig, Ovidiu Constantin Baltatu, Palash Chandra Banik, Aduragbemi Banke-Thomas, Martina Barchitta, Mainak Bardhan, Till Winfried Bärnighausen, Hiba Jawdat Barqawi, Amadou Barrow, Mohammad-Mahdi Bastan, Sanjay Basu, Nebiyou Simegnew Bayileyegn, Alehegn Bekele, Uzma Iqbal Belgaumi, Michelle L Bell, Olorunjuwon Omolaja Bello, Apostolos Beloukas, Salaheddine Bendak, Fiona B Bennitt, Eduardo Bernabe, Akshaya Srikanth Bhagavathula, Dinesh Bhandari, Sonu Bhaskar, Ajay Nagesh Bhat, Vivek Bhat, Gurjit Kaur Bhatti, Jasvinder Singh Bhatti, Zulfiqar A Bhutta, Boris Bikbov, Bijit Biswas, Virginia Bodolica, Aadam Olalekan Bodunrin, Srinivasa Rao Bolla, Archith Boloor, Berrak Bora Basara, Hamed Borhany, Souad Bouaoud, Soufiane Boufous, Rupert Bourne, Dejana Braithwaite, Luisa C Brant, Michael Brauer, Dana Bryazka, Lemma N Bulto, Danilo Buonsenso, Katrin Burkart, Nadeem Shafique Butt, Florentino Luciano Caetano dos Santos, Jack Cagney, Chao Cao, Sinclair Carr, Juan J Carrero, Joao Mauricio Castaldelli-Maia, Carlos A Castañeda-Orjuela, Ferrán Catalá-López, Christopher R Cederroth, Joshua Chadwick, Chiranjib

Chakraborty, Raymond N C Chan, Joht Singh Chandan, Rama Mohan Chandika, Catherine S Chen, Ching-Yu Cheng, Abdulaal Chitheer, William C S Cho, Bryan Chong, Hitesh Chopra, Rajiv Chowdhury, Dinh-Toi Chu, Eric Chung, Aaron J Cohen, Alyssa Columbus, Joao Conde, Paolo Angelo Cortesi, Michael H Criqui, Natália Cruz-Martins, Xiaochen Dai, Giovanni Damiani, Lucio D'Anna, Aso Mohammad Darwesh, Fernando Pio De la Hoz, Alejandro de la Torre-Luque, Louisa Degenhardt, Cristian Del Bo', Chalachew Kassaw Demoze, Nikolaos Dervenis, Hardik Dineshbhai Desai, Rupak Desai, Vinoth Gnana Chellaiyan Devanbu, Arkadeep Dhali, Mandira Lamichhane Dhimal, Meghnath Dhimal, Sameer Dhingra, Vishal R Dhulipala, Diana Dias da Silva, Daniel Diaz, Michael J Diaz, Thanh Chi Do, Thao Huynh Phuong Do, Masoud Dodangeh, Klara Georgieva Dokova, Fariba Dorostkar, Rajkumar Doshi, Leila Doshmangir, Robert Kokou Dowou, Tim Robert Driscoll, Haneil Larson Dsouza, Bruce B Duncan, Andre Rodrigues Duraes, Senbagam Duraisamy, Anar Dushpanova, Paulina Agnieszka Dzianach, David Edvardsson, Aziz Eftekharimehrabad, Ebrahim Eini, Michael Ekholuenetale, Temitope Cyrus Ekundayo, Rabie Adel El Arab, Maysaa El Sayed Zaki, Faris El-Dahiyat, Mohammed Elshaer, Sayed Vahid Esmaeili, Adeniyi Francis Fagbamigbe, Omotayo Francis Fagbule, Saman Fahimi, Luca Falzone, Andre Faro, Ali Fatehizadeh, Valery L Feigin, Seyed-Mohammad Fereshtehnejad, Alize J Ferrari, Ida Fitriana, Carsten Flohr, David Flood, Luisa S Flor, Morenike Oluwatoyin Folayan, Blima Fux, Sridevi G, Peter Andras Gaal, Santosh Gaihre, Emmanuela Gakidou, Yaseen Galali, Silvano Gallus, Balasankar Ganesan, William M Gardner, Tilaye Gebru Gebi, Tesfay B B Gebremariam, Teferi Gebru Gebremeskel, Lemma Getacher, Fariba Ghassemi, Gabriela Fernanda Gil, Alem Girmay, Laszlo Göbölös, Myron Anthony Godinho, Mahaveer Golechha, Pouya Goleij, Nelson G M Gomes, Sameer Vali Gopalani, Alessandra C Goulart, Giuseppe Grosso, Shi-Yang Guan, Avirup Guha, Anish Kumar Gupta, Rajat Das Gupta, Rajeev Gupta, Sapna Gupta, Vijai Kumar Gupta, Nils Haep, Dariush Haghmorad, Demewoz Haile, Alemayehu Hailu, Adel Hajj Ali, Aram Halimi, Brian J Hall, Rabih Halwani, Nadia M Hamdy, Harapan Harapan, Josep Maria Haro, Mohammad Hasanian, Mahgol Sadat Hassan Zadeh Tabatabaei, Shokoufeh Hassani, Soheil Hassanipour, Hadi Hassankhani, Johannes Haubold, Rasmus J Havmoeller, Simon I Hay, Jeffrey J Hebert, Mehdi Hemmati, Claire A Henson, Molly E Herbert, Claudiu Herteliu, Nguyen Quoc Hoan, Julia Hon, Nobuyuki Horita, Mehdi Hosseinzadeh, Chengxi Hu, Md Nazmul Huda, Erin N Hulland, Michael Hultström, Nawfal R Hussein, Hong-Han Huynh, Segun Emmanuel Ibitoye, Nayu Ikeda, Kevin S Ikuta, Olayinka Stephen Ilesanmi, Lalu Muhammad Irham, Nahlah Elkudssiah Ismail, Gaetano Isola, Vinothini J, Jalil Jaafari, Kathryn H Jacobsen, Haitham Jahrami, Abhishek Jaiswal, Mihajlo Jakovljevic, Reza Jalilzadeh Yengejeh, Tahereh Javaheri, Shubha Jayaram, Bijay Mukesh Jeswani, Nabi Jomehzadeh, Jost B Jonas, Tamas Joo, Abel Joseph, Vivek Joshi, Charity Ehimwenma Joshua, Jacek Jerzy Jozwiak, Mikk Jürisson, Billingsley Kaambwa, Zubair Kabir, Vidya Kadashetti, Himal Kandel, Neeti Kapoor, André Karch, Faizan Zaffar Kashoo, Nicholas J Kassebaum, Norito Kawakami, Neda Kaydi, Gbenga A Kayode, Peter Njenga Keiyoro, Laura Kemmer, Yousef Saleh Khader, Morteza Abdullatif Khafaie, Himanshu Khajuria, Mariam Khalil, Faham Khamesipour, M Nuruzzaman Khan, Maseer Khan, Khaled Khatab, Mahalaqua Nazli Khatib, Atulya Aman Khosla, Min Seo Kim, Yun Jin Kim, Adnan Kisa, Sezer Kisa, Luke D Knibbs, Ann Kristin Skrindo Knudsen, Gerbrand Koren, Oleksii Korzh, Soewarta Kosen, Nikhil Kothari, Parvaiz A Koul, Sindhura Lakshmi Koulmane Laxminarayana, Kewal Krishan, Vijay Krishnamoorthy, Barthelemy Kuate Defo, Burcu Kucuk Bicer, Nuworza Kugbey, Mukhtar Kulimbet, Ashish Kumar, Vijay Kumar, Asep Kusnali, Dian Kusuma, Muhammad Awwal Ladan, Chandrakant Lahariya, Daphne Teck Ching Lai, Dharmesh Kumar Lal, Savita Lasrado, Huu-Hoai Le, Nhi Huu Hanh Le, Thao Thi Thu Le, Janet L Leasher, Caterina Ledda, Munjae Lee, Seung Won Lee, Shaun Wen Huey Lee, James Leigh, Yichong Li, Lee-Ling Lim, Stephen S Lim, Gang Liu, Jue Liu, Shiwei Liu, Wei Liu, Xuefeng Liu, Erand Llanaj, Rubén López-Bueno,

Stefan Lorkowski, Paulo A Lotufo, Rafael Lozano, Jailos Lubinda, Zheng Feei Ma, Kelsey Lynn Maass, Monika Machoy, Mohammed Magdy Abd El Razek, Azzam A Maghazachi, D R Mahadeshwara Prasad, Sandeep B Maharaj, Elham Mahmoudi, Azeem Majeed, Kashish Malhotra, Deborah Carvalho Malta, Borhan Mansouri, Mohammad Ali Mansournia, Lorenzo Giovanni Mantovani, Joemer C Maravilla, Francisco Rogerlândio Martins-Melo, Sharmeen Maryam, Alexander G Mathioudakis, Jishanth Mattumpuram, Andrea Maugeri, Michael A McPhail, Enkeleint A Mechili, Khalid Mehmood, Entezar Mehrabi Nasab, Toni Meier, Fabiola Mejia-Rodriguez, Tesfahun Mekene Meto, Ritesh G Menezes, Atte Meretoja, Bezawit Afework Mesfin, Sachith Mettananda, Tomasz Miazgowski, Irmina Maria Michalek, Ted R Miller, Erkin M Mirrakhimov, Vinaytosh Mishra, Chaitanya Mittal, Babak Moazen, Abdollah Mohammadian-Hafshejani, Mustapha Mohammed, Salahuddin Mohammed, Shafiu Mohammed, Ali H Mokdad, Lorenzo Monasta, Mohammad Ali Moni, Catrin E Moore, Maryam Moradi, Yousef Moradi, Jakub Morze, Rohith Motappa, Vincent Mougin, Ahmed Msherghi, Sumaira Mubarik, Ulrich Otto Mueller, Francesk Mulita, Efrén Murillo-Zamora, Christopher J L Murray, Ana-Maria Musina, Ghulam Mustafa, Saravanan Muthupandian, Raman Muthusamy, Ayoub Nafei, Ahamarshan Jayaraman Nagarajan, Shankar Prasad Nagaraju, Gabriele Nagel, Mohsen Naghavi, Ganesh R Naik, Sreenivas Narasimha Swamy, Bruno Ramos Nascimento, Zuhair S Natto, Biswa Prakash Nayak, Ionut Negoi, Ruxandra Irina Negoi, Josephine W Ngunjiri, Dang H Nguyen, Nhien Ngoc Y Nguyen, Phat Tuan Nguyen, Van Thanh Nguyen, Robina Khan Niazi, Sneha Ingle Nicholson, Jing Nie, Chukwudi A Nnaji, Shuhei Nomura, Bo Norrving, Jean Jacques Noubiap, Jerry John Nutor, Bogdan Oancea, Ismail A Odetokun, Akinkunmi Paul Okekunle, Osaretin Christabel Okonji, Andrew T Olagunju, Matthew Idowu Olatubi, Gláucia Maria Moraes Oliveira, Bolajoko Olubukunola Olusanya, Jacob Olusegun Olusanya, Yinka Doris Oluwafemi, Hany A Omar, Ahmed Omar Bali, Sokking Ong, Obinna E Onwujekwe, Heather M Orpana, Alberto Ortiz, Samuel M Ostroff, Adrian Otoiu, Stanislav S Otstavnov, Amel Ouyahia, Mayowa O Owolabi, Mahesh Padukudru P A, Kevin Pacheco-Barrios, Jagadish Rao Padubidri, Songhomitra Panda-Jonas, Paraskevi Papadopoulou, Romil R Parikh, Nicholas Parsons, Maja Pasovic, Shankargouda Patil, Shrikant Pawar, Paolo Pedersini, Veincent Christian Filipino Pepito, Emmanuel K Peprah, Prince Peprah, Mario F P Peres, Arokiasamy Perianayagam, Simone Perna, Konrad Pesudovs, Hoang Tran Pham, Anil K Philip, Daniela Pierannunzio, David M Pigott, Zahra Zahid Piracha, Dietrich Plass, Kevan R Polkinghorne, Ramesh Poluru, Maarten J Postma, Naeimeh Pourtaheri, Sergio I Prada, Jalandhar Pradhan, Elton Junio Sady Prates, Jagadeesh Puvvula, Asma Saleem Qazi, Raghu Anekal Radhakrishnan, Quinn Rafferty, Nasiru Raheem, Fakher Rahim, Md Jillur Rahim, Vafa Rahimi-Movaghar, Amir Masoud Rahmani, Bita Rahmani, Mohammad Rahmanian, Sathish Rajaa, Vinoth Rajendran, Shakthi Kumaran Ramasamy, Sheena Ramazanu, Juwel Rana, Chhabi Lal Ranabhat, Chythra R Rao, Mithun Rao, Sowmya J Rao, Sina Rashedi, Vahid Rashedi, Zubair Ahmed Ratan, Santosh Kumar Rauniyar, Salman Rawaf, Reza Rawassizadeh, Christian Razo, Lennart Reifels, Bhageerathy Reshmi, Maryam Rezaei, Taeho Gregory Rhee, Antonio Luiz P Ribeiro, Hannah Elizabeth Robinson-Oden, Mónica Rodrigues, Jefferson Antonio Buendia Rodriguez, Leonardo Roever, Debby Syahru Romadlon, Luca Ronfani, Jennifer Jacqueline Rosauer, Gregory A Roth, Kunle Rotimi, Himanshu Sekhar Rout, Enrico Rubagotti, Susan Fred Rumisha, Tilleye Runghien, Michele Russo, Aly M A Saad, Korosh Saber, Siamak Sabour, Perminder S Sachdev, Basema Saddik, Adam Saddler, Bashdar Abuzed Sadee, Ehsan Sadeghi, Elham Sadeghi Majd, Umar Saeed, Rajesh Sagar, Zahra Saif, Mirza Rizwan Sajid, Joseph W Sakshaug, Afeez Abolarinwa Salami, Marwa Rashad Salem, Hossein Samadi Kafil, Sara Samadzadeh, Vijaya Paul Samuel, Abdallah M Samy, Juan Sanabria, Francesca Sanna, Damian Francesco Santomauro, Milena M Santric-Milicevic, Babak Saravi, Brijesh Sathian, Monika Sawhney, Md Abu Sayeed, Maria Inês Schmidt, Aletta Elisabeth Schutte, David C Schwebel, Mohammad

H Semreen, Subramanian Senthilkumaran, Dragos Serban, Marc L Serre, Yashendra Sethi, Pritik A Shah, Masood Ali Shaikh, Summaiya Zareen Shaikh, Sunder Sham, Muhammad Aaqib Shamim, Mehran Shams-Beyranvand, Abhishek Shankar, Javad Sharifi-Rad, Vishal Sharma, Rajesh P Shastry, Somia Shehzadi, Jiabin Shen, Adithi Shetty, B Suresh Kumar Shetty, Aminu Shittu, K M Shivakumar, Sina Shool, Sunil Shrestha, Kanwar Hamza Shuja, Luís Manuel Lopes Rodrigues Silva, Abhinav Singh, Baljinder Singh, Jasvinder A Singh, Paramdeep Singh, Shravan Sivakumar, Natia Skhvitaridze, Sameh S M Soliman, Reed J D Sorensen, Ireneous N Soyiri, Michael Spartalis, Cory N Spencer, Chandrashekhar T Sreeramareddy, Jeffrey D Stanaway, Muhammad Haroon Stanikzai, Caroline Stein, Fridolin Steinbeis, Mark A Stokes, Muhammad Suleman, Rizwan Suliankatchi Abdulkader, Johan Sundström, Chandan Kumar Swain, Lukasz Szarpak, Mindy D Szeto, Payam Tabaee Damavandi, Rafael Tabarés-Seisdedos, Shima Tabatabai, Mohammad Tabish, Yasaman Taheri Abkenar, Moslem Taheri Soodejani, Amir Taherkhani, Ker-Kan Tan, Nuno Taveira, Yibekal Manaye Tefera, Pugazhenthan Thangaraju, Nihal Thomas, Nikhil Kenny Thomas, Ruoyan Tobe-Gai, Marcos Roberto Tovani-Palone, Khaled Trabelsi, Jasmine T Tran, Mai Thi Ngoc Tran, Domenico Trico, Indang Trihandini, Christopher E Troeger, Sana Ullah, Muhammad Umair, Srikanth Umakanthan, Bhaskaran Unnikrishnan, Era Upadhyay, Jibrin Sammani Usman, Jef Van den Eynde, Aaron van Donkelaar, Shoban Babu Varthya, Tommi Juhani Vasankari, Narayanaswamy Venketasubramanian, Nicholas Alexander Verghese, Georgios-Ioannis Verras, Victor E Villalobos-Daniel, Vasily Vlassov, Bay Vo, Simona Ruxandra Volovat, Theo Vos, Yasir Waheed, Shu Wang, Abdul Waris, Daniel J Weiss, Taweewat Wiangkham, Caroline Wilkerson, Peter Willeit, Yen Jun Wong, Utoomporn Wongsin, Felicia Wu, Juan Xia, Suowen Xu, Yvonne Yiru Xu, Kazumasa Yamagishi, Pengpeng Ye, Siyan Yi, Zeamanuel Anteneh Yigzaw, Dehui Yin, Dong Keon Yon, Naohiro Yonemoto, Mustafa Z Younis, Chuanhua Yu, Yong Yu, Siddhesh Zadey, Josefina Zakzuk, Ghazal G Z Zandieh, Iman Zare, Mikhail Sergeevich Zastrozhin, Chunxia Zhai, Magdalena Zielińska, Stephanie R M Zimsen, Thomas Zoller, and Alimuddin Zumla.

Developing methods or computational machinery

Cristiana Abbafati, Armita Abedi, Girma Beressa Aboye, Rufus Adesoji Adedoyin, Bashir Aden, Qorinah Estiningtyas Sakilah Adnani, Saira Afzal, Noah Ahmad, Muktar Beshir Ahmed, Safoora Ahmed, Hossein Akbarialiabad, Salah Al Awaidy, Najim Z Alshahrani, Walid Al-Zyoud, Hubert Amu, Susan C Anenberg, Davood Anvari, Sumadi Lukman Anwar, Aleksandr Y Aravkin, Michael Benjamin Arndt, Charlie Ashbaugh, Michael Assmus, Ahmed Y Azzam, Mohammad-Mahdi Bastan, Akshaya Srikanth Bhagavathula, Sonu Bhaskar, Aadam Olalekan Bodunrin, Milad Bonakdar Hashemi, Hamed Borhany, Souad Bouaoud, Rupert Bourne, Michael Brauer, Dana Bryazka, Katrin Burkart, Fan Cao, Sinclair Carr, Kelly M Cercy, Catherine S Chen, William C S Cho, Rebecca M Cogen, Aaron J Cohen, Jessica A Cruz, Garland T Culbreth, Xiaochen Dai, Aso Mohammad Darwesh, Louisa Degenhardt, Chalachew Kassaw Demoze, Hardik Dineshbhai Desai, Thanh Chi Do, Deepa Dongarwar, Robert Kokou Dowou, Paulina Agnieszka Dzianach, Michael Ekholuenetale, Maysaa El Sayed Zaki, Sayed Vahid Esmaeili, Adeniyi Francis Fagbamigbe, Ali Fatehizadeh, Alize J Ferrari, Carsten Flohr, Luisa S Flor, Sridevi G, William M Gardner, Gabriela Fernanda Gil, Nora M Gilbertson, Alem Girmay, Shi-Yang Guan, Demewoz Haile, Adel Hajj Ali, Mohammad Jahid Hasan, Simon I Hay, Mohammad Heidari, Mehdi Hosseinzadeh, Chantal K Huynh, Hong-Han Huynh, Lalu Muhammad Irham, Gaetano Isola, Vinothini J, Haitham Jahrami, Tahereh Javaheri, Bijay Mukesh Jeswani, Charity Ehimwenma Joshua, Daniel John Kapner, Faizan Zaffar Kashoo, Nicholas J Kassebaum, Peter Njenga Keiyoro, Mahalaqua Nazli Khatib, Adnan Kisa, Chandrakant Lahariya, Huu-Hoai Le, Nhi Huu Hanh Le, Thao Thi Thu Le, James Leigh, Haley Lescinsky, Erand Llanaj, Kelsey Lynn Maass, Mohammed Magdy Abd El Razek, Borhan Mansouri, Michael A McPhail, Madeline E Moberg, Salahuddin Mohammed, Shafiu Mohammed, Ali H Mokdad, Mohammad Ali Moni, Yousef Moradi, Francesk Mulita,

Christopher J L Murray, Ayoub Nafei, Mohsen Naghavi, Josephine W Ngunjiri, Phat Tuan Nguyen, Van Thanh Nguyen, Michal Ordak, Amel Ouyahia, Hoang Tran Pham, Brandon V Pickering, Zahra Zahid Piracha, Hadi Raeisi Shahraki, Amir Masoud Rahmani, Vinoth Rajendran, Giridhara Rathnaiah Babu, Reza Rawassizadeh, Christian Razo, Maryam Rezaei, Mónica Rodrigues, Jennifer Jacqueline Rosauer, Gregory A Roth, Himanshu Sekhar Rout, Enrico Rubagotti, Susan Fred Rumisha, Korosh Saber, Adam Saddler, Elham Sadeghi Majd, Umar Saeed, Abdallah M Samy, Francesca Sanna, Damian Francesco Santomauro, Ganesh Kumar Saya, Mehdi Sayyah, Austin E Schumacher, Yashendra Sethi, Yi Song, Reed J D Sorensen, Michael Spartalis, Sandra Spearman, Cory N Spencer, Jeffrey D Stanaway, Muhammad Haroon Stanikzai, Caitlyn Steiner, Muhammad Suleman, Chandan Kumar Swain, Yibekal Manaye Tefera, Roman Topor-Madry, Mai Thi Ngoc Tran, Christopher E Troeger, Muhammad Umair, Victor E Villalobos-Daniel, Bay Vo, Theo Vos, Daniel J Weiss, Eli J Weiss, Shadrach Wilson, Yen Jun Wong, Dong Keon Yon, Ghazal G Z Zandieh, Mikhail Sergeevich Zastrozhin, Chunxia Zhai, Meixin Zhang, Yang Zhao, and Peng Zheng.

Providing critical feedback on methods or results

Kalkidan Hassen Abate, Yohannes Habtegiorgis Abate, Cristiana Abbafati, Rouzbeh Abbasgholizadeh, Madineh Akram Abbasi, Mohammadreza Abbasian, Mitra Abbasifard, Samar Abd ElHafeez, Sherief Abd-Elsalam, Parsa Abdi, Mohammad Abdollahi, Meriem Abdoun, Deldar Morad Abdulah, Auwal Abdullahi, Mesfin Abebe, Aidin Abedi, Armita Abedi, Tadesse M Abegaz, Roberto Ariel Abeldaño Zuñiga, Temesgen Lera Abiso, Richard Gyan Aboagye, Hassan Abolhassani, Mohamed Abouzid, Girma Beressa Aboye, Lucas Guimarães Abreu, Hasan Abualruz, Bilyaminu Abubakar, Eman Abu-Gharbieh, Hana Jihad Jihad Abukhadijah, Salahdein Aburuz, Ahmed Abu-Zaid, Mesafint Molla Adane, Isaac Yeboah Addo, Rufus Adesoji Adedoyin, Victor Adekanmbi, Juliana Bunmi Adetunji, Temitayo Esther Adeyeoluwa, Rishan Adha, Amin Adibi, Qorinah Estiningtyas Sakilah Adnani, Leticia Akua Adzigbli, Aanuoluwapo Adeyimika Afolabi, Rotimi Felix Afolabi, Shadi Afyouni, Muhammad Sohail Afzal, Saira Afzal, Suneth Buddhika Agampodi, Faith Agbozo, Shahin Aghamiri, Antonella Agodi, Anurag Agrawal, Williams Agyemang-Duah, Bright Opoku Ahinkorah, Aqeel Ahmad, Danish Ahmad, Firdos Ahmad, Shahzaib Ahmad, Tauseef Ahmad, Ali Ahmed, Anisuddin Ahmed, Ayman Ahmed, Luai A Ahmed, Muktar Beshir Ahmed, Syed Anees Ahmed, Gizachew Taddesse Akalu, Essona Matatom Akara, Hossein Akbarialiabad, Karolina Akinosoglou, Tomi Akinyemiju, Sreelatha Akkala, Salah Al Awaidy, Syed Mahfuz Al Hasan, Fares Alahdab, Tareq Mohammed Ali AL-Ahdal, Samer O Alalalmeh, Ziyad Al-Aly, Khurshid Alam, Nazmul Alam, Fahad Mashhour Alanezi, Turki M Alanzi, Almaza Albakri, Mohammad T AlBataineh, Mulubirhan Assefa Alemayohu, Yihun Mulugeta Alemu, Bassam Al-Fatly, Adel Ali Saeed Al-Gheethi, Khairat Al-Habbal, Khalid F Alhabib, Robert Kaba Alhassan, Abid Ali, Iman Ali, Liaqat Ali, Mohammed Usman Ali, Rafat Ali, Syed Shujait Shujait Ali, Gianfranco Alicandro, Sheikh Mohammad Alif, Syed Mohamed Aljunid, Sabah Al-Marwani, Hesham M Al-Mekhlafi, Sami Almustanyir, Mahmoud A Alomari, Jaber S Alqahtani, Ahmed Yaseen Alqutaibi, Rajaa M Al-Raddadi, Rami Hani Al-Rifai, Sahel Majed Alrousan, Salman Khalifah Al-Sabah, Najim Z Alshahrani, Zaid Altaany, Awais Altaf, Jaffar A Al-Tawfiq, Khalid A Altirkawi, Nelson Alvis-Guzman, Nelson J Alvis-Zakzuk, Hassan Alwafi, Mohammad Sami Al-Wardat, Yaser Mohammed Al-Worafi, Hany Aly, Safwat Aly, Karem H Alzoubi, Walid Al-Zyoud, Reza Amani, Sohrab Amiri, Hubert Amu, Dickson A Amugsi, Ganiyu Adeniyi Amusa, Robert Ancuceanu, Deanna Anderlini, Pedro Prata Andrade, Catalina Liliana Andrei, Tudorel Andrei, Susan C Anenberg, Dhanalakshmi Angappan, Colin Angus, Afifa Anjum, Amir Anoushiravani, Ernoiz Antriyandarti, Boluwatife Stephen Anuoluwa, Davood Anvari, Saeid Anvari, Saleha Anwar, Sumadi Lukman Anwar, Razique Anwer, Ekenedilichukwu Emmanuel Anyabolo, Anayochukwu Edward Anyasodor, Geminn Louis Carace Apostol, Jalal Arabloo, Mosab Arafat, Aleksandr Y Aravkin, Demelash Areda, Brhane Berhe Aregawi, Benedetta Armocida, Mahwish Arooj, Anton A Artamonov,

Kurnia Dwi Artanti, Idowu Thomas Aruleba, Ashokan Arumugam, Akram M Asbeutah, Saeed Asgary, Akeza Awealom Asgedom, Charlie Ashbaugh, Mubarek Yesse Ashemo, Tahira Ashraf, Amir Askarinejad, Thomas Astell-Burt, Mohammad Athar, Seyyed Shamsadin Athari, Prince Atorkey, Alok Atreya, Avinash Aujayeb, Marcel Ausloos, Leticia Avila-Burgos, Andargie Abate Awoke, Beatriz Paulina Ayala Quintanilla, Haleh Ayatollahi, Sina Azadnajafabad, Rui M S Azevedo, Gulrez Shah Azhar, Hosein Azizi, Ahmed Y Azzam, Insa Linnea Backhaus, Muhammad Badar, Ashish D Badiye, Arvind Bagga, Nasser Bagheri, Sara Bagherieh, Pegah Bahrami Taghanaki, Ruhai Bai, Atif Amin Baig, Jennifer L Baker, Madhan Balasubramanian, Ovidiu Constantin Baltatu, Soham Bandyopadhyay, Biswajit Banik, Palash Chandra Banik, Aduragbemi Banke-Thomas, Hansi Bansal, Martina Barchitta, Mainak Bardhan, Suzanne Lyn Barker-Collo, Till Winfried Bärnighausen, Hiba Jawdat Barqawi, Lope H Barrero, Amadou Barrow, Sandra Barteit, Zarrin Basharat, João Diogo Basso, Mohammad-Mahdi Bastan, Sanjay Basu, Sai Batchu, Kavita Batra, Ravi Batra, Bernhard T Baune, Mohsen Bayati, Nebiyou Simegnew Bayileyegn, Thomas Beaney, Amir Hossein Behnoush, Alehegn Bekele, Uzma Iqbal Belgaumi, Arielle Wilder Bell, Michelle L Bell, Olorunjuwon Omolaja Bello, Luis Belo, Apostolos Beloukas, Derrick A Bennett, Isabela M Bensenor, Habib Benzian, Azizullah Beran, Zombor Berezvai, Eduardo Bernabe, Robert S Bernstein, Akshaya Srikanth Bhagavathula, Neeraj Bhala, Dinesh Bhandari, Nikha Bhardwaj, Pankaj Bhardwaj, Sonu Bhaskar, Ajay Nagesh Bhat, Vivek Bhat, Gurjit Kaur Bhatti, Jasvinder Singh Bhatti, Manpreet S Bhatti, Rajbir Bhatti, Mohiuddin Ahmed Bhuiyan, Zulfiqar A Bhutta, Boris Bikbov, Jessica Devin Bishai, Bijit Biswas, Raaj Kishore Biswas, Micheal Kofi Boachie, Moses John Bockarie, Virginia Bodolica, Aadam Olalekan Bodunrin, Eyob Ketema Bogale, Archith Boloor, Milad Bonakdar Hashemi, Sri Harsha Boppana, Hamed Borhany, Alejandro Botero Carvajal, Souad Bouaoud, Rupert Bourne, Christopher Boxe, Dejana Braithwaite, Luisa C Brant, Michael Brauer, Nicholas J K Breitborde, Hermann Brenner, Andrey Nikolaevich Briko, Gabrielle Britton, Annie J Browne, Andre R Brunoni, Dana Bryazka, Norma B Bulamu, Lemma N Bulto, Danilo Buonsenso, Katrin Burkart, Richard A Burns, Reinhard Busse, Yasser Bustanji, Nadeem Shafique Butt, Zahid A Butt, Florentino Luciano Caetano dos Santos, Jack Cagney, Lucero Cahuana-Hurtado, Luis Alberto Cámera, Ismael R Campos-Nonato, Chao Cao, Fan Cao, Yubin Cao, Angelo Capodici, Rosario Cárdenas, Sinclair Carr, Juan J Carrero, Márcia Carvalho, Joao Mauricio Castaldelli-Maia, Carlos A Castañeda-Orjuela, Ferrán Catalá-López, Alberico L Catapano, Christopher R Cederroth, Luca Cegolon, Muthia Cenderadewi, Ester Cerin, Joshua Chadwick, Chiranjib Chakraborty, Promit Ananyo Chakraborty, Raymond N C Chan, Joht Singh Chandan, Rama Mohan Chandika, An-Tian Chen, Haowei Chen, Meng Xuan Chen, Mingling Chen, Esther T W Cheng, Nicolas Cherbuin, Fatemeh Chichagi, Odgerel Chimed-Ochir, Ritesh Chimoriya, Jesus Lorenzo Chirinos-Caceres, William C S Cho, Bryan Chong, Hitesh Chopra, Rajiv Chowdhury, Devasahayam J Christopher, Dinh-Toi Chu, Isaac Sunday Chukwu, Eric Chung, Sheng-Chia Chung, Muhammad Chutiyami, Iolanda Cioffi, Aaron J Cohen, Alyssa Columbus, Joao Conde, Alexandru Corlateanu, Samuele Cortese, Paolo Angelo Cortesi, Vera Marisa Costa, Simona Costanzo, Michael H Criqui, Natália Cruz-Martins, Garland T Culbreth, Alanna Gomes da Silva, Omid Dadras, Xiaochen Dai, Patience Unekwuojo Daikwo, Lachlan L Dalli, Giovanni Damiani, Emanuele D'Amico, Lucio D'Anna, Aso Mohammad Darwesh, Jai K Das, Subasish Das, Nihar Ranjan Dash, Mohsen Dashti, Claudio Alberto Dávila-Cervantes, Alejandro de la Torre-Luque, Diego De Leo, Shayom Debopadhaya, Louisa Degenhardt, Cristian Del Bo', Ivan Delgado-Enciso, Chalachew Kassaw Demoze, Nikolaos Dervenis, Emina Dervišević, Hardik Dineshbhai Desai, Rupak Desai, Vinoth Gnana Chellaiyan Devanbu, Arkadeep Dhali, Kuldeep Dhama, Amol S Dhane, Mandira Lamichhane Dhimal, Meghnath Dhimal, Sameer Dhingra, Vishal R Dhulipala, Raja Ram Dhungana, Diana Dias da Silva, Daniel Diaz, Michael J Diaz, Adriana Dima, Delaney D Ding, Shirin Djalalinia, Thanh Chi Do, Thao Huynh Phuong Do,

Camila Bruneli do Prado, Masoud Dodangeh, Klara Georgieva Dokova, Deepa Dongarwar, Mario D'Oria, Fariba Dorostkar, E Ray Dorsey, Rajkumar Doshi, Leila Doshmangir, Robert Kokou Dowou, Tim Robert Driscoll, Haneil Larson Dsouza, Samuel C Dumith, Senbagam Duraisamy, Anar Dushpanova, Paulina Agnieszka Dzianach, Arkadiusz Marian Dziedzic, Alireza Ebrahimi, Chidiebere Peter Echieh, Abdelaziz Ed-Dra, Hisham Atan Edinur, David Edvardsson, Kristina Edvardsson, Aziz Eftekharimehrabad, Ebrahim Eini, Michael Ekholuenetale, Temitope Cyrus Ekundayo, Rabie Adel El Arab, Maysaa El Sayed Zaki, Faris El-Dahiyat, Noha Mousaad Elemam, Frank J Elgar, Ghada Metwally Tawfik ElGohary, Hala Rashad Elhabashy, Muhammed Elhadi, Ahmed O Elmehrath, Omar Abdelsadek Abdou Elmeligy, Mohammed Elshaer, Ibrahim Elsohaby, Theophilus I Emeto, Negin Esfandiari, Babak Eshrati, Majid Eslami, Sayed Vahid Esmaeili, Farshid Etaee, Natalia Fabin, Adeniyi Francis Fagbamigbe, Omotayo Francis Fagbule, Saman Fahimi, Luca Falzone, Mohammad Fareed, Carla Sofia e Sá Farinha, Andre Faro, Folorunso Oludayo Fasina, Ali Fatehizadeh, Timur Fazylov, Valery L Feigin, Xiaoqi Feng, Seyed-Mohammad Fereshtehnejad, Abdullah Hamid Feroze, Pietro Ferrara, Getahun Fetensa, Bikila Regassa Feyisa, Irina Filip, Florian Fischer, Ida Fitriana, Joanne Flavel, Carsten Flohr, David Flood, Luisa S Flor, Nataliya A Foigt, Morenike Oluwatoyin Folayan, Lisa M Force, Matteo Foschi, Richard Charles Franklin, Alberto Freitas, Sara D Friedman, Blima Fux, Sridevi G, Peter Andras Gaal, Santosh Gaihre, Márió Gajdács, Emmanuela Gakidou, Yaseen Galali, Aravind P Gandhi, Balasankar Ganesan, Mohammad Arfat Ganiyani, Vanessa Garcia, William M Gardner, Rupesh K Gautam, Tilaye Gebru Gebi, Miglas W Gebregergis, Mesfin Gebrehiwot, Tesfay B B Gebremariam, Teferi Gebru Gebremeskel, Urge Gerema, Lemma Getacher, Genanew K a Getahun, Molla Getie, Amir Ghaffari Jolfayi, Alireza Ghajar, MohammadReza Ghasemi, Ghazal Ghasempour Dabaghi, Afsaneh Ghasemzadeh, Fariba Ghassemi, Ramy Mohamed Ghazy, Ali Gholami, Ali Gholamrezanezhad, Artyom Urievich Gil, Gabriela Fernanda Gil, Paramjit Singh Gill, Tiffany K Gill, Ebisa Zerihun Gindaba, Alem Girmay, James C Glasbey, Elena V Gnedovskaya, Laszlo Göbölös, Myron Anthony Godinho, Mahaveer Golechha, Davide Golinelli, Nelson G M Gomes, Houman Goudarzi, Anmol Goyal, Simon Matthew Graham, Michal Grivna, Giuseppe Grosso, Shi-Yang Guan, Mohammed Ibrahim Mohialdeen Gubari, Avirup Guha, Stefano Guicciardi, Snigdha Gulati, David Gulisashvili, Damitha Asanga Gunawardane, Cui Guo, Anish Kumar Gupta, Bhawna Gupta, Rahul Gupta, Rajat Das Gupta, Sapna Gupta, Veer Bala Gupta, Vijai Kumar Gupta, Vivek Kumar Gupta, Farrokh Habibzadeh, Parham Habibzadeh, Tesfahun Simon Hadaro, Nils Haep, Hamed Haghi-Aminjan, Dariush Haghmorad, Demewoz Haile, Alemayehu Hailu, Adel Hajj Ali, Aram Halimi, Brian J Hall, Sebastian Haller, Rabih Halwani, Randah R Hamadeh, Nadia M Hamdy, Samer Hamidi, Ahmad Hammoud, Asif Hanif, Nasrin Hanifi, Zaim Anan Haq, Md Rabiul Haque, Harapan Harapan, Arief Hargono, Josep Maria Haro, Ahmed I Hasaballah, Ikramul Hasan, Mohammad Jahid Hasan, Hamidreza Hasani, Md Saquib Hasnain, Ikrama Hassan, Mahgol Sadat Hassan Zadeh Tabatabaei, Shokoufeh Hassani, Soheil Hassanipour, Hadi Hassankhani, Johannes Haubold, Rasmus J Havmoeller, Simon I Hay, Omar E Hegazi, Tadele Yohannes Hegena, Golnaz Heidari, Mohammad Heidari, Bartosz Helfer, Mehdi Hemmati, Claire A Henson, Claudiu Herteliu, Kamal Hezam, Thomas Kwadwo Hinneh, Yuta Hiraike, Nguyen Quoc Hoan, Ramesh Holla, Mohammad Enamul Hoque, Sahadat Hossain, Seyed Ehsan Hosseini, Hassan Hosseinzadeh, Mehdi Hosseinzadeh, Mihaela Hostiuc, Mohamed Hsairi, Chengxi Hu, Md Nazmul Huda, Michael Hultström, Kiavash Hushmandi, Javid Hussain, Nawfal R Hussein, Hong-Han Huynh, Segun Emmanuel Ibitoye, Oluwatope Olaniyi Idowu, Audrey L Ihler, Kevin S Ikuta, Olayinka Stephen Ilesanmi, Irena M Ilic, Milena D Ilic, Mohammad Tarique Imam, Mustapha Immurana, Leeberk Raja Inbaraj, Lalu Muhammad Irham, Mustafa Alhaji Isa, Md Rabiul Islam, Faisal Ismail, Nahlah Elkudssiah Ismail, Gaetano Isola, Masao Iwagami, Chidozie C D Iwu, Chinwe Juliana Iwu-Jaja, Vinothini J, Jalil Jaafari, Louis Jacob, Kathryn H Jacobsen, Farhad Jadidi-Niaragh, Nader

Jahanmehr, Haitham Jahrami, Nityanand Jain, Ammar Abdulrahman Jairoun, Abhishek Jaiswal, Mihajlo Jakovljevic, Reza Jalilzadeh Yengejeh, Roland Dominic G Jamora, Abubakar Ibrahim Jatau, Sabzali Javadov, Tahereh Javaheri, Shubha Jayaram, Jayakumar Jeganathan, Bijay Mukesh Jeswani, Heng Jiang, Mohammad Jokar, Nabi Jomehzadeh, Jost B Jonas, Tamas Joo, Abel Joseph, Nitin Joseph, Vivek Joshi, Charity Ehimwenma Joshua, Jacek Jerzy Jozwiak, Mikk Jürisson, Billingsley Kaambwa, Ali Kabir, Zubair Kabir, Vidya Kadashetti, Ethan M Kahn, Rizwan Kalani, Feroze Kaliyadan, Sanjay Kalra, Rajesh Kamath, Thanigaivelan Kanagasabai, Tanuj Kanchan, Himal Kandel, Edmund Wedam Kanmiki, Kehinde Kazeem Kanmodi, Sushil Kumar Kansal, Neeti Kapoor, Efstratios Karagiannidis, Mehrdad Karajizadeh, Paschalis Karakasis, Ibraheem M Karaye, André Karch, Hanie Karimi, Shilpi Karmakar, Faizan Zaffar Kashoo, Woldeteklehaymanot Dagne Kassahun, Nicholas J Kassebaum, Norito Kawakami, Neda Kaydi, Gbenga A Kayode, Foad Kazemi, Peter Njenga Keiyoro, Laura Kemmer, John H Kempen, Jessica A Kerr, Emmanuelle Kesse-Guyot, Yousef Saleh Khader, Morteza Abdullatif Khafaie, Himanshu Khajuria, Amirmohammad Khalaji, Alireza Khalilian, Faham Khamesipour, Asaduzzaman Khan, M Nuruzzaman Khan, Maseer Khan, Mohammad Jobair Khan, Moien AB Khan, Shaghayegh Khanmohammadi, Khaled Khatab, Haitham Khatatbeh, Moawiah Mohammad Khatatbeh, Mahalaqua Nazli Khatib, Armin Khavandegar, Feriha Fatima Khidri, Moein Khormali, Zahra Khorrami, Atulya Aman Khosla, Haneen Khreis, Helda Khusun, Zemene Demelash Kifle, Kwanghyun Kim, Min Seo Kim, Yun Jin Kim, Ruth W Kimokoti, Adnan Kisa, Sezer Kisa, Luke D Knibbs, David S Q Koh, Ali-Asghar Kolahi, Farzad Kompani, Jianqiu Kong, Gerbrand Koren, Miikka Korja, Vladimir Andreevich Korshunov, Oleksii Korzh, Nikhil Kothari, Sindhura Lakshmi Koulmane Laxminarayana, Kewal Krishan, Vijay Krishnamoorthy, Yuvaraj Krishnamoorthy, Bindu Krishnan, Kris J Krohn, Barthelemy Kuate Defo, Burcu Kucuk Bicer, Md Abdul Kuddus, Mohammed Kuddus, Nuworza Kugbey, Ilari Kuitunen, Mukhtar Kulimbet, Vishnutheertha Kulkarni, Ashish Kumar, Nithin Kumar, Vijay Kumar, Satyajit Kundu, Om P Kurmi, Asep Kusnali, Dian Kusuma, Carlo La Vecchia, Muhammad Awwal Ladan, Chandrakant Lahariya, Daphne Teck Ching Lai, Dharmesh Kumar Lal, Tea Lallukka, Judit Lám, Qing Lan, Tuo Lan, Iván Landires, Francesco Lanfranchi, Van Charles Lansingh, Bagher Larijani, Anders O Larsson, Savita Lasrado, Paolo Lauriola, Huu-Hoai Le, Long Khanh Dao Le, Nhi Huu Hanh Le, Thao Thi Thu Le, Caterina Ledda, Munjae Lee, Seung Won Lee, Shaun Wen Huey Lee, Yo Han Lee, Kate E LeGrand, James Leigh, Elvynna Leong, Temesgen L Lerango, Ming-Chieh Li, Wang-Zhong Li, Wei Li, Zhihui Li, Virendra S Ligade, Lee-Ling Lim, Stephen S Lim, Ro-Ting Lin, Shuzhi Lin, Chaojie Liu, Gang Liu, Jinli Liu, Jue Liu, Richard T Liu, Wei Liu, Xiaofeng Liu, Xuefeng Liu, Katherine M Livingstone, Erand Llanaj, Rubén López-Bueno, Stefan Lorkowski, Paulo A Lotufo, Jailos Lubinda, Giancarlo Lucchetti, Lisha Luo, Zheng Feei Ma, Mahmoud Mabrok, Nikolaos Machairas, Monika Machoy, Asma Mafhoumi, Mohammed Magdy Abd El Razek, Azzam A Maghazachi, D R Mahadeshwara Prasad, Mansour Adam Mahmoud, Elham Mahmoudi, Azeem Majeed, Omar Mohamed Makram, Konstantinos Christos Makris, Satyaveni Malasala, Venkatesh Maled, Kashish Malhotra, Ahmad Azam Malik, Iram Malik, Deborah Carvalho Malta, Abdullah A Mamun, Yosef Manla, Ali Mansour, Borhan Mansouri, Pejman Mansouri, Marjan Mansourian, Mohammad Ali Mansournia, Emmanuel Manu, Hamid Reza Marateb, Joemer C Maravilla, Ramon Martinez-Piedra, Santi Martini, Francisco Rogerlândio Martins-Melo, Miquel Martorell, Sharmeen Maryam, Yasith Mathangasinghe, Alexander G Mathioudakis, Fernanda Penido Matozinhos, Jishanth Mattumpuram, Andrea Maugeri, Pallab K Maulik, Mahsa Mayeli, Mohsen Mazidi, John J McGrath, Martin McKee, Michael A McPhail, Steven M McPhail, Enkeleint A Mechili, Asim Mehmood, Kamran Mehrabani-Zeinabad, Entezar Mehrabi Nasab, Toni Meier, Tesfahun Mekene Meto, Birye Dessalegn Mekonnen, Ritesh G Menezes, Belayneh Mengist, George A Mensah, Laverne G Mensah, Alexios-Fotios A Mentis, Sultan Ayoub Meo, Atte Meretoja, Tuomo J Meretoja, Abera M Mersha,

Bezawit Afework Mesfin, Tomislav Mestrovic, Kukulege Chamila Dinushi Mettananda, Sachith Mettananda, Tomasz Miazgowski, Georgia Micha, Irmina Maria Michalek, Ana Carolina Micheletti Gomide Nogueira de Sá, Ted R Miller, Mojde Mirarefin, Andreea Mirica, Erkin M Mirrakhimov, Arvin Mirshahi, Maryam Mirzaei, Ajay Kumar Mishra, Vinaytosh Mishra, Philip B Mitchell, Prasanna Mithra, Chaitanya Mittal, Babak Moazen, Madeline E Moberg, Ashraf Mohamadkhani, Abdalla Z Mohamed, Ahmed Ismail Mohamed, Jama Mohamed, Mouhand F H Mohamed, Nouh Saad Mohamed, Saeed Mohammadi, Abdollah Mohammadian-Hafshejani, Noushin Mohammadifard, Mustapha Mohammed, Salahuddin Mohammed, Shafiu Mohammed, Ali H Mokdad, Lorenzo Monasta, Stefania Mondello, Mohammad Ali Moni, AmirAli Moodi Ghalibaf, Catrin E Moore, Maryam Moradi, Yousef Moradi, Paula Moraga, Lidia Morawska, Rafael Silveira Moreira, Negar Morovatdar, Shane Douglas Morrison, Reza Mosaddeghi Heris, Elias Mossialos, Rohith Motappa, Ahmed Msherghi, Sumaira Mubarik, Lorenzo Muccioli, Ulrich Otto Mueller, Francesk Mulita, Kavita Munjal, Efrén Murillo-Zamora, Christopher J L Murray, Ana-Maria Musina, Ghulam Mustafa, Sathish Muthu, Saravanan Muthupandian, Raman Muthusamy, Muhammad Muzaffar, Woojae Myung, Ayoub Nafei, Ahamarshan Jayaraman Nagarajan, Shankar Prasad Nagaraju, Mohsen Naghavi, Pirouz Naghavi, Ganesh R Naik, Gurudatta Naik, Firzan Nainu, Tapas Sadasivan Nair, Dhairya P Nanavaty, Vinay Nangia, Sreenivas Narasimha Swamy, Bruno Ramos Nascimento, Gustavo G Nascimento, Abdulqadir J Nashwan, Zuhair S Natto, Javaid Nauman, Samidi N K Navaratna, Muhammad Naveed, Biswa Prakash Nayak, Vinod C Nayak, Rawlance Ndejjo, Sabina Onyinye Nduaguba, Hadush Negash, Ionut Negoi, Ruxandra Irina Negoi, Seyed Aria Nejadghaderi, Chakib Nejjari, Samata Nepal, Subas Neupane, Marie Ng, Georges Nguefack-Tsague, Josephine W Ngunjiri, Dang H Nguyen, Nhien Ngoc Y Nguyen, Phat Tuan Nguyen, Phuong The Nguyen, Van Thanh Nguyen, Duc Nguyen Tran Minh, Robina Khan Niazi, Ali Nikoobar, Amin Reza Nikpoor, Dina Nur Anggraini Ningrum, Chukwudi A Nnaji, Efaq Ali Noman, Shuhei Nomura, Nafise Noroozi, Bo Norrving, Jean Jacques Noubiap, Chisom Adaobi Nri-Ezedi, George Ntaios, Mengistu H Nunemo, Dieta Nurrika, Jerry John Nutor, Bogdan Oancea, Ismail A Odetokun, Martin James O'Donnell, Michael Safo Oduro, Hassan Okati-Aliabad, Akinkunmi Paul Okekunle, Osaretin Christabel Okonji, Andrew T Olagunju, Omotola O Olasupo, Matthew Idowu Olatubi, Arão Belitardo Oliveira, Gláucia Maria Moraes Oliveira, Abdulhakeem Abayomi Olorukooba, Isaac Iyinoluwa Olufadewa, Bolajoko Olubukunola Olusanya, Jacob Olusegun Olusanya, Yinka Doris Oluwafemi, Hany A Omar, Ahmed Omar Bali, Goran Latif Omer, Sokking Ong, Obinna E Onwujekwe, Kenneth Ikenna Onyedibe, Anita Frimpomaa Oppong, Michal Ordak, Raffaele Ornello, Heather M Orpana, Esteban Ortiz-Prado, Wael M S Osman, Samuel M Ostroff, Uchechukwu Levi Osuagwu, Adrian Otoiu, Nikita Otstavnov, Stanislav S Otstavnov, Amel Ouyahia, Mayowa O Owolabi, Oyetunde T Oyeyemi, Mahesh Padukudru P A, Kevin Pacheco-Barrios, Alicia Padron-Monedero, Jagadish Rao Padubidri, Pramod Kumar Pal, Tamás Palicz, Feng Pan, Hai-Feng Pan, Adrian Pana, Sujogya K Panda, Songhomitra Panda-Jonas, Ashok Pandey, Seithikurippu R Pandi-Perumal, Helena Ullyartha Pangaribuan, Ioannis Pantazopoulos, Anca Mihaela Pantea Stoian, Paraskevi Papadopoulou, Pragyan Paramita Parija, Romil R Parikh, Seoyeon Park, Sungchul Park, Nicholas Parsons, Ava Pashaei, Maja Pasovic, Roberto Passera, Shankargouda Patil, Dimitrios Patoulias, Uttam Paudel, Shrikant Pawar, Amy E Peden, Paolo Pedersini, Minjin Peng, Veincent Christian Filipino Pepito, Emmanuel K Peprah, Prince Peprah, Mario F P Peres, Arokiasamy Perianayagam, Konrad Pesudovs, Ionela-Roxana Petcu, Fanny Emily Petermann-Rocha, Hoang Tran Pham, Anil K Philip, Michael R Phillips, Daniela Pierannunzio, Manon Pigeolet, Zahra Zahid Piracha, Michael A Piradov, Mapa Prabhath Piyasena, Dietrich Plass, Evgenii Plotnikov, Kevan R Polkinghorne, Ramesh Poluru, Constance Dimity Pond, Maarten J Postma, Govinda Raj Poudel, Ahmad Pour-Rashidi, Akram Pourshams, Naeimeh Pourtaheri, Disha Prabhu, Sergio I Prada, Jalandhar Pradhan,

Pranil Man Singh Pradhan, Manya Prasad, Elton Junio Sady Prates, Hery Purnobasuki, Bharathi M Purohit, Jagadeesh Puvvula, Nameer Hashim Qasim, Ibrahim Qattea, Asma Saleem Qazi, Gangzhen Qian, Suli Qiu, Mehrdad Rabiee Rad, Amir Radfar, Raghu Anekal Radhakrishnan, Venkatraman Radhakrishnan, Hadi Raeisi Shahraki, Quinn Rafferty, Alireza Rafiei, Alberto Raggi, Pankaja Raghav Raghav, Fakher Rahim, Md Jillur Rahim, Mahban Rahimifard, Vafa Rahimi-Movaghar, Md Obaidur Rahman, Muhammad Aziz Rahman, Amir Masoud Rahmani, Bita Rahmani, Mohammad Rahmanian, Nazanin Rahmanian, Vahid Rahmanian, Setyaningrum Rahmawaty, Sathish Rajaa, Vinoth Rajendran, Prashant Rajput, Mahmoud Mohammed Ramadan, Shakthi Kumaran Ramasamy, Premkumar Ramasubramani, Pramod W Ramteke, Juwel Rana, Kritika Rana, Chhabi Lal Ranabhat, Amey Rane, Usha Rani, Chythra R Rao, Mithun Rao, Puja C Rao, Sowmya J Rao, Sina Rashedi, Vahid Rashedi, Mahsa Rashidi, Mohammad-Mahdi Rashidi, Zubair Ahmed Ratan, Giridhara Rathnaiah Babu, Santosh Kumar Rauniyar, Ilari Rautalin, David Laith Rawaf, Salman Rawaf, Reza Rawassizadeh, Christian Razo, Zinabu Ferede Ferede Reda, Murali Mohan Rama Krishna Reddy, Elrashdy Moustafa Mohamed Redwan, Lennart Reifels, Marissa B Reitsma, Bhageerathy Reshmi, Serge Resnikoff, Stefano Restaino, Luis Felipe Reyes, Maryam Rezaei, Nazila Rezaei, Negar Rezaei, Mohsen Rezaeian, Taeho Gregory Rhee, Antonio Luiz P Ribeiro, Jennifer Rickard, Célia Fortuna Rodrigues, Mónica Rodrigues, Jefferson Antonio Buendia Rodriguez, Leonardo Roever, Debby Syahru Romadlon, Gholamreza Roshandel, Gregory A Roth, Kunle Rotimi, Himanshu Sekhar Rout, Nitai Roy, Enrico Rubagotti, Susan Fred Rumisha, Michele Russo, Sacha Walde Ruzzante, Chandan S N, Aly M A Saad, Korosh Saber, Maha Mohamed Saber-Ayad, Siamak Sabour, Rajesh Sachdeva, Basema Saddik, Bashdar Abuzed Sadee, Ehsan Sadeghi, Elham Sadeghi Majd, Mohammad Reza Saeb, Umar Saeed, Mehdi Safari, Sare Safi, Sher Zaman Safi, Rajesh Sagar, Fatemeh Saheb Sharif-Askari, Narjes Saheb Sharif-Askari, Soumya Swaroop Sahoo, Zahra Saif, Mirza Rizwan Sajid, Joseph W Sakshaug, Payman Salamati, Afeez Abolarinwa Salami, Leili Salehi, Sana Salehi, Marwa Rashad Salem, Dauda Salihu, Giovanni A Salum, Hossein Samadi Kafil, Sara Samadzadeh, Yoseph Leonardo Samodra, Vijaya Paul Samuel, Abdallah M Samy, Juan Sanabria, Rama Krishna Sanjeev, Damian Francesco Santomauro, Milena M Santric-Milicevic, Made Ary Sarasmita, Sivan Yegnanarayana Iyer Saraswathy, Babak Saravi, Yaser Sarikhani, Rodrigo Sarmiento-Suárez, Gargi Sachin Sarode, Sachin C Sarode, Benn Sartorius, Arash Sarveazad, Brijesh Sathian, Monika Sawhney, Ganesh Kumar Saya, Abu Sayeed, Md Abu Sayeed, Mehdi Sayyah, Christophe Schinckus, Art Schuermans, Aletta Elisabeth Schutte, Michaël Schwarzinger, David C Schwebel, Falk Schwendicke, Subramanian Senthilkumaran, Dragos Serban, Marc L Serre, Yashendra Sethi, Mahan Shafie, Humaira Shah, Nilay S Shah, Pritik A Shah, Syed Mahboob Shah, Ataollah Shahbandi, Amira A Shaheen, Samiah Shahid, Wajeehah Shahid, Hamid R Shahsavari, Moyad Jamal Shahwan, Masood Ali Shaikh, Summaiya Zareen Shaikh, Muhammad Aaqib Shamim, Mehran Shams-Beyranvand, Mohammad Ali Shamshirgaran, Mohammad Anas Shamsi, Mohd Shanawaz, Abhishek Shankar, Sadaf Sharfaei, Amin Sharifan, Javad Sharifi-Rad, Ujjawal Sharma, Vishal Sharma, Rajesh P Shastry, Amin Shavandi, Amr Mohamed Elsayed Shehabeldine, Somia Shehzadi, Aziz Sheikh, Jiabin Shen, Amir Shiani, Desalegn Shiferaw, Mika Shigematsu, Min-Jeong Shin, Rahman Shiri, Aminu Shittu, Ivy Shiue, K M Shivakumar, Sina Shool, Seyed Afshin Shorofi, Rajan Shrestha, Sunil Shrestha, Kanwar Hamza Shuja, Kerem Shuval, Yafei Si, Emmanuel Edwar Siddig, Diego Augusto Santos Silva, Luís Manuel Lopes Rodrigues Silva, Soraia Silva, Thales Philipe R Silva, Abhinav Singh, Balbir Bagicha Singh, Baljinder Singh, Garima Singh, Harmanjit Singh, Jasvinder A Singh, Mahendra Singh, Narinder Pal Singh, Paramdeep Singh, Surjit Singh, Robert Sinto, Shravan Sivakumar, Samarjeet Singh Siwal, Søren T Skou, David A Sleet, Farrukh Sobia, Matiwos Soboka, Bogdan Socea, Sameh S M Soliman, Ranjani Somayaji, Yi Song, Reed J D Sorensen, Joan B Soriano, Ireneous N Soyiri, Michael Spartalis, Cory N Spencer, Chandrashekhar T

Sreeramareddy, Jeffrey D Stanaway, Muhammad Haroon Stanikzai, Caroline Stein, Fridolin Steinbeis, Caitlyn Steiner, Leo Stockfelt, Mark A Stokes, Kurt Straif, Saverio Stranges, Vetriselvan Subramaniyan, Muhammad Suleman, Rizwan Suliankatchi Abdulkader, David Sunkersing, Katharina S Sunnerhagen, Vinay Suresh, Chandan Kumar Swain, Lukasz Szarpak, Mindy D Szeto, Payam Tabaee Damavandi, Rafael Tabarés-Seisdedos, Seyyed Mohammad Tabatabaei, Ozra Tabatabaei Malazy, Seyed-Amir Tabatabaeizadeh, Shima Tabatabai, Celine Tabche, Santosh Kumar Tadakamadla, Yasaman Taheri Abkenar, Jabeen Taiba, Iman M Talaat, Jacques Lukenze Tamuzi, Ker-Kan Tan, Haosu Tang, Nuno Taveira, Yibekal Manaye Tefera, Worku Animaw Temesgen, Mohamad-Hani Temsah, Masayuki Teramoto, Dufera Rikitu Terefa, Enoch Teye-Kwadjo, Ramna Thakur, Pugazhenthan Thangaraju, Kavumpurathu Raman Thankappan, Rekha Thapar, Rasiah Thayakaran, Sathish Thirunavukkarasu, Nihal Thomas, Nikhil Kenny Thomas, Jing Tian, Ales Tichopad, Jansje Henny Vera Ticoalu, Tenaw Yimer Tiruye, Ruoyan Tobe-Gai, Tadesse Tolossa, Marcello Tonelli, Roman Topor-Madry, Fotis Topouzis, Mathilde Touvier, Marcos Roberto Tovani-Palone, Khaled Trabelsi, Jasmine T Tran, Mai Thi Ngoc Tran, Nghia Minh Tran, Domenico Trico, Indang Trihandini, Christopher E Troeger, Samuel Joseph Tromans, Evangelia Eirini Tsermpini, Munkhtuya Tumurkhuu, Aniefiok John Udoakang, Arit Udoh, Atta Ullah, Saeed Ullah, Sana Ullah, Muhammad Umair, Srikanth Umakanthan, Bhaskaran Unnikrishnan, Era Upadhyay, Daniele Urso, Jibrin Sammani Usman, Omid Vakili, Rohollah Valizadeh, Jef Van den Eynde, Priya Vart, Shoban Babu Varthya, Tommi Juhani Vasankari, Siavash Vaziri, Narayanaswamy Venketasubramanian, Madhur Verma, Massimiliano Veroux, Georgios-Ioannis Verras, Dominique Vervoort, Jorge Hugo Villafañe, Victor E Villalobos-Daniel, Gabriela Ines Villanueva, Manish Vinayak, Francesco S Violante, Bay Vo, Stein Emil Vollset, Simona Ruxandra Volovat, Theo Vos, Isidora S Vujcic, Yasir Waheed, Cong Wang, Fang Wang, Shu Wang, Yanzhong Wang, Yuan-Pang Wang, Mary Njeri Wanjau, Muhammad Waqas, Paul Ward, Abdul Waris, Emebet Gashaw Wassie, Kosala Gayan Weerakoon, Haftom Legese Legese Weldetinsaa, Yi Feng Wen, Taweewat Wiangkham, Nuwan Darshana Wickramasinghe, Caroline Wilkerson, Peter Willeit, Yen Jun Wong, Utoomporn Wongsin, Chenkai Wu, Dongze Wu, Felicia Wu, Zenghong Wu, Juan Xia, Hong Xiao, Xiaoyue Xu, Mukesh Kumar Yadav, Lin Yang, Yuichiro Yano, Habib Yaribeygi, Yuichi Yasufuku, Pengpeng Ye, Renjulal Yesodharan, Subah Abderehim Yesuf, Saber Yezli, Siyan Yi, Arzu Yiğit, Zeamanuel Anteneh Yigzaw, Dehui Yin, Paul Yip, Malede Berihun Yismaw, Dong Keon Yon, Naohiro Yonemoto, Yuyi You, Mustafa Z Younis, Zabihollah Yousefi, Chuanhua Yu, Yong Yu, Siddhesh Zadey, Vesna Zadnik, Fathiah Zakham, Nazar Zaki, Josefina Zakzuk, Giulia Zamagni, Sojib Bin Zaman, Ghazal G Z Zandieh, Aurora Zanghì, Fatemeh Zarimeidani, Mikhail Sergeevich Zastrozhin, Youjie Zeng, Chunxia Zhai, Anthony Lin Zhang, Haijun Zhang, Liqun Zhang, Meixin Zhang, Yunquan Zhang, Zhenyu Zhang, Zhi-Jiang Zhang, Hanqing Zhao, Yang Zhao, Peng Zheng, Chenwen Zhong, Jingjing Zhou, Juexiao Zhou, Shangcheng Zhou, Bin Zhu, Lei Zhu, Zhaohua Zhu, Boback Ziaeian, Magdalena Zielińska, Ghazal Zoghi, Alimuddin Zumla, and Samer H Zyoud.

Drafting the work or revising it critically for important intellectual content

Yohannes Habtegiorgis Abate, Cristiana Abbafati, Rouzbeh Abbasgholizadeh, Mohsen Abbasi-Kangevari, Samar Abd ElHafeez, Sherief Abd-Elsalam, Parsa Abdi, Mohammad Abdollahi, Auwal Abdullahi, Mesfin Abebe, Aidin Abedi, Armita Abedi, Roberto Ariel Abeldaño Zuñiga, Olumide Abiodun, Hassan Abolhassani, Mohamed Abouzid, Girma Beressa Aboye, Lucas Guimarães Abreu, Eman Abu-Gharbieh, Hana Jihad Jihad Abukhadijah, Salahdein Aburuz, Ahmed Abu-Zaid, Mesafint Molla Adane, Isaac Yeboah Addo, Giovanni Addolorato, Rufus Adesoji Adedoyin, Victor Adekanmbi, Juliana Bunmi Adetunji, Amin Adibi, Qorinah Estiningtyas Sakilah Adnani, Aanuoluwapo Adeyimika Afolabi, Rotimi Felix Afolabi, Muhammad Sohail Afzal, Saira Afzal, Suneth Buddhika Agampodi, Faith Agbozo, Antonella Agodi, Bright

Opoku Ahinkorah, Danish Ahmad, Firdos Ahmad, Anisuddin Ahmed, Ayman Ahmed, Luai A Ahmed, Muktar Beshir Ahmed, Safoora Ahmed, Syed Anees Ahmed, Marjan Ajami, Essona Matatom Akara, Hossein Akbarialiabad, Shiva Akhlaghi, Karolina Akinosoglou, Tomi Akinyemiju, Mohammed Ahmed Akkaif, Sreelatha Akkala, Blessing Akombi-Inyang, Salah Al Awaidy, Fares Alahdab, Samer O Alalalmeh, Tariq A Alalwan, Khurshid Alam, Almaza Albakri, Mohammad T AlBataineh, Wafa A Aldhaleei, Mulubirhan Assefa Alemayohu, Bassam Al-Fatly, Khairat Al-Habbal, Abid Ali, Amjad Ali, Liaqat Ali, Mohammed Usman Ali, Rafat Ali, Syed Shujait Shujait Ali, Waad Ali, Gianfranco Alicandro, Sami Almustanyir, Mahmoud A Alomari, Jordi Alonso, Jaber S Alqahtani, Ahmad Alrawashdeh, Rami Hani Al-Rifai, Sahel Majed Alrousan, Salman Khalifah Al-Sabah, Najim Z Alshahrani, Zaid Altaany, Awais Altaf, Jaffar A Al-Tawfiq, Deborah Oyine Aluh, Nelson Alvis-Guzman, Nelson J Alvis-Zakzuk, Hassan Alwafi, Mohammad Sami Al-Wardat, Yaser Mohammed Al-Worafi, Hany Aly, Safwat Aly, Karem H Alzoubi, Walid Al-Zyoud, Masous Aman Mohammadi, Reza Amani, Sohrab Amiri, Mohammad Hosein Amirzade-Iranaq, Enrico Ammirati, Hubert Amu, Dickson A Amugsi, Ganiyu Adeniyi Amusa, Robert Ancuceanu, Deanna Anderlini, Pedro Prata Andrade, Dhanalakshmi Angappan, Colin Angus, Abhishek Anil, Sneha Anil, Afifa Anjum, Amir Anoushiravani, Ippazio Cosimo Antonazzo, Catherine M Antony, Ernoiz Antriyandarti, Boluwatife Stephen Anuoluwa, Saeid Anvari, Anayochukwu Edward Anyasodor, Geminn Louis Carace Apostol, Jalal Arabloo, Razman Arabzadeh Bahri, Mosab Arafat, Brhane Berhe Aregawi, Abdulfatai Aremu, Benedetta Armocida, Michael Benjamin Arndt, Johan Ärnlöv, Mahwish Arooj, Kurnia Dwi Artanti, Ashokan Arumugam, Akram M Asbeutah, Saeed Asgary, Akeza Awealom Asgedom, Charlie Ashbaugh, Amir Askarinejad, Seyyed Shamsadin Athari, Prince Atorkey, Alok Atreya, Avinash Aujayeb, Marcel Ausloos, Andargie Abate Awoke, Beatriz Paulina Ayala Quintanilla, Carlos Ayestas Portugal, Sina Azadnajafabad, Rui M S Azevedo, Gulrez Shah Azhar, Ahmed Y Azzam, Insa Linnea Backhaus, Muhammad Badar, Ashish D Badiye, Soroush Baghdadi, Sara Bagherieh, Ruhai Bai, Atif Amin Baig, Jennifer L Baker, Shankar M Bakkannavar, Madhan Balasubramanian, Ovidiu Constantin Baltatu, Kiran Bam, Soham Bandyopadhyay, Aduragbemi Banke-Thomas, Hansi Bansal, Martina Barchitta, Mainak Bardhan, Erfan Bardideh, Suzanne Lyn Barker-Collo, Till Winfried Bärnighausen, Francesco Barone-Adesi, Hiba Jawdat Barqawi, Lope H Barrero, Amadou Barrow, Afisu Basiru, João Diogo Basso, Mohammad-Mahdi Bastan, Sanjay Basu, Bernhard T Baune, Nebiyou Simegnew Bayileyegn, Thomas Beaney, Amir Hossein Behnoush, Maryam Beiranvand, Yannick Béjot, Alehegn Bekele, Uzma Iqbal Belgaumi, Arielle Wilder Bell, Michelle L Bell, Muhammad Bashir Bello, Olorunjuwon Omolaja Bello, Luis Belo, Apostolos Beloukas, Salaheddine Bendak, Derrick A Bennett, Isabela M Bensenor, Habib Benzian, Azizullah Beran, Zombor Berezvai, Paulo J G Bettencourt, Akshaya Srikanth Bhagavathula, Neeraj Bhala, Dinesh Bhandari, Sonu Bhaskar, Ajay Nagesh Bhat, Vivek Bhat, Gurjit Kaur Bhatti, Jasvinder Singh Bhatti, Manpreet S Bhatti, Rajbir Bhatti, Mohiuddin Ahmed Bhuiyan, Boris Bikbov, Jessica Devin Bishai, Catherine Bisignano, Atanu Biswas, Bijit Biswas, Raaj Kishore Biswas, Tone Bjørge, Micheal Kofi Boachie, Hosea Boakye, Moses John Bockarie, Virginia Bodolica, Aadam Olalekan Bodunrin, Milad Bonakdar Hashemi, Sri Harsha Boppana, Hamed Borhany, Alejandro Botero Carvajal, Soufiane Boufous, Christopher Boxe, Dejana Braithwaite, Luisa C Brant, Amanpreet Brar, Michael Brauer, Nicholas J K Breitborde, Susanne Breitner, Hermann Brenner, Gabrielle Britton, Colin Stewart Brown, Norma B Bulamu, Danilo Buonsenso, Richard A Burns, Reinhard Busse, Yasser Bustanji, Florentino Luciano Caetano dos Santos, Jack Cagney, Lucero Cahuana-Hurtado, Daniela Calina, Luis Alberto Cámera, Luciana Aparecida Campos, Ismael R Campos-Nonato, Angelo Capodici, Sinclair Carr, Giulia Carreras, Juan J Carrero, Andrea Carugno, Felix Carvalho, Márcia Carvalho, Joao Mauricio Castaldelli-Maia, Carlos A Castañeda-Orjuela, Giulio Castelpietra, Ferrán Catalá-López, Alberico L Catapano, Maria Sofia Cattaruzza, Arthur Caye, Christopher R Cederroth, Luca

Cegolon, Muthia Cenderadewi, Ester Cerin, Joshua Chadwick, Chiranjib Chakraborty, Sandip Chakraborty, Jeffrey Shi Kai Chan, Joht Singh Chandan, Rama Mohan Chandika, Pankaj Chaturvedi, An-Tian Chen, Haowei Chen, Meng Xuan Chen, Simiao Chen, Nicolas Cherbuin, Gerald Chi, Ritesh Chimoriya, Patrick R Ching, Jesus Lorenzo Chirinos-Caceres, William C S Cho, Bryan Chong, Hitesh Chopra, Rajiv Chowdhury, Devasahayam J Christopher, Dinh-Toi Chu, Eric Chung, Muhammad Chutiyami, Iolanda Cioffi, Alyssa Columbus, Joao Conde, Samuele Cortese, Paolo Angelo Cortesi, Vera Marisa Costa, Michael H Criqui, Natália Cruz-Martins, Alanna Gomes da Silva, Xiaochen Dai, Zhaoli Dai, Giovanni Damiani, Emanuele D'Amico, Lucio D'Anna, Nihar Ranjan Dash, Mohsen Dashti, Claudio Alberto Dávila-Cervantes, Nicole Davis Weaver, Dragos Virgil Davitoiu, Fernando Pio De la Hoz, Alejandro de la Torre-Luque, Louisa Degenhardt, Ivan Delgado-Enciso, Juana Maria Delgado-Saborit, Chalachew Kassaw Demoze, Edgar Denova-Gutiérrez, Nikolaos Dervenis, Emina Dervišević, Hardik Dineshbhai Desai, Rupak Desai, Syed Masudur Rahman Dewan, Amol S Dhane, Mandira Lamichhane Dhimal, Meghnath Dhimal, Sameer Dhingra, Vishal R Dhulipala, Diana Dias da Silva, Daniel Diaz, Luis Antonio Diaz, Michael J Diaz, Adriana Dima, Delaney D Ding, Monica Dinu, Thanh Chi Do, Camila Bruneli do Prado, Masoud Dodangeh, Sushil Dohare, Wanyue Dong, Mario D'Oria, Rajkumar Doshi, Leila Doshmangir, Robert Kokou Dowou, Tim Robert Driscoll, Ashel Chelsea Dsouza, Haneil Larson Dsouza, Samuel C Dumith, Bruce B Duncan, Senbagam Duraisamy, Arkadiusz Marian Dziedzic, David Edvardsson, Ferry Efendi, Ebrahim Eini, Michael Ekholuenetale, Rabie Adel El Arab, Maysaa El Sayed Zaki, Faris El-Dahiyat, Noha Mousaad Elemam, Frank J Elgar, Ghada Metwally Tawfik ElGohary, Muhammed Elhadi, Ahmed O Elmehrath, Omar Abdelsadek Abdou Elmeligy, Ibrahim Elsohaby, Theophilus I Emeto, Negin Esfandiari, Majid Eslami, Sayed Vahid Esmaeili, Farshid Etaee, Natalia Fabin, Adeniyi Francis Fagbamigbe, Omotayo Francis Fagbule, Saman Fahimi, Luca Falzone, Mohammad Fareed, Carla Sofia e Sá Farinha, MoezAlIslam Ezzat Mahmoud Faris, Pawan Sirwan Faris, Andre Faro, Folorunso Oludayo Fasina, Ali Fatehizadeh, Nelsensius Klau Fauk, Timur Fazylov, Seyed-Mohammad Fereshtehnejad, Pietro Ferrara, Nuno Ferreira, Getahun Fetensa, Bikila Regassa Feyisa, Irina Filip, Florian Fischer, Ida Fitriana, Joanne Flavel, Carsten Flohr, David Flood, Nataliya A Foigt, Morenike Oluwatoyin Folayan, Lisa M Force, Daniela Fortuna, Matteo Foschi, Alberto Freitas, Sara D Friedman, Blima Fux, Sridevi G, Peter Andras Gaal, Santosh Gaihre, Márió Gajdács, Emmanuela Gakidou, Yaseen Galali, Silvano Gallus, Aravind P Gandhi, Balasankar Ganesan, Mohammad Arfat Ganiyani, Vanessa Garcia, Ravindra K Garg, Rupesh K Gautam, Tilaye Gebru Gebi, Miglas W Gebregergis, Lemma Getacher, Fataneh Ghadirian, Sadegh Ghafarian, Khalid Yaser Ghailan, Alireza Ghajar, MohammadReza Ghasemi, Ghazal Ghasempour Dabaghi, Afsaneh Ghasemzadeh, Fariba Ghassemi, Ramy Mohamed Ghazy, Nasim Gholizadeh, Mahsa Ghorbani, Gabriela Fernanda Gil, Paramjit Singh Gill, Tiffany K Gill, Ebisa Zerihun Gindaba, Alem Girmay, James C Glasbey, Elena V Gnedovskaya, Laszlo Göbölös, Myron Anthony Godinho, Amit Goel, Davide Golinelli, Sameer Vali Gopalani, Giuseppe Gorini, Houman Goudarzi, Alessandra C Goulart, Mahdi Gouravani, Anmol Goyal, Michal Grivna, Shi-Yang Guan, Giovanni Guarducci, Avirup Guha, Stefano Guicciardi, Snigdha Gulati, David Gulisashvili, Cui Guo, Bhawna Gupta, Mohak Gupta, Rahul Gupta, Rajat Das Gupta, Rajeev Gupta, Sapna Gupta, Veer Bala Gupta, Vivek Kumar Gupta, Farrokh Habibzadeh, Parham Habibzadeh, Tesfahun Simon Hadaro, Zahra Hadian, Nils Haep, Hamed Haghi-Aminjan, Dariush Haghmorad, Alemayehu Hailu, Adel Hajj Ali, Esam S Halboub, Aram Halimi, Brian J Hall, Rabih Halwani, Randah R Hamadeh, Nadia M Hamdy, Sajid Hameed, Ahmad Hammoud, Nasrin Hanifi, Zaim Anan Haq, Harapan Harapan, Josep Maria Haro, Ahmed I Hasaballah, Mohammad Jahid Hasan, S M Mahmudul Hasan, Hamidreza Hasani, Nadim Hashmeh, Md Saquib Hasnain, Amr Hassan, Mahgol Sadat Hassan Zadeh Tabatabaei, Johannes Haubold, Rasmus J Havmoeller, Simon I Hay, Jeffrey J Hebert, Omar E Hegazi, Tadele Yohannes Hegena, Golnaz Heidari,

Bartosz Helfer, Mehdi Hemmati, Claudiu Herteliu, Kamal Hezam, Yuta Hiraike, Nguyen Quoc Hoan, Ramesh Holla, Nobuyuki Horita, Sahadat Hossain, Seyed Ehsan Hosseini, Sorin Hostiuc, Hanno Hoven, Johnathan M Hsu, Junjie Huang, Md Nazmul Huda, Erin N Hulland, Kiavash Hushmandi, Javid Hussain, Hong-Han Huynh, Segun Emmanuel Ibitoye, Audrey L Ihler, Olayinka Stephen Ilesanmi, Irena M Ilic, Milena D Ilic, Mustapha Immurana, Lalu Muhammad Irham, Mustafa Alhaji Isa, Md Rabiul Islam, Faisal Ismail, Nahlah Elkudssiah Ismail, Hiroyasu Iso, Gaetano Isola, Chidozie C D Iwu, Chinwe Juliana Iwu-Jaja, Vinothini J, Louis Jacob, Kathryn H Jacobsen, Kasra Jahankhani, Nader Jahanmehr, Haitham Jahrami, Akhil Jain, Nityanand Jain, Abhishek Jaiswal, Mihajlo Jakovljevic, Roland Dominic G Jamora, Abubakar Ibrahim Jatau, Shubha Jayaram, Bijay Mukesh Jeswani, Jost B Jonas, Tamas Joo, Abel Joseph, Nitin Joseph, Charity Ehimwenma Joshua, Jacek Jerzy Jozwiak, Mikk Jürisson, Ali Kabir, Vidya Kadashetti, Ethan M Kahn, Rizwan Kalani, Feroze Kaliyadan, Sanjay Kalra, Rajesh Kamath, Thanigaivelan Kanagasabai, Himal Kandel, Edmund Wedam Kanmiki, Kehinde Kazeem Kanmodi, Sushil Kumar Kansal, Neeti Kapoor, Efstratios Karagiannidis, Mehrdad Karajizadeh, Paschalis Karakasis, Shama D Karanth, André Karch, Asima Karim, Hanie Karimi, Shilpi Karmakar, Faizan Zaffar Kashoo, Hengameh Kasraei, Nicholas J Kassebaum, Srinivasa Vittal Katikireddi, Joonas H Kauppila, Gbenga A Kayode, Foad Kazemi, Peter Njenga Keiyoro, John H Kempen, Jessica A Kerr, Emmanuelle Kesse-Guyot, Himanshu Khajuria, Amirmohammad Khalaji, M Nuruzzaman Khan, Maseer Khan, Mohammad Jobair Khan, Moien AB Khan, Shaghayegh Khanmohammadi, Khaled Khatab, Haitham Khatatbeh, Moawiah Mohammad Khatatbeh, Mahalaqua Nazli Khatib, Hamid Reza Khayat Kashani, Feriha Fatima Khidri, Elaheh Khodadoust, Atulya Aman Khosla, Mahmood Khosrowjerdi, Haneen Khreis, Helda Khusun, Kwanghyun Kim, Min Seo Kim, Yun Jin Kim, Adnan Kisa, Sezer Kisa, Ann Kristin Skrindo Knudsen, Farzad Kompani, Jianqiu Kong, Oleksii Korzh, Parvaiz A Koul, Sindhura Lakshmi Koulmane Laxminarayana, Kewal Krishan, Yuvaraj Krishnamoorthy, Barthelemy Kuate Defo, Burcu Kucuk Bicer, Md Abdul Kuddus, Mohammed Kuddus, Nuworza Kugbey, Ilari Kuitunen, Mukhtar Kulimbet, Vishnutheertha Kulkarni, Vijay Kumar, Satyajit Kundu, Om P Kurmi, Dian Kusuma, Tezer Kutluk, Carlo La Vecchia, Muhammad Awwal Ladan, Lucie Laflamme, Chandrakant Lahariya, Tea Lallukka, Judit Lám, Iván Landires, Berthold Langguth, Ariane Laplante-Lévesque, Bagher Larijani, Anders O Larsson, Savita Lasrado, Huu-Hoai Le, Nhi Huu Hanh Le, Thao Thi Thu Le, Caterina Ledda, Munjae Lee, Paul H Lee, Kate E LeGrand, Elvynna Leong, Janni Leung, Wang-Zhong Li, Wei Li, Zhihui Li, Lee-Ling Lim, Chaojie Liu, Richard T Liu, Wei Liu, Xuefeng Liu, Katherine M Livingstone, Erand Llanaj, Ayush Lohiya, Rubén López-Bueno, Platon D Lopukhov, Stefan Lorkowski, Giancarlo Lucchetti, Hengliang lv, Hawraz Ibrahim M Amin, Zheng Feei Ma, Mahmoud Mabrok, Nikolaos Machairas, Monika Machoy, Asma Mafhoumi, Mohammed Magdy Abd El Razek, Mansour Adam Mahmoud, Elham Mahmoudi, Omar Mohamed Makram, Venkatesh Maled, Kashish Malhotra, Ahmad Azam Malik, Lesibana Anthony Malinga, Deborah Carvalho Malta, Abdullah A Mamun, Ana Laura Manda, Borhan Mansouri, Marjan Mansourian, Lorenzo Giovanni Mantovani, Emmanuel Manu, Hamid Reza Marateb, Gabriel Martinez, Ramon Martinez-Piedra, Francisco Rogerlândio Martins-Melo, Miquel Martorell, Wolfgang Marx, Sharmeen Maryam, Yasith Mathangasinghe, Alexander G Mathioudakis, Fernanda Penido Matozinhos, Jishanth Mattumpuram, Andrea Maugeri, Pallab K Maulik, Mahsa Mayeli, Antonio Mazzotti, Anna Laura W McKowen, Susan A McLaughlin, Steven M McPhail, Enkeleint A Mechili, Asim Mehmood, Kamran Mehrabani-Zeinabad, Entezar Mehrabi Nasab, Toni Meier, Tesfahun Mekene Meto, Ritesh G Menezes, George A Mensah, Alexios-Fotios A Mentis, Sultan Ayoub Meo, Atte Meretoja, Tuomo J Meretoja, Tomislav Mestrovic, Kukulege Chamila Dinushi Mettananda, Sachith Mettananda, Tomasz Miazgowski, Georgia Micha, Irmina Maria Michalek, Ana Carolina Micheletti Gomide Nogueira de Sá, Ted R Miller, Mojgan Mirghafourvand, Antonio Mirijello, Arvin Mirshahi, Philip B Mitchell,

Prasanna Mithra, Chaitanya Mittal, Babak Moazen, Gabriele Mocciaro, Abdalla Z Mohamed, Mouhand F H Mohamed, Nouh Saad Mohamed, Esmaeil Mohammadi, Abdollah Mohammadian-Hafshejani, Hussen Mohammed, Mustapha Mohammed, Salahuddin Mohammed, Shafiu Mohammed, Ali H Mokdad, Lorenzo Monasta, Stefania Mondello, Mohammad Ali Moni, AmirAli Moodi Ghalibaf, Catrin E Moore, Maryam Moradi, Yousef Moradi, Paula Moraga, Rafael Silveira Moreira, Shane Douglas Morrison, Jakub Morze, Reza Mosaddeghi Heris, Parsa Mousavi, Ahmed Msherghi, Ulrich Otto Mueller, Francesk Mulita, BV Murlimanju, Christopher J L Murray, Ana-Maria Musina, Ghulam Mustafa, Sathish Muthu, Saravanan Muthupandian, Muhammad Muzaffar, Ayoub Nafei, Ahamarshan Jayaraman Nagarajan, Shankar Prasad Nagaraju, Gabriele Nagel, Mohsen Naghavi, Soroush Najdaghi, Noureddin Nakhostin Ansari, Dhairya P Nanavaty, Sreenivas Narasimha Swamy, Delaram Narimani Davani, Bruno Ramos Nascimento, Gustavo G Nascimento, Abdulqadir J Nashwan, Zuhair S Natto, Javaid Nauman, Samidi N K Navaratna, Biswa Prakash Nayak, Vinod C Nayak, Sabina Onyinye Nduaguba, Hadush Negash, Ionut Negoi, Ruxandra Irina Negoi, Mohammad Hadi Nematollahi, Samata Nepal, Subas Neupane, Georges Nguefack-Tsague, Josephine W Ngunjiri, Dang H Nguyen, Phat Tuan Nguyen, Van Thanh Nguyen, Duc Nguyen Tran Minh, Robina Khan Niazi, Amin Reza Nikpoor, Bo Norrving, Jean Jacques Noubiap, Chisom Adaobi Nri-Ezedi, George Ntaios, Mpiko Ntsekhe, Jerry John Nutor, Bogdan Oancea, Ismail A Odetokun, Martin James O'Donnell, Adesola Adenike Ogunfowokan, Abiola Ogunkoya, In-Hwan Oh, Sylvester Reuben Okeke, Osaretin Christabel Okonji, Andrew T Olagunju, Matthew Idowu Olatubi, Gláucia Maria Moraes Oliveira, Abdulhakeem Abayomi Olorukooba, Bolajoko Olubukunola Olusanya, Jacob Olusegun Olusanya, Hany A Omar, Obinna E Onwujekwe, Kenneth Ikenna Onyedibe, Michal Ordak, Verner N Orish, Raffaele Ornello, Heather M Orpana, Alberto Ortiz, Esteban Ortiz-Prado, Wael M S Osman, Samuel M Ostroff, Uchechukwu Levi Osuagwu, Adrian Otoiu, Nikita Otstavnov, Amel Ouyahia, Mayowa O Owolabi, Ifeoluwa Temitayo Oyeyemi, Oyetunde T Oyeyemi, Mahesh Padukudru P A, Kevin Pacheco-Barrios, Alicia Padron-Monedero, Jagadish Rao Padubidri, Tamás Palicz, Feng Pan, Sujogya K Panda, Songhomitra Panda-Jonas, Ashok Pandey, Seithikurippu R Pandi-Perumal, Ioannis Pantazopoulos, Anca Mihaela Pantea Stoian, Pragyan Paramita Parija, Romil R Parikh, Sungchul Park, Nicholas Parsons, Roberto Passera, Shankargouda Patil, Dimitrios Patoulias, Venkata Suresh Patthipati, Uttam Paudel, Shrikant Pawar, Hamidreza Pazoki Toroudi, Amy E Peden, Paolo Pedersini, Umberto Pensato, Veincent Christian Filipino Pepito, Arokiasamy Perianayagam, Norberto Perico, Konrad Pesudovs, Ionela-Roxana Petcu, Fanny Emily Petermann-Rocha, Hoang Tran Pham, Michael R Phillips, Daniela Pierannunzio, Zahra Zahid Piracha, Michael A Piradov, Enrico Pisoni, Mapa Prabhath Piyasena, Dimitri Poddighe, Ramesh Poluru, Djordje S Popovic, Fabio Porru, Maarten J Postma, Govinda Raj Poudel, Sergio I Prada, Jalandhar Pradhan, Pranil Man Singh Pradhan, Manya Prasad, Elton Junio Sady Prates, Nameer Hashim Qasim, Ibrahim Qattea, Asma Saleem Qazi, Suli Qiu, Mehrdad Rabiee Rad, Amir Radfar, Venkatraman Radhakrishnan, Hadi Raeisi Shahraki, Alberto Raggi, Pankaja Raghav Raghav, Fakher Rahim, Mahban Rahimifard, Vafa Rahimi-Movaghar, Bita Rahmani, Mohammad Rahmanian, Masoud Rahmati, Diego Raimondo, Sathish Rajaa, Vinoth Rajendran, Prashant Rajput, Mahmoud Mohammed Ramadan, Shakthi Kumaran Ramasamy, Kritika Rana, Chhabi Lal Ranabhat, Usha Rani, Annemarei Ranta, Chythra R Rao, Mithun Rao, Puja C Rao, Sowmya J Rao, Davide Rasella, Vahid Rashedi, Ashkan Rasouli-Saravani, Giridhara Rathnaiah Babu, Santosh Kumar Rauniyar, Ilari Rautalin, David Laith Rawaf, Salman Rawaf, Elrashdy Moustafa Mohamed Redwan, Giuseppe Remuzzi, Bhageerathy Reshmi, Serge Resnikoff, Luis Felipe Reyes, Maryam Rezaei, Nazila Rezaei, Mavra A Riaz, Jennifer Rickard, Mónica Rodrigues, Jefferson Antonio Buendia Rodriguez, Leonardo Roever, Luca Ronfani, Gholamreza Roshandel, Morteza Rostamian, Gregory A Roth, Kunle Rotimi, Himanshu Sekhar Rout, Bedanta Roy, Nitai Roy, Enrico

Rubagotti, Guilherme de Andrade Ruela, Susan Fred Rumisha, Michele Russo, Sacha Walde Ruzzante, Chandan S N, Aly M A Saad, Korosh Saber, Maha Mohamed Saber-Ayad, Simona Sacco, Perminder S Sachdev, Rajesh Sachdeva, Basema Saddik, Bashdar Abuzed Sadee, Masoumeh Sadeghi, Elham Sadeghi Majd, Umar Saeed, Mehdi Safari, Rajesh Sagar, Dominic Sagoe, Fatemeh Saheb Sharif-Askari, Narjes Saheb Sharif-Askari, Amirhossein Sahebkar, Soumya Swaroop Sahoo, Monalisha Sahu, Zahra Saif, Mirza Rizwan Sajid, Nasir Salam, Afeez Abolarinwa Salami, Luciane B Salaroli, Marwa Rashad Salem, Mohammed Z Y Salem, Dauda Salihu, Sohrab Salimi, Giovanni A Salum, Sara Samadzadeh, Abdallah M Samy, Juan Sanabria, Milena M Santric-Milicevic, Aswini Saravanan, Babak Saravi, Yaser Sarikhani, Rodrigo Sarmiento-Suárez, Gargi Sachin Sarode, Sachin C Sarode, Arash Sarveazad, Davide Sattin, Monika Sawhney, Ganesh Kumar Saya, Abu Sayeed, Md Abu Sayeed, Christophe Schinckus, Maria Inês Schmidt, Art Schuermans, Aletta Elisabeth Schutte, David C Schwebel, Siddharthan Selvaraj, Mohammad H Semreen, Dragos Serban, Yashendra Sethi, Mahan Shafie, Nilay S Shah, Pritik A Shah, Syed Mahboob Shah, Samiah Shahid, Moyad Jamal Shahwan, Ali S Shalash, Muhammad Aaqib Shamim, Mehran Shams-Beyranvand, Mohammad Ali Shamshirgaran, Mohammad Anas Shamsi, Mohd Shanawaz, Abhishek Shankar, Amin Sharifan, Manoj Sharma, Ujjawal Sharma, Vishal Sharma, Rajesh P Shastry, Amr Mohamed Elsayed Shehabeldine, Pavanchand H Shetty, Mika Shigematsu, Aminu Shittu, K M Shivakumar, Velizar Shivarov, Sina Shool, Seyed Afshin Shorofi, Sunil Shrestha, Kerem Shuval, Emmanuel Edwar Siddig, Diego Augusto Santos Silva, Soraia Silva, Thales Philipe R Silva, Colin R Simpson, Abhinav Singh, Balbir Bagicha Singh, Harmanjit Singh, Jasvinder A Singh, Narinder Pal Singh, Paramdeep Singh, Robert Sinto, Shravan Sivakumar, Natia Skhvitaridze, Søren T Skou, David A Sleet, Bogdan Socea, Shahabaddin Solaimanian, Ranjan Solanki, Shipra Solanki, Sameh S M Soliman, Ranjani Somayaji, Joan B Soriano, Ireneous N Soyiri, Michael Spartalis, Chandrashekhar T Sreeramareddy, Panagiotis Stachteas, Jeffrey D Stanaway, Muhammad Haroon Stanikzai, Dan J Stein, Fridolin Steinbeis, Sabine Steinke, Paschalis Steiropoulos, Mark A Stokes, Saverio Stranges, Narayan Subedi, Muhammad Suleman, Johan Sundström, David Sunkersing, Katharina S Sunnerhagen, Chandan Kumar Swain, Lukasz Szarpak, Mindy D Szeto, Payam Tabaee Damavandi, Rafael Tabarés-Seisdedos, Ozra Tabatabaei Malazy, Seyed-Amir Tabatabaeizadeh, Shima Tabatabai, Celine Tabche, Mohammad Tabish, Santosh Kumar Tadakamadla, Yasaman Taheri Abkenar, Ken Takahashi, Iman M Talaat, Jacques Lukenze Tamuzi, Ker-Kan Tan, Nathan Y Tat, Nuno Taveira, Yibekal Manaye Tefera, Arash Tehrani-Banihashemi, Mohamad-Hani Temsah, Masayuki Teramoto, Dufera Rikitu Terefa, Ramna Thakur, Pugazhenthan Thangaraju, Sathish Thirunavukkarasu, Nihal Thomas, Jing Tian, Ales Tichopad, Tenaw Yimer Tiruye, Musliu Adetola Tolani, Marcello Tonelli, Roman Topor-Madry, Mathilde Touvier, Marcos Roberto Tovani-Palone, Khaled Trabelsi, Jasmine T Tran, Mai Thi Ngoc Tran, Nghia Minh Tran, Domenico Trico, Samuel Joseph Tromans, Thien Tan Tri Tai Truyen, Aristidis Tsatsakis, Evangelia Eirini Tsermpini, Aniefiok John Udoakang, Arit Udoh, Srikanth Umakanthan, Brigid Unim, Bhaskaran Unnikrishnan, Era Upadhyay, Daniele Urso, Jibrin Sammani Usman, Asokan Govindaraj Vaithinathan, Omid Vakili, Mario Valenti, Jef Van den Eynde, Orsolya Varga, Shoban Babu Varthya, Tommi Juhani Vasankari, Milena Vasic, Narayanaswamy Venketasubramanian, Madhur Verma, Massimiliano Veroux, Georgios-Ioannis Verras, Dominique Vervoort, Jorge Hugo Villafañe, Leonardo Villani, Manish Vinayak, Francesco S Violante, Vasily Vlassov, Stein Emil Vollset, Simona Ruxandra Volovat, Theo Vos, Cong Wang, Fang Wang, Shu Wang, Yanzhong Wang, Yuan-Pang Wang, Paul Ward, Emebet Gashaw Wassie, Kosala Gayan Weerakoon, Robert G Weintraub, Taweewat Wiangkham, Nuwan Darshana Wickramasinghe, Peter Willeit, Yen Jun Wong, Dongze Wu, Juan Xia, Hong Xiao, Mukesh Kumar Yadav, Sajad Yaghoubi, Kazumasa Yamagishi, Lin Yang, Yuichiro Yano, Yuichi Yasufuku, Saber Yezli, Arzu Yiğit, Dong Keon Yon, Naohiro Yonemoto, Vesna Zadnik,

Sojib Bin Zaman, Ghazal G Z Zandieh, Aurora Zanghì, Heather J Zar, Iman Zare, Fatemeh Zarimeidani, Mikhail Sergeevich Zastrozhin, Youjie Zeng, Chunxia Zhai, Haijun Zhang, Xiu-Ju George Zhao, Yang Zhao, Yong Zhao, Chenwen Zhong, Zhaohua Zhu, Makan Ziafati, Magdalena Zielińska, Ghazal Zoghi, Thomas Zoller, Alimuddin Zumla, Sa'ed H Zyoud, and Samer H Zyoud.

Managing the estimation or publications process

Catherine M Antony, Aleksandr Y Aravkin, Charlie Ashbaugh, Michael Assmus, Michael Brauer, Catherine S Chen, Xiaochen Dai, Kara Estep, Alize J Ferrari, Lisa M Force, Emmanuela Gakidou, Hailey Hagins, Simon I Hay, Nicholas J Kassebaum, Molly B Kassel, Kris J Krohn, Kate E LeGrand, Anna Laura W McKowen, Susan A McLaughlin, Ali H Mokdad, Erin C Mullany, Christopher J L Murray, Mohsen Naghavi, Erin M O'Connell, Samuel M Ostroff, Maja Pasovic, David M Pigott, Puja C Rao, Jeffrey D Stanaway, Caitlyn Steiner, and Katherine M Wells.
